# *Fortunella venosa* (Champ. ex Benth.) C. C. Huang and *F. hindsii* (Champ. ex Benth.) Swingle as Independent Species: Evidence From Morphology and Molecular Systematics and Taxonomic Revision of *Fortunella* (Rutaceae)

**DOI:** 10.3389/fpls.2022.867659

**Published:** 2022-05-12

**Authors:** Ting Wang, Ling-Ling Chen, Hui-Juan Shu, Fang You, Xiao-Li Liang, Jun Li, Jing Ren, Vincent Okelo Wanga, Fredrick Munyao Mutie, Xiu-Zhen Cai, Ke-Ming Liu, Guang-Wan Hu

**Affiliations:** ^1^College of Life Sciences, Hunan Normal University, Changsha, China; ^2^Department of Ecological Environment of Hunan Province, Changsha, China; ^3^Key Laboratory of Plant Germplasm Enhancement and Specialty Agriculture, Wuhan Botanical Garden, Chinese Academy of Sciences, Wuhan, China; ^4^UCAS, University of Chinese Academy of Sciences, Beijing, China

**Keywords:** *Fortunella* Swingle, *Citrus* Linn., *Fortunella venosa*, *Fortunella hindsii*, independent species, taxonomic revision

## Abstract

Recently, the systematic status of *Fortunella* Swingle and its taxonomy has attracted much attention. Flora of China incorporates *Fortunella* into *Citrus* Linn. and treats all species of the traditional *Fortunella* as one species, namely *Citrus japonica* (Thunb.) Swingle. Furthermore, *F. venosa* (Champ. ex Benth.) C. C. Huang and *F. hindsii* (Champ. ex Benth.) Swingle are currently considered as synonyms of *C. japonica*. In this paper, morphological, palynological, and phylogenetic analyses were used to systematically explore the taxonomic status of traditional *Fortunella*. The key morphological features that differed among the *Fortunella* species were the leaf and the petiole hence could be key in its taxonomic classification of the species. Additionally, pollen morphological analysis based on the pollen size, germination grooves, polar, and equatorial axes also supported the separation of the species. The results of the phylogenetic analysis showed that each of the three species clustered separately, hence strongly supporting the conclusion of independent species. In addition, the phylogenetic analysis showed that the two genera clustered closely together hence our results support the incorporation of *Fortunella* into *Citrus*. Based on the above, this article has revised the classification of the traditional *Fortunella* and determined that this genus has three species, namely; *F. venosa*, *F. hindsii*, and *F. japonica*. *F. venosa* and *F. hindsii* are placed in the *Citrus* as separate species, and their species names still use the previous specific epithet. The revised scientific names of the new combinations of *F. venosa* and *F. hindsii* are as follows: *Citrus venosa* (Champ. ex Benth.) K. M. Liu, X. Z. Cai, and G. W. Hu, *comb. nov*. and *Citrus hindsii* (Champ. ex Benth.) K. M. Liu, G. W. Hu, and X. Z. Cai, *comb. nov*. *F. venosa* is the original species of *Fortunella*, *F. venosa* and *F. hindsii* are both listed as the second-class key protected wild plants in China. Therefore, the establishment of the taxonomic status of *F. venosa* and *F. hindsii* not only deepens our understanding, importance, and the complexity of the systematic classification of *Fortunella*, but is also significant for global biodiversity conservation, genetic resources for breeding purposes, and population genetics.

## Introduction

*Fortunella* Swingle is a genus in Rutaceae Juss. and Subfam Aurantioideae Engl., whose species grow as shrubs or small trees. The taxonomy and systematics of *Fortunella* have over the past been a puzzle with several authors agreeing on 6 described species numbers within the genus (Huang, 1997; Khalvashi and Mermarne, 2014; Kong et al., 2020), distributed in Southeast Asia. In 1846, the genus was introduced in Europe by a Scottish traveler and botanist, Robert Fortune, and after subsequent cultivation, it was widely recognized in Europe and named after Robert Fortune in the 1880s. The genus *Fortunella* was described in 1915 by Swingle, at this onset of classification (Swingle, 1915), he described 4 species based on leaf, fruit, seed morphological traits. The four species described were *F. margarita* (Lour.) Swingle., *F. japonica* (Thunb.) Swingle., *F. crassifolia* Swingle. and *F. hindsii* (Champ. ex Benth.) Swingle. However, subsequent studies brought out differing opinions with different authors publishing their insights into the new species to enrich the number. In 1928, *F. swinglei* Tanaka was published in Malaysia (Tanaka, 1928). Huang published a new species of *F. bawangica* C. C. Huang and a new combination of *F. venosa* (Champ. ex Benth.) C. C. Huang from China (Huang, 1991). In 1997, two species, *F. venosa* and *F. bawangica* were added to the Flora Reipublicae Popularis Sinicae (Huang, 1997) upon its publication. Hence *Fortunella* had five species in the Flora Reipublicae Popularis Sinicae, including *F. margarita*, *F. japonica*, *F. hindsii*, *F. venosa*, and *F. bawangica*. The classification of *Fortunella* has been difficult because *Fortunella* and *Citrus* are very closely related, and can easily hybridize, resulting in difficulties in their classification (Wu et al., 2017). Thus, the classification of this genus has been puzzling plant taxonomists. Up to now, the traditional systematics of *Fortunella* is still controversial and many scholars insist that *Fortunella* should be regarded as an independent genus (Khalvashi and Mermarne, 2014; Jose et al., 2015; Xu et al., 2019). Especially in the latest List of National Key Protected Wild Plants in China (443,253,634.html),^1^ *Fortunella* is still regarded as an independent genus. It is considered that *Citrus* and *Fortunella* are sister groups. Mabberley, on the other hand, merged *Fortunella*, *Eremocitrus* Swingle, and *Microcitrus* Swingle into the *Citrus* Linn. based on morphological and molecular evidence when revising the Australian *Fortunella* and related genera in 1998 (Mabberley, 1998). In 2008, Zhang merged *Fortunella* into the genus *Citrus* when compiling the Flora of China (Zhang et al., 2008). The APGIV^2^ plant classification system revised in 2016 also incorporates *Fortunella* into the genus *Citrus*. Additionally, the classification within the *Fortunella* group is also controversial. Not only is *Fortunella* regarded as an independent genus in China’s most recent national key of protected plant list, but the two species, *F. venosa* and *F. hindsii*, are also regarded as independent species of the genus.^3^ Swingle classified only 4 species within the genus (Swingle, 1915). However, Khalvashi, Kong, and Huang classified six species within the genus (Huang, 1997; Khalvashi and Mermarne, 2014; Kong et al., 2020). But, in the Flora of China (Zhang et al., 2008), the traditional genus *Fortunella* is treated as one species, namely *C. japonica*, and the other species of the original group are synonyms (the scientific name of *F. bawangica* is not treated). Therefore, up to now, there is no unified thought on the number of *Fortunella* species.

*Fortunella venosa* (Champ. ex Hook.) C. C. Huang is an evergreen shrub, usually 0.25–1 m high, with simple leaves, and ellipsoid or subglobose fruit, which is 6–8 mm in diameter, and fruit yellow or orange when matured (Huang, 1997). Similar to *Citrus*, its fruits are aromatic and edible (Huang et al., 2010). The species is of high ornamental value and can bloom throughout the year through artificial means/processes. Different parts of the plant can be used as medicine, with the effect of cooling and detoxifying the body, and can help prevent many diseases (Huang et al., 2010; Liu, 2014). The distribution range is restricted to northern latitudes 25°50′–27°50′. The range is slightly northerly than that of the tetraploid *F. hindsii* (Huang, 1997). On the other hand, *F. hindsii* is a shrub, usually 1–3 m high, and bears fruits of 8–10 mm in diameter. Its roots are used in medicine to treat phlegm, cough, and other symptoms. Due to the influence of cold weather and anthropogenic activities, the number of wild plants of *F. venosa* and *F. hindsii* is rapidly declining (Huang et al., 2010). In 2004, *F. venosa* was listed as an endangered species in the Red List of Chinese Species (Wang and Xie, 2004). In 2021, the two species were listed in the list of national second-class key protected wild plants in China’s List of National Key Protected Wild Plants (4,443,253,634. html).^4^

During evolution, the typical characteristic plant tissue structure evolved, which has remained stable in various plants for a long time and varies between plant species (Liu, 1991). As a result, integrating morphological data with phylogenetic analysis is important because it reveals a series of phenotypic traits that characterize molecular taxonomy and thus assists systematists in proposing species and their cladistic relationships (Lee and Palci, 2015). Morphological features such as fruit type and flower characters have been used in the classification of Rutaceae (Huang, 1997; Zhang et al., 2008). Similarly, plant morphology is also one of the means of classification of *Fortunella*.

Pollen is an intimate part of plant reproduction (Hornick et al., 2021), and plays an important role in the life cycle of angiosperms plants (Zhang et al., 2016). Pollen morphology and ornamentation are important bases for the detailed classification of plants (Sharma, 1968; Cai et al., 2008). Pollen ornamentation is exquisite and delicate, with a complex exine structure, having varying shape characteristics among different species (Wu et al., 2018b). Its morphological characteristics are controlled by genes (Fang, 2002), and are less affected by external factors (Li et al., 2018). Additionally, it is highly genetically conserved and can provide a lot of information for taxonomic classification (Fang, 2002).

Previous researches reinforce the importance of pollen morphology in the identification and characterization of species of Rutaceae (Singhal et al., 1983; Victor and Van Wyk, 1998; Ulukuş et al., 2016; Dutra and Gasparino, 2018). Although *Citrus* s.l (sensu lato) is a large genus with high ornamental and edibility values, there is a paucity of studies on pollen morphology, with only sporadic reports on individual species (Shao and Fan, 2002; Lin et al., 2013; Mou et al., 2013; Cheng et al., 2016). Through the study of pollen shape, size, and outer wall decoration, to establish the quantitative index of pollen morphology of *Fortunella* and explore its relationship with species evolution and classification. The palynological evidence provides evidence for the taxonomic revision and systematic study of this group.

The aim of this study is to explore the phylogenetic relationship between *Fortunella* and *Citrus* and to determine the exact species placed under *Fortunella*. To achieve these goals, this research used the plastome to reconstruct the phylogeny through the sampling of dense taxa of the entire section. The structural characteristics of plastomes can be used to study genetic diversity and species evolution, and play an important role in formulating germplasm resources protection policies (Zhang et al., 2021). In the past 20 years, plastid DNA sequences have been widely used in plant phylogenetic studies (Bausher et al., 2006; Su et al., 2014; Asaf et al., 2018; Feng et al., 2020; Wang et al., 2021). Complete plastome sequences can provide valuable data sets to solve complex evolutionary relationships (Cui et al., 2006; Jansen et al., 2007; Moore et al., 2010). This study provides new plastid genomic resources for *Fortunella*, which will facilitate its genetic diversity assessment and plant molecular identification.

## Materials and Methods

### Material Collection

All the species of *Fortunella* used in this study were collected from more than 10 provinces in China (*F. japonica* is all naturally distributed in the provinces of the material, involving a total of 10 provinces with 11 materials). Photographs were taken during the botanical field investigation, and the corresponding buds were collected and stored in the FAA fixative. Young leaves were immediately packed in sealed bags for DNA extraction and stored after drying with silica gel. To avoid experimental errors caused by intermediate hybridization, all materials of *Fortunella* were collected from wild individuals, except for *F. margarita* which had no wild representatives (Detailed voucher specimen information is provided in Supplementary Table S1). Voucher specimens were deposited at the Herbarium of Hunan Normal University (HNNU).

### Experimental Method

#### Microscopic Observation of Ovary Structures

The transverse section of the ovary was obtained by the freehand sectioning method according to Zhang (Zhang et al., 2015). The sections were observed and photographed for recording under an objective lens of 40x using an optical microscope.

#### SEM Observation of Pollen Structures

Pollen from three species was collected for scanning electron microscope (SEM) observation. The pollen was rinsed in phosphoric acid buffer before being dehydrated and dried in increments of 60, 70, 80, 90, and 95% ethanol. They were afterward coated with vacuum spray gold plating using JEOL JFC-1600 ion sputtering instrument. They were then observed under JEOL JSM-649OLV scanning electron microscope (SEM) with a working voltage of 20KV. The typical and representative visual fields were selected, and the population, individual, polar view, equatorial view, and mesh morphology of pollen were photographed and recorded 300 times, 3,000 times, and 10,000 times, respectively. Representative pollen grains were selected, then the polar axis length (P), equatorial axis length (E), mesh ridge width (W), and mesh diameter (D) were measured, and the polar axis length (P) and equatorial axis length (E) ratio calculated, i.e., the value of P/E. After measuring 20 groups of data for each type of pollen, the average value was calculated in excel for pollen description, we followed the established standard methods. For pollen description, refer to Introduction to Palynology and Walker’s (1974) (Moore and Webb, 1978; Wei, 2003).

#### Sequencing of Plastid Genome and ITS

The optimized cetyltrimethylammonium bromide (CTAB; Doyle, 1991) method was used to extract total DNA from 0.5 g |silica gel dried plant leaves. After PCR amplification (Supplementary Table S2), the plastome and ITS gene fragments were sequenced using the second-generation sequencing platform Illumina of novogen company in Beijing and the second-generation sequencing platform of Tsingke Bio’s company. The sequence completed by the sequencing is uploaded to the NCBI accession number, see Supplementary Table S3.

#### Assembly and Annotation of the Plastome

The raw sequence data were assembled using GetOrganelle version 1. 7. 4, and the complete plastome was generated (Jin et al., 2020). The final assembly map was visualized using Bandage (Wick et al., 2015) to examine the automatically generated plastome. The assembled plastid genome was annotated using Plastid Genome Annotator (PGA) software (Qu et al., 2019) with the initial reference as *Amborella trichopoda* (GenBank accession number: GCA_U000471905.1). The published genome of *Citrus maxima* (Burm.) ^*^Merr (MN782007) was used as a reference for further annotation. The Geneious software was used to manually annotate and supplement problematic genes (Camacho et al., 2009; Bankevich et al., 2012; Langmead and Salzberg, 2012). The annotated complete plastid genome was submitted to the GenBank, and the accession number can be found in Supplementary Table S3. The whole plastome circle map was drawn by Organelle genome Draw (OGDRAW) online software (Lohse et al., 2007).

#### Analysis of the Phylogenetic Relationship

To further understand the position of *F. venosa* and *F. hindsii* in Rutaceae. Phylogenetic relationships were analyzed using three sets of data: complete plastome sequences (whole-genome tree), protein-coding genes (PCGs; CDS tree), and ITS (fragment tree). Phylogenetic trees were reconstructed using 24 complete plastid genomes obtained in this study and 12 plastid genomes downloaded from the NCBI database (Supplementary Table S3). *Atalantia kwangtungensis* Merrill and Chun ex Swingle was selected as the outgroup. The outgroup was chosen based on the current APGIV classification system and the tree of life phylogeny. Using MAFFTv7 (Katoh and Standley, 2013), plastid sequences were aligned and manually checked if necessary. The aligned sequences were used to analyze variable sites, informative sites, and nucleotide diversity using the software BioEdit (Hall et al., 2011) and DnaSP v.5.0 (Librado and Rozas, 2009). Model Finder (Kalyaanamoorthy et al., 2017) was used to determine the most appropriate DNA sequence evolution model for the data set. The most suitable models for Bayesian and IQ-tree analysis are GTR + F + I + G4 and GTR + F + R3, respectively. Based on the 81 CDS of the 36 species, after CDS splicing alignment, all positions containing gaps and missing data were eliminated, and the sequence alignment was 23,404 amino acids. The phylogenetic relationships were estimated using the ML, PhyML, and BI analyses done using the IQ-Tree and MrBayes, respectively, integrated with Phylosuite (Zhang et al., 2020). The CDS tree was constructed using ML, PhyML, and BI methods. The BI method was used to construct a phylogenetic tree with the entire plastid genome and ITS gene fragments as the auxiliary (Zhang et al., 2020). The reconstructed tree was visualized using FigTree version 1.4.4 (Rambaut, 2018).

## Results

### Morphological Characteristics of *F. japonica*, *F. venosa*, and *F. hindsii*

**Figure 1:**
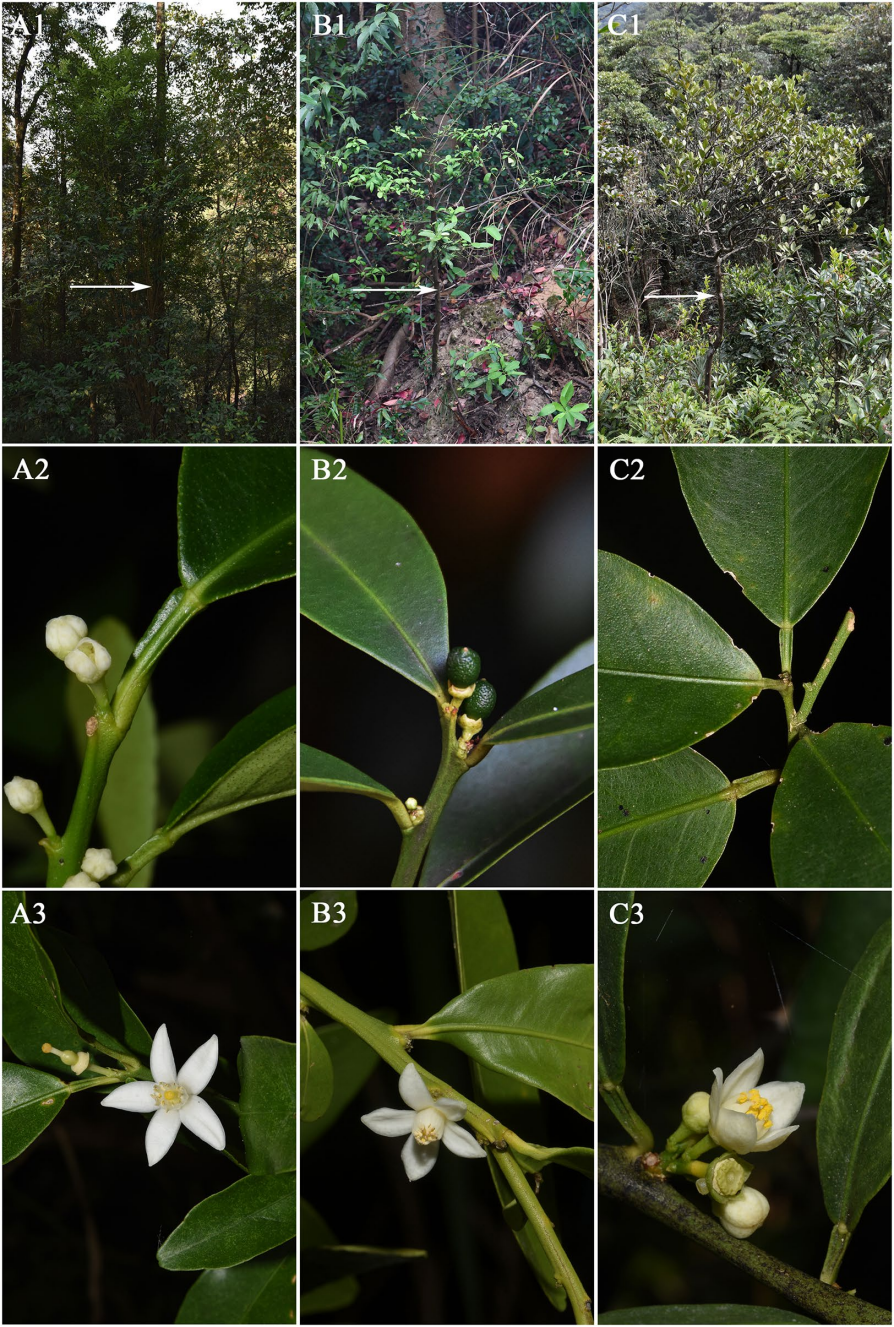
Comparison of morphological characteristics of Fortunella. **A**. *F. japonica* **B**. *F. venosa* **C**. *F. hindsii*. 1. Whole plant 2. Leaf type 3. Flower.

**Figure 2:**
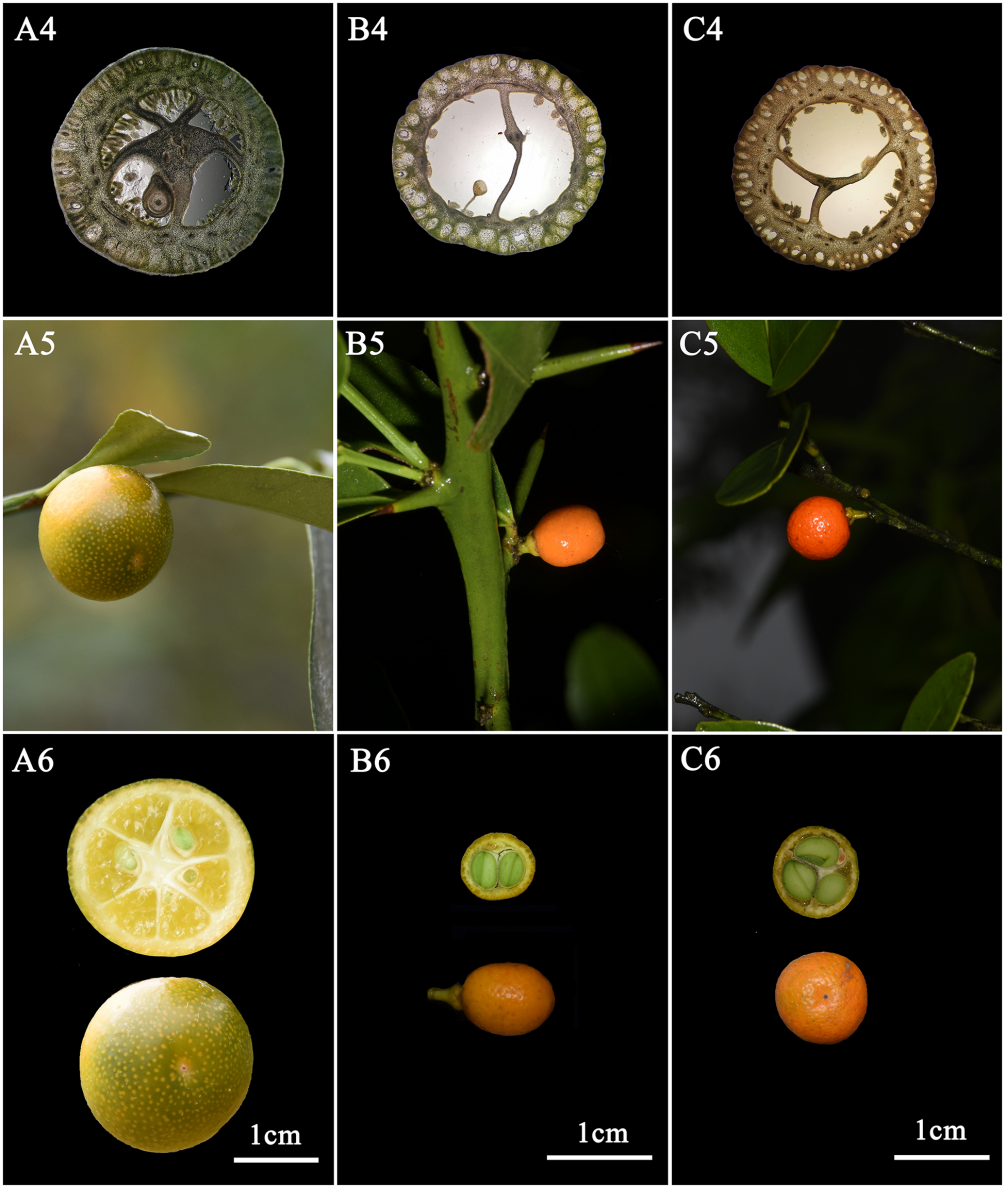
Comparison of morphological characteristics of Fortunella. **A**. *F. japonica* **B**. *F. venosa* **C**. *F. hindsii*. 3. Ovary cross-section 4. Fruit 5. Fruit cross-section.

*F. venosa* and *F. hindsii* as traditional plants of *Fortunella* are different from *F. japonica* in many morphological characteristics and should be independent species, respectively, according to our results. For example, life form, leaf type, Fruit size, etc. A detailed morphological comparison of those species was conducted (Figures 1, 2; Table 1). The above characteristics reveal that there are obvious differences among *F. venosa*, *F. hindsii*, and *F. japonica*, which should be regarded as independent species.

**Table 1:** Comparison of morphological characteristics of *Fortunella.*

Species name	*F. japonica*	*F. venosa*	*F. hindsii*
Life form	Small trees or shrubs, usually 2–9 m tall, trunk usually slender	Small shrubs, usually 0.25-1 m tall, trunk usually not slender	Shrubs, usually not more than 3 m tall, trunk usually not slender
Leaf type	Leaves 1-foliolate, that is, there is a joint at the joint between the petiole and the leaf	Simple leaves, that is, there is no joint at the joint between the petiole and the leaf	Leaves 1-foliolate, extremely rare and single leaf
Petiole length	6–10 mm long	1–3(−5) mm long	6–9 mm long
Number of ovary locules	4–6	2–4	3–4
Stamen number	(15–)20–25	10–15	20 (19–21)
Fruit size (diameter)	(16–)20–25 mm	6–8 mm	8–10 mm
Fruit shape	Spherical	Ellipsoidal or sub-spheroidal	Spherical

### Palynological Characteristics of *F. japonica*, *F. venosa*, and *F. hindsii*

**Figure 3:**
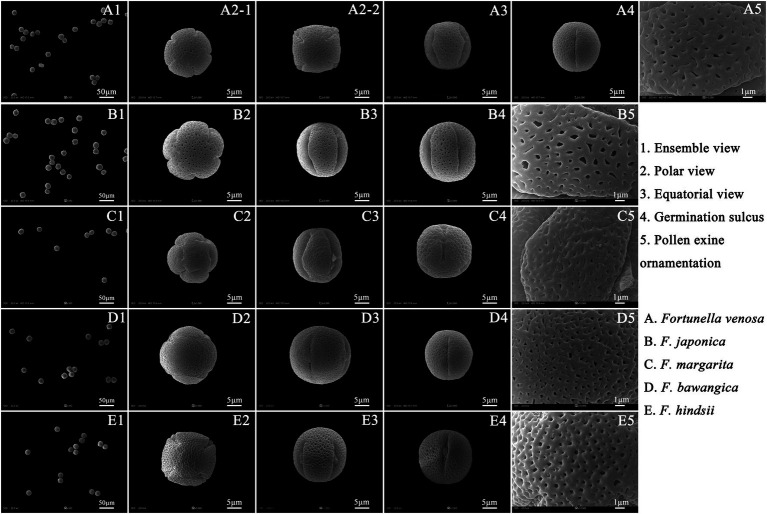
Scanning electron microscopic observation of the pollen morphology.

Pollen’s shape was similar, with all of them being spherical. The polar view was round with 4–5 valve splits, and 4–5 corporates; the equatorial view was nearly round with 1–2 corporates (Figure 3; Table 2). Among them, *F. venosa* had both 4 and 5 corporates, *F. japonica* had 4 and 5 corporates, and the *F. hindsii* has 4 corporates. This is the characteristic that *F. hindsii* is easily distinguishable from *F. venosa* and *F. japonica*. In general, the pollen of *F. japonica* (16.86–20.78) * (15.26–20.11) μm was slightly larger than that of *F. venosa* (16.33–18.95) * (14.26–17.30) μm and *F. hindsii* (17.50–19.90) * (17.21–19.53) μm (Table 2). The maximum ratio (P/E) of the polar axis (P) to the equatorial axis (E) is *F. venosa*, which was 1.0897.

**Table 2:** Pollen morphological characteristics of 3 *Fortunella.*

Species name	*F. venosa* 1	*F. venosa* 2	*F. japonica*	*F. hindsii*
Germination grooves number	4, 5	4, 5	4, 5	4
Polar axis (μm)	17.135 (16.33–18.95)	17.451 (18.25–20.02)	19.121 (16.86–20.78)	18.546 (17.50–19.90)
Equatorial axis (μm)	15.725 (14.26–17.30)	16.161 (18.23–19.86)	18.276 (15.26–20.11)	18.214 (17.21–19.53)
P/E value	1.0897 (1.02–1.15)	1.0798 (1.01–1.08)	1.0452 (1.00–1.19)	1.0183 (1.00–1.04)
Murus width (μm)	0.7330 (0.68–0.74)	0.7343 (0.68–0.82)	0.6897 (0.51–0.91)	0.5406 (0.51–0.57)
Lumen diameter (μm)	0.0681 (0.04–0.12)	0.0666 (0.06–0.09)	0.0667 (0.02–0.21)	0.0633 (0.05–0.08)
Mesh quantity under the same area	23.2 (22–24)	22.8 (21–25)	32.2 (18–46)	40 (35–47)

The exine ornamentation of *F. venosa*, *F. japonica*, *F. hindsii*, *F. margarita*, and *F. bawangica*. Was reticulate, with shallow foveolate meshes and irregular perforations. There is also a strong similarity between the mesh shape and the mesh ridge (The ridge was flat and broad or slightly protuberant, and the surface was smooth). But there were differences in the number of meshes under the same area. The number of meshes under 25 μm^2^ are all greater than 20, and the maximum number of *F. hindsii* can reach 41, *F. japonica* was 32.2. This micromorphological feature is the palynological basis for *F. venosa* and *F. hindsii* as independent species.

### Plastid Genome Characteristics of *F. japonica*, *F. venosa*, and *F. hindsii*

**Figure 4:**
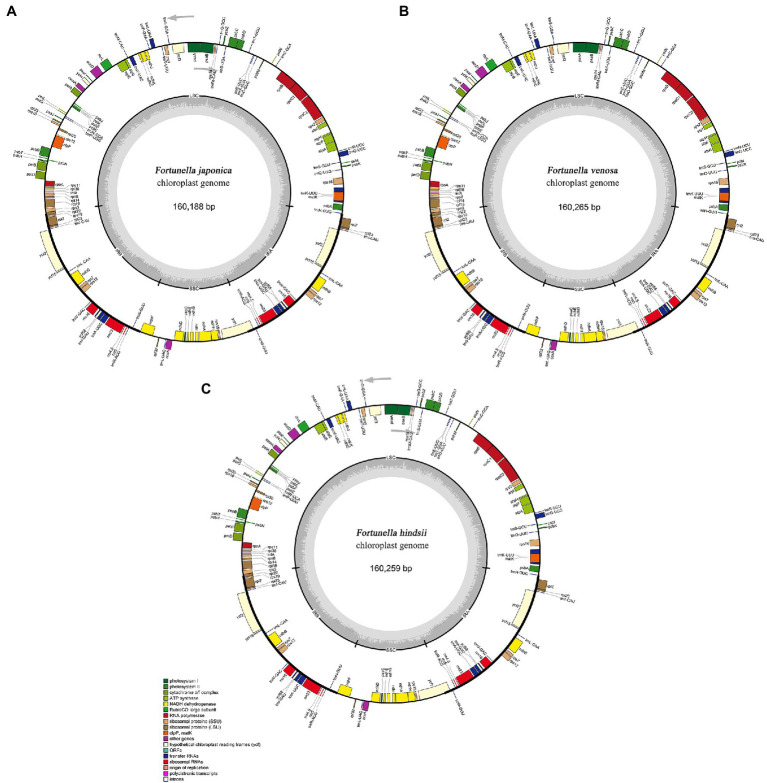
Gene maps of three traditional Fortunella chloroplast genomes: **(A)** *F. japonica*; **(B)** *F. venosa*; **(C)** *F. hindsii*. Genes located outside the circle are transcribed clockwise, and genes located inside the circle are transcribed counterclockwise. The colored bands belong to different functional groups of protein-coding genes. Grey circles indicate the small single-copy region (SSC), the large single-copy region (LSC), and the two inverted repeats (IRa, IRb).

All of the base coverage reads used to assemble the chloroplast genome were more than 750 times. The chloroplast genome of the 3 species has been submitted to the GenBank of the National Center for Biotechnology Information (NCBI; Supplementary Table S3). The lengths of the three chloroplast genomes ranged from 160,229 bp (*F. japonica*) to 160,265 bp (*F. venosa*; Table 3). It has a typical ring-shaped four-part structure, as do most angiosperms (Figure 4). The two IR regions are separated by an LSC region and an SSC region. The total number of genes ranged between 134 and 135, with protein-coding genes (CDS) being 88–89 in number, and similar gene types among the species’ plastomes (Table 4). The rRNA and tRNA numbers were 8 and 37, respectively, and the three species were identical. The total GC of the entire cp genome was 34.8%. However, there were differences in the distribution of GC content among the regions, with the GC content of the IR region being significantly higher than that of the LSC or SSC (Table 3).

**Table 3:** Summaries of complete chloroplast genomes of *Fortunella.*

Species	*F. venosa*	*F. japonica*	*F. hindsii*
Genome size (bp)	160,265	160,229	160,248
Large single copy (LSC, bp)	87,597	87,564	87,574
Small single copy (SSC, bp)	18,732	18,721	18,728
Inverted repeat (IR, bp)	26,968	26,972	26,973
Gene	134	134	134
protein-coding genes	89	89	90
rRNA	8	8	8
tRNA	37	37	37
GC content (%)			
Total genome	38.4	38.4	38.4
LSC	36.8	36.7	36.7
SSC	33.2	33.2	33.2
IR	43.0	42.0	42.9
GenBank accession	MZ457935	MN495932	OM773621

**Table 4:** Genes present and functional gene category in *Fortunella.*

**Category**	**Group of genes**	**Name of genes**
Self-replication	Ribosomal protein (LSU)	*rpl2**^a^*,*^c^**, rpl14, rpl16**^a^**, rpl20, rpl22**^c^**, rpl23**^c^**, rpl33, rpl32, rpl36*
	Ribosomal proteins (SSU)	*rps2, rps3, rps4, rps7**^c^**, rps8, rps11, rps12**^a^*,*^c^**, rps14, rps15, rps16**^a^**, rps18, rps19**^c^*
	DNA-dependent RNA polymerase	*rpoA, rpoB, rpoC1* *^a^* *, rpoC2*
	rRNA genes	*rrn4.5* *^c^* *, rrn5* *^c^* *, rrn16* *^c^* *, rrn23* *^c^*
	tRNA genes	*trnA-UGC**^a^*,*^c^**, trnC-GCA, trnD-GUC, trnE-UUC, trnF-GAA, trnfM-CAU*
		*trnG-GCC, trnG-UCC**^a^**, trnH-GUG, trnI-CAU**^c^**, trnI-GAU**^a^*,*^c^**, trnK-UUU*
		*trnL-CAA* *^c^* *, trnL-UAA* *^a^* *, trnL-UAG, trnM-CAU, trnN-GUU* *^c^* *, trnP-UGG*
		*trnQ-UUG, trnR-ACG* *^c^* *, trnR-UCU, trnS-GCU, trnS-GGA, trnS-UGA, trnT-GGU*
		*trnT-UGU, trnV-GAC* *^c^* *, trnV-UAC* *^a^* *, trnW-CCA, trnY-GUA*
Photosynthesis	Photosystem I	*psaA, psaB, psaC, psaI, psaJ*
	Photosystem II	*psbA, psbB, psbC, psbD, psbE, psbF, psbH, psbI, psbJ, psbK, psbL, psbM*
		*psbN, psbT, psbZ*
	NADPH dehydrogenase	*ndhA**^a^**, ndhB**^a^*,*^c^*, *ndhC*, *ndhD*, *ndhE*, *ndhF*, *ndhG*, *ndhH*, *ndhI*, *ndhJ*, *ndhK*
	ATP synthase	*atpA, atpB, atpE, atpF* *^a^* *, atpH, atpI*
	Cytochrome c-type synthesis	*petA, petB* *^a^* *, petD* *^a^* *, petG, petL, petN*
	Rubisco	*rbcL*
Other genes	Maturase	*matK*
	Cytochrome c-type synthesis	*ccsA*
	Carbon metabolism	*cemA*
	Fatty acid synthesis	*accD*
	Transfer initiation factor	*infA*
	Proteolysis	*clpP* ^b^
Unknown	Conserved open reading frames	*ycf1, ycf2* *^c^* *, ycf3* ^b^ *, ycf4, ycf68* *^c^* *, ycf15* *^c^*

a Genes have one intron.

b Genes have two introns.

c Genes located in the inverted repeats.

### Phylogenetic Analysis

**Figure 5:**
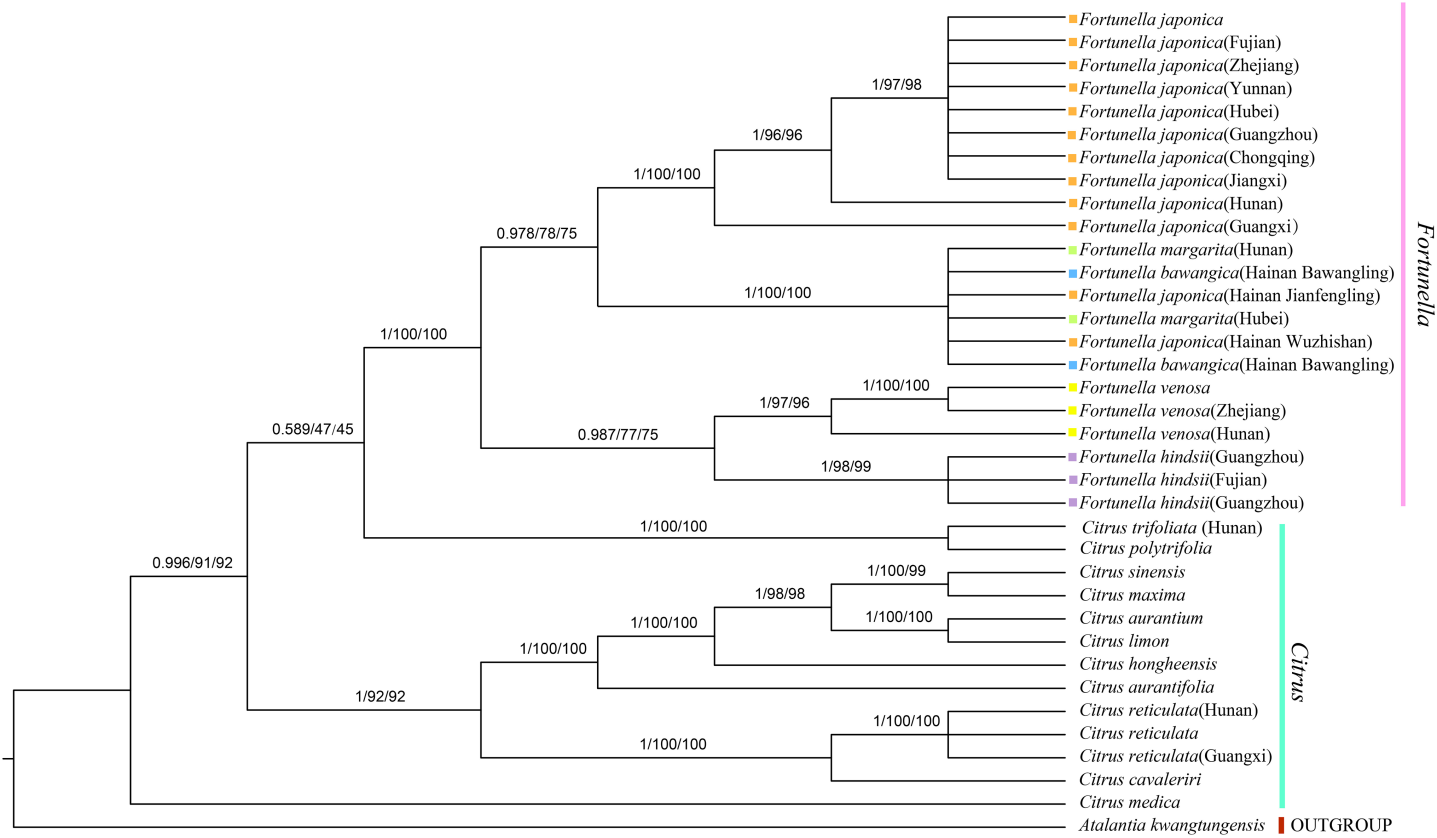
Phylogenetic tree generation using 81 CDS from 36 species common in Rutaceae species. *Atalantia kwangtungensis* was used as the outgroup. The numbers above the branch represent bootstrap support value for BI/ML/PhyML methods.

**Figure 6:**
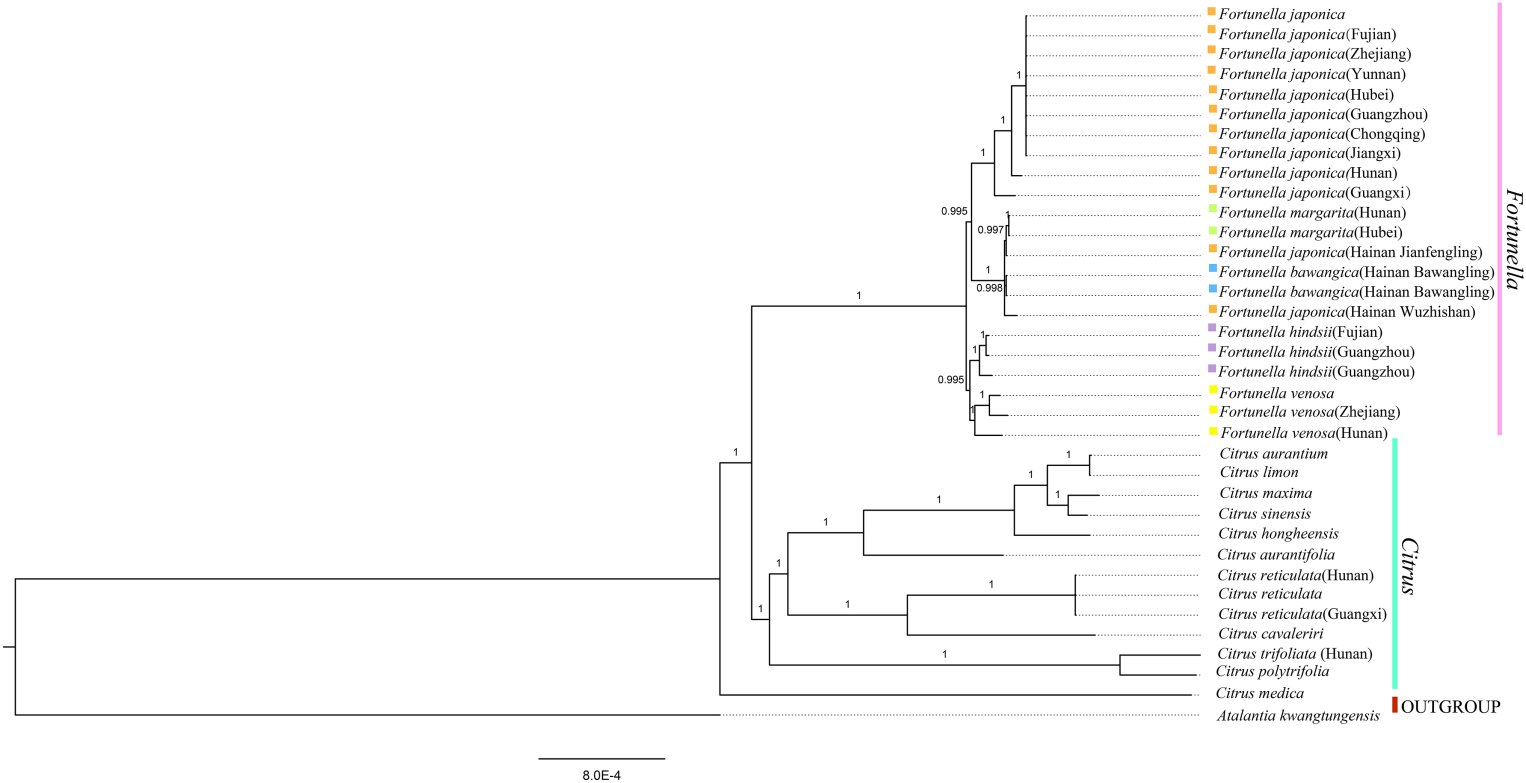
Phylogenetic trees based on BI of Rutaceae species based on whole plastid genome sequences, with one species *Atalantia kwangtungensis* used as outgroup. The Bayesian inference (BI) tree with posterior probabilities values on the branches.

**Figure 7:**
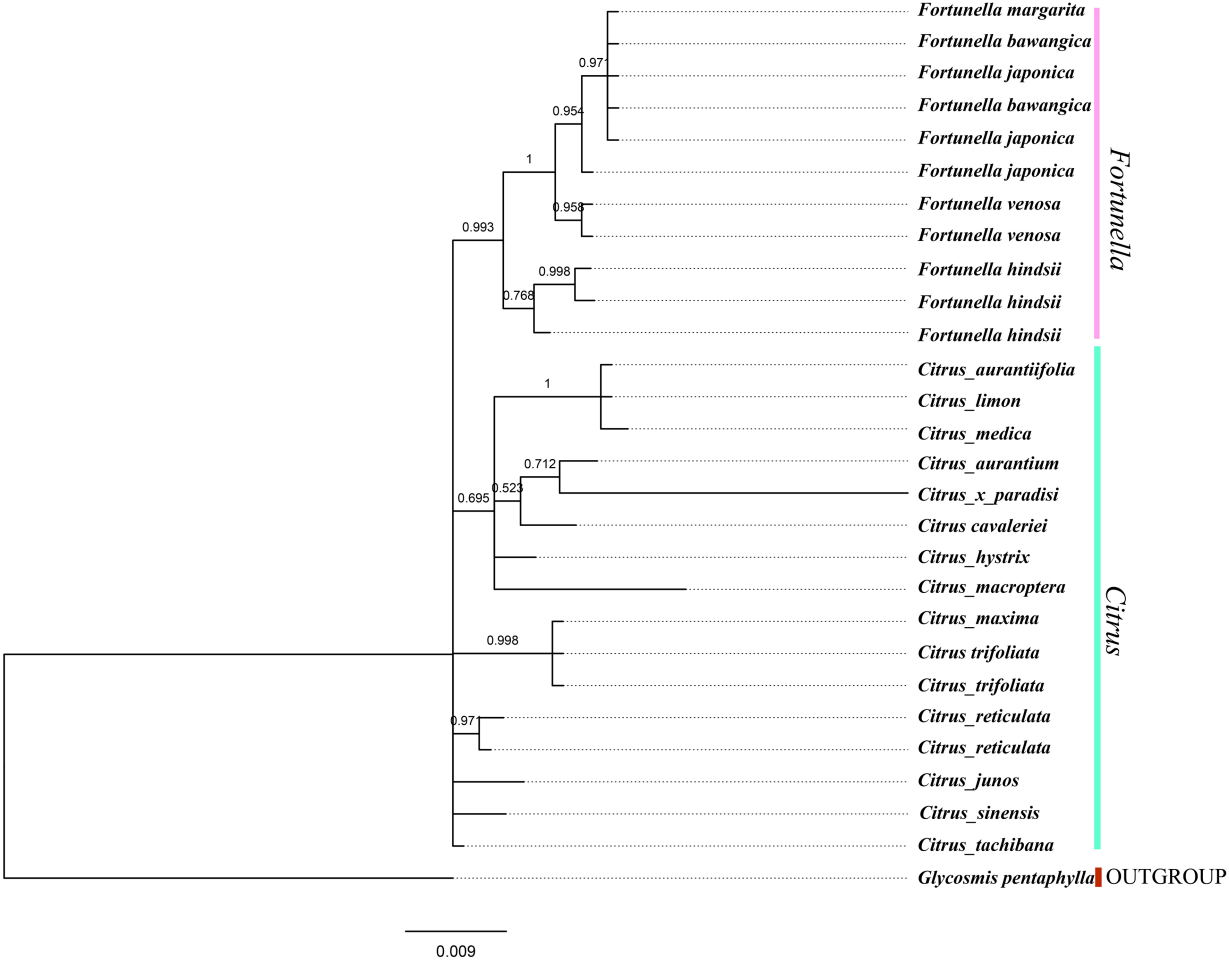
ITS fragment tree, phylogenetic tree inferred from Bayesian analysis, with one species *Glycosmis pentaphylla* used as outgroup. Values above the branches are Bayesian posterior probabilities.

Supplementary Table S4, S5 contain matrix information for plastid genomes and ITS. DnaSP v5 analysis shows that the plastid genome has 2,889 variable (polymorphic) sites, 1847 Singleton variable sites, and 1,042 informative sites. The nucleotide polymorphism PI is 0.00227. In general, from the results of the phylogenetic tree, *Fortunella* species clustered into a monophyletic group (Figures 5–7). In the phylogenetic tree, *Fortunella* contained 3 branches and a total of 10 nodes and all nodes had strong bootstrap support (BS) of 99–100%. Among them, there is a branch of *F. venosa*, a branch of *F. hindsii*, a branch of *F. japonica*, *F. margarita*, and *F. bawangica*. These results strongly support the classification of *F. venosa* and *F. hindsii* as independent species. The phylogenetic tree also showed that *Fortunella* and *Citrus* are closely related with high bootstrap support (100%). This result had the same topology as the data validated by phylogenetic methods based on whole plastid DNA markers and ITS. The bootstrap support values varied slightly for each tree topology. The topological structure of CDS is basically the same as that of the plastid genome tree, but it is quite different from the topological structure of the ITS tree, especially the systematic location of *Citrus* species, which may be caused by the inconsistency of the topological structure of the tree caused by low taxon sampling.

### Taxonomic Treatment

New combinations of two independent species of Rutaceae.

According to a previous phylogenetic study, the traditional *Fortunella* was merged into *Citrus* s.l (Zhang et al., 2008). Within *Citrus*, *F. japonica*, *F. venosa*, and *F. hindsii* are currently retained as synonyms of *C. japonica*. However, this study showed that *Citrus* and *Fortunella* should be treated as one genus and that *F. venosa* and *F. hindsii* had significant differences morphologically and phylogenetically, hence the two should be treated as independent species. Therefore, two new combinations of *F. venosa* and *F. hindsii* are proposed in this paper.

#### *Citrus venosa* (Champ. ex Benth.) K. M. Liu, X. Z. Cai and G. W. Hu, *comb. nov*.

*Sclerostylis venosa* Champ. ex Benth. in Hook. Journ. Bot. Kew Misc. 3: 327. 1851.; *Fortunella hindsii* var. *chintou* Swingle in Journ. Wash. Acad. Sci. 21: 130. 1940.; *Fortunella venosa* (Champ. ex Benth.) Huang (1991).

Type: —No type specimen of this species exists, specimen checked: Yunnan, Binchuan Xian, *S. Q. Ding 1,463* (noetype, designated here), (Herb. Yunnan Inst.Agricult., Sect. Horticu lt.).

#### *Citrus hindsii* (Champ. ex Benth.) K. M. Liu, G. W. Hu, and X. Z. Cai, *comb. nov*.

*Sclerostylis hindsii* Champ. ex Benth. in Hook. Journ. Bot. Kew Mise. 3: 327.1851; *Atalanthia hindsii* Oliv. ex Benth. Fl. Hongk. 51.1861; *Fortunella hindsii* (Champ. ex Benth.) Swingle in Journ. Wash. Acad. Sci. 5: 175. 1915.

Type: —CHINA: Hongkong, 1841, *R. B. Hinds, s.n.*, (lectotype: K[K000717994] image! designated by Swingle in 1861).

## Discussion

### The Key Basis of the Classification of the *Fortunella* Group

The taxonomy focuses only on ovary locules, ovule was existed controversial (Huang, 1997; Zhang et al., 2008); hence, we are changing the narrative. After reviewing numerous specimens and field investigations, we found that the leaf shape, petiole length, and fruit size of *Fortunella* species were fairly stable within and between populations (Although the fruit size of *F. margarita* and some hybrids varied greatly under cultivation conditions, our discussion here is mainly aimed at wild species), indicating that these traits are the key characteristics of the *Fortunella* group. The leaves of seed plants evolved from the original branch system (Efroni et al., 2010). Leaf types, such as simple leaf and compound leaf, are an important part of plant morphological characters and are also the basis of traditional and modern classification. Simple and compound leaves may represent different stages or processes of evolution. Efroni et al. (2010) pointed out that, based on a large amount of genetic evidence, the ontogeny and morphology of simple and compound leaves are determined by different genetic materials, which have common potential in different environments. Fruit size is a common basis for many taxonomists (Pojarkova, 1971; Song and Hong, 2021). Combined with field investigations, we found that the fruit size of wild *Fortunella* species is a very stable trait, so fruit size was used as the key evidence. After comparing and analyzing the morphological characteristics of *Fortunella*, it is suggested that *F. venosa* and *F. hindsii* with smaller fruits (6–8 mm in diameter) should be separated from *F. japonica* with significantly larger fruits (1.6–2.5 cm in diameter). Separate the two different species of a single leaf, with petiole length1-3(−5) mm (*F. venosa*), and a single compound leaf, with petiole length 6–9 mm (*F. hindsii*). According to the general law of morphological evolution (Efroni et al., 2010; Meyer and Purugganan, 2013; J S (Pat) Heslop-Harrison, 2017), especially the evolutionary relationship from simple leaf to compound leaf (Efroni et al., 2010), there is no doubt that *C. venosa* is the original species of this group. It is simple leaves, ovary 2–4 locules, stamens 10–15, small fruit, and so on, are the original characteristics of the group. *C. japonica* belongs to the evolutional type, single compound leaf (simple leaflet), ovary 4–6 locules, stamens (15-) 20–25, large fruit, etc. which are the secondary characteristics of this group. *C. hindsii* belongs to the transitional type, which is characterized by simple leaflets, which are rare and a simple leaf. Here we emphasize the taxonomic value of leaf type and fruit size in this group, hoping to further understand their relationship by discussing the morphological boundaries and differentiation between different species.

According to Erdtman’s standard of pollen size classification, pollen grains in *Fortunella* are all small and medium-sized (Erdtman, 1969). The pollen morphology and data analysis showed that there are significant differences in pollen size, germination groove, polar and equatorial axis, etc., which support independent species. For example, the number of germination grooves: *F. japonica* 4 and 5 grooves, *F. venosa* 4 and 5 grooves, *F. hindsii* 4 grooves. The pollen morphological characteristics of *Fortunella* can be used as the basis for its interspecific classification, which is consistent with previous studies (Zhang et al., 2003; Cheng et al., 2016). However, most researchers only use SEM to observe the external morphology of pollen, rarely studying the internal structure of the pollen (Hu et al., 2021; Xu et al., 2021), thus important information critical for classification may be lost, making the results deviate from those obtained through other methods.

The present phylogenetic analysis has greatly deepened the understanding of the evolutionary relationship between *Fortunella* and *Citrus*. The phylogenetic results are consistent with the research of previous studies (Araújo et al., 2003; Wu et al., 2018a; Xu et al., 2019; Wang et al., 2021). Although our results clarify the phylogenetic relationship between *Fortunella* and *Citrus*, *Citrus* remains a complex group, hence further taxon sampling is necessary to fully understand the phylogenetic relationships in this group and the related genera. The availability of the plastid genome provides a powerful genetic resource for molecular phylogenetic studies of the wild plant resources, and with the advent of more plastid full genome sequences, the plastid genome is also expected to play a role in helping to resolve deeper phylogenetic relationships.

### About the System Status of the *Fortunella* Group and Related Classification Issues

Based on morphological and molecular biological data, the authors consider that *F. venosa* and *F. hindsii* are independent species; *F. margarita* and *F. bawangica* are very similar to *F. japonica*, there is no essential difference, they belong to the same species. Therefore, the author classified the traditional *Fortunella* into three independent species:

#### *Citrus venosa* (Champ. ex Benth.) K. M. Liu, X. Z. Cai, and G. W. Hu, *comb. nov.*

*Sclerostylis venosa* Champ. ex Benth. in Hook. Journ. Bot. Kew Misc. 3: 327. 1851; *Fortunella hindsii* var. *chintou* Swingle in Journ. Wash. Acad. Sci. 21: 130. 1940. *Fortunella venosa* (Champ. ex Benth.) Huang (1991).

A small shrub, usually 0.25–1 m tall, Leaves simple with a very short petiole, usually 1–3 mm long, Ovary 2-4-loculate. Fruit small, 6–8 mm in diameter. This species is significantly different from other species of this group. It is easy to be recognized in the field and can be regarded as a “good species” in classification. So far, many scholars have identified it as an independent species (Wang et al., 2021).^5^

**Figure 8:**
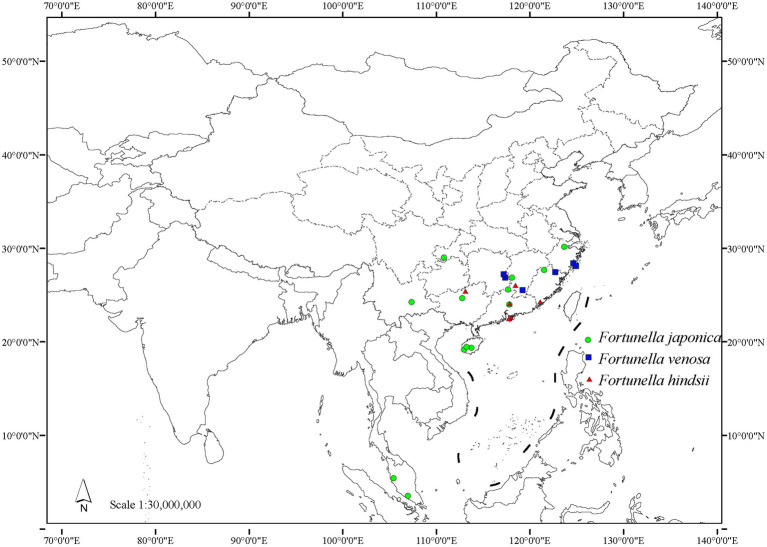
Distribution map of traditional *Fortunella*.

Distribution and habitat: endemic to China, distributed in Hunan, Jiangxi, Fujian, and Zhejiang (Figure 8). Most of them occur in steep hillsides and ditches of the evergreen broad-leaved secondary forest at an altitude of 200–500 m and sometimes form small communities.

#### *Citrus hindsii* (Champ. ex Benth.) K. M. Liu, G. W. Hu and X. Z. cai, *Comb. nov*.

*Sclerostylis venosa* Champ. ex Benth. ex Benth.in Hook. Journ. Bot. Kew Misc. 3: 327. 1851; *Atalanthia hindsii* Oliv. ex Benth. Fl. Hongk. 51.1861. *Fortunella hindsii* (Champ. ex Benth.) Swingle in Journ. Wash. Acad. Sci. 5: 175. 1915.

A shrub, usually 1–3 m tall. Leaves 1-foliolate, with a joint between petiole and blade, simple leaves extremely rare, petiole 6–9 mm long. Ovary 3–4 loculate. Fruit small, 8–10 mm in diameter. This species is significantly different from other species in this group and easy to identify. The List of National Key Protected Wild Plants (Xu et al., 2019; 164,754,443,253,634.html),^6^ and other scholars (Zhu et al., 2019) also recognized it as an independent species.

Distribution and habitat: distributed in Hong Kong, Guangdong, Guangxi, Fujian, and Jiangxi provinces in China (Figure 8). It is mostly found in evergreen broad-leaved secondary forest hillside sparse forests in the mountains of southern central subtropics of China, at an altitude of 150–650 m.

#### *Citrus japonica* Thunb.

Nova Acta Regiae Soc. Sci. Upsal. 3: 208. 1780. *F. japonica* (Thunb.) Swingle; *F. margarita* (Thunb.) Swingle; *F. crassifolia* Swingle.; *F. obovata* Tanaka; *F. bawangica* C.C.Huang; *F. swinglei* Tanaka.

Small trees or shrubs, usually 2–9 m tall, trunk usually slender. Leaves 1-foliolate, with a joint between petiole and blade, petiole length 6–10 mm. Ovary 4–6 loculate. Fruit (16-) 20–25 mm in diameter. This species is significantly different from other species in this group and is easy to be recognized in the field. For the taxonomic status of this species, almost all scholars have recognized it as an independent species (Swingle, 1915; Zhang et al., 2008), but it should be pointed out that although Flora of China (Zhang et al., 2008) also uses the same Chinese name and scientific name, its Synonyms include *F. venosa* and *F. hindsii* in this regard, the author does not agree with the latter treatment.

#### Distribution and Habitat

Mainly distributed in China, also in Malaysia, and cultivated in Japan, Australia, Cuba, Dominican Republic, Gambia, Haiti, Japan, South Korea, etc. In China, it is distributed in Hainan, Guangdong, Guangxi, Yunnan, Hunan, Jiangxi, Fujian, Zhejiang, Chongqing, etc. (Figure 8). Born in the steep hillside forests of evergreen broad-leaved forests in the southern subtropics and central subtropics, at an altitude of 465–1,300 m.

## Key to the Species of Traditional *Fortunella*

1. Small trees or shrubs, 2-9 m tall, trunk usually slender; Single compound leaf; Fruit large, 16-25 mm in diameter. ldots 1. *Citrus japonica*

+ Shrubs or small shrubs, 0.25–3 m high; Single leaf or single compound leaf; small fruit, 6–10 mm in diameter. ldots (2)

2. Simple leaf; Petiole very short, usually 1–3 mm long; Small shrubs, usually 0.25- 1 m tall. ldots 2. *C. venosa*

+ Single compound leaves, very rare and simple; petiole longer, 6–9 mm long; shrubs, usually 1–3 m tall. ldots 3. *C. hindsii.*

### Status and Taxonomic Treatment of *F. swinglei* and *F. bawangica* C. C. Huang

*F. swinglei* also known as Ma la ya jin gan, is a new species published by Tanaka in Société Bot. Fr in commemorating of swinglei. It is native to Malaysia and is a Malaysian cultivar (Tanaka, 1928). We have carefully studied its original description, that is, stem without thorns; large evergreen shrubs 1–2 m tall or small trees 3–5 m tall; fruit diameter 1.5–3 cm; single compound leaf. Therefore, we believe that there is no essential difference between it and *C. japonica* (its stem without thorns is a cultivated variation). Agree with Lim (2012) about being a synonym for *C. japonica*.

*F. bawangica* is a new species of *Fortunella*, which was reported by Huang (1991) based on specimens collected from Bawangling, Changjiang County, Hainan Province. To determine the taxonomic identity of this name, the authors carried out field investigations on the origin of its type specimens from 28 August to 30 August 2020, and 28 May 28 to 3 June 2021, respectively, and successfully collected specimens. The results showed that the petiole length, which was used as the main basis for distinguishing *C. japonica* and other species in Flora Reipublicae Popularis Sinicae, was a rather unstable character in populations, especially in its germinating branches, which could not be used as the basis for its species selection. Its immature fruit is pear-shaped, that is, the base is narrow and elongated into a short stalk shape (Huang, 1997; Jiang et al., 2009), which can also be seen in *C. japonica* and other plants of this group. According to the author’s field investigation, *F. bawangica* is only distributed in the Bawangling area of Changjiang County, Hainan, China, while *C. japonica* is distributed in Guangdong, Fujian, Zhejiang, Chongqing, Yunnan, Hunan, Jiangxi, and other places (sporadic distribution), but Hainan is the most concentrated and main distribution area. Wild individuals or populations of this species exist in Yaxian, Dongfang, Qiongzhong, Chengmai, Ledong, and Wuzhishan in Hainan, especially in the mountain evergreen broad-leaved forests of Jianfengling and Bawangling in Hainan. The distribution areas of *F. bawangica* and *F. japonica* are highly overlapped in the Bawangling area of Changjiang County, Hainan Province, so we suspect that there may be natural hybridization. According to the molecular results of this study, the traditional *Fortunella*, *F. japonica*, *F. bawangica*, and *F. margarita* were clustered together with a high bootstrap support rate, which indicated that *F. bawangica* and *F. japonica* were closely related. Based on the analysis of morphological characters, the author believes that *F. bawangica* is probably a hybrid type related to *F. japonica*. Accordingly, it is treated as a synonym of *C. japonica* in this paper.

## Conclusion

This study analyzed and compared the characteristics of traditional *Fortunella* in morphology, anatomy, palynology, and molecular biology. The results showed that *Fortunella* and *Citrus* were closely related and overlapped in morphology, and could not be completely separated from each other in molecular systematics, hence this study supports the incorporation of *Fortunella* into *Citrus*. *F. venosa*, *F. hindsii*, and *F. japonica* have morphological discontinuity and differences and should be regarded as independent species. *C. venosa* is the original species of *Fortunella* and an important genetic resource of *Citrus* fruit. The establishment of the taxonomic status of *C. venosa* and *C. hindsii* is of great significance for the global genetic diversity assessment, phylogeny, and population genetics of *Fortunella*.

## Data Availability Statement

The datasets presented in this study can be found in online repositories. The names of the repository/repositories and accession number(s) can be found in the article/Supplementary Material.

## Author Contributions

The field survey was completed by TW, L-LC, K-ML, FY, X-LL, H-JS, JL, and JR. TW and X-ZC conducted data analysis. The drafting of manuscripts and diagrams were prepared by TW, L-LC, X-ZC, G-WH, and K-ML. The revision and manuscript editing were completed by TW, VW, FM, FY, X-ZC, and G-WH. The proofreading of the English manuscript was completed by TW, L-LC, X-ZC, and G-WH. Resource acquisition and funding were provided by G-WH, X-ZC, and K-ML. All authors contributed to the article and approved the submitted version.

## Funding

This study was supported by National Science and Technology Fundamental Resources Investigation Program of China (grant Nos. 2019FY101800 and 2019FY101812); Investigation of Forest Tree Germplasm Resources in Hunan Province [Xiangcainongzhi (2015) 91]; Compilation of Rural Biosafety Planning in Hunan Province [XiangcaiZihuanzhi (2020) No. 41].

## Conflict of Interest

The authors declare that the research was conducted in the absence of any commercial or financial relationships that could be construed as a potential conflict of interest.

The Reviewer W-BY declared a shared parent affiliation with JR, VW, and FM at the time of the review.

## Publisher’s Note

All claims expressed in this article are solely those of the authors and do not necessarily represent those of their affiliated organizations, or those of the publisher, the editors and the reviewers. Any product that may be evaluated in this article, or claim that may be made by its manufacturer, is not guaranteed or endorsed by the publisher.

## Footnotes

^1^ http://www.forestry.gov.cn/main/3951/20210908/164754

^2^ http://www.mobot.org/MOBOT/research/APweb/

^3^ http://www.forestry.gov.cn/main/3951/20210908/164754443253634.html

^4^ http://www.forestry.gov.cn/main/3951/20210908/16475

^5^ http://www.forestry.gov.cn/main/3951/20210908/164754443253634.html

^6^ http://www.forestry.gov.cn/main/3951/20210908/
