# Supplementary material for: Fortunella venosa (Champ. ex Benth.) C. C. Huang and F. hindsii (Champ. ex Benth.) Swingle as Independent Species: Evidence From Morphology and Molecular Systematics and Taxonomic Revision of Fortunella (Rutaceae)
Source: Front Plant Sci. 2022 May 12;13:867659. doi: 10.3389/fpls.2022.867659 (PMC9133918; doi:10.3389/fpls.2022.867659)
Supplement: Supplementary file 3 [file Table_3.DOCX]

The amplification procedure is as follows:

(1) Pre-denaturation at 95°C 4 min

(2) Denaturation at 95°C 30 s

(3) Annealing at 55℃ 30 s 33 cycles

(4) extension 72℃ 30 s

(5) extension 72℃ 5 min

The PCR amplification system is 25 μl, and its composition is as follows:

| Total | 25 μl |
| --- | --- |
| reverse primer ITS4 | 1.0 μl |
| Forward primer ITS5 | 1.0 μl |
| Mix | 12.0 μl |
| H_2_o | 12.0 μl |
| DNA template | 1.0 μl |
