# Supplementary material for: Fortunella venosa (Champ. ex Benth.) C. C. Huang and F. hindsii (Champ. ex Benth.) Swingle as Independent Species: Evidence From Morphology and Molecular Systematics and Taxonomic Revision of Fortunella (Rutaceae)
Source: Front Plant Sci. 2022 May 12;13:867659. doi: 10.3389/fpls.2022.867659 (PMC9133918; doi:10.3389/fpls.2022.867659)
Supplement: Supplementary file 5 [file Table_5.DOCX]

10 20 30 40 50 60

....|....|....|....|....|....|....|....|....|....|....|....|

**Citrus_reticulata_1**  **CCAGTTGATATCACCGGCGGCGGGAGGGGGTGC-GCGTCCGCAACGGGCGCTCCTCCTTC**

**Citrus_trifoliata**  **CCAGTTGATGTCACCGGCGGCGGGAGGGGGTGC-GCGTCCGCAGCGGGCGCTCCTCCTTC**

**Fortunella_bawangica_1** **CCAGTTGATATCACCGGCGGCGGGAGGGGGTAC-GCGTCCGCAACGGGCGCTCCTCCTTC**

**Fortunella_bawangica_2** **CCAGTTGATATCACCGGCGGCGGGAGGGGGTGC-GCGTCCGCAACGGGCGCTCCTCCTTC**

**Fortunella_hindsii_1**  **CCAGTTGATATCACCGGCGGCGGGAGGGGGTGC-GCGTCCGCAACGGGCGCTCCTCCTTC**

**Fortunella_hindsii_2**  **CCAGTTGATATCACCGGCGGCGGGAGGGGGTGC-GCGTCCGCAACGGGCGCTCCTCCTTC**

**Fortunella_hindsii_3**  **CCAGTTGATATCACCGGCGGCGGGAGGGGGTGC-GCGTCCGCAACGGGCGCTCCTCCTTC**

**Fortunella_japonica_1**  **CCAGTTGATATCACCGGCGGCGGGAGGGGGTGC-GCGTCCGCAACGGGCGCTCCTCCTTC**

**Fortunella_japonica_2**  **CCAGTTGATATCACCGGCGGCGGGAGGGGGTGC-GCGTCCGCAACGGGCGCTCCTCCTTC**

**Fortunella_japonica_3**  **CCAGTTGATATCACCGGCGGCGGGAGGGGGTGC-GCGTCCGCAACGGGCGCTCCTCCTTC**

**Fortunella_margarita_2** **CCAGTTGATATCACCGGCGGCGGGAGGGGGTGC-GCGTCCGCAACGGGCGCTCCTCCTTC**

**Fortunella_venosa_1**  **CCTGTTGATATCACCGGCGGCGGGAGGGGGTAC-GCGTCCGCAACGGGCGCTCCTCCTTC**

**Fortunella_venosa_2**  **CCTGTTGATATCACCGGCGGCGGGAGGGGGTAC-GCGTCCGCAACGGGCGCTCCTCCTTC**

**Citrus_aurantiifolia**  **---------ATCACCGGCGGCGGGAGGGGGGA--GCGTAAACAGAGGGGGCTCCTCCTTC**

**Citrus_aurantium**  **--------------------------------------------CGGGCGCTCCTCCTTC**

**Citrus_hystrix**  **CCAGTTGATATCACCGGCGGCGGGAGGGGGGAT-GCGTCCGCAGCGGGCGCTCCTCCTTC**

**Citrus_cavaleriei**  **CCAGTTGATATCACCGGCGGCGGGAGGGGGGAC-GCGTCCGCAGCGGGCGCTCCTCCTTC**

**Citrus_junos**  **CCAGTTGATATCACCGGCGGCGGGAGGGGGTGC-GCGTCCGCAACGGGCGCTCCTCCTTC**

**Citrus_limon**  **CCAGTTGATATCACCGGCGGCGGGAGGGGGGAT-GCGTCCGCAGCGGGCGCTCCTCCTTC**

**Citrus_tachibana**  **CYAGTTGATATCACCGGCGGCGGGAGGGGGKGC-GCGTCCGCAACKGGCGCTCCTCCTTC**

**Glycosmis_pentaphylla**  **CCAGTTAGAACCACCGGCCGCGGGAGGGGGGGGAGCGCTCCTAGCGTGCGCCCCCGCCTC**

**Poncirus_trifoliata**  **CCAGTTGATGTCACCGGCGGCGGGAGGGGGTGC-GCGTCCGCAGCGGGCGCTCCTCCTTC**

**Citrus_sinensis**  **CCAGTTGATATCACCGGCGGCGGGAGGGGGGGC-GCGTCCGCAACGGGCGCTCCTCCTTC**

**Citrus_reticulata**  **CCAGTTGATATCACCGGCGGCGGGAGGGGGTGC-GCATCCGCAACGGGCGCTCCTCCTTC**

**Citrus_x_paradisi**  **----------TCACCGGCGGCGGGAGGGGGGGC-GCGTCCGCAACGGGCGCTCCTCCTTC**

**Citrus_maxima**  **CCAGTTGATGTCACCGGCGGCGGGAGGGGGTGC-GCGTCCGCAGCGGGCGCTCCTCCTTC**

**Citrus_medica**  **---------------------------------------------------------TTC**

**Citrus_macroptera**  **CCAGTTGATATCACTGGCGTCGGGAGGGGGGAT-GCGTCCGCAGCGGGCGCTCCTCCTTC**

70 80 90 100 110 120

....|....|....|....|....|....|....|....|....|....|....|....|

**Citrus_reticulata_1**  **CCGCCCC-ATGCC-GCGGGGAGAGGGACTCGTCCCGCTCCCGGCTGGCGAAACAACGAAC**

**Citrus_trifoliata**  **CCGCCCC-ACGCC-GCGGGGAGAGGGACTCGTCCCGCTCCCGGCCGGCGAAACAACGAAC**

**Fortunella_bawangica_1** **CCGCCCC-ACGCC-ACGGGGAGAGGGACTCATCCCGCTCCCGGCTGGCGAAACAACGAAC**

**Fortunella_bawangica_2** **CCGCCCC-ACGCC-ACGGGGAGAGGGACTCATCCCGCTCCCGGCTGGCGAAACAACGAAC**

**Fortunella_hindsii_1**  **CCGCCCC-ACGCC-GCGGGGAGAGGGACTCGTCCCGCTCCCGGCTGGCGAAACAACGAAC**

**Fortunella_hindsii_2**  **CCGCCCC-ACGCC-GCGGGGAGAGGGACTCGTCCCGCTCCCGGCTGGCGAAACAACGAAC**

**Fortunella_hindsii_3**  **CCGCCCC-ACGCC-GCGGGGAGAGGGACTCGTCCCGCTCCCGGCTGGCGAAACAACGAAC**

**Fortunella_japonica_1**  **CCGCCCC-ACGCC-ACGGGGAGAGGGACTCATCCCGCTCCCGGCTGGCGAAACAACGAAC**

**Fortunella_japonica_2**  **CCGCCCC-ACGCC-ACGGGGAGAGGGACTCATCCCGCTCCCGGCTGGCGAAACAACGAAC**

**Fortunella_japonica_3**  **CCGCCCC-ACGCC-ACGGGGAGAGGGACTCATCCCGCTCCCGGCTGGCGAAACAACGAAC**

**Fortunella_margarita_2** **CCGCCCC-ACGCC-ACGGGGAGAGGGACTCATCCCGCTCCCGGCTGGCGAAACAACGAAC**

**Fortunella_venosa_1**  **CCGCCCC-ACGCC-ACGGGGAGAGGGACTCATCCCGCTCCCGGCTGGCGAAACAACGAAC**

**Fortunella_venosa_2**  **CCGCCCC-ACGCC-ACGGGGAGAGGGACTCATCCCGCTCCCGGCTGGCGAAACAACGAAC**

**Citrus_aurantiifolia**  **CCGCCCC-ACGCC-GCGGGGAGAGGGACTCGTTCTGCTCCCGGCTGGCGAAACAACAAAC**

**Citrus_aurantium**  **CCGCCCC-ACGCC-GCGGGGAGAGGGACTCGTCCCGCTCCTGGCTGGCGAAACAACGAAC**

**Citrus_hystrix**  **TCGCCCC-ACGCC-GCGGGGAGAGGGACTCGTCCCGCTCCCGGCTGGCGAAACAACGAAC**

**Citrus_cavaleriei**  **CCGCCCCCACGCC-GCGGGGAGAGGGACTCGTCCCGCTCCCGGCTGGCGAAACAACGAAC**

**Citrus_junos**  **CCGCCCC-ATGCC-GCGGGGAGAGGGACTCGTCCCGCTCCCGGCTGGCGAAACAACGAAC**

**Citrus_limon**  **CCGCCCC-ACGCC-GCGGGGAGAGGGACTCGTTCTGCTCCCGGCTGGCGAAACAACAAAC**

**Citrus_tachibana**  **CCGCCCC-AYGCC-GCGGGGAGAGGGACTCGTCCCGCTCCYGGCTGGCGAAACAACGAAC**

**Glycosmis_pentaphylla**  **CCGCGCC-CCGCC-TCGGGGAGGGGGACTCGTCCCTCTCCCCGC-GGCGGAACAACGAAC**

**Poncirus_trifoliata**  **CCGCCCC-ACGCC-GCGGGGAGAGGGACTCGTCCCGCTCCCGGCCGGCGAAACAACGAAC**

**Citrus_sinensis**  **CCGCCCC-ATGCC-GCGGGGAGAGGGACTCGTCCCGCTCCCGGCTGGCGAAACAACGAAC**

**Citrus_reticulata**  **CCGCCCC-ATGCC-GCGGGGAGAGGGACTCGTCCCGCTCCCGGCTGGCGAAACAACGAAC**

**Citrus_x_paradisi**  **CCGCCCC-ACGCC-GCGGGGAGAGGGACTCGTCCCGCTCCTGGCTGGCAAAACAACGAAC**

**Citrus_maxima**  **CCGCCCC-ACGCC-GCGGGGAGAGGGACTCGTCCCGCTCCCGGCCGGCGAAACAACGAAC**

**Citrus_medica**  **CCGCCCC-ACGCCCGCGGGGAGAGGGACTCGTTCTGCTCCCGGCTGGCGAAACAACAAAC**

**Citrus_macroptera**  **CCGCCCC-ACGCC-GCGGGGAGAGGGACTCGTCCCGCTCCCGGCTGGCGAAACAACGAAC**

130 140 150 160 170 180

....|....|....|....|....|....|....|....|....|....|....|....|

**Citrus_reticulata_1**  **CCCCGGCGCGGACTGCGCCA-AGGAAATCTAACGAGAGAGCACGCTCCCGCGGCCCCG--**

**Citrus_trifoliata**  **CCCCGGCGCGGACCGCGCCA-AGGAAATCTAACGAGAGAGCACGCTCCCGCGGCCCCG--**

**Fortunella_bawangica_1** **CCCCGGCGCGGACTGCGCCA-AGGAAATTTAACGAGAGAGCACGCTCCCGCGGCCCCG--**

**Fortunella_bawangica_2** **CCCCGGCGCGGACTGCGCCA-AGGAAATTTAACGAGAGAGCACGCTCCCGCGGCCCCG--**

**Fortunella_hindsii_1**  **CCCCGGCGCGGACTGCGCCA-AGGAAATCTAACGAGAGAGCACGCTACCGCGGCCCCG--**

**Fortunella_hindsii_2**  **CCCCGGCGCGGACTGCGCCA-AGGAAATCTAACGAGAGAGCACGCTCCCGCGGCCCCG--**

**Fortunella_hindsii_3**  **CCCCGGCGCGGACTGCGCCA-AGGAAATCTAACGAGAGAGCACGCTACCGCGGCCCCG--**

**Fortunella_japonica_1**  **CCCCGGCGCGGACTGCGCCA-AGGAAATTTAACGAGAGAGCACGCTCCCGCGGCCCCG--**

**Fortunella_japonica_2**  **CCCCGGCGCGGACTGCGCCA-AGGAAATTTAACGAGAGAGCACGCTCCCGCGGCCCCG--**

**Fortunella_japonica_3**  **CCCCGGCGCGGACTGCGCCA-AGGAAATTTAACGAGAGAGCACGCTCCCGCGGCCCCG--**

**Fortunella_margarita_2** **CCCCGGCGCGGACTGCGCCA-AGGAAATTTAACGAGAGAGCACGCTCCCGCGGCCCCG--**

**Fortunella_venosa_1**  **CCCCGGCGCGGACTGCGCCA-AGGAAATCTAACGAGAGAGCACGCTCCCGCGGCCCCG--**

**Fortunella_venosa_2**  **CCCCGGCGCGGACTGCGCCA-AGGAAATCTAACGAGAGAGCACGCTCCCGCGGCCCCG--**

**Citrus_aurantiifolia**  **CCCCGGCGCGGACTGCGCCA-AGGAAATCTAACGAGAGAGCACGCTCCCGCGGC------**

**Citrus_aurantium**  **CCCCGGCGCGGACTGCGCCA-AGGAAATCTAACGAGAGAGCACGCTCCCGCGGCCCCGGA**

**Citrus_hystrix**  **CCCCGGCGCGGACTGCGCCA-AGGAAATCTAACGAGAGAGCACGCTCCCGCGGCCCCG--**

**Citrus_cavaleriei**  **CCCCGGCGCGGACTGCGCCA-AGGAAATCTAACGAGAGAGCATGCTCCTGCGGCCCCG--**

**Citrus_junos**  **CCCCGGCGCGGACTGCGCCA-AGGAAATCTAACGAGAGAGCACGCTCCTGCGGCCCCG--**

**Citrus_limon**  **CCCCGGCGCGGACTGCGCCACAGGAAATCTAACGAGAGAGCACGCTCCCGCGGC------**

**Citrus_tachibana**  **CCCCGGCGCGGACTGCGCCA-AGGAAATCTAACGAGAGAGCACGCTCCCGCGGCCCCG--**

**Glycosmis_pentaphylla**  **CCCCGGCGCGGACCGCGCCA-AGGAAATCCAACGGGAGAGCGCGCTCCCGCGGCCCCG--**

**Poncirus_trifoliata**  **CCCCGGCGCGGACCGCGCCA-AGGAAATCTAACGAGAGAGCACGCTCCCGCGGCCCCG--**

**Citrus_sinensis**  **CCCCGGCGCGGACTGCGCCA-AGGAAATCTAACGAGAGAGCACGCTCCCGCGGCCCCG--**

**Citrus_reticulata**  **CCCCGGCGCGGACTGCGCCA-AGGAAATCTAACGAGAGAGCACGCTCCCGCGGCCCCG--**

**Citrus_x_paradisi**  **CCCCGGCGCGGACTGCGCCA-AGGAAATCTAACGAGAGAGCACGCTCCCGCGGCCCCGGA**

**Citrus_maxima**  **CCCCGGCGCGGACCGCGCCA-AGGAAATCTAACGAGAGAGCACGCTCCCGCGGCCCCG--**

**Citrus_medica**  **CCCCGGCGCGGACTGCGCCA-AGGAAATCTAACGAGAGAGCACGCTCCCGCGGC------**

**Citrus_macroptera**  **CCCCGGCGCGGACTGCGCCA-AGGAAATCTAACGAGAGAGCACGCTCCTGCGGCCCCCCC**

190 200 210 220 230 240

....|....|....|....|....|....|....|....|....|....|....|....|

**Citrus_reticulata_1**  **GAGACGGTGCGCCGCGGGGTGCGGCGCCTTCTTTCACATGT-ATCCAAAACGACTCTCGG**

**Citrus_trifoliata**  **GAGACGGTGCGCCGCGGGGTGCGGCGCCTTCTTTCACATGT-ATCCAAAACGACTCTCGG**

**Fortunella_bawangica_1** **GAGACGGTGCGCCGCGGGGTGCGGCGCCTTCTTTCACATGT-ATCCAAAACGACTCTCGG**

**Fortunella_bawangica_2** **GAGACGGTGCGCCGCGGGGTGCGGCGCCTTCTTTCACATGT-ATCCAAAACGACTCTCGG**

**Fortunella_hindsii_1**  **GAGACGGTGCGCCGCGGGGTGCGGTGCCTTCTTTCACATGT-ATCCAAAACGACTCTCGG**

**Fortunella_hindsii_2**  **GAGACGGTGCGCCGCGGGGTGCGGCGCCTTCTTTCACATGT-ATCCAAAACGACTCTCGG**

**Fortunella_hindsii_3**  **GAGACGGTGCGCCGCGGGGTGCGGTGCCTTCTTTCACATGT-ATCCAAAACGACTCTCGG**

**Fortunella_japonica_1**  **GAGACGGTGCGCCGCGGGGTGCGGCGCCTTCTTTCACATGT-ATCCAAAACGACTCTCGG**

**Fortunella_japonica_2**  **GAGACGGTGCGCCGCGGGGTGCGGCGCCTTCTTTCACATGT-ATCCAAAACGACTCTCGG**

**Fortunella_japonica_3**  **GAGACGGTGCGCCGCGGGGTGCGGCGCCTTCTTTCACATGT-ATCCAAAACGACTCTCGG**

**Fortunella_margarita_2** **GAGACGGTGCGCCGCGGGGTGCGGCGCCTTCTTTCACATGT-ATCCAAAACGACTCTCGG**

**Fortunella_venosa_1**  **GAGACGGTGCGCCGCGGGGTGCGGCGCCTTCTTTCACATGTTATCCAAAACGACTCTCGG**

**Fortunella_venosa_2**  **GAGACGGTGCGCCGCGGGGTGCGGCGCCTTCTTTCACATGTTATCCAAAACGACTCTCGG**

**Citrus_aurantiifolia**  **-------------------------GCCTTCTTTCACATGT-ATCCAAAACGACTCTCGG**

**Citrus_aurantium**  **GACGGTGCGC-TGCGGGG-TGCGGTGCCTTCTTTCACATGT-ATCCAAAACGACTCTCGG**

**Citrus_hystrix**  **GAGACGGTGCGCCGCGGGGTGCGGCGCCTTCTTTCACATGC-ATCCAAAACGACTCTCGG**

**Citrus_cavaleriei**  **GAGACGGTGCGCCGCGGGGTGCGGCGCCTTCTTTCACATGT-ATCCAAAACGACTCTCGG**

**Citrus_junos**  **GAGACGGTGCGCCGCGGGGTGCGGCGCCTTCTTTCACATGT-ATCCAAAACGACTCTCGG**

**Citrus_limon**  **-------------------------GCCTTCTTTCACATGT-ATCCAAAACGACTCTCGG**

**Citrus_tachibana**  **GAGACGGTGCGCCGCGGGGYGCGGCGCCTTCTTTCACATGT-ATCCAAAACGACTCTCGG**

**Glycosmis_pentaphylla**  **GACACGGTGCGCCGCGGGACGCCGCGCCTTCTATTACTCGT-ATCCAAAACGACTCTCGG**

**Poncirus_trifoliata**  **GAGACGGTGCGCCGCGGGGTGCGGCGCCTTCTTTCACATGT-ATCCAAAACGACTCTCGG**

**Citrus_sinensis**  **GAGACGGTGCGCCGCGGGGTGCGGCGCCTTCTTTCACATGT-ATCCAAAACGACTCTCGG**

**Citrus_reticulata**  **GAGACGGTGCGCCGCGGGGTGCGGCGCCTTCTTTCACATGT-ATCCAAAACGACTCTCGG**

**Citrus_x_paradisi**  **GAGGGGGCGGGTGCGGGGGTGCGGCGCCTTCTTTCACATGT-ATCCAAAACGACTCTCGG**

**Citrus_maxima**  **GAGACGGTGCGCCGCGGGGTGCGGCGCCTTCTTTCACATGT-ATCCAAAACGACTCTCGG**

**Citrus_medica**  **-------------------------GCCTTCTTTCACATGT-ATCCAAAACGACTCTCGG**

**Citrus_macroptera**  **ACCCCGGTGCGCCGCGGGGTGCGGCGCCTTCTTTCACATGT-ATCTAAAATGACTCTCGG**

250 260 270 280 290 300

....|....|....|....|....|....|....|....|....|....|....|....|

**Citrus_reticulata_1**  **CAACGGATATCTCGGCTCTCGCATCGATGAAGAACGTAGCGAAATGCGATACTTGGTGTG**

**Citrus_trifoliata**  **CAACGGATATCTCGGCTCTCGCATCGATGAAGAACGTAGCGAAATGCGATACTTGGTGTG**

**Fortunella_bawangica_1** **CAACGGATATCTCGGCTCTCGCATCGATGAAGAACGTAGCGAAATGCGATACTTGGTGTG**

**Fortunella_bawangica_2** **CAACGGATATCTCGGCTCTCGCATCGATGAAGAACGTAGCGAAATGCGATACTTGGTGTG**

**Fortunella_hindsii_1**  **CAACGGATATCTCGGCTCTCGCATCGATGAAGAACGTAGCGAAATGCGATACTTGGTGTG**

**Fortunella_hindsii_2**  **CAACGGATATCTCGGCTCTCGCATCGATGAAGAACGTAGCGAAATGCGATACTTGGTGTG**

**Fortunella_hindsii_3**  **CAACGGATATCTCGGCTCTCGCATCGATGAAGAACGTAGCGAAATGCGATACTTGGTGTG**

**Fortunella_japonica_1**  **CAACGGATATCTCGGCTCTCGCATCGATGAAGAACGTAGCGAAATGCGATACTTGGTGTG**

**Fortunella_japonica_2**  **CAACGGATATCTCGGCTCTCGCATCGATGAAGAACGTAGCGAAATGCGATACTTGGTGTG**

**Fortunella_japonica_3**  **CAACGGATATCTCGGCTCTCGCATCGATGAAGAACGTAGCGAAATGCGATACTTGGTGTG**

**Fortunella_margarita_2** **CAACGGATATCTCGGCTCTCGCATCGATGAAGAACGTAGCGAAATGCGATACTTGGTGTG**

**Fortunella_venosa_1**  **CAACGGATATCTCGGCTCTCGCATCGATGAAGAACGTAGCGAAATGCGATACTTGGTGTG**

**Fortunella_venosa_2**  **CAACGGATATCTCGGCTCTCGCATCGATGAAGAACGTAGCGAAATGCGATACTTGGTGTG**

**Citrus_aurantiifolia**  **CAACGGATATCTCGGCTCTCGCATCGATGAAGAACGTAGCGAAATGCGATACTTGGTGTG**

**Citrus_aurantium**  **CAACGGATATCTCGGCTCTCGCATCGATGAAGAACGTAGCGAAATGCGATACTTGGTGTG**

**Citrus_hystrix**  **CAACGGATATCTCGGCTCTCGCATCGATGAAGAACGTAGCGAAATGCGATACTTGGTGTG**

**Citrus_cavaleriei**  **CAACGGATATCTCGGCTCTCGCATCGATGAAGAACGTAGCGAAATGCGATACTTGGTGTG**

**Citrus_junos**  **CAACGGATATCTCGGCTCTCGCATCGATGAAGAACGTAGCGAAATGCGATACTTGGTGTG**

**Citrus_limon**  **CAACGGATATCTCGGCTCTCGCATCGATGAAGAACGTAGCGAAATGCGATACTTGGTGTG**

**Citrus_tachibana**  **CAACGGATATCTCGGCTCTCGCATCGATGAAGAACGTAGCGRAATGCGATACTTGGTGTG**

**Glycosmis_pentaphylla**  **CAACGGATATCTCGGCTCTCGCATCGATGAAGAACGTAGCGAAATGCGATACTTGGTGTG**

**Poncirus_trifoliata**  **CAACGGATATCTCGGCTCTCGCATCGATGAAGAACGTAGCGAAATGCGATACTTGGTGTG**

**Citrus_sinensis**  **CAACGGATATCTCGGCTCTCGCATCGATGAAGAACGTAGCGAAATGCGATACTTGGTGTG**

**Citrus_reticulata**  **CAACGGATATCTCGGCTCTCGCATCGATGAAGAACGTAGCGAAATGCGATACTTGGTGTG**

**Citrus_x_paradisi**  **CAACGGATATCTCGGCTCTCGCATCGATGAAGAACGTATCGAAATGCGATACTTGGTGTG**

**Citrus_maxima**  **CAACGGATATCTCGGCTCTCGCATCGATGAAGAACGTAGCGAAATGCGATACTTGGTGTG**

**Citrus_medica**  **CAACGGATATCTCGGCTCTCGCATCGATGAAGAACGTAGCGAAATGCGATACTTGGTGTG**

**Citrus_macroptera**  **CAACGGATATCTCGGCTCTCGCATCGATGAAGAACGTAGCGAAATGCGATACTTGGTGTG**

310 320 330 340 350 360

....|....|....|....|....|....|....|....|....|....|....|....|

**Citrus_reticulata_1**  **AATTGCAGAATCCCGTGAACCATCGAGTCTTTGAACGCAAGTTGCGCCCCAAGCCATTAG**

**Citrus_trifoliata**  **AATTGCAGAATCCCGTGAACCATCGAGTCTTTGAACGCAAGTTGCGCCCCAAGCCATTAG**

**Fortunella_bawangica_1** **AATTGCAGAATCCCGTGAACCATCGAGTCTTTGAACGCAAGTTGCGCCCCAAGCCATTAG**

**Fortunella_bawangica_2** **AATTGCAGAATCCCGTGAACCATCGAGTCTTTGAACGCAAGTTGCGCCCCAAGCCATTAG**

**Fortunella_hindsii_1**  **AATTGCAGAATCCCGTGAACCATCGAGTCTTTGAACGCAAGTTGCGCCCCAAGCCATTAG**

**Fortunella_hindsii_2**  **AATTGCAGAATCCCGTGAACCATCGAGTCTTTGAACGCAAGTTGCGCCCCAAGCCATTAG**

**Fortunella_hindsii_3**  **AATTGCAGAATCCCGTGAACCATCGAGTCTTTGAACGCAAGTTGCGCCCCAAGCCATTAG**

**Fortunella_japonica_1**  **AATTGCAGAATCCCGTGAACCATCGAGTCTTTGAACGCAAGTTGCGCCCCAAGCCATTAG**

**Fortunella_japonica_2**  **AATTGCAGAATCCCGTGAACCATCGAGTCTTTGAACGCAAGTTGCGCCCCAAGCCATTAG**

**Fortunella_japonica_3**  **AATTGCAGAATCCCGTGAACCATCGAGTCTTTGAACGCAAGTTGCGCCCCAAGCCATTAG**

**Fortunella_margarita_2** **AATTGCAGAATCCCGTGAACCATCGAGTCTTTGAACGCAAGTTGCGCCCCAAGCCATTAG**

**Fortunella_venosa_1**  **AATTGCAGAATCCCGTGAACCATCGAGTCTTTGAACGCAAGTTGCGCCCCAAGCCATTAG**

**Fortunella_venosa_2**  **AATTGCAGAATCCCGTGAACCATCGAGTCTTTGAACGCAAGTTGCGCCCCAAGCCATTAG**

**Citrus_aurantiifolia**  **AATTGCAGAATCCCGTGAACCATCGAGTCTTTGAACGCAAGTTGCGCCCCAAGCCATTAG**

**Citrus_aurantium**  **AATTGCAGAATCCCGTGAACCATCGAGTCTTTGAACGCAAGTTGCGCCCCAAGCCATTAG**

**Citrus_hystrix**  **AATTGCAGAATCCCGTGAACCATCGAGTCTTTGAACGCAAGTTGCGCCCCAAGCCATTAG**

**Citrus_cavaleriei**  **AATTGCAGAATCCCGTGAACCATCGAGTCTTTGAACGCAAGTTGCGCCCCAAGCCATTAG**

**Citrus_junos**  **AATTGCAGAATCCCGTGAACCATCGAGTCTTTGAACGCAAGTTGCGCCCCAAGCCATTAG**

**Citrus_limon**  **AATTGCAGAATCCCGTGAACCATCGAGTCTTTGAACGCAAGTTGCGCCCCAAGCCATTAG**

**Citrus_tachibana**  **AATTGCAGAATCCCGTGAACCATCGAGTCTTTGAACGCAAGTTGCGCCCCAAGCCATTAG**

**Glycosmis_pentaphylla**  **AATTGCAGAATCCCGTGAACCATCGAGTCTTTGAACGCAAGTTGCGCCCCAAGCCATCAG**

**Poncirus_trifoliata**  **AATTGCAGAATCCCGTGAACCATCGAGTCTTTGAACGCAAGTTGCGCCCCAAGCCATTAG**

**Citrus_sinensis**  **AATTGCAGAATCCCGTGAACCATCGAGTCTTTGAACGCAAGTTGCGCCCCAAGCCATTAG**

**Citrus_reticulata**  **AATTGCAGAATCCCGTGAACCATCGAGTCTTTGAACGCAAGTTGCGCCCCAAGCCATTAG**

**Citrus_x_paradisi**  **AATTGCAGAATCCCGTGAACCATCGAGTCTTTGAACGCAAGTTGCGCCCCAAGCCATTTA**

**Citrus_maxima**  **AATTGCAGAATCCCGTGAACCATCGAGTCTTTGAACGCAAGTTGCGCCCCAAGCCATTAG**

**Citrus_medica**  **AATTGCAGAATCCCGTGAACCATCGAGTCTTTGAACGCAAGTTGCGCCCCAAGCCATTAG**

**Citrus_macroptera**  **AATTGCAGAATCCCGTGAACCATCGAGTCTTTGAACGCAAGTTGCGCCCCAAGCCATTAG**

370 380 390 400 410 420

....|....|....|....|....|....|....|....|....|....|....|....|

**Citrus_reticulata_1**  **GCCGAGGGCACGTCTGCCTGGGTGTCACGCATCGTTGCCCCACCCCACCCCCCCAAAC--**

**Citrus_trifoliata**  **GCCGAGGGCACGTCTGCCTGGGTGTCACGCATCGTTGCCCCACCCCACCCCCCCAAAC--**

**Fortunella_bawangica_1** **GCCGAGGGCACGTCTGCCTGGGTGTCACGCATCGTTGCTCCACCCCACCCCCCCAAAC--**

**Fortunella_bawangica_2** **GCCGAGGGCACGTCTGCCTGGGTGTCACGCATCGTTGCTCCACCCCACCCCCCCAAAC--**

**Fortunella_hindsii_1**  **GCCGAGGGCACGTCTGCCTGGGTGTCACGCATCGTTGCTCCACCCCACCCCCCCAAAC--**

**Fortunella_hindsii_2**  **GCCGAGGGCACGTCTGCCTGGGTGTCACGCATCGTTGCTCCACCCCACCCCCCCAAAC--**

**Fortunella_hindsii_3**  **GCCGAGGGCACGTCTGCCTGGGTGTCACGCATCGTTGCTCCACCCCACCCCCCCAAAC--**

**Fortunella_japonica_1**  **GCCGAGGGCACGTCTGCCTGGGTGTCACGCATCGTTGCTCCACCCCACCCCCCCAAAC--**

**Fortunella_japonica_2**  **GCCGAGGGCACGTCTGCCTGGGTGTCACGCATCGTTGCTCCACCCCACCCCCCCAAAC--**

**Fortunella_japonica_3**  **GCCGAGGGCACGTCTGCCTGGGTGTCACGCATCGTTGCTCCACCCCACCCCCCCAAAC--**

**Fortunella_margarita_2** **GCCGAGGGCACGTCTGCCTGGGTGTCACGCATCGTTGCTCCACCCCACCCCCCCAAAC--**

**Fortunella_venosa_1**  **GCCGAGGGCACGTCTGCCTGGGTGTCACGCATCGTTGCTCCACCCCACCCCCCCAAAC--**

**Fortunella_venosa_2**  **GCCGAGGGCACGTCTGCCTGGGTGTCACGCATCGTTGCTCCACCCCACCCCCCCAAAC--**

**Citrus_aurantiifolia**  **GCCGAGGGCACGTCTGCCTGGGTGTCACGCATCGTTGCCCCACCCCACCCCCCCAAAC--**

**Citrus_aurantium**  **GCCGAGGGCACGTCTGCCTGGGTGTCACGCATCGTTGCCCCACCCCACCCCCCCAAAC--**

**Citrus_hystrix**  **GCCGAGGGCACGTCTGCCTGGGTGTCACGCATCGTTGCCCCACCCCACCCCCCCAAAA--**

**Citrus_cavaleriei**  **GCCGAGGGCACGTCTGCCTGGGTGTCACGCATCGTTGCCCCACCCCACCCCCC-AAAC--**

**Citrus_junos**  **GCCGAGGGCACGTCTGCCTGGGCGTCACGCATCGTTGCCCCACCCCACCCCCCCAAAC--**

**Citrus_limon**  **GCCGAGGGCACGTCTGCCTGGGTGTCACGCATCGTTGCCCCACCCCACCCCCCCAAAC--**

**Citrus_tachibana**  **GCCGAGGGCACGTCTGCCTGGGTGTCACGCATCGTTGCCCCACCCCACCCCSCCAAAC--**

**Glycosmis_pentaphylla**  **GCCGAGGGCACGTCTGCCTGGGTGTCACGCATCGCTGCCCCACCCCGCTGCACCCCCCCG**

**Poncirus_trifoliata**  **GCCGAGGGCACGTCTGCCTGGGTGTCACGCATCGnnnnnnnnnnnnnnnnnnnnnnnnAA**

**Citrus_sinensis**  **GCCGAGGGCACGTCTGCCTGGGTGTCACGCATCGTTGCCCCACCCCACCCCCCCAAAC--**

**Citrus_reticulata**  **GCCGAGGGCACGTCTGCCTGGGTGTCACGCATCGTTGCCCCACCCCACCCCCCCAAAC--**

**Citrus_x_paradisi**  **GCCGAGGGCATGTCTGCTTGAGTCTCAGGCATCGCGGCCCCACCCCACCCCCGCAAAC--**

**Citrus_maxima**  **GCCGAGGGCACGTCTGCCTGGGTGTCACGCATCGTTGCCCCACCCCACCCCCCCAAAC--**

**Citrus_medica**  **GCCGAGGGCACGTCTGCCTGGGTGTCACGCATCGTTGCCCCACCCCACCCCCCCAAAC--**

**Citrus_macroptera**  **GCCGAGGGCACGTCTGCCTGGGTGTCACGCATCGTTGCCCCACCCCACCCCCCCAAAC--**

430 440 450 460 470 480

....|....|....|....|....|....|....|....|....|....|....|....|

**Citrus_reticulata_1**  **--CAAGGC--GGGGGCCCCGGGGTGCGG--GCGGAGATTGGCCTCCCGTGCGCTGACCGC**

**Citrus_trifoliata**  **--CAAGGC--GGGGGCCCCGGGGTGCGG--GCGGAGATTGGCCTCCCGTGCGCTGACCGC**

**Fortunella_bawangica_1** **--CAAGGC--GGGGGCCCCGGGGTGCGG--GCGGAGATTGGCCTCCCGTGCGCTGACCGC**

**Fortunella_bawangica_2** **--CAAGGC--GGGGGCCCCGGGGTGCGG--GCGGAGATTGGCCTCCCGTGCGCTGACCGC**

**Fortunella_hindsii_1**  **--CAAGGC--GGGGGCCCCGGGGTGCGG--GCGGAGATTGGCCTCCCGTGCGCTGACCGC**

**Fortunella_hindsii_2**  **--CAAGGC--GGGGGCCCCGGGGTGCGG--GCGGAGATTGGCCTCCCGTGCGCTGACCGC**

**Fortunella_hindsii_3**  **--CAAGGC--GGGGGCCCCGGGGTGCGG--GCGGAGATTGGCCTCCCGTGCGCTGACCGC**

**Fortunella_japonica_1**  **--CAAGGC--GGGGGCCCCGGGGTGCGG--GCGGAGATTGGCCTCCCGTGCGCTGACCGC**

**Fortunella_japonica_2**  **--CAAGGC--GGGGGCCCCGGGGTGCGG--GCGGAGATTGGCCTCCCGTGCGCTGACCGC**

**Fortunella_japonica_3**  **--CAAGGC--GGGGGCCCCGGGGTGCGG--GCGGAGATTGGCCTCCCGTGCGCTGACCGC**

**Fortunella_margarita_2** **--CAAGGC--GGGGGCCCCGGGGTGCGG--GCGGAGATTGGCCTCCCGTGCGCTGACCGC**

**Fortunella_venosa_1**  **--CAAGGC--GGGGGCCCCGGGGTGCGG--GCGGAGATTGGCCTCCCGTGCGCTGACCGC**

**Fortunella_venosa_2**  **--CAAGGC--GGGGGCCCCGGGGTGCGG--GCGGAGATTGGCCTCCCGTGCGCTGACCGC**

**Citrus_aurantiifolia**  **--CAAGGC--GGGGGCCCCGGGGTGCGG--GCGGAGATTGGCCTCCCGTGCGCTGACCGC**

**Citrus_aurantium**  **--CAAGGC--GGGGGCCCTGGGGTGCGG--GCGGAGATTGGCCTCCCGTGCGCTGACCGC**

**Citrus_hystrix**  **-CCAAGGC--GGGGGCCCCGGGGTGCGG--GCGGAGATTGGCCTCCCGTGCGCTGACCGC**

**Citrus_cavaleriei**  **--CAAGGC--GGGGGCCCTGGGGTGCGG--GCGGAGATTGGCCTCCCGTGCGCTGACCGC**

**Citrus_junos**  **--CAAGGC--GGGGGCCCCGGGGTGCGG--GCGGAGATTGGCCTCCCGTGCGCTGACCGC**

**Citrus_limon**  **--CAAGGC--GGGGGCCCCGGGGTGCGG--GCGGAGATTGGCCTCCCGTGCGCTGACCGC**

**Citrus_tachibana**  **--CAAGGC--GGGGGCCCCGGGGTGCGG--GCGGAGATTGGCCTCCCGTGCGYTGACCGC**

**Glycosmis_pentaphylla**  **ACCTACGT-GGGGGGCACGGCGGTGCGG--GCGGACATTGGCCTCCCGTGCGCTCCCCGC**

**Poncirus_trifoliata**  **ACCAAGGC--GGGGGCCCCGGGGTGCGG--GCGGAGATTGGCCTCCCGTGCGCTGACCGC**

**Citrus_sinensis**  **--CAAGGGCGGGGGGCCCCGGGGTGCGG--GCGGAGATTGGCCTCCCGTGCGCTGACCGC**

**Citrus_reticulata**  **--CAAGGC--GGGGGCCCCGGGGTGCGG--GCGGAGATTGGCCTCCCGTGCGCTGACCGC**

**Citrus_x_paradisi**  **--CAAGGC--GGGGGCCCTGGGGTGCGGTGGCGGAGTTTGGCCTCCCGTGCGCTGACCGC**

**Citrus_maxima**  **--CAAGGC--GGGGGCCCCGGGGTGCGG--GCGGAGATTGGCCTCCCGTGCGCTGACCGC**

**Citrus_medica**  **--CAAGGC--GGGGGCCCCGGGGTGCGG--GCGGAGATTGGCCTCCCGTGCGCTGACCGC**

**Citrus_macroptera**  **--CAAGGC--GGGGGCCCCGGGGTGCGG--GCGGAGATTGGCCTCCCGTGCGCTGACTGC**

490 500 510 520 530 540

....|....|....|....|....|....|....|....|....|....|....|....|

**Citrus_reticulata_1**  **TCGCGGTTGGCCCAAATCTGAGTCCTCGGCGAGCGAAGCCGCGGCGATCGGTGGTGAAAC**

**Citrus_trifoliata**  **TCGCGGTTGGCCCAAACACGAGTCCTCGGCGACCGAAGCCGCGGCGATCGGTGGCGAAAC**

**Fortunella_bawangica_1** **TCGCGGTTGGCCCAAATCTGAGTCCTCGGCGACCGAAGCCGCGGCGATCGGTGGCGAAAC**

**Fortunella_bawangica_2** **TCGCGGCTGGCCCAAATCTGAGTCCTCGGCGACCGAAGCCGCGGCGATCGGTGGCGAAAC**

**Fortunella_hindsii_1**  **TCGCGGTTGGCCCAAATTTGAGTCCTCGGCGACCGAAGCCGCGGCGATCGGTGGCGAAAC**

**Fortunella_hindsii_2**  **TCGCGGTTGGCCCAAATTTGAGTCCTCGGCGACCGAAGCCGCGGCGATCGGTGGCGAAAC**

**Fortunella_hindsii_3**  **TCGCGGTTGGCCCAAATTTGAGTCCTCGGCGACCGAAGCCGCGGCGATCGGTGGCGAAAC**

**Fortunella_japonica_1**  **TCGCGGCTGGCCCAAATCTGAGTCCTCGGCGACCGAAGCCGCGGCGATCGGTGGCGAAAC**

**Fortunella_japonica_2**  **TCGCGGCTGGCCCAAATCTGAGTCCTCGGCGACCGAAGCCGCGGCGATCGGTGGCGAAAC**

**Fortunella_japonica_3**  **TCGCGGCTGGCCCAAATCTGAGTCCTCGGCGACCGAAGCCGCGGCGATCGGTGGCGAAAC**

**Fortunella_margarita_2** **TCGCGGCTGGCCCAAATCTGAGTCCTCGGCGACCGAAGCCGCGGCGATCGGTGGCGAAAC**

**Fortunella_venosa_1**  **TCGCGGTTGGCCCAAATCTGAGTCCTCGGCGACCGAAGCCGTGGCGATCGGTGGCGAAAC**

**Fortunella_venosa_2**  **TCGCGGTTGGCCCAAATCTGAGTCCTCGGCGACCGAAGCCGTGGCGATCGGTGGCGAAAC**

**Citrus_aurantiifolia**  **TCGCGGTTGGCCCAAATATGAGTCCTCGGCGACCGAAGCCGCGGCGATCGGTGGTGAAAC**

**Citrus_aurantium**  **TCGCGGTTGGCCCAAATATGAGTCCTCGGCGACCGAAGCCGCGGCGATCGGTGGTGAAAC**

**Citrus_hystrix**  **TCGCGGTTGGCCCAAATATGAGTCCTCGGCGACCGAAGCCGCGGCGATCGGTGGTGAAAC**

**Citrus_cavaleriei**  **TCGCGGTTGGCCCAAATATGAGTCCTCGGCGACCGAAGCCGCGGCGATCGGTGGTGAAAC**

**Citrus_junos**  **TCGCGGTTGGCCCAAATCTGAGTCCTCGGCGACCGAAGCCGCGGCGATCGGTGGTGAAAC**

**Citrus_limon**  **TCGCGGTTGGCCCAAATATGAGTCCTCGGCGACCGAAGCCGCGGCGATCGGTGGTGAAAC**

**Citrus_tachibana**  **TSGCGGTTGGCCCAAATYTGAGTCCTCGGCGACCGAAGCYGCGGCGATCGGTGGTGAAAC**

**Glycosmis_pentaphylla**  **TCGCGGTTGGCCCAAATCCGAGTCCTCGGCGACCGGAGCCGCGGCGTTCGGTGGTGAAAC**

**Poncirus_trifoliata**  **TCGCGGTTGGCCCAAACACGAGTCCTCGGCGACCGAAGCCGCGGCGATCGGTGGCGAAAC**

**Citrus_sinensis**  **TCGCGGTTGGCCCAAATATGAGTCCTCGGCGACCGAAGCCGCGGCGATCGGTGGTGAAAC**

**Citrus_reticulata**  **TCGCGGTTGGCCCAAATTTGAGTCCTCGGCGAGCGAAGCCGCGGCGATCGGTGGTGAAAC**

**Citrus_x_paradisi**  **TCGCGGTTGGCCCAAATATGAGTCCTCGGCGACCGAAGCCGCGGCGATCGGTGGTGAAAC**

**Citrus_maxima**  **TCGCGGTTGGCCCAAACACGAGTCCTCGGCGACCGAAGCCGCGGCGATCGGTGGCGAAAC**

**Citrus_medica**  **TCGCGGTTGGCCCAAATATGAGTCCTCGGCGACCGAAGCCGCGGCGATCGGTGGTGAAAC**

**Citrus_macroptera**  **TCGCGGTTGGCCCAAATATGAGTCCTCGGCGACCGAAGCCGCGGCGATCGGTGGTGAAAC**

550 560 570 580 590 600

....|....|....|....|....|....|....|....|....|....|....|....|

**Citrus_reticulata_1**  **AAAAGCCTCTCGAGCTCCCGCCGCGCGC--CCGGTC-TCCGAGTGGGGACTCTGCGGCCC**

**Citrus_trifoliata**  **AAAAGCCTCTCGAGCTCCCGCCGCGCGC--CCGGTC-TCCGAGCGGGGACTCTGCGGCCC**

**Fortunella_bawangica_1** **AAAAGCCTCTCGAGCTCCCGCCGCGCGC--CCGGTC-TCCGAGTGGGGACTCTGCGACCC**

**Fortunella_bawangica_2** **AAAAGCCTCTCGAGCTCCCGCCGCGCGC--CCGGTC-TCCGAGTGGGGACTCTGCGACCC**

**Fortunella_hindsii_1**  **AAAAGCCTCTCGAGCTCCCGCCGCGCGC--CCGGTC-TCCGAGTGGGGACTCTGCGACCC**

**Fortunella_hindsii_2**  **AAAAGCCTCTCGAGCTCCCGCCGCGCGC--CCGGTC-TCTGAGTGGGGACTCTGCGACCC**

**Fortunella_hindsii_3**  **AAAAGCCTCTCGAGCTCCCGCCGCGCGC--CCGGTC-TCTGAGTGGGGACTCTGCGACCC**

**Fortunella_japonica_1**  **AAAAGCCTCTCGAGCTCCCGCCGCGCGC--CCGGTC-TCCGAGTGGGGACTCTGCGACCC**

**Fortunella_japonica_2**  **AAAAGCCTCTCGAGCTCCCGCCGCGCGC--CCGGTC-TCCGAGTGGGGACTCTGCGACCC**

**Fortunella_japonica_3**  **AAAAGCCTCTCGAGCTCCCGCCGCGCGC--CCGGTC-TCCGAGTGGGGACTCTGCGACCC**

**Fortunella_margarita_2** **AAAAGCCTCTCGAGCTCCCGCCGCGCGC--CCGGTC-TCCGAGTGGGGACTCTGCGACCC**

**Fortunella_venosa_1**  **AAAAGCCTCTCGAGCTCCCGCCGCGCGC--CCGGTC-TCCGAGTGGGGACTCTGCGACCC**

**Fortunella_venosa_2**  **AAAAGCCTCTCGAGCTCCCGCCGCGCGC--CCGGTC-TCCGAGTGGGGACTCTGCGACCC**

**Citrus_aurantiifolia**  **AAA-GCCTCTCGAGCTCCCGCCGCGCGC--CCGGTC-TCCAAGTGTGGACTCTGCGACCC**

**Citrus_aurantium**  **AAA-GCCTCTCGAGCTCCCGCCGCGCGC--CCGGTC-TCCAAGTGTGGACTCTGCGACCC**

**Citrus_hystrix**  **AAA-GCCTCTCGAGCTCCCGCCGCGCGC--CCGGTC-TCCAAGTGTGGACTCTGCGACCC**

**Citrus_cavaleriei**  **AAA-GCCTCTCGTGCTCCCGCCGCGCGC--CCGGTC-TCCAAGTGTGGACTCTGCGACCC**

**Citrus_junos**  **AAAAGCCTCTCGAGCTCCCGCCGCGCAC--CCGGTC-TCCGAGTGGGGACTCTGCGGCCC**

**Citrus_limon**  **AAA-GCCTCTCGAGCTCCCGCCGCGCGC--CCGGTC-TCCAAGTGTGGACTCTGCGACCC**

**Citrus_tachibana**  **AAAAGCCTCTCGAGCTCCCGCCGCGCGC--CCGGTC-TCCGAGTGGGGACTCTGCGRCCC**

**Glycosmis_pentaphylla**  **AAAAGCCTCTCGAGCTCCCGCCGCGCGCGCCCGGTCACCCCTGTGGGGACTCCGCGGCCC**

**Poncirus_trifoliata**  **AAAAGCCTCTCGAGCTCCCGCCGCGCGC--CCGGTC-TCCGAGCGGGGACTCTGCGGCCC**

**Citrus_sinensis**  **AAAGGC------------------------------------------------------**

**Citrus_reticulata**  **AAAAGCCTCTCGAGCTCCCGCCGCGCGC--CCGGTC-TCCGAGTGGGGACTCTGCGGCCC**

**Citrus_x_paradisi**  **AAA-GCCTCTCGAGCTCCCGCCGCGCGC--CCGGTC-TCCGAGTGTGGACTCTGCGGCCC**

**Citrus_maxima**  **AAAAGCCTCTCGAGCTCCCGCCGCGCGC--CCGGTC-TCCGAGCGGGGACTCTGCGGCCC**

**Citrus_medica**  **AAA-GCCTCTCGAGCTCCCGCCGCGCGC--CCGGTC-TCCAAGTGTGTACTCTGCGACCC**

**Citrus_macroptera**  **AAA-GCCTCTTGAGCTCCCGCCGTGCGC--CCGGTC-TCCAAGTGTGGACTCTGCGACCC**

610 620 630 640

....|....|....|....|....|....|....|....|....|

**Citrus_reticulata_1**  **TGAAGCTCCGCGCAAGC----GGCGCTCGCATTGCGACC---CCA**

**Citrus_trifoliata**  **TGAAGCTCCGCGCAAGCAAGCGGCGCTCGCATCGCGACC---CCA**

**Fortunella_bawangica_1** **TGAAGCTCCGCGCAAGC----GGCGCTCGCATCGCGACC---CCA**

**Fortunella_bawangica_2** **TGAAGCTCCGCGCAAGC----GGCGCTCGCATCGCGACC---CCA**

**Fortunella_hindsii_1**  **TGAAGCTCCGCGCAAGC----GGCGCTCGCATCGCGACC---CCA**

**Fortunella_hindsii_2**  **TGAAGCTCCGCGCAAGC----GGCGCTCGCATCGCGACC---CCA**

**Fortunella_hindsii_3**  **TGAAGCTCCGCGCAAGC----GGCGCTCGCATCGCGACC---CCA**

**Fortunella_japonica_1**  **TGAAGCTCCGCGCAAGC----GGCGCTCGCATCGCGACC---CCA**

**Fortunella_japonica_2**  **TGAAGCTCCGCGCAAGC----GGCGCTCGCATCGCGACC---CCA**

**Fortunella_japonica_3**  **TGAAGCTCCGCGCAAGC----GGCGCTCGCATCGCGACC---CCA**

**Fortunella_margarita_2** **TGAAGCTCCGCGCAAGC----GGCGCTCGCATCGCGACC---CCA**

**Fortunella_venosa_1**  **TGAAGCTCCGCGCAAGC----GGCGCTCGCATCGCGACC---CCA**

**Fortunella_venosa_2**  **TGAAGCTCCGCGCAAGC----GGCGCTCGCATCGCGACC---CCA**

**Citrus_aurantiifolia**  **TGAAGCTCCGTTCCAAC----GGCGCTCGCATCGCGACC---CCA**

**Citrus_aurantium**  **TGAAGCTCCGCGCAAGC----GGC---------------------**

**Citrus_hystrix**  **TGAAGCTCCGCGCAAGC----GGCGCTCGCATTGCGACC---CCA**

**Citrus_cavaleriei**  **TGAAGCTCCGCGCAAGC----GGCGCTCGCATTGCGACC---CCA**

**Citrus_junos**  **TGATGCTCCGCGCAAGC----GGCGCTCGCATTGCGACC---CCA**

**Citrus_limon**  **TGAAGCTCCGTT---------------------------------**

**Citrus_tachibana**  **TGAAGCTCCGCGCAAGC----GGCGCTCGCATYGCGACC---CCA**

**Glycosmis_pentaphylla**  **AGACGCTCCGCGCCAGC----GGCGCTCGCATCGCGACC---CCA**

**Poncirus_trifoliata**  **TGAAGCTCCGCGCAAGCAAGCGGCGCTCGCATCGCGACC---C-A**

**Citrus_sinensis**  **---------------------------------------------**

**Citrus_reticulata**  **TGAAGCTCCGCGCAAGC----GGCGCTCGCATTGCGACC---CC-**

**Citrus_x_paradisi**  **TGAAGCTCCGCGCAAGC----GGCGCTCGCAT-GCGACC---CCA**

**Citrus_maxima**  **TGAAGCTCCGCGCAAGC----GGCGCTCGCATT------------**

**Citrus_medica**  **TGAAGCTCCGTTCCAAC----GGCGCTCGCATCGCGACGAGACCA**

**Citrus_macroptera**  **TGAAGCTCTGCGCAAGC----GGCGCTC-----------------**
