# Supplementary material for: Fortunella venosa (Champ. ex Benth.) C. C. Huang and F. hindsii (Champ. ex Benth.) Swingle as Independent Species: Evidence From Morphology and Molecular Systematics and Taxonomic Revision of Fortunella (Rutaceae)
Source: Front Plant Sci. 2022 May 12;13:867659. doi: 10.3389/fpls.2022.867659 (PMC9133918; doi:10.3389/fpls.2022.867659)
Supplement: Supplementary file 4 [file Table_4.DOCX]

10	20	30	40	50	60

....|....|....|....|....|....|....|....|....|....|....|....|

**Atalantia_kwangtungens** **atagctatgggcgaacgacgggaattgaacccgcgcatggtggattcacaatccactgcc**

**Citrus_reticulata_1** **ataactatgggcgaacgacgggaattgaacccgcgcatggtggattcacaatccactgcc**

**Citrus_reticulata_2** **ataactatgggcgaacgacgggaattgaacccgcgcatggtggattcacaatccactgcc**

**Citrus_trifoliata_3** **ctagctatgggcgaacgacgggaattgaacccgcgcatggtggattcacaatccactgcc**

**Citrus_aurantiifolia** **-------tgggcgaacgacgggaattgaacccgcgcgtggtggattcacaatccactgcc**

**Citrus_aurantium** **-----------cgaacgacgggaattgaacccgcgcatggtggattcacaatccactgcc**

**Citrus_cavaleriei** **ataactatgggcgaacgacgggaattgaacccgcgcatggtggattcacaatccactgcc**

**Citrus_hongheensis** **-----------cgaacgacgggaattgaacccgcgcatggtggattcacaatccactgcc**

**Citrus_limon** **-----------cgaacgacgggaattgaacccgcgcatggtggattcacaatccactgcc**

**Citrus_maxima** **atagctatgggcgaacgacgggaattgaacccgcgcatggtggattcacaatccactgcc**

**Citrus_medica** **ataactatgggcgaacgacgggaattgaacccgcgcatggtggattcacaatccactgcc**

**Citrus_polytrifolia** **-------tgggcgaacgacgggaattgaacccgcgcatggtggattcacaatccactgcc**

**Citrus_reticulata** **ataactatgggcgaacgacgggaattgaacccgcgcatggtggattcacaatccactgcc**

**Citrus_sinensis** **-------tgggcgaacgacgggaattgaacccgcgcatggtggattcacaatccactgcc**

**Fortunella_bawangica_1** **atagctatgggcgaacgacgggaattgaacccgcgcatggtggattcacaatccactgcc**

**Fortunella_bawangica_2** **atagctatgggcgaacgacgggaattgaacccgcgcatggtggattcacaatccactgcc**

**Fortunella_hindsii_1** **atagctatgggcgaacgacgggaattgaacccgcgcatggtggattcacaatccactgcc**

**Fortunella_hindsii_2** **atagctatgggcgaacgacgggaattgaacccgcgcatggtggattcacaatccactgcc**

**Fortunella_hindsii_3** **atagctatgggcgaacgacgggaattgaacccgcgcatggtggattcacaatccactgcc**

**Fortunella_japonica_1** **------atgggcgaacgacgggaattgaacccgcgcatggtggattcacaatccactgcc**

**Fortunella_japonica_2** **atagctatgggcgaacgacgggaattgaacccgcgcatggtggattcacaatccactgcc**

**Fortunella_japonica_3** **atagctatgggcgaacgacgggaattgaacccgcgcatggtggattcacaatccactgcc**

**Fortunella_japonica_4** **atagctatgggcgaacgacgggaattgaacccgcgcatggtggattcacaatccactgcc**

**Fortunella_japonica_5** **atagctatgggcgaacgacgggaattgaacccgcgcatggtggattcacaatccactgcc**

**Fortunella_japonica_6** **atagctatgggcgaacgacgggaattgaacccgcgcatggtggattcacaatccactgcc**

**Fortunella_japonica_7** **atagctatgggcgaacgacgggaattgaacccgcgcatggtggattcacaatccactgcc**

**Fortunella_japonica_8** **atagctatgggcgaacgacgggaattgaacccgcgcatggtggattcacaatccactgcc**

**Fortunella_japonica_9** **atagctatgggcgaacgacgggaattgaacccgcgcatggtggattcacaatccactgcc**

**Fortunella_japonica_10** **atagctatgggcgaacgacgggaattgaacccgcgcatggtggattcacaatccactgcc**

**Fortunella_japonica_11** **atagctatgggcgaacgacgggaattgaacccgcgcatggtggattcacaatccactgcc**

**Fortunella_japonica_12** **atagctatgggcgaacgacgggaattgaacccgcgcatggtggattcacaatccactgcc**

**Fortunella_margarita_1** **atagctatgggcgaacgacgggaattgaacccgcgcatggtggattcacaatccactgcc**

**Fortunella_margarita_2** **atagctatgggcgaacgacgggaattgaacccgcgcatggtggattcacaatccactgcc**

**Fortunella_venosa_1** **atagctatgggcgaacgacgggaattgaacccgcgcatggtggattcacaatccactgcc**

**Fortunella_venosa_2** **atagctatgggcgaacgacgggaattgaacccgcgcatggtggattcacaatccactgcc**

**Fortunella_venosa_3** **atagctatgggcgaacgacgggaattgaacccgcgcatggtggattcacaatccactgcc**

70	80	90	100	110	120

....|....|....|....|....|....|....|....|....|....|....|....|

**Atalantia_kwangtungens** **ttgatccacttggctacatccgcccctccgctatttacacaattttgattttgaaataca**

**Citrus_reticulata_1** **ttgatccacttggctacatccgcccctccgctatttacaca------attttgaaataca**

**Citrus_reticulata_2** **ttgatccacttggctacatccgcccctccgctatttacaca------attttgaaataca**

**Citrus_trifoliata_3** **ttgatccacttggctacatccgcccctccgctatttacaca------attttgaaatgca**

**Citrus_aurantiifolia** **ttgatccacttggctacatccgcccctccgctatttacaca------attttgaaataca**

**Citrus_aurantium** **ttgatccacttggctacatccgcccctccgctatttacaca------attttgaaataca**

**Citrus_cavaleriei** **ttgatccacttggctacatccgcccctccactatttacaca------attttgaaataca**

**Citrus_hongheensis** **ttgatccacttggctacatccgcccctccgctatttacaca------attttgaaataca**

**Citrus_limon** **ttgatccacttggctacatccgcccctccgctatttacaca------attttgaaataca**

**Citrus_maxima** **ttgatccacttggctacatccgcccctccgctatttacaca------attttgaaataca**

**Citrus_medica** **ttgatccacttggctacatccgcccctccgctatttacaca------attttgaaataca**

**Citrus_polytrifolia** **ttgatccacttggctacatccgcccctccgctatttacaca------attttgaaatgca**

**Citrus_reticulata** **ttgatccacttggctacatccgcccctccgctatttacaca------attttgaaataca**

**Citrus_sinensis** **ttgatccacttggctacatccgcccctccgctatttacaca------attttgaaataca**

**Fortunella_bawangica_1** **ttgatccacttggctacatccgcccctccgctatttacaca------attttgaaataca**

**Fortunella_bawangica_2** **ttgatccacttggctacatccgcccctccgctatttacaca------attttgaaataca**

**Fortunella_hindsii_1** **ttgatccacttggctacatccgcccctccgctatttacaca------attttgaaataca**

**Fortunella_hindsii_2** **ttgatccacttggctacatccgcccctccgctatttacaca------attttgaaataca**

**Fortunella_hindsii_3** **ttgatccacttggctacatccgcccctccgctatttacaca------attttgaaataca**

**Fortunella_japonica_1** **ttgatccacttggctacatccgcccctccgctatttacaca------attttgaaataca**

**Fortunella_japonica_2** **ttgatccacttggctacatccgcccctccgctatttacaca------attttgaaataca**

**Fortunella_japonica_3** **ttgatccacttggctacatccgcccctccgctatttacaca------attttgaaataca**

**Fortunella_japonica_4** **ttgatccacttggctacatccgcccctccgctatttacaca------attttgaaataca**

**Fortunella_japonica_5** **ttgatccacttggctacatccgcccctccgctatttacaca------attttgaaataca**

**Fortunella_japonica_6** **ttgatccacttggctacatccgcccctccgctatttacaca------attttgaaataca**

**Fortunella_japonica_7** **ttgatccacttggctacatccgcccctccgctatttacaca------attttgaaataca**

**Fortunella_japonica_8** **ttgatccacttggctacatccgcccctccgctatttacaca------attttgaaataca**

**Fortunella_japonica_9** **ttgatccacttggctacatccgcccctccgctatttacaca------attttgaaataca**

**Fortunella_japonica_10** **ttgatccacttggctacatccgcccctccgcgatttacaca------attttgaaataca**

**Fortunella_japonica_11** **ttgatccacttggctacatccgcccctccgctatttacaca------attttgaaataca**

**Fortunella_japonica_12** **ttgatccacttggctacatccgcccctccgctatttacaca------attttgaaataca**

**Fortunella_margarita_1** **ttgatccacttggctacatccgcccctccgcgatttacaca------attttgaaataca**

**Fortunella_margarita_2** **ttgatccacttggctacatccgcccctccgcgatttacaca------attttgaaataca**

**Fortunella_venosa_1** **ttgatccacttggctacatccgcccctccgctatttacaca------attttgaaataca**

**Fortunella_venosa_2** **ttgatccacttggctacatccgcccctccgctatttacaca------attttgaaataca**

**Fortunella_venosa_3** **ttgatccacttggctacatccgcccctccgctatttacaca------attttgaaataca**

130	140	150	160	170	180

....|....|....|....|....|....|....|....|....|....|....|....|

**Atalantia_kwangtungens** **aagatctcaaatccaaccattgataattt---gataatttttttatcttatgaagagcca**

**Citrus_reticulata_1** **aagatctcaaattcaaccattgattatttttgtttatcttatcttacttatgaagagcca**

**Citrus_reticulata_2** **aagatctcaaattcaaccattgattatttttgtttatcttatcttacttatgaagagcca**

**Citrus_trifoliata_3** **aagatctcaaattcaaccattgattatttttgtttatcttatcttacttatgaagagcca**

**Citrus_aurantiifolia** **aagatctcaaattcaaccattgattatttttgtttatcttatcttacttatgaagagcca**

**Citrus_aurantium** **aagatctaaaattcaaccattgattatttttgtttatcttatcttacttatgaagagcca**

**Citrus_cavaleriei** **aagatctcaaa---------tgattat-----------ttatcttacttatgaagagcca**

**Citrus_hongheensis** **aagatctaaaattcaaccattgattatttttgtttatcttatcttacttatgaagagcca**

**Citrus_limon** **aagatctaaaattcaaccattgattatttttgtttatcttatcttacttatgaagagcca**

**Citrus_maxima** **aagatctaaaattcaaccattgattatttttgtttatcttatcttacttatgaagagcca**

**Citrus_medica** **aagatctcaaattcaaccattgattatttttgtttatcttatcttacttatgaagaacca**

**Citrus_polytrifolia** **aagatctcaaattcaaccattgattatttttgtttatcttatcttacttatgaagagcca**

**Citrus_reticulata** **aagatctcaaattcaaccattgattatttttgtttatcttatcttacttatgaagagcca**

**Citrus_sinensis** **aagatctaaaattcaaccattgattatttttgtttatcttatcttacttatgaagagcca**

**Fortunella_bawangica_1** **aagatctcaaattcaaccattgattatttttgtttatcttatcttacttatgaagagcca**

**Fortunella_bawangica_2** **aagatctcaaattcaaccattgattatttttgtttatcttatcttacttatgaagagcca**

**Fortunella_hindsii_1** **aagatctcaaattcaaccattgattatttttgtttatcttatcttacttatgaagagcca**

**Fortunella_hindsii_2** **aagatctcaaattcaaccattgattatttttgtttatcttatcttacttatgaagagcca**

**Fortunella_hindsii_3** **aagatctcaaattcaaccattgattatttttgtttatcttatcttacttatgaagagcca**

**Fortunella_japonica_1** **aagatctcaaattcaaccattgattatttttgtttatcttatcttacttatgaagagcca**

**Fortunella_japonica_2** **aagatctcaaattcaaccattgattatttttgtttatcttatcttacttatgaagagcca**

**Fortunella_japonica_3** **aagatctcaaattcaaccattgattatttttgtttatcttatcttacttatgaagagcca**

**Fortunella_japonica_4** **aagatctcaaattcaaccattgattatttttgtttatcttatcttacttatgaagagcca**

**Fortunella_japonica_5** **aagatctcaaattcaaccattgattatttttgtttatcttatcttacttatgaagagcca**

**Fortunella_japonica_6** **aagatctcaaattcaaccattgattatttttgtttatcttatcttacttatgaagagcca**

**Fortunella_japonica_7** **aagatctcaaattcaaccattgattatttttgtttatcttatcttacttatgaagagcca**

**Fortunella_japonica_8** **aagatctcaaattcaaccattgattatttttgtttatcttatcttacttatgaagagcca**

**Fortunella_japonica_9** **aagatctcaaattcaaccattgattatttttgtttatcttatcttacttatgaagagcca**

**Fortunella_japonica_10** **aagatctcaaattcaaccattgattatttttgtttatcttatcttacttatgaagagcca**

**Fortunella_japonica_11** **aagatctcaaattcaaccattgattatttttgtttatcttatcttacttatgaagagcca**

**Fortunella_japonica_12** **aagatctcaaattcaaccattgattatttttgtttatcttatcttacttatgaagagcca**

**Fortunella_margarita_1** **aagatctcaaattcaaccattgattatttttgtttatcttatcttacttatgaagagcca**

**Fortunella_margarita_2** **aagatctcaaattcaaccattgattatttttgtttatcttatcttacttatgaagagcca**

**Fortunella_venosa_1** **aagatctcaaattcaaccattgattatttttgtttatcttatcttacttatgaagagcca**

**Fortunella_venosa_2** **aagatctcaaattcaaccattgattatttttgtttatcttatcttacttatgaagagcca**

**Fortunella_venosa_3** **aagatctcaaattcaaccattgattatttttgtttatcttatcttacttatgaagagcca**

190	200	210	220	230	240

....|....|....|....|....|....|....|....|....|....|....|....|

**Atalantia_kwangtungens** **aatgaagatcgaagggcagaaaactacaacctttctattgtcttttttcttttctataaa**

**Citrus_reticulata_1** **aatgaagatcgaagggcagaaaactacaacctttctattgtcttttttctttgctatgaa**

**Citrus_reticulata_2** **aatgaagatcgaagggcagaaaactacaacctttctattgtcttttttctttgctatgaa**

**Citrus_trifoliata_3** **aatgaagatcgaagggcagaaaactacaacctttctattgtcttttttctttgctatgaa**

**Citrus_aurantiifolia** **aatgaagatcgaagggaagaaaactacaacctttctattgtcttttttctttgctatgaa**

**Citrus_aurantium** **aatgaagatcgaagagcagaaaactacaacctttctattgtcttttttctttgctatgaa**

**Citrus_cavaleriei** **aatgaagatcgaagggcagaaaactacaacctttctattgtcttttttctttactatgaa**

**Citrus_hongheensis** **aatgaagatcgaagagcagaaaactacaacctttctattgtcttttttctttgctatgaa**

**Citrus_limon** **aatgaagatcgaagagcagaaaactacaacctttctattgtcttttttctttgctatgaa**

**Citrus_maxima** **aatgaagatcgaagagcagaaaactacaacctttctattggcttttttctttgctatgaa**

**Citrus_medica** **aatgaaaatcgaagggcagaaaactacaacctttctattgtcttttttctttgctatgca**

**Citrus_polytrifolia** **aatgaagatcgaagggcagaaaactacaacctttctattgtcttttttctttgctatgaa**

**Citrus_reticulata** **aatgaagatcgaagggcagaaaactacaacctttctattgtcttttttctttgctatgaa**

**Citrus_sinensis** **aatgaagatcgaagagcagaaaactacaacctttctattgtcttttttctttgctatgaa**

**Fortunella_bawangica_1** **aatgaagatcgaagggcagaaaactacaacctttctattgtcttttttctttgctatgaa**

**Fortunella_bawangica_2** **aatgaagatcgaagggcagaaaactacaacctttctattgtcttttttctttgctatgaa**

**Fortunella_hindsii_1** **aatgaagatcgaagggcagaaaactacaacctttctattgtcttttttctttgctatgaa**

**Fortunella_hindsii_2** **aatgaagatcgaagggcagaaaactacaacctttctattgtcttttttctttgctatgaa**

**Fortunella_hindsii_3** **aatgaagatcgaagggcagaaaactacaacctttctattgtcttttttctttgctatgaa**

**Fortunella_japonica_1** **aatgaagatcgaagggcagaaaactacaacctttctattgtcttttttctttgctatgaa**

**Fortunella_japonica_2** **aatgaagatcgaagggcagaaaactacaacctttctattgtcttttttctttgctatgaa**

**Fortunella_japonica_3** **aatgaagatcgaagggcagaaaactacaacctttctattgtcttttttctttgctatgaa**

**Fortunella_japonica_4** **aatgaagatcgaagggcagaaaactacaacctttctattgtcttttttctttgctatgaa**

**Fortunella_japonica_5** **aatgaagatcgaagggcagaaaactacaacctttctattgtcttttttctttgctatgaa**

**Fortunella_japonica_6** **aatgaagatcgaagggcagaaaactacaacctttctattgtcttttttctttgctatgaa**

**Fortunella_japonica_7** **aatgaagatcgaagggcagaaaactacaacctttctattgtcttttttctttgctatgaa**

**Fortunella_japonica_8** **aatgaagatcgaagggcagaaaactacaacctttctattgtcttttttctttgctatgaa**

**Fortunella_japonica_9** **aatgaagatcgaagggcagaaaactacaacctttctattgtcttttttctttgctatgaa**

**Fortunella_japonica_10** **aatgaagatcgaagggcagaaaactacaacctttctattgtcttttttctttgctatgaa**

**Fortunella_japonica_11** **aatgaagatcgaagggcagaaaactacaacctttctattgtcttttttctttgctatgaa**

**Fortunella_japonica_12** **aatgaagatcgaagggcagaaaactacaacctttctattgtcttttttctttgctatgaa**

**Fortunella_margarita_1** **aatgaagatcgaagggcagaaaactacaacctttctattgtcttttttctttgctatgaa**

**Fortunella_margarita_2** **aatgaagatcgaagggcagaaaactacaacctttctattgtcttttttctttgctatgaa**

**Fortunella_venosa_1** **aatgaagatcgaagggcagaaaactacaacctttctattgtcttttttctttgctatgaa**

**Fortunella_venosa_2** **aatgaagatcgaagggcagaaaactacaacctttctattgtcttttttctttgctatgaa**

**Fortunella_venosa_3** **aatgaagatcgaagggcagaaaactacaacctttctattgtcttttttctttgctatgaa**

250	260	270	280	290	300

....|....|....|....|....|....|....|....|....|....|....|....|

**Atalantia_kwangtungens** **atctataaaatctataaaatgaactgtaaaattagaaaatgcgactctaatttctaatta**

**Citrus_reticulata_1** **att------------------aactgtaaaattagaaaatgcgactctaatttctaatta**

**Citrus_reticulata_2** **att------------------aactgtaaaattagaaaatgcgactctaatttctaatta**

**Citrus_trifoliata_3** **att------------------aactgtaaaattagaaaatgcgactcgactttctaatta**

**Citrus_aurantiifolia** **att------------------aactgtaaaattcgaaaatgcgactctaatttctaatta**

**Citrus_aurantium** **att------------------aactgtaaaattagaaa--------ctaatttctaatta**

**Citrus_cavaleriei** **att------------------aactgtaaaattcgaaaatgcgactctaatttcgaatta**

**Citrus_hongheensis** **att------------------aactgtaaaattagaaaatgcgactctaatttctaatta**

**Citrus_limon** **att------------------aactgtaaaattagaaa--------ctaatttctaatta**

**Citrus_maxima** **att------------------aactgtaaaattagaaa--------ctaatttctaatta**

**Citrus_medica** **att------------------aactgtaaaattagaaaatgcgactcgaatttctaatta**

**Citrus_polytrifolia** **att------------------aactgtaaaattagaaaatgcgactcgactttctaatta**

**Citrus_reticulata** **att------------------aactgtaaaattagaaaatgcgactctaatttctaatta**

**Citrus_sinensis** **att------------------aactgtaaaattagaaa--------ctaatttctaatta**

**Fortunella_bawangica_1** **att------------------aactgtaaaattataaaatacgactctaatttctaatta**

**Fortunella_bawangica_2** **att------------------aactgtaaaattataaaatacgactctaatttctaatta**

**Fortunella_hindsii_1** **att------------------aactgtaaaattataaaatgcgactctaatttctaatta**

**Fortunella_hindsii_2** **att------------------aactgtaaaattataaaatgcgactctaatttctaatta**

**Fortunella_hindsii_3** **att------------------aactgtaaaattataaaatgcgactctaatttctaatta**

**Fortunella_japonica_1** **att------------------aactgtaaaattataaaatgcgactctaatttctaatta**

**Fortunella_japonica_2** **att------------------aactgtaaaattataaaatgcgactctaatttctaatta**

**Fortunella_japonica_3** **att------------------aactgtaaaattataaaatgcgactctaatttctaatta**

**Fortunella_japonica_4** **att------------------aactgtaaaattataaaatgcgactctaatttctaatta**

**Fortunella_japonica_5** **att------------------aactgtaaaattataaaatgcgactctaatttctaatta**

**Fortunella_japonica_6** **att------------------aactgtaaaattataaaatgcgactctaatttctaatta**

**Fortunella_japonica_7** **att------------------aactgtaaaattataaaatgcgactctaatttctaatta**

**Fortunella_japonica_8** **att------------------aactgtaaaattataaaatgcgactctaatttctaatta**

**Fortunella_japonica_9** **att------------------aactgtaaaattataaaatgcgactctaatttctaatta**

**Fortunella_japonica_10** **att------------------aactgtaaaattataaaatacgactctaatttctaatta**

**Fortunella_japonica_11** **att------------------aactgtaaaattataaaatgcgactctaatttctaatta**

**Fortunella_japonica_12** **att------------------aactgtaaaattataaaatgcgactctaatttctaatta**

**Fortunella_margarita_1** **att------------------aactgtaaaattataaaatacgactctaatttctaatta**

**Fortunella_margarita_2** **att------------------aactgtaaaattataaaatacgactctaatttctaatta**

**Fortunella_venosa_1** **att------------------aactgtaaaattataaaatgcgactctaatttctaatta**

**Fortunella_venosa_2** **att------------------aactgtaaaattataaaatgcgactctaatttctaatta**

**Fortunella_venosa_3** **att------------------aactgtaaaattataaaatgcgactctaatttctaatta**

310	320	330	340	350	360

....|....|....|....|....|....|....|....|....|....|....|....|

**Atalantia_kwangtungens** **ataatctaattaataaa-------attcgaaatttattaatttattagtagtattagcgc**

**Citrus_reticulata_1** **ataatctaa-taataaa-------attagaaatttattaatttattagtagtattagcgc**

**Citrus_reticulata_2** **ataatctaa-taataaa-------attagaaatttattaatttattagtagtattagcgc**

**Citrus_trifoliata_3** **ataatctaa-taagaaa-------attaga--------aatttattagtagtattagcgc**

**Citrus_aurantiifolia** **ataatctaa-taataaa-------attataaatttactaatttattagtagtattagcgc**

**Citrus_aurantium** **ataatctaa-taataaa-------attataaatttagtaatttattagtagtattagcgc**

**Citrus_cavaleriei** **ataatctaa-taataaa-------attaga--------aatttattagtagtattagcgc**

**Citrus_hongheensis** **ataatctaa-taataaa-------attagaaatttagtaatttattagtagtattagcgc**

**Citrus_limon** **ataatctaa-taataaa-------attataaatttagtaatttattagtagtattagcgc**

**Citrus_maxima** **ataatctaa-taataaa-------attagaaatttagtaatttattagtagtattagcgc**

**Citrus_medica** **ataatctaa-taataaa-------attaga--------aatttattagtagtattagcgc**

**Citrus_polytrifolia** **ataatctaa-taagaaa-------attata--------aatttattagtagtattagcgc**

**Citrus_reticulata** **ataatctaa-taataaa-------attagaaatttattaatttattagtagtattagcgc**

**Citrus_sinensis** **ataatctaa-taataaa-------attagaaatttagtaatttattagtagtattagcgc**

**Fortunella_bawangica_1** **ataatctaa-taataaataataacattcgaaatttattaatttattagtagtattagcgc**

**Fortunella_bawangica_2** **ataatctaa-taataaataataacattcgaaatttattaatttattagtagtattagcgc**

**Fortunella_hindsii_1** **ataatctaa-taataaataataacattcgaaatttattaatttattagtagtattagcgc**

**Fortunella_hindsii_2** **ataatctaa-taataaataataacattcgaaatttattaatttattagtagtattagcgc**

**Fortunella_hindsii_3** **ataatctaa-taataaataataacattcgaaatttattaatttattagtagtattagcgc**

**Fortunella_japonica_1** **ataatctaa-taataaataataacattcgaaatttattaatttattagtagtattagcgc**

**Fortunella_japonica_2** **ataatctaa-taataaataataacattcgaaatttattaatttattagtagtattagcgc**

**Fortunella_japonica_3** **ataatctaa-taataaataataacattcgaaatttattaatttattagtagtattagcgc**

**Fortunella_japonica_4** **ataatctaa-taataaataataacattcgaaatttattaatttattagtagtattagcgc**

**Fortunella_japonica_5** **ataatctaa-taataaataataacattcgaaatttattaatttattagtagtattagcgc**

**Fortunella_japonica_6** **ataatctaa-taataaataataacattcgaaatttattaatttattagtagtattagcgc**

**Fortunella_japonica_7** **ataatctaa-taataaataataacattcgaaatttattaatttattagtagtattagcgc**

**Fortunella_japonica_8** **ataatctaa-taataaataataacattcgaaatttattaatttattagtagtattagcgc**

**Fortunella_japonica_9** **ataatctaa-taataaataataacattcgaaatttattaatttattagtagtattagcgc**

**Fortunella_japonica_10** **ataatctaa-taataaataataacattcgaaatttattaatttattagtagtattagcgc**

**Fortunella_japonica_11** **ataatctaa-taataaataataacattcgaaatttattaatttattagtagtattagcgc**

**Fortunella_japonica_12** **ataatctaa-taataaataataacattcgaaatttattaatttattagtagtattagcgc**

**Fortunella_margarita_1** **ataatctaa-taataaataataacattcgaaatttattaatttattagtagtattagcgc**

**Fortunella_margarita_2** **ataatctaa-taataaataataacattcgaaatttattaatttattagtagtattagcgc**

**Fortunella_venosa_1** **ataatctaa-taataaataataacattcgaaatttattaatttattagtagtattagcgc**

**Fortunella_venosa_2** **ataatctaa-taataaataataacattcgaaatttattaatttattagtagtattagcgc**

**Fortunella_venosa_3** **ataatctaa-taataaataataacattcgaaatttattaatttattagtagtattagcgc**

370	380	390	400	410	420

....|....|....|....|....|....|....|....|....|....|....|....|

**Atalantia_kwangtungens** **atagcaaaaatat-----ctaaatc-------------aaataaacgaaagaaaaagcat**

**Citrus_reticulata_1** **ataccaacaata------tcatact-------------aaatcaaagaaag-aaaagcat**

**Citrus_reticulata_2** **ataccaacaata------tcatact-------------aaatcaaagaaag-aaaagcat**

**Citrus_trifoliata_3** **ataccaacaata------tcatact-------------aaatcaaagaaag-aaaagcat**

**Citrus_aurantiifolia** **ataccaataata------tcatactaaatcaaagaaagaaatcaaagaaag-aaaagcat**

**Citrus_aurantium** **ataccaacaata------tcatact-------------aaatcaaagaaag-aaaagcat**

**Citrus_cavaleriei** **ataccaacaatatcatactcatact-------------aaatcaaagaaag-aaaagcat**

**Citrus_hongheensis** **ataccaacaata------tcatact-------------aaatcaaagaaag-aaaagcat**

**Citrus_limon** **ataccaacaata------tcatact-------------aaatcaaagaaag-aaaagcat**

**Citrus_maxima** **ataccaacaata------tcatact-------------aaatcaaagaaag-aaaagcat**

**Citrus_medica** **ataccaacaata------tcatact-------------aaatcaaagaaag-aaaagcat**

**Citrus_polytrifolia** **ataccaacaata------tcatact-------------aaatcaaagaaag-aaaagcat**

**Citrus_reticulata** **ataccaacaata------tcatact-------------aaatcaaagaaag-aaaagcat**

**Citrus_sinensis** **ataccaacaata------tcatact-------------aaatcaaagaaag-aaaagcat**

**Fortunella_bawangica_1** **ataccaacaata------tcatact-------------aaatcaaagaaag-aaaagcat**

**Fortunella_bawangica_2** **ataccaacaata------tcatact-------------aaatcaaagaaag-aaaagcat**

**Fortunella_hindsii_1** **ataccaacaata------tcatact-------------aaatcaaagaaag-aaaagcat**

**Fortunella_hindsii_2** **ataccaacaata------tcatact-------------aaatcaaagaaag-aaaagcat**

**Fortunella_hindsii_3** **ataccaacaata------tcatact-------------aaatcaaagaaag-aaaagcat**

**Fortunella_japonica_1** **ataccaacaata------tcatact-------------aaatcaaagaaag-aaaagcat**

**Fortunella_japonica_2** **ataccaacaata------tcatact-------------aaatcaaagaaag-aaaagcat**

**Fortunella_japonica_3** **ataccaacaata------tcatact-------------aaatcaaagaaag-aaaagcat**

**Fortunella_japonica_4** **ataccaacaata------tcatact-------------aaatcaaagaaag-aaaagcat**

**Fortunella_japonica_5** **ataccaacaata------tcatact-------------aaatcaaagaaag-aaaagcat**

**Fortunella_japonica_6** **ataccaacaata------tcatact-------------aaatcaaagaaag-aaaagcat**

**Fortunella_japonica_7** **ataccaacaata------tcatact-------------aaatcaaagaaag-aaaagcat**

**Fortunella_japonica_8** **ataccaacaata------tcatact-------------aaatcaaagaaag-aaaagcat**

**Fortunella_japonica_9** **ataccaacaata------tcatact-------------aaatcaaagaaag-aaaagcat**

**Fortunella_japonica_10** **ataccaacaata------tcatact-------------aaatcaaagaaag-aaaagcat**

**Fortunella_japonica_11** **ataccaacaata------tcatact-------------aaatcaaagaaag-aaaagcat**

**Fortunella_japonica_12** **ataccaacaata------tcatact-------------aaatcaaagaaag-aaaagcat**

**Fortunella_margarita_1** **ataccaacaata------tcatact-------------aaatcaaagaaag-aaaagcat**

**Fortunella_margarita_2** **ataccaacaata------tcatact-------------aaatcaaagaaag-aaaagcat**

**Fortunella_venosa_1** **ataccaacaata------tcatact-------------aaatcaaagaaag-aaaagcat**

**Fortunella_venosa_2** **ataccaacaata------tcatact-------------aaatcaaagaaag-aaaagcat**

**Fortunella_venosa_3** **ataccaacaata------tcatact-------------aaatcaaagaaag-aaaagcat**

430	440	450	460	470	480

....|....|....|....|....|....|....|....|....|....|....|....|

**Atalantia_kwangtungens** **aaaaatact-----t----aaaaaaaaaaaaaaaatgaactaaaaaactactaaagaacc**

**Citrus_reticulata_1** **aaaaatact-----t-------aaaaaaaaaaaaatgaact-aaaaactaataaagaacc**

**Citrus_reticulata_2** **aaaaatact-----t-------aaaaaaaaaaaaatgaact-aaaaactaataaagaacc**

**Citrus_trifoliata_3** **aaaaataataaaaaa-aaaaaaaaaaaaaaaaaaatgaatt-aaaaactaataaagaacc**

**Citrus_aurantiifolia** **aaaaatact-----t------aaaaaaaaaaaaaatgaact-aaaaactaataaagaacc**

**Citrus_aurantium** **aaaaatact-----t-aaacaaaaaaaaaaaaaaatgaact-aaaaactaataaagaacc**

**Citrus_cavaleriei** **aaaaatact-----t-------aaaaaaaaaaaaatgaact-aaaaactaataaagaacc**

**Citrus_hongheensis** **aaaaatact-----t-aaacaaaaaaaaaaaaa---gaact-aaaaactaataaagaacc**

**Citrus_limon** **aaaaatact-----t-aaacaaaaaaaaaaaaaaatgaact-aaaaactaataaagaacc**

**Citrus_maxima** **aaaaatact-----t-aaacaaaaaaaaaaaaaaatgaact-aaaaactaataaagaacc**

**Citrus_medica** **aaaaatact-----t----------aaaaaaaaaatgaact-aaaaactaataaagaacc**

**Citrus_polytrifolia** **aaaaatact-----t-acaaaaaaaaaaaaaaaaatgaatt-aaaaactaataaagaacc**

**Citrus_reticulata** **aaaaatact-----t-------aaaaaaaaaaaaatgaact-aaaaactaataaagaacc**

**Citrus_sinensis** **aaaaatact-----t-aaac-aaaaaaaaaaaaaatgaact-aaaaactaataaagaacc**

**Fortunella_bawangica_1** **aaaaatact-----t---aaaaaaaaaaaaaaaaatgaact-aaaaactaataaagaacc**

**Fortunella_bawangica_2** **aaaaatact-----t---aaaaaaaaaaaaaaaaatgaact-aaaaactaataaagaacc**

**Fortunella_hindsii_1** **aaaaatact-----t-aaaaaaaaaaaaaaaaaaatgaact-aaaaactaataaagaacc**

**Fortunella_hindsii_2** **aaaaatact-----t-aaaaaaaaaaaaaaaaaaatgaact-aaaaactaataaagaacc**

**Fortunella_hindsii_3** **aaaaatact-----taaaaaaaaaaaaaaaaaaaatgaact-aaaaactaataaagaacc**

**Fortunella_japonica_1** **aaaaatact-----t-aaaaaa-aaaaaaaaaaaatgaact-aaaaactaataaagaacc**

**Fortunella_japonica_2** **aaaaatact-----t-aaaaa--aaaaaaaaaaaatgaact-aaaaactaataaagaacc**

**Fortunella_japonica_3** **aaaaatact-----t-aaaaaa-aaaaaaaaaaaatgaact-aaaaactaataaagaacc**

**Fortunella_japonica_4** **aaaaatact-----t-aaaaaa-aaaaaaaaaaaatgaact-aaaaactaataaagaacc**

**Fortunella_japonica_5** **aaaaatact-----t-aaaaaa-aaaaaaaaaaaatgaact-aaaaactaataaagaacc**

**Fortunella_japonica_6** **aaaaatact-----t-aaaaaa-aaaaaaaaaaaatgaact-aaaaactaataaagaacc**

**Fortunella_japonica_7** **aaaaatact-----t-aaaaaa-aaaaaaaaaaaatgaact-aaaaactaataaagaacc**

**Fortunella_japonica_8** **aaaaatact-----t-aaaaaa-aaaaaaaaaaaatgaact-aaaaactaataaagaacc**

**Fortunella_japonica_9** **aaaaatact-----t-aaaaaa-aaaaaaaaaaaatgaact-aaaaactaataaagaacc**

**Fortunella_japonica_10** **aaaaatact-----t---aaaaaaaaaaaaaaaaatgaact-aaaaactaataaagaacc**

**Fortunella_japonica_11** **aaaaatact-----t-aaaaaaaaaaaaaaaaaaatgaact-aaaaactaataaagaacc**

**Fortunella_japonica_12** **aaaaatact-----t-aaaaaa-aaaaaaaaaaaatgaact-aaaaactaataaagaacc**

**Fortunella_margarita_1** **aaaaatact-----t---aaaaaaaaaaaaaaaaatgaact-aaaaactaataaagaacc**

**Fortunella_margarita_2** **aaaaatact-----t---aaaaaaaaaaaaaaaaatgaact-aaaaactaataaagaacc**

**Fortunella_venosa_1** **aaaaatact-----t-----aaaaaaaaaaaaaaatgaact-aaaaactaataaagaacc**

**Fortunella_venosa_2** **aaaaatact-----t---aaaaaaaaaaaaaaaaatgaact-aaaaactaataaagaacc**

**Fortunella_venosa_3** **aaaaatact-----t-aaaaaaaaaaaaaaaaaaatgaact-aaaaactaataaagaacc**

490	500	510	520	530	540

....|....|....|....|....|....|....|....|....|....|....|....|

**Atalantia_kwangtungens** **ccgataaagaaacccg-------actaaataacggatcaatactgacccccagct-gggg**

**Citrus_reticulata_1** **ccgataaagaaacccg-------actaaataacggatcaatactgacccccagctggggg**

**Citrus_reticulata_2** **ccgataaagaaacccg-------actaaataacggatcaatactgacccccagctggggg**

**Citrus_trifoliata_3** **ccgataaagaaacccgactaaatactaaataacggatcaatactgacccccagctggggg**

**Citrus_aurantiifolia** **ccgataaagaaacccg-------actaaataacggatcaatactgacccccagctggggg**

**Citrus_aurantium** **ccgataaagaaacccg-------actaaataacggatcaatactgacccccagctggggg**

**Citrus_cavaleriei** **ccgataaagaaacccg-------actaaataacggatcaatactgacccccagctggggg**

**Citrus_hongheensis** **ccgataaagaaacccg-------actaaataacggatcaatactgacccccagctggggg**

**Citrus_limon** **ccgataaagaaacccg-------actaaataacggatcaatactgacccccagctggggg**

**Citrus_maxima** **ccgataaagaaacccg-------actaaataacggatcaatactgacccccagctggggg**

**Citrus_medica** **ccgataaagaaacc--------gactaaataacggatcaatactgacccccagctggggg**

**Citrus_polytrifolia** **ccgataaagaaacccgactaaatactaaataacggatcaatactgaccccccgctggggg**

**Citrus_reticulata** **ccgataaagaaacccg-------actaaataacggatcaatactgacccccagctggggg**

**Citrus_sinensis** **ccgataaagaaacccg-------actaaataacggatcaatactgacccccagctggggg**

**Fortunella_bawangica_1** **ccgataaagaaacccg-------actaaataacggatcaatactgacccccagctggggg**

**Fortunella_bawangica_2** **ccgataaagaaacccg-------actaaataacggatcaatactgacccccagctggggg**

**Fortunella_hindsii_1** **ccgataaagaaacccg-------actaaataacggatcaatactgacccccagctggggg**

**Fortunella_hindsii_2** **ccgataaagaaacccg-------actaaataacggatcaatactgacccccagctggggg**

**Fortunella_hindsii_3** **ccgataaagaaacccg-------actaaataacggatcaatactgacccccagctggggg**

**Fortunella_japonica_1** **ccgataaagaaacccg-------actaaataacggatcaatactgacccccagctggggg**

**Fortunella_japonica_2** **ccgataaagaaacccg-------actaaataacggatcaatactgacccccagctggggg**

**Fortunella_japonica_3** **ccgataaagaaacccg-------actaaataacggatcaatactgacccccagctggggg**

**Fortunella_japonica_4** **ccgataaagaaacccg-------actaaataacggatcaatactgacccccagctggggg**

**Fortunella_japonica_5** **ccgataaagaaacccg-------actaaataacggatcaatactgacccccagctggggg**

**Fortunella_japonica_6** **ccgataaagaaacccg-------actaaataacggatcaatactgacccccagctggggg**

**Fortunella_japonica_7** **ccgataaagaaacccg-------actaaataacggatcaatactgacccccagctggggg**

**Fortunella_japonica_8** **ccgataaagaaacccg-------actaaataacggatcaatactgacccccagctggggg**

**Fortunella_japonica_9** **ccgataaagaaacccg-------actaaataacggatcaatactgacccccagctggggg**

**Fortunella_japonica_10** **ccgataaagaaacccg-------actaaataacggatcaatactgacccccagctggggg**

**Fortunella_japonica_11** **ccgataaagaaacccg-------actaaataacggatcaatactgacccccagctggggg**

**Fortunella_japonica_12** **ccgataaagaaacccg-------actaaataacggatcaatactgacccccagctggggg**

**Fortunella_margarita_1** **ccgataaagaaacccg-------actaaataacggatcaatactgacccccagctggggg**

**Fortunella_margarita_2** **ccgataaagaaacccg-------actaaataacggatcaatactgacccccagctggggg**

**Fortunella_venosa_1** **ccgataaagaaacccg-------actaaataacggatcaatactgacccccagctggggg**

**Fortunella_venosa_2** **ccgataaagaaacccg-------actaaataacggatcaatactgacccccagctggggg**

**Fortunella_venosa_3** **ccgataaagaaacccg-------actaaataacggatcaatactgacccccagctggggg**

550	560	570	580	590	600

....|....|....|....|....|....|....|....|....|....|....|....|

**Atalantia_kwangtungens** **tcaatattgatccgttattttcaaaaacccgcctacacaaagaccaaaatcttatccatt**

**Citrus_reticulata_1** **tcagtattgatccgttattttcaaaaacccgcctacacaaagaccaaaatcttacccatt**

**Citrus_reticulata_2** **tcagtattgatccgttattttcaaaaacccgcctacacaaagaccaaaatcttacccatt**

**Citrus_trifoliata_3** **tcagtattgatccgttattttcaaaaacccgcctacacaaagaccaaaatcttacccatt**

**Citrus_aurantiifolia** **tcagtattgatccgttattttcaaaaacccgcctacacaaagaccaaaatcttacccatt**

**Citrus_aurantium** **tcagtattgatccgttattttcaaaaacccgcctacacaaagaccaaaatcttacccatt**

**Citrus_cavaleriei** **tcagtattgatccgttattttcaaaaacccgcctacacaaagaccaaaatcttacccatt**

**Citrus_hongheensis** **tcagtattgatccgttattttcaaaaacccgcctacacaaagaccaaaatcttacccatt**

**Citrus_limon** **tcagtattgatccgttattttcaaaaacccgcctacacaaagaccaaaatcttacccatt**

**Citrus_maxima** **tcagtattgatccgttattttcaaaaacccgcctacacaaagaccaaaatcttacccatt**

**Citrus_medica** **tcagtattgatccgttattttcaaaaacccgcctacacaaagaccaaaatcttacccatt**

**Citrus_polytrifolia** **tcagtattgatccgttattttcaaaaacccgcctacacaaagaccaaaatcttacccatt**

**Citrus_reticulata** **tcagtattgatccgttattttcaaaaacccgcctacacaaagaccaaaatcttacccatt**

**Citrus_sinensis** **tcagtattgatccgttattttcaaaaacccgcctacacaaagaccaaaatcttacccatt**

**Fortunella_bawangica_1** **tcagtattgatccgttattttcaaaaacccgcctacacaaagaccaaaatcttacccatt**

**Fortunella_bawangica_2** **tcagtattgatccgttattttcaaaaacccgcctacacaaagaccaaaatcttacccatt**

**Fortunella_hindsii_1** **tcagtattgatccgttattttcaaaaacccgcctacacaaagaccaaaatcttacccatt**

**Fortunella_hindsii_2** **tcagtattgatccgttattttcaaaaacccgcctacacaaagaccaaaatcttacccatt**

**Fortunella_hindsii_3** **tcagtattgatccgttattttcaaaaacccgcctacacaaagaccaaaatcttacccatt**

**Fortunella_japonica_1** **tcagtattgatccgttattttcaaaaacccgcctacacaaagaccaaaatcttacccatt**

**Fortunella_japonica_2** **tcagtattgatccgttattttcaaaaacccgcctacacaaagaccaaaatcttacccatt**

**Fortunella_japonica_3** **tcagtattgatccgttattttcaaaaacccgcctacacaaagaccaaaatcttacccatt**

**Fortunella_japonica_4** **tcagtattgatccgttattttcaaaaacccgcctacacaaagaccaaaatcttacccatt**

**Fortunella_japonica_5** **tcagtattgatccgttattttcaaaaacccgcctacacaaagaccaaaatcttacccatt**

**Fortunella_japonica_6** **tcagtattgatccgttattttcaaaaacccgcctacacaaagaccaaaatcttacccatt**

**Fortunella_japonica_7** **tcagtattgatccgttattttcaaaaacccgcctacacaaagaccaaaatcttacccatt**

**Fortunella_japonica_8** **tcagtattgatccgttattttcaaaaacccgcctacacaaagaccaaaatcttacccatt**

**Fortunella_japonica_9** **tcagtattgatccgttattttcaaaaacccgcctacacaaagaccaaaatcttacccatt**

**Fortunella_japonica_10** **tcagtattgatccgttattttcaaaaacccgcctacacaaagaccaaaatcttacccatt**

**Fortunella_japonica_11** **tcagtattgatccgttattttcaaaaacccgcctacacaaagaccaaaatcttacccatt**

**Fortunella_japonica_12** **tcagtattgatccgttattttcaaaaacccgcctacacaaagaccaaaatcttacccatt**

**Fortunella_margarita_1** **tcagtattgatccgttattttcaaaaacccgcctacacaaagaccaaaatcttacccatt**

**Fortunella_margarita_2** **tcagtattgatccgttattttcaaaaacccgcctacacaaagaccaaaatcttacccatt**

**Fortunella_venosa_1** **tcagtattgatccgttattttcaaaaacccgcctacacaaagaccaaaatcttacccatt**

**Fortunella_venosa_2** **tcagtattgatccgttattttcaaaaacccgcctacacaaagaccaaaatcttacccatt**

**Fortunella_venosa_3** **tcagtattgatccgttattttcaaaaacccgcctacacaaagaccaaaatcttacccatt**

610	620	630	640	650	660

....|....|....|....|....|....|....|....|....|....|....|....|

**Atalantia_kwangtungens** **tgtagatggggcttcaatagcagctaggtctagagggaagttatgagcattacgttcatg**

**Citrus_reticulata_1** **tgtagatggggcttcaatagcagctaggtctagagggaagttatgagcattacgttcatg**

**Citrus_reticulata_2** **tgtagatggggcttcaatagcagctaggtctagagggaagttatgagcattacgttcatg**

**Citrus_trifoliata_3** **tgtagatggggcttcaatagcagctaggtctagagggaagttatgagcattacgttcatg**

**Citrus_aurantiifolia** **tgtagatggggcttcaatagcagctaggtctagagggaagttatgagcattacgttcatg**

**Citrus_aurantium** **tgtagatggggcttcaatagcagctaggtctagagggaagttatgagcattacgttcatg**

**Citrus_cavaleriei** **tgtagatggggcttcaatagcagctaggtctagagggaagttatgagcattacgttcatg**

**Citrus_hongheensis** **tgtagatggggcttcaatagcagctaggtctagagggaagttatgagcattacgttcatg**

**Citrus_limon** **tgtagatggggcttcaatagcagctaggtctagagggaagttatgagcattacgttcatg**

**Citrus_maxima** **tgtagatggggcttcaatagcagctaggtctagagggaagttatgagcattacgttcatg**

**Citrus_medica** **tgtagatggggcttcaatagcagctaggtctagagggaagttatgagcattacgttcatg**

**Citrus_polytrifolia** **tgtagatggggcttcaatagcagctaggtctagagggaagttatgagcattacgttcatg**

**Citrus_reticulata** **tgtagatggggcttcaatagcagctaggtctagagggaagttatgagcattacgttcatg**

**Citrus_sinensis** **tgtagatggggcttcaatagcagctaggtctagagggaagttatgagcattacgttcatg**

**Fortunella_bawangica_1** **tgtagatggggcttcaatagcagctaggtctagagggaagttatgagcattacgttcatg**

**Fortunella_bawangica_2** **tgtagatggggcttcaatagcagctaggtctagagggaagttatgagcattacgttcatg**

**Fortunella_hindsii_1** **tgtagatggggcttcaatagcagctaggtctagagggaagttatgagcattacgttcatg**

**Fortunella_hindsii_2** **tgtagatggggcttcaatagcagctaggtctagagggaagttatgagcattacgttcatg**

**Fortunella_hindsii_3** **tgtagatggggcttcaatagcagctaggtctagagggaagttatgagcattacgttcatg**

**Fortunella_japonica_1** **tgtagatggggcttcaatagcagctaggtctagagggaagttatgagcattacgttcatg**

**Fortunella_japonica_2** **tgtagatggggcttcaatagcagctaggtctagagggaagttatgagcattacgttcatg**

**Fortunella_japonica_3** **tgtagatggggcttcaatagcagctaggtctagagggaagttatgagcattacgttcatg**

**Fortunella_japonica_4** **tgtagatggggcttcaatagcagctaggtctagagggaagttatgagcattacgttcatg**

**Fortunella_japonica_5** **tgtagatggggcttcaatagcagctaggtctagagggaagttatgagcattacgttcatg**

**Fortunella_japonica_6** **tgtagatggggcttcaatagcagctaggtctagagggaagttatgagcattacgttcatg**

**Fortunella_japonica_7** **tgtagatggggcttcaatagcagctaggtctagagggaagttatgagcattacgttcatg**

**Fortunella_japonica_8** **tgtagatggggcttcaatagcagctaggtctagagggaagttatgagcattacgttcatg**

**Fortunella_japonica_9** **tgtagatggggcttcaatagcagctaggtctagagggaagttatgagcattacgttcatg**

**Fortunella_japonica_10** **tgtagatggggcttcaatagcagctaggtctagagggaagttatgagcattacgttcatg**

**Fortunella_japonica_11** **tgtagatggggcttcaatagcagctaggtctagagggaagttatgagcattacgttcatg**

**Fortunella_japonica_12** **tgtagatggggcttcaatagcagctaggtctagagggaagttatgagcattacgttcatg**

**Fortunella_margarita_1** **tgtagatggggcttcaatagcagctaggtctagagggaagttatgagcattacgttcatg**

**Fortunella_margarita_2** **tgtagatggggcttcaatagcagctaggtctagagggaagttatgagcattacgttcatg**

**Fortunella_venosa_1** **tgtagatggggcttcaatagcagctaggtctagagggaagttatgagcattacgttcatg**

**Fortunella_venosa_2** **tgtagatggggcttcaatagcagctaggtctagagggaagttatgagcattacgttcatg**

**Fortunella_venosa_3** **tgtagatggggcttcaatagcagctaggtctagagggaagttatgagcattacgttcatg**

670	680	690	700	710	720

....|....|....|....|....|....|....|....|....|....|....|....|

**Atalantia_kwangtungens** **cataacttccataccaaggttagcacgattaataatatcagcccaggtattaattacacg**

**Citrus_reticulata_1** **cataacttccataccaaggttagcacgattaataatatcagcccaggtattaattacacg**

**Citrus_reticulata_2** **cataacttccataccaaggttagcacgattaataatatcagcccaggtattaattacacg**

**Citrus_trifoliata_3** **cataacttccataccaaggttagcacgattaataatatcagcccaggtattaattacacg**

**Citrus_aurantiifolia** **cataacttccataccaaggttagcacgattaataatatcagcccaggtattaattacacg**

**Citrus_aurantium** **cataacttccataccaaggttagcacgattaataatatcagcccaggtattaattacacg**

**Citrus_cavaleriei** **cataacttccataccaaggttagcacgattaataatatcagcccaggtattaattacacg**

**Citrus_hongheensis** **cataacttccataccaaggttagcacgattaataatatcagcccaggtattaattacacg**

**Citrus_limon** **cataacttccataccaaggttagcacgattaataatatcagcccaggtattaattacacg**

**Citrus_maxima** **cataacttccataccaaggttagcacgattaataatatcagcccaggtattaattacacg**

**Citrus_medica** **cataacttccataccaaggttagcacgattaataatatcagcccaggtattaattacacg**

**Citrus_polytrifolia** **cataacttccataccaaggttagcacgattaataatatcagcccaggtattaattacacg**

**Citrus_reticulata** **cataacttccataccaaggttagcacgattaataatatcagcccaggtattaattacacg**

**Citrus_sinensis** **cataacttccataccaaggttagcacgattaataatatcagcccaggtattaattacacg**

**Fortunella_bawangica_1** **cataacttccataccaaggttagcacgattaataatatcagcccaggtattaattacacg**

**Fortunella_bawangica_2** **cataacttccataccaaggttagcacgattaataatatcagcccaggtattaattacacg**

**Fortunella_hindsii_1** **cataacttccataccaaggttagcacgattaataatatcagcccaggtattaattacacg**

**Fortunella_hindsii_2** **cataacttccataccaaggttagcacgattaataatatcagcccaggtattaattacacg**

**Fortunella_hindsii_3** **cataacttccataccaaggttagcacgattaataatatcagcccaggtattaattacacg**

**Fortunella_japonica_1** **cataacttccataccaaggttagcacgattaataatatcagcccaggtattaattacacg**

**Fortunella_japonica_2** **cataacttccataccaaggttagcacgattaataatatcagcccaggtattaattacacg**

**Fortunella_japonica_3** **cataacttccataccaaggttagcacgattaataatatcagcccaggtattaattacacg**

**Fortunella_japonica_4** **cataacttccataccaaggttagcacgattaataatatcagcccaggtattaattacacg**

**Fortunella_japonica_5** **cataacttccataccaaggttagcacgattaataatatcagcccaggtattaattacacg**

**Fortunella_japonica_6** **cataacttccataccaaggttagcacgattaataatatcagcccaggtattaattacacg**

**Fortunella_japonica_7** **cataacttccataccaaggttagcacgattaataatatcagcccaggtattaattacacg**

**Fortunella_japonica_8** **cataacttccataccaaggttagcacgattaataatatcagcccaggtattaattacacg**

**Fortunella_japonica_9** **cataacttccataccaaggttagcacgattaataatatcagcccaggtattaattacacg**

**Fortunella_japonica_10** **cataacttccataccaaggttagcacgattaataatatcagcccaggtattaattacacg**

**Fortunella_japonica_11** **cataacttccataccaaggttagcacgattaataatatcagcccaggtattaattacacg**

**Fortunella_japonica_12** **cataacttccataccaaggttagcacgattaataatatcagcccaggtattaattacacg**

**Fortunella_margarita_1** **cataacttccataccaaggttagcacgattaataatatcagcccaggtattaattacacg**

**Fortunella_margarita_2** **cataacttccataccaaggttagcacgattaataatatcagcccaggtattaattacacg**

**Fortunella_venosa_1** **cataacttccataccaaggttagcacgattaataatatcagcccaggtattaattacacg**

**Fortunella_venosa_2** **cataacttccataccaaggttagcacgattaataatatcagcccaggtattaattacacg**

**Fortunella_venosa_3** **cataacttccataccaaggttagcacgattaataatatcagcccaggtattaattacacg**

730	740	750	760	770	780

....|....|....|....|....|....|....|....|....|....|....|....|

**Atalantia_kwangtungens** **accttgactatcaactacagattggttgaaattgaaaccatttaggttgaaagccatagt**

**Citrus_reticulata_1** **accttgactatcaactacagattggttgaaattgaaaccatttaagttgaaagccatagt**

**Citrus_reticulata_2** **accttgactatcaactacagattggttgaaattgaaaccatttaagttgaaagccatagt**

**Citrus_trifoliata_3** **accttgactatcaactacagattggttgaaattgaaaccatttaagttgaaagccatagt**

**Citrus_aurantiifolia** **accttgactatcaactacagattggttgaaattgaaaccatttaagttgaaagccatagt**

**Citrus_aurantium** **accttgactatcaactacagattggttgaaattgaaaccatttaagttgaaagccatagt**

**Citrus_cavaleriei** **accttgactatcaactacagattggttgaaattgaaaccatttaagttgaaagccatagt**

**Citrus_hongheensis** **accttgactatcaactacagattggttgaaattgaaaccatttaagttgaaagccatagt**

**Citrus_limon** **accttgactatcaactacagattggttgaaattgaaaccatttaagttgaaagccatagt**

**Citrus_maxima** **accttgactatcaactacagattggttgaaattgaaaccatttaagttgaaagccatagt**

**Citrus_medica** **accttgactatcaactacagattggttgaaattgaaaccatttaagttgaaagccatagt**

**Citrus_polytrifolia** **accttgactatcaactacagattggttgaaattgaaaccatttaagttgaaagccatagt**

**Citrus_reticulata** **accttgactatcaactacagattggttgaaattgaaaccatttaagttgaaagccatagt**

**Citrus_sinensis** **accttgactatcaactacagattggttgaaattgaaaccatttaagttgaaagccatagt**

**Fortunella_bawangica_1** **accttgactatcaactacagattggttgaaattgaaaccatttaagttgaaagccatagt**

**Fortunella_bawangica_2** **accttgactatcaactacagattggttgaaattgaaaccatttaagttgaaagccatagt**

**Fortunella_hindsii_1** **accttgactatcaactacagattggttgaaattgaaaccatttaagttgaaagccatagt**

**Fortunella_hindsii_2** **accttgactatcaactacagattggttgaaattgaaaccatttaagttgaaagccatagt**

**Fortunella_hindsii_3** **accttgactatcaactacagattggttgaaattgaaaccatttaagttgaaagccatagt**

**Fortunella_japonica_1** **accttgactatcaactacagattggttgaaattgaaaccatttaagttgaaagccatagt**

**Fortunella_japonica_2** **accttgactatcaactacagattggttgaaattgaaaccatttaagttgaaagccatagt**

**Fortunella_japonica_3** **accttgactatcaactacagattggttgaaattgaaaccatttaagttgaaagccatagt**

**Fortunella_japonica_4** **accttgactatcaactacagattggttgaaattgaaaccatttaagttgaaagccatagt**

**Fortunella_japonica_5** **accttgactatcaactacagattggttgaaattgaaaccatttaagttgaaagccatagt**

**Fortunella_japonica_6** **accttgactatcaactacagattggttgaaattgaaaccatttaagttgaaagccatagt**

**Fortunella_japonica_7** **accttgactatcaactacagattggttgaaattgaaaccatttaagttgaaagccatagt**

**Fortunella_japonica_8** **accttgactatcaactacagattggttgaaattgaaaccatttaagttgaaagccatagt**

**Fortunella_japonica_9** **accttgactatcaactacagattggttgaaattgaaaccatttaagttgaaagccatagt**

**Fortunella_japonica_10** **accttgactatcaactacagattggttgaaattgaaaccatttaagttgaaagccatagt**

**Fortunella_japonica_11** **accttgactatcaactacagattggttgaaattgaaaccatttaagttgaaagccatagt**

**Fortunella_japonica_12** **accttgactatcaactacagattggttgaaattgaaaccatttaagttgaaagccatagt**

**Fortunella_margarita_1** **accttgactatcaactacagattggttgaaattgaaaccatttaagttgaaagccatagt**

**Fortunella_margarita_2** **accttgactatcaactacagattggttgaaattgaaaccatttaagttgaaagccatagt**

**Fortunella_venosa_1** **accttgactatcaactacagattggttgaaattgaaaccatttaagttgaaagccatagt**

**Fortunella_venosa_2** **accttgactatcaactacagattggttgaaattgaaaccatttaagttgaaagccatagt**

**Fortunella_venosa_3** **accttgactatcaactacagattggttgaaattgaaaccatttaagttgaaagccatagt**

790	800	810	820	830	840

....|....|....|....|....|....|....|....|....|....|....|....|

**Atalantia_kwangtungens** **gctaatacctaaagcagtgaaccagatacctactacaggccaagcagccaggaagaaatg**

**Citrus_reticulata_1** **gctaatacctaaagcagtgaaccagatacctactacaggccaagcagccaggaagaaatg**

**Citrus_reticulata_2** **gctaatacctaaagcagtgaaccagatacctactacaggccaagcagccaggaagaaatg**

**Citrus_trifoliata_3** **gctaatacctaaagcagtgaaccagatacctactacaggccaagcagccaggaagaaatg**

**Citrus_aurantiifolia** **gctaatacctaaagcagtgaaccagatacctactacaggccaagcagccaggaagaaatg**

**Citrus_aurantium** **gctaatacctaaagcagtgaaccagatacctactacaggccaagcagccaggaagaaatg**

**Citrus_cavaleriei** **gctaatacctaaagcagtgaaccagatacctactacaggccaagcagccaggaagaaatg**

**Citrus_hongheensis** **gctaatacctaaagcagtgaaccagatacctactacaggccaagcagccaggaagaaatg**

**Citrus_limon** **gctaatacctaaagcagtgaaccagatacctactacaggccaagcagccaggaagaaatg**

**Citrus_maxima** **gctaatacctaaagcagtgaaccagatacctactacaggccaagcagccaggaagaaatg**

**Citrus_medica** **gctaatacctaaagcagtgaaccagatacctactacaggccaagcagccaggaagaaatg**

**Citrus_polytrifolia** **gctaatacctaaagcagtgaaccagatacctactacaggccaagcagccaggaagaaatg**

**Citrus_reticulata** **gctaatacctaaagcagtgaaccagatacctactacaggccaagcagccaggaagaaatg**

**Citrus_sinensis** **gctaatacctaaagcagtgaaccagatacctactacaggccaagcagccaggaagaaatg**

**Fortunella_bawangica_1** **gctaatacctaaagaagtgaaccagatacctactacaggccaagcagccaggaagaaatg**

**Fortunella_bawangica_2** **gctaatacctaaagaagtgaaccagatacctactacaggccaagcagccaggaagaaatg**

**Fortunella_hindsii_1** **gctaatacctaaagaagtgaaccagatacctactacaggccaagcagccaggaagaaatg**

**Fortunella_hindsii_2** **gctaatacctaaagaagtgaaccagatacctactacaggccaagcagccaggaagaaatg**

**Fortunella_hindsii_3** **gctaatacctaaagaagtgaaccagatacctactacaggccaagcagccaggaagaaatg**

**Fortunella_japonica_1** **gctaatacctaaagaagtgaaccagatacctactacaggccaagcagccaggaagaaatg**

**Fortunella_japonica_2** **gctaatacctaaagaagtgaaccagatacctactacaggccaagcagccaggaagaaatg**

**Fortunella_japonica_3** **gctaatacctaaagaagtgaaccagatacctactacaggccaagcagccaggaagaaatg**

**Fortunella_japonica_4** **gctaatacctaaagaagtgaaccagatacctactacaggccaagcagccaggaagaaatg**

**Fortunella_japonica_5** **gctaatacctaaagaagtgaaccagatacctactacaggccaagcagccaggaagaaatg**

**Fortunella_japonica_6** **gctaatacctaaagaagtgaaccagatacctactacaggccaagcagccaggaagaaatg**

**Fortunella_japonica_7** **gctaatacctaaagaagtgaaccagatacctactacaggccaagcagccaggaagaaatg**

**Fortunella_japonica_8** **gctaatacctaaagaagtgaaccagatacctactacaggccaagcagccaggaagaaatg**

**Fortunella_japonica_9** **gctaatacctaaagaagtgaaccagatacctactacaggccaagcagccaggaagaaatg**

**Fortunella_japonica_10** **gctaatacctaaagaagtgaaccagatacctactacaggccaagcagccaggaagaaatg**

**Fortunella_japonica_11** **gctaatacctaaagaagtgaaccagatacctactacaggccaagcagccaggaagaaatg**

**Fortunella_japonica_12** **gctaatacctaaagaagtgaaccagatacctactacaggccaagcagccaggaagaaatg**

**Fortunella_margarita_1** **gctaatacctaaagaagtgaaccagatacctactacaggccaagcagccaggaagaaatg**

**Fortunella_margarita_2** **gctaatacctaaagaagtgaaccagatacctactacaggccaagcagccaggaagaaatg**

**Fortunella_venosa_1** **gctaatacctaaagaagtgaaccagatacctactacaggccaagcagccaggaagaaatg**

**Fortunella_venosa_2** **gctaatacctaaagaagtgaaccagatacctactacaggccaagcagccaggaagaaatg**

**Fortunella_venosa_3** **gctaatacctaaagaagtgaaccagatacctactacaggccaagcagccaggaagaaatg**

850	860	870	880	890	900

....|....|....|....|....|....|....|....|....|....|....|....|

**Atalantia_kwangtungens** **taaagaacgagaattgttgaaactagcatattggaagatcaatcggccaaaataaccgtg**

**Citrus_reticulata_1** **taaagaacgagaattgttgaaactagcatattggaagatcaatcggccaaaataaccgtg**

**Citrus_reticulata_2** **taaagaacgagaattgttgaaactagcatattggaagatcaatcggccaaaataaccgtg**

**Citrus_trifoliata_3** **taaagaacgagaattgttgaaactagcatattggaagatcaatcggccaaaataaccgtg**

**Citrus_aurantiifolia** **taaagaacgagaattgttgaaactagcatattggaagatcaatcggccaaaataaccgtg**

**Citrus_aurantium** **taaagaacgagaattgttgaaactagcatattggaagatcaatcggccaaaataaccgtg**

**Citrus_cavaleriei** **taaagaacgagaattgttgaaactagcatattggaagatcaatcggccaaaataaccgtg**

**Citrus_hongheensis** **taaagaacgagaattgttgaaactagcatattggaagatcaatcggccaaaataaccgtg**

**Citrus_limon** **taaagaacgagaattgttgaaactagcatattggaagatcaatcggccaaaataaccgtg**

**Citrus_maxima** **taaagaacgagaattgttgaaactagcatattggaagatcaatcggccaaaataaccgtg**

**Citrus_medica** **taaagaacgagaattgttgaaactagcatattggaagatcaatcggccaaaataaccgtg**

**Citrus_polytrifolia** **taaagaacgagaattgttgaaactagcatattggaagatcaatcggccaaaataaccgtg**

**Citrus_reticulata** **taaagaacgagaattgttgaaactagcatattggaagatcaatcggccaaaataaccgtg**

**Citrus_sinensis** **taaagaacgagaattgttgaaactagcatattggaagatcaatcggccaaaataaccgtg**

**Fortunella_bawangica_1** **taaagaacgagaattgttgaaactagcatattggaagatcaatcggccaaaataaccgtg**

**Fortunella_bawangica_2** **taaagaacgagaattgttgaaactagcatattggaagatcaatcggccaaaataaccgtg**

**Fortunella_hindsii_1** **taaagaacgagaattgttgaaactagcatattggaagatcaatcggccaaaataaccgtg**

**Fortunella_hindsii_2** **taaagaacgagaattgttgaaactagcatattggaagatcaatcggccaaaataaccgtg**

**Fortunella_hindsii_3** **taaagaacgagaattgttgaaactagcatattggaagatcaatcggccaaaataaccgtg**

**Fortunella_japonica_1** **taaagaacgagaattgttgaaactagcatattggaagatcaatcggccaaaataaccgtg**

**Fortunella_japonica_2** **taaagaacgagaattgttgaaactagcatattggaagatcaatcggccaaaataaccgtg**

**Fortunella_japonica_3** **taaagaacgagaattgttgaaactagcatattggaagatcaatcggccaaaataaccgtg**

**Fortunella_japonica_4** **taaagaacgagaattgttgaaactagcatattggaagatcaatcggccaaaataaccgtg**

**Fortunella_japonica_5** **taaagaacgagaattgttgaaactagcatattggaagatcaatcggccaaaataaccgtg**

**Fortunella_japonica_6** **taaagaacgagaattgttgaaactagcatattggaagatcaatcggccaaaataaccgtg**

**Fortunella_japonica_7** **taaagaacgagaattgttgaaactagcatattggaagatcaatcggccaaaataaccgtg**

**Fortunella_japonica_8** **taaagaacgagaattgttgaaactagcatattggaagatcaatcggccaaaataaccgtg**

**Fortunella_japonica_9** **taaagaacgagaattgttgaaactagcatattggaagatcaatcggccaaaataaccgtg**

**Fortunella_japonica_10** **taaagaacgagaattgttgaaactagcatattggaagatcaatcggccaaaataaccgtg**

**Fortunella_japonica_11** **taaagaacgagaattgttgaaactagcatattggaagatcaatcggccaaaataaccgtg**

**Fortunella_japonica_12** **taaagaacgagaattgttgaaactagcatattggaagatcaatcggccaaaataaccgtg**

**Fortunella_margarita_1** **taaagaacgagaattgttgaaactagcatattggaagatcaatcggccaaaataaccgtg**

**Fortunella_margarita_2** **taaagaacgagaattgttgaaactagcatattggaagatcaatcggccaaaataaccgtg**

**Fortunella_venosa_1** **taaagaacgagaattgttgaaactagcatattggaagatcaatcggccaaaataaccgtg**

**Fortunella_venosa_2** **taaagaacgagaattgttgaaactagcatattggaagatcaatcggccaaaataaccgtg**

**Fortunella_venosa_3** **taaagaacgagaattgttgaaactagcatattggaagatcaatcggccaaaataaccgtg**

910	920	930	940	950	960

....|....|....|....|....|....|....|....|....|....|....|....|

**Atalantia_kwangtungens** **agcagctacgatattataagtttcttcctcttgaccgaatctgtaaccttcattagcgga**

**Citrus_reticulata_1** **agcagctacgatattataagtttcttcctcttgaccgaatctgtaacctgcattagcaga**

**Citrus_reticulata_2** **agcagctacgatattataagtttcttcctcttgaccgaatctgtaacctgcattagcaga**

**Citrus_trifoliata_3** **agcagctacgatattataagtttcttcctcttgaccgaatctgtaacctgcattagcaga**

**Citrus_aurantiifolia** **agcagctacgatattataagtttcttcctcttgaccgaatctgtaacctgcattagcaga**

**Citrus_aurantium** **agcagctacgatattataagtttcttcctcttgaccgaatctgtaacctgcattagcaga**

**Citrus_cavaleriei** **agcagctacgatattataagtttcttcctcttgaccgaatctgtaacctgcattagcaga**

**Citrus_hongheensis** **agcagctacgatattataagtttcttcctcttgaccgaatctgtaacctgcattagcaga**

**Citrus_limon** **agcagctacgatattataagtttcttcctcttgaccgaatctgtaacctgcattagcaga**

**Citrus_maxima** **agcagctacgatattataagtttcttcctcttgaccgaatctgtaacctgcattagcaga**

**Citrus_medica** **agcagctacgatattataagtttcttcctcttgaccgaatctgtaacctgcattagcaga**

**Citrus_polytrifolia** **agcagctacgatattataagtttcttcctcttgaccgaatctgtaacctgcattagcaga**

**Citrus_reticulata** **agcagctacgatattataagtttcttcctcttgaccgaatctgtaacctgcattagcaga**

**Citrus_sinensis** **agcagctacgatattataagtttcttcctcttgaccgaatctgtaacctgcattagcaga**

**Fortunella_bawangica_1** **agcagctacgatattataagtttcttcctcttgaccgaatctgtaacctgcattagcaga**

**Fortunella_bawangica_2** **agcagctacgatattataagtttcttcctcttgaccgaatctgtaacctgcattagcaga**

**Fortunella_hindsii_1** **agcagctacgatattataagtttcttcctcttgaccgaatctgtaacctgcattagcaga**

**Fortunella_hindsii_2** **agcagctacgatattataagtttcttcctcttgaccgaatctgtaacctgcattagcaga**

**Fortunella_hindsii_3** **agcagctacgatattataagtttcttcctcttgaccgaatctgtaacctgcattagcaga**

**Fortunella_japonica_1** **agcagctacgatattataagtttcttcctcttgaccgaatctgtaacctgcattagcaga**

**Fortunella_japonica_2** **agcagctacgatattataagtttcttcctcttgaccgaatctgtaacctgcattagcaga**

**Fortunella_japonica_3** **agcagctacgatattataagtttcttcctcttgaccgaatctgtaacctgcattagcaga**

**Fortunella_japonica_4** **agcagctacgatattataagtttcttcctcttgaccgaatctgtaacctgcattagcaga**

**Fortunella_japonica_5** **agcagctacgatattataagtttcttcctcttgaccgaatctgtaacctgcattagcaga**

**Fortunella_japonica_6** **agcagctacgatattataagtttcttcctcttgaccgaatctgtaacctgcattagcaga**

**Fortunella_japonica_7** **agcagctacgatattataagtttcttcctcttgaccgaatctgtaacctgcattagcaga**

**Fortunella_japonica_8** **agcagctacgatattataagtttcttcctcttgaccgaatctgtaacctgcattagcaga**

**Fortunella_japonica_9** **agcagctacgatattataagtttcttcctcttgaccgaatctgtaacctgcattagcaga**

**Fortunella_japonica_10** **agcagctacgatattataagtttcttcctcttgaccgaatctgtaacctgcattagcaga**

**Fortunella_japonica_11** **agcagctacgatattataagtttcttcctcttgaccgaatctgtaacctgcattagcaga**

**Fortunella_japonica_12** **agcagctacgatattataagtttcttcctcttgaccgaatctgtaacctgcattagcaga**

**Fortunella_margarita_1** **agcagctacgatattataagtttcttcctcttgaccgaatctgtaacctgcattagcaga**

**Fortunella_margarita_2** **agcagctacgatattataagtttcttcctcttgaccgaatctgtaacctgcattagcaga**

**Fortunella_venosa_1** **agcagctacgatattataagtttcttcctcttgaccgaatctgtaacctgcattagcaga**

**Fortunella_venosa_2** **agcagctacgatattataagtttcttcctcttgaccgaatctgtaacctgcattagcaga**

**Fortunella_venosa_3** **agcagctacgatattataagtttcttcctcttgaccgaatctgtaacctgcattagcaga**

970	980	990	1000	1010	1020

....|....|....|....|....|....|....|....|....|....|....|....|

**Atalantia_kwangtungens** **ttcattttctgtggtttccctgatcaaactagaggttaccaaggaaccatgcatagcact**

**Citrus_reticulata_1** **ttcattttctgtggtttccctgatcaaactagaggttaccaaggaaccatgcatagcact**

**Citrus_reticulata_2** **ttcattttctgtggtttccctgatcaaactagaggttaccaaggaaccatgcatagcact**

**Citrus_trifoliata_3** **ttcattttctgtggtttccctgatcaaactagaggttaccaaggaaccatgcatagcact**

**Citrus_aurantiifolia** **ttcattttctgtggtttccctgatcaaactagaggttaccaaggaaccatgcatagcact**

**Citrus_aurantium** **ttcattttctgtggtttccctgatcaaactagaggttaccaaggaaccatgcatagcact**

**Citrus_cavaleriei** **ttcattttctgtggtttccctgatcaaactagaggttaccaaggaaccatgcatagcact**

**Citrus_hongheensis** **ttcattttctgtggtttccctgatcaaactagaggttaccaaggaaccatgcatagcact**

**Citrus_limon** **ttcattttctgtggtttccctgatcaaactagaggttaccaaggaaccatgcatagcact**

**Citrus_maxima** **ttcattttctgtggtttccctgatcaaactagaggttaccaaggaaccatgcatagcact**

**Citrus_medica** **ttcattttctgtggtttccctgatcaaactagaggttaccaaggaaccatgcatagcact**

**Citrus_polytrifolia** **ttcattttctgtggtttccctgatcaaactagaggttaccaaggaaccatgcatagcact**

**Citrus_reticulata** **ttcattttctgtggtttccctgatcaaactagaggttaccaaggaaccatgcatagcact**

**Citrus_sinensis** **ttcattttctgtggtttccctgatcaaactagaggttaccaaggaaccatgcatagcact**

**Fortunella_bawangica_1** **ttcattttctgtggtttccctgatcaaactagaggttaccaaggaaccatgcatagcact**

**Fortunella_bawangica_2** **ttcattttctgtggtttccctgatcaaactagaggttaccaaggaaccatgcatagcact**

**Fortunella_hindsii_1** **ttcattttctgtggtttccctgatcaaactagaggttaccaaggaaccatgcatagcact**

**Fortunella_hindsii_2** **ttcattttctgtggtttccctgatcaaactagaggttaccaaggaaccatgcatagcact**

**Fortunella_hindsii_3** **ttcattttctgtggtttccctgatcaaactagaggttaccaaggaaccatgcatagcact**

**Fortunella_japonica_1** **ttcattttctgtggtttccctgatcaaactagaggttaccaaggaaccatgcatagcact**

**Fortunella_japonica_2** **ttcattttctgtggtttccctgatcaaactagaggttaccaaggaaccatgcatagcact**

**Fortunella_japonica_3** **ttcattttctgtggtttccctgatcaaactagaggttaccaaggaaccatgcatagcact**

**Fortunella_japonica_4** **ttcattttctgtggtttccctgatcaaactagaggttaccaaggaaccatgcatagcact**

**Fortunella_japonica_5** **ttcattttctgtggtttccctgatcaaactagaggttaccaaggaaccatgcatagcact**

**Fortunella_japonica_6** **ttcattttctgtggtttccctgatcaaactagaggttaccaaggaaccatgcatagcact**

**Fortunella_japonica_7** **ttcattttctgtggtttccctgatcaaactagaggttaccaaggaaccatgcatagcact**

**Fortunella_japonica_8** **ttcattttctgtggtttccctgatcaaactagaggttaccaaggaaccatgcatagcact**

**Fortunella_japonica_9** **ttcattttctgtggtttccctgatcaaactagaggttaccaaggaaccatgcatagcact**

**Fortunella_japonica_10** **ttcattttctgtggtttccctgatcaaactagaggttaccaaggaaccatgcatagcact**

**Fortunella_japonica_11** **ttcattttctgtggtttccctgatcaaactagaggttaccaaggaaccatgcatagcact**

**Fortunella_japonica_12** **ttcattttctgtggtttccctgatcaaactagaggttaccaaggaaccatgcatagcact**

**Fortunella_margarita_1** **ttcattttctgtggtttccctgatcaaactagaggttaccaaggaaccatgcatagcact**

**Fortunella_margarita_2** **ttcattttctgtggtttccctgatcaaactagaggttaccaaggaaccatgcatagcact**

**Fortunella_venosa_1** **ttcattttctgtggtttccctgatcaaactagaggttaccaaggaaccatgcatagcact**

**Fortunella_venosa_2** **ttcattttctgtggtttccctgatcaaactagaggttaccaaggaaccatgcatagcact**

**Fortunella_venosa_3** **ttcattttctgtggtttccctgatcaaactagaggttaccaaggaaccatgcatagcact**

1030	1040	1050	1060	1070	1080

....|....|....|....|....|....|....|....|....|....|....|....|

**Atalantia_kwangtungens** **gaatagggagccgccgaatacaccagctacgcctaacatgtggaatgggtgcataaggat**

**Citrus_reticulata_1** **gaatagggagccgccgaatacaccagctacgcctaacatgtggaatgggtgcataaggat**

**Citrus_reticulata_2** **gaatagggagccgccgaatacaccagctacgcctaacatgtggaatgggtgcataaggat**

**Citrus_trifoliata_3** **gaatagggagccgccgaatacaccagctacgcctaacatgtggaatgggtgcataaggat**

**Citrus_aurantiifolia** **gaatagggagccgccgaatacaccagctacgcctaacatgtggaatgggtgcataaggat**

**Citrus_aurantium** **gaatagggagccgccgaatacaccagctacgcctaacatgtggaatgggtgcataaggat**

**Citrus_cavaleriei** **gaatagggagccgccgaatacaccagctacgcctaacatgtggaatgggtgcataaggat**

**Citrus_hongheensis** **gaatagggagccgccgaatacaccagctacgcctaacatgtggaatgggtgcataaggat**

**Citrus_limon** **gaatagggagccgccgaatacaccagctacgcctaacatgtggaatgggtgcataaggat**

**Citrus_maxima** **gaatagggagccgccgaatacaccagctacgcctaacatgtggaatgggtgcataaggat**

**Citrus_medica** **gaatagggagccgccgaatacaccagctacgcctaacatgtggaatgggtgcataaggat**

**Citrus_polytrifolia** **gaatagggagccgccgaatacaccagctacgcctaacatgtggaatgggtgcataaggat**

**Citrus_reticulata** **gaatagggagccgccgaatacaccagctacgcctaacatgtggaatgggtgcataaggat**

**Citrus_sinensis** **gaatagggagccgccgaatacaccagctacgcctaacatgtggaatgggtgcataaggat**

**Fortunella_bawangica_1** **gaatagggagccgccgaatacaccagctacgcctaacatgtggaatgggtgcataaggat**

**Fortunella_bawangica_2** **gaatagggagccgccgaatacaccagctacgcctaacatgtggaatgggtgcataaggat**

**Fortunella_hindsii_1** **gaatagggagccgccgaatacaccagctacgcctaacatgtggaatgggtgcataaggat**

**Fortunella_hindsii_2** **gaatagggagccgccgaatacaccagctacgcctaacatgtggaatgggtgcataaggat**

**Fortunella_hindsii_3** **gaatagggagccgccgaatacaccagctacgcctaacatgtggaatgggtgcataaggat**

**Fortunella_japonica_1** **gaatagggagccgccgaatacaccagctacgcctaacatgtggaatgggtgcataaggat**

**Fortunella_japonica_2** **gaatagggagccgccgaatacaccagctacgcctaacatgtggaatgggtgcataaggat**

**Fortunella_japonica_3** **gaatagggagccgccgaatacaccagctacgcctaacatgtggaatgggtgcataaggat**

**Fortunella_japonica_4** **gaatagggagccgccgaatacaccagctacgcctaacatgtggaatgggtgcataaggat**

**Fortunella_japonica_5** **gaatagggagccgccgaatacaccagctacgcctaacatgtggaatgggtgcataaggat**

**Fortunella_japonica_6** **gaatagggagccgccgaatacaccagctacgcctaacatgtggaatgggtgcataaggat**

**Fortunella_japonica_7** **gaatagggagccgccgaatacaccagctacgcctaacatgtggaatgggtgcataaggat**

**Fortunella_japonica_8** **gaatagggagccgccgaatacaccagctacgcctaacatgtggaatgggtgcataaggat**

**Fortunella_japonica_9** **gaatagggagccgccgaatacaccagctacgcctaacatgtggaatgggtgcataaggat**

**Fortunella_japonica_10** **gaatagggagccgccgaatacaccagctacgcctaacatgtggaatgggtgcataaggat**

**Fortunella_japonica_11** **gaatagggagccgccgaatacaccagctacgcctaacatgtggaatgggtgcataaggat**

**Fortunella_japonica_12** **gaatagggagccgccgaatacaccagctacgcctaacatgtggaatgggtgcataaggat**

**Fortunella_margarita_1** **gaatagggagccgccgaatacaccagctacgcctaacatgtggaatgggtgcataaggat**

**Fortunella_margarita_2** **gaatagggagccgccgaatacaccagctacgcctaacatgtggaatgggtgcataaggat**

**Fortunella_venosa_1** **gaatagggagccgccgaatacaccagctacgcctaacatgtggaatgggtgcataaggat**

**Fortunella_venosa_2** **gaatagggagccgccgaatacaccagctacgcctaacatgtggaatgggtgcataaggat**

**Fortunella_venosa_3** **gaatagggagccgccgaatacaccagctacgcctaacatgtggaatgggtgcataaggat**

1090	1100	1110	1120	1130	1140

....|....|....|....|....|....|....|....|....|....|....|....|

**Atalantia_kwangtungens** **gttgtgctcagcctgaaatacaatcatgaagttgaaagtaccagagattcctagaggcat**

**Citrus_reticulata_1** **gttgtgctcagcctggaatacaatcatgaagttgaaagtaccagagattcctagaggcat**

**Citrus_reticulata_2** **gttgtgctcagcctggaatacaatcatgaagttgaaagtaccagagattcctagaggcat**

**Citrus_trifoliata_3** **attgtgctcagcctggaatacaatcatgaagttgaaagtaccagagattcctagaggcat**

**Citrus_aurantiifolia** **gttgtgctcagcctggaatacaatcatgaagttgaaagtaccagatattcctagaggcat**

**Citrus_aurantium** **gttgtgctcagcctggaatacaatcatgaaattgaaagtaccagagattcctagaggcat**

**Citrus_cavaleriei** **gttgtgctcagcctggaatacaatcatgaagttgaaagtaccagagattcctagaggcat**

**Citrus_hongheensis** **gttgtgctcagcctggaatacaatcatgaaattgaaagtaccagagattcctagaggcat**

**Citrus_limon** **gttgtgctcagcctggaatacaatcatgaaattgaaagtaccagagattcctagaggcat**

**Citrus_maxima** **gttgtgctcagcctggaatacaatcatgaaattgaaagtaccagagattcctagaggcat**

**Citrus_medica** **gttgtgctcagcctggaatacaatcatgaagttgaaagtaccagagattcctagaggcat**

**Citrus_polytrifolia** **attgtgctcagcctggaatacaatcatgaagttgaaagtaccagagattcctagaggcat**

**Citrus_reticulata** **gttgtgctcagcctggaatacaatcatgaagttgaaagtaccagagattcctagaggcat**

**Citrus_sinensis** **gttgtgctcagcctggaatacaatcatgaaattgaaagtaccagagattcctagaggcat**

**Fortunella_bawangica_1** **gttgtgctcagcctggaatacaatcatgaagttgaaagtaccagagattcctagaggcat**

**Fortunella_bawangica_2** **gttgtgctcagcctggaatacaatcatgaagttgaaagtaccagagattcctagaggcat**

**Fortunella_hindsii_1** **gttgtgctcagcctggaatacaatcatgaagttgaaagtaccagagattcctagaggcat**

**Fortunella_hindsii_2** **gttgtgctcagcctggaatacaatcatgaagttgaaagtaccagagattcctagaggcat**

**Fortunella_hindsii_3** **gttgtgctcagcctggaatacaatcatgaagttgaaagtaccagagattcctagaggcat**

**Fortunella_japonica_1** **gttgtgctcagcctggaatacaatcatgaagttgaaagtaccagagattcctagaggcat**

**Fortunella_japonica_2** **gttgtgctcagcctggaatacaatcatgaagttgaaagtaccagagattcctagaggcat**

**Fortunella_japonica_3** **gttgtgctcagcctggaatacaatcatgaagttgaaagtaccagagattcctagaggcat**

**Fortunella_japonica_4** **gttgtgctcagcctggaatacaatcatgaagttgaaagtaccagagattcctagaggcat**

**Fortunella_japonica_5** **gttgtgctcagcctggaatacaatcatgaagttgaaagtaccagagattcctagaggcat**

**Fortunella_japonica_6** **gttgtgctcagcctggaatacaatcatgaagttgaaagtaccagagattcctagaggcat**

**Fortunella_japonica_7** **gttgtgctcagcctggaatacaatcatgaagttgaaagtaccagagattcctagaggcat**

**Fortunella_japonica_8** **gttgtgctcagcctggaatacaatcatgaagttgaaagtaccagagattcctagaggcat**

**Fortunella_japonica_9** **gttgtgctcagcctggaatacaatcatgaagttgaaagtaccagagattcctagaggcat**

**Fortunella_japonica_10** **gttgtgctcagcctggaatacaatcatgaagttgaaagtaccagagattcctagaggcat**

**Fortunella_japonica_11** **gttgtgctcagcctggaatacaatcatgaagttgaaagtaccagagattcctagaggcat**

**Fortunella_japonica_12** **gttgtgctcagcctggaatacaatcatgaagttgaaagtaccagagattcctagaggcat**

**Fortunella_margarita_1** **gttgtgctcagcctggaatacaatcatgaagttgaaagtaccagagattcctagaggcat**

**Fortunella_margarita_2** **gttgtgctcagcctggaatacaatcatgaagttgaaagtaccagagattcctagaggcat**

**Fortunella_venosa_1** **gttgtgctcagcctggaatacaatcatgaagttgaaagtaccagagattcctagaggcat**

**Fortunella_venosa_2** **gttgtgctcagcctggaatacaatcatgaagttgaaagtaccagagattcctagaggcat**

**Fortunella_venosa_3** **gttgtgctcagcctggaatacaatcatgaagttgaaagtaccagagattcctagaggcat**

1150	1160	1170	1180	1190	1200

....|....|....|....|....|....|....|....|....|....|....|....|

**Atalantia_kwangtungens** **accatcagaaaaacttccttgaccgattgggtagatcaagaaaacagcagccgctgctgc**

**Citrus_reticulata_1** **accatcagaaaaacttccttgaccgattgggtagatcaagaaaacagcagtcgctgctgc**

**Citrus_reticulata_2** **accatcagaaaaacttccttgaccgattgggtagatcaagaaaacagcagtcgctgctgc**

**Citrus_trifoliata_3** **accatcagaaaaacttccttgaccgattgggtagatcaagaaaacagcagtcgctgctgc**

**Citrus_aurantiifolia** **accatcagaaaaacttccttgaccgattgggtagatcaagaaaacagcagtcgctgctgc**

**Citrus_aurantium** **accatcagaaaaacttccttgaccgattgggtagatcaagaaaacagcagtcgctgctgc**

**Citrus_cavaleriei** **accatcagaaaaacttccttgaccgattgggtagatcaagaaaacagcagtcgctgctgc**

**Citrus_hongheensis** **accatcagaaaaacttccttgaccgattgggtagatcaagaaaacagcagtcgctgctgc**

**Citrus_limon** **accatcagaaaaacttccttgaccgattgggtagatcaagaaaacagcagtcgctgctgc**

**Citrus_maxima** **accatcagaaaaacttccttgaccgattgggtagatcaagaaaacagcagtcgctgctgc**

**Citrus_medica** **accatcagaaaaacttccttgaccgattgggtagatcaagaaaacagcagtcgctgctgc**

**Citrus_polytrifolia** **accatcagaaaaacttccttgaccgattgggtagatcaagaaaacagcagtcgctgctgc**

**Citrus_reticulata** **accatcagaaaaacttccttgaccgattgggtagatcaagaaaacagcagtcgctgctgc**

**Citrus_sinensis** **accatcagaaaaacttccttgaccgattgggtagatcaagaaaacagcagtcgctgctgc**

**Fortunella_bawangica_1** **accatcagaaaaacttccttgaccgattgggtagatcaagaaaacagcagtcgctgctgc**

**Fortunella_bawangica_2** **accatcagaaaaacttccttgaccgattgggtagatcaagaaaacagcagtcgctgctgc**

**Fortunella_hindsii_1** **accatcagaaaaacttccttgaccgattgggtagatcaagaaaacagcagtcgctgctgc**

**Fortunella_hindsii_2** **accatcagaaaaacttccttgaccgattgggtagatcaagaaaacagcagtcgctgctgc**

**Fortunella_hindsii_3** **accatcagaaaaacttccttgaccgattgggtagatcaagaaaacagcagtcgctgctgc**

**Fortunella_japonica_1** **accatcagaaaaacttccttgaccgattgggtagatcaagaaaacagcagtcgctgctgc**

**Fortunella_japonica_2** **accatcagaaaaacttccttgaccgattgggtagatcaagaaaacagcagtcgctgctgc**

**Fortunella_japonica_3** **accatcagaaaaacttccttgaccgattgggtagatcaagaaaacagcagtcgctgctgc**

**Fortunella_japonica_4** **accatcagaaaaacttccttgaccgattgggtagatcaagaaaacagcagtcgctgctgc**

**Fortunella_japonica_5** **accatcagaaaaacttccttgaccgattgggtagatcaagaaaacagcagtcgctgctgc**

**Fortunella_japonica_6** **accatcagaaaaacttccttgaccgattgggtagatcaagaaaacagcagtcgctgctgc**

**Fortunella_japonica_7** **accatcagaaaaacttccttgaccgattgggtagatcaagaaaacagcagtcgctgctgc**

**Fortunella_japonica_8** **accatcagaaaaacttccttgaccgattgggtagatcaagaaaacagcagtcgctgctgc**

**Fortunella_japonica_9** **accatcagaaaaacttccttgaccgattgggtagatcaagaaaacagcagtcgctgctgc**

**Fortunella_japonica_10** **accatcagaaaaacttccttgaccgattgggtagatcaagaaaacagcagtcgctgctgc**

**Fortunella_japonica_11** **accatcagaaaaacttccttgaccgattgggtagatcaagaaaacagcagtcgctgctgc**

**Fortunella_japonica_12** **accatcagaaaaacttccttgaccgattgggtagatcaagaaaacagcagtcgctgctgc**

**Fortunella_margarita_1** **accatcagaaaaacttccttgaccgattgggtagatcaagaaaacagcagtcgctgctgc**

**Fortunella_margarita_2** **accatcagaaaaacttccttgaccgattgggtagatcaagaaaacagcagtcgctgctgc**

**Fortunella_venosa_1** **accatcagaaaaacttccttgaccgattgggtagatcaagaaaacagcagtcgctgctgc**

**Fortunella_venosa_2** **accatcagaaaaacttccttgaccgattgggtagatcaagaaaacagcagtcgctgctgc**

**Fortunella_venosa_3** **accatcagaaaaacttccttgaccgattgggtagatcaagaaaacagcagtcgctgctgc**

1210	1220	1230	1240	1250	1260

....|....|....|....|....|....|....|....|....|....|....|....|

**Atalantia_kwangtungens** **aacaggagctgaatatgcaacagcaatccaaggacgcatacccagacggaaactaagttc**

**Citrus_reticulata_1** **aacaggagctgaatatgcaacagcaatccaaggacgcatacccagacggaaactaagttc**

**Citrus_reticulata_2** **aacaggagctgaatatgcaacagcaatccaaggacgcatacccagacggaaactaagttc**

**Citrus_trifoliata_3** **aacaggagctgaatatgcaacagcaatccaaggacgcatacccagacggaaactaagttc**

**Citrus_aurantiifolia** **aacaggagctgaatatgcaacagcaatccaaggacgcatacccagacggaaactaagttc**

**Citrus_aurantium** **aacaggagctgaatatgcaacagcaatccaaggacgcatacccagacggaaactaagttc**

**Citrus_cavaleriei** **aacaggagctgaatatgcaacagcaatccaaggacgcatacccagacggaaactaagttc**

**Citrus_hongheensis** **aacaggagctgaatatgcaacagcaatccaaggacgcatacccagacggaaactaagttc**

**Citrus_limon** **aacaggagctgaatatgcaacagcaatccaaggacgcatacccagacggaaactaagttc**

**Citrus_maxima** **aacaggagctgaatatgcaacagcaatccaaggacgcatacccagacggaaactaagttc**

**Citrus_medica** **aacaggagctgaatatgcaacagcaatccaaggacgcatacccagacggaaactaagttc**

**Citrus_polytrifolia** **aacaggagctgaatatgcaacagcaatccaaggacgcatacccagacggaaactaagttc**

**Citrus_reticulata** **aacaggagctgaatatgcaacagcaatccaaggacgcatacccagacggaaactaagttc**

**Citrus_sinensis** **aacaggagctgaatatgcaacagcaatccaaggacgcatacccagacggaaactaagttc**

**Fortunella_bawangica_1** **aacaggagctgaatatgcaacagcaatccaaggacgcatacccagacggaaactaagttc**

**Fortunella_bawangica_2** **aacaggagctgaatatgcaacagcaatccaaggacgcatacccagacggaaactaagttc**

**Fortunella_hindsii_1** **aacaggagctgaatatgcaacagcaatccaaggacgcatacccagacggaaactaagttc**

**Fortunella_hindsii_2** **aacaggagctgaatatgcaacagcaatccaaggacgcatacccagacggaaactaagttc**

**Fortunella_hindsii_3** **aacaggagctgaatatgcaacagcaatccaaggacgcatacccagacggaaactaagttc**

**Fortunella_japonica_1** **aacaggagctgaatatgcaacagcaatccaaggacgcatacccagacggaaactaagttc**

**Fortunella_japonica_2** **aacaggagctgaatatgcaacagcaatccaaggacgcatacccagacggaaactaagttc**

**Fortunella_japonica_3** **aacaggagctgaatatgcaacagcaatccaaggacgcatacccagacggaaactaagttc**

**Fortunella_japonica_4** **aacaggagctgaatatgcaacagcaatccaaggacgcatacccagacggaaactaagttc**

**Fortunella_japonica_5** **aacaggagctgaatatgcaacagcaatccaaggacgcatacccagacggaaactaagttc**

**Fortunella_japonica_6** **aacaggagctgaatatgcaacagcaatccaaggacgcatacccagacggaaactaagttc**

**Fortunella_japonica_7** **aacaggagctgaatatgcaacagcaatccaaggacgcatacccagacggaaactaagttc**

**Fortunella_japonica_8** **aacaggagctgaatatgcaacagcaatccaaggacgcatacccagacggaaactaagttc**

**Fortunella_japonica_9** **aacaggagctgaatatgcaacagcaatccaaggacgcatacccagacggaaactaagttc**

**Fortunella_japonica_10** **aacaggagctgaatatgcaacagcaatccaaggacgcatacccagacggaaactaagttc**

**Fortunella_japonica_11** **aacaggagctgaatatgcaacagcaatccaaggacgcatacccagacggaaactaagttc**

**Fortunella_japonica_12** **aacaggagctgaatatgcaacagcaatccaaggacgcatacccagacggaaactaagttc**

**Fortunella_margarita_1** **aacaggagctgaatatgcaacagcaatccaaggacgcatacccagacggaaactaagttc**

**Fortunella_margarita_2** **aacaggagctgaatatgcaacagcaatccaaggacgcatacccagacggaaactaagttc**

**Fortunella_venosa_1** **aacaggagctgaatatgcaacagcaatccaaggacgcatacccagacggaaactaagttc**

**Fortunella_venosa_2** **aacaggagctgaatatgcaacagcaatccaaggacgcatacccagacggaaactaagttc**

**Fortunella_venosa_3** **aacaggagctgaatatgcaacagcaatccaaggacgcatacccagacggaaactaagttc**

1270	1280	1290	1300	1310	1320

....|....|....|....|....|....|....|....|....|....|....|....|

**Atalantia_kwangtungens** **ccactcacgacccatgtaacaagctacaccaagtaagaagtgtagaacaattagctcata**

**Citrus_reticulata_1** **ccactcacgacccatgtaacaagctacaccaagtaagaagtgtagaacaattagctcata**

**Citrus_reticulata_2** **ccactcacgacccatgtaacaagctacaccaagtaagaagtgtagaacaattagctcata**

**Citrus_trifoliata_3** **ccactcacgacccatgtaacaagctacaccaagtaagaagtgtagaacaattagctcata**

**Citrus_aurantiifolia** **ccactcacgacccatgtaacaagctacaccaagtaagaagtgtagaacaattagctcata**

**Citrus_aurantium** **ccactcacgacccatgtaacaagctacaccaagtaagaagtgtagaacaattagctcata**

**Citrus_cavaleriei** **ccactcacgacccatgtaacaagctacaccaagtaagaagtgtagaacaattagctcata**

**Citrus_hongheensis** **ccactcacgacccatgtaacaagctacaccaagtaagaagtgtagaacaattagctcata**

**Citrus_limon** **ccactcacgacccatgtaacaagctacaccaagtaagaagtgtagaacaattagctcata**

**Citrus_maxima** **ccactcacgacccatgtaacaagctacaccaagtaagaagtgtagaacaattagctcata**

**Citrus_medica** **ccactcacgacccatgtaacaagctacaccaagtaagaagtgtagaacaattagctcata**

**Citrus_polytrifolia** **ccactcacgacccatgtaacaagctacaccaagtaagaagtgtagaacaattagctcata**

**Citrus_reticulata** **ccactcacgacccatgtaacaagctacaccaagtaagaagtgtagaacaattagctcata**

**Citrus_sinensis** **ccactcacgacccatgtaacaagctacaccaagtaagaagtgtagaacaattagctcata**

**Fortunella_bawangica_1** **ccactcacgacccatgtaacaagctacaccaagtaagaagtgtagaacaattagctcata**

**Fortunella_bawangica_2** **ccactcacgacccatgtaacaagctacaccaagtaagaagtgtagaacaattagctcata**

**Fortunella_hindsii_1** **ccactcacgacccatgtaacaagctacaccaagtaagaagtgtagaacaattagctcata**

**Fortunella_hindsii_2** **ccactcacgacccatgtaacaagctacaccaagtaagaagtgtagaacaattagctcata**

**Fortunella_hindsii_3** **ccactcacgacccatgtaacaagctacaccaagtaagaagtgtagaacaattagctcata**

**Fortunella_japonica_1** **ccactcacgacccatgtaacaagctacaccaagtaagaagtgtagaacaattagctcata**

**Fortunella_japonica_2** **ccactcacgacccatgtaacaagctacaccaagtaagaagtgtagaacaattagctcata**

**Fortunella_japonica_3** **ccactcacgacccatgtaacaagctacaccaagtaagaagtgtagaacaattagctcata**

**Fortunella_japonica_4** **ccactcacgacccatgtaacaagctacaccaagtaagaagtgtagaacaattagctcata**

**Fortunella_japonica_5** **ccactcacgacccatgtaacaagctacaccaagtaagaagtgtagaacaattagctcata**

**Fortunella_japonica_6** **ccactcacgacccatgtaacaagctacaccaagtaagaagtgtagaacaattagctcata**

**Fortunella_japonica_7** **ccactcacgacccatgtaacaagctacaccaagtaagaagtgtagaacaattagctcata**

**Fortunella_japonica_8** **ccactcacgacccatgtaacaagctacaccaagtaagaagtgtagaacaattagctcata**

**Fortunella_japonica_9** **ccactcacgacccatgtaacaagctacaccaagtaagaagtgtagaacaattagctcata**

**Fortunella_japonica_10** **ccactcacgacccatgtaacaagctacaccaagtaagaagtgtagaacaattagctcata**

**Fortunella_japonica_11** **ccactcacgacccatgtaacaagctacaccaagtaagaagtgtagaacaattagctcata**

**Fortunella_japonica_12** **ccactcacgacccatgtaacaagctacaccaagtaagaagtgtagaacaattagctcata**

**Fortunella_margarita_1** **ccactcacgacccatgtaacaagctacaccaagtaagaagtgtagaacaattagctcata**

**Fortunella_margarita_2** **ccactcacgacccatgtaacaagctacaccaagtaagaagtgtagaacaattagctcata**

**Fortunella_venosa_1** **ccactcacgacccatgtaacaagctacaccaagtaagaagtgtagaacaattagctcata**

**Fortunella_venosa_2** **ccactcacgacccatgtaacaagctacaccaagtaagaagtgtagaacaattagctcata**

**Fortunella_venosa_3** **ccactcacgacccatgtaacaagctacaccaagtaagaagtgtagaacaattagctcata**

1330	1340	1350	1360	1370	1380

....|....|....|....|....|....|....|....|....|....|....|....|

**Atalantia_kwangtungens** **aggaccgccattgtataaccattcatcaacggatgccgcttcccatatcgggtaaaaatg**

**Citrus_reticulata_1** **aggaccgccattgtataaccattcatcaacggatgccgcttcccatatcgggtaaaaatg**

**Citrus_reticulata_2** **aggaccgccattgtataaccattcatcaacggatgccgcttcccatatcgggtaaaaatg**

**Citrus_trifoliata_3** **aggaccgccattgtataaccattcatcaacggatgccgcttcccatatcgggtaaaaatg**

**Citrus_aurantiifolia** **aggaccgccattgtataaccattcatcaacggatgccgcttcccatatcgggtaaaaatg**

**Citrus_aurantium** **aggaccgccattgtataaccattcatcaacggatgccgcttcccatatcgggtaaaaatg**

**Citrus_cavaleriei** **aggaccgccattgtataaccattcatcaacggatgccgcttcccatatcgggtaaaaatg**

**Citrus_hongheensis** **aggaccgccattgtataaccattcatcaacggatgccgcttcccatatcgggtaaaaatg**

**Citrus_limon** **aggaccgccattgtataaccattcatcaacggatgccgcttcccatatcgggtaaaaatg**

**Citrus_maxima** **aggaccgccattgtataaccattcatcaacggatgccgcttcccatatcgggtaaaaatg**

**Citrus_medica** **aggaccgccattgtataaccattcatcaacggatgccgcttcccatatcgggtaaaaatg**

**Citrus_polytrifolia** **aggaccgccattgtataaccattcatcaacggatgccgcttcccatatcgggtaaaaatg**

**Citrus_reticulata** **aggaccgccattgtataaccattcatcaacggatgccgcttcccatatcgggtaaaaatg**

**Citrus_sinensis** **aggaccgccattgtataaccattcatcaacggatgccgcttcccatatcgggtaaaaatg**

**Fortunella_bawangica_1** **aggaccgccattgtataaccattcatcaacggatgccgcttcccatatcgggtaaaaatg**

**Fortunella_bawangica_2** **aggaccgccattgtataaccattcatcaacggatgccgcttcccatatcgggtaaaaatg**

**Fortunella_hindsii_1** **aggaccgccattgtataaccattcatcaacggatgccgcttcccatatcgggtaaaaatg**

**Fortunella_hindsii_2** **aggaccgccattgtataaccattcatcaacggatgccgcttcccatatcgggtaaaaatg**

**Fortunella_hindsii_3** **aggaccgccattgtataaccattcatcaacggatgccgcttcccatatcgggtaaaaatg**

**Fortunella_japonica_1** **aggaccgccattgtataaccattcatcaacggatgccgcttcccatatcgggtaaaaatg**

**Fortunella_japonica_2** **aggaccgccattgtataaccattcatcaacggatgccgcttcccatatcgggtaaaaatg**

**Fortunella_japonica_3** **aggaccgccattgtataaccattcatcaacggatgccgcttcccatatcgggtaaaaatg**

**Fortunella_japonica_4** **aggaccgccattgtataaccattcatcaacggatgccgcttcccatatcgggtaaaaatg**

**Fortunella_japonica_5** **aggaccgccattgtataaccattcatcaacggatgccgcttcccatatcgggtaaaaatg**

**Fortunella_japonica_6** **aggaccgccattgtataaccattcatcaacggatgccgcttcccatatcgggtaaaaatg**

**Fortunella_japonica_7** **aggaccgccattgtataaccattcatcaacggatgccgcttcccatatcgggtaaaaatg**

**Fortunella_japonica_8** **aggaccgccattgtataaccattcatcaacggatgccgcttcccatatcgggtaaaaatg**

**Fortunella_japonica_9** **aggaccgccattgtataaccattcatcaacggatgccgcttcccatatcgggtaaaaatg**

**Fortunella_japonica_10** **aggaccgccattgtataaccattcatcaacggatgccgcttcccatatcgggtaaaaatg**

**Fortunella_japonica_11** **aggaccgccattgtataaccattcatcaacggatgccgcttcccatatcgggtaaaaatg**

**Fortunella_japonica_12** **aggaccgccattgtataaccattcatcaacggatgccgcttcccatatcgggtaaaaatg**

**Fortunella_margarita_1** **aggaccgccattgtataaccattcatcaacggatgccgcttcccatatcgggtaaaaatg**

**Fortunella_margarita_2** **aggaccgccattgtataaccattcatcaacggatgccgcttcccatatcgggtaaaaatg**

**Fortunella_venosa_1** **aggaccgccattgtataaccattcatcaacggatgccgcttcccatatcgggtaaaaatg**

**Fortunella_venosa_2** **aggaccgccattgtataaccattcatcaacggatgccgcttcccatatcgggtaaaaatg**

**Fortunella_venosa_3** **aggaccgccattgtataaccattcatcaacggatgccgcttcccatatcgggtaaaaatg**

1390	1400	1410	1420	1430	1440

....|....|....|....|....|....|....|....|....|....|....|....|

**Atalantia_kwangtungens** **caaaccgatagctgcagaagtaggaataatcgcaccagaaataatattgtttccataaag**

**Citrus_reticulata_1** **caaacctatagctgcagaagtaggaataatcgcaccagaaataatattgtttccataaag**

**Citrus_reticulata_2** **caaacctatagctgcagaagtaggaataatcgcaccagaaataatattgtttccataaag**

**Citrus_trifoliata_3** **caaacctatagctgcagaagtaggaataatcgcaccagaaataatattgtttccataaag**

**Citrus_aurantiifolia** **caaacctatagctgcagaagtaggaataatcgcaccagaaataatattgtttccataaag**

**Citrus_aurantium** **caaacctatagctgcagaagtaggaataatcgcaccagaaataatattgtttccataaag**

**Citrus_cavaleriei** **caaacctatagctgcagaagtaggaataatcgcaccagaaataatattgtttccataaag**

**Citrus_hongheensis** **caaacctatagctgcagaagtaggaataatcgcaccagaaataatattgtttccataaag**

**Citrus_limon** **caaacctatagctgcagaagtaggaataatcgcaccagaaataatattgtttccataaag**

**Citrus_maxima** **caaacctatagctgcagaagtaggaataatcgcaccagaaataatattgtttccataaag**

**Citrus_medica** **caaacctatagctgcagaagtaggaataatcgcaccagaaataatattgtttccataaag**

**Citrus_polytrifolia** **caaacctatagctgcagaagtaggaataatcgcaccagaaataatattgtttccataaag**

**Citrus_reticulata** **caaacctatagctgcagaagtaggaataatcgcaccagaaataatattgtttccataaag**

**Citrus_sinensis** **caaacctatagctgcagaagtaggaataatcgcaccagaaataatattgtttccataaag**

**Fortunella_bawangica_1** **caaacctatagctgcagaagtaggaataatcgcaccagaaataatattgtttccataaag**

**Fortunella_bawangica_2** **caaacctatagctgcagaagtaggaataatcgcaccagaaataatattgtttccataaag**

**Fortunella_hindsii_1** **caaacctatagctgcagaagtaggaataatcgcaccagaaataatattgtttccataaag**

**Fortunella_hindsii_2** **caaacctatagctgcagaagtaggaataatcgcaccagaaataatattgtttccataaag**

**Fortunella_hindsii_3** **caaacctatagctgcagaagtaggaataatcgcaccagaaataatattgtttccataaag**

**Fortunella_japonica_1** **caaacctatagctgcagaagtaggaataatcgcaccagaaataatattgtttccataaag**

**Fortunella_japonica_2** **caaacctatagctgcagaagtaggaataatcgcaccagaaataatattgtttccataaag**

**Fortunella_japonica_3** **caaacctatagctgcagaagtaggaataatcgcaccagaaataatattgtttccataaag**

**Fortunella_japonica_4** **caaacctatagctgcagaagtaggaataatcgcaccagaaataatattgtttccataaag**

**Fortunella_japonica_5** **caaacctatagctgcagaagtaggaataatcgcaccagaaataatattgtttccataaag**

**Fortunella_japonica_6** **caaacctatagctgcagaagtaggaataatcgcaccagaaataatattgtttccataaag**

**Fortunella_japonica_7** **caaacctatagctgcagaagtaggaataatcgcaccagaaataatattgtttccataaag**

**Fortunella_japonica_8** **caaacctatagctgcagaagtaggaataatcgcaccagaaataatattgtttccataaag**

**Fortunella_japonica_9** **caaacctatagctgcagaagtaggaataatcgcaccagaaataatattgtttccataaag**

**Fortunella_japonica_10** **caaacctatagctgcagaagtaggaataatcgcaccagaaataatattgtttccataaag**

**Fortunella_japonica_11** **caaacctatagctgcagaagtaggaataatcgcaccagaaataatattgtttccataaag**

**Fortunella_japonica_12** **caaacctatagctgcagaagtaggaataatcgcaccagaaataatattgtttccataaag**

**Fortunella_margarita_1** **caaacctatagctgcagaagtaggaataatcgcaccagaaataatattgtttccataaag**

**Fortunella_margarita_2** **caaacctatagctgcagaagtaggaataatcgcaccagaaataatattgtttccataaag**

**Fortunella_venosa_1** **caaacctatagctgcagaagtaggaataatcgcaccagaaataatattgtttccataaag**

**Fortunella_venosa_2** **caaacctatagctgcagaagtaggaataatcgcaccagaaataatattgtttccataaag**

**Fortunella_venosa_3** **caaacctatagctgcagaagtaggaataatcgcaccagaaataatattgtttccataaag**

1450	1460	1470	1480	1490	1500

....|....|....|....|....|....|....|....|....|....|....|....|

**Atalantia_kwangtungens** **tagagatccagaaacaggttcacgaataccgtcaatatctactggaggagcagcaatgaa**

**Citrus_reticulata_1** **tagagatccagaaacaggttcacgaataccgtcaatatctactggaggagcagcaatgaa**

**Citrus_reticulata_2** **tagagatccagaaacaggttcacgaataccgtcaatatctactggaggagcagcaatgaa**

**Citrus_trifoliata_3** **tagagatccagaaacaggttcacgaataccgtcaatatctactggaggagcagcaatgaa**

**Citrus_aurantiifolia** **tagagatccagaaacaggttcacgaataccgtcaatatctactggaggagcagcaatgaa**

**Citrus_aurantium** **tagagatccagaaacaggttcacgaataccgtcaatatctactggaggagcagcaatgaa**

**Citrus_cavaleriei** **tagagatccagaaacaggttcacgaataccgtcaatatctactggaggagcagcaatgaa**

**Citrus_hongheensis** **tagagatccagaaacaggttcacgaataccgtcaatatctactggaggagcagcaatgaa**

**Citrus_limon** **tagagatccagaaacaggttcacgaataccgtcaatatctactggaggagcagcaatgaa**

**Citrus_maxima** **tagagatccagaaacaggttcacgaataccgtcaatatctactggaggagcagcaatgaa**

**Citrus_medica** **cagagatccagaaacaggttcacgaataccgtcaatatctactggaggagcagcaatgaa**

**Citrus_polytrifolia** **tagagatccagaaacaggttcacgaataccgtcaatatctactggaggagcagcaatgaa**

**Citrus_reticulata** **tagagatccagaaacaggttcacgaataccgtcaatatctactggaggagcagcaatgaa**

**Citrus_sinensis** **tagagatccagaaacaggttcacgaataccgtcaatatctactggaggagcagcaatgaa**

**Fortunella_bawangica_1** **tagagatccagaaacaggttcacgaataccgtcaatatctactggaggagcagcaatgaa**

**Fortunella_bawangica_2** **tagagatccagaaacaggttcacgaataccgtcaatatctactggaggagcagcaatgaa**

**Fortunella_hindsii_1** **tagagatccagaaacaggttcacgaataccgtcaatatctactggaggagcagcaatgaa**

**Fortunella_hindsii_2** **tagagatccagaaacaggttcacgaataccgtcaatatctactggaggagcagcaatgaa**

**Fortunella_hindsii_3** **tagagatccagaaacaggttcacgaataccgtcaatatctactggaggagcagcaatgaa**

**Fortunella_japonica_1** **tagagatccagaaacaggttcacgaataccgtcaatatctactggaggagcagcaatgaa**

**Fortunella_japonica_2** **tagagatccagaaacaggttcacgaataccgtcaatatctactggaggagcagcaatgaa**

**Fortunella_japonica_3** **tagagatccagaaacaggttcacgaataccgtcaatatctactggaggagcagcaatgaa**

**Fortunella_japonica_4** **tagagatccagaaacaggttcacgaataccgtcaatatctactggaggagcagcaatgaa**

**Fortunella_japonica_5** **tagagatccagaaacaggttcacgaataccgtcaatatctactggaggagcagcaatgaa**

**Fortunella_japonica_6** **tagagatccagaaacaggttcacgaataccgtcaatatctactggaggagcagcaatgaa**

**Fortunella_japonica_7** **tagagatccagaaacaggttcacgaataccgtcaatatctactggaggagcagcaatgaa**

**Fortunella_japonica_8** **tagagatccagaaacaggttcacgaataccgtcaatatctactggaggagcagcaatgaa**

**Fortunella_japonica_9** **tagagatccagaaacaggttcacgaataccgtcaatatctactggaggagcagcaatgaa**

**Fortunella_japonica_10** **tagagatccagaaacaggttcacgaataccgtcaatatctactggaggagcagcaatgaa**

**Fortunella_japonica_11** **tagagatccagaaacaggttcacgaataccgtcaatatctactggaggagcagcaatgaa**

**Fortunella_japonica_12** **tagagatccagaaacaggttcacgaataccgtcaatatctactggaggagcagcaatgaa**

**Fortunella_margarita_1** **tagagatccagaaacaggttcacgaataccgtcaatatctactggaggagcagcaatgaa**

**Fortunella_margarita_2** **tagagatccagaaacaggttcacgaataccgtcaatatctactggaggagcagcaatgaa**

**Fortunella_venosa_1** **tagagatccagaaacaggttcacgaataccgtcaatatctactggaggagcagcaatgaa**

**Fortunella_venosa_2** **tagagatccagaaacaggttcacgaataccgtcaatatctactggaggagcagcaatgaa**

**Fortunella_venosa_3** **tagagatccagaaacaggttcacgaataccgtcaatatctactggaggagcagcaatgaa**

1510	1520	1530	1540	1550	1560

....|....|....|....|....|....|....|....|....|....|....|....|

**Atalantia_kwangtungens** **ggcgataataaatacagaagttgcggtcaataaagtagggatcatcaaaacaccaaacca**

**Citrus_reticulata_1** **ggcgataataaatacagaagttgcggtcaataaagtagggatcatcaaaacaccaaacca**

**Citrus_reticulata_2** **ggcgataataaatacagaagttgcggtcaataaagtagggatcatcaaaacaccaaacca**

**Citrus_trifoliata_3** **ggcgataataaatacagaagttgcggtcaataaagtagggatcatcaaaacaccaaacca**

**Citrus_aurantiifolia** **ggcgataataaatacagaagttgcggtcaataaagtagggatcatcaaaacaccaaacca**

**Citrus_aurantium** **ggcgataataaatacagaagttgcggtcaataaagtagggatcatcaaaacaccaaacca**

**Citrus_cavaleriei** **ggcgataataaatacagaagttgcggtcaataaagtagggatcatcaaaacaccaaacca**

**Citrus_hongheensis** **ggcgataataaatacagaagttgcggtcaataaagtagggatcatcaaaacaccaaacca**

**Citrus_limon** **ggcgataataaatacagaagttgcggtcaataaagtagggatcatcaaaacaccaaacca**

**Citrus_maxima** **ggcgataataaatacagaagttgcggtcaataaagtagggatcatcaaaacaccaaacca**

**Citrus_medica** **ggcgataataaatacagaagttgcggtcaataaagtagggatcatcaaaacaccaaacca**

**Citrus_polytrifolia** **ggcgataataaatacagaagttgcggtcaataaagtagggatcatcaaaacaccaaacca**

**Citrus_reticulata** **ggcgataataaatacagaagttgcggtcaataaagtagggatcatcaaaacaccaaacca**

**Citrus_sinensis** **ggcgataataaatacagaagttgcggtcaataaagtagggatcatcaaaacaccaaacca**

**Fortunella_bawangica_1** **ggcgataataaatacagaagttgcggtcaataaagtagggatcatcaaaacaccaaacca**

**Fortunella_bawangica_2** **ggcgataataaatacagaagttgcggtcaataaagtagggatcatcaaaacaccaaacca**

**Fortunella_hindsii_1** **ggcgataataaatacagaagttgcggtcaataaagtagggatcatcaaaacaccaaacca**

**Fortunella_hindsii_2** **ggcgataataaatacagaagttgcggtcaataaagtagggatcatcaaaacaccaaacca**

**Fortunella_hindsii_3** **ggcgataataaatacagaagttgcggtcaataaagtagggatcatcaaaacaccaaacca**

**Fortunella_japonica_1** **ggcgataataaatacagaagttgcggtcaataaagtagggatcatcaaaacaccaaacca**

**Fortunella_japonica_2** **ggcgataataaatacagaagttgcggtcaataaagtagggatcatcaaaacaccaaacca**

**Fortunella_japonica_3** **ggcgataataaatacagaagttgcggtcaataaagtagggatcatcaaaacaccaaacca**

**Fortunella_japonica_4** **ggcgataataaatacagaagttgcggtcaataaagtagggatcatcaaaacaccaaacca**

**Fortunella_japonica_5** **ggcgataataaatacagaagttgcggtcaataaagtagggatcatcaaaacaccaaacca**

**Fortunella_japonica_6** **ggcgataataaatacagaagttgcggtcaataaagtagggatcatcaaaacaccaaacca**

**Fortunella_japonica_7** **ggcgataataaatacagaagttgcggtcaataaagtagggatcatcaaaacaccaaacca**

**Fortunella_japonica_8** **ggcgataataaatacagaagttgcggtcaataaagtagggatcatcaaaacaccaaacca**

**Fortunella_japonica_9** **ggcgataataaatacagaagttgcggtcaataaagtagggatcatcaaaacaccaaacca**

**Fortunella_japonica_10** **ggcgataataaatacagaagttgcggtcaataaagtagggatcatcaaaacaccaaacca**

**Fortunella_japonica_11** **ggcgataataaatacagaagttgcggtcaataaagtagggatcatcaaaacaccaaacca**

**Fortunella_japonica_12** **ggcgataataaatacagaagttgcggtcaataaagtagggatcatcaaaacaccaaacca**

**Fortunella_margarita_1** **ggcgataataaatacagaagttgcggtcaataaagtagggatcatcaaaacaccaaacca**

**Fortunella_margarita_2** **ggcgataataaatacagaagttgcggtcaataaagtagggatcatcaaaacaccaaacca**

**Fortunella_venosa_1** **ggcgataataaatacagaagttgcggtcaataaagtagggatcatcaaaacaccaaacca**

**Fortunella_venosa_2** **ggcgataataaatacagaagttgcggtcaataaagtagggatcatcaaaacaccaaacca**

**Fortunella_venosa_3** **ggcgataataaatacagaagttgcggtcaataaagtagggatcatcaaaacaccaaacca**

1570	1580	1590	1600	1610	1620

....|....|....|....|....|....|....|....|....|....|....|....|

**Atalantia_kwangtungens** **accaatgtaaagtcggttttcagtgctagttatccagttacagaagcgaccccataggcg**

**Citrus_reticulata_1** **accaatgtaaagacggttttcagtgctagttatccagttacagaagcgaccccataggcg**

**Citrus_reticulata_2** **accaatgtaaagacggttttcagtgctagttatccagttacagaagcgaccccataggcg**

**Citrus_trifoliata_3** **accaatgtaaaggcggttttcagtgctagttatccagttacagaagcgaccccataggcg**

**Citrus_aurantiifolia** **accaatgtaaaggcggttttcagtgctagttatccagttacagaagcgaccccataggcg**

**Citrus_aurantium** **accaatgtaaaggcggttttcagtgctagttatccagttacagaagcgaccccataggcg**

**Citrus_cavaleriei** **accaatgtaaagacggttttcagtgctagttatccagttacagaagcgaccccataggcg**

**Citrus_hongheensis** **accaatgtaaaggcggttttcagtgctagttatccagttacagaagcgaccccataggcg**

**Citrus_limon** **accaatgtaaaggcggttttcagtgctagttatccagttacagaagcgaccccataggcg**

**Citrus_maxima** **accaatgtaaaggcggttttcagtgctagttatccagttacagaagcgaccccataggcg**

**Citrus_medica** **accaatgtaaaggcggttttcagtgctagttatccagttacagaagcgaccccataggcg**

**Citrus_polytrifolia** **accaatgtaaaggcggttttcagtgctagttatccagttacagaagcgaccccataggcg**

**Citrus_reticulata** **accaatgtaaagacggttttcagtgctagttatccagttacagaagcgaccccataggcg**

**Citrus_sinensis** **accaatgtaaaggcggttttcagtgctagttatccagttacagaagcgaccccataggcg**

**Fortunella_bawangica_1** **accaatgtaaaggcggttttcagtgctagttatccagttacagaagcgaccccataggcg**

**Fortunella_bawangica_2** **accaatgtaaaggcggttttcagtgctagttatccagttacagaagcgaccccataggcg**

**Fortunella_hindsii_1** **accaatgtaaaggcggttttcagtgctagttatccagttacagaagcgaccccataggcg**

**Fortunella_hindsii_2** **accaatgtaaaggcggttttcagtgctagttatccagttacagaagcgaccccataggcg**

**Fortunella_hindsii_3** **accaatgtaaaggcggttttcagtgctagttatccagttacagaagcgaccccataggcg**

**Fortunella_japonica_1** **accaatgtaaaggcggttttcagtgctagttatccagttacagaagcgaccccataggcg**

**Fortunella_japonica_2** **accaatgtaaaggcggttttcagtgctagttatccagttacagaagcgaccccataggcg**

**Fortunella_japonica_3** **accaatgtaaaggcggttttcagtgctagttatccagttacagaagcgaccccataggcg**

**Fortunella_japonica_4** **accaatgtaaaggcggttttcagtgctagttatccagttacagaagcgaccccataggcg**

**Fortunella_japonica_5** **accaatgtaaaggcggttttcagtgctagttatccagttacagaagcgaccccataggcg**

**Fortunella_japonica_6** **accaatgtaaaggcggttttcagtgctagttatccagttacagaagcgaccccataggcg**

**Fortunella_japonica_7** **accaatgtaaaggcggttttcagtgctagttatccagttacagaagcgaccccataggcg**

**Fortunella_japonica_8** **accaatgtaaaggcggttttcagtgctagttatccagttacagaagcgaccccataggcg**

**Fortunella_japonica_9** **accaatgtaaaggcggttttcagtgctagttatccagttacagaagcgaccccataggcg**

**Fortunella_japonica_10** **accaatgtaaaggcggttttcagtgctagttatccagttacagaagcgaccccataggcg**

**Fortunella_japonica_11** **accaatgtaaaggcggttttcagtgctagttatccagttacagaagcgaccccataggcg**

**Fortunella_japonica_12** **accaatgtaaaggcggttttcagtgctagttatccagttacagaagcgaccccataggcg**

**Fortunella_margarita_1** **accaatgtaaaggcggttttcagtgctagttatccagttacagaagcgaccccataggcg**

**Fortunella_margarita_2** **accaatgtaaaggcggttttcagtgctagttatccagttacagaagcgaccccataggcg**

**Fortunella_venosa_1** **accaatgtaaaggcggttttcagtgctagttatccagttacagaagcgaccccataggcg**

**Fortunella_venosa_2** **accaatgtaaaggcggttttcagtgctagttatccagttacagaagcgaccccataggcg**

**Fortunella_venosa_3** **accaatgtaaaggcggttttcagtgctagttatccagttacagaagcgaccccataggcg**

1630	1640	1650	1660	1670	1680

....|....|....|....|....|....|....|....|....|....|....|....|

**Atalantia_kwangtungens** **ttcgctttcgcgtctctctaaaattgcagtcatggtaaaatcttggtttatttaatcatc**

**Citrus_reticulata_1** **ttcgctttcgcgtctctctaaaattgcagtcatggtaaaatcttggtttatttaatcatc**

**Citrus_reticulata_2** **ttcgctttcgcgtctctctaaaattgcagtcatggtaaaatcttggtttatttaatcatc**

**Citrus_trifoliata_3** **ttcgctttcgcgtctctctaaaattgcagtcatggtaaaatcttggtttatttaatcatc**

**Citrus_aurantiifolia** **ttcgctttcgcgtctctctaaaattgcagtcatggtaaaatcttggtttatgtaatcatc**

**Citrus_aurantium** **ttcgctttcgcgtctctctaaaattgcagtcatggtaaaatcttggtttatttaatcatc**

**Citrus_cavaleriei** **ttcgctttcgcgtctctctaaaattgcagtcatggtaaaatcttggtttatttaatcatc**

**Citrus_hongheensis** **ttcgctttcgcgtctctctaaaattgcagtcatggtaaaatcttggtttatttaatcatc**

**Citrus_limon** **ttcgctttcgcgtctctctaaaattgcagtcatggtaaaatcttggtttatttaatcatc**

**Citrus_maxima** **ttcgctttcgcgtctctctaaaattgcagtcatggtaaaatcttggtttatttaatcatc**

**Citrus_medica** **ttcgctttcgcgtctctctaaaattgcagtcatggtaaaatcttggtttatttaatcatc**

**Citrus_polytrifolia** **ttcgctttcgcgtctctctaaaattgcagtcatggtaaaatcttggtttatttaatcatc**

**Citrus_reticulata** **ttcgctttcgcgtctctctaaaattgcagtcatggtaaaatcttggtttatttaatcatc**

**Citrus_sinensis** **ttcgctttcgcgtctctctaaaattgcagtcatggtaaaatcttggtttatttaatcatc**

**Fortunella_bawangica_1** **ttcgctttcgcgtctctctaaaattgcagtcatggtaaaatcttggtttatttaatcatc**

**Fortunella_bawangica_2** **ttcgctttcgcgtctctctaaaattgcagtcatggtaaaatcttggtttatttaatcatc**

**Fortunella_hindsii_1** **ttcgctttcgcgtctctctaaaattgcagtcatggtaaaatcttggtttatttaatcatc**

**Fortunella_hindsii_2** **ttcgctttcgcgtctctctaaaattgcagtcatggtaaaatcttggtttatttaatcatc**

**Fortunella_hindsii_3** **ttcgctttcgcgtctctctaaaattgcagtcatggtaaaatcttggtttatttaatcatc**

**Fortunella_japonica_1** **ttcgctttcgcgtctctctaaaattgcagtcatggtaaaatcttggtttatttaatcatc**

**Fortunella_japonica_2** **ttcgctttcgcgtctctctaaaattgcagtcatggtaaaatcttggtttatttaatcatc**

**Fortunella_japonica_3** **ttcgctttcgcgtctctctaaaattgcagtcatggtaaaatcttggtttatttaatcatc**

**Fortunella_japonica_4** **ttcgctttcgcgtctctctaaaattgcagtcatggtaaaatcttggtttatttaatcatc**

**Fortunella_japonica_5** **ttcgctttcgcgtctctctaaaattgcagtcatggtaaaatcttggtttatttaatcatc**

**Fortunella_japonica_6** **ttcgctttcgcgtctctctaaaattgcagtcatggtaaaatcttggtttatttaatcatc**

**Fortunella_japonica_7** **ttcgctttcgcgtctctctaaaattgcagtcatggtaaaatcttggtttatttaatcatc**

**Fortunella_japonica_8** **ttcgctttcgcgtctctctaaaattgcagtcatggtaaaatcttggtttatttaatcatc**

**Fortunella_japonica_9** **ttcgctttcgcgtctctctaaaattgcagtcatggtaaaatcttggtttatttaatcatc**

**Fortunella_japonica_10** **ttcgctttcgcgtctctctaaaattgcagtcatggtaaaatcttggtttatttaatcatc**

**Fortunella_japonica_11** **ttcgctttcgcgtctctctaaaattgcagtcatggtaaaatcttggtttatttaatcatc**

**Fortunella_japonica_12** **ttcgctttcgcgtctctctaaaattgcagtcatggtaaaatcttggtttatttaatcatc**

**Fortunella_margarita_1** **ttcgctttcgcgtctctctaaaattgcagtcatggtaaaatcttggtttatttaatcatc**

**Fortunella_margarita_2** **ttcgctttcgcgtctctctaaaattgcagtcatggtaaaatcttggtttatttaatcatc**

**Fortunella_venosa_1** **ttcgctttcgcgtctctctaaaattgcagtcatggtaaaatcttggtttatttaatcatc**

**Fortunella_venosa_2** **ttcgctttcgcgtctctctaaaattgcagtcatggtaaaatcttggtttatttaatcatc**

**Fortunella_venosa_3** **ttcgctttcgcgtctctctaaaattgcagtcatggtaaaatcttggtttatttaatcatc**

1690	1700	1710	1720	1730	1740

....|....|....|....|....|....|....|....|....|....|....|....|

**Atalantia_kwangtungens** **agggactcccaagcatacaaattctgcaaaaagcgagaattgagggcttgttattcaaca**

**Citrus_reticulata_1** **agggactcccaagcatacaaattctgcaaaaagcgagaattgagggcttgttattcaaca**

**Citrus_reticulata_2** **agggactcccaagcatacaaattctgcaaaaagcgagaattgagggcttgttattcaaca**

**Citrus_trifoliata_3** **agggactcccaagcatacaaattctgcaaaaagcgagaattgagggcttgttattcaaca**

**Citrus_aurantiifolia** **agggactcccaagcatacaaattctgcaaaaagcgagaattgagggcttgttattcaaca**

**Citrus_aurantium** **agggactcccaagcatacaaattctgcaaaaagcgagaattgagggcttgttattcaaca**

**Citrus_cavaleriei** **agggactcccaagcatacaaattctgcaaaaagcgagaattgagggcttgttattcaaca**

**Citrus_hongheensis** **agggactcccaagcatacaaattctgcaaaaagcgagaattgagggcttgttattcaaca**

**Citrus_limon** **agggactcccaagcatacaaattctgcaaaaagcgagaattgagggcttgttattcaaca**

**Citrus_maxima** **agggactcccaagcatacaaattctgcaaaaagcgagaattgagggcttgttattcaaca**

**Citrus_medica** **agggactcccaagcatacaaattctgcaaaaagcgagaattgagggcttgttattcaaca**

**Citrus_polytrifolia** **agggactcccaagcatacaaattctgcaaaaagcgagaattgagggcttgttattcaaca**

**Citrus_reticulata** **agggactcccaagcatacaaattctgcaaaaagcgagaattgagggcttgttattcaaca**

**Citrus_sinensis** **agggactcccaagcatacaaattctgcaaaaagcgagaattgagggcttgttattcaaca**

**Fortunella_bawangica_1** **agggactcccaagcatacaaattctgcaaaaagcgagaattgagggcttgttattcaaca**

**Fortunella_bawangica_2** **agggactcccaagcatacaaattctgcaaaaagcgagaattgagggcttgttattcaaca**

**Fortunella_hindsii_1** **agggactcccaagcatacaaattctgcaaaaagcgagaattgagggcttgttattcaaca**

**Fortunella_hindsii_2** **agggactcccaagcatacaaattctgcaaaaagcgagaattgagggcttgttattcaaca**

**Fortunella_hindsii_3** **agggactcccaagcatacaaattctgcaaaaagcgagaattgagggcttgttattcaaca**

**Fortunella_japonica_1** **agggactcccaagcatacaaattctgcaaaaagcgagaattgagggcttgttattcaaca**

**Fortunella_japonica_2** **agggactcccaagcatacaaattctgcaaaaagcgagaattgagggcttgttattcaaca**

**Fortunella_japonica_3** **agggactcccaagcatacaaattctgcaaaaagcgagaattgagggcttgttattcaaca**

**Fortunella_japonica_4** **agggactcccaagcatacaaattctgcaaaaagcgagaattgagggcttgttattcaaca**

**Fortunella_japonica_5** **agggactcccaagcatacaaattctgcaaaaagcgagaattgagggcttgttattcaaca**

**Fortunella_japonica_6** **agggactcccaagcatacaaattctgcaaaaagcgagaattgagggcttgttattcaaca**

**Fortunella_japonica_7** **agggactcccaagcatacaaattctgcaaaaagcgagaattgagggcttgttattcaaca**

**Fortunella_japonica_8** **agggactcccaagcatacaaattctgcaaaaagcgagaattgagggcttgttattcaaca**

**Fortunella_japonica_9** **agggactcccaagcatacaaattctgcaaaaagcgagaattgagggcttgttattcaaca**

**Fortunella_japonica_10** **agggactcccaagcatacaaattctgcaaaaagcgagaattgagggcttgttattcaaca**

**Fortunella_japonica_11** **agggactcccaagcatacaaattctgcaaaaagcgagaattgagggcttgttattcaaca**

**Fortunella_japonica_12** **agggactcccaagcatacaaattctgcaaaaagcgagaattgagggcttgttattcaaca**

**Fortunella_margarita_1** **agggactcccaagcatacaaattctgcaaaaagcgagaattgagggcttgttattcaaca**

**Fortunella_margarita_2** **agggactcccaagcatacaaattctgcaaaaagcgagaattgagggcttgttattcaaca**

**Fortunella_venosa_1** **agggactcccaagcatacaaattctgcaaaaagcgagaattgagggcttgttattcaaca**

**Fortunella_venosa_2** **agggactcccaagcatacaaattctgcaaaaagcgagaattgagggcttgttattcaaca**

**Fortunella_venosa_3** **agggactcccaagcatacaaattctgcaaaaagcgagaattgagggcttgttattcaaca**

1750	1760	1770	1780	1790	1800

....|....|....|....|....|....|....|....|....|....|....|....|

**Atalantia_kwangtungens** **gtataacatgacttatatctccgtgtcaaccaacaatccatagataaatattcatgatct**

**Citrus_reticulata_1** **gtataacatgacttatatctccgtgtcaaccaacaatccatagataaatattcatg----**

**Citrus_reticulata_2** **gtataacatgacttatatctccgtgtcaaccaacaatccatagataaatattcatg----**

**Citrus_trifoliata_3** **gtataacatgacttatatcttcgtgtcaaccaacaatccatagataaatattcatg----**

**Citrus_aurantiifolia** **gtataacatgacttatatctccgtgtcaaccaacaatccatagataaatattcatg----**

**Citrus_aurantium** **gtataacatgacttatatctccgtgtcaaccaacaatccatagataaatattcatg----**

**Citrus_cavaleriei** **gtataacatgacttatatctccgtgtcaaccaacaatccatagataaatattcatg----**

**Citrus_hongheensis** **gtataacatgacttatatctccgtgtcaaccaacaatccatagataaatattcatg----**

**Citrus_limon** **gtataacatgacttatatctccgtgtcaaccaacaatccatagataaatattcatg----**

**Citrus_maxima** **gtataacatgacttatatctccgtgtcaaccaacaatccatagataaatattcatg----**

**Citrus_medica** **gtataacatgacttatatctccgtgtcaaccaacaatccatagataaatattcatg----**

**Citrus_polytrifolia** **gtataacatgacttatatcttcgtgtcaaccaacaatccatagataaatattcatg----**

**Citrus_reticulata** **gtataacatgacttatatctccgtgtcaaccaacaatccatagataaatattcatg----**

**Citrus_sinensis** **gtataacatgacttatatctccgtgtcaaccaacaatccatagataaatattcatg----**

**Fortunella_bawangica_1** **gtataacatgacttatatctccgtgtcaaccaacaatccatagataaatattcatg----**

**Fortunella_bawangica_2** **gtataacatgacttatatctccgtgtcaaccaacaatccatagataaatattcatg----**

**Fortunella_hindsii_1** **gtataacatgacttatatctccgtgtcaaccaacaatccatagataaatattcatg----**

**Fortunella_hindsii_2** **gtataacatgacttatatctccgtgtcaaccaacaatccatagataaatattcatg----**

**Fortunella_hindsii_3** **gtataacatgacttatatctccgtgtcaaccaacaatccatagataaatattcatg----**

**Fortunella_japonica_1** **gtataacatgacttatatctccgtgtcaaccaacaatccatagataaatattcatg----**

**Fortunella_japonica_2** **gtataacatgacttatatctccgtgtcaaccaacaatccatagataaatattcatg----**

**Fortunella_japonica_3** **gtataacatgacttatatctccgtgtcaaccaacaatccatagataaatattcatg----**

**Fortunella_japonica_4** **gtataacatgacttatatctccgtgtcaaccaacaatccatagataaatattcatg----**

**Fortunella_japonica_5** **gtataacatgacttatatctccgtgtcaaccaacaatccatagataaatattcatg----**

**Fortunella_japonica_6** **gtataacatgacttatatctccgtgtcaaccaacaatccatagataaatattcatg----**

**Fortunella_japonica_7** **gtataacatgacttatatctccgtgtcaaccaacaatccatagataaatattcatg----**

**Fortunella_japonica_8** **gtataacatgacttatatctccgtgtcaaccaacaatccatagataaatattcatg----**

**Fortunella_japonica_9** **gtataacatgacttatatctccgtgtcaaccaacaatccatagataaatattcatg----**

**Fortunella_japonica_10** **gtataacatgacttatatctccgtgtcaaccaacaatccatagataaatattcatg----**

**Fortunella_japonica_11** **gtataacatgacttatatctccgtgtcaaccaacaatccatagataaatattcatg----**

**Fortunella_japonica_12** **gtataacatgacttatatctccgtgtcaaccaacaatccatagataaatattcatg----**

**Fortunella_margarita_1** **gtataacatgacttatatctccgtgtcaaccaacaatccatagataaatattcatg----**

**Fortunella_margarita_2** **gtataacatgacttatatctccgtgtcaaccaacaatccatagataaatattcatg----**

**Fortunella_venosa_1** **gtataacatgacttatatctccgtgtcaaccaacaatccatagataaatattcatg----**

**Fortunella_venosa_2** **gtataacatgacttatatctccgtgtcaaccaacaatccatagataaatattcatg----**

**Fortunella_venosa_3** **gtataacatgacttatatctccgtgtcaaccaacaatccatagataaatattcatg----**

1810	1820	1830	1840	1850	1860

....|....|....|....|....|....|....|....|....|....|....|....|

**Atalantia_kwangtungens** **tatcttatctagattccgattcagaaaaaat-----------------------------**

**Citrus_reticulata_1** **-atcttgtctagattccgattccgaataaattattattattat-----------------**

**Citrus_reticulata_2** **-atcttgtctagattccgattccgaataaattattattattat-----------------**

**Citrus_trifoliata_3** **-atcttgtctagattccgattcagaataaattattattattatttttttttt-ttttttt**

**Citrus_aurantiifolia** **-atcttgtctagattccgattcagaataaattattattattattat------------tt**

**Citrus_aurantium** **-atcttgtctagattccgattcagaataaattattattattatta--------------t**

**Citrus_cavaleriei** **-atcttgtctagattccgattcagaataaattattattattattct------------tt**

**Citrus_hongheensis** **-atcttgtctagattccgattcagaataaattattattattattt---------------**

**Citrus_limon** **-atcttgtctagattccgattcagaataaattattattattatta--------------t**

**Citrus_maxima** **-atcttgtctagattccgattcagaataaattattattattattat------------tt**

**Citrus_medica** **-atcttgtctagattccgattcagaataaattattattattattt---------------**

**Citrus_polytrifolia** **-atcttgtctagattccgattcagaataaattattattattatttttttttt-tttttga**

**Citrus_reticulata** **-atcttgtctagattccgattccgaataaattattattattat-----------------**

**Citrus_sinensis** **-atcttgtctagattccgattcagaataaattattattattatta--------------t**

**Fortunella_bawangica_1** **-atcttgtctagattccgattcagaataaattattattattattaattatta--tttttt**

**Fortunella_bawangica_2** **-atcttgtctagattccgattcagaataaattattattattattaattatta--tttttt**

**Fortunella_hindsii_1** **-atcttgtctagattccgattcagaataaattattattattattaattattatttttttt**

**Fortunella_hindsii_2** **-atcttgtctagattccgattcagaataaattattattattattaattatta-ttttttt**

**Fortunella_hindsii_3** **-atcttgtctagattccgattcagaataaattattattattattaattatta-ttttttt**

**Fortunella_japonica_1** **-atcttgtctagattccgattcagaataaattattattattattaattatta--tttttt**

**Fortunella_japonica_2** **-atcttgtctagattccgattcagaataaattattattattattaattatta--tttttt**

**Fortunella_japonica_3** **-atcttgtctagattccgattcagaataaattattattattattaattatta--tttttt**

**Fortunella_japonica_4** **-atcttgtctagattccgattcagaataaattattattattattaattatta--tttttt**

**Fortunella_japonica_5** **-atcttgtctagattccgattcagaataaattattattattattaattatta--tttttt**

**Fortunella_japonica_6** **-atcttgtctagattccgattcagaataaattattattattattaattatta--tttttt**

**Fortunella_japonica_7** **-atcttgtctagattccgattcagaataaattattattattattaattatta--tttttt**

**Fortunella_japonica_8** **-atcttgtctagattccgattcagaataaattattattattattaattatta--tttttt**

**Fortunella_japonica_9** **-atcttgtctagattccgattcagaataaattattattattattaattatta--tttttt**

**Fortunella_japonica_10** **-atcttgtctagattccgattcagaataaattattattattattaattatta--tttttt**

**Fortunella_japonica_11** **-atcttgtctagattccgattcagaataaattattattattattaattatta--tttttt**

**Fortunella_japonica_12** **-atcttgtctagattccgattcagaataaattattattattattaattatta--tttttt**

**Fortunella_margarita_1** **-atcttgtctagattccgattcagaataaattattattattattaattatta-ttttttt**

**Fortunella_margarita_2** **-atcttgtctagattccgattcagaataaattattattattattaattatta-ttttttt**

**Fortunella_venosa_1** **-atcttgtctagattccgattcagaataaattattattattattaattattc--ttttt-**

**Fortunella_venosa_2** **-atcttgtctagattccgattcagaataaattattattattattaattatta--ttttt-**

**Fortunella_venosa_3** **-atcttgtctagattccgattcagaataaattattattattattaattatta--tttttt**

1870	1880	1890	1900	1910	1920

....|....|....|....|....|....|....|....|....|....|....|....|

**Atalantia_kwangtungens** **gcttttttttttttttgaagtagtaagttcaaaaaattgaatacctattta-tatcgata**

**Citrus_reticulata_1** **--tattttttttttttgaagtagtaagttaaaaaaattgaatacctattta-tatcgata**

**Citrus_reticulata_2** **--tattttttttttttgaagtagtaagttaaaaaaattgaatacctattta-tatcgata**

**Citrus_trifoliata_3** **ttttttatttttttttgaagtagtaagttaaaaaaattgaatacctattta-tatcgata**

**Citrus_aurantiifolia** **tttttttttttattttgaagtagtaagttaaaaaaattgaatacctattta-tatcgata**

**Citrus_aurantium** **ttttttttattattttgaagtagtaagttaaaaaaattgaatacctattta-tatcgata**

**Citrus_cavaleriei** **ttttttttttttttttgaagtagtaagttaaaaaaatggaatacctattta-tatcgata**

**Citrus_hongheensis** **---------tttttttgaagtagtaagttaaaaaaatggaatacctattta-tatcgata**

**Citrus_limon** **ttttttttattattttgaagtagtaagttaaaaaaattgaatacctattta-tatcgata**

**Citrus_maxima** **ttttttttattattttgaagtagtaagttaaaaaaattgaatacctattta-tatcgata**

**Citrus_medica** **--tttttttttattttgaagtagtaagttaaaaaaattgaatacctattta-tatcgata**

**Citrus_polytrifolia** **agtagtaagttaaaaagaagtagtaagttaaaaaaattgaatacctattta-tatcgata**

**Citrus_reticulata** **--tattttttttttttgaagtagtaagttaaaaaaattgaatacctattta-tatcgata**

**Citrus_sinensis** **ttttttttattattttgaagtagtaagttaaaaaaattgaatacctattta-tatcgata**

**Fortunella_bawangica_1** **ttttttattttattttgaagtagtaagttaaaaaaattgaatacctatttc-tatcgata**

**Fortunella_bawangica_2** **ttttttattttattttgaagtagtaagttaaaaaaattgaatacctatttc-tatcgata**

**Fortunella_hindsii_1** **ttttttattttattttgaagtagtaagttaaaaaaattgaatacctatttc-tatcgata**

**Fortunella_hindsii_2** **ttttttattttattttgaagtagtaagttaaaaaaattgaatacctatttc-tatcgata**

**Fortunella_hindsii_3** **ttttttattttattttgaagtagtaagttaaaaaaattgaatacctatttc-tatcgata**

**Fortunella_japonica_1** **t-ttttattttattttgaagtagtaagttaaaaaaattgaatacctatttc-tatcgata**

**Fortunella_japonica_2** **--ttttattttattttgaagtagtaagttaaaaaaattgaatacctatttcatatcgata**

**Fortunella_japonica_3** **t-ttttattttattttgaagtagtaagttaaaaaaattgaatacctatttc-tatcgata**

**Fortunella_japonica_4** **t-ttttattttattttgaagtagtaagttaaaaaaattgaatacctatttc-tatcgata**

**Fortunella_japonica_5** **t-ttttattttattttgaagtagtaagttaaaaaaattgaatacctatttc-tatcgata**

**Fortunella_japonica_6** **t-ttttattttattttgaagtagtaagttaaaaaaattgaatacctatttc-tatcgata**

**Fortunella_japonica_7** **t-ttttattttattttgaagtagtaagttaaaaaaattgaatacctatttc-tatcgata**

**Fortunella_japonica_8** **t-ttttattttattttgaagtagtaagttaaaaaaattgaatacctatttc-tatcgata**

**Fortunella_japonica_9** **t-ttttattttattttgaagtagtaagttaaaaaaattgaatacctatttc-tatcgata**

**Fortunella_japonica_10** **ttttttattttattttgaagtagtaagttaaaaaaattgaatacctatttc-tatcgata**

**Fortunella_japonica_11** **ttttttattttattttgaagtagtaagttaaaaaaattgaatacctatttc-tatcgata**

**Fortunella_japonica_12** **t-ttttattttattttgaagtagtaagttaaaaaaattgaatacctatttc-tatcgata**

**Fortunella_margarita_1** **ttttttattttattttgaagtagtaagttaaaaaaattgaatacctatttc-tatcgata**

**Fortunella_margarita_2** **ttttttattttattttgaagtagtaagttaaaaaaattgaatacctatttc-tatcgata**

**Fortunella_venosa_1** **ttttttattttattttgaagtagtaagttaaaaaaattgaatacctatttc-tatcgata**

**Fortunella_venosa_2** **ttttttattttattttgaagtagtaagttaaaaaaattgaatacctatttc-tatcgata**

**Fortunella_venosa_3** **ttttttattttattttgaagtagtaagttaaaaaaattgaatacctatttc-tatcgata**

1930	1940	1950	1960	1970	1980

....|....|....|....|....|....|....|....|....|....|....|....|

**Atalantia_kwangtungens** **tatgatg-------tcaatataataatcagaatgggttgcccgggactcgaacccggaac**

**Citrus_reticulata_1** **tatgatg-------tcaatataataataagaatgggttgcccgggactcgaacccggaac**

**Citrus_reticulata_2** **tatgatg-------tcaatataataataagaatgggttgcccgggactcgaacccggaac**

**Citrus_trifoliata_3** **tatgatg-------tcaatataataataagaatgggttgcccgggactcgaacccggaac**

**Citrus_aurantiifolia** **tatgatg-------tcaatataataataagaatgggttgcccgggactcgaacccggaac**

**Citrus_aurantium** **tatgatg-------tcaatataataataagaatgggttgcccgggactcgaacccggaac**

**Citrus_cavaleriei** **tatgatg-------tcaatataataataagaatgggttgcccgggactcgaacccggaac**

**Citrus_hongheensis** **tatgatg-------tcaatataataataagaatgggttgcccgggactcgaacccggaac**

**Citrus_limon** **tatgatg-------tcaatataataataagaatgggttgcccgggactcgaacccggaac**

**Citrus_maxima** **tatgatg-------tcaatataataataagaatgggttgcccgggactcgaacccggaac**

**Citrus_medica** **tatgatg-------tcaatataataataagaatgggttgcccgggactcgaacccggaac**

**Citrus_polytrifolia** **tatgatg-------tcaatataataataagaatgggttgcccgggactcgaacccggaac**

**Citrus_reticulata** **tatgatg-------tcaatataataataagaatgggttgcccgggactcgaacccggaac**

**Citrus_sinensis** **tatgatg-------tcaatataataataagaatgggttgcccgggactcgaacccggaac**

**Fortunella_bawangica_1** **tatgatg-------tcaatataataataagaatgggttgcccgggactcgaacccggaac**

**Fortunella_bawangica_2** **tatgatg-------tcaatataataataagaatgggttgcccgggactcgaacccggaac**

**Fortunella_hindsii_1** **tatgatg-------tcaatataataataagaatgggttgcccgggactcgaacccggaac**

**Fortunella_hindsii_2** **tatgatgtcaatattcaatataataataagaatgggttgcccgggactcgaacccggaac**

**Fortunella_hindsii_3** **tatgatg-------tcaatataataataagaatgggttgcccgggactcgaacccggaac**

**Fortunella_japonica_1** **tatgatg-------tcaatataataataagaatgggttgcccgggactcgaacccggaac**

**Fortunella_japonica_2** **tatgatg-------tcaatataataataagaatgggttgcccgggactcgaacccggaac**

**Fortunella_japonica_3** **tatgatg-------tcaatataataataagaatgggttgcccgggactcgaacccggaac**

**Fortunella_japonica_4** **tatgatg-------tcaatataataataagaatgggttgcccgggactcgaacccggaac**

**Fortunella_japonica_5** **tatgatg-------tcaatataataataagaatgggttgcccgggactcgaacccggaac**

**Fortunella_japonica_6** **tatgatg-------tcaatataataataagaatgggttgcccgggactcgaacccggaac**

**Fortunella_japonica_7** **tatgatg-------tcaatataataataagaatgggttgcccgggactcgaacccggaac**

**Fortunella_japonica_8** **tatgatg-------tcaatataataataagaatgggttgcccgggactcgaacccggaac**

**Fortunella_japonica_9** **tatgatg-------tcaatataataataagaatgggttgcccgggactcgaacccggaac**

**Fortunella_japonica_10** **tatgatg-------tcaatataataataagaatgggttgcccgggactcgaacccggaac**

**Fortunella_japonica_11** **tatgatg-------tcaatataataataagaatgggttgcccgggactcgaacccggaac**

**Fortunella_japonica_12** **tatgatg-------tcaatataataataagaatgggttgcccgggactcgaacccggaac**

**Fortunella_margarita_1** **tatgatg-------tcaatataataataagaatgggttgcccgggactcgaacccggaac**

**Fortunella_margarita_2** **tatgatg-------tcaatataataataagaatgggttgcccgggactcgaacccggaac**

**Fortunella_venosa_1** **tatgatg-------tcaatataataataagaatgggttgcccgggactcgaacccggaac**

**Fortunella_venosa_2** **tatgatg-------tcaatataataataagaatgggttgcccgggactcgaacccggaac**

**Fortunella_venosa_3** **tatgatg-------tcaatataataataagaatgggttgcccgggactcgaacccggaac**

1990	2000	2010	2020	2030	2040

....|....|....|....|....|....|....|....|....|....|....|....|

**Atalantia_kwangtungens** **tagtcggatggagtagaaaacttccttgttaaataggtaaacaaaatccctccccaagcc**

**Citrus_reticulata_1** **tagtcggatggagtagaaaacttccttgttaaataggtaaaaaaaatccctccccaagcc**

**Citrus_reticulata_2** **tagtcggatggagtagaaaacttccttgttaaataggtaaaaaaaatccctccccaagcc**

**Citrus_trifoliata_3** **tagtcggatggagtagaaaacttccttgtgaaataggtaaacaaaatccctccccaagcc**

**Citrus_aurantiifolia** **tagtcggatggagtagaaaacttccttgttaaataggtaaacaaaatccctccccaagcc**

**Citrus_aurantium** **tagtcggatggagtagaaaacttccttgttaaataggtaaacaaaatccctccccaagcc**

**Citrus_cavaleriei** **tagtcggatggagtagaaaacttccttgttaaataggtaaaaaaaatccctccccaagcc**

**Citrus_hongheensis** **tagtcggatggagtagaaaacttccttgttaaataggtaaacaaaatccctccccaagcc**

**Citrus_limon** **tagtcggatggagtagaaaacttccttgttaaataggtaaacaaaatccctccccaagcc**

**Citrus_maxima** **tagtcggatggagtagaaaacttccttgttaaataggtaaacaaaatctctccccaagcc**

**Citrus_medica** **tagtcggatggagtagaaaacttccttgttaaataggtaaacaaaatccctccccaagcc**

**Citrus_polytrifolia** **tagtcggatggagtagaaaacttccttgtgaaataggtaaacaaaatccctccccaagcc**

**Citrus_reticulata** **tagtcggatggagtagaaaacttccttgttaaataggtaaaaaaaatccctccccaagcc**

**Citrus_sinensis** **tagtcggatggagtagaaaacttccttgttaaataggtaaacaaaatccctccccaagcc**

**Fortunella_bawangica_1** **tagtcggatggagtagaaaacttccttgttaaataggtaaacaaaatccctccccaagcc**

**Fortunella_bawangica_2** **tagtcggatggagtagaaaacttccttgttaaataggtaaacaaaatccctccccaagcc**

**Fortunella_hindsii_1** **tagtcggatggagtagaaaacttccttgttaaataggtaaacaaaatccctccccaagcc**

**Fortunella_hindsii_2** **tagtcggatggagtagaaaacttccttgttaaataggtaaacaaaatccctccccaagcc**

**Fortunella_hindsii_3** **tagtcggatggagtagaaaacttccttgttaaataggtaaacaaaatccctccccaagcc**

**Fortunella_japonica_1** **tagtcggatggagtagaaaacttccttgttaaataggtaaacaaaatccctccccaagcc**

**Fortunella_japonica_2** **tagtcggatggagtagaaaacttccttgttaaataggtaaacaaaatccctccccaagcc**

**Fortunella_japonica_3** **tagtcggatggagtagaaaacttccttgttaaataggtaaacaaaatccctccccaagcc**

**Fortunella_japonica_4** **tagtcggatggagtagaaaacttccttgttaaataggtaaacaaaatccctccccaagcc**

**Fortunella_japonica_5** **tagtcggatggagtagaaaacttccttgttaaataggtaaacaaaatccctccccaagcc**

**Fortunella_japonica_6** **tagtcggatggagtagaaaacttccttgttaaataggtaaacaaaatccctccccaagcc**

**Fortunella_japonica_7** **tagtcggatggagtagaaaacttccttgttaaataggtaaacaaaatccctccccaagcc**

**Fortunella_japonica_8** **tagtcggatggagtagaaaacttccttgttaaataggtaaacaaaatccctccccaagcc**

**Fortunella_japonica_9** **tagtcggatggagtagaaaacttccttgttaaataggtaaacaaaatccctccccaagcc**

**Fortunella_japonica_10** **tagtcggatggagtagaaaacttccttgttaaataggtaaacaaaatccctccccaagcc**

**Fortunella_japonica_11** **tagtcggatggagtagaaaacttccttgttaaataggtaaacaaaatccctccccaagcc**

**Fortunella_japonica_12** **tagtcggatggagtagaaaacttccttgttaaataggtaaacaaaatccctccccaagcc**

**Fortunella_margarita_1** **tagtcggatggagtagaaaacttccttgttaaataggtaaacaaaatccctccccaagcc**

**Fortunella_margarita_2** **tagtcggatggagtagaaaacttccttgttaaataggtaaacaaaatccctccccaagcc**

**Fortunella_venosa_1** **tagtcggatggagtagaaaacttccttgttaaataggtaaacaaaatccctccccaagcc**

**Fortunella_venosa_2** **tagtcggatggagtagaaaacttccttgttaaataggtaaacaaaatccctccccaagcc**

**Fortunella_venosa_3** **tagtcggatggagtagaaaacttccttgttaaataggtaaacaaaatccctccccaagcc**

2050	2060	2070	2080	2090	2100

....|....|....|....|....|....|....|....|....|....|....|....|

**Atalantia_kwangtungens** **gtgcatgcatttttcattgcacacagctttccctatgtataacatcaaaaactccctttc**

**Citrus_reticulata_1** **gtgcatgcatttttcattgcacacagctttccctatgtataacatcaaaaactccctttc**

**Citrus_reticulata_2** **gtgcatgcatttttcattgcacacagctttccctatgtataacatcaaaaactccctttc**

**Citrus_trifoliata_3** **gtgcatgcatttttcattgcacacagctttccctatgtataacatcaaaaactccctttc**

**Citrus_aurantiifolia** **gtgcatgcatttttcattgcacacagctttccctatgtataacatcaaaaactccctttc**

**Citrus_aurantium** **gtgcatgcatttttcattgcacacagctttccctatgtataacatcaaaaactccctttc**

**Citrus_cavaleriei** **gtgcatgcatttttcattgcacacagctttccctatgtataacatcaaaaactccctttc**

**Citrus_hongheensis** **gtgcatgcatttttcattgcacacagctttccctatgtataacatcaaaaactccctttc**

**Citrus_limon** **gtgcatgcatttttcattgcacacagctttccctatgtataacatcaaaaactccctttc**

**Citrus_maxima** **gtgcatgcatttttcattgcacacagctttccctatgtataacatcaaaaactccctttc**

**Citrus_medica** **gtgcatgcatttttcattgcacacagctttccctatgtataacatcaaaaactccctttc**

**Citrus_polytrifolia** **gtgcatgcatttttcattgcacacagctttccctatgtataacatcaaaaactccctttc**

**Citrus_reticulata** **gtgcatgcatttttcattgcacacagctttccctatgtataacatcaaaaactccctttc**

**Citrus_sinensis** **gtgcatgcatttttcattgcacacagctttccctatgtataacatcaaaaactccctttc**

**Fortunella_bawangica_1** **gtgcatgcatttttcattgcacacagctttccctatgtataacatcaaaaactccctttc**

**Fortunella_bawangica_2** **gtgcatgcatttttcattgcacacagctttccctatgtataacatcaaaaactccctttc**

**Fortunella_hindsii_1** **gtgcatgcatttttcattgcacacagctttccctatgtataacatcaaaaactccctttc**

**Fortunella_hindsii_2** **gtgcatgcatttttcattgcacacagctttccctatgtataacatcaaaaactccctttc**

**Fortunella_hindsii_3** **gtgcatgcatttttcattgcacacagctttccctatgtataacatcaaaaactccctttc**

**Fortunella_japonica_1** **gtgcatgcatttttcattgcacacagctttccctatgtataacatcaaaaactccctttc**

**Fortunella_japonica_2** **gtgcatgcatttttcattgcacacagctttccctatgtataacatcaaaaactccctttc**

**Fortunella_japonica_3** **gtgcatgcatttttcattgcacacagctttccctatgtataacatcaaaaactccctttc**

**Fortunella_japonica_4** **gtgcatgcatttttcattgcacacagctttccctatgtataacatcaaaaactccctttc**

**Fortunella_japonica_5** **gtgcatgcatttttcattgcacacagctttccctatgtataacatcaaaaactccctttc**

**Fortunella_japonica_6** **gtgcatgcatttttcattgcacacagctttccctatgtataacatcaaaaactccctttc**

**Fortunella_japonica_7** **gtgcatgcatttttcattgcacacagctttccctatgtataacatcaaaaactccctttc**

**Fortunella_japonica_8** **gtgcatgcatttttcattgcacacagctttccctatgtataacatcaaaaactccctttc**

**Fortunella_japonica_9** **gtgcatgcatttttcattgcacacagctttccctatgtataacatcaaaaactccctttc**

**Fortunella_japonica_10** **gtgcatgcatttttcattgcacacagctttccctatgtataacatcaaaaactccctttc**

**Fortunella_japonica_11** **gtgcatgcatttttcattgcacacagctttccctatgtataacatcaaaaactccctttc**

**Fortunella_japonica_12** **gtgcatgcatttttcattgcacacagctttccctatgtataacatcaaaaactccctttc**

**Fortunella_margarita_1** **gtgcatgcatttttcattgcacacagctttccctatgtataacatcaaaaactccctttc**

**Fortunella_margarita_2** **gtgcatgcatttttcattgcacacagctttccctatgtataacatcaaaaactccctttc**

**Fortunella_venosa_1** **gtgcatgcatttttcattgcacacagctttccctatgtataacatcaaaaactccctttc**

**Fortunella_venosa_2** **gtgcatgcatttttcattgcacacagctttccctatgtataacatcaaaaactccctttc**

**Fortunella_venosa_3** **gtgcatgcatttttcattgcacacagctttccctatgtataacatcaaaaactccctttc**

2110	2120	2130	2140	2150	2160

....|....|....|....|....|....|....|....|....|....|....|....|

**Atalantia_kwangtungens** **ttcccaaatccctaactaagacact------aaaaaagttgaataactcaattaatcaac**

**Citrus_reticulata_1** **ttcccaaatc-----ctaagacact------aaaaaagttgaataactcaattaatcaac**

**Citrus_reticulata_2** **ttcccaaatc-----ctaagacact------aaaaaagttgaataactcaattaatcaac**

**Citrus_trifoliata_3** **ttcccaaatc-----ctaagacact------aaaaaagttgaataactcacttaatcaac**

**Citrus_aurantiifolia** **ttcccaaatc-----ctaagacact------caaaaagttgaataactcaattaatcaac**

**Citrus_aurantium** **ttcccaaatc-----ctaagacatt------aaaaaagttgaataactcaattaatcaac**

**Citrus_cavaleriei** **ttcccaaatc-----ctaagacact------aaaaaagttgaataactcaattaatcaac**

**Citrus_hongheensis** **ttcccaaatc-----ctaagacact------aaaaaagttgaataactcaattaatcaac**

**Citrus_limon** **ttcccaaatc-----ctaagacatt------aaaaaagttgaataactcaattaatcaac**

**Citrus_maxima** **ttcccaaatc-----ctaagacattaaaaaaaaaaaagttgaataactcaattaatcaac**

**Citrus_medica** **ttcccaaatc-----ctaagacact------aaaaaagttgaataactcaattaatcaac**

**Citrus_polytrifolia** **ttcccaaatc-----ctaagacact------aaaaaagttgaataactcaattaatcaac**

**Citrus_reticulata** **ttcccaaatc-----ctaagacact------aaaaaagttgaataactcaattaatcaac**

**Citrus_sinensis** **ttcccaaatc-----ctaagacatt------aaaaaagttgaataactcaattaatcaac**

**Fortunella_bawangica_1** **ttcccaaatc-----ctaagacact------aaaaaagttgaataactcaattaatcaac**

**Fortunella_bawangica_2** **ttcccaaatc-----ctaagacact------aaaaaagttgaataactcaattaatcaac**

**Fortunella_hindsii_1** **ttcccaaatc-----ctaagacact------aaaaaagttgaataactcaattaatcaac**

**Fortunella_hindsii_2** **ttcccaaatc-----ctaagacact------aaaaaagttgaataactcaattaatcaac**

**Fortunella_hindsii_3** **ttcccaaatc-----ctaagacact------aaaaaagttgaataactcaattaatcaac**

**Fortunella_japonica_1** **ttcccaaatc-----ctaagacact------aaaaaagttgaataactcaattaatcaac**

**Fortunella_japonica_2** **ttcccaaatc-----ctaagacact------aaaaaagttgaataactcaattaatcaac**

**Fortunella_japonica_3** **ttcccaaatc-----ctaagacact------aaaaaagttgaataactcaattaatcaac**

**Fortunella_japonica_4** **ttcccaaatc-----ctaagacact------aaaaaagttgaataactcaattaatcaac**

**Fortunella_japonica_5** **ttcccaaatc-----ctaagacact------aaaaaagttgaataactcaattaatcaac**

**Fortunella_japonica_6** **ttcccaaatc-----ctaagacact------aaaaaagttgaataactcaattaatcaac**

**Fortunella_japonica_7** **ttcccaaatc-----ctaagacact------aaaaaagttgaataactcaattaatcaac**

**Fortunella_japonica_8** **ttcccaaatc-----ctaagacact------aaaaaagttgaataactcaattaatcaac**

**Fortunella_japonica_9** **ttcccaaatc-----ctaagacact------aaaaaagttgaataactcaattaatcaac**

**Fortunella_japonica_10** **ttcccaaatc-----ctaagacact------aaaaaagttgaataactcaattaatcaac**

**Fortunella_japonica_11** **ttcccaaatc-----ctaagacact------aaaaaagttgaataactcaattaatcaac**

**Fortunella_japonica_12** **ttcccaaatc-----ctaagacact------aaaaaagttgaataactcaattaatcaac**

**Fortunella_margarita_1** **ttcccaaatc-----ctaagacact------aaaaaagttgaataactcaattaatcaac**

**Fortunella_margarita_2** **ttcccaaatc-----ctaagacact------aaaaaagttgaataactcaattaatcaac**

**Fortunella_venosa_1** **ttcccaaatc-----ctaagacact------aaaaaagttgaataactcaattaatcaac**

**Fortunella_venosa_2** **ttcccaaatc-----ctaagacact------aaaaaagttgaataactcaattaatcaac**

**Fortunella_venosa_3** **ttcccaaatc-----ctaagacact------aaaaaagttgaataactcaattaatcaac**

2170	2180	2190	2200	2210	2220

....|....|....|....|....|....|....|....|....|....|....|....|

**Atalantia_kwangtungens** **ctttattttattattcttattgtatgaacatt------taatacaaatgaatgaattttt**

**Citrus_reticulata_1** **ctttattttattattcttattgtatgaacatt------taatacaaatgaatgcattttt**

**Citrus_reticulata_2** **ctttattttattattcttattgtatgaacatt------taatacaaatgaatgcattttt**

**Citrus_trifoliata_3** **ctttattttattattcttattgtatgaacatttaatactaatacaaatgaatgaattttt**

**Citrus_aurantiifolia** **ctttattttattattcttattgtatgaacatt------taatacaaatgaatgaattttt**

**Citrus_aurantium** **ctttattttattattcttattgtatgaacatt------taatacaaatgaatgaattttt**

**Citrus_cavaleriei** **ctttattttattattcttattgtctgaacatt------taatacaaatgaatgaattttt**

**Citrus_hongheensis** **ctttattttattattcttattgtatgaacatt------taatacaaatgaatgaattttt**

**Citrus_limon** **ctttattttattattcttattgtatgaacatt------taatacaaatgaatgaattttt**

**Citrus_maxima** **ctttattttattattcttattgtatgaacatt------taatacaaatgaatgaattttt**

**Citrus_medica** **ctttattttattattcttattgtatgaacatt------taatacaaatgaatgaattttt**

**Citrus_polytrifolia** **ctttattttattattcttattgtatgaacatttaatactaatacaaatgaatgaattttt**

**Citrus_reticulata** **ctttattttattattcttattgtatgaacatt------taatacaaatgaatgcattttt**

**Citrus_sinensis** **ctttattttattattcttattgtatgaacatt------taatacaaatgaatgaattttt**

**Fortunella_bawangica_1** **ctttattttattattcttattgtatgaacatt------taatacaaatgaatgaattttt**

**Fortunella_bawangica_2** **ctttattttattattcttattgtatgaacatt------taatacaaatgaatgaattttt**

**Fortunella_hindsii_1** **ctttattttattattcttattgtatgaacatt------taatacaaatgaatgaattttt**

**Fortunella_hindsii_2** **ctttattttattattcttattgtatgaacatt------taatacaaatgaatgaattttt**

**Fortunella_hindsii_3** **ctttattttattattcttattgtatgaacatt------taatacaaatgaatgaattttt**

**Fortunella_japonica_1** **ctttattttattattcttattgtatgaacatt------taatacaaatgaatgaattttt**

**Fortunella_japonica_2** **ctttattttattattcttattgtatgaacatt------taatacaaatgaatgaattttt**

**Fortunella_japonica_3** **ctttattttattattcttattgtatgaacatt------taatacaaatgaatgaattttt**

**Fortunella_japonica_4** **ctttattttattattcttattgtatgaacatt------taatacaaatgaatgaattttt**

**Fortunella_japonica_5** **ctttattttattattcttattgtatgaacatt------taatacaaatgaatgaattttt**

**Fortunella_japonica_6** **ctttattttattattcttattgtatgaacatt------taatacaaatgaatgaattttt**

**Fortunella_japonica_7** **ctttattttattattcttattgtatgaacatt------taatacaaatgaatgaattttt**

**Fortunella_japonica_8** **ctttattttattattcttattgtatgaacatt------taatacaaatgaatgaattttt**

**Fortunella_japonica_9** **ctttattttattattcttattgtatgaacatt------taatacaaatgaatgaattttt**

**Fortunella_japonica_10** **ctttattttattattcttattgtatgaacatt------taatacaaatgaatgaattttt**

**Fortunella_japonica_11** **ctttattttattattcttattgtatgaacatt------taatacaaatgaatgaattttt**

**Fortunella_japonica_12** **ctttattttattattcttattgtatgaacatt------taatacaaatgaatgaattttt**

**Fortunella_margarita_1** **ctttattttattattcttattgtatgaacatt------taatacaaatgaatgaattttt**

**Fortunella_margarita_2** **ctttattttattattcttattgtatgaacatt------taatacaaatgaatgaattttt**

**Fortunella_venosa_1** **ctttattttattattcttattgtatgaacatt------taatacaaatgaatgaattttt**

**Fortunella_venosa_2** **ctttattttattattcttattgtatgaacatt------taatacaaatgaatgaattttt**

**Fortunella_venosa_3** **ctttattttattattcttattgtatgaacatt------taatacaaatgaatgaattttt**

2230	2240	2250	2260	2270	2280

....|....|....|....|....|....|....|....|....|....|....|....|

**Atalantia_kwangtungens** **ttgttatcgattgattatttaggaatcatttccaaattacatagtctcgtaaccaatcat**

**Citrus_reticulata_1** **ttgttatcgattgattgtttaggaatcatttcaaaatgacatagtctcgtaaccaatcat**

**Citrus_reticulata_2** **ttgttatcgattgattgtttaggaatcatttcaaaatgacatagtctcgtaaccaatcat**

**Citrus_trifoliata_3** **ttgttatcgattgattgtttaggaatcatttcaaaatgacatagtctcgtaaccaatcat**

**Citrus_aurantiifolia** **ttgttatcgattgattgtttaggaatcatttcaaaatgacatagtctcgtaaccaatcat**

**Citrus_aurantium** **ttgttatcgattgattgtttaggaatcatttcaaaatgacatagtctcgtaaccaatcat**

**Citrus_cavaleriei** **ttgttatcgattgattgtttaggaatcatttcaaaatgacatagtctcgtaaccaatcat**

**Citrus_hongheensis** **ttgttatcgattgattgtttaggaatcatttcaaaatgacatagtctcgtaaccaatcat**

**Citrus_limon** **ttgttatcgattgattgtttaggaatcatttcaaaatgacatagtctcgtaaccaatcat**

**Citrus_maxima** **ttgttatcgattgattgtttaggaatcatttcaaaatgacatagtctcgtaaccaatcat**

**Citrus_medica** **ttgttatcgattgattgtttaggaatcatttcaaaatgacatagtctcgtaaccaatcat**

**Citrus_polytrifolia** **ttgttatcgattgattgtttaggaatcatttcaaaatgacatagtctcgtaaccaatcat**

**Citrus_reticulata** **ttgttatcgattgattgtttaggaatcatttcaaaatgacatagtctcgtaaccaatcat**

**Citrus_sinensis** **ttgttatcgattgattgtttaggaatcatttcaaaatgacatagtctcgtaaccaatcat**

**Fortunella_bawangica_1** **ttgttatcgattgattgtttaggaatcatttcaaaatgacatagtctcgtaaccaatcat**

**Fortunella_bawangica_2** **ttgttatcgattgattgtttaggaatcatttcaaaatgacatagtctcgtaaccaatcat**

**Fortunella_hindsii_1** **ttgttatcgattgattgtttaggaatcatttcaaaatgacatagtctcgtaaccaatcat**

**Fortunella_hindsii_2** **ttgttatcgattgattgtttaggaatcatttcaaaatgacatagtctcgtaaccaatcat**

**Fortunella_hindsii_3** **ttgttatcgattgattgtttaggaatcatttcaaaatgacatagtctcgtaaccaatcat**

**Fortunella_japonica_1** **ttgttatcgattgattgtttaggaatcatttcaaaatgacatagtctcgtaaccaatcat**

**Fortunella_japonica_2** **ttgttatcgattgattgtttaggaatcatttcaaaatgacatagtctcgtaaccaatcat**

**Fortunella_japonica_3** **ttgttatcgattgattgtttaggaatcatttcaaaatgacatagtctcgtaaccaatcat**

**Fortunella_japonica_4** **ttgttatcgattgattgtttaggaatcatttcaaaatgacatagtctcgtaaccaatcat**

**Fortunella_japonica_5** **ttgttatcgattgattgtttaggaatcatttcaaaatgacatagtctcgtaaccaatcat**

**Fortunella_japonica_6** **ttgttatcgattgattgtttaggaatcatttcaaaatgacatagtctcgtaaccaatcat**

**Fortunella_japonica_7** **ttgttatcgattgattgtttaggaatcatttcaaaatgacatagtctcgtaaccaatcat**

**Fortunella_japonica_8** **ttgttatcgattgattgtttaggaatcatttcaaaatgacatagtctcgtaaccaatcat**

**Fortunella_japonica_9** **ttgttatcgattgattgtttaggaatcatttcaaaatgacatagtctcgtaaccaatcat**

**Fortunella_japonica_10** **ttgttatcgattgattgtttaggaatcatttcaaaatgacatagtctcgtaaccaatcat**

**Fortunella_japonica_11** **ttgttatcgattgattgtttaggaatcatttcaaaatgacatagtctcgtaaccaatcat**

**Fortunella_japonica_12** **ttgttatcgattgattgtttaggaatcatttcaaaatgacatagtctcgtaaccaatcat**

**Fortunella_margarita_1** **ttgttatcgattgattgtttaggaatcatttcaaaatgacatagtctcgtaaccaatcat**

**Fortunella_margarita_2** **ttgttatcgattgattgtttaggaatcatttcaaaatgacatagtctcgtaaccaatcat**

**Fortunella_venosa_1** **ttgttatcgattgattgtttaggaatcatttcaaaatgacatagtctcgtaaccaatcat**

**Fortunella_venosa_2** **ttgttatcgattgattgtttaggaatcatttcaaaatgacatagtctcgtaaccaatcat**

**Fortunella_venosa_3** **ttgttatcgattgattgtttaggaatcatttcaaaatgacatagtctcgtaaccaatcat**

2290	2300	2310	2320	2330	2340

....|....|....|....|....|....|....|....|....|....|....|....|

**Atalantia_kwangtungens** **ttataattaaccagatcgttgatacaaaaaatatccaaataccaaatccgtcctctatat**

**Citrus_reticulata_1** **tgacaattaaccagatcgttgatacaaaaaatatccaaataccaaatccgtcctctatat**

**Citrus_reticulata_2** **tgacaattaaccagatcgttgatacaaaaaatatccaaataccaaatccgtcctctatat**

**Citrus_trifoliata_3** **tgataattaaccagatcgttgatacaaaaaatatccaaataccaaatccgtcctctatat**

**Citrus_aurantiifolia** **tgataattaaccagatcgttgatacaaaaaatatccaaataccaaatccgtcctctatat**

**Citrus_aurantium** **tgataattaaccagatcgttgatacaaaaaatatccaaataccaaatccgtcctctatat**

**Citrus_cavaleriei** **tgacaattaaccagatcgttgatacaaaaaatatccaaataccaaatccgtcctctatat**

**Citrus_hongheensis** **tgataattaaccagatcgttgatacaaaaaatatccaaataccaaatccgtcctctatat**

**Citrus_limon** **tgataattaaccagatcgttgatacaaaaaatatccaaataccaaatccgtcctctatat**

**Citrus_maxima** **tgataattaaccagatcgttgatacaaaaaatatccaaataccaaatccgtcctctatat**

**Citrus_medica** **tgataattaaccagatcgttgatacaaaaaatatccaaataccaaatccgtcctctatat**

**Citrus_polytrifolia** **tgataattaaccagatcgttgatacaaaaaatatccaaataccaaatccgtcctctatat**

**Citrus_reticulata** **tgacaattaaccagatcgttgatacaaaaaatatccaaataccaaatccgtcctctatat**

**Citrus_sinensis** **tgataattaaccagatcgttgatacaaaaaatatccaaataccaaatccgtcctctatat**

**Fortunella_bawangica_1** **tgataattaaacagatcgttgatacaaaaaatatccaaataccaaatccgtcctctatat**

**Fortunella_bawangica_2** **tgataattaaacagatcgttgatacaaaaaatatccaaataccaaatccgtcctctatat**

**Fortunella_hindsii_1** **tgataattaaacagatcgttgatacaaaaaatatccaaataccaaatccgtcctctatat**

**Fortunella_hindsii_2** **tgataattaaacagatcgttgatacaaaaaatatccaaataccaaatccgtcctctatat**

**Fortunella_hindsii_3** **tgataattaaacagatcgttgatacaaaaaatatccaaataccaaatccgtcctctatat**

**Fortunella_japonica_1** **tgataattaaacagatcgttgatacaaaaaatatccaaataccaaatccgtcctctatat**

**Fortunella_japonica_2** **tgataattaaacagatcgttgatacaaaaaatatccaaataccaaatccgtcctctatat**

**Fortunella_japonica_3** **tgataattaaacagatcgttgatacaaaaaatatccaaataccaaatccgtcctctatat**

**Fortunella_japonica_4** **tgataattaaacagatcgttgatacaaaaaatatccaaataccaaatccgtcctctatat**

**Fortunella_japonica_5** **tgataattaaacagatcgttgatacaaaaaatatccaaataccaaatccgtcctctatat**

**Fortunella_japonica_6** **tgataattaaacagatcgttgatacaaaaaatatccaaataccaaatccgtcctctatat**

**Fortunella_japonica_7** **tgataattaaacagatcgttgatacaaaaaatatccaaataccaaatccgtcctctatat**

**Fortunella_japonica_8** **tgataattaaacagatcgttgatacaaaaaatatccaaataccaaatccgtcctctatat**

**Fortunella_japonica_9** **tgataattaaacagatcgttgatacaaaaaatatccaaataccaaatccgtcctctatat**

**Fortunella_japonica_10** **tgataattaaacagatcgttgatacaaaaaatatccaaataccaaatccgtcctctatat**

**Fortunella_japonica_11** **tgataattaaacagatcgttgatacaaaaaatatccaaataccaaatccgtcctctatat**

**Fortunella_japonica_12** **tgataattaaacagatcgttgatacaaaaaatatccaaataccaaatccgtcctctatat**

**Fortunella_margarita_1** **tgataattaaacagatcgttgatacaaaaaatatccaaataccaaatccgtcctctatat**

**Fortunella_margarita_2** **tgataattaaacagatcgttgatacaaaaaatatccaaataccaaatccgtcctctatat**

**Fortunella_venosa_1** **tgataattaaacagatcgttgatacaaaaaatatccaaataccaaatccgtcctctatat**

**Fortunella_venosa_2** **tgataattaaacagatcgttgatacaaaaaatatccaaataccaaatccgtcctctatat**

**Fortunella_venosa_3** **tgataattaaacagatcgttgatacaaaaaatatccaaataccaaatccgtcctctatat**

2350	2360	2370	2380	2390	2400

....|....|....|....|....|....|....|....|....|....|....|....|

**Atalantia_kwangtungens** **aaatagaacctacgcgaagtagacgaagctcttggaaagagtaaagcaagaacgtgttct**

**Citrus_reticulata_1** **aaatagaacctacgcgaagtagacgaagctcttggaaagagtaaagcaagaacgtgttct**

**Citrus_reticulata_2** **aaatagaacctacgcgaagtagacgaagctcttggaaagagtaaagcaagaacgtgttct**

**Citrus_trifoliata_3** **aaatagaacctacgcgaagtagacgaagctcttggaaagagtaaagcaagaacgtgttct**

**Citrus_aurantiifolia** **aaatagaacctacgcgaagtagacgaagctcttggaacgagtaaagcaagaacgtgttct**

**Citrus_aurantium** **aaatagaacctacgcgaagtagacgaagctcttggaaagagtaaagcaagaacgtgttct**

**Citrus_cavaleriei** **aaatagaacctacgcgaagtagacgaagctcttggaaagagtaaagcaagaacgtgttct**

**Citrus_hongheensis** **aaatagaacctacgcgaagtagacgaagctcttggaaagagtaaagcaagaacgtgttct**

**Citrus_limon** **aaatagaacctacgcgaagtagacgaagctcttggaaagagtaaagcaagaacgtgttct**

**Citrus_maxima** **aaatagaacctacgcgaagtagacgaagctcttggaaagagtaaagcaagaacgtgttct**

**Citrus_medica** **aaatagaacctacgcgaagtagacgaagctcctggaaaaagtaaagcaagaacgtgttct**

**Citrus_polytrifolia** **aaatagaacctacgcgaagtagacgaagctcttggaaagagtaaagcaagaacgtgttct**

**Citrus_reticulata** **aaatagaacctacgcgaagtagacgaagctcttggaaagagtaaagcaagaacgtgttct**

**Citrus_sinensis** **aaatagaacctacgcgaagtagacgaagctcttggaaagagtaaagcaagaacgtgttct**

**Fortunella_bawangica_1** **aaatagaacctacgcgaagtagacgaagctcttggaaagagtaaagcaagaacgtgttct**

**Fortunella_bawangica_2** **aaatagaacctacgcgaagtagacgaagctcttggaaagagtaaagcaagaacgtgttct**

**Fortunella_hindsii_1** **aaatagaacctacgcgaagtagacgaagctcttggaaagagtaaagcaagaacgtgttct**

**Fortunella_hindsii_2** **aaatagaacctacgcgaagtagacgaagctcttggaaagagtaaagcaagaacgtgttct**

**Fortunella_hindsii_3** **aaatagaacctacgcgaagtagacgaagctcttggaaagagtaaagcaagaacgtgttct**

**Fortunella_japonica_1** **aaatagaacctacgcgaagtagacgaagctcttggaaagagtaaagcaagaacgtgttct**

**Fortunella_japonica_2** **aaatagaacctacgcgaagtagacgaagctcttggaaagagtaaagcaagaacgtgttct**

**Fortunella_japonica_3** **aaatagaacctacgcgaagtagacgaagctcttggaaagagtaaagcaagaacgtgttct**

**Fortunella_japonica_4** **aaatagaacctacgcgaagtagacgaagctcttggaaagagtaaagcaagaacgtgttct**

**Fortunella_japonica_5** **aaatagaacctacgcgaagtagacgaagctcttggaaagagtaaagcaagaacgtgttct**

**Fortunella_japonica_6** **aaatagaacctacgcgaagtagacgaagctcttggaaagagtaaagcaagaacgtgttct**

**Fortunella_japonica_7** **aaatagaacctacgcgaagtagacgaagctcttggaaagagtaaagcaagaacgtgttct**

**Fortunella_japonica_8** **aaatagaacctacgcgaagtagacgaagctcttggaaagagtaaagcaagaacgtgttct**

**Fortunella_japonica_9** **aaatagaacctacgcgaagtagacgaagctcttggaaagagtaaagcaagaacgtgttct**

**Fortunella_japonica_10** **aaatagaacctacgcgaagtagacgaagctcttggaaagagtaaagcaagaacgtgttct**

**Fortunella_japonica_11** **aaatagaacctacgcgaagtagacgaagctcttggaaagagtaaagcaagaacgtgttct**

**Fortunella_japonica_12** **aaatagaacctacgcgaagtagacgaagctcttggaaagagtaaagcaagaacgtgttct**

**Fortunella_margarita_1** **aaatagaacctacgcgaagtagacgaagctcttggaaagagtaaagcaagaacgtgttct**

**Fortunella_margarita_2** **aaatagaacctacgcgaagtagacgaagctcttggaaagagtaaagcaagaacgtgttct**

**Fortunella_venosa_1** **aaatagaacctacgcgaagtagacgaagctcttggaaagagtaaagcaagaacgtgttct**

**Fortunella_venosa_2** **aaatagaacctacgcgaagtagacgaagctcttggaaagagtaaagcaagaacgtgttct**

**Fortunella_venosa_3** **aaatagaacctacgcgaagtagacgaagctcttggaaagagtaaagcaagaacgtgttct**

2410	2420	2430	2440	2450	2460

....|....|....|....|....|....|....|....|....|....|....|....|

**Atalantia_kwangtungens** **tcctccataaggaattcttccaataatgccgaacctaatctttttaaaaaagcgcgtaca**

**Citrus_reticulata_1** **tcctccataaggaattcttccaataattccgaacctaatctttgtaaaaaagcgcgtaca**

**Citrus_reticulata_2** **tcctccataaggaattcttccaataattccgaacctaatctttgtaaaaaagcgcgtaca**

**Citrus_trifoliata_3** **tcctccataaggaattcttccaataattccgaacctaatctttttaaaaaagcgcgtaca**

**Citrus_aurantiifolia** **tcctccataaggaattcttccaataattccgaacctaatctttttaaaaaagcgcgtaca**

**Citrus_aurantium** **tcctccataaggaattcttccaataattccgaacctaatctttttaaaaaagcgcgtaca**

**Citrus_cavaleriei** **tcctccataaggaattcttccaataattccgaacctaatctttttaaaaaagcgcgtaca**

**Citrus_hongheensis** **tcctccataaggaattcttccaataattccgaacctaatctttttaaaaaagcgcgtaca**

**Citrus_limon** **tcctccataaggaattcttccaataattccgaacctaatctttttaaaaaagcgcgtaca**

**Citrus_maxima** **tcctccataaggaattcttccaataattccgaacctaatctttttaaaaaagcgcgtaca**

**Citrus_medica** **tcctccataaggaattcttccaataattccgaacctaatctttttaaaaaagcgcgtaca**

**Citrus_polytrifolia** **tcctccataaggaattcttccaataattccgaacctaatctttttaaaaaagcgcgtaca**

**Citrus_reticulata** **tcctccataaggaattcttccaataattccgaacctaatctttgtaaaaaagcgcgtaca**

**Citrus_sinensis** **tcctccataaggaattcttccaataattccgaacctaatctttttaaaaaagcgcgtaca**

**Fortunella_bawangica_1** **tcctccataaggaattcttccaataattccgaacctaatctttttaaaaaagcgcgtaca**

**Fortunella_bawangica_2** **tcctccataaggaattcttccaataattccgaacctaatctttttaaaaaagcgcgtaca**

**Fortunella_hindsii_1** **tcctccataaggaattcttccaataattccgaacctaatctttttaaaaaagcgcgtaca**

**Fortunella_hindsii_2** **tcctccataaggaattcttccaataattccgaacctaatctttttaaaaaagcgcgtaca**

**Fortunella_hindsii_3** **tcctccataaggaattcttccaataattccgaacctaatctttttaaaaaagcgcgtaca**

**Fortunella_japonica_1** **tcctccataaggaattcttccaataattccgaacctaatctttttaaaaaagcgcgtaca**

**Fortunella_japonica_2** **tcctccataaggaattcttccaataattccgaacctaatctttttaaaaaagcgcgtaca**

**Fortunella_japonica_3** **tcctccataaggaattcttccaataattccgaacctaatctttttaaaaaagcgcgtaca**

**Fortunella_japonica_4** **tcctccataaggaattcttccaataattccgaacctaatctttttaaaaaagcgcgtaca**

**Fortunella_japonica_5** **tcctccataaggaattcttccaataattccgaacctaatctttttaaaaaagcgcgtaca**

**Fortunella_japonica_6** **tcctccataaggaattcttccaataattccgaacctaatctttttaaaaaagcgcgtaca**

**Fortunella_japonica_7** **tcctccataaggaattcttccaataattccgaacctaatctttttaaaaaagcgcgtaca**

**Fortunella_japonica_8** **tcctccataaggaattcttccaataattccgaacctaatctttttaaaaaagcgcgtaca**

**Fortunella_japonica_9** **tcctccataaggaattcttccaataattccgaacctaatctttttaaaaaagcgcgtaca**

**Fortunella_japonica_10** **tcctccataaggaattcttccaataattccgaacctaatctttttaaaaaagcgcgtaca**

**Fortunella_japonica_11** **tcctccataaggaattcttccaataattccgaacctaatctttttaaaaaagcgcgtaca**

**Fortunella_japonica_12** **tcctccataaggaattcttccaataattccgaacctaatctttttaaaaaagcgcgtaca**

**Fortunella_margarita_1** **tcctccataaggaattcttccaataattccgaacctaatctttttaaaaaagcgcgtaca**

**Fortunella_margarita_2** **tcctccataaggaattcttccaataattccgaacctaatctttttaaaaaagcgcgtaca**

**Fortunella_venosa_1** **tcctccataaggaattcttccaataattccgaacctaatctttttaaaaaagcgcgtaca**

**Fortunella_venosa_2** **tcctccataaggaattcttccaataattccgaacctaatctttttaaaaaagcgcgtaca**

**Fortunella_venosa_3** **tcctccataaggaattcttccaataattccgaacctaatctttttaaaaaagcgcgtaca**

2470	2480	2490	2500	2510	2520

....|....|....|....|....|....|....|....|....|....|....|....|

**Atalantia_kwangtungens** **gtacttttatgtttacgaaccaaacttttaacacaagaaagccgaagtatatattttact**

**Citrus_reticulata_1** **gtacttttatgtttacgaaccaaacttttaacacaagaaagccgaagtatatattttact**

**Citrus_reticulata_2** **gtacttttatgtttacgaaccaaacttttaacacaagaaagccgaagtatatattttact**

**Citrus_trifoliata_3** **gtacttttatgtttacgaaccaaacttttaacacaagaaagccgaagtatatattttact**

**Citrus_aurantiifolia** **gtacttttatgtttacgaaccaaacttttaacacaagaaagccgaagtatatattttact**

**Citrus_aurantium** **gtacttttatgtttacgaaccaaacttttaacacaagaaagccgaagtatatattttact**

**Citrus_cavaleriei** **gtacttttatgtttacgaaccaaacttttaacacaagaaagccgaagtatatattttact**

**Citrus_hongheensis** **gtacttttatgtttacgaaccaaacttttaacacaagaaagccgaagtatatattttact**

**Citrus_limon** **gtacttttatgtttacgaaccaaacttttaacacaagaaagccgaagtatatattttact**

**Citrus_maxima** **gtacttttatgtttacgaaccaaacttttaacacaagaaagccgaagtatatattttact**

**Citrus_medica** **gtacttttatgtttacgaaccaaacttttaacacaagaaagccgaagtatatattttact**

**Citrus_polytrifolia** **gtacttttatgtttacgaaccaaacttttaacacaagaaagccgaagtatatattttact**

**Citrus_reticulata** **gtacttttatgtttacgaaccaaacttttaacacaagaaagccgaagtatatattttact**

**Citrus_sinensis** **gtacttttatgtttacgaaccaaacttttaacacaagaaagccgaagtatatattttact**

**Fortunella_bawangica_1** **gtacttttatgtttacgaaccaaacttttaacacaagaaagccgaagtatatattttact**

**Fortunella_bawangica_2** **gtacttttatgtttacgaaccaaacttttaacacaagaaagccgaagtatatattttact**

**Fortunella_hindsii_1** **gtacttttatgtttacgaaccaaacttttaacacaagaaagccgaagtatatattttact**

**Fortunella_hindsii_2** **gtacttttatgtttacgaaccaaacttttaacacaagaaagccgaagtatatattttact**

**Fortunella_hindsii_3** **gtacttttatgtttacgaaccaaacttttaacacaagaaagccgaagtatatattttact**

**Fortunella_japonica_1** **gtacttttatgtttacgaaccaaacttttaacacaagaaagccgaagtatatattttact**

**Fortunella_japonica_2** **gtacttttatgtttacgaaccaaacttttaacacaagaaagccgaagtatatattttact**

**Fortunella_japonica_3** **gtacttttatgtttacgaaccaaacttttaacacaagaaagccgaagtatatattttact**

**Fortunella_japonica_4** **gtacttttatgtttacgaaccaaacttttaacacaagaaagccgaagtatatattttact**

**Fortunella_japonica_5** **gtacttttatgtttacgaaccaaacttttaacacaagaaagccgaagtatatattttact**

**Fortunella_japonica_6** **gtacttttatgtttacgaaccaaacttttaacacaagaaagccgaagtatatattttact**

**Fortunella_japonica_7** **gtacttttatgtttacgaaccaaacttttaacacaagaaagccgaagtatatattttact**

**Fortunella_japonica_8** **gtacttttatgtttacgaaccaaacttttaacacaagaaagccgaagtatatattttact**

**Fortunella_japonica_9** **gtacttttatgtttacgaaccaaacttttaacacaagaaagccgaagtatatattttact**

**Fortunella_japonica_10** **gtacttttatgtttacgaaccaaacttttaacacaagaaagccgaagtatatattttact**

**Fortunella_japonica_11** **gtacttttatgtttacgaaccaaacttttaacacaagaaagccgaagtatatattttact**

**Fortunella_japonica_12** **gtacttttatgtttacgaaccaaacttttaacacaagaaagccgaagtatatattttact**

**Fortunella_margarita_1** **gtacttttatgtttacgaaccaaacttttaacacaagaaagccgaagtatatattttact**

**Fortunella_margarita_2** **gtacttttatgtttacgaaccaaacttttaacacaagaaagccgaagtatatattttact**

**Fortunella_venosa_1** **gtacttttatgtttacgaaccaaacttttaacacaagaaagccgaagtatatattttact**

**Fortunella_venosa_2** **gtacttttatgtttacgaaccaaacttttaacacaagaaagccgaagtatatattttact**

**Fortunella_venosa_3** **gtacttttatgtttacgaaccaaacttttaacacaagaaagccgaagtatatattttact**

2530	2540	2550	2560	2570	2580

....|....|....|....|....|....|....|....|....|....|....|....|

**Atalantia_kwangtungens** **cgatacaaacttttttttgttgaagatccgctgtaataatgagaaagatttctgcataga**

**Citrus_reticulata_1** **cgatacaaactttttttttttgaagatccgctgtaataatgagaaagatttctacatata**

**Citrus_reticulata_2** **cgatacaaactttttttttttgaagatccgctgtaataatgagaaagatttctacatata**

**Citrus_trifoliata_3** **cgatacaaactttttttttttgaagatccgctgtaataatgagaaagatttctacatata**

**Citrus_aurantiifolia** **cgatacaaactttttttttttgaagatccgctgtaataatgagaaagatttctacatata**

**Citrus_aurantium** **cgatacaaactttttttttttgaagatccgctgtaataatgagaaagatttctacatata**

**Citrus_cavaleriei** **cgatacaaactttttttttttgaagatccgctgtaataatgagaaagatttctacatata**

**Citrus_hongheensis** **cgatacaaactttttttttttgaagatccgctgtaataatgagaaagatttctacatata**

**Citrus_limon** **cgatacaaactttttttttttgaagatccgctgtaataatgagaaagatttctacatata**

**Citrus_maxima** **cgatacaaactttttttttttgaagatccgctgtaataatgagaaagatttctacatata**

**Citrus_medica** **cgatacaaactttttttttttgaagatccgctgtaataatgagaaagatttctacatata**

**Citrus_polytrifolia** **cgatacaaactttttttttttgaagatccgctgtaataatgagaaagatttctacatata**

**Citrus_reticulata** **cgatacaaactttttttttttgaagatccgctgtaataatgagaaagatttctacatata**

**Citrus_sinensis** **cgatacaaactttttttttttgaagatccgctgtaataatgagaaagatttctacatata**

**Fortunella_bawangica_1** **cgatacaaactttttttttttgaagatccgctgtaataatgagaaagatttctacatata**

**Fortunella_bawangica_2** **cgatacaaactttttttttttgaagatccgctgtaataatgagaaagatttctacatata**

**Fortunella_hindsii_1** **cgatacaaactttttttttttgaagatccgctgtaataatgagaaagatttctacatata**

**Fortunella_hindsii_2** **cgatacaaactttttttttttgaagatccgctgtaataatgagaaagatttctacatata**

**Fortunella_hindsii_3** **cgatacaaactttttttttttgaagatccgctgtaataatgagaaagatttctacatata**

**Fortunella_japonica_1** **cgatacaaactttttttttttgaagatccgctgtaataatgagaaagatttctacatata**

**Fortunella_japonica_2** **cgatacaaactttttttttttgaagatccgctgtaataatgagaaagatttctacatata**

**Fortunella_japonica_3** **cgatacaaactttttttttttgaagatccgctgtaataatgagaaagatttctacatata**

**Fortunella_japonica_4** **cgatacaaactttttttttttgaagatccgctgtaataatgagaaagatttctacatata**

**Fortunella_japonica_5** **cgatacaaactttttttttttgaagatccgctgtaataatgagaaagatttctacatata**

**Fortunella_japonica_6** **cgatacaaactttttttttttgaagatccgctgtaataatgagaaagatttctacatata**

**Fortunella_japonica_7** **cgatacaaactttttttttttgaagatccgctgtaataatgagaaagatttctacatata**

**Fortunella_japonica_8** **cgatacaaactttttttttttgaagatccgctgtaataatgagaaagatttctacatata**

**Fortunella_japonica_9** **cgatacaaactttttttttttgaagatccgctgtaataatgagaaagatttctacatata**

**Fortunella_japonica_10** **cgatacaaactttttttttttgaagatccgctgtaataatgagaaagatttctacatata**

**Fortunella_japonica_11** **cgatacaaactttttttttttgaagatccgctgtaataatgagaaagatttctacatata**

**Fortunella_japonica_12** **cgatacaaactttttttttttgaagatccgctgtaataatgagaaagatttctacatata**

**Fortunella_margarita_1** **cgatacaaactttttttttttgaagatccgctgtaataatgagaaagatttctacatata**

**Fortunella_margarita_2** **cgatacaaactttttttttttgaagatccgctgtaataatgagaaagatttctacatata**

**Fortunella_venosa_1** **cgatacaaactttttttttttgaagatccgctgtaataatgagaaagatttctacatata**

**Fortunella_venosa_2** **cgatacaaactttttttttttgaagatccgctgtaataatgagaaagatttctacatata**

**Fortunella_venosa_3** **cgatacaaactttttttttttgaagatccgctgtaataatgagaaagatttctacatata**

2590	2600	2610	2620	2630	2640

....|....|....|....|....|....|....|....|....|....|....|....|

**Atalantia_kwangtungens** **cgcacaaatcggtcgataagatgagaatcagagaaatcggcccaagtcgacttactgata**

**Citrus_reticulata_1** **cgcacaaatcggtcgataagatgagaatcagagaaatcggaccaagtcgacttactgata**

**Citrus_reticulata_2** **cgcacaaatcggtcgataagatgagaatcagagaaatcggaccaagtcgacttactgata**

**Citrus_trifoliata_3** **cgcacaaatcggtcgataagatgagaatcagagaaatcggaccaagtcgacttactgata**

**Citrus_aurantiifolia** **cgcacaaatcggtcgataagatgagaatcagagaaatcggaccaagtcgacttactgata**

**Citrus_aurantium** **cgcacaaatcggtcgataagatgagaatcagagaaatcggaccaagtcgacttactgata**

**Citrus_cavaleriei** **cgcacaaatcggtcgataagatgagaatcagagaaatcggaccaagtcgacttactgata**

**Citrus_hongheensis** **cgcacaaatcggtcgataagatgagaatcagagaaatcggaccaagtcgacttactgata**

**Citrus_limon** **cgcacaaatcggtcgataagatgagaatcagagaaatcggaccaagtcgacttactgata**

**Citrus_maxima** **cgcacaaatcggtcgataagatgagaatcagagaaatcggaccaagtcgacttactgata**

**Citrus_medica** **cgcacaaatcggtcgataagatgagaatcagagaaatcggaccaagtcgacttactgata**

**Citrus_polytrifolia** **cgcacaaatcggtcgataagatgagaatcagagaaatcggaccaagtcgacttactgata**

**Citrus_reticulata** **cgcacaaatcggtcgataagatgagaatcagagaaatcggaccaagtcgacttactgata**

**Citrus_sinensis** **cgcacaaatcggtcgataagatgagaatcagagaaatcggaccaagtcgacttactgata**

**Fortunella_bawangica_1** **cgcacaaatcggtcgataagatgagaatccgagaaatcggaccaagtcgacttactgata**

**Fortunella_bawangica_2** **cgcacaaatcggtcgataagatgagaatccgagaaatcggaccaagtcgacttactgata**

**Fortunella_hindsii_1** **cgcacaaatcggtcgataagatgagaatccgagaaatcggaccaagtcgacttactgata**

**Fortunella_hindsii_2** **cgcacaaatcggtcgataagatgagaatccgagaaatcggaccaagtcgacttactgata**

**Fortunella_hindsii_3** **cgcacaaatcggtcgataagatgagaatccgagaaatcggaccaagtcgacttactgata**

**Fortunella_japonica_1** **cgcacaaatcggtcgataagatgagaatccgagaaatcggaccaagtcgacttactgata**

**Fortunella_japonica_2** **cgcacaaatcggtcgataagatgagaatccgagaaatcggaccaagtcgacttactgata**

**Fortunella_japonica_3** **cgcacaaatcggtcgataagatgagaatccgagaaatcggaccaagtcgacttactgata**

**Fortunella_japonica_4** **cgcacaaatcggtcgataagatgagaatccgagaaatcggaccaagtcgacttactgata**

**Fortunella_japonica_5** **cgcacaaatcggtcgataagatgagaatccgagaaatcggaccaagtcgacttactgata**

**Fortunella_japonica_6** **cgcacaaatcggtcgataagatgagaatccgagaaatcggaccaagtcgacttactgata**

**Fortunella_japonica_7** **cgcacaaatcggtcgataagatgagaatccgagaaatcggaccaagtcgacttactgata**

**Fortunella_japonica_8** **cgcacaaatcggtcgataagatgagaatccgagaaatcggaccaagtcgacttactgata**

**Fortunella_japonica_9** **cgcacaaatcggtcgataagatgagaatccgagaaatcggaccaagtcgacttactgata**

**Fortunella_japonica_10** **cgcacaaatcggtcgataagatgagaatccgagaaatcggaccaagtcgacttactgata**

**Fortunella_japonica_11** **cgcacaaatcggtcgataagatgagaatccgagaaatcggaccaagtcgacttactgata**

**Fortunella_japonica_12** **cgcacaaatcggtcgataagatgagaatccgagaaatcggaccaagtcgacttactgata**

**Fortunella_margarita_1** **cgcacaaatcggtcgataagatgagaatccgagaaatcggaccaagtcgacttactgata**

**Fortunella_margarita_2** **cgcacaaatcggtcgataagatgagaatccgagaaatcggaccaagtcgacttactgata**

**Fortunella_venosa_1** **cgcacaaatcggtcgataagatgagaatccgagaaatcggaccaagtcgacttactgata**

**Fortunella_venosa_2** **cgcacaaatcggtcgataagatgagaatccgagaaatcggaccaagtcgacttactgata**

**Fortunella_venosa_3** **cgcacaaatcggtcgataagatgagaatccgagaaatcggaccaagtcgacttactgata**

2650	2660	2670	2680	2690	2700

....|....|....|....|....|....|....|....|....|....|....|....|

**Atalantia_kwangtungens** **ggatgccctaatgcgttacaaaaccgcgccttagtcaatgatccaatcaaatgaataatt**

**Citrus_reticulata_1** **ggatgccctaatgcgttacaaaaccgcgccttagtcaatgatccaatcagatgaataatt**

**Citrus_reticulata_2** **ggatgccctaatgcgttacaaaaccgcgccttagtcaatgatccaatcagatgaataatt**

**Citrus_trifoliata_3** **ggatgccctaatgcgttacaaaaccgcgccttagtcaatgatccaatcagatgaataatt**

**Citrus_aurantiifolia** **ggatgccctaatgcgttacaaaaccgcgccttagtcaatgatccaatcagatgaataatt**

**Citrus_aurantium** **ggatgccctaatgcgttacaaaaccgcgccttagtcaatgatccaatcagatgaataatt**

**Citrus_cavaleriei** **ggatgccctaatgcgttacaaaaccgcgccttagtcaatgatccaatcagatgaataatt**

**Citrus_hongheensis** **ggatgccctaatgcgttacaaaaccgcgccttagtcaatgatccaatcagatgaataatt**

**Citrus_limon** **ggatgccctaatgcgttacaaaaccgcgccttagtcaatgatccaatcagatgaataatt**

**Citrus_maxima** **ggatgccctaatgcgttacaaaaccgcgccttagtcaatgatccaatcagatgaataatt**

**Citrus_medica** **ggatgccctaatgcgttacaaaaccgcgccttagtcaatgatccaatcagatgaataatt**

**Citrus_polytrifolia** **ggatgccctaatgcgttacaaaaccgcgccttagtcaatgatccaatcagatgaataatt**

**Citrus_reticulata** **ggatgccctaatgcgttacaaaaccgcgccttagtcaatgatccaatcagatgaataatt**

**Citrus_sinensis** **ggatgccctaatgcgttacaaaaccgcgccttagtcaatgatccaatcagatgaataatt**

**Fortunella_bawangica_1** **ggatgccctaatgcgttacaaaaccgcgccttagtcaatgatccaatcagatgaataatt**

**Fortunella_bawangica_2** **ggatgccctaatgcgttacaaaaccgcgccttagtcaatgatccaatcagatgaataatt**

**Fortunella_hindsii_1** **ggatgccctaatgcgttacaaaaccgcgccttagtcaatgatccaatcagatgaataatt**

**Fortunella_hindsii_2** **ggatgccctaatgcgttacaaaaccgcgccttagtcaatgatccaatcagatgaataatt**

**Fortunella_hindsii_3** **ggatgccctaatgcgttacaaaaccgcgccttagtcaatgatccaatcagatgaataatt**

**Fortunella_japonica_1** **ggatgccctaatgcgttacaaaaccgcgccttagtcaatgatccaatcagatgaataatt**

**Fortunella_japonica_2** **ggatgccctaatgcgttacaaaaccgcgccttagtcaatgatccaatcagatgaataatt**

**Fortunella_japonica_3** **ggatgccctaatgcgttacaaaaccgcgccttagtcaatgatccaatcagatgaataatt**

**Fortunella_japonica_4** **ggatgccctaatgcgttacaaaaccgcgccttagtcaatgatccaatcagatgaataatt**

**Fortunella_japonica_5** **ggatgccctaatgcgttacaaaaccgcgccttagtcaatgatccaatcagatgaataatt**

**Fortunella_japonica_6** **ggatgccctaatgcgttacaaaaccgcgccttagtcaatgatccaatcagatgaataatt**

**Fortunella_japonica_7** **ggatgccctaatgcgttacaaaaccgcgccttagtcaatgatccaatcagatgaataatt**

**Fortunella_japonica_8** **ggatgccctaatgcgttacaaaaccgcgccttagtcaatgatccaatcagatgaataatt**

**Fortunella_japonica_9** **ggatgccctaatgcgttacaaaaccgcgccttagtcaatgatccaatcagatgaataatt**

**Fortunella_japonica_10** **ggatgccctaatgcgttacaaaaccgcgccttagtcaatgatccaatcagatgaataatt**

**Fortunella_japonica_11** **ggatgccctaatgcgttacaaaaccgcgccttagtcaatgatccaatcagatgaataatt**

**Fortunella_japonica_12** **ggatgccctaatgcgttacaaaaccgcgccttagtcaatgatccaatcagatgaataatt**

**Fortunella_margarita_1** **ggatgccctaatgcgttacaaaaccgcgccttagtcaatgatccaatcagatgaataatt**

**Fortunella_margarita_2** **ggatgccctaatgcgttacaaaaccgcgccttagtcaatgatccaatcagatgaataatt**

**Fortunella_venosa_1** **ggatgccctaatgcgttacaaaaccgcgccttagtcaatgatccaatcagatgaataatt**

**Fortunella_venosa_2** **ggatgccctaatgcgttacaaaaccgcgccttagtcaatgatccaatcagatgaataatt**

**Fortunella_venosa_3** **ggatgccctaatgcgttacaaaaccgcgccttagtcaatgatccaatcagatgaataatt**

2710	2720	2730	2740	2750	2760

....|....|....|....|....|....|....|....|....|....|....|....|

**Atalantia_kwangtungens** **ggaacggtcgtatcgaccttcttcatagaattacctattagaaatgaattttctagcatt**

**Citrus_reticulata_1** **ggaacggtcgtatcgaccttcttcatagaattatctattagaaatgaattttctagcatt**

**Citrus_reticulata_2** **ggaacggtcgtatcgaccttcttcatagaattatctattagaaatgaattttctagcatt**

**Citrus_trifoliata_3** **ggaacggtcgtatcgaccttcttcatagaattatctattagaaatgaattttctagcatt**

**Citrus_aurantiifolia** **ggaacggtcgtatcgaccttcttcatagaattatctattagaaatgaattttctagcatt**

**Citrus_aurantium** **ggaacggtcgtatcgaccttcttcatagaattatctattagaaatgaattttctagcatt**

**Citrus_cavaleriei** **ggaacggtcgtatcgaccttcttcatagaattatctattagaaatgaattttctagcatt**

**Citrus_hongheensis** **ggaacggtcgtatcgaccttcttcatagaattatctattagaaatgaattttctagcatt**

**Citrus_limon** **ggaacggtcgtatcgaccttcttcatagaattatctattagaaatgaattttctagcatt**

**Citrus_maxima** **ggaacggtcgtatcgaccttcttcatagaattatctattagaaatgaattttctagcatt**

**Citrus_medica** **ggaacggtcgtatcgaccttcttcatagaattatctattagaaatgaattttctagcatt**

**Citrus_polytrifolia** **ggaacggtcgtatcgaccttcttcatagaattatctattagaaatgaattttctagcatt**

**Citrus_reticulata** **ggaacggtcgtatcgaccttcttcatagaattatctattagaaatgaattttctagcatt**

**Citrus_sinensis** **ggaacggtcgtatcgaccttcttcatagaattatctattagaaatgaattttctagcatt**

**Fortunella_bawangica_1** **ggaacggtcgtatcgaccttcttcacagaattatctattagaaatgaattttctagcatt**

**Fortunella_bawangica_2** **ggaacggtcgtatcgaccttcttcacagaattatctattagaaatgaattttctagcatt**

**Fortunella_hindsii_1** **ggaacggtcgtatcgaccttcttcacagaattatctattagaaatgaattttctagcatt**

**Fortunella_hindsii_2** **ggaacggtcgtatcgaccttcttcacagaattatctattagaaatgaattttctagcatt**

**Fortunella_hindsii_3** **ggaacggtcgtatcgaccttcttcacagaattatctattagaaatgaattttctagcatt**

**Fortunella_japonica_1** **ggaacggtcgtatcgaccttcttcacagaattatctattagaaatgaattttctagcatt**

**Fortunella_japonica_2** **ggaacggtcgtatcgaccttcttcacagaattatctattagaaatgaattttctagcatt**

**Fortunella_japonica_3** **ggaacggtcgtatcgaccttcttcacagaattatctattagaaatgaattttctagcatt**

**Fortunella_japonica_4** **ggaacggtcgtatcgaccttcttcacagaattatctattagaaatgaattttctagcatt**

**Fortunella_japonica_5** **ggaacggtcgtatcgaccttcttcacagaattatctattagaaatgaattttctagcatt**

**Fortunella_japonica_6** **ggaacggtcgtatcgaccttcttcacagaattatctattagaaatgaattttctagcatt**

**Fortunella_japonica_7** **ggaacggtcgtatcgaccttcttcacagaattatctattagaaatgaattttctagcatt**

**Fortunella_japonica_8** **ggaacggtcgtatcgaccttcttcacagaattatctattagaaatgaattttctagcatt**

**Fortunella_japonica_9** **ggaacggtcgtatcgaccttcttcacagaattatctattagaaatgaattttctagcatt**

**Fortunella_japonica_10** **ggaacggtcgtatcgaccttcttcacagaattatctattagaaatgaattttctagcatt**

**Fortunella_japonica_11** **ggaacggtcgtatcgaccttcttcacagaattatctattagaaatgaattttctagcatt**

**Fortunella_japonica_12** **ggaacggtcgtatcgaccttcttcacagaattatctattagaaatgaattttctagcatt**

**Fortunella_margarita_1** **ggaacggtcgtatcgaccttcttcacagaattatctattagaaatgaattttctagcatt**

**Fortunella_margarita_2** **ggaacggtcgtatcgaccttcttcacagaattatctattagaaatgaattttctagcatt**

**Fortunella_venosa_1** **ggaacggtcgtatcgaccttcttcacagaattatctattagaaatgaattttctagcatt**

**Fortunella_venosa_2** **ggaacggtcgtatcgaccttcttcacagaattatctattagaaatgaattttctagcatt**

**Fortunella_venosa_3** **ggaacggtcgtatcgaccttcttcacagaattatctattagaaatgaattttctagcatt**

2770	2780	2790	2800	2810	2820

....|....|....|....|....|....|....|....|....|....|....|....|

**Atalantia_kwangtungens** **tgactccgtaccaacaaagaatttagtcgcacaccggaaagatagcccagaaagttaata**

**Citrus_reticulata_1** **tgactccgtaccaacaaagaatttagtcgcacaccggaaagatagcccagaaagttaata**

**Citrus_reticulata_2** **tgactccgtaccaacaaagaatttagtcgcacaccggaaagatagcccagaaagttaata**

**Citrus_trifoliata_3** **tgactccgtaccaacaaagaatttagtcgcacaccggaaagatagcccagaaagttaata**

**Citrus_aurantiifolia** **tgactccgtaccaacaaagaatttagtcgcacaccggaaagatagcccagaaagttaata**

**Citrus_aurantium** **tgactccgtaccaacaaagaatttagtcgcacaccggaaagatagcccagaaagttaata**

**Citrus_cavaleriei** **tgactccgtaccaacaaagaatttagtcgcacaccggaaagatagcccagaaagttaata**

**Citrus_hongheensis** **tgactccgtaccaacaaagaatttagtcgcacaccggaaagatagcccagaaaggtaata**

**Citrus_limon** **tgactccgtaccaacaaagaatttagtcgcacaccggaaagatagcccagaaagttaata**

**Citrus_maxima** **tgactccgtaccaacaaagaatttagtcgcacaccggaaagatagcccagaaagttaata**

**Citrus_medica** **tgactccgtaccaacaaagaatttagtcgcacaccggaaagatagcccagaaagttaata**

**Citrus_polytrifolia** **tgactccgtaccaacaaagaatttagtcgcacaccggaaagatagcccagaaagttaata**

**Citrus_reticulata** **tgactccgtaccaacaaagaatttagtcgcacaccggaaagatagcccagaaagttaata**

**Citrus_sinensis** **tgactccgtaccaacaaagaatttagtcgcacaccggaaagatagcccagaaagttaata**

**Fortunella_bawangica_1** **tgactccgtaccaacaaagaatttagtcgcacaccggaaagatagcccagaaagttaata**

**Fortunella_bawangica_2** **tgactccgtaccaacaaagaatttagtcgcacaccggaaagatagcccagaaagttaata**

**Fortunella_hindsii_1** **tgactccgtaccaacaaagaatttagtcgcacaccggaaagatagcccagaaagttaata**

**Fortunella_hindsii_2** **tgactccgtaccaacaaagaatttagtcgcacaccggaaagatagcccagaaagttaata**

**Fortunella_hindsii_3** **tgactccgtaccaacaaagaatttagtcgcacaccggaaagatagcccagaaagttaata**

**Fortunella_japonica_1** **tgactccgtaccaacaaagaatttagtcgcacaccggaaagatagcccagaaagttaata**

**Fortunella_japonica_2** **tgactccgtaccaacaaagaatttagttgcacaccggaaagatagcccagaaagttaata**

**Fortunella_japonica_3** **tgactccgtaccaacaaagaatttagtcgcacaccggaaagatagcccagaaagttaata**

**Fortunella_japonica_4** **tgactccgtaccaacaaagaatttagtcgcacaccggaaagatagcccagaaagttaata**

**Fortunella_japonica_5** **tgactccgtaccaacaaagaatttagtcgcacaccggaaagatagcccagaaagttaata**

**Fortunella_japonica_6** **tgactccgtaccaacaaagaatttagtcgcacaccggaaagatagcccagaaagttaata**

**Fortunella_japonica_7** **tgactccgtaccaacaaagaatttagtcgcacaccggaaagatagcccagaaagttaata**

**Fortunella_japonica_8** **tgactccgtaccaacaaagaatttagtcgcacaccggaaagatagcccagaaagttaata**

**Fortunella_japonica_9** **tgactccgtaccaacaaagaatttagtcgcacaccggaaagatagcccagaaagttaata**

**Fortunella_japonica_10** **tgactccgtaccaacaaagaatttagtcgcacaccggaaagatagcccagaaagttaata**

**Fortunella_japonica_11** **tgactccgtaccaacaaagaatttagtcgcacaccggaaagatagcccagaaagttaata**

**Fortunella_japonica_12** **tgactccgtaccaacaaagaatttagtcgcacaccggaaagatagcccagaaagttaata**

**Fortunella_margarita_1** **tgactccgtaccaacaaagaatttagtcgcacaccggaaagatagcccagaaagttaata**

**Fortunella_margarita_2** **tgactccgtaccaacaaagaatttagtcgcacaccggaaagatagcccagaaagttaata**

**Fortunella_venosa_1** **tgactccgtaccaacaaagaatttagtcgcacaccggaaagatagcccagaaagttaata**

**Fortunella_venosa_2** **tgactccgtaccaacaaagaatttagtcgcacaccggaaagatagcccagaaagttaata**

**Fortunella_venosa_3** **tgactccgtaccaacaaagaatttagtcgcacaccggaaagatagcccagaaagttaata**

2830	2840	2850	2860	2870	2880

....|....|....|....|....|....|....|....|....|....|....|....|

**Atalantia_kwangtungens** **gcgtacttgcctaagtataagtggtttagctgaacccttcctggttgagaagacacgtga**

**Citrus_reticulata_1** **gcgtacttgcctaagtataagtggtttagctgaacccttcctggttgagaagacgcgtga**

**Citrus_reticulata_2** **gcgtacttgcctaagtataagtggtttagctgaacccttcctggttgagaagacgcgtga**

**Citrus_trifoliata_3** **gcgtacttgcctaagtataagtggtttagctgaacccttcctggttgagaagacgcgtga**

**Citrus_aurantiifolia** **gcgtacttgcctaagtataagtggtttagctgaacccttcctggttgagaagacgcgtga**

**Citrus_aurantium** **gcgtacttgcctaagtataagtggtttagctgaacccttcctggttgagaagacgcgtga**

**Citrus_cavaleriei** **gcgtacttgcctaagtataagtggtttagctgaacccttcctggttgagaagacgcgtga**

**Citrus_hongheensis** **gcgtacttgcctaagtataagtggtttagctgaacccttcctggttgagaagacgcgtga**

**Citrus_limon** **gcgtacttgcctaagtataagtggtttagctgaacccttcctggttgagaagacgcgtga**

**Citrus_maxima** **gcgtacttgcctaagtataagtggtttagctgaacccttcctggttgagaagacgcgtga**

**Citrus_medica** **gcgtacttgcctaagtataagtggtttagctgaacccttcctggttgagaagacgcgtga**

**Citrus_polytrifolia** **gcgtacttgcctaagtataagtggtttagctgaacccttcctggttgagaagacgcgtga**

**Citrus_reticulata** **gcgtacttgcctaagtataagtggtttagctgaacccttcctggttgagaagacgcgtga**

**Citrus_sinensis** **gcgtacttgcctaagtataagtggtttagctgaacccttcctggttgagaagacgcgtga**

**Fortunella_bawangica_1** **gcgtacttgcctaagtataagtggtttagctgaacccttcctggttgagaagacgcgtga**

**Fortunella_bawangica_2** **gcgtacttgcctaagtataagtggtttagctgaacccttcctggttgagaagacgcgtga**

**Fortunella_hindsii_1** **gcgtacttgcctaagtataagtggtttagctgaacccttcctggttgagaagacgcgtga**

**Fortunella_hindsii_2** **gcgtacttgcctaagtataagtggtttagctgaacccttcctggttgagaagacgcgtga**

**Fortunella_hindsii_3** **gcgtacttgcctaagtataagtggtttagctgaacccttcctggttgagaagacgcgtga**

**Fortunella_japonica_1** **gcgtacttgcctaagtataagtggtttagctgaacccttcctggttgagaagacgcgtga**

**Fortunella_japonica_2** **gcgtacttgcctaagtataagtggtttagctgaacccttcctggttgagaagacgcgtga**

**Fortunella_japonica_3** **gcgtacttgcctaagtataagtggtttagctgaacccttcctggttgagaagacgcgtga**

**Fortunella_japonica_4** **gcgtacttgcctaagtataagtggtttagctgaacccttcctggttgagaagacgcgtga**

**Fortunella_japonica_5** **gcgtacttgcctaagtataagtggtttagctgaacccttcctggttgagaagacgcgtga**

**Fortunella_japonica_6** **gcgtacttgcctaagtataagtggtttagctgaacccttcctggttgagaagacgcgtga**

**Fortunella_japonica_7** **gcgtacttgcctaagtataagtggtttagctgaacccttcctggttgagaagacgcgtga**

**Fortunella_japonica_8** **gcgtacttgcctaagtataagtggtttagctgaacccttcctggttgagaagacgcgtga**

**Fortunella_japonica_9** **gcgtacttgcctaagtataagtggtttagctgaacccttcctggttgagaagacgcgtga**

**Fortunella_japonica_10** **gcgtacttgcctaagtataagtggtttagctgaacccttcctggttgagaagacgcgtga**

**Fortunella_japonica_11** **gcgtacttgcctaagtataagtggtttagctgaacccttcctggttgagaagacgcgtga**

**Fortunella_japonica_12** **gcgtacttgcctaagtataagtggtttagctgaacccttcctggttgagaagacgcgtga**

**Fortunella_margarita_1** **gcgtacttgcctaagtataagtggtttagctgaacccttcctggttgagaagacgcgtga**

**Fortunella_margarita_2** **gcgtacttgcctaagtataagtggtttagctgaacccttcctggttgagaagacgcgtga**

**Fortunella_venosa_1** **gcgtacttgcctaagtataagtggtttagctgaacccttcctggttgagaagacgcgtga**

**Fortunella_venosa_2** **gcgtacttgcctaagtataagtggtttagctgaacccttcctggttgagaagacgcgtga**

**Fortunella_venosa_3** **gcgtacttgcctaagtataagtggtttagctgaacccttcctggttgagaagacgcgtga**

2890	2900	2910	2920	2930	2940

....|....|....|....|....|....|....|....|....|....|....|....|

**Atalantia_kwangtungens** **aaatgccattgccataaaccgacaaggtaatatttccatttattcatcagaagaggcgta**

**Citrus_reticulata_1** **aaatgccattgccataaaccgacaaggtaatatttccatttattcatcagaagaggcgta**

**Citrus_reticulata_2** **aaatgccattgccataaaccgacaaggtaatatttccatttattcatcagaagaggcgta**

**Citrus_trifoliata_3** **aaatgccattgccataaaccgacaaggtaatatttccatttattcatcagaagaggcgta**

**Citrus_aurantiifolia** **aaatgccattgccataaaccgacaaggtaatatttccatttattcatcagaagaggcgta**

**Citrus_aurantium** **aaatgccattgccataaaccgacaaggtaatatttccatttattcatcagaagaggcgta**

**Citrus_cavaleriei** **aaatgccattgccataaaccgacaaggtaatatttccatttattcatcagaagaggcgta**

**Citrus_hongheensis** **aaatgccattgccataaaccgacaaggtaatatttccatttattcatcagaagaggcgta**

**Citrus_limon** **aaatgccattgccataaaccgacaaggtaatatttccatttattcatcagaagaggcgta**

**Citrus_maxima** **aaatgccattgccataaaccgacaaggtaatatttccatttattcatcagaagaggcgta**

**Citrus_medica** **aaatgccattgccataaaccgacaaggtaatatttccatttattcatcagaagaggcgta**

**Citrus_polytrifolia** **aaatgccattgccataaaccgacaaggtaatatttccatttattcatcagaagaggcgta**

**Citrus_reticulata** **aaatgccattgccataaaccgacaaggtaatatttccatttattcatcagaagaggcgta**

**Citrus_sinensis** **aaatgccattgccataaaccgacaaggtaatatttccatttattcatcagaagaggcgta**

**Fortunella_bawangica_1** **aaatgccattgccataaaccgacaaggtaatatttccatttattcatcagaagaggcgta**

**Fortunella_bawangica_2** **aaatgccattgccataaaccgacaaggtaatatttccatttattcatcagaagaggcgta**

**Fortunella_hindsii_1** **aaatgccattgccataaaccgacaaggtaatatttccatttattcatcagaagaggcgta**

**Fortunella_hindsii_2** **aaatgccattgccataaaccgacaaggtaatatttccatttattcatcagaagaggcgta**

**Fortunella_hindsii_3** **aaatgccattgccataaaccgacaaggtaatatttccatttattcatcagaagaggcgta**

**Fortunella_japonica_1** **aaatgccattgccataaaccgacaaggtaatatttccatttattcatcagaagaggcgta**

**Fortunella_japonica_2** **aaatgccattgccataaaccgacaaggtaatatttccatttattcatcagaagaggcgta**

**Fortunella_japonica_3** **aaatgccattgccataaaccgacaaggtaatatttccatttattcatcagaagaggcgta**

**Fortunella_japonica_4** **aaatgccattgccataaaccgacaaggtaatatttccatttattcatcagaagaggcgta**

**Fortunella_japonica_5** **aaatgccattgccataaaccgacaaggtaatatttccatttattcatcagaagaggcgta**

**Fortunella_japonica_6** **aaatgccattgccataaaccgacaaggtaatatttccatttattcatcagaagaggcgta**

**Fortunella_japonica_7** **aaatgccattgccataaaccgacaaggtaatatttccatttattcatcagaagaggcgta**

**Fortunella_japonica_8** **aaatgccattgccataaaccgacaaggtaatatttccatttattcatcagaagaggcgta**

**Fortunella_japonica_9** **aaatgccattgccataaaccgacaaggtaatatttccatttattcatcagaagaggcgta**

**Fortunella_japonica_10** **aaatgccattgccataaaccgacaaggtaatatttccatttattcatcagaagaggcgta**

**Fortunella_japonica_11** **aaatgccattgccataaaccgacaaggtaatatttccatttattcatcagaagaggcgta**

**Fortunella_japonica_12** **aaatgccattgccataaaccgacaaggtaatatttccatttattcatcagaagaggcgta**

**Fortunella_margarita_1** **aaatgccattgccataaaccgacaaggtaatatttccatttattcatcagaagaggcgta**

**Fortunella_margarita_2** **aaatgccattgccataaaccgacaaggtaatatttccatttattcatcagaagaggcgta**

**Fortunella_venosa_1** **aaatgccattgccataaaccgacaaggtaatatttccatttattcatcagaagaggcgta**

**Fortunella_venosa_2** **aaatgccattgccataaaccgacaaggtaatatttccatttattcatcagaagaggcgta**

**Fortunella_venosa_3** **aaatgccattgccataaaccgacaaggtaatatttccatttattcatcagaagaggcgta**

2950	2960	2970	2980	2990	3000

....|....|....|....|....|....|....|....|....|....|....|....|

**Atalantia_kwangtungens** **tcctttgaagccaaaatggcttttccttgatatctaacataatgcatgaaaggatccttg**

**Citrus_reticulata_1** **tcctttgaagccaaaatggattttccttgatatctaacataatgcatgaaaggatccttg**

**Citrus_reticulata_2** **tcctttgaagccaaaatggattttccttgatatctaacataatgcatgaaaggatccttg**

**Citrus_trifoliata_3** **tcctttgaagccaaaatggattttccttgatatctaacataatgcatgaaaggatccttg**

**Citrus_aurantiifolia** **tcctttgaagccaaaatggattttccttgatatctaacataatgcatgaaaggatccttg**

**Citrus_aurantium** **tcctttgaagccaaaatggattttccttgatatctaacataatgcatgaaaggatccttg**

**Citrus_cavaleriei** **tcctttgaagccaaaatggattttccttgatatctaacataatgcatgaaaggatccttg**

**Citrus_hongheensis** **tcctttgaagccaaaatggattttccttgatatctaacataatgcatgaaaggatccttg**

**Citrus_limon** **tcctttgaagccaaaatggattttccttgatatctaacataatgcatgaaaggatccttg**

**Citrus_maxima** **tcctttgaagccaaaatggattttccttgatatctaacataatgcatgaaaggatccttg**

**Citrus_medica** **tcctttgaagccaaaatggattttccttgatatctaacataatgcatgaaaggatccttg**

**Citrus_polytrifolia** **tcctttgaagccaaaatggattttccttgatatctaacataatgcatgaaaggatccttg**

**Citrus_reticulata** **tcctttgaagccaaaatggattttccttgatatctaacataatgcatgaaaggatccttg**

**Citrus_sinensis** **tcctttgaagccaaaatggattttccttgatatctaacataatgcatgaaaggatccttg**

**Fortunella_bawangica_1** **tcctttgaagccaaaatggattttccttgatatctaacataatgcatgaaaggatccttg**

**Fortunella_bawangica_2** **tcctttgaagccaaaatggattttccttgatatctaacataatgcatgaaaggatccttg**

**Fortunella_hindsii_1** **tcctttgaagccaaaatggattttccttgatatctaacataatgcatgaaaggatccttg**

**Fortunella_hindsii_2** **tcctttgaagccaaaatggattttccttgatatctaacataatgcatgaaaggatccttg**

**Fortunella_hindsii_3** **tcctttgaagccaaaatggattttccttgatatctaacataatgcatgaaaggatccttg**

**Fortunella_japonica_1** **tcctttgaagccaaaatggattttccttgatatctaacataatgcatgaaaggatccttg**

**Fortunella_japonica_2** **tcctttgaagccaaaatggattttccttgatatctaacataatgcatgaaaggatccttg**

**Fortunella_japonica_3** **tcctttgaagccaaaatggattttccttgatatctaacataatgcatgaaaggatccttg**

**Fortunella_japonica_4** **tcctttgaagccaaaatggattttccttgatatctaacataatgcatgaaaggatccttg**

**Fortunella_japonica_5** **tcctttgaagccaaaatggattttccttgatatctaacataatgcatgaaaggatccttg**

**Fortunella_japonica_6** **tcctttgaagccaaaatggattttccttgatatctaacataatgcatgaaaggatccttg**

**Fortunella_japonica_7** **tcctttgaagccaaaatggattttccttgatatctaacataatgcatgaaaggatccttg**

**Fortunella_japonica_8** **tcctttgaagccaaaatggattttccttgatatctaacataatgcatgaaaggatccttg**

**Fortunella_japonica_9** **tcctttgaagccaaaatggattttccttgatatctaacataatgcatgaaaggatccttg**

**Fortunella_japonica_10** **tcctttgaagccaaaatggattttccttgatatctaacataatgcatgaaaggatccttg**

**Fortunella_japonica_11** **tcctttgaagccaaaatggattttccttgatatctaacataatgcatgaaaggatccttg**

**Fortunella_japonica_12** **tcctttgaagccaaaatggattttccttgatatctaacataatgcatgaaaggatccttg**

**Fortunella_margarita_1** **tcctttgaagccaaaatggattttccttgatatctaacataatgcatgaaaggatccttg**

**Fortunella_margarita_2** **tcctttgaagccaaaatggattttccttgatatctaacataatgcatgaaaggatccttg**

**Fortunella_venosa_1** **tcctttgaagccaaaatggattttccttgatatctaacataatgcatgaaaggatccttg**

**Fortunella_venosa_2** **tcctttgaagccaaaatggattttccttgatatctaacataatgcatgaaaggatccttg**

**Fortunella_venosa_3** **tcctttgaagccaaaatggattttccttgatatctaacataatgcatgaaaggatccttg**

3010	3020	3030	3040	3050	3060

....|....|....|....|....|....|....|....|....|....|....|....|

**Atalantia_kwangtungens** **aacaaccctaagatgtcctgaaaatctttagcaaagacttcgacaagatgttcgactttt**

**Citrus_reticulata_1** **accaaccctaagatgtcctgaaaatctttagcaaagacttcgacaagatgttcgactttt**

**Citrus_reticulata_2** **accaaccctaagatgtcctgaaaatctttagcaaagacttcgacaagatgttcgactttt**

**Citrus_trifoliata_3** **accaaccctaagatgtcctgaaaatctttcgcaaagacttcgacaagatgttcgactttt**

**Citrus_aurantiifolia** **accaaccctaagatgtcctgaaaatctttagcaaagacttcgacaagatgttcgactttt**

**Citrus_aurantium** **accaaccctaagatgtcctgaaaatctttagcaaagacttcgacaagatgttcgactttt**

**Citrus_cavaleriei** **accaaccctaagatgtcctgaaaatctttagcaaagacttcgacaagatgttcgactttt**

**Citrus_hongheensis** **accaaccctaagatgtcctgaaaatctttagcaaagacttcgacaagatgttcgactttt**

**Citrus_limon** **accaaccctaagatgtcctgaaaatctttagcaaagacttcgacaagatgttcgactttt**

**Citrus_maxima** **accaaccctaagatgtcctgaaaatctttagcaaagacttcgacaagatgttcgactttt**

**Citrus_medica** **accaaccctaagatgtcctgaaaatctgtagcaaagacttcgacaagatgttcgactttt**

**Citrus_polytrifolia** **accaaccctaagatgtcctgaaaatctttcgcaaagacttcgacaagatgttcgactttt**

**Citrus_reticulata** **accaaccctaagatgtcctgaaaatctttagcaaagacttcgacaagatgttcgactttt**

**Citrus_sinensis** **accaaccctaagatgtcctgaaaatctttagcaaagacttcgacaagatgttcgactttt**

**Fortunella_bawangica_1** **accaaccctaagatgtcctgaaaatctttagcaaagacttcgacaagatgttcgactttt**

**Fortunella_bawangica_2** **accaaccctaagatgtcctgaaaatctttagcaaagacttcgacaagatgttcgactttt**

**Fortunella_hindsii_1** **accaaccctaagatgtcctgaaaatctttagcaaagacttcgacaagatgttcgactttt**

**Fortunella_hindsii_2** **accaaccctaagatgtcctgaaaatctttagcaaagacttcgacaagatgttcgactttt**

**Fortunella_hindsii_3** **accaaccctaagatgtcctgaaaatctttagcaaagacttcgacaagatgttcgactttt**

**Fortunella_japonica_1** **accaaccctaagatgtcctgaaaatctttagcaaagacttcgacaagatgttcgactttt**

**Fortunella_japonica_2** **accaaccctaagatgtcctgaaaatctttagcaaagacttcgacaagatgttcgactttt**

**Fortunella_japonica_3** **accaaccctaagatgtcctgaaaatctttagcaaagacttcgacaagatgttcgactttt**

**Fortunella_japonica_4** **accaaccctaagatgtcctgaaaatctttagcaaagacttcgacaagatgttcgactttt**

**Fortunella_japonica_5** **accaaccctaagatgtcctgaaaatctttagcaaagacttcgacaagatgttcgactttt**

**Fortunella_japonica_6** **accaaccctaagatgtcctgaaaatctttagcaaagacttcgacaagatgttcgactttt**

**Fortunella_japonica_7** **accaaccctaagatgtcctgaaaatctttagcaaagacttcgacaagatgttcgactttt**

**Fortunella_japonica_8** **accaaccctaagatgtcctgaaaatctttagcaaagacttcgacaagatgttcgactttt**

**Fortunella_japonica_9** **accaaccctaagatgtcctgaaaatctttagcaaagacttcgacaagatgttcgactttt**

**Fortunella_japonica_10** **accaaccctaagatgtcctgaaaatctttagcaaagacttcgacaagatgttcgactttt**

**Fortunella_japonica_11** **accaaccctaagatgtcctgaaaatctttagcaaagacttcgacaagatgttcgactttt**

**Fortunella_japonica_12** **accaaccctaagatgtcctgaaaatctttagcaaagacttcgacaagatgttcgactttt**

**Fortunella_margarita_1** **accaaccctaagatgtcctgaaaatctttagcaaagacttcgacaagatgttcgactttt**

**Fortunella_margarita_2** **accaaccctaagatgtcctgaaaatctttagcaaagacttcgacaagatgttcgactttt**

**Fortunella_venosa_1** **accaaccctaagatgtcctgaaaatctttagcaaagacttcgacaagatgttcgactttt**

**Fortunella_venosa_2** **accaaccctaagatgtcctgaaaatctttagcaaagacttcgacaagatgttcgactttt**

**Fortunella_venosa_3** **accaaccctaagatgtcctgaaaatctttagcaaagacttcgacaagatgttcgactttt**

3070	3080	3090	3100	3110	3120

....|....|....|....|....|....|....|....|....|....|....|....|

**Atalantia_kwangtungens** **ccatagaaatacgttcgctcaacgaggactcgagaggatgttgattgtaaatgagacgat**

**Citrus_reticulata_1** **ccatagaaatacgttcgctcaacgaggactcgaaaggatgttgattgtaaatgagacgat**

**Citrus_reticulata_2** **ccatagaaatacgttcgctcaacgaggactcgaaaggatgttgattgtaaatgagacgat**

**Citrus_trifoliata_3** **ccatagaaatacgttcgctcaacgaggactcgaaaggatgttgattgtaaatgagacgat**

**Citrus_aurantiifolia** **ccatagaaatacgttcgctcaacaaggacttgaaaggatgttgattgtaaatgagacgat**

**Citrus_aurantium** **ccatagaaatacgttcgctcaacgaggacttgaaaggatgttgattgtaaatgagacgat**

**Citrus_cavaleriei** **ccatagaaatacgttcgctcaacgaggactcgaaaggatgttgattgtaaatgagacgat**

**Citrus_hongheensis** **ccatagaaatacgttcgctcaacgaggacttgaaaggatgttgattgtaaatgagacgat**

**Citrus_limon** **ccatagaaatacgttcgctcaacgaggacttgaaaggatgttgattgtaaatgagacgat**

**Citrus_maxima** **ccatagaaatacgttcgctcaacgaggacttgaaaggatgttgattgtaaatgagacgat**

**Citrus_medica** **ccatagaaatacgttcgctcaacgaggactcgaaaggatgttgattgtaaatgcgacgat**

**Citrus_polytrifolia** **ccatagaaatacgttcgctcaacgaggactcgaaaggatgttgattgtaaatgagacgat**

**Citrus_reticulata** **ccatagaaatacgttcgctcaacgaggactcgaaaggatgttgattgtaaatgagacgat**

**Citrus_sinensis** **ccatagaaatacgttcgctcaacgaggacttgaaaggatgttgattgtaaatgagacgat**

**Fortunella_bawangica_1** **ccatagaaatacgttcgctcaacgaggactcgaaaggatgttgattgtaaatgagacgat**

**Fortunella_bawangica_2** **ccatagaaatacgttcgctcaacgaggactcgaaaggatgttgattgtaaatgagacgat**

**Fortunella_hindsii_1** **ccatagaaatacgttcgctcaacgaggactcgaaaggatgttgattgtaaatgagacgat**

**Fortunella_hindsii_2** **ccatagaaatacgttcgctcaacgaggactcgaaaggatgttgattgtaaatgagacgat**

**Fortunella_hindsii_3** **ccatagaaatacgttcgctcaacgaggactcgaaaggatgttgattgtaaatgagacgat**

**Fortunella_japonica_1** **ccatagaaatacgttcgctcaacgaggactcgaaaggatgttgattgtaaatgagacgat**

**Fortunella_japonica_2** **ccatagaaatacgttcgctcaacgaggactcgaaaggatgttgattgtaaatgagacgat**

**Fortunella_japonica_3** **ccatagaaatacgttcgctcaacgaggactcgaaaggatgttgattgtaaatgagacgat**

**Fortunella_japonica_4** **ccatagaaatacgttcgctcaacgaggactcgaaaggatgttgattgtaaatgagacgat**

**Fortunella_japonica_5** **ccatagaaatacgttcgctcaacgaggactcgaaaggatgttgattgtaaatgagacgat**

**Fortunella_japonica_6** **ccatagaaatacgttcgctcaacgaggactcgaaaggatgttgattgtaaatgagacgat**

**Fortunella_japonica_7** **ccatagaaatacgttcgctcaacgaggactcgaaaggatgttgattgtaaatgagacgat**

**Fortunella_japonica_8** **ccatagaaatacgttcgctcaacgaggactcgaaaggatgttgattgtaaatgagacgat**

**Fortunella_japonica_9** **ccatagaaatacgttcgctcaacgaggactcgaaaggatgttgattgtaaatgagacgat**

**Fortunella_japonica_10** **ccatagaaatacgttcgctcaacgaggactcgaaaggatgttgattgtaaatgagacgat**

**Fortunella_japonica_11** **ccatagaaatacgttcgctcaacgaggactcgaaaggatgttgattgtaaatgagacgat**

**Fortunella_japonica_12** **ccatagaaatacgttcgctcaacgaggactcgaaaggatgttgattgtaaatgagacgat**

**Fortunella_margarita_1** **ccatagaaatacgttcgctcaacgaggactcgaaaggatgttgattgtaaatgagacgat**

**Fortunella_margarita_2** **ccatagaaatacgttcgctcaacgaggactcgaaaggatgttgattgtaaatgagacgat**

**Fortunella_venosa_1** **ccatagaaatacgttcgctcaacgaggactcgaaaggatgttgattgtaaatgagacgat**

**Fortunella_venosa_2** **ccatagaaatacgttcgctcaacgaggactcgaaaggatgttgattgtaaatgagacgat**

**Fortunella_venosa_3** **ccatagaaatacgttcgctcaacgaggactcgaaaggatgttgattgtaaatgagacgat**

3130	3140	3150	3160	3170	3180

....|....|....|....|....|....|....|....|....|....|....|....|

**Atalantia_kwangtungens** **tggttacagagaaaaaagaggatggattcatattcatatacatgagaattatatagaaac**

**Citrus_reticulata_1** **tggttacagagaaaaaagaggatggattcatattcatatacatgagaattatatagaaac**

**Citrus_reticulata_2** **tggttacagagaaaaaagaggatggattcatattcatatacatgagaattatatagaaac**

**Citrus_trifoliata_3** **tggttacagagaaaaaagaggatggattcatattcatatacatgagaattatatagaaac**

**Citrus_aurantiifolia** **tggttacagagaaaaaagaggatggattcatattcatatacatgagaattatatagaaac**

**Citrus_aurantium** **tggttacagagaaaaaagaggatggattcatattcatatacatgagaattatatagaaac**

**Citrus_cavaleriei** **tggttacagagaaaaaagaggatggattcatattcatatacatgagaattatatagaaac**

**Citrus_hongheensis** **tggttacagagaaaaaagaggatggattcatattcatatacatgagaattatatagaaac**

**Citrus_limon** **tggttacagagaaaaaagaggatggattcatattcatatacatgagaattatatagaaac**

**Citrus_maxima** **tggttacagagaaaaaagaggatggattcatattcatatacatgagaattatatagaaac**

**Citrus_medica** **tggttacataaaaacaagaggatggattcatattcatatacatgagaattatatagaaac**

**Citrus_polytrifolia** **tggttacagagaaaaaagaggatggattcatattcatatacatgagaattatatagaaac**

**Citrus_reticulata** **tggttacagagaaaaaagaggatggattcatattcatatacatgagaattatatagaaac**

**Citrus_sinensis** **tggttacagagaaaaaagaggatggattcatattcatatacatgagaattatatagaaac**

**Fortunella_bawangica_1** **tggttacagagaaaaaagaggatggattcatattcatatacatgagaattatatagaaac**

**Fortunella_bawangica_2** **tggttacagagaaaaaagaggatggattcatattcatatacatgagaattatatagaaac**

**Fortunella_hindsii_1** **tggttacagagaaaaaagaggatggattcatattcatatacatgagaattatatagaaac**

**Fortunella_hindsii_2** **tggttacagagaaaaaagaggatggattcatattcatatacatgagaattatatagaaac**

**Fortunella_hindsii_3** **tggttacagagaaaaaagaggatggattcatattcatatacatgagaattatatagaaac**

**Fortunella_japonica_1** **tggttacagagaaaaaagaggatggattcatattcatatacatgagaattatatagaaac**

**Fortunella_japonica_2** **tggttacagagaaaaaagaggatggattcatattcatatacatgagaattatatagaaac**

**Fortunella_japonica_3** **tggttacagagaaaaaagaggatggattcatattcatatacatgagaattatatagaaac**

**Fortunella_japonica_4** **tggttacagagaaaaaagaggatggattcatattcatatacatgagaattatatagaaac**

**Fortunella_japonica_5** **tggttacagagaaaaaagaggatggattcatattcatatacatgagaattatatagaaac**

**Fortunella_japonica_6** **tggttacagagaaaaaagaggatggattcatattcatatacatgagaattatatagaaac**

**Fortunella_japonica_7** **tggttacagagaaaaaagaggatggattcatattcatatacatgagaattatatagaaac**

**Fortunella_japonica_8** **tggttacagagaaaaaagaggatggattcatattcatatacatgagaattatatagaaac**

**Fortunella_japonica_9** **tggttacagagaaaaaagaggatggattcatattcatatacatgagaattatatagaaac**

**Fortunella_japonica_10** **tggttacagagaaaaaagaggatggattcatattcatatacatgagaattatatagaaac**

**Fortunella_japonica_11** **tggttacagagaaaaaagaggatggattcatattcatatacatgagaattatatagaaac**

**Fortunella_japonica_12** **tggttacagagaaaaaagaggatggattcatattcatatacatgagaattatatagaaac**

**Fortunella_margarita_1** **tggttacagagaaaaaagaggatggattcatattcatatacatgagaattatatagaaac**

**Fortunella_margarita_2** **tggttacagagaaaaaagaggatggattcatattcatatacatgagaattatatagaaac**

**Fortunella_venosa_1** **tggttacagagaaaaaagaggatggattcatattcatatacatgagaattatatagaaac**

**Fortunella_venosa_2** **tggttacagagaaaaaagaggatggattcatattcatatacatgagaattatatagaaac**

**Fortunella_venosa_3** **tggttacagagaaaaaagaggatggattcatattcatatacatgagaattatatagaaac**

3190	3200	3210	3220	3230	3240

....|....|....|....|....|....|....|....|....|....|....|....|

**Atalantia_kwangtungens** **aataacaatcttggattacttttaaaaaaaacagaaatagagttctttggagtaataaga**

**Citrus_reticulata_1** **aataacaatcttggattactttttaaaaaaacagaaatagagttctttggagtaataaga**

**Citrus_reticulata_2** **aataacaatcttggattactttttaaaaaaacagaaatagagttctttggagtaataaga**

**Citrus_trifoliata_3** **aataacaatcttggattactttttaaaaaaacagaaatagagttctttggagtaataaga**

**Citrus_aurantiifolia** **aataacaatcttggattactttttaaaaaaacagaaatagagttctttggagtaataaga**

**Citrus_aurantium** **aataacaatcttggattacttttaaaaaaaaaagaaatagagttctttggagtaataaga**

**Citrus_cavaleriei** **aataacaatcttggattactttttaaaaaaacagaaatagagttctttggagtaataaga**

**Citrus_hongheensis** **aataacaatcttggattactttttaaaaaaacagaaatagagttctttggagtaataaga**

**Citrus_limon** **aataacaatcttggattactttttaaaaaaaaagaaatagagttctttggagtaataaga**

**Citrus_maxima** **aataacaatcttggattactttttaaaaaaacagaaatagagttctttggagtaataaga**

**Citrus_medica** **aataacaatcttggattactttttaaaaaaccagaaatagagttctttggagtaataaga**

**Citrus_polytrifolia** **aataacaatcttggattactttttaaaaaaacagaaatagagttctttggagtaataaga**

**Citrus_reticulata** **aataacaatcttggattactttttaaaaaaacagaaatagagttctttggagtaataaga**

**Citrus_sinensis** **aataacaatcttggattactttttaaaaaaacagaaatagagttctttggagtaataaga**

**Fortunella_bawangica_1** **aataacaatcttggattactttttaaaaaaacagaaatagagttctttggagtaataaga**

**Fortunella_bawangica_2** **aataacaatcttggattactttttaaaaaaacagaaatagagttctttggagtaataaga**

**Fortunella_hindsii_1** **aataacaatcttggattactttttaaaaaaacagaaatagagttctttggagtaataaga**

**Fortunella_hindsii_2** **aataacaatcttggattactttttaaaaaaacagaaatagagttctttggagtaataaga**

**Fortunella_hindsii_3** **aataacaatcttggattactttttaaaaaaacagaaatagagttctttggagtaataaga**

**Fortunella_japonica_1** **aataacaatcttggattactttttaaaaaaacagaaatagagttctttggagtaataaga**

**Fortunella_japonica_2** **aataacaatcttggattactttttaaaaaaacagaaatagagttctttggagtaataaga**

**Fortunella_japonica_3** **aataacaatcttggattactttttaaaaaaacagaaatagagttctttggagtaataaga**

**Fortunella_japonica_4** **aataacaatcttggattactttttaaaaaaacagaaatagagttctttggagtaataaga**

**Fortunella_japonica_5** **aataacaatcttggattactttttaaaaaaacagaaatagagttctttggagtaataaga**

**Fortunella_japonica_6** **aataacaatcttggattactttttaaaaaaacagaaatagagttctttggagtaataaga**

**Fortunella_japonica_7** **aataacaatcttggattactttttaaaaaaacagaaatagagttctttggagtaataaga**

**Fortunella_japonica_8** **aataacaatcttggattactttttaaaaaaacagaaatagagttctttggagtaataaga**

**Fortunella_japonica_9** **aataacaatcttggattactttttaaaaaaacagaaatagagttctttggagtaataaga**

**Fortunella_japonica_10** **aataacaatcttggattactttttaaaaaaacagaaatagagttctttggagtaataaga**

**Fortunella_japonica_11** **aataacaatcttggattactttttaaaaaaacagaaatagagttctttggagtaataaga**

**Fortunella_japonica_12** **aataacaatcttggattactttttaaaaaaacagaaatagagttctttggagtaataaga**

**Fortunella_margarita_1** **aataacaatcttggattactttttaaaaaaacagaaatagagttctttggagtaataaga**

**Fortunella_margarita_2** **aataacaatcttggattactttttaaaaaaacagaaatagagttctttggagtaataaga**

**Fortunella_venosa_1** **aataacaatcttggattactttttaaaaaaacagaaatagagttctttggagtaataaga**

**Fortunella_venosa_2** **aataacaatcttggattactttttaaaaaaacagaaatagagttctttggagtaataaga**

**Fortunella_venosa_3** **aataacaatcttggattactttttaaaaaaacagaaatagagttctttggagtaataaga**

3250	3260	3270	3280	3290	3300

....|....|....|....|....|....|....|....|....|....|....|....|

**Atalantia_kwangtungens** **ctgttcgaattaaaatactcgtggagaaagaaccgtaataaatgtaaagaagaggcatcc**

**Citrus_reticulata_1** **ctgttcgaattaaaatactcgtggagaaagaaccgtaataaatgtaaagaagaggcatcc**

**Citrus_reticulata_2** **ctgttcgaattaaaatactcgtggagaaagaaccgtaataaatgtaaagaagaggcatcc**

**Citrus_trifoliata_3** **ctgttcgaattaaaatactcgtggagaaagaaacgtaataaatgtaaagaagaggcatcc**

**Citrus_aurantiifolia** **ctgttcgaattaaaatactcgtggagaaagaaccgtaataaatgtaaagaagaggcatcc**

**Citrus_aurantium** **ctgttcgaattaaaatactcgtggagaaagaaccgtaataaatgtaaagaagaggcatcc**

**Citrus_cavaleriei** **ctgttcgaattaaaatactcgtggagaaagaaccgtaataaatgtaaagaagaggcatcc**

**Citrus_hongheensis** **ctgttcgaattaaaatactcgtggagaaagaaccgtaataaatgtaaagaagaggcatcc**

**Citrus_limon** **ctgttcgaattaaaatactcgtggagaaagaaccgtaataaatgtaaagaagaggcatcc**

**Citrus_maxima** **ctgttcgaattaaaatactcgtggagaaagaaccgtaataaatgtaaagaagaggcatcc**

**Citrus_medica** **ctgttcgaattaaaatactcgtggagaaagaaccgtaataaatgtaaagaagaggcatcc**

**Citrus_polytrifolia** **ctgttcgaattaaaatactcgtggagaaagaaccgtaataaatgtaaagaagaggcatcc**

**Citrus_reticulata** **ctgttcgaattaaaatactcgtggagaaagaaccgtaataaatgtaaagaagaggcatcc**

**Citrus_sinensis** **ctgttcgaattaaaatactcgtggagaaagaaccgtaataaatgtaaagaagaggcatcc**

**Fortunella_bawangica_1** **ctgttcgaattaaaatactcgtggagaaagaaccgtaataaatgtaaagaagaggcatcc**

**Fortunella_bawangica_2** **ctgttcgaattaaaatactcgtggagaaagaaccgtaataaatgtaaagaagaggcatcc**

**Fortunella_hindsii_1** **ctgttcgaattaaaatactcgtggagaaagaaccgtaataaatgtaaagaagaggcatcc**

**Fortunella_hindsii_2** **ctgttcgaattaaaatactcgtggagaaagaaccgtaataaatgtaaagaagaggcatcc**

**Fortunella_hindsii_3** **ctgttcgaattaaaatactcgtggagaaagaaccgtaataaatgtaaagaagaggcatcc**

**Fortunella_japonica_1** **ctgttcgaattaaaatactcgtggagaaagaaccgtaataaatgtaaagaagaggcatcc**

**Fortunella_japonica_2** **ctgttcgaattaaaatactcgtggagaaagaaccgtaataaatgtaaagaagaggcatcc**

**Fortunella_japonica_3** **ctgttcgaattaaaatactcgtggagaaagaaccgtaataaatgtaaagaagaggcatcc**

**Fortunella_japonica_4** **ctgttcgaattaaaatactcgtggagaaagaaccgtaataaatgtaaagaagaggcatcc**

**Fortunella_japonica_5** **ctgttcgaattaaaatactcgtggagaaagaaccgtaataaatgtaaagaagaggcatcc**

**Fortunella_japonica_6** **ctgttcgaattaaaatactcgtggagaaagaaccgtaataaatgtaaagaagaggcatcc**

**Fortunella_japonica_7** **ctgttcgaattaaaatactcgtggagaaagaaccgtaataaatgtaaagaagaggcatcc**

**Fortunella_japonica_8** **ctgttcgaattaaaatactcgtggagaaagaaccgtaataaatgtaaagaagaggcatcc**

**Fortunella_japonica_9** **ctgttcgaattaaaatactcgtggagaaagaaccgtaataaatgtaaagaagaggcatcc**

**Fortunella_japonica_10** **ctgttcgaattaaaatactcgtggagaaagaaccgtaataaatgtaaagaagaggcatcc**

**Fortunella_japonica_11** **ctgttcgaattaaaatactcgtggagaaagaaccgtaataaatgtaaagaagaggcatcc**

**Fortunella_japonica_12** **ctgttcgaattaaaatactcgtggagaaagaaccgtaataaatgtaaagaagaggcatcc**

**Fortunella_margarita_1** **ctgttcgaattaaaatactcgtggagaaagaaccgtaataaatgtaaagaagaggcatcc**

**Fortunella_margarita_2** **ctgttcgaattaaaatactcgtggagaaagaaccgtaataaatgtaaagaagaggcatcc**

**Fortunella_venosa_1** **ctgttcgaattaaaatactcgtggagaaagaaccgtaataaatgtaaagaagaggcatcc**

**Fortunella_venosa_2** **ctgttcgaattaaaatactcgtggagaaagaaccgtaataaatgtaaagaagaggcatcc**

**Fortunella_venosa_3** **ctgttcgaattaaaatactcgtggagaaagaaccgtaataaatgtaaagaagaggcatcc**

3310	3320	3330	3340	3350	3360

....|....|....|....|....|....|....|....|....|....|....|....|

**Atalantia_kwangtungens** **tttacccattcgcgaaaggtttgaaccaagatttcgggacaaatggggtggggtattcgt**

**Citrus_reticulata_1** **tttacccattcgcgaagggtttgaaccaagatttcgggacaaatggggtagggtattcgt**

**Citrus_reticulata_2** **tttacccattcgcgaagggtttgaaccaagatttcgggacaaatggggtagggtattcgt**

**Citrus_trifoliata_3** **tttacccattcgcgaagggtttgaaccaagatttcgggacaaatggggtagggtattcgt**

**Citrus_aurantiifolia** **tttacccattcgcgaagggtttgaaccaagatttcgggacaaatggggtagggtattcgt**

**Citrus_aurantium** **tttacccattcgcgaagggtttgaaccaagatttcgggacaaatggggtagggtattcgt**

**Citrus_cavaleriei** **tttacccattcgcgaagggtttgaaccaagatttcgggacaaatggggtagggtattcgt**

**Citrus_hongheensis** **tttacccattcgcgaagggtttgaaccaagatttcgggacaaatggggtagggtattcgt**

**Citrus_limon** **tttacccattcgcgaagggtttgaaccaagatttcgggacaaatggggtagggtattcgt**

**Citrus_maxima** **tttacccattcgcgaagggtttgaaccaagatttcgggacaaatggggtagggtattcgt**

**Citrus_medica** **tttacccattcgcgaagggtttgaaccaagatttcgggacaaatggggtagggtattcgt**

**Citrus_polytrifolia** **tttacccattcgcgaagggtttgaaccaagatttcgggacaaatggggtagggtattcgt**

**Citrus_reticulata** **tttacccattcgcgaagggtttgaaccaagatttcgggacaaatggggtagggtattcgt**

**Citrus_sinensis** **tttacccattcgcgaagggtttgaaccaagatttcgggacaaatggggtagggtattcgt**

**Fortunella_bawangica_1** **tttacccattcgcgaagggtttgaaccaagatttcgggacaaatggggtagggtattcgt**

**Fortunella_bawangica_2** **tttacccattcgcgaagggtttgaaccaagatttcgggacaaatggggtagggtattcgt**

**Fortunella_hindsii_1** **tttacccattcgcgaagggtttgaaccaagatttcgggacaaatggggtagggtattcgt**

**Fortunella_hindsii_2** **tttacccattcgcgaagggtttgaaccaagatttcgggacaaatggggtagggtattcgt**

**Fortunella_hindsii_3** **tttacccattcgcgaagggtttgaaccaagatttcgggacaaatggggtagggtattcgt**

**Fortunella_japonica_1** **tttacccattcgcgaagggtttgaaccaagatttcgggacaaatggggtagggtattcgt**

**Fortunella_japonica_2** **tttacccattcgcgaagggtttgaaccaagatttcgggacaaatggggtagggtattcgt**

**Fortunella_japonica_3** **tttacccattcgcgaagggtttgaaccaagatttcgggacaaatggggtagggtattcgt**

**Fortunella_japonica_4** **tttacccattcgcgaagggtttgaaccaagatttcgggacaaatggggtagggtattcgt**

**Fortunella_japonica_5** **tttacccattcgcgaagggtttgaaccaagatttcgggacaaatggggtagggtattcgt**

**Fortunella_japonica_6** **tttacccattcgcgaagggtttgaaccaagatttcgggacaaatggggtagggtattcgt**

**Fortunella_japonica_7** **tttacccattcgcgaagggtttgaaccaagatttcgggacaaatggggtagggtattcgt**

**Fortunella_japonica_8** **tttacccattcgcgaagggtttgaaccaagatttcgggacaaatggggtagggtattcgt**

**Fortunella_japonica_9** **tttacccattcgcgaagggtttgaaccaagatttcgggacaaatggggtagggtattcgt**

**Fortunella_japonica_10** **tttacccattcgcgaagggtttgaaccaagatttcgggacaaatggggtagggtattcgt**

**Fortunella_japonica_11** **tttacccattcgcgaagggtttgaaccaagatttcgggacaaatggggtagggtattcgt**

**Fortunella_japonica_12** **tttacccattcgcgaagggtttgaaccaagatttcgggacaaatggggtagggtattcgt**

**Fortunella_margarita_1** **tttacccattcgcgaagggtttgaaccaagatttcgggacaaatggggtagggtattcgt**

**Fortunella_margarita_2** **tttacccattcgcgaagggtttgaaccaagatttcgggacaaatggggtagggtattcgt**

**Fortunella_venosa_1** **tttacccattcgcgaagggtttgaaccaagatttcgggacaaatggggtagggtattcgt**

**Fortunella_venosa_2** **tttacccattcgcgaagggtttgaaccaagatttcgggacaaatggggtagggtattcgt**

**Fortunella_venosa_3** **tttacccattcgcgaagggtttgaaccaagatttcgggacaaatggggtagggtattcgt**

3370	3380	3390	3400	3410	3420

....|....|....|....|....|....|....|....|....|....|....|....|

**Atalantia_kwangtungens** **acatctaacacataatttaaatgggacaatttatcctcgaaaaaaggaaatattgaatga**

**Citrus_reticulata_1** **acatctaacacataatttaaatgggacaatttatcctcgaaaaaaggaaatattgaatga**

**Citrus_reticulata_2** **acatctaacacataatttaaatgggacaatttatcctcgaaaaaaggaaatattgaatga**

**Citrus_trifoliata_3** **acatctaacacataatttaaatgggacaatttatcctcgaaaaaaggaaatattgaatga**

**Citrus_aurantiifolia** **acatctaacacataatttaaatgggacaatttatcctcgaaaaaaggaaatattgaatga**

**Citrus_aurantium** **acatctaacacataatttaaatgggacaatttatcctcgaaaaaaggaaatattgaatga**

**Citrus_cavaleriei** **acatctaacacataatttaaatgggacaatttatcctcgaaaaaaggaaatattgaatga**

**Citrus_hongheensis** **acatctaacacataatttaaatgggacaatttatcctcgaaaaaaggaaatattgaatga**

**Citrus_limon** **acatctaacacataatttaaatgggacaatttatcctcgaaaaaaggaaatattgaatga**

**Citrus_maxima** **acatctaacacataatttaaatgggacaatttatcctcgaaaaaaggaaatattgaatga**

**Citrus_medica** **acatctaacacataatttaaatgggacaatttatcctcgaaaaaaggaaatattgaatga**

**Citrus_polytrifolia** **acatctaacacataatttaaatgggacaatttatcctcgaaaaaaggaaatattgaatga**

**Citrus_reticulata** **acatctaacacataatttaaatgggacaatttatcctcgaaaaaaggaaatattgaatga**

**Citrus_sinensis** **acatctaacacataatttaaatgggacaatttatcctcgaaaaaaggaaatattgaatga**

**Fortunella_bawangica_1** **acatctaacacataatttaaatgggacaatttatcctcgaaaaaaggaaatattgaatga**

**Fortunella_bawangica_2** **acatctaacacataatttaaatgggacaatttatcctcgaaaaaaggaaatattgaatga**

**Fortunella_hindsii_1** **acatctaacacataatttaaatgggacaatttatcctcgaaaaaaggaaatattgaatga**

**Fortunella_hindsii_2** **acatctaacacataatttaaatgggacaatttatcctcgaaaaaaggaaatattgaatga**

**Fortunella_hindsii_3** **acatctaacacataatttaaatgggacaatttatcctcgaaaaaaggaaatattgaatga**

**Fortunella_japonica_1** **acatctaacacataatttaaatgggacaatttatcctcgaaaaaaggaaatattgaatga**

**Fortunella_japonica_2** **acatctaacacataatttaaatgggacaatttatcctcgaaaaaaggaaatattgaatga**

**Fortunella_japonica_3** **acatctaacacataatttaaatgggacaatttatcctcgaaaaaaggaaatattgaatga**

**Fortunella_japonica_4** **acatctaacacataatttaaatgggacaatttatcctcgaaaaaaggaaatattgaatga**

**Fortunella_japonica_5** **acatctaacacataatttaaatgggacaatttatcctcgaaaaaaggaaatattgaatga**

**Fortunella_japonica_6** **acatctaacacataatttaaatgggacaatttatcctcgaaaaaaggaaatattgaatga**

**Fortunella_japonica_7** **acatctaacacataatttaaatgggacaatttatcctcgaaaaaaggaaatattgaatga**

**Fortunella_japonica_8** **acatctaacacataatttaaatgggacaatttatcctcgaaaaaaggaaatattgaatga**

**Fortunella_japonica_9** **acatctaacacataatttaaatgggacaatttatcctcgaaaaaaggaaatattgaatga**

**Fortunella_japonica_10** **acatctaacacataatttaaatgggacaatttatcctcgaaaaaaggaaatattgaatga**

**Fortunella_japonica_11** **acatctaacacataatttaaatgggacaatttatcctcgaaaaaaggaaatattgaatga**

**Fortunella_japonica_12** **acatctaacacataatttaaatgggacaatttatcctcgaaaaaaggaaatattgaatga**

**Fortunella_margarita_1** **acatctaacacataatttaaatgggacaatttatcctcgaaaaaaggaaatattgaatga**

**Fortunella_margarita_2** **acatctaacacataatttaaatgggacaatttatcctcgaaaaaaggaaatattgaatga**

**Fortunella_venosa_1** **acatctaacacataatttaaatgggacaatttatcctcgaaaaaaggaaatattgaatga**

**Fortunella_venosa_2** **acatctaacacataatttaaatgggacaatttatcctcgaaaaaaggaaatattgaatga**

**Fortunella_venosa_3** **acatctaacacataatttaaatgggacaatttatcctcgaaaaaaggaaatattgaatga**

3430	3440	3450	3460	3470	3480

....|....|....|....|....|....|....|....|....|....|....|....|

**Atalantia_kwangtungens** **attgattggaaattaggcgatttttccatttctttcccttctaaaaaagctaccaaccgt**

**Citrus_reticulata_1** **attgattggaaattaggcgatttttcaatttctttcccttctaaaaaagccaccaaccgt**

**Citrus_reticulata_2** **attgattggaaattaggcgatttttcaatttctttcccttctaaaaaagccaccaaccgt**

**Citrus_trifoliata_3** **attgattggaaattaggcgatttttcaatttcgttcccttctaaaaaagccaccaaccgt**

**Citrus_aurantiifolia** **attgattggaaattaggcgatttttcaatttctttcccttctaaaaaagccaccaaccgt**

**Citrus_aurantium** **attgattggaaattaggcgatttttcaatttctttcccttctaaaaaagccaccaaccgt**

**Citrus_cavaleriei** **attgattggaaattaggcgatttttcaatttctttcccttctaaaaaagccaccaaccgt**

**Citrus_hongheensis** **attgattggaaattaggcgatttttcaatttctttcccttctaaaaaagccaccaaccgt**

**Citrus_limon** **attgattggaaattaggcgatttttcaatttctttcccttctaaaaaagccaccaaccgt**

**Citrus_maxima** **attgattggaaattaggcgatttttcaatttctttcccttctaaaaaagccaacaaccgt**

**Citrus_medica** **attgattggaaattaggcgctttttcaatttctttcccttctaaaaaagccaccaaccgt**

**Citrus_polytrifolia** **attgattggaaattaggcgatttttcaatttcgttcccttctaaaaaagccaccaaccgt**

**Citrus_reticulata** **attgattggaaattaggcgatttttcaatttctttcccttctaaaaaagccaccaaccgt**

**Citrus_sinensis** **attgattggaaattaggcgatttttcaatttctttcccttctaaaaaagccaccaaccgt**

**Fortunella_bawangica_1** **attgattggaaattaggcgatttttcaatttctttcccttctaaaaaagccaccaaccgt**

**Fortunella_bawangica_2** **attgattggaaattaggcgatttttcaatttctttcccttctaaaaaagccaccaaccgt**

**Fortunella_hindsii_1** **attgattggaaattaggcgatttttcaatttctttcccttctaaaaaagccaccaaccgt**

**Fortunella_hindsii_2** **attgattggaaattaggcgatttttcaatttctttcccttctaaaaaagccaccaaccgt**

**Fortunella_hindsii_3** **attgattggaaattaggcgatttttcaatttctttcccttctaaaaaagccaccaaccgt**

**Fortunella_japonica_1** **attgattggaaattaggcgatttttcaatttctttcccttctaaaaaagccaccaaccgt**

**Fortunella_japonica_2** **attgattggaaattaggcgatttttcaatttctttcccttctaaaaaagccaccaaccgt**

**Fortunella_japonica_3** **attgattggaaattaggcgatttttcaatttctttcccttctaaaaaagccaccaaccgt**

**Fortunella_japonica_4** **attgattggaaattaggcgatttttcaatttctttcccttctaaaaaagccaccaaccgt**

**Fortunella_japonica_5** **attgattggaaattaggcgatttttcaatttctttcccttctaaaaaagccaccaaccgt**

**Fortunella_japonica_6** **attgattggaaattaggcgatttttcaatttctttcccttctaaaaaagccaccaaccgt**

**Fortunella_japonica_7** **attgattggaaattaggcgatttttcaatttctttcccttctaaaaaagccaccaaccgt**

**Fortunella_japonica_8** **attgattggaaattaggcgatttttcaatttctttcccttctaaaaaagccaccaaccgt**

**Fortunella_japonica_9** **attgattggaaattaggcgatttttcaatttctttcccttctaaaaaagccaccaaccgt**

**Fortunella_japonica_10** **attgattggaaattaggcgatttttcaatttctttcccttctaaaaaagccaccaaccgt**

**Fortunella_japonica_11** **attgattggaaattaggcgatttttcaatttctttcccttctaaaaaagccaccaaccgt**

**Fortunella_japonica_12** **attgattggaaattaggcgatttttcaatttctttcccttctaaaaaagccaccaaccgt**

**Fortunella_margarita_1** **attgattggaaattaggcgatttttcaatttctttcccttctaaaaaagccaccaaccgt**

**Fortunella_margarita_2** **attgattggaaattaggcgatttttcaatttctttcccttctaaaaaagccaccaaccgt**

**Fortunella_venosa_1** **attgattggaaattaggcgatttttcaatttctttcccttctaaaaaagccaccaaccgt**

**Fortunella_venosa_2** **attgattggaaattaggcgatttttcaatttctttcccttctaaaaaagccaccaaccgt**

**Fortunella_venosa_3** **attgattggaaattaggcgatttttcaatttctttcccttctaaaaaagccaccaaccgt**

3490	3500	3510	3520	3530	3540

....|....|....|....|....|....|....|....|....|....|....|....|

**Atalantia_kwangtungens** **agggaaaatggaatttccaccacagcagcaaatacctctgatataatttgagaatacaac**

**Citrus_reticulata_1** **agggaaaatggaatttccaccacagcagcaaatacctctgatataatttgagaatataac**

**Citrus_reticulata_2** **agggaaaatggaatttccaccacagcagcaaatacctctgatataatttgagaatataac**

**Citrus_trifoliata_3** **agggaaaatggaatttccaccacagcagcaaatacctctgatataatttgagaatataac**

**Citrus_aurantiifolia** **agggaaaatggaatttccaccacagcagcaaatacctctgatataatttgagaatataac**

**Citrus_aurantium** **agggaaaatggaatttccaccacagcagcaaatacctctgatataatttgagaatataac**

**Citrus_cavaleriei** **agggaaaatggaatttccaccacagcagcaaatacctctgatataatttgagaatataac**

**Citrus_hongheensis** **agggaaaatggaatttccaccacagcagcaaatacctctgatataatttgagaatataac**

**Citrus_limon** **agggaaaatggaatttccaccacagcagcaaatacctctgatataatttgagaatataac**

**Citrus_maxima** **agggaaaatggaatttccaccacagcagcaaatacctctgatataatttgagaatataac**

**Citrus_medica** **agggaaaatggaatttccaccccggcagcaaatacctctgatataatttgagaatataac**

**Citrus_polytrifolia** **agggaaaatggaatttccaccacagcagcaaatacctctgatataatttgagaatataac**

**Citrus_reticulata** **agggaaaatggaatttccaccacagcagcaaatacctctgatataatttgagaatataac**

**Citrus_sinensis** **agggaaaatggaatttccaccacagcagcaaatacctctgatataatttgagaatataac**

**Fortunella_bawangica_1** **agggaaaatggaatttccaccacagcagcaaatacctctgatataatttgagaatataac**

**Fortunella_bawangica_2** **agggaaaatggaatttccaccacagcagcaaatacctctgatataatttgagaatataac**

**Fortunella_hindsii_1** **agggaaaatggaatttccaccacagcagcaaatacctctgatataatttgagaatataac**

**Fortunella_hindsii_2** **agggaaaatggaatttccaccacagcagcaaatacctctgatataatttgagaatataac**

**Fortunella_hindsii_3** **agggaaaatggaatttccaccacagcagcaaatacctctgatataatttgagaatataac**

**Fortunella_japonica_1** **agggaaaatggaatttccaccacagcagcaaatacctctgatataatttgagaatataac**

**Fortunella_japonica_2** **agggaaaatggaatttccaccacagcagcaaatacctctgatataatttgagaatataac**

**Fortunella_japonica_3** **agggaaaatggaatttccaccacagcagcaaatacctctgatataatttgagaatataac**

**Fortunella_japonica_4** **agggaaaatggaatttccaccacagcagcaaatacctctgatataatttgagaatataac**

**Fortunella_japonica_5** **agggaaaatggaatttccaccacagcagcaaatacctctgatataatttgagaatataac**

**Fortunella_japonica_6** **agggaaaatggaatttccaccacagcagcaaatacctctgatataatttgagaatataac**

**Fortunella_japonica_7** **agggaaaatggaatttccaccacagcagcaaatacctctgatataatttgagaatataac**

**Fortunella_japonica_8** **agggaaaatggaatttccaccacagcagcaaatacctctgatataatttgagaatataac**

**Fortunella_japonica_9** **agggaaaatggaatttccaccacagcagcaaatacctctgatataatttgagaatataac**

**Fortunella_japonica_10** **agggaaaatggaatttccaccacagcagcaaatacctctgatataatttgagaatataac**

**Fortunella_japonica_11** **agggaaaatggaatttccaccacagcagcaaatacctctgatataatttgagaatataac**

**Fortunella_japonica_12** **agggaaaatggaatttccaccacagcagcaaatacctctgatataatttgagaatataac**

**Fortunella_margarita_1** **agggaaaatggaatttccaccacagcagcaaatacctctgatataatttgagaatataac**

**Fortunella_margarita_2** **agggaaaatggaatttccaccacagcagcaaatacctctgatataatttgagaatataac**

**Fortunella_venosa_1** **agggaaaatggaatttccaccacagcagcaaatacctctgatataatttgagaatataac**

**Fortunella_venosa_2** **agggaaaatggaatttccaccacagcagcaaatacctctgatataatttgagaatataac**

**Fortunella_venosa_3** **agggaaaatggaatttccaccacagcagcaaatacctctgatataatttgagaatataac**

3550	3560	3570	3580	3590	3600

....|....|....|....|....|....|....|....|....|....|....|....|

**Atalantia_kwangtungens** **ttattgttgtgcccaaaaattggattttgattcgaatcattagcagcaatactcaaattt**

**Citrus_reticulata_1** **ttattgttgtgcccaaaaatcggattttgattcgaatcattagcaacaatactcaaatta**

**Citrus_reticulata_2** **ttattgttgtgcccaaaaatcggattttgattcgaatcattagcaacaatactcaaatta**

**Citrus_trifoliata_3** **ttattgttgtgcccaaaaatcggattttgattcgaatcattagcagcaatactcaaatta**

**Citrus_aurantiifolia** **ttattgttgtgcccaaaaatcggattttgattcgaatcattagcagcaatactcaaatta**

**Citrus_aurantium** **ttattgttgtgcccaaaaatcggattttgattcgaatcattagcagcaatactcaaatta**

**Citrus_cavaleriei** **ttattgttgtgcccaaaaatcggattttgattcgaatcattagcaacaatactcaaatta**

**Citrus_hongheensis** **ttattgttgtgcccaaaaataggattttgattcgaatcattagcagcaatactcaaatta**

**Citrus_limon** **ttattgttgtgcccaaaaatcggattttgattcgaatcattagcagcaatactcaaatta**

**Citrus_maxima** **ttattgttgtgcccaaaaatcggattttgattcgaatcattagcagcaatactcaaatta**

**Citrus_medica** **ttattgttgtgcccaaaaatcggattttgattcgaatcattagcagcaatactcaaatta**

**Citrus_polytrifolia** **ttattgttgtgcccaaaaatcggattttgattcgaatcattagcagcaatactcaaatta**

**Citrus_reticulata** **ttattgttgtgcccaaaaatcggattttgattcgaatcattagcaacaatactcaaatta**

**Citrus_sinensis** **ttattgttgtgcccaaaaatcggattttgattcgaatcattagcagcaatactcaaatta**

**Fortunella_bawangica_1** **ttattgttgtgcccaaaaatcggattttgattcgaatcattagcagcaatactaaaatta**

**Fortunella_bawangica_2** **ttattgttgtgcccaaaaatcggattttgattcgaatcattagcagcaatactaaaatta**

**Fortunella_hindsii_1** **ttattgttgtgcccaaaaatcggattttgattcgaatcattagcagcaatactaaaatta**

**Fortunella_hindsii_2** **ttattgttgtgcccaaaaatcggattttgattcgaatcattagcagcaatactaaaatta**

**Fortunella_hindsii_3** **ttattgttgtgcccaaaaatcggattttgattcgaatcattagcagcaatactaaaatta**

**Fortunella_japonica_1** **ttattgttgtgcccaaaaatcggattttgattcgaatcattagcagcaatactaaaatta**

**Fortunella_japonica_2** **ttattgttgtgcccaaaaatcggattttgattcgaatcattagcagcaatactaaaatta**

**Fortunella_japonica_3** **ttattgttgtgcccaaaaatcggattttgattcgaatcattagcagcaatactaaaatta**

**Fortunella_japonica_4** **ttattgttgtgcccaaaaatcggattttgattcgaatcattagcagcaatactaaaatta**

**Fortunella_japonica_5** **ttattgttgtgcccaaaaatcggattttgattcgaatcattagcagcaatactaaaatta**

**Fortunella_japonica_6** **ttattgttgtgcccaaaaatcggattttgattcgaatcattagcagcaatactaaaatta**

**Fortunella_japonica_7** **ttattgttgtgcccaaaaatcggattttgattcgaatcattagcagcaatactaaaatta**

**Fortunella_japonica_8** **ttattgttgtgcccaaaaatcggattttgattcgaatcattagcagcaatactaaaatta**

**Fortunella_japonica_9** **ttattgttgtgcccaaaaatcggattttgattcgaatcattagcagcaatactaaaatta**

**Fortunella_japonica_10** **ttattgttgtgcccaaaaatcggattttgattcgaatcattagcagcaatactaaaatta**

**Fortunella_japonica_11** **ttattgttgtgcccaaaaatcggattttgattcgaatcattagcagcaatactaaaatta**

**Fortunella_japonica_12** **ttattgttgtgcccaaaaatcggattttgattcgaatcattagcagcaatactaaaatta**

**Fortunella_margarita_1** **ttattgttgtgcccaaaaatcggattttgattcgaatcattagcagcaatactaaaatta**

**Fortunella_margarita_2** **ttattgttgtgcccaaaaatcggattttgattcgaatcattagcagcaatactaaaatta**

**Fortunella_venosa_1** **ttattgttgtgcccaaaaatcggattttgattcgaatcattagcagcaatactaaaatta**

**Fortunella_venosa_2** **ttattgttgtgcccaaaaatcggattttgattcaaatcattagcagcaatactaaaatta**

**Fortunella_venosa_3** **ttattgttgtgcccaaaaatcggattttgattcgaatcattagcagcaatactaaaatta**

3610	3620	3630	3640	3650	3660

....|....|....|....|....|....|....|....|....|....|....|....|

**Atalantia_kwangtungens** **atctgttgatacattctagtaattaaccgtttcacacttagtgaactagatttattgtca**

**Citrus_reticulata_1** **atccgttgatacattctagtaattaaccgtttcacacttagtgaactagatttattgtca**

**Citrus_reticulata_2** **atccgttgatacattctagtaattaaccgtttcacacttagtgaactagatttattgtca**

**Citrus_trifoliata_3** **atccgttgatacattctagtaattaaccgtttcacacttagtgaactagatttattgtca**

**Citrus_aurantiifolia** **atccgttgatacattctagtaattaaccgtttcacacttagtgaactagatttattgtca**

**Citrus_aurantium** **atccgttgatacattctagtaattaaccgtttcacacttagtgaactagatttattgtca**

**Citrus_cavaleriei** **atccgttgatacattctagtaattaaccgtttcacacttagtgaactagatttattgtca**

**Citrus_hongheensis** **atccgttgatacattctagtaattaaccgtttcacacttagtgaactagatttattgtca**

**Citrus_limon** **atccgttgatacattctagtaattaaccgtttcacacttagtgaactagatttattgtca**

**Citrus_maxima** **atccgttgatacattctagtaattaaccgtttcacacttagtgaactagatttattgtca**

**Citrus_medica** **atccgttgatacattctagtaattaaccgtttcacacttagtgaactagatttattgtca**

**Citrus_polytrifolia** **atccgttgatacattctagtaattaaccgtttcacacttagtgaactagatttattgtca**

**Citrus_reticulata** **atccgttgatacattctagtaattaaccgtttcacacttagtgaactagatttattgtca**

**Citrus_sinensis** **atccgttgatacattctagtaattaaccgtttcacacttagtgaactagatttattgtca**

**Fortunella_bawangica_1** **atccgttgatacattctagtaattaaccgtttcacacttagtgaactagatttattgtca**

**Fortunella_bawangica_2** **atccgttgatacattctagtaattaaccgtttcacacttagtgaactagatttattgtca**

**Fortunella_hindsii_1** **atccgttgatacattctagtaattaaccgtttcacacttagtgaactagatttattgtca**

**Fortunella_hindsii_2** **atccgttgatacattctagtaattaaccgtttcacacttagtgaactagatttattgtca**

**Fortunella_hindsii_3** **atccgttgatacattctagtaattaaccgtttcacacttagtgaactagatttattgtca**

**Fortunella_japonica_1** **atccgttgatacattctagtaattaaccgtttcacacttagtgaactagatttattgtca**

**Fortunella_japonica_2** **atccgttgatacattctagtaattaaccgtttcacacttagtgaactagatttattgtca**

**Fortunella_japonica_3** **atccgttgatacattctagtaattaaccgtttcacacttagtgaactagatttattgtca**

**Fortunella_japonica_4** **atccgttgatacattctagtaattaaccgtttcacacttagtgaactagatttattgtca**

**Fortunella_japonica_5** **atccgttgatacattctagtaattaaccgtttcacacttagtgaactagatttattgtca**

**Fortunella_japonica_6** **atccgttgatacattctagtaattaaccgtttcacacttagtgaactagatttattgtca**

**Fortunella_japonica_7** **atccgttgatacattctagtaattaaccgtttcacacttagtgaactagatttattgtca**

**Fortunella_japonica_8** **atccgttgatacattctagtaattaaccgtttcacacttagtgaactagatttattgtca**

**Fortunella_japonica_9** **atccgttgatacattctagtaattaaccgtttcacacttagtgaactagatttattgtca**

**Fortunella_japonica_10** **atccgttgatacattctagtaattaaccgtttcacacttagtgaactagatttattgtca**

**Fortunella_japonica_11** **atccgttgatacattctagtaattaaccgtttcacacttagtgaactagatttattgtca**

**Fortunella_japonica_12** **atccgttgatacattctagtaattaaccgtttcacacttagtgaactagatttattgtca**

**Fortunella_margarita_1** **atccgttgatacattctagtaattaaccgtttcacacttagtgaactagatttattgtca**

**Fortunella_margarita_2** **atccgttgatacattctagtaattaaccgtttcacacttagtgaactagatttattgtca**

**Fortunella_venosa_1** **atccgttgatacattctagtaattaaccgtttcacacttagtgaactagatttattgtca**

**Fortunella_venosa_2** **atccgttgatacattctagtaattaaccgtttcacacttagtgaactagatttattgtca**

**Fortunella_venosa_3** **atccgttgatacattctagtaattaaccgtttcacacttagtgaactagatttattgtca**

3670	3680	3690	3700	3710	3720

....|....|....|....|....|....|....|....|....|....|....|....|

**Atalantia_kwangtungens** **taaaacccaccttccaatgacatcatcgagctatttaaaccatgatcatgagcaagtaca**

**Citrus_reticulata_1** **taaaacccaccttccaatgacatcatcgagctatttaaaccatgatcatgagcaagtaca**

**Citrus_reticulata_2** **taaaacccaccttccaatgacatcatcgagctatttaaaccatgatcatgagcaagtaca**

**Citrus_trifoliata_3** **taaaacccaccttccaatgacatcatcgagctatttaaaccatgatcatgagcaagtaca**

**Citrus_aurantiifolia** **taaaacccaccttccaatgacatcatcgagctatttaaaccatgatcatgagcaagtaca**

**Citrus_aurantium** **taaaacccaccttccaatgacatcatcgagctatttaaaccatgatcatgagcaagtaca**

**Citrus_cavaleriei** **taaaacccaccttccaatgacatcatcgagctatttaaaccatgatcatgagcaagtaca**

**Citrus_hongheensis** **taaaacccaccttccaatgacatcatcgagctatttaaaccatgatcatgagcaagtaca**

**Citrus_limon** **taaaacccaccttccaatgacatcatcgagctatttaaaccatgatcatgagcaagtaca**

**Citrus_maxima** **taaaacccaccttccaatgacatcatcgagctatttaaaccatgatcatgagcaagtaca**

**Citrus_medica** **taaaacccaccttccaatgacatcatcgagctatttaaaccatgatcatgagcaagtaca**

**Citrus_polytrifolia** **taaaacccaccttccaatgacatcatcgagctatttaaaccatgatcatgagcaagtaca**

**Citrus_reticulata** **taaaacccaccttccaatgacatcatcgagctatttaaaccatgatcatgagcaagtaca**

**Citrus_sinensis** **taaaacccaccttccaatgacatcatcgagctatttaaaccatgatcatgagcaagtaca**

**Fortunella_bawangica_1** **taaaacccaccttccaatgacatcatcgagctatttaaaccatgatcatgagcaagtaca**

**Fortunella_bawangica_2** **taaaacccaccttccaatgacatcatcgagctatttaaaccatgatcatgagcaagtaca**

**Fortunella_hindsii_1** **taaaacccaccttccaatgacatcatcgagctatttaaaccatgatcatgagcaagtaca**

**Fortunella_hindsii_2** **taaaacccaccttccaatgacatcatcgagctatttaaaccatgatcatgagcaagtaca**

**Fortunella_hindsii_3** **taaaacccaccttccaatgacatcatcgagctatttaaaccatgatcatgagcaagtaca**

**Fortunella_japonica_1** **taaaacccaccttccaatgacatcatcgagctatttaaaccatgatcatgagcaagtaca**

**Fortunella_japonica_2** **taaaacccaccttccaatgacatcatcgagctatttaaaccatgatcatgagcaagtaca**

**Fortunella_japonica_3** **taaaacccaccttccaatgacatcatcgagctatttaaaccatgatcatgagcaagtaca**

**Fortunella_japonica_4** **taaaacccaccttccaatgacatcatcgagctatttaaaccatgatcatgagcaagtaca**

**Fortunella_japonica_5** **taaaacccaccttccaatgacatcatcgagctatttaaaccatgatcatgagcaagtaca**

**Fortunella_japonica_6** **taaaacccaccttccaatgacatcatcgagctatttaaaccatgatcatgagcaagtaca**

**Fortunella_japonica_7** **taaaacccaccttccaatgacatcatcgagctatttaaaccatgatcatgagcaagtaca**

**Fortunella_japonica_8** **taaaacccaccttccaatgacatcatcgagctatttaaaccatgatcatgagcaagtaca**

**Fortunella_japonica_9** **taaaacccaccttccaatgacatcatcgagctatttaaaccatgatcatgagcaagtaca**

**Fortunella_japonica_10** **taaaacccaccttccaatgacatcatcgagctatttaaaccatgatcatgagcaagtaca**

**Fortunella_japonica_11** **taaaacccaccttccaatgacatcatcgagctatttaaaccatgatcatgagcaagtaca**

**Fortunella_japonica_12** **taaaacccaccttccaatgacatcatcgagctatttaaaccatgatcatgagcaagtaca**

**Fortunella_margarita_1** **taaaacccaccttccaatgacatcatcgagctatttaaaccatgatcatgagcaagtaca**

**Fortunella_margarita_2** **taaaacccaccttccaatgacatcatcgagctatttaaaccatgatcatgagcaagtaca**

**Fortunella_venosa_1** **taaaacccaccttccaatgacatcatcgagctatttaaaccatgatcatgagcaagtaca**

**Fortunella_venosa_2** **taaaacccaccttccaatgacatcatcgagctatttaaaccatgatcatgagcaagtaca**

**Fortunella_venosa_3** **taaaacccaccttccaatgacatcatcgagctatttaaaccatgatcatgagcaagtaca**

3730	3740	3750	3760	3770	3780

....|....|....|....|....|....|....|....|....|....|....|....|

**Atalantia_kwangtungens** **taaatatactcccgaaaaataagtgggtataggaagtcgtgttgttgagatctatctagt**

**Citrus_reticulata_1** **taaatatactcccgaaaaagaagtgggtataggaagtcgtgttgttgagatctatctagt**

**Citrus_reticulata_2** **taaatatactcccgaaaaagaagtgggtataggaagtcgtgttgttgagatctatctagt**

**Citrus_trifoliata_3** **taaatatactcccgaaaaagaagtgggtataggaagtcgtgttgttgagatctatctagt**

**Citrus_aurantiifolia** **taaatatactcccgaaaaagaagtgggtataggaagtcgtgttgttgagatctatctagt**

**Citrus_aurantium** **taaatatactcccgaaaaagaagtgggtataggaagtcgtgttgttgagatctatctagt**

**Citrus_cavaleriei** **taaatatactcctgaaaaagaagtgggtataggaagtcgtgttgttgagatctatctagt**

**Citrus_hongheensis** **taaatatactcccgaaaaagaagtgggtataggaagtcgtgttgttgagatctatctagt**

**Citrus_limon** **taaatatactcccgaaaaagaagtgggtataggaagtcgtgttgttgagatctatctagt**

**Citrus_maxima** **taaatatactcccgaaaaagaagtgggtataggaagtcgtgttgttgagatctatctagt**

**Citrus_medica** **taaatatactcccgaaaaagaagtgggtataggaagtcgtgttgttgagatctatctagt**

**Citrus_polytrifolia** **taaatatactcccgaaaaagaagtgggtataggaagtcgtgttgttgagatctatctagt**

**Citrus_reticulata** **taaatatactcccgaaaaagaagtgggtataggaagtcgtgttgttgagatctatctagt**

**Citrus_sinensis** **taaatatactcccgaaaaagaagtgggtataggaagtcgtgttgttgagatctatctagt**

**Fortunella_bawangica_1** **taaatatactcccgaaaaagaagtgggtataggaagtcgtgttgttgagatctatctagt**

**Fortunella_bawangica_2** **taaatatactcccgaaaaagaagtgggtataggaagtcgtgttgttgagatctatctagt**

**Fortunella_hindsii_1** **taaatatactcccgaaaaagaagtgggtataggaagtcgtgttgttgagatctatctagt**

**Fortunella_hindsii_2** **taaatatactcccgaaaaagaagtgggtataggaagtcgtgttgttgagatctatctagt**

**Fortunella_hindsii_3** **taaatatactcccgaaaaagaagtgggtataggaagtcgtgttgttgagatctatctagt**

**Fortunella_japonica_1** **taaatatactcccgaaaaagaagtgggtataggaagtcgtgttgttgagatctatctagt**

**Fortunella_japonica_2** **taaatatactcccgaaaaagaagtgggtataggaagtcgtgttgttgagatctatctagt**

**Fortunella_japonica_3** **taaatatactcccgaaaaagaagtgggtataggaagtcgtgttgttgagatctatctagt**

**Fortunella_japonica_4** **taaatatactcccgaaaaagaagtgggtataggaagtcgtgttgttgagatctatctagt**

**Fortunella_japonica_5** **taaatatactcccgaaaaagaagtgggtataggaagtcgtgttgttgagatctatctagt**

**Fortunella_japonica_6** **taaatatactcccgaaaaagaagtgggtataggaagtcgtgttgttgagatctatctagt**

**Fortunella_japonica_7** **taaatatactcccgaaaaagaagtgggtataggaagtcgtgttgttgagatctatctagt**

**Fortunella_japonica_8** **taaatatactcccgaaaaagaagtgggtataggaagtcgtgttgttgagatctatctagt**

**Fortunella_japonica_9** **taaatatactcccgaaaaagaagtgggtataggaagtcgtgttgttgagatctatctagt**

**Fortunella_japonica_10** **taaatatactcccgaaaaagaagtgggtataggaagtcgtgttgttgagatctatctagt**

**Fortunella_japonica_11** **taaatatactcccgaaaaagaagtgggtataggaagtcgtgttgttgagatctatctagt**

**Fortunella_japonica_12** **taaatatactcccgaaaaagaagtgggtataggaagtcgtgttgttgagatctatctagt**

**Fortunella_margarita_1** **taaatatactcccgaaaaagaagtgggtataggaagtcgtgttgttgagatctatctagt**

**Fortunella_margarita_2** **taaatatactcccgaaaaagaagtgggtataggaagtcgtgttgttgagatctatctagt**

**Fortunella_venosa_1** **taaatatactcccgaaaaagaagtgggtataggaagtcgtgttgttgagatctatctagt**

**Fortunella_venosa_2** **taaatatactcccgaaaaagaagtgggtataggaagtcgtgttgttgagatctatctagt**

**Fortunella_venosa_3** **taaatatactcccgaaaaagaagtgggtataggaagtcgtgttgttgagatctatctagt**

3790	3800	3810	3820	3830	3840

....|....|....|....|....|....|....|....|....|....|....|....|

**Atalantia_kwangtungens** **gctaaatatacttgaaattcctccatttgaaattcgattaaaaaaaaaaacaacaaaggt**

**Citrus_reticulata_1** **tctaaatatacttgaaattcctccatttgaaattcgattaaa---------aacaaaggt**

**Citrus_reticulata_2** **tctaaatatacttgaaattcctccatttgaaattcgattaaa---------aacaaaggt**

**Citrus_trifoliata_3** **tctaaatatacttgaaattcctccatttgaaattcgattaaa---------aacaagggt**

**Citrus_aurantiifolia** **tctaaatatacttgaaattcctccatttgaaattcgattaaa---------aacaaaggt**

**Citrus_aurantium** **tctaaatatacttgaaattcctccatttgaaattcgattaaa---------aacaaaggt**

**Citrus_cavaleriei** **tctaaatatacttgaaattcctccatttgaaattcgattaaa---------aacaaaggt**

**Citrus_hongheensis** **tctaaatatacttgaaattcctccatttgaaattcgattaaa---------aacaaaggt**

**Citrus_limon** **tctaaatatacttgaaattcctccatttgaaattcgattaaa---------aacaaaggt**

**Citrus_maxima** **tctaaatatacttgaaattcctccatttgaaattcgattaaa---------aacaaaggt**

**Citrus_medica** **tctaaatatacttgaaattcctccatttgaaattcgattaaa---------aacaaaggt**

**Citrus_polytrifolia** **tctaaatatacttgaaattcctccatttgaaattcgattaaa---------aacaagggt**

**Citrus_reticulata** **tctaaatatacttgaaattcctccatttgaaattcgattaaa---------aacaaaggt**

**Citrus_sinensis** **tctaaatatacttgaaattcctccatttgaaattcgattaaa---------aacaaaggt**

**Fortunella_bawangica_1** **tctaaatatacttgaaattcctccatttgaaattcgattaaa---------aacaaaggt**

**Fortunella_bawangica_2** **tctaaatatacttgaaattcctccatttgaaattcgattaaa---------aacaaaggt**

**Fortunella_hindsii_1** **tctaaatatacttgaaattcctccatttgaaattcgattaaa---------aacaaaggt**

**Fortunella_hindsii_2** **tctaaatatacttgaaattcctccatttgaaattcgattaaa---------aacaaaggt**

**Fortunella_hindsii_3** **tctaaatatacttgaaattcctccatttgaaattcgattaaa---------aacaaaggt**

**Fortunella_japonica_1** **tctaaatatacttgaaattcctccatttgaaattcgattaaa---------aacaaaggt**

**Fortunella_japonica_2** **tctaaatatacttgaaattcctccatttgaaattcgattaaa---------aacaaaggt**

**Fortunella_japonica_3** **tctaaatatacttgaaattcctccatttgaaattcgattaaa---------aacaaaggt**

**Fortunella_japonica_4** **tctaaatatacttgaaattcctccatttgaaattcgattaaa---------aacaaaggt**

**Fortunella_japonica_5** **tctaaatatacttgaaattcctccatttgaaattcgattaaa---------aacaaaggt**

**Fortunella_japonica_6** **tctaaatatacttgaaattcctccatttgaaattcgattaaa---------aacaaaggt**

**Fortunella_japonica_7** **tctaaatatacttgaaattcctccatttgaaattcgattaaa---------aacaaaggt**

**Fortunella_japonica_8** **tctaaatatacttgaaattcctccatttgaaattcgattaaa---------aacaaaggt**

**Fortunella_japonica_9** **tctaaatatacttgaaattcctccatttgaaattcgattaaa---------aacaaaggt**

**Fortunella_japonica_10** **tctaaatatacttgaaattcctccatttgaaattcgattaaa---------aacaaaggt**

**Fortunella_japonica_11** **tctaaatatacttgaaattcctccatttgaaattcgattaaa---------aacaaaggt**

**Fortunella_japonica_12** **tctaaatatacttgaaattcctccatttgaaattcgattaaa---------aacaaaggt**

**Fortunella_margarita_1** **tctaaatatacttgaaattcctccatttgaaattcgattaaa---------aacaaaggt**

**Fortunella_margarita_2** **tctaaatatacttgaaattcctccatttgaaattcgattaaa---------aacaaaggt**

**Fortunella_venosa_1** **tctaaatatacttgaaattcctccatttgaaattcgattaaa---------aacaaaggt**

**Fortunella_venosa_2** **tctaaatatacttgaaattcctccatttgaaattcgattaaa---------aacaaaggt**

**Fortunella_venosa_3** **tctaaatatacttgaaattcctccatttgaaattcgattaaa---------aacaaaggt**

3850	3860	3870	3880	3890	3900

....|....|....|....|....|....|....|....|....|....|....|....|

**Atalantia_kwangtungens** **aagggatttagtgagcgatcaaacgatacatagtgcgatacagtgaaaacaaagtattgt**

**Citrus_reticulata_1** **aagggatttagtgagcgatcaaacgatacatagtgcgatacggtgaaaacaaagtattgt**

**Citrus_reticulata_2** **aagggatttagtgagcgatcaaacgatacatagtgcgatacggtgaaaacaaagtattgt**

**Citrus_trifoliata_3** **aagggatttagtgagcgatcaaacgatacatagtgcgatacggttaaaacaaagtattgt**

**Citrus_aurantiifolia** **acgggatttagtgagcgatcaaacgatacatagtgcgatacggtgaaaacaaagtattgt**

**Citrus_aurantium** **aagggatttagtgagcgatcaaacgatacatagtgcgatacggtgaaaacaaagtattgt**

**Citrus_cavaleriei** **aagggatttagtgagcgatcaaacgatacatagtgcgatacggtgaaaacaaagtattgt**

**Citrus_hongheensis** **aagggatttagtgagcgatcaaacgatacatagtgcgatacggtgaaaacaaagtattgt**

**Citrus_limon** **aagggatttagtgagcgatcaaacgatacatagtgcgatacggtgaaaacaaagtattgt**

**Citrus_maxima** **aagggatttagtgagcgatcaaacgatacatagtgcgatacggtgaaaacaaagtattgt**

**Citrus_medica** **aagggatttagtgagcgatcaaacgatacatagtgcgatacggtgaaaacaaagtattgt**

**Citrus_polytrifolia** **aagggatttagtgagcgatcaaacgatacatagtgcgatacggttaaaacaaagtattgt**

**Citrus_reticulata** **aagggatttagtgagcgatcaaacgatacatagtgcgatacggtgaaaacaaagtattgt**

**Citrus_sinensis** **aagggatttagtgagcgatcaaacgatacatagtgcgatacggtgaaaacaaagtattgt**

**Fortunella_bawangica_1** **aagggatttagtgagcgatcaaacgatacatagtgcgatacggtgaaaacaaagtattgt**

**Fortunella_bawangica_2** **aagggatttagtgagcgatcaaacgatacatagtgcgatacggtgaaaacaaagtattgt**

**Fortunella_hindsii_1** **aagggatttagtgagcgatcaaacgatacatagtgcgatacggtgaaaacaaagtattgt**

**Fortunella_hindsii_2** **aagggatttagtgagcgatcaaacgatacatagtgcgatacggtgaaaacaaagtattgt**

**Fortunella_hindsii_3** **aagggatttagtgagcgatcaaacgatacatagtgcgatacggtgaaaacaaagtattgt**

**Fortunella_japonica_1** **aagggatttagtgagcgatcaaacgatacatagtgcgatacggtgaaaacaaagtattgt**

**Fortunella_japonica_2** **aagggatttagtgagcgatcaaacgatacatagtgcgatacggtgaaaacaaagtattgt**

**Fortunella_japonica_3** **aagggatttagtgagcgatcaaacgatacatagtgcgatacggtgaaaacaaagtattgt**

**Fortunella_japonica_4** **aagggatttagtgagcgatcaaacgatacatagtgcgatacggtgaaaacaaagtattgt**

**Fortunella_japonica_5** **aagggatttagtgagcgatcaaacgatacatagtgcgatacggtgaaaacaaagtattgt**

**Fortunella_japonica_6** **aagggatttagtgagcgatcaaacgatacatagtgcgatacggtgaaaacaaagtattgt**

**Fortunella_japonica_7** **aagggatttagtgagcgatcaaacgatacatagtgcgatacggtgaaaacaaagtattgt**

**Fortunella_japonica_8** **aagggatttagtgagcgatcaaacgatacatagtgcgatacggtgaaaacaaagtattgt**

**Fortunella_japonica_9** **aagggatttagtgagcgatcaaacgatacatagtgcgatacggtgaaaacaaagtattgt**

**Fortunella_japonica_10** **aagggatttagtgagcgatcaaacgatacatagtgcgatacggtgaaaacaaagtattgt**

**Fortunella_japonica_11** **aagggatttagtgagcgatcaaacgatacatagtgcgatacggtgaaaacaaagtattgt**

**Fortunella_japonica_12** **aagggatttagtgagcgatcaaacgatacatagtgcgatacggtgaaaacaaagtattgt**

**Fortunella_margarita_1** **aagggatttagtgagcgatcaaacgatacatagtgcgatacggtgaaaacaaagtattgt**

**Fortunella_margarita_2** **aagggatttagtgagcgatcaaacgatacatagtgcgatacggtgaaaacaaagtattgt**

**Fortunella_venosa_1** **aagggatttagtgagcgatcaaacgatacatagtgcgatacggtgaaaacaaagtattgt**

**Fortunella_venosa_2** **aagggatttagtgagcgatcaaacgatacatagtgcgatacggtgaaaacaaagtattgt**

**Fortunella_venosa_3** **aagggatttagtgagcgatcaaacgatacatagtgcgatacggtgaaaacaaagtattgt**

3910	3920	3930	3940	3950	3960

....|....|....|....|....|....|....|....|....|....|....|....|

**Atalantia_kwangtungens** **agtaaaaaaagtagataccttgaaaatgaaaatgggtagactcatcaccggattctctat**

**Citrus_reticulata_1** **agtaaaaaaagtggatacct------tgaaaatgggtagactcatcaccggattctctat**

**Citrus_reticulata_2** **agtaaaaaaagtggatacct------tgaaaatgggtagactcatcaccggattctctat**

**Citrus_trifoliata_3** **agtaaaaaaagtggatacct------tgaaaatgggtagactcatcaccggattctctat**

**Citrus_aurantiifolia** **agt-aaaaaagtggatacct------tgaaaatgggtagactcatcaccggattctctat**

**Citrus_aurantium** **agt-aaaaaagtggatacct------tgaaaatgggtagactcatcaccggattctctat**

**Citrus_cavaleriei** **agtaaaaaaagtggatacct------tgaaaatggatagactcatcaccggattctctat**

**Citrus_hongheensis** **agt-aaaaaagtggatacct------tgaaaatgggtagactcatcaccggattctctat**

**Citrus_limon** **agt-aaaaaagtggatacct------tgaaaatgggtagactcatcaccggattctctat**

**Citrus_maxima** **agt-aaaaaagtggatacct------tgaaaatgggtagactcatcaccggattctctat**

**Citrus_medica** **agtaaaaaaagtggatacct------tgaaaatgggtagactcatcaccggattctctat**

**Citrus_polytrifolia** **agtaaaaaaagtggatacct------tgaaaatgggtagactcatcaccggattctctat**

**Citrus_reticulata** **agtaaaaaaagtggatacct------tgaaaatgggtagactcatcaccggattctctat**

**Citrus_sinensis** **agt-aaaaaagtggatacct------tgaaaatgggtagactcatcaccggattctctat**

**Fortunella_bawangica_1** **agtaaaaaaagtggatacct------tgaaaatgggtagactcatcaccggattctctat**

**Fortunella_bawangica_2** **agtaaaaaaagtggatacct------tgaaaatgggtagactcatcaccggattctctat**

**Fortunella_hindsii_1** **agtaaaaaaagtggatacct------tgaaaatgggtagactcatcaccggattctctat**

**Fortunella_hindsii_2** **agtaaaaaaagtggatacct------tgaaaatgggtagactcatcaccggattctctat**

**Fortunella_hindsii_3** **agtaaaaaaagtggatacct------tgaaaatgggtagactcatcaccggattctctat**

**Fortunella_japonica_1** **agtaaaaaaagtggatacct------tgaaaatgggtagactcatcaccggattctctat**

**Fortunella_japonica_2** **agtaaaaaaagtggatacct------tgaaaatgggtagactcatcaccggattctctat**

**Fortunella_japonica_3** **agtaaaaaaagtggatacct------tgaaaatgggtagactcatcaccggattctctat**

**Fortunella_japonica_4** **agtaaaaaaagtggatacct------tgaaaatgggtagactcatcaccggattctctat**

**Fortunella_japonica_5** **agtaaaaaaagtggatacct------tgaaaatgggtagactcatcaccggattctctat**

**Fortunella_japonica_6** **agtaaaaaaagtggatacct------tgaaaatgggtagactcatcaccggattctctat**

**Fortunella_japonica_7** **agtaaaaaaagtggatacct------tgaaaatgggtagactcatcaccggattctctat**

**Fortunella_japonica_8** **agtaaaaaaagtggatacct------tgaaaatgggtagactcatcaccggattctctat**

**Fortunella_japonica_9** **agtaaaaaaagtggatacct------tgaaaatgggtagactcatcaccggattctctat**

**Fortunella_japonica_10** **agtaaaaaaagtggatacct------tgaaaatgggtagactcatcaccggattctctat**

**Fortunella_japonica_11** **agtaaaaaaagtggatacct------tgaaaatgggtagactcatcaccggattctctat**

**Fortunella_japonica_12** **agtaaaaaaagtggatacct------tgaaaatgggtagactcatcaccggattctctat**

**Fortunella_margarita_1** **agtaaaaaaagtggatacct------tgaaaatgggtagactcatcaccggattctctat**

**Fortunella_margarita_2** **agtaaaaaaagtggatacct------tgaaaatgggtagactcatcaccggattctctat**

**Fortunella_venosa_1** **agtaaaaaaagtggatacct------tgaaaatgggtagactcatcaccggattctctat**

**Fortunella_venosa_2** **agtaaaaaaagtggatacct------tgaaaatgggtagactcatcaccggattctctat**

**Fortunella_venosa_3** **agtaaaaaaagtggatacct------tgaaaatgggtagactcatcaccggattctctat**

3970	3980	3990	4000	4010	4020

....|....|....|....|....|....|....|....|....|....|....|....|

**Atalantia_kwangtungens** **cctctcattttgagttaatttaattggtttatgttt------gttatagtataactaagt**

**Citrus_reticulata_1** **cctctcattttgagttaatttaattggtttatgtttgttatagttatagtataactaagt**

**Citrus_reticulata_2** **cctctcattttgagttaatttaattggtttatgtttgttatagttatagtataactaagt**

**Citrus_trifoliata_3** **cctctcattttgagttaatttaattggtttatgtttgttatagttatagtataactaagt**

**Citrus_aurantiifolia** **cctctcattttgagttaatttaattggtttatgtttgttatagttatagtataactaagt**

**Citrus_aurantium** **cctctcattttgagttaatttaattggtttatgtttgttatagttatagtataactaagt**

**Citrus_cavaleriei** **cctctcattttgagttaatttaattggtttatgtttgttatagttatagtataactaagt**

**Citrus_hongheensis** **cctctcattttgagttaatttaattggtttatgtttgttatagttatagtataactaagt**

**Citrus_limon** **cctctcattttgagttaatttaattggtttatgtttgttatagttatagtataactaagt**

**Citrus_maxima** **cctctcattttgagttaatttaattggtttatgtttgttatagttatagtataactaagt**

**Citrus_medica** **cctctcattttgagttaatttaattggtttatgtttgttatagttatagtataactaagt**

**Citrus_polytrifolia** **cctctcattttgagttaatttaattggtttatgtttgttatagttatagtataactaagt**

**Citrus_reticulata** **cctctcattttgagttaatttaattggtttatgtttgttatagttatagtataactaagt**

**Citrus_sinensis** **cctctcattttgagttaatttaattggtttatgtttgttatagttatagtataactaagt**

**Fortunella_bawangica_1** **cctctcattttgagttaatttaattggtttatgtttgttatagttatagtataactaagt**

**Fortunella_bawangica_2** **cctctcattttgagttaatttaattggtttatgtttgttatagttatagtataactaagt**

**Fortunella_hindsii_1** **cctctcattttgagttaatttaattggtttatgtttgttatagttatagtataactaagt**

**Fortunella_hindsii_2** **cctctcattttgagttaatttaattggtttatgtttgttatagttatagtataactaagt**

**Fortunella_hindsii_3** **cctctcattttgagttaatttaattggtttatgtttgttatagttatagtataactaagt**

**Fortunella_japonica_1** **cctctcattttgagttaatttaattggtttatgtttgttatagttatagtataactaagt**

**Fortunella_japonica_2** **cctctcattttgagttaatttaattggtttatgtttgttatagttatagtataactaagt**

**Fortunella_japonica_3** **cctctcattttgagttaatttaattggtttatgtttgttatagttatagtataactaagt**

**Fortunella_japonica_4** **cctctcattttgagttaatttaattggtttatgtttgttatagttatagtataactaagt**

**Fortunella_japonica_5** **cctctcattttgagttaatttaattggtttatgtttgttatagttatagtataactaagt**

**Fortunella_japonica_6** **cctctcattttgagttaatttaattggtttatgtttgttatagttatagtataactaagt**

**Fortunella_japonica_7** **cctctcattttgagttaatttaattggtttatgtttgttatagttatagtataactaagt**

**Fortunella_japonica_8** **cctctcattttgagttaatttaattggtttatgtttgttatagttatagtataactaagt**

**Fortunella_japonica_9** **cctctcattttgagttaatttaattggtttatgtttgttatagttatagtataactaagt**

**Fortunella_japonica_10** **cctctcattttgagttaatttaattggtttatgtttgttatagttatagtataactaagt**

**Fortunella_japonica_11** **cctctcattttgagttaatttaattggtttatgtttgttatagttatagtataactaagt**

**Fortunella_japonica_12** **cctctcattttgagttaatttaattggtttatgtttgttatagttatagtataactaagt**

**Fortunella_margarita_1** **cctctcattttgagttaatttaattggtttatgtttgttatagttatagtataactaagt**

**Fortunella_margarita_2** **cctctcattttgagttaatttaattggtttatgtttgttatagttatagtataactaagt**

**Fortunella_venosa_1** **cctctcattttgagttaatttaattggtttatgtttgttatagttatagtataactaagt**

**Fortunella_venosa_2** **cctctcattttgagttaatttaattggtttatgtttgttatagttatagtataactaagt**

**Fortunella_venosa_3** **cctctcattttgagttaatttaattggtttatgtttgttatagttatagtataactaagt**

4030	4040	4050	4060	4070	4080

....|....|....|....|....|....|....|....|....|....|....|....|

**Atalantia_kwangtungens** **ggttagaaaccctttattttttcactccaatcgctcttttgattttggaaaa-----aac**

**Citrus_reticulata_1** **ggttagaaaccctttattttttcactccaatcgctcttttgattttggaaaaaaaaaaac**

**Citrus_reticulata_2** **ggttagaaaccctttattttttcactccaatcgctcttttgattttggaaaaaaaaaaac**

**Citrus_trifoliata_3** **ggttagaaaccctttattttttcactccaatcgctcttttgattttggaaaaa----aac**

**Citrus_aurantiifolia** **ggttagaaaccctttattttttcactccaatcgctcttttgattttggaaaaa----aac**

**Citrus_aurantium** **ggttagaaaccctttattttttcactccaatcgctcttttgattttggaaaaaa---aac**

**Citrus_cavaleriei** **ggttagaaaccctttattttttcactccaatcgctcttttgattttggaaaaaaaacaac**

**Citrus_hongheensis** **ggttagaaaccctttattttttcactccaatcgctcttttgattttggaaaaaa---aac**

**Citrus_limon** **ggttagaaaccctttattttttcactccaatcgctcttttgattttggaaaaaa---aac**

**Citrus_maxima** **ggttagaaaccctttattttttcactccaatcgctcttttgattttggaaaaaa---aac**

**Citrus_medica** **ggttagaaaccctttattttttcactccaatcgctcttttgattttggaaaaa----aac**

**Citrus_polytrifolia** **ggttagaaaccctttattttttcactccaatcgctcttttgattttggaaaaa----aac**

**Citrus_reticulata** **ggttagaaaccctttattttttcactccaatcgctcttttgattttggaaaaaaaaaaac**

**Citrus_sinensis** **ggttagaaaccctttattttttcactccaatcgctcttttgattttggaaaaaa---aac**

**Fortunella_bawangica_1** **ggttagaaaccctttattttttcactccaatcgctcttttgattttggaaaaaa---aac**

**Fortunella_bawangica_2** **ggttagaaaccctttattttttcactccaatcgctcttttgattttggaaaaaa---aac**

**Fortunella_hindsii_1** **ggttagaaaccctttattttttcactccaatcgctcttttgattttggaaaaaa---aac**

**Fortunella_hindsii_2** **ggttagaaaccctttattttttcactccaatcgctcttttgattttggaaaaaa---aac**

**Fortunella_hindsii_3** **ggttagaaaccctttattttttcactccaatcgctcttttgattttggaaaaaa---aac**

**Fortunella_japonica_1** **ggttagaaaccctttattttttcactccaatcgctcttttgattttggaaaaaa---aac**

**Fortunella_japonica_2** **ggttagaaaccctttattttttcactccaatcgctcttttgattttggaaaaaa---aac**

**Fortunella_japonica_3** **ggttagaaaccctttattttttcactccaatcgctcttttgattttggaaaaaa---aac**

**Fortunella_japonica_4** **ggttagaaaccctttattttttcactccaatcgctcttttgattttggaaaaaa---aac**

**Fortunella_japonica_5** **ggttagaaaccctttattttttcactccaatcgctcttttgattttggaaaaaa---aac**

**Fortunella_japonica_6** **ggttagaaaccctttattttttcactccaatcgctcttttgattttggaaaaaa---aac**

**Fortunella_japonica_7** **ggttagaaaccctttattttttcactccaatcgctcttttgattttggaaaaaa---aac**

**Fortunella_japonica_8** **ggttagaaaccctttattttttcactccaatcgctcttttgattttggaaaaaa---aac**

**Fortunella_japonica_9** **ggttagaaaccctttattttttcactccaatcgctcttttgattttggaaaaaa---aac**

**Fortunella_japonica_10** **ggttagaaaccctttattttttcactccaatcgctcttttgattttggaaaaaa---aac**

**Fortunella_japonica_11** **ggttagaaaccctttattttttcactccaatcgctcttttgattttggaaaaaa---aac**

**Fortunella_japonica_12** **ggttagaaaccctttattttttcactccaatcgctcttttgattttggaaaaaa---aac**

**Fortunella_margarita_1** **ggttagaaaccctttattttttcactccaatcgctcttttgattttggaaaaaa---aac**

**Fortunella_margarita_2** **ggttagaaaccctttattttttcactccaatcgctcttttgattttggaaaaaa---aac**

**Fortunella_venosa_1** **ggttagaaaccctttattttttcactccaatcgctcttttgattttggaaaaaa---aac**

**Fortunella_venosa_2** **ggttagaaaccctttattttttcactccaatcgctcttttgattttggaaaaaa---aac**

**Fortunella_venosa_3** **ggttagaaaccctttattttttcactccaatcgctcttttgattttggaaaaaa---aac**

4090	4100	4110	4120	4130	4140

....|....|....|....|....|....|....|....|....|....|....|....|

**Atalantia_kwangtungens** **aactatatttatcaatatactgcttcttctacacattcagctacaatccataatagggac**

**Citrus_reticulata_1** **aactctatttatcaatatactgcttcttctacacattcagttacaacccataatagggac**

**Citrus_reticulata_2** **aactctatttatcaatatactgcttcttctacacattcagttacaacccataatagggac**

**Citrus_trifoliata_3** **aactatatttatcaatatactgcttcttctacacattcagttacaacccataatagggac**

**Citrus_aurantiifolia** **aactatatttatcaatatactgcttcttctacacattcagttacaacccataatagggac**

**Citrus_aurantium** **aactatatttatcaatatactgcttcttctacacattcagttacaacccataatagggac**

**Citrus_cavaleriei** **ga---tatttatcaatatactgcttcttctacacattcagttacaacccataatagggac**

**Citrus_hongheensis** **aactatatttatcaatatactgcttcttctacacattcagttacaacccataatagggac**

**Citrus_limon** **aactatatttatcaatatactgcttcttctacacattcagttacaacccataatagggac**

**Citrus_maxima** **aactatatttatcaatatactgcttcttctacacattcagttacaacccataatagggac**

**Citrus_medica** **aactatatttatcaatatactgcttcttctacacattcagttacaacccataatagggac**

**Citrus_polytrifolia** **aactatatttatcaatatactgcttcttctacacattcagttacaacccataatagggac**

**Citrus_reticulata** **aactctatttatcaatatactgcttcttctacacattcagttacaacccataatagggac**

**Citrus_sinensis** **aactatatttatcaatatactgcttcttctacacattcagttacaacccataatagggac**

**Fortunella_bawangica_1** **aactatatttatcaatatactgcttcttctacacattcagttacaacccataatagggac**

**Fortunella_bawangica_2** **aactatatttatcaatatactgcttcttctacacattcagttacaacccataatagggac**

**Fortunella_hindsii_1** **aactatatttatcaatatactgcttcttctacacattcagttacaacccataatagggac**

**Fortunella_hindsii_2** **aactatatttatcaatatactgcttcttctacacattcagttacaacccataatagggac**

**Fortunella_hindsii_3** **aactatatttatcaatatactgcttcttctacacattcagttacaacccataatagggac**

**Fortunella_japonica_1** **aactatatttatcaatatactgcttcttctacacattcagttacaacccataatagggac**

**Fortunella_japonica_2** **aactatatttatcaatatactgcttcttctacacattcagttacaacccataatagggac**

**Fortunella_japonica_3** **aactatatttatcaatatactgcttcttctacacattcagttacaacccataatagggac**

**Fortunella_japonica_4** **aactatatttatcaatatactgcttcttctacacattcagttacaacccataatagggac**

**Fortunella_japonica_5** **aactatatttatcaatatactgcttcttctacacattcagttacaacccataatagggac**

**Fortunella_japonica_6** **aactatatttatcaatatactgcttcttctacacattcagttacaacccataatagggac**

**Fortunella_japonica_7** **aactatatttatcaatatactgcttcttctacacattcagttacaacccataatagggac**

**Fortunella_japonica_8** **aactatatttatcaatatactgcttcttctacacattcagttacaacccataatagggac**

**Fortunella_japonica_9** **aactatatttatcaatatactgcttcttctacacattcagttacaacccataatagggac**

**Fortunella_japonica_10** **aactatatttatcaatatactgcttcttctacacattcagttacaacccataatagggac**

**Fortunella_japonica_11** **aactatatttatcaatatactgcttcttctacacattcagttacaacccataatagggac**

**Fortunella_japonica_12** **aactatatttatcaatatactgcttcttctacacattcagttacaacccataatagggac**

**Fortunella_margarita_1** **aactatatttatcaatatactgcttcttctacacattcagttacaacccataatagggac**

**Fortunella_margarita_2** **aactatatttatcaatatactgcttcttctacacattcagttacaacccataatagggac**

**Fortunella_venosa_1** **aactatatttatcaatatactgcttcttctacacattcagttacaacccataatagggac**

**Fortunella_venosa_2** **aactatatttatcaatatactgcttcttctacacattcagttacaacccataatagggac**

**Fortunella_venosa_3** **aactatatttatcaatatactgcttcttctacacattcagttacaacccataatagggac**

4150	4160	4170	4180	4190	4200

....|....|....|....|....|....|....|....|....|....|....|....|

**Atalantia_kwangtungens** **tcgctaatacttaggactcattaaataaatcaataatcccctcatgggaaaacctttccc**

**Citrus_reticulata_1** **ccgctaatacttaggactcattaaataaatcgataatcccctcatgggaaaacctttccc**

**Citrus_reticulata_2** **ccgctaatacttaggactcattaaataaatcgataatcccctcatgggaaaacctttccc**

**Citrus_trifoliata_3** **ccgctaatacttaggactcattaaataaatcgataatcccctcatgggaaaacctttccc**

**Citrus_aurantiifolia** **ccgctaatacttaggactcattaaataaatcgataatcccctcatgggaaaacctttccc**

**Citrus_aurantium** **ccgctaatacttaggactcattaaataaatcgataatcccctcatgggaaaacctttccc**

**Citrus_cavaleriei** **ccgctaatacttaggactcattaaataaatcgataatcccctcatgggaaaacctttccc**

**Citrus_hongheensis** **ccgctaatacttaggactcattaaataaatcgataatcccctcatgggaaaacctttccc**

**Citrus_limon** **ccgctaatacttaggactcattaaataaatcgataatcccctcatgggaaaacctttccc**

**Citrus_maxima** **ccgctaatacttaggactcattaaataaatcgataatcccctcatgggaaaacctttccc**

**Citrus_medica** **ccgctaatacttaggactcattaaataaatcgataatcccctcatgggaaaacctttccc**

**Citrus_polytrifolia** **ccgctaatacttaggactcattaaataaatcgataatcccctcatgggaaaacctttccc**

**Citrus_reticulata** **ccgctaatacttaggactcattaaataaatcgataatcccctcatgggaaaacctttccc**

**Citrus_sinensis** **ccgctaatacttaggactcattaaataaatcgataatcccctcatgggaaaacctttccc**

**Fortunella_bawangica_1** **ccgctaatacttaggactcattaaataaatcgataatcccctcatgggaaaacctttccc**

**Fortunella_bawangica_2** **ccgctaatacttaggactcattaaataaatcgataatcccctcatgggaaaacctttccc**

**Fortunella_hindsii_1** **ccgctaatacttaggactcattaaataaatcgataatcccctcatgggaaaacctttccc**

**Fortunella_hindsii_2** **ccgctaatacttaggactcattaaataaatcgataatcccctcatgggaaaacctttccc**

**Fortunella_hindsii_3** **ccgctaatacttaggactcattaaataaatcgataatcccctcatgggaaaacctttccc**

**Fortunella_japonica_1** **ccgctaatacttaggactcattaaataaatcgataatcccctcatgggaaaacctttccc**

**Fortunella_japonica_2** **ccgctaatacttaggactcattaaataaatcgataatcccctcatgggaaaacctttccc**

**Fortunella_japonica_3** **ccgctaatacttaggactcattaaataaatcgataatcccctcatgggaaaacctttccc**

**Fortunella_japonica_4** **ccgctaatacttaggactcattaaataaatcgataatcccctcatgggaaaacctttccc**

**Fortunella_japonica_5** **ccgctaatacttaggactcattaaataaatcgataatcccctcatgggaaaacctttccc**

**Fortunella_japonica_6** **ccgctaatacttaggactcattaaataaatcgataatcccctcatgggaaaacctttccc**

**Fortunella_japonica_7** **ccgctaatacttaggactcattaaataaatcgataatcccctcatgggaaaacctttccc**

**Fortunella_japonica_8** **ccgctaatacttaggactcattaaataaatcgataatcccctcatgggaaaacctttccc**

**Fortunella_japonica_9** **ccgctaatacttaggactcattaaataaatcgataatcccctcatgggaaaacctttccc**

**Fortunella_japonica_10** **ccgctaatacttaggactcattaaataaatcgataatcccctcatgggaaaacctttccc**

**Fortunella_japonica_11** **ccgctaatacttaggactcattaaataaatcgataatcccctcatgggaaaacctttccc**

**Fortunella_japonica_12** **ccgctaatacttaggactcattaaataaatcgataatcccctcatgggaaaacctttccc**

**Fortunella_margarita_1** **ccgctaatacttaggactcattaaataaatcgataatcccctcatgggaaaacctttccc**

**Fortunella_margarita_2** **ccgctaatacttaggactcattaaataaatcgataatcccctcatgggaaaacctttccc**

**Fortunella_venosa_1** **ccgctaatacttaggactcattaaataaatcgataatcccctcatgggaaaacctttccc**

**Fortunella_venosa_2** **ccgctaatacttaggactcattaaataaatcgataatcccctcatgggaaaacctttccc**

**Fortunella_venosa_3** **ccgctaatacttaggactcattaaataaatcgataatcccctcatgggaaaacctttccc**

4210	4220	4230	4240	4250	4260

....|....|....|....|....|....|....|....|....|....|....|....|

**Atalantia_kwangtungens** **cgcgttaggaactaatatctttttaacgtttaattagatcggataatcattcaaattaag**

**Citrus_reticulata_1** **cgcgttaggaactaatatctttttaacgtttaattagatcggataatcattcaaattaag**

**Citrus_reticulata_2** **cgcgttaggaactaatatctttttaacgtttaattagatcggataatcattcaaattaag**

**Citrus_trifoliata_3** **cgcgttaggaactaatatctttttaacgtttaattagatcggataatcattcaaattaag**

**Citrus_aurantiifolia** **cgcgttaggaactaatatctttttaacgtttaattagatcggataatcattcaaattaag**

**Citrus_aurantium** **cgcgttaggaactaatatctttttaacgtttaattagatcggataatcattcaaattaag**

**Citrus_cavaleriei** **cgcgttaggaactaatatctttttaacgtttaattagatcggataatcattcaaattaag**

**Citrus_hongheensis** **cgcgttaggaactaatatctttttaacgtttaattagatcggataatcattcaaattaag**

**Citrus_limon** **cgcgttaggaactaatatctttttaacgtttaattagatcggataatcattcaaattaag**

**Citrus_maxima** **cgcgttaggaactaatatctttttaacgtttaattagatcggataatcattcaaattaag**

**Citrus_medica** **cgcgttaggaactaatatctttttaacgtttaattagatcggataatcattcaaattaag**

**Citrus_polytrifolia** **cgcgttaggaactaatatctttttaacgtttaattagatcggataatcattcaaattaag**

**Citrus_reticulata** **cgcgttaggaactaatatctttttaacgtttaattagatcggataatcattcaaattaag**

**Citrus_sinensis** **cgcgttaggaactaatatctttttaacgtttaattagatcggataatcattcaaattaag**

**Fortunella_bawangica_1** **cgcgttaggaactaatatctttttaacgtttaattagatcggataatcattcaaattaag**

**Fortunella_bawangica_2** **cgcgttaggaactaatatctttttaacgtttaattagatcggataatcattcaaattaag**

**Fortunella_hindsii_1** **cgcgttaggaactaatatctttttaacgtttaattagatcggataatcattcaaattaag**

**Fortunella_hindsii_2** **cgcgttaggaactaatatctttttaacgtttaattagatcggataatcattcaaattaag**

**Fortunella_hindsii_3** **cgcgttaggaactaatatctttttaacgtttaattagatcggataatcattcaaattaag**

**Fortunella_japonica_1** **cgcgttaggaactaatatctttttaacgtttaattagatcggataatcattcaaattaag**

**Fortunella_japonica_2** **cgcgttaggaactaatatctttttaacgtttaattagatcggataatcattcaaattaag**

**Fortunella_japonica_3** **cgcgttaggaactaatatctttttaacgtttaattagatcggataatcattcaaattaag**

**Fortunella_japonica_4** **cgcgttaggaactaatatctttttaacgtttaattagatcggataatcattcaaattaag**

**Fortunella_japonica_5** **cgcgttaggaactaatatctttttaacgtttaattagatcggataatcattcaaattaag**

**Fortunella_japonica_6** **cgcgttaggaactaatatctttttaacgtttaattagatcggataatcattcaaattaag**

**Fortunella_japonica_7** **cgcgttaggaactaatatctttttaacgtttaattagatcggataatcattcaaattaag**

**Fortunella_japonica_8** **cgcgttaggaactaatatctttttaacgtttaattagatcggataatcattcaaattaag**

**Fortunella_japonica_9** **cgcgttaggaactaatatctttttaacgtttaattagatcggataatcattcaaattaag**

**Fortunella_japonica_10** **cgcgttaggaactaatatctttttaacgtttaattagatcggataatcattcaaattaag**

**Fortunella_japonica_11** **cgcgttaggaactaatatctttttaacgtttaattagatcggataatcattcaaattaag**

**Fortunella_japonica_12** **cgcgttaggaactaatatctttttaacgtttaattagatcggataatcattcaaattaag**

**Fortunella_margarita_1** **cgcgttaggaactaatatctttttaacgtttaattagatcggataatcattcaaattaag**

**Fortunella_margarita_2** **cgcgttaggaactaatatctttttaacgtttaattagatcggataatcattcaaattaag**

**Fortunella_venosa_1** **cgcgttaggaactaatatctttttaacgtttaattagatcggataatcattcaaattaag**

**Fortunella_venosa_2** **cgcgttaggaactaatatctttttaacgtttaattagatcggataatcattcaaattaag**

**Fortunella_venosa_3** **cgcgttaggaactaatatctttttaacgtttaattagatcggataatcattcaaattaag**

4270	4280	4290	4300	4310	4320

....|....|....|....|....|....|....|....|....|....|....|....|

**Atalantia_kwangtungens** **aaccgaagctcgttactttttgtttccctataattggaaccctagggctctgtccattta**

**Citrus_reticulata_1** **aaccgaagctcgttactttttgtttccctataattggaaccctcgggctctatccattta**

**Citrus_reticulata_2** **aaccgaagctcgttactttttgtttccctataattggaaccctcgggctctatccattta**

**Citrus_trifoliata_3** **aaccgaagctcgttactttttgtttccctataattggaaccctagggctctatccattta**

**Citrus_aurantiifolia** **aaccgaagctcgttactttttgtttccctataattggaaccctagggctctatccattta**

**Citrus_aurantium** **aaccgaagctcgttactttttgtttccctataattggaaccctagggctctatccattta**

**Citrus_cavaleriei** **aaccgaagctcgttactttttgtttccctataattggaaccctagggctctatccattta**

**Citrus_hongheensis** **aaccgaagctcgttactttttgtttccctataattggaaccctagggctctatccattta**

**Citrus_limon** **aaccgaagctcgttactttttgtttccctataattggaaccctagggctctatccattta**

**Citrus_maxima** **aaccgaagctcgttactttttgtttccctataattggaaccctagggctctatccattta**

**Citrus_medica** **aaccgaagctcgttactttttgtttccctataattggaaccctagggctctatccattta**

**Citrus_polytrifolia** **aaccgaagctcgttactttttgtttccctataattggaaccctagggctctatccattta**

**Citrus_reticulata** **aaccgaagctcgttactttttgtttccctataattggaaccctcgggctctatccattta**

**Citrus_sinensis** **aaccgaagctcgttactttttgtttccctataattggaaccctagggctctatccattta**

**Fortunella_bawangica_1** **aaccgaagctcgttactttttgtttccctataattggaaccctagggctctatccattta**

**Fortunella_bawangica_2** **aaccgaagctcgttactttttgtttccctataattggaaccctagggctctatccattta**

**Fortunella_hindsii_1** **aaccgaagctcgttactttttgtttccctataattggaaccctagggctctatccattta**

**Fortunella_hindsii_2** **aaccgaagctcgttactttttgtttccctataattggaaccctagggctctatccattta**

**Fortunella_hindsii_3** **aaccgaagctcgttactttttgtttccctataattggaaccctagggctctatccattta**

**Fortunella_japonica_1** **aaccgaagctcgttactttttgtttccctataattggaaccctagggctctatccattta**

**Fortunella_japonica_2** **aaccgaagctcgttactttttgtttccctataattggaaccctagggctctatccattta**

**Fortunella_japonica_3** **aaccgaagctcgttactttttgtttccctataattggaaccctagggctctatccattta**

**Fortunella_japonica_4** **aaccgaagctcgttactttttgtttccctataattggaaccctagggctctatccattta**

**Fortunella_japonica_5** **aaccgaagctcgttactttttgtttccctataattggaaccctagggctctatccattta**

**Fortunella_japonica_6** **aaccgaagctcgttactttttgtttccctataattggaaccctagggctctatccattta**

**Fortunella_japonica_7** **aaccgaagctcgttactttttgtttccctataattggaaccctagggctctatccattta**

**Fortunella_japonica_8** **aaccgaagctcgttactttttgtttccctataattggaaccctagggctctatccattta**

**Fortunella_japonica_9** **aaccgaagctcgttactttttgtttccctataattggaaccctagggctctatccattta**

**Fortunella_japonica_10** **aaccgaagctcgttactttttgtttccctataattggaaccctagggctctatccattta**

**Fortunella_japonica_11** **aaccgaagctcgttactttttgtttccctataattggaaccctagggctctatccattta**

**Fortunella_japonica_12** **aaccgaagctcgttactttttgtttccctataattggaaccctagggctctatccattta**

**Fortunella_margarita_1** **aaccgaagctcgttactttttgtttccctataattggaaccctagggctctatccattta**

**Fortunella_margarita_2** **aaccgaagctcgttactttttgtttccctataattggaaccctagggctctatccattta**

**Fortunella_venosa_1** **aaccgaagctcgttactttttgtttccctataattggaaccctagggctctatccattta**

**Fortunella_venosa_2** **aaccgaagctcgttactttttgtttccctataattggaaccctagggctctatccattta**

**Fortunella_venosa_3** **aaccgaagctcgttactttttgtttccctataattggaaccctagggctctatccattta**

4330	4340	4350	4360	4370	4380

....|....|....|....|....|....|....|....|....|....|....|....|

**Atalantia_kwangtungens** **ttcactcgacccaactcttaatta----aatatt-------aaattgattttgttccgcg**

**Citrus_reticulata_1** **ttcactcgacccaactctta--------ataatt-------atatttattttgttccgcg**

**Citrus_reticulata_2** **ttcactcgacccaactctta--------ataatt-------atatttattttgttccgcg**

**Citrus_trifoliata_3** **ttcactcgacccaactctta--------ataatt-------atatttattttgttccgca**

**Citrus_aurantiifolia** **ttcactcgacccaactcttaataattatataatt-------atatttattttgttccgcg**

**Citrus_aurantium** **ttaactcgacccaactctta--------ataattatatttaatatttattttgttccgcg**

**Citrus_cavaleriei** **ttcactcgacccaactctta--------ataatt-------atattgattttgttccgcg**

**Citrus_hongheensis** **ttaactcgacccaactctta--------ataatt-------atatttattttgttccgcg**

**Citrus_limon** **ttaactcgacccaactctta--------ataattatatttaatatttattttgttccgcg**

**Citrus_maxima** **ttaactcgacccaactctta--------ataatt-------atatttattttgttccgcg**

**Citrus_medica** **ttcactcgacccaactcttaatta----ttaatt-------atatttattttgttccgcg**

**Citrus_polytrifolia** **ttcactcgacccaactctta--------ataatt-------atatttattttgttccgcg**

**Citrus_reticulata** **ttcactcgacccaactctta--------ataatt-------atatttattttgttccgcg**

**Citrus_sinensis** **ttaactcgacccaactctta--------ataatt-------atatttattttgttccgcg**

**Fortunella_bawangica_1** **ttcactcgacccaactctta--------ataatt-------atatttattttgttccgcg**

**Fortunella_bawangica_2** **ttcactcgacccaactctta--------ataatt-------atatttattttgttccgcg**

**Fortunella_hindsii_1** **ttcactcgacccaactctta--------ataatt-------atatttattttgttccgcg**

**Fortunella_hindsii_2** **ttcactcgacccaactctta--------ataatt-------atatttattttgttccgcg**

**Fortunella_hindsii_3** **ttcactcgacccaactctta--------ataatt-------atatttattttgttccgcg**

**Fortunella_japonica_1** **ttcactcgacccaactctta--------ataatt-------atatttattttgttccgcg**

**Fortunella_japonica_2** **ttcactcgacccaactctta--------ataatt-------atatttattttgttccgcg**

**Fortunella_japonica_3** **ttcactcgacccaactctta--------ataatt-------atatttattttgttccgcg**

**Fortunella_japonica_4** **ttcactcgacccaactctta--------ataatt-------atatttattttgttccgcg**

**Fortunella_japonica_5** **ttcactcgacccaactctta--------ataatt-------atatttattttgttccgcg**

**Fortunella_japonica_6** **ttcactcgacccaactctta--------ataatt-------atatttattttgttccgcg**

**Fortunella_japonica_7** **ttcactcgacccaactctta--------ataatt-------atatttattttgttccgcg**

**Fortunella_japonica_8** **ttcactcgacccaactctta--------ataatt-------atatttattttgttccgcg**

**Fortunella_japonica_9** **ttcactcgacccaactctta--------ataatt-------atatttattttgttccgcg**

**Fortunella_japonica_10** **ttcactcgacccaactctta--------ataatt-------atatttattttgttccgcg**

**Fortunella_japonica_11** **ttcactcgacccaactctta--------ataatt-------atatttattttgttccgcg**

**Fortunella_japonica_12** **ttcactcgacccaactctta--------ataatt-------atatttattttgttccgcg**

**Fortunella_margarita_1** **ttcactcgacccaactctta--------ataatt-------atatttattttgttccgcg**

**Fortunella_margarita_2** **ttcactcgacccaactctta--------ataatt-------atatttattttgttccgcg**

**Fortunella_venosa_1** **ttcactcgacccaactctta--------ataatt-------atatttattttgttccgcg**

**Fortunella_venosa_2** **ttcactcgacccaactctta--------ataatt-------atatttattttgttccgcg**

**Fortunella_venosa_3** **ttcactcgacccaactctta--------ataatt-------atatttattttgttccgcg**

4390	4400	4410	4420	4430	4440

....|....|....|....|....|....|....|....|....|....|....|....|

**Atalantia_kwangtungens** **ccaagaattcaaacttggttttatagcgattgaacaagaataaaagattctcaaaattat**

**Citrus_reticulata_1** **ccaagaattcaaacttggttttatagcgattgaacaagaataaaatattctcaaaattat**

**Citrus_reticulata_2** **ccaagaattcaaacttggttttatagcgattgaacaagaataaaatattctcaaaattat**

**Citrus_trifoliata_3** **ccaagaattcaaacttggttttatagcgattgaacaagaataaactattctcaaaattat**

**Citrus_aurantiifolia** **ccaagaattcaaacttggttttatagcgattgaacaagaataaaatattctcaaaattat**

**Citrus_aurantium** **ccaagaattcaaacttggttttatagcgattgaacaagaataaaatattctcaaaattat**

**Citrus_cavaleriei** **ccaagaattcaaacttggttttatagcgattgaacaagaataaaatattctcaaaattat**

**Citrus_hongheensis** **ccaagaattcaaacttggttttatagcgattgaacaagaataaaatattctcaaaattat**

**Citrus_limon** **ccaagaattcaaacttggttttatagcgattgaacaagaataaaatattctcaaaattat**

**Citrus_maxima** **ccaagaattcaaacttggttttatagcgattgaacaagaataaaatattctcaaaattat**

**Citrus_medica** **ccaagaattcaaacttggttttatagcgattgaacaagaataaaatattctcaaaattat**

**Citrus_polytrifolia** **ccaagaattcaaacttggttttatagcgattgaacaagaataaactattctcaaaattat**

**Citrus_reticulata** **ccaagaattcaaacttggttttatagcgattgaacaagaataaaatattctcaaaattat**

**Citrus_sinensis** **ccaagaattcaaacttggttttatagcgattgaacaagaataaaatattctcaaaattat**

**Fortunella_bawangica_1** **ccaagaattcaaacttggttttataacgattgaacaagaataaaatattctaaaaattat**

**Fortunella_bawangica_2** **ccaagaattcaaacttggttttataacgattgaacaagaataaaatattctaaaaattat**

**Fortunella_hindsii_1** **ccaagaattcaaacttggttttatagcgattgaacaagaataaaatattctcaaaattat**

**Fortunella_hindsii_2** **ccaagaattcaaacttggttttatagcgattgaacaagaataaaatattctcaaaattat**

**Fortunella_hindsii_3** **ccaagaattcaaacttggttttatagcgattgaacaagaataaaatattctcaaaattat**

**Fortunella_japonica_1** **ccaagaattcaaacttggttttatagcgattgaacaagaataaaatattctcaaaattat**

**Fortunella_japonica_2** **ccaagaattcaaacttggttttatagcgattgaacaagaataaaatattctcaaaattat**

**Fortunella_japonica_3** **ccaagaattcaaacttggttttatagcgattgaacaagaataaaatattctcaaaattat**

**Fortunella_japonica_4** **ccaagaattcaaacttggttttatagcgattgaacaagaataaaatattctcaaaattat**

**Fortunella_japonica_5** **ccaagaattcaaacttggttttatagcgattgaacaagaataaaatattctcaaaattat**

**Fortunella_japonica_6** **ccaagaattcaaacttggttttatagcgattgaacaagaataaaatattctcaaaattat**

**Fortunella_japonica_7** **ccaagaattcaaacttggttttatagcgattgaacaagaataaaatattctcaaaattat**

**Fortunella_japonica_8** **ccaagaattcaaacttggttttatagcgattgaacaagaataaaatattctcaaaattat**

**Fortunella_japonica_9** **ccaagaattcaaacttggttttatagcgattgaacaagaataaaatattctcaaaattat**

**Fortunella_japonica_10** **ccaagaattcaaacttggttttataacgattgaacaagaataaaatattctaaaaattat**

**Fortunella_japonica_11** **ccaagaattcaaacttggttttatagcgattgaacaagaataaaatattctcaaaattat**

**Fortunella_japonica_12** **ccaagaattcaaacttggttttatagcgattgaacaagaataaaatattctcaaaattat**

**Fortunella_margarita_1** **ccaagaattcaaacttggttttataacgattgaacaagaataaaatattctaaaaattat**

**Fortunella_margarita_2** **ccaagaattcaaacttggttttataacgattgaacaagaataaaatattctaaaaattat**

**Fortunella_venosa_1** **ccaagaattcaaacttggttttatagcgattgaacaagaataaaatattctcaaaattat**

**Fortunella_venosa_2** **ccaagaattcaaacttggttttatagcgattgaacaagaataaaatattctcaaaattat**

**Fortunella_venosa_3** **ccaagaattcaaacttggttttatagcgattgaacaagaataaaatattctcaaaattat**

4450	4460	4470	4480	4490	4500

....|....|....|....|....|....|....|....|....|....|....|....|

**Atalantia_kwangtungens** **ccattgatacgacatgctgttttttccattcattcctttcaggatcagtcgcggtcttat**

**Citrus_reticulata_1** **ccattgatacgacatgctgttttttccattcattcctttcaggatcagtcgcagtcttac**

**Citrus_reticulata_2** **ccattgatacgacatgctgttttttccattcattcctttcaggatcagtcgcagtcttac**

**Citrus_trifoliata_3** **ccattgatacgacatgctgttttttccattcattcctttcaggatcagtcgcagtcttac**

**Citrus_aurantiifolia** **ccattgatacgacatgctgttttttccattcattcctttcaggatcagtcgcagtcttac**

**Citrus_aurantium** **ccattgatacgacatgctgttttttccattcattcctttcaggatcagtcgcagtcttac**

**Citrus_cavaleriei** **ccattgatacgacatgctgttttttccattcattcctttcaggatcagtcgcagtcttac**

**Citrus_hongheensis** **ccattgatacgacatgctgttttttccattcattcctttcaggatcagtcgcagtcttac**

**Citrus_limon** **ccattgatacgacatgctgttttttccattcattcctttcaggatcagtcgcagtcttac**

**Citrus_maxima** **ccattgatacgacatgctgttttttccattcattcctttcaggatcagtcgcagtcttac**

**Citrus_medica** **ccattgatacgacatactgttttttccattcattcctttcaggatcagtcgcagtcttac**

**Citrus_polytrifolia** **ccattgatacgacatgctgttttttccattcattcctttcaggatcagtcgcagtcttac**

**Citrus_reticulata** **ccattgatacgacatgctgttttttccattcattcctttcaggatcagtcgcagtcttac**

**Citrus_sinensis** **ccattgatacgacatgctgttttttccattcattcctttcaggatcagtcgcagtcttac**

**Fortunella_bawangica_1** **ccattgatacgacatgctgttttttccattcattcctttcaggatcagtcgcagtcttac**

**Fortunella_bawangica_2** **ccattgatacgacatgctgttttttccattcattcctttcaggatcagtcgcagtcttac**

**Fortunella_hindsii_1** **ccattgatacgacatgctgttttttccattcattcctttcaggatcagtcgcagtcttac**

**Fortunella_hindsii_2** **ccattgatacgacatgctgttttttccattcattcctttcaggatcagtcgcagtcttac**

**Fortunella_hindsii_3** **ccattgatacgacatgctgttttttccattcattcctttcaggatcagtcgcagtcttac**

**Fortunella_japonica_1** **ccattgatacgacatgctgttttttccattcattcctttcaggatcagtcgcagtcttac**

**Fortunella_japonica_2** **ccattgatacgacatgctgttttttccattcattcctttcaggatcagtcgcagtcttac**

**Fortunella_japonica_3** **ccattgatacgacatgctgttttttccattcattcctttcaggatcagtcgcagtcttac**

**Fortunella_japonica_4** **ccattgatacgacatgctgttttttccattcattcctttcaggatcagtcgcagtcttac**

**Fortunella_japonica_5** **ccattgatacgacatgctgttttttccattcattcctttcaggatcagtcgcagtcttac**

**Fortunella_japonica_6** **ccattgatacgacatgctgttttttccattcattcctttcaggatcagtcgcagtcttac**

**Fortunella_japonica_7** **ccattgatacgacatgctgttttttccattcattcctttcaggatcagtcgcagtcttac**

**Fortunella_japonica_8** **ccattgatacgacatgctgttttttccattcattcctttcaggatcagtcgcagtcttac**

**Fortunella_japonica_9** **ccattgatacgacatgctgttttttccattcattcctttcaggatcagtcgcagtcttac**

**Fortunella_japonica_10** **ccattgatacgacatgctgttttttccattcattcctttcaggatcagtcgcagtcttac**

**Fortunella_japonica_11** **ccattgatacgacatgctgttttttccattcattcctttcaggatcagtcgcagtcttac**

**Fortunella_japonica_12** **ccattgatacgacatgctgttttttccattcattcctttcaggatcagtcgcagtcttac**

**Fortunella_margarita_1** **ccattgatacgacatgctgttttttccattcattcctttcaggatcagtcgcagtcttac**

**Fortunella_margarita_2** **ccattgatacgacatgctgttttttccattcattcctttcaggatcagtcgcagtcttac**

**Fortunella_venosa_1** **ccattgatacgacatgctgttttttccattcattcctttcaggatcagtcgcagtcttac**

**Fortunella_venosa_2** **ccattgatacgacatgctgttttttccattcattcctttcaggatcagtcgcagtcttac**

**Fortunella_venosa_3** **ccattgatacgacatgctgttttttccattcattcctttcaggatcagtcgcagtcttac**

4510	4520	4530	4540	4550	4560

....|....|....|....|....|....|....|....|....|....|....|....|

**Atalantia_kwangtungens** **aaactctcccgaagatttgtacgaattctttgcttcatagaaatgtgtaaaagatgctag**

**Citrus_reticulata_1** **aaactctcccgaagatttggacgaattctttgcttcatagaaatgtgtaaaagatgctag**

**Citrus_reticulata_2** **aaactctcccgaagatttggacgaattctttgcttcatagaaatgtgtaaaagatgctag**

**Citrus_trifoliata_3** **aaactctcccgaagatttggacgaattctttgcttcatagaaatgtgtaaaagatgctag**

**Citrus_aurantiifolia** **aaactctcccgaagatttggacgaattctttgcttcatagaaatgtgtaaaagatgctag**

**Citrus_aurantium** **aaactctcccgaagatttggacgaattctttgcttcatagaaatgtgtaaaagatgctag**

**Citrus_cavaleriei** **aaactctcccgaagatttggacgaattctttgcttcatagaaatgtgtaaaagatgctag**

**Citrus_hongheensis** **aaactctcccgaagatttggacgaattctttgcttcatagaaatgtgtaaaagatgctag**

**Citrus_limon** **aaactctcccgaagatttggacgaattctttgcttcatagaaatgtgtaaaagatgctag**

**Citrus_maxima** **aaactctcccgaagatttggacgaattctttgcttcatagaaatgtgtaaaagatgctag**

**Citrus_medica** **aaactctcccgaagatttggacgaattctttgcttcatagaaatgtgtaaaagatgctag**

**Citrus_polytrifolia** **aaactctcccgaagatttggacgaattctttgcttcatagaaatgtgtaaaagatgctag**

**Citrus_reticulata** **aaactctcccgaagatttggacgaattctttgcttcatagaaatgtgtaaaagatgctag**

**Citrus_sinensis** **aaactctcccgaagatttggacgaattctttgcttcatagaaatgtgtaaaagatgctag**

**Fortunella_bawangica_1** **aaactctcccgaagatttggacgaattctttgcttcatagaaatgtgtaaaagatgctag**

**Fortunella_bawangica_2** **aaactctcccgaagatttggacgaattctttgcttcatagaaatgtgtaaaagatgctag**

**Fortunella_hindsii_1** **aaactctcccgaagatttggacgaattctttgcttcatagaaatgtgtaaaagatgctag**

**Fortunella_hindsii_2** **aaactctcccgaagatttggacgaattctttgcttcatagaaatgtgtaaaagatgctag**

**Fortunella_hindsii_3** **aaactctcccgaagatttggacgaattctttgcttcatagaaatgtgtaaaagatgctag**

**Fortunella_japonica_1** **aaactctcccgaagatttggacgaattctttgcttcatagaaatgtgtaaaagatgctag**

**Fortunella_japonica_2** **aaactctcccgaagatttggacgaattctttgcttcatagaaatgtgtaaaagatgctag**

**Fortunella_japonica_3** **aaactctcccgaagatttggacgaattctttgcttcatagaaatgtgtaaaagatgctag**

**Fortunella_japonica_4** **aaactctcccgaagatttggacgaattctttgcttcatagaaatgtgtaaaagatgctag**

**Fortunella_japonica_5** **aaactctcccgaagatttggacgaattctttgcttcatagaaatgtgtaaaagatgctag**

**Fortunella_japonica_6** **aaactctcccgaagatttggacgaattctttgcttcatagaaatgtgtaaaagatgctag**

**Fortunella_japonica_7** **aaactctcccgaagatttggacgaattctttgcttcatagaaatgtgtaaaagatgctag**

**Fortunella_japonica_8** **aaactctcccgaagatttggacgaattctttgcttcatagaaatgtgtaaaagatgctag**

**Fortunella_japonica_9** **aaactctcccgaagatttggacgaattctttgcttcatagaaatgtgtaaaagatgctag**

**Fortunella_japonica_10** **aaactctcccgaagatttggacgaattctttgcttcatagaaatgtgtaaaagatgctag**

**Fortunella_japonica_11** **aaactctcccgaagatttggacgaattctttgcttcatagaaatgtgtaaaagatgctag**

**Fortunella_japonica_12** **aaactctcccgaagatttggacgaattctttgcttcatagaaatgtgtaaaagatgctag**

**Fortunella_margarita_1** **aaactctcccgaagatttggacgaattctttgcttcatagaaatgtgtaaaagatgctag**

**Fortunella_margarita_2** **aaactctcccgaagatttggacgaattctttgcttcatagaaatgtgtaaaagatgctag**

**Fortunella_venosa_1** **aaactctcccgaagatttggacgaattctttgcttcatagaaatgtgtaaaagatgctag**

**Fortunella_venosa_2** **aaactctcccgaagatttggacgaattctttgcttcatagaaatgtgtaaaagatgctag**

**Fortunella_venosa_3** **aaactctcccgaagatttggacgaattctttgcttcatagaaatgtgtaaaagatgctag**

4570	4580	4590	4600	4610	4620

....|....|....|....|....|....|....|....|....|....|....|....|

**Atalantia_kwangtungens** **ccgtacttaaaagccgagtactctaccgttgagttagcaacccaaataatttgaatagat**

**Citrus_reticulata_1** **ccgtacttaaaagccgagtactctaccgttgagttagcaacccaaataattcgaatgggt**

**Citrus_reticulata_2** **ccgtacttaaaagccgagtactctaccgttgagttagcaacccaaataattcgaatgggt**

**Citrus_trifoliata_3** **ccgtacttaaaagccgagtactctaccgttgagttagcaacccaaataattcgaatgggt**

**Citrus_aurantiifolia** **ccgtacttaaaagccgagtactctaccgttgagttagcaacccaaataattcgaatgggt**

**Citrus_aurantium** **ccgtacttaaaagccgagtactctaccgttgagttagcaacccaaataattcgaatgggt**

**Citrus_cavaleriei** **ccgtacttaaaagccgagtactctaccgttgagttagcaacccaaataattcgaatgggt**

**Citrus_hongheensis** **ccgtacttaaaagccgagtactctaccgttgagttagcaacccaaataattcgaatgggt**

**Citrus_limon** **ccgtacttaaaagccgagtactctaccgttgagttagcaacccaaataattcgaatgggt**

**Citrus_maxima** **ccgtacttaaaagccgagtactctaccgttgagttagcaacccaaataattcgaatgggt**

**Citrus_medica** **ccgtacttaaaagccgagtactctaccgttgagttagcaacccaaataattcgaatgggt**

**Citrus_polytrifolia** **ccgtacttaaaagccgagtactctaccgttgagttagcaacccaaataattcgaatgggt**

**Citrus_reticulata** **ccgtacttaaaagccgagtactctaccgttgagttagcaacccaaataattcgaatgggt**

**Citrus_sinensis** **ccgtacttaaaagccgagtactctaccgttgagttagcaacccaaataattcgaatgggt**

**Fortunella_bawangica_1** **ccgtacttaaaagccgagtactctaccgttgagttagcaacccaaataattcgaatgggt**

**Fortunella_bawangica_2** **ccgtacttaaaagccgagtactctaccgttgagttagcaacccaaataattcgaatgggt**

**Fortunella_hindsii_1** **ccgtacttaaaagccgagtactctaccgttgagttagcaacccaaataattcgaatgggt**

**Fortunella_hindsii_2** **ccgtacttaaaagccgagtactctaccgttgagttagcaacccaaataattcgaatgggt**

**Fortunella_hindsii_3** **ccgtacttaaaagccgagtactctaccgttgagttagcaacccaaataattcgaatgggt**

**Fortunella_japonica_1** **ccgtacttaaaagccgagtactctaccgttgagttagcaacccaaataattcgaatgggt**

**Fortunella_japonica_2** **ccgtacttaaaagccgagtactctaccgttgagttagcaacccaaataattcgaatgggt**

**Fortunella_japonica_3** **ccgtacttaaaagccgagtactctaccgttgagttagcaacccaaataattcgaatgggt**

**Fortunella_japonica_4** **ccgtacttaaaagccgagtactctaccgttgagttagcaacccaaataattcgaatgggt**

**Fortunella_japonica_5** **ccgtacttaaaagccgagtactctaccgttgagttagcaacccaaataattcgaatgggt**

**Fortunella_japonica_6** **ccgtacttaaaagccgagtactctaccgttgagttagcaacccaaataattcgaatgggt**

**Fortunella_japonica_7** **ccgtacttaaaagccgagtactctaccgttgagttagcaacccaaataattcgaatgggt**

**Fortunella_japonica_8** **ccgtacttaaaagccgagtactctaccgttgagttagcaacccaaataattcgaatgggt**

**Fortunella_japonica_9** **ccgtacttaaaagccgagtactctaccgttgagttagcaacccaaataattcgaatgggt**

**Fortunella_japonica_10** **ccgtacttaaaagccgagtactctaccgttgagttagcaacccaaataattcgaatgggt**

**Fortunella_japonica_11** **ccgtacttaaaagccgagtactctaccgttgagttagcaacccaaataattcgaatgggt**

**Fortunella_japonica_12** **ccgtacttaaaagccgagtactctaccgttgagttagcaacccaaataattcgaatgggt**

**Fortunella_margarita_1** **ccgtacttaaaagccgagtactctaccgttgagttagcaacccaaataattcgaatgggt**

**Fortunella_margarita_2** **ccgtacttaaaagccgagtactctaccgttgagttagcaacccaaataattcgaatgggt**

**Fortunella_venosa_1** **ccgtacttaaaagccgagtactctaccgttgagttagcaacccaaataattcgaatgggt**

**Fortunella_venosa_2** **ccgtacttaaaagccgagtactctaccgttgagttagcaacccaaataattcgaatgggt**

**Fortunella_venosa_3** **ccgtacttaaaagccgagtactctaccgttgagttagcaacccaaataattcgaatgggt**

4630	4640	4650	4660	4670	4680

....|....|....|....|....|....|....|....|....|....|....|....|

**Atalantia_kwangtungens** **agatagaatcggaat--acaaataaat------aaaaaaaaaaatggaatttcccaacgg**

**Citrus_reticulata_1** **agatagaatcggaat--aaaaataaat------aaaaaaaaaaatggaatttcaaaacgg**

**Citrus_reticulata_2** **agatagaatcggaat--aaaaataaat------aaaaaaaaaaatggaatttcaaaacgg**

**Citrus_trifoliata_3** **agatagaatcggaat--aaaaataaataaaaaaaaaaaaaaaaatggaatttcaaaacgg**

**Citrus_aurantiifolia** **agatagaatcggaat--aaaaataaat-------aaaaaaaaaattgaatttcaaaacgg**

**Citrus_aurantium** **agatagaatcggaat--aaaaaaaaat-----aaaaaaaaaaaaaggaatttcaaaacgg**

**Citrus_cavaleriei** **agatagaatcggaat--aaaaataaat-------caaaaaaaaatggaatttcaaaacgg**

**Citrus_hongheensis** **agatagaatcggaataaaaaaaaaaat-----aaaaaaaaaaaaaggaatttcaaaacgg**

**Citrus_limon** **agatagaatcggaat--aaaaaaaaat-----aaaaaaaaaaaaaggaatttcaaaacgg**

**Citrus_maxima** **agatagaatcggaat--aaaaaaaaat-----aaaaaaaaaaaaaggaatttcaaaacgg**

**Citrus_medica** **agatagaatcggaat--aaaaataaat---taaaaaaaaaaaaatggaatttcaaaacgg**

**Citrus_polytrifolia** **agatagaatcggaat--aaaaataaat-aaaaaaaaaaaaaaaatggaatttcaaaacgg**

**Citrus_reticulata** **agatagaatcggaat--aaaaataaat------aaaaaaaaaaatggaatttcaaaacgg**

**Citrus_sinensis** **agatagaatcggaat--aaaaaaaaat------aaaaaaaaaaaaggaatttcaaaacgg**

**Fortunella_bawangica_1** **agatagaatcggaat-aaaaataaaat------aaaaaaaaaaattgaatttcaaaacgg**

**Fortunella_bawangica_2** **agatagaatcggaat-aaaaataaaat------aaaaaaaaaaattgaatttcaaaacgg**

**Fortunella_hindsii_1** **agatagaatcggaat-aaaaataaaat----aaaaaaaaaaaaattgaatttcaaaacgg**

**Fortunella_hindsii_2** **agatagaatcggaat-aaaaataaaat----aaaaaaaaaaaaattgaatttcaaaacgg**

**Fortunella_hindsii_3** **agatagaatcggaat-aaaaataaaat----aaaaaaaaaaaaattgaatttcaaaacgg**

**Fortunella_japonica_1** **agatagaatcggaat-aaaaataaaat--------aaaaaaaaattgaatttcaaaacgg**

**Fortunella_japonica_2** **agatagaatcggaat-aaaaataaaat-----aaaaaaaaaaaattgaatttcaaaacgg**

**Fortunella_japonica_3** **agatagaatcggaat-aaaaataaaat--------aaaaaaaaattgaatttcaaaacgg**

**Fortunella_japonica_4** **agatagaatcggaat-aaaaataaaat--------aaaaaaaaattgaatttcaaaacgg**

**Fortunella_japonica_5** **agatagaatcggaat-aaaaataaaat--------aaaaaaaaattgaatttcaaaacgg**

**Fortunella_japonica_6** **agatagaatcggaat-aaaaataaaat--------aaaaaaaaattgaatttcaaaacgg**

**Fortunella_japonica_7** **agatagaatcggaat-aaaaataaaat--------aaaaaaaaattgaatttcaaaacgg**

**Fortunella_japonica_8** **agatagaatcggaat-aaaaataaaat--------aaaaaaaaattgaatttcaaaacgg**

**Fortunella_japonica_9** **agatagaatcggaat-aaaaataaaat--------aaaaaaaaattgaatttcaaaacgg**

**Fortunella_japonica_10** **agatagaatcggaat-aaaaataaaat------aaaaaaaaaaattgaatttcaaaacgg**

**Fortunella_japonica_11** **agatagaatcggaat-aaaaataaaat-----a-aaaaaaaaaattgaatttcaaaacgg**

**Fortunella_japonica_12** **agatagaatcggaat-aaaaataaaat--------aaaaaaaaattgaatttcaaaacgg**

**Fortunella_margarita_1** **agatagaatcggaat-aaaaataaaat------aaaaaaaaaaattgaatttcaaaacgg**

**Fortunella_margarita_2** **agatagaatcggaat-aaaaataaaat------aaaaaaaaaaattgaatttcaaaacgg**

**Fortunella_venosa_1** **agatagaatcggaat-aaaaataaaat-----aaaaaaaaaaaattgaatttcaaaacgg**

**Fortunella_venosa_2** **agatagaatcggaat-aaaaataaaat------aaaaaaaaaaattgaatttcaaaacgg**

**Fortunella_venosa_3** **agatagaatcggaat-aaaaataaaat------aaaaaaaaaaattgaatttcaaaacgg**

4690	4700	4710	4720	4730	4740

....|....|....|....|....|....|....|....|....|....|....|....|

**Atalantia_kwangtungens** **aatcaaaaaatgaaattagcacgaactcgaatcaaaaaaatttctaattgattctgaaca**

**Citrus_reticulata_1** **aatcaaaaaatgaaattagcaagaactcgaatcaaaaaaatttctaatcgattttgaaca**

**Citrus_reticulata_2** **aatcaaaaaatgaaattagcaagaactcgaatcaaaaaaatttctaatcgattttgaaca**

**Citrus_trifoliata_3** **aatcaaaaaatgaaattagcaagaactcgaatcaaaaaaatttctaatcgattttgaaca**

**Citrus_aurantiifolia** **aatcaaaaaatgaaattagcaagaactcgaatcaaaaaaatttctaatcgattttgaaca**

**Citrus_aurantium** **aatcaaaaaatgaaattagcaagaactcgaatcaaaaaaatttctaatcgattttgaaca**

**Citrus_cavaleriei** **aatcaaaaaatgaaattagcaagaactcgaatcaaaaaaatttctaatcgattttgaaca**

**Citrus_hongheensis** **aatcaaaaaatgaaattagcaagaactcgaatcaaaaaaatttctaatcgattttgaaca**

**Citrus_limon** **aatcaaaaaatgaaattagcaagaactcgaatcaaaaaaatttctaatcgattttgaaca**

**Citrus_maxima** **aatcaaaaaatgaaattagcaagaactcgaatcaaaaaaatttctaatcgattttgaaca**

**Citrus_medica** **aatcaaaaaatgaaattagcaagaactcgaatcaaaaaaatttctaatcgattttgaaca**

**Citrus_polytrifolia** **aatcaaaaaatgaaattagcaagaactcgaatcaaaaaaatttctaatcgattttgaaca**

**Citrus_reticulata** **aatcaaaaaatgaaattagcaagaactcgaatcaaaaaaatttctaatcgattttgaaca**

**Citrus_sinensis** **aatcaaaaaatgaaattagcaagaactcgaatcaaaaaaatttctaatcgattttgaaca**

**Fortunella_bawangica_1** **aatcaaaaaattaaattagcaagaactcgaatcaaaaaaatttctaatcgattttgaaca**

**Fortunella_bawangica_2** **aatcaaaaaattaaattagcaagaactcgaatcaaaaaaatttctaatcgattttgaaca**

**Fortunella_hindsii_1** **aatcaaaaaattaaattagcaagaactcgaatcaaaaaaatttctaatcgattttgaaca**

**Fortunella_hindsii_2** **aatcaaaaaattaaattagcaagaactcgaatcaaaaaaatttctaatcgattttgaaca**

**Fortunella_hindsii_3** **aatcaaaaaattaaattagcaagaactcgaatcaaaaaaatttctaatcgattttgaaca**

**Fortunella_japonica_1** **aatcaaaaaattaaattagcaagaactcgaatcaaaaaaatttctaatcgattttgaaca**

**Fortunella_japonica_2** **aatcaaaaaattaaattagcaagaactcgaatcaaaaaaatttctaatcgattttgaaca**

**Fortunella_japonica_3** **aatcaaaaaattaaattagcaagaactcgaatcaaaaaaatttctaatcgattttgaaca**

**Fortunella_japonica_4** **aatcaaaaaattaaattagcaagaactcgaatcaaaaaaatttctaatcgattttgaaca**

**Fortunella_japonica_5** **aatcaaaaaattaaattagcaagaactcgaatcaaaaaaatttctaatcgattttgaaca**

**Fortunella_japonica_6** **aatcaaaaaattaaattagcaagaactcgaatcaaaaaaatttctaatcgattttgaaca**

**Fortunella_japonica_7** **aatcaaaaaattaaattagcaagaactcgaatcaaaaaaatttctaatcgattttgaaca**

**Fortunella_japonica_8** **aatcaaaaaattaaattagcaagaactcgaatcaaaaaaatttctaatcgattttgaaca**

**Fortunella_japonica_9** **aatcaaaaaattaaattagcaagaactcgaatcaaaaaaatttctaatcgattttgaaca**

**Fortunella_japonica_10** **aatcaaaaaattaaattagcaagaactcgaatcaaaaaaatttctaatcgattttgaaca**

**Fortunella_japonica_11** **aatcaaaaaattaaattagcaagaactcgaatcaaaaaaatttctaatcgattttgaaca**

**Fortunella_japonica_12** **aatcaaaaaattaaattagcaagaactcgaatcaaaaaaatttctaatcgattttgaaca**

**Fortunella_margarita_1** **aatcaaaaaattaaattagcaagaactcgaatcaaaaaaatttctaatcgattttgaaca**

**Fortunella_margarita_2** **aatcaaaaaattaaattagcaagaactcgaatcaaaaaaatttctaatcgattttgaaca**

**Fortunella_venosa_1** **aatcaaaaaattaaattagcaagaactcgaatcaaaaaaatttctaatcgattttgaaca**

**Fortunella_venosa_2** **aatcaaaaaattaaattagcaagaactcgaatcaaaaaaatttctaatcgattttgaaca**

**Fortunella_venosa_3** **aatcaaaaaattaaattagcaagaactcgaatcaaaaaaatttctaatcgattttgaaca**

4750	4760	4770	4780	4790	4800

....|....|....|....|....|....|....|....|....|....|....|....|

**Atalantia_kwangtungens** **taaaaaaaagacaaccaaaacctatggaacggagctaaatgggcccatttctccatgatc**

**Citrus_reticulata_1** **taaaaaaaagacaaccaaaacctatggaacggagataaatgggcccagttctccatgatc**

**Citrus_reticulata_2** **taaaaaaaagacaaccaaaacctatggaacggagataaatgggcccagttctccatgatc**

**Citrus_trifoliata_3** **taaaaaaaagacaaccaaaacctatggaacggagataaatgggcccatttctccatgatc**

**Citrus_aurantiifolia** **taaaaaaaagacaaccaaaacctatggaacggagataaatgggtccatttctccatgatc**

**Citrus_aurantium** **taaaaaaaagacaaccaaaacctatggaacggagataaatgggcccatttctccatgaga**

**Citrus_cavaleriei** **taaaaaaaagacaaccaaaacctatggaacggagataaatgggcccagttctccatgatc**

**Citrus_hongheensis** **taaaaaaaagacaaccaaaacctatggaacggagataaatgggcccatttctccatgaga**

**Citrus_limon** **taaaaaaaagacaaccaaaacctatggaacggagataaatgggcccatttctccatgaga**

**Citrus_maxima** **taaaaaaaagacaaccaaaacctatggaacggagataaatgggcccatttctccatgaga**

**Citrus_medica** **taaaaaaaagacaaccaaaacctatggaacggagataaatgggcccatttctccatgatc**

**Citrus_polytrifolia** **taaaaaaaagacaaccaaaacctatggaacggagataaatgggcccatttctccatgatc**

**Citrus_reticulata** **taaaaaaaagacaaccaaaacctatggaacggagataaatgggcccagttctccatgatc**

**Citrus_sinensis** **taaaaaaaagacaaccaaaacctatggaacggagataaatgggcccatttctccatgaga**

**Fortunella_bawangica_1** **taaaaaaaagacaaccaaaacctatggaacggagataaatgggcccatttctccatgatc**

**Fortunella_bawangica_2** **taaaaaaaagacaaccaaaacctatggaacggagataaatgggcccatttctccatgatc**

**Fortunella_hindsii_1** **taaaaaaaagacaaccaaaacctatggaacggagataaatgggcccatttctccatgatc**

**Fortunella_hindsii_2** **taaaaaaaagacaaccaaaacctatggaacggagataaatgggcccatttctccatgatc**

**Fortunella_hindsii_3** **taaaaaaaagacaaccaaaacctatggaacggagataaatgggcccatttctccatgatc**

**Fortunella_japonica_1** **taaaaaaaagacaaccaaaacctatggaacggagataaatgggcccatttctccatgatc**

**Fortunella_japonica_2** **taaaaaaaagacaaccaaaacctatggaacggagataaatgggcccatttctccatgatc**

**Fortunella_japonica_3** **taaaaaaaagacaaccaaaacctatggaacggagataaatgggcccatttctccatgatc**

**Fortunella_japonica_4** **taaaaaaaagacaaccaaaacctatggaacggagataaatgggcccatttctccatgatc**

**Fortunella_japonica_5** **taaaaaaaagacaaccaaaacctatggaacggagataaatgggcccatttctccatgatc**

**Fortunella_japonica_6** **taaaaaaaagacaaccaaaacctatggaacggagataaatgggcccatttctccatgatc**

**Fortunella_japonica_7** **taaaaaaaagacaaccaaaacctatggaacggagataaatgggcccatttctccatgatc**

**Fortunella_japonica_8** **taaaaaaaagacaaccaaaacctatggaacggagataaatgggcccatttctccatgatc**

**Fortunella_japonica_9** **taaaaaaaagacaaccaaaacctatggaacggagataaatgggcccatttctccatgatc**

**Fortunella_japonica_10** **taaaaaaaagacaaccaaaacctatggaacggagataaatgggcccatttctccatgatc**

**Fortunella_japonica_11** **taaaaaaaagacaaccaaaacctatggaacggagataaatgggcccatttctccatgatc**

**Fortunella_japonica_12** **taaaaaaaagacaaccaaaacctatggaacggagataaatgggcccatttctccatgatc**

**Fortunella_margarita_1** **taaaaaaaagacaaccaaaacctatggaacggagataaatgggcccatttctccatgatc**

**Fortunella_margarita_2** **taaaaaaaagacaaccaaaacctatggaacggagataaatgggcccatttctccatgatc**

**Fortunella_venosa_1** **taaaaaaaagacaaccaaaacctatggaacggagataaatgggcccatttctccatgatc**

**Fortunella_venosa_2** **taaaaaaaagacaaccaaaacctatggaacggagataaatgggcccatttctccatgatc**

**Fortunella_venosa_3** **taaaaaaaagacaaccaaaacctatggaacggagataaatgggcccatttctccatgatc**

4810	4820	4830	4840	4850	4860

....|....|....|....|....|....|....|....|....|....|....|....|

**Atalantia_kwangtungens** **aaattatatttgatcaatacaatattgtcaatatgacattgaatattgaatagcaagata**

**Citrus_reticulata_1** **aaattatatttgttcactacaatattgtcaatatgacattgaatattga-----------**

**Citrus_reticulata_2** **aaattatatttgttcactacaatattgtcaatatgacattgaatattga-----------**

**Citrus_trifoliata_3** **aaattatatttgttcactacaatattgtcaatattacattgaatattga-----------**

**Citrus_aurantiifolia** **aaattatatttgttcactacaatattgtcaatatgacattgaatattga-----------**

**Citrus_aurantium** **aaattatatttgttcactacaatattgtcaatatgacattgaatattga-----------**

**Citrus_cavaleriei** **aaattatatttgttcactacaatattgtcaatatgacattgaatattga-----------**

**Citrus_hongheensis** **aaattatatttgttcactacaatattgtcaatatgacattgaatattga-----------**

**Citrus_limon** **aaattatatttgttcactacaatattgtcaatatgacattgaatattga-----------**

**Citrus_maxima** **aaattatatttgttcactacaatattgtcaatatgacattgaatattga-----------**

**Citrus_medica** **caattatatttgttcactacaatattgtcaatatgacattgaatattgaata--------**

**Citrus_polytrifolia** **aaattatatttgttcactacaatattgtcaatattacattgaatattga-----------**

**Citrus_reticulata** **aaattatatttgttcactacaatattgtcaatatgacattgaatattga-----------**

**Citrus_sinensis** **aaattatatttgttcactacaatattgtcaatatgacattgaatattga-----------**

**Fortunella_bawangica_1** **aaattatatttgttcactacaatattgtcaatatgacattgaatattga-----------**

**Fortunella_bawangica_2** **aaattatatttgttcactacaatattgtcaatatgacattgaatattga-----------**

**Fortunella_hindsii_1** **aaattatatttgttcactacaatattgtcaatatgacattgaatattga-----------**

**Fortunella_hindsii_2** **aaattatatttgttcactacaatattgtcaatatgacattgaatattga-----------**

**Fortunella_hindsii_3** **aaattatatttgttcactacaatattgtcaatatgacattgaatattga-----------**

**Fortunella_japonica_1** **aaattatatttgttcactacaatattgtcaatatgacattgaatattga-----------**

**Fortunella_japonica_2** **aaattatatttgttcactacaatattgtcaatatgacattgaatattga-----------**

**Fortunella_japonica_3** **aaattatatttgttcactacaatattgtcaatatgacattgaatattga-----------**

**Fortunella_japonica_4** **aaattatatttgttcactacaatattgtcaatatgacattgaatattga-----------**

**Fortunella_japonica_5** **aaattatatttgttcactacaatattgtcaatatgacattgaatattga-----------**

**Fortunella_japonica_6** **aaattatatttgttcactacaatattgtcaatatgacattgaatattga-----------**

**Fortunella_japonica_7** **aaattatatttgttcactacaatattgtcaatatgacattgaatattga-----------**

**Fortunella_japonica_8** **aaattatatttgttcactacaatattgtcaatatgacattgaatattga-----------**

**Fortunella_japonica_9** **aaattatatttgttcactacaatattgtcaatatgacattgaatattga-----------**

**Fortunella_japonica_10** **aaattatatttgttcactacaatattgtcaatatgacattgaatattga-----------**

**Fortunella_japonica_11** **aaattatatttgttcactacaatattgtcaatatgacattgaatattga-----------**

**Fortunella_japonica_12** **aaattatatttgttcactacaatattgtcaatatgacattgaatattga-----------**

**Fortunella_margarita_1** **aaattatatttgttcactacaatattgtcaatatgacattgaatattga-----------**

**Fortunella_margarita_2** **aaattatatttgttcactacaatattgtcaatatgacattgaatattga-----------**

**Fortunella_venosa_1** **aaattatatttgttcactacaatattgtcaatatgacattgaatattga-----------**

**Fortunella_venosa_2** **aaattatatttgttcactacaatattgtcaatatgacattgaatattga-----------**

**Fortunella_venosa_3** **aaattatatttgttcactacaatattgtcaatatgacattgaatattga-----------**

4870	4880	4890	4900	4910	4920

....|....|....|....|....|....|....|....|....|....|....|....|

**Atalantia_kwangtungens** **ttgaatagcaagattgagaaaatactaaaaaaatacaataac------------------**

**Citrus_reticulata_1** **----atagcaagattgagaaaatactaaaaacatacaataac------------------**

**Citrus_reticulata_2** **----atagcaagattgagaaaatactaaaaacatacaataac------------------**

**Citrus_trifoliata_3** **----atagcaagattgagaaaatactaaaaacatacaataac------------------**

**Citrus_aurantiifolia** **----atagcaagattgagaaaatactaaaaacatacaataac------------------**

**Citrus_aurantium** **----atagcaagattgagaaaatactaaaaacatacaataac------------------**

**Citrus_cavaleriei** **----atagcaagattgagaaaatactaaaaacatacaataac------------------**

**Citrus_hongheensis** **----atagc-----------aatactaaaaacatacaataacaaaaa----------caa**

**Citrus_limon** **----atagcaagattgagaaaatactaaaaacatacaataac------------------**

**Citrus_maxima** **----atagcaagattgagaaaatactaaaaacatacaataac------------------**

**Citrus_medica** **ttgaatagcaagattgagaaaatactaaaaacatacaataacaaaaaaacatacaataac**

**Citrus_polytrifolia** **----atagcaagattgagaaaatactaaaaacatacaataac------------------**

**Citrus_reticulata** **----atagcaagattgagaaaatactaaaaacatacaataac------------------**

**Citrus_sinensis** **----atagcaagattgagaaaatactaaaaacatacaataac------------------**

**Fortunella_bawangica_1** **----atagcaagattgagaaaatactaaaaacatacaataac------------------**

**Fortunella_bawangica_2** **----atagcaagattgagaaaatactaaaaacatacaataac------------------**

**Fortunella_hindsii_1** **----atagcaagattgagaaaatactaaaaacatacaataac------------------**

**Fortunella_hindsii_2** **----atagcaagattgagaaaatactaaaaacatacaataac------------------**

**Fortunella_hindsii_3** **----atagcaagattgagaaaatactaaaaacatacaataac------------------**

**Fortunella_japonica_1** **----atagcaagattgagaaaatactaaaaacatacaataac------------------**

**Fortunella_japonica_2** **----atagcaagattgagaaaatactaaaaacatacaataac------------------**

**Fortunella_japonica_3** **----atagcaagattgagaaaatactaaaaacatacaataac------------------**

**Fortunella_japonica_4** **----atagcaagattgagaaaatactaaaaacatacaataac------------------**

**Fortunella_japonica_5** **----atagcaagattgagaaaatactaaaaacatacaataac------------------**

**Fortunella_japonica_6** **----atagcaagattgagaaaatactaaaaacatacaataac------------------**

**Fortunella_japonica_7** **----atagcaagattgagaaaatactaaaaacatacaataac------------------**

**Fortunella_japonica_8** **----atagcaagattgagaaaatactaaaaacatacaataac------------------**

**Fortunella_japonica_9** **----atagcaagattgagaaaatactaaaaacatacaataac------------------**

**Fortunella_japonica_10** **----atagcaagattgagaaaatactaaaaacatacaataac------------------**

**Fortunella_japonica_11** **----atagcaagattgagaaaatactaaaaacatacaataac------------------**

**Fortunella_japonica_12** **----atagcaagattgagaaaatactaaaaacatacaataac------------------**

**Fortunella_margarita_1** **----atagcaagattgagaaaatactaaaaacatacaataac------------------**

**Fortunella_margarita_2** **----atagcaagattgagaaaatactaaaaacatacaataac------------------**

**Fortunella_venosa_1** **----atagcaagattgagaaaatactaaaaacatacaataac------------------**

**Fortunella_venosa_2** **----atagcaagattgagaaaatactaaaaacatacaataac------------------**

**Fortunella_venosa_3** **----atagcaagattgagaaaatactaaaaacatacaataac------------------**

4930	4940	4950	4960	4970	4980

....|....|....|....|....|....|....|....|....|....|....|....|

**Atalantia_kwangtungens** **aaaaacaaaaagagttaattggttatttattaaattgaattcaaatccaaataacaaata**

**Citrus_reticulata_1** **aaaaacaaaaagagttaattggttatttattaaattgaattcaaatccaaataacaaatc**

**Citrus_reticulata_2** **aaaaacaaaaagagttaattggttatttattaaattgaattcaaatccaaataacaaatc**

**Citrus_trifoliata_3** **aaaaacaaaaagagttaattggttatttattaaattgaattcaaatccaaataacaaatc**

**Citrus_aurantiifolia** **aaaaacaaaaagagttaattggttatttattaaattgaattcaaatccaaataacaaatc**

**Citrus_aurantium** **aaaaacaaaaagagttaattggttatttattaaattgaattcaaatccaaataacaaatc**

**Citrus_cavaleriei** **aaaaacaaaaagagttaattggttatttattaaattgaattcaaatccaaataacaaatc**

**Citrus_hongheensis** **aaaaacaaaaagagttaattggttatttattaaattgaattcaaatccaaataacaaatc**

**Citrus_limon** **aaaaacaaaaagagttaattggttatttattaaattgaattcaaatccaaataacaaatc**

**Citrus_maxima** **aaaaacaaaaagagttaattggttatttattaaattgaattcaaatccaaataacaaatc**

**Citrus_medica** **aaaaaccaaaagagttaattggttatttattaaattgaattcaaatccaaataacaaatc**

**Citrus_polytrifolia** **aaaaacaaaaagagttaattggttatttattaaattgaattcaaatccaaataacaaatc**

**Citrus_reticulata** **aaaaacaaaaagagttaattggttatttattaaattgaattcaaatccaaataacaaatc**

**Citrus_sinensis** **aaaaacaaaaagagttaattggttatttattaaattgaattcaaatccaaataacaaatc**

**Fortunella_bawangica_1** **aaaaacaaaaagagttaattggttatttattaaattgaattcaaatccaaataacaaatc**

**Fortunella_bawangica_2** **aaaaacaaaaagagttaattggttatttattaaattgaattcaaatccaaataacaaatc**

**Fortunella_hindsii_1** **aaaaacaaaaagagttaattggttatttattaaattgaattcaaatccaaataacaaatc**

**Fortunella_hindsii_2** **aaaaacaaaaagagttaattggttatttattaaattgaattcaaatccaaataacaaatc**

**Fortunella_hindsii_3** **aaaaacaaaaagagttaattggttatttattaaattgaattcaaatccaaataacaaatc**

**Fortunella_japonica_1** **aaaaacaaaaagagttaattggttatttattaaattgaattcaaatccaaataacaaatc**

**Fortunella_japonica_2** **aaaaacaaaaagagttaattggttatttattaaattgaattcaaatccaaataacaaatc**

**Fortunella_japonica_3** **aaaaacaaaaagagttaattggttatttattaaattgaattcaaatccaaataacaaatc**

**Fortunella_japonica_4** **aaaaacaaaaagagttaattggttatttattaaattgaattcaaatccaaataacaaatc**

**Fortunella_japonica_5** **aaaaacaaaaagagttaattggttatttattaaattgaattcaaatccaaataacaaatc**

**Fortunella_japonica_6** **aaaaacaaaaagagttaattggttatttattaaattgaattcaaatccaaataacaaatc**

**Fortunella_japonica_7** **aaaaacaaaaagagttaattggttatttattaaattgaattcaaatccaaataacaaatc**

**Fortunella_japonica_8** **aaaaacaaaaagagttaattggttatttattaaattgaattcaaatccaaataacaaatc**

**Fortunella_japonica_9** **aaaaacaaaaagagttaattggttatttattaaattgaattcaaatccaaataacaaatc**

**Fortunella_japonica_10** **aaaaacaaaaagagttaattggttatttattaaattgaattcaaatccaaataacaaatc**

**Fortunella_japonica_11** **aaaaacaaaaagagttaattggttatttattaaattgaattcaaatccaaataacaaatc**

**Fortunella_japonica_12** **aaaaacaaaaagagttaattggttatttattaaattgaattcaaatccaaataacaaatc**

**Fortunella_margarita_1** **aaaaacaaaaagagttaattggttatttattaaattgaattcaaatccaaataacaaatc**

**Fortunella_margarita_2** **aaaaacaaaaagagttaattggttatttattaaattgaattcaaatccaaataacaaatc**

**Fortunella_venosa_1** **aaaaacaaaaagagttaattggttatttattaaattgaattcaaatccaaataacaaatc**

**Fortunella_venosa_2** **aaaaacaaaaagagttaattggttatttattaaattgaattcaaatccaaataacaaatc**

**Fortunella_venosa_3** **aaaaacaaaaagagttaattggttatttattaaattgaattcaaatccaaataacaaatc**

4990	5000	5010	5020	5030	5040

....|....|....|....|....|....|....|....|....|....|....|....|

**Atalantia_kwangtungens** **aaattatatatatattaataaata-gataattat--atattaataaata-----------**

**Citrus_reticulata_1** **aaattatatattaataaatagatataataaatatggaaattaataaataatatctaaaga**

**Citrus_reticulata_2** **aaattatatattaataaatagatataataaatatggaaattaataaataatatctaaaga**

**Citrus_trifoliata_3** **aaattatatattaataaatagatataataaatatggaaattaataaataatatctaaaga**

**Citrus_aurantiifolia** **aaattatatattaataaatagatataataaatatggaaattaataaataatatctaaaga**

**Citrus_aurantium** **aaattatatattaataaatagatataataaatatggaaattaataaataatatctaaaga**

**Citrus_cavaleriei** **aaattatatattaataaatagatataataaatatggaaattaataaataatatctaaaga**

**Citrus_hongheensis** **aaattatatattaataaatagatataataaatatggaaattaataaataatatctaaaga**

**Citrus_limon** **aaattatatattaataaatagatataataaatatggaaattaataaataatatctaaaga**

**Citrus_maxima** **aaattatatattaataaatagatataataaatatggaaattaataaataatatctaaaga**

**Citrus_medica** **aaattttatattaataaatagatataataaatgtggaaattaataaataatatctaaaga**

**Citrus_polytrifolia** **aaattatatattaataaatagatataataaatatggaaattaataaataatatctaaaga**

**Citrus_reticulata** **aaattatatattaataaatagatataataaatatggaaattaataaataatatctaaaga**

**Citrus_sinensis** **aaattatatattaataaatagatataataaatatggaaattaataaataatatctaaaga**

**Fortunella_bawangica_1** **aaattatatattaataaatagatataataaatatggaaattaagaaataatatctaaaga**

**Fortunella_bawangica_2** **aaattatatattaataaatagatataataaatatggaaattaagaaataatatctaaaga**

**Fortunella_hindsii_1** **aaattatatattaataaatagatataataaatatggaaattaagaaataatatctaaaga**

**Fortunella_hindsii_2** **aaattatatattaataaatagatataataaatatggaaattaagaaataatatctaaaga**

**Fortunella_hindsii_3** **aaattatatattaataaatagatataataaatatggaaattaagaaataatatctaaaga**

**Fortunella_japonica_1** **aaattatatattaataaatagatataataaatatggaaattaagaaataatatctaaaga**

**Fortunella_japonica_2** **aaattatatattaataaatagatataataaatatggaaattaagaaataatatctaaaga**

**Fortunella_japonica_3** **aaattatatattaataaatagatataataaatatggaaattaagaaataatatctaaaga**

**Fortunella_japonica_4** **aaattatatattaataaatagatataataaatatggaaattaagaaataatatctaaaga**

**Fortunella_japonica_5** **aaattatatattaataaatagatataataaatatggaaattaagaaataatatctaaaga**

**Fortunella_japonica_6** **aaattatatattaataaatagatataataaatatggaaattaagaaataatatctaaaga**

**Fortunella_japonica_7** **aaattatatattaataaatagatataataaatatggaaattaagaaataatatctaaaga**

**Fortunella_japonica_8** **aaattatatattaataaatagatataataaatatggaaattaagaaataatatctaaaga**

**Fortunella_japonica_9** **aaattatatattaataaatagatataataaatatggaaattaagaaataatatctaaaga**

**Fortunella_japonica_10** **aaattatatattaataaatagatataataaatatggaaattaagaaataatatctaaaga**

**Fortunella_japonica_11** **aaattatatattaataaatagatataataaatatggaaattaagaaataatatctaaaga**

**Fortunella_japonica_12** **aaattatatattaataaatagatataataaatatggaaattaagaaataatatctaaaga**

**Fortunella_margarita_1** **aaattatatattaataaatagatataataaatatggaaattaagaaataatatctaaaga**

**Fortunella_margarita_2** **aaattatatattaataaatagatataataaatatggaaattaagaaataatatctaaaga**

**Fortunella_venosa_1** **aaattatatattaataaatagatataataaatatggaaattaagaaataatatctaaaga**

**Fortunella_venosa_2** **aaattatatattaataaatagatataataaatatggaaattaagaaataatatctaaaga**

**Fortunella_venosa_3** **aaattatatattaataaatagatataataaatatggaaattaagaaataatatctaaaga**

5050	5060	5070	5080	5090	5100

....|....|....|....|....|....|....|....|....|....|....|....|

**Atalantia_kwangtungens** **-----------gaaaaactttaagaatactt--------ttttagagaaagacttg----**

**Citrus_reticulata_1** **attagataaataaaaaactttaagaatactt--------ttttagagaaagacttgaa--**

**Citrus_reticulata_2** **attagataaataaaaaactttaagaatactt--------ttttagagaaagacttgaa--**

**Citrus_trifoliata_3** **attagataaataaaaaactttaagaatactt--------ttttagagaaagactt-----**

**Citrus_aurantiifolia** **attagataaataaaaaactttaagaatactt--------ttttagagaaagactt-----**

**Citrus_aurantium** **attagataaataaaaaactttaagaatacttttttagagttttagagaaagactt-----**

**Citrus_cavaleriei** **attagataaataaaaaactttaagaatactt--------ttttagagaaagacttg----**

**Citrus_hongheensis** **attagataaataaaaaactttaagaatacttttttagagttttagagaaagactt-----**

**Citrus_limon** **attagataaataaaaaactttaagaatacttttttagagttttagagaaagactt-----**

**Citrus_maxima** **attagataaataaaaaactttaagaatacttttttagagttttagagaaagactt-----**

**Citrus_medica** **attagataaataaaaaactttaagaatactt--------ttttagagaaagacttgaaaa**

**Citrus_polytrifolia** **attagataaataaaaaactttaagaatactt--------ttttagagaaagactt-----**

**Citrus_reticulata** **attagataaataaaaaactttaagaatactt--------ttttagagaaagacttgaa--**

**Citrus_sinensis** **attagataaataaaaaactttaagaatacttttttagagttttagagaaagactt-----**

**Fortunella_bawangica_1** **attagataaataaaaaactttaagaatactt--------ttttagagaaagacttgaaaa**

**Fortunella_bawangica_2** **attagataaataaaaaactttaagaatactt--------ttttagagaaagacttgaaaa**

**Fortunella_hindsii_1** **attagataaataaaaaactttaagaatactt--------ttttagagaaagacttgaa--**

**Fortunella_hindsii_2** **attagataaataaaaaactttaagaatactt--------ttttagagaaagacttgaa--**

**Fortunella_hindsii_3** **attagataaataaaaaactttaagaatactt--------ttttagagaaagacttgaaa-**

**Fortunella_japonica_1** **attagataaataaaaaactttaagaatactt--------ttttagagaaagacttgaa--**

**Fortunella_japonica_2** **attagataaataaaaaactttaagaatactt--------ttttagagaaagacttgaaa-**

**Fortunella_japonica_3** **attagataaataaaaaactttaagaatactt--------ttttagagaaagacttgaa--**

**Fortunella_japonica_4** **attagataaataaaaaactttaagaatactt--------ttttagagaaagacttgaa--**

**Fortunella_japonica_5** **attagataaataaaaaactttaagaatactt--------ttttagagaaagacttgaa--**

**Fortunella_japonica_6** **attagataaataaaaaactttaagaatactt--------ttttagagaaagacttgaa--**

**Fortunella_japonica_7** **attagataaataaaaaactttaagaatactt--------ttttagagaaagacttgaa--**

**Fortunella_japonica_8** **attagataaataaaaaactttaagaatactt--------ttttagagaaagacttgaa--**

**Fortunella_japonica_9** **attagataaataaaaaactttaagaatactt--------ttttagagaaagacttgaa--**

**Fortunella_japonica_10** **attagataaataaaaaactttaagaatactt--------ttttagagaaagacttg-aaa**

**Fortunella_japonica_11** **attagataaataaaaaactttaagaatactt--------ttttagagaaagacttgaaa-**

**Fortunella_japonica_12** **attagataaataaaaaactttaagaatactt--------ttttagagaaagacttgaa--**

**Fortunella_margarita_1** **attagataaataaaaaactttaagaatactt--------ttttagagaaagacttg-aaa**

**Fortunella_margarita_2** **attagataaataaaaaactttaagaatactt--------ttttagagaaagacttg-aaa**

**Fortunella_venosa_1** **attagataaataaaaaactttaagaatactt--------ttttagagaaagacttgaa--**

**Fortunella_venosa_2** **attagataaataaaaaactttaagaatactt--------ttttagagaaagacttgaa--**

**Fortunella_venosa_3** **attagataaataaaaaactttaagaatactt--------ttttagagaaagacttgaa--**

5110	5120	5130	5140	5150	5160

....|....|....|....|....|....|....|....|....|....|....|....|

**Atalantia_kwangtungens** **----aaaaaaccgaaaagaaataggtctgaatagaacaaaaggggggatagtaaagaaaa**

**Citrus_reticulata_1** **aaaaaaaaaaccgaaaagaaataggtctgaatagaacaaaaggggggatagtaaagaaaa**

**Citrus_reticulata_2** **aaaaaaaaaaccgaaaagaaataggtctgaatagaacaaaaggggggatagtaaagaaaa**

**Citrus_trifoliata_3** **gaaaaaaaaaccgaaaagaaataggtctgaatagaacaaaaggggggatagtaaagaaaa**

**Citrus_aurantiifolia** **caaaaaaaaaccgaaaagaaataggtctgaatagaacaaaaggggggatagtaaagaaaa**

**Citrus_aurantium** **caaaaaaaaaccgaaaagaaataggtctgaatagaacaaaaggggggatagtaaagaaaa**

**Citrus_cavaleriei** **aaaaaaaaaaccgaaaagaaataggtctgaatagaacaaaaggggggatagtaaagaaaa**

**Citrus_hongheensis** **caaaaaaaaaccgaaaagaaataggtctgaatagaacaaaaggggggatagtaaagaaaa**

**Citrus_limon** **caaaaaaaaaccgaaaagaaataggtctgaatagaacaaaaggggggatagtaaagaaaa**

**Citrus_maxima** **caaaaaaaaaccgaaaagaaataggtctgaatagaacaaaaggggggatagtaaagaaaa**

**Citrus_medica** **aaaaaaaaaaccgaaaagaaataggtctgaatagaacaaaaggggggatagtaaagaaaa**

**Citrus_polytrifolia** **gaaaaaaaaaccgaaaagaaataggtctgaatagaacaaaaggggggatagtaaagaaaa**

**Citrus_reticulata** **aaaaaaaaaaccgaaaagaaataggtctgaatagaacaaaaggggggatagtaaagaaaa**

**Citrus_sinensis** **caaaaaaaaaccgaaaagaaataggtctgaatagaacaaaaggggggatagtaaagaaaa**

**Fortunella_bawangica_1** **aaaaaaaaaaccgaaaagaaataggtctgaatagaacaaaaggggggatagtaaagaaaa**

**Fortunella_bawangica_2** **aaaaaaaaaaccgaaaagaaataggtctgaatagaacaaaaggggggatagtaaagaaaa**

**Fortunella_hindsii_1** **-aaaaaaaaaccgaaaagaaataggtctgaatagaacaaaaggggggatagtaaagaaaa**

**Fortunella_hindsii_2** **-aaaaaaaaaccgaaaagaaataggtctgaatagaacaaaaggggggatagtaaagaaaa**

**Fortunella_hindsii_3** **-aaaaaaaaaccgaaaagaaataggtctgaatagaacaaaaggggggatagtaaagaaaa**

**Fortunella_japonica_1** **-aaaaaaaaaccgaaaagaaataggtctgaatagaacaaaaggggggatagtaaagaaaa**

**Fortunella_japonica_2** **aaaaaaaaaaccgaaaagaaataggtctgaatagaacaaaaggggggatagtaaagaaaa**

**Fortunella_japonica_3** **-aaaaaaaaaccgaaaagaaataggtctgaatagaacaaaaggggggatagtaaagaaaa**

**Fortunella_japonica_4** **-aaaaaaaaaccgaaaagaaataggtctgaatagaacaaaaggggggatagtaaagaaaa**

**Fortunella_japonica_5** **-aaaaaaaaaccgaaaagaaataggtctgaatagaacaaaaggggggatagtaaagaaaa**

**Fortunella_japonica_6** **-aaaaaaaaaccgaaaagaaataggtctgaatagaacaaaaggggggatagtaaagaaaa**

**Fortunella_japonica_7** **-aaaaaaaaaccgaaaagaaataggtctgaatagaacaaaaggggggatagtaaagaaaa**

**Fortunella_japonica_8** **-aaaaaaaaaccgaaaagaaataggtctgaatagaacaaaaggggggatagtaaagaaaa**

**Fortunella_japonica_9** **-aaaaaaaaaccgaaaagaaataggtctgaatagaacaaaaggggggatagtaaagaaaa**

**Fortunella_japonica_10** **aaaaaaaaaaccgaaaagaaataggtctgaatagaacaaaaggggggatagtaaagaaaa**

**Fortunella_japonica_11** **-aaaaaaaaaccgaaaagaaataggtctgaatagaacaaaaggggggatagtaaagaaaa**

**Fortunella_japonica_12** **-aaaaaaaaaccgaaaagaaataggtctgaatagaacaaaaggggggatagtaaagaaaa**

**Fortunella_margarita_1** **aaaaaaaaaaccgaaaagaaataggtctgaatagaacaaaaggggggatagtaaagaaaa**

**Fortunella_margarita_2** **aaaaaaaaaaccgaaaagaaataggtctgaatagaacaaaaggggggatagtaaagaaaa**

**Fortunella_venosa_1** **--aaaaaaaaccgaaaagaaataggtctgaatagaacaaaaggggggatagtaaagaaaa**

**Fortunella_venosa_2** **--aaaaaaaaccgaaaagaaataggtctgaatagaacaaaaggggggatagtaaagaaaa**

**Fortunella_venosa_3** **--aaaaaaaaccgaaaagaaataggtctgaatagaacaaaaggggggatagtaaagaaaa**

5170	5180	5190	5200	5210	5220

....|....|....|....|....|....|....|....|....|....|....|....|

**Atalantia_kwangtungens** **aattatacaaaactagataaagcgcatccacaccctcttttttgttctattccttaatag**

**Citrus_reticulata_1** **aattatacaaaactagataaagcgcatccacaccctcttttttgttctattccttaatag**

**Citrus_reticulata_2** **aattatacaaaactagataaagcgcatccacaccctcttttttgttctattccttaatag**

**Citrus_trifoliata_3** **aattatacaaaactagataaagcgcatccacaccctcttttttgttctattccttaataa**

**Citrus_aurantiifolia** **aattatacaaaactagataaagcgcatccacaccctcttttttgttctattccttaatag**

**Citrus_aurantium** **aattatacaaaactagataaagcgcatccacaccctcttttttgttctattccttaatag**

**Citrus_cavaleriei** **aattatacaaaactagataaagcgcatccacaccctcttttttgttctattccttaatag**

**Citrus_hongheensis** **aattatacaaaactagataaagcgcatccacaccctcttttttgttctattccttaatag**

**Citrus_limon** **aattatacaaaactagataaagcgcatccacaccctcttttttgttctattccttaatag**

**Citrus_maxima** **aattatacaaaactagataaagcgcatccacaccctcttttttgttctattccttaatag**

**Citrus_medica** **aattatacaaaactagataaagcgcatccacaccctcttttttgttctattccttaatag**

**Citrus_polytrifolia** **aattatacaaaactagataaagcgcatccacaccctcttttttgttctattccttaataa**

**Citrus_reticulata** **aattatacaaaactagataaagcgcatccacaccctcttttttgttctattccttaatag**

**Citrus_sinensis** **aattatacaaaactagataaagcgcatccacaccctcttttttgttctattccttaatag**

**Fortunella_bawangica_1** **aattatacaaaactagataaagcgcatccacaccctcttttttgttctattccttaatag**

**Fortunella_bawangica_2** **aattatacaaaactagataaagcgcatccacaccctcttttttgttctattccttaatag**

**Fortunella_hindsii_1** **aattatacaaaactagataaagcgcatccacaccctcttttttgttctattccttaatag**

**Fortunella_hindsii_2** **aattatacaaaactagataaagcgcatccacaccctcttttttgttctattccttaatag**

**Fortunella_hindsii_3** **aattatacaaaactagataaagcgcatccacaccctcttttttgttctattccttaatag**

**Fortunella_japonica_1** **aattatacaaaactagataaagcgcatccacaccctcttttttgttctattccttaatag**

**Fortunella_japonica_2** **aattatacaaaactagataaagcgcatccacaccctcttttttgttctattccttaatag**

**Fortunella_japonica_3** **aattatacaaaactagataaagcgcatccacaccctcttttttgttctattccttaatag**

**Fortunella_japonica_4** **aattatacaaaactagataaagcgcatccacaccctcttttttgttctattccttaatag**

**Fortunella_japonica_5** **aattatacaaaactagataaagcgcatccacaccctcttttttgttctattccttaatag**

**Fortunella_japonica_6** **aattatacaaaactagataaagcgcatccacaccctcttttttgttctattccttaatag**

**Fortunella_japonica_7** **aattatacaaaactagataaagcgcatccacaccctcttttttgttctattccttaatag**

**Fortunella_japonica_8** **aattatacaaaactagataaagcgcatccacaccctcttttttgttctattccttaatag**

**Fortunella_japonica_9** **aattatacaaaactagataaagcgcatccacaccctcttttttgttctattccttaatag**

**Fortunella_japonica_10** **aattatacaaaactagataaagcgcatccacaccctcttttttgttctattccttaatag**

**Fortunella_japonica_11** **aattatacaaaactagataaagcgcatccacaccctcttttttgttctattccttaatag**

**Fortunella_japonica_12** **aattatacaaaactagataaagcgcatccacaccctcttttttgttctattccttaatag**

**Fortunella_margarita_1** **aattatacaaaactagataaagcgcatccacaccctcttttttgttctattccttaatag**

**Fortunella_margarita_2** **aattatacaaaactagataaagcgcatccacaccctcttttttgttctattccttaatag**

**Fortunella_venosa_1** **aattatacaaaactagataaagcgcatccacaccctcttttttgttctattccttaatag**

**Fortunella_venosa_2** **aattatacaaaactagataaagcgcatccacaccctcttttttgttctattccttaatag**

**Fortunella_venosa_3** **aattatacaaaactagataaagcgcatccacaccctcttttttgttctattccttaatag**

5230	5240	5250	5260	5270	5280

....|....|....|....|....|....|....|....|....|....|....|....|

**Atalantia_kwangtungens** **aatttcgtttgattaagactaagttctgtaaaaacccccgctttctgtgaaatatcatga**

**Citrus_reticulata_1** **aatttcgtttgattaagactaacttctgtaaaaacccccgctttctgtgaaatatcatga**

**Citrus_reticulata_2** **aatttcgtttgattaagactaacttctgtaaaaacccccgctttctgtgaaatatcatga**

**Citrus_trifoliata_3** **aatttcgtttgattaagactaacttctgtaaaaacccccgctttctgtgaaatatcatga**

**Citrus_aurantiifolia** **aatttcgtttgattaagactaacttctgtaaaaacccccgctttctgtgaaatatcatga**

**Citrus_aurantium** **aatttcgtttgattaagactaacttctgtaaaaacccccgctttctgtgaaatatcatga**

**Citrus_cavaleriei** **aatttcgtttgattaagactaacttctgtaaaaacccccgctttctgtgaaatatcatga**

**Citrus_hongheensis** **aatttcgtttgattaagactaacttctgtaaaaacccccgctttctgtgaaatatcatga**

**Citrus_limon** **aatttcgtttgattaagactaacttctgtaaaaacccccgctttctgtgaaatatcatga**

**Citrus_maxima** **aatttcgtttgattaagactaacttctgtaaaaacccccgctttctgtgaaatatcatga**

**Citrus_medica** **aatttcgtttgattaagactaacttctgtaaaaacccccgctttctgtgaaatatcatga**

**Citrus_polytrifolia** **aatttagtttgattaagactaacttctgtaaaaacccccgctttctgtgaaatatcatga**

**Citrus_reticulata** **aatttcgtttgattaagactaacttctgtaaaaacccccgctttctgtgaaatatcatga**

**Citrus_sinensis** **aatttcgtttgattaagactaacttctgtaaaaacccccgctttctgtgaaatatcatga**

**Fortunella_bawangica_1** **aatttcgtttgattaagactaacttctgtaaaaacccccgctttctgtgaaatatcatga**

**Fortunella_bawangica_2** **aatttcgtttgattaagactaacttctgtaaaaacccccgctttctgtgaaatatcatga**

**Fortunella_hindsii_1** **aatttcgtttgattaagactaacttctgtaaaaacccccgctttctgtgaaatatcatga**

**Fortunella_hindsii_2** **aatttcgtttgattaagactaacttctgtaaaaacccccgctttctgtgaaatatcatga**

**Fortunella_hindsii_3** **aatttcgtttgattaagactaacttctgtaaaaacccccgctttctgtgaaatatcatga**

**Fortunella_japonica_1** **aatttcgtttgattaagactaacttctgtaaaaacccccgctttctgtgaaatatcatga**

**Fortunella_japonica_2** **aatttcgtttgattaagactaacttctgtaaaaacccccgctttctgtgaaatatcatga**

**Fortunella_japonica_3** **aatttcgtttgattaagactaacttctgtaaaaacccccgctttctgtgaaatatcatga**

**Fortunella_japonica_4** **aatttcgtttgattaagactaacttctgtaaaaacccccgctttctgtgaaatatcatga**

**Fortunella_japonica_5** **aatttcgtttgattaagactaacttctgtaaaaacccccgctttctgtgaaatatcatga**

**Fortunella_japonica_6** **aatttcgtttgattaagactaacttctgtaaaaacccccgctttctgtgaaatatcatga**

**Fortunella_japonica_7** **aatttcgtttgattaagactaacttctgtaaaaacccccgctttctgtgaaatatcatga**

**Fortunella_japonica_8** **aatttcgtttgattaagactaacttctgtaaaaacccccgctttctgtgaaatatcatga**

**Fortunella_japonica_9** **aatttcgtttgattaagactaacttctgtaaaaacccccgctttctgtgaaatatcatga**

**Fortunella_japonica_10** **aatttcgtttgattaagactaacttctgtaaaaacccccgctttctgtgaaatatcatga**

**Fortunella_japonica_11** **aatttcgtttgattaagactaacttctgtaaaaacccccgctttctgtgaaatatcatga**

**Fortunella_japonica_12** **aatttcgtttgattaagactaacttctgtaaaaacccccgctttctgtgaaatatcatga**

**Fortunella_margarita_1** **aatttcgtttgattaagactaacttctgtaaaaacccccgctttctgtgaaatatcatga**

**Fortunella_margarita_2** **aatttcgtttgattaagactaacttctgtaaaaacccccgctttctgtgaaatatcatga**

**Fortunella_venosa_1** **aatttcgtttgattaagactaacttctgtaaaaacccccgctttctgtgaaatatcatga**

**Fortunella_venosa_2** **aatttcgtttgattaagactaacttctgtaaaaacccccgctttctgtgaaatatcatga**

**Fortunella_venosa_3** **aatttcgtttgattaagactaacttctgtaaaaacccccgctttctgtgaaatatcatga**

5290	5300	5310	5320	5330	5340

....|....|....|....|....|....|....|....|....|....|....|....|

**Atalantia_kwangtungens** **acggttcctgtaggttgagcgcccttggcaaggaaatatagaatagcaggaacatttaaa**

**Citrus_reticulata_1** **acggttcctgtaggttgagcgcccttggcaaggaaatatagaatagcaggaacatttaaa**

**Citrus_reticulata_2** **acggttcctgtaggttgagcgcccttggcaaggaaatatagaatagcaggaacatttaaa**

**Citrus_trifoliata_3** **acggttcctgtaggttgagcgcccttggcaaggaaatatagaatagcaggaacatttaaa**

**Citrus_aurantiifolia** **acggttcctgtaggttgagcgcccttggcaaggaaatatagaatagcaggaacatttaaa**

**Citrus_aurantium** **acggttcctgtaggttgagcgcccttggcaaggaaatatagaatagcaggaacatttaaa**

**Citrus_cavaleriei** **acggttcctgtaggttgagcgcccttggcaaggaaatatagaatagcaggaacatttaaa**

**Citrus_hongheensis** **acggttcctgtaggttgagcgcccttggcaaggaaatatagaatagcaggaacatttaaa**

**Citrus_limon** **acggttcctgtaggttgagcgcccttggcaaggaaatatagaatagcaggaacatttaaa**

**Citrus_maxima** **acggttcctgtaggttgagcgcccttggcaaggaaatatagaatagcaggaacatttaaa**

**Citrus_medica** **acggttcctgtaggttgagcgcctttggcaaggaaatatagaatagcaggaacatttaaa**

**Citrus_polytrifolia** **acggttcctgtaggttgagcgcccttggcaaggaaatatagaatagcaggaacatttaaa**

**Citrus_reticulata** **acggttcctgtaggttgagcgcccttggcaaggaaatatagaatagcaggaacatttaaa**

**Citrus_sinensis** **acggttcctgtaggttgagcgcccttggcaaggaaatatagaatagcaggaacatttaaa**

**Fortunella_bawangica_1** **acggttcctgtaggttgagcgcccttggcaaggaaatatagaatagcaggaacatttaaa**

**Fortunella_bawangica_2** **acggttcctgtaggttgagcgcccttggcaaggaaatatagaatagcaggaacatttaaa**

**Fortunella_hindsii_1** **acggttcctgtaggttgagcgcccttggcaaggaaatatagaatagcaggaacatttaaa**

**Fortunella_hindsii_2** **acggttcctgtaggttgagcgcccttggcaaggaaatatagaatagcaggaacatttaaa**

**Fortunella_hindsii_3** **acggttcctgtaggttgagcgcccttggcaaggaaatatagaatagcaggaacatttaaa**

**Fortunella_japonica_1** **acggttcctgtaggttgagcgcccttggcaaggaaatatataatagcaggaacatttaaa**

**Fortunella_japonica_2** **acggttcctgtaggttgagcgcccttggcaaggaaatatataatagcaggaacatttaaa**

**Fortunella_japonica_3** **acggttcctgtaggttgagcgcccttggcaaggaaatatataatagcaggaacatttaaa**

**Fortunella_japonica_4** **acggttcctgtaggttgagcgcccttggcaaggaaatatataatagcaggaacatttaaa**

**Fortunella_japonica_5** **acggttcctgtaggttgagcgcccttggcaaggaaatatataatagcaggaacatttaaa**

**Fortunella_japonica_6** **acggttcctgtaggttgagcgcccttggcaaggaaatatataatagcaggaacatttaaa**

**Fortunella_japonica_7** **acggttcctgtaggttgagcgcccttggcaaggaaatatataatagcaggaacatttaaa**

**Fortunella_japonica_8** **acggttcctgtaggttgagcgcccttggcaaggaaatatataatagcaggaacatttaaa**

**Fortunella_japonica_9** **acggttcctgtaggttgagcgcccttggcaaggaaatatataatagcaggaacatttaaa**

**Fortunella_japonica_10** **acggttcctgtaggttgagcgcccttggcaaggaaatatagaatagcaggaacatttaaa**

**Fortunella_japonica_11** **acggttcctgtaggttgagcgcccttggcaaggaaatatataatagcaggaacatttaaa**

**Fortunella_japonica_12** **acggttcctgtaggttgagcgcccttggcaaggaaatatataatagcaggaacatttaaa**

**Fortunella_margarita_1** **acggttcctgtaggttgagcgcccttggcaaggaaatatagaatagcaggaacatttaaa**

**Fortunella_margarita_2** **acggttcctgtaggttgagcgcccttggcaaggaaatatagaatagcaggaacatttaaa**

**Fortunella_venosa_1** **acggttcctgtaggttgagcgcccttggcaaggaaatatagaatagcaggaacatttaaa**

**Fortunella_venosa_2** **acggttcctgtaggttgagcgcccttggcaaggaaatatagaatagcaggaacatttaaa**

**Fortunella_venosa_3** **acggttcctgtaggttgagcgcccttggcaaggaaatatagaatagcaggaacatttaaa**

5350	5360	5370	5380	5390	5400

....|....|....|....|....|....|....|....|....|....|....|....|

**Atalantia_kwangtungens** **tgggtttgattatttattggatcataaaaacccactttccgaagatctcttccttctctt**

**Citrus_reticulata_1** **tgggtttgattatttatcggatcataaaaacccactttccgaagatctcttccttctctt**

**Citrus_reticulata_2** **tgggtttgattatttatcggatcataaaaacccactttccgaagatctcttccttctctt**

**Citrus_trifoliata_3** **tgggtttgattatttatcggatcataaaaacccactttccgaagatctcttccttctctt**

**Citrus_aurantiifolia** **tgggtttgattatttatcggatcataaaaacccactttccgaagatctcttccttctctt**

**Citrus_aurantium** **tgggtttgattatttatcggatcataaaaacccactttccgaagatctcttccttctctt**

**Citrus_cavaleriei** **tgggtttgattatttatcggatcataaaaacccactttccgaagatctcttccttctctt**

**Citrus_hongheensis** **tgggtttgattatttatcggatcataaaaacccactttccgaagatctcttccttctctt**

**Citrus_limon** **tgggtttgattatttatcggatcataaaaacccactttccgaagatctcttccttctctt**

**Citrus_maxima** **tgggtttgattatttatcggatcataaaaacccactttccgaagatctcttccttctctt**

**Citrus_medica** **tgggttttattatttatcggatcataaaaacctactttccgaagatctcttccttctctt**

**Citrus_polytrifolia** **tgggtttgattatttatcggatcataaaaacccactttccgaagatctcttccttctctt**

**Citrus_reticulata** **tgggtttgattatttatcggatcataaaaacccactttccgaagatctcttccttctctt**

**Citrus_sinensis** **tgggtttgattatttatcggatcataaaaacccactttccgaagatctcttccttctctt**

**Fortunella_bawangica_1** **tgggtttgattatttatcggatcataaaaacccactttccgaagatctcttccttctctt**

**Fortunella_bawangica_2** **tgggtttgattatttatcggatcataaaaacccactttccgaagatctcttccttctctt**

**Fortunella_hindsii_1** **tgggtttgattatttatcggatcataaaaacccactttccgaagatctcttccttctctt**

**Fortunella_hindsii_2** **tgggtttgattatttatcggatcataaaaacccactttccgaagatctcttccttctctt**

**Fortunella_hindsii_3** **tgggtttgattatttatcggatcataaaaacccactttccgaagatctcttccttctctt**

**Fortunella_japonica_1** **tgggtttgattatttatcggatcataaaaacccactttccgaagatctcttccttctctt**

**Fortunella_japonica_2** **tgggtttgattatttatcggatcataaaaacccactttccgaagatctcttccttctctt**

**Fortunella_japonica_3** **tgggtttgattatttatcggatcataaaaacccactttccgaagatctcttccttctctt**

**Fortunella_japonica_4** **tgggtttgattatttatcggatcataaaaacccactttccgaagatctcttccttctctt**

**Fortunella_japonica_5** **tgggtttgattatttatcggatcataaaaacccactttccgaagatctcttccttctctt**

**Fortunella_japonica_6** **tgggtttgattatttatcggatcataaaaacccactttccgaagatctcttccttctctt**

**Fortunella_japonica_7** **tgggtttgattatttatcggatcataaaaacccactttccgaagatctcttccttctctt**

**Fortunella_japonica_8** **tgggtttgattatttatcggatcataaaaacccactttccgaagatctcttccttctctt**

**Fortunella_japonica_9** **tgggtttgattatttatcggatcataaaaacccactttccgaagatctcttccttctctt**

**Fortunella_japonica_10** **tgggtttgattatttatcggatcataaaaacccactttccgaagatctcttccttctctt**

**Fortunella_japonica_11** **tgggtttgattatttatcggatcataaaaacccactttccgaagatctcttccttctctt**

**Fortunella_japonica_12** **tgggtttgattatttatcggatcataaaaacccactttccgaagatctcttccttctctt**

**Fortunella_margarita_1** **tgggtttgattatttatcggatcataaaaacccactttccgaagatctcttccttctctt**

**Fortunella_margarita_2** **tgggtttgattatttatcggatcataaaaacccactttccgaagatctcttccttctctt**

**Fortunella_venosa_1** **tgggtttgattatttatcggatcataaaaacccactttccgaagatctcttccttctctt**

**Fortunella_venosa_2** **tgggtttgattatttatcggatcataaaaacccactttccgaagatctcttccttctctt**

**Fortunella_venosa_3** **tgggtttgattatttatcggatcataaaaacccactttccgaagatctcttccttctctt**

5410	5420	5430	5440	5450	5460

....|....|....|....|....|....|....|....|....|....|....|....|

**Atalantia_kwangtungens** **cgggatcgaccatcaattgcaacgattcgataaacggctcattgggatagatatagatga**

**Citrus_reticulata_1** **cgggatcgaccatcaattgcaacgattcgataaacgactcattgggatagatatagatga**

**Citrus_reticulata_2** **cgggatcgaccatcaattgcaacgattcgataaacgactcattgggatagatatagatga**

**Citrus_trifoliata_3** **cgggatcgaccatcaattgcaacgattcgataaacggctcattgggatagatatagatga**

**Citrus_aurantiifolia** **cgggatcgaccatcaattgcaacgattcgataaacggctcattgggatagatatagatga**

**Citrus_aurantium** **cgggatcgaccatcaattgcaacgattcgataaacggctcattgggatagatatagatga**

**Citrus_cavaleriei** **cgggatcgaccatcaattgcaacgattcgataaacggctcattgggatagatatagatga**

**Citrus_hongheensis** **cgggatcgaccatcaattgcaacgattcgataaacggctcattgggatagatatagatga**

**Citrus_limon** **cgggatcgaccatcaattgcaacgattcgataaacggctcattgggatagatatagatga**

**Citrus_maxima** **cgggatcgaccatcaattgcaacgattcgataaacggctcattgggatagatatagatga**

**Citrus_medica** **cgggatcgaccatcaattgcaacgattcgataaacggctcattgggatagatatagatga**

**Citrus_polytrifolia** **cgggatcgaccatcaattgcaacgattcgataaacggctcattgggatagatatagatga**

**Citrus_reticulata** **cgggatcgaccatcaattgcaacgattcgataaacgactcattgggatagatatagatga**

**Citrus_sinensis** **cgggatcgaccatcaattgcaacgattcgataaacggctcattgggatagatatagatga**

**Fortunella_bawangica_1** **cgggatcgaccatcaattgcaacgattcgataaacggctcattgggatagatatagatga**

**Fortunella_bawangica_2** **cgggatcgaccatcaattgcaacgattcgataaacggctcattgggatagatatagatga**

**Fortunella_hindsii_1** **cgggatcgaccatcaattgcaacgattcgataaacggctcattgggatagatatagatga**

**Fortunella_hindsii_2** **cgggatcgaccatcaattgcaacgattcgataaacggctcattgggatagatatagatga**

**Fortunella_hindsii_3** **cgggatcgaccatcaattgcaacgattcgataaacggctcattgggatagatatagatga**

**Fortunella_japonica_1** **cgggatcgaccatcaattgcaacgattcgataaacggctcattgggatagatatagatga**

**Fortunella_japonica_2** **cgggatcgaccatcaattgcaacgattcgataaacggctcattgggatagatatagatga**

**Fortunella_japonica_3** **cgggatcgaccatcaattgcaacgattcgataaacggctcattgggatagatatagatga**

**Fortunella_japonica_4** **cgggatcgaccatcaattgcaacgattcgataaacggctcattgggatagatatagatga**

**Fortunella_japonica_5** **cgggatcgaccatcaattgcaacgattcgataaacggctcattgggatagatatagatga**

**Fortunella_japonica_6** **cgggatcgaccatcaattgcaacgattcgataaacggctcattgggatagatatagatga**

**Fortunella_japonica_7** **cgggatcgaccatcaattgcaacgattcgataaacggctcattgggatagatatagatga**

**Fortunella_japonica_8** **cgggatcgaccatcaattgcaacgattcgataaacggctcattgggatagatatagatga**

**Fortunella_japonica_9** **cgggatcgaccatcaattgcaacgattcgataaacggctcattgggatagatatagatga**

**Fortunella_japonica_10** **cgggatcgaccatcaattgcaacgattcgataaacggctcattgggatagatatagatga**

**Fortunella_japonica_11** **cgggatcgaccatcaattgcaacgattcgataaacggctcattgggatagatatagatga**

**Fortunella_japonica_12** **cgggatcgaccatcaattgcaacgattcgataaacggctcattgggatagatatagatga**

**Fortunella_margarita_1** **cgggatcgaccatcaattgcaacgattcgataaacggctcattgggatagatatagatga**

**Fortunella_margarita_2** **cgggatcgaccatcaattgcaacgattcgataaacggctcattgggatagatatagatga**

**Fortunella_venosa_1** **cgggatcgaccatcaattgcaacgattcgataaacggctcattgggatagatatagatga**

**Fortunella_venosa_2** **cgggatcgaccatcaattgcaacgattcgataaacggctcattgggatagatatagatga**

**Fortunella_venosa_3** **cgggatcgaccatcaattgcaacgattcgataaacggctcattgggatagatatagatga**

5470	5480	5490	5500	5510	5520

....|....|....|....|....|....|....|....|....|....|....|....|

**Atalantia_kwangtungens** **acagtaccccccctagaaacgtataagaagttttctcctcgtacggctcgagaaaaacaa**

**Citrus_reticulata_1** **acagtaccccccctagaaacgtataagaagttttctcctcgtacggctcgagaaaaaaaa**

**Citrus_reticulata_2** **acagtaccccccctagaaacgtataagaagttttctcctcgtacggctcgagaaaaaaaa**

**Citrus_trifoliata_3** **acagtaccccccctagaaacgtataagaagttttctcctcgtacggctcgag-aaaacaa**

**Citrus_aurantiifolia** **acagtaccccccctagaaacgtataagaagttttctcctcgtacggctcgag-aaaacaa**

**Citrus_aurantium** **acagtaccccccctagaaacgtataagaagttttctcctcgtacggctcgag-aaaacaa**

**Citrus_cavaleriei** **acagtaccccccctagaaacgtataagaagttttctcctcgtacggctcgag-aaaacaa**

**Citrus_hongheensis** **acagtaccccccctagaaacgtataagaagttttctcctcgtacggctcgag-aaaacaa**

**Citrus_limon** **acagtaccccccctagaaacgtataagaagttttctcctcgtacggctcgag-aaaacaa**

**Citrus_maxima** **acagtaccccccctagaaacgtataagaagttttctcctcgtacggctcgag-aaaacaa**

**Citrus_medica** **acagtaccccccctagaaacgtataagaagttttctcctcgtacggctcgagaaaaacaa**

**Citrus_polytrifolia** **acagtaccccccctagaaacgtataagaagttttctcctcgtacggctcgag-aaaacaa**

**Citrus_reticulata** **acagtaccccccctagaaacgtataagaagttttctcctcgtacggctcgagaaaaaaaa**

**Citrus_sinensis** **acagtaccccccctagaaacgtataagaagttttctcctcgtacggctcgag-aaaacaa**

**Fortunella_bawangica_1** **acagtaccccccctagaaacgtataagaagttttctcctcgtacggctcgag-aaaacaa**

**Fortunella_bawangica_2** **acagtaccccccctagaaacgtataagaagttttctcctcgtacggctcgag-aaaacaa**

**Fortunella_hindsii_1** **acagtaccccccctagaaacgtataagaagttttctcctcgtacggctcgag-aaaacaa**

**Fortunella_hindsii_2** **acagtaccccccctagaaacgtataagaagttttctcctcgtacggctcgag-aaaacaa**

**Fortunella_hindsii_3** **acagtaccccccctagaaacgtataagaagttttctcctcgtacggctcgag-aaaacaa**

**Fortunella_japonica_1** **acagtaccccccctagaaacgtataagaagttttctcctcgtacggctcgag-aaaacaa**

**Fortunella_japonica_2** **acagtaccccccctagaaacgtataagaagttttctcctcgtacggctcgag-aaaacaa**

**Fortunella_japonica_3** **acagtaccccccctagaaacgtataagaagttttctcctcgtacggctcgag-aaaacaa**

**Fortunella_japonica_4** **acagtaccccccctagaaacgtataagaagttttctcctcgtacggctcgag-aaaacaa**

**Fortunella_japonica_5** **acagtaccccccctagaaacgtataagaagttttctcctcgtacggctcgag-aaaacaa**

**Fortunella_japonica_6** **acagtaccccccctagaaacgtataagaagttttctcctcgtacggctcgag-aaaacaa**

**Fortunella_japonica_7** **acagtaccccccctagaaacgtataagaagttttctcctcgtacggctcgag-aaaacaa**

**Fortunella_japonica_8** **acagtaccccccctagaaacgtataagaagttttctcctcgtacggctcgag-aaaacaa**

**Fortunella_japonica_9** **acagtaccccccctagaaacgtataagaagttttctcctcgtacggctcgag-aaaacaa**

**Fortunella_japonica_10** **acagtaccccccctagaaacgtataagaagttttctcctcgtacggctcgag-aaaacaa**

**Fortunella_japonica_11** **acagtaccccccctagaaacgtataagaagttttctcctcgtacggctcgag-aaaacaa**

**Fortunella_japonica_12** **acagtaccccccctagaaacgtataagaagttttctcctcgtacggctcgag-aaaacaa**

**Fortunella_margarita_1** **acagtaccccccctagaaacgtataagaagttttctcctcgtacggctcgag-aaaacaa**

**Fortunella_margarita_2** **acagtaccccccctagaaacgtataagaagttttctcctcgtacggctcgag-aaaacaa**

**Fortunella_venosa_1** **acagtaccccccctagaaacgtataagaagttttctcctcgtacggctcgag-aaaacaa**

**Fortunella_venosa_2** **acagtaccccccctagaaacgtataagaagttttctcctcgtacggctcgag-aaaacaa**

**Fortunella_venosa_3** **acagtaccccccctagaaacgtataagaagttttctcctcgtacggctcgag-aaaacaa**

5530	5540	5550	5560	5570	5580

....|....|....|....|....|....|....|....|....|....|....|....|

**Atalantia_kwangtungens** **aatggttcgaagtgatagttatgtctatgtatagaattagaatgtaaaatagatctataa**

**Citrus_reticulata_1** **aatggttagaagtgacagttatgtctatgtatagaattagaatgtcaaatagatctataa**

**Citrus_reticulata_2** **aatggttagaagtgacagttatgtctatgtatagaattagaatgtcaaatagatctataa**

**Citrus_trifoliata_3** **aatggttagaagtgatagttatgtctatgtatagaagtagaatgtcaaatagatctataa**

**Citrus_aurantiifolia** **aatggttcgaagtgatagttatgtctatgtatagaattagaatgtcaaatagatctataa**

**Citrus_aurantium** **aatggttcgaagtgatagttatgtctatgtatagaattagaatgtcaaatagatctataa**

**Citrus_cavaleriei** **aatggttcgaagtgatagttatgtctatgtatagaattagaatgtcaaatagatctataa**

**Citrus_hongheensis** **aatggttcgaagtgatagttatgtctatgtatagaattagaatgtcaaatagatctataa**

**Citrus_limon** **aatggttcgaagtgatagttatgtctatgtatagaattagaatgtcaaatagatctataa**

**Citrus_maxima** **aatggttcgaagtgatagttatgtctatgtatagaattagaatgtcaaatagatctataa**

**Citrus_medica** **aatggttagaagtgatagttatgtctatgtatagaattagaatgtcaaatagatctataa**

**Citrus_polytrifolia** **aatggttagaagtgatagttatgtctatgtatagaagtagaatgtcaaatagatctataa**

**Citrus_reticulata** **aatggttagaagtgacagttatgtctatgtatagaattagaatgtcaaatagatctataa**

**Citrus_sinensis** **aatggttcgaagtgatagttatgtctatgtatagaattagaatgtcaaatagatctataa**

**Fortunella_bawangica_1** **aatggttagaagtgatagttatgtctatgtatagaattagaatgtcaaatagatctataa**

**Fortunella_bawangica_2** **aatggttagaagtgatagttatgtctatgtatagaattagaatgtcaaatagatctataa**

**Fortunella_hindsii_1** **aatggttagaagtgatagttatgtctatgtatagaattagaatgtcaaatagatctataa**

**Fortunella_hindsii_2** **aatggttagaagtgatagttatgtctatgtatagaattagaatgtcaaatagatctataa**

**Fortunella_hindsii_3** **aatggttagaagtgatagttatgtctatgtatagaattagaatgtcaaatagatctataa**

**Fortunella_japonica_1** **aatggttagaagtgatagttatgtctatgtatagaattagaatgtcaaatagatctataa**

**Fortunella_japonica_2** **aatggttagaagtgatagttatgtctatgtatagaattagaatgtcaaatagatctataa**

**Fortunella_japonica_3** **aatggttagaagtgatagttatgtctatgtatagaattagaatgtcaaatagatctataa**

**Fortunella_japonica_4** **aatggttagaagtgatagttatgtctatgtatagaattagaatgtcaaatagatctataa**

**Fortunella_japonica_5** **aatggttagaagtgatagttatgtctatgtatagaattagaatgtcaaatagatctataa**

**Fortunella_japonica_6** **aatggttagaagtgatagttatgtctatgtatagaattagaatgtcaaatagatctataa**

**Fortunella_japonica_7** **aatggttagaagtgatagttatgtctatgtatagaattagaatgtcaaatagatctataa**

**Fortunella_japonica_8** **aatggttagaagtgatagttatgtctatgtatagaattagaatgtcaaatagatctataa**

**Fortunella_japonica_9** **aatggttagaagtgatagttatgtctatgtatagaattagaatgtcaaatagatctataa**

**Fortunella_japonica_10** **aatggttagaagtgatagttatgtctatgtatagaattagaatgtcaaatagatctataa**

**Fortunella_japonica_11** **aatggttagaagtgatagttatgtctatgtatagaattagaatgtcaaatagatctataa**

**Fortunella_japonica_12** **aatggttagaagtgatagttatgtctatgtatagaattagaatgtcaaatagatctataa**

**Fortunella_margarita_1** **aatggttagaagtgatagttatgtctatgtatagaattagaatgtcaaatagatctataa**

**Fortunella_margarita_2** **aatggttagaagtgatagttatgtctatgtatagaattagaatgtcaaatagatctataa**

**Fortunella_venosa_1** **aatggttagaagtgatagttatgtctatgtatagaattagaatgtcaaatagatctataa**

**Fortunella_venosa_2** **aatggttagaagtgatagttatgtctatgtatagaattagaatgtcaaatagatctataa**

**Fortunella_venosa_3** **aatggttagaagtgatagttatgtctatgtatagaattagaatgtcaaatagatctataa**

5590	5600	5610	5620	5630	5640

....|....|....|....|....|....|....|....|....|....|....|....|

**Atalantia_kwangtungens** **aataatcaaatcaattagagatttaagttattctttttttgtttctttgtcattttcaaa**

**Citrus_reticulata_1** **aataatcaaatcaattagagatttaagttattctttttttgtttctttgtcattttcaaa**

**Citrus_reticulata_2** **aataatcaaatcaattagagatttaagttattctttttttgtttctttgtcattttcaaa**

**Citrus_trifoliata_3** **aataatcaaatcaattagagatttaagttattctttttttgtttctttgtcattttccaa**

**Citrus_aurantiifolia** **aataatcaaatcaattagagatttaagttattctttttttgtttctttgtcattttcaaa**

**Citrus_aurantium** **aataatcaaatcaattagagatttaagttattctttttttgtttctttgtcattttcaaa**

**Citrus_cavaleriei** **aataatcaaatcaattagagatttaagttattctttttttgtttctttgtcattttcaaa**

**Citrus_hongheensis** **aataatcaaatcaattagagatttaagttattctttttttgtttctttgtcattttcaaa**

**Citrus_limon** **aataatcaaatcaattagagatttaagttattctttttttgtttctttgtcattttcaaa**

**Citrus_maxima** **aataatcaaatcaattagagatttaagttattctttttttgtttctttgtcattttcaaa**

**Citrus_medica** **aataatcaaatcaattatagatttaagttattctttttttgtttcgttgtcattttcaaa**

**Citrus_polytrifolia** **aataatcaaatcaattagagatttaagttattctttttttgtttctttgtcattttccaa**

**Citrus_reticulata** **aataatcaaatcaattagagatttaagttattctttttttgtttctttgtcattttcaaa**

**Citrus_sinensis** **aataatcaaatcaattagagatttaagttattctttttttgtttctttgtcattttcaaa**

**Fortunella_bawangica_1** **aataatcaaatcaattagagatttaagttattctttttttgtttctttgtcattttcaaa**

**Fortunella_bawangica_2** **aataatcaaatcaattagagatttaagttattctttttttgtttctttgtcattttcaaa**

**Fortunella_hindsii_1** **aataatcaaatcaattagagatttaagttattctttttttgtttctttgtcattttcaaa**

**Fortunella_hindsii_2** **aataatcaaatcaattagagatttaagttattctttttttgtttctttgtcattttcaaa**

**Fortunella_hindsii_3** **aataatcaaatcaattagagatttaagttattctttttttgtttctttgtcattttcaaa**

**Fortunella_japonica_1** **aataatcaaatcaattagagatttaagttattctttttttgtttctttgtcattttcaaa**

**Fortunella_japonica_2** **aataatcaaatcaattagagatttaagttattctttttttgtttctttgtcattttcaaa**

**Fortunella_japonica_3** **aataatcaaatcaattagagatttaagttattctttttttgtttctttgtcattttcaaa**

**Fortunella_japonica_4** **aataatcaaatcaattagagatttaagttattctttttttgtttctttgtcattttcaaa**

**Fortunella_japonica_5** **aataatcaaatcaattagagatttaagttattctttttttgtttctttgtcattttcaaa**

**Fortunella_japonica_6** **aataatcaaatcaattagagatttaagttattctttttttgtttctttgtcattttcaaa**

**Fortunella_japonica_7** **aataatcaaatcaattagagatttaagttattctttttttgtttctttgtcattttcaaa**

**Fortunella_japonica_8** **aataatcaaatcaattagagatttaagttattctttttttgtttctttgtcattttcaaa**

**Fortunella_japonica_9** **aataatcaaatcaattagagatttaagttattctttttttgtttctttgtcattttcaaa**

**Fortunella_japonica_10** **aataatcaaatcaattagagatttaagttattctttttttgtttctttgtcattttcaaa**

**Fortunella_japonica_11** **aataatcaaatcaattagagatttaagttattctttttttgtttctttgtcattttcaaa**

**Fortunella_japonica_12** **aataatcaaatcaattagagatttaagttattctttttttgtttctttgtcattttcaaa**

**Fortunella_margarita_1** **aataatcaaatcaattagagatttaagttattctttttttgtttctttgtcattttcaaa**

**Fortunella_margarita_2** **aataatcaaatcaattagagatttaagttattctttttttgtttctttgtcattttcaaa**

**Fortunella_venosa_1** **aataatcaaatcaattagagatttaagttattctttttttgtttctttgtcattttcaaa**

**Fortunella_venosa_2** **aataatcaaatcaattagagatttaagttattctttttttgtttctttgtcattttcaaa**

**Fortunella_venosa_3** **aataatcaaatcaattagagatttaagttattctttttttgtttctttgtcattttcaaa**

5650	5660	5670	5680	5690	5700

....|....|....|....|....|....|....|....|....|....|....|....|

**Atalantia_kwangtungens** **agaaacaaaaaaagcattcgtactctcataactcaagttagataagtttcaaagcccagc**

**Citrus_reticulata_1** **agaaacaaaaaaagaattcgtactctcataactcaagttagataagtttcaacgcccagc**

**Citrus_reticulata_2** **agaaacaaaaaaagaattcgtactctcataactcaagttagataagtttcaacgcccagc**

**Citrus_trifoliata_3** **agaaacaaaaaaagaattcgtactctcataactcaagttagataagtttcaacgcccagc**

**Citrus_aurantiifolia** **agaaacaaaaaaagaattcgtactctcataactcaagttagataagtttcaacgcccagc**

**Citrus_aurantium** **agaaacaaaaaaagaattcgtactctcataactcaagttagataagtttcaacgcccagc**

**Citrus_cavaleriei** **agaaacaaaaaaagaattcgtactctcataactcaagttagataagtttcaacgcccagc**

**Citrus_hongheensis** **agaaacaaaaaaagaattagtactctcataactcaagttagataagtttcaacgcccagc**

**Citrus_limon** **agaaacaaaaaaagaattcgtactctcataactcaagttagataagtttcaacgcccagc**

**Citrus_maxima** **agaaacaaaaaaagaattcgtactctcataactcaagttagataagtttcaacgcccagc**

**Citrus_medica** **agaaacaaaaaaagaattcgtactctcataactcaagttagataagtttcaacgcccagc**

**Citrus_polytrifolia** **agaaacaaaaaaagaattcgtactctcataactcaagttagataagtttcaacgcccagc**

**Citrus_reticulata** **agaaacaaaaaaagaattcgtactctcataactcaagttagataagtttcaacgcccagc**

**Citrus_sinensis** **agaaacaaaaaaagaattcgtactctcataactcaagttagataagtttcaacgcccagc**

**Fortunella_bawangica_1** **agaaacaaaaaaagaattcgtactctcataactcaagttagataagtttcaacgcccagc**

**Fortunella_bawangica_2** **agaaacaaaaaaagaattcgtactctcataactcaagttagataagtttcaacgcccagc**

**Fortunella_hindsii_1** **agaaacaaaaaaagaattcgtactctcataactcaagttagataagtttcaacgcccagc**

**Fortunella_hindsii_2** **agaaacaaaaaaagaattcgtactctcataactcaagttagataagtttcaacgcccagc**

**Fortunella_hindsii_3** **agaaacaaaaaaagaattcgtactctcataactcaagttagataagtttcaacgcccagc**

**Fortunella_japonica_1** **agaaacaaaaaaagaattcgtactctcataactcaagttagataagtttcaacgcccagc**

**Fortunella_japonica_2** **agaaacaaaaaaagaattcgtactctcataactcaagttagataagtttcaacgcccagc**

**Fortunella_japonica_3** **agaaacaaaaaaagaattcgtactctcataactcaagttagataagtttcaacgcccagc**

**Fortunella_japonica_4** **agaaacaaaaaaagaattcgtactctcataactcaagttagataagtttcaacgcccagc**

**Fortunella_japonica_5** **agaaacaaaaaaagaattcgtactctcataactcaagttagataagtttcaacgcccagc**

**Fortunella_japonica_6** **agaaacaaaaaaagaattcgtactctcataactcaagttagataagtttcaacgcccagc**

**Fortunella_japonica_7** **agaaacaaaaaaagaattcgtactctcataactcaagttagataagtttcaacgcccagc**

**Fortunella_japonica_8** **agaaacaaaaaaagaattcgtactctcataactcaagttagataagtttcaacgcccagc**

**Fortunella_japonica_9** **agaaacaaaaaaagaattcgtactctcataactcaagttagataagtttcaacgcccagc**

**Fortunella_japonica_10** **agaaacaaaaaaagaattcgtactctcataactcaagttagataagtttcaacgcccagc**

**Fortunella_japonica_11** **agaaacaaaaaaagaattcgtactctcataactcaagttagataagtttcaacgcccagc**

**Fortunella_japonica_12** **agaaacaaaaaaagaattcgtactctcataactcaagttagataagtttcaacgcccagc**

**Fortunella_margarita_1** **agaaacaaaaaaagaattcgtactctcataactcaagttagataagtttcaacgcccagc**

**Fortunella_margarita_2** **agaaacaaaaaaagaattcgtactctcataactcaagttagataagtttcaacgcccagc**

**Fortunella_venosa_1** **agaaacaaaaaaagaattcgtactctcataactcaagttagataagtttcaacgcccagc**

**Fortunella_venosa_2** **agaaacaaaaaaagaattcgtactctcataactcaagttagataagtttcaacgcccagc**

**Fortunella_venosa_3** **agaaacaaaaaaagaattcgtactctcataactcaagttagataagtttcaacgcccagc**

5710	5720	5730	5740	5750	5760

....|....|....|....|....|....|....|....|....|....|....|....|

**Atalantia_kwangtungens** **cgaaaatccttaggcatttccttttttgagcggtctcaagccccttttttgtttgtctcg**

**Citrus_reticulata_1** **cgaaaatccttaggcatttccttttttgagcggtctcaagccccttttttgtttgtctcg**

**Citrus_reticulata_2** **cgaaaatccttaggcatttccttttttgagcggtctcaagccccttttttgtttgtctcg**

**Citrus_trifoliata_3** **cgaaaatccttaggcatttccttttttgagcggtctcaagccccttttttgtttgtctcg**

**Citrus_aurantiifolia** **cgaaaatccttaggcatttccttttttgagcggtctcaagccccttttttgtttgtctcg**

**Citrus_aurantium** **cgaaaatccttaggcatttccttttttgagcggtctcaagccccttttttgtttgtctcg**

**Citrus_cavaleriei** **cgaaaatccttaggcatttccttttttgagcggtctcaagccccttttttgtttgtctcg**

**Citrus_hongheensis** **cgaaaatccttaggcatttccttttttgagcggtctcaagccccttttttgtttgtctcg**

**Citrus_limon** **cgaaaatccttaggcatttccttttttgagcggtctcaagccccttttttgtttgtctcg**

**Citrus_maxima** **cgaaaatccttaggcatttccttttttgagcggtctcaagccccttttttgtttgtctcg**

**Citrus_medica** **cgaaaatccttaggcatttccttttttgagcggtctcaagccccttttttgtttgtctcg**

**Citrus_polytrifolia** **cgaaaatccttaggcatttccttttttgagcggtctcaagccccttttttgtttgtctcg**

**Citrus_reticulata** **cgaaaatccttaggcatttccttttttgagcggtctcaagccccttttttgtttgtctcg**

**Citrus_sinensis** **cgaaaatccttaggcatttccttttttgagcggtctcaagccccttttttgtttgtctcg**

**Fortunella_bawangica_1** **cgaaaatccttaggcatttccttttttgagcggtctcaagccccttttttgtttgtctcg**

**Fortunella_bawangica_2** **cgaaaatccttaggcatttccttttttgagcggtctcaagccccttttttgtttgtctcg**

**Fortunella_hindsii_1** **cgaaaatccttaggcatttccttttttgagcggtctcaagccccttttttgtttgtctcg**

**Fortunella_hindsii_2** **cgaaaatccttaggcatttccttttttgagcggtctcaagccccttttttgtttgtctcg**

**Fortunella_hindsii_3** **cgaaaatccttaggcatttccttttttgagcggtctcaagccccttttttgtttgtctcg**

**Fortunella_japonica_1** **cgaaaatccttaggcatttccttttttgagcggtctcaagccccttttttgtttgtctcg**

**Fortunella_japonica_2** **cgaaaatccttaggcatttccttttttgagcggtctcaagccccttttttgtttgtctcg**

**Fortunella_japonica_3** **cgaaaatccttaggcatttccttttttgagcggtctcaagccccttttttgtttgtctcg**

**Fortunella_japonica_4** **cgaaaatccttaggcatttccttttttgagcggtctcaagccccttttttgtttgtctcg**

**Fortunella_japonica_5** **cgaaaatccttaggcatttccttttttgagcggtctcaagccccttttttgtttgtctcg**

**Fortunella_japonica_6** **cgaaaatccttaggcatttccttttttgagcggtctcaagccccttttttgtttgtctcg**

**Fortunella_japonica_7** **cgaaaatccttaggcatttccttttttgagcggtctcaagccccttttttgtttgtctcg**

**Fortunella_japonica_8** **cgaaaatccttaggcatttccttttttgagcggtctcaagccccttttttgtttgtctcg**

**Fortunella_japonica_9** **cgaaaatccttaggcatttccttttttgagcggtctcaagccccttttttgtttgtctcg**

**Fortunella_japonica_10** **cgaaaatccttaggcatttccttttttgagcggtctcaagccccttttttgtttgtctcg**

**Fortunella_japonica_11** **cgaaaatccttaggcatttccttttttgagcggtctcaagccccttttttgtttgtctcg**

**Fortunella_japonica_12** **cgaaaatccttaggcatttccttttttgagcggtctcaagccccttttttgtttgtctcg**

**Fortunella_margarita_1** **cgaaaatccttaggcatttccttttttgagcggtctcaagccccttttttgtttgtctcg**

**Fortunella_margarita_2** **cgaaaatccttaggcatttccttttttgagcggtctcaagccccttttttgtttgtctcg**

**Fortunella_venosa_1** **cgaaaatccttaggcatttccttttttgagcggtctcaagccccttttttgtttgtctcg**

**Fortunella_venosa_2** **cgaaaatccttaggcatttccttttttgagcggtctcaagccccttttttgtttgtctcg**

**Fortunella_venosa_3** **cgaaaatccttaggcatttccttttttgagcggtctcaagccccttttttgtttgtctcg**

5770	5780	5790	5800	5810	5820

....|....|....|....|....|....|....|....|....|....|....|....|

**Atalantia_kwangtungens** **tttcgaatcgaatcgatttttggattggattgaattgttgagacaattgaaaaatgggat**

**Citrus_reticulata_1** **tttcgaatcgaatctatttttggattggattgaattgttgagacaattgaaaaatggtat**

**Citrus_reticulata_2** **tttcgaatcgaatctatttttggattggattgaattgttgagacaattgaaaaatggtat**

**Citrus_trifoliata_3** **tttcgaatcgaatctatttttggattggattgaattgttgagacaattgaaaaatggtat**

**Citrus_aurantiifolia** **tttcgaatcgaatctattttttgattggattgaattgttgagacaattgaaaaatggtat**

**Citrus_aurantium** **tttcgaatcgaatctatttttggattggattgaattgttgagacaattgaaaaatggtat**

**Citrus_cavaleriei** **tttcgaatcgaatctatttttggattggattgaattgttgagacaattgaaaaatggtat**

**Citrus_hongheensis** **tttcgaatcgaatctatttttggattggattgaattgttgagacaattgaaaaatggtat**

**Citrus_limon** **tttcgaatcgaatctatttttggattggattgaattgttgagacaattgaaaaatggtat**

**Citrus_maxima** **tttcgaatcgaatctatttttggattggattgaattgttgagacaattgaaaaatggtat**

**Citrus_medica** **tttcgaatcgaatcgatttttggattggattgaattgttgagacaattgaaaaatggtat**

**Citrus_polytrifolia** **tttcgaatcgaatctatttttggattggattgaattgttgagacaattgaaaaatggtat**

**Citrus_reticulata** **tttcgaatcgaatctatttttggattggattgaattgttgagacaattgaaaaatggtat**

**Citrus_sinensis** **tttcgaatcgaatccatttttggattggattgaattgttgagacaattgaaaaatggtat**

**Fortunella_bawangica_1** **tttcgaatcgaatctatttttggattggattgaattgttgagacaattgaaaaatggtat**

**Fortunella_bawangica_2** **tttcgaatcgaatctatttttggattggattgaattgttgagacaattgaaaaatggtat**

**Fortunella_hindsii_1** **tttcgaatcgaatctatttttggattggattgaattgttgagacaattgaaaaatggtat**

**Fortunella_hindsii_2** **tttcgaatcgaatctatttttggattggattgaattgttgagacaattgaaaaatggtat**

**Fortunella_hindsii_3** **tttcgaatcgaatctatttttggattggattgaattgttgagacaattgaaaaatggtat**

**Fortunella_japonica_1** **tttcgaatcgaatctatttttggattggattgaattgttgagacaattgaaaaatggtat**

**Fortunella_japonica_2** **tttcgaatcgaatctatttttggattggattgaattgttgagacaattgaaaaatggtat**

**Fortunella_japonica_3** **tttcgaatcgaatctatttttggattggattgaattgttgagacaattgaaaaatggtat**

**Fortunella_japonica_4** **tttcgaatcgaatctatttttggattggattgaattgttgagacaattgaaaaatggtat**

**Fortunella_japonica_5** **tttcgaatcgaatctatttttggattggattgaattgttgagacaattgaaaaatggtat**

**Fortunella_japonica_6** **tttcgaatcgaatctatttttggattggattgaattgttgagacaattgaaaaatggtat**

**Fortunella_japonica_7** **tttcgaatcgaatctatttttggattggattgaattgttgagacaattgaaaaatggtat**

**Fortunella_japonica_8** **tttcgaatcgaatctatttttggattggattgaattgttgagacaattgaaaaatggtat**

**Fortunella_japonica_9** **tttcgaatcgaatctatttttggattggattgaattgttgagacaattgaaaaatggtat**

**Fortunella_japonica_10** **tttcgaatcgaatctatttttggattggattgaattgttgagacaattgaaaaatggtat**

**Fortunella_japonica_11** **tttcgaatcgaatctatttttggattggattgaattgttgagacaattgaaaaatggtat**

**Fortunella_japonica_12** **tttcgaatcgaatctatttttggattggattgaattgttgagacaattgaaaaatggtat**

**Fortunella_margarita_1** **tttcgaatcgaatctatttttggattggattgaattgttgagacaattgaaaaatggtat**

**Fortunella_margarita_2** **tttcgaatcgaatctatttttggattggattgaattgttgagacaattgaaaaatggtat**

**Fortunella_venosa_1** **tttcgaatcgaatctatttttggattggattgaattgttgagacaattgaaaaatggtat**

**Fortunella_venosa_2** **tttcgaatcgaatctatttttggattggattgaattgttgagacaattgaaaaatggtat**

**Fortunella_venosa_3** **tttcgaatcgaatcgatttttggattggattgaattgttgagacaattgaaaaatggtat**

5830	5840	5850	5860	5870	5880

....|....|....|....|....|....|....|....|....|....|....|....|

**Atalantia_kwangtungens** **ttccttgttccaggatcctttatctttgctttgaatcattaggtttagacattacttcgg**

**Citrus_reticulata_1** **ttccttgttccaggatcctttatctttgctttgaatcattaggtttagacattacttcgg**

**Citrus_reticulata_2** **ttccttgttccaggatcctttatctttgctttgaatcattaggtttagacattacttcgg**

**Citrus_trifoliata_3** **ttccttgttccaggatccgttatctttgctttgaatcattaggtttagacattacttcgg**

**Citrus_aurantiifolia** **ttccttgttccaggatcctttatctttgctttgaatcattaggtttagacattacttcgg**

**Citrus_aurantium** **ttccttgttccaggatcctttatctttgctttgaatcattaggtttagacattacttcgg**

**Citrus_cavaleriei** **ttccttgttccaggatcctttatctttgctttgaatcattaggtttagacattacttcgg**

**Citrus_hongheensis** **ttccttgttccaggatcctttatctttgctttgaatcattaggtttagacattacttcgg**

**Citrus_limon** **ttccttgttccaggatcctttatctttgctttgaatcattaggtttagacattacttcgg**

**Citrus_maxima** **ttccttgttccaggatcctttatctttgctttgaatcattaggtttagacattacttcgg**

**Citrus_medica** **ttccttgttccaggatcctttatctttgctttgaatcattaggtttagacattacttcgg**

**Citrus_polytrifolia** **ttccttgttccaggatccgttatctttgctttgaatcattaggtttagacattacttcgg**

**Citrus_reticulata** **ttccttgttccaggatcctttatctttgctttgaatcattaggtttagacattacttcgg**

**Citrus_sinensis** **ttccttgttccaggatcctttatctttgctttgaatcattaggtttagacattacttcgg**

**Fortunella_bawangica_1** **ttccttgttccaggatcctttatctttgctttgaatcattaggtttagacattacttcgg**

**Fortunella_bawangica_2** **ttccttgttccaggatcctttatctttgctttgaatcattaggtttagacattacttcgg**

**Fortunella_hindsii_1** **ttccttgttccaggatcctttatctttgctttgaatcattaggtttagacattacttcgg**

**Fortunella_hindsii_2** **ttccttgttccaggatcctttatctttgctttgaatcattaggtttagacattacttcgg**

**Fortunella_hindsii_3** **ttccttgttccaggatcctttatctttgctttgaatcattaggtttagacattacttcgg**

**Fortunella_japonica_1** **ttccttgttccaggatcctttatctttgctttgaatcattaggtttagacattacttcgg**

**Fortunella_japonica_2** **ttccttgttccaggatcctttatctttgctttgaatcattaggtttagacattacttcgg**

**Fortunella_japonica_3** **ttccttgttccaggatcctttatctttgctttgaatcattaggtttagacattacttcgg**

**Fortunella_japonica_4** **ttccttgttccaggatcctttatctttgctttgaatcattaggtttagacattacttcgg**

**Fortunella_japonica_5** **ttccttgttccaggatcctttatctttgctttgaatcattaggtttagacattacttcgg**

**Fortunella_japonica_6** **ttccttgttccaggatcctttatctttgctttgaatcattaggtttagacattacttcgg**

**Fortunella_japonica_7** **ttccttgttccaggatcctttatctttgctttgaatcattaggtttagacattacttcgg**

**Fortunella_japonica_8** **ttccttgttccaggatcctttatctttgctttgaatcattaggtttagacattacttcgg**

**Fortunella_japonica_9** **ttccttgttccaggatcctttatctttgctttgaatcattaggtttagacattacttcgg**

**Fortunella_japonica_10** **ttccttgttccaggatcctttatctttgctttgaatcattaggtttagacattacttcgg**

**Fortunella_japonica_11** **ttccttgttccaggatcctttatctttgctttgaatcattaggtttagacattacttcgg**

**Fortunella_japonica_12** **ttccttgttccaggatcctttatctttgctttgaatcattaggtttagacattacttcgg**

**Fortunella_margarita_1** **ttccttgttccaggatcctttatctttgctttgaatcattaggtttagacattacttcgg**

**Fortunella_margarita_2** **ttccttgttccaggatcctttatctttgctttgaatcattaggtttagacattacttcgg**

**Fortunella_venosa_1** **ttccttgttccaggatcctttatctttgctttgaatcattaggtttagacattacttcgg**

**Fortunella_venosa_2** **ttccttgttccaggatcctttatctttgctttgaatcattaggtttagacattacttcgg**

**Fortunella_venosa_3** **ttccttgttccaggatcctttatctttgctttgaatcattaggtttagacattacttcgg**

5890	5900	5910	5920	5930	5940

....|....|....|....|....|....|....|....|....|....|....|....|

**Atalantia_kwangtungens** **tgatctttaacgttttttattttaaaaaatggcagcaacacacccccttttgatttcttt**

**Citrus_reticulata_1** **tgatctttaacgttttttattttaaaaaatggcagcaacacacccccttttgatttcttt**

**Citrus_reticulata_2** **tgatctttaacgttttttattttaaaaaatggcagcaacacacccccttttgatttcttt**

**Citrus_trifoliata_3** **tgatctttaacgttttttattttaaaaaatggcagcaacacacccccttttgatttcttt**

**Citrus_aurantiifolia** **tgatctttaacgttttttattttaaaaaatggcagcaacacacccccttttgatttcttt**

**Citrus_aurantium** **tgatctttaacgttttttattttaaaaaatggcagcaacacacccccttttgatttcttt**

**Citrus_cavaleriei** **tgatctttaacgttttttattttaaaaaatggcagcaacacacccccttttgatttcttt**

**Citrus_hongheensis** **tgatctttaacgttttttattttaaaaaatggcagcaacacacccccttttgatttcttt**

**Citrus_limon** **tgatctttaacgttttttattttaaaaaatggcagcaacacacccccttttgatttcttt**

**Citrus_maxima** **tgatctttaacgttttttattttaaaaaatggcagcaacacacccccttttgatttcttt**

**Citrus_medica** **tgatctttaacgttttttatttttaaaaatggcagcaacacacccccttttgatttcttt**

**Citrus_polytrifolia** **tgatctttaacgttttttattttaaaaaatggcagcaacacacccccttttgatttcttt**

**Citrus_reticulata** **tgatctttaacgttttttattttaaaaaatggcagcaacacacccccttttgatttcttt**

**Citrus_sinensis** **tgatctttaacgttttttattttaaaaaatggcagcaacacacccccttttgatttcttt**

**Fortunella_bawangica_1** **tgatctttaacgttttttattttaaaaaatggcagcaacacacccccttttgatttcttt**

**Fortunella_bawangica_2** **tgatctttaacgttttttattttaaaaaatggcagcaacacacccccttttgatttcttt**

**Fortunella_hindsii_1** **tgatctttaacgttttttattttaaaaaatggcagcaacacacccccttttgatttcttt**

**Fortunella_hindsii_2** **tgatctttaacgttttttattttaaaaaatggcagcaacacacccccttttgatttcttt**

**Fortunella_hindsii_3** **tgatctttaacgttttttattttaaaaaatggcagcaacacacccccttttgatttcttt**

**Fortunella_japonica_1** **tgatctttaacgttttttattttaaaaaatggcagcaacacacccccttttgatttcttt**

**Fortunella_japonica_2** **tgatctttaacgttttttattttaaaaaatggcagcaacacacccccttttgatttcttt**

**Fortunella_japonica_3** **tgatctttaacgttttttattttaaaaaatggcagcaacacacccccttttgatttcttt**

**Fortunella_japonica_4** **tgatctttaacgttttttattttaaaaaatggcagcaacacacccccttttgatttcttt**

**Fortunella_japonica_5** **tgatctttaacgttttttattttaaaaaatggcagcaacacacccccttttgatttcttt**

**Fortunella_japonica_6** **tgatctttaacgttttttattttaaaaaatggcagcaacacacccccttttgatttcttt**

**Fortunella_japonica_7** **tgatctttaacgttttttattttaaaaaatggcagcaacacacccccttttgatttcttt**

**Fortunella_japonica_8** **tgatctttaacgttttttattttaaaaaatggcagcaacacacccccttttgatttcttt**

**Fortunella_japonica_9** **tgatctttaacgttttttattttaaaaaatggcagcaacacacccccttttgatttcttt**

**Fortunella_japonica_10** **tgatctttaacgttttttattttaaaaaatggcagcaacacacccccttttgatttcttt**

**Fortunella_japonica_11** **tgatctttaacgttttttattttaaaaaatggcagcaacacacccccttttgatttcttt**

**Fortunella_japonica_12** **tgatctttaacgttttttattttaaaaaatggcagcaacacacccccttttgatttcttt**

**Fortunella_margarita_1** **tgatctttaacgttttttattttaaaaaatggcagcaacacacccccttttgatttcttt**

**Fortunella_margarita_2** **tgatctttaacgttttttattttaaaaaatggcagcaacacacccccttttgatttcttt**

**Fortunella_venosa_1** **tgatctttaacgttttttattttaaaaaatggcagcaacacacccccttttgatttcttt**

**Fortunella_venosa_2** **tgatctttaacgttttttattttaaaaaatggcagcaacacacccccttttgatttcttt**

**Fortunella_venosa_3** **tgatctttaacgttttttattttaaaaaatggcagcaacacacccccttttgatttcttt**

5950	5960	5970	5980	5990	6000

....|....|....|....|....|....|....|....|....|....|....|....|

**Atalantia_kwangtungens** **ctatcaaagaatcatacgaacaattgattcctacaggatacacttttggtagaaaaagtt**

**Citrus_reticulata_1** **ctatcaaagaatcatacgaacaattgattcctacaggatacacttttggtagaaaaagtt**

**Citrus_reticulata_2** **ctatcaaagaatcatacgaacaattgattcctacaggatacacttttggtagaaaaagtt**

**Citrus_trifoliata_3** **ctatcaaagaatcatacgaacaattgattcctacaggatacacttttggtagaaaaagtt**

**Citrus_aurantiifolia** **ttatcaaagaatcatacgaacaattgattcctacaggatacacttttggtagaaaaagtt**

**Citrus_aurantium** **ctatcaaagaatcatacgaacaattgattcctacaggatacacttttggtagaaaaagtt**

**Citrus_cavaleriei** **ctatcaaagaatcatacgaacaattgattcctacaggatacacttttggtagaaaaagtt**

**Citrus_hongheensis** **ctatcaaagaatcatacgaacaattgattcctacaggatacacttttggtagaaaaagtt**

**Citrus_limon** **ctatcaaagaatcatacgaacaattgattcctacaggatacacttttggtagaaaaagtt**

**Citrus_maxima** **ctatcaaagaatcatacgaacaattgattcctacaggatacacttttggtagaaaaagtt**

**Citrus_medica** **ctatcaaagaatcataggaacaattgattcctacaggatacacttttggtagaaaaagtt**

**Citrus_polytrifolia** **ctatcaaagaatcatacgaacaattgattcctacaggatacacttttggtagaaaaagtt**

**Citrus_reticulata** **ctatcaaagaatcatacgaacaattgattcctacaggatacacttttggtagaaaaagtt**

**Citrus_sinensis** **ctatcaaagaatcatacgaacaattgattcctacaggatacacttttggtagaaaaagtt**

**Fortunella_bawangica_1** **ctatcaaagaatcatacgaacaattgattcctacaggatacacttttggtagaaaaagtt**

**Fortunella_bawangica_2** **ctatcaaagaatcatacgaacaattgattcctacaggatacacttttggtagaaaaagtt**

**Fortunella_hindsii_1** **ctatcaaagaatcatacgaacaattgattcctacaggatacacttttggtagaaaaagtt**

**Fortunella_hindsii_2** **ctatcaaagaatcatacgaacaattgattcctacaggatacacttttggtagaaaaagtt**

**Fortunella_hindsii_3** **ctatcaaagaatcatacgaacaattgattcctacaggatacacttttggtagaaaaagtt**

**Fortunella_japonica_1** **ctatcaaagaatcatacgaacaattgattcctacaggatacacttttggtagaaaaagtt**

**Fortunella_japonica_2** **ctatcaaagaatcatacgaacaattgattcctacaggatacacttttggtagaaaaagtt**

**Fortunella_japonica_3** **ctatcaaagaatcatacgaacaattgattcctacaggatacacttttggtagaaaaagtt**

**Fortunella_japonica_4** **ctatcaaagaatcatacgaacaattgattcctacaggatacacttttggtagaaaaagtt**

**Fortunella_japonica_5** **ctatcaaagaatcatacgaacaattgattcctacaggatacacttttggtagaaaaagtt**

**Fortunella_japonica_6** **ctatcaaagaatcatacgaacaattgattcctacaggatacacttttggtagaaaaagtt**

**Fortunella_japonica_7** **ctatcaaagaatcatacgaacaattgattcctacaggatacacttttggtagaaaaagtt**

**Fortunella_japonica_8** **ctatcaaagaatcatacgaacaattgattcctacaggatacacttttggtagaaaaagtt**

**Fortunella_japonica_9** **ctatcaaagaatcatacgaacaattgattcctacaggatacacttttggtagaaaaagtt**

**Fortunella_japonica_10** **ctatcaaagaatcatacgaacaattgattcctacaggatacacttttggtagaaaaagtt**

**Fortunella_japonica_11** **ctatcaaagaatcatacgaacaattgattcctacaggatacacttttggtagaaaaagtt**

**Fortunella_japonica_12** **ctatcaaagaatcatacgaacaattgattcctacaggatacacttttggtagaaaaagtt**

**Fortunella_margarita_1** **ctatcaaagaatcatacgaacaattgattcctacaggatacacttttggtagaaaaagtt**

**Fortunella_margarita_2** **ctatcaaagaatcatacgaacaattgattcctacaggatacacttttggtagaaaaagtt**

**Fortunella_venosa_1** **atatcaaagaatcatacgaacaattgattcctacaggatacacttttggtagaaaaagtt**

**Fortunella_venosa_2** **atatcaaagaatcatacgaacaattgattcctacaggatacacttttggtagaaaaagtt**

**Fortunella_venosa_3** **ctatcaaagaatcatacgaacaattgattcctacaggatacacttttggtagaaaaagtt**

6010	6020	6030	6040	6050	6060

....|....|....|....|....|....|....|....|....|....|....|....|

**Atalantia_kwangtungens** **ttcccaattccaacaaattttccccttgcttttggatttcaaaattgctcgaatcagatc**

**Citrus_reticulata_1** **ttcccaattccaacaaattttccccttgcttttggatttcaaaattgctcgaatcagatc**

**Citrus_reticulata_2** **ttcccaattccaacaaattttccccttgcttttggatttcaaaattgctcgaatcagatc**

**Citrus_trifoliata_3** **ttcccaattccaacaaattttacccttgcttttggatttcaaaattgctcgaatcagatc**

**Citrus_aurantiifolia** **ttcccaattccaacaaattttccccttgcttttggatttcaaaattgctcgaatcagatc**

**Citrus_aurantium** **ttcccaattccaacaaattttccccttgcttttggatttcaaaattgctcgaatcagatc**

**Citrus_cavaleriei** **ttcccaattccaacaaattttccccttgcttttggatttcaaaattgctcgaatcagatc**

**Citrus_hongheensis** **ttcccaattccaacaaattttccccttgcttttggatttcaaaattgctcgaatcagatc**

**Citrus_limon** **ttcccaattccaacaaattttccccttgcttttggatttcaaaattgctcgaatcagatc**

**Citrus_maxima** **ttcccaattccaacaaattttccccttgcttttggatttcaaaattgctcgaatcagatc**

**Citrus_medica** **ttcccaattccaacaaaatttccccttgcttttggatttcaaaattgctcgaatcagatc**

**Citrus_polytrifolia** **ttcccaattccaacaaattttccccttgcttttggatttcaaaattgctcgaatcagatc**

**Citrus_reticulata** **ttcccaattccaacaaattttccccttgcttttggatttcaaaattgctcgaatcagatc**

**Citrus_sinensis** **ttcccaattccaacaaattttccccttgcttttggatttcaaaattgctcgaatcagatc**

**Fortunella_bawangica_1** **ttcccaattccaacaaattttccccttgcttttggatttcaaaattgctcgaatcagatc**

**Fortunella_bawangica_2** **ttcccaattccaacaaattttccccttgcttttggatttcaaaattgctcgaatcagatc**

**Fortunella_hindsii_1** **ttcccaattccaacaaattttccccttgcttttggatttcaaaattgctcgaatcagatc**

**Fortunella_hindsii_2** **ttcccaattccaacaaattttccccttgcttttggatttcaaaattgctcgaatcagatc**

**Fortunella_hindsii_3** **ttcccaattccaacaaattttccccttgcttttggatttcaaaattgctcgaatcagatc**

**Fortunella_japonica_1** **ttcccaattccaacaaattttccccttgcttttggatttcaaaattgctcgaatcagatc**

**Fortunella_japonica_2** **ttcccaattccaacaaattttccccttgcttttggatttcaaaattgctcgaatcagatc**

**Fortunella_japonica_3** **ttcccaattccaacaaattttccccttgcttttggatttcaaaattgctcgaatcagatc**

**Fortunella_japonica_4** **ttcccaattccaacaaattttccccttgcttttggatttcaaaattgctcgaatcagatc**

**Fortunella_japonica_5** **ttcccaattccaacaaattttccccttgcttttggatttcaaaattgctcgaatcagatc**

**Fortunella_japonica_6** **ttcccaattccaacaaattttccccttgcttttggatttcaaaattgctcgaatcagatc**

**Fortunella_japonica_7** **ttcccaattccaacaaattttccccttgcttttggatttcaaaattgctcgaatcagatc**

**Fortunella_japonica_8** **ttcccaattccaacaaattttccccttgcttttggatttcaaaattgctcgaatcagatc**

**Fortunella_japonica_9** **ttcccaattccaacaaattttccccttgcttttggatttcaaaattgctcgaatcagatc**

**Fortunella_japonica_10** **ttcccaattccaacaaattttccccttgcttttggatttcaaaattgctcgaatcagatc**

**Fortunella_japonica_11** **ttcccaattccaacaaattttccccttgcttttggatttcaaaattgctcgaatcagatc**

**Fortunella_japonica_12** **ttcccaattccaacaaattttccccttgcttttggatttcaaaattgctcgaatcagatc**

**Fortunella_margarita_1** **ttcccaattccaacaaattttccccttgcttttggatttcaaaattgctcgaatcagatc**

**Fortunella_margarita_2** **ttcccaattccaacaaattttccccttgcttttggatttcaaaattgctcgaatcagatc**

**Fortunella_venosa_1** **ttcccaattccaacaaattttccccttacttttggatttcaaaattgctcgaatcagatc**

**Fortunella_venosa_2** **ttcccaattccaacaaattttccccttgcttttggatttcaaaattgctcgaatcagatc**

**Fortunella_venosa_3** **ttcccaattccaacaaattttccccttgcttttggatttcaaaattgctcgaatcagatc**

6070	6080	6090	6100	6110	6120

....|....|....|....|....|....|....|....|....|....|....|....|

**Atalantia_kwangtungens** **ctttcgatttctatatcgaaaattgacttacgaagttttttccaacttattgattggtat**

**Citrus_reticulata_1** **ctttcgatttctatatcgaaaatttacttacgaagttttttccaacttattgattggtat**

**Citrus_reticulata_2** **ctttcgatttctatatcgaaaatttacttacgaagttttttccaacttattgattggtat**

**Citrus_trifoliata_3** **ctttcgatttctatatcgaaaatttacttacgaagttttttccaacttattgattggtat**

**Citrus_aurantiifolia** **ctttcgatttctatatcgaaaatttacttacgaagttttttccaacttattgattggtat**

**Citrus_aurantium** **ctttcgatttctatatcgaaaatttacttacgaagttttttccaacttattgattggtat**

**Citrus_cavaleriei** **ctttcgatttctatatcgaaaatttacttacgaagttttttccaacttattgattggtat**

**Citrus_hongheensis** **ctttcgatttctatatcgaaaatttacttacgaagttttttccaacttattgattggtat**

**Citrus_limon** **ctttcgatttctatatcgaaaatttacttacgaagttttttccaacttattgattggtat**

**Citrus_maxima** **ctttcgatttctatatcgaaaatttacttacgaagttttttccaacttattgattggtat**

**Citrus_medica** **ctttcgatttctatatcgaaaatttacttacgaagttttttccaacttattgattggtat**

**Citrus_polytrifolia** **ctttcgatttctatatcgaaaatttacttacgaagttttttccaacttattgattggtat**

**Citrus_reticulata** **ctttcgatttctatatcgaaaatttacttacgaagttttttccaacttattgattggtat**

**Citrus_sinensis** **ctttcgatttctatatcgaaaatttacttacgaagttttttccaacttattgattggtat**

**Fortunella_bawangica_1** **ctttcgatttctatatcgaaaatttacttacgaagttttttccaacttattgattggtat**

**Fortunella_bawangica_2** **ctttcgatttctatatcgaaaatttacttacgaagttttttccaacttattgattggtat**

**Fortunella_hindsii_1** **ctttcgatttctatatcgaaaatttacttacgaagttttttccaacttattgattggtat**

**Fortunella_hindsii_2** **ctttcgatttctatatcgaaaatttacttacgaagttttttccaacttattgattggtat**

**Fortunella_hindsii_3** **ctttcgatttctatatcgaaaatttacttacgaagttttttccaacttattgattggtat**

**Fortunella_japonica_1** **ctttcgatttctatatcgaaaatttacttacgaagttttttccaacttattgattggtat**

**Fortunella_japonica_2** **ctttcgatttctatatcgaaaatttacttacgaagttttttccaacttattgattggtat**

**Fortunella_japonica_3** **ctttcgatttctatatcgaaaatttacttacgaagttttttccaacttattgattggtat**

**Fortunella_japonica_4** **ctttcgatttctatatcgaaaatttacttacgaagttttttccaacttattgattggtat**

**Fortunella_japonica_5** **ctttcgatttctatatcgaaaatttacttacgaagttttttccaacttattgattggtat**

**Fortunella_japonica_6** **ctttcgatttctatatcgaaaatttacttacgaagttttttccaacttattgattggtat**

**Fortunella_japonica_7** **ctttcgatttctatatcgaaaatttacttacgaagttttttccaacttattgattggtat**

**Fortunella_japonica_8** **ctttcgatttctatatcgaaaatttacttacgaagttttttccaacttattgattggtat**

**Fortunella_japonica_9** **ctttcgatttctatatcgaaaatttacttacgaagttttttccaacttattgattggtat**

**Fortunella_japonica_10** **ctttcgatttctatatcgaaaatttacttacgaagttttttccaacttattgattggtat**

**Fortunella_japonica_11** **ctttcgatttctatatcgaaaatttacttacgaagttttttccaacttattgattggtat**

**Fortunella_japonica_12** **ctttcgatttctatatcgaaaatttacttacgaagttttttccaacttattgattggtat**

**Fortunella_margarita_1** **ctttcgatttctatatcgaaaatttacttacgaagttttttccaacttattgattggtat**

**Fortunella_margarita_2** **ctttcgatttctatatcgaaaatttacttacgaagttttttccaacttattgattggtat**

**Fortunella_venosa_1** **ctttcgatttctatatcgaaaatttacttacgaagttttttccaacttattgattggtat**

**Fortunella_venosa_2** **ctttcgatttctatatcgaaaatttacttacgaagttttttccaacttattgattggtat**

**Fortunella_venosa_3** **ctttcgatttctatatcgaaaatttacttacgaagttttttccaacttattgattggtat**

6130	6140	6150	6160	6170	6180

....|....|....|....|....|....|....|....|....|....|....|....|

**Atalantia_kwangtungens** **taaccctagatccttgcccctgagaaatgaataaatactttatactcgagctccatcctg**

**Citrus_reticulata_1** **taaccctagatccttgcccctgagaaaggaataaatactttatactcgagctccatcctg**

**Citrus_reticulata_2** **taaccctagatccttgcccctgagaaaggaataaatactttatactcgagctccatcctg**

**Citrus_trifoliata_3** **taaccctagatccttgcccctgagaaaggaataaatactttatactcgagctccatcctg**

**Citrus_aurantiifolia** **taaccctagatccttgcccctgagaaaggaataaatactttatactcgagctccatcctg**

**Citrus_aurantium** **taaccctagatccgtgcccctgagaaaggaataaatactttatactcgagctccatcctg**

**Citrus_cavaleriei** **taaccctagatccttgcccctgagaaaggaataaatactttatactcgagctccatcctg**

**Citrus_hongheensis** **taaccctagatccgtgcccctgagaaaggaataaatactttatactcgagctccatcctg**

**Citrus_limon** **taaccctagatccgtgcccctgagaaaggaataaatactttatactcgagctccatcctg**

**Citrus_maxima** **taaccctagatccgtgcccctgagaaaggaataaatactttatactcgagctccatcctg**

**Citrus_medica** **taaccctagatccttgcccctgagaaaggaataaatactttatactcgagctccatcctg**

**Citrus_polytrifolia** **taaccctagatccttgcccctgagaaaggaataaatactttatactcgagctccatcctg**

**Citrus_reticulata** **taaccctagatccttgcccctgagaaaggaataaatactttatactcgagctccatcctg**

**Citrus_sinensis** **taaccctagatccgtgcccctgagaaaggaataaatactttatactcgagctccatcctg**

**Fortunella_bawangica_1** **taaccctagatccttgcccctgagaaaggaataaatactttatactcgagctccatcctg**

**Fortunella_bawangica_2** **taaccctagatccttgcccctgagaaaggaataaatactttatactcgagctccatcctg**

**Fortunella_hindsii_1** **taaccctagatccttgcccctgagaaaggaataaatactttatactcgagctccatcctg**

**Fortunella_hindsii_2** **taaccctagatccttgcccctgagaaaggaataaatactttatactcgagctccatcctg**

**Fortunella_hindsii_3** **taaccctagatccttgcccctgagaaaggaataaatactttatactcgagctccatcctg**

**Fortunella_japonica_1** **taaccctagatccttgcccctgagaaaggaataaatactttatactcgagctccatcctg**

**Fortunella_japonica_2** **taaccctagatccttgcccctgagaaaggaataaatactttatactcgagctccatcctg**

**Fortunella_japonica_3** **taaccctagatccttgcccctgagaaaggaataaatactttatactcgagctccatcctg**

**Fortunella_japonica_4** **taaccctagatccttgcccctgagaaaggaataaatactttatactcgagctccatcctg**

**Fortunella_japonica_5** **taaccctagatccttgcccctgagaaaggaataaatactttatactcgagctccatcctg**

**Fortunella_japonica_6** **taaccctagatccttgcccctgagaaaggaataaatactttatactcgagctccatcctg**

**Fortunella_japonica_7** **taaccctagatccttgcccctgagaaaggaataaatactttatactcgagctccatcctg**

**Fortunella_japonica_8** **taaccctagatccttgcccctgagaaaggaataaatactttatactcgagctccatcctg**

**Fortunella_japonica_9** **taaccctagatccttgcccctgagaaaggaataaatactttatactcgagctccatcctg**

**Fortunella_japonica_10** **taaccctagatccttgcccctgagaaaggaataaatactttatactcgagctccatcctg**

**Fortunella_japonica_11** **taaccctagatccttgcccctgagaaaggaataaatactttatactcgagctccatcctg**

**Fortunella_japonica_12** **taaccctagatccttgcccctgagaaaggaataaatactttatactcgagctccatcctg**

**Fortunella_margarita_1** **taaccctagatccttgcccctgagaaaggaataaatactttatactcgagctccatcctg**

**Fortunella_margarita_2** **taaccctagatccttgcccctgagaaaggaataaatactttatactcgagctccatcctg**

**Fortunella_venosa_1** **taaccctagatccttgcccctgagaaaggaataaatactttatactcgagctccatcctg**

**Fortunella_venosa_2** **taaccctagatccttgcccctgagaaaggaataaatactttatactcgagctccatcctg**

**Fortunella_venosa_3** **taaccctagatccttgcccctgagaaaggaataaatactttatactcgagctccatcctg**

6190	6200	6210	6220	6230	6240

....|....|....|....|....|....|....|....|....|....|....|....|

**Atalantia_kwangtungens** **tactagttcccttaccccccc---aaaaaacttgaggattctaatagaacagaagaaaaa**

**Citrus_reticulata_1** **tactagttccattaccccccc--caaaaaacttgaggattctaatagaactgaagaaaaa**

**Citrus_reticulata_2** **tactagttccattaccccccc--caaaaaacttgaggattctaatagaactgaagaaaaa**

**Citrus_trifoliata_3** **tactagttccattaccccccccaaaaaaaacttgaggattctaatagaacagaagaaaaa**

**Citrus_aurantiifolia** **tactagttccattaccccccc--aaaaaaacttgaggattctaatagaacagaagaaaaa**

**Citrus_aurantium** **tactagttccattaccccccc--aaaaaaacttgaggattctaatagaacagaagaaaaa**

**Citrus_cavaleriei** **tactagttccattaccccccc--aaaaaaacttgaggattctaatagaacagaagaaaaa**

**Citrus_hongheensis** **tactagttccattaccccccc--aaaaaaacttgaggattctaatagaacagaagaaaaa**

**Citrus_limon** **tactagttccattaccccccc--aaaaaaacttgaggattctaatagaacagaagaaaaa**

**Citrus_maxima** **tactagttccattaccccccc--aaaaaaacttgaggattctaatagaacagaagaaaaa**

**Citrus_medica** **tactagttccattacctcccc--aaaaaaacttgaggattctaatagaaccgaagaaaaa**

**Citrus_polytrifolia** **tactagttccattacccccccc-aaaaaaacttgaggattctaatagaacagaagaaaaa**

**Citrus_reticulata** **tactagttccattaccccccc--caaaaaacttgaggattctaatagaactgaagaaaaa**

**Citrus_sinensis** **tactagttccattaccccccc--aaaaaaacttgaggattctaatagaacagaagaaaaa**

**Fortunella_bawangica_1** **tactagttccattaccccccc--aaaaaaacttgaggattctaatagaacagaagaaaaa**

**Fortunella_bawangica_2** **tactagttccattaccccccc--aaaaaaacttgaggattctaatagaacagaagaaaaa**

**Fortunella_hindsii_1** **tactagttccattaccccccc--aaaaaaacttgaggattctaatagaacagaagaaaaa**

**Fortunella_hindsii_2** **tactagttccattaccccccc--aaaaaaacttgaggattctaatagaacagaagaaaaa**

**Fortunella_hindsii_3** **tactagttccattaccccccc--aaaaaaacttgaggattctaatagaacagaagaaaaa**

**Fortunella_japonica_1** **tactagttccattaccccccc--aaaaaaacttgaggattctaatagaacagaagaaaaa**

**Fortunella_japonica_2** **tactagttccattaccccccc--aaaaaaacttgaggattctaatagaacagaagaaaaa**

**Fortunella_japonica_3** **tactagttccattaccccccc--aaaaaaacttgaggattctaatagaacagaagaaaaa**

**Fortunella_japonica_4** **tactagttccattaccccccc--aaaaaaacttgaggattctaatagaacagaagaaaaa**

**Fortunella_japonica_5** **tactagttccattaccccccc--aaaaaaacttgaggattctaatagaacagaagaaaaa**

**Fortunella_japonica_6** **tactagttccattaccccccc--aaaaaaacttgaggattctaatagaacagaagaaaaa**

**Fortunella_japonica_7** **tactagttccattaccccccc--aaaaaaacttgaggattctaatagaacagaagaaaaa**

**Fortunella_japonica_8** **tactagttccattaccccccc--aaaaaaacttgaggattctaatagaacagaagaaaaa**

**Fortunella_japonica_9** **tactagttccattaccccccc--aaaaaaacttgaggattctaatagaacagaagaaaaa**

**Fortunella_japonica_10** **tactagttccattaccccccc--aaaaaaacttgaggattctaatagaacagaagaaaaa**

**Fortunella_japonica_11** **tactagttccattaccccccc--aaaaaaacttgaggattctaatagaacagaagaaaaa**

**Fortunella_japonica_12** **tactagttccattaccccccc--aaaaaaacttgaggattctaatagaacagaagaaaaa**

**Fortunella_margarita_1** **tactagttccattaccccccc--aaaaaaacttgaggattctaatagaacagaagaaaaa**

**Fortunella_margarita_2** **tactagttccattaccccccc--aaaaaaacttgaggattctaatagaacagaagaaaaa**

**Fortunella_venosa_1** **tactagttccattaccccccc--aaaaaaacttgaggattctaatagaacagaagaaaaa**

**Fortunella_venosa_2** **tactagttccattaccccccc--aaaaaaacttgaggattctaatagaacagaagaaaaa**

**Fortunella_venosa_3** **tactagttccattaccccccc--aaaaaaacttgaggattctaatagaacagaagaaaaa**

6250	6260	6270	6280	6290	6300

....|....|....|....|....|....|....|....|....|....|....|....|

**Atalantia_kwangtungens** **atgtcgagccaagagcccattcatataaaatcgaaaacggtggatgtaaaaaacaaatcc**

**Citrus_reticulata_1** **atgtcgagccaagagcccattcatataaaatagaaaacggtggatgtaaaaaacaaatcc**

**Citrus_reticulata_2** **atgtcgagccaagagcccattcatataaaatagaaaacggtggatgtaaaaaacaaatcc**

**Citrus_trifoliata_3** **atgtcgagccaagagcccattcatataaaatcgaaaacggtggatgtaaaaaacaaatcc**

**Citrus_aurantiifolia** **atgtcgagccaagagcccattcatataaaatcgaaaacggtggatgtaaaaaacaaatcc**

**Citrus_aurantium** **atgtcgagccaagagcccattcatataaaatcgaaaatggtggatgtaaaaaacaaatcc**

**Citrus_cavaleriei** **atgtcgagccaagagcccattcatataaaatcgaaaacggtggatgtaaaaaacaaatcc**

**Citrus_hongheensis** **atgtcgagccaagagcccattcatataaaatcgaaaatggtggatgtaaaaaacaaatcc**

**Citrus_limon** **atgtcgagccaagagcccattcatataaaatcgaaaatggtggatgtaaaaaacaaatcc**

**Citrus_maxima** **atgtcgagccaagagcccattcatataaaatcgaaaatggtggatgtaaaaaacaaatcc**

**Citrus_medica** **atgtcgagccaagagccccttcagataaaatcgaaaacggtggatgtaaaaaacaaatcc**

**Citrus_polytrifolia** **atgtcgagccaagagcccattcatataaaatcgaaaacggtggatgtaaaaaacaaatcc**

**Citrus_reticulata** **atgtcgagccaagagcccattcatataaaatagaaaacggtggatgtaaaaaacaaatcc**

**Citrus_sinensis** **atgtcgagccaagagcccattcatataaaatcgaaaatggtggatgtaaaaaacaaatcc**

**Fortunella_bawangica_1** **atgtcgagccaagagcccattcatataaaatcgaaaacggtggatgtaaaaaacaaatcc**

**Fortunella_bawangica_2** **atgtcgagccaagagcccattcatataaaatcgaaaacggtggatgtaaaaaacaaatcc**

**Fortunella_hindsii_1** **atgtcgagccaagagcccattcatataaaatcgaaaacggtggatgtaaaaaacaaatcc**

**Fortunella_hindsii_2** **atgtcgagccaagagcccattcatataaaatcgaaaacggtggatgtaaaaaacaaatcc**

**Fortunella_hindsii_3** **atgtcgagccaagagcccattcatataaaatcgaaaacggtggatgtaaaaaacaaatcc**

**Fortunella_japonica_1** **atgtcgagccaagagcccattcatataaaatcgaaaacggtggatgtaaaaaacaaatcc**

**Fortunella_japonica_2** **atgtcgagccaagagcccattcatataaaatcgaaaacggtggatgtaaaaaacaaatcc**

**Fortunella_japonica_3** **atgtcgagccaagagcccattcatataaaatcgaaaacggtggatgtaaaaaacaaatcc**

**Fortunella_japonica_4** **atgtcgagccaagagcccattcatataaaatcgaaaacggtggatgtaaaaaacaaatcc**

**Fortunella_japonica_5** **atgtcgagccaagagcccattcatataaaatcgaaaacggtggatgtaaaaaacaaatcc**

**Fortunella_japonica_6** **atgtcgagccaagagcccattcatataaaatcgaaaacggtggatgtaaaaaacaaatcc**

**Fortunella_japonica_7** **atgtcgagccaagagcccattcatataaaatcgaaaacggtggatgtaaaaaacaaatcc**

**Fortunella_japonica_8** **atgtcgagccaagagcccattcatataaaatcgaaaacggtggatgtaaaaaacaaatcc**

**Fortunella_japonica_9** **atgtcgagccaagagcccattcatataaaatcgaaaacggtggatgtaaaaaacaaatcc**

**Fortunella_japonica_10** **atgtcgagccaagagcccattcatataaaatcgaaaacggtggatgtaaaaaacaaatcc**

**Fortunella_japonica_11** **atgtcgagccaagagcccattcatataaaatcgaaaacggtggatgtaaaaaacaaatcc**

**Fortunella_japonica_12** **atgtcgagccaagagcccattcatataaaatcgaaaacggtggatgtaaaaaacaaatcc**

**Fortunella_margarita_1** **atgtcgagccaagagcccattcatataaaatcgaaaacggtggatgtaaaaaacaaatcc**

**Fortunella_margarita_2** **atgtcgagccaagagcccattcatataaaatcgaaaacggtggatgtaaaaaacaaatcc**

**Fortunella_venosa_1** **atgtcgagccaagagcccattcatataaaatcgaaaacggtggatgtaaaaaacaaatcc**

**Fortunella_venosa_2** **atgtcgagccaagagcccattcatataaaatcgaaaacggtggatgtaaaaaacaaatcc**

**Fortunella_venosa_3** **atgtcgagccaagagcccattcatataaaatcgaaaacggtggatgtaaaaaacaaatcc**

6310	6320	6330	6340	6350	6360

....|....|....|....|....|....|....|....|....|....|....|....|

**Atalantia_kwangtungens** **acagcggatcatgtccttcaagtcgcacgttgctttctaccacatcgtttcaaacgaagt**

**Citrus_reticulata_1** **acagcggatcatgtccttcaagtcgcacgttgctttctaccacatcgtttcaaacgaagt**

**Citrus_reticulata_2** **acagcggatcatgtccttcaagtcgcacgttgctttctaccacatcgtttcaaacgaagt**

**Citrus_trifoliata_3** **acagcggatcatgtccttcaagtcgcacgttgctttctaccacatcgtttcaaacgaagt**

**Citrus_aurantiifolia** **acagcggatcatgtccttcaagtcgcacgttgctttctaccacatcgtttcaaacgaagt**

**Citrus_aurantium** **acagcggatcatgtccttcaagtcgcacgttgctttctaccacatcgtttcaaacgaagt**

**Citrus_cavaleriei** **acagcggatcatgtccttcaagtcgcacgttgctttctaccacatcgtttcaaacgaagt**

**Citrus_hongheensis** **acagcggatcatgtccttcaagtcgcacgttgctttctaccacatcgtttcaaacgaagt**

**Citrus_limon** **acagcggatcatgtccttcaagtcgcacgttgctttctaccacatcgtttcaaacgaagt**

**Citrus_maxima** **acagcggatcatgtccttcaagtcgcacgttgctttctaccacatcgtttcaaacgaagt**

**Citrus_medica** **acagcggatcatgtccttcaagtcgcacgttgctttctaccacatcgtttcaaacgaagt**

**Citrus_polytrifolia** **acagcggatcatgtccttcaagtcgcacgttgctttctaccacatcgtttcaaacgaagt**

**Citrus_reticulata** **acagcggatcatgtccttcaagtcgcacgttgctttctaccacatcgtttcaaacgaagt**

**Citrus_sinensis** **acagcggatcatgtccttcaagtcgcacgttgctttctaccacatcgtttcaaacgaagt**

**Fortunella_bawangica_1** **acagcggatcatgtccttcaagtcgcacgttgctttctaccacatcgtttcaaacgaagt**

**Fortunella_bawangica_2** **acagcggatcatgtccttcaagtcgcacgttgctttctaccacatcgtttcaaacgaagt**

**Fortunella_hindsii_1** **acagcggatcatgtccttcaagtcgcacgttgctttctaccacatcgtttcaaacgaagt**

**Fortunella_hindsii_2** **acagcggatcatgtccttcaagtcgcacgttgctttctaccacatcgtttcaaacgaagt**

**Fortunella_hindsii_3** **acagcggatcatgtccttcaagtcgcacgttgctttctaccacatcgtttcaaacgaagt**

**Fortunella_japonica_1** **acagcggatcatgtccttcaagtcgcacgttgctttctaccacatcgtttcaaacgaagt**

**Fortunella_japonica_2** **acagcggatcatgtccttcaagtcgcacgttgctttctaccacatcgtttcaaacgaagt**

**Fortunella_japonica_3** **acagcggatcatgtccttcaagtcgcacgttgctttctaccacatcgtttcaaacgaagt**

**Fortunella_japonica_4** **acagcggatcatgtccttcaagtcgcacgttgctttctaccacatcgtttcaaacgaagt**

**Fortunella_japonica_5** **acagcggatcatgtccttcaagtcgcacgttgctttctaccacatcgtttcaaacgaagt**

**Fortunella_japonica_6** **acagcggatcatgtccttcaagtcgcacgttgctttctaccacatcgtttcaaacgaagt**

**Fortunella_japonica_7** **acagcggatcatgtccttcaagtcgcacgttgctttctaccacatcgtttcaaacgaagt**

**Fortunella_japonica_8** **acagcggatcatgtccttcaagtcgcacgttgctttctaccacatcgtttcaaacgaagt**

**Fortunella_japonica_9** **acagcggatcatgtccttcaagtcgcacgttgctttctaccacatcgtttcaaacgaagt**

**Fortunella_japonica_10** **acagcggatcatgtccttcaagtcgcacgttgctttctaccacatcgtttcaaacgaagt**

**Fortunella_japonica_11** **acagcggatcatgtccttcaagtcgcacgttgctttctaccacatcgtttcaaacgaagt**

**Fortunella_japonica_12** **acagcggatcatgtccttcaagtcgcacgttgctttctaccacatcgtttcaaacgaagt**

**Fortunella_margarita_1** **acagcggatcatgtccttcaagtcgcacgttgctttctaccacatcgtttcaaacgaagt**

**Fortunella_margarita_2** **acagcggatcatgtccttcaagtcgcacgttgctttctaccacatcgtttcaaacgaagt**

**Fortunella_venosa_1** **acagcggatcatgtccttcaagtcgcacgttgctttctaccacatcgtttcaaacgaagt**

**Fortunella_venosa_2** **acagcggatcatgtccttcaagtcgcacgttgctttctaccacatcgtttcaaacgaagt**

**Fortunella_venosa_3** **acagcggatcatgtccttcaagtcgcacgttgctttctaccacatcgtttcaaacgaagt**

6370	6380	6390	6400	6410	6420

....|....|....|....|....|....|....|....|....|....|....|....|

**Atalantia_kwangtungens** **tttaccataacatttctctagtttggaaccggtatgtaattgattccagtatggaatcat**

**Citrus_reticulata_1** **tttaccataacatttctctagtttggaaccggtatgtaattgattccagtatggaatcat**

**Citrus_reticulata_2** **tttaccataacatttctctagtttggaaccggtatgtaattgattccagtatggaatcat**

**Citrus_trifoliata_3** **tttaccataacatttctctagtttggaaccggtatgtaattgattccagtatggaatcat**

**Citrus_aurantiifolia** **tttaccataacatttctctagtttggaaccggtatgtaattgattccagtatggaatcat**

**Citrus_aurantium** **tttaccataacatttctctagtttggaaccggtatgtaattgattccagtatggaatcat**

**Citrus_cavaleriei** **tttaccataacatttctctagtttggaaccggtatgtaattgattccagtatggaatcat**

**Citrus_hongheensis** **tttaccataacatttctctagtttggaaccggtatgtaattgattccagtatggaatcat**

**Citrus_limon** **tttaccataacatttctctagtttggaaccggtatgtaattgattccagtatggaatcat**

**Citrus_maxima** **tttaccataacatttctctagtttggaaccggtatgtaattgattccagtatggaatcat**

**Citrus_medica** **tttaccataacatttctctagtttggaaccggtatgtaattgattccagtatggaatcat**

**Citrus_polytrifolia** **tttaccataacatttctctagtttggaaccggtatgtaattgattccagtatggaatcat**

**Citrus_reticulata** **tttaccataacatttctctagtttggaaccggtatgtaattgattccagtatggaatcat**

**Citrus_sinensis** **tttaccataacatttctctagtttggaaccggtatgtaattgattccagtatggaatcat**

**Fortunella_bawangica_1** **tttaccataacatttctctagtttggaaccggtatgtaattgattccagtatggaatcat**

**Fortunella_bawangica_2** **tttaccataacatttctctagtttggaaccggtatgtaattgattccagtatggaatcat**

**Fortunella_hindsii_1** **tttaccataacatttctctagtttggaaccggtatgtaattgattccagtatggaatcat**

**Fortunella_hindsii_2** **tttaccataacatttctctagtttggaaccggtatgtaattgattccagtatggaatcat**

**Fortunella_hindsii_3** **tttaccataacatttctctagtttggaaccggtatgtaattgattccagtatggaatcat**

**Fortunella_japonica_1** **tttaccataacatttctctagtttggaaccggtatgtaattgattccagtatggaatcat**

**Fortunella_japonica_2** **tttaccataacatttctctagtttggaaccggtatgtaattgattccagtatggaatcat**

**Fortunella_japonica_3** **tttaccataacatttctctagtttggaaccggtatgtaattgattccagtatggaatcat**

**Fortunella_japonica_4** **tttaccataacatttctctagtttggaaccggtatgtaattgattccagtatggaatcat**

**Fortunella_japonica_5** **tttaccataacatttctctagtttggaaccggtatgtaattgattccagtatggaatcat**

**Fortunella_japonica_6** **tttaccataacatttctctagtttggaaccggtatgtaattgattccagtatggaatcat**

**Fortunella_japonica_7** **tttaccataacatttctctagtttggaaccggtatgtaattgattccagtatggaatcat**

**Fortunella_japonica_8** **tttaccataacatttctctagtttggaaccggtatgtaattgattccagtatggaatcat**

**Fortunella_japonica_9** **tttaccataacatttctctagtttggaaccggtatgtaattgattccagtatggaatcat**

**Fortunella_japonica_10** **tttaccataacatttctctagtttggaaccggtatgtaattgattccagtatggaatcat**

**Fortunella_japonica_11** **tttaccataacatttctctagtttggaaccggtatgtaattgattccagtatggaatcat**

**Fortunella_japonica_12** **tttaccataacatttctctagtttggaaccggtatgtaattgattccagtatggaatcat**

**Fortunella_margarita_1** **tttaccataacatttctctagtttggaaccggtatgtaattgattccagtatggaatcat**

**Fortunella_margarita_2** **tttaccataacatttctctagtttggaaccggtatgtaattgattccagtatggaatcat**

**Fortunella_venosa_1** **tttaccataacatttctctagtttggaaccggtatgtaattgattccagtatggaatcat**

**Fortunella_venosa_2** **tttaccataacatttctctagtttggaaccggtatgtaattgattccagtatggaatcat**

**Fortunella_venosa_3** **tttaccataacatttctctagtttggaaccggtatgtaattgattccagtatggaatcat**

6430	6440	6450	6460	6470	6480

....|....|....|....|....|....|....|....|....|....|....|....|

**Atalantia_kwangtungens** **gaatagtcattgcttcggtcgatacacagccttttttccttgtatatgaagggaatattt**

**Citrus_reticulata_1** **gaatagtcattgcttcggtcgatacacagacttttttccttgtatatgaagggaatattt**

**Citrus_reticulata_2** **gaatagtcattgcttcggtcgatacacagacttttttccttgtatatgaagggaatattt**

**Citrus_trifoliata_3** **gaatagtcattgcttcggtcgatacacagacttttttccttgtatatgaagggaatattt**

**Citrus_aurantiifolia** **gaatagtcattgcttcggtcgatacacagacttttttccttgtatatgaagggaatattt**

**Citrus_aurantium** **gaatagtcattgcttcggtcgatacacagacttttttccttgtatatgaagggaatattt**

**Citrus_cavaleriei** **gaatagtcattgcttcggtcgatacacagacttttttccttgtatatgaagggaatattt**

**Citrus_hongheensis** **gaatagtcattgcttcggtcgatacacagacttttttccttgtatatgaagggaatattt**

**Citrus_limon** **gaatagtcattgcttcggtcgatacacagacttttttccttgtatatgaagggaatattt**

**Citrus_maxima** **gaatagtcattgcttcggtcgatacacagacttttttccttgtatatgaagggaatattt**

**Citrus_medica** **gaatagtcattgcttcggtcgatacacagacttttttccttgtatatgaagggaatattt**

**Citrus_polytrifolia** **gaatagtcattgcttcggtcgatacacagacttttttccttgtatatgaagggaatattt**

**Citrus_reticulata** **gaatagtcattgcttcggtcgatacacagacttttttccttgtatatgaagggaatattt**

**Citrus_sinensis** **gaatagtcattgcttcggtcgatacacagacttttttccttgtatatgaagggaatattt**

**Fortunella_bawangica_1** **gaatagtcattgcttcggtcgatacacagacttttttccttgtatatgaagggaatattt**

**Fortunella_bawangica_2** **gaatagtcattgcttcggtcgatacacagacttttttccttgtatatgaagggaatattt**

**Fortunella_hindsii_1** **gaatagtcattgcttcggtcgatacacagacttttttccttgtatatgaagggaatattt**

**Fortunella_hindsii_2** **gaatagtcattgcttcggtcgatacacagacttttttccttgtatatgaagggaatattt**

**Fortunella_hindsii_3** **gaatagtcattgcttcggtcgatacacagacttttttccttgtatatgaagggaatattt**

**Fortunella_japonica_1** **gaatagtcattgcttcggtcgatacacagacttttttccttgtatatgaagggaatattt**

**Fortunella_japonica_2** **gaatagtcattgcttcggtcgatacacagacttttttccttgtatatgaagggaatattt**

**Fortunella_japonica_3** **gaatagtcattgcttcggtcgatacacagacttttttccttgtatatgaagggaatattt**

**Fortunella_japonica_4** **gaatagtcattgcttcggtcgatacacagacttttttccttgtatatgaagggaatattt**

**Fortunella_japonica_5** **gaatagtcattgcttcggtcgatacacagacttttttccttgtatatgaagggaatattt**

**Fortunella_japonica_6** **gaatagtcattgcttcggtcgatacacagacttttttccttgtatatgaagggaatattt**

**Fortunella_japonica_7** **gaatagtcattgcttcggtcgatacacagacttttttccttgtatatgaagggaatattt**

**Fortunella_japonica_8** **gaatagtcattgcttcggtcgatacacagacttttttccttgtatatgaagggaatattt**

**Fortunella_japonica_9** **gaatagtcattgcttcggtcgatacacagacttttttccttgtatatgaagggaatattt**

**Fortunella_japonica_10** **gaatagtcattgcttcggtcgatacacagacttttttccttgtatatgaagggaatattt**

**Fortunella_japonica_11** **gaatagtcattgcttcggtcgatacacagacttttttccttgtatatgaagggaatattt**

**Fortunella_japonica_12** **gaatagtcattgcttcggtcgatacacagacttttttccttgtatatgaagggaatattt**

**Fortunella_margarita_1** **gaatagtcattgcttcggtcgatacacagacttttttccttgtatatgaagggaatattt**

**Fortunella_margarita_2** **gaatagtcattgcttcggtcgatacacagacttttttccttgtatatgaagggaatattt**

**Fortunella_venosa_1** **gaatagtcattgcttcggtcgatacacagacttttttccttgtatatgaagggaatattt**

**Fortunella_venosa_2** **gaatagtcattgcttcggtcgatacacagacttttttccttgtatatgaagggaatattt**

**Fortunella_venosa_3** **gaatagtcattgcttcggtcgatacacagacttttttccttgtatatgaagggaatattt**

6490	6500	6510	6520	6530	6540

....|....|....|....|....|....|....|....|....|....|....|....|

**Atalantia_kwangtungens** **aggttgttagtcgttcatccggaatcctgaaatgaaagagcaaatcagaattacaaaaat**

**Citrus_reticulata_1** **aggttgttagtcgttcatccggaatcctgaaatgaaagagaaaatcagaattacaaaaat**

**Citrus_reticulata_2** **aggttgttagtcgttcatccggaatcctgaaatgaaagagaaaatcagaattacaaaaat**

**Citrus_trifoliata_3** **aggttgttagtcgttcatccggaatcctgaaatgaaagagaaaatcagaattacaaaaat**

**Citrus_aurantiifolia** **aggttgttagtcgttcatccggaatcctgaaatgaaagagaaaatcagaattacaaaaat**

**Citrus_aurantium** **aggttgttagtcgttcatccggaatcctgaaatgaaagagaaaatcagaattacaaaaat**

**Citrus_cavaleriei** **aggttgttagtcgttcatccggaatcctgaaatgaaagagaaaatcagaattacaaaaat**

**Citrus_hongheensis** **aggttgttagtcgttcatccggaatcctgaaatgaaagagaaaatcagaattacaaaaat**

**Citrus_limon** **aggttgttagtcgttcatccggaatcctgaaatgaaagagaaaatcagaattacaaaaat**

**Citrus_maxima** **aggttgttagtcgttcatccggaatcctgaaatgaaaaagaaaatcagaattacaaaaat**

**Citrus_medica** **aggttgttagtcgttcatccggaatcctgaaatgaaagagcaaatcagaattacaaaaat**

**Citrus_polytrifolia** **aggttgttagtcgttcatccggaatcctgaaatgaaagagaaaatcagaattacaaaaat**

**Citrus_reticulata** **aggttgttagtcgttcatccggaatcctgaaatgaaagagaaaatcagaattacaaaaat**

**Citrus_sinensis** **aggttgttagtcgttcatccggaatcctgaaatgaaaaagaaaatcagaattacaaaaat**

**Fortunella_bawangica_1** **aggttgttagtcgttcatccggaatcctgaaatgaaagagaaaatcagaattacaaaaat**

**Fortunella_bawangica_2** **aggttgttagtcgttcatccggaatcctgaaatgaaagagaaaatcagaattacaaaaat**

**Fortunella_hindsii_1** **aggttgttagtcgttcatccggaatcctgaaatgaaagagaaaatcagaattacaaaaat**

**Fortunella_hindsii_2** **aggttgttagtcgttcatccggaatcctgaaatgaaagagaaaatcagaattacaaaaat**

**Fortunella_hindsii_3** **aggttgttagtcgttcatccggaatcctgaaatgaaagagaaaatcagaattacaaaaat**

**Fortunella_japonica_1** **aggttgttagtcgttcatccggaatcctgaaatgaaagagaaaatcagaattacaaaaat**

**Fortunella_japonica_2** **aggttgttagtcgttcatccggaatcctgaaatgaaagagaaaatcagaattacaaaaat**

**Fortunella_japonica_3** **aggttgttagtcgttcatccggaatcctgaaatgaaagagaaaatcagaattacaaaaat**

**Fortunella_japonica_4** **aggttgttagtcgttcatccggaatcctgaaatgaaagagaaaatcagaattacaaaaat**

**Fortunella_japonica_5** **aggttgttagtcgttcatccggaatcctgaaatgaaagagaaaatcagaattacaaaaat**

**Fortunella_japonica_6** **aggttgttagtcgttcatccggaatcctgaaatgaaagagaaaatcagaattacaaaaat**

**Fortunella_japonica_7** **aggttgttagtcgttcatccggaatcctgaaatgaaagagaaaatcagaattacaaaaat**

**Fortunella_japonica_8** **aggttgttagtcgttcatccggaatcctgaaatgaaagagaaaatcagaattacaaaaat**

**Fortunella_japonica_9** **aggttgttagtcgttcatccggaatcctgaaatgaaagagaaaatcagaattacaaaaat**

**Fortunella_japonica_10** **aggttgttagtcgttcatccggaatcctgaaatgaaagagaaaatcagaattacaaaaat**

**Fortunella_japonica_11** **aggttgttagtcgttcatccggaatcctgaaatgaaagagaaaatcagaattacaaaaat**

**Fortunella_japonica_12** **aggttgttagtcgttcatccggaatcctgaaatgaaagagaaaatcagaattacaaaaat**

**Fortunella_margarita_1** **aggttgttagtcgttcatccggaatcctgaaatgaaagagaaaatcagaattacaaaaat**

**Fortunella_margarita_2** **aggttgttagtcgttcatccggaatcctgaaatgaaagagaaaatcagaattacaaaaat**

**Fortunella_venosa_1** **aggttgttagtcgttcatccggaatcctgaaatgaaagagaaaatcagaattacaaaaat**

**Fortunella_venosa_2** **aggttgttagtcgttcatccggaatcctgaaatgaaagagaaaatcagaattacaaaaat**

**Fortunella_venosa_3** **aggttgttaggcgttcatccggaatcctgaaatgaaagagaaaatcagaattacaaaaat**

6550	6560	6570	6580	6590	6600

....|....|....|....|....|....|....|....|....|....|....|....|

**Atalantia_kwangtungens** **gggcgatttctgtagctatctgaacaattttcgatttctgtagctatctgaacaattttc**

**Citrus_reticulata_1** **gggcgatttctgtatctatcagatta-------------------gactgaacaattttc**

**Citrus_reticulata_2** **gggcgatttctgtatctatcagatta-------------------gactgaacaattttc**

**Citrus_trifoliata_3** **gggcgatttctgtatctatcagatta-------------------gactgaacaattttc**

**Citrus_aurantiifolia** **gggcgatttccgtatctatcagatta-------------------gactgaactattttc**

**Citrus_aurantium** **gggcgatttccgtatctatcagatta-------------------gactgaacaattttc**

**Citrus_cavaleriei** **gggcgatttctgtatctatcagatta-------------------gactgaacaattttc**

**Citrus_hongheensis** **gggcgatttccgtatctatcagatta-------------------gactgaacaattttc**

**Citrus_limon** **gggcgatttccgtatctatcagatta-------------------gactgaacaattttc**

**Citrus_maxima** **gggcgatttccgtatctatcagatta-------------------gactgaacaattttc**

**Citrus_medica** **gggcgatttccgtatctatcagatta-------------------gactgaacaattttc**

**Citrus_polytrifolia** **gggcgatttctgtatctatcagatta-------------------gactgaacaattttc**

**Citrus_reticulata** **gggcgatttctgtatctatcagatta-------------------gactgaacaattttc**

**Citrus_sinensis** **gggcgatttccgtatctatcagatta-------------------gactgaacaattttc**

**Fortunella_bawangica_1** **gggcgatttctgtatctatcagatta-------------------gactgaacaattttc**

**Fortunella_bawangica_2** **gggcgatttctgtatctatcagatta-------------------gactgaacaattttc**

**Fortunella_hindsii_1** **gggcgatttctgtatctatcagatta-------------------gactgaacaattttc**

**Fortunella_hindsii_2** **gggcgatttctgtatctatcagatta-------------------gactgaacaattttc**

**Fortunella_hindsii_3** **gggcgatttctgtatctatcagatta-------------------gactgaacaattttc**

**Fortunella_japonica_1** **gggcgatttctgtatctatcagatta-------------------gactgaacaattttc**

**Fortunella_japonica_2** **gggcgatttctgtatctatcagatta-------------------gactgaacaattttc**

**Fortunella_japonica_3** **gggcgatttctgtatctatcagatta-------------------gactgaacaattttc**

**Fortunella_japonica_4** **gggcgatttctgtatctatcagatta-------------------gactgaacaattttc**

**Fortunella_japonica_5** **gggcgatttctgtatctatcagatta-------------------gactgaacaattttc**

**Fortunella_japonica_6** **gggcgatttctgtatctatcagatta-------------------gactgaacaattttc**

**Fortunella_japonica_7** **gggcgatttctgtatctatcagatta-------------------gactgaacaattttc**

**Fortunella_japonica_8** **gggcgatttctgtatctatcagatta-------------------gactgaacaattttc**

**Fortunella_japonica_9** **gggcgatttctgtatctatcagatta-------------------gactgaacaattttc**

**Fortunella_japonica_10** **gggcgatttctgtatctatcagatta-------------------gactgaacaattttc**

**Fortunella_japonica_11** **gggcgatttctgtatctatcagatta-------------------gactgaacaattttc**

**Fortunella_japonica_12** **gggcgatttctgtatctatcagatta-------------------gactgaacaattttc**

**Fortunella_margarita_1** **gggcgatttctgtatctatcagatta-------------------gactgaacaattttc**

**Fortunella_margarita_2** **gggcgatttctgtatctatcagatta-------------------gactgaacaattttc**

**Fortunella_venosa_1** **gggcgatttctgtatctatcagatta-------------------gactgaacaattttc**

**Fortunella_venosa_2** **gggcgatttctgtatctatcagatta-------------------gactgaacaattttc**

**Fortunella_venosa_3** **gggcgatttctgtatctatcagatta-------------------gactgaacaattttc**

6610	6620	6630	6640	6650	6660

....|....|....|....|....|....|....|....|....|....|....|....|

**Atalantia_kwangtungens** **gttggaagtaattatgtatattgtatgttaaatatttaatttaacaggaagataagggct**

**Citrus_reticulata_1** **gttggaagtaattatgtatattgtatgttaaatatttaatttaacaggaagataagggct**

**Citrus_reticulata_2** **gttggaagtaattatgtatattgtatgttaaatatttaatttaacaggaagataagggct**

**Citrus_trifoliata_3** **gttggaagtaattatgtatattgtatgttaaatatttaatttaacaggaagataagggct**

**Citrus_aurantiifolia** **gttggaagtaattatgtatattgtatgttaaatatttaatttaacaggaagataagggct**

**Citrus_aurantium** **gttggaagtaattatgtatattgtatgttaaatatttaatttaacaggaagataagggct**

**Citrus_cavaleriei** **gttggaagtaattatgtatattgtatgttaaatatttaatttaacaggaagataagggct**

**Citrus_hongheensis** **gttggaagtaattatgtatattgtatgttaaatatttaatttaacaggaagataagggct**

**Citrus_limon** **gttggaagtaattatgtatattgtatgttaaatatttaatttaacaggaagataagggct**

**Citrus_maxima** **gttggaagtaattatgtatattgtatgttaaatatttaatttaacaggaagataagggct**

**Citrus_medica** **gttggaagtaattatgtatattgtatgttaaatatttaatttaccaggaagataagggct**

**Citrus_polytrifolia** **gttggaagtaattatgtatattgtatgttaaatatttaatttaacaggaagataagggct**

**Citrus_reticulata** **gttggaagtaattatgtatattgtatgttaaatatttaatttaacaggaagataagggct**

**Citrus_sinensis** **gttggaagtaattatgtatattgtatgttaaatatttaatttaacaggaagataagggct**

**Fortunella_bawangica_1** **gttggaagtaattatgtatattgtatgttaaatatttaatttaacaggaagataagggct**

**Fortunella_bawangica_2** **gttggaagtaattatgtatattgtatgttaaatatttaatttaacaggaagataagggct**

**Fortunella_hindsii_1** **gttggaagtaattatgtatattgtatgttaaatatttaatttaacaggaagataagggct**

**Fortunella_hindsii_2** **gttggaagtaattatgtatattgtatgttaaatatttaatttaacaggaagataagggct**

**Fortunella_hindsii_3** **gttggaagtaattatgtatattgtatgttaaatatttaatttaacaggaagataagggct**

**Fortunella_japonica_1** **gttggaagtaattatgtatattgtatgttaaatatttaatttaacaggaagataagggct**

**Fortunella_japonica_2** **gttggaagtaattatgtatattgtatgttaaatatttaatttaacaggaagataagggct**

**Fortunella_japonica_3** **gttggaagtaattatgtatattgtatgttaaatatttaatttaacaggaagataagggct**

**Fortunella_japonica_4** **gttggaagtaattatgtatattgtatgttaaatatttaatttaacaggaagataagggct**

**Fortunella_japonica_5** **gttggaagtaattatgtatattgtatgttaaatatttaatttaacaggaagataagggct**

**Fortunella_japonica_6** **gttggaagtaattatgtatattgtatgttaaatatttaatttaacaggaagataagggct**

**Fortunella_japonica_7** **gttggaagtaattatgtatattgtatgttaaatatttaatttaacaggaagataagggct**

**Fortunella_japonica_8** **gttggaagtaattatgtatattgtatgttaaatatttaatttaacaggaagataagggct**

**Fortunella_japonica_9** **gttggaagtaattatgtatattgtatgttaaatatttaatttaacaggaagataagggct**

**Fortunella_japonica_10** **gttggaagtaattatgtatattgtatgttaaatatttaatttaacaggaagataagggct**

**Fortunella_japonica_11** **gttggaagtaattatgtatattgtatgttaaatatttaatttaacaggaagataagggct**

**Fortunella_japonica_12** **gttggaagtaattatgtatattgtatgttaaatatttaatttaacaggaagataagggct**

**Fortunella_margarita_1** **gttggaagtaattatgtatattgtatgttaaatatttaatttaacaggaagataagggct**

**Fortunella_margarita_2** **gttggaagtaattatgtatattgtatgttaaatatttaatttaacaggaagataagggct**

**Fortunella_venosa_1** **gttggaagtaattatgtatattgtatgttaaatatttaatttaacaggaagataagggct**

**Fortunella_venosa_2** **gttggaagtaattatgtatattgtatgttaaatatttaatttaacaggaagataagggct**

**Fortunella_venosa_3** **gttggaagtaattatgtatattgtatgttaaatatttaatttaacaggaagataagggct**

6670	6680	6690	6700	6710	6720

....|....|....|....|....|....|....|....|....|....|....|....|

**Atalantia_kwangtungens** **cacaaatcttaaaaagcaaaaggacggtatctctgaaatagaaggatagcaatccatgcg**

**Citrus_reticulata_1** **cacaaatcttaaaaagcaaaaggacgttatctctgaaatagaaggatagcaatccatgca**

**Citrus_reticulata_2** **cacaaatcttaaaaagcaaaaggacgttatctctgaaatagaaggatagcaatccatgca**

**Citrus_trifoliata_3** **cacaaatcttaaaaagcaaaaggacgttatctctgaaatagaaggatagcaatccatgca**

**Citrus_aurantiifolia** **cacaaatcttaaaaagcaaaaggacgttatctctgaaatagaaggatagcaatccatgca**

**Citrus_aurantium** **cacaaatcttaaaaagcaaaaggacgttatctctgaaatagaaggatagcaatccatgca**

**Citrus_cavaleriei** **cacaaatcttaaaaagcaaaaggacgttatctctgaaatagaaggatagcaatccatgca**

**Citrus_hongheensis** **cacaaatcttaaaaagcaaaaggacgttatctctgaaatagaaggatagcaatccatgca**

**Citrus_limon** **cacaaatcttaaaaagcaaaaggacgttatctctgaaatagaaggatagcaatccatgca**

**Citrus_maxima** **cacaaatcttaaaaagcaaaaggacgttatctctgaaatagaaggatagcaatccatgca**

**Citrus_medica** **cacaaatcttaaaaagcaaaaggacgttatctctgaaatagaaggatagcaatccatgca**

**Citrus_polytrifolia** **cacaaatcttaaaaagcaaaaggacattatctctgaaatagaaggatagcaatccatgca**

**Citrus_reticulata** **cacaaatcttaaaaagcaaaaggacgttatctctgaaatagaaggatagcaatccatgca**

**Citrus_sinensis** **cacaaatcttaaaaagcaaaaggacgttatctctgaaatagaaggatagcaatccatgca**

**Fortunella_bawangica_1** **cacaaatcttaaaaagcaaaaggacgttatctctgaaatagaaggatagcaatccatgca**

**Fortunella_bawangica_2** **cacaaatcttaaaaagcaaaaggacgttatctctgaaatagaaggatagcaatccatgca**

**Fortunella_hindsii_1** **cacaaatcttaaaaagcaaaaggacgttatctctgaaatagaaggatagcaatccatgca**

**Fortunella_hindsii_2** **cacaaatcttaaaaagcaaaaggacgttatctctgaaatagaaggatagcaatccatgca**

**Fortunella_hindsii_3** **cacaaatcttaaaaagcaaaaggacgttatctctgaaatagaaggatagcaatccatgca**

**Fortunella_japonica_1** **cacaaatcttaaaaagcaaaaggacgttatctctgaaatagaaggatagcaatccatgca**

**Fortunella_japonica_2** **cacaaatcttaaaaagcaaaaggacgttatctctgaaatagaaggatagcaatccatgca**

**Fortunella_japonica_3** **cacaaatcttaaaaagcaaaaggacgttatctctgaaatagaaggatagcaatccatgca**

**Fortunella_japonica_4** **cacaaatcttaaaaagcaaaaggacgttatctctgaaatagaaggatagcaatccatgca**

**Fortunella_japonica_5** **cacaaatcttaaaaagcaaaaggacgttatctctgaaatagaaggatagcaatccatgca**

**Fortunella_japonica_6** **cacaaatcttaaaaagcaaaaggacgttatctctgaaatagaaggatagcaatccatgca**

**Fortunella_japonica_7** **cacaaatcttaaaaagcaaaaggacgttatctctgaaatagaaggatagcaatccatgca**

**Fortunella_japonica_8** **cacaaatcttaaaaagcaaaaggacgttatctctgaaatagaaggatagcaatccatgca**

**Fortunella_japonica_9** **cacaaatcttaaaaagcaaaaggacgttatctctgaaatagaaggatagcaatccatgca**

**Fortunella_japonica_10** **cacaaatcttaaaaagcaaaaggacgttatctctgaaatagaaggatagcaatccatgca**

**Fortunella_japonica_11** **cacaaatcttaaaaagcaaaaggacgttatctctgaaatagaaggatagcaatccatgca**

**Fortunella_japonica_12** **cacaaatcttaaaaagcaaaaggacgttatctctgaaatagaaggatagcaatccatgca**

**Fortunella_margarita_1** **cacaaatcttaaaaagcaaaaggacgttatctctgaaatagaaggatagcaatccatgca**

**Fortunella_margarita_2** **cacaaatcttaaaaagcaaaaggacgttatctctgaaatagaaggatagcaatccatgca**

**Fortunella_venosa_1** **cacaaatcttaaaaagcaaaaggacgttatctctgaaatagaaggatagcaatccatgca**

**Fortunella_venosa_2** **cacaaatcttaaaaagcaaaaggacgttatctctgaaatagaaggatagcaatccatgca**

**Fortunella_venosa_3** **cacaaatcttaaaaagcaaaaggacgttatctctgaaatagaaggatagcaatccatgca**

6730	6740	6750	6760	6770	6780

....|....|....|....|....|....|....|....|....|....|....|....|

**Atalantia_kwangtungens** **gataagcctttcctcaaattatgtgttgtttctttggcttgggcttcagattcaataatc**

**Citrus_reticulata_1** **tataagcctttcctcaaattatgtgtcgtttctttggcttgggcttcagattcaataatc**

**Citrus_reticulata_2** **tataagcctttcctcaaattatgtgtcgtttctttggcttgggcttcagattcaataatc**

**Citrus_trifoliata_3** **tataagcctttcctcaaattatgtgtcgtttctttggcttgggcttccgattcaataatc**

**Citrus_aurantiifolia** **tataagcctttcctcaaattatgtgtcgtttctttggcttgggcttcagattcaataatc**

**Citrus_aurantium** **tataagcctttcctcaaattatgtgtcgtttctttggcttgggcttcagattcaataatc**

**Citrus_cavaleriei** **tataagcctttcctcaaattatgtgtcgtttctttggcttgggcttcagattcaataatc**

**Citrus_hongheensis** **tataagcctttcctcaaattatgggtcgtttctttggcttgggcttcagattcaataatc**

**Citrus_limon** **tataagcctttcctcaaattatgtgtcgtttctttggcttgggcttcagattcaataatc**

**Citrus_maxima** **tataagcctttcctcaaattatgtgtcgtttctttggcttgggcttcagattcaataatc**

**Citrus_medica** **tataagcctttcctcaaattatgtgtcgtttctttggcttgggcttcagattcaataatc**

**Citrus_polytrifolia** **tataagcctttcctcaaattatgtgtcgtttctttggcttgggcttccgattcaataatc**

**Citrus_reticulata** **tataagcctttcctcaaattatgtgtcgtttctttggcttgggcttcagattcaataatc**

**Citrus_sinensis** **tataagcctttcctcaaattatgtgtcgtttctttggcttgggcttcagattcaataatc**

**Fortunella_bawangica_1** **tataagcctttcctcaaattatgtgtcgtttctttggcttgggcttcagattcaataatc**

**Fortunella_bawangica_2** **tataagcctttcctcaaattatgtgtcgtttctttggcttgggcttcagattcaataatc**

**Fortunella_hindsii_1** **tataagcctttcctcaaattatgtgtcgtttctttggcttgggcttcagattcaataatc**

**Fortunella_hindsii_2** **tataagcctttcctcaaattatgtgtcgtttctttggcttgggcttcagattcaataatc**

**Fortunella_hindsii_3** **tataagcctttcctcaaattatgtgtcgtttctttggcttgggcttcagattcaataatc**

**Fortunella_japonica_1** **tataagcctttcctcaaattatgtgtcgtttctttggcttgggcttcagattcaataatc**

**Fortunella_japonica_2** **tataagcctttcctcaaattatgtgtcgtttctttggcttgggcttcagattcaataatc**

**Fortunella_japonica_3** **tataagcctttcctcaaattatgtgtcgtttctttggcttgggcttcagattcaataatc**

**Fortunella_japonica_4** **tataagcctttcctcaaattatgtgtcgtttctttggcttgggcttcagattcaataatc**

**Fortunella_japonica_5** **tataagcctttcctcaaattatgtgtcgtttctttggcttgggcttcagattcaataatc**

**Fortunella_japonica_6** **tataagcctttcctcaaattatgtgtcgtttctttggcttgggcttcagattcaataatc**

**Fortunella_japonica_7** **tataagcctttcctcaaattatgtgtcgtttctttggcttgggcttcagattcaataatc**

**Fortunella_japonica_8** **tataagcctttcctcaaattatgtgtcgtttctttggcttgggcttcagattcaataatc**

**Fortunella_japonica_9** **tataagcctttcctcaaattatgtgtcgtttctttggcttgggcttcagattcaataatc**

**Fortunella_japonica_10** **tataagcctttcctcaaattatgtgtcgtttctttggcttgggcttcagattcaataatc**

**Fortunella_japonica_11** **tataagcctttcctcaaattatgtgtcgtttctttggcttgggcttcagattcaataatc**

**Fortunella_japonica_12** **tataagcctttcctcaaattatgtgtcgtttctttggcttgggcttcagattcaataatc**

**Fortunella_margarita_1** **tataagcctttcctcaaattatgtgtcgtttctttggcttgggcttcagattcaataatc**

**Fortunella_margarita_2** **tataagcctttcctcaaattatgtgtcgtttctttggcttgggcttcagattcaataatc**

**Fortunella_venosa_1** **tataagcctttcctcaaattatgtgtcgtttctttggcttgggcttcagattcaataatc**

**Fortunella_venosa_2** **tataagcctttcctcaaattatgtgtcgtttctttggcttgggcttcagattcaataatc**

**Fortunella_venosa_3** **tataagcctttcctcaaattatgtgtcgtttctttggcttgggcttcagattcaataatc**

6790	6800	6810	6820	6830	6840

....|....|....|....|....|....|....|....|....|....|....|....|

**Atalantia_kwangtungens** **tttccccaaattcaaaataaaa------------------------------------ta**

**Citrus_reticulata_1** **tttccccaaattcaaaataaaataaataaaatagaaaataaagtaaatagtagaaaattc**

**Citrus_reticulata_2** **tttccccaaattcaaaataaaataaataaaatagaaaataaagtaaatagtagaaaattc**

**Citrus_trifoliata_3** **tttccccaaattaaaaataaaataaatcaaatagaaaataaagtaaatagtataaagtta**

**Citrus_aurantiifolia** **tttccccaaattcaaaataaaataaataaaatagaaaataaagtaaatagtataaaattc**

**Citrus_aurantium** **tttccccaaattcaaaataaaataaataaaatagaaaataaagtaaatagtataaaattc**

**Citrus_cavaleriei** **tttccccaaattcaaaataaaataaataaaatagaaaataaagtaaatagtataaaattc**

**Citrus_hongheensis** **tttccccaaattcaaaataaaataaataaaatagaaaataaagtaaatagtataaaattc**

**Citrus_limon** **tttccccaaattcaaaataaaataaataaaatagaaaataaagtaaatagtataaaattc**

**Citrus_maxima** **tttccccaaattcaaaataaaataaataaaatagaaaataaagtaaatagtataaaattc**

**Citrus_medica** **tttccccaaattcaaaataaaataaataaaatataaaataaagtaaatagtataaaa-ta**

**Citrus_polytrifolia** **tttccccaaattaaaaataaaataaatcaaatagaaaataaagtaaatagtataaagtta**

**Citrus_reticulata** **tttccccaaattcaaaataaaataaataaaatagaaaataaagtaaatagtagaaaattc**

**Citrus_sinensis** **tttccccaaattcaaaataaaataaataaaatagaaaataaagtaaatagtataaaattc**

**Fortunella_bawangica_1** **tttccccaaattcaaaataaaataaataaaatagaaaataaagtaaatagtataaaatta**

**Fortunella_bawangica_2** **tttccccaaattcaaaataaaataaataaaatagaaaataaagtaaatagtataaaatta**

**Fortunella_hindsii_1** **tttccccaaattcaaaataaaataaataaaatagaaaataaagtaaatagtataaaatta**

**Fortunella_hindsii_2** **tttccccaaattcaaaataaaataaataaaatagaaaataaagtaaatagtataaaatta**

**Fortunella_hindsii_3** **tttccccaaattcaaaataaaataaataaaatagaaaataaagtaaatagtataaaatta**

**Fortunella_japonica_1** **tttccccaaattcaaaataaaataaataaaatagaaaataaagtaaatagtataaaatta**

**Fortunella_japonica_2** **tttccccaaattcaaaataaaataaataaaatagaaaataaagtaaatagtataaaatta**

**Fortunella_japonica_3** **tttccccaaattcaaaataaaataaataaaatagaaaataaagtaaatagtataaaatta**

**Fortunella_japonica_4** **tttccccaaattcaaaataaaataaataaaatagaaaataaagtaaatagtataaaatta**

**Fortunella_japonica_5** **tttccccaaattcaaaataaaataaataaaatagaaaataaagtaaatagtataaaatta**

**Fortunella_japonica_6** **tttccccaaattcaaaataaaataaataaaatagaaaataaagtaaatagtataaaatta**

**Fortunella_japonica_7** **tttccccaaattcaaaataaaataaataaaatagaaaataaagtaaatagtataaaatta**

**Fortunella_japonica_8** **tttccccaaattcaaaataaaataaataaaatagaaaataaagtaaatagtataaaatta**

**Fortunella_japonica_9** **tttccccaaattcaaaataaaataaataaaatagaaaataaagtaaatagtataaaatta**

**Fortunella_japonica_10** **tttccccaaattcaaaataaaataaataaaatagaaaataaagtaaatagtataaaatta**

**Fortunella_japonica_11** **tttccccaaattcaaaataaaataaataaaatagaaaataaagtaaatagtataaaatta**

**Fortunella_japonica_12** **tttccccaaattcaaaataaaataaataaaatagaaaataaagtaaatagtataaaatta**

**Fortunella_margarita_1** **tttccccaaattcaaaataaaataaataaaatagaaaataaagtaaatagtataaaatta**

**Fortunella_margarita_2** **tttccccaaattcaaaataaaataaataaaatagaaaataaagtaaatagtataaaatta**

**Fortunella_venosa_1** **tttccccaaattcaaaataaaataaataaaatagaaaataaagtaaatagtataaaatta**

**Fortunella_venosa_2** **tttccccaaattcaaaataaaataaatcaaatagaaaataaagtaaatagtataaaatta**

**Fortunella_venosa_3** **tttccccaaattcaaaataaaataaataaaatagaaaataaagtaaatagtataaaatta**

6850	6860	6870	6880	6890	6900

....|....|....|....|....|....|....|....|....|....|....|....|

**Atalantia_kwangtungens** **aagtaaatagactatatgaataaccc-cgtctcaattgttattccagagatttacaatta**

**Citrus_reticulata_1** **aagtaaatagactatatgaataacccccgtctcaattgttattccagagatttacaatta**

**Citrus_reticulata_2** **aagtaaatagactatatgaataacccccgtctcaattgttattccagagatttacaatta**

**Citrus_trifoliata_3** **aagtaaatagactatctgaataacccccgtctcaattgttattccagagatttacaatta**

**Citrus_aurantiifolia** **aagtaaatagactatatgaataacccccgtctcaattgttattccagagatttacaatta**

**Citrus_aurantium** **aagtaaatagactatatgaataacccccgtctcaattgttattccagagatttacaatta**

**Citrus_cavaleriei** **aagtaaatagactatatgaataacccccgtctcaattgttattccagagatttacaatta**

**Citrus_hongheensis** **aagtaaatagactatatgaataacccccgtctcaattgttattccagagatttacaatta**

**Citrus_limon** **aagtaaatagactatatgaataacccccgtctcaattgttattccagagatttacaatta**

**Citrus_maxima** **aagtaaatagactatatgaataacccccgtctcaattgttattccagagatttacaatta**

**Citrus_medica** **aagtaaatagactatatgaataacccccgtctcaattgttattccagagatttacaatta**

**Citrus_polytrifolia** **aagtaaatagactatctgaataacccccgtctcaattgttattccagagatttacaatta**

**Citrus_reticulata** **aagtaaatagactatatgaataacccccgtctcaattgttattccagagatttacaatta**

**Citrus_sinensis** **aagtaaatagactatatgaataacccccgtctcaattgttattccagagatttacaatta**

**Fortunella_bawangica_1** **aagtaaatagactatatgaataacccccgtctcaattgttattccagagatttacaatta**

**Fortunella_bawangica_2** **aagtaaatagactatatgaataacccccgtctcaattgttattccagagatttacaatta**

**Fortunella_hindsii_1** **aagtaaatagactatatgaataacccccgtctcaattgttattccagagatttacaatta**

**Fortunella_hindsii_2** **aagtaaatagactatatgaataacccccgtctcaattgttattccagagatttacaatta**

**Fortunella_hindsii_3** **aagtaaatagactatatgaataacccccgtctcaattgttattccagagatttacaatta**

**Fortunella_japonica_1** **aagtaaatagactatatgaataacccccgtctcaattgttattccagagatttacaatta**

**Fortunella_japonica_2** **aagtaaatagactatatgaataacccccgtctcaattgttattccagagatttacaatta**

**Fortunella_japonica_3** **aagtaaatagactatatgaataacccccgtctcaattgttattccagagatttacaatta**

**Fortunella_japonica_4** **aagtaaatagactatatgaataacccccgtctcaattgttattccagagatttacaatta**

**Fortunella_japonica_5** **aagtaaatagactatatgaataacccccgtctcaattgttattccagagatttacaatta**

**Fortunella_japonica_6** **aagtaaatagactatatgaataacccccgtctcaattgttattccagagatttacaatta**

**Fortunella_japonica_7** **aagtaaatagactatatgaataacccccgtctcaattgttattccagagatttacaatta**

**Fortunella_japonica_8** **aagtaaatagactatatgaataacccccgtctcaattgttattccagagatttacaatta**

**Fortunella_japonica_9** **aagtaaatagactatatgaataacccccgtctcaattgttattccagagatttacaatta**

**Fortunella_japonica_10** **aagtaaatagactatatgaataacccccgtctcaattgttattccagagatttacaatta**

**Fortunella_japonica_11** **aagtaaatagactatatgaataacccccgtctcaattgttattccagagatttacaatta**

**Fortunella_japonica_12** **aagtaaatagactatatgaataacccccgtctcaattgttattccagagatttacaatta**

**Fortunella_margarita_1** **aagtaaatagactatatgaataacccccgtctcaattgttattccagagatttacaatta**

**Fortunella_margarita_2** **aagtaaatagactatatgaataacccccgtctcaattgttattccagagatttacaatta**

**Fortunella_venosa_1** **aagtaaatagactatatgaataacccccgtctcaattgttattccagagatttacaatta**

**Fortunella_venosa_2** **aagtaaatagactatatgaataacccccgtctcaattgttattccagagatttacaatta**

**Fortunella_venosa_3** **aagtaaatagactatatgaataacccccgtctcaattgttattccagagatttacaatta**

6910	6920	6930	6940	6950	6960

....|....|....|....|....|....|....|....|....|....|....|....|

**Atalantia_kwangtungens** **ttcattaaaaattaaaaaaagcccaagaaaaaataccgaaaaatttgggttagaatcaaa**

**Citrus_reticulata_1** **ttcatt-------aaaaaaagaccaag-------accgcaaaatttgggttagaatcaaa**

**Citrus_reticulata_2** **ttcatt-------aaaaaaagaccaag-------accgcaaaatttgggttagaatcaaa**

**Citrus_trifoliata_3** **ttcatt--------aaaaaagaccaag-------accgcaaaatttgggttagaatcaaa**

**Citrus_aurantiifolia** **ttcatt--------aaaaaagaccaag-------accgcaaaatttgggttagaatcaaa**

**Citrus_aurantium** **ttcatt-------aaaaaaagaccaag-------accgcaaaatttgggttagaatcaaa**

**Citrus_cavaleriei** **ttcatt-------aaaaaaagaccaag-------accgcaaaatttgggttagaatcaaa**

**Citrus_hongheensis** **ttcatt-------aaaaaaagaccaag-------accgcaaaatttgggttagaatcaaa**

**Citrus_limon** **ttcatt-------aaaaaaagaccaag-------accgcaaaatttgggttagaatcaaa**

**Citrus_maxima** **ttcatt-------aaaaaaagaccaag-------accgcaaaatttgggttagaatcaaa**

**Citrus_medica** **ttcatt-------aaaaaaagaccaag-------accgcaaaatttgggttagaatcaaa**

**Citrus_polytrifolia** **ttcatt--------aaaaaagaccaag-------accgcaaaatttgggttagaatcaaa**

**Citrus_reticulata** **ttcatt-------aaaaaaagaccaag-------accgcaaaatttgggttagaatcaaa**

**Citrus_sinensis** **ttcatt-------aaaaaaagaccaag-------accgcaaaatttgggttagaatcaaa**

**Fortunella_bawangica_1** **ttcatt-------aaaaaaagaccaag-------accgcaaaatttgggttagaatcaaa**

**Fortunella_bawangica_2** **ttcatt-------aaaaaaagaccaag-------accgcaaaatttgggttagaatcaaa**

**Fortunella_hindsii_1** **ttcatt-------aaaaaaagaccaag-------accgcaaaatttgggttagaatcaaa**

**Fortunella_hindsii_2** **ttcatt-------aaaaaaagaccaag-------accgcaaaatttgggttagaatcaaa**

**Fortunella_hindsii_3** **ttcatt-------aaaaaaagaccaag-------accgcaaaatttgggttagaatcaaa**

**Fortunella_japonica_1** **ttcatt-------aaaaaaagaccaag-------accgcaaaatttgggttagaatcaaa**

**Fortunella_japonica_2** **ttcatt-------aaaaaaagaccaag-------accgcaaaatttgggttagaatcaaa**

**Fortunella_japonica_3** **ttcatt-------aaaaaaagaccaag-------accgcaaaatttgggttagaatcaaa**

**Fortunella_japonica_4** **ttcatt-------aaaaaaagaccaag-------accgcaaaatttgggttagaatcaaa**

**Fortunella_japonica_5** **ttcatt-------aaaaaaagaccaag-------accgcaaaatttgggttagaatcaaa**

**Fortunella_japonica_6** **ttcatt-------aaaaaaagaccaag-------accgcaaaatttgggttagaatcaaa**

**Fortunella_japonica_7** **ttcatt-------aaaaaaagaccaag-------accgcaaaatttgggttagaatcaaa**

**Fortunella_japonica_8** **ttcatt-------aaaaaaagaccaag-------accgcaaaatttgggttagaatcaaa**

**Fortunella_japonica_9** **ttcatt-------aaaaaaagaccaag-------accgcaaaatttgggttagaatcaaa**

**Fortunella_japonica_10** **ttcatt-------aaaaaaagaccaag-------accgcaaaatttgggttagaatcaaa**

**Fortunella_japonica_11** **ttcatt-------aaaaaaagaccaag-------accgcaaaatttgggttagaatcaaa**

**Fortunella_japonica_12** **ttcatt-------aaaaaaagaccaag-------accgcaaaatttgggttagaatcaaa**

**Fortunella_margarita_1** **ttcatt-------aaaaaaagaccaag-------accgcaaaatttgggttagaatcaaa**

**Fortunella_margarita_2** **ttcatt-------aaaaaaagaccaag-------accgcaaaatttgggttagaatcaaa**

**Fortunella_venosa_1** **ttcatt-------aaaaaaagaccaag-------accgcaaaatttgggttagaatcaaa**

**Fortunella_venosa_2** **ttcatt-------aaaaaaagaccaag-------accgcaaaatttgggttagaatcaaa**

**Fortunella_venosa_3** **ttcatt-------aaaaaaagaccaag-------accgcaaaatttgggttagaatcaaa**

6970	6980	6990	7000	7010	7020

....|....|....|....|....|....|....|....|....|....|....|....|

**Atalantia_kwangtungens** **gagcctccattccatatagagcgggattattggttaatgagatttggaccgacatcgata**

**Citrus_reticulata_1** **gagcctccattccatatagagcgggattattggttaatgaaatttggaccgacaccgata**

**Citrus_reticulata_2** **gagcctccattccatatagagcgggattattggttaatgaaatttggaccgacaccgata**

**Citrus_trifoliata_3** **gagcctccattccatatagagcgggattattggttaatgaaatttggaccgacaccgata**

**Citrus_aurantiifolia** **gagcctccattccatatagagcgggattattggttaatgaaatttggaccgacaccgata**

**Citrus_aurantium** **gagcctccattccatatagagcgggattattggttaatgaaatttggaccgacaccgata**

**Citrus_cavaleriei** **gagcctccattccatatagagcgggattattggttaatgaaatttggaccgacaccgata**

**Citrus_hongheensis** **gagcctccattccatatagagcggaattattggttaatgaaatttggaccgacaccgata**

**Citrus_limon** **gagcctccattccatatagagcgggattattggttaatgaaatttggaccgacaccgata**

**Citrus_maxima** **gagcctccattccatatagagcgggattattggttaatgaaatttggaccgacaccgata**

**Citrus_medica** **gagcctccattccatatagagcgggattattggttaatgagatttggaccgacaccgata**

**Citrus_polytrifolia** **gagcctccattccatatagagcgggattattggttaatgaaatttggaccgacacccata**

**Citrus_reticulata** **gagcctccattccatatagagcgggattattggttaatgaaatttggaccgacaccgata**

**Citrus_sinensis** **gagcctccattccatatagagcgggattattggttaatgaaatttggaccgacaccgata**

**Fortunella_bawangica_1** **gagcctccattccatatagagcgggattattggttaatgaaatttggaccgacaccgata**

**Fortunella_bawangica_2** **gagcctccattccatatagagcgggattattggttaatgaaatttggaccgacaccgata**

**Fortunella_hindsii_1** **gagcctccattccatatagagcgggattattggttaatgaaatttggaccgacaccgata**

**Fortunella_hindsii_2** **gagcctccattccatatagagcgggattattggttaatgaaatttggaccgacaccgata**

**Fortunella_hindsii_3** **gagcctccattccatatagagcgggattattggttaatgaaatttggaccgacaccgata**

**Fortunella_japonica_1** **gagcctccattccatatagagcgggattattggttaatgaaatttggaccgacaccgata**

**Fortunella_japonica_2** **gagcctccattccatatagagcgggattattggttaatgaaatttggaccgacaccgata**

**Fortunella_japonica_3** **gagcctccattccatatagagcgggattattggttaatgaaatttggaccgacaccgata**

**Fortunella_japonica_4** **gagcctccattccatatagagcgggattattggttaatgaaatttggaccgacaccgata**

**Fortunella_japonica_5** **gagcctccattccatatagagcgggattattggttaatgaaatttggaccgacaccgata**

**Fortunella_japonica_6** **gagcctccattccatatagagcgggattattggttaatgaaatttggaccgacaccgata**

**Fortunella_japonica_7** **gagcctccattccatatagagcgggattattggttaatgaaatttggaccgacaccgata**

**Fortunella_japonica_8** **gagcctccattccatatagagcgggattattggttaatgaaatttggaccgacaccgata**

**Fortunella_japonica_9** **gagcctccattccatatagagcgggattattggttaatgaaatttggaccgacaccgata**

**Fortunella_japonica_10** **gagcctccattccatatagagcgggattattggttaatgaaatttggaccgacaccgata**

**Fortunella_japonica_11** **gagcctccattccatatagagcgggattattggttaatgaaatttggaccgacaccgata**

**Fortunella_japonica_12** **gagcctccattccatatagagcgggattattggttaatgaaatttggaccgacaccgata**

**Fortunella_margarita_1** **gagcctccattccatatagagcgggattattggttaatgaaatttggaccgacaccgata**

**Fortunella_margarita_2** **gagcctccattccatatagagcgggattattggttaatgaaatttggaccgacaccgata**

**Fortunella_venosa_1** **gagcctccattccatatagagcgggattattggttaatgaaatttggaccgacaccgata**

**Fortunella_venosa_2** **gagcctccattccatatagagcgggattattggttaatgaaatttggaccgacaccgata**

**Fortunella_venosa_3** **gagcctccattccatatagagcgggattattggttaatgaaatttggaccgacaccgata**

7030	7040	7050	7060	7070	7080

....|....|....|....|....|....|....|....|....|....|....|....|

**Atalantia_kwangtungens** **ttcaaatcggtatctttcattgctcactaactcttatctactcccaccccgaattctttc**

**Citrus_reticulata_1** **ttcaaatcggtatctttcattgctcactaactcttacctactcccaccccgaattctttc**

**Citrus_reticulata_2** **ttcaaatcggtatctttcattgctcactaactcttacctactcccaccccgaattctttc**

**Citrus_trifoliata_3** **ttcaaatcggtatctttcattgctcactaactcttacctactcccaccccgaattctttc**

**Citrus_aurantiifolia** **ttcaaatcggtatctttcattgctcactaactcttacctactcccaccccgaattctttc**

**Citrus_aurantium** **ttcaaatcggtatctttcattgctcactaactcttacctactcccaccccgaattctttc**

**Citrus_cavaleriei** **ttcaaatcggtatctttcattgctcactaactcttacctactcccaccccgaattctttc**

**Citrus_hongheensis** **ttcaaatcggtatctttcattgctcactaactcttacctactcccaccccgaattctttc**

**Citrus_limon** **ttcaaatcggtatctttcattgctcactaactcttacctactcccaccccgaattctttc**

**Citrus_maxima** **ttcaaatcggtatctttcattgctcactaactcttacctactcccaccccgaattctttc**

**Citrus_medica** **ttcaaatcggtatctttcattgctcactaactcttacctactcccaccccgaattctttc**

**Citrus_polytrifolia** **ttcaaatcggtatctttcattgctcactaactcttacctactcccaccccgaattctttc**

**Citrus_reticulata** **ttcaaatcggtatctttcattgctcactaactcttacctactcccaccccgaattctttc**

**Citrus_sinensis** **ttcaaatcggtatctttcattgctcactaactcttacctactcccaccccgaattctttc**

**Fortunella_bawangica_1** **ttcaaatcggtatctttcattgctcactaactcttacttactcccaccccgaattctttc**

**Fortunella_bawangica_2** **ttcaaatcggtatctttcattgctcactaactcttacttactcccaccccgaattctttc**

**Fortunella_hindsii_1** **ttcaaatcggtatctttcattgctcactaactcttacttactcccaccccgaattctttc**

**Fortunella_hindsii_2** **ttcaaatcggtatctttcattgctcactaactcttacttactcccaccccgaattctttc**

**Fortunella_hindsii_3** **ttcaaatcggtatctttcattgctcactaactcttacttactcccaccccgaattctttc**

**Fortunella_japonica_1** **ttcaaatcggtatctttcattgctcactaactcttacttactcccaccccgaattctttc**

**Fortunella_japonica_2** **ttcaaatcggtatctttcattgctcactaactcttacttactcccaccccgaattctttc**

**Fortunella_japonica_3** **ttcaaatcggtatctttcattgctcactaactcttacttactcccaccccgaattctttc**

**Fortunella_japonica_4** **ttcaaatcggtatctttcattgctcactaactcttacttactcccaccccgaattctttc**

**Fortunella_japonica_5** **ttcaaatcggtatctttcattgctcactaactcttacttactcccaccccgaattctttc**

**Fortunella_japonica_6** **ttcaaatcggtatctttcattgctcactaactcttacttactcccaccccgaattctttc**

**Fortunella_japonica_7** **ttcaaatcggtatctttcattgctcactaactcttacttactcccaccccgaattctttc**

**Fortunella_japonica_8** **ttcaaatcggtatctttcattgctcactaactcttacttactcccaccccgaattctttc**

**Fortunella_japonica_9** **ttcaaatcggtatctttcattgctcactaactcttacttactcccaccccgaattctttc**

**Fortunella_japonica_10** **ttcaaatcggtatctttcattgctcactaactcttacttactcccaccccgaattctttc**

**Fortunella_japonica_11** **ttcaaatcggtatctttcattgctcactaactcttacttactcccaccccgaattctttc**

**Fortunella_japonica_12** **ttcaaatcggtatctttcattgctcactaactcttacttactcccaccccgaattctttc**

**Fortunella_margarita_1** **ttcaaatcggtatctttcattgctcactaactcttacttactcccaccccgaattctttc**

**Fortunella_margarita_2** **ttcaaatcggtatctttcattgctcactaactcttacttactcccaccccgaattctttc**

**Fortunella_venosa_1** **ttcaaatcggtatctttcattgctcactaactcttacttactcccaccccgaattctttc**

**Fortunella_venosa_2** **ttcaaatcggtatctttcattgctcactaactcttacttactcccaccccgaattctttc**

**Fortunella_venosa_3** **ttcaaatcggtatctttcattgctcactaactcttacttactcccaccccgaattctttc**

7090	7100	7110	7120	7130	7140

....|....|....|....|....|....|....|....|....|....|....|....|

**Atalantia_kwangtungens** **tgtgggaatcctaaataaaaaataaaaaaaaaaagatcccattttacggaatgctagact**

**Citrus_reticulata_1** **tgtgggaatcctaaataaataaaaaaaaaaaaaagatcccattttacggaatgctagact**

**Citrus_reticulata_2** **tgtgggaatcctaaataaataaaaaaaaaaaaaagatcccattttacggaatgctagact**

**Citrus_trifoliata_3** **tgtgggaatcc----taaat----------------------------------------**

**Citrus_aurantiifolia** **tgtgggaatcctaaataaat----------------------------------------**

**Citrus_aurantium** **tgtgggaatcctaaataaat-aaaaaaaaaaaaagatcccattttacggaatgctagact**

**Citrus_cavaleriei** **tgtgggaatcctaaataaat--caaaaaaaaaaagatcccattttacggaatgctagact**

**Citrus_hongheensis** **tgtgggaatcctaaataaat---aaaaaaaaaaagatcccattttacggaatgctagact**

**Citrus_limon** **tgtgggaatcctaaataaat-aaaaaaaaaaaaagatcccattttacggaatgctagact**

**Citrus_maxima** **tgtgggaatcctaaataaat--aaaaaaaaaaaagatcccattttacggaatgctagact**

**Citrus_medica** **tgtgggaatcctaaataaat--aaaaaaaaaaaagatcccattttacggaatgctagact**

**Citrus_polytrifolia** **tgtgggaatcctaaataaat----------------------------------------**

**Citrus_reticulata** **tgtgggaatcctaaataaataaaaaaaaaaaaaagatcccattttacggaatgctagact**

**Citrus_sinensis** **tgtgggaatcctaaataaat-aaaaaaaaaaaaagatcccattttacggaatgctagact**

**Fortunella_bawangica_1** **tgtgggaatcctaaataaat----aaaaaaaaaagatcccattttacggaatgctagact**

**Fortunella_bawangica_2** **tgtgggaatcctaaataaat----aaaaaaaaaagatcccattttacggaatgctagact**

**Fortunella_hindsii_1** **tgtgggaatcctaaataaat----aaaaaaaaaagatcccattttacggaatgctagact**

**Fortunella_hindsii_2** **tgtgggaatcctaaataaat----aaaaaaaaaagatcccattttacggaatgctagact**

**Fortunella_hindsii_3** **tgtgggaatcctaaataaat----aaaaaaaaaagatcccattttacggaatgctagact**

**Fortunella_japonica_1** **tgtgggaatcctaaataaat---aaaaaaaaaaagatcccattttacggaatgctagact**

**Fortunella_japonica_2** **tgtgggaatcctaaataaat---aaaaaaaaaaagatcccattttacggaatgctagact**

**Fortunella_japonica_3** **tgtgggaatcctaaataaat---aaaaaaaaaaagatcccattttacggaatgctagact**

**Fortunella_japonica_4** **tgtgggaatcctaaataaat---aaaaaaaaaaagatcccattttacggaatgctagact**

**Fortunella_japonica_5** **tgtgggaatcctaaataaat---aaaaaaaaaaagatcccattttacggaatgctagact**

**Fortunella_japonica_6** **tgtgggaatcctaaataaat---aaaaaaaaaaagatcccattttacggaatgctagact**

**Fortunella_japonica_7** **tgtgggaatcctaaataaat---aaaaaaaaaaagatcccattttacggaatgctagact**

**Fortunella_japonica_8** **tgtgggaatcctaaataaat---aaaaaaaaaaagatcccattttacggaatgctagact**

**Fortunella_japonica_9** **tgtgggaatcctaaataaat---aaaaaaaaaaagatcccattttacggaatgctagact**

**Fortunella_japonica_10** **tgtgggaatcctaaataaat----aaaaaaaaaagatcccattttacggaatgctagact**

**Fortunella_japonica_11** **tgtgggaatcctaaataaat---aaaaaaaaaaagatcccattttacggaatgctagact**

**Fortunella_japonica_12** **tgtgggaatcctaaataaat---aaaaaaaaaaagatcccattttacggaatgctagact**

**Fortunella_margarita_1** **tgtgggaatcctaaataaat----aaaaaaaaaagatcccattttacggaatgctagact**

**Fortunella_margarita_2** **tgtgggaatcctaaataaat----aaaaaaaaaagatcccattttacggaatgctagact**

**Fortunella_venosa_1** **tgtgggaatcctaaataaat----aaaaaaaaaagatcccattttacggaatgctagact**

**Fortunella_venosa_2** **tgtgggaatcctaaataaat----aaaaaaaaaagatcccattttacggaatgctagact**

**Fortunella_venosa_3** **tgtgggaatcctaaataaat----aaaaaaaaaagatcccattttacggaatgctagact**

7150	7160	7170	7180	7190	7200

....|....|....|....|....|....|....|....|....|....|....|....|

**Atalantia_kwangtungens** **attttgtatagatacaaagacaaaagggcccagattaagtcattgtttcctttctaattt**

**Citrus_reticulata_1** **attttgtatagatacaaagacaaaagggcccagattaagtcattgtttcctttctaattt**

**Citrus_reticulata_2** **attttgtatagatacaaagacaaaagggcccagattaagtcattgtttcctttctaattt**

**Citrus_trifoliata_3** **------------------------------------------------------------**

**Citrus_aurantiifolia** **------------------------------------------------------------**

**Citrus_aurantium** **attttgtatagatacaaagacaaaagggcccagattaagtcattgtttcctttctaattt**

**Citrus_cavaleriei** **attttgtatagatacaaagacaaaagggcccagattaagtcattgtttcctttctaattt**

**Citrus_hongheensis** **attttgtatagatacaaagacaaaagggcccagattaagtcattgtttcctttctaattt**

**Citrus_limon** **attttgtatagatacaaagacaaaagggcccagattaagtcattgtttcctttctaattt**

**Citrus_maxima** **attttgtatagatacaaagacaaaagggcccagattaagtcattgtttcctttctaattt**

**Citrus_medica** **attttgtatagatacaaagacaaaagggcccagattaagtcattgtttcctttctaattt**

**Citrus_polytrifolia** **------------------------------------------------------------**

**Citrus_reticulata** **attttgtatagatacaaagacaaaagggcccagattaagtcattgtttcctttctaattt**

**Citrus_sinensis** **attttgtatagatacaaagacaaaagggcccagattaagtcattgtttcctttctaattt**

**Fortunella_bawangica_1** **attttgtatagatacaaagacaaaagggcccagattaagtcattgtttcctttctaattt**

**Fortunella_bawangica_2** **attttgtatagatacaaagacaaaagggcccagattaagtcattgtttcctttctaattt**

**Fortunella_hindsii_1** **attttgtatagatacaaagacaaaagggcccagattaagtcattgtttcctttctaattt**

**Fortunella_hindsii_2** **attttgtatagatacaaagacaaaagggcccagattaagtcattgtttcctttctaattt**

**Fortunella_hindsii_3** **attttgtatagatacaaagacaaaagggcccagattaagtcattgtttcctttctaattt**

**Fortunella_japonica_1** **attttgtatagatacaaagacaaaagggcccagattaagtcattgtttcctttctaattt**

**Fortunella_japonica_2** **attttgtatagatacaaagacaaaagggcccagattaagtcattgtttcctttctaattt**

**Fortunella_japonica_3** **attttgtatagatacaaagacaaaagggcccagattaagtcattgtttcctttctaattt**

**Fortunella_japonica_4** **attttgtatagatacaaagacaaaagggcccagattaagtcattgtttcctttctaattt**

**Fortunella_japonica_5** **attttgtatagatacaaagacaaaagggcccagattaagtcattgtttcctttctaattt**

**Fortunella_japonica_6** **attttgtatagatacaaagacaaaagggcccagattaagtcattgtttcctttctaattt**

**Fortunella_japonica_7** **attttgtatagatacaaagacaaaagggcccagattaagtcattgtttcctttctaattt**

**Fortunella_japonica_8** **attttgtatagatacaaagacaaaagggcccagattaagtcattgtttcctttctaattt**

**Fortunella_japonica_9** **attttgtatagatacaaagacaaaagggcccagattaagtcattgtttcctttctaattt**

**Fortunella_japonica_10** **attttgtatagatacaaagacaaaagggcccagattaagtcattgtttcctttctaattt**

**Fortunella_japonica_11** **attttgtatagatacaaagacaaaagggcccagattaagtcattgtttcctttctaattt**

**Fortunella_japonica_12** **attttgtatagatacaaagacaaaagggcccagattaagtcattgtttcctttctaattt**

**Fortunella_margarita_1** **attttgtatagatacaaagacaaaagggcccagattaagtcattgtttcctttctaattt**

**Fortunella_margarita_2** **attttgtatagatacaaagacaaaagggcccagattaagtcattgtttcctttctaattt**

**Fortunella_venosa_1** **attttgtatagatacaaagacaaaagggcccagattaagtcattgtttcctttctaattt**

**Fortunella_venosa_2** **attttgtatagatacaaagacaaaagggcccagattaagtcattgtttcctttctaattt**

**Fortunella_venosa_3** **attttgtatagatacaaagacaaaagggcccagattaagtcattgtttcctttctaattt**

7210	7220	7230	7240	7250	7260

....|....|....|....|....|....|....|....|....|....|....|....|

**Atalantia_kwangtungens** **-----gttgttttttattttaatctaacctagtcttacttaagagacttaatcccgaatt**

**Citrus_reticulata_1** **-----gttgttttttattttaatctaacctagtcttacttaagagacttaatcccgaatt**

**Citrus_reticulata_2** **-----gttgttttttattttaatctaacctagtcttacttaagagacttaatcccgaatt**

**Citrus_trifoliata_3** **------------------------------------------------------------**

**Citrus_aurantiifolia** **------------------------------------------------------------**

**Citrus_aurantium** **-----gttgttttttattttaatctaacctagtcttacttaagagacttaatcccgaatt**

**Citrus_cavaleriei** **-----gttgttttttattttaatctaacctagtcttacttaagagacttaatcccgaatt**

**Citrus_hongheensis** **-----gttgttttttattttaatctaacctagtcttacttaagagacttaatcccgaatt**

**Citrus_limon** **-----gttgttttttattttaatctaacctagtcttacttaagagacttaatcccgaatt**

**Citrus_maxima** **-----gttgttttttattttaatctaacctagtcttacttaagagacttaatcccgaatt**

**Citrus_medica** **aatttgttgttttttattttaatctaacctagtcttacttaagagacttaatcccgaatt**

**Citrus_polytrifolia** **------------------------------------------------------------**

**Citrus_reticulata** **-----gttgttttttattttaatctaacctagtcttacttaagagacttaatcccgaatt**

**Citrus_sinensis** **-----gttgttttttattttaatctaacctagtcttacttaagagacttaatcccgaatt**

**Fortunella_bawangica_1** **-----gttgttttttattttaatctaacctagtcttacttaagagacttaatcccgaatt**

**Fortunella_bawangica_2** **-----gttgttttttattttaatctaacctagtcttacttaagagacttaatcccgaatt**

**Fortunella_hindsii_1** **-----gttgttttttattttaatctaacctagtcttacttaagagacttaatcccgaatt**

**Fortunella_hindsii_2** **-----gttgttttttattttaatctaacctagtcttacttaagagacttaatcccgaatt**

**Fortunella_hindsii_3** **-----gttgttttttattttaatctaacctagtcttacttaagagacttaatcccgaatt**

**Fortunella_japonica_1** **-----gttgttttttattttaatctaacctagtcttacttaagagacttaatcccgaatt**

**Fortunella_japonica_2** **-----gttgttttttattttaatctaacctagtcttacttaagagacttaatcccgaatt**

**Fortunella_japonica_3** **-----gttgttttttattttaatctaacctagtcttacttaagagacttaatcccgaatt**

**Fortunella_japonica_4** **-----gttgttttttattttaatctaacctagtcttacttaagagacttaatcccgaatt**

**Fortunella_japonica_5** **-----gttgttttttattttaatctaacctagtcttacttaagagacttaatcccgaatt**

**Fortunella_japonica_6** **-----gttgttttttattttaatctaacctagtcttacttaagagacttaatcccgaatt**

**Fortunella_japonica_7** **-----gttgttttttattttaatctaacctagtcttacttaagagacttaatcccgaatt**

**Fortunella_japonica_8** **-----gttgttttttattttaatctaacctagtcttacttaagagacttaatcccgaatt**

**Fortunella_japonica_9** **-----gttgttttttattttaatctaacctagtcttacttaagagacttaatcccgaatt**

**Fortunella_japonica_10** **-----gttgttttttattttaatctaacctagtcttacttaagagacttaatcccgaatt**

**Fortunella_japonica_11** **-----gttgttttttattttaatctaacctagtcttacttaagagacttaatcccgaatt**

**Fortunella_japonica_12** **-----gttgttttttattttaatctaacctagtcttacttaagagacttaatcccgaatt**

**Fortunella_margarita_1** **-----gttgttttttattttaatctaacctagtcttacttaagagacttaatcccgaatt**

**Fortunella_margarita_2** **-----gttgttttttattttaatctaacctagtcttacttaagagacttaatcccgaatt**

**Fortunella_venosa_1** **-----gttgttttttattttaatctaacctagtcttacttaagagacttaatcccgaatt**

**Fortunella_venosa_2** **-----gttgttttttattttaatctaacctagtcttacttaagagacttaatcccgaatt**

**Fortunella_venosa_3** **-----gttgttttttattttaatctaacctagtcttacttaagagacttaatcccgaatt**

7270	7280	7290	7300	7310	7320

....|....|....|....|....|....|....|....|....|....|....|....|

**Atalantia_kwangtungens** **caatcagtaccaggaataccctcttgtttagaattccgaaatgaaaagagaa------ta**

**Citrus_reticulata_1** **caatcagtaccaggaataccctcttgtttagaattccgaaatgaaaagagaa------ta**

**Citrus_reticulata_2** **caatcagtaccaggaataccctcttgtttagaattccgaaatgaaaagagaa------ta**

**Citrus_trifoliata_3** **------------------------------------------------------------**

**Citrus_aurantiifolia** **------------------------------------------------------------**

**Citrus_aurantium** **caatcagtaccaggaataccctcttgtttagaattccgaaatgaaaagagaa------ta**

**Citrus_cavaleriei** **ccatcagtaccaggaataccctcttgtttagaattccgaaatgaaaagagaa------ta**

**Citrus_hongheensis** **caatcagtaccaggaataccctcttgtttagaattccgaaatgaaaagagaa------ta**

**Citrus_limon** **caatcagtaccaggaataccctcttgtttagaattccgaaatgaaaagagaa------ta**

**Citrus_maxima** **caatcagtaccaggaataccctcttgtttagaattccgaaatgaaaagagaa------ta**

**Citrus_medica** **caatcagtatcaggaataccctcttgtttagaattccgaaatgaaaagagaa------ta**

**Citrus_polytrifolia** **------------------------------------------------------------**

**Citrus_reticulata** **caatcagtaccaggaataccctcttgtttagaattccgaaatgaaaagagaa------ta**

**Citrus_sinensis** **caatcagtaccaggaataccctcttgtttagaattccgaaatgaaaagagaa------ta**

**Fortunella_bawangica_1** **caatcagtaccaggaataccctcttgtttagaattccgaaatgaaaagagaa------ta**

**Fortunella_bawangica_2** **caatcagtaccaggaataccctcttgtttagaattccgaaatgaaaagagaa------ta**

**Fortunella_hindsii_1** **caatcagtaccaggaataccctcttgtttagaattccgaaatgaaaagagaa------ta**

**Fortunella_hindsii_2** **caatcagtaccaggaataccctcttgtttagaattccgaaatgaaaagagaa------ta**

**Fortunella_hindsii_3** **caatcagtaccaggaataccctcttgtttagaattccgaaatgaaaagagaa------ta**

**Fortunella_japonica_1** **caatcagtaccaggaataccctcttgtttagaattccgaaatgaaaagagaa------ta**

**Fortunella_japonica_2** **caatcagtaccaggaataccctcttgtttagaattccgaaatgaaaagagaataataata**

**Fortunella_japonica_3** **caatcagtaccaggaataccctcttgtttagaattccgaaatgaaaagagaa------ta**

**Fortunella_japonica_4** **caatcagtaccaggaataccctcttgtttagaattccgaaatgaaaagagaa------ta**

**Fortunella_japonica_5** **caatcagtaccaggaataccctcttgtttagaattccgaaatgaaaagagaa------ta**

**Fortunella_japonica_6** **caatcagtaccaggaataccctcttgtttagaattccgaaatgaaaagagaa------ta**

**Fortunella_japonica_7** **caatcagtaccaggaataccctcttgtttagaattccgaaatgaaaagagaa------ta**

**Fortunella_japonica_8** **caatcagtaccaggaataccctcttgtttagaattccgaaatgaaaagagaa------ta**

**Fortunella_japonica_9** **caatcagtaccaggaataccctcttgtttagaattccgaaatgaaaagagaa------ta**

**Fortunella_japonica_10** **caatcagtaccaggaataccctcttgtttagaattccgaaatgaaaagagaa------ta**

**Fortunella_japonica_11** **caatcagtaccaggaataccctcttgtttagaattccgaaatgaaaagagaa------ta**

**Fortunella_japonica_12** **caatcagtaccaggaataccctcttgtttagaattccgaaatgaaaagagaa------ta**

**Fortunella_margarita_1** **caatcagtaccaggaataccctcttgtttagaattccgaaatgaaaagagaa------ta**

**Fortunella_margarita_2** **caatcagtaccaggaataccctcttgtttagaattccgaaatgaaaagagaa------ta**

**Fortunella_venosa_1** **caatcagtaccaggaataccctcttgtttagaattccgaaatgaaaagagaa------ta**

**Fortunella_venosa_2** **caatcagtaccaggaataccctcttgtttagaattccgaaatgaaaagagaa------ta**

**Fortunella_venosa_3** **caatcagtaccaggaataccctcttgtttagaattccgaaatgaaaagagaa------ta**

7330	7340	7350	7360	7370	7380

....|....|....|....|....|....|....|....|....|....|....|....|

**Atalantia_kwangtungens** **attgggactgtaaaaagataggaatggggaggctttcacatttctatttagaatggatct**

**Citrus_reticulata_1** **attgggattctaaaaagataggaatggggaggctttcccatttcgatttagaacggatct**

**Citrus_reticulata_2** **attgggattctaaaaagataggaatggggaggctttcccatttcgatttagaacggatct**

**Citrus_trifoliata_3** **------------------------------------------------------------**

**Citrus_aurantiifolia** **------------------------------------------------------------**

**Citrus_aurantium** **attgggattctaaaaagataggaatggggaggctttcccatttcgatttagaatggatct**

**Citrus_cavaleriei** **attgggattctaaaaagataggaatggggaggctttcccatttcgatttagaacggatct**

**Citrus_hongheensis** **attgggattctaaaaagataggaatggggaggctttcccatttcgatttagaatggatct**

**Citrus_limon** **attgggattctaaaaagataggaatggggaggctttcccatttcgatttagaatggatct**

**Citrus_maxima** **attgggattctaaaaagataggaatggggaggctttcccatttcgatttagaatggatct**

**Citrus_medica** **attgggattctaaaaagataggaatggggaggctttcccatttcgatttcgaatggattt**

**Citrus_polytrifolia** **------------------------------------------------------------**

**Citrus_reticulata** **attgggattctaaaaagataggaatggggaggctttcccatttcgatttagaacggatct**

**Citrus_sinensis** **attgggattctaaaaagataggaatggggaggctttcccatttcgatttagaatggatct**

**Fortunella_bawangica_1** **attgggattctaaaaagataggaatggggaggctttcccatttcgatttagaatggatct**

**Fortunella_bawangica_2** **attgggattctaaaaagataggaatggggaggctttcccatttcgatttagaatggatct**

**Fortunella_hindsii_1** **attgggattctaaaaagataggaatggggaggctttcccatttcgatttagaatggatct**

**Fortunella_hindsii_2** **attgggattctaaaaagataggaatggggaggctttcccatttcgatttagaatggatct**

**Fortunella_hindsii_3** **attgggattctaaaaagataggaatggggaggctttcccatttcgatttagaatggatct**

**Fortunella_japonica_1** **attgggattctaaaaagataggaatggggaggctttcccatttcgatttagaatggatct**

**Fortunella_japonica_2** **attgggattctaaaaagataggaatggggaggctttcccatttcgatttagaatggatct**

**Fortunella_japonica_3** **attgggattctaaaaagataggaatggggaggctttcccatttcgatttagaatggatct**

**Fortunella_japonica_4** **attgggattctaaaaagataggaatggggaggctttcccatttcgatttagaatggatct**

**Fortunella_japonica_5** **attgggattctaaaaagataggaatggggaggctttcccatttcgatttagaatggatct**

**Fortunella_japonica_6** **attgggattctaaaaagataggaatggggaggctttcccatttcgatttagaatggatct**

**Fortunella_japonica_7** **attgggattctaaaaagataggaatggggaggctttcccatttcgatttagaatggatct**

**Fortunella_japonica_8** **attgggattctaaaaagataggaatggggaggctttcccatttcgatttagaatggatct**

**Fortunella_japonica_9** **attgggattctaaaaagataggaatggggaggctttcccatttcgatttagaatggatct**

**Fortunella_japonica_10** **attgggattctaaaaagataggaatggggaggctttcccatttcgatttagaatggatct**

**Fortunella_japonica_11** **attgggattctaaaaagataggaatggggaggctttcccatttcgatttagaatggatct**

**Fortunella_japonica_12** **attgggattctaaaaagataggaatggggaggctttcccatttcgatttagaatggatct**

**Fortunella_margarita_1** **attgggattctaaaaagataggaatggggaggctttcccatttcgatttagaatggatct**

**Fortunella_margarita_2** **attgggattctaaaaagataggaatggggaggctttcccatttcgatttagaatggatct**

**Fortunella_venosa_1** **attgggattctaaaaagataggaatggggaggctttcccatttcgatttagaatggatct**

**Fortunella_venosa_2** **attgggattctaaaaagataggaatggggaggctttcccatttcgatttagaatggatct**

**Fortunella_venosa_3** **attgggattctaaaaagataggaatggggaggctttcccatttcgatttagaatggatct**

7390	7400	7410	7420	7430	7440

....|....|....|....|....|....|....|....|....|....|....|....|

**Atalantia_kwangtungens** **ttgaaaattcaactcga-----gggggggcgtaagacttttctacgaaactcgcttaaat**

**Citrus_reticulata_1** **ttgaaaattcaattcga----ggggggggcgtcagacttttctacgaaactcgcttaaat**

**Citrus_reticulata_2** **ttgaaaattcaattcga----ggggggggcgtcagacttttctacgaaactcgcttaaat**

**Citrus_trifoliata_3** **------------------------------------------------------------**

**Citrus_aurantiifolia** **------------------------------------------------------------**

**Citrus_aurantium** **ttgaaaattcaattcga----ggggggggcgtcagacttttctacgaaactcgcttaaat**

**Citrus_cavaleriei** **ttgaaaattcaattcgaggggggggggggcgtcagacttttctacgaaactcgcttaaat**

**Citrus_hongheensis** **ttgaaaattcaattcga----ggggggggcgtcagacttttctacgaaactcgcttaaag**

**Citrus_limon** **ttgaaaattcaattcga----ggggggggcgtcagacttttctacgaaactcgcttaaat**

**Citrus_maxima** **ttgaaaattcaattcga----ggggggggcgtcagacttttctacgaaactcgcttaaat**

**Citrus_medica** **ttgaaaattcaattcga-gggggggggggcgtcagacttttctacgaaactcgcttaaat**

**Citrus_polytrifolia** **------------------------------------------------------------**

**Citrus_reticulata** **ttgaaaattcaattcga----ggggggggcgtcagacttttctacgaaactcgcttaaat**

**Citrus_sinensis** **ttgaaaattcaattcga----ggggggggcgtcagacttttctacgaaactcgcttaaat**

**Fortunella_bawangica_1** **ttgaaaattcaattcga----ggggggggcgtcagagttttctacgaaactcgcttaaat**

**Fortunella_bawangica_2** **ttgaaaattcaattcga----ggggggggcgtcagagttttctacgaaactcgcttaaat**

**Fortunella_hindsii_1** **ttgaaaattcaattcga----ggggggggcgtcagagttttctacgaaactcgcttaaat**

**Fortunella_hindsii_2** **ttgaaaattcaattcga----ggggggggcgtcagagttttctacgaaactcgcttaaat**

**Fortunella_hindsii_3** **ttgaaaattcaattcga----ggggggggcgtcagagttttctacgaaactcgcttaaat**

**Fortunella_japonica_1** **ttgaaaattcaattcga----ggggggggcgtcagagttttctacgaaactcgcttaaat**

**Fortunella_japonica_2** **ttgaaaattcaattcga----ggggggggcgtcagagttttctacgaaactcgcttaaat**

**Fortunella_japonica_3** **ttgaaaattcaattcga----ggggggggcgtcagagttttctacgaaactcgcttaaat**

**Fortunella_japonica_4** **ttgaaaattcaattcga----ggggggggcgtcagagttttctacgaaactcgcttaaat**

**Fortunella_japonica_5** **ttgaaaattcaattcga----ggggggggcgtcagagttttctacgaaactcgcttaaat**

**Fortunella_japonica_6** **ttgaaaattcaattcga----ggggggggcgtcagagttttctacgaaactcgcttaaat**

**Fortunella_japonica_7** **ttgaaaattcaattcga----ggggggggcgtcagagttttctacgaaactcgcttaaat**

**Fortunella_japonica_8** **ttgaaaattcaattcga----ggggggggcgtcagagttttctacgaaactcgcttaaat**

**Fortunella_japonica_9** **ttgaaaattcaattcga----ggggggggcgtcagagttttctacgaaactcgcttaaat**

**Fortunella_japonica_10** **ttgaaaattcaattcga----ggggggggcgtcagagttttctacgaaactcgcttaaat**

**Fortunella_japonica_11** **ttgaaaattcaattcga----ggggggggcgtcagagttttctacgaaactcgcttaaat**

**Fortunella_japonica_12** **ttgaaaattcaattcga----ggggggggcgtcagagttttctacgaaactcgcttaaat**

**Fortunella_margarita_1** **ttgaaaattcaattcga----ggggggggcgtcagagttttctacgaaactcgcttaaat**

**Fortunella_margarita_2** **ttgaaaattcaattcga----ggggggggcgtcagagttttctacgaaactcgcttaaat**

**Fortunella_venosa_1** **ttgaaaattcaattcga---gggggggggcgtcagagttttctacgaaactcgcttaaat**

**Fortunella_venosa_2** **ttgaaaattcaattcga---gggggggggcgtcagagttttctacgaaactcgcttaaat**

**Fortunella_venosa_3** **ttgaaaattcaattcga---gggggggggcgtcagagttttctacgaaactcgcttaaat**

7450	7460	7470	7480	7490	7500

....|....|....|....|....|....|....|....|....|....|....|....|

**Atalantia_kwangtungens** **at----gaaagtgaatga------aagaggggggcatacgcaaaaacgcccatccaacgt**

**Citrus_reticulata_1** **at----gaaagtgaatgaaagaggaagaggggggcatacgcaaaaacgcccatccaacgt**

**Citrus_reticulata_2** **at----gaaagtgaatgaaagaggaagaggggggcatacgcaaaaacgcccatccaacgt**

**Citrus_trifoliata_3** **------------------------------------------------------------**

**Citrus_aurantiifolia** **------------------------------------------------------------**

**Citrus_aurantium** **at----gaaagtgaatgaaagaggaagaggggggcatacgcaaaaacgcccatccaacgt**

**Citrus_cavaleriei** **at----gaaagtgaatgaaagaggaagaggggggcatacgcaaaaacgcccatccaacgt**

**Citrus_hongheensis** **at----gaaagtgaatgaaagaggaagaggggggcatacgcaaaaacgcccatccaacgt**

**Citrus_limon** **at----gaaagtgaatgaaagaggaagaggggggcatacgcaaaaacgcccatccaacgt**

**Citrus_maxima** **at----gaaagtgaatgaaagaggaagaggggggcatacgcaaaaacgcccatccaacgt**

**Citrus_medica** **atgaaagaaagtgaatgaaagaggaagaggggggcatacgcaaaaacgcccatccaacgt**

**Citrus_polytrifolia** **------------------------------------------------------------**

**Citrus_reticulata** **at----gaaagtgaatgaaagaggaagaggggggcatacgcaaaaacgcccatccaacgt**

**Citrus_sinensis** **at----gaaagtgaatgaaagaggaagaggggggcatacgcaaaaacgcccatccaacgt**

**Fortunella_bawangica_1** **at----gaaagtgaatgaaagaggaagaggggggcatacgcaaaaacgcccatccaacgt**

**Fortunella_bawangica_2** **at----gaaagtgaatgaaagaggaagaggggggcatacgcaaaaacgcccatccaacgt**

**Fortunella_hindsii_1** **at----gaaagtgaatgaaagaggaagaggggggcatacgcaaaaacgcccatccaacgt**

**Fortunella_hindsii_2** **at----gaaagtgaatgaaagaggaagaggggggcatacgcaaaaacgcccatccaacgt**

**Fortunella_hindsii_3** **at----gaaagtgaatgaaagaggaagaggggggcatacgcaaaaacgcccatccaacgt**

**Fortunella_japonica_1** **at----gaaagtgaatgaaagaggaagaggggggcatacgcaaaaacgcccatccaacgt**

**Fortunella_japonica_2** **at----gaaagtgaatgaaagaggaagaggggggcatacgcaaaaacgcccatccaacgt**

**Fortunella_japonica_3** **at----gaaagtgaatgaaagaggaagaggggggcatacgcaaaaacgcccatccaacgt**

**Fortunella_japonica_4** **at----gaaagtgaatgaaagaggaagaggggggcatacgcaaaaacgcccatccaacgt**

**Fortunella_japonica_5** **at----gaaagtgaatgaaagaggaagaggggggcatacgcaaaaacgcccatccaacgt**

**Fortunella_japonica_6** **at----gaaagtgaatgaaagaggaagaggggggcatacgcaaaaacgcccatccaacgt**

**Fortunella_japonica_7** **at----gaaagtgaatgaaagaggaagaggggggcatacgcaaaaacgcccatccaacgt**

**Fortunella_japonica_8** **at----gaaagtgaatgaaagaggaagaggggggcatacgcaaaaacgcccatccaacgt**

**Fortunella_japonica_9** **at----gaaagtgaatgaaagaggaagaggggggcatacgcaaaaacgcccatccaacgt**

**Fortunella_japonica_10** **at----gaaagtgaatgaaagaggaagaggggggcatacgcaaaaacgcccatccaacgt**

**Fortunella_japonica_11** **at----gaaagtgaatgaaagaggaagaggggggcatacgcaaaaacgcccatccaacgt**

**Fortunella_japonica_12** **at----gaaagtgaatgaaagaggaagaggggggcatacgcaaaaacgcccatccaacgt**

**Fortunella_margarita_1** **at----gaaagtgaatgaaagaggaagaggggggcatacgcaaaaacgcccatccaacgt**

**Fortunella_margarita_2** **at----gaaagtgaatgaaagaggaagaggggggcatacgcaaaaacgcccatccaacgt**

**Fortunella_venosa_1** **at----gaaagtgaatgaaagaggaagaggggggcatacgcaaaaacgcccatccaacgt**

**Fortunella_venosa_2** **at----gaaagtgaatgaaagaggaagaggggggcatacgcaaaaacgcccatccaacgt**

**Fortunella_venosa_3** **at----gaaagtgaatgaaagaggaagaggggggcatacgcaaaaacgcccatccaacgt**

7510	7520	7530	7540	7550	7560

....|....|....|....|....|....|....|....|....|....|....|....|

**Atalantia_kwangtungens** **tttttaattcaaactcttgtgatcgatgttccccacttgcggggaccacaaatgcattca**

**Citrus_reticulata_1** **ttttggattcaaactcttgtgatcgatgttccccgcttgcggggaccacaaatgcattca**

**Citrus_reticulata_2** **ttttggattcaaactcttgtgatcgatgttccccgcttgcggggaccacaaatgcattca**

**Citrus_trifoliata_3** **------------------------------------------------------------**

**Citrus_aurantiifolia** **------------------------------------------------------------**

**Citrus_aurantium** **tttttaattcaaactcttgtgatcgatgttccccgcttgcggggaccacaaatgcattca**

**Citrus_cavaleriei** **tttttaattcaaactcttgtgatcgatgttccccgcttgcggggaccacaaatgcattca**

**Citrus_hongheensis** **tttttaattcaaactcttgtgatcgatgttccccgcttgcggggaccacaaatgcattca**

**Citrus_limon** **tttttaattcaaactcttgtgatcgatgttccccgcttgcggggaccacaaatgcattca**

**Citrus_maxima** **tttttaattcaaactcttgtgatcgatgttccccgcttgcggggaccacaaatgcattca**

**Citrus_medica** **tttttaattcaaactcttgtgatcgatgttccccgcttgcggggaacacaaatgcattca**

**Citrus_polytrifolia** **------------------------------------------------------------**

**Citrus_reticulata** **ttttggattcaaactcttgtgatcgatgttccccgcttgcggggaccacaaatgcattca**

**Citrus_sinensis** **tttttaattcaaactcttgtgatcgatgttccccgcttgcggggaccacaaatgcattca**

**Fortunella_bawangica_1** **tttttaattcaaactcttgtgatcgatgttccccgcttgcggggaccacaaatgcattca**

**Fortunella_bawangica_2** **tttttaattcaaactcttgtgatcgatgttccccgcttgcggggaccacaaatgcattca**

**Fortunella_hindsii_1** **tttttaattcaaactcttgtgatcgatgttccccgcttgcggggaccacaaatgcattca**

**Fortunella_hindsii_2** **tttttaattcaaactcttgtgatcgatgttccccgcttgcggggaccacaaatgcattca**

**Fortunella_hindsii_3** **tttttaattcaaactcttgtgatcgatgttccccgcttgcggggaccacaaatgcattca**

**Fortunella_japonica_1** **tttttaattcaaactcttgtgatcgatgttccccgcttgcggggaccacaaatgcattca**

**Fortunella_japonica_2** **tttttaattcaaactcttgtgatcgatgttccccgcttgcggggaccacaaatgcattca**

**Fortunella_japonica_3** **tttttaattcaaactcttgtgatcgatgttccccgcttgcggggaccacaaatgcattca**

**Fortunella_japonica_4** **tttttaattcaaactcttgtgatcgatgttccccgcttgcggggaccacaaatgcattca**

**Fortunella_japonica_5** **tttttaattcaaactcttgtgatcgatgttccccgcttgcggggaccacaaatgcattca**

**Fortunella_japonica_6** **tttttaattcaaactcttgtgatcgatgttccccgcttgcggggaccacaaatgcattca**

**Fortunella_japonica_7** **tttttaattcaaactcttgtgatcgatgttccccgcttgcggggaccacaaatgcattca**

**Fortunella_japonica_8** **tttttaattcaaactcttgtgatcgatgttccccgcttgcggggaccacaaatgcattca**

**Fortunella_japonica_9** **tttttaattcaaactcttgtgatcgatgttccccgcttgcggggaccacaaatgcattca**

**Fortunella_japonica_10** **tttttaattcaaactcttgtgatcgatgttccccgcttgcggggaccacaaatgcattca**

**Fortunella_japonica_11** **tttttaattcaaactcttgtgatcgatgttccccgcttgcggggaccacaaatgcattca**

**Fortunella_japonica_12** **tttttaattcaaactcttgtgatcgatgttccccgcttgcggggaccacaaatgcattca**

**Fortunella_margarita_1** **tttttaattcaaactcttgtgatcgatgttccccgcttgcggggaccacaaatgcattca**

**Fortunella_margarita_2** **tttttaattcaaactcttgtgatcgatgttccccgcttgcggggaccacaaatgcattca**

**Fortunella_venosa_1** **tttttaattcaaactcttgtgatcgatgttccccgcttgcggggaccacaaatgcattca**

**Fortunella_venosa_2** **tttttaattcaaactcttgtgatcgatgttccccgcttgcggggaccacaaatgcattca**

**Fortunella_venosa_3** **tttttaattcaaactcttgtgatcgatgttccccgcttgcggggaccacaaatgcattca**

7570	7580	7590	7600	7610	7620

....|....|....|....|....|....|....|....|....|....|....|....|

**Atalantia_kwangtungens** **gttcaatcttcgtattatttgaattcgattcaatttgtgaaaaaagatcttgttgaaaaa**

**Citrus_reticulata_1** **gttccattttcgtattatttgaattcgattcaatttgtgaaaaaagatccggttgagaaa**

**Citrus_reticulata_2** **gttccattttcgtattatttgaattcgattcaatttgtgaaaaaagatccggttgagaaa**

**Citrus_trifoliata_3** **------------------------------------------------------------**

**Citrus_aurantiifolia** **------------------------------------------------------------**

**Citrus_aurantium** **gttccattttcgtattatttgaattcgattcaatttgtgaaaaaagatctggttgagaaa**

**Citrus_cavaleriei** **gttccattttcgtattatttgaattcgattcaatttgtgaaaaaggatctggttgagaaa**

**Citrus_hongheensis** **gttccattttcgtattatttgaattcgattcaatttgtgaaaaaagatctggttgagaaa**

**Citrus_limon** **gttccattttcgtattatttgaattcgattcaatttgtgaaaaaagatctggttgagaaa**

**Citrus_maxima** **gttccattttcgtattatttgaattcgattcaatttgtgaaaaaagatctggttgagaaa**

**Citrus_medica** **gttccattttcgtattatttgaattcgattcaatttgtgaaaaaagatctggttgagaaa**

**Citrus_polytrifolia** **------------------------------------------------------------**

**Citrus_reticulata** **gttccattttcgtattatttgaattcgattcaatttgtgaaaaaagatccggttgagaaa**

**Citrus_sinensis** **gttccattttcgtattatttggattcgattcaatttgtgaaaaaagatctggttgagaaa**

**Fortunella_bawangica_1** **gttccattttcgtattatttgaattcgattcaatttgtgaaaaaagatctggttgagaaa**

**Fortunella_bawangica_2** **gttccattttcgtattatttgaattcgattcaatttgtgaaaaaagatctggttgagaaa**

**Fortunella_hindsii_1** **gttccattttcgtattatttgaattcgattcaatttgtgaaaaaagatctggttgagaaa**

**Fortunella_hindsii_2** **gttccattttcgtattatttgaattcgattcaatttgtgaaaaaagatctggttgagaaa**

**Fortunella_hindsii_3** **gttccattttcgtattatttgaattcgattcaatttgtgaaaaaagatctggttgagaaa**

**Fortunella_japonica_1** **gttccattttcgtattatttgaattcgattcaatttgtgaaaaaagatctggttgagaaa**

**Fortunella_japonica_2** **gttccattttcgtattatttgaattcgattcaatttgtgaaaaaagatctggttgagaaa**

**Fortunella_japonica_3** **gttccattttcgtattatttgaattcgattcaatttgtgaaaaaagatctggttgagaaa**

**Fortunella_japonica_4** **gttccattttcgtattatttgaattcgattcaatttgtgaaaaaagatctggttgagaaa**

**Fortunella_japonica_5** **gttccattttcgtattatttgaattcgattcaatttgtgaaaaaagatctggttgagaaa**

**Fortunella_japonica_6** **gttccattttcgtattatttgaattcgattcaatttgtgaaaaaagatctggttgagaaa**

**Fortunella_japonica_7** **gttccattttcgtattatttgaattcgattcaatttgtgaaaaaagatctggttgagaaa**

**Fortunella_japonica_8** **gttccattttcgtattatttgaattcgattcaatttgtgaaaaaagatctggttgagaaa**

**Fortunella_japonica_9** **gttccattttcgtattatttgaattcgattcaatttgtgaaaaaagatctggttgagaaa**

**Fortunella_japonica_10** **gttccattttcgtattatttgaattcgattcaatttgtgaaaaaagatctggttgagaaa**

**Fortunella_japonica_11** **gttccattttcgtattatttgaattcgattcaatttgtgaaaaaagatctggttgagaaa**

**Fortunella_japonica_12** **gttccattttcgtattatttgaattcgattcaatttgtgaaaaaagatctggttgagaaa**

**Fortunella_margarita_1** **gttccattttcgtattatttgaattcgattcaatttgtgaaaaaagatctggttgagaaa**

**Fortunella_margarita_2** **gttccattttcgtattatttgaattcgattcaatttgtgaaaaaagatctggttgagaaa**

**Fortunella_venosa_1** **gttccattttcgtattatttgaattcgattcaatttgtgaaaaaagatctggttgagaaa**

**Fortunella_venosa_2** **gttccattttcgtattatttgaattcgattcaatttgtgaaaaaagatctggttgagaaa**

**Fortunella_venosa_3** **gttccattttcgtattatttgaattcgattcaatttgtgaaaaaagatctggttgagaaa**

7630	7640	7650	7660	7670	7680

....|....|....|....|....|....|....|....|....|....|....|....|

**Atalantia_kwangtungens** **ataatttttttttttatttttaatt------------------------agaaaaaaaaa**

**Citrus_reticulata_1** **ataatttttt---------tttattcga------aaaaaaa--------aaaaaaaaaaa**

**Citrus_reticulata_2** **ataatttttt---------tttattcg-------aaaaaaa--------aaaaaaaaaaa**

**Citrus_trifoliata_3** **-------------------------------------------------aaaaaaaaaaa**

**Citrus_aurantiifolia** **---------------------------------------------------aaaaaaaaa**

**Citrus_aurantium** **ataattttt----------ttaattcgaa----aaaaaaaa--------aaaaaaagaaa**

**Citrus_cavaleriei** **ataatttttttttttttttttttttcg-------------a--------ataaaaaaaaa**

**Citrus_hongheensis** **ataattttt----------ttaattcga------aaaaaaa--------aaaaaaagaaa**

**Citrus_limon** **ataattttt----------ttaattcgaa----aaaaaaaa--------aaaaaaagaaa**

**Citrus_maxima** **ataattttt----------ttaattcga------aaaaaaa--------aaaaaaagaaa**

**Citrus_medica** **ataattttt----------ttaattcgaaaa------------------aaaaaaaaaaa**

**Citrus_polytrifolia** **-------------------------------------------------aaaaaaaaaaa**

**Citrus_reticulata** **ataatttttt---------tttattcg-------aaaaaaa--------aaaaaaaaaaa**

**Citrus_sinensis** **ataattttt----------ttaattcg--------aaaaaa--------aaaaaaagaaa**

**Fortunella_bawangica_1** **ataattttt----------ttaattcgaaaaaaaaaaaaaa---ga---aaaaaaaaaaa**

**Fortunella_bawangica_2** **ataattttt----------ttaattcgaaaaaaaaaaaaaa---ga---aaaaaaaaaaa**

**Fortunella_hindsii_1** **ataattttt----------ttaattcgaaaaaaaaaagaa---------aaaaaaaaaaa**

**Fortunella_hindsii_2** **ataattttt----------ttaattcgaaaaaaaaaag-a---------aaaaaaaaaaa**

**Fortunella_hindsii_3** **ataattttt----------ttaattcgaaaaaaaaaaaaa---------gaaaaaaaaaa**

**Fortunella_japonica_1** **ataattttt----------ttaattcgaaaaaaaaaaaaa----gaa--aaaaaaaaaaa**

**Fortunella_japonica_2** **ataattttt----------ttaattcgaaaaaaaaaaaaaa---gaa--aaaaaaaaaaa**

**Fortunella_japonica_3** **ataattttt----------ttaattcgaaaaaaaaaaaaa----gaa--aaaaaaaaaaa**

**Fortunella_japonica_4** **ataattttt----------ttaattcgaaaaaaaaaaaaa----gaa--aaaaaaaaaaa**

**Fortunella_japonica_5** **ataattttt----------ttaattcgaaaaaaaaaaaaa----gaa--aaaaaaaaaaa**

**Fortunella_japonica_6** **ataattttt----------ttaattcgaaaaaaaaaaaaa----gaa--aaaaaaaaaaa**

**Fortunella_japonica_7** **ataattttt----------ttaattcgaaaaaaaaaaaaa----gaa--aaaaaaaaaaa**

**Fortunella_japonica_8** **ataattttt----------ttaattcgaaaaaaaaaaaaa----gaa--aaaaaaaaaaa**

**Fortunella_japonica_9** **ataattttt----------ttaattcgaaaaaaaaaaaaa----gaa--aaaaaaaaaaa**

**Fortunella_japonica_10** **ataattttt----------ttaattcgaaaaaaaaaaaaaa---ga---aaaaaaaaaaa**

**Fortunella_japonica_11** **ataattttt----------ttaattcgaaaaaaaaaaaaaaaagaaa--aaaaaaaaaaa**

**Fortunella_japonica_12** **ataattttt----------ttaattcgaaaaaaaaaaaaa----gaa--aaaaaaaaaaa**

**Fortunella_margarita_1** **ataattttt----------ttaattcgaaaaaaaaaaaaaa---ga---aaaaaaaaaaa**

**Fortunella_margarita_2** **ataattttt----------ttaattcgaaaaaaaaaaaaaa---ga---aaaaaaaaaaa**

**Fortunella_venosa_1** **ataattttt----------ttaattcgaaaaaaaaaaaaaa---aaga-aaaaaaaaaaa**

**Fortunella_venosa_2** **ataattttt----------ttaattcgaaaaaaaaaaaaaa---aaaagaaaaaaaaaaa**

**Fortunella_venosa_3** **ataattttt----------ttaattcgaaaaaaaaaaaaaa---aaaa-aaaaaaaaaaa**

7690	7700	7710	7720	7730	7740

....|....|....|....|....|....|....|....|....|....|....|....|

**Atalantia_kwangtungens** **aagacgtacacgtatattttatcaatatatatacttaagagggttgctcaaaagggaatt**

**Citrus_reticulata_1** **aagacgtacacgtatattttatca--atatatacttaagagggttgcgcaaacgggaatt**

**Citrus_reticulata_2** **aagacgtacacgtatattttatca--atatatacttaagagggttgcgcaaacgggaatt**

**Citrus_trifoliata_3** **aagacgtacacgtatattttatca--atatatacttaagagggttgctcaaacgggaatt**

**Citrus_aurantiifolia** **aagacgtacacgtatattttatca--atatatacttaagagggttgctcaaacgggaatt**

**Citrus_aurantium** **aagacgtacacgtatattttatca--atatatacttaagagggttgctcaaacgggaatt**

**Citrus_cavaleriei** **aagacgtacacgtatattttatca--atatatacttaagagggttgcgcaaacgggaatt**

**Citrus_hongheensis** **aagacgtacacgtatattttatca--atatatacttaagagggttgctcaaacgggaatt**

**Citrus_limon** **aagacgtacacgtatattttatca--atatatacttaagagggttgctcaaacgggaatt**

**Citrus_maxima** **aagacgtacacgtatattttatca--atatatacttaagagggttgctcaaacgggaatt**

**Citrus_medica** **aagacgtacacgtatattttatca--atatatacttaagagggttgctcaaacgggaatt**

**Citrus_polytrifolia** **aagacgtacacgtatattttatca--atatatacttaagagggttgctcaaacgggaatt**

**Citrus_reticulata** **aagacgtacacgtatattttatca--atatatacttaagagggttgcgcaaacgggaatt**

**Citrus_sinensis** **aagacgtacacgtatattttatca--atatatacttaagagggttgctcaaacgggaatt**

**Fortunella_bawangica_1** **aagacgtacacgtatattttatca--atatatacttaagagggttgctcaaacgggaatt**

**Fortunella_bawangica_2** **aagacgtacacgtatattttatca--atatatacttaagagggttgctcaaacgggaatt**

**Fortunella_hindsii_1** **aagacgtacacgtatattttatca--atatatacttaagagggttgctcaaacgggaatt**

**Fortunella_hindsii_2** **aagacgtacacgtatattttatca--atatatacttaagagggttgctcaaacgggaatt**

**Fortunella_hindsii_3** **aagacgtacacgtatattttatca--atatatacttaagagggttgctcaaacgggaatt**

**Fortunella_japonica_1** **aagacgtacacgtatattttatca--atatatacttaagagggttgctcaaacgggaatt**

**Fortunella_japonica_2** **aagacgtacacgtatattttatca--atatatacttaagagggttgctcaaacgggaatt**

**Fortunella_japonica_3** **aagacgtacacgtatattttatca--atatatacttaagagggttgctcaaacgggaatt**

**Fortunella_japonica_4** **aagacgtacacgtatattttatca--atatatacttaagagggttgctcaaacgggaatt**

**Fortunella_japonica_5** **aagacgtacacgtatattttatca--atatatacttaagagggttgctcaaacgggaatt**

**Fortunella_japonica_6** **aagacgtacacgtatattttatca--atatatacttaagagggttgctcaaacgggaatt**

**Fortunella_japonica_7** **aagacgtacacgtatattttatca--atatatacttaagagggttgctcaaacgggaatt**

**Fortunella_japonica_8** **aagacgtacacgtatattttatca--atatatacttaagagggttgctcaaacgggaatt**

**Fortunella_japonica_9** **aagacgtacacgtatattttatca--atatatacttaagagggttgctcaaacgggaatt**

**Fortunella_japonica_10** **aagacgtacacgtatattttatca--atatatacttaagagggttgctcaaacgggaatt**

**Fortunella_japonica_11** **aagacgtacacgtatattttatca--atatatacttaagagggttgctcaaacgggaatt**

**Fortunella_japonica_12** **aagacgtacacgtatattttatca--atatatacttaagagggttgctcaaacgggaatt**

**Fortunella_margarita_1** **aagacgtacacgtatattttatca--atatatacttaagagggttgctcaaacgggaatt**

**Fortunella_margarita_2** **aagacgtacacgtatattttatca--atatatacttaagagggttgctcaaacgggaatt**

**Fortunella_venosa_1** **aagacgtacacgtatattttatca--atatatacttaagagggttgctcaaacgggaatt**

**Fortunella_venosa_2** **aagacgtacacgtatattttatca--atatatacttaagagggttgctcaaacgggaatt**

**Fortunella_venosa_3** **aagacgtacacgtatattttatca--atatatacttaagagggttgctcaaacgggaatt**

7750	7760	7770	7780	7790	7800

....|....|....|....|....|....|....|....|....|....|....|....|

**Atalantia_kwangtungens** **gaaaattttgattctgcccggtctgggacggaaggattcgaacctccgaataccgggacc**

**Citrus_reticulata_1** **gaaaatttttattctgcccggtctgggacggaaggattcgaacctccgaataccgggacc**

**Citrus_reticulata_2** **gaaaatttttattctgcccggtctgggacggaaggattcgaacctccgaataccgggacc**

**Citrus_trifoliata_3** **gaaaatttttattctgcccggtctgggacggaaggattcgaacctccgaataccgggacc**

**Citrus_aurantiifolia** **gaaaatttttattctgcccggtctgggacggaaggattcgaacctccgaataccgggacc**

**Citrus_aurantium** **gaaaatttttattctgcccggtctgggacggaaggattcgaacctccgaataccgggacc**

**Citrus_cavaleriei** **gaaaatttttattctgcccggtctgggacggaaggattcgaacctccgaataccgggacc**

**Citrus_hongheensis** **gcaaatttttattctgcccggtctgggacggaaggattcgaacctccgaataccgggacc**

**Citrus_limon** **gaaaatttttattctgcccggtctgggacggaaggattcgaacctccgaataccgggacc**

**Citrus_maxima** **gaaaatttttattctgcccggtctgggacggaaggattcgaacctccgaataccgggacc**

**Citrus_medica** **gaaaatttttattctgcccggtctgggacggaaggattcgaacctccgaataccgggacc**

**Citrus_polytrifolia** **gaaaatttttattctgcccggtctgggacggaaggattcgaacctccgaataccgggacc**

**Citrus_reticulata** **gaaaatttttattctgcccggtctgggacggaaggattcgaacctccgaataccgggacc**

**Citrus_sinensis** **gaaaatttttattctgcccggtctgggacggaaggattcgaacctccgaataccgggacc**

**Fortunella_bawangica_1** **gaaaatttttattctgcccggtctgggacggaaggattcgaacctccgaataccgggacc**

**Fortunella_bawangica_2** **gaaaatttttattctgcccggtctgggacggaaggattcgaacctccgaataccgggacc**

**Fortunella_hindsii_1** **gaaaatttttattctgcccggtctgggacggaaggattcgaacctccgaataccgggacc**

**Fortunella_hindsii_2** **gaaaatttttattctgcccggtctgggacggaaggattcgaacctccgaataccgggacc**

**Fortunella_hindsii_3** **gaaaatttttattctgcccggtctgggacggaaggattcgaacctccgaataccgggacc**

**Fortunella_japonica_1** **gaaaatttttattctgcccggtctgggacggaaggattcgaacctccgaataccgggacc**

**Fortunella_japonica_2** **gaaaatttttattctgcccggtctgggacggaaggattcgaacctccgaataccgggacc**

**Fortunella_japonica_3** **gaaaatttttattctgcccggtctgggacggaaggattcgaacctccgaataccgggacc**

**Fortunella_japonica_4** **gaaaatttttattctgcccggtctgggacggaaggattcgaacctccgaataccgggacc**

**Fortunella_japonica_5** **gaaaatttttattctgcccggtctgggacggaaggattcgaacctccgaataccgggacc**

**Fortunella_japonica_6** **gaaaatttttattctgcccggtctgggacggaaggattcgaacctccgaataccgggacc**

**Fortunella_japonica_7** **gaaaatttttattctgcccggtctgggacggaaggattcgaacctccgaataccgggacc**

**Fortunella_japonica_8** **gaaaatttttattctgcccggtctgggacggaaggattcgaacctccgaataccgggacc**

**Fortunella_japonica_9** **gaaaatttttattctgcccggtctgggacggaaggattcgaacctccgaataccgggacc**

**Fortunella_japonica_10** **gaaaatttttattctgcccggtctgggacggaaggattcgaacctccgaataccgggacc**

**Fortunella_japonica_11** **gaaaatttttattctgcccggtctgggacggaaggattcgaacctccgaataccgggacc**

**Fortunella_japonica_12** **gaaaatttttattctgcccggtctgggacggaaggattcgaacctccgaataccgggacc**

**Fortunella_margarita_1** **gaaaatttttattctgcccggtctgggacggaaggattcgaacctccgaataccgggacc**

**Fortunella_margarita_2** **gaaaatttttattctgcccggtctgggacggaaggattcgaacctccgaataccgggacc**

**Fortunella_venosa_1** **gaaaatttttattctgcccggtctgggacggaaggattcgaacctccgaataccgggacc**

**Fortunella_venosa_2** **gaaaatttttattctgcccggtctgggacggaaggattcgaacctccgaataccgggacc**

**Fortunella_venosa_3** **gaaaatttttattctgcccggtctgggacggaaggattcgaacctccgaataccgggacc**

7810	7820	7830	7840	7850	7860

....|....|....|....|....|....|....|....|....|....|....|....|

**Atalantia_kwangtungens** **aaaacccgttgccttaccacttggcgacgccccatttcaatttcgatttggtactaatac**

**Citrus_reticulata_1** **aaaacccgttgccttaccacttggcgacgccccatttcaatttcgatttggtac------**

**Citrus_reticulata_2** **aaaacccgttgccttaccacttggcgacgccccatttcaatttcgatttggtac------**

**Citrus_trifoliata_3** **aaaacccgttgccttaccacttggcgacgccccatttcaatttcgatttggtac------**

**Citrus_aurantiifolia** **aaaacccgttgccttaccacttggcgacgccccatttgaatttcgatttggtac------**

**Citrus_aurantium** **aaaacccgttgccttaccacttggcgacgccccatttcaatttcgatttggtac------**

**Citrus_cavaleriei** **aaaacccgttgccttaccacttggcgacgccccatttcaatttcgatttggtac------**

**Citrus_hongheensis** **aaaacccgttgccttaccacttggcgacgccccatttcaatttcgatttggtac------**

**Citrus_limon** **aaaacccgttgccttaccacttggcgacgccccatttcaatttcgatttggtac------**

**Citrus_maxima** **aaaacccgttgccttaccacttggcgacgccccatttcaatttcgatttggtac------**

**Citrus_medica** **aaaacccgttgccttaccacttggcgacgccccatttcaatttcgatttggtac------**

**Citrus_polytrifolia** **aaaacccgttgccttaccacttggcgacgccccatttcaatttcgatttggtac------**

**Citrus_reticulata** **aaaacccgttgccttaccacttggcgacgccccatttcaatttcgatttggtac------**

**Citrus_sinensis** **aaaacccgttgccttaccacttggcgacgccccatttcaatttcgatttggtac------**

**Fortunella_bawangica_1** **aaaacccgttgccttaccacttggcgacgccccatttcaatttcgatttggtac------**

**Fortunella_bawangica_2** **aaaacccgttgccttaccacttggcgacgccccatttcaatttcgatttggtac------**

**Fortunella_hindsii_1** **aaaacccgttgccttaccacttggcgacgccccatttcaatttcgatttggtac------**

**Fortunella_hindsii_2** **aaaacccgttgccttaccacttggcgacgccccatttcaatttcgatttggtac------**

**Fortunella_hindsii_3** **aaaacccgttgccttaccacttggcgacgccccatttcaatttcgatttggtac------**

**Fortunella_japonica_1** **aaaacccgttgccttaccacttggcgacgccccatttcaatttcgatttggtac------**

**Fortunella_japonica_2** **aaaacccgttgccttaccacttggcgacgccccatttcaatttcgatttggtac------**

**Fortunella_japonica_3** **aaaacccgttgccttaccacttggcgacgccccatttcaatttcgatttggtac------**

**Fortunella_japonica_4** **aaaacccgttgccttaccacttggcgacgccccatttcaatttcgatttggtac------**

**Fortunella_japonica_5** **aaaacccgttgccttaccacttggcgacgccccatttcaatttcgatttggtac------**

**Fortunella_japonica_6** **aaaacccgttgccttaccacttggcgacgccccatttcaatttcgatttggtac------**

**Fortunella_japonica_7** **aaaacccgttgccttaccacttggcgacgccccatttcaatttcgatttggtac------**

**Fortunella_japonica_8** **aaaacccgttgccttaccacttggcgacgccccatttcaatttcgatttggtac------**

**Fortunella_japonica_9** **aaaacccgttgccttaccacttggcgacgccccatttcaatttcgatttggtac------**

**Fortunella_japonica_10** **aaaacccgttgccttaccacttggcgacgccccatttcaatttcgatttggtac------**

**Fortunella_japonica_11** **aaaacccgttgccttaccacttggcgacgccccatttcaatttcgatttggtac------**

**Fortunella_japonica_12** **aaaacccgttgccttaccacttggcgacgccccatttcaatttcgatttggtac------**

**Fortunella_margarita_1** **aaaacccgttgccttaccacttggcgacgccccatttcaatttcgatttggtac------**

**Fortunella_margarita_2** **aaaacccgttgccttaccacttggcgacgccccatttcaatttcgatttggtac------**

**Fortunella_venosa_1** **aaaacccgttgccttaccacttggcgacgccccatttcaatttcgatttggtac------**

**Fortunella_venosa_2** **aaaacccgttgccttaccacttggcgacgccccatttcaatttcgatttggtac------**

**Fortunella_venosa_3** **aaaacccgttgccttaccacttggcgacgccccatttcaatttcgatttggtac------**

7870	7880	7890	7900	7910	7920

....|....|....|....|....|....|....|....|....|....|....|....|

**Atalantia_kwangtungens** **taataaagagtaccattggtattggctgttcgtcaattccagtccaaagcccaaaaatga**

**Citrus_reticulata_1** **taataaagagtaccagtggtattggctgttcgtcaattccagtccaaagccccaaaattc**

**Citrus_reticulata_2** **taataaagagtaccagtggtattggctgttcgtcaattccagtccaaagccccaaaattc**

**Citrus_trifoliata_3** **taataaagagtaccagtggtattggctgttcgtcaattccaggccaaagccccaaaattc**

**Citrus_aurantiifolia** **taataaagagtaccagtggtattggctgttcgtcaattccagtccaaagccccaaaattc**

**Citrus_aurantium** **taataaagagtaccagtggtattggctgttcgtcaattccagtccaaagccccaaaattc**

**Citrus_cavaleriei** **taataaagagtaccagtggtattggctgttcgtcaattccagtccaaagccccaaaattc**

**Citrus_hongheensis** **taataaagagtacctgtggtattggctgttcgtcaattccagtccaaagccccaaaattc**

**Citrus_limon** **taataaagagtaccagtggtattggctgttcgtcaattccagtccaaagccccaaaattc**

**Citrus_maxima** **taataaagagtaccagtggtattggctgttcgtcaattccagtccaaagccccaaaattc**

**Citrus_medica** **taataaagagtaccagtggtattggctgttcgtcaattccagtccaaagccccaaaatta**

**Citrus_polytrifolia** **taataaagagtaccagtggtattggctgttcgtcaattccaggccaaagccccaaaattc**

**Citrus_reticulata** **taataaagagtaccagtggtattggctgttcgtcaattccagtccaaagccccaaaattc**

**Citrus_sinensis** **taataaagagtaccagtggtattggctgttcgtcaattccagtccaaagccccaaaattc**

**Fortunella_bawangica_1** **taataaagagtaccagtggtattggctgttcgtcaattccactccaaagccccaaaattc**

**Fortunella_bawangica_2** **taataaagagtaccagtggtattggctgttcgtcaattccactccaaagccccaaaattc**

**Fortunella_hindsii_1** **taataaagagtaccagtggtattggctgttcgtcaattccactccaaagccccaaaattc**

**Fortunella_hindsii_2** **taataaagagtaccagtggtattggctgttcgtcaattccactccaaagccccaaaattc**

**Fortunella_hindsii_3** **taataaagagtaccagtggtattggctgttcgtcaattccactccaaagccccaaaattc**

**Fortunella_japonica_1** **taataaagagtaccagtggtattggctgttcgtcaattccactccaaagccccaaaattc**

**Fortunella_japonica_2** **taataaagagtaccagtggtattggctgttcgtcaattccactccaaagccccaaaattc**

**Fortunella_japonica_3** **taataaagagtaccagtggtattggctgttcgtcaattccactccaaagccccaaaattc**

**Fortunella_japonica_4** **taataaagagtaccagtggtattggctgttcgtcaattccactccaaagccccaaaattc**

**Fortunella_japonica_5** **taataaagagtaccagtggtattggctgttcgtcaattccactccaaagccccaaaattc**

**Fortunella_japonica_6** **taataaagagtaccagtggtattggctgttcgtcaattccactccaaagccccaaaattc**

**Fortunella_japonica_7** **taataaagagtaccagtggtattggctgttcgtcaattccactccaaagccccaaaattc**

**Fortunella_japonica_8** **taataaagagtaccagtggtattggctgttcgtcaattccactccaaagccccaaaattc**

**Fortunella_japonica_9** **taataaagagtaccagtggtattggctgttcgtcaattccactccaaagccccaaaattc**

**Fortunella_japonica_10** **taataaagagtaccagtggtattggctgttcgtcaattccactccaaagccccaaaattc**

**Fortunella_japonica_11** **taataaagagtaccagtggtattggctgttcgtcaattccactccaaagccccaaaattc**

**Fortunella_japonica_12** **taataaagagtaccagtggtattggctgttcgtcaattccactccaaagccccaaaattc**

**Fortunella_margarita_1** **taataaagagtaccagtggtattggctgttcgtcaattccactccaaagccccaaaattc**

**Fortunella_margarita_2** **taataaagagtaccagtggtattggctgttcgtcaattccactccaaagccccaaaattc**

**Fortunella_venosa_1** **taataaagagtaccagtggtattggctgttcgtcaattccactccaaagccccaaaattc**

**Fortunella_venosa_2** **taataaagagtaccagtggtattggctgttcgtcaattccactccaaagccccaaaattc**

**Fortunella_venosa_3** **taataaagagtaccagtggtattggctgttcgtcaattccactccaaagccccaaaattc**

7930	7940	7950	7960	7970	7980

....|....|....|....|....|....|....|....|....|....|....|....|

**Atalantia_kwangtungens** **gattctgattttagtgttaggattttgacacgtgtaggtgtaaaatataacttaatttat**

**Citrus_reticulata_1** **gattctgattttagtgttaggattttgacacatgtaggtgtaaaatacaacttaatttat**

**Citrus_reticulata_2** **gattctgattttagtgttaggattttgacacatgtaggtgtaaaatacaacttaatttat**

**Citrus_trifoliata_3** **gattctgattttagtgttaggattttgacacatgtaggtgtaaaatacaacttaatttat**

**Citrus_aurantiifolia** **gattctgattttagtgttaggattttgacacatgtaggtgtaaaatacaacttaatttat**

**Citrus_aurantium** **gattctgattttagtgttaggattttgacacatataggtgtaaaatacaacttaatttat**

**Citrus_cavaleriei** **gattctgattttagtgttaggattttgacacatgtaggtgtaaaatacaacttaatttat**

**Citrus_hongheensis** **g-ttctgattttagtgttaggattttgacacatataggtgtaaaatacaacttaatttat**

**Citrus_limon** **gattctgattttagtgttaggattttgacacatataggtgtaaaatacaacttaatttat**

**Citrus_maxima** **gattctgattttagtgttaggattttgacacatataggtgtaaaatacaacttaatttat**

**Citrus_medica** **gattctgattttagtgttaggattttgacacatgtaggtgtaaaatacaacttaatttat**

**Citrus_polytrifolia** **gattctgattttagtgttaggattttgacacatgtaggtgtaaaatacaacttaatttat**

**Citrus_reticulata** **gattctgattttagtgttaggattttgacacatgtaggtgtaaaatacaacttaatttat**

**Citrus_sinensis** **gattctgattttagtgttaggattttgacacatataggtgtaaaatacaacttaatttat**

**Fortunella_bawangica_1** **gattctgattttagtgttaggattttgacacatgtaggtgtaaaatacaacttaatttat**

**Fortunella_bawangica_2** **gattctgattttagtgttaggattttgacacatgtaggtgtaaaatacaacttaatttat**

**Fortunella_hindsii_1** **gattctgattttagtgttaggattttgacacatgtaggtgtaaaatacaacttaatttat**

**Fortunella_hindsii_2** **gattctgattttagtgttaggattttgacacatgtaggtgtaaaatacaacttaatttat**

**Fortunella_hindsii_3** **gattctgattttagtgttaggattttgacacatgtaggtgtaaaatacaacttaatttat**

**Fortunella_japonica_1** **gattctgattttagtgttaggattttgacacatgtaggtgtaaaatacaacttaatttat**

**Fortunella_japonica_2** **gattctgattttagtgttaggattttgacacatgtaggtgtaaaatacaacttaatttat**

**Fortunella_japonica_3** **gattctgattttagtgttaggattttgacacatgtaggtgtaaaatacaacttaatttat**

**Fortunella_japonica_4** **gattctgattttagtgttaggattttgacacatgtaggtgtaaaatacaacttaatttat**

**Fortunella_japonica_5** **gattctgattttagtgttaggattttgacacatgtaggtgtaaaatacaacttaatttat**

**Fortunella_japonica_6** **gattctgattttagtgttaggattttgacacatgtaggtgtaaaatacaacttaatttat**

**Fortunella_japonica_7** **gattctgattttagtgttaggattttgacacatgtaggtgtaaaatacaacttaatttat**

**Fortunella_japonica_8** **gattctgattttagtgttaggattttgacacatgtaggtgtaaaatacaacttaatttat**

**Fortunella_japonica_9** **gattctgattttagtgttaggattttgacacatgtaggtgtaaaatacaacttaatttat**

**Fortunella_japonica_10** **gattctgattttagtgttaggattttgacacatgtaggtgtaaaatacaacttaatttat**

**Fortunella_japonica_11** **gattctgattttagtgttaggattttgacacatgtaggtgtaaaatacaacttaatttat**

**Fortunella_japonica_12** **gattctgattttagtgttaggattttgacacatgtaggtgtaaaatacaacttaatttat**

**Fortunella_margarita_1** **gattctgattttagtgttaggattttgacacatgtaggtgtaaaatacaacttaatttat**

**Fortunella_margarita_2** **gattctgattttagtgttaggattttgacacatgtaggtgtaaaatacaacttaatttat**

**Fortunella_venosa_1** **gattctgattttagtgttaggattttgacacatgtaggtgtaaaatacaacttaatttat**

**Fortunella_venosa_2** **gattctgattttagtgttaggattttgacacatgtaggtgtaaaatacaacttaatttat**

**Fortunella_venosa_3** **gattctgattttagtgttaggattttgacacatgtaggtgtaaaatacaacttaatttat**

7990	8000	8010	8020	8030	8040

....|....|....|....|....|....|....|....|....|....|....|....|

**Atalantia_kwangtungens** **tgatcattacatagaattcaattaagatattgtatgaaagtatgatttcttcaattctca**

**Citrus_reticulata_1** **tgatcattacatagaattcaattaagatattgtatgaaagtatgatttcttcaattctca**

**Citrus_reticulata_2** **tgatcattacatagaattcaattaagatattgtatgaaagtatgatttcttcaattctca**

**Citrus_trifoliata_3** **tgatcattacatagaattcaattaagatattgtatgaaagtatgatttcttcaattctta**

**Citrus_aurantiifolia** **tgatcattacatagaattcaattaagatattgtatgaaagtatgatttcttcaattctca**

**Citrus_aurantium** **tgatcattacatagaattcaattaagatattgtatgaaagtatgatttcttcaattctca**

**Citrus_cavaleriei** **tgatcattacatagaattcaattaagatattgtatgaaagtatgatttcttcaattctca**

**Citrus_hongheensis** **tgatcattacatagaattcaattaagatattgtatgaaagtatgatttcttcaattctca**

**Citrus_limon** **tgatcattacatagaattcaattaagatattgtatgaaagtatgatttcttcaattctca**

**Citrus_maxima** **tgatcattacatagaattcaattaagatattgtatgaaagtatgatttcttcaattctca**

**Citrus_medica** **tgatcattacatagaattcaattaagatattgtatgaaagtatgatttcttcaattctca**

**Citrus_polytrifolia** **tgatcattacatagaattcaattaagatattgtatgaaagtatgatttcttcaattctta**

**Citrus_reticulata** **tgatcattacatagaattcaattaagatattgtatgaaagtatgatttcttcaattctca**

**Citrus_sinensis** **tgatcattacatagaattcaattaagatattgtatgaaagtatgatttcttcaattctca**

**Fortunella_bawangica_1** **tgatcattacatagaattcaattaagatattgtatgaaagtatgatttcttcaattctca**

**Fortunella_bawangica_2** **tgatcattacatagaattcaattaagatattgtatgaaagtatgatttcttcaattctca**

**Fortunella_hindsii_1** **tgatcattacatagaattcaattaagatattgtatgaaagtatgatttcttcaattctca**

**Fortunella_hindsii_2** **tgatcattacatagaattcaattaagatattgtatgaaagtatgatttcttcaattctca**

**Fortunella_hindsii_3** **tgatcattacatagaattcaattaagatattgtatgaaagtatgatttcttcaattctca**

**Fortunella_japonica_1** **tgatcattacatagaattcaattaagatattgtatgaaagtatgatttcttcaattctca**

**Fortunella_japonica_2** **tgatcattacatagaattcaattaagatattgtatgaaagtatgatttcttcaattctca**

**Fortunella_japonica_3** **tgatcattacatagaattcaattaagatattgtatgaaagtatgatttcttcaattctca**

**Fortunella_japonica_4** **tgatcattacatagaattcaattaagatattgtatgaaagtatgatttcttcaattctca**

**Fortunella_japonica_5** **tgatcattacatagaattcaattaagatattgtatgaaagtatgatttcttcaattctca**

**Fortunella_japonica_6** **tgatcattacatagaattcaattaagatattgtatgaaagtatgatttcttcaattctca**

**Fortunella_japonica_7** **tgatcattacatagaattcaattaagatattgtatgaaagtatgatttcttcaattctca**

**Fortunella_japonica_8** **tgatcattacatagaattcaattaagatattgtatgaaagtatgatttcttcaattctca**

**Fortunella_japonica_9** **tgatcattacatagaattcaattaagatattgtatgaaagtatgatttcttcaattctca**

**Fortunella_japonica_10** **tgatcattacatagaattcaattaagatattgtatgaaagtatgatttcttcaattctca**

**Fortunella_japonica_11** **tgatcattacatagaattcaattaagatattgtatgaaagtatgatttcttcaattctca**

**Fortunella_japonica_12** **tgatcattacatagaattcaattaagatattgtatgaaagtatgatttcttcaattctca**

**Fortunella_margarita_1** **tgatcattacatagaattcaattaagatattgtatgaaagtatgatttcttcaattctca**

**Fortunella_margarita_2** **tgatcattacatagaattcaattaagatattgtatgaaagtatgatttcttcaattctca**

**Fortunella_venosa_1** **tgatcattacatagaattcaattaagatattgtatgaaagtatgatttcttcaattctca**

**Fortunella_venosa_2** **tgatcattacatagaattcaattaagatattgtatgaaagtatgatttcttcaattctca**

**Fortunella_venosa_3** **tgatcattacatagaattcaattaagatattgtatgaaagtatgatttcttcaattctca**

8050	8060	8070	8080	8090	8100

....|....|....|....|....|....|....|....|....|....|....|....|

**Atalantia_kwangtungens** **cccgagaatagaagtatttttgttttgattgggtcggttccaagaa-gttttttttgtct**

**Citrus_reticulata_1** **cacgagaatagaaggatttttgttttgattgggtcggttccaagaa-gttttttttgtct**

**Citrus_reticulata_2** **cacgagaatagaaggatttttgttttgattgggtcggttccaagaa-gttttttttgtct**

**Citrus_trifoliata_3** **cacgagaatagaaggatttttgttttgattgggtcggttccaagaattttttttttttct**

**Citrus_aurantiifolia** **cacgagaatagaaggatttttgttttgattgggtcggttccaagaa-gttttttttgtct**

**Citrus_aurantium** **cacgagaatagaaggatttttgttttgattgggtcggttccaagaa-gttttttttgtct**

**Citrus_cavaleriei** **cacgagaatagaaggatttttgttttgattgggtcggttccaagaa-gttttttttgtct**

**Citrus_hongheensis** **cacgagaatagaaggatttttgttttgattgggtcggttccaagaa-gttttttttgtct**

**Citrus_limon** **cacgagaatagaaggatttttgttttgattgggtcggttccaagaa-gttttttttgtct**

**Citrus_maxima** **cacgagaatagaaggatttttgttttgattgggtcggttccaagaa-gttttttttgtct**

**Citrus_medica** **cacgagaatagaaggatttttgttttgattgggtcggttccaagaa-gttttttttgtct**

**Citrus_polytrifolia** **cacgagaatagaaggatttttgttttgattgggtcggttccaagaa--ttttttttttct**

**Citrus_reticulata** **cacgagaatagaaggatttttgttttgattgggtcggttccaagaa-gttttttttgtct**

**Citrus_sinensis** **cacgagaatagaaggatttttgttttgattgggtcggttccaagaa-gttttttttgtct**

**Fortunella_bawangica_1** **cacgagaatagaaggatttttgttttgattgggtcggttccaagaa-gttttttttgtct**

**Fortunella_bawangica_2** **cacgagaatagaaggatttttgttttgattgggtcggttccaagaa-gttttttttgtct**

**Fortunella_hindsii_1** **cacgagaatagaaggatttttgttttgattgggtcggttccaagaa-gttttttttgtct**

**Fortunella_hindsii_2** **cacgagaatagaaggatttttgttttgattgggtcggttccaagaa-gttttttttgtct**

**Fortunella_hindsii_3** **cacgagaatagaaggatttttgttttgattgggtcggttccaagaa-gttttttttgtct**

**Fortunella_japonica_1** **cacgagaatagaaggatttttgttttgattgggtcggttccaagaa-gttttttttgtct**

**Fortunella_japonica_2** **cacgagaatagaaggatttttgttttgattgggtcggttccaagaa-gttttttttgtct**

**Fortunella_japonica_3** **cacgagaatagaaggatttttgttttgattgggtcggttccaagaa-gttttttttgtct**

**Fortunella_japonica_4** **cacgagaatagaaggatttttgttttgattgggtcggttccaagaa-gttttttttgtct**

**Fortunella_japonica_5** **cacgagaatagaaggatttttgttttgattgggtcggttccaagaa-gttttttttgtct**

**Fortunella_japonica_6** **cacgagaatagaaggatttttgttttgattgggtcggttccaagaa-gttttttttgtct**

**Fortunella_japonica_7** **cacgagaatagaaggatttttgttttgattgggtcggttccaagaa-gttttttttgtct**

**Fortunella_japonica_8** **cacgagaatagaaggatttttgttttgattgggtcggttccaagaa-gttttttttgtct**

**Fortunella_japonica_9** **cacgagaatagaaggatttttgttttgattgggtcggttccaagaa-gttttttttgtct**

**Fortunella_japonica_10** **cacgagaatagaaggatttttgttttgattgggtcggttccaagaa-gttttttttgtct**

**Fortunella_japonica_11** **cacgagaatagaaggatttttgttttgattgggtcggttccaagaa-gttttttttgtct**

**Fortunella_japonica_12** **cacgagaatagaaggatttttgttttgattgggtcggttccaagaa-gttttttttgtct**

**Fortunella_margarita_1** **cacgagaatagaaggatttttgttttgattgggtcggttccaagaa-gttttttttgtct**

**Fortunella_margarita_2** **cacgagaatagaaggatttttgttttgattgggtcggttccaagaa-gttttttttgtct**

**Fortunella_venosa_1** **cacgagaatagaaggatttttgttttgattgggtcggttccaagaa-attttttttgtct**

**Fortunella_venosa_2** **cacgagaatagaaggatttttgttttgattgggtcggttccaagaa-gttttttttgtct**

**Fortunella_venosa_3** **cacgagaatagaaggatttttgttttgattgggtcggttccaagaa-gttttttttgtct**

8110	8120	8130	8140	8150	8160

....|....|....|....|....|....|....|....|....|....|....|....|

**Atalantia_kwangtungens** **accttactttcttttcttcatttttatcttatatcaataagatattaataactcaatcaa**

**Citrus_reticulata_1** **accttactttcttttcttcatttttatcttatatcaataagatattaataactcaatcaa**

**Citrus_reticulata_2** **accttactttcttttcttcatttttatcttatatcaataagatattaataactcaatcaa**

**Citrus_trifoliata_3** **accttactttcttttcttcatttttatcttatatcaataagatattaataactcaatcaa**

**Citrus_aurantiifolia** **accttactttcttttcttcatttttatcttatatcaataagatattaataactcaatcaa**

**Citrus_aurantium** **accttactttcttttcttcatttttatcttatatcaataagatattaataactcaatcaa**

**Citrus_cavaleriei** **accttactttgttttcttcatttttatcttatatcaataagatattaataactcaatcaa**

**Citrus_hongheensis** **accttactttcttttcttcatttttatcttatatcaataagatattaataactcaatcaa**

**Citrus_limon** **accttactttcttttcttcatttttatcttatatcaataagatattaataactcaatcaa**

**Citrus_maxima** **accttactttcttttcttcatttttatcttatatcaataagatattaataactcaatcaa**

**Citrus_medica** **accttactgtcttttcttcatttttatcttatatcaataagatattaataactcaatcaa**

**Citrus_polytrifolia** **accttactttcttttcttcatttttatcttatatcaataagatattaataactcaatcaa**

**Citrus_reticulata** **accttactttcttttcttcatttttatcttatatcaataagatattaataactcaatcaa**

**Citrus_sinensis** **accttactttcttttcttcatttttatcttatatcaataagatattaataactcaatcaa**

**Fortunella_bawangica_1** **accttactttcttttcttcatttttatcttatatcaataagatattaataactcaatcaa**

**Fortunella_bawangica_2** **accttactttcttttcttcatttttatcttatatcaataagatattaataactcaatcaa**

**Fortunella_hindsii_1** **accttactttcttttcttcatttttatcttatatcaataagatattaataactcaatcaa**

**Fortunella_hindsii_2** **accttactttcttttcttcatttttatcttatatcaataagatattaataactcaatcaa**

**Fortunella_hindsii_3** **accttactttcttttcttcatttttatcttatatcaataagatattaataactcaatcaa**

**Fortunella_japonica_1** **accttactttcttttcttcatttttatcttatatcaataagatattaataactcaatcaa**

**Fortunella_japonica_2** **accttactttcttttcttcatttttatcttatatcaataagatattaataactcaatcaa**

**Fortunella_japonica_3** **accttactttcttttcttcatttttatcttatatcaataagatattaataactcaatcaa**

**Fortunella_japonica_4** **accttactttcttttcttcatttttatcttatatcaataagatattaataactcaatcaa**

**Fortunella_japonica_5** **accttactttcttttcttcatttttatcttatatcaataagatattaataactcaatcaa**

**Fortunella_japonica_6** **accttactttcttttcttcatttttatcttatatcaataagatattaataactcaatcaa**

**Fortunella_japonica_7** **accttactttcttttcttcatttttatcttatatcaataagatattaataactcaatcaa**

**Fortunella_japonica_8** **accttactttcttttcttcatttttatcttatatcaataagatattaataactcaatcaa**

**Fortunella_japonica_9** **accttactttcttttcttcatttttatcttatatcaataagatattaataactcaatcaa**

**Fortunella_japonica_10** **accttactttcttttcttcatttttatcttatatcaataagatattaataactcaatcaa**

**Fortunella_japonica_11** **accttactttcttttcttcatttttatcttatatcaataagatattaataactcaatcaa**

**Fortunella_japonica_12** **accttactttcttttcttcatttttatcttatatcaataagatattaataactcaatcaa**

**Fortunella_margarita_1** **accttactttcttttcttcatttttatcttatatcaataagatattaataactcaatcaa**

**Fortunella_margarita_2** **accttactttcttttcttcatttttatcttatatcaataagatattaataactcaatcaa**

**Fortunella_venosa_1** **accttactttcttttcttcatttttatcttatatcaataagatattaataactcaatcaa**

**Fortunella_venosa_2** **accttactttcttttcttcatttttatcttatatcaataagatattaataactcaatcaa**

**Fortunella_venosa_3** **accttactttcttttcttcatttttatcttatatcaataagatattaataactcaatcaa**

8170	8180	8190	8200	8210	8220

....|....|....|....|....|....|....|....|....|....|....|....|

**Atalantia_kwangtungens** **aataaaattatctccaagaacaaaatgtttgttatgcttaatatcttaagtttaatgtat**

**Citrus_reticulata_1** **aataaaattatctccaagaacaaaatgtttgttatgcttaatatctttagtttaatgtat**

**Citrus_reticulata_2** **aataaaattatctccaagaacaaaatgtttgttatgcttaatatctttagtttaatgtat**

**Citrus_trifoliata_3** **aataaaattatctccaagaacaaaatgtttgttatgcttaatatctttagtttaatgtat**

**Citrus_aurantiifolia** **aataaaattatctccaagaacaaaatgtttgttatgcttaatatctttagtttaatgtat**

**Citrus_aurantium** **aataaaattatctccaagaacaaaatgtttgttatgcttaatatctttagtttaatgtat**

**Citrus_cavaleriei** **aataaaattatctccaagaacaaaatgtttgttatgcttaatatctttagtttaatgtat**

**Citrus_hongheensis** **aataaaattatctccaagaacaaaatgtttgttatgcttaatatctttagtttaatgtat**

**Citrus_limon** **aataaaattatctccaagaacaaaatgtttgttatgcttaatatctttagtttaatgtat**

**Citrus_maxima** **aataaaattatctccaagaacaaaatgtttgttatgcttaatatctttagtttaatgtat**

**Citrus_medica** **aataaaattatctccaagaacaaaatgtttgttatgcttaatatctttagtttaatgtat**

**Citrus_polytrifolia** **aataaaattatctccaagaacaaaatgtttgttatgcttaatatctttagtttaatgtat**

**Citrus_reticulata** **aataaaattatctccaagaacaaaatgtttgttatgcttaatatctttagtttaatgtat**

**Citrus_sinensis** **aataaaattatctccaagaacaaaatgtttgttatgcttaatatctttagtttaatgtat**

**Fortunella_bawangica_1** **aataaaattatctccaagaacaaaatgtttgttatgcttaatatctttagtttaatgtat**

**Fortunella_bawangica_2** **aataaaattatctccaagaacaaaatgtttgttatgcttaatatctttagtttaatgtat**

**Fortunella_hindsii_1** **aataaaattatctccaagaacaaaatgtttgttatgcttaatatctttagtttaatgtat**

**Fortunella_hindsii_2** **aataaaattatctccaagaacaaaatgtttgttatgcttaatatctttagtttaatgtat**

**Fortunella_hindsii_3** **aataaaattatctccaagaacaaaatgtttgttatgcttaatatctttagtttaatgtat**

**Fortunella_japonica_1** **aataaaattatctccaagaacaaaatgtttgttatgcttaatatctttagtttaatgtat**

**Fortunella_japonica_2** **aataaaattatctccaagaacaaaatgtttgttatgcttaatatctttagtttaatgtat**

**Fortunella_japonica_3** **aataaaattatctccaagaacaaaatgtttgttatgcttaatatctttagtttaatgtat**

**Fortunella_japonica_4** **aataaaattatctccaagaacaaaatgtttgttatgcttaatatctttagtttaatgtat**

**Fortunella_japonica_5** **aataaaattatctccaagaacaaaatgtttgttatgcttaatatctttagtttaatgtat**

**Fortunella_japonica_6** **aataaaattatctccaagaacaaaatgtttgttatgcttaatatctttagtttaatgtat**

**Fortunella_japonica_7** **aataaaattatctccaagaacaaaatgtttgttatgcttaatatctttagtttaatgtat**

**Fortunella_japonica_8** **aataaaattatctccaagaacaaaatgtttgttatgcttaatatctttagtttaatgtat**

**Fortunella_japonica_9** **aataaaattatctccaagaacaaaatgtttgttatgcttaatatctttagtttaatgtat**

**Fortunella_japonica_10** **aataaaattatctccaagaacaaaatgtttgttatgcttaatatctttagtttaatgtat**

**Fortunella_japonica_11** **aataaaattatctccaagaacaaaatgtttgttatgcttaatatctttagtttaatgtat**

**Fortunella_japonica_12** **aataaaattatctccaagaacaaaatgtttgttatgcttaatatctttagtttaatgtat**

**Fortunella_margarita_1** **aataaaattatctccaagaacaaaatgtttgttatgcttaatatctttagtttaatgtat**

**Fortunella_margarita_2** **aataaaattatctccaagaacaaaatgtttgttatgcttaatatctttagtttaatgtat**

**Fortunella_venosa_1** **aataaaattatctccaagaacaaaatgtttgttatgcttaatatctttagtttaatgtat**

**Fortunella_venosa_2** **aataaaattatctccaagaacaaaatgtttgttatgcttaatatctttagtttaatgtat**

**Fortunella_venosa_3** **aataaaattatctccaagaacaaaatgtttgttatgcttaatatctttagtttaatgtat**

8230	8240	8250	8260	8270	8280

....|....|....|....|....|....|....|....|....|....|....|....|

**Atalantia_kwangtungens** **atctgtcttaattctgccctttattcgagtagttttttattcgctaaattacccgaggcc**

**Citrus_reticulata_1** **atctgtcttaattctgccctttattcgagtagttttttattcgctaaattacccgaggcc**

**Citrus_reticulata_2** **atctgtcttaattctgccctttattcgagtagttttttattcgctaaattacccgaggcc**

**Citrus_trifoliata_3** **atctgtcttaattctgccctttattcgagtagttttttattcgctaaattacccgaggcc**

**Citrus_aurantiifolia** **atctgtcttaattctgccctttattcgagtagttttttattcgctaaattacccgaggcc**

**Citrus_aurantium** **atctgtcttaattctgccctttattcgagtagttttttattcgctaaattacccgaggcc**

**Citrus_cavaleriei** **atctgtcttaattctgccctttattcgagtagttttttattcgctaaattacccgaggcc**

**Citrus_hongheensis** **atctgtcttaattctgccctttattcgagtagttttttattcgctaaattacccgaggcc**

**Citrus_limon** **atctgtcttaattctgccctttattcgagtagttttttattcgctaaattacccgaggcc**

**Citrus_maxima** **atctgtcttaattctgccctttattcgagtagttttttattcgctaaattacccgaggcc**

**Citrus_medica** **atctgtcttaattctgccctttattcgagtagttttttattcgctaaattacccgaggcc**

**Citrus_polytrifolia** **atctgtcttaattctgccctttattcgagtagttttttattcgctaaattacccgaggcc**

**Citrus_reticulata** **atctgtcttaattctgccctttattcgagtagttttttattcgctaaattacccgaggcc**

**Citrus_sinensis** **atctgtcttaattctgccctttattcgagtagttttttattcgctaaattacccgaggcc**

**Fortunella_bawangica_1** **atctgtcttaattctgccctttattcgagtagttttttattcgctaaattacccgaggcc**

**Fortunella_bawangica_2** **atctgtcttaattctgccctttattcgagtagttttttattcgctaaattacccgaggcc**

**Fortunella_hindsii_1** **atctgtcttaattctgccctttattcgagtagttttttattcgctaaattacccgaggcc**

**Fortunella_hindsii_2** **atctgtcttaattctgccctttattcgagtagttttttattcgctaaattacccgaggcc**

**Fortunella_hindsii_3** **atctgtcttaattctgccctttattcgagtagttttttattcgctaaattacccgaggcc**

**Fortunella_japonica_1** **atctgtcttaattctgccctttattcgagtagttttttattcgctaaattacccgaggcc**

**Fortunella_japonica_2** **atctgtcttaattctgccctttattcgagtagttttttattcgctaaattacccgaggcc**

**Fortunella_japonica_3** **atctgtcttaattctgccctttattcgagtagttttttattcgctaaattacccgaggcc**

**Fortunella_japonica_4** **atctgtcttaattctgccctttattcgagtagttttttattcgctaaattacccgaggcc**

**Fortunella_japonica_5** **atctgtcttaattctgccctttattcgagtagttttttattcgctaaattacccgaggcc**

**Fortunella_japonica_6** **atctgtcttaattctgccctttattcgagtagttttttattcgctaaattacccgaggcc**

**Fortunella_japonica_7** **atctgtcttaattctgccctttattcgagtagttttttattcgctaaattacccgaggcc**

**Fortunella_japonica_8** **atctgtcttaattctgccctttattcgagtagttttttattcgctaaattacccgaggcc**

**Fortunella_japonica_9** **atctgtcttaattctgccctttattcgagtagttttttattcgctaaattacccgaggcc**

**Fortunella_japonica_10** **atctgtcttaattctgccctttattcgagtagttttttattcgctaaattacccgaggcc**

**Fortunella_japonica_11** **atctgtcttaattctgccctttattcgagtagttttttattcgctaaattacccgaggcc**

**Fortunella_japonica_12** **atctgtcttaattctgccctttattcgagtagttttttattcgctaaattacccgaggcc**

**Fortunella_margarita_1** **atctgtcttaattctgccctttattcgagtagttttttattcgctaaattacccgaggcc**

**Fortunella_margarita_2** **atctgtcttaattctgccctttattcgagtagttttttattcgctaaattacccgaggcc**

**Fortunella_venosa_1** **atctgtcttaattctgccctttattcgagtagttttttattcgctaaattacccgaggcc**

**Fortunella_venosa_2** **atctgtcttaattctgccctttattcgagtagttttttattcgctaaattacccgaggcc**

**Fortunella_venosa_3** **atctgtcttaattctgccctttattcgagtagttttttattcgctaaattacccgaggcc**

8290	8300	8310	8320	8330	8340

....|....|....|....|....|....|....|....|....|....|....|....|

**Atalantia_kwangtungens** **tacgcttttttgaatccaattgtagatgttatgccagtaatacctgttctattttttctc**

**Citrus_reticulata_1** **tacgcttttttgaatccaattgtagatgttatgccagtaatacctgttctattttttctc**

**Citrus_reticulata_2** **tacgcttttttgaatccaattgtagatgttatgccagtaatacctgttctattttttctc**

**Citrus_trifoliata_3** **tacgcttttttgaatccaattgtagatgttatgccagtaatacctcttctattttttctc**

**Citrus_aurantiifolia** **tacgcttttttgaatccaattgtagatgttatgccagtaatacctgttctattttttctc**

**Citrus_aurantium** **tacgcttttttgaatccaattgtagatgttatgccagtaatacctgttctattttttctc**

**Citrus_cavaleriei** **tacgcttttttgaatccaattgtagatgttatgccagtaatacctgttctattttttctc**

**Citrus_hongheensis** **tacgcttttttgaatccaattgtagatgttatgccagtaatacctgttctattttttctc**

**Citrus_limon** **tacgcttttttgaatccaattgtagatgttatgccagtaatacctgttctattttttctc**

**Citrus_maxima** **tacgcttttttgaatccaattgtagatattatgccagtaatacctgttctattttttctc**

**Citrus_medica** **tacgcttttttgaatccaattgtagatgttatgccagtaatacctgttctattttttctc**

**Citrus_polytrifolia** **tacgcttttttgaatccaattgtagatgttatgccagtaatacctcttctattttttctc**

**Citrus_reticulata** **tacgcttttttgaatccaattgtagatgttatgccagtaatacctgttctattttttctc**

**Citrus_sinensis** **tacgcttttttgaatccaattgtagatgttatgccagtaatacctgttctattttttctc**

**Fortunella_bawangica_1** **tacgcttttttgaatccaattgtagatgttatgccagtaatacctgttctattttttctc**

**Fortunella_bawangica_2** **tacgcttttttgaatccaattgtagatgttatgccagtaatacctgttctattttttctc**

**Fortunella_hindsii_1** **tacgcttttttgaatccaattgtagatgttatgccagtaatacctgttctattttttctc**

**Fortunella_hindsii_2** **tacgcttttttgaatccaattgtagatgttatgccagtaatacctgttctattttttctc**

**Fortunella_hindsii_3** **tacgcttttttgaatccaattgtagatgttatgccagtaatacctgttctattttttctc**

**Fortunella_japonica_1** **tacgcttttttgaatccaattgtagatgttatgccagtaatacctgttctattttttctc**

**Fortunella_japonica_2** **tacgcttttttgaatccaattgtagatgttatgccagtaatacctgttctattttttctc**

**Fortunella_japonica_3** **tacgcttttttgaatccaattgtagatgttatgccagtaatacctgttctattttttctc**

**Fortunella_japonica_4** **tacgcttttttgaatccaattgtagatgttatgccagtaatacctgttctattttttctc**

**Fortunella_japonica_5** **tacgcttttttgaatccaattgtagatgttatgccagtaatacctgttctattttttctc**

**Fortunella_japonica_6** **tacgcttttttgaatccaattgtagatgttatgccagtaatacctgttctattttttctc**

**Fortunella_japonica_7** **tacgcttttttgaatccaattgtagatgttatgccagtaatacctgttctattttttctc**

**Fortunella_japonica_8** **tacgcttttttgaatccaattgtagatgttatgccagtaatacctgttctattttttctc**

**Fortunella_japonica_9** **tacgcttttttgaatccaattgtagatgttatgccagtaatacctgttctattttttctc**

**Fortunella_japonica_10** **tacgcttttttgaatccaattgtagatgttatgccagtaatacctgttctattttttctc**

**Fortunella_japonica_11** **tacgcttttttgaatccaattgtagatgttatgccagtaatacctgttctattttttctc**

**Fortunella_japonica_12** **tacgcttttttgaatccaattgtagatgttatgccagtaatacctgttctattttttctc**

**Fortunella_margarita_1** **tacgcttttttgaatccaattgtagatgttatgccagtaatacctgttctattttttctc**

**Fortunella_margarita_2** **tacgcttttttgaatccaattgtagatgttatgccagtaatacctgttctattttttctc**

**Fortunella_venosa_1** **tacgcttttttgaatccaattgtagatgttatgccagtaatacctgttctattttttctc**

**Fortunella_venosa_2** **tacgcttttttgaatccaattgtagatgttatgccagtaatacctgttctattttttctc**

**Fortunella_venosa_3** **tacgcttttttgaatccaattgtagatgttatgccagtaatacctgttctattttttctc**

8350	8360	8370	8380	8390	8400

....|....|....|....|....|....|....|....|....|....|....|....|

**Atalantia_kwangtungens** **ttagcctttgtttggcaagctgctgtaagttttcgatgagatctttaatattgtgctaga**

**Citrus_reticulata_1** **ttagcctttgtttggcaagctgctgtaagttttcgataagatctttaatattgtgctaga**

**Citrus_reticulata_2** **ttagcctttgtttggcaagctgctgtaagttttcgataagatctttaatattgtgctaga**

**Citrus_trifoliata_3** **ttagcctttgtttggcaagctgctgtaagttttcgataagatctttaatattgtgctaga**

**Citrus_aurantiifolia** **ttagcctttgtttggcaagctgctgtaagttttcgataagatctttaatattgtgctaga**

**Citrus_aurantium** **ttagcctttgtttggcaagctgctgtaagttttcgataagatctttaatattgtgctaga**

**Citrus_cavaleriei** **ttagcctttgtttggcaagctgctgtaagttttcgataagatctttaatattgtgctaga**

**Citrus_hongheensis** **ttagcctttgtttggcaagctgctgtaagttttcgataagatctttaatattgtgctaga**

**Citrus_limon** **ttagcctttgtttggcaagctgctgtaagttttcgataagatctttaatattgtgctaga**

**Citrus_maxima** **ttagcctttgtttggcaagctgctgtaagttttcgataagatctttaatattgtgctaga**

**Citrus_medica** **ttagcctttgtttggcaagctgctgtaagttttcgataagatctttaatattgtgctaga**

**Citrus_polytrifolia** **ttagcctttgtttggcaagctgctgtaagttttcgataagatctttaatattgtgctaga**

**Citrus_reticulata** **ttagcctttgtttggcaagctgctgtaagttttcgataagatctttaatattgtgctaga**

**Citrus_sinensis** **ttagcctttgtttggcaagctgctgtaagttttcgataagatctttaatattgtgctaga**

**Fortunella_bawangica_1** **ttagcctttgtttggcaagctgctgtaagttttcgataagatctttaatattgtgctaga**

**Fortunella_bawangica_2** **ttagcctttgtttggcaagctgctgtaagttttcgataagatctttaatattgtgctaga**

**Fortunella_hindsii_1** **ttagcctttgtttggcaagctgctgtaagttttcgataagatctttaatattgtgctaga**

**Fortunella_hindsii_2** **ttagcctttgtttggcaagctgctgtaagttttcgataagatctttaatattgtgctaga**

**Fortunella_hindsii_3** **ttagcctttgtttggcaagctgctgtaagttttcgataagatctttaatattgtgctaga**

**Fortunella_japonica_1** **ttagcctttgtttggcaagctgctgtaagttttcgataagatctttaatattgtgctaga**

**Fortunella_japonica_2** **ttagcctttgtttggcaagctgctgtaagttttcgataagatctttaatattgtgctaga**

**Fortunella_japonica_3** **ttagcctttgtttggcaagctgctgtaagttttcgataagatctttaatattgtgctaga**

**Fortunella_japonica_4** **ttagcctttgtttggcaagctgctgtaagttttcgataagatctttaatattgtgctaga**

**Fortunella_japonica_5** **ttagcctttgtttggcaagctgctgtaagttttcgataagatctttaatattgtgctaga**

**Fortunella_japonica_6** **ttagcctttgtttggcaagctgctgtaagttttcgataagatctttaatattgtgctaga**

**Fortunella_japonica_7** **ttagcctttgtttggcaagctgctgtaagttttcgataagatctttaatattgtgctaga**

**Fortunella_japonica_8** **ttagcctttgtttggcaagctgctgtaagttttcgataagatctttaatattgtgctaga**

**Fortunella_japonica_9** **ttagcctttgtttggcaagctgctgtaagttttcgataagatctttaatattgtgctaga**

**Fortunella_japonica_10** **ttagcctttgtttggcaagctgctgtaagttttcgataagatctttaatattgtgctaga**

**Fortunella_japonica_11** **ttagcctttgtttggcaagctgctgtaagttttcgataagatctttaatattgtgctaga**

**Fortunella_japonica_12** **ttagcctttgtttggcaagctgctgtaagttttcgataagatctttaatattgtgctaga**

**Fortunella_margarita_1** **ttagcctttgtttggcaagctgctgtaagttttcgataagatctttaatattgtgctaga**

**Fortunella_margarita_2** **ttagcctttgtttggcaagctgctgtaagttttcgataagatctttaatattgtgctaga**

**Fortunella_venosa_1** **ttagcctttgtttggcaagctgctgtaagttttcgataagatctttaatattgtgctaga**

**Fortunella_venosa_2** **ttagcctttgtttggcaagctgctgtaagttttcgataagatctttaatattgtgctaga**

**Fortunella_venosa_3** **ttagcctttgtttggcaagctgctgtaagttttcgataagatctttaatattgtgctaga**

8410	8420	8430	8440	8450	8460

....|....|....|....|....|....|....|....|....|....|....|....|

**Atalantia_kwangtungens** **aaaattcatgatttattctagttcg-------aaaaaaaaaat----------tctaaca**

**Citrus_reticulata_1** **aaaattcatgatttattctagttcg-------aaaaaaaaaat----------tctaaca**

**Citrus_reticulata_2** **aaaattcatgatttattctagttcg-------aaaaaaaaaat----------tctaaca**

**Citrus_trifoliata_3** **aaaattcatgatttattctagttcg---aaaaaaaaaaaaaat----------tctaaca**

**Citrus_aurantiifolia** **aaaattcatgatttattctagttcgaaaaaaaaaaaaaaaaat----------tctaaca**

**Citrus_aurantium** **aaaattcatgatttattctagttcg---------aaaaaaaat----------tctaaca**

**Citrus_cavaleriei** **aaaattcatgatttattctagttcg------aaaaaaaaaaat----------tctaaca**

**Citrus_hongheensis** **aaaattcatgatttattctagttcg-----aaaaaaaaaaattctaacaattatctaaca**

**Citrus_limon** **aaaattcatgatttattctagttcg---------aaaaaaaat----------tctaaca**

**Citrus_maxima** **aaaattcatgatttattctagttcg-----aaaaaaaaaaaat----------tctaaca**

**Citrus_medica** **aaaattcatgatttattctagttcg-----aaaaaaaaaaattctaacaa---ctaatca**

**Citrus_polytrifolia** **aaaattcatgatttattctagttcg----aaaaaaaaaaaaat----------tctaaca**

**Citrus_reticulata** **aaaattcatgatttattctagttcg-------aaaaaaaaaat----------tctaaca**

**Citrus_sinensis** **aaaattcatgatttattctagttcg-----aaaaaaaaaaaat----------tctaaca**

**Fortunella_bawangica_1** **aaaattcatgatttattctagttcg-----aaaaaaaaaaaat----------tctaaca**

**Fortunella_bawangica_2** **aaaattcatgatttattctagttcg-----aaaaaaaaaaaat----------tctaaca**

**Fortunella_hindsii_1** **aaaattcatgatttattctagttcg----aaaaaaaaaaaaat----------tctaaca**

**Fortunella_hindsii_2** **aaaattcatgatttattctagttcg----aaaaaaaaaaaaat----------tctaaca**

**Fortunella_hindsii_3** **aaaattcatgatttattctagttcg----aaaaaaaaaaaaat----------tctaaca**

**Fortunella_japonica_1** **aaaattcatgatttattctagttcg-----aaaaaaaaaaaat----------tctaaca**

**Fortunella_japonica_2** **aaaattcatgatttattctagttcg------aaaaaaaaaaat----------tctaaca**

**Fortunella_japonica_3** **aaaattcatgatttattctagttcg-----aaaaaaaaaaaat----------tctaaca**

**Fortunella_japonica_4** **aaaattcatgatttattctagttcg-----aaaaaaaaaaaat----------tctaaca**

**Fortunella_japonica_5** **aaaattcatgatttattctagttcg-----aaaaaaaaaaaat----------tctaaca**

**Fortunella_japonica_6** **aaaattcatgatttattctagttcg-----aaaaaaaaaaaat----------tctaaca**

**Fortunella_japonica_7** **aaaattcatgatttattctagttcg-----aaaaaaaaaaaat----------tctaaca**

**Fortunella_japonica_8** **aaaattcatgatttattctagttcg-----aaaaaaaaaaaat----------tctaaca**

**Fortunella_japonica_9** **aaaattcatgatttattctagttcg-----aaaaaaaaaaaat----------tctaaca**

**Fortunella_japonica_10** **aaaattcatgatttattctagttcg-----aaaaaaaaaaaat----------tctaaca**

**Fortunella_japonica_11** **aaaattcatgatttattctagttcg-----aaaaaaaaaaaat----------tctaaca**

**Fortunella_japonica_12** **aaaattcatgatttattctagttcg-----aaaaaaaaaaaat----------tctaaca**

**Fortunella_margarita_1** **aaaattcatgatttattctagttcg-----aaaaaaaaaaaat----------tctaaca**

**Fortunella_margarita_2** **aaaattcatgatttattctagttcg-----aaaaaaaaaaaat----------tctaaca**

**Fortunella_venosa_1** **aaaattcatgatttattctagttcg-----aaaaaaaaaaaat----------tctaaca**

**Fortunella_venosa_2** **aaaattcatgatttattctagttcg-----aaaaaaaaaaaat----------tctaaca**

**Fortunella_venosa_3** **aaaattcatgatttattctagttcg-----aaaaaaaaaaaat----------tctaaca**

8470	8480	8490	8500	8510	8520

....|....|....|....|....|....|....|....|....|....|....|....|

**Atalantia_kwangtungens** **attaataaataagagcagatgagtcttacaatatgaacgaacccccgcctca--------**

**Citrus_reticulata_1** **attaataaataagagcagatgagtcttacaatatgaacgaacccccgcctca--------**

**Citrus_reticulata_2** **attaataaataagagcagatgagtcttacaatatgaacgaacccccgcctca--------**

**Citrus_trifoliata_3** **attaataaataagagcagatgagtcttacaatatgaacgaacccccgcctca--------**

**Citrus_aurantiifolia** **attaataaataagagcagatgagtcttacaatatgaacgaacccccgcctca--------**

**Citrus_aurantium** **attaataaataagagcagatgagtcttacaatatgaacgaacccccgcctcaattcaaac**

**Citrus_cavaleriei** **attaataaataagagcagatgagtcttacaatatgaacgaacccccgcctca--------**

**Citrus_hongheensis** **attaataaataagagcagatgagtcttacaatatgaacgaacccccgcctcaattcaaac**

**Citrus_limon** **attaataaataagagcagatgagtcttacaatatgaacgaacccccgcctcaattcaaac**

**Citrus_maxima** **attaataaataagagcagatgagtcttacaatatgaacgaacccccgcctcaattcaaac**

**Citrus_medica** **attaataaataagagcagatgagtcttacaatatgaacgaacccccgcctcaattcaaaa**

**Citrus_polytrifolia** **attaataaataagagcagatgagtcttacaatatgaacgaacccccgcctca--------**

**Citrus_reticulata** **attaataaataagagcagatgagtcttacaatatgaacgaacccccgcctca--------**

**Citrus_sinensis** **attaataaataagagcagatgagtcttacaatatgaacgaacccccgcctcaattcaaac**

**Fortunella_bawangica_1** **attaataaataagagcagatgagtcttacaatatgaacgaacccccgcctca--------**

**Fortunella_bawangica_2** **attaataaataagagcagatgagtcttacaatatgaacgaacccccgcctca--------**

**Fortunella_hindsii_1** **attaataaataagagcagatgagtcttacaatatgaacgaacccccgcctca--------**

**Fortunella_hindsii_2** **attaataaataagagcagatgagtcttacaatatgaacgaacccccgcctca--------**

**Fortunella_hindsii_3** **attaataaataagagcagatgagtcttacaatatgaacgaacccccgcctca--------**

**Fortunella_japonica_1** **attaataaataagagcagatgagtcttacaatatgaacgaacccccgcctca--------**

**Fortunella_japonica_2** **attaataaataagagcagatgagtcttacaatatgaacgaacccccgcctca--------**

**Fortunella_japonica_3** **attaataaataagagcagatgagtcttacaatatgaacgaacccccgcctca--------**

**Fortunella_japonica_4** **attaataaataagagcagatgagtcttacaatatgaacgaacccccgcctca--------**

**Fortunella_japonica_5** **attaataaataagagcagatgagtcttacaatatgaacgaacccccgcctca--------**

**Fortunella_japonica_6** **attaataaataagagcagatgagtcttacaatatgaacgaacccccgcctca--------**

**Fortunella_japonica_7** **attaataaataagagcagatgagtcttacaatatgaacgaacccccgcctca--------**

**Fortunella_japonica_8** **attaataaataagagcagatgagtcttacaatatgaacgaacccccgcctca--------**

**Fortunella_japonica_9** **attaataaataagagcagatgagtcttacaatatgaacgaacccccgcctca--------**

**Fortunella_japonica_10** **attaataaataagagcagatgagtcttacaatatgaacgaacccccgcctca--------**

**Fortunella_japonica_11** **attaataaataagagcagatgagtcttacaatatgaacgaacccccgcctca--------**

**Fortunella_japonica_12** **attaataaataagagcagatgagtcttacaatatgaacgaacccccgcctca--------**

**Fortunella_margarita_1** **attaataaataagagcagatgagtcttacaatatgaacgaacccccgcctca--------**

**Fortunella_margarita_2** **attaataaataagagcagatgagtcttacaatatgaacgaacccccgcctca--------**

**Fortunella_venosa_1** **attaataaataagagcagatgagtcttacaatatgaacgaacccccgcctca--------**

**Fortunella_venosa_2** **attaataaataagagcagatgagtcttacaatatgaacgaacccccgcctca--------**

**Fortunella_venosa_3** **attaataaataagagcagatgagtcttacaatatgaacgaacccccgcctca--------**

8530	8540	8550	8560	8570	8580

....|....|....|....|....|....|....|....|....|....|....|....|

**Atalantia_kwangtungens** **attcaaacaatgaaattcttggggaaccgcgatccattttgatcccccatttgctatttg**

**Citrus_reticulata_1** **attcaaacaatgaaattcttggggaaccgcgatccattttgatcccccatttgctatttg**

**Citrus_reticulata_2** **attcaaacaatgaaattcttggggaaccgcgatccattttgatcccccatttgctatttg**

**Citrus_trifoliata_3** **attcaaacaatgaaattcttgtggaaccgcgatccattttgatcccccatttgctatttg**

**Citrus_aurantiifolia** **attcaaacaatgaaattcttggggaaccgcgatccattttgatcccccatttgctatttg**

**Citrus_aurantium** **attcaaacaatgaaattcttggggaaccgcgatccattttgatcccccatttgctatttg**

**Citrus_cavaleriei** **attcaaacaatgaaattcttggggaaccgcgatccattttgatcccccatttgctatttg**

**Citrus_hongheensis** **attcaaacaatgaaattcttggggaaccgcgatccattttgatcccccatttgctatttg**

**Citrus_limon** **attcaaacaatgaaattcttggggaaccgcgatccattttgatcccccatttgctatttg**

**Citrus_maxima** **attcaaacaatgaaattcttggggaaccgcgatccattttgatcccccatttgctatttg**

**Citrus_medica** **attcaaacaatgaaattcttggggaaccgcaatccattttgatcccccatttgctatttg**

**Citrus_polytrifolia** **attcaaacaatgaaattcttggggaaccgcgatccattttgatcccccatttgctatttg**

**Citrus_reticulata** **attcaaacaatgaaattcttggggaaccgcgatccattttgatcccccatttgctatttg**

**Citrus_sinensis** **attcaaacaatgaaattcttggggaaccgcgatccattttgatcccccatttgctatttg**

**Fortunella_bawangica_1** **attcaaacaatgaaatttttggggaaccgcgatccattttgatcccccatttgctatttg**

**Fortunella_bawangica_2** **attcaaacaatgaaatttttggggaaccgcgatccattttgatcccccatttgctatttg**

**Fortunella_hindsii_1** **attcaaacaatgaaattcttggggaaccgcgatccattttgatcccccatttgctatttg**

**Fortunella_hindsii_2** **attcaaacaatgaaattcttggggaaccgcgatccattttgatcccccatttgctatttg**

**Fortunella_hindsii_3** **attcaaacaatgaaattcttggggaaccgcgatccattttgatcccccatttgctatttg**

**Fortunella_japonica_1** **attcaaacaatgaaattcttggggaaccgcgatccattttgatcccccatttgctatttg**

**Fortunella_japonica_2** **attcaaacaatgaaattcttggggaaccgcgatccattttgatcccccatttgctatttg**

**Fortunella_japonica_3** **attcaaacaatgaaattcttggggaaccgcgatccattttgatcccccatttgctatttg**

**Fortunella_japonica_4** **attcaaacaatgaaattcttggggaaccgcgatccattttgatcccccatttgctatttg**

**Fortunella_japonica_5** **attcaaacaatgaaattcttggggaaccgcgatccattttgatcccccatttgctatttg**

**Fortunella_japonica_6** **attcaaacaatgaaattcttggggaaccgcgatccattttgatcccccatttgctatttg**

**Fortunella_japonica_7** **attcaaacaatgaaattcttggggaaccgcgatccattttgatcccccatttgctatttg**

**Fortunella_japonica_8** **attcaaacaatgaaattcttggggaaccgcgatccattttgatcccccatttgctatttg**

**Fortunella_japonica_9** **attcaaacaatgaaattcttggggaaccgcgatccattttgatcccccatttgctatttg**

**Fortunella_japonica_10** **attcaaacaatgaaatttttggggaaccgcgatccattttgatcccccatttgctatttg**

**Fortunella_japonica_11** **attcaaacaatgaaattcttggggaaccgcgatccattttgatcccccatttgctatttg**

**Fortunella_japonica_12** **attcaaacaatgaaattcttggggaaccgcgatccattttgatcccccatttgctatttg**

**Fortunella_margarita_1** **attcaaacaatgaaatttttggggaaccgcgatccattttgatcccccatttgctatttg**

**Fortunella_margarita_2** **attcaaacaatgaaatttttggggaaccgcgatccattttgatcccccatttgctatttg**

**Fortunella_venosa_1** **attcaaacaatgaaattcttggggaaccgcgatccattttgatcccccatttgctatttg**

**Fortunella_venosa_2** **attcaaacaatgaaattcttggggaaccgcgatccattttgatcccccatttgctatttg**

**Fortunella_venosa_3** **attcaaacaatgaaattcttggggaaccgcgatccattttgatcccccatttgctatttg**

8590	8600	8610	8620	8630	8640

....|....|....|....|....|....|....|....|....|....|....|....|

**Atalantia_kwangtungens** **-------ttgattatgaccctttttctttttcactgaaaccatcttatattagagccaac**

**Citrus_reticulata_1** **caatttgttgattatgacc------ctttttcactgaaaccatcttatattagagccaac**

**Citrus_reticulata_2** **caatttgttgattatgacc------ctttttcactgaaaccatcttatattagagccaac**

**Citrus_trifoliata_3** **caatttgttgattatgacc------ctttttcactgaaaccatcttatattatagccaac**

**Citrus_aurantiifolia** **caatttgttgattatgacc------ctttttcactgaaaccatcttatattagagccaac**

**Citrus_aurantium** **caattt--------tgacc------ctttttcactgaaaccatcttatattagagccaac**

**Citrus_cavaleriei** **caatttgttgattatgacc------ctttttcactgaaaccatcttatattagagccaac**

**Citrus_hongheensis** **caattt--------tgacc------ctttttcactgaaaccatcttatattagagccaac**

**Citrus_limon** **caattt--------tgacc------ctttttcactgaaaccatcttatattagagccaac**

**Citrus_maxima** **caattt--------tgacc------ctttttcactgaaaccatcttatattagagccaac**

**Citrus_medica** **caatttgttgattaggacc------ctttttcactgaaaccatcttatattagagccaac**

**Citrus_polytrifolia** **caatttgttgattatgacc------ctttttcactgaaaccatcttatattatagccaac**

**Citrus_reticulata** **caatttgttgattatgacc------ctttttcactgaaaccatcttatattagagccaac**

**Citrus_sinensis** **caattt--------tgacc------ctttttcactgaaaccatcttatattagagccaac**

**Fortunella_bawangica_1** **caatttgttgattatgacc------ctttttcactgaaaccatcttatattagagccaac**

**Fortunella_bawangica_2** **caatttgttgattatgacc------ctttttcactgaaaccatcttatattagagccaac**

**Fortunella_hindsii_1** **caatttgttgattatgacc------ctttttcactgaaaccatcttatattagagccaac**

**Fortunella_hindsii_2** **caatttgttgattatgacc------ctttttcactgaaaccatcttatattagagccaac**

**Fortunella_hindsii_3** **caatttgttgattatgacc------ctttttcactgaaaccatcttatattagagccaac**

**Fortunella_japonica_1** **caatttgttgattatgacc------ctttttcactgaaaccatcttatattagagccaac**

**Fortunella_japonica_2** **caatttgttgattatgacc------ctttttcactgaaaccatcttatattagagccaac**

**Fortunella_japonica_3** **caatttgttgattatgacc------ctttttcactgaaaccatcttatattagagccaac**

**Fortunella_japonica_4** **caatttgttgattatgacc------ctttttcactgaaaccatcttatattagagccaac**

**Fortunella_japonica_5** **caatttgttgattatgacc------ctttttcactgaaaccatcttatattagagccaac**

**Fortunella_japonica_6** **caatttgttgattatgacc------ctttttcactgaaaccatcttatattagagccaac**

**Fortunella_japonica_7** **caatttgttgattatgacc------ctttttcactgaaaccatcttatattagagccaac**

**Fortunella_japonica_8** **caatttgttgattatgacc------ctttttcactgaaaccatcttatattagagccaac**

**Fortunella_japonica_9** **caatttgttgattatgacc------ctttttcactgaaaccatcttatattagagccaac**

**Fortunella_japonica_10** **caatttgttgattatgacc------ctttttcactgaaaccatcttatattagagccaac**

**Fortunella_japonica_11** **caatttgttgattatgacc------ctttttcactgaaaccatcttatattagagccaac**

**Fortunella_japonica_12** **caatttgttgattatgacc------ctttttcactgaaaccatcttatattagagccaac**

**Fortunella_margarita_1** **caatttgttgattatgacc------ctttttcactgaaaccatcttatattagagccaac**

**Fortunella_margarita_2** **caatttgttgattatgacc------ctttttcactgaaaccatcttatattagagccaac**

**Fortunella_venosa_1** **caatttgttgattatgacc------ctttttcactgaaaccatcttatattagagccaac**

**Fortunella_venosa_2** **caatttgttgattatgacc------ctttttcactgaaacaatcttatattagagccaac**

**Fortunella_venosa_3** **caatttgttgattatgacc------ctttttcactgaaaccatcttatattagagccaac**

8650	8660	8670	8680	8690	8700

....|....|....|....|....|....|....|....|....|....|....|....|

**Atalantia_kwangtungens** **caaaatgtcgaattctaattgtgtgaatttcatttctgaaaacgattttctatttagaga**

**Citrus_reticulata_1** **caaaatgtagaattctaattgtgtgaatttaatttatgaaaacgattttctatttagaga**

**Citrus_reticulata_2** **caaaatgtagaattctaattgtgtgaatttaatttatgaaaacgattttctatttagaga**

**Citrus_trifoliata_3** **caaaatgtagaattctaattgtgtgaatttaatttatgaaaacgattttctatttagaga**

**Citrus_aurantiifolia** **caaaatgtagaattctaattgtgtgaatttaatttatgaaaacgattttctatttagaga**

**Citrus_aurantium** **caaaatgtagaattctaattgtgtgaatttaatttatgaaaacgattttctatttagaga**

**Citrus_cavaleriei** **caaaatgtagaattctaattgtgtgaatttaatttatgaaaacgattttcgatttagaga**

**Citrus_hongheensis** **caaaatgtagaattctaattgtgtgaatttaatttatgaaaacgattttctatttagaga**

**Citrus_limon** **caaaatgtagaattctaattgtgtgaatttaatttatgaaaacgattttctatttagaga**

**Citrus_maxima** **caaaatgtagaattctaattgtgtgaatttaatttatgaaaacgattttctatttagaga**

**Citrus_medica** **caaaatgtagaattctaattgtgtgaatttaatttatgaaaacgattttctatttagaga**

**Citrus_polytrifolia** **caaaatgtagaattctaattgtgtgaatttaatttatgaaaacgattttctatttagaga**

**Citrus_reticulata** **caaaatgtagaattctaattgtgtgaatttaatttatgaaaacgattttctatttagaga**

**Citrus_sinensis** **caaaatgtagaattctaattgtgtgaatttaatttatgaaaacgattttctatttagaga**

**Fortunella_bawangica_1** **caaaatgtagaattctaattgtgtgaatttaatttatgaaaacgattttctatttagaga**

**Fortunella_bawangica_2** **caaaatgtagaattctaattgtgtgaatttaatttatgaaaacgattttctatttagaga**

**Fortunella_hindsii_1** **caaaatgtagaattctaattgtgtgaatttaatttatgaaaacgattttctatttagaga**

**Fortunella_hindsii_2** **caaaatgtagaattctaattgtgtgaatttaatttatgaaaacgattttctatttagaga**

**Fortunella_hindsii_3** **caaaatgtagaattctaattgtgtgaatttaatttatgaaaacgattttctatttagaga**

**Fortunella_japonica_1** **caaaatgtagaattctaattgtgtgaatttaatttatgaaaacgattttctatttagaga**

**Fortunella_japonica_2** **caaaatgtagaattctaattgtgtgaatttaatttatgaaaacgattttctatttagaga**

**Fortunella_japonica_3** **caaaatgtagaattctaattgtgtgaatttaatttatgaaaacgattttctatttagaga**

**Fortunella_japonica_4** **caaaatgtagaattctaattgtgtgaatttaatttatgaaaacgattttctatttagaga**

**Fortunella_japonica_5** **caaaatgtagaattctaattgtgtgaatttaatttatgaaaacgattttctatttagaga**

**Fortunella_japonica_6** **caaaatgtagaattctaattgtgtgaatttaatttatgaaaacgattttctatttagaga**

**Fortunella_japonica_7** **caaaatgtagaattctaattgtgtgaatttaatttatgaaaacgattttctatttagaga**

**Fortunella_japonica_8** **caaaatgtagaattctaattgtgtgaatttaatttatgaaaacgattttctatttagaga**

**Fortunella_japonica_9** **caaaatgtagaattctaattgtgtgaatttaatttatgaaaacgattttctatttagaga**

**Fortunella_japonica_10** **caaaatgtagaattctaattgtgtgaatttaatttatgaaaacgattttctatttagaga**

**Fortunella_japonica_11** **caaaatgtagaattctaattgtgtgaatttaatttatgaaaacgattttctatttagaga**

**Fortunella_japonica_12** **caaaatgtagaattctaattgtgtgaatttaatttatgaaaacgattttctatttagaga**

**Fortunella_margarita_1** **caaaatgtagaattctaattgtgtgaatttaatttatgaaaacgattttctatttagaga**

**Fortunella_margarita_2** **caaaatgtagaattctaattgtgtgaatttaatttatgaaaacgattttctatttagaga**

**Fortunella_venosa_1** **caaaatgtagaattctaattgtgtgaatttaatttatgaaaacgattttctatttagaga**

**Fortunella_venosa_2** **caaaatgtagaattctaattgtgtgaatttaatttatgaaaacgattttctatttagaga**

**Fortunella_venosa_3** **caaaatgtagaattctaattgtgtgaatttaatttatgaaaacgattttctatttagaga**

8710	8720	8730	8740	8750	8760

....|....|....|....|....|....|....|....|....|....|....|....|

**Atalantia_kwangtungens** **atcaaattctgaaaacgattttctatttagagaatcaaattctaaaaagaacttcatttc**

**Citrus_reticulata_1** **atc---------------------------------aaattctaaaaagaacttcatttc**

**Citrus_reticulata_2** **atc---------------------------------aaattctaaaaagaacttcatttc**

**Citrus_trifoliata_3** **atc---------------------------------aaattctaaaaagaacttcatttc**

**Citrus_aurantiifolia** **atc---------------------------------aaattctaaaaagaacttcatttc**

**Citrus_aurantium** **atc---------------------------------aaattctaaaaagaacttcatttc**

**Citrus_cavaleriei** **atc---------------------------------aaattataaaaagaacttcatttc**

**Citrus_hongheensis** **atc---------------------------------aaattctaaaaagaacttcatttc**

**Citrus_limon** **atc---------------------------------aaattctaaaaagaacttcatttc**

**Citrus_maxima** **atc---------------------------------aaattctaaaaagaacttcatttc**

**Citrus_medica** **atc---------------------------------aaattctaaaaagaacttcatttc**

**Citrus_polytrifolia** **atc---------------------------------aaattctaaaaagaacttcatttc**

**Citrus_reticulata** **atc---------------------------------aaattctaaaaagaacttcatttc**

**Citrus_sinensis** **atc---------------------------------aaattctaaaaagaacttcatttc**

**Fortunella_bawangica_1** **atcaaattc-------------------------taaaattctaaaaagaacttcatttc**

**Fortunella_bawangica_2** **atcaaattc-------------------------taaaattctaaaaagaacttcatttc**

**Fortunella_hindsii_1** **atcaaattc-------------------------taaaattctaaaaagaacttcatttc**

**Fortunella_hindsii_2** **atcaaattc-------------------------taaaattctaaaaagaacttcatttc**

**Fortunella_hindsii_3** **atcaaattc-------------------------taaaattctaaaaagaacttcatttc**

**Fortunella_japonica_1** **atcaaattc-------------------------taaaattctaaaaagaacttcatttc**

**Fortunella_japonica_2** **atcaaattc-------------------------taaaattctaaaaagaacttcatttc**

**Fortunella_japonica_3** **atcaaattc-------------------------taaaattctaaaaagaacttcatttc**

**Fortunella_japonica_4** **atcaaattc-------------------------taaaattctaaaaagaacttcatttc**

**Fortunella_japonica_5** **atcaaattc-------------------------taaaattctaaaaagaacttcatttc**

**Fortunella_japonica_6** **atcaaattc-------------------------taaaattctaaaaagaacttcatttc**

**Fortunella_japonica_7** **atcaaattc-------------------------taaaattctaaaaagaacttcatttc**

**Fortunella_japonica_8** **atcaaattc-------------------------taaaattctaaaaagaacttcatttc**

**Fortunella_japonica_9** **atcaaattc-------------------------taaaattctaaaaagaacttcatttc**

**Fortunella_japonica_10** **atcaaattc-------------------------taaaattctaaaaagaacttcatttc**

**Fortunella_japonica_11** **atcaaattc-------------------------taaaattctaaaaagaacttcatttc**

**Fortunella_japonica_12** **atcaaattc-------------------------taaaattctaaaaagaacttcatttc**

**Fortunella_margarita_1** **atcaaattc-------------------------taaaattctaaaaagaacttcatttc**

**Fortunella_margarita_2** **atcaaattc-------------------------taaaattctaaaaagaacttcatttc**

**Fortunella_venosa_1** **atcaaattc-------------------------taaaattctaaaaagaacttcatttc**

**Fortunella_venosa_2** **atcaaattc-------------------------taaaattctaaaaagaacttcatttc**

**Fortunella_venosa_3** **atcaaattc-------------------------taaaattctaaaaagaacttcatttc**

8770	8780	8790	8800	8810	8820

....|....|....|....|....|....|....|....|....|....|....|....|

**Atalantia_kwangtungens** **ttgatgtcaaaattggtgtcaaaattggatatgtagtataaaaatagagaatctattctc**

**Citrus_reticulata_1** **ttgatgtcaaaat------------tggatatgtagtataaaaatagagaatctattctc**

**Citrus_reticulata_2** **ttgatgtcaaaat------------tggatatgtagtataaaaatagagaatctattctc**

**Citrus_trifoliata_3** **ttgatgtcaaaat------------tggatatgtagtataaaaatagagaatctattctc**

**Citrus_aurantiifolia** **ttgatgtcaaaat------------tggatatgtagtataaaaatagagaatctattctc**

**Citrus_aurantium** **ttgatgtcaaaat------------tggatatgtagtataaaaatagagaatctattctc**

**Citrus_cavaleriei** **ttgatgtcaaaat------------tggatatgtagtataaaaatagagaatctattctc**

**Citrus_hongheensis** **ttgatgtcaaaat------------tggatatgtagtataaaaatagagaatctattctc**

**Citrus_limon** **ttgatgtcaaaat------------tggatatgtagtataaaaatagagaatctattctc**

**Citrus_maxima** **ttgatgtcaaaat------------tggatatgtagtataaaaatagagaatctattctc**

**Citrus_medica** **ttgatgtcaaaat------------tggatatgtagtataaaaatagagaatctattctc**

**Citrus_polytrifolia** **ttgatgtcaaaat------------tggatatgtagtataaaaatagagaatctattctc**

**Citrus_reticulata** **ttgatgtcaaaat------------tggatatgtagtataaaaatagagaatctattctc**

**Citrus_sinensis** **ttgatgtcaaaat------------tggatatgtagtataaaaatagagaatctattctc**

**Fortunella_bawangica_1** **ttgatgtcaaaat------------tggatatgtagtataaaaatagagaatctattctc**

**Fortunella_bawangica_2** **ttgatgtcaaaat------------tggatatgtagtataaaaatagagaatctattctc**

**Fortunella_hindsii_1** **ttgatgtcaaaat------------tggatatgtagtataaaaatagagaatctattctc**

**Fortunella_hindsii_2** **ttgatgtcaaaat------------tggatatgtagtataaaaatagagaatctattctc**

**Fortunella_hindsii_3** **ttgatgtcaaaat------------tggatatgtagtataaaaatagagaatctattctc**

**Fortunella_japonica_1** **ttgatgtcaaaat------------tggatatgtagtataaaaatagagaatctattctc**

**Fortunella_japonica_2** **ttgatgtcaaaat------------tggatatgtagtataaaaatagagaatctattctc**

**Fortunella_japonica_3** **ttgatgtcaaaat------------tggatatgtagtataaaaatagagaatctattctc**

**Fortunella_japonica_4** **ttgatgtcaaaat------------tggatatgtagtataaaaatagagaatctattctc**

**Fortunella_japonica_5** **ttgatgtcaaaat------------tggatatgtagtataaaaatagagaatctattctc**

**Fortunella_japonica_6** **ttgatgtcaaaat------------tggatatgtagtataaaaatagagaatctattctc**

**Fortunella_japonica_7** **ttgatgtcaaaat------------tggatatgtagtataaaaatagagaatctattctc**

**Fortunella_japonica_8** **ttgatgtcaaaat------------tggatatgtagtataaaaatagagaatctattctc**

**Fortunella_japonica_9** **ttgatgtcaaaat------------tggatatgtagtataaaaatagagaatctattctc**

**Fortunella_japonica_10** **ttgatgtcaaaat------------tggatatgtagtataaaaatagagaatctattctc**

**Fortunella_japonica_11** **ttgatgtcaaaat------------tggatatgtagtataaaaatagagaatctattctc**

**Fortunella_japonica_12** **ttgatgtcaaaat------------tggatatgtagtataaaaatagagaatctattctc**

**Fortunella_margarita_1** **ttgatgtcaaaat------------tggatatgtagtataaaaatagagaatctattctc**

**Fortunella_margarita_2** **ttgatgtcaaaat------------tggatatgtagtataaaaatagagaatctattctc**

**Fortunella_venosa_1** **ttgatgtcaaaat------------tggatatgtagtataaaaatagagaatctattctc**

**Fortunella_venosa_2** **ttgatgtcaaaat------------tggatatgtagtataaaaatagagaatctattctc**

**Fortunella_venosa_3** **ttgatgtcaaaat------------tggatatgtagtataaaaatagagaatctattctc**

8830	8840	8850	8860	8870	8880

....|....|....|....|....|....|....|....|....|....|....|....|

**Atalantia_kwangtungens** **tttttccttttccc------caaaaaccttatttggagattgtgtaatgcttactctcaa**

**Citrus_reticulata_1** **tttttccttttccc------caaaaacctgatttggagattgtgtaatgcttactctcaa**

**Citrus_reticulata_2** **tttttccttttccc------caaaaacctgatttggagattgtgtaatgcttactctcaa**

**Citrus_trifoliata_3** **tttttccttttccccaaaaacaaaaacccgatttggagattgtgtaatgcttactctcaa**

**Citrus_aurantiifolia** **tttttccttttccc------caaaaacctgatttggagattgtgtaatgcttactctcaa**

**Citrus_aurantium** **tttttccttttccc------caaaaacctgatttggagattgtgtaatgcttactctcaa**

**Citrus_cavaleriei** **tttttccttttccc------caaaaacctgatttggagattgtgtaatgcttactctcaa**

**Citrus_hongheensis** **tttttccttttccc------caaaaacctgatttggagattgtgtaatgcttactctcaa**

**Citrus_limon** **tttttccttttccc------caaaaacctgatttggagattgtgtaatgcttactctcaa**

**Citrus_maxima** **tttttccttttccc------caaaaacctgatttggagattgtgtaatgcttactctcaa**

**Citrus_medica** **tttttccttttccc------caaaaacctgatttggagattgtgtaatgcttactctcaa**

**Citrus_polytrifolia** **tttttccttttccc------caaaaacccgatttggagattgtgtaatgcttactctcaa**

**Citrus_reticulata** **tttttccttttccc------caaaaacctgatttggagattgtgtaatgcttactctcaa**

**Citrus_sinensis** **tttttccttttccc------caaaaacctgatttggagattgtgtaatgcttactctcaa**

**Fortunella_bawangica_1** **tttttccttttccc------aaaaaacctgatttggagattgtgtaatgcttactctcaa**

**Fortunella_bawangica_2** **tttttccttttccc------aaaaaacctgatttggagattgtgtaatgcttactctcaa**

**Fortunella_hindsii_1** **tttttccttttccc------aaaaaacctgatttggagattgtgtaatgcttactctcaa**

**Fortunella_hindsii_2** **tttttccttttccc------aaaaaacctgatttggagattgtgtaatgcttactctcaa**

**Fortunella_hindsii_3** **tttttccttttccc------aaaaaacctgatttggagattgtgtaatgcttactctcaa**

**Fortunella_japonica_1** **tttttccttttccc------aaaaaacctgatttggagattgtgtaatgcttactctcaa**

**Fortunella_japonica_2** **tttttccttttccc------aaaaaacctgatttggagattgtgtaatgcttactctcaa**

**Fortunella_japonica_3** **tttttccttttccc------aaaaaacctgatttggagattgtgtaatgcttactctcaa**

**Fortunella_japonica_4** **tttttccttttccc------aaaaaacctgatttggagattgtgtaatgcttactctcaa**

**Fortunella_japonica_5** **tttttccttttccc------aaaaaacctgatttggagattgtgtaatgcttactctcaa**

**Fortunella_japonica_6** **tttttccttttccc------aaaaaacctgatttggagattgtgtaatgcttactctcaa**

**Fortunella_japonica_7** **tttttccttttccc------aaaaaacctgatttggagattgtgtaatgcttactctcaa**

**Fortunella_japonica_8** **tttttccttttccc------aaaaaacctgatttggagattgtgtaatgcttactctcaa**

**Fortunella_japonica_9** **tttttccttttccc------aaaaaacctgatttggagattgtgtaatgcttactctcaa**

**Fortunella_japonica_10** **tttttccttttccc------aaaaaacctgatttggagattgtgtaatgcttactctcaa**

**Fortunella_japonica_11** **tttttccttttccc------aaaaaacctgatttggagattgtgtaatgcttactctcaa**

**Fortunella_japonica_12** **tttttccttttccc------aaaaaacctgatttggagattgtgtaatgcttactctcaa**

**Fortunella_margarita_1** **tttttccttttccc------aaaaaacctgatttggagattgtgtaatgcttactctcaa**

**Fortunella_margarita_2** **tttttccttttccc------aaaaaacctgatttggagattgtgtaatgcttactctcaa**

**Fortunella_venosa_1** **tttttccttttccc------aaaaaacctgatttggagattgtgtaatgcttactctcaa**

**Fortunella_venosa_2** **tttttccttttccc------aaaaaacctgatttggagattgtgtaatgcttactctcaa**

**Fortunella_venosa_3** **tttttccttttccc------aaaaaacctgatttggagattgtgtaatgcttactctcaa**

8890	8900	8910	8920	8930	8940

....|....|....|....|....|....|....|....|....|....|....|....|

**Atalantia_kwangtungens** **actctttgtttacaccgtagtgatattctttgtttcgctcttcatcttcggattcctgtc**

**Citrus_reticulata_1** **actctttgtttacaccgtagtgatattctttgtttcgctcttcatcttcggattcctgtc**

**Citrus_reticulata_2** **actctttgtttacaccgtagtgatattctttgtttcgctcttcatcttcggattcctgtc**

**Citrus_trifoliata_3** **actctttgtttacaccgtagtgatattctttgtttcgctcttcatcttcggattcctgtc**

**Citrus_aurantiifolia** **actctttgtttacaccgtagtgatattctttgtttcgctcttcatcttcggattcctgtc**

**Citrus_aurantium** **actctttgtttacaccgtagtgatattctttgtttcgctcttcatcttcggattcctgtc**

**Citrus_cavaleriei** **actctttgtttacaccgtagtgatattctttgtttcgctcttcatcttcggattcctgtc**

**Citrus_hongheensis** **actctttgtttacaccgtagtgatattctttgtttcgctcttcatcttcggattcctgtc**

**Citrus_limon** **actctttgtttacaccgtagtgatattctttgtttcgctcttcatcttcggattcctgtc**

**Citrus_maxima** **actctttgtttacaccgtagtgatattctttgtttcgctcttcatcttcggattcctgtc**

**Citrus_medica** **actctttgtttacaccgtagtgatattctttgtttcgctcttcatcttcggattcctgtc**

**Citrus_polytrifolia** **actctttgtttacaccgtagtgatattctttgtttcgctcttcatcttcggattcctgtc**

**Citrus_reticulata** **actctttgtttacaccgtagtgatattctttgtttcgctcttcatcttcggattcctgtc**

**Citrus_sinensis** **actctttgtttacaccgtagtgatattctttgtttcgctcttcatcttcggattcctgtc**

**Fortunella_bawangica_1** **actctttgtttacaccgtagtgatattctttgtttcgctcttcatcttcggattcctgtc**

**Fortunella_bawangica_2** **actctttgtttacaccgtagtgatattctttgtttcgctcttcatcttcggattcctgtc**

**Fortunella_hindsii_1** **actctttgtttacaccgtagtgatattctttgtttcgctcttcatcttcggattcctgtc**

**Fortunella_hindsii_2** **actctttgtttacaccgtagtgatattctttgtttcgctcttcatcttcggattcctgtc**

**Fortunella_hindsii_3** **actctttgtttacaccgtagtgatattctttgtttcgctcttcatcttcggattcctgtc**

**Fortunella_japonica_1** **actctttgtttacaccgtagtgatattctttgtttcgctcttcatcttcggattcctgtc**

**Fortunella_japonica_2** **actctttgtttacaccgtagtgatattctttgtttcgctcttcatcttcggattcctgtc**

**Fortunella_japonica_3** **actctttgtttacaccgtagtgatattctttgtttcgctcttcatcttcggattcctgtc**

**Fortunella_japonica_4** **actctttgtttacaccgtagtgatattctttgtttcgctcttcatcttcggattcctgtc**

**Fortunella_japonica_5** **actctttgtttacaccgtagtgatattctttgtttcgctcttcatcttcggattcctgtc**

**Fortunella_japonica_6** **actctttgtttacaccgtagtgatattctttgtttcgctcttcatcttcggattcctgtc**

**Fortunella_japonica_7** **actctttgtttacaccgtagtgatattctttgtttcgctcttcatcttcggattcctgtc**

**Fortunella_japonica_8** **actctttgtttacaccgtagtgatattctttgtttcgctcttcatcttcggattcctgtc**

**Fortunella_japonica_9** **actctttgtttacaccgtagtgatattctttgtttcgctcttcatcttcggattcctgtc**

**Fortunella_japonica_10** **actctttgtttacaccgtagtgatattctttgtttcgctcttcatcttcggattcctgtc**

**Fortunella_japonica_11** **actctttgtttacaccgtagtgatattctttgtttcgctcttcatcttcggattcctgtc**

**Fortunella_japonica_12** **actctttgtttacaccgtagtgatattctttgtttcgctcttcatcttcggattcctgtc**

**Fortunella_margarita_1** **actctttgtttacaccgtagtgatattctttgtttcgctcttcatcttcggattcctgtc**

**Fortunella_margarita_2** **actctttgtttacaccgtagtgatattctttgtttcgctcttcatcttcggattcctgtc**

**Fortunella_venosa_1** **actctttgtttacaccgtagtgatattctttgtttcgctcttcatcttcggattcctgtc**

**Fortunella_venosa_2** **actctttgtttacaccgtagtgatattctttgtttcgctcttcatcttcggattcctgtc**

**Fortunella_venosa_3** **actctttgtttacaccgtagtgatattctttgtttcgctcttcatcttcggattcctgtc**

8950	8960	8970	8980	8990	9000

....|....|....|....|....|....|....|....|....|....|....|....|

**Atalantia_kwangtungens** **taatgatccagggcgtaatcccggacgcgaagaataaaaaaggaaggggttgcttacttt**

**Citrus_reticulata_1** **taatgatccagggcgtaatcccggacgcgaagaataaaaaaggaaggagttgcttacttt**

**Citrus_reticulata_2** **taatgatccagggcgtaatcccggacgcgaagaataaaaaaggaaggagttgcttacttt**

**Citrus_trifoliata_3** **taatgatccagggcgtaatcccggacgcgaagaataaaaaaggaaggagttgcttacttt**

**Citrus_aurantiifolia** **taatgatccagggcgtaatcccggacgcgaagaataaaaaaggaaggagttgcttacttt**

**Citrus_aurantium** **taatgatccagggcgtaatcccggacgcgaagaataaaaaaggaaggagttgcttacttt**

**Citrus_cavaleriei** **taatgatccagggcgtaatcccggacgcgaagaataaaaaaggaaggagttgcttacttt**

**Citrus_hongheensis** **taatgatccagggcgtaatcccggacgcgaagaataaaaaaggaaggagttgcttacttt**

**Citrus_limon** **taatgatccagggcgtaatcccggacgcgaagaataaaaaaggaaggagttgcttacttt**

**Citrus_maxima** **taatgatccagggcgtaatcccggacgcgaagaataaaaaaggaaggagttgcttacttt**

**Citrus_medica** **taatgatccagggcgtaatcccggacgcgaagaataaaaaaggaaggagttgcttacttt**

**Citrus_polytrifolia** **taatgatccagggcgtaatcccggacgcgaagaataaaaaaggaaggagttgcttacttt**

**Citrus_reticulata** **taatgatccagggcgtaatcccggacgcgaagaataaaaaaggaaggagttgcttacttt**

**Citrus_sinensis** **taatgatccagggcgtaatcccggacgcgaagaataaaaaaggaaggagttgcttacttt**

**Fortunella_bawangica_1** **taatgatccagggcgtaatcccggacgcgaagaataaaaaaggaaggagttgcttacttt**

**Fortunella_bawangica_2** **taatgatccagggcgtaatcccggacgcgaagaataaaaaaggaaggagttgcttacttt**

**Fortunella_hindsii_1** **taatgatccagggcgtaatcccggacgcgaagaataaaaaaggaaggagttgcttacttt**

**Fortunella_hindsii_2** **taatgatccagggcgtaatcccggacgcgaagaataaaaaaggaaggagttgcttacttt**

**Fortunella_hindsii_3** **taatgatccagggcgtaatcccggacgcgaagaataaaaaaggaaggagttgcttacttt**

**Fortunella_japonica_1** **taatgatccagggcgtaatcccggacgcgaagaataaaaaaggaaggagttgcttacttt**

**Fortunella_japonica_2** **taatgatccagggcgtaatcccggacgcgaagaataaaaaaggaaggagttgcttacttt**

**Fortunella_japonica_3** **taatgatccagggcgtaatcccggacgcgaagaataaaaaaggaaggagttgcttacttt**

**Fortunella_japonica_4** **taatgatccagggcgtaatcccggacgcgaagaataaaaaaggaaggagttgcttacttt**

**Fortunella_japonica_5** **taatgatccagggcgtaatcccggacgcgaagaataaaaaaggaaggagttgcttacttt**

**Fortunella_japonica_6** **taatgatccagggcgtaatcccggacgcgaagaataaaaaaggaaggagttgcttacttt**

**Fortunella_japonica_7** **taatgatccagggcgtaatcccggacgcgaagaataaaaaaggaaggagttgcttacttt**

**Fortunella_japonica_8** **taatgatccagggcgtaatcccggacgcgaagaataaaaaaggaaggagttgcttacttt**

**Fortunella_japonica_9** **taatgatccagggcgtaatcccggacgcgaagaataaaaaaggaaggagttgcttacttt**

**Fortunella_japonica_10** **taatgatccagggcgtaatcccggacgcgaagaataaaaaaggaaggagttgcttacttt**

**Fortunella_japonica_11** **taatgatccagggcgtaatcccggacgcgaagaataaaaaaggaaggagttgcttacttt**

**Fortunella_japonica_12** **taatgatccagggcgtaatcccggacgcgaagaataaaaaaggaaggagttgcttacttt**

**Fortunella_margarita_1** **taatgatccagggcgtaatcccggacgcgaagaataaaaaaggaaggagttgcttacttt**

**Fortunella_margarita_2** **taatgatccagggcgtaatcccggacgcgaagaataaaaaaggaaggagttgcttacttt**

**Fortunella_venosa_1** **taatgatccagggcgtaatcccggacgcgaagaataaaaaaggaaggagttgcttacttt**

**Fortunella_venosa_2** **taatgatccagggcgtaatcccggacgcgaagaataaaaaaggaaggagttgcttacttt**

**Fortunella_venosa_3** **taatgatccagggcgtaatcccggacgcgaagaataaaaaaggaaggagttgcttacttt**

9010	9020	9030	9040	9050	9060

....|....|....|....|....|....|....|....|....|....|....|....|

**Atalantia_kwangtungens** **attcgtataaatgttcttaggattttttgattcc------ctgttactattaaaaa-taa**

**Citrus_reticulata_1** **attcgtataaacgttcttaggattttttgattcc------cagttactattaaaaa----**

**Citrus_reticulata_2** **attcgtataaacgttcttaggattttttgattcc------cagttactattaaaaa----**

**Citrus_trifoliata_3** **attcgtataaacgttcttaggattttttgattcc------cagttactattaaaaa----**

**Citrus_aurantiifolia** **attcgtataaacgttcttaggattttttgattcccagttacagttactattaaaaa----**

**Citrus_aurantium** **attcgtataaacgttcttaggattttttgattcc------cagttactattaaaaa----**

**Citrus_cavaleriei** **attcgtataaacgttcttaggattttttgattcc------cagttactattaaaaa----**

**Citrus_hongheensis** **attcgtataaacgttcttaggattttttgattcc------cagttactattaaaaa----**

**Citrus_limon** **attcgtataaacgttcttaggattttttgattcc------cagttactattaaaaa----**

**Citrus_maxima** **attcgtataaacgttcttaggattttttgattcc------cagttactattaaaaa----**

**Citrus_medica** **attcgtataaacgttcttaggattttttgattcc------cagttactattaaaaataaa**

**Citrus_polytrifolia** **attcgtataaacgttcttaggattttttgattcc------cagttactattaaaaa----**

**Citrus_reticulata** **attcgtataaacgttcttaggattttttgattcc------cagttactattaaaaa----**

**Citrus_sinensis** **attcgtataaacgttcttaggattttttgattcc------cagttactattaaaaa----**

**Fortunella_bawangica_1** **attcgtataaacgttcttaggattttttgattcc------cagttactattaaaaa----**

**Fortunella_bawangica_2** **attcgtataaacgttcttaggattttttgattcc------cagttactattaaaaa----**

**Fortunella_hindsii_1** **attcgtataaacgttcttaggattttttgattcc------cagttactattaaaaa----**

**Fortunella_hindsii_2** **attcgtataaacgttcttaggattttttgattcc------cagttactattaaaaa----**

**Fortunella_hindsii_3** **attcgtataaacgttcttaggattttttgattcc------cagttactattaaaaa----**

**Fortunella_japonica_1** **attcgtataaacgttcttaggattttttgattcc------cagttactattaaaaa----**

**Fortunella_japonica_2** **attcgtataaacgttcttaggattttttgattcc------cagttactattaaaaa----**

**Fortunella_japonica_3** **attcgtataaacgttcttaggattttttgattcc------cagttactattaaaaa----**

**Fortunella_japonica_4** **attcgtataaacgttcttaggattttttgattcc------cagttactattaaaaa----**

**Fortunella_japonica_5** **attcgtataaacgttcttaggattttttgattcc------cagttactattaaaaa----**

**Fortunella_japonica_6** **attcgtataaacgttcttaggattttttgattcc------cagttactattaaaaa----**

**Fortunella_japonica_7** **attcgtataaacgttcttaggattttttgattcc------cagttactattaaaaa----**

**Fortunella_japonica_8** **attcgtataaacgttcttaggattttttgattcc------cagttactattaaaaa----**

**Fortunella_japonica_9** **attcgtataaacgttcttaggattttttgattcc------cagttactattaaaaa----**

**Fortunella_japonica_10** **attcgtataaacgttcttaggattttttgattcc------cagttactattaaaaa----**

**Fortunella_japonica_11** **attcgtataaacgttcttaggattttttgattcc------cagttactattaaaaa----**

**Fortunella_japonica_12** **attcgtataaacgttcttaggattttttgattcc------cagttactattaaaaa----**

**Fortunella_margarita_1** **attcgtataaacgttcttaggattttttgattcc------cagttactattaaaaa----**

**Fortunella_margarita_2** **attcgtataaacgttcttaggattttttgattcc------cagttactattaaaaa----**

**Fortunella_venosa_1** **attcgtataaacgttcttaggattttttgattcc------cagttactattaaaaa----**

**Fortunella_venosa_2** **attcgtataaacgttcttaggattttttgattcc------cagttactattaaaaa----**

**Fortunella_venosa_3** **attcgtataaacgttcttaggattttttgattcc------cagttactattaaaaa----**

9070	9080	9090	9100	9110	9120

....|....|....|....|....|....|....|....|....|....|....|....|

**Atalantia_kwangtungens** **agtaaaataaaaataaagtaaaagtgttcgctaaattcgctaaagttaaattttaaagat**

**Citrus_reticulata_1** **-------------taaagtaaaagtgttcgctaa---------aggtaaatttgaaaaat**

**Citrus_reticulata_2** **-------------taaagtaaaagtgttcgctaa---------aggtaaatttgaaaaat**

**Citrus_trifoliata_3** **-------------gaaagtaaaagtgttcgctaa---------agttcaatttgaaaaat**

**Citrus_aurantiifolia** **-------------taaagtaaaagtgttcgctaa---------agttaaatttgaaaaat**

**Citrus_aurantium** **-------------taaagtaaaagtgttcgctaa---------agttaaatttgaaaaat**

**Citrus_cavaleriei** **-------------taaagtaaaagtgttcgctaa---------aggtaaatttgaaaaat**

**Citrus_hongheensis** **-------------taaagtaaaagtgttcgctaa---------agttaaatttgaaaaat**

**Citrus_limon** **-------------taaagtaaaagtgttcgctaa---------agttaaatttgaaaaat**

**Citrus_maxima** **-------------taaagtaaaagtgttcgctaa---------agttaaatttgaaaaat**

**Citrus_medica** **aatcaagtaaaaataaagtaaaagtgttcgctaa---------agttaaatttgaaaaat**

**Citrus_polytrifolia** **-------------gaaagtaaaagtgttcgctaa---------agttcaatttgaaaaat**

**Citrus_reticulata** **-------------taaagtaaaagtgttcgctaa---------aggtaaatttgaaaaat**

**Citrus_sinensis** **-------------taaagtaaaagtgttcgctaa---------agttaaatttgaaaaat**

**Fortunella_bawangica_1** **-------------taaagtaaaagtgttcgctaa---------agttaaatttgaaaaat**

**Fortunella_bawangica_2** **-------------taaagtaaaagtgttcgctaa---------agttaaatttgaaaaat**

**Fortunella_hindsii_1** **-------------taaagtaaaagtgttcgctaa---------agttaaatttgaaaaat**

**Fortunella_hindsii_2** **-------------taaagtaaaagtgttcgctaa---------agttaaatttgaaaaat**

**Fortunella_hindsii_3** **-------------taaagtaaaagtgttcgctaa---------agttaaatttgaaaaat**

**Fortunella_japonica_1** **-------------taaagtaaaagtgttcgctaa---------agttaaatttgaaaaat**

**Fortunella_japonica_2** **-------------taaagtaaaagtgttcgctaa---------agttaaatttgaaaaat**

**Fortunella_japonica_3** **-------------taaagtaaaagtgttcgctaa---------agttaaatttgaaaaat**

**Fortunella_japonica_4** **-------------taaagtaaaagtgttcgctaa---------agttaaatttgaaaaat**

**Fortunella_japonica_5** **-------------taaagtaaaagtgttcgctaa---------agttaaatttgaaaaat**

**Fortunella_japonica_6** **-------------taaagtaaaagtgttcgctaa---------agttaaatttgaaaaat**

**Fortunella_japonica_7** **-------------taaagtaaaagtgttcgctaa---------agttaaatttgaaaaat**

**Fortunella_japonica_8** **-------------taaagtaaaagtgttcgctaa---------agttaaatttgaaaaat**

**Fortunella_japonica_9** **-------------taaagtaaaagtgttcgctaa---------agttaaatttgaaaaat**

**Fortunella_japonica_10** **-------------taaagtaaaagtgttcgctaa---------agttaaatttgaaaaat**

**Fortunella_japonica_11** **-------------taaagtaaaagtgttcgctaa---------agttaaatttgaaaaat**

**Fortunella_japonica_12** **-------------taaagtaaaagtgttcgctaa---------agttaaatttgaaaaat**

**Fortunella_margarita_1** **-------------taaagtaaaagtgttcgctaa---------agttaaatttgaaaaat**

**Fortunella_margarita_2** **-------------taaagtaaaagtgttcgctaa---------agttaaatttgaaaaat**

**Fortunella_venosa_1** **-------------taaagtaaaagtgttcgctaa---------agttaaatttgaaaaat**

**Fortunella_venosa_2** **-------------taaagtaaaagtgttcgctaa---------agttaaatttgaaaaat**

**Fortunella_venosa_3** **-------------taaagtaaaagtgttcgctaa---------agttaaatttgaaaaat**

9130	9140	9150	9160	9170	9180

....|....|....|....|....|....|....|....|....|....|....|....|

**Atalantia_kwangtungens** **accaagccatcgaccgaaacggaaagagagggattcgaaccctcggtacgaataactcgt**

**Citrus_reticulata_1** **accaagccatcgcccgaaacggaaagagagggattcgaaccctcggtacgaataactcgt**

**Citrus_reticulata_2** **accaagccatcgcccgaaacggaaagagagggattcgaaccctcggtacgaataactcgt**

**Citrus_trifoliata_3** **accaagccatcgcccgaaacggaaagagagggattcgaaccctcggtacgaataactcgt**

**Citrus_aurantiifolia** **accaagccatcgcccgaaacggaaagagagggattcgaaccctcggtacgaataactcgt**

**Citrus_aurantium** **accaagccatcgcccgaaacggaaagagagggattcgaaccctcggtacgaagaactcgt**

**Citrus_cavaleriei** **accaagccatcgcccgaaacggaaagagagggattcgaaccctcggtacgaataactcgt**

**Citrus_hongheensis** **accaagccatcgcccgaaacggaaagagagggattcgaaccctcggtacgaagaactcgt**

**Citrus_limon** **accaagccatcgcccgaaacggaaagagagggattcgaaccctcggtacgaagaactcgt**

**Citrus_maxima** **accaagccatcgcccgaaacggaaagagagggattcgaaccctcggtacgaagaactcgt**

**Citrus_medica** **accaagccatcacccgaaacggaaagagagggattcgaaccctcggtacgaataactcgt**

**Citrus_polytrifolia** **accaagccatcgcccgaaacggaaagagagggattcgaaccctcggtacgaataactcgt**

**Citrus_reticulata** **accaagccatcgcccgaaacggaaagagagggattcgaaccctcggtacgaataactcgt**

**Citrus_sinensis** **accaagccatcgcccgaaacggaaagagagggattcgaaccctcggtacgaagaactcgt**

**Fortunella_bawangica_1** **accaagccatcgcccgaaacggaaagagagggattcgaaccctcggtacgaataactcgt**

**Fortunella_bawangica_2** **accaagccatcgcccgaaacggaaagagagggattcgaaccctcggtacgaataactcgt**

**Fortunella_hindsii_1** **accaagccatcgcccgaaacggaaagagagggattcgaaccctcggtacgaataactcgt**

**Fortunella_hindsii_2** **accaagccatcgcccgaaacggaaagagagggattcgaaccctcggtacgaataactcgt**

**Fortunella_hindsii_3** **accaagccatcgcccgaaacggaaagagagggattcgaaccctcggtacgaataactcgt**

**Fortunella_japonica_1** **accaagccatcgcccgaaacggaaagagagggattcgaaccctcggtacgaataactcgt**

**Fortunella_japonica_2** **accaagccatcgcccgaaacggaaagagagggattcgaaccctcggtacgaataactcgt**

**Fortunella_japonica_3** **accaagccatcgcccgaaacggaaagagagggattcgaaccctcggtacgaataactcgt**

**Fortunella_japonica_4** **accaagccatcgcccgaaacggaaagagagggattcgaaccctcggtacgaataactcgt**

**Fortunella_japonica_5** **accaagccatcgcccgaaacggaaagagagggattcgaaccctcggtacgaataactcgt**

**Fortunella_japonica_6** **accaagccatcgcccgaaacggaaagagagggattcgaaccctcggtacgaataactcgt**

**Fortunella_japonica_7** **accaagccatcgcccgaaacggaaagagagggattcgaaccctcggtacgaataactcgt**

**Fortunella_japonica_8** **accaagccatcgcccgaaacggaaagagagggattcgaaccctcggtacgaataactcgt**

**Fortunella_japonica_9** **accaagccatcgcccgaaacggaaagagagggattcgaaccctcggtacgaataactcgt**

**Fortunella_japonica_10** **accaagccatcgcccgaaacggaaagagagggattcgaaccctcggtacgaataactcgt**

**Fortunella_japonica_11** **accaagccatcgcccgaaacggaaagagagggattcgaaccctcggtacgaataactcgt**

**Fortunella_japonica_12** **accaagccatcgcccgaaacggaaagagagggattcgaaccctcggtacgaataactcgt**

**Fortunella_margarita_1** **accaagccatcgcccgaaacggaaagagagggattcgaaccctcggtacgaataactcgt**

**Fortunella_margarita_2** **accaagccatcgcccgaaacggaaagagagggattcgaaccctcggtacgaataactcgt**

**Fortunella_venosa_1** **accaagccatcgcccgaaacggaaagagagggattcgaaccctcggtacgaataactcgt**

**Fortunella_venosa_2** **accaagccatcgcccgaaacggaaagagagggattcgaaccctcggtacgaataactcgt**

**Fortunella_venosa_3** **accaagccatcgcccgaaacggaaagagagggattcgaaccctcggtacgaataactcgt**

9190	9200	9210	9220	9230	9240

....|....|....|....|....|....|....|....|....|....|....|....|

**Atalantia_kwangtungens** **acaccggattagcaatccgacgctttaatccactcagccatctctcctaattg------a**

**Citrus_reticulata_1** **acaccggattagcaatccgacgctttaatccactcagccatctctcctaattg-------**

**Citrus_reticulata_2** **acaccggattagcaatccgacgctttaatccactcagccatctctcctaattgaaaataa**

**Citrus_trifoliata_3** **acaccggattagcaatccgacgctttaatccactcagccatctctcctaattg------a**

**Citrus_aurantiifolia** **acaccggattagcaatccgacgctttaatccactcagccatctctcctaattg-------**

**Citrus_aurantium** **acaccggattagcaatccgacgctttaatccactcagccatctctcctaattg-------**

**Citrus_cavaleriei** **acaccggattagcaatccgacgctttaatccactcagccatctctcctaattg-------**

**Citrus_hongheensis** **acaccggattagcaatccgacgctttaatccactcagccatctctcctaattg-------**

**Citrus_limon** **acaccggattagcaatccgacgctttaatccactcagccatctctcctaattg-------**

**Citrus_maxima** **acaccggattagcaatccgacgctttaatccactcagccatctctcctaattg-------**

**Citrus_medica** **acaccggattagcaatccgacgctttaatccactcagccatctctcctaattg-----aa**

**Citrus_polytrifolia** **acaccggattagcaatccgacgctttaatccactcagccatctctcctaattg------a**

**Citrus_reticulata** **acaccggattagcaatccgacgctttaatccactcagccatctctcctaattgaaaataa**

**Citrus_sinensis** **acaccggattagcaatccgacgctttaatccactcagccatctctcctaattg-------**

**Fortunella_bawangica_1** **acaccggattagcaatccgacgctttaatccactcagccatctctcctaattga----aa**

**Fortunella_bawangica_2** **acaccggattagcaatccgacgctttaatccactcagccatctctcctaattga----aa**

**Fortunella_hindsii_1** **acaccggattagcaatccgacgctttaatccactcagccatctctcctaattg-------**

**Fortunella_hindsii_2** **acaccggattagcaatccgacgctttaatccactcagccatctctcctaattg------a**

**Fortunella_hindsii_3** **acaccggattagcaatccgacgctttaatccactcagccatctctcctaattg-------**

**Fortunella_japonica_1** **acaccggattagcaatccgacgctttaatccactcagccatctctcctaattg------a**

**Fortunella_japonica_2** **acaccggattagcaatccgacgctttaatccactcagccatctctcctaattg------a**

**Fortunella_japonica_3** **acaccggattagcaatccgacgctttaatccactcagccatctctcctaattg------a**

**Fortunella_japonica_4** **acaccggattagcaatccgacgctttaatccactcagccatctctcctaattg------a**

**Fortunella_japonica_5** **acaccggattagcaatccgacgctttaatccactcagccatctctcctaattg------a**

**Fortunella_japonica_6** **acaccggattagcaatccgacgctttaatccactcagccatctctcctaattg------a**

**Fortunella_japonica_7** **acaccggattagcaatccgacgctttaatccactcagccatctctcctaattg------a**

**Fortunella_japonica_8** **acaccggattagcaatccgacgctttaatccactcagccatctctcctaattg------a**

**Fortunella_japonica_9** **acaccggattagcaatccgacgctttaatccactcagccatctctcctaattg------a**

**Fortunella_japonica_10** **acaccggattagcaatccgacgctttaatccactcagccatctctcctaattg-------**

**Fortunella_japonica_11** **acaccggattagcaatccgacgctttaatccactcagccatctctcctaattg------a**

**Fortunella_japonica_12** **acaccggattagcaatccgacgctttaatccactcagccatctctcctaattg------a**

**Fortunella_margarita_1** **acaccggattagcaatccgacgctttaatccactcagccatctctcctaattg-------**

**Fortunella_margarita_2** **acaccggattagcaatccgacgctttaatccactcagccatctctcctaattg-------**

**Fortunella_venosa_1** **acaccggattagcaatccgacgctttaatccactcagccatctctcctaattg-------**

**Fortunella_venosa_2** **acaccggattagcaatccgacgctttaatccactcagccatctctcctaattg-------**

**Fortunella_venosa_3** **acaccggattagcaatccgacgctttaatccactcagccatctctcctaattg-------**

9250	9260	9270	9280	9290	9300

....|....|....|....|....|....|....|....|....|....|....|....|

**Atalantia_kwangtungens** **aaaaaa-------aaa-----aaaaataattactatgttacattactctcctaattgaaa**

**Citrus_reticulata_1** **aaaata-------aaa-aa-taaaaaata-------------------------------**

**Citrus_reticulata_2** **aaaata-------aaa-aa-taaaaaata-------------------------------**

**Citrus_trifoliata_3** **aaaaaaaaaaaagaaataa--aaaaaaaa-------------------------------**

**Citrus_aurantiifolia** **aaaata-------aaa-a-------aaaa-------------------------------**

**Citrus_aurantium** **aaaata-------aaa-aa-taaaaaaaa-------------------------------**

**Citrus_cavaleriei** **aaaata-------aaa-a---taaaaaaa-------------------------------**

**Citrus_hongheensis** **aaaata-------aaa--------aaaaa-------------------------------**

**Citrus_limon** **aaaata-------aaa-aa-taaaaaaaa-------------------------------**

**Citrus_maxima** **aaaata-------aaaaaa-taaaaaaaa-------------------------------**

**Citrus_medica** **aaaaaa---------------aaaaaaaa-------------------------------**

**Citrus_polytrifolia** **aaaaaaaaaaaaaaaagaaataaaaaaaa-------------------------------**

**Citrus_reticulata** **aaaata-------aaa-aa-taaaaaata-------------------------------**

**Citrus_sinensis** **aaaata-------aaa-----aaaaaaaa-------------------------------**

**Fortunella_bawangica_1** **aaaaaa-------aaa-----aaaaaaaattactatgttacattac--------------**

**Fortunella_bawangica_2** **aaaaaa-------aaa-----aaaaaaaattactatgttacattac--------------**

**Fortunella_hindsii_1** **-aaaaa-------aaa-----aaaaaaaattactatgttacattac--------------**

**Fortunella_hindsii_2** **aaaaaa-------aaa-----aaaaaaaattactatgttacattac--------------**

**Fortunella_hindsii_3** **aaaaaa-------aaa-----aaaaaaaattactatgttacattac--------------**

**Fortunella_japonica_1** **aaaaaaaaaaaaaaaa-----aaaaaaaattactatgttacattac--------------**

**Fortunella_japonica_2** **aaa----------aaa-----aaaaaaaattactatgttacattac--------------**

**Fortunella_japonica_3** **aaaaaaaaaaaaaaaa-----aaaaaaaattactatgttacattac--------------**

**Fortunella_japonica_4** **aaaaaaaaaaaaaaaa-----aaaaaaaattactatgttacattac--------------**

**Fortunella_japonica_5** **aaaaaaaaaaaaaaaa-----aaaaaaaattactatgttacattac--------------**

**Fortunella_japonica_6** **aaaaaaaaaaaaaaaa-----aaaaaaaattactatgttacattac--------------**

**Fortunella_japonica_7** **aaaaaaaaaaaaaaaa-----aaaaaaaattactatgttacattac--------------**

**Fortunella_japonica_8** **aaaaaaaaaaaaaaaa-----aaaaaaaattactatgttacattac--------------**

**Fortunella_japonica_9** **aaaaaaaaaaaaaaaa-----aaaaaaaattactatgttacattac--------------**

**Fortunella_japonica_10** **-aaaaa-------aaa-----aaaaaaaattactatgttacattac--------------**

**Fortunella_japonica_11** **aaaaaa-------aaa-----aaaaaaaattactatgttacattac--------------**

**Fortunella_japonica_12** **aaaaaaaaaaaaaaaa-----aaaaaaaattactatgttacattac--------------**

**Fortunella_margarita_1** **aaaaaa-------aaa-----aaaaaaaattactatgttacattac--------------**

**Fortunella_margarita_2** **aaaaaa-------aaa-----aaaaaaaattactatgttacattac--------------**

**Fortunella_venosa_1** **aaaaaa-------aaa-----aaaaaaaattactatgttacattac--------------**

**Fortunella_venosa_2** **aaaaaa-------aaa-----aaaaaaaattactatgttacattac--------------**

**Fortunella_venosa_3** **aaaaaa-------aaa-----aaaaaaaattactatgttacattac--------------**

9310	9320	9330	9340	9350	9360

....|....|....|....|....|....|....|....|....|....|....|....|

**Atalantia_kwangtungens** **aaaaaaaaaaaataattactatgttacattacacaaaaaatactgcttgaaaaagcccgt**

**Citrus_reticulata_1** **------------aaattactatgttacattacacaaaaaataatgcttgaaaaagcccgt**

**Citrus_reticulata_2** **------------aaattactatgttacattacacaaaaaataatgcttgaaaaagcccgt**

**Citrus_trifoliata_3** **------------aaaggactatgttacattacacaaaaaagaatgcttgaaaaagcccgt**

**Citrus_aurantiifolia** **------------aaatgactatgttacattacacaaaaaataatgcttgaaaaagcccgt**

**Citrus_aurantium** **------------aaatgactatgttacattacacaaaaaataatgcttgaaaaagcccgt**

**Citrus_cavaleriei** **------------aaattactatgttacattacacaaaaaataatgcttgaaaaagcccgt**

**Citrus_hongheensis** **------------aaatgactatgttacattacacaaaaaataatgcttgaaaaagcccgt**

**Citrus_limon** **------------aaatgactatgttacattacacaaaaaataatgcttgaaaaagcccgt**

**Citrus_maxima** **------------aaatgactatgttacattacacaaaaaataatgcttgaaaaagcccgt**

**Citrus_medica** **------------aaattactatgttacattacacaaaaaataatgcttgaaaaagcccgt**

**Citrus_polytrifolia** **------------aaaggactatgttacattacacaaaaaagaatgcttgaaaaagcccgt**

**Citrus_reticulata** **------------aaattactatgttacattacacaaaaaataatgcttgaaaaagcccgt**

**Citrus_sinensis** **------------aaatgactatgttacattacacaaaaaataatgcttgaaaaagcccgt**

**Fortunella_bawangica_1** **----------acaaattactatgttacattacacaaaaaataatgcttgaaaaagcccgt**

**Fortunella_bawangica_2** **----------acaaattactatgttacattacacaaaaaataatgcttgaaaaagcccgt**

**Fortunella_hindsii_1** **----------acaaattactatgttacattacacaaaaaataatgcttgaaaaagcccgt**

**Fortunella_hindsii_2** **----------acaaattactatgttacattacacaaaaaataatgcttgaaaaagcccgt**

**Fortunella_hindsii_3** **----------acaaattactatgttacattacacaaaaaataatgcttgaaaaagcccgt**

**Fortunella_japonica_1** **----------acaaattactatgttacattacacaaaaaataatgcttgaaaaagcccgt**

**Fortunella_japonica_2** **----------acaaattactatgttacattacacaaaaaataatgcttgaaaaagcccgt**

**Fortunella_japonica_3** **----------acaaattactatgttacattacacaaaaaataatgcttgaaaaagcccgt**

**Fortunella_japonica_4** **----------acaaattactatgttacattacacaaaaaataatgcttgaaaaagcccgt**

**Fortunella_japonica_5** **----------acaaattactatgttacattacacaaaaaataatgcttgaaaaagcccgt**

**Fortunella_japonica_6** **----------acaaattactatgttacattacacaaaaaataatgcttgaaaaagcccgt**

**Fortunella_japonica_7** **----------acaaattactatgttacattacacaaaaaataatgcttgaaaaagcccgt**

**Fortunella_japonica_8** **----------acaaattactatgttacattacacaaaaaataatgcttgaaaaagcccgt**

**Fortunella_japonica_9** **----------acaaattactatgttacattacacaaaaaataatgcttgaaaaagcccgt**

**Fortunella_japonica_10** **----------acaaattactatgttacattacacaaaaaataatgcttgaaaaagcccgt**

**Fortunella_japonica_11** **----------acaaattactatgttacattacacaaaaaataatgcttgaaaaagcccgt**

**Fortunella_japonica_12** **----------acaaattactatgttacattacacaaaaaataatgcttgaaaaagcccgt**

**Fortunella_margarita_1** **----------acaaattactatgttacattacacaaaaaataatgcttgaaaaagcccgt**

**Fortunella_margarita_2** **----------acaaattactatgttacattacacaaaaaataatgcttgaaaaagcccgt**

**Fortunella_venosa_1** **----------acaaattactatgttacattacacaaaaaataatgcttgaaaaagcccgt**

**Fortunella_venosa_2** **----------acaaattactatgttacattacacaaaaaataatgcttgaaaaagcccgt**

**Fortunella_venosa_3** **----------acaaattactatgttacattacacaaaaaataatgcttgaaaaagcccgt**

9370	9380	9390	9400	9410	9420

....|....|....|....|....|....|....|....|....|....|....|....|

**Atalantia_kwangtungens** **ctttacttgattttctatctttactttcgatattctcttctatattctctagaagagaat**

**Citrus_reticulata_1** **ctttacttgattttctatctttactttctatattctcttctagagaatatagaagagaat**

**Citrus_reticulata_2** **ctttacttgattttctatctttactttctatattctcttctagagaatatagaagagaat**

**Citrus_trifoliata_3** **ctttacttgattttctatctttactttctatattctcttct------------agagaat**

**Citrus_aurantiifolia** **ctttacttgattttctatctttactttctatattctcttctagagaatatagaagagaat**

**Citrus_aurantium** **ctttacttgattttctatctttact------------ttctatattctctagaagagaat**

**Citrus_cavaleriei** **ctttacttgattttctatctttactttctatattctcttctagagaatatagaagagaat**

**Citrus_hongheensis** **ctttacttgattttctatctttactttctatattctcttctagagaatatagaagagaat**

**Citrus_limon** **ctttacttgattttctatctttact------------ttctatattctctagaagagaat**

**Citrus_maxima** **ctttacttgattttctatctttactttctatattctcttctatattctctagaagagaat**

**Citrus_medica** **ctttacttgattttctatctttactttctatattctcttctatattctcttctagagaat**

**Citrus_polytrifolia** **ctttacttgattttctatctttactttctatattctcttct------------agagaat**

**Citrus_reticulata** **ctttacttgattttctatctttactttctatattctcttctagagaatatagaagagaat**

**Citrus_sinensis** **ctttacttgattttctatctttactttctatattctcttctatattctctagaagagaat**

**Fortunella_bawangica_1** **ctttacttgattttctatctttactttctatattctcttct------------agagaat**

**Fortunella_bawangica_2** **ctttacttgattttctatctttactttctatattctcttct------------agagaat**

**Fortunella_hindsii_1** **ctttacttgattttctatctttactttctatattctcttct------------agagaat**

**Fortunella_hindsii_2** **ctttacttgattttctatctttactttctatattctcttct------------agagaat**

**Fortunella_hindsii_3** **ctttacttgattttctatctttactttctatattctcttct------------agagaat**

**Fortunella_japonica_1** **ctttacttgattttctatctttactttctatattctcttct------------agagaat**

**Fortunella_japonica_2** **ctttacttgattttctatctttactttctatattctcttct------------agagaat**

**Fortunella_japonica_3** **ctttacttgattttctatctttactttctatattctcttct------------agagaat**

**Fortunella_japonica_4** **ctttacttgattttctatctttactttctatattctcttct------------agagaat**

**Fortunella_japonica_5** **ctttacttgattttctatctttactttctatattctcttct------------agagaat**

**Fortunella_japonica_6** **ctttacttgattttctatctttactttctatattctcttct------------agagaat**

**Fortunella_japonica_7** **ctttacttgattttctatctttactttctatattctcttct------------agagaat**

**Fortunella_japonica_8** **ctttacttgattttctatctttactttctatattctcttct------------agagaat**

**Fortunella_japonica_9** **ctttacttgattttctatctttactttctatattctcttct------------agagaat**

**Fortunella_japonica_10** **ctttacttgattttctatctttactttctatattctcttct------------agagaat**

**Fortunella_japonica_11** **ctttacttgattttctatctttactttctatattctcttct------------agagaat**

**Fortunella_japonica_12** **ctttacttgattttctatctttactttctatattctcttct------------agagaat**

**Fortunella_margarita_1** **ctttacttgattttctatctttactttctatattctcttct------------agagaat**

**Fortunella_margarita_2** **ctttacttgattttctatctttactttctatattctcttct------------agagaat**

**Fortunella_venosa_1** **ctttacttgattttctatctttactttctatattctcttct------------agagaat**

**Fortunella_venosa_2** **ctttacttgattttctatctttactttctatattctcttct------------agagaat**

**Fortunella_venosa_3** **ctttacttgattttctatctttactttctatattctcttct------------agagaat**

9430	9440	9450	9460	9470	9480

....|....|....|....|....|....|....|....|....|....|....|....|

**Atalantia_kwangtungens** **atcgaaagtaaattaattatatagctcatagtagctcatagaaaatatagcttatagaat**

**Citrus_reticulata_1** **atagaaagtaaattgattata----------tagctcatagaaaatatagcttatagaat**

**Citrus_reticulata_2** **atagaaagtaaattgattata----------tagctcatagaaaatatagcttatagaat**

**Citrus_trifoliata_3** **atagaaagtaaattgattata----------tagctcatagaaaacatagcttatagaat**

**Citrus_aurantiifolia** **atagaaagtaaattgattata----------tagctcatagaaaatatagcttatagaat**

**Citrus_aurantium** **atagaaagtaaattgattata----------tagctcatagaaaatatagcttatagaat**

**Citrus_cavaleriei** **atagaaagtaaattgattata----------tagctcatagaaaatatagcttatagaat**

**Citrus_hongheensis** **atagaaagtaaattgattata----------tagctcatagaaaatatagcttatagaat**

**Citrus_limon** **atagaaagtaaattgattata----------tagctcatagaaaatatagcttatagaat**

**Citrus_maxima** **atagaaagtaaattgattata----------tagctcatagaaaatatagcttatagaat**

**Citrus_medica** **atagaaagtaaattgattata----------tagctcatagaaaatatagcttatagaat**

**Citrus_polytrifolia** **atagaaagtaaattgattata----------tagctcatagaaaacatagcttatagaat**

**Citrus_reticulata** **atagaaagtaaattgattata----------tagctcatagaaaatatagcttatagaat**

**Citrus_sinensis** **atagaaagtaaattgattata----------tagctcatagaaaatatagcttatagaat**

**Fortunella_bawangica_1** **atagaaagtaaattgattata----------tagctcatagaaaatatagcttatagaat**

**Fortunella_bawangica_2** **atagaaagtaaattgattata----------tagctcatagaaaatatagcttatagaat**

**Fortunella_hindsii_1** **atagaaagtaaattgattata----------tagctcatagaaaatatagcttatagaat**

**Fortunella_hindsii_2** **atagaaagtaaattgattata----------tagctcatagaaaatatagcttatagaat**

**Fortunella_hindsii_3** **atagaaagtaaattgattata----------tagctcatagaaaatatagcttatagaat**

**Fortunella_japonica_1** **atagaaagtaaattgattata----------tagctcatagaaaatatagcttatagaat**

**Fortunella_japonica_2** **atagaaagtaaattgattata----------tagctcatagaaaatatagcttatagaat**

**Fortunella_japonica_3** **atagaaagtaaattgattata----------tagctcatagaaaatatagcttatagaat**

**Fortunella_japonica_4** **atagaaagtaaattgattata----------tagctcatagaaaatatagcttatagaat**

**Fortunella_japonica_5** **atagaaagtaaattgattata----------tagctcatagaaaatatagcttatagaat**

**Fortunella_japonica_6** **atagaaagtaaattgattata----------tagctcatagaaaatatagcttatagaat**

**Fortunella_japonica_7** **atagaaagtaaattgattata----------tagctcatagaaaatatagcttatagaat**

**Fortunella_japonica_8** **atagaaagtaaattgattata----------tagctcatagaaaatatagcttatagaat**

**Fortunella_japonica_9** **atagaaagtaaattgattata----------tagctcatagaaaatatagcttatagaat**

**Fortunella_japonica_10** **atagaaagtaaattgattata----------tagctcatagaaaatatagcttatagaat**

**Fortunella_japonica_11** **atagaaagtaaattgattata----------tagctcatagaaaatatagcttatagaat**

**Fortunella_japonica_12** **atagaaagtaaattgattata----------tagctcatagaaaatatagcttatagaat**

**Fortunella_margarita_1** **atagaaagtaaattgattata----------tagctcatagaaaatatagcttatagaat**

**Fortunella_margarita_2** **atagaaagtaaattgattata----------tagctcatagaaaatatagcttatagaat**

**Fortunella_venosa_1** **atagaaagtaaattgattata----------tagctcatagaaaatatagcttatagaat**

**Fortunella_venosa_2** **atagaaagtaaattgattata----------tagctcatagaaaatatagcttatagaat**

**Fortunella_venosa_3** **atagaaagtaaattgattata----------tagctcatagaaaatatagcttatagaat**

9490	9500	9510	9520	9530	9540

....|....|....|....|....|....|....|....|....|....|....|....|

**Atalantia_kwangtungens** **tttagaatttcttttttttttattgtcttttgtattctattatataaatagatataactt**

**Citrus_reticulata_1** **ttc----------ttttttttattgtcttttgtattctattatataaatagatataactt**

**Citrus_reticulata_2** **ttc----------ttttttttattgtcttttgtattctattatataaatagatataactt**

**Citrus_trifoliata_3** **ttc----------ttttttttattgtcttttgtattctattatataaatagatataactt**

**Citrus_aurantiifolia** **ttc----------ttttttttattgtcttttgtattctattatataaatagatataactt**

**Citrus_aurantium** **ttct--------tttttttttattgtcttttgtattctattatataaatagatataactt**

**Citrus_cavaleriei** **ttc----------ttttttttattgtcttttgtattctattatataaatagatataactt**

**Citrus_hongheensis** **ttc----------ttttttttattgtcttttgtattctattatataaatagatataactt**

**Citrus_limon** **ttct--------tttttttttattgtcttttgtattctattatataaatagatataactt**

**Citrus_maxima** **ttc---------tttttttttattgtcttttgtattctattatataaatagatataactt**

**Citrus_medica** **ttc----------ttttttttattgtcttttgtattctattatataaatagatataactt**

**Citrus_polytrifolia** **ttc----------ttttttttattgtcttttgtattctattatataaatagatataactt**

**Citrus_reticulata** **ttc----------ttttttttattgtcttttgtattctattatataaatagatataactt**

**Citrus_sinensis** **ttc---------tttttttttattgtcttttgtattctattatataaatagatataactt**

**Fortunella_bawangica_1** **tta----------ttttttttattgtcttttgtattctattatataaatagatataactt**

**Fortunella_bawangica_2** **tta----------ttttttttattgtcttttgtattctattatataaatagatataactt**

**Fortunella_hindsii_1** **tta----------ttttttttattgtcttttgtattctattatataaatagatataactt**

**Fortunella_hindsii_2** **tta----------ttttttttattgtcttttgtattctattatataaatagatataactt**

**Fortunella_hindsii_3** **tta----------ttttttttattgtcttttgtattctattatataaatagatataactt**

**Fortunella_japonica_1** **tta----------ttttttttattgtcttttgtattctattatataaatagatataactt**

**Fortunella_japonica_2** **tta----------ttttttttattgtcttttgtattctattatataaatagatataactt**

**Fortunella_japonica_3** **tta----------ttttttttattgtcttttgtattctattatataaatagatataactt**

**Fortunella_japonica_4** **tta----------ttttttttattgtcttttgtattctattatataaatagatataactt**

**Fortunella_japonica_5** **tta----------ttttttttattgtcttttgtattctattatataaatagatataactt**

**Fortunella_japonica_6** **tta----------ttttttttattgtcttttgtattctattatataaatagatataactt**

**Fortunella_japonica_7** **tta----------ttttttttattgtcttttgtattctattatataaatagatataactt**

**Fortunella_japonica_8** **tta----------ttttttttattgtcttttgtattctattatataaatagatataactt**

**Fortunella_japonica_9** **tta----------ttttttttattgtcttttgtattctattatataaatagatataactt**

**Fortunella_japonica_10** **tta----------ttttttttattgtcttttgtattctattatataaatagatataactt**

**Fortunella_japonica_11** **tta----------ttttttttattgtcttttgtattctattatataaatagatataactt**

**Fortunella_japonica_12** **tta----------ttttttttattgtcttttgtattctattatataaatagatataactt**

**Fortunella_margarita_1** **tta----------ttttttttattgtcttttgtattctattatataaatagatataactt**

**Fortunella_margarita_2** **tta----------ttttttttattgtcttttgtattctattatataaatagatataactt**

**Fortunella_venosa_1** **tta----------ttttttttattgtcttttgtattctattatataaatagatataactt**

**Fortunella_venosa_2** **tta----------ttttttttattgtcttttgtattctattatataaatagatataactt**

**Fortunella_venosa_3** **tta----------ttttttttattgtcttttgtattctattatataaatagatataactt**

9550	9560	9570	9580	9590	9600

....|....|....|....|....|....|....|....|....|....|....|....|

**Atalantia_kwangtungens** **ttatcagcaattccatttcgatcatttttagaaaaaaaaaaaataaagaaagcattcgaa**

**Citrus_reticulata_1** **ttatcagcaattccatttctatcatttttag--aaaattaaaataaagaaagtattcgaa**

**Citrus_reticulata_2** **ttatcagcaattccatttctatcatttttag--aaaattaaaataaagaaagtattcgaa**

**Citrus_trifoliata_3** **ttatcagcaattccatttctatcatttttag--aaaattaaaataaagaaagtattcgaa**

**Citrus_aurantiifolia** **ttatcagcaattccatttctatcatttttcg--aaaattaaaataaagaaagtattcgaa**

**Citrus_aurantium** **ttatcagcaattccatttctatcatttttcg--aaaattaaaataaagaaagtattcgaa**

**Citrus_cavaleriei** **ttatcagcaattccatttctatcatttttag--aaaattaaaataaagaaagtattcgaa**

**Citrus_hongheensis** **ttatcagcaattccatttctatcatttttcg--aaaattaaaataaagaaagtattcgaa**

**Citrus_limon** **ttatcagcaattccatttctatcatttttcg--aaaattaaaataaagaaagtattcgaa**

**Citrus_maxima** **ttatcagcaattccatttctatcatttttcg--aaaattaaaataaagaaagtattcgaa**

**Citrus_medica** **ttatcagcaattccatttctatcatttttag--aaaatgcaaataaagaaagtattcgaa**

**Citrus_polytrifolia** **ttatcagcaattccatttctatcatttttag--aaaattaaaataaagaaagtattcgaa**

**Citrus_reticulata** **ttatcagcaattccatttctatcatttttag--aaaattaaaataaagaaagtattcgaa**

**Citrus_sinensis** **ttatcagcaattccatttctatcatttttcg--aaaattaaaataaagaaagtattcgaa**

**Fortunella_bawangica_1** **ttatcagcaattccatttctatcatttttag--aaaattaaaataaagaaagtattcgaa**

**Fortunella_bawangica_2** **ttatcagcaattccatttctatcatttttag--aaaattaaaataaagaaagtattcgaa**

**Fortunella_hindsii_1** **ttatcagcaattccatttctatcatttttag--aaaattaaaataaagaaagtattcgaa**

**Fortunella_hindsii_2** **ttatcagcaattccatttctatcatttttag--aaaattaaaataaagaaagtattcgaa**

**Fortunella_hindsii_3** **ttatcagcaattccatttctatcatttttag--aaaattaaaataaagaaagtattcgaa**

**Fortunella_japonica_1** **ttatcagcaattccatttctatcatttttag--aaaattaaaataaagaaagtattcgaa**

**Fortunella_japonica_2** **ttatcagcaattccatttctatcatttttag--aaaattaaaataaagaaagtattcgaa**

**Fortunella_japonica_3** **ttatcagcaattccatttctatcatttttag--aaaattaaaataaagaaagtattcgaa**

**Fortunella_japonica_4** **ttatcagcaattccatttctatcatttttag--aaaattaaaataaagaaagtattcgaa**

**Fortunella_japonica_5** **ttatcagcaattccatttctatcatttttag--aaaattaaaataaagaaagtattcgaa**

**Fortunella_japonica_6** **ttatcagcaattccatttctatcatttttag--aaaattaaaataaagaaagtattcgaa**

**Fortunella_japonica_7** **ttatcagcaattccatttctatcatttttag--aaaattaaaataaagaaagtattcgaa**

**Fortunella_japonica_8** **ttatcagcaattccatttctatcatttttag--aaaattaaaataaagaaagtattcgaa**

**Fortunella_japonica_9** **ttatcagcaattccatttctatcatttttag--aaaattaaaataaagaaagtattcgaa**

**Fortunella_japonica_10** **ttatcagcaattccatttctatcatttttag--aaaattaaaataaagaaagtattcgaa**

**Fortunella_japonica_11** **ttatcagcaattccatttctatcatttttag--aaaattaaaataaagaaagtattcgaa**

**Fortunella_japonica_12** **ttatcagcaattccatttctatcatttttag--aaaattaaaataaagaaagtattcgaa**

**Fortunella_margarita_1** **ttatcagcaattccatttctatcatttttag--aaaattaaaataaagaaagtattcgaa**

**Fortunella_margarita_2** **ttatcagcaattccatttctatcatttttag--aaaattaaaataaagaaagtattcgaa**

**Fortunella_venosa_1** **ttatcagcaattccatttctatcatttttag--aaaattaaaataaagaaagtattcgaa**

**Fortunella_venosa_2** **ttatcagcaattccatttctatcatttttag--aaaattaaaataaagaaagtattcgaa**

**Fortunella_venosa_3** **ttatcagcaattccatttctatcatttttag--aaaattaaaataaagaaagtattcgaa**

9610	9620	9630	9640	9650	9660

....|....|....|....|....|....|....|....|....|....|....|....|

**Atalantia_kwangtungens** **agagataaaagag--aaaaaaataaaga-----atatctctttaatttcattcaacaagg**

**Citrus_reticulata_1** **agagataaaatag--aaaaaaataaagaaaagaatatctctttaatttcgttcaacgggg**

**Citrus_reticulata_2** **agagataaaatag--aaaaaaataaagaaaagaatatctctttaatttcgttcaacgggg**

**Citrus_trifoliata_3** **agagataaaatag--aaaaaaataaagaaaagaatatctctttaatttcgttcaacgggg**

**Citrus_aurantiifolia** **agagataaaatagaaaaaaaaataaagaaaagaatatctctttaatttcgttcaacgggg**

**Citrus_aurantium** **agagataaaatag--aaaaaaataaagaaaagaatatctctttaatttcgttcaacgggg**

**Citrus_cavaleriei** **agagataaaatag--aaaaaaataaagaaaagaatatctctttaatttcgttcaacgggg**

**Citrus_hongheensis** **agagataaaatag--aaaaaaataaagaaaagaatatctctttaatttcgttcaacgggg**

**Citrus_limon** **agagataaaatag--aaaaaaataaagaaaagaatatctctttaatttcgttcaacgggg**

**Citrus_maxima** **agagataaaatag--aaaaaaataaagaaaagaatatctctttaatttcgttcaacgggg**

**Citrus_medica** **agagataaaatag--aaaaaaataaagaaaagaatatctctttaatttcgttcaacgggg**

**Citrus_polytrifolia** **agagataaaatag--aaaaaaataaagaaaagaatatctctttaatttcgttcaacgggg**

**Citrus_reticulata** **agagataaaatag--aaaaaaataaagaaaagaatatctctttaatttcgttcaacgggg**

**Citrus_sinensis** **agagataaaatag--aaaaaaataaagaaaagaatatctctttaatttcgttcaacgggg**

**Fortunella_bawangica_1** **agaaataaaatag--aaaaaaataaagaaaagaatatctctttaatttcgttcaacgggg**

**Fortunella_bawangica_2** **agaaataaaatag--aaaaaaataaagaaaagaatatctctttaatttcgttcaacgggg**

**Fortunella_hindsii_1** **agaaataaaatag--aaaaaaataaagaaaagaatatctctttaatttcgttcaacgggg**

**Fortunella_hindsii_2** **agaaataaaatag--aaaaaaataaagaaaagaatatctctttaatttcgttcaacgggg**

**Fortunella_hindsii_3** **agaaataaaatag--aaaaaaataaagaaaagaatatctctttaatttcgttcaacgggg**

**Fortunella_japonica_1** **agaaataaaatag--aaaaaaataaagaaaagaatatctctttaatttcgttcaacgggg**

**Fortunella_japonica_2** **agaaataaaatag--aaaaaaataaagaaaagaatatctctttaatttcgttcaacgggg**

**Fortunella_japonica_3** **agaaataaaatag--aaaaaaataaagaaaagaatatctctttaatttcgttcaacgggg**

**Fortunella_japonica_4** **agaaataaaatag--aaaaaaataaagaaaagaatatctctttaatttcgttcaacgggg**

**Fortunella_japonica_5** **agaaataaaatag--aaaaaaataaagaaaagaatatctctttaatttcgttcaacgggg**

**Fortunella_japonica_6** **agaaataaaatag--aaaaaaataaagaaaagaatatctctttaatttcgttcaacgggg**

**Fortunella_japonica_7** **agaaataaaatag--aaaaaaataaagaaaagaatatctctttaatttcgttcaacgggg**

**Fortunella_japonica_8** **agaaataaaatag--aaaaaaataaagaaaagaatatctctttaatttcgttcaacgggg**

**Fortunella_japonica_9** **agaaataaaatag--aaaaaaataaagaaaagaatatctctttaatttcgttcaacgggg**

**Fortunella_japonica_10** **agaaataaaatag--aaaaaaataaagaaaagaatatctctttaatttcgttcaacgggg**

**Fortunella_japonica_11** **agaaataaaatag--aaaaaaataaagaaaagaatatctctttaatttcgttcaacgggg**

**Fortunella_japonica_12** **agaaataaaatag--aaaaaaataaagaaaagaatatctctttaatttcgttcaacgggg**

**Fortunella_margarita_1** **agaaataaaatag--aaaaaaataaagaaaagaatatctctttaatttcgttcaacgggg**

**Fortunella_margarita_2** **agaaataaaatag--aaaaaaataaagaaaagaatatctctttaatttcgttcaacgggg**

**Fortunella_venosa_1** **agaaataaaatag--aaaaaaataaagaaaagaatatctctttaatttcgttcaacgggg**

**Fortunella_venosa_2** **agaaataaaatag--aaaaaaataaagaaaagaatatctctttaatttcgttcaacgggg**

**Fortunella_venosa_3** **agaaataaaatag--aaaaaaataaagaaaagaatatctctttaatttcgttcaacgggg**

9670	9680	9690	9700	9710	9720

....|....|....|....|....|....|....|....|....|....|....|....|

**Atalantia_kwangtungens** **ccttttttatttccacttccacggcctggcctggtcagtacccagccgggcctttttttg**

**Citrus_reticulata_1** **ccttttttatttccacttccacggcctggcctggtcagtacccagccgggcctttttttg**

**Citrus_reticulata_2** **ccttttttatttccacttccacggcctggcctggtcagtacccagccgggcctttttttg**

**Citrus_trifoliata_3** **ccttttttatttccacttccacggcctggcctggtcagtacccagccgggcctttttttg**

**Citrus_aurantiifolia** **ccttttttatttccacttccacggcctggcctggtcagtacccagccgggcctttttttg**

**Citrus_aurantium** **ccttttttatttccacttccacggcctggcctggtcagtacccagccgggcctttttttg**

**Citrus_cavaleriei** **ccctttttatttccacttccacggcctggcctggtcagtacccagccgggcctttttttg**

**Citrus_hongheensis** **ccttttttatttccacttccacggcctggcctggtcagtacccagccgggcctttttttg**

**Citrus_limon** **ccttttttatttccacttccacggcctggcctggtcagtacccagccgggcctttttttg**

**Citrus_maxima** **ccttttttatttccacttccacggcctggcctggtcagtacccagccgggcctttttttg**

**Citrus_medica** **ccttttttatttccacttccacggcctggcctggtcagtacccagccgggcctttttttg**

**Citrus_polytrifolia** **ccttttttatttccacttccacggcctggcctggtcagtacccagccgggcctttttttg**

**Citrus_reticulata** **ccttttttatttccacttccacggcctggcctggtcagtacccagccgggcctttttttg**

**Citrus_sinensis** **ccttttttatttccacttccacggcctggcctggtcagtacccagccgggcctttttttg**

**Fortunella_bawangica_1** **ccttttttatttccacttccacggcctggcctggtcagtacccagccgggcctttttttg**

**Fortunella_bawangica_2** **ccttttttatttccacttccacggcctggcctggtcagtacccagccgggcctttttttg**

**Fortunella_hindsii_1** **ccttttttatttccacttccacggcctggcctggtcagtacccagccgggcctttttttg**

**Fortunella_hindsii_2** **ccttttttatttccacttccacggcctggcctggtcagtacccagccgggcctttttttg**

**Fortunella_hindsii_3** **ccttttttatttccacttccacggcctggcctggtcagtacccagccgggcctttttttg**

**Fortunella_japonica_1** **ccttttttatttccacttccacggcctggcctggtcagtacccagccgggcctttttttg**

**Fortunella_japonica_2** **ccttttttatttccacttccacggcctggcctggtcagtacccagccgggcctttttttg**

**Fortunella_japonica_3** **ccttttttatttccacttccacggcctggcctggtcagtacccagccgggcctttttttg**

**Fortunella_japonica_4** **ccttttttatttccacttccacggcctggcctggtcagtacccagccgggcctttttttg**

**Fortunella_japonica_5** **ccttttttatttccacttccacggcctggcctggtcagtacccagccgggcctttttttg**

**Fortunella_japonica_6** **ccttttttatttccacttccacggcctggcctggtcagtacccagccgggcctttttttg**

**Fortunella_japonica_7** **ccttttttatttccacttccacggcctggcctggtcagtacccagccgggcctttttttg**

**Fortunella_japonica_8** **ccttttttatttccacttccacggcctggcctggtcagtacccagccgggcctttttttg**

**Fortunella_japonica_9** **ccttttttatttccacttccacggcctggcctggtcagtacccagccgggcctttttttg**

**Fortunella_japonica_10** **ccttttttatttccacttccacggcctggcctggtcagtacccagccgggcctttttttg**

**Fortunella_japonica_11** **ccttttttatttccacttccacggcctggcctggtcagtacccagccgggcctttttttg**

**Fortunella_japonica_12** **ccttttttatttccacttccacggcctggcctggtcagtacccagccgggcctttttttg**

**Fortunella_margarita_1** **ccttttttatttccacttccacggcctggcctggtcagtacccagccgggcctttttttg**

**Fortunella_margarita_2** **ccttttttatttccacttccacggcctggcctggtcagtacccagccgggcctttttttg**

**Fortunella_venosa_1** **ccttttttatttccacttccacggcctggcctggtcagtacccagccgggcctttttttg**

**Fortunella_venosa_2** **ccttttttatttccacttccacggcctggcctggtcagtacccagccgggcctttttttg**

**Fortunella_venosa_3** **ccttttttatttccacttccacggcctggcctggtcagtacccagccgggcctttttttg**

9730	9740	9750	9760	9770	9780

....|....|....|....|....|....|....|....|....|....|....|....|

**Atalantia_kwangtungens** **ttctaatgacccataccgctgaaccgtagaaagggcgcgaaccataccataaaaaaacga**

**Citrus_reticulata_1** **ttctaatgacccataccgctgaaccgtagaaagggtgcgaaccataccataaaaaaacga**

**Citrus_reticulata_2** **ttctaatgacccataccgctgaaccgtagaaagggtgcgaaccataccataaaaaaacga**

**Citrus_trifoliata_3** **ttctaatgacccataccgctgaaccgtagaaagggtgcgaaccataccataaaaaaacga**

**Citrus_aurantiifolia** **ttctaatgacccataccgctgaaccgtagaaagggtgcgaaccataccataaaaaaatga**

**Citrus_aurantium** **ttctaatgacccataccgctgaaccgtagaaagggtgcgaaccataccataaaaaaacga**

**Citrus_cavaleriei** **ttctaatgacccataccgctgaaccgtagaaagggtgcgaaccataccataaaaaaacga**

**Citrus_hongheensis** **ttctaatgacccataccgctgaaccgtagaaagggtgcgaaccataccataaaaaaacga**

**Citrus_limon** **ttctaatgacccataccgctgaaccgtagaaagggtgcgaaccataccataaaaaaacga**

**Citrus_maxima** **ttctaatgacccataccgctgaaccgtagaaagggtgcgaaccataccataaaaaaacga**

**Citrus_medica** **ttctaatgacccataccgctgaaccgtagaaagggtgcgaaccataccataaaaaaacga**

**Citrus_polytrifolia** **ttctaatgacccataccgctgaaccgtagaaagggtgcgaaccataccataaaaaaacga**

**Citrus_reticulata** **ttctaatgacccataccgctgaaccgtagaaagggtgcgaaccataccataaaaaaacga**

**Citrus_sinensis** **ttctaatgacccataccgctgaaccgtagaaagggtgcgaaccataccataaaaaaacga**

**Fortunella_bawangica_1** **ttctaatgacccataccgctgaaccgtagaaagggtgcgaaccataccataaaaaaacga**

**Fortunella_bawangica_2** **ttctaatgacccataccgctgaaccgtagaaagggtgcgaaccataccataaaaaaacga**

**Fortunella_hindsii_1** **ttctaatgacccataccgctgaaccgtagaaagggtgcgaaccataccataaaaaaacga**

**Fortunella_hindsii_2** **ttctaatgacccataccgctgaaccgtagaaagggtgcgaaccataccataaaaaaacga**

**Fortunella_hindsii_3** **ttctaatgacccataccgctgaaccgtagaaagggtgcgaaccataccataaaaaaacga**

**Fortunella_japonica_1** **ttctaatgacccataccgctgaaccgtagaaagggtgcgaaccataccataaaaaaacga**

**Fortunella_japonica_2** **ttctaatgacccataccgctgaaccgtagaaagggtgcgaaccataccataaaaaaacga**

**Fortunella_japonica_3** **ttctaatgacccataccgctgaaccgtagaaagggtgcgaaccataccataaaaaaacga**

**Fortunella_japonica_4** **ttctaatgacccataccgctgaaccgtagaaagggtgcgaaccataccataaaaaaacga**

**Fortunella_japonica_5** **ttctaatgacccataccgctgaaccgtagaaagggtgcgaaccataccataaaaaaacga**

**Fortunella_japonica_6** **ttctaatgacccataccgctgaaccgtagaaagggtgcgaaccataccataaaaaaacga**

**Fortunella_japonica_7** **ttctaatgacccataccgctgaaccgtagaaagggtgcgaaccataccataaaaaaacga**

**Fortunella_japonica_8** **ttctaatgacccataccgctgaaccgtagaaagggtgcgaaccataccataaaaaaacga**

**Fortunella_japonica_9** **ttctaatgacccataccgctgaaccgtagaaagggtgcgaaccataccataaaaaaacga**

**Fortunella_japonica_10** **ttctaatgacccataccgctgaaccgtagaaagggtgcgaaccataccataaaaaaacga**

**Fortunella_japonica_11** **ttctaatgacccataccgctgaaccgtagaaagggtgcgaaccataccataaaaaaacga**

**Fortunella_japonica_12** **ttctaatgacccataccgctgaaccgtagaaagggtgcgaaccataccataaaaaaacga**

**Fortunella_margarita_1** **ttctaatgacccataccgctgaaccgtagaaagggtgcgaaccataccataaaaaaacga**

**Fortunella_margarita_2** **ttctaatgacccataccgctgaaccgtagaaagggtgcgaaccataccataaaaaaacga**

**Fortunella_venosa_1** **ttctaatgacccataccgctgaaccgtagaaagggtgcgaaccataccataaaaaaacga**

**Fortunella_venosa_2** **ttctaatgacccataccgctgaaccgtagaaagggtgcgaaccataccataaaaaaacga**

**Fortunella_venosa_3** **ttctaatgacccataccgctgaaccgtagaaagggtgcgaaccataccataaaaaaacga**

9790	9800	9810	9820	9830	9840

....|....|....|....|....|....|....|....|....|....|....|....|

**Atalantia_kwangtungens** **tttatttgaatttgatttgaaaaccaaaaaatgaaatatttgttattgtattcaaaggaa**

**Citrus_reticulata_1** **tttatttgaatttgatttgaaaaccacaaaatgaaatacttgttattgtattcaaagg--**

**Citrus_reticulata_2** **tttatttgaatttgatttgaaaaccacaaaatgaaatacttgttattgtattcaaagg--**

**Citrus_trifoliata_3** **tttattttaatttgatttgaaaaccacaaaatgaaatacttgttattgtattcaaagg--**

**Citrus_aurantiifolia** **tttatttgaatttgatttgaaaaccacaaaatgaaatccttgttattgtattcaaagg--**

**Citrus_aurantium** **tttatttgaatttgatttgaaaaccacaaaatgaaatccttgttattgtattcaaagg--**

**Citrus_cavaleriei** **tttatttgaatttgatttgaaaaccacaaaatgaaatacttgttattgtattcaaagg--**

**Citrus_hongheensis** **tttatttgaatttgatttgaaaaccacaaaatgaaatccttgttattgtattcaaagg--**

**Citrus_limon** **tttatttgaatttgatttgaaaaccacaaaatgaaatccttgttattgtattcaaagg--**

**Citrus_maxima** **tttatttgaatttgatttgaaaaccacaaaatgaaatccttgttattgtattcaaagg--**

**Citrus_medica** **tttatttgaatttgatttgaaaaccacaaaatgaaatacttgttattgtattcaaagg--**

**Citrus_polytrifolia** **tttattttaatttgatttgaaaaccacaaaatgaaatacttgttattgtattcaaagg--**

**Citrus_reticulata** **tttatttgaatttgatttgaaaaccacaaaatgaaatacttgttattgtattcaaagg--**

**Citrus_sinensis** **tttatttgaatttgatttgaaaaccacaaaatgaaatccttgttattgtattcaaagg--**

**Fortunella_bawangica_1** **tttatttgaatttgatttgaaaaccacaaaatgaaatacttgttattgtattcaaagg--**

**Fortunella_bawangica_2** **tttatttgaatttgatttgaaaaccacaaaatgaaatacttgttattgtattcaaagg--**

**Fortunella_hindsii_1** **tttatttgaatttgatttgaaaaccacaaaatgaaatacttgttattgtattcaaagg--**

**Fortunella_hindsii_2** **tttatttgaatttgatttgaaaaccacaaaatgaaatacttgttattgtattcaaagg--**

**Fortunella_hindsii_3** **tttatttgaatttgatttgaaaaccacaaaatgaaatacttgttattgtattcaaagg--**

**Fortunella_japonica_1** **tttatttgaatttgatttgaaaaccacaaaatgaaatacttgttattgtattcaaagg--**

**Fortunella_japonica_2** **tttatttgaatttgatttgaaaaccacaaaatgaaatacttgttattgtattcaaagg--**

**Fortunella_japonica_3** **tttatttgaatttgatttgaaaaccacaaaatgaaatacttgttattgtattcaaagg--**

**Fortunella_japonica_4** **tttatttgaatttgatttgaaaaccacaaaatgaaatacttgttattgtattcaaagg--**

**Fortunella_japonica_5** **tttatttgaatttgatttgaaaaccacaaaatgaaatacttgttattgtattcaaagg--**

**Fortunella_japonica_6** **tttatttgaatttgatttgaaaaccacaaaatgaaatacttgttattgtattcaaagg--**

**Fortunella_japonica_7** **tttatttgaatttgatttgaaaaccacaaaatgaaatacttgttattgtattcaaagg--**

**Fortunella_japonica_8** **tttatttgaatttgatttgaaaaccacaaaatgaaatacttgttattgtattcaaagg--**

**Fortunella_japonica_9** **tttatttgaatttgatttgaaaaccacaaaatgaaatacttgttattgtattcaaagg--**

**Fortunella_japonica_10** **tttatttgaatttgatttgaaaaccacaaaatgaaatacttgttattgtattcaaagg--**

**Fortunella_japonica_11** **tttatttgaatttgatttgaaaaccacaaaatgaaatacttgttattgtattcaaagg--**

**Fortunella_japonica_12** **tttatttgaatttgatttgaaaaccacaaaatgaaatacttgttattgtattcaaagg--**

**Fortunella_margarita_1** **tttatttgaatttgatttgaaaaccacaaaatgaaatacttgttattgtattcaaagg--**

**Fortunella_margarita_2** **tttatttgaatttgatttgaaaaccacaaaatgaaatacttgttattgtattcaaagg--**

**Fortunella_venosa_1** **tttatttgaatttgatttgaaaaccacaaaatgaaatacttgttattgtattcaaagg--**

**Fortunella_venosa_2** **tttatttgaatttgatttgaaaaccacaaaatgaaatacttgttattgtattcaaagg--**

**Fortunella_venosa_3** **tttatttgaatttgatttgaaaaccacaaaatgaaatacttgttattgtattcaaagg--**

9850	9860	9870	9880	9890	9900

....|....|....|....|....|....|....|....|....|....|....|....|

**Atalantia_kwangtungens** **caaaatatttgttattggattcaaaggaacaataaaaagaaggactatttccattctttt**

**Citrus_reticulata_1** **------------------------------------------------------------**

**Citrus_reticulata_2** **------------------------------------------------------------**

**Citrus_trifoliata_3** **------------------------------------------------------------**

**Citrus_aurantiifolia** **------------------------------------------------------------**

**Citrus_aurantium** **------------------------------------------------------------**

**Citrus_cavaleriei** **------------------------------------------------------------**

**Citrus_hongheensis** **------------------------------------------------------------**

**Citrus_limon** **------------------------------------------------------------**

**Citrus_maxima** **------------------------------------------------------------**

**Citrus_medica** **------------------------------------------------------------**

**Citrus_polytrifolia** **------------------------------------------------------------**

**Citrus_reticulata** **------------------------------------------------------------**

**Citrus_sinensis** **------------------------------------------------------------**

**Fortunella_bawangica_1** **------------------------------------------------------------**

**Fortunella_bawangica_2** **------------------------------------------------------------**

**Fortunella_hindsii_1** **------------------------------------------------------------**

**Fortunella_hindsii_2** **------------------------------------------------------------**

**Fortunella_hindsii_3** **------------------------------------------------------------**

**Fortunella_japonica_1** **------------------------------------------------------------**

**Fortunella_japonica_2** **------------------------------------------------------------**

**Fortunella_japonica_3** **------------------------------------------------------------**

**Fortunella_japonica_4** **------------------------------------------------------------**

**Fortunella_japonica_5** **------------------------------------------------------------**

**Fortunella_japonica_6** **------------------------------------------------------------**

**Fortunella_japonica_7** **------------------------------------------------------------**

**Fortunella_japonica_8** **------------------------------------------------------------**

**Fortunella_japonica_9** **------------------------------------------------------------**

**Fortunella_japonica_10** **------------------------------------------------------------**

**Fortunella_japonica_11** **------------------------------------------------------------**

**Fortunella_japonica_12** **------------------------------------------------------------**

**Fortunella_margarita_1** **------------------------------------------------------------**

**Fortunella_margarita_2** **------------------------------------------------------------**

**Fortunella_venosa_1** **------------------------------------------------------------**

**Fortunella_venosa_2** **------------------------------------------------------------**

**Fortunella_venosa_3** **------------------------------------------------------------**

9910	9920	9930	9940	9950	9960

....|....|....|....|....|....|....|....|....|....|....|....|

**Atalantia_kwangtungens** **gattgaatcaggaatcaaagtttgtatttggtgtgtggataaaagttattggattcaaag**

**Citrus_reticulata_1** **------------------------------------------------------------**

**Citrus_reticulata_2** **------------------------------------------------------------**

**Citrus_trifoliata_3** **------------------------------------------------------------**

**Citrus_aurantiifolia** **------------------------------------------------------------**

**Citrus_aurantium** **------------------------------------------------------------**

**Citrus_cavaleriei** **------------------------------------------------------------**

**Citrus_hongheensis** **------------------------------------------------------------**

**Citrus_limon** **------------------------------------------------------------**

**Citrus_maxima** **------------------------------------------------------------**

**Citrus_medica** **------------------------------------------------------------**

**Citrus_polytrifolia** **------------------------------------------------------------**

**Citrus_reticulata** **------------------------------------------------------------**

**Citrus_sinensis** **------------------------------------------------------------**

**Fortunella_bawangica_1** **------------------------------------------------------------**

**Fortunella_bawangica_2** **------------------------------------------------------------**

**Fortunella_hindsii_1** **------------------------------------------------------------**

**Fortunella_hindsii_2** **------------------------------------------------------------**

**Fortunella_hindsii_3** **------------------------------------------------------------**

**Fortunella_japonica_1** **------------------------------------------------------------**

**Fortunella_japonica_2** **------------------------------------------------------------**

**Fortunella_japonica_3** **------------------------------------------------------------**

**Fortunella_japonica_4** **------------------------------------------------------------**

**Fortunella_japonica_5** **------------------------------------------------------------**

**Fortunella_japonica_6** **------------------------------------------------------------**

**Fortunella_japonica_7** **------------------------------------------------------------**

**Fortunella_japonica_8** **------------------------------------------------------------**

**Fortunella_japonica_9** **------------------------------------------------------------**

**Fortunella_japonica_10** **------------------------------------------------------------**

**Fortunella_japonica_11** **------------------------------------------------------------**

**Fortunella_japonica_12** **------------------------------------------------------------**

**Fortunella_margarita_1** **------------------------------------------------------------**

**Fortunella_margarita_2** **------------------------------------------------------------**

**Fortunella_venosa_1** **------------------------------------------------------------**

**Fortunella_venosa_2** **------------------------------------------------------------**

**Fortunella_venosa_3** **------------------------------------------------------------**

9970	9980	9990	10000	10010	10020

....|....|....|....|....|....|....|....|....|....|....|....|

**Atalantia_kwangtungens** **gaacaataaaaagaaggactatttccattcttttgattgaatcaggaatcaaagtttgta**

**Citrus_reticulata_1** **-aacaataaaaagaaggactatttccattcttttgattgaatcaagaataaaaatttgta**

**Citrus_reticulata_2** **-aacaataaaaagaaggactatttccattcttttgattgaatcaagaataaaaatttgta**

**Citrus_trifoliata_3** **-aacaataaaaagaaggactatttccattcttttgattgaatcaagaatcaaaatttgta**

**Citrus_aurantiifolia** **-aacaataaaaagaaggactatttccattcttttgattgaatcaagaatcaaaatttgta**

**Citrus_aurantium** **-aacaataaaaagaaggactatttccattcttttgattgaatcaagaatccaaatttgta**

**Citrus_cavaleriei** **-aacaataaaaagaaggactatttccattcttttgattgaatcaagaatcaaaatttgta**

**Citrus_hongheensis** **-aacaataaaaagaaggactatttccattcttttgattgaatcaagaatccaaatttgta**

**Citrus_limon** **-aacaataaaaagaaggactatttccattcttttgattgaatcaagaatccaaatttgta**

**Citrus_maxima** **-aacaataaaaagaaggactatttccattcttttgattgaatcaagaatccaaatttgta**

**Citrus_medica** **-aacaataaaaagaaggactatttccattcttttgattgaatcaagaataaaaattagta**

**Citrus_polytrifolia** **-aacaataaaaagaaggactatttccattcttttgattgaatcaagaa------------**

**Citrus_reticulata** **-aacaataaaaagaaggactatttccattcttttgattgaatcaagaataaaaatttgta**

**Citrus_sinensis** **-aacaataaaaagaaggactatttccattcttttgattgaatcaagaatccaaatttgta**

**Fortunella_bawangica_1** **-aacaataaaaagaaggactatttccattcttttgattgaatcaagaataaaaatttgta**

**Fortunella_bawangica_2** **-aacaataaaaagaaggactatttccattcttttgattgaatcaagaataaaaatttgta**

**Fortunella_hindsii_1** **-aacaataaaaagaaggactatttccattcttttgattgaatcaagaataaaaatttgta**

**Fortunella_hindsii_2** **-aacaataaaaagaaggactatttccattcttttgattgaatcaagaataaaaatttgta**

**Fortunella_hindsii_3** **-aacaataaaaagaaggactatttccattcttttgattgaatcaagaataaaaatttgta**

**Fortunella_japonica_1** **-aacaataaaaagaaggactatttccattcttttgattgaatcaagaatcaaaatttgta**

**Fortunella_japonica_2** **-aacaataaaaagaaggactatttccattcttttgattgaatcaagaataaaaatttgta**

**Fortunella_japonica_3** **-aacaataaaaagaaggactatttccattcttttgattgaatcaagaatcaaaatttgta**

**Fortunella_japonica_4** **-aacaataaaaagaaggactatttccattcttttgattgaatcaagaatcaaaatttgta**

**Fortunella_japonica_5** **-aacaataaaaagaaggactatttccattcttttgattgaatcaagaatcaaaatttgta**

**Fortunella_japonica_6** **-aacaataaaaagaaggactatttccattcttttgattgaatcaagaatcaaaatttgta**

**Fortunella_japonica_7** **-aacaataaaaagaaggactatttccattcttttgattgaatcaagaatcaaaatttgta**

**Fortunella_japonica_8** **-aacaataaaaagaaggactatttccattcttttgattgaatcaagaatcaaaatttgta**

**Fortunella_japonica_9** **-aacaataaaaagaaggactatttccattcttttgattgaatcaagaatcaaaatttgta**

**Fortunella_japonica_10** **-aacaataaaaagaaggactatttccattcttttgattgaatcaagaataaaaatttgta**

**Fortunella_japonica_11** **-aacaataaaaagaaggactatttccattcttttgattgaatcaagaatcaaaatttgta**

**Fortunella_japonica_12** **-aacaataaaaagaaggactatttccattcttttgattgaatcaagaatcaaaatttgta**

**Fortunella_margarita_1** **-aacaataaaaagaaggactatttccattcttttgattgaatcaagaataaaaatttgta**

**Fortunella_margarita_2** **-aacaataaaaagaaggactatttccattcttttgattgaatcaagaataaaaatttgta**

**Fortunella_venosa_1** **-aacaataaaaagaaggactatttccattcttttgattgaatcaagaataaaaatttgta**

**Fortunella_venosa_2** **-aacaataaaaagaaggactatttccattcttttgattgaatcaagaataaaaatttgta**

**Fortunella_venosa_3** **-aacaataaaaagaaggactatttccattcttttgattgaatcaagaataaaaatttgta**

10030	10040	10050	10060	10070	10080

....|....|....|....|....|....|....|....|....|....|....|....|

**Atalantia_kwangtungens** **tttggtgtgtggataaaagttattttgtcgagccatatctgtcaaaattcgtccaacaaa**

**Citrus_reticulata_1** **tttggtgtgtggataaaagttattttgtcgagccatatctgtcaaaattcgtccaacaaa**

**Citrus_reticulata_2** **tttggtgtgtggataaaagttattttgtcgagccatatctgtcaaaattcgtccaacaaa**

**Citrus_trifoliata_3** **tttggtgtgtggataaaagttattttgtcgagccatatctgtcaaaattcgtccaacaaa**

**Citrus_aurantiifolia** **tttggtgtgtggataaaagttattttgtcgagccatatctgtcaaaattcgtccaacaaa**

**Citrus_aurantium** **tttggtgtgtggataaaagttattttgtcgagccatatctgtcaaaattcgtccaacaaa**

**Citrus_cavaleriei** **tttggtgtgtggataaaagtaattttctcgagccatatctgtcaaaattcgtccaacaaa**

**Citrus_hongheensis** **tttggtgtgtggataaaagttattttgtcgagccatatctgtcaaaattcgtccaacaaa**

**Citrus_limon** **tttggtgtgtggataaaagttattttgtcgagccatatctgtcaaaattcgtccaacaaa**

**Citrus_maxima** **tttggtgtgtggataaaagttattttgtcgagccatatctgtcaaaattcgtccaacaaa**

**Citrus_medica** **tttggtgtgtggataaaagttattttgtcgagccatatctgtcaaaattagtccaacaaa**

**Citrus_polytrifolia** **---------------------------------------------------tccaacaaa**

**Citrus_reticulata** **tttggtgtgtggataaaagttattttgtcgagccatatctgtcaaaattcgtccaacaaa**

**Citrus_sinensis** **tttggtgtgtggataaaagttattttgtcgagccatatctgtcaaaattcgtccaacaaa**

**Fortunella_bawangica_1** **tttggtgtgtggataaaagttattttgtcgagccatatctgtcaaaattcgtccaacaaa**

**Fortunella_bawangica_2** **tttggtgtgtggataaaagttattttgtcgagccatatctgtcaaaattcgtccaacaaa**

**Fortunella_hindsii_1** **tttggtgtgtggataaaagttattttgtcgagccatatctgtcaaaattcgtccaacaaa**

**Fortunella_hindsii_2** **tttggtgtgtggataaaagttattttgtcgagccatatctgtcaaaattcgtccaacaaa**

**Fortunella_hindsii_3** **tttggtgtgtggataaaagttattttgtcgagccatatctgtcaaaattcgtccaacaaa**

**Fortunella_japonica_1** **tttggtgtgtggataaaagttattttgtcgagccatatctgtcaaaattcgtccaacaaa**

**Fortunella_japonica_2** **tttggtgtgtggataaaagttattttgtcgagccatatctgtcaaaattcgtccaacaaa**

**Fortunella_japonica_3** **tttggtgtgtggataaaagttattttgtcgagccatatctgtcaaaattcgtccaacaaa**

**Fortunella_japonica_4** **tttggtgtgtggataaaagttattttgtcgagccatatctgtcaaaattcgtccaacaaa**

**Fortunella_japonica_5** **tttggtgtgtggataaaagttattttgtcgagccatatctgtcaaaattcgtccaacaaa**

**Fortunella_japonica_6** **tttggtgtgtggataaaagttattttgtcgagccatatctgtcaaaattcgtccaacaaa**

**Fortunella_japonica_7** **tttggtgtgtggataaaagttattttgtcgagccatatctgtcaaaattcgtccaacaaa**

**Fortunella_japonica_8** **tttggtgtgtggataaaagttattttgtcgagccatatctgtcaaaattcgtccaacaaa**

**Fortunella_japonica_9** **tttggtgtgtggataaaagttattttgtcgagccatatctgtcaaaattcgtccaacaaa**

**Fortunella_japonica_10** **tttggtgtgtggataaaagttattttgtcgagccatatctgtcaaaattcgtccaacaaa**

**Fortunella_japonica_11** **tttggtgtgtggataaaagttattttgtcgagccatatctgtcaaaattcgtccaacaaa**

**Fortunella_japonica_12** **tttggtgtgtggataaaagttattttgtcgagccatatctgtcaaaattcgtccaacaaa**

**Fortunella_margarita_1** **tttggtgtgtggataaaagttattttgtcgagccatatctgtcaaaattcgtccaacaaa**

**Fortunella_margarita_2** **tttggtgtgtggataaaagttattttgtcgagccatatctgtcaaaattcgtccaacaaa**

**Fortunella_venosa_1** **tttggtgtgtggataaaagttattttgtcgagccatatctgtcaaaattcgtccaacaaa**

**Fortunella_venosa_2** **tttggtgtgtggataaaagttattttgtcgagccatatctgtcaaaattcgtccaacaaa**

**Fortunella_venosa_3** **tttggtgtgtggataaaagttattttgtcgagccatatctgtcaaaattcgtccaacaaa**

10090	10100	10110	10120	10130	10140

....|....|....|....|....|....|....|....|....|....|....|....|

**Atalantia_kwangtungens** **agaaattg------ttttgaattattaaatttagatttaaacgaaataactcctccttta**

**Citrus_reticulata_1** **agaaattg------ttttgaattattaaatttag-------------------ttattta**

**Citrus_reticulata_2** **agaaattg------ttttgaattattaaatttag-------------------ttattta**

**Citrus_trifoliata_3** **agaaattg------ttttgaattattcaatttag-------------------ttattta**

**Citrus_aurantiifolia** **agaaattg------ttttgaattattaaatttag-------------------ttattta**

**Citrus_aurantium** **agaaattg------ttttgaattattaaatttag-------------------ttattta**

**Citrus_cavaleriei** **agaaattg------ttttgaattattaaatttag-------------------ttattta**

**Citrus_hongheensis** **agaaattg------ttttgaattattaaatttag-------------------ttattta**

**Citrus_limon** **agaaattg------ttttgaattattaaatttag-------------------ttattta**

**Citrus_maxima** **agaaattg------ttttgaattattaaatttag-------------------ttattta**

**Citrus_medica** **agaaattgttttgattttgaattattaaatttag-------------------ttattta**

**Citrus_polytrifolia** **agaaattg------ttttgaattattcaatttag-------------------ttattta**

**Citrus_reticulata** **agaaattg------ttttgaattattaaatttag-------------------ttattta**

**Citrus_sinensis** **agaaattg------ttttgaattattaaatttag-------------------ttattta**

**Fortunella_bawangica_1** **agaaattg------ttttgaattattaaatttag-------------------ttattta**

**Fortunella_bawangica_2** **agaaattg------ttttgaattattaaatttag-------------------ttattta**

**Fortunella_hindsii_1** **agaaattg------ttttgaattattaaatttag-------------------ttattta**

**Fortunella_hindsii_2** **agaaattg------ttttgaattattaaatttag-------------------ttattta**

**Fortunella_hindsii_3** **agaaattg------ttttgaattattcaatttag-------------------ttattta**

**Fortunella_japonica_1** **agaaattg------ttttgaattattaaatttag-------------------ttattta**

**Fortunella_japonica_2** **agaaattg------ttttgaattattaaatttag-------------------ttattta**

**Fortunella_japonica_3** **agaaattg------ttttgaattattaaatttag-------------------ttattta**

**Fortunella_japonica_4** **agaaattg------ttttgaattattaaatttag-------------------ttattta**

**Fortunella_japonica_5** **agaaattg------ttttgaattattaaatttag-------------------ttattta**

**Fortunella_japonica_6** **agaaattg------ttttgaattattaaatttag-------------------ttattta**

**Fortunella_japonica_7** **agaaattg------ttttgaattattaaatttag-------------------ttattta**

**Fortunella_japonica_8** **agaaattg------ttttgaattattaaatttag-------------------ttattta**

**Fortunella_japonica_9** **agaaattg------ttttgaattattaaatttag-------------------ttattta**

**Fortunella_japonica_10** **agaaattg------ttttgaattattaaatttag-------------------ttattta**

**Fortunella_japonica_11** **agaaattg------ttttgaattattaaatttag-------------------ttattta**

**Fortunella_japonica_12** **agaaattg------ttttgaattattaaatttag-------------------ttattta**

**Fortunella_margarita_1** **agaaattg------ttttgaattattaaatttag-------------------ttattta**

**Fortunella_margarita_2** **agaaattg------ttttgaattattaaatttag-------------------ttattta**

**Fortunella_venosa_1** **agaaattg------ttttgaattattaaatttag-------------------ttattta**

**Fortunella_venosa_2** **agaaattg------ttttgaattattaaatttag-------------------ttattta**

**Fortunella_venosa_3** **agaaattg------ttttgaattattaaatttag-------------------ttattta**

10150	10160	10170	10180	10190	10200

....|....|....|....|....|....|....|....|....|....|....|....|

**Atalantia_kwangtungens** **aacgaaataactcctcctttacgaggactccttacaaagacgtcaacgttctaatttcga**

**Citrus_reticulata_1** **aacgaaataactcctcctttacgaggactcctgacaaagacgtcaacgttctaatttcga**

**Citrus_reticulata_2** **aacgaaataactcctcctttacgaggactcctgacaaagacgtcaacgttctaatttcga**

**Citrus_trifoliata_3** **aacgaaataactcctcctttacgaggactcctgacaaagacgtcaacgttctaatttcga**

**Citrus_aurantiifolia** **aacgaaataactcctcctttacgaggactcctgacaaagacgtcaacgttctaatttcga**

**Citrus_aurantium** **aacgaaataactcctcctttacgaggactcctgacaaagacgtcaacgttctaatttcca**

**Citrus_cavaleriei** **aacgaaataactcctcctttacgaggactcctgacaaagacgtcaacgttctaatttcga**

**Citrus_hongheensis** **aacgaaataactcctcctttacgaggactcctgacaaagacgtcaacgttctaatttcca**

**Citrus_limon** **aacgaaataactcctcctttacgaggactcctgacaaagacgtcaacgttctaatttcca**

**Citrus_maxima** **aacgaaataactcctcctttacgaggactcctgacaaagacgtcaacgttctaatttcca**

**Citrus_medica** **aacgaaataactcctcctttacgaggactcctgacaaagacgtcaacgttctaatttcga**

**Citrus_polytrifolia** **aacgaaataactcctcctttacgaggactcctgacaaagacgtcaacgttctaatttcga**

**Citrus_reticulata** **aacgaaataactcctcctttacgaggactcctgacaaagacgtcaacgttctaatttcga**

**Citrus_sinensis** **aacgaaataactcctcctttacgaggactcctgacaaagacgtcaacgttctaatttcca**

**Fortunella_bawangica_1** **aacgaaataactcctcctttacgaggactcctgacaaaga----------ctaatttcga**

**Fortunella_bawangica_2** **aacgaaataactcctcctttacgaggactcctgacaaaga----------ctaatttcga**

**Fortunella_hindsii_1** **aacgaaataactcctcctttacgaggactcctgacaaaga----------ctaatttcga**

**Fortunella_hindsii_2** **aacgaaataactcctcctttacgaggactcctgacaaaga----------ctaatttcga**

**Fortunella_hindsii_3** **aacgaaataactcctcctttacgaggactcctgacaaaga----------ctaatttcga**

**Fortunella_japonica_1** **aacgaaataactcctcctttacgaggactcctgacaaaga----------ctaatttcga**

**Fortunella_japonica_2** **aacgaaataactcctcctttacgaggactcctgacaaaga----------ctaatttcga**

**Fortunella_japonica_3** **aacgaaataactcctcctttacgaggactcctgacaaaga----------ctaatttcga**

**Fortunella_japonica_4** **aacgaaataactcctcctttacgaggactcctgacaaaga----------ctaatttcga**

**Fortunella_japonica_5** **aacgaaataactcctcctttacgaggactcctgacaaaga----------ctaatttcga**

**Fortunella_japonica_6** **aacgaaataactcctcctttacgaggactcctgacaaaga----------ctaatttcga**

**Fortunella_japonica_7** **aacgaaataactcctcctttacgaggactcctgacaaaga----------ctaatttcga**

**Fortunella_japonica_8** **aacgaaataactcctcctttacgaggactcctgacaaaga----------ctaatttcga**

**Fortunella_japonica_9** **aacgaaataactcctcctttacgaggactcctgacaaaga----------ctaatttcga**

**Fortunella_japonica_10** **aacgaaataactcctcctttacgaggactcctgacaaaga----------ctaatttcga**

**Fortunella_japonica_11** **aacgaaataactcctcctttacgaggactcctgacaaaga----------ctaatttcga**

**Fortunella_japonica_12** **aacgaaataactcctcctttacgaggactcctgacaaaga----------ctaatttcga**

**Fortunella_margarita_1** **aacgaaataactcctcctttacgaggactcctgacaaaga----------ctaatttcga**

**Fortunella_margarita_2** **aacgaaataactcctcctttacgaggactcctgacaaaga----------ctaatttcga**

**Fortunella_venosa_1** **aacgaaataactcctcctttacgaggactcctgacaaaga----------ctaatttcga**

**Fortunella_venosa_2** **aacgaaataactcctcctttacgaggactcctgacaaaga----------ctaatttcga**

**Fortunella_venosa_3** **aacgaaataactcctcctttacgaggactcctgacaaaga----------ctaatttcga**

10210	10220	10230	10240	10250	10260

....|....|....|....|....|....|....|....|....|....|....|....|

**Atalantia_kwangtungens** **agtttcatttcatgattattttgaatttctgtcctatcttgattacatctgattctcttg**

**Citrus_reticulata_1** **attttcatttcatgattattttaaatttatgtcctatcttgattacatctgattctcttg**

**Citrus_reticulata_2** **attttcatttcatgattattttaaatttatgtcctatcttgattacatctgattctcttg**

**Citrus_trifoliata_3** **attttcatttcatgattattttaaatttatgtcctatcttgattacatctgattctcttg**

**Citrus_aurantiifolia** **attttcatttcatgattattttaaatttatgtcctatcttgattacatctgattctcttg**

**Citrus_aurantium** **cttttcatttcatgattattttaaatttatgtcctatcttgattacatctgattctcttg**

**Citrus_cavaleriei** **attttcatttcatgattattttaaatttatgtcctatcttgattacatctgattctcttg**

**Citrus_hongheensis** **attttcatttcatgattattttaaatttatgtcctatcttgattacatctgattctcttg**

**Citrus_limon** **cttttcatttcatgattattttaaatttatgtcctatcttgattacatctgattctcttg**

**Citrus_maxima** **attttcatttcatgattattttaaatttatgtcctatcttgattacatctgattctcttg**

**Citrus_medica** **attttcatttcatgattattttaaatttatgtcctatcttgattacatctgattctcttg**

**Citrus_polytrifolia** **attttcatttcatgattattttaaatttatgtcctatcttgattacatccgattctcttg**

**Citrus_reticulata** **attttcatttcatgattattttaaatttatgtcctatcttgattacatctgattctcttg**

**Citrus_sinensis** **attttcatttcatgattattttaaatttatgtcctatcttgattacatctgattctcttg**

**Fortunella_bawangica_1** **attttcatttcatgattattttaaatttatgtcctatcttgattacatctgattctcttg**

**Fortunella_bawangica_2** **attttcatttcatgattattttaaatttatgtcctatcttgattacatctgattctcttg**

**Fortunella_hindsii_1** **attttcatttcatgattattttaaatttatgtcctatcttgattacatctgattctcttg**

**Fortunella_hindsii_2** **attttcatttcatgattattttaaatttatgtcctatcttgattacatctgattctcttg**

**Fortunella_hindsii_3** **attttcatttcatgattattttaaatttatgtcctatcttgattacatctgattctcttg**

**Fortunella_japonica_1** **attttcatttcatgattattttaaatttatgtcctatcttgattacatctgattctcttg**

**Fortunella_japonica_2** **attttcatttcatgattattttaaatttatgtcctatcttgattacatctgattctcttg**

**Fortunella_japonica_3** **attttcatttcatgattattttaaatttatgtcctatcttgattacatctgattctcttg**

**Fortunella_japonica_4** **attttcatttcatgattattttaaatttatgtcctatcttgattacatctgattctcttg**

**Fortunella_japonica_5** **attttcatttcatgattattttaaatttatgtcctatcttgattacatctgattctcttg**

**Fortunella_japonica_6** **attttcatttcatgattattttaaatttatgtcctatcttgattacatctgattctcttg**

**Fortunella_japonica_7** **attttcatttcatgattattttaaatttatgtcctatcttgattacatctgattctcttg**

**Fortunella_japonica_8** **attttcatttcatgattattttaaatttatgtcctatcttgattacatctgattctcttg**

**Fortunella_japonica_9** **attttcatttcatgattattttaaatttatgtcctatcttgattacatctgattctcttg**

**Fortunella_japonica_10** **attttcatttcatgattattttaaatttatgtcctatcttgattacatctgattctcttg**

**Fortunella_japonica_11** **attttcatttcatgattattttaaatttatgtcctatcttgattacatctgattctcttg**

**Fortunella_japonica_12** **attttcatttcatgattattttaaatttatgtcctatcttgattacatctgattctcttg**

**Fortunella_margarita_1** **attttcatttcatgattattttaaatttatgtcctatcttgattacatctgattctcttg**

**Fortunella_margarita_2** **attttcatttcatgattattttaaatttatgtcctatcttgattacatctgattctcttg**

**Fortunella_venosa_1** **attttcatttcatgattattttaaatttatgtcctatcttgattacatctgattctcttg**

**Fortunella_venosa_2** **attttcatttcatgattattttaaatttatgtcctatcttgattacatctgattctcttg**

**Fortunella_venosa_3** **attttcatttcatgattattttaaatttatgtcctatcttgattacatctgattctcttg**

10270	10280	10290	10300	10310	10320

....|....|....|....|....|....|....|....|....|....|....|....|

**Atalantia_kwangtungens** **ttcgacaaagggccctttttatacaataatcgcattgtagcgggtatagtttagtggtaa**

**Citrus_reticulata_1** **ttcgacaaagggccctttttatacaataatcgcattgtagcgggtatagtttagtggtaa**

**Citrus_reticulata_2** **ttcgacaaagggccctttttatacaataatcgcattgtagcgggtatagtttagtggtaa**

**Citrus_trifoliata_3** **ttcgacaaagggccctttttttacaataatcgcattgtagcgggtatagtttagtggtaa**

**Citrus_aurantiifolia** **ttcgacaaagggccctttttatacaataatcgcattgtagcgggtatagtttagtggtaa**

**Citrus_aurantium** **ttcgacaaagggccctttttatacaataatcgcattgtagcgggtatagtttagtggtaa**

**Citrus_cavaleriei** **ttcgacaaagggccctttttatacaataatcgcattgtagcgggtatagtttagtggtaa**

**Citrus_hongheensis** **ttcgacaaagggccctttttatacaataatcgcattgtagcgggtatagtttagtggtaa**

**Citrus_limon** **ttcgacaaagggccctttttatacaataatcgcattgtagcgggtatagtttagtggtaa**

**Citrus_maxima** **ttcgacaaagggccctttttatacaataatcgcattgtagcgggtatagtttagtggtaa**

**Citrus_medica** **ttcgacaaagggccctttttatacaataatcgcattgtagcgggtatagtttagtggtaa**

**Citrus_polytrifolia** **ttcgacaaagggccctttttttacaataatcgcattgtagcgggtatagtttagtggtaa**

**Citrus_reticulata** **ttcgacaaagggccctttttatacaataatcgcattgtagcgggtatagtttagtggtaa**

**Citrus_sinensis** **ttcgacaaagggccctttttatacaataatcgcattgtagcgggtatagtttagtggtaa**

**Fortunella_bawangica_1** **ttcgacaaagggccctttttatacaataatcgcattgtagcgggtatagtttagtggtaa**

**Fortunella_bawangica_2** **ttcgacaaagggccctttttatacaataatcgcattgtagcgggtatagtttagtggtaa**

**Fortunella_hindsii_1** **ttcgacaaagggccctttttatacaataatcgcattgtagcgggtatagtttagtggtaa**

**Fortunella_hindsii_2** **ttcgacaaagggccctttttatacaataatcgcattgtagcgggtatagtttagtggtaa**

**Fortunella_hindsii_3** **ttcgacaaagggccctttttatacaataatcgcattgtagcgggtatagtttagtggtaa**

**Fortunella_japonica_1** **ttcgacaaagggccctttttatacaataatcgcattgtagcgggtatagtttagtggtaa**

**Fortunella_japonica_2** **ttcgacaaagggccctttttatacaataatcgcattgtagcgggtatagtttagtggtaa**

**Fortunella_japonica_3** **ttcgacaaagggccctttttatacaataatcgcattgtagcgggtatagtttagtggtaa**

**Fortunella_japonica_4** **ttcgacaaagggccctttttatacaataatcgcattgtagcgggtatagtttagtggtaa**

**Fortunella_japonica_5** **ttcgacaaagggccctttttatacaataatcgcattgtagcgggtatagtttagtggtaa**

**Fortunella_japonica_6** **ttcgacaaagggccctttttatacaataatcgcattgtagcgggtatagtttagtggtaa**

**Fortunella_japonica_7** **ttcgacaaagggccctttttatacaataatcgcattgtagcgggtatagtttagtggtaa**

**Fortunella_japonica_8** **ttcgacaaagggccctttttatacaataatcgcattgtagcgggtatagtttagtggtaa**

**Fortunella_japonica_9** **ttcgacaaagggccctttttatacaataatcgcattgtagcgggtatagtttagtggtaa**

**Fortunella_japonica_10** **ttcgacaaagggccctttttatacaataatcgcattgtagcgggtatagtttagtggtaa**

**Fortunella_japonica_11** **ttcgacaaagggccctttttatacaataatcgcattgtagcgggtatagtttagtggtaa**

**Fortunella_japonica_12** **ttcgacaaagggccctttttatacaataatcgcattgtagcgggtatagtttagtggtaa**

**Fortunella_margarita_1** **ttcgacaaagggccctttttatacaataatcgcattgtagcgggtatagtttagtggtaa**

**Fortunella_margarita_2** **ttcgacaaagggccctttttatacaataatcgcattgtagcgggtatagtttagtggtaa**

**Fortunella_venosa_1** **ttcgacaaagggccctttttatacaataatcgcattgtagcgggtatagtttagtggtaa**

**Fortunella_venosa_2** **ttcgacaaagggccctttttatacaataatcgcattgtagcgggtatagtttagtggtaa**

**Fortunella_venosa_3** **ttcgacaaagggccctttttatacaataatcgcattgtagcgggtatagtttagtggtaa**

10330	10340	10350	10360	10370	10380

....|....|....|....|....|....|....|....|....|....|....|....|

**Atalantia_kwangtungens** **aagtgtgattcgttcaattaatccccttaatagttaaggggtccttcagtttaattgatc**

**Citrus_reticulata_1** **aagtgtgattcgttcaattaatccccttaatagttaaggggtccttcaatttaattgata**

**Citrus_reticulata_2** **aagtgtgattcgttcaattaatccccttaatagttaaggggtccttcaatttaattgata**

**Citrus_trifoliata_3** **aagtgtgattcgttcaattaatccccttaatagttaaggggtccttcagttaaattgata**

**Citrus_aurantiifolia** **aagtgtgattcgttcaattaatccccttaatagttaaggggtccttcagtttaattgata**

**Citrus_aurantium** **aagtgtgattcgttcaattaatccccttaatagttaaggggtccttcagtttaattgata**

**Citrus_cavaleriei** **aagtgtgattcgttcaattaatccccttaatagttaaggggtccttcaatttaattgata**

**Citrus_hongheensis** **aagtgtgattcgttcaattaatccccttaatagttaaggggtccttcagtttaattgata**

**Citrus_limon** **aagtgtgattcgttcaattaatccccttaatagttaaggggtccttcagtttaattgata**

**Citrus_maxima** **aagtgtgattcgttcaattaatccccttaatagttaaggggtccttcagtttaattgata**

**Citrus_medica** **aagtgtgattcgttcaattaatccccttaatagttaaggggtccttcagtttaatttata**

**Citrus_polytrifolia** **aagtgtgattcgttcaattaatccccttaatagttaaggggtccttcagttaaattgata**

**Citrus_reticulata** **aagtgtgattcgttcaattaatccccttaatagttaaggggtccttcaatttaattgata**

**Citrus_sinensis** **aagtgtgattcgttcaattaatccccttaatagttaaggggtccttcagtttaattgata**

**Fortunella_bawangica_1** **aagtgtgattcgttcaattaatccccttaatggttaaggggtccttcagtttaattgata**

**Fortunella_bawangica_2** **aagtgtgattcgttcaattaatccccttaatggttaaggggtccttcagtttaattgata**

**Fortunella_hindsii_1** **aagtgtgattcgttcaattaatccccttaatggttaaggggtccttcagtttaattgata**

**Fortunella_hindsii_2** **aagtgtgattcgttcaattaatccccttaatggttaaggggtccttcagtttaattgata**

**Fortunella_hindsii_3** **aagtgtgattcgttcaattaatccccttaatggttaaggggtccttcagtttaattgata**

**Fortunella_japonica_1** **aagtgtgattcgttcaattaatccccttaatggttaaggggtccttcagtttaattgata**

**Fortunella_japonica_2** **aagtgtgattcgttcaattaatccccttaatggttaaggggtccttcagtttaattgata**

**Fortunella_japonica_3** **aagtgtgattcgttcaattaatccccttaatggttaaggggtccttcagtttaattgata**

**Fortunella_japonica_4** **aagtgtgattcgttcaattaatccccttaatggttaaggggtccttcagtttaattgata**

**Fortunella_japonica_5** **aagtgtgattcgttcaattaatccccttaatggttaaggggtccttcagtttaattgata**

**Fortunella_japonica_6** **aagtgtgattcgttcaattaatccccttaatggttaaggggtccttcagtttaattgata**

**Fortunella_japonica_7** **aagtgtgattcgttcaattaatccccttaatggttaaggggtccttcagtttaattgata**

**Fortunella_japonica_8** **aagtgtgattcgttcaattaatccccttaatggttaaggggtccttcagtttaattgata**

**Fortunella_japonica_9** **aagtgtgattcgttcaattaatccccttaatggttaaggggtccttcagtttaattgata**

**Fortunella_japonica_10** **aagtgtgattcgttcaattaatccccttaatggttaaggggtccttcagtttaattgata**

**Fortunella_japonica_11** **aagtgtgattcgttcaattaatccccttaatggttaaggggtccttcagtttaattgata**

**Fortunella_japonica_12** **aagtgtgattcgttcaattaatccccttaatggttaaggggtccttcagtttaattgata**

**Fortunella_margarita_1** **aagtgtgattcgttcaattaatccccttaatggttaaggggtccttcagtttaattgata**

**Fortunella_margarita_2** **aagtgtgattcgttcaattaatccccttaatggttaaggggtccttcagtttaattgata**

**Fortunella_venosa_1** **aagtgtgattcgttcaattaatccccttaatggttaaggggtccttcagtttaattgata**

**Fortunella_venosa_2** **aagtgtgattcgttcaattaatccccttaatggttaaggggtccttcagtttaattgata**

**Fortunella_venosa_3** **aagtgtgattcgttcaattaatccccttaatggttaaggggtccttcagtttaattgata**

10390	10400	10410	10420	10430	10440

....|....|....|....|....|....|....|....|....|....|....|....|

**Atalantia_kwangtungens** **tttcaatcaaaaactttatttcttaaaaggattaaatttgaatcctttcactctaaaatt**

**Citrus_reticulata_1** **tttcaatcaaaaactttatttcttaaaaggattaaatttgaatcctttcactctcaaatt**

**Citrus_reticulata_2** **tttcaatcaaaaactttatttcttaaaaggattaaatttgaatcctttcactctcaaatt**

**Citrus_trifoliata_3** **tttcaataaaaaactttatttcttaaaaggattaaattttaatcctttcactctcaaatt**

**Citrus_aurantiifolia** **tttcaatcaaaaactttatttcttaaaaggattaaattttaatcctttcactctcaaatt**

**Citrus_aurantium** **tttcaatcaaaaactttatttcttaaaaggattcaattttaatcctttcactctcaaatt**

**Citrus_cavaleriei** **tttcaatcaaaaactttatttcttaaaaggattcaattttaatcctttcactctcaaatt**

**Citrus_hongheensis** **tttcaatcaaaaactttatttcttaaaaggattcaattttaatcctttcactctcaaatt**

**Citrus_limon** **tttcaatcaaaaactttatttcttaaaaggattcaattttaatcctttcactctcaaatt**

**Citrus_maxima** **tttcaatcaaaaactttatttcttaaaaggattcaattttaatcctttcactctcaaatt**

**Citrus_medica** **tttcaatcaaaaactttatttcttaaaaggattaaattttaatcctttcactctcaaatt**

**Citrus_polytrifolia** **tttcaataaaaaactttatttcttaaaaggattaaattttaatcctttcactctcaaatt**

**Citrus_reticulata** **tttcaatcaaaaactttatttcttaaaaggattaaatttgaatcctttcactctcaaatt**

**Citrus_sinensis** **tttcaatcaaaaactttatttcttaaaaggattcaattttaatcctttcactctcaaatt**

**Fortunella_bawangica_1** **tttcaatcaaaaactttatttcttaaaaggattaaattttaatcctttcactctcaaatt**

**Fortunella_bawangica_2** **tttcaatcaaaaactttatttcttaaaaggattaaattttaatcctttcactctcaaatt**

**Fortunella_hindsii_1** **tttcaatcaaaaactttatttcttaaaaggattaaattttaatcctttcactctcaaatt**

**Fortunella_hindsii_2** **tttcaatcaaaaactttatttcttaaaaggattaaattttaatcctttcactctcaaatt**

**Fortunella_hindsii_3** **tttcaatcaaaaactttatttcttaaaaggattaaattttaatcctttcactctcaaatt**

**Fortunella_japonica_1** **tttcaatcaaaaactttatttcttaaaaggattaaattttaatcctttcactctcaaatt**

**Fortunella_japonica_2** **tttcaatcaaaaactttatttcttaaaaggattaaattttaatcctttcactctcaaatt**

**Fortunella_japonica_3** **tttcaatcaaaaactttatttcttaaaaggattaaattttaatcctttcactctcaaatt**

**Fortunella_japonica_4** **tttcaatcaaaaactttatttcttaaaaggattaaattttaatcctttcactctcaaatt**

**Fortunella_japonica_5** **tttcaatcaaaaactttatttcttaaaaggattaaattttaatcctttcactctcaaatt**

**Fortunella_japonica_6** **tttcaatcaaaaactttatttcttaaaaggattaaattttaatcctttcactctcaaatt**

**Fortunella_japonica_7** **tttcaatcaaaaactttatttcttaaaaggattaaattttaatcctttcactctcaaatt**

**Fortunella_japonica_8** **tttcaatcaaaaactttatttcttaaaaggattaaattttaatcctttcactctcaaatt**

**Fortunella_japonica_9** **tttcaatcaaaaactttatttcttaaaaggattaaattttaatcctttcactctcaaatt**

**Fortunella_japonica_10** **tttcaatcaaaaactttatttcttaaaaggattaaattttaatcctttcactctcaaatt**

**Fortunella_japonica_11** **tttcaatcaaaaactttatttcttaaaaggattaaattttaatcctttcactctcaaatt**

**Fortunella_japonica_12** **tttcaatcaaaaactttatttcttaaaaggattaaattttaatcctttcactctcaaatt**

**Fortunella_margarita_1** **tttcaatcaaaaactttatttcttaaaaggattaaattttaatcctttcactctcaaatt**

**Fortunella_margarita_2** **tttcaatcaaaaactttatttcttaaaaggattaaattttaatcctttcactctcaaatt**

**Fortunella_venosa_1** **tttcaatcaaaaactttatttcttaaaaggattaaattttaatcctttcactctcaaatt**

**Fortunella_venosa_2** **tttcaatcaaaaactttatttcttaaaaggattaaattttaatcctttcactctcaaatt**

**Fortunella_venosa_3** **tttcaatcaaaaactttatttcttaaaaggattaaattttaatcctttcactctcaaatt**

10450	10460	10470	10480	10490	10500

....|....|....|....|....|....|....|....|....|....|....|....|

**Atalantia_kwangtungens** **aaaataaaagattcggagaaaaatatacattctcgtgatttgtatccaagagtcaattcg**

**Citrus_reticulata_1** **taaataaaagattcggagaaaaatatccgttctcgtgatttgtatccaagagtcaattcg**

**Citrus_reticulata_2** **taaataaaagattcggagaaaaatatccgttctcgtgatttgtatccaagagtcaattcg**

**Citrus_trifoliata_3** **taaataaaagattcggagaaaaatatccgttctcgtgatttgtatccaagagtcaattcg**

**Citrus_aurantiifolia** **taaataaaagattcggagaaaaatatccgttctcgtgatttgtatccaagagtcaattcg**

**Citrus_aurantium** **taaataaaagattcggagaaaaatatccgttctcgtgatttgtatccaagagtcaattcg**

**Citrus_cavaleriei** **taaataaaagattcggagaaaaatatccgttctcgtgatttgtatccaagagtcaattcg**

**Citrus_hongheensis** **taaataaaagattcggagaaaaatatccgttctcgtgatttgtatccaagagtcaattcg**

**Citrus_limon** **taaataaaagattcggagaaaaatatccgttctcgtgatttgtatccaagagtcaattcg**

**Citrus_maxima** **taaataaaagattcggagaaaaatatccgttctcgtgatttgtatccaagagtcaattcg**

**Citrus_medica** **taaataaaagattcggagaaaaatatccgttctcgtgatttgtatccaagagtcaattcg**

**Citrus_polytrifolia** **taaataaaagattcggagaaaaatatccgttctcgtgatttgtatccaagagtcaattcg**

**Citrus_reticulata** **taaataaaagattcggagaaaaatatccgttctcgtgatttgtatccaagagtcaattcg**

**Citrus_sinensis** **taaataaaagattcggagaaaaatatccgttctcgtgatttgtatccaagagtcaattcg**

**Fortunella_bawangica_1** **taaataaaagattcggagaaaaatatccgttctcgtgatttgtatccaagagtcaattcg**

**Fortunella_bawangica_2** **taaataaaagattcggagaaaaatatccgttctcgtgatttgtatccaagagtcaattcg**

**Fortunella_hindsii_1** **taaataaaagattcggagaaaaatatccgttctcgtgatttgtatccaagagtcaattcg**

**Fortunella_hindsii_2** **taaataaaagattcggagaaaaatatccgttctcgtgatttgtatccaagagtcaattcg**

**Fortunella_hindsii_3** **taaataaaagattcggagaaaaatatccgttctcgtgatttgtatccaagagtcaattcg**

**Fortunella_japonica_1** **taaataaaagattcggagaaaaatatccgttctcgtgatttgtatccaagagtcaattcg**

**Fortunella_japonica_2** **aaaataaaagattcggagaaaaatatccgttctcgtgatttgtatccaagagtcaattcg**

**Fortunella_japonica_3** **taaataaaagattcggagaaaaatatccgttctcgtgatttgtatccaagagtcaattcg**

**Fortunella_japonica_4** **taaataaaagattcggagaaaaatatccgttctcgtgatttgtatccaagagtcaattcg**

**Fortunella_japonica_5** **taaataaaagattcggagaaaaatatccgttctcgtgatttgtatccaagagtcaattcg**

**Fortunella_japonica_6** **taaataaaagattcggagaaaaatatccgttctcgtgatttgtatccaagagtcaattcg**

**Fortunella_japonica_7** **taaataaaagattcggagaaaaatatccgttctcgtgatttgtatccaagagtcaattcg**

**Fortunella_japonica_8** **taaataaaagattcggagaaaaatatccgttctcgtgatttgtatccaagagtcaattcg**

**Fortunella_japonica_9** **taaataaaagattcggagaaaaatatccgttctcgtgatttgtatccaagagtcaattcg**

**Fortunella_japonica_10** **taaataaaagattcggagaaaaatatccgttctcgtgatttgtatccaagagtcaattcg**

**Fortunella_japonica_11** **taaataaaagattcggagaaaaatatccgttctcgtgatttgtatccaagagtcaattcg**

**Fortunella_japonica_12** **taaataaaagattcggagaaaaatatccgttctcgtgatttgtatccaagagtcaattcg**

**Fortunella_margarita_1** **taaataaaagattcggagaaaaatatccgttctcgtgatttgtatccaagagtcaattcg**

**Fortunella_margarita_2** **taaataaaagattcggagaaaaatatccgttctcgtgatttgtatccaagagtcaattcg**

**Fortunella_venosa_1** **taaataaaagattcggagaaaaatatccgttctcgtgatttgtatccaagagtcaattcg**

**Fortunella_venosa_2** **taaataaaagattcggagaaaaatatccgttctcgtgatttgtatccaagagtcaattcg**

**Fortunella_venosa_3** **taaataaaagattcggagaaaaatatccgttctcgtgatttgtatccaagagtcaattcg**

10510	10520	10530	10540	10550	10560

....|....|....|....|....|....|....|....|....|....|....|....|

**Atalantia_kwangtungens** **aaattgacaatttggattatgaaattgcgaaacataa---------------tttttttt**

**Citrus_reticulata_1** **aaattgacaatttggattatgaaattgcgaaacataat--ttttttt-tttttttttttt**

**Citrus_reticulata_2** **aaattgacaatttggattatgaaattgcgaaacataat--ttttttt-tttttttttttt**

**Citrus_trifoliata_3** **aaattgacaatttggattatgaaattgcgaaacataattttgttttt-tttttttttttt**

**Citrus_aurantiifolia** **aaattgacaatttggattatgaaattgcgaaacataa-------ttt-tgtttttttttt**

**Citrus_aurantium** **aaattgacaatttggattatgaaattgcgaaacataa------tttt-gttttttgtttt**

**Citrus_cavaleriei** **aaattgacaatttggattatgaaattgcgaaacataa-------ttt-tttttttttttt**

**Citrus_hongheensis** **aaattgacaatttggattatgaaattgcgaaacataa------tttt-gttttttgtttt**

**Citrus_limon** **aaattgacaatttggattatgaaattgcgaaacataa------tttt-gttttttgtttt**

**Citrus_maxima** **aaattgacaatttggattatgaaattgcgaaacataa------tttt-gttttttgtttt**

**Citrus_medica** **aaattgacaatttggattatgaaattgcgaaacataatt---------tttttttttttt**

**Citrus_polytrifolia** **aaattgacaatttggattatgaaattgcgaaacataattttgttttt-tttttttttttt**

**Citrus_reticulata** **aaattgacaatttggattatgaaattgcgaaacataat--ttttttt-tttttttttttt**

**Citrus_sinensis** **aaattgacaatttggattatgaaattgcgaaacataa------tttt-gttttttgtttt**

**Fortunella_bawangica_1** **aa---------ttggattatgaaattgcgaaacataa-----ttttg-tttttttttttt**

**Fortunella_bawangica_2** **aa---------ttggattatgaaattgcgaaacataa-----ttttg-tttttttttttt**

**Fortunella_hindsii_1** **aa---------ttggattatgaaattgcgaaacataa-----ttttg-tttttttttttt**

**Fortunella_hindsii_2** **aa---------ttggattatgaaattgcgaaacataa-----ttttg-tttttttttttt**

**Fortunella_hindsii_3** **aa---------ttggattatgaaattgcgaaacataa-----ttttg---tttttttttt**

**Fortunella_japonica_1** **aa---------ttggattatgaaattgcgaaacataa-----ttttg-tttttttttttt**

**Fortunella_japonica_2** **aa---------ttggattatgaaattgcgaaacataa-----ttttg---tttttttttt**

**Fortunella_japonica_3** **aa---------ttggattatgaaattgcgaaacataa-----ttttg-tttttttttttt**

**Fortunella_japonica_4** **aa---------ttggattatgaaattgcgaaacataa-----ttttg-tttttttttttt**

**Fortunella_japonica_5** **aa---------ttggattatgaaattgcgaaacataa-----ttttg-tttttttttttt**

**Fortunella_japonica_6** **aa---------ttggattatgaaattgcgaaacataa-----ttttg-tttttttttttt**

**Fortunella_japonica_7** **aa---------ttggattatgaaattgcgaaacataa-----ttttg-tttttttttttt**

**Fortunella_japonica_8** **aa---------ttggattatgaaattgcgaaacataa-----ttttg-tttttttttttt**

**Fortunella_japonica_9** **aa---------ttggattatgaaattgcgaaacataa-----ttttg-tttttttttttt**

**Fortunella_japonica_10** **aa---------ttggattatgaaattgcgaaacataa-----ttttg-tttttttttttt**

**Fortunella_japonica_11** **aa---------ttggattatgaaattgcgaaacataa-----ttttg-tttttttttttt**

**Fortunella_japonica_12** **aa---------ttggattatgaaattgcgaaacataa-----ttttg-tttttttttttt**

**Fortunella_margarita_1** **aa---------ttggattatgaaattgcgaaacataa-----ttttg-tttttttttttt**

**Fortunella_margarita_2** **aa---------ttggattatgaaattgcgaaacataa-----ttttg-tttttttttttt**

**Fortunella_venosa_1** **aa---------ttggattatgaaattgcgaaacataa-----ttttgttttttttttttt**

**Fortunella_venosa_2** **aa---------ttggattatgaaattgcgaaacataa-----ttttg-tttttttttttt**

**Fortunella_venosa_3** **aa---------ttggattatgaaattgcgaaacataa-----ttttg-tttttttttttt**

10570	10580	10590	10600	10610	10620

....|....|....|....|....|....|....|....|....|....|....|....|

**Atalantia_kwangtungens** **ttaattggatcaatacttccaattttttaagtataagtaaaggatccatggataaagata**

**Citrus_reticulata_1** **tgaattggatcaatacttccaattttttaagtataagtaaaggatccatggataaagata**

**Citrus_reticulata_2** **tgaattggatcaatacttccaattttttaagtataagtaaaggatccatggataaagata**

**Citrus_trifoliata_3** **--aattggatcaatacttccaattttttaagtataagtaaaggatccatggataaagata**

**Citrus_aurantiifolia** **tgaattggatcaatacttccaattttttaagtataagtaaaggatccatggataaagata**

**Citrus_aurantium** **tgaattggatcaatacttccaattttttaagtataagtaaaggatccatggataaagata**

**Citrus_cavaleriei** **tgaattggatcaatacttccaattttttaagtataagtaaaggatccatggataaagata**

**Citrus_hongheensis** **tgaattggatcaatacttccaattttttaagtataagtaaaggatccatggataaagata**

**Citrus_limon** **tgaattggatcaatacttccaattttttaagtataagtaaaggatccatggataaagata**

**Citrus_maxima** **tgaattggatcaatacttccaattttttaagtataagtaaaggatccatggataaagata**

**Citrus_medica** **tgaattggatcaatacttccaattttttaagtataagtaaaggatccatggataaagata**

**Citrus_polytrifolia** **tgaattggatcaatacttccaattttttaagtataagtaaaggatccatggataaagata**

**Citrus_reticulata** **tgaattggatcaatacttccaattttttaagtataagtaaaggatccatggataaagata**

**Citrus_sinensis** **tgaattggatcaatacttccaattttttaagtataagtaaaggatccatggataaagata**

**Fortunella_bawangica_1** **tgaattggatcaatacttccaattttttaagtataagtaaaggatccatggataaagata**

**Fortunella_bawangica_2** **tgaattggatcaatacttccaattttttaagtataagtaaaggatccatggataaagata**

**Fortunella_hindsii_1** **tgaattggatcaatacttccaattttttaagtataagtaaaggatccatggataaagata**

**Fortunella_hindsii_2** **tgaattggatcaatacttccaattttttaagtataagtaaaggatccatggataaagata**

**Fortunella_hindsii_3** **tgaattggatcaatacttccaattttttaagtataagtaaaggatccatggataaagata**

**Fortunella_japonica_1** **tgaattggatcaatacttccaattttttaagtataagtaaaggatccatggataaagata**

**Fortunella_japonica_2** **tgaattggatcaatacttccaattttttaagtataagtaaaggatccatggataaagata**

**Fortunella_japonica_3** **tgaattggatcaatacttccaattttttaagtataagtaaaggatccatggataaagata**

**Fortunella_japonica_4** **tgaattggatcaatacttccaattttttaagtataagtaaaggatccatggataaagata**

**Fortunella_japonica_5** **tgaattggatcaatacttccaattttttaagtataagtaaaggatccatggataaagata**

**Fortunella_japonica_6** **tgaattggatcaatacttccaattttttaagtataagtaaaggatccatggataaagata**

**Fortunella_japonica_7** **tgaattggatcaatacttccaattttttaagtataagtaaaggatccatggataaagata**

**Fortunella_japonica_8** **tgaattggatcaatacttccaattttttaagtataagtaaaggatccatggataaagata**

**Fortunella_japonica_9** **tgaattggatcaatacttccaattttttaagtataagtaaaggatccatggataaagata**

**Fortunella_japonica_10** **tgaattggatcaatacttccaattttttaagtataagtaaaggatccatggataaagata**

**Fortunella_japonica_11** **tgaattggatcaatacttccaattttttaagtataagtaaaggatccatggataaagata**

**Fortunella_japonica_12** **tgaattggatcaatacttccaattttttaagtataagtaaaggatccatggataaagata**

**Fortunella_margarita_1** **tgaattggatcaatacttccaattttttaagtataagtaaaggatccatggataaagata**

**Fortunella_margarita_2** **tgaattggatcaatacttccaattttttaagtataagtaaaggatccatggataaagata**

**Fortunella_venosa_1** **tgaattggatcaatacttccaattttttaagtataagtaaaggatccatggataaagata**

**Fortunella_venosa_2** **tgaattggatcaatacttccaattttttaagtataagtaaaggatccatggataaagata**

**Fortunella_venosa_3** **tgaattggatcaatacttccaattttttaagtataagtaaaggatccatggataaagata**

10630	10640	10650	10660	10670	10680

....|....|....|....|....|....|....|....|....|....|....|....|

**Atalantia_kwangtungens** **gaaaagtctagttcgaatcgtaactaaatcttcaattttggttttttataggttagattg**

**Citrus_reticulata_1** **gaaaagtctagttcgaatcgtaactaaatcttcaattttggttttttataggttagattg**

**Citrus_reticulata_2** **gaaaagtctagttcgaatcgtaactaaatcttcaattttggttttttataggttagattg**

**Citrus_trifoliata_3** **gaaaagtctagtttgaatcgtaactaaatcttcaattttggttttttataggttagattg**

**Citrus_aurantiifolia** **gaaaagtctagttcgaatcgtaactaaatcttcaattttggttttttataggttagattg**

**Citrus_aurantium** **gaaaagtctagttcgaatcgtaactaaatcttcaattttggttttttataggttagattg**

**Citrus_cavaleriei** **gaaaagtctagttcgaatcgtaactaaatcttcaattttggttttttataggttagattg**

**Citrus_hongheensis** **gaaaagtctagttcgaatcgtaactaaatcttcaattttggttttttataggttagattg**

**Citrus_limon** **gaaaagtctagttcgaatcgtaactaaatcttcaattttggttttttataggttagattg**

**Citrus_maxima** **gaaaagtctagttcgaatcgtaactaaatcttcaattttggttttttataggttagattg**

**Citrus_medica** **gaaaagtctagttcgaatcgtaactaaatcttcaattttggttttttataggttagattg**

**Citrus_polytrifolia** **gaaaagtctagtttgaatcgtaactaaatcttcaattttggttttttataggttagattg**

**Citrus_reticulata** **gaaaagtctagttcgaatcgtaactaaatcttcaattttggttttttataggttagattg**

**Citrus_sinensis** **gaaaagtctagttcgaatcgtaactaaatcttcaattttggttttttataggttagattg**

**Fortunella_bawangica_1** **gaaaagtctagttcgaatcgtaactaaatcttcaattttggttttttataggttagattg**

**Fortunella_bawangica_2** **gaaaagtctagttcgaatcgtaactaaatcttcaattttggttttttataggttagattg**

**Fortunella_hindsii_1** **gaaaagtctagttcgaatcgtaactaaatcttcaattttggttttttataggttagattg**

**Fortunella_hindsii_2** **gaaaagtctagttcgaatcgtaactaaatcttcaattttggttttttataggttagattg**

**Fortunella_hindsii_3** **gaaaagtctagttcgaatcgtaactaaatcttcaattttggttttttataggttagattg**

**Fortunella_japonica_1** **gaaaagtctagttcgaatcgtaactaaatcttcaattttggttttttataggttagattg**

**Fortunella_japonica_2** **gaaaagtctagttcgaatcgtaactaaatcttcaattttggttttttctaggttagattg**

**Fortunella_japonica_3** **gaaaagtctagttcgaatcgtaactaaatcttcaattttggttttttataggttagattg**

**Fortunella_japonica_4** **gaaaagtctagttcgaatcgtaactaaatcttcaattttggttttttataggttagattg**

**Fortunella_japonica_5** **gaaaagtctagttcgaatcgtaactaaatcttcaattttggttttttataggttagattg**

**Fortunella_japonica_6** **gaaaagtctagttcgaatcgtaactaaatcttcaattttggttttttataggttagattg**

**Fortunella_japonica_7** **gaaaagtctagttcgaatcgtaactaaatcttcaattttggttttttataggttagattg**

**Fortunella_japonica_8** **gaaaagtctagttcgaatcgtaactaaatcttcaattttggttttttataggttagattg**

**Fortunella_japonica_9** **gaaaagtctagttcgaatcgtaactaaatcttcaattttggttttttataggttagattg**

**Fortunella_japonica_10** **gaaaagtctagttcgaatcgtaactaaatcttcaattttggttttttataggttagattg**

**Fortunella_japonica_11** **gaaaagtctagttcgaatcgtaactaaatcttcaattttggttttttataggttagattg**

**Fortunella_japonica_12** **gaaaagtctagttcgaatcgtaactaaatcttcaattttggttttttataggttagattg**

**Fortunella_margarita_1** **gaaaagtctagttcgaatcgtaactaaatcttcaattttggttttttataggttagattg**

**Fortunella_margarita_2** **gaaaagtctagttcgaatcgtaactaaatcttcaattttggttttttataggttagattg**

**Fortunella_venosa_1** **gaaaagtctagttcgaatcgtaactaaatcttcaattttggttttttataggttagattg**

**Fortunella_venosa_2** **gaaaagtctagttcgaatcgtaactaaatcttcaattttggttttttataggttagattg**

**Fortunella_venosa_3** **gaaaagtctagttcgaatcgtaactaaatcttcaattttggttttttataggttagattg**

10690	10700	10710	10720	10730	10740

....|....|....|....|....|....|....|....|....|....|....|....|

**Atalantia_kwangtungens** **aagcaaaatagctattaaatgatcactctagtttactagagacatcaccatattctatta**

**Citrus_reticulata_1** **aagcaaaatagctattaaatgatcactctagtttactagagacatcaacatattctat--**

**Citrus_reticulata_2** **aagcaaaatagctattaaatgatcactctagtttactagagacatcaacatattctat--**

**Citrus_trifoliata_3** **aagcaaaatagctattaaatgatcactctagtttactagagacatcaacgtattctat--**

**Citrus_aurantiifolia** **aagcaaaatagctattaaatgatcactctagtttactagagacatcaacgtattctat--**

**Citrus_aurantium** **aagcaaaatagctattaaatgatcactctagtttactagagacatcaacgtattctat--**

**Citrus_cavaleriei** **aagcaaaatagctattaaatgatcactctagtttactagagacatcaacgtattctat--**

**Citrus_hongheensis** **aagcaaaatagctattaaatgatcactctagtttactagagacatcaacgtattctat--**

**Citrus_limon** **aagcaaaatagctattaaatgatcactctagtttactagagacatcaacgtattctat--**

**Citrus_maxima** **aagcaaaatagctattaaatgatcactctagtttactagagacatcaacgtattctat--**

**Citrus_medica** **aagcaaaatagctattaaatgatcactctagtttactagagacatcaacgtattctat--**

**Citrus_polytrifolia** **aagcaaaatagctattaaatgatcactctagtttactagagacatcaacgtattctat--**

**Citrus_reticulata** **aagcaaaatagctattaaatgatcactctagtttactagagacatcaacatattctat--**

**Citrus_sinensis** **aagcaaaatagctattaaatgatcactctagtttactagagacatcaacgtattctat--**

**Fortunella_bawangica_1** **aagcaaaatagctattaaatgatcactctagtttactagagacatcaacgtattctat--**

**Fortunella_bawangica_2** **aagcaaaatagctattaaatgatcactctagtttactagagacatcaacgtattctat--**

**Fortunella_hindsii_1** **aagcaaaatagctattaaatgatcactctagtttactagagacatcaacgtattctat--**

**Fortunella_hindsii_2** **aagcaaaatagctattaaatgatcactctagtttactagagacatcaacgtattctat--**

**Fortunella_hindsii_3** **aagcaaaatagctattaaatgatcactctagtttactagagacatcaacgtattctat--**

**Fortunella_japonica_1** **aagcaaaatagctattaaatgatcactctagtttactagagacatcaacgtattctat--**

**Fortunella_japonica_2** **aagcaaaatagctattaaatgatcactctagtttactagagacatcaacgtattctat--**

**Fortunella_japonica_3** **aagcaaaatagctattaaatgatcactctagtttactagagacatcaacgtattctat--**

**Fortunella_japonica_4** **aagcaaaatagctattaaatgatcactctagtttactagagacatcaacgtattctat--**

**Fortunella_japonica_5** **aagcaaaatagctattaaatgatcactctagtttactagagacatcaacgtattctat--**

**Fortunella_japonica_6** **aagcaaaatagctattaaatgatcactctagtttactagagacatcaacgtattctat--**

**Fortunella_japonica_7** **aagcaaaatagctattaaatgatcactctagtttactagagacatcaacgtattctat--**

**Fortunella_japonica_8** **aagcaaaatagctattaaatgatcactctagtttactagagacatcaacgtattctat--**

**Fortunella_japonica_9** **aagcaaaatagctattaaatgatcactctagtttactagagacatcaacgtattctat--**

**Fortunella_japonica_10** **aagcaaaatagctattaaatgatcactctagtttactagagacatcaacgtattctat--**

**Fortunella_japonica_11** **aagcaaaatagctattaaatgatcactctagtttactagagacatcaacgtattctat--**

**Fortunella_japonica_12** **aagcaaaatagctattaaatgatcactctagtttactagagacatcaacgtattctat--**

**Fortunella_margarita_1** **aagcaaaatagctattaaatgatcactctagtttactagagacatcaacgtattctat--**

**Fortunella_margarita_2** **aagcaaaatagctattaaatgatcactctagtttactagagacatcaacgtattctat--**

**Fortunella_venosa_1** **aagcaaaatagctattaaatgatcactctagtttactagagacatcaacgtattctat--**

**Fortunella_venosa_2** **aagcaaaatagctattaaatgatcactctagtttactagagacatcaacgtattctat--**

**Fortunella_venosa_3** **aagcaaaatagctattaaatgatcactctagtttactagagacatcaacgtattctat--**

10750	10760	10770	10780	10790	10800

....|....|....|....|....|....|....|....|....|....|....|....|

**Atalantia_kwangtungens** **aatgatcactctagtttactagagacatcaccatattctattttttagtttagctcgggg**

**Citrus_reticulata_1** **----------------------------------------ttttttagtttagctcgggg**

**Citrus_reticulata_2** **----------------------------------------ttttttagtttagctcgggg**

**Citrus_trifoliata_3** **----------------------------------------ttttttagtttagctcgggg**

**Citrus_aurantiifolia** **----------------------------------------ttttttagtttagctcgggg**

**Citrus_aurantium** **----------------------------------------ttttttagtttagctcgggg**

**Citrus_cavaleriei** **----------------------------------------ttttttagtttagctcgggg**

**Citrus_hongheensis** **----------------------------------------ttttttagtttagctcgggg**

**Citrus_limon** **----------------------------------------ttttttagtttagctcgggg**

**Citrus_maxima** **----------------------------------------ttttttagtttagctcgggg**

**Citrus_medica** **----------------------------------------ttttttagtttagctcgggg**

**Citrus_polytrifolia** **----------------------------------------ttttttagtttagctcgggg**

**Citrus_reticulata** **----------------------------------------ttttttagtttagctcgggg**

**Citrus_sinensis** **----------------------------------------ttttttagtttagctcgggg**

**Fortunella_bawangica_1** **----------------------------------------ttttttagtttagctcgggg**

**Fortunella_bawangica_2** **----------------------------------------ttttttagtttagctcgggg**

**Fortunella_hindsii_1** **----------------------------------------ttttttagtttagctcgggg**

**Fortunella_hindsii_2** **----------------------------------------ttttttagtttagctcgggg**

**Fortunella_hindsii_3** **----------------------------------------ttttttagtttagctcgggg**

**Fortunella_japonica_1** **----------------------------------------tttttgagtttagctcgggg**

**Fortunella_japonica_2** **----------------------------------------tttttgagtttagctcgggg**

**Fortunella_japonica_3** **----------------------------------------tttttgagtttagctcgggg**

**Fortunella_japonica_4** **----------------------------------------tttttgagtttagctcgggg**

**Fortunella_japonica_5** **----------------------------------------tttttgagtttagctcgggg**

**Fortunella_japonica_6** **----------------------------------------tttttgagtttagctcgggg**

**Fortunella_japonica_7** **----------------------------------------tttttgagtttagctcgggg**

**Fortunella_japonica_8** **----------------------------------------tttttgagtttagctcgggg**

**Fortunella_japonica_9** **----------------------------------------tttttgagtttagctcgggg**

**Fortunella_japonica_10** **----------------------------------------ttttttagtttagctcgggg**

**Fortunella_japonica_11** **----------------------------------------tttttgagtttagctcgggg**

**Fortunella_japonica_12** **----------------------------------------tttttgagtttagctcgggg**

**Fortunella_margarita_1** **----------------------------------------ttttttagtttagctcgggg**

**Fortunella_margarita_2** **----------------------------------------ttttttagtttagctcgggg**

**Fortunella_venosa_1** **----------------------------------------ttttttagtttagctcgggg**

**Fortunella_venosa_2** **----------------------------------------ttttttagtttagctcgggg**

**Fortunella_venosa_3** **----------------------------------------ttttttagtttagctcgggg**

10810	10820	10830	10840	10850	10860

....|....|....|....|....|....|....|....|....|....|....|....|

**Atalantia_kwangtungens** **gaaacaaaaaacttttcctaaggattctttcaaatagaaatagagaacgaagtaactaga**

**Citrus_reticulata_1** **gaaacaaaaaacttttcctaaggattctttcaaatagaaatagagaacgaagtaactaga**

**Citrus_reticulata_2** **gaaacaaaaaacttttcctaaggattctttcaaatagaaatagagaacgaagtaactaga**

**Citrus_trifoliata_3** **gaaacaaaaaacttttcctaaggattctttcaaatagaaatagagaacgaagtaactaga**

**Citrus_aurantiifolia** **gaaacaaaaaacttttcctaaggattctttcaaatagaaatagagaacgaagtaactaga**

**Citrus_aurantium** **gaaacaaaaaacttttcctaaggattctttcaaatagaaatagagaacgaagtaactaga**

**Citrus_cavaleriei** **gaaacaaaaaacttttcctaaggattctttcaaatagaaatagagaacgaagtaactaga**

**Citrus_hongheensis** **gaaacaaaaaacttttcctaaggattctttcaaatagaaatagagaacgaagtaactaga**

**Citrus_limon** **gaaacaaaaaacttttcctaaggattctttcaaatagaaatagagaacgaagtaactaga**

**Citrus_maxima** **gaaacaaaaaacttttcctaaggattctttcaaatagaaatagagaacgaagtaactaga**

**Citrus_medica** **gaaacaaaaaacttttcctaaggattctttcaaatagaaatagagaacgaagtaactaga**

**Citrus_polytrifolia** **gaaacaaaaaacttttcctaaggattctttcaaatagaaatagagaacgaagtaactaga**

**Citrus_reticulata** **gaaacaaaaaacttttcctaaggattctttcaaatagaaatagagaacgaagtaactaga**

**Citrus_sinensis** **gaaacaaaaaacttttcctaaggattctttcaaatagaaatagagaacgaagtaactaga**

**Fortunella_bawangica_1** **gaaacaaaaaacttttcctaaggattctttcaaatagaaatagagaacgaagtaactaga**

**Fortunella_bawangica_2** **gaaacaaaaaacttttcctaaggattctttcaaatagaaatagagaacgaagtaactaga**

**Fortunella_hindsii_1** **gaaacaaaaaacttttcctaaggattctttcaaatagaaatagagaacgaagtaactaga**

**Fortunella_hindsii_2** **gaaacaaaaaacttttcctaaggattctttcaaatagaaatagagaacgaagtaactaga**

**Fortunella_hindsii_3** **gaaacaaaaaacttttcctaaggattctttcaaatagaaatagagaacgaagtaactaga**

**Fortunella_japonica_1** **gaaacaaaaaacttttcctaaggattctttcaaatagaaatagagaacgaagtaactaga**

**Fortunella_japonica_2** **gaaacaaaaaacttttcctaaggattctttcaaatagaaatagagaacgaagtaactaga**

**Fortunella_japonica_3** **gaaacaaaaaacttttcctaaggattctttcaaatagaaatagagaacgaagtaactaga**

**Fortunella_japonica_4** **gaaacaaaaaacttttcctaaggattctttcaaatagaaatagagaacgaagtaactaga**

**Fortunella_japonica_5** **gaaacaaaaaacttttcctaaggattctttcaaatagaaatagagaacgaagtaactaga**

**Fortunella_japonica_6** **gaaacaaaaaacttttcctaaggattctttcaaatagaaatagagaacgaagtaactaga**

**Fortunella_japonica_7** **gaaacaaaaaacttttcctaaggattctttcaaatagaaatagagaacgaagtaactaga**

**Fortunella_japonica_8** **gaaacaaaaaacttttcctaaggattctttcaaatagaaatagagaacgaagtaactaga**

**Fortunella_japonica_9** **gaaacaaaaaacttttcctaaggattctttcaaatagaaatagagaacgaagtaactaga**

**Fortunella_japonica_10** **gaaacaaaaaacttttcctaaggattctttcaaatagaaatagagaacgaagtaactaga**

**Fortunella_japonica_11** **gaaacaaaaaacttttcctaaggattctttcaaatagaaatagagaacgaagtaactaga**

**Fortunella_japonica_12** **gaaacaaaaaacttttcctaaggattctttcaaatagaaatagagaacgaagtaactaga**

**Fortunella_margarita_1** **gaaacaaaaaacttttcctaaggattctttcaaatagaaatagagaacgaagtaactaga**

**Fortunella_margarita_2** **gaaacaaaaaacttttcctaaggattctttcaaatagaaatagagaacgaagtaactaga**

**Fortunella_venosa_1** **gaaacaaaaaacttttcctaaggattctttcaaatagaaatagagaacgaagtaactaga**

**Fortunella_venosa_2** **gaaacaaaaaacttttcctaaggattctttcaaatagaaatagagaacgaagtaactaga**

**Fortunella_venosa_3** **gaaacaaaaaacttttcctaaggattctttcaaatagaaatagagaacgaagtaactaga**

10870	10880	10890	10900	10910	10920

....|....|....|....|....|....|....|....|....|....|....|....|

**Atalantia_kwangtungens** **aagagtgggattgttataatcctactcttctagagggatcatctagaaagcagattgttt**

**Citrus_reticulata_1** **aagattggtattgttataatcccactcttctagagggatcatctagaaagcggattgttt**

**Citrus_reticulata_2** **aagattggtattgttataatcccactcttctagagggatcatctagaaagcggattgttt**

**Citrus_trifoliata_3** **aagattggtattgttataatcccactcttctagagggatcatctagaaagcggattgttt**

**Citrus_aurantiifolia** **aagattggtattgttataatcccactcttctagagggatcatctagaaagcggattgttt**

**Citrus_aurantium** **aagattggtattgttataatcccactcttctagagggatcatctagaaagcggattgttt**

**Citrus_cavaleriei** **aagattggtattgttataatcccactcttctagagggatcatctagaaagcggattgttt**

**Citrus_hongheensis** **aagattggtattgttataatcccactcttctagagggatcatctagaaagcggattgttt**

**Citrus_limon** **aagattggtattgttataatcccactcttctagagggatcatctagaaagcggattgttt**

**Citrus_maxima** **aagattggtattgttataatcccactcttctagagggatcatctagaaagcggattgttt**

**Citrus_medica** **aagattggtattgttataatcccactcttctagagggatcatctagaaagcggattgttt**

**Citrus_polytrifolia** **aagattggtattgttataatcccactcttctagagggatcatctagaaagcggatttttt**

**Citrus_reticulata** **aagattggtattgttataatcccactcttctagagggatcatctagaaagcggattgttt**

**Citrus_sinensis** **aagattggtattgttataatcccactcttctagagggatcatctagaaagcggattgttt**

**Fortunella_bawangica_1** **aagattggtattgttataatccccctcttctagagggatcatctagaaagcggattgttt**

**Fortunella_bawangica_2** **aagattggtattgttataatccccctcttctagagggatcatctagaaagcggattgttt**

**Fortunella_hindsii_1** **aagattggtattgttataatccccctcttctagagggatcatctagaaagcggattgttt**

**Fortunella_hindsii_2** **aagattggtattgttataatccccctcttctagagggatcatctagaaagcggattgttt**

**Fortunella_hindsii_3** **aagattggtattgttataatccccctcttctagagggatcatctagaaagcggattgttt**

**Fortunella_japonica_1** **aagattggtattgttataatccccctcttctagagggatcatctagaaagcggattgttt**

**Fortunella_japonica_2** **aagattggtattgttataatccccctcttctagagggatcatctagaaagcggattgttt**

**Fortunella_japonica_3** **aagattggtattgttataatccccctcttctagagggatcatctagaaagcggattgttt**

**Fortunella_japonica_4** **aagattggtattgttataatccccctcttctagagggatcatctagaaagcggattgttt**

**Fortunella_japonica_5** **aagattggtattgttataatccccctcttctagagggatcatctagaaagcggattgttt**

**Fortunella_japonica_6** **aagattggtattgttataatccccctcttctagagggatcatctagaaagcggattgttt**

**Fortunella_japonica_7** **aagattggtattgttataatccccctcttctagagggatcatctagaaagcggattgttt**

**Fortunella_japonica_8** **aagattggtattgttataatccccctcttctagagggatcatctagaaagcggattgttt**

**Fortunella_japonica_9** **aagattggtattgttataatccccctcttctagagggatcatctagaaagcggattgttt**

**Fortunella_japonica_10** **aagattggtattgttataatccccctcttctagagggatcatctagaaagcggattgttt**

**Fortunella_japonica_11** **aagattggtattgttataatccccctcttctagagggatcatctagaaagcggattgttt**

**Fortunella_japonica_12** **aagattggtattgttataatccccctcttctagagggatcatctagaaagcggattgttt**

**Fortunella_margarita_1** **aagattggtattgttataatccccctcttctagagggatcatctagaaagcggattgttt**

**Fortunella_margarita_2** **aagattggtattgttataatccccctcttctagagggatcatctagaaagcggattgttt**

**Fortunella_venosa_1** **aagattggtattgttataatccccctcttctagagggatcatctagaaagcggattgttt**

**Fortunella_venosa_2** **aagattggtattgttataatccccctcttctagagggatcatctagaaagcggattgttt**

**Fortunella_venosa_3** **aagattggtattgttataatccccctcttctagagggatcatctagaaagcggattgttt**

10930	10940	10950	10960	10970	10980

....|....|....|....|....|....|....|....|....|....|....|....|

**Atalantia_kwangtungens** **tgaattcattcagacaaaaaaagctgacatagatgttatgggtcgacttttgttattttg**

**Citrus_reticulata_1** **tgaattcattcagacaaaaaaagctgacatagatgttatgggtcgacttttgttattttg**

**Citrus_reticulata_2** **tgaattcattcagacaaaaaaagctgacatagatgttatgggtcgacttttgttattttg**

**Citrus_trifoliata_3** **tgaattcattcagacaaaaaaagctgacatagatgttatgggtcgacttttgtta-tttg**

**Citrus_aurantiifolia** **tgaattcattcagacaaaaaaagctgacatagatgttatgggtcgacttttgttattttg**

**Citrus_aurantium** **tgaattcattcagacaaaaaaagctgacatagatgttatgggtcgacttttgttattttg**

**Citrus_cavaleriei** **tgaattcattcagacaaaaaaagctgacatagatgttatgggtcgacttttgttattttg**

**Citrus_hongheensis** **tgaattcattcagacaaaaaaagctgacatagatgttatgggtcgacttttgttattttg**

**Citrus_limon** **tgaattcattcagacaaaaaaagctgacatagatgttatgggtcgacttttgttattttg**

**Citrus_maxima** **tgaattcattcagacaaaaaaagctgacatagatgttatgggtcgacttttgttattttg**

**Citrus_medica** **tgaattcattcagacaaaaaaagctgacatagatgttatgggtcgacttttgttattttg**

**Citrus_polytrifolia** **tgaattcattcagacaaaaaaagctgacatagatgttatgggtcgacttttgtta-tttg**

**Citrus_reticulata** **tgaattcattcagacaaaaaaagctgacatagatgttatgggtcgacttttgttattttg**

**Citrus_sinensis** **tgaattcattcagacaaaaaaagctgacatagatgttatgggtcgacttttgttattttg**

**Fortunella_bawangica_1** **tgaattcattcagacaaaaaaagctgacatagatgttatgggtcgacttttgttattttg**

**Fortunella_bawangica_2** **tgaattcattcagacaaaaaaagctgacatagatgttatgggtcgacttttgttattttg**

**Fortunella_hindsii_1** **tgaattcattcagacaaaaaaagctgacatagatgttatgggtcgacttttgttattttg**

**Fortunella_hindsii_2** **tgaattcattcagacaaaaaaagctgacatagatgttatgggtcgacttttgttattttg**

**Fortunella_hindsii_3** **tgaattcattcagacaaaaaaagctgacatagatgttatgggtcgacttttgttattttg**

**Fortunella_japonica_1** **tgaattcattcagacaaaaaaagctgacatagatgttatgggtcgacttttgttattttg**

**Fortunella_japonica_2** **tgaattcattcagacaaaaaaagctgacatagatgttatgggtcgacttttgttattttg**

**Fortunella_japonica_3** **tgaattcattcagacaaaaaaagctgacatagatgttatgggtcgacttttgttattttg**

**Fortunella_japonica_4** **tgaattcattcagacaaaaaaagctgacatagatgttatgggtcgacttttgttattttg**

**Fortunella_japonica_5** **tgaattcattcagacaaaaaaagctgacatagatgttatgggtcgacttttgttattttg**

**Fortunella_japonica_6** **tgaattcattcagacaaaaaaagctgacatagatgttatgggtcgacttttgttattttg**

**Fortunella_japonica_7** **tgaattcattcagacaaaaaaagctgacatagatgttatgggtcgacttttgttattttg**

**Fortunella_japonica_8** **tgaattcattcagacaaaaaaagctgacatagatgttatgggtcgacttttgttattttg**

**Fortunella_japonica_9** **tgaattcattcagacaaaaaaagctgacatagatgttatgggtcgacttttgttattttg**

**Fortunella_japonica_10** **tgaattcattcagacaaaaaaagctgacatagatgttatgggtcgacttttgttattttg**

**Fortunella_japonica_11** **tgaattcattcagacaaaaaaagctgacatagatgttatgggtcgacttttgttattttg**

**Fortunella_japonica_12** **tgaattcattcagacaaaaaaagctgacatagatgttatgggtcgacttttgttattttg**

**Fortunella_margarita_1** **tgaattcattcagacaaaaaaagctgacatagatgttatgggtcgacttttgttattttg**

**Fortunella_margarita_2** **tgaattcattcagacaaaaaaagctgacatagatgttatgggtcgacttttgttattttg**

**Fortunella_venosa_1** **tgaattcattcagacaaaaaaagctgacatagatgttatgggtcgacttttgttattttg**

**Fortunella_venosa_2** **tgaattcattcagacaaaaaaagctgacatagatgttatgggtcgacttttgttattttg**

**Fortunella_venosa_3** **tgaattcattcagacaaaaaaagctgacatagatgttatgggtcgacttttgttattttg**

10990	11000	11010	11020	11030	11040

....|....|....|....|....|....|....|....|....|....|....|....|

**Atalantia_kwangtungens** **atcccggcttctctctgggaatatatcaaacttccataaaggagccgaatgaccccaaag**

**Citrus_reticulata_1** **ctcccggcttctctctgggaatatagcaaacttccataaaggagccgaatgaccccaaag**

**Citrus_reticulata_2** **ctcccggcttctctctgggaatatagcaaacttccataaaggagccgaatgaccccaaag**

**Citrus_trifoliata_3** **ctcccggcttctctctgggaatatagcaaacttccataaaggagccgaatgaccccaaag**

**Citrus_aurantiifolia** **ctcccggcttctctctgggaatatagcaaacttccataaaggagccgaatgaccccaaag**

**Citrus_aurantium** **ctcccggcttctctctgggaatatagcaaacttccataaaggagccgaatgaccccaaag**

**Citrus_cavaleriei** **ctcccggcttctctctgggaatatagcaaacttccataaaggagccgaatgaccccaaag**

**Citrus_hongheensis** **ctcccggcttctctctgggaatatagcaaacttccataaaggagccgaatgaccccaaag**

**Citrus_limon** **ctcccggcttctctctgggaatatagcaaacttccataaaggagccgaatgaccccaaag**

**Citrus_maxima** **ctcccggcttctctctgggaatatagcaaacttccataaaggagccgaatgaccccaaag**

**Citrus_medica** **ctcccggcttctctctgggaatatagcaaacttccataaaggagccgaatgaccccaaag**

**Citrus_polytrifolia** **ctcccggcttctctctgggaatatagcaaacttccataaaggagccgaatgaccccaaag**

**Citrus_reticulata** **ctcccggcttctctctgggaatatagcaaacttccataaaggagccgaatgaccccaaag**

**Citrus_sinensis** **ctcccggcttctctctgggaatatagcaaacttccataaaggagccgaatgaccccaaag**

**Fortunella_bawangica_1** **ctcccggcttctctctgggaatatagcaaacttccataaaggagccgaatgaacccaaag**

**Fortunella_bawangica_2** **ctcccggcttctctctgggaatatagcaaacttccataaaggagccgaatgaacccaaag**

**Fortunella_hindsii_1** **ctcccggcttctctctgggaatatagcaaacttccataaaggagccgaatgaacccaaag**

**Fortunella_hindsii_2** **ctcccggcttctctctgggaatatagcaaacttccataaaggagccgaatgaacccaaag**

**Fortunella_hindsii_3** **ctcccggcttctctctgggaatatagcaaacttccataaaggagccgaatgaacccaaag**

**Fortunella_japonica_1** **ctcccggcttctctctgggaatatagcaaacttccataaaggagccgaatgaacccaaag**

**Fortunella_japonica_2** **ctcccggcttctctctgggaatatagcaaacttccataaaggagccgaatgaacccaaag**

**Fortunella_japonica_3** **ctcccggcttctctctgggaatatagcaaacttccataaaggagccgaatgaacccaaag**

**Fortunella_japonica_4** **ctcccggcttctctctgggaatatagcaaacttccataaaggagccgaatgaacccaaag**

**Fortunella_japonica_5** **ctcccggcttctctctgggaatatagcaaacttccataaaggagccgaatgaacccaaag**

**Fortunella_japonica_6** **ctcccggcttctctctgggaatatagcaaacttccataaaggagccgaatgaacccaaag**

**Fortunella_japonica_7** **ctcccggcttctctctgggaatatagcaaacttccataaaggagccgaatgaacccaaag**

**Fortunella_japonica_8** **ctcccggcttctctctgggaatatagcaaacttccataaaggagccgaatgaacccaaag**

**Fortunella_japonica_9** **ctcccggcttctctctgggaatatagcaaacttccataaaggagccgaatgaacccaaag**

**Fortunella_japonica_10** **ctcccggcttctctctgggaatatagcaaacttccataaaggagccgaatgaacccaaag**

**Fortunella_japonica_11** **ctcccggcttctctctgggaatatagcaaacttccataaaggagccgaatgaacccaaag**

**Fortunella_japonica_12** **ctcccggcttctctctgggaatatagcaaacttccataaaggagccgaatgaacccaaag**

**Fortunella_margarita_1** **ctcccggcttctctctgggaatatagcaaacttccataaaggagccgaatgaacccaaag**

**Fortunella_margarita_2** **ctcccggcttctctctgggaatatagcaaacttccataaaggagccgaatgaacccaaag**

**Fortunella_venosa_1** **ctcccggcttctctctgggaatatagcaaacttccataaaggagccgaatgaacccaaag**

**Fortunella_venosa_2** **ctcccggcttctctctgggaatatagcaaacttccataaaggagccgaatgaacccaaag**

**Fortunella_venosa_3** **ctcccggcttctctctgggaatatagcaaacttccataaaggagccgaatgaacccaaag**

11050	11060	11070	11080	11090	11100

....|....|....|....|....|....|....|....|....|....|....|....|

**Atalantia_kwangtungens** **tttcatgttcggttttgaattagcggcgtt-aaaaagtatgaatcgacgtcgactataac**

**Citrus_reticulata_1** **tttcatgttcggttttgaattagcggcgtt-aaaaagtatgaatcgacgtctactataac**

**Citrus_reticulata_2** **tttcatgttcggttttgaattagcggcgtt-aaaaagtatgaatcgacgtctactataac**

**Citrus_trifoliata_3** **tttcatgttcggttttgaattagcggcgtt-aaaaagtatgaatcgacgtctactataac**

**Citrus_aurantiifolia** **tttcatgttcggttttgaattagcggcgttaaaaaagtatgaatcgacgtctactataac**

**Citrus_aurantium** **tttcatgttcggttttgaattagcggcgtt-aaaaagtatgaatcgacgtctactataac**

**Citrus_cavaleriei** **tttcatgttcggttttgaattagcggcgtt-aaaaagtatgaatcgacgtctactataac**

**Citrus_hongheensis** **tttcatgttcggttttgaattagcggcgtt-aaaaagtatgaatcgacgtctactataac**

**Citrus_limon** **tttcatgttcggttttgaattagcggcgtt-aaaaagtatgaatcgacgtctactataac**

**Citrus_maxima** **tttcatgttcggttttgaattagcggcgtt-aaaaagtatgaatcgacgtctactataac**

**Citrus_medica** **tttcatgttcggttttgaattagcggcgtt-aaaaagtatgaatcgacgtctactataac**

**Citrus_polytrifolia** **tttcatgttcggttttgaattagcggcgtt-aaaaagtatgaatcgacgtctactataac**

**Citrus_reticulata** **tttcatgttcggttttgaattagcggcgtt-aaaaagtatgaatcgacgtctactataac**

**Citrus_sinensis** **tttcatgttcggttttgaattagcggcgtt-aaaaagtatgaatcgacgtctactataac**

**Fortunella_bawangica_1** **tttcatgttcggttttgaattagcggcgtt-aaaaagtatgaatcgacgtctactataac**

**Fortunella_bawangica_2** **tttcatgttcggttttgaattagcggcgtt-aaaaagtatgaatcgacgtctactataac**

**Fortunella_hindsii_1** **tttcatgttcggttttgaattagcggcgtt-aaaaagtatgaatcgacgtctactataac**

**Fortunella_hindsii_2** **tttcatgttcggttttgaattagcggcgtt-aaaaagtatgaatcgacgtctactataac**

**Fortunella_hindsii_3** **tttcatgttcggttttgaattagcggcgtt-aaaaagtatgaatcgacgtctactataac**

**Fortunella_japonica_1** **tttcatgttcggttttgaattagcggcgtt-aaaaagtatgaatcgacgtctactataac**

**Fortunella_japonica_2** **tttcatgttcggttttgaattagcggcgtt-aaaaagtatgaatcgacgtctactataac**

**Fortunella_japonica_3** **tttcatgttcggttttgaattagcggcgtt-aaaaagtatgaatcgacgtctactataac**

**Fortunella_japonica_4** **tttcatgttcggttttgaattagcggcgtt-aaaaagtatgaatcgacgtctactataac**

**Fortunella_japonica_5** **tttcatgttcggttttgaattagcggcgtt-aaaaagtatgaatcgacgtctactataac**

**Fortunella_japonica_6** **tttcatgttcggttttgaattagcggcgtt-aaaaagtatgaatcgacgtctactataac**

**Fortunella_japonica_7** **tttcatgttcggttttgaattagcggcgtt-aaaaagtatgaatcgacgtctactataac**

**Fortunella_japonica_8** **tttcatgttcggttttgaattagcggcgtt-aaaaagtatgaatcgacgtctactataac**

**Fortunella_japonica_9** **tttcatgttcggttttgaattagcggcgtt-aaaaagtatgaatcgacgtctactataac**

**Fortunella_japonica_10** **tttcatgttcggttttgaattagcggcgtt-aaaaagtatgaatcgacgtctactataac**

**Fortunella_japonica_11** **tttcatgttcggttttgaattagcggcgtt-aaaaagtatgaatcgacgtctactataac**

**Fortunella_japonica_12** **tttcatgttcggttttgaattagcggcgtt-aaaaagtatgaatcgacgtctactataac**

**Fortunella_margarita_1** **tttcatgttcggttttgaattagcggcgtt-aaaaagtatgaatcgacgtctactataac**

**Fortunella_margarita_2** **tttcatgttcggttttgaattagcggcgtt-aaaaagtatgaatcgacgtctactataac**

**Fortunella_venosa_1** **tttcatgttcggttttgaattagcggcgtt-aaaaagtatgaatcgacgtctactataac**

**Fortunella_venosa_2** **tttcatgttcggttttgaattagcggcgtt-aaaaagtatgaatcgacgtctactataac**

**Fortunella_venosa_3** **tttcatgttcggttttgaattagcggcgtt-aaaaagtatgaatcgacgtctactataac**

11110	11120	11130	11140	11150	11160

....|....|....|....|....|....|....|....|....|....|....|....|

**Atalantia_kwangtungens** **ccctagccttccaagctaacgatgcgggttcgattcccgctacccgctccatatgataat**

**Citrus_reticulata_1** **ccctagccttccaagctaacgatgcgggttcgattcccgctacccgctccatatgataat**

**Citrus_reticulata_2** **ccctagccttccaagctaacgatgcgggttcgattcccgctacccgctccatatgataat**

**Citrus_trifoliata_3** **ccctagccttccaagctaacgatgcgggttcgattcccgctacccgctccatatgataat**

**Citrus_aurantiifolia** **ccctagccttccaagctaacgatgcgggttcgattcccgctacccgctccatatgataat**

**Citrus_aurantium** **ccctagccttccaagctaacgatgcgggttcgattcccgctacccgctccatatgataat**

**Citrus_cavaleriei** **ccctagccttccaagctaacgatgcgggttcgattcccgctacccgctccatatgataat**

**Citrus_hongheensis** **ccctagccttccaagctaacgatgcgggttcgattcccgctacccgctccatatgataat**

**Citrus_limon** **ccctagccttccaagctaacgatgcgggttcgattcccgctacccgctccatatgataat**

**Citrus_maxima** **ccctagccttccaagctaacgatgcgggttcgattcccgctacccgctccatatgataat**

**Citrus_medica** **ccctagccttccaagctaacgatgcgggttcgattcccgctacccgctccatatgataat**

**Citrus_polytrifolia** **ccctagccttccaagctaacgatgcgggttcgattcccgctacccgctccatatgataat**

**Citrus_reticulata** **ccctagccttccaagctaacgatgcgggttcgattcccgctacccgctccatatgataat**

**Citrus_sinensis** **ccctagccttccaagctaacgatgcgggttcgattcccgctacccgctccatatgataat**

**Fortunella_bawangica_1** **ccctagccttccaagctaacgatgcgggttcgattcccgctacccgctccatatgataat**

**Fortunella_bawangica_2** **ccctagccttccaagctaacgatgcgggttcgattcccgctacccgctccatatgataat**

**Fortunella_hindsii_1** **ccctagccttccaagctaacgatgcgggttcgattcccgctacccgctccatatgataat**

**Fortunella_hindsii_2** **ccctagccttccaagctaacgatgcgggttcgattcccgctacccgctccatatgataat**

**Fortunella_hindsii_3** **ccctagccttccaagctaacgatgcgggttcgattcccgctacccgctccatatgataat**

**Fortunella_japonica_1** **ccctagccttccaagctaacgatgcgggttcgattcccgctacccgctccatatgataat**

**Fortunella_japonica_2** **ccctagccttccaagctaacgatgcgggttcgattcccgctacccgctccatatgataat**

**Fortunella_japonica_3** **ccctagccttccaagctaacgatgcgggttcgattcccgctacccgctccatatgataat**

**Fortunella_japonica_4** **ccctagccttccaagctaacgatgcgggttcgattcccgctacccgctccatatgataat**

**Fortunella_japonica_5** **ccctagccttccaagctaacgatgcgggttcgattcccgctacccgctccatatgataat**

**Fortunella_japonica_6** **ccctagccttccaagctaacgatgcgggttcgattcccgctacccgctccatatgataat**

**Fortunella_japonica_7** **ccctagccttccaagctaacgatgcgggttcgattcccgctacccgctccatatgataat**

**Fortunella_japonica_8** **ccctagccttccaagctaacgatgcgggttcgattcccgctacccgctccatatgataat**

**Fortunella_japonica_9** **ccctagccttccaagctaacgatgcgggttcgattcccgctacccgctccatatgataat**

**Fortunella_japonica_10** **ccctagccttccaagctaacgatgcgggttcgattcccgctacccgctccatatgataat**

**Fortunella_japonica_11** **ccctagccttccaagctaacgatgcgggttcgattcccgctacccgctccatatgataat**

**Fortunella_japonica_12** **ccctagccttccaagctaacgatgcgggttcgattcccgctacccgctccatatgataat**

**Fortunella_margarita_1** **ccctagccttccaagctaacgatgcgggttcgattcccgctacccgctccatatgataat**

**Fortunella_margarita_2** **ccctagccttccaagctaacgatgcgggttcgattcccgctacccgctccatatgataat**

**Fortunella_venosa_1** **ccctagccttccaagctaacgatgcgggttcgattcccgctacccgctccatatgataat**

**Fortunella_venosa_2** **ccctagccttccaagctaacgatgcgggttcgattcccgctacccgctccatatgataat**

**Fortunella_venosa_3** **ccctagccttccaagctaacgatgcgggttcgattcccgctacccgctccatatgataat**

11170	11180	11190	11200	11210	11220

....|....|....|....|....|....|....|....|....|....|....|....|

**Atalantia_kwangtungens** **tatatcaattagtatatcaactcggcgatgcatt-------aattaaagtttacttaatg**

**Citrus_reticulata_1** **tatatcagttagtatatcaactcggcgatgcatt-------aattaaagtttacttaatg**

**Citrus_reticulata_2** **tatatcagttagtatatcaactcggcgatgcatt-------aattaaagtttacttaatg**

**Citrus_trifoliata_3** **gatatcagttagtatatcaactcggcgatgcattaattattaattaaagtttacttaatg**

**Citrus_aurantiifolia** **tatatcagttagtatatcaactcggcgatgcatt-------aagtaaactttacttaatg**

**Citrus_aurantium** **tatatcagttagtatatcaactcggcgatgcatt-------aagtaaagtttacttaatg**

**Citrus_cavaleriei** **tatatcagttagtatatcaactcggcgatgcatt-------aattaaagtttacttaatg**

**Citrus_hongheensis** **tatatcagttagtatatcaactcggcgatgcatt-------aagtaaagtttacttaatg**

**Citrus_limon** **tatatcagttagtatatcaactcggcgatgcatt-------aagtaaagtttacttaatg**

**Citrus_maxima** **tatatcagttagtatatcaactcggcgatgcatt-------aagtaaagtttacttaatg**

**Citrus_medica** **tatatcagttagtatatcaactcggcgatgcatt-------aattaaagtttacttaatg**

**Citrus_polytrifolia** **gatatcagttagtatatcaactcggcgatgcattaattattaattaaagtttacttaatg**

**Citrus_reticulata** **tatatcagttagtatatcaactcggcgatgcatt-------aattaaagtttacttaatg**

**Citrus_sinensis** **tatatcagttagtatatcaactcggcgatgcatt-------aagtaaagtttacttaatg**

**Fortunella_bawangica_1** **tatatcagttagtatatcaactcggcgatgcattaattaaaaattaaagtttacttaatg**

**Fortunella_bawangica_2** **tatatcagttagtatatcaactcggcgatgcattaattaaaaattaaagtttacttaatg**

**Fortunella_hindsii_1** **tatatcagttagtatatcaactcggcgatgcattaattaaaaattaaagtttacttaatg**

**Fortunella_hindsii_2** **tatatcagttagtatatcaactcggcgatgcattaattaaaaattaaagtttacttaatg**

**Fortunella_hindsii_3** **tatatcagttagtatatcaactcggcgatgcattaattaaaaattaaagtttacttaatg**

**Fortunella_japonica_1** **tatatcagttagtatatcaactcggcgatgcattaattaaaaattaaagtttacttaatg**

**Fortunella_japonica_2** **tatatcagttagtatatcaactcggcgatgcattaattaaaaattaaagtttacttaatg**

**Fortunella_japonica_3** **tatatcagttagtatatcaactcggcgatgcattaattaaaaattaaagtttacttaatg**

**Fortunella_japonica_4** **tatatcagttagtatatcaactcggcgatgcattaattaaaaattaaagtttacttaatg**

**Fortunella_japonica_5** **tatatcagttagtatatcaactcggcgatgcattaattaaaaattaaagtttacttaatg**

**Fortunella_japonica_6** **tatatcagttagtatatcaactcggcgatgcattaattaaaaattaaagtttacttaatg**

**Fortunella_japonica_7** **tatatcagttagtatatcaactcggcgatgcattaattaaaaattaaagtttacttaatg**

**Fortunella_japonica_8** **tatatcagttagtatatcaactcggcgatgcattaattaaaaattaaagtttacttaatg**

**Fortunella_japonica_9** **tatatcagttagtatatcaactcggcgatgcattaattaaaaattaaagtttacttaatg**

**Fortunella_japonica_10** **tatatcagttagtatatcaactcggcgatgcattaattaaaaattaaagtttacttaatg**

**Fortunella_japonica_11** **tatatcagttagtatatcaactcggcgatgcattaattaaaaattaaagtttacttaatg**

**Fortunella_japonica_12** **tatatcagttagtatatcaactcggcgatgcattaattaaaaattaaagtttacttaatg**

**Fortunella_margarita_1** **tatatcagttagtatatcaactcggcgatgcattaattaaaaattaaagtttacttaatg**

**Fortunella_margarita_2** **tatatcagttagtatatcaactcggcgatgcattaattaaaaattaaagtttacttaatg**

**Fortunella_venosa_1** **tatatcagttagtatatcaactcggcgatgcattaattaaaaattaaagtttacttaatg**

**Fortunella_venosa_2** **tatatcagttagtatatcaactcggcgatgcattaattaaaaattaaagtttacttaatg**

**Fortunella_venosa_3** **tatatcagttagtatatcaactcggcgatgcattaattaaaaattaaagtttacttaatg**

11230	11240	11250	11260	11270	11280

....|....|....|....|....|....|....|....|....|....|....|....|

**Atalantia_kwangtungens** **catcgccaaattgaatatgaaagtccttaaattatatcacatattctttatgctttccga**

**Citrus_reticulata_1** **catcgccaagttgaatatgaaagtcctgaaattatatcacatattctttatgctttccga**

**Citrus_reticulata_2** **catcgccaagttgaatatgaaagtcctgaaattatatcacatattctttatgctttccga**

**Citrus_trifoliata_3** **catcgccaagttgaatatgaaagtcctgaaattatatcacatattctttatgctttccga**

**Citrus_aurantiifolia** **catcgccaagttgaatatgaaagtcctgaaattatatcacatattctttatgctttccga**

**Citrus_aurantium** **catcgccaagttgaatatgaaagtcctgaaattatatcacatattctttatgctttccga**

**Citrus_cavaleriei** **catcgccaagttgaatatgaaagtcctgaaattatatcacatattctttatgctttccga**

**Citrus_hongheensis** **catcgccaagttgaatatgaaagtcctgaaattatatcacatattctttatgctttccga**

**Citrus_limon** **catcgccaagttgaatatgaaagtcctgaaattatatcacatattctttatgctttccga**

**Citrus_maxima** **catcgccaagttgaatatgaaagtcctgaaattatatcacatattctttatgctttccga**

**Citrus_medica** **catcgccaagttgaatatgaaagtcctgaaattatatcacatattctttatgctttccga**

**Citrus_polytrifolia** **catcgccaagttgaatatgaaagtcctgaaattatatcacatattctttatgctttccga**

**Citrus_reticulata** **catcgccaagttgaatatgaaagtcctgaaattatatcacatattctttatgctttccga**

**Citrus_sinensis** **catcgccaagttgaatatgaaagtcctgaaattatatcacatattctttatgctttccga**

**Fortunella_bawangica_1** **catcgccaagttgaatatgaaagtcctgaaattatatcacatattctttatgctttccga**

**Fortunella_bawangica_2** **catcgccaagttgaatatgaaagtcctgaaattatatcacatattctttatgctttccga**

**Fortunella_hindsii_1** **catcgccaagttgaatatgaaagtcctgaaattatatcacatattctttatgctttccga**

**Fortunella_hindsii_2** **catcgccaagttgaatatgaaagtcctgaaattatatcacatattctttatgctttccga**

**Fortunella_hindsii_3** **catcgccaagttgaatatgaaagtcctgaaattatatcacatattctttatgctttccga**

**Fortunella_japonica_1** **catcgccaagttgaatatgaaagtcctgaaattatatcacatattctttatgctttccga**

**Fortunella_japonica_2** **catcgccaagttgaatatgaaagtcctgaaattatatcacatattctttatgctttccga**

**Fortunella_japonica_3** **catcgccaagttgaatatgaaagtcctgaaattatatcacatattctttatgctttccga**

**Fortunella_japonica_4** **catcgccaagttgaatatgaaagtcctgaaattatatcacatattctttatgctttccga**

**Fortunella_japonica_5** **catcgccaagttgaatatgaaagtcctgaaattatatcacatattctttatgctttccga**

**Fortunella_japonica_6** **catcgccaagttgaatatgaaagtcctgaaattatatcacatattctttatgctttccga**

**Fortunella_japonica_7** **catcgccaagttgaatatgaaagtcctgaaattatatcacatattctttatgctttccga**

**Fortunella_japonica_8** **catcgccaagttgaatatgaaagtcctgaaattatatcacatattctttatgctttccga**

**Fortunella_japonica_9** **catcgccaagttgaatatgaaagtcctgaaattatatcacatattctttatgctttccga**

**Fortunella_japonica_10** **catcgccaagttgaatatgaaagtcctgaaattatatcacatattctttatgctttccga**

**Fortunella_japonica_11** **catcgccaagttgaatatgaaagtcctgaaattatatcacatattctttatgctttccga**

**Fortunella_japonica_12** **catcgccaagttgaatatgaaagtcctgaaattatatcacatattctttatgctttccga**

**Fortunella_margarita_1** **catcgccaagttgaatatgaaagtcctgaaattatatcacatattctttatgctttccga**

**Fortunella_margarita_2** **catcgccaagttgaatatgaaagtcctgaaattatatcacatattctttatgctttccga**

**Fortunella_venosa_1** **catcgccaagttgaatatgaaagtcctgaaattatatcacatattctttatgctttccga**

**Fortunella_venosa_2** **catcgccaagttgaatatgaaagtcctgaaattatatcacatattctttatgctttccga**

**Fortunella_venosa_3** **catcgccaagttgaatatgaaagtcctgaaattatatcacatattctttatgctttccga**

11290	11300	11310	11320	11330	11340

....|....|....|....|....|....|....|....|....|....|....|....|

**Atalantia_kwangtungens** **agaagagataggaaagaaaaatgcgaa---------------------------aaaaaa**

**Citrus_reticulata_1** **agaagagataggaaagaaaaatgcgaaaaaaaaaaa-a----------a-----aagaaa**

**Citrus_reticulata_2** **agaagagataggaaagaaaaatgcgaaaaaaaaaaa-a----------a-----aagaaa**

**Citrus_trifoliata_3** **agaagagataggaaagaaaaatgcgaaaaaaaaa------------------------aa**

**Citrus_aurantiifolia** **agaagagataggaaagaaaaatgcgaaaaaaaaaaaaacaaaataaata-----aaaaaa**

**Citrus_aurantium** **agaagagataggaaagaaaaatgcg--aaaaaaaaaaa-taaataaata---aaaaaaaa**

**Citrus_cavaleriei** **agaagagatcggaaagaaaaatgcgaaaaaaaaaaaga----------a-----aaaaaa**

**Citrus_hongheensis** **agaagagataggaaagaaaaatgcgaaaaaaaaaaaaa-taaataaata-----aaaaaa**

**Citrus_limon** **agaagagataggaaagaaaaatgcg--aaaaaaaaaaa-taaataaata---aaaaaaaa**

**Citrus_maxima** **agaagagataggaaagaaaaattcg---aaaaaaaaaa-taaataaataaataaaaaaaa**

**Citrus_medica** **agaagagataggaaagaaaaatgcgaaaaaaaaaaaaa-aaaaga---------aaaaaa**

**Citrus_polytrifolia** **agaagagataggaaagaaaaatgcgaaaaaaaaaaaaa-aaaacaaa-------aaataa**

**Citrus_reticulata** **agaagagataggaaagaaaaatgcgaaaaaaaaaaa-a----------a-----aagaaa**

**Citrus_sinensis** **agaagagataggaaagaaaaatgcg--aaaaaaaaaaa-taaataaataaataaaaaaaa**

**Fortunella_bawangica_1** **agaagagataggaaagaaaaacgcg--------aaaaa----------------aaaaaa**

**Fortunella_bawangica_2** **agaagagataggaaagaaaaacgcg--------aaaaa----------------aaaaaa**

**Fortunella_hindsii_1** **agaagagataggaaagaaaaacgcg-----------aa----------------aaaaaa**

**Fortunella_hindsii_2** **agaagagataggaaagaaaaacgcg-----------aa----------------aaaaaa**

**Fortunella_hindsii_3** **agaagagataggaaagaaaaacgcg-----------aa----------------aaaaaa**

**Fortunella_japonica_1** **agaagagataggaaagaaaaacgcg-------aaaaaa----------------aaaaaa**

**Fortunella_japonica_2** **agaagagataggaaagaaaaacgcg---------aaaa----------------aaaaaa**

**Fortunella_japonica_3** **agaagagataggaaagaaaaacgcg-------aaaaaa----------------aaaaaa**

**Fortunella_japonica_4** **agaagagataggaaagaaaaacgcg-------aaaaaa----------------aaaaaa**

**Fortunella_japonica_5** **agaagagataggaaagaaaaacgcg-------aaaaaa----------------aaaaaa**

**Fortunella_japonica_6** **agaagagataggaaagaaaaacgcg-------aaaaaa----------------aaaaaa**

**Fortunella_japonica_7** **agaagagataggaaagaaaaacgcg-------aaaaaa----------------aaaaaa**

**Fortunella_japonica_8** **agaagagataggaaagaaaaacgcg-------aaaaaa----------------aaaaaa**

**Fortunella_japonica_9** **agaagagataggaaagaaaaacgcg-------aaaaaa----------------aaaaaa**

**Fortunella_japonica_10** **agaagagataggaaagaaaaacgcg--------aaaaa----------------aaaaaa**

**Fortunella_japonica_11** **agaagagataggaaagaaaaacgcg-------aaaaaa----------------aaaaaa**

**Fortunella_japonica_12** **agaagagataggaaagaaaaacgcg-------aaaaaa----------------aaaaaa**

**Fortunella_margarita_1** **agaagagataggaaagaaaaacgcga------aaaaaa----------------aaaaaa**

**Fortunella_margarita_2** **agaagagataggaaagaaaaacgcga------aaaaaa----------------aaaaaa**

**Fortunella_venosa_1** **agaagagataggaaagaaaaacgcg--------aaaaa----------------aaaaaa**

**Fortunella_venosa_2** **agaagagataggaaagaaaaacgcg--------aaaaa----------------aaaaaa**

**Fortunella_venosa_3** **agaagagataggaaagaaaaacgcg--------aaaaa----------------aaaaaa**

11350	11360	11370	11380	11390	11400

....|....|....|....|....|....|....|....|....|....|....|....|

**Atalantia_kwangtungens** **aaaaataggaatgggaatgaacggcgtccattgtctaatggataggacagaggtcttcta**

**Citrus_reticulata_1** **aaaattaggaatgggaatgaaaggcgtccattgtctaatggataggacagaggtcttcta**

**Citrus_reticulata_2** **aaaattaggaatgggaatgaaaggcgtccattgtctaatggataggacagaggtcttcta**

**Citrus_trifoliata_3** **aaaattaggaatgggaatgaaaggcgtccattgtctaatggataggacagaggtcttcta**

**Citrus_aurantiifolia** **aaaattaggaatgggaatgaaaggcgtccattgtctaatggataggacagaggtcttcta**

**Citrus_aurantium** **aaaattaggaatgggaatgaaaggcgtccattgtctaatggataggacagaggtcttcta**

**Citrus_cavaleriei** **aaaattaggaatgggaatgaaaggcgtccattgtctaatggataggacagaggtcttcta**

**Citrus_hongheensis** **aaaattaggaatgggaatgaaaggcgtccattgtctaatggataggacagaggtcttcta**

**Citrus_limon** **aaaattaggaatgggaatgaaaggcgtccattgtctaatggataggacagaggtcttcta**

**Citrus_maxima** **aaaattaggaatgggaatgaaaggcgtccattgtctaatggataggacagaggtcttcta**

**Citrus_medica** **aaaattaggaatgggaatgaaaggcgtccattgtctaatggataggacagaggtcttcta**

**Citrus_polytrifolia** **aaaattagaaatgggaatgaaaggcgtccattgtctaatggataggacagaggtcttcta**

**Citrus_reticulata** **aaaattaggaatgggaatgaaaggcgtccattgtctaatggataggacagaggtcttcta**

**Citrus_sinensis** **aaaattaggaatgggaatgaaaggcgtccattgtctaatggataggacagaggtcttcta**

**Fortunella_bawangica_1** **aaaattaggaatgggaatgaaaggcgtccattgtctaatggataggacagaggtcttcta**

**Fortunella_bawangica_2** **aaaattaggaatgggaatgaaaggcgtccattgtctaatggataggacagaggtcttcta**

**Fortunella_hindsii_1** **aaaattaggaatgggaatgaaaggcgtccattgtctaatggataggacagaggtcttcta**

**Fortunella_hindsii_2** **aaaattaggaatgggaatgaaaggcgtccattgtctaatggataggacagaggtcttcta**

**Fortunella_hindsii_3** **aaaattaggaatgggaatgaaaggcgtccattgtctaatggataggacagaggtcttcta**

**Fortunella_japonica_1** **aaaattaggaatgggaatgaaaggcgtccattgtctaatggataggacagaggtcttcta**

**Fortunella_japonica_2** **aaaattaggaatgggaatgaaaggcgtccattgtctaatggataggacagaggtcttcta**

**Fortunella_japonica_3** **aaaattaggaatgggaatgaaaggcgtccattgtctaatggataggacagaggtcttcta**

**Fortunella_japonica_4** **aaaattaggaatgggaatgaaaggcgtccattgtctaatggataggacagaggtcttcta**

**Fortunella_japonica_5** **aaaattaggaatgggaatgaaaggcgtccattgtctaatggataggacagaggtcttcta**

**Fortunella_japonica_6** **aaaattaggaatgggaatgaaaggcgtccattgtctaatggataggacagaggtcttcta**

**Fortunella_japonica_7** **aaaattaggaatgggaatgaaaggcgtccattgtctaatggataggacagaggtcttcta**

**Fortunella_japonica_8** **aaaattaggaatgggaatgaaaggcgtccattgtctaatggataggacagaggtcttcta**

**Fortunella_japonica_9** **aaaattaggaatgggaatgaaaggcgtccattgtctaatggataggacagaggtcttcta**

**Fortunella_japonica_10** **aaaattaggaatgggaatgaaaggcgtccattgtctaatggataggacagaggtcttcta**

**Fortunella_japonica_11** **aaaattaggaatgggaatgaaaggcgtccattgtctaatggataggacagaggtcttcta**

**Fortunella_japonica_12** **aaaattaggaatgggaatgaaaggcgtccattgtctaatggataggacagaggtcttcta**

**Fortunella_margarita_1** **aaaattaggaatgggaatgaaaggcgtccattgtctaatggataggacagaggtcttcta**

**Fortunella_margarita_2** **aaaattaggaatgggaatgaaaggcgtccattgtctaatggataggacagaggtcttcta**

**Fortunella_venosa_1** **aaaattaggaatgggaatgaaaggcgtccattgtctaatggataggacagaggtcttcta**

**Fortunella_venosa_2** **aaaattaggaatgggaatgaaaggcgtccattgtctaatggataggacagaggtcttcta**

**Fortunella_venosa_3** **aaaattaggaatgggaatgaaaggcgtccattgtctaatggataggacagaggtcttcta**

11410	11420	11430	11440	11450	11460

....|....|....|....|....|....|....|....|....|....|....|....|

**Atalantia_kwangtungens** **aacctttggtataggttcaaatcctattggacgcaattttttgccatttccccatattct**

**Citrus_reticulata_1** **aacctttggtataggttcaaatcctattggacgcaattttttgccatttccccatattct**

**Citrus_reticulata_2** **aacctttggtataggttcaaatcctattggacgcaattttttgccatttccccatattct**

**Citrus_trifoliata_3** **aacctttggtataggttcaaatcctattggacgcaattttttgccatttccccatattct**

**Citrus_aurantiifolia** **aacctttggtataggttcaaatcctattggacgcaattttttgccatttccccatattct**

**Citrus_aurantium** **aacctttggtataggttcaaatcctattggacgcaattttttgccatttccccatattct**

**Citrus_cavaleriei** **aacctttggtataggttcaaatcctattggacgcaattttttgccatttccccatattct**

**Citrus_hongheensis** **aacctttggtataggttcaaatcctattggacgcaattttttgccatttccccatattct**

**Citrus_limon** **aacctttggtataggttcaaatcctattggacgcaattttttgccatttccccatattct**

**Citrus_maxima** **aacctttggtataggttcaaatcctattggacgcaattttttgccatttccccatattct**

**Citrus_medica** **aacctttggtataggttcaaatcctattggacgcaattttttgccatttccccatattct**

**Citrus_polytrifolia** **aacctttggtataggttcaaatcctattggacgcaattttttgccatttccccatattct**

**Citrus_reticulata** **aacctttggtataggttcaaatcctattggacgcaattttttgccatttccccatattct**

**Citrus_sinensis** **aacctttggtataggttcaaatcctattggacgcaattttttgccatttccccatattct**

**Fortunella_bawangica_1** **aacctttggtataggttcaaatcctattggacgcaattttttgccatttccccatattct**

**Fortunella_bawangica_2** **aacctttggtataggttcaaatcctattggacgcaattttttgccatttccccatattct**

**Fortunella_hindsii_1** **aacctttggtataggttcaaatcctattggacgcaattttttgccatttccccatattct**

**Fortunella_hindsii_2** **aacctttggtataggttcaaatcctattggacgcaattttttgccatttccccatattct**

**Fortunella_hindsii_3** **aacctttggtataggttcaaatcctattggacgcaattttttgccatttccccatattct**

**Fortunella_japonica_1** **aacctttggtataggttcaaatcctattggacgcaattttttgccatttccccatattct**

**Fortunella_japonica_2** **aacctttggtataggttcaaatcctattggacgcaattttttgccatttccccatattct**

**Fortunella_japonica_3** **aacctttggtataggttcaaatcctattggacgcaattttttgccatttccccatattct**

**Fortunella_japonica_4** **aacctttggtataggttcaaatcctattggacgcaattttttgccatttccccatattct**

**Fortunella_japonica_5** **aacctttggtataggttcaaatcctattggacgcaattttttgccatttccccatattct**

**Fortunella_japonica_6** **aacctttggtataggttcaaatcctattggacgcaattttttgccatttccccatattct**

**Fortunella_japonica_7** **aacctttggtataggttcaaatcctattggacgcaattttttgccatttccccatattct**

**Fortunella_japonica_8** **aacctttggtataggttcaaatcctattggacgcaattttttgccatttccccatattct**

**Fortunella_japonica_9** **aacctttggtataggttcaaatcctattggacgcaattttttgccatttccccatattct**

**Fortunella_japonica_10** **aacctttggtataggttcaaatcctattggacgcaattttttgccatttccccatattct**

**Fortunella_japonica_11** **aacctttggtataggttcaaatcctattggacgcaattttttgccatttccccatattct**

**Fortunella_japonica_12** **aacctttggtataggttcaaatcctattggacgcaattttttgccatttccccatattct**

**Fortunella_margarita_1** **aacctttggtataggttcaaatcctattggacgcaattttttgccatttccccatattct**

**Fortunella_margarita_2** **aacctttggtataggttcaaatcctattggacgcaattttttgccatttccccatattct**

**Fortunella_venosa_1** **aacctttggtataggttcaaatcctattggacgcaattttttgccatttccccatattct**

**Fortunella_venosa_2** **aacctttggtataggttcaaatcctattggacgcaattttttgccatttccccatattct**

**Fortunella_venosa_3** **aacctttggtataggttcaaatcctattggacgcaattttttgccatttccccatattct**

11470	11480	11490	11500	11510	11520

....|....|....|....|....|....|....|....|....|....|....|....|

**Atalantia_kwangtungens** **attacataataat-atattcttattcgattattattattaataata-------aattatt**

**Citrus_reticulata_1** **attacataataataatattcttattcgattattattattaataataaattaataattagt**

**Citrus_reticulata_2** **attacataataataatattcttattcgattattattattaataataaattaataattagt**

**Citrus_trifoliata_3** **attacataataat-atattcttattcg---attattattaataataaattaataattatt**

**Citrus_aurantiifolia** **attacataataat-atattcttattcgattattattattaataataaattaataattatt**

**Citrus_aurantium** **attacataataat-atattcttattcgattattattattaataataaattaataattatt**

**Citrus_cavaleriei** **attacataataat-atattcttattcgattattattattaataataaattaataattatt**

**Citrus_hongheensis** **attacataataat-atattcttattcgattattattattaataataaattaataattatt**

**Citrus_limon** **attacataataat-atattcttattcgattattattattaataataaattaataattatt**

**Citrus_maxima** **attacataataat-atattcttattcgattattattattaataataaattaataattatt**

**Citrus_medica** **attacataataat-atattcttattcgattattattattaataataaattaataattatt**

**Citrus_polytrifolia** **attacataataat-atattcttattcg---attattattaataataaattaataattatt**

**Citrus_reticulata** **attacataataataatattcttattcgattattattattaataataaattaataattagt**

**Citrus_sinensis** **attacataataat-atattcttattcgattattattattaataataaattaataattatt**

**Fortunella_bawangica_1** **attacagaataat-atattcttattcgattattattattaataataaattattaattatt**

**Fortunella_bawangica_2** **attacagaataat-atattcttattcgattattattattaataataaattattaattatt**

**Fortunella_hindsii_1** **attacagaataat-atattcttattcgattattattattaataataaattattaattatt**

**Fortunella_hindsii_2** **attacagaataat-atattcttattcgattattattattaataataaattattaattatt**

**Fortunella_hindsii_3** **attacagaataat-atattcttattcgattattattattaataataaattattaattatt**

**Fortunella_japonica_1** **attacagaataat-atattcttattcgattattattattaataataaattattaattatt**

**Fortunella_japonica_2** **attacagaataat-atattcttattcgattattattattaataataaattattaattatt**

**Fortunella_japonica_3** **attacagaataat-atattcttattcgattattattattaataataaattattaattatt**

**Fortunella_japonica_4** **attacagaataat-atattcttattcgattattattattaataataaattattaattatt**

**Fortunella_japonica_5** **attacagaataat-atattcttattcgattattattattaataataaattattaattatt**

**Fortunella_japonica_6** **attacagaataat-atattcttattcgattattattattaataataaattattaattatt**

**Fortunella_japonica_7** **attacagaataat-atattcttattcgattattattattaataataaattattaattatt**

**Fortunella_japonica_8** **attacagaataat-atattcttattcgattattattattaataataaattattaattatt**

**Fortunella_japonica_9** **attacagaataat-atattcttattcgattattattattaataataaattattaattatt**

**Fortunella_japonica_10** **attacagaataat-atattcttattcgattattattattaataataaattattaattatt**

**Fortunella_japonica_11** **attacagaataat-atattcttattcgattattattattaataataaattattaattatt**

**Fortunella_japonica_12** **attacagaataat-atattcttattcgattattattattaataataaattattaattatt**

**Fortunella_margarita_1** **attacagaataat-atattcttattcgattattattattaataataaattattaattatt**

**Fortunella_margarita_2** **attacagaataat-atattcttattcgattattattattaataataaattattaattatt**

**Fortunella_venosa_1** **attacagaataat-atattcttattcgattattattattaataataaattattaattatt**

**Fortunella_venosa_2** **attacagaataat-atattcttattcgattattattattaataataaattattaattatt**

**Fortunella_venosa_3** **attacagaataat-atattcttattcgattattattattaataataaattattaattatt**

11530	11540	11550	11560	11570	11580

....|....|....|....|....|....|....|....|....|....|....|....|

**Atalantia_kwangtungens** **atattaa--------tcaattttgatttgaaataataaatagagtaataaattg------**

**Citrus_reticulata_1** **atattaa--------tcaattttgatttgaaataataaatagagtaatatattg------**

**Citrus_reticulata_2** **atattaa--------tcaattttgatttgaaataataaatagagtaatatattg------**

**Citrus_trifoliata_3** **atattaa--------taaattttgatttgaaataataaatagagtaatatattg------**

**Citrus_aurantiifolia** **atattaa--------taaattttgatttgaaataataaatagagtaatatattg------**

**Citrus_aurantium** **atattaa--------taaattttgatttgaaataataaatagagtaatatattg------**

**Citrus_cavaleriei** **atattaa--------tcaattttgatttgaaataataaatagagtaatatattg------**

**Citrus_hongheensis** **atattaa--------taaattttgatttgaaataataaatagagtaatatattg------**

**Citrus_limon** **atattaa--------taaattttgatttgaaataataaatagagtaatatattg------**

**Citrus_maxima** **atattaataaatttttaaattttgatttgaaataataaatagagtaatatattgaataat**

**Citrus_medica** **atattaa--------taaattttgatttgaaataatcaatagagtaatatattg------**

**Citrus_polytrifolia** **atattaa--------taaattttgatttgaaataataaatagagtaatatattg------**

**Citrus_reticulata** **atattaa--------tcaattttgatttgaaataataaatagagtaatatattg------**

**Citrus_sinensis** **atattaa--------taaattttgatttgaaataataaatagagtaatatattg------**

**Fortunella_bawangica_1** **atattaa--------taaattttgatttgaaataataaatagagtaatatattg------**

**Fortunella_bawangica_2** **atattaa--------taaattttgatttgaaataataaatagagtaatatattg------**

**Fortunella_hindsii_1** **atattaa--------tcaattttgatttgaaataataaatagagtaatatattg------**

**Fortunella_hindsii_2** **atattaa--------tcaattttgatttgaaataataaatagagtaatatattg------**

**Fortunella_hindsii_3** **atattaa--------tcaattttgatttgaaataataaatagagtaatatattg------**

**Fortunella_japonica_1** **atattaa--------taaattttgatttgaaataataaatagagtaatatattg------**

**Fortunella_japonica_2** **atattaa--------taaattttgatttgaaataataaatagagtaatatattg------**

**Fortunella_japonica_3** **atattaa--------taaattttgatttgaaataataaatagagtaatatattg------**

**Fortunella_japonica_4** **atattaa--------taaattttgatttgaaataataaatagagtaatatattg------**

**Fortunella_japonica_5** **atattaa--------taaattttgatttgaaataataaatagagtaatatattg------**

**Fortunella_japonica_6** **atattaa--------taaattttgatttgaaataataaatagagtaatatattg------**

**Fortunella_japonica_7** **atattaa--------taaattttgatttgaaataataaatagagtaatatattg------**

**Fortunella_japonica_8** **atattaa--------taaattttgatttgaaataataaatagagtaatatattg------**

**Fortunella_japonica_9** **atattaa--------taaattttgatttgaaataataaatagagtaatatattg------**

**Fortunella_japonica_10** **atattaa--------taaattttgatttgaaataataaatagagtaatatattg------**

**Fortunella_japonica_11** **ataataa--------taaattttgatttgaaataataaatagagtaatatattg------**

**Fortunella_japonica_12** **atattaa--------taaattttgatttgaaataataaatagagtaatatattg------**

**Fortunella_margarita_1** **atattaa--------taaattttgatttgaaataataaatagagtaatatattg------**

**Fortunella_margarita_2** **atattaa--------taaattttgatttgaaataataaatagagtaatatattg------**

**Fortunella_venosa_1** **atattaa--------taaattttgatttgaaataataaatagagtaatatattg------**

**Fortunella_venosa_2** **atattaa--------taaattttgatttgaaataataaatagagtaatatattg------**

**Fortunella_venosa_3** **atattaa--------taaattttgatttgaaataataaatagagtaatatattg------**

11590	11600	11610	11620	11630	11640

....|....|....|....|....|....|....|....|....|....|....|....|

**Atalantia_kwangtungens** **-aataattattcaactatttaattactta---------tata------------------**

**Citrus_reticulata_1** **-aataattattcaaatattttattactta---------tatatatttatagatta-----**

**Citrus_reticulata_2** **-aataattattcaaatattttattactta---------tatatatttatagatta-----**

**Citrus_trifoliata_3** **-aataattattcaaatattttattactta---------tatatatttatatatat-----**

**Citrus_aurantiifolia** **-aataattattcaaatattttattacttatatatattttatatatttatatatta-----**

**Citrus_aurantium** **-aataattattcaaatattttattactta---------tatatatttctatatta-----**

**Citrus_cavaleriei** **-aataattattcaaatattttattactta---------tatatatttagatattc-----**

**Citrus_hongheensis** **-aataattattcaaatattttattactta---------tatatatttatatatta-----**

**Citrus_limon** **-aataattattcaaatattttattactta---------tatatatttctatatta-----**

**Citrus_maxima** **taataattattcaaatattttattactta---------tatatatttatatatta-----**

**Citrus_medica** **-aataattattcaaatattttattactta-----------------tatatatta-----**

**Citrus_polytrifolia** **-aataattattcaaatattttattactta---------tatatatttatatatta-----**

**Citrus_reticulata** **-aataattattcaaatattttattactta---------tatatatttatagatta-----**

**Citrus_sinensis** **-aataattattcaaatattttattactta---------tatatatttatatatta-----**

**Fortunella_bawangica_1** **-aataattattcaaatattttattactta---------tatatatttatatatta----t**

**Fortunella_bawangica_2** **-aataattattcaaatattttattactta---------tatatatttatatatta----t**

**Fortunella_hindsii_1** **-aataattattcaaatattttattactta---------tatatatttatatatta---tt**

**Fortunella_hindsii_2** **-aataattattcaaatattttattactta---------tatatatttatatatta---tt**

**Fortunella_hindsii_3** **-aataattattcaaatattttattactta---------tatatatttatatatta---tt**

**Fortunella_japonica_1** **-aataattattcaaatattttattactta---------tatatatttatatatta---tt**

**Fortunella_japonica_2** **-aataattattcaaatattttattactta---------tatatatttatatatta----t**

**Fortunella_japonica_3** **-aataattattcaaatattttattactta---------tatatatttatatatta---tt**

**Fortunella_japonica_4** **-aataattattcaaatattttattactta---------tatatatttatatatta---tt**

**Fortunella_japonica_5** **-aataattattcaaatattttattactta---------tatatatttatatatta---tt**

**Fortunella_japonica_6** **-aataattattcaaatattttattactta---------tatatatttatatatta---tt**

**Fortunella_japonica_7** **-aataattattcaaatattttattactta---------tatatatttatatatta---tt**

**Fortunella_japonica_8** **-aataattattcaaatattttattactta---------tatatatttatatatta---tt**

**Fortunella_japonica_9** **-aataattattcaaatattttattactta---------tatatatttatatatta---tt**

**Fortunella_japonica_10** **-aataattattcaaatattttattactta---------tatatatttatatatta----t**

**Fortunella_japonica_11** **-aataattattcaaatattttattactta---------tatatatttatatatta----t**

**Fortunella_japonica_12** **-aataattattcaaatattttattactta---------tatatatttatatatta---tt**

**Fortunella_margarita_1** **-aataattattcaaatattttattactta---------tatatatttatatatta----t**

**Fortunella_margarita_2** **-aataattattcaaatattttattactta---------tatatatttatatatta----t**

**Fortunella_venosa_1** **-aataattattcaaatattttattactta---------tatatatttatatattattttt**

**Fortunella_venosa_2** **-aataattattcaaatattttattactta---------tatatatttatatatta-tttt**

**Fortunella_venosa_3** **-aataattattcaaatattttattactta---------tatatatttatatatta-tttt**

11650	11660	11670	11680	11690	11700

....|....|....|....|....|....|....|....|....|....|....|....|

**Atalantia_kwangtungens** **-----ttatttttttttttaatattattaagtattataaagtaagaataataaaagtaag**

**Citrus_reticulata_1** **-----ttattttttttt--tatattattaagtattat---------------aaagtaag**

**Citrus_reticulata_2** **-----ttattttttttt--tatattattaagtattat---------------aaagtaag**

**Citrus_trifoliata_3** **----ttttttttttaatattatattattaagtattat---------------aaagtaag**

**Citrus_aurantiifolia** **-----tttttttttt----tatattattaagtattat---------------aaagtaag**

**Citrus_aurantium** **----------ttttttttttttattattaagtattat---------------aaagtaag**

**Citrus_cavaleriei** **-----tttttttttttttatatattattaagtattat---------------aaagtaag**

**Citrus_hongheensis** **-----tttttttttttttttttattattaagtattat---------------aaagtaag**

**Citrus_limon** **----------ttttttttttttattattaagtattat---------------aaagtaag**

**Citrus_maxima** **----------ttttttttttttattattaagtattat---------------aaagtaag**

**Citrus_medica** **-----ttttttttttttttaatattattaagtattat---------------aaagtaag**

**Citrus_polytrifolia** **----tttttttttt----taatattattaagtattat---------------aaagtaag**

**Citrus_reticulata** **-----ttattttttttt--tatattattaagtattat---------------aaagtaag**

**Citrus_sinensis** **---------tttttttttttttattattaagtattat---------------aaagtaag**

**Fortunella_bawangica_1** **tttttttttttattttttgaatattattaagtattat---------------aaagtaag**

**Fortunella_bawangica_2** **tttttttttttattttttgaatattattaagtattat---------------aaagtaag**

**Fortunella_hindsii_1** **tttttttttttattttttgaatattattaagtattat---------------aaagtaag**

**Fortunella_hindsii_2** **tttttttttttattttttgaatattattaagtattat---------------aaagtaag**

**Fortunella_hindsii_3** **tttttgtttttattttttgaatattattaagtattat---------------aaagtaag**

**Fortunella_japonica_1** **tttttttttttattttttgaatattattaagtattat---------------aaagtaag**

**Fortunella_japonica_2** **tttttttttttattttttgaatattattaagtattat---------------aaagtaag**

**Fortunella_japonica_3** **tttttttttttattttttgaatattattaagtattat---------------aaagtaag**

**Fortunella_japonica_4** **tttttttttttattttttgaatattattaagtattat---------------aaagtaag**

**Fortunella_japonica_5** **tttttttttttattttttgaatattattaagtattat---------------aaagtaag**

**Fortunella_japonica_6** **tttttttttttattttttgaatattattaagtattat---------------aaagtaag**

**Fortunella_japonica_7** **tttttttttttattttttgaatattattaagtattat---------------aaagtaag**

**Fortunella_japonica_8** **tttttttttttattttttgaatattattaagtattat---------------aaagtaag**

**Fortunella_japonica_9** **tttttttttttattttttgaatattattaagtattat---------------aaagtaag**

**Fortunella_japonica_10** **tttttttttttattttttgaatattattaagtattat---------------aaagtaag**

**Fortunella_japonica_11** **ttttttttttgattttttgaatattattaagtattat---------------aaagtaag**

**Fortunella_japonica_12** **tttttttttttattttttgaatattattaagtattat---------------aaagtaag**

**Fortunella_margarita_1** **tttttttttttattttttgaatattattaagtattat---------------aaagtaag**

**Fortunella_margarita_2** **tttttttttttattttttgaatattattaagtattat---------------aaagtaag**

**Fortunella_venosa_1** **ttttttttttttttttttgaatattattaagtattat---------------aaagtaag**

**Fortunella_venosa_2** **tttttttttttattttttgaatattattaagtattat---------------aaagtaag**

**Fortunella_venosa_3** **tttttttttttattttttgaatattattaagtattat---------------aaagtaag**

11710	11720	11730	11740	11750	11760

....|....|....|....|....|....|....|....|....|....|....|....|

**Atalantia_kwangtungens** **aataagagtggttttttatttataattgttcctgaagtagaaaacgttccttttgttcct**

**Citrus_reticulata_1** **aataagagtggttttttatttataattgttcctgaagtagaaaacgttccttttgttcct**

**Citrus_reticulata_2** **aataagagtggttttttatttataattgttcctgaagtagaaaacgttccttttgttcct**

**Citrus_trifoliata_3** **aataagagtggttttttatttataattgttcctgaagtagaaaacgttccttttgttcct**

**Citrus_aurantiifolia** **aataagagtggttttttatttataattgttcctgaagtagaaaacgttccttttgttcct**

**Citrus_aurantium** **aataagagtggttttttatttagaattgttcctgaagtagaaaacgttccttttgttcct**

**Citrus_cavaleriei** **aataagagtggttttttatttataattgttcctgaagtagaaaacgttccttttgttcct**

**Citrus_hongheensis** **aataagagtggttttttatttagaattgttcctgaagtagaaaacgttccttttgttcct**

**Citrus_limon** **aataagagtggttttttatttagaattgttcctgaagtagaaaacgttccttttgttcct**

**Citrus_maxima** **aataagagtggttttttatttagaattgttcctgaagtagaaaacgttccttttgttcct**

**Citrus_medica** **aataagagtggttttttatttataattgttcctgaagtagaaaacgttccttttgttcct**

**Citrus_polytrifolia** **aataagagtggttttttatttataattgttcctgaagtagaaaacgttccttttgttcct**

**Citrus_reticulata** **aataagagtggttttttatttataattgttcctgaagtagaaaacgttccttttgttcct**

**Citrus_sinensis** **aataagagtggttttttatttagaattgttcctgaagtagaaaacgttccttttgttcct**

**Fortunella_bawangica_1** **aataagagtggttttttatttataattgttcctgaagtagaaaacgttccttttgttcct**

**Fortunella_bawangica_2** **aataagagtggttttttatttataattgttcctgaagtagaaaacgttccttttgttcct**

**Fortunella_hindsii_1** **aataagagtggttttttatttataattgttcctgaagtagaaaacgttccttttgttcct**

**Fortunella_hindsii_2** **aataagagtggttttttatttataattgttcctgaagtagaaaacgttccttttgttcct**

**Fortunella_hindsii_3** **aataagagtggttttttatttataattgttcctgaagtagaaaacgttccttttgttcct**

**Fortunella_japonica_1** **aataagagtggttttttatttataattgttcctgaagtagaaaacgttccttttgttcct**

**Fortunella_japonica_2** **aataagagtggttttttatttataattgttcctgaagtagaaaacgttccttttgttcct**

**Fortunella_japonica_3** **aataagagtggttttttatttataattgttcctgaagtagaaaacgttccttttgttcct**

**Fortunella_japonica_4** **aataagagtggttttttatttataattgttcctgaagtagaaaacgttccttttgttcct**

**Fortunella_japonica_5** **aataagagtggttttttatttataattgttcctgaagtagaaaacgttccttttgttcct**

**Fortunella_japonica_6** **aataagagtggttttttatttataattgttcctgaagtagaaaacgttccttttgttcct**

**Fortunella_japonica_7** **aataagagtggttttttatttataattgttcctgaagtagaaaacgttccttttgttcct**

**Fortunella_japonica_8** **aataagagtggttttttatttataattgttcctgaagtagaaaacgttccttttgttcct**

**Fortunella_japonica_9** **aataagagtggttttttatttataattgttcctgaagtagaaaacgttccttttgttcct**

**Fortunella_japonica_10** **aataagagtggttttttatttataattgttcctgaagtagaaaacgttccttttgttcct**

**Fortunella_japonica_11** **aataagagtggttttttatttataattgttcctgaagtagaaaacgttccttttgttcct**

**Fortunella_japonica_12** **aataagagtggttttttatttataattgttcctgaagtagaaaacgttccttttgttcct**

**Fortunella_margarita_1** **aataagagtggttttttatttataattgttcctgaagtagaaaacgttccttttgttcct**

**Fortunella_margarita_2** **aataagagtggttttttatttataattgttcctgaagtagaaaacgttccttttgttcct**

**Fortunella_venosa_1** **aataagagtggttttttatttataattgttcctgaagtagaaaacgttccttttgttcct**

**Fortunella_venosa_2** **aataagagtggttttttatttataattgttcctgaagtagaaaacgttccttttgttcct**

**Fortunella_venosa_3** **aataagagtggttttttatttataattgttcctgaagtagaaaacgttccttttgttcct**

11770	11780	11790	11800	11810	11820

....|....|....|....|....|....|....|....|....|....|....|....|

**Atalantia_kwangtungens** **gaatagcttcttgcaaaagggcttctgcttcgtcggtgaatatcttggtagaagatatta**

**Citrus_reticulata_1** **gaatagcttcttgcaaaagggcttctgcttcttcggtgaatatcttggtagaagatatta**

**Citrus_reticulata_2** **gaatagcttcttgcaaaagggcttctgcttcttcggtgaatatcttggtagaagatatta**

**Citrus_trifoliata_3** **gaatagcttcttgcaaaagggcttctgcttcttcggtgaatatcttggtagaagatatta**

**Citrus_aurantiifolia** **gaatagcttcttgcaaaagggcttctgcttcttcggtgaatatcttggtagaagatatta**

**Citrus_aurantium** **gaatagcttcttgcaaaagggcttctgcttcttcggtgaatatcttggtagaagatatta**

**Citrus_cavaleriei** **gaatagcttcttgcaaaagggcttctgcttcttcggtgaatatcttggtagaagatatta**

**Citrus_hongheensis** **gaatagcttcttgcaaaagggcttctgcttcttcggtgaatatcttggtagaagatatta**

**Citrus_limon** **gaatagcttcttgcaaaagggcttctgcttcttcggtgaatatcttggtagaagatatta**

**Citrus_maxima** **gaatagcttcttgcaaaagggcttctgcttcttcggtgaatatcttggtagaagatatta**

**Citrus_medica** **gaatagcttcttgcaaaagggcttctgcttcttcggtgaatatcttggtagaagatatta**

**Citrus_polytrifolia** **gaatagcttcttgcaaaagggcttctgcttcttcggtgaatatcttggtagaagatatta**

**Citrus_reticulata** **gaatagcttcttgcaaaagggcttctgcttcttcggtgaatatcttggtagaagatatta**

**Citrus_sinensis** **gaatagcttcttgcaaaagggcttctgcttcttcggtgaatatcttggtagaagatatta**

**Fortunella_bawangica_1** **gaatagcttcttgcaaaagggcttctgcttcttcggtgaatatcttggtagaagatatta**

**Fortunella_bawangica_2** **gaatagcttcttgcaaaagggcttctgcttcttcggtgaatatcttggtagaagatatta**

**Fortunella_hindsii_1** **gaatagcttcttgcaaaagggcttctgcttcttcggtgaatatcttggtagaagatatta**

**Fortunella_hindsii_2** **gaatagcttcttgcaaaagggcttctgcttcttcggtgaatatcttggtagaagatatta**

**Fortunella_hindsii_3** **gaatagcttcttgcaaaagggcttctgcttcttcggtgaatatcttggtagaagatatta**

**Fortunella_japonica_1** **gaatagcttcttgcaaaagggcttctgcttcttcggtgaatatcttggtagaagatatta**

**Fortunella_japonica_2** **gaatagcttcttgcaaaagggcttctgcttcttcggtgaatatcttggtagaagatatta**

**Fortunella_japonica_3** **gaatagcttcttgcaaaagggcttctgcttcttcggtgaatatcttggtagaagatatta**

**Fortunella_japonica_4** **gaatagcttcttgcaaaagggcttctgcttcttcggtgaatatcttggtagaagatatta**

**Fortunella_japonica_5** **gaatagcttcttgcaaaagggcttctgcttcttcggtgaatatcttggtagaagatatta**

**Fortunella_japonica_6** **gaatagcttcttgcaaaagggcttctgcttcttcggtgaatatcttggtagaagatatta**

**Fortunella_japonica_7** **gaatagcttcttgcaaaagggcttctgcttcttcggtgaatatcttggtagaagatatta**

**Fortunella_japonica_8** **gaatagcttcttgcaaaagggcttctgcttcttcggtgaatatcttggtagaagatatta**

**Fortunella_japonica_9** **gaatagcttcttgcaaaagggcttctgcttcttcggtgaatatcttggtagaagatatta**

**Fortunella_japonica_10** **gaatagcttcttgcaaaagggcttctgcttcttcggtgaatatcttggtagaagatatta**

**Fortunella_japonica_11** **gaatagcttcttgcaaaagggcttctgcttcttcggtgaatatcttggtagaagatatta**

**Fortunella_japonica_12** **gaatagcttcttgcaaaagggcttctgcttcttcggtgaatatcttggtagaagatatta**

**Fortunella_margarita_1** **gaatagcttcttgcaaaagggcttctgcttcttcggtgaatatcttggtagaagatatta**

**Fortunella_margarita_2** **gaatagcttcttgcaaaagggcttctgcttcttcggtgaatatcttggtagaagatatta**

**Fortunella_venosa_1** **gaatagcttcttgcaaaagggcttctgcttcttcggtgaatatcttggtagaagatatta**

**Fortunella_venosa_2** **gaatagcttcttgcaaaagggcttctgcttcttcggtgaatatcttggtagaagatatta**

**Fortunella_venosa_3** **gaatagcttcttgcaaaagggcttctgcttcttcggtgaatatcttggtagaagatatta**

11830	11840	11850	11860	11870	11880

....|....|....|....|....|....|....|....|....|....|....|....|

**Atalantia_kwangtungens** **tttcttggaactgaggtttattcgtttttaagtaagtacgtaactcaacgagaaattttc**

**Citrus_reticulata_1** **tttcttggaactgaggtttattcgtttttaagtaagtacgtaactcaacgagaaattttc**

**Citrus_reticulata_2** **tttcttggaactgaggtttattcgtttttaagtaagtacgtaactcaacgagaaattttc**

**Citrus_trifoliata_3** **tttcttggaactgaggtttattcgtttttaagtaagtacgtaactcaacgagaaattttc**

**Citrus_aurantiifolia** **tttcttggaactgaggtttattcgtttttaagtaagtacgtaactcaacgagaaattttc**

**Citrus_aurantium** **tttcttggaactgaggtttattcgtttttaagtaagtacgtaactcaacgagaaattttc**

**Citrus_cavaleriei** **tttcttggaactgaggtttattcgtttttaagtaagtacgtaactcaacgagaaattttc**

**Citrus_hongheensis** **tttcttggaactgaggtttattcgtttttaagtaagtacgtaactcaacgagaaattttc**

**Citrus_limon** **tttcttggaactgaggtttattcgtttttaagtaagtacgtaactcaacgagaaattttc**

**Citrus_maxima** **tttcttggaactgaggtttattcgtttttaagtaagtacgtaactcaacgagaaattttc**

**Citrus_medica** **tttcttggaactgaggtttattcgtttttaagtaagtacgtaactcaacgagaaattttc**

**Citrus_polytrifolia** **tttcttggaactgaggtttattcgtttttaagtaagtacgtaactcaacgagaaattttc**

**Citrus_reticulata** **tttcttggaactgaggtttattcgtttttaagtaagtacgtaactcaacgagaaattttc**

**Citrus_sinensis** **tttcttggaactgaggtttattcgtttttaagtaagtacgtaactcaacgagaaattttc**

**Fortunella_bawangica_1** **tttcttggaactgaggtttattcgtttttaagtaagtacgtaactcaacgagaaattttc**

**Fortunella_bawangica_2** **tttcttggaactgaggtttattcgtttttaagtaagtacgtaactcaacgagaaattttc**

**Fortunella_hindsii_1** **tttcttggaactgaggtttattcgtttttaagtaagtacgtaactcaacgagaaattttc**

**Fortunella_hindsii_2** **tttcttggaactgaggtttattcgtttttaagtaagtacgtaactcaacgagaaattttc**

**Fortunella_hindsii_3** **tttcttggaactgaggtttattcgtttttaagtaagtacgtaactcaacgagaaattttc**

**Fortunella_japonica_1** **tttcttggaactgaggtttattcgtttttaagtaagtacgtaactcaacgagaaattttc**

**Fortunella_japonica_2** **tttcttggaactgaggtttattcgtttttaagtaagtacgtaactcaacgagaaattttc**

**Fortunella_japonica_3** **tttcttggaactgaggtttattcgtttttaagtaagtacgtaactcaacgagaaattttc**

**Fortunella_japonica_4** **tttcttggaactgaggtttattcgtttttaagtaagtacgtaactcaacgagaaattttc**

**Fortunella_japonica_5** **tttcttggaactgaggtttattcgtttttaagtaagtacgtaactcaacgagaaattttc**

**Fortunella_japonica_6** **tttcttggaactgaggtttattcgtttttaagtaagtacgtaactcaacgagaaattttc**

**Fortunella_japonica_7** **tttcttggaactgaggtttattcgtttttaagtaagtacgtaactcaacgagaaattttc**

**Fortunella_japonica_8** **tttcttggaactgaggtttattcgtttttaagtaagtacgtaactcaacgagaaattttc**

**Fortunella_japonica_9** **tttcttggaactgaggtttattcgtttttaagtaagtacgtaactcaacgagaaattttc**

**Fortunella_japonica_10** **tttcttggaactgaggtttattcgtttttaagtaagtacgtaactcaacgagaaattttc**

**Fortunella_japonica_11** **tttcttggaactgaggtttattcgtttttaagtaagtacgtaactcaacgagaaattttc**

**Fortunella_japonica_12** **tttcttggaactgaggtttattcgtttttaagtaagtacgtaactcaacgagaaattttc**

**Fortunella_margarita_1** **tttcttggaactgaggtttattcgtttttaagtaagtacgtaactcaacgagaaattttc**

**Fortunella_margarita_2** **tttcttggaactgaggtttattcgtttttaagtaagtacgtaactcaacgagaaattttc**

**Fortunella_venosa_1** **tttcttggaactgaggtttattcgtttttaagtaagtacgtaactcaacgagaaattttc**

**Fortunella_venosa_2** **tttcttggaactgaggtttattcgtttttaagtaagtacgtaactcaacgagaaattttc**

**Fortunella_venosa_3** **tttcttggaactgaggtttattcgtttttaagtaagtacgtaactcaacgagaaattttc**

11890	11900	11910	11920	11930	11940

....|....|....|....|....|....|....|....|....|....|....|....|

**Atalantia_kwangtungens** **ttacttgaccaatttctaatgaatcaagataaccattcgttcctgtataaatagtcatta**

**Citrus_reticulata_1** **ttacttgaccaatttctaatgaatcaagataaccattcgttcctgtataaatagtcatta**

**Citrus_reticulata_2** **ttacttgaccaatttctaatgaatcaagataaccattcgttcctgtataaatagtcatta**

**Citrus_trifoliata_3** **ttacttgaccaatttctaatgaatcaagataaccattcgttcctgtataaatagtcatta**

**Citrus_aurantiifolia** **ttacttgaccaatttctaatgaatcaagataaccattcgttcctgtataaatagtcatta**

**Citrus_aurantium** **ttacttgaccaatttctaatgaatcaagataaccattcgttcctgtataaatagtcatta**

**Citrus_cavaleriei** **ttacttgaccaatttctaatgaatcaagataaccattcgttcctgtataaatagtcatta**

**Citrus_hongheensis** **ttacttgaccaatttctaatgaatcaagataaccattcgttcctgtataaatagtcatta**

**Citrus_limon** **ttacttgaccaatttctaatgaatcaagataaccattcgttcctgtataaatagtcatta**

**Citrus_maxima** **ttacttgaccaatttctaatgaatcaagataaccattcgttcctgtataaatagtcatta**

**Citrus_medica** **ttacttgaacaatttctaatgaatcaagataaccattcgttcctgtataaatagtcatta**

**Citrus_polytrifolia** **ttacttgaccaatttctaatgaatcaagataaccattcgttcctgtataaatagtcatta**

**Citrus_reticulata** **ttacttgaccaatttctaatgaatcaagataaccattcgttcctgtataaatagtcatta**

**Citrus_sinensis** **ttacttgaccaatttctaatgaatcaagataaccattcgttcctgtataaatagtcatta**

**Fortunella_bawangica_1** **ttacttgaccaatttctaatgaatcaagataaccattcgttcctgtataaatagtcatta**

**Fortunella_bawangica_2** **ttacttgaccaatttctaatgaatcaagataaccattcgttcctgtataaatagtcatta**

**Fortunella_hindsii_1** **ttacttgaccaatttctaatgaatcaagataaccattcgttcctgtataaatagtcatta**

**Fortunella_hindsii_2** **ttacttgaccaatttctaatgaatcaagataaccattcgttcctgtataaatagtcatta**

**Fortunella_hindsii_3** **ttacttgaccaatttctaatgaatcaagataaccattcgttcctgtataaatagtcatta**

**Fortunella_japonica_1** **ttacttgaccaatttctaatgaatcaagataaccattcgttcctgtataaatagtcatta**

**Fortunella_japonica_2** **ttacttgaccaatttctaatgaatcaagataaccattcgttcctgtataaatagtcatta**

**Fortunella_japonica_3** **ttacttgaccaatttctaatgaatcaagataaccattcgttcctgtataaatagtcatta**

**Fortunella_japonica_4** **ttacttgaccaatttctaatgaatcaagataaccattcgttcctgtataaatagtcatta**

**Fortunella_japonica_5** **ttacttgaccaatttctaatgaatcaagataaccattcgttcctgtataaatagtcatta**

**Fortunella_japonica_6** **ttacttgaccaatttctaatgaatcaagataaccattcgttcctgtataaatagtcatta**

**Fortunella_japonica_7** **ttacttgaccaatttctaatgaatcaagataaccattcgttcctgtataaatagtcatta**

**Fortunella_japonica_8** **ttacttgaccaatttctaatgaatcaagataaccattcgttcctgtataaatagtcatta**

**Fortunella_japonica_9** **ttacttgaccaatttctaatgaatcaagataaccattcgttcctgtataaatagtcatta**

**Fortunella_japonica_10** **ttacttgaccaatttctaatgaatcaagataaccattcgttcctgtataaatagtcatta**

**Fortunella_japonica_11** **ttacttgaccaatttctaatgaatcaagataaccattcgttcctgtataaatagtcatta**

**Fortunella_japonica_12** **ttacttgaccaatttctaatgaatcaagataaccattcgttcctgtataaatagtcatta**

**Fortunella_margarita_1** **ttacttgaccaatttctaatgaatcaagataaccattcgttcctgtataaatagtcatta**

**Fortunella_margarita_2** **ttacttgaccaatttctaatgaatcaagataaccattcgttcctgtataaatagtcatta**

**Fortunella_venosa_1** **ttacttgaccaatttctaatgaatcaagataaccattcgttcctgtataaatagtcatta**

**Fortunella_venosa_2** **ttacttgaccaatttctaatgaatcaagataaccattcgttcctgtataaatagtcatta**

**Fortunella_venosa_3** **ttacttgaccaatttctaatgaatcaagataaccattcgttcctgtataaatagtcatta**

11950	11960	11970	11980	11990	12000

....|....|....|....|....|....|....|....|....|....|....|....|

**Atalantia_kwangtungens** **tctgttcttccaccgtgagaggggctgcttgggcctgtttgagcaactcacgtaagcgtt**

**Citrus_reticulata_1** **tctgttcttccaccgtgagaggggctgcttgggcctgtttgagcaactcacgtaagcgtt**

**Citrus_reticulata_2** **tctgttcttccaccgtgagaggggctgcttgggcctgtttgagcaactcacgtaagcgtt**

**Citrus_trifoliata_3** **tctgttcttccaccgtgagaggggctgcttgggcctgtttgagcaactcacgtaagcgtt**

**Citrus_aurantiifolia** **tctgttcttccaccgtgagaggggctgcttgggcctgtttgagcaactcacgtaagcgtt**

**Citrus_aurantium** **tctgttcttccaccgtgagaggggctgcttgggcctgtttgagcaactcacgtaagcgtt**

**Citrus_cavaleriei** **tctgttcttccaccgtgagaggggctgcttgggcctgtttgagcaactcacgtaagcgtt**

**Citrus_hongheensis** **tctgttcttccaccgtgagaggggctgcttgggcctgtttgagcaactcacgtaagcgtt**

**Citrus_limon** **tctgttcttccaccgtgagaggggctgcttgggcctgtttgagcaactcacgtaagcgtt**

**Citrus_maxima** **tctgttcttccaccgtgagaggggctgcttgggcctgtttgagcaactcacgtaagcgtt**

**Citrus_medica** **tctgttcttccaccgtgagaggggctgcttgggcctgtttgagcaactcacgtaagcgtt**

**Citrus_polytrifolia** **tctgttcttccaccgtgagaggggctgcttgggcctgtttgagcaactcacgtaagcgtt**

**Citrus_reticulata** **tctgttcttccaccgtgagaggggctgcttgggcctgtttgagcaactcacgtaagcgtt**

**Citrus_sinensis** **tctgttcttccaccgtgagaggggctgcttgggcctgtttgagcaactcacgtaagcgtt**

**Fortunella_bawangica_1** **tctgttcttccaccgtgagaggggctgcttgggcctgtttgagcaactcacgtaagcgtt**

**Fortunella_bawangica_2** **tctgttcttccaccgtgagaggggctgcttgggcctgtttgagcaactcacgtaagcgtt**

**Fortunella_hindsii_1** **tctgttcttccaccgtgagaggggctgcttgggcctgtttgagcaactcacgtaagcgtt**

**Fortunella_hindsii_2** **tctgttcttccaccgtgagaggggctgcttgggcctgtttgagcaactcacgtaagcgtt**

**Fortunella_hindsii_3** **tctgttcttccaccgtgagaggggctgcttgggcctgtttgagcaactcacgtaagcgtt**

**Fortunella_japonica_1** **tctgttcttccaccgtgagaggggctgcttgggcctgtttgagcaactcacgtaagcgtt**

**Fortunella_japonica_2** **tctgttcttccaccgtgagaggggctgcttgggcctgtttgagcaactcacgtaagcgtt**

**Fortunella_japonica_3** **tctgttcttccaccgtgagaggggctgcttgggcctgtttgagcaactcacgtaagcgtt**

**Fortunella_japonica_4** **tctgttcttccaccgtgagaggggctgcttgggcctgtttgagcaactcacgtaagcgtt**

**Fortunella_japonica_5** **tctgttcttccaccgtgagaggggctgcttgggcctgtttgagcaactcacgtaagcgtt**

**Fortunella_japonica_6** **tctgttcttccaccgtgagaggggctgcttgggcctgtttgagcaactcacgtaagcgtt**

**Fortunella_japonica_7** **tctgttcttccaccgtgagaggggctgcttgggcctgtttgagcaactcacgtaagcgtt**

**Fortunella_japonica_8** **tctgttcttccaccgtgagaggggctgcttgggcctgtttgagcaactcacgtaagcgtt**

**Fortunella_japonica_9** **tctgttcttccaccgtgagaggggctgcttgggcctgtttgagcaactcacgtaagcgtt**

**Fortunella_japonica_10** **tctgttcttccaccgtgagaggggctgcttgggcctgtttgagcaactcacgtaagcgtt**

**Fortunella_japonica_11** **tctgttcttccaccgtgagaggggctgcttgggcctgtttgagcaactcacgtaagcgtt**

**Fortunella_japonica_12** **tctgttcttccaccgtgagaggggctgcttgggcctgtttgagcaactcacgtaagcgtt**

**Fortunella_margarita_1** **tctgttcttccaccgtgagaggggctgcttgggcctgtttgagcaactcacgtaagcgtt**

**Fortunella_margarita_2** **tctgttcttccaccgtgagaggggctgcttgggcctgtttgagcaactcacgtaagcgtt**

**Fortunella_venosa_1** **tctgttcttccaccgtgagaggggctgcttgggcctgtttgagcaactcacgtaagcgtt**

**Fortunella_venosa_2** **tctgttcttccaccgtgagaggggctgcttgggcctgtttgagcaactcacgtaagcgtt**

**Fortunella_venosa_3** **tctgttcttccaccgtgagaggggctgcttgggcctgtttgagcaactcacgtaagcgtt**

12010	12020	12030	12040	12050	12060

....|....|....|....|....|....|....|....|....|....|....|....|

**Atalantia_kwangtungens** **gaccccttgccaattggttctgagtagctttatcgagatcagaagcaaattgtgcaaagg**

**Citrus_reticulata_1** **gaccccttgccaattggttctgagtagctttatcgagatcagaagcaaattgtgcaaagg**

**Citrus_reticulata_2** **gaccccttgccaattggttctgagtagctttatcgagatcagaagcaaattgtgcaaagg**

**Citrus_trifoliata_3** **gaccccttgccaattggttctgagtagctttatcgagatcagaagcaaattgtgcaaagg**

**Citrus_aurantiifolia** **gaccccttgccaattggttctgagtagctttatcgagatcagaagcaaattgtgcaaagg**

**Citrus_aurantium** **gaccccttgccaattggttctgagtagctttatcgagatcagaagcaaattgtgcaaagg**

**Citrus_cavaleriei** **gaccccttgccaattggttctgagtagctttatcgagatcagaagcaaattgtgcaaagg**

**Citrus_hongheensis** **gaccccttgccaattggttctgagtagctttatcgagatcagaagcaaattgtgcaaagg**

**Citrus_limon** **gaccccttgccaattggttctgagtagctttatcgagatcagaagcaaattgtgcaaagg**

**Citrus_maxima** **gaccccttgccaattggttctgagtagctttatcgagatcagaagcaaattgtgcaaagg**

**Citrus_medica** **gaccccttgccaattggttctgagtagctttatcgagatcagaagcaaattgtgcaaagg**

**Citrus_polytrifolia** **gaccccttgccaattggttctgagtagctttatcgagatcagaagcaaattgtgcaaagg**

**Citrus_reticulata** **gaccccttgccaattggttctgagtagctttatcgagatcagaagcaaattgtgcaaagg**

**Citrus_sinensis** **gaccccttgccaattggttctgagtagctttatcgagatcagaagcaaattgtgcaaagg**

**Fortunella_bawangica_1** **gaccccttgccaattggttctgagtagctttatcgagatcagaagcaaattgtgcaaagg**

**Fortunella_bawangica_2** **gaccccttgccaattggttctgagtagctttatcgagatcagaagcaaattgtgcaaagg**

**Fortunella_hindsii_1** **gaccccttgccaattggttctgagtagctttatcgagatcagaagcaaattgtgcaaagg**

**Fortunella_hindsii_2** **gaccccttgccaattggttctgagtagctttatcgagatcagaagcaaattgtgcaaagg**

**Fortunella_hindsii_3** **gaccccttgccaattggttctgagtagctttatcgagatcagaagcaaattgtgcaaagg**

**Fortunella_japonica_1** **gaccccttgccaattggttctgagtagctttatcgagatcagaagcaaattgtgcaaagg**

**Fortunella_japonica_2** **gaccccttgccaattggttctgagtagctttatcgagatcagaagcaaattgtgcaaagg**

**Fortunella_japonica_3** **gaccccttgccaattggttctgagtagctttatcgagatcagaagcaaattgtgcaaagg**

**Fortunella_japonica_4** **gaccccttgccaattggttctgagtagctttatcgagatcagaagcaaattgtgcaaagg**

**Fortunella_japonica_5** **gaccccttgccaattggttctgagtagctttatcgagatcagaagcaaattgtgcaaagg**

**Fortunella_japonica_6** **gaccccttgccaattggttctgagtagctttatcgagatcagaagcaaattgtgcaaagg**

**Fortunella_japonica_7** **gaccccttgccaattggttctgagtagctttatcgagatcagaagcaaattgtgcaaagg**

**Fortunella_japonica_8** **gaccccttgccaattggttctgagtagctttatcgagatcagaagcaaattgtgcaaagg**

**Fortunella_japonica_9** **gaccccttgccaattggttctgagtagctttatcgagatcagaagcaaattgtgcaaagg**

**Fortunella_japonica_10** **gaccccttgccaattggttctgagtagctttatcgagatcagaagcaaattgtgcaaagg**

**Fortunella_japonica_11** **gaccccttgccaattggttctgagtagctttatcgagatcagaagcaaattgtgcaaagg**

**Fortunella_japonica_12** **gaccccttgccaattggttctgagtagctttatcgagatcagaagcaaattgtgcaaagg**

**Fortunella_margarita_1** **gaccccttgccaattggttctgagtagctttatcgagatcagaagcaaattgtgcaaagg**

**Fortunella_margarita_2** **gaccccttgccaattggttctgagtagctttatcgagatcagaagcaaattgtgcaaagg**

**Fortunella_venosa_1** **gaccccttgccaattggttctgagtagctttatcgagatcagaagcaaattgtgcaaagg**

**Fortunella_venosa_2** **gaccccttgccaattggttctgagtagctttatcgagatcagaagcaaattgtgcaaagg**

**Fortunella_venosa_3** **gaccccttgccaattggttctgagtagctttatcgagatcagaagcaaattgtgcaaagg**

12070	12080	12090	12100	12110	12120

....|....|....|....|....|....|....|....|....|....|....|....|

**Atalantia_kwangtungens** **cttctaattctgcgaattgtgccaattccaattttaatttgccagctacttgtttcatag**

**Citrus_reticulata_1** **cttctaattctgcgaattgtgccaattccaattttaatttgccggctacttgtttcatag**

**Citrus_reticulata_2** **cttctaattctgcgaattgtgccaattccaattttaatttgccggctacttgtttcatag**

**Citrus_trifoliata_3** **cttctaattctgcgaattgtgccaattccaattttaatttgccggctacttgtttcatag**

**Citrus_aurantiifolia** **cttctaattctgcgaattgtgccaattccaattttaatttgccggctacttgtttcatag**

**Citrus_aurantium** **cttctaattctgcgaattgtgccaattccaattttaatttgccggctacttgtttcatag**

**Citrus_cavaleriei** **cttctaattctgcgaattgtgccaattccaattttaatttgccggctacttgtttcatag**

**Citrus_hongheensis** **cttctaattctgcgaattgtgccaattccaattttaatttgccggctacttgtttcatag**

**Citrus_limon** **cttctaattctgcgaattgtgccaattccaattttaatttgccggctacttgtttcatag**

**Citrus_maxima** **cttctaattctgcgaattgtgccaattccaattttaatttgccggctacttgtttcatag**

**Citrus_medica** **cttctaattctgcgaattgtgccaattccaattttaatttgccggctacttgtttcatag**

**Citrus_polytrifolia** **cttctaattctgcgaattgtgccaattccaattttaatttgccggctacttgtttcatag**

**Citrus_reticulata** **cttctaattctgcgaattgtgccaattccaattttaatttgccggctacttgtttcatag**

**Citrus_sinensis** **cttctaattctgcgaattgtgccaattccaattttaatttgccggctacttgtttcatag**

**Fortunella_bawangica_1** **cttctaattctgcgaattgtgccaattccaattttaatttgccggctacttgtttcatag**

**Fortunella_bawangica_2** **cttctaattctgcgaattgtgccaattccaattttaatttgccggctacttgtttcatag**

**Fortunella_hindsii_1** **cttctaattctgcgaattgtgccaattccaattttaatttgccggctacttgtttcatag**

**Fortunella_hindsii_2** **cttctaattctgcgaattgtgccaattccaattttaatttgccggctacttgtttcatag**

**Fortunella_hindsii_3** **cttctaattctgcgaattgtgccaattccaattttaatttgccggctacttgtttcatag**

**Fortunella_japonica_1** **cttctaattctgcgaattgtgccaattccaattttaatttgccggctacttgtttcatag**

**Fortunella_japonica_2** **cttctaattctgcgaattgtgccaattccaattttaatttgccggctacttgtttcatag**

**Fortunella_japonica_3** **cttctaattctgcgaattgtgccaattccaattttaatttgccggctacttgtttcatag**

**Fortunella_japonica_4** **cttctaattctgcgaattgtgccaattccaattttaatttgccggctacttgtttcatag**

**Fortunella_japonica_5** **cttctaattctgcgaattgtgccaattccaattttaatttgccggctacttgtttcatag**

**Fortunella_japonica_6** **cttctaattctgcgaattgtgccaattccaattttaatttgccggctacttgtttcatag**

**Fortunella_japonica_7** **cttctaattctgcgaattgtgccaattccaattttaatttgccggctacttgtttcatag**

**Fortunella_japonica_8** **cttctaattctgcgaattgtgccaattccaattttaatttgccggctacttgtttcatag**

**Fortunella_japonica_9** **cttctaattctgcgaattgtgccaattccaattttaatttgccggctacttgtttcatag**

**Fortunella_japonica_10** **cttctaattctgcgaattgtgccaattccaattttaatttgccggctacttgtttcatag**

**Fortunella_japonica_11** **cttctaattctgcgaattgtgccaattccaattttaatttgccggctacttgtttcatag**

**Fortunella_japonica_12** **cttctaattctgcgaattgtgccaattccaattttaatttgccggctacttgtttcatag**

**Fortunella_margarita_1** **cttctaattctgcgaattgtgccaattccaattttaatttgccggctacttgtttcatag**

**Fortunella_margarita_2** **cttctaattctgcgaattgtgccaattccaattttaatttgccggctacttgtttcatag**

**Fortunella_venosa_1** **cttctaattctgcgaattgtgccaattccaattttaatttgccggctacttgtttcatag**

**Fortunella_venosa_2** **cttctaattctgcgaattgtgccaattccaattttaatttgccggctacttgtttcatag**

**Fortunella_venosa_3** **cttctaattctgcgaattgtgccaattccaattttaatttgccggctacttgtttcatag**

12130	12140	12150	12160	12170	12180

....|....|....|....|....|....|....|....|....|....|....|....|

**Atalantia_kwangtungens** **ctttaatttgagctgcggatcctactctagagacggaaatacccacattaatagcgggtc**

**Citrus_reticulata_1** **ctttaatttgagctgcggatcctactcgagagacggaaatacccacattaatagcgggtc**

**Citrus_reticulata_2** **ctttaatttgagctgcggatcctactcgagagacggaaatacccacattaatagcgggtc**

**Citrus_trifoliata_3** **ctttaatttgagctgcggatcctactcgcgagacggaaatacccacattaatagcgggtc**

**Citrus_aurantiifolia** **ctttaatttgagctgcggatcctactcgagagacggaaatacccacattaatagcgggtc**

**Citrus_aurantium** **ctttaatttgagctgcggatcctactcgagagacggaaatacccacattaatagcgggtc**

**Citrus_cavaleriei** **ctttaatttgagctgcggatcctactcgagagacggaaatacccacattaatagcgggtc**

**Citrus_hongheensis** **ctttaatttgagctgcggatcctactcgagagacggaaatacccacattaatagcgggtc**

**Citrus_limon** **ctttaatttgagctgcggatcctactcgagagacggaaatacccacattaatagcgggtc**

**Citrus_maxima** **ctttaatttgagctgcggatcctactcgagagacggaaatacccacattaatagcgggtc**

**Citrus_medica** **ctttaatttgagctgcggatcctactcgagagacggaaatacccacattaatagcgggtc**

**Citrus_polytrifolia** **ctttaatttgagctgcggatcctactcgcgagacggaaatacccacattaatagcgggtc**

**Citrus_reticulata** **ctttaatttgagctgcggatcctactcgagagacggaaatacccacattaatagcgggtc**

**Citrus_sinensis** **ctttaatttgagctgcggatcctactcgagagacggaaatacccacattaatagcgggtc**

**Fortunella_bawangica_1** **ctttaatttgagctgcggatcctactcgagagacggaaatacccacattaatagcgggtc**

**Fortunella_bawangica_2** **ctttaatttgagctgcggatcctactcgagagacggaaatacccacattaatagcgggtc**

**Fortunella_hindsii_1** **ctttaatttgagctgcggatcctactcgagagacggaaatacccacattaatagcgggtc**

**Fortunella_hindsii_2** **ctttaatttgagctgcggatcctactcgagagacggaaatacccacattaatagcgggtc**

**Fortunella_hindsii_3** **ctttaatttgagctgcggatcctactcgagagacggaaatacccacattaatagcgggtc**

**Fortunella_japonica_1** **ctttaatttgagctgcggatcctactcgagagacggaaatacccacattaatagcgggtc**

**Fortunella_japonica_2** **ctttaatttgagctgcggatcctactcgagagacggaaatacccacattaatagcgggtc**

**Fortunella_japonica_3** **ctttaatttgagctgcggatcctactcgagagacggaaatacccacattaatagcgggtc**

**Fortunella_japonica_4** **ctttaatttgagctgcggatcctactcgagagacggaaatacccacattaatagcgggtc**

**Fortunella_japonica_5** **ctttaatttgagctgcggatcctactcgagagacggaaatacccacattaatagcgggtc**

**Fortunella_japonica_6** **ctttaatttgagctgcggatcctactcgagagacggaaatacccacattaatagcgggtc**

**Fortunella_japonica_7** **ctttaatttgagctgcggatcctactcgagagacggaaatacccacattaatagcgggtc**

**Fortunella_japonica_8** **ctttaatttgagctgcggatcctactcgagagacggaaatacccacattaatagcgggtc**

**Fortunella_japonica_9** **ctttaatttgagctgcggatcctactcgagagacggaaatacccacattaatagcgggtc**

**Fortunella_japonica_10** **ctttaatttgagctgcggatcctactcgagagacggaaatacccacattaatagcgggtc**

**Fortunella_japonica_11** **ctttaatttgagctgcggatcctactcgagagacggaaatacccacattaatagcgggtc**

**Fortunella_japonica_12** **ctttaatttgagctgcggatcctactcgagagacggaaatacccacattaatagcgggtc**

**Fortunella_margarita_1** **ctttaatttgagctgcggatcctactcgagagacggaaatacccacattaatagcgggtc**

**Fortunella_margarita_2** **ctttaatttgagctgcggatcctactcgagagacggaaatacccacattaatagcgggtc**

**Fortunella_venosa_1** **ctttaatttgagctgcggatcctactcgagagacggaaatacccacattaatagcgggtc**

**Fortunella_venosa_2** **ctttaatttgagctgcggatcctactcgagagacggaaatacccacattaatagcgggtc**

**Fortunella_venosa_3** **ctttaatttgagctgcggatcctactcgagagacggaaatacccacattaatagcgggtc**

12190	12200	12210	12220	12230	12240

....|....|....|....|....|....|....|....|....|....|....|....|

**Atalantia_kwangtungens** **tgattccagcattgaataaatccgcggataagaatatttgcccgtctgtaatggaaatta**

**Citrus_reticulata_1** **tgattccagcattgaataaatccgcggataagaatatttgcccgtctgtaatggaaatta**

**Citrus_reticulata_2** **tgattccagcattgaataaatccgcggataagaatatttgcccgtctgtaatggaaatta**

**Citrus_trifoliata_3** **tgattccagcattgaataaatccgcggataagaatatttgcccgtctgtaatggaaatta**

**Citrus_aurantiifolia** **tgattccagcattgaataaatccgcggataagaatatttgcccgtctgtaatggaaatta**

**Citrus_aurantium** **tgattccagcattgaataaatccgcggataagaatatttgcccgtctgtaatggaaatta**

**Citrus_cavaleriei** **tgattccagcattgaataaatccgcggataagaatatttgcccgtctgtaatggaaatta**

**Citrus_hongheensis** **tgattccagcattgaataaatccgcggataagaatatttgcccgtctgtaatggaaatta**

**Citrus_limon** **tgattccagcattgaataaatccgcggataagaatatttgcccgtctgtaatggaaatta**

**Citrus_maxima** **tgattccagcattgaataaatccgcggataagaatatttgcccgtctgtaatggaaatta**

**Citrus_medica** **tgattccagcattgaataaatccgcggataagaatatttgcccgtctgtaatggaaatta**

**Citrus_polytrifolia** **tgattccagcattgaataaatccgcggataagaatatttgcccgtctgtaatggaaatta**

**Citrus_reticulata** **tgattccagcattgaataaatccgcggataagaatatttgcccgtctgtaatggaaatta**

**Citrus_sinensis** **tgattccagcattgaataaatccgcggataagaatatttgcccgtctgtaatggaaatta**

**Fortunella_bawangica_1** **tgattccagcattgaataaatccgcggataagaatatttgcccgtctgtaatggaaatta**

**Fortunella_bawangica_2** **tgattccagcattgaataaatccgcggataagaatatttgcccgtctgtaatggaaatta**

**Fortunella_hindsii_1** **tgattccagcattgaataaatccgcggataagaatatttgcccgtctgtaatggaaatta**

**Fortunella_hindsii_2** **tgattccagcattgaataaatccgcggataagaatatttgcccgtctgtaatggaaatta**

**Fortunella_hindsii_3** **tgattccagcattgaataaatccgcggataagaatatttgcccgtctgtaatggaaatta**

**Fortunella_japonica_1** **tgattccagcattgaataaatccgcggataagaatatttgcccgtctgtaatggaaatta**

**Fortunella_japonica_2** **tgattccagcattgaataaatccgcggataagaatatttgcccgtctgtaatggaaatta**

**Fortunella_japonica_3** **tgattccagcattgaataaatccgcggataagaatatttgcccgtctgtaatggaaatta**

**Fortunella_japonica_4** **tgattccagcattgaataaatccgcggataagaatatttgcccgtctgtaatggaaatta**

**Fortunella_japonica_5** **tgattccagcattgaataaatccgcggataagaatatttgcccgtctgtaatggaaatta**

**Fortunella_japonica_6** **tgattccagcattgaataaatccgcggataagaatatttgcccgtctgtaatggaaatta**

**Fortunella_japonica_7** **tgattccagcattgaataaatccgcggataagaatatttgcccgtctgtaatggaaatta**

**Fortunella_japonica_8** **tgattccagcattgaataaatccgcggataagaatatttgcccgtctgtaatggaaatta**

**Fortunella_japonica_9** **tgattccagcattgaataaatccgcggataagaatatttgcccgtctgtaatggaaatta**

**Fortunella_japonica_10** **tgattccagcattgaataaatccgcggataagaatatttgcccgtctgtaatggaaatta**

**Fortunella_japonica_11** **tgattccagcattgaataaatccgcggataagaatatttgcccgtctgtaatggaaatta**

**Fortunella_japonica_12** **tgattccagcattgaataaatccgcggataagaatatttgcccgtctgtaatggaaatta**

**Fortunella_margarita_1** **tgattccagcattgaataaatccgcggataagaatatttgcccgtctgtaatggaaatta**

**Fortunella_margarita_2** **tgattccagcattgaataaatccgcggataagaatatttgcccgtctgtaatggaaatta**

**Fortunella_venosa_1** **tgattccagcattgaataaatccgcggataagaatatttgcccgtctgtaatggaaatta**

**Fortunella_venosa_2** **tgattccagcattgaataaatccgcggataagaatatttgcccgtctgtaatggaaatta**

**Fortunella_venosa_3** **tgattccagcattgaataaatccgcggataagaatatttgcccgtctgtaatggaaatta**

12250	12260	12270	12280	12290	12300

....|....|....|....|....|....|....|....|....|....|....|....|

**Atalantia_kwangtungens** **cattagtaggaatataagccgaaacatctcccgactgggtctcaactattggtaaagcag**

**Citrus_reticulata_1** **cattagtaggaatataagccgaaacatctcccgactgggtctcaactattggtaaagcgg**

**Citrus_reticulata_2** **cattagtaggaatataagccgaaacatctcccgactgggtctcaactattggtaaagcgg**

**Citrus_trifoliata_3** **cattagtaggaatataagccgaaacatctcccgactgggtctcaactattggtaaagcgg**

**Citrus_aurantiifolia** **cattagtaggaatataagccgaaacatctcccgactgggtctcaactattggtaaagcgg**

**Citrus_aurantium** **cattagtaggaatataagccgaaacatctcccgactgggtctcaactattggtaaagcgg**

**Citrus_cavaleriei** **cattagtaggaatataagccgaaacatctcccgactgggtctcaactattggtaaagcgg**

**Citrus_hongheensis** **cattagtaggaatataagccgaaacatctcccgactgggtctcaactattggtaaagcgg**

**Citrus_limon** **cattagtaggaatataagccgaaacatctcccgactgggtctcaactattggtaaagcgg**

**Citrus_maxima** **cattagtaggaatataagccgaaacatctcccgactgggtctcaactattggtaaagcgg**

**Citrus_medica** **cattagtaggaatataagccgaaacatctcccgactgggtctcaactattggtaaagcgg**

**Citrus_polytrifolia** **cattagtaggaatataagccgaaacatctcccgactgggtctcaactattggtaaagcgg**

**Citrus_reticulata** **cattagtaggaatataagccgaaacatctcccgactgggtctcaactattggtaaagcgg**

**Citrus_sinensis** **cattagtaggaatataagccgaaacatctcccgactgggtctcaactattggtaaagcgg**

**Fortunella_bawangica_1** **cattagtaggaatataagccgaaacatctcccgactgggtctcaactattggtaaagcgg**

**Fortunella_bawangica_2** **cattagtaggaatataagccgaaacatctcccgactgggtctcaactattggtaaagcgg**

**Fortunella_hindsii_1** **cattagtaggaatataagccgaaacatctcccgactgggtctcaactattggtaaagcgg**

**Fortunella_hindsii_2** **cattagtaggaatataagccgaaacatctcccgactgggtctcaactattggtaaagcgg**

**Fortunella_hindsii_3** **cattagtaggaatataagccgaaacatctcccgactgggtctcaactattggtaaagcgg**

**Fortunella_japonica_1** **cattagtaggaatataagccgaaacatctcccgactgggtctcaactattggtaaagcgg**

**Fortunella_japonica_2** **cattagtaggaatataagccgaaacatctcccgactgggtctcaactattggtaaagcgg**

**Fortunella_japonica_3** **cattagtaggaatataagccgaaacatctcccgactgggtctcaactattggtaaagcgg**

**Fortunella_japonica_4** **cattagtaggaatataagccgaaacatctcccgactgggtctcaactattggtaaagcgg**

**Fortunella_japonica_5** **cattagtaggaatataagccgaaacatctcccgactgggtctcaactattggtaaagcgg**

**Fortunella_japonica_6** **cattagtaggaatataagccgaaacatctcccgactgggtctcaactattggtaaagcgg**

**Fortunella_japonica_7** **cattagtaggaatataagccgaaacatctcccgactgggtctcaactattggtaaagcgg**

**Fortunella_japonica_8** **cattagtaggaatataagccgaaacatctcccgactgggtctcaactattggtaaagcgg**

**Fortunella_japonica_9** **cattagtaggaatataagccgaaacatctcccgactgggtctcaactattggtaaagcgg**

**Fortunella_japonica_10** **cattagtaggaatataagccgaaacatctcccgactgggtctcaactattggtaaagcgg**

**Fortunella_japonica_11** **cattagtaggaatataagccgaaacatctcccgactgggtctcaactattggtaaagcgg**

**Fortunella_japonica_12** **cattagtaggaatataagccgaaacatctcccgactgggtctcaactattggtaaagcgg**

**Fortunella_margarita_1** **cattagtaggaatataagccgaaacatctcccgactgggtctcaactattggtaaagcgg**

**Fortunella_margarita_2** **cattagtaggaatataagccgaaacatctcccgactgggtctcaactattggtaaagcgg**

**Fortunella_venosa_1** **cattagtaggaatataagccgaaacatctcccgactgggtctcaactattggtaaagcgg**

**Fortunella_venosa_2** **cattagtaggaatataagccgaaacatctcccgactgggtctcaactattggtaaagcgg**

**Fortunella_venosa_3** **cattagtaggaatataagccgaaacatctcccgactgggtctcaactattggtaaagcgg**

12310	12320	12330	12340	12350	12360

....|....|....|....|....|....|....|....|....|....|....|....|

**Atalantia_kwangtungens** **tcatacttccttcacctaactgcgaacctaatttcgcggctctttccaaaagtcgtgaat**

**Citrus_reticulata_1** **tcatacttccttcgcctaactgcgaacctaatttcgcggctctttccaaaagtcgtgaat**

**Citrus_reticulata_2** **tcatacttccttcgcctaactgcgaacctaatttcgcggctctttccaaaagtcgtgaat**

**Citrus_trifoliata_3** **tcatacttccttcgcctaactgcgaacctaatttcgcggctctttccaaaagtcgtgaat**

**Citrus_aurantiifolia** **tcatacttccttcgcctaactgcgaacctaatttcgcggctctttccaaaagtcgtgaat**

**Citrus_aurantium** **tcatacttccttcgcctaactgcgaacctaatttcgcggctctttccaaaagtcgtgaat**

**Citrus_cavaleriei** **tcatacttccttcgcctaactgcgaacctaatttcgcggctctttccaaaagtcgtgaat**

**Citrus_hongheensis** **tcatacttccttcgcctaactgcgaacctaatttcgcggctctttccaaaagtcgtgaat**

**Citrus_limon** **tcatacttccttcgcctaactgcgaacctaatttcgcggctctttccaaaagtcgtgaat**

**Citrus_maxima** **tcatacttccttcgcctaactgcgaacctaatttcgcggctctttccaaaagtcgtgaat**

**Citrus_medica** **tcatacttccttcgcctaactgcgaacctaatttagcggctctttccaaaagtcgtgaat**

**Citrus_polytrifolia** **tcatacttccttcgcctaactgcgaacctaatttcgcggctctttccaaaagtcgtgaat**

**Citrus_reticulata** **tcatacttccttcgcctaactgcgaacctaatttcgcggctctttccaaaagtcgtgaat**

**Citrus_sinensis** **tcatacttccttcgcctaactgcgaacctaatttcgcggctctttccaaaagtcgtgaat**

**Fortunella_bawangica_1** **tcatacttccttcgcctaactgcgaacctaatttcgcggctctttccaaaagtcgtgaat**

**Fortunella_bawangica_2** **tcatacttccttcgcctaactgcgaacctaatttcgcggctctttccaaaagtcgtgaat**

**Fortunella_hindsii_1** **tcatacttccttcgcctaactgcgaacctaatttcgcggctctttccaaaagtcgtgaat**

**Fortunella_hindsii_2** **tcatacttccttcgcctaactgcgaacctaatttcgcggctctttccaaaagtcgtgaat**

**Fortunella_hindsii_3** **tcatacttccttcgcctaactgcgaacctaatttcgcggctctttccaaaagtcgtgaat**

**Fortunella_japonica_1** **tcatacttccttcgcctaactgcgaacctaatttcgcggctctttccaaaagtcgtgaat**

**Fortunella_japonica_2** **tcatacttccttcgcctaactgcgaacctaatttcgcggctctttccaaaagtcgtgaat**

**Fortunella_japonica_3** **tcatacttccttcgcctaactgcgaacctaatttcgcggctctttccaaaagtcgtgaat**

**Fortunella_japonica_4** **tcatacttccttcgcctaactgcgaacctaatttcgcggctctttccaaaagtcgtgaat**

**Fortunella_japonica_5** **tcatacttccttcgcctaactgcgaacctaatttcgcggctctttccaaaagtcgtgaat**

**Fortunella_japonica_6** **tcatacttccttcgcctaactgcgaacctaatttcgcggctctttccaaaagtcgtgaat**

**Fortunella_japonica_7** **tcatacttccttcgcctaactgcgaacctaatttcgcggctctttccaaaagtcgtgaat**

**Fortunella_japonica_8** **tcatacttccttcgcctaactgcgaacctaatttcgcggctctttccaaaagtcgtgaat**

**Fortunella_japonica_9** **tcatacttccttcgcctaactgcgaacctaatttcgcggctctttccaaaagtcgtgaat**

**Fortunella_japonica_10** **tcatacttccttcgcctaactgcgaacctaatttcgcggctctttccaaaagtcgtgaat**

**Fortunella_japonica_11** **tcatacttccttcgcctaactgcgaacctaatttcgcggctctttccaaaagtcgtgaat**

**Fortunella_japonica_12** **tcatacttccttcgcctaactgcgaacctaatttcgcggctctttccaaaagtcgtgaat**

**Fortunella_margarita_1** **tcatacttccttcgcctaactgcgaacctaatttcgcggctctttccaaaagtcgtgaat**

**Fortunella_margarita_2** **tcatacttccttcgcctaactgcgaacctaatttcgcggctctttccaaaagtcgtgaat**

**Fortunella_venosa_1** **tcatacttccttcgcctaactgcgaacctaatttcgcggctctttccaaaagtcgtgaat**

**Fortunella_venosa_2** **tcatacttccttcgcctaactgcgaacctaatttcgcggctctttccaaaagtcgtgaat**

**Fortunella_venosa_3** **tcatacttccttcgcctaactgcgaacctaatttcgcggctctttccaaaagtcgtgaat**

12370	12380	12390	12400	12410	12420

....|....|....|....|....|....|....|....|....|....|....|....|

**Atalantia_kwangtungens** **gcaaataaaaaacatctccgggataagcttcgcgaccaggtggtcttcgtaatagaagag**

**Citrus_reticulata_1** **gcaaataaaaaacatctccgggataagcttcgcgacccggtggtcttcgtaatagaagag**

**Citrus_reticulata_2** **gcaaataaaaaacatctccgggataagcttcgcgacccggtggtcttcgtaatagaagag**

**Citrus_trifoliata_3** **gcaaataaaaaacatctccgggataagcttcgcgacccggtggtcttcgtaatagaagag**

**Citrus_aurantiifolia** **gcaaataaaaaacatctccgggataagcttcgcgacccggtggtcttcgtaatagaagag**

**Citrus_aurantium** **gcaaataaaaaacatctccgggataagcttcgcgacccggtggtcttcgtaatagaagag**

**Citrus_cavaleriei** **gcaaataaaaaacatctccgggataagcttcgcgacccggtggtcttcgtaatagaagag**

**Citrus_hongheensis** **gcaaataaaaaacatctccgggataagcttcgcgacccggtggtcttcgtaatagaagag**

**Citrus_limon** **gcaaataaaaaacatctccgggataagcttcgcgacccggtggtcttcgtaatagaagag**

**Citrus_maxima** **gcaaataaaaaacatctccgggataagcttcgcgacccggtggtcttcgtaatagaagag**

**Citrus_medica** **gcaaataaaaaacatctccgggataagcttcgcgacccggtggtcttcgtaatagaagag**

**Citrus_polytrifolia** **gcaaataaaaaacatctccgggataagcttcgcgacccggtggtcttcgtaatagaagag**

**Citrus_reticulata** **gcaaataaaaaacatctccgggataagcttcgcgacccggtggtcttcgtaatagaagag**

**Citrus_sinensis** **gcaaataaaaaacatctccgggataagcttcgcgacccggtggtcttcgtaatagaagag**

**Fortunella_bawangica_1** **gcaaataaaaaacatctccgggataagcttcgcgacccggtggtcttcgtaatagaagag**

**Fortunella_bawangica_2** **gcaaataaaaaacatctccgggataagcttcgcgacccggtggtcttcgtaatagaagag**

**Fortunella_hindsii_1** **gcaaataaaaaacatctccgggataagcttcgcgacccggtggtcttcgtaatagaagag**

**Fortunella_hindsii_2** **gcaaataaaaaacatctccgggataagcttcgcgacccggtggtcttcgtaatagaagag**

**Fortunella_hindsii_3** **gcaaataaaaaacatctccgggataagcttcgcgacccggtggtcttcgtaatagaagag**

**Fortunella_japonica_1** **gcaaataaaaaacatctccgggataagcttcgcgacccggtggtcttcgtaatagaagag**

**Fortunella_japonica_2** **gcaaataaaaaacatctccgggataagcttcgcgacccggtggtcttcgtaatagaagag**

**Fortunella_japonica_3** **gcaaataaaaaacatctccgggataagcttcgcgacccggtggtcttcgtaatagaagag**

**Fortunella_japonica_4** **gcaaataaaaaacatctccgggataagcttcgcgacccggtggtcttcgtaatagaagag**

**Fortunella_japonica_5** **gcaaataaaaaacatctccgggataagcttcgcgacccggtggtcttcgtaatagaagag**

**Fortunella_japonica_6** **gcaaataaaaaacatctccgggataagcttcgcgacccggtggtcttcgtaatagaagag**

**Fortunella_japonica_7** **gcaaataaaaaacatctccgggataagcttcgcgacccggtggtcttcgtaatagaagag**

**Fortunella_japonica_8** **gcaaataaaaaacatctccgggataagcttcgcgacccggtggtcttcgtaatagaagag**

**Fortunella_japonica_9** **gcaaataaaaaacatctccgggataagcttcgcgacccggtggtcttcgtaatagaagag**

**Fortunella_japonica_10** **gcaaataaaaaacatctccgggataagcttcgcgacccggtggtcttcgtaatagaagag**

**Fortunella_japonica_11** **gcaaataaaaaacatctccgggataagcttcgcgacccggtggtcttcgtaatagaagag**

**Fortunella_japonica_12** **gcaaataaaaaacatctccgggataagcttcgcgacccggtggtcttcgtaatagaagag**

**Fortunella_margarita_1** **gcaaataaaaaacatctccgggataagcttcgcgacccggtggtcttcgtaatagaagag**

**Fortunella_margarita_2** **gcaaataaaaaacatctccgggataagcttcgcgacccggtggtcttcgtaatagaagag**

**Fortunella_venosa_1** **gcaaataaaaaacatctccgggataagcttcgcgacccggtggtcttcgtaatagaagag**

**Fortunella_venosa_2** **gcaaataaaaaacatctccgggataagcttcgcgacccggtggtcttcgtaatagaagag**

**Fortunella_venosa_3** **gcaaataaaaaacatctccgggataagcttcgcgacccggtggtcttcgtaatagaagag**

12430	12440	12450	12460	12470	12480

....|....|....|....|....|....|....|....|....|....|....|....|

**Atalantia_kwangtungens** **acatttgtcgataagcctgtgcttgtttggagggatcatcataaatgattaaagtgtgtc**

**Citrus_reticulata_1** **acatttgtcgataagcctgtgcttgtttggagggatcatcataaatgattaaagtgtgtc**

**Citrus_reticulata_2** **acatttgtcgataagcctgtgcttgtttggagggatcatcataaatgattaaagtgtgtc**

**Citrus_trifoliata_3** **acatttgtcgataagcctgtgcttgtttggagggatcatcataaatgattaaagtgtgtc**

**Citrus_aurantiifolia** **acatttgtcgataagcctgtgcttgtttggagggatcatcataaatgattaaagtgtgtc**

**Citrus_aurantium** **acatttgtcgataagcctgtgcttgtttggagggatcatcataaatgattaaagtgtgtc**

**Citrus_cavaleriei** **acatttgtcgataagcctgtgcttgtttggagggatcatcataaatgattaaagtgtgtc**

**Citrus_hongheensis** **acatttgtcgataagcctgtgcttgtttggagggatcatcataaatgattaaagtgtgtc**

**Citrus_limon** **acatttgtcgataagcctgtgcttgtttggagggatcatcataaatgattaaagtgtgtc**

**Citrus_maxima** **acatttgtcgataagcctgtgcttgtttggagggatcatcataaatgattaaagtgtgtc**

**Citrus_medica** **acatttgtcgataagcctgtgcttgtttggagggatcatcataaatgattaaagtgtgtc**

**Citrus_polytrifolia** **acatttgtcgataagcctgtgcttgtttggagggatcatcataaatgattaaagtgtgtc**

**Citrus_reticulata** **acatttgtcgataagcctgtgcttgtttggagggatcatcataaatgattaaagtgtgtc**

**Citrus_sinensis** **acatttgtcgataagcctgtgcttgtttggagggatcatcataaatgattaaagtgtgtc**

**Fortunella_bawangica_1** **acatttgtcgataagcctgtgcttgtttggagggatcatcataaatgattaaagtgtgtc**

**Fortunella_bawangica_2** **acatttgtcgataagcctgtgcttgtttggagggatcatcataaatgattaaagtgtgtc**

**Fortunella_hindsii_1** **acatttgtcgataagcctgtgcttgtttggagggatcatcataaatgattaaagtgtgtc**

**Fortunella_hindsii_2** **acatttgtcgataagcctgtgcttgtttggagggatcatcataaatgattaaagtgtgtc**

**Fortunella_hindsii_3** **acatttgtcgataagcctgtgcttgtttggagggatcatcataaatgattaaagtgtgtc**

**Fortunella_japonica_1** **acatttgtcgataagcctgtgcttgtttggagggatcatcataaatgattaaagtgtgtc**

**Fortunella_japonica_2** **acatttgtcgataagcctgtgcttgtttggagggatcatcataaatgattaaagtgtgtc**

**Fortunella_japonica_3** **acatttgtcgataagcctgtgcttgtttggagggatcatcataaatgattaaagtgtgtc**

**Fortunella_japonica_4** **acatttgtcgataagcctgtgcttgtttggagggatcatcataaatgattaaagtgtgtc**

**Fortunella_japonica_5** **acatttgtcgataagcctgtgcttgtttggagggatcatcataaatgattaaagtgtgtc**

**Fortunella_japonica_6** **acatttgtcgataagcctgtgcttgtttggagggatcatcataaatgattaaagtgtgtc**

**Fortunella_japonica_7** **acatttgtcgataagcctgtgcttgtttggagggatcatcataaatgattaaagtgtgtc**

**Fortunella_japonica_8** **acatttgtcgataagcctgtgcttgtttggagggatcatcataaatgattaaagtgtgtc**

**Fortunella_japonica_9** **acatttgtcgataagcctgtgcttgtttggagggatcatcataaatgattaaagtgtgtc**

**Fortunella_japonica_10** **acatttgtcgataagcctgtgcttgtttggagggatcatcataaatgattaaagtgtgtc**

**Fortunella_japonica_11** **acatttgtcgataagcctgtgcttgtttggagggatcatcataaatgattaaagtgtgtc**

**Fortunella_japonica_12** **acatttgtcgataagcctgtgcttgtttggagggatcatcataaatgattaaagtgtgtc**

**Fortunella_margarita_1** **acatttgtcgataagcctgtgcttgtttggagggatcatcataaatgattaaagtgtgtc**

**Fortunella_margarita_2** **acatttgtcgataagcctgtgcttgtttggagggatcatcataaatgattaaagtgtgtc**

**Fortunella_venosa_1** **acatttgtcgataagcctgtgcttgtttggagggatcatcataaatgattaaagtgtgtc**

**Fortunella_venosa_2** **acatttgtcgataagcctgtgcttgtttggagggatcatcataaatgattaaagtgtgtc**

**Fortunella_venosa_3** **acatttgtcgataagcctgtgcttgtttggagggatcatcataaatgattaaagtgtgtc**

12490	12500	12510	12520	12530	12540

....|....|....|....|....|....|....|....|....|....|....|....|

**Atalantia_kwangtungens** **gttcacggtacataaaatattcagctagagctgcgcccgtataaggagcgaggtattgta**

**Citrus_reticulata_1** **gttcacggtacataaaatattcagctagagctgcgcccgtataaggagcgaggtattgta**

**Citrus_reticulata_2** **gttcacggtacataaaatattcagctagagctgcgcccgtataaggagcgaggtattgta**

**Citrus_trifoliata_3** **gttgacggtacataaaatattcagctagagctgcgcccgtataaggagcgaggtattgta**

**Citrus_aurantiifolia** **gttcacggtacataaaatattcagctagagctgcgcccgtataaggagcgaggtattgta**

**Citrus_aurantium** **gttcacggtacataaaatattcagctagagctgcgcccgtataaggagcgaggtattgta**

**Citrus_cavaleriei** **gttcacggtacataaaatattcagctagagctgcgcccgtataaggagcgaggtattgta**

**Citrus_hongheensis** **gttcacggtacataaaatattcagctagagctgcgcccgtataaggagcgaggtattgta**

**Citrus_limon** **gttcacggtacataaaatattcagctagagctgcgcccgtataaggagcgaggtattgta**

**Citrus_maxima** **gttcacggtacataaaatattcagctagagctgcgcccgtataaggagcgaggtattgta**

**Citrus_medica** **gttcacggtacataaaatattcagctagagctgcgcccgtataaggagcgaggtattgta**

**Citrus_polytrifolia** **gttgacggtacataaaatattcagctagagctgcgcccgtataaggagcgaggtattgta**

**Citrus_reticulata** **gttcacggtacataaaatattcagctagagctgcgcccgtataaggagcgaggtattgta**

**Citrus_sinensis** **gttcacggtacataaaatattcagctagagctgcgcccgtataaggagcgaggtattgta**

**Fortunella_bawangica_1** **gttcacggtacataaaatattcagctagagctgcgcccgtataaggagcgaggtattgta**

**Fortunella_bawangica_2** **gttcacggtacataaaatattcagctagagctgcgcccgtataaggagcgaggtattgta**

**Fortunella_hindsii_1** **gttcacggtacataaaatattcagctagagctgcgcccgtataaggagcgaggtattgta**

**Fortunella_hindsii_2** **gttcacggtacataaaatattcagctagagctgcgcccgtataaggagcgaggtattgta**

**Fortunella_hindsii_3** **gttcacggtacataaaatattcagctagagctgcgcccgtataaggagcgaggtattgta**

**Fortunella_japonica_1** **gttcacggtacataaaatattcagctagagctgcgcccgtataaggagcgaggtattgta**

**Fortunella_japonica_2** **gttcacggtacataaaatattcagctagagctgcgcccgtataaggagcgaggtattgta**

**Fortunella_japonica_3** **gttcacggtacataaaatattcagctagagctgcgcccgtataaggagcgaggtattgta**

**Fortunella_japonica_4** **gttcacggtacataaaatattcagctagagctgcgcccgtataaggagcgaggtattgta**

**Fortunella_japonica_5** **gttcacggtacataaaatattcagctagagctgcgcccgtataaggagcgaggtattgta**

**Fortunella_japonica_6** **gttcacggtacataaaatattcagctagagctgcgcccgtataaggagcgaggtattgta**

**Fortunella_japonica_7** **gttcacggtacataaaatattcagctagagctgcgcccgtataaggagcgaggtattgta**

**Fortunella_japonica_8** **gttcacggtacataaaatattcagctagagctgcgcccgtataaggagcgaggtattgta**

**Fortunella_japonica_9** **gttcacggtacataaaatattcagctagagctgcgcccgtataaggagcgaggtattgta**

**Fortunella_japonica_10** **gttcacggtacataaaatattcagctagagctgcgcccgtataaggagcgaggtattgta**

**Fortunella_japonica_11** **gttcacggtacataaaatattcagctagagctgcgcccgtataaggagcgaggtattgta**

**Fortunella_japonica_12** **gttcacggtacataaaatattcagctagagctgcgcccgtataaggagcgaggtattgta**

**Fortunella_margarita_1** **gttcacggtacataaaatattcagctagagctgcgcccgtataaggagcgaggtattgta**

**Fortunella_margarita_2** **gttcacggtacataaaatattcagctagagctgcgcccgtataaggagcgaggtattgta**

**Fortunella_venosa_1** **gttcacggtacataaaatattcagctagagctgcgcccgtataaggagcgaggtattgta**

**Fortunella_venosa_2** **gttcacggtacataaaatattcagctagagctgcgcccgtataaggagcgaggtattgta**

**Fortunella_venosa_3** **gttcacggtacataaaatattcagctagagctgcgcccgtataaggagcgaggtattgta**

12550	12560	12570	12580	12590	12600

....|....|....|....|....|....|....|....|....|....|....|....|

**Atalantia_kwangtungens** **acgtagcgggggaatccgccgtttcggctaccacaatcgtgtattccattgcccccctct**

**Citrus_reticulata_1** **acgtagcgggggaatccgccgtttcggctaccacaatagtgtagtccattgcccccctct**

**Citrus_reticulata_2** **acgtagcgggggaatccgccgtttcggctaccacaatagtgtagtccattgcccccctct**

**Citrus_trifoliata_3** **acgtagcgggggaatccgccgtttcggctaccacaatagtgtagtccattgcccccctct**

**Citrus_aurantiifolia** **acgtagcgggggaatccgccgtttcggctaccacaatagtgtagtccattgcccccctct**

**Citrus_aurantium** **acgtagcgggggaatccgccgtttcggctaccacaatagtgtagtccattgcccccctct**

**Citrus_cavaleriei** **acgtagcgggggaatccgccgtttcggctaccacaatagtgtagtccattgcccccctct**

**Citrus_hongheensis** **acgtagcgggggaatccgccgtttcggctaccacaatagtgtagtccattgcccccctct**

**Citrus_limon** **acgtagcgggggaatccgccgtttcggctaccacaatagtgtagtccattgcccccctct**

**Citrus_maxima** **acgtagcgggggaatccgccgtttcggctaccacaatagtgtagtccattgcccccctct**

**Citrus_medica** **acgtagcgggggaatccgccgtttcggataccacaatagtgtagtccattgcccccctct**

**Citrus_polytrifolia** **acgtagcgggggaatccgccgtttcggctaccacaatagtgtagtccattgcccccctct**

**Citrus_reticulata** **acgtagcgggggaatccgccgtttcggctaccacaatagtgtagtccattgcccccctct**

**Citrus_sinensis** **acgtagcgggggaatccgccgtttcggctaccacaatagtgtagtccattgcccccctct**

**Fortunella_bawangica_1** **acgtagcgggggaatccgccgtttcggctaccacaatagtgtagtccattgcccccctct**

**Fortunella_bawangica_2** **acgtagcgggggaatccgccgtttcggctaccacaatagtgtagtccattgcccccctct**

**Fortunella_hindsii_1** **acgtagcgggggaatccgccgtttcggctaccacaatagtgtagtccattgcccccctct**

**Fortunella_hindsii_2** **acgtagcgggggaatccgccgtttcggctaccacaatagtgtagtccattgcccccctct**

**Fortunella_hindsii_3** **acgtagcgggggaatccgccgtttcggctaccacaatagtgtagtccattgcccccctct**

**Fortunella_japonica_1** **acgtagcgggggaatccgccgtttcggctaccacaatagtgtagtccattgcccccctct**

**Fortunella_japonica_2** **acgtagcgggggaatccgccgtttcggctaccacaatagtgtagtccattgcccccctct**

**Fortunella_japonica_3** **acgtagcgggggaatccgccgtttcggctaccacaatagtgtagtccattgcccccctct**

**Fortunella_japonica_4** **acgtagcgggggaatccgccgtttcggctaccacaatagtgtagtccattgcccccctct**

**Fortunella_japonica_5** **acgtagcgggggaatccgccgtttcggctaccacaatagtgtagtccattgcccccctct**

**Fortunella_japonica_6** **acgtagcgggggaatccgccgtttcggctaccacaatagtgtagtccattgcccccctct**

**Fortunella_japonica_7** **acgtagcgggggaatccgccgtttcggctaccacaatagtgtagtccattgcccccctct**

**Fortunella_japonica_8** **acgtagcgggggaatccgccgtttcggctaccacaatagtgtagtccattgcccccctct**

**Fortunella_japonica_9** **acgtagcgggggaatccgccgtttcggctaccacaatagtgtagtccattgcccccctct**

**Fortunella_japonica_10** **acgtagcgggggaatccgccgtttcggctaccacaatagtgtagtccattgcccccctct**

**Fortunella_japonica_11** **acgtagcgggggaatccgccgtttcggctaccacaatagtgtagtccattgcccccctct**

**Fortunella_japonica_12** **acgtagcgggggaatccgccgtttcggctaccacaatagtgtagtccattgcccccctct**

**Fortunella_margarita_1** **acgtagcgggggaatccgccgtttcggctaccacaatagtgtagtccattgcccccctct**

**Fortunella_margarita_2** **acgtagcgggggaatccgccgtttcggctaccacaatagtgtagtccattgcccccctct**

**Fortunella_venosa_1** **acgtagcgggggaatccgccgtttcggctaccacaatagtgtagtccattgcccccctct**

**Fortunella_venosa_2** **acgtagcgggggaatccgccgtttcggctaccacaatagtgtagtccattgcccccctct**

**Fortunella_venosa_3** **acgtagcgggggaatccgccgtttcggctaccacaatagtgtagtccattgcccccctct**

12610	12620	12630	12640	12650	12660

....|....|....|....|....|....|....|....|....|....|....|....|

**Atalantia_kwangtungens** **cctggaaagtactcactacctgagccacagaagatgctttttgaccaatagctacataaa**

**Citrus_reticulata_1** **cctggaaagtattcactacctgagccacagaagatgctttttgaccaatagctacataaa**

**Citrus_reticulata_2** **cctggaaagtattcactacctgagccacagaagatgctttttgaccaatagctacataaa**

**Citrus_trifoliata_3** **cctggaaagtattcactacctgagccacagaagatgctttttgaccaatagctacataaa**

**Citrus_aurantiifolia** **cctggaaagtattcactacctgagccacagaagatgctttttgaccaatagctacataaa**

**Citrus_aurantium** **cctggaaagtattcactacctgagccacagaagatgctttttgaccaatagctacataaa**

**Citrus_cavaleriei** **cctggaaagtattcactacctgagccacagaagatgctttttgaccaatagctacataaa**

**Citrus_hongheensis** **cctggaaagtattcactacctgagccacagaagatgctttttgaccaatagctacataaa**

**Citrus_limon** **cctggaaagtattcactacctgagccacagaagatgctttttgaccaatagctacataaa**

**Citrus_maxima** **cctggaaagtattcactacctgagccacagaagatgctttttgaccaatagctacataaa**

**Citrus_medica** **cctggaaagtattcactacctgagccacagaagatgctttttgaccaatagctacataaa**

**Citrus_polytrifolia** **cctggaaagtattcactacctgagccacagaagatgctttttgaccaatagctacataaa**

**Citrus_reticulata** **cctggaaagtattcactacctgagccacagaagatgctttttgaccaatagctacataaa**

**Citrus_sinensis** **cctggaaagtattcactacctgagccacagaagatgctttttgaccaatagctacataaa**

**Fortunella_bawangica_1** **cctggaaagtattcactacctgagccacagaagatgctttttgaccaatagctacataaa**

**Fortunella_bawangica_2** **cctggaaagtattcactacctgagccacagaagatgctttttgaccaatagctacataaa**

**Fortunella_hindsii_1** **cctggaaagtattcactacctgagccacagaagatgctttttgaccaatagctacataaa**

**Fortunella_hindsii_2** **cctggaaagtattcactacctgagccacagaagatgctttttgaccaatagctacataaa**

**Fortunella_hindsii_3** **cctggaaagtattcactacctgagccacagaagatgctttttgaccaatagctacataaa**

**Fortunella_japonica_1** **cctggaaagtattcactacctgagccacagaagatgctttttgaccaatagctacataaa**

**Fortunella_japonica_2** **cctggaaagtattcactacctgagccacagaagatgctttttgaccaatagctacataaa**

**Fortunella_japonica_3** **cctggaaagtattcactacctgagccacagaagatgctttttgaccaatagctacataaa**

**Fortunella_japonica_4** **cctggaaagtattcactacctgagccacagaagatgctttttgaccaatagctacataaa**

**Fortunella_japonica_5** **cctggaaagtattcactacctgagccacagaagatgctttttgaccaatagctacataaa**

**Fortunella_japonica_6** **cctggaaagtattcactacctgagccacagaagatgctttttgaccaatagctacataaa**

**Fortunella_japonica_7** **cctggaaagtattcactacctgagccacagaagatgctttttgaccaatagctacataaa**

**Fortunella_japonica_8** **cctggaaagtattcactacctgagccacagaagatgctttttgaccaatagctacataaa**

**Fortunella_japonica_9** **cctggaaagtattcactacctgagccacagaagatgctttttgaccaatagctacataaa**

**Fortunella_japonica_10** **cctggaaagtattcactacctgagccacagaagatgctttttgaccaatagctacataaa**

**Fortunella_japonica_11** **cctggaaagtattcactacctgagccacagaagatgctttttgaccaatagctacataaa**

**Fortunella_japonica_12** **cctggaaagtattcactacctgagccacagaagatgctttttgaccaatagctacataaa**

**Fortunella_margarita_1** **cctggaaagtattcactacctgagccacagaagatgctttttgaccaatagctacataaa**

**Fortunella_margarita_2** **cctggaaagtattcactacctgagccacagaagatgctttttgaccaatagctacataaa**

**Fortunella_venosa_1** **cctggaaagtattcactacctgagccacagaagatgctttttgaccaatagctacataaa**

**Fortunella_venosa_2** **cctggaaagtattcactacctgagccacagaagatgctttttgaccaatagctacataaa**

**Fortunella_venosa_3** **cctggaaagtattcactacctgagccacagaagatgctttttgaccaatagctacataaa**

12670	12680	12690	12700	12710	12720

....|....|....|....|....|....|....|....|....|....|....|....|

**Atalantia_kwangtungens** **cacatattacattttgcccttgttggttgagaatcgtatccgtggctactgccgttttac**

**Citrus_reticulata_1** **cacatattacattttgcccttgttggttgagaatcgtatccgtggctactgccgttttac**

**Citrus_reticulata_2** **cacatattacattttgcccttgttggttgagaatcgtatccgtggctactgccgttttac**

**Citrus_trifoliata_3** **cacatattacattttgcccttgttggttgagaatcgtatccgtggctactgccgttttac**

**Citrus_aurantiifolia** **cacatattacattttgcccttgttggttgagaatcgtatccgtggctactgccgttttac**

**Citrus_aurantium** **cacatattacattttgcccttgttggttgagaatcgtatccgtggctactgccgttttac**

**Citrus_cavaleriei** **cacatattacattttgcccttgttggttgagaatcgtatccgtggctactgccgttttac**

**Citrus_hongheensis** **cacatattacattttgcccttgttggttgagaatcgtatccgtggctactgccgttttac**

**Citrus_limon** **cacatattacattttgcccttgttggttgagaatcgtatccgtggctactgccgttttac**

**Citrus_maxima** **cacatattacattttgcccttgttggttgagaatcgtatccgtggctactgccgttttac**

**Citrus_medica** **cacatattacattttgcccttgttggttgagaatcgtatccgtggctactgccgttttac**

**Citrus_polytrifolia** **cacatattacattttgcccttgttggttgagaatcgtatccgtggctactgccgttttac**

**Citrus_reticulata** **cacatattacattttgcccttgttggttgagaatcgtatccgtggctactgccgttttac**

**Citrus_sinensis** **cacatattacattttgcccttgttggttgagaatcgtatccgtggctactgccgttttac**

**Fortunella_bawangica_1** **cacatattacattttgcccttgttggttgagaatcgtatccgtggctactgccgttttac**

**Fortunella_bawangica_2** **cacatattacattttgcccttgttggttgagaatcgtatccgtggctactgccgttttac**

**Fortunella_hindsii_1** **cacatattacattttgcccttgttggttgagaatcgtatccgtggctactgccgttttac**

**Fortunella_hindsii_2** **cacatattacattttgcccttgttggttgagaatcgtatccgtggctactgccgttttac**

**Fortunella_hindsii_3** **cacatattacattttgcccttgttggttgagaatcgtatccgtggctactgccgttttac**

**Fortunella_japonica_1** **cacatattacattttgcccttgttggttgagaatcgtatccgtggctactgccgttttac**

**Fortunella_japonica_2** **cacatattacattttgcccttgttggttgagaatcgtatccgtggctactgccgttttac**

**Fortunella_japonica_3** **cacatattacattttgcccttgttggttgagaatcgtatccgtggctactgccgttttac**

**Fortunella_japonica_4** **cacatattacattttgcccttgttggttgagaatcgtatccgtggctactgccgttttac**

**Fortunella_japonica_5** **cacatattacattttgcccttgttggttgagaatcgtatccgtggctactgccgttttac**

**Fortunella_japonica_6** **cacatattacattttgcccttgttggttgagaatcgtatccgtggctactgccgttttac**

**Fortunella_japonica_7** **cacatattacattttgcccttgttggttgagaatcgtatccgtggctactgccgttttac**

**Fortunella_japonica_8** **cacatattacattttgcccttgttggttgagaatcgtatccgtggctactgccgttttac**

**Fortunella_japonica_9** **cacatattacattttgcccttgttggttgagaatcgtatccgtggctactgccgttttac**

**Fortunella_japonica_10** **cacatattacattttgcccttgttggttgagaatcgtatccgtggctactgccgttttac**

**Fortunella_japonica_11** **cacatattacattttgcccttgttggttgagaatcgtatccgtggctactgccgttttac**

**Fortunella_japonica_12** **cacatattacattttgcccttgttggttgagaatcgtatccgtggctactgccgttttac**

**Fortunella_margarita_1** **cacatattacattttgcccttgttggttgagaatcgtatccgtggctactgccgttttac**

**Fortunella_margarita_2** **cacatattacattttgcccttgttggttgagaatcgtatccgtggctactgccgttttac**

**Fortunella_venosa_1** **cacatattacattttgcccttgttggttgagaatcgtatccgtggctactgccgttttac**

**Fortunella_venosa_2** **cacatattacattttgcccttgttggttgagaatcgtatccgtggctactgccgttttac**

**Fortunella_venosa_3** **cacatattacattttgcccttgttggttgagaatcgtatccgtggctactgccgttttac**

12730	12740	12750	12760	12770	12780

....|....|....|....|....|....|....|....|....|....|....|....|

**Atalantia_kwangtungens** **cggtctgtctgtccccaataattaattctcgctgaccgcgtcctatagggatcatcgaat**

**Citrus_reticulata_1** **cggtctgtctgtccccaataattaattctcgctgaccgcgtccgatagggatcatcgaat**

**Citrus_reticulata_2** **cggtctgtctgtccccaataattaattctcgctgaccgcgtccgatagggatcatcgaat**

**Citrus_trifoliata_3** **cggtctgtctgtccccaataattaattctcgctgaccgcgtccgatagggatcatcgaat**

**Citrus_aurantiifolia** **cggtctgtctgtccccaataattaattctcgctgaccgcgtccgatagggatcatcgaat**

**Citrus_aurantium** **cggtctgtctgtccccaataattaattctcgctgaccgcgtccgatagggatcatcgaat**

**Citrus_cavaleriei** **cggtctgtctgtccccaataattaattctcgctgaccgcgtccgatagggatcatcgaat**

**Citrus_hongheensis** **cggtctgtctgtccccaataattaattctcgctgaccgcgtccgatagggatcatcgaat**

**Citrus_limon** **cggtctgtctgtccccaataattaattctcgctgaccgcgtccgatagggatcatcgaat**

**Citrus_maxima** **cggtctgtctgtccccaataattaattctcgctgaccgcgtccgatagggatcatcgaat**

**Citrus_medica** **cggtctgtctgtccccaataattaattctcgctgaccgcgtcctatagggatcatcgaat**

**Citrus_polytrifolia** **cggtctgtctgtccccaataattaattctcgctgaccgcgtccgatagggatcatcgaat**

**Citrus_reticulata** **cggtctgtctgtccccaataattaattctcgctgaccgcgtccgatagggatcatcgaat**

**Citrus_sinensis** **cggtctgtctgtccccaataattaattctcgctgaccgcgtccgatagggatcatcgaat**

**Fortunella_bawangica_1** **cggtctgtctgtccccaataattaattctcgctgaccgcgtccgatagggatcatcgaat**

**Fortunella_bawangica_2** **cggtctgtctgtccccaataattaattctcgctgaccgcgtccgatagggatcatcgaat**

**Fortunella_hindsii_1** **cggtctgtctgtccccaataattaattctcgctgaccgcgtccgatagggatcatcgaat**

**Fortunella_hindsii_2** **cggtctgtctgtccccaataattaattctcgctgaccgcgtccgatagggatcatcgaat**

**Fortunella_hindsii_3** **cggtctgtctgtccccaataattaattctcgctgaccgcgtccgatagggatcatcgaat**

**Fortunella_japonica_1** **cggtctgtctgtccccaataattaattctcgctgaccgcgtccgatagggatcatcgaat**

**Fortunella_japonica_2** **cggtctgtctgtccccaataattaattctcgctgaccgcgtccgatagggatcatcgaat**

**Fortunella_japonica_3** **cggtctgtctgtccccaataattaattctcgctgaccgcgtccgatagggatcatcgaat**

**Fortunella_japonica_4** **cggtctgtctgtccccaataattaattctcgctgaccgcgtccgatagggatcatcgaat**

**Fortunella_japonica_5** **cggtctgtctgtccccaataattaattctcgctgaccgcgtccgatagggatcatcgaat**

**Fortunella_japonica_6** **cggtctgtctgtccccaataattaattctcgctgaccgcgtccgatagggatcatcgaat**

**Fortunella_japonica_7** **cggtctgtctgtccccaataattaattctcgctgaccgcgtccgatagggatcatcgaat**

**Fortunella_japonica_8** **cggtctgtctgtccccaataattaattctcgctgaccgcgtccgatagggatcatcgaat**

**Fortunella_japonica_9** **cggtctgtctgtccccaataattaattctcgctgaccgcgtccgatagggatcatcgaat**

**Fortunella_japonica_10** **cggtctgtctgtccccaataattaattctcgctgaccgcgtccgatagggatcatcgaat**

**Fortunella_japonica_11** **cggtctgtctgtccccaataattaattctcgctgaccgcgtccgatagggatcatcgaat**

**Fortunella_japonica_12** **cggtctgtctgtccccaataattaattctcgctgaccgcgtccgatagggatcatcgaat**

**Fortunella_margarita_1** **cggtctgtctgtccccaataattaattctcgctgaccgcgtccgatagggatcatcgaat**

**Fortunella_margarita_2** **cggtctgtctgtccccaataattaattctcgctgaccgcgtccgatagggatcatcgaat**

**Fortunella_venosa_1** **cggtctgtctgtccccaataattaattctcgctgaccgcgtccgatagggatcatcgaat**

**Fortunella_venosa_2** **cggtctgtctgtccccaataattaattctcgctgaccgcgtccgatagggatcatcgaat**

**Fortunella_venosa_3** **cggtctgtctgtccccaataattaattctcgctgaccgcgtccgatagggatcatcgaat**

12790	12800	12810	12820	12830	12840

....|....|....|....|....|....|....|....|....|....|....|....|

**Atalantia_kwangtungens** **caatagcaataagtcctgtttgaagaggctcatatacggaacgtctcgaaataatacccg**

**Citrus_reticulata_1** **caatagcaataagtcctgtttgaagaggctcatatacggaacgtctcgaaataatacccg**

**Citrus_reticulata_2** **caatagcaataagtcctgtttgaagaggctcatatacggaacgtctcgaaataatacccg**

**Citrus_trifoliata_3** **caatagcaataagtcctgtttgaagaggctcatatacggaacgtctcgaaataatccccg**

**Citrus_aurantiifolia** **caatagcaataagtcctgtttgaagaggctcatatacggaacgtctcgaaataatacccg**

**Citrus_aurantium** **caatagcaataagtcctgtttgaagaggctcatatacggaacgtctcgaaataatacccg**

**Citrus_cavaleriei** **caatagcaataagtcctgtttgaagaggctcatatacggaacgtctcgaaataatacccg**

**Citrus_hongheensis** **caatagcaataagtcctgtttgaagaggctcatatacggaacgtctcgaaataatacccg**

**Citrus_limon** **caatagcaataagtcctgtttgaagaggctcatatacggaacgtctcgaaataatacccg**

**Citrus_maxima** **caatagcaataagtcctgtttgaagaggctcatatacggaacgtctcgaaataatacccg**

**Citrus_medica** **caatagcaataagtcctgtttgaagaggctcatatacggaacgtctcgaaataatacccg**

**Citrus_polytrifolia** **caatagcaataagtcctgtttgaagaggctcatatacggaacgtctcgaaataatccccg**

**Citrus_reticulata** **caatagcaataagtcctgtttgaagaggctcatatacggaacgtctcgaaataatacccg**

**Citrus_sinensis** **caatagcaataagtcctgtttgaagaggctcatatacggaacgtctcgaaataatacccg**

**Fortunella_bawangica_1** **caatagcaataagtcctgtttgaagaggctcatatacggaacgtctcgaaataatacccg**

**Fortunella_bawangica_2** **caatagcaataagtcctgtttgaagaggctcatatacggaacgtctcgaaataatacccg**

**Fortunella_hindsii_1** **caatagcaataagtcctgtttgaagaggctcatatacggaacgtctcgaaataatacccg**

**Fortunella_hindsii_2** **caatagcaataagtcctgtttgaagaggctcatatacggaacgtctcgaaataatacccg**

**Fortunella_hindsii_3** **caatagcaataagtcctgtttgaagaggctcatatacggaacgtctcgaaataatacccg**

**Fortunella_japonica_1** **caatagcaataagtcctgtttgaagaggctcatatacggaacgtctcgaaataatacccg**

**Fortunella_japonica_2** **caatagcaataagtcctgtttgaagaggctcatatacggaacgtctcgaaataatacccg**

**Fortunella_japonica_3** **caatagcaataagtcctgtttgaagaggctcatatacggaacgtctcgaaataatacccg**

**Fortunella_japonica_4** **caatagcaataagtcctgtttgaagaggctcatatacggaacgtctcgaaataatacccg**

**Fortunella_japonica_5** **caatagcaataagtcctgtttgaagaggctcatatacggaacgtctcgaaataatacccg**

**Fortunella_japonica_6** **caatagcaataagtcctgtttgaagaggctcatatacggaacgtctcgaaataatacccg**

**Fortunella_japonica_7** **caatagcaataagtcctgtttgaagaggctcatatacggaacgtctcgaaataatacccg**

**Fortunella_japonica_8** **caatagcaataagtcctgtttgaagaggctcatatacggaacgtctcgaaataatacccg**

**Fortunella_japonica_9** **caatagcaataagtcctgtttgaagaggctcatatacggaacgtctcgaaataatacccg**

**Fortunella_japonica_10** **caatagcaataagtcctgtttgaagaggctcatatacggaacgtctcgaaataatacccg**

**Fortunella_japonica_11** **caatagcaataagtcctgtttgaagaggctcatatacggaacgtctcgaaataatacccg**

**Fortunella_japonica_12** **caatagcaataagtcctgtttgaagaggctcatatacggaacgtctcgaaataatacccg**

**Fortunella_margarita_1** **caatagcaataagtcctgtttgaagaggctcatatacggaacgtctcgaaataatacccg**

**Fortunella_margarita_2** **caatagcaataagtcctgtttgaagaggctcatatacggaacgtctcgaaataatacccg**

**Fortunella_venosa_1** **caatagcaataagtcctgtttgaagaggctcatatacggaacgtctcgaaataatacccg**

**Fortunella_venosa_2** **caatagcaataagtcctgtttgaagaggctcatatacggaacgtctcgaaataatacccg**

**Fortunella_venosa_3** **caatagcaataagtcctgtttgaagaggctcatatacggaacgtctcgaaataatacccg**

12850	12860	12870	12880	12890	12900

....|....|....|....|....|....|....|....|....|....|....|....|

**Atalantia_kwangtungens** **gagcaggggattcaattaatctagattcagaagttgaaatttcacctcgaccatcaatag**

**Citrus_reticulata_1** **gagcaggggattcaattaatcgagattcagaagctgaaatttcacctcgaccatcaatag**

**Citrus_reticulata_2** **gagcaggggattcaattaatcgagattcagaagctgaaatttcacctcgaccatcaatag**

**Citrus_trifoliata_3** **gagcaggggattcaattaatcgagattcagaagctggaatttcacctcgaccatcaatag**

**Citrus_aurantiifolia** **gagcaggggattcaattaatcgagattcagaagctgaaatttcacctcgaccatcaatag**

**Citrus_aurantium** **gagcaggggattcaattaatcgagattcagaagctgaaatttcacctcgaccatcaatag**

**Citrus_cavaleriei** **gagcaggggattcaattaatcgagattcagaagctgaaatttcacctcgaccatcaatag**

**Citrus_hongheensis** **gagcaggggattcaattaatcgagattcagaagctgaaatttcacctcgaccatcaatag**

**Citrus_limon** **gagcaggggattcaattaatcgagattcagaagctgaaatttcacctcgaccatcaatag**

**Citrus_maxima** **gagcaggggattcaattaatcgagattcagaagctgaaatttcacctcgaccatcaatag**

**Citrus_medica** **gagcaggggattcaattaatcgagattcagaagctgaaatttcacctcgaccatcaatag**

**Citrus_polytrifolia** **gagcaggggattcaattaatcgagattcagaagctggaatttcacctcgaccatcaatag**

**Citrus_reticulata** **gagcaggggattcaattaatcgagattcagaagctgaaatttcacctcgaccatcaatag**

**Citrus_sinensis** **gagcaggggattcaattaatcgagattcagaagctgaaatttcacctcgaccatcaatag**

**Fortunella_bawangica_1** **gagcaggggattcaattaatcgagattcagaagctgaaatttcacctcgaccatcaatag**

**Fortunella_bawangica_2** **gagcaggggattcaattaatcgagattcagaagctgaaatttcacctcgaccatcaatag**

**Fortunella_hindsii_1** **gagcaggggattcaattaatcgagattcagaagctgaaatttcacctcgaccatcaatag**

**Fortunella_hindsii_2** **gagcaggggattcaattaatcgagattcagaagctgaaatttcacctcgaccatcaatag**

**Fortunella_hindsii_3** **gagcaggggattcaattaatcgagattcagaagctgaaatttcacctcgaccatcaatag**

**Fortunella_japonica_1** **gagcaggggattcaattaatcgagattcagaagctgaaatttcacctcgaccatcaatag**

**Fortunella_japonica_2** **gagcaggggattcaattaatcgagattcagaagctgaaatttcacctcgaccatcaatag**

**Fortunella_japonica_3** **gagcaggggattcaattaatcgagattcagaagctgaaatttcacctcgaccatcaatag**

**Fortunella_japonica_4** **gagcaggggattcaattaatcgagattcagaagctgaaatttcacctcgaccatcaatag**

**Fortunella_japonica_5** **gagcaggggattcaattaatcgagattcagaagctgaaatttcacctcgaccatcaatag**

**Fortunella_japonica_6** **gagcaggggattcaattaatcgagattcagaagctgaaatttcacctcgaccatcaatag**

**Fortunella_japonica_7** **gagcaggggattcaattaatcgagattcagaagctgaaatttcacctcgaccatcaatag**

**Fortunella_japonica_8** **gagcaggggattcaattaatcgagattcagaagctgaaatttcacctcgaccatcaatag**

**Fortunella_japonica_9** **gagcaggggattcaattaatcgagattcagaagctgaaatttcacctcgaccatcaatag**

**Fortunella_japonica_10** **gagcaggggattcaattaatcgagattcagaagctgaaatttcacctcgaccatcaatag**

**Fortunella_japonica_11** **gagcaggggattcaattaatcgagattcagaagctgaaatttcacctcgaccatcaatag**

**Fortunella_japonica_12** **gagcaggggattcaattaatcgagattcagaagctgaaatttcacctcgaccatcaatag**

**Fortunella_margarita_1** **gagcaggggattcaattaatcgagattcagaagctgaaatttcacctcgaccatcaatag**

**Fortunella_margarita_2** **gagcaggggattcaattaatcgagattcagaagctgaaatttcacctcgaccatcaatag**

**Fortunella_venosa_1** **gagcaggggattcaattaatcgagattcagaagctgaaatttcacctcgaccatcaatag**

**Fortunella_venosa_2** **gagcaggggattcaattaatcgagattcagaagctgaaatttcacctcgaccatcaatag**

**Fortunella_venosa_3** **gagcaggggattcaattaatcgagattcagaagctgaaatttcacctcgaccatcaatag**

12910	12920	12930	12940	12950	12960

....|....|....|....|....|....|....|....|....|....|....|....|

**Atalantia_kwangtungens** **gtttagccagggcatttataacacgacctaaataagcctcgcttactggtatctgagcaa**

**Citrus_reticulata_1** **gtttagccagggcatttataacacgacctaaataagcctcgcttactggtatctgagcaa**

**Citrus_reticulata_2** **gtttagccagggcatttataacacgacctaaataagcctcgcttactggtatctgagcaa**

**Citrus_trifoliata_3** **gtttagccagggcatttataacacgacctaaataagcctcgcttactggtatctgagcaa**

**Citrus_aurantiifolia** **gtttagccagggcatttataacacgacctaaataagcctcgcttactggtatctgagcaa**

**Citrus_aurantium** **gtttagccagggcatttataacacgacctaaataagcctcgcttactggtatctgagcaa**

**Citrus_cavaleriei** **gtttagccagggcatttataacacgacctaaataagcctcgcttactggtatctgagcaa**

**Citrus_hongheensis** **gtttagccagggcatttataacacgacctaaataagcctcgcttactggtatctgagcaa**

**Citrus_limon** **gtttagccagggcatttataacacgacctaaataagcctcgcttactggtatctgagcaa**

**Citrus_maxima** **gtttagccagggcatttataacacgacctaaataagcctcgcttactggtatctgagcaa**

**Citrus_medica** **gtttagccagggcatttataacacgacctaaataagcctcgcttactggtatctgagcaa**

**Citrus_polytrifolia** **gtttagccagggcatttataacacgacctaaataagcctcgcttactggtatctgagcaa**

**Citrus_reticulata** **gtttagccagggcatttataacacgacctaaataagcctcgcttactggtatctgagcaa**

**Citrus_sinensis** **gtttagccagggcatttataacacgacctaaataagcctcgcttactggtatctgagcaa**

**Fortunella_bawangica_1** **gtttagccagggcatttataacacgacctaaataagcctcgcttactggtatctgagcaa**

**Fortunella_bawangica_2** **gtttagccagggcatttataacacgacctaaataagcctcgcttactggtatctgagcaa**

**Fortunella_hindsii_1** **gtttagccagggcatttataacacgacctaaataagcctcgcttactggtatctgagcaa**

**Fortunella_hindsii_2** **gtttagccagggcatttataacacgacctaaataagcctcgcttactggtatctgagcaa**

**Fortunella_hindsii_3** **gtttagccagggcatttataacacgacctaaataagcctcgcttactggtatctgagcaa**

**Fortunella_japonica_1** **gtttagccagggcatttataacacgacctaaataagcctcgcttactggtatctgagcaa**

**Fortunella_japonica_2** **gtttagccagggcatttataacacgacctaaataagcctcgcttactggtatctgagcaa**

**Fortunella_japonica_3** **gtttagccagggcatttataacacgacctaaataagcctcgcttactggtatctgagcaa**

**Fortunella_japonica_4** **gtttagccagggcatttataacacgacctaaataagcctcgcttactggtatctgagcaa**

**Fortunella_japonica_5** **gtttagccagggcatttataacacgacctaaataagcctcgcttactggtatctgagcaa**

**Fortunella_japonica_6** **gtttagccagggcatttataacacgacctaaataagcctcgcttactggtatctgagcaa**

**Fortunella_japonica_7** **gtttagccagggcatttataacacgacctaaataagcctcgcttactggtatctgagcaa**

**Fortunella_japonica_8** **gtttagccagggcatttataacacgacctaaataagcctcgcttactggtatctgagcaa**

**Fortunella_japonica_9** **gtttagccagggcatttataacacgacctaaataagcctcgcttactggtatctgagcaa**

**Fortunella_japonica_10** **gtttagccagggcatttataacacgacctaaataagcctcgcttactggtatctgagcaa**

**Fortunella_japonica_11** **gtttagccagggcatttataacacgacctaaataagcctcgcttactggtatctgagcaa**

**Fortunella_japonica_12** **gtttagccagggcatttataacacgacctaaataagcctcgcttactggtatctgagcaa**

**Fortunella_margarita_1** **gtttagccagggcatttataacacgacctaaataagcctcgcttactggtatctgagcaa**

**Fortunella_margarita_2** **gtttagccagggcatttataacacgacctaaataagcctcgcttactggtatctgagcaa**

**Fortunella_venosa_1** **gtttagccagggcatttataacacgacctaaataagcctcgcttactggtatctgagcaa**

**Fortunella_venosa_2** **gtttagccagggcatttataacacgacctaaataagcctcgcttactggtatctgagcaa**

**Fortunella_venosa_3** **gtttagccagggcatttataacacgacctaaataagcctcgcttactggtatctgagcaa**

12970	12980	12990	13000	13010	13020

....|....|....|....|....|....|....|....|....|....|....|....|

**Atalantia_kwangtungens** **tttttccggttgcttttacagaacttccttcttgtatcattaaaccgtcacccattaaaa**

**Citrus_reticulata_1** **tttttccggttgcttttacagaacttccttcttgtatcattaaaccgtcacccattaaaa**

**Citrus_reticulata_2** **tttttccggttgcttttacagaacttccttcttgtatcattaaaccgtcacccattaaaa**

**Citrus_trifoliata_3** **tttttccggttgcttttacagaacttccttcttgtatcattaaaccgtcacccattaaaa**

**Citrus_aurantiifolia** **tttttccggttgcttttacagaacttccttcttgtatcattaaaccgtcacccattaaaa**

**Citrus_aurantium** **tttttccggttgcttttacagaacttccttcttgtatcattaaaccgtcacccattaaaa**

**Citrus_cavaleriei** **tttttccagttgcttttacagaacttccttcttgtatcattaaaccgtcacccattaaaa**

**Citrus_hongheensis** **tttttccggttgcttttacagaacttccttcttgtatcattaaaccgtcacccattaaaa**

**Citrus_limon** **tttttccggttgcttttacagaacttccttcttgtatcattaaaccgtcacccattaaaa**

**Citrus_maxima** **tttttccggttgcttttacagaacttccttcttgtatcattaaaccgtcacccattaaaa**

**Citrus_medica** **tttttccggttgcttttacagaacttccttcttgtatcattaaaccgtcacccattaaaa**

**Citrus_polytrifolia** **tttttccggttgcttttacagaacttccttcttgtatcattaaaccgtcacccattaaaa**

**Citrus_reticulata** **tttttccggttgcttttacagaacttccttcttgtatcattaaaccgtcacccattaaaa**

**Citrus_sinensis** **tttttccggttgcttttacagaacttccttcttgtatcattaaaccgtcacccattaaaa**

**Fortunella_bawangica_1** **tttttccggttgcttttacagaacttccttcttgtatcattaaaccgtcacccattaaaa**

**Fortunella_bawangica_2** **tttttccggttgcttttacagaacttccttcttgtatcattaaaccgtcacccattaaaa**

**Fortunella_hindsii_1** **tttttccggttgcttttacagaacttccttcttgtatcattaaaccgtcacccattaaaa**

**Fortunella_hindsii_2** **tttttccggttgcttttacagaacttccttcttgtatcattaaaccgtcacccattaaaa**

**Fortunella_hindsii_3** **tttttccggttgcttttacagaacttccttcttgtatcattaaaccgtcacccattaaaa**

**Fortunella_japonica_1** **tttttccggttgcttttacagaacttccttcttgtatcattaaaccgtcacccattaaaa**

**Fortunella_japonica_2** **tttttccggttgcttttacagaacttccttcttgtatcattaaaccgtcacccattaaaa**

**Fortunella_japonica_3** **tttttccggttgcttttacagaacttccttcttgtatcattaaaccgtcacccattaaaa**

**Fortunella_japonica_4** **tttttccggttgcttttacagaacttccttcttgtatcattaaaccgtcacccattaaaa**

**Fortunella_japonica_5** **tttttccggttgcttttacagaacttccttcttgtatcattaaaccgtcacccattaaaa**

**Fortunella_japonica_6** **tttttccggttgcttttacagaacttccttcttgtatcattaaaccgtcacccattaaaa**

**Fortunella_japonica_7** **tttttccggttgcttttacagaacttccttcttgtatcattaaaccgtcacccattaaaa**

**Fortunella_japonica_8** **tttttccggttgcttttacagaacttccttcttgtatcattaaaccgtcacccattaaaa**

**Fortunella_japonica_9** **tttttccggttgcttttacagaacttccttcttgtatcattaaaccgtcacccattaaaa**

**Fortunella_japonica_10** **tttttccggttgcttttacagaacttccttcttgtatcattaaaccgtcacccattaaaa**

**Fortunella_japonica_11** **tttttccggttgcttttacagaacttccttcttgtatcattaaaccgtcacccattaaaa**

**Fortunella_japonica_12** **tttttccggttgcttttacagaacttccttcttgtatcattaaaccgtcacccattaaaa**

**Fortunella_margarita_1** **tttttccggttgcttttacagaacttccttcttgtatcattaaaccgtcacccattaaaa**

**Fortunella_margarita_2** **tttttccggttgcttttacagaacttccttcttgtatcattaaaccgtcacccattaaaa**

**Fortunella_venosa_1** **tttttccggttgcttttacagaacttccttcttgtatcattaaaccgtcacccattaaaa**

**Fortunella_venosa_2** **tttttccggttgcttttacagaacttccttcttgtatcattaaaccgtcacccattaaaa**

**Fortunella_venosa_3** **tttttccggttgcttttacagaacttccttcttgtatcattaaaccgtcacccattaaaa**

13030	13040	13050	13060	13070	13080

....|....|....|....|....|....|....|....|....|....|....|....|

**Atalantia_kwangtungens** **caacaccaacattatttgattccaaattcagagcaatgcctattgtaccctcttcaaatt**

**Citrus_reticulata_1** **caacaccaacattagttgattccaaattcagagcaatgcctattgtaccctcttcaaatt**

**Citrus_reticulata_2** **caacaccaacattagttgattccaaattcagagcaatgcctattgtaccctcttcaaatt**

**Citrus_trifoliata_3** **caacaccaacattagttgattccaaattcagagcaatgcctattgtaccctcttcaaatt**

**Citrus_aurantiifolia** **caacaccaacattagttgattccaaattcagagcaatgcctattgtaccctcttcaaatt**

**Citrus_aurantium** **caacaccaacattagttgattccaaattcagagcaatgcctattgtaccctcttcaaatt**

**Citrus_cavaleriei** **caacaccaacattagttgattccaaattcagagcaatgcctattgtaccctcttcaaatt**

**Citrus_hongheensis** **caacaccaacattagttgattccaaattcagagcaatgcctattgtaccctcttcaaatt**

**Citrus_limon** **caacaccaacattagttgattccaaattcagagcaatgcctattgtaccctcttcaaatt**

**Citrus_maxima** **caacaccaacattagttgattccaaattcagagcaatgcctattgtaccctcttcaaatt**

**Citrus_medica** **caacaccaacattagttgattccaaattcagagcaatgcctattgtaccctcttcaaatt**

**Citrus_polytrifolia** **caacaccaacattagttgattccaaattcagagcaatgcctattgtaccctcttcaaatt**

**Citrus_reticulata** **caacaccaacattagttgattccaaattcagagcaatgcctattgtaccctcttcaaatt**

**Citrus_sinensis** **caacaccaacattagttgattccaaattcagagcaatgcctattgtaccctcttcaaatt**

**Fortunella_bawangica_1** **caacaccaacattagttgattccaaattcagagcaatgcctattgtaccctcttcaaatt**

**Fortunella_bawangica_2** **caacaccaacattagttgattccaaattcagagcaatgcctattgtaccctcttcaaatt**

**Fortunella_hindsii_1** **caacaccaacattagttgattccaaattcagagcaatgcctattgtaccctcttcaaatt**

**Fortunella_hindsii_2** **caacaccaacattagttgattccaaattcagagcaatgcctattgtaccctcttcaaatt**

**Fortunella_hindsii_3** **caacaccaacattagttgattccaaattcagagcaatgcctattgtaccctcttcaaatt**

**Fortunella_japonica_1** **caacaccaacattagttgattccaaattcagagcaatgcctattgtaccctcttcaaatt**

**Fortunella_japonica_2** **caacaccaacattagttgattccaaattcagagcaatgcctattgtaccctcttcaaatt**

**Fortunella_japonica_3** **caacaccaacattagttgattccaaattcagagcaatgcctattgtaccctcttcaaatt**

**Fortunella_japonica_4** **caacaccaacattagttgattccaaattcagagcaatgcctattgtaccctcttcaaatt**

**Fortunella_japonica_5** **caacaccaacattagttgattccaaattcagagcaatgcctattgtaccctcttcaaatt**

**Fortunella_japonica_6** **caacaccaacattagttgattccaaattcagagcaatgcctattgtaccctcttcaaatt**

**Fortunella_japonica_7** **caacaccaacattagttgattccaaattcagagcaatgcctattgtaccctcttcaaatt**

**Fortunella_japonica_8** **caacaccaacattagttgattccaaattcagagcaatgcctattgtaccctcttcaaatt**

**Fortunella_japonica_9** **caacaccaacattagttgattccaaattcagagcaatgcctattgtaccctcttcaaatt**

**Fortunella_japonica_10** **caacaccaacattagttgattccaaattcagagcaatgcctattgtaccctcttcaaatt**

**Fortunella_japonica_11** **caacaccaacattagttgattccaaattcagagcaatgcctattgtaccctcttcaaatt**

**Fortunella_japonica_12** **caacaccaacattagttgattccaaattcagagcaatgcctattgtaccctcttcaaatt**

**Fortunella_margarita_1** **caacaccaacattagttgattccaaattcagagcaatgcctattgtaccctcttcaaatt**

**Fortunella_margarita_2** **caacaccaacattagttgattccaaattcagagcaatgcctattgtaccctcttcaaatt**

**Fortunella_venosa_1** **caacaccaacattagttgattccaaattcagagcaatgcctattgtaccctcttcaaatt**

**Fortunella_venosa_2** **caacaccaacattagttgattccaaattcagagcaatgcctattgtaccctcttcaaatt**

**Fortunella_venosa_3** **caacaccaacattagttgattccaaattcagagcaatgcctattgtaccctcttcaaatt**

13090	13100	13110	13120	13130	13140

....|....|....|....|....|....|....|....|....|....|....|....|

**Atalantia_kwangtungens** **ctactaattcacctgccattacttcatcaagaccataaatacgggcgatgccgtcgccta**

**Citrus_reticulata_1** **ctactaattcacctgccattacttcatcaagaccataaatacgggcgatgccgtcgccta**

**Citrus_reticulata_2** **ctactaattcacctgccattacttcatcaagaccataaatacgggcgatgccgtcgccta**

**Citrus_trifoliata_3** **ctactaattcacctgccattacttcatcaagaccataaatacgggcgatgccgtcgccta**

**Citrus_aurantiifolia** **ctactaattcacttgccattacttcatcaagaccataaatacgggcgatgccgtcgccta**

**Citrus_aurantium** **ctactaattcacctgccattacttcatcaagaccataaatacgggcgatgccgtcgccta**

**Citrus_cavaleriei** **ctactaattcacctgccattacttcatcaagaccataaatacgggcgataccgtcgccta**

**Citrus_hongheensis** **ctactaattcacctgccattacttcatcaagaccataaatacgggcgatgccgtcgccta**

**Citrus_limon** **ctactaattcacctgccattacttcatcaagaccataaatacgggcgatgccgtcgccta**

**Citrus_maxima** **ctactaattcacctgccattacttcatcaagaccataaatacgggcgatgccgtcgccta**

**Citrus_medica** **ctactaattcacctgccattacttcatcaagaccataaatacgggcgatgccgtcgccta**

**Citrus_polytrifolia** **ctactaattcacctgccattacttcatcaagaccataaatacgggcgatgccgtcgccta**

**Citrus_reticulata** **ctactaattcacctgccattacttcatcaagaccataaatacgggcgatgccgtcgccta**

**Citrus_sinensis** **ctactaattcacctgccattacttcatcaagaccataaatacgggcgatgccgtcgccta**

**Fortunella_bawangica_1** **ctactaattcacctgccattacttcatcaagaccataaatacgggcgatgccgtcgccta**

**Fortunella_bawangica_2** **ctactaattcacctgccattacttcatcaagaccataaatacgggcgatgccgtcgccta**

**Fortunella_hindsii_1** **ctactaattcacctgccattacttcatcaagaccataaatacgggcgatgccgtcgccta**

**Fortunella_hindsii_2** **ctactaattcacctgccattacttcatcaagaccataaatacgggcgatgccgtcgccta**

**Fortunella_hindsii_3** **ctactaattcacctgccattacttcatcaagaccataaatacgggcgatgccgtcgccta**

**Fortunella_japonica_1** **ctactaattcacctgccattacttcatcaagaccataaatacgggcgatgccgtcgccta**

**Fortunella_japonica_2** **ctactaattcacctgccattacttcatcaagaccataaatacgggcgatgccgtcgccta**

**Fortunella_japonica_3** **ctactaattcacctgccattacttcatcaagaccataaatacgggcgatgccgtcgccta**

**Fortunella_japonica_4** **ctactaattcacctgccattacttcatcaagaccataaatacgggcgatgccgtcgccta**

**Fortunella_japonica_5** **ctactaattcacctgccattacttcatcaagaccataaatacgggcgatgccgtcgccta**

**Fortunella_japonica_6** **ctactaattcacctgccattacttcatcaagaccataaatacgggcgatgccgtcgccta**

**Fortunella_japonica_7** **ctactaattcacctgccattacttcatcaagaccataaatacgggcgatgccgtcgccta**

**Fortunella_japonica_8** **ctactaattcacctgccattacttcatcaagaccataaatacgggcgatgccgtcgccta**

**Fortunella_japonica_9** **ctactaattcacctgccattacttcatcaagaccataaatacgggcgatgccgtcgccta**

**Fortunella_japonica_10** **ctactaattcacctgccattacttcatcaagaccataaatacgggcgatgccgtcgccta**

**Fortunella_japonica_11** **ctactaattcacctgccattacttcatcaagaccataaatacgggcgatgccgtcgccta**

**Fortunella_japonica_12** **ctactaattcacctgccattacttcatcaagaccataaatacgggcgatgccgtcgccta**

**Fortunella_margarita_1** **ctactaattcacctgccattacttcatcaagaccataaatacgggcgatgccgtcgccta**

**Fortunella_margarita_2** **ctactaattcacctgccattacttcatcaagaccataaatacgggcgatgccgtcgccta**

**Fortunella_venosa_1** **ctactaattcacctgccattacttcatcaagaccataaatacgggcgatgccgtcgccta**

**Fortunella_venosa_2** **ctactaattcacctgccattacttcatcaagaccataaatacgggcgatgccgtcgccta**

**Fortunella_venosa_3** **ctactaattcacctgccattacttcatcaagaccataaatacgggcgatgccgtcgccta**

13150	13160	13170	13180	13190	13200

....|....|....|....|....|....|....|....|....|....|....|....|

**Atalantia_kwangtungens** **cttgaagtacggtcccaatatttacaatctttacttctctattatattgctcaatacgtt**

**Citrus_reticulata_1** **cttgaagtacggtcccaatatttacaatctttacttctctattatattgctcaatacgtt**

**Citrus_reticulata_2** **cttgaagtacggtcccaatatttacaatctttacttctctattatattgctcaatacgtt**

**Citrus_trifoliata_3** **cttgaagtacggtcccaatatttacaatctttacttctctattatattgctcaatacgtt**

**Citrus_aurantiifolia** **cttgaagtacggtcccaatatttacaatctttacttctctattatattgctcaatacgtt**

**Citrus_aurantium** **cttgaagtacggtcccaatatttacaatctttacttctctattatattgctcaatacgtt**

**Citrus_cavaleriei** **cttgaagtacggtcccaatatttacaatctttacttctctattatattgctcaatacgtt**

**Citrus_hongheensis** **cttgaagtacggtcccaatatttacaatctttacttctctattatattgctcaatacgtt**

**Citrus_limon** **cttgaagtacggtcccaatatttacaatctttacttctctattatattgctcaatacgtt**

**Citrus_maxima** **cttgaagtacggtcccaatatttacaatctttacttctctattatattgctcaatacgtt**

**Citrus_medica** **cttgaagtacggtcccaatatttacaatctttacttctctattatattgctcaatacgtt**

**Citrus_polytrifolia** **cttgaagtacggtcccaatatttacaatctttacttctctattatattgctcaatacgtt**

**Citrus_reticulata** **cttgaagtacggtcccaatatttacaatctttacttctctattatattgctcaatacgtt**

**Citrus_sinensis** **cttgaagtacggtcccaatatttacaatctttacttctctattatattgctcaatacgtt**

**Fortunella_bawangica_1** **cttgaagtacggtcccaatatttacaatctttacttctctattatattgctcaatacgtt**

**Fortunella_bawangica_2** **cttgaagtacggtcccaatatttacaatctttacttctctattatattgctcaatacgtt**

**Fortunella_hindsii_1** **cttgaagtacggtcccaatatttacaatctttacttctctattatattgctcaatacgtt**

**Fortunella_hindsii_2** **cttgaagtacggtcccaatatttacaatctttacttctctattatattgctcaatacgtt**

**Fortunella_hindsii_3** **cttgaagtacggtcccaatatttacaatctttacttctctattatattgctcaatacgtt**

**Fortunella_japonica_1** **cttgaagtacggtcccaatatttacaatctttacttctctattatattgctcaatacgtt**

**Fortunella_japonica_2** **cttgaagtacggtcccaatatttacaatctttacttctctattatattgctcaatacgtt**

**Fortunella_japonica_3** **cttgaagtacggtcccaatatttacaatctttacttctctattatattgctcaatacgtt**

**Fortunella_japonica_4** **cttgaagtacggtcccaatatttacaatctttacttctctattatattgctcaatacgtt**

**Fortunella_japonica_5** **cttgaagtacggtcccaatatttacaatctttacttctctattatattgctcaatacgtt**

**Fortunella_japonica_6** **cttgaagtacggtcccaatatttacaatctttacttctctattatattgctcaatacgtt**

**Fortunella_japonica_7** **cttgaagtacggtcccaatatttacaatctttacttctctattatattgctcaatacgtt**

**Fortunella_japonica_8** **cttgaagtacggtcccaatatttacaatctttacttctctattatattgctcaatacgtt**

**Fortunella_japonica_9** **cttgaagtacggtcccaatatttacaatctttacttctctattatattgctcaatacgtt**

**Fortunella_japonica_10** **cttgaagtacggtcccaatatttacaatctttacttctctattatattgctcaatacgtt**

**Fortunella_japonica_11** **cttgaagtacggtcccaatatttacaatctttacttctctattatattgctcaatacgtt**

**Fortunella_japonica_12** **cttgaagtacggtcccaatatttacaatctttacttctctattatattgctcaatacgtt**

**Fortunella_margarita_1** **cttgaagtacggtcccaatatttacaatctttacttctctattatattgctcaatacgtt**

**Fortunella_margarita_2** **cttgaagtacggtcccaatatttacaatctttacttctctattatattgctcaatacgtt**

**Fortunella_venosa_1** **cttgaagtacggtcccaatatttacaatctttacttctctattatattgctcaatacgtt**

**Fortunella_venosa_2** **cttgaagtacggtcccaatatttacaatctttacttctctattatattgctcaatacgtt**

**Fortunella_venosa_3** **cttgaagtacggtcccaatatttacaatctttacttctctattatattgctcaatacgtt**

13210	13220	13230	13240	13250	13260

....|....|....|....|....|....|....|....|....|....|....|....|

**Atalantia_kwangtungens** **cgcggataatattactaatttcgtcggctttaattgttgccataagtatttcttaattct**

**Citrus_reticulata_1** **cgcggataatattactaatttcgtcggctttaattgttgccataagtatttcttaattct**

**Citrus_reticulata_2** **cgcggataatattactaatttcgtcggctttaattgttgccataagtatttcttaattct**

**Citrus_trifoliata_3** **cgcggataatattactaatttcgtcggctttaattgttgccataagtatttcttaattc-**

**Citrus_aurantiifolia** **cgcggataatattactaatttcgtcggctttaattgttgtcataagtatttcttaattct**

**Citrus_aurantium** **cgcggataatattactaatttcgtcggctttaattgttgtcataagtatttcttaattct**

**Citrus_cavaleriei** **cgcggataatattactaatttcgtcggctttaattgttgccataagtatttcttaattct**

**Citrus_hongheensis** **cgcggataatattactaatttcgtcggctttaattgttgtcataagtatttcttaattct**

**Citrus_limon** **cgcggataatattactaatttcgtcggctttaattgttgtcataagtatttcttaattct**

**Citrus_maxima** **cgcggataatattactaatttcgtcggctttaattgttgtcataagtatttcttaattct**

**Citrus_medica** **cgcggataatattactaatttcgtcggctttaattgttgccataagtatttcttaattat**

**Citrus_polytrifolia** **cgcggataatattactaatttcgtcggctttaattgttgccataagtatttcttaattct**

**Citrus_reticulata** **cgcggataatattactaatttcgtcggctttaattgttgccataagtatttcttaattct**

**Citrus_sinensis** **cgcggataatattactaatttcgtcggctttaattgttgtcataagtatttcttaattct**

**Fortunella_bawangica_1** **cgcggataatattactaatttcgtcggctttaattgttgccataagtatttcttaattct**

**Fortunella_bawangica_2** **cgcggataatattactaatttcgtcggctttaattgttgccataagtatttcttaattct**

**Fortunella_hindsii_1** **cgcggataatattactaatttcgtcggctttaattgttgccataagtatttcttaattct**

**Fortunella_hindsii_2** **cgcggataatattactaatttcgtcggctttaattgttgccataagtatttcttaattct**

**Fortunella_hindsii_3** **cgcggataatattactaatttcgtcggctttaattgttgccataagtatttcttaattct**

**Fortunella_japonica_1** **cgcggataatattactaatttcgtcggctttaattgttgccataagtatttcttaattct**

**Fortunella_japonica_2** **cgcggataatattactaatttcgtcggctttaattgttgccataagtatttcttaattct**

**Fortunella_japonica_3** **cgcggataatattactaatttcgtcggctttaattgttgccataagtatttcttaattct**

**Fortunella_japonica_4** **cgcggataatattactaatttcgtcggctttaattgttgccataagtatttcttaattct**

**Fortunella_japonica_5** **cgcggataatattactaatttcgtcggctttaattgttgccataagtatttcttaattct**

**Fortunella_japonica_6** **cgcggataatattactaatttcgtcggctttaattgttgccataagtatttcttaattct**

**Fortunella_japonica_7** **cgcggataatattactaatttcgtcggctttaattgttgccataagtatttcttaattct**

**Fortunella_japonica_8** **cgcggataatattactaatttcgtcggctttaattgttgccataagtatttcttaattct**

**Fortunella_japonica_9** **cgcggataatattactaatttcgtcggctttaattgttgccataagtatttcttaattct**

**Fortunella_japonica_10** **cgcggataatattactaatttcgtcggctttaattgttgccataagtatttcttaattct**

**Fortunella_japonica_11** **cgcggataatattactaatttcgtcggctttaattgttgccataagtatttcttaattct**

**Fortunella_japonica_12** **cgcggataatattactaatttcgtcggctttaattgttgccataagtatttcttaattct**

**Fortunella_margarita_1** **cgcggataatattactaatttcgtcggctttaattgttgccataagtatttcttaattct**

**Fortunella_margarita_2** **cgcggataatattactaatttcgtcggctttaattgttgccataagtatttcttaattct**

**Fortunella_venosa_1** **cgcggataatattactaatttcgtcggctttaattgttgccataagtatttcttaattct**

**Fortunella_venosa_2** **cgcggataatattactaatttcgtcggctttaattgttgccataagtatttcttaattct**

**Fortunella_venosa_3** **cgcggataatattactaatttcgtcggctttaattgttgccataagtatttcttaattct**

13270	13280	13290	13300	13310	13320

....|....|....|....|....|....|....|....|....|....|....|....|

**Atalantia_kwangtungens** **tttttttcttcc-------------aaaaaaaaataatgtgcctacagtaaaaggactaa**

**Citrus_reticulata_1** **tttttttctt----------aaaaaaaaaaaaaataatgtgcctacagtaaaaggactaa**

**Citrus_reticulata_2** **tttttttctt----------aaaaaaaaaaaaaataatgtgcctacagtaaaaggactaa**

**Citrus_trifoliata_3** **-tttttttttct-----tcaaaaaaaaaaaaaaataatgtgcctacagtaaaaggactaa**

**Citrus_aurantiifolia** **tttttttcttca----aaaaaaaaaaaaaaaaaataatgtgcctacagtaaaaggactaa**

**Citrus_aurantium** **tttttttctt-------caaaaaaaaaaaaaaaataatgtgcctacagtaaaaggactaa**

**Citrus_cavaleriei** **ttttttttctt------aaaaaaaaaaaaaaaaataatgtgcctacagtaaaaggactaa**

**Citrus_hongheensis** **tttttttctt-------caaaaaaaaaaaaaaaataatgtgcctacagtaaaaggactaa**

**Citrus_limon** **tttttttctt-------caaaaaaaaaaaaaaaataatgtgcctacagtaaaaggactaa**

**Citrus_maxima** **tttttttctt-------aaaaaaaaaaaaaaaaataatgtgcctacagtaaaaggactaa**

**Citrus_medica** **ttttttttcttcaaaaaaaaaaaaaaaaaaaaaaaaatgtgcctacag-aaaaggactaa**

**Citrus_polytrifolia** **ttttttttttct-----tcaaaaaaaaaaaaaaataatgtgcctacagtaaaaggactaa**

**Citrus_reticulata** **tttttttctt----------aaaaaaaaaaaaaataatgtgcctacagtaaaaggactaa**

**Citrus_sinensis** **tttttttctt-------aaaaaaaaaaaaaaaaataatgtgcctacagtaaaaggactaa**

**Fortunella_bawangica_1** **tttttttcttc------aaaaaaaaaaaaaaaaataatgtgcctacagtaaaaggactaa**

**Fortunella_bawangica_2** **tttttttcttc------aaaaaaaaaaaaaaaaataatgtgcctacagtaaaaggactaa**

**Fortunella_hindsii_1** **tttttttcttc------aaaaaaaaaaaaaaaaataatgtgcctacagtaaaaggactaa**

**Fortunella_hindsii_2** **tttttttcttc------aaaaaaaaaaaaaaaaataatgtgcctacagtaaaaggactaa**

**Fortunella_hindsii_3** **tttttttcttc------aaaaaaaaaaaaaaaaataatgtgcctacagtaaaaggactaa**

**Fortunella_japonica_1** **tttttttcttc------aaaaaaaaaaaaaaaaataatgtgcctacagtaaaaggactaa**

**Fortunella_japonica_2** **tttttttcttc------aaaaaaaaaaaaaaaaataatgtgcctacagtaaaaggactaa**

**Fortunella_japonica_3** **tttttttcttc------aaaaaaaaaaaaaaaaataatgtgcctacagtaaaaggactaa**

**Fortunella_japonica_4** **tttttttcttc------aaaaaaaaaaaaaaaaataatgtgcctacagtaaaaggactaa**

**Fortunella_japonica_5** **tttttttcttc------aaaaaaaaaaaaaaaaataatgtgcctacagtaaaaggactaa**

**Fortunella_japonica_6** **tttttttcttc------aaaaaaaaaaaaaaaaataatgtgcctacagtaaaaggactaa**

**Fortunella_japonica_7** **tttttttcttc------aaaaaaaaaaaaaaaaataatgtgcctacagtaaaaggactaa**

**Fortunella_japonica_8** **tttttttcttc------aaaaaaaaaaaaaaaaataatgtgcctacagtaaaaggactaa**

**Fortunella_japonica_9** **tttttttcttc------aaaaaaaaaaaaaaaaataatgtgcctacagtaaaaggactaa**

**Fortunella_japonica_10** **tttttttcttc------aaaaaaaaaaaaaaaaataatgtgcctacagtaaaaggactaa**

**Fortunella_japonica_11** **tttttttcttc------aaaaaaaaaaaaaaaaataatgtgcctacagtaaaaggactaa**

**Fortunella_japonica_12** **tttttttcttc------aaaaaaaaaaaaaaaaataatgtgcctacagtaaaaggactaa**

**Fortunella_margarita_1** **tttttttcttc------aaaaaaaaaaaaaaaaataatgtgcctacagtaaaaggactaa**

**Fortunella_margarita_2** **tttttttcttc------aaaaaaaaaaaaaaaaataatgtgcctacagtaaaaggactaa**

**Fortunella_venosa_1** **tttttttcttc-------aaaaaaaaaaaaaaaataatgtgcctacagtaaaaggactaa**

**Fortunella_venosa_2** **tttttttcttc-------aaaaaaaaaaaaaaaataatgtgcctacagtaaaaggactaa**

**Fortunella_venosa_3** **tttttttcttc-------aaaaaaaaaaaaaaaataatgtgcctacagtaaaaggactaa**

13330	13340	13350	13360	13370	13380

....|....|....|....|....|....|....|....|....|....|....|....|

**Atalantia_kwangtungens** **tcagttatttctttcatcgctccaaacatgccaatattggcactaatggtacgtaaatgt**

**Citrus_reticulata_1** **tccgttatttctttcatcgctccaaacatgccaatattggcactaatggtacgtaaatgt**

**Citrus_reticulata_2** **tccgttatttctttcatcgctccaaacatgccaatattggcactaatggtacgtaaatgt**

**Citrus_trifoliata_3** **tcagttatttctttcatcgctccaaacatgccaatattggcactaatggtacgtaaatgt**

**Citrus_aurantiifolia** **tcagttatttctttcatcgctccaaacatgccaatattggcactaatggtacgtaaatgt**

**Citrus_aurantium** **tcagttatttctttcatcgctccaaacatgccaatattggcactaatggtacgtaaatgt**

**Citrus_cavaleriei** **tcagttatttctttcatcgctccaaacatgccaatattggcactaatggtacgtaaatgt**

**Citrus_hongheensis** **tcagttatttctttcatcgctccaaacatgccaatattggcactaatggtacgtaaatgt**

**Citrus_limon** **tcagttatttctttcatcgctccaaacatgccaatattggcactaatggtacgtaaatgt**

**Citrus_maxima** **tcagttatttctttcatcgctccaaacatgccaatattggcactaatggtacgtaaatgt**

**Citrus_medica** **t--agtttttttttcatcgctccaaacatgccaatattggcactaatggtacgtaaatgt**

**Citrus_polytrifolia** **tcagttatttctttcatcgctccaaacatgccaatattggcactaatggtacgtaaatgt**

**Citrus_reticulata** **tccgttatttctttcatcgctccaaacatgccaatattggcactaatggtacgtaaatgt**

**Citrus_sinensis** **tcagttatttctttcatcgctccaaacatgccaatattggcactaatggtacgtaaatgt**

**Fortunella_bawangica_1** **tcagttatttctttcatcgctccaaacatgccaatattggcactaatggtacgtaaatgt**

**Fortunella_bawangica_2** **tcagttatttctttcatcgctccaaacatgccaatattggcactaatggtacgtaaatgt**

**Fortunella_hindsii_1** **tcagttatttctttcatcgctccaaacatgccaatattggcactaatggtacgtaaatgt**

**Fortunella_hindsii_2** **tcagttatttctttcatcgctccaaacatgccaatattggcactaatggtacgtaaatgt**

**Fortunella_hindsii_3** **tcagttatttctttcatcgctccaaacatgccaatattggcactaatggtacgtaaatgt**

**Fortunella_japonica_1** **tcagttatttctttcatcgctccaaacatgccaatattggcactaatggtacgtaaatgt**

**Fortunella_japonica_2** **tcagttatttctttcatcgctccaaacatgccaatattggcactaatggtacgtaaatgt**

**Fortunella_japonica_3** **tcagttatttctttcatcgctccaaacatgccaatattggcactaatggtacgtaaatgt**

**Fortunella_japonica_4** **tcagttatttctttcatcgctccaaacatgccaatattggcactaatggtacgtaaatgt**

**Fortunella_japonica_5** **tcagttatttctttcatcgctccaaacatgccaatattggcactaatggtacgtaaatgt**

**Fortunella_japonica_6** **tcagttatttctttcatcgctccaaacatgccaatattggcactaatggtacgtaaatgt**

**Fortunella_japonica_7** **tcagttatttctttcatcgctccaaacatgccaatattggcactaatggtacgtaaatgt**

**Fortunella_japonica_8** **tcagttatttctttcatcgctccaaacatgccaatattggcactaatggtacgtaaatgt**

**Fortunella_japonica_9** **tcagttatttctttcatcgctccaaacatgccaatattggcactaatggtacgtaaatgt**

**Fortunella_japonica_10** **tcagttatttctttcatcgctccaaacatgccaatattggcactaatggtacgtaaatgt**

**Fortunella_japonica_11** **tcagttatttctttcatcgctccaaacatgccaatattggcactaatggtacgtaaatgt**

**Fortunella_japonica_12** **tcagttatttctttcatcgctccaaacatgccaatattggcactaatggtacgtaaatgt**

**Fortunella_margarita_1** **tcagttatttctttcatcgctccaaacatgccaatattggcactaatggtacgtaaatgt**

**Fortunella_margarita_2** **tcagttatttctttcatcgctccaaacatgccaatattggcactaatggtacgtaaatgt**

**Fortunella_venosa_1** **tcagttatttctttcatcgctccaaacatgccaatattggcactaatggtacgtaaatgt**

**Fortunella_venosa_2** **tcagttatttctttcatcgctccaaacatgccaatattggcactaatggtacgtaaatgt**

**Fortunella_venosa_3** **tcagttatttctttcatcgctccaaacatgccaatattggcactaatggtacgtaaatgt**

13390	13400	13410	13420	13430	13440

....|....|....|....|....|....|....|....|....|....|....|....|

**Atalantia_kwangtungens** **aactccttgttcaaacaactattcagagttccgagcgctccttgtaaagcttgttggaaa**

**Citrus_reticulata_1** **aactccttgttcaaacaactattcagagttccgagcgctccttgtaaagcttgttggaaa**

**Citrus_reticulata_2** **aactccttgttcaaacaactattcagagttccgagcgctccttgtaaagcttgttggaaa**

**Citrus_trifoliata_3** **aactccttgttcaaacaactattcagagttccgagcgctccttgtaaagcttgttggaaa**

**Citrus_aurantiifolia** **aactccttgttcaaacaactattcagagttccgagcgctccttgtaaagcttgttggaaa**

**Citrus_aurantium** **aactccttgttcaaacaactattcagagttccgagggctccttgtaaagcttgttggaaa**

**Citrus_cavaleriei** **aattccttgttcaaacaactattcagagttccgagcgctccttgtaaagcttgttggaaa**

**Citrus_hongheensis** **aactccttgttcaaacaactattcagagttccgagcgctccttgtaaagcttgttggaaa**

**Citrus_limon** **aactccttgttcaaacaactattcagagttccgagggctccttgtaaagcttgttggaaa**

**Citrus_maxima** **aactccttgttcaaacaactattcagagttccgagcgctccttgtaaagcttgttggaaa**

**Citrus_medica** **aactccttgttcaaacaactattcagagttccgagcgctccttgtaaagcttgttggaaa**

**Citrus_polytrifolia** **aactccttgttcaaacaactattcagagttccgagcgctccttgtaaagcttgttggaaa**

**Citrus_reticulata** **aactccttgttcaaacaactattcagagttccgagcgctccttgtaaagcttgttggaaa**

**Citrus_sinensis** **aactccttgttcaaacaactattcagagttccgagcgctccttgtaaagcttgttggaaa**

**Fortunella_bawangica_1** **aactccttgttcaaacaactattcagagttccgagcgctccttgtaaagcttgttggaaa**

**Fortunella_bawangica_2** **aactccttgttcaaacaactattcagagttccgagcgctccttgtaaagcttgttggaaa**

**Fortunella_hindsii_1** **aactccttgttcaaacaactattcagagttccgagcgctccttgtaaagcttgttggaaa**

**Fortunella_hindsii_2** **aactccttgttcaaacaactattcagagttccgagcgctccttgtaaagcttgttggaaa**

**Fortunella_hindsii_3** **aactccttgttcaaacaactattcagagttccgagcgctccttgtaaagcttgttggaaa**

**Fortunella_japonica_1** **aactccttgttcaaacaactattcagagttccgagcgctccttgtaaagcttgttggaaa**

**Fortunella_japonica_2** **aactccttgttcaaacaactattcagagttccgagcgctccttgtaaagcttgttggaaa**

**Fortunella_japonica_3** **aactccttgttcaaacaactattcagagttccgagcgctccttgtaaagcttgttggaaa**

**Fortunella_japonica_4** **aactccttgttcaaacaactattcagagttccgagcgctccttgtaaagcttgttggaaa**

**Fortunella_japonica_5** **aactccttgttcaaacaactattcagagttccgagcgctccttgtaaagcttgttggaaa**

**Fortunella_japonica_6** **aactccttgttcaaacaactattcagagttccgagcgctccttgtaaagcttgttggaaa**

**Fortunella_japonica_7** **aactccttgttcaaacaactattcagagttccgagcgctccttgtaaagcttgttggaaa**

**Fortunella_japonica_8** **aactccttgttcaaacaactattcagagttccgagcgctccttgtaaagcttgttggaaa**

**Fortunella_japonica_9** **aactccttgttcaaacaactattcagagttccgagcgctccttgtaaagcttgttggaaa**

**Fortunella_japonica_10** **aactccttgttcaaacaactattcagagttccgagcgctccttgtaaagcttgttggaaa**

**Fortunella_japonica_11** **aactccttgttcaaacaactattcagagttccgagcgctccttgtaaagcttgttggaaa**

**Fortunella_japonica_12** **aactccttgttcaaacaactattcagagttccgagcgctccttgtaaagcttgttggaaa**

**Fortunella_margarita_1** **aactccttgttcaaacaactattcagagttccgagcgctccttgtaaagcttgttggaaa**

**Fortunella_margarita_2** **aactccttgttcaaacaactattcagagttccgagcgctccttgtaaagcttgttggaaa**

**Fortunella_venosa_1** **aactccttgttcaaacaactattcagagttccgagcgctccttgtaaagcttgttggaaa**

**Fortunella_venosa_2** **aactccttgttcaaacaactattcagagttccgagcgctccttgtaaagcttgttggaaa**

**Fortunella_venosa_3** **aactccttgttcaaacaactattcagagttccgagcgctccttgtaaagcttgttggaaa**

13450	13460	13470	13480	13490	13500

....|....|....|....|....|....|....|....|....|....|....|....|

**Atalantia_kwangtungens** **acccgttgtcggacttgattaattgctctttgttgttcaaaatgaatggtttcatttttg**

**Citrus_reticulata_1** **acccgttgtcggacttgattaattgctctttgttgttcaaaatgaatggtttcatttttg**

**Citrus_reticulata_2** **acccgttgtcggacttgattaattgctctttgttgttcaaaatgaatggtttcatttttg**

**Citrus_trifoliata_3** **acccgttgtcggacttgattaattgttctttgttgttcaaaatgaatggtttcatttttg**

**Citrus_aurantiifolia** **acccgttgtcggacttgattaattgctctttgttgttcaaaatgaatggtttcatttttg**

**Citrus_aurantium** **acccgttgtcggacttgattaattgctctttgttgttcaaaatgaatggtttcatttttg**

**Citrus_cavaleriei** **acccgttgtcggacttgattaattgctctttgttgttcaaaatgaatggtttcatttttg**

**Citrus_hongheensis** **acccgttgtcggacttgattaattgctctttgttgttcaaaatgaatggtttcatttttg**

**Citrus_limon** **acccgttgtcggacttgattaattgctctttgttgttcaaaatgaatggtttcatttttg**

**Citrus_maxima** **acccgttgtcggacttgattaattgctctttgttgttcaaaatgaatggtttcatttttg**

**Citrus_medica** **acccgttgtcggacttgattaattgctctttgttgttcaaaatgaatggtttcatttttg**

**Citrus_polytrifolia** **acccgttgtcggacttgattaattgttctttgttgttcaaaatgaatggtttcatttttg**

**Citrus_reticulata** **acccgttgtcggacttgattaattgctctttgttgttcaaaatgaatggtttcatttttg**

**Citrus_sinensis** **acccgttgtcggacttgattaattgctctttgttgttcaaaatgaatggtttcatttttg**

**Fortunella_bawangica_1** **acccgttgtcggacttgattaattgctctttgttgttcaaaatgaatggtttcatttttg**

**Fortunella_bawangica_2** **acccgttgtcggacttgattaattgctctttgttgttcaaaatgaatggtttcatttttg**

**Fortunella_hindsii_1** **acccgttgtcggacttgattaattgctctttgttgttcaaaatgaatggtttcatttttg**

**Fortunella_hindsii_2** **acccgttgtcggacttgattaattgctctttgttgttcaaaatgaatggtttcatttttg**

**Fortunella_hindsii_3** **acccgttgtcggacttgattaattgctctttgttgttcaaaatgaatggtttcatttttg**

**Fortunella_japonica_1** **acccgttgtcggacttgattaattgctctttgttgttcaaaatgaatggtttcatttttg**

**Fortunella_japonica_2** **acccgttgtcggacttgattaattgctctttgttgttcaaaatgaatggtttcatttttg**

**Fortunella_japonica_3** **acccgttgtcggacttgattaattgctctttgttgttcaaaatgaatggtttcatttttg**

**Fortunella_japonica_4** **acccgttgtcggacttgattaattgctctttgttgttcaaaatgaatggtttcatttttg**

**Fortunella_japonica_5** **acccgttgtcggacttgattaattgctctttgttgttcaaaatgaatggtttcatttttg**

**Fortunella_japonica_6** **acccgttgtcggacttgattaattgctctttgttgttcaaaatgaatggtttcatttttg**

**Fortunella_japonica_7** **acccgttgtcggacttgattaattgctctttgttgttcaaaatgaatggtttcatttttg**

**Fortunella_japonica_8** **acccgttgtcggacttgattaattgctctttgttgttcaaaatgaatggtttcatttttg**

**Fortunella_japonica_9** **acccgttgtcggacttgattaattgctctttgttgttcaaaatgaatggtttcatttttg**

**Fortunella_japonica_10** **acccgttgtcggacttgattaattgctctttgttgttcaaaatgaatggtttcatttttg**

**Fortunella_japonica_11** **acccgttgtcggacttgattaattgctctttgttgttcaaaatgaatggtttcatttttg**

**Fortunella_japonica_12** **acccgttgtcggacttgattaattgctctttgttgttcaaaatgaatggtttcatttttg**

**Fortunella_margarita_1** **acccgttgtcggacttgattaattgctctttgttgttcaaaatgaatggtttcatttttg**

**Fortunella_margarita_2** **acccgttgtcggacttgattaattgctctttgttgttcaaaatgaatggtttcatttttg**

**Fortunella_venosa_1** **acccgttgtcggacttgattaattgctctttgttgttcaaaatgaatggtttcatttttg**

**Fortunella_venosa_2** **acccgttgtcggacttgattaattgctctttgttgttcaaaatgaatggtttcatttttg**

**Fortunella_venosa_3** **acccgttgtcggacttgattaattgctctttgttgttcaaaatgaatggtttcatttttg**

13510	13520	13530	13540	13550	13560

....|....|....|....|....|....|....|....|....|....|....|....|

**Atalantia_kwangtungens** **taattttctaattgatccaaagtcttataagttgaattaatcaaattgaatttttctcgt**

**Citrus_reticulata_1** **taattttctaattgatccaaagtcttataagttgaattaatcaaattgaatttttctcgt**

**Citrus_reticulata_2** **taattttctaattgatccaaagtcttataagttgaattaatcaaattgaatttttctcgt**

**Citrus_trifoliata_3** **taattttctaattgatccaaagtcttataagttgaattaatcaaattgaatttttctcgt**

**Citrus_aurantiifolia** **taattttctaattgatccaaagtcttataagttgaattaatcaaattgaatttttctcgt**

**Citrus_aurantium** **taattttctaattgatccaaagtcttataagttgaattaatcaaattgaatttttctcgt**

**Citrus_cavaleriei** **taattttctaattgatccaaagtcttataagttgaattaatcaaattgaatttttctcgt**

**Citrus_hongheensis** **taattttctaattgatccaaagtcttataagttgaattaatcaaattgaatttttctcgt**

**Citrus_limon** **taattttctaattgatccaaagtcttataagttgaattaatcaaattgaatttttctcgt**

**Citrus_maxima** **taattttctaattgatccaaagtcttataagttgaattaatcaaattgaatttttctcgt**

**Citrus_medica** **taattttctaattgatccaaagtcttataagttgaattaatcaaattgaatttttctcgt**

**Citrus_polytrifolia** **taattttctaattgatccaaagtcttataagttgaattaatcaaattgaatttttctcgt**

**Citrus_reticulata** **taattttctaattgatccaaagtcttataagttgaattaatcaaattgaatttttctcgt**

**Citrus_sinensis** **taattttctaattgatccaaagtcttataagttgaattaatcaaattgaatttttctcgt**

**Fortunella_bawangica_1** **taattttctaattgatccaaagtcttataagttgaattaatcaaattgaatttttctcgt**

**Fortunella_bawangica_2** **taattttctaattgatccaaagtcttataagttgaattaatcaaattgaatttttctcgt**

**Fortunella_hindsii_1** **taattttctaattgatccaaagtcttataagttgaattaatcaaattgaatttttctcgt**

**Fortunella_hindsii_2** **taattttctaattgatccaaagtcttataagttgaattaatcaaattgaatttttctcgt**

**Fortunella_hindsii_3** **taattttctaattgatccaaagtcttataagttgaattaatcaaattgaatttttcgcgt**

**Fortunella_japonica_1** **taattttctaattgatccaaagtcttataagttgaattaatcaaattgaatttttctcgt**

**Fortunella_japonica_2** **taattttctaattgatccaaagtcttataagttgaattaatcaaattgaatttttctcgt**

**Fortunella_japonica_3** **taattttctaattgatccaaagtcttataagttgaattaatcaaattgaatttttctcgt**

**Fortunella_japonica_4** **taattttctaattgatccaaagtcttataagttgaattaatcaaattgaatttttctcgt**

**Fortunella_japonica_5** **taattttctaattgatccaaagtcttataagttgaattaatcaaattgaatttttctcgt**

**Fortunella_japonica_6** **taattttctaattgatccaaagtcttataagttgaattaatcaaattgaatttttctcgt**

**Fortunella_japonica_7** **taattttctaattgatccaaagtcttataagttgaattaatcaaattgaatttttctcgt**

**Fortunella_japonica_8** **taattttctaattgatccaaagtcttataagttgaattaatcaaattgaatttttctcgt**

**Fortunella_japonica_9** **taattttctaattgatccaaagtcttataagttgaattaatcaaattgaatttttctcgt**

**Fortunella_japonica_10** **taattttctaattgatccaaagtcttataagttgaattaatcaaattgaatttttctcgt**

**Fortunella_japonica_11** **taattttctaattgatccaaagtcttataagttgaattaatcaaattgaatttttctcgt**

**Fortunella_japonica_12** **taattttctaattgatccaaagtcttataagttgaattaatcaaattgaatttttctcgt**

**Fortunella_margarita_1** **taattttctaattgatccaaagtcttataagttgaattaatcaaattgaatttttctcgt**

**Fortunella_margarita_2** **taattttctaattgatccaaagtcttataagttgaattaatcaaattgaatttttctcgt**

**Fortunella_venosa_1** **taattttctaattgatccaaagtcttataagttgaattaatcaaattgaatttttctcgt**

**Fortunella_venosa_2** **taattttctaattgatccaaagtcttataagttgaattaatcaaattgaatttttctcgt**

**Fortunella_venosa_3** **taattttctaattgatccaaagtcttataagttgaattaatcaaattgaatttttctcgt**

13570	13580	13590	13600	13610	13620

....|....|....|....|....|....|....|....|....|....|....|....|

**Atalantia_kwangtungens** **tctatctcagagtatccattcactcgaaactgatctgcttctatttcgatttttcgtaac**

**Citrus_reticulata_1** **tctatctcagagtatccattcactcgaaactgatctgcttctatttctatttttcgtaac**

**Citrus_reticulata_2** **tctatctcagagtatccattcactcgaaactgatctgcttctatttctatttttcgtaac**

**Citrus_trifoliata_3** **tctatctcagagtatccattcactcgaaactgatctgcttctatttctatttttcgtaac**

**Citrus_aurantiifolia** **tctatctcagagtatccattcactcgaaactgatcggcttctatttctatttttcgtaac**

**Citrus_aurantium** **tctatctccgagtatccattcactcgaaactgatctgcttctatttctatttttcgtaac**

**Citrus_cavaleriei** **tctatctcagagtatccattcactcgaaactgatctgcttctatttctatttttcgtaac**

**Citrus_hongheensis** **tctatctcagagtatccattcactcgaaactgatctgcttctatttctatttttcgtaac**

**Citrus_limon** **tctatctccgagtatccattcactcgaaactgatctgcttctatttctatttttcgtaac**

**Citrus_maxima** **tctatctcagagtatccattcactcgaaactgatctgcttctatttctatttttcgtaac**

**Citrus_medica** **tctatctcagagtatccattcactcgaaactgatctgcttctatttctatttttcgtaac**

**Citrus_polytrifolia** **tctatctcagagtatccattcactcgaaactgatctgcttctatttctatttttcgtaac**

**Citrus_reticulata** **tctatctcagagtatccattcactcgaaactgatctgcttctatttctatttttcgtaac**

**Citrus_sinensis** **tctatctcagagtatccattcactcgaaactgatctgcttctatttctatttttcgtaac**

**Fortunella_bawangica_1** **tctatctcagagtatccattcactcgaaactgatctgcttctatttctatttttcgtaac**

**Fortunella_bawangica_2** **tctatctcagagtatccattcactcgaaactgatctgcttctatttctatttttcgtaac**

**Fortunella_hindsii_1** **tctatctcagagtatccattcactcgaaactgatctgcttctatttctatttttcgtaac**

**Fortunella_hindsii_2** **tctatctcagagtatccattcactcgaaactgatctgcttctatttctatttttcgtaac**

**Fortunella_hindsii_3** **tctatctcagagtatccattcactcgaaactgatctgcttctatttctatttttcgtaac**

**Fortunella_japonica_1** **tctatctcagagtatccattcactcgaaactgatctgcttctatttctatttttcgtaac**

**Fortunella_japonica_2** **tctatctcagagtatccattcactcgaaactgatctgcttctatttctatttttcgtaac**

**Fortunella_japonica_3** **tctatctcagagtatccattcactcgaaactgatctgcttctatttctatttttcgtaac**

**Fortunella_japonica_4** **tctatctcagagtatccattcactcgaaactgatctgcttctatttctatttttcgtaac**

**Fortunella_japonica_5** **tctatctcagagtatccattcactcgaaactgatctgcttctatttctatttttcgtaac**

**Fortunella_japonica_6** **tctatctcagagtatccattcactcgaaactgatctgcttctatttctatttttcgtaac**

**Fortunella_japonica_7** **tctatctcagagtatccattcactcgaaactgatctgcttctatttctatttttcgtaac**

**Fortunella_japonica_8** **tctatctcagagtatccattcactcgaaactgatctgcttctatttctatttttcgtaac**

**Fortunella_japonica_9** **tctatctcagagtatccattcactcgaaactgatctgcttctatttctatttttcgtaac**

**Fortunella_japonica_10** **tctatctcagagtatccattcactcgaaactgatctgcttctatttctatttttcgtaac**

**Fortunella_japonica_11** **tctatctcagagtatccattcactcgaaactgatctgcttctatttctatttttcgtaac**

**Fortunella_japonica_12** **tctatctcagagtatccattcactcgaaactgatctgcttctatttctatttttcgtaac**

**Fortunella_margarita_1** **tctatctcagagtatccattcactcgaaactgatctgcttctatttctatttttcgtaac**

**Fortunella_margarita_2** **tctatctcagagtatccattcactcgaaactgatctgcttctatttctatttttcgtaac**

**Fortunella_venosa_1** **tctatctcagagtatccattcactcgaaactgatctgcttctatttctatttttcgtaac**

**Fortunella_venosa_2** **tctatctcagagtatccattcactcgaaactgatctgcttctatttctatttttcgtaac**

**Fortunella_venosa_3** **tctatctcagagtatccattcactcgaaactgatctgcttctatttctatttttcgtaac**

13630	13640	13650	13660	13670	13680

....|....|....|....|....|....|....|....|....|....|....|....|

**Atalantia_kwangtungens** **cgggcccgggctttttccagccgttcaatggcccctctctgcagttcttctgaatttcga**

**Citrus_reticulata_1** **cgggcccgggctttttccagccgttcaatggcccctctctgcagttcttctgaatttcga**

**Citrus_reticulata_2** **cgggcccgggctttttccagccgttcaatggcccctctctgcagttcttctgaatttcga**

**Citrus_trifoliata_3** **cgggcccgggctttttccagccgttcaatggcccctctctgcagttcttctgaatttcga**

**Citrus_aurantiifolia** **cgggcccgggctttttccagccgttcaatggcccctctctgcagttcttctgaatttcga**

**Citrus_aurantium** **cgggcccgggctttttccagccgttcaatggcccctctctgcagttcttctgaatttcga**

**Citrus_cavaleriei** **cgggcccgggctttttccagccgttcaatggcccctctctgcagttcttctgaatttcga**

**Citrus_hongheensis** **cgggcccgggctttttccagccgttcaatggcccctctctgcagttcttctgaatttcga**

**Citrus_limon** **cgggcccgggctttttccagccgttcaatggcccctctctgcagttcttctgaatttcga**

**Citrus_maxima** **cgggcccgggctttttccagccgttcaatggcccctctctgcagttcttctgaatttcga**

**Citrus_medica** **cgggcccgggctttttccagccgttcaatggcccctctctgcagttcttctgaatttcga**

**Citrus_polytrifolia** **cgggcccgggctttttccagccgttcaatggcccctctctgcagttcttctgaatttcga**

**Citrus_reticulata** **cgggcccgggctttttccagccgttcaatggcccctctctgcagttcttctgaatttcga**

**Citrus_sinensis** **cgggcccgggctttttccagccgttcaatggcccctctctgcagttcttctgaatttcga**

**Fortunella_bawangica_1** **cgggcccgggctttttccagccgttcaatggcccctctctgcagttcttctgaatttcga**

**Fortunella_bawangica_2** **cgggcccgggctttttccagccgttcaatggcccctctctgcagttcttctgaatttcga**

**Fortunella_hindsii_1** **cgggcccgggctttttccagccgttcaatggcccctctctgcagttcttctgaatttcga**

**Fortunella_hindsii_2** **cgggcccgggctttttccagccgttcaatggcccctctctgcagttcttctgaatttcga**

**Fortunella_hindsii_3** **cgggcccgggctttttccagccgttcaatggcccctctctgcagttcttctgaatttcga**

**Fortunella_japonica_1** **cgggcccgggctttttccagccgttcaatggcccctctctgcagttcttctgaatttcga**

**Fortunella_japonica_2** **cgggcccgggctttttccagccgttcaatggcccctctctgcagttcttctgaatttcga**

**Fortunella_japonica_3** **cgggcccgggctttttccagccgttcaatggcccctctctgcagttcttctgaatttcga**

**Fortunella_japonica_4** **cgggcccgggctttttccagccgttcaatggcccctctctgcagttcttctgaatttcga**

**Fortunella_japonica_5** **cgggcccgggctttttccagccgttcaatggcccctctctgcagttcttctgaatttcga**

**Fortunella_japonica_6** **cgggcccgggctttttccagccgttcaatggcccctctctgcagttcttctgaatttcga**

**Fortunella_japonica_7** **cgggcccgggctttttccagccgttcaatggcccctctctgcagttcttctgaatttcga**

**Fortunella_japonica_8** **cgggcccgggctttttccagccgttcaatggcccctctctgcagttcttctgaatttcga**

**Fortunella_japonica_9** **cgggcccgggctttttccagccgttcaatggcccctctctgcagttcttctgaatttcga**

**Fortunella_japonica_10** **cgggcccgggctttttccagccgttcaatggcccctctctgcagttcttctgaatttcga**

**Fortunella_japonica_11** **cgggcccgggctttttccagccgttcaatggcccctctctgcagttcttctgaatttcga**

**Fortunella_japonica_12** **cgggcccgggctttttccagccgttcaatggcccctctctgcagttcttctgaatttcga**

**Fortunella_margarita_1** **cgggcccgggctttttccagccgttcaatggcccctctctgcagttcttctgaatttcga**

**Fortunella_margarita_2** **cgggcccgggctttttccagccgttcaatggcccctctctgcagttcttctgaatttcga**

**Fortunella_venosa_1** **cgggcccgggctttttccagccgttcaatggcccctctctgcagttcttctgaatttcga**

**Fortunella_venosa_2** **cgggcccgggctttttccagccgttcaatggcccctctctgcagttcttctgaatttcga**

**Fortunella_venosa_3** **cgggcccgggctttttccagccgttcaatggcccctctctgcagttcttctgaatttcga**

13690	13700	13710	13720	13730	13740

....|....|....|....|....|....|....|....|....|....|....|....|

**Atalantia_kwangtungens** **atactattcaaaatcctcagtttgcgattatctaataaatcacttaatgaaagtagatta**

**Citrus_reticulata_1** **atactattcaaaatcctcagtttgcgattatctaataaatcacttaatgaaagtagatta**

**Citrus_reticulata_2** **atactattcaaaatcctcagtttgcgattatctaataaatcacttaatgaaagtagatta**

**Citrus_trifoliata_3** **atactattcaaaatcctcagtttgcgattatctaataaatcacttaatgaaagtagatta**

**Citrus_aurantiifolia** **atactattcaaaatcctcagtttgcgattatctaataaatcacttaatgaaagtagatta**

**Citrus_aurantium** **atactattcaaaatcctcagtttgcgattatctaataaatcacttaatgaaagtagatta**

**Citrus_cavaleriei** **atactattcaaaatcctcagtttgcgattatctaataaatcacttaatgaaagtagatta**

**Citrus_hongheensis** **atactattcaaaatcctcagtttgcgattatctaataaatcacttaatgaaagtagatta**

**Citrus_limon** **atactattcaaaatcctcagtttgcgattatctaataaatcacttaatgaaagtagatta**

**Citrus_maxima** **atactattcaaaatcctcagtttgcgattatctaataaatcacttaatgaaagtagatta**

**Citrus_medica** **atactattcaaaatcctcagtttgcgattatctaataaatcacttaatgaaagtagatta**

**Citrus_polytrifolia** **atactattcaaaatcctcagtttgcgattatctaataaatcacttaatgaaagtagatta**

**Citrus_reticulata** **atactattcaaaatcctcagtttgcgattatctaataaatcacttaatgaaagtagatta**

**Citrus_sinensis** **atactattcaaaatcctcagtttgcgattatctaataaatcacttaatgaaagtagatta**

**Fortunella_bawangica_1** **atactattcaaaatcctcagtttgcgattatctaataaatcacttaatgaaagtagatta**

**Fortunella_bawangica_2** **atactattcaaaatcctcagtttgcgattatctaataaatcacttaatgaaagtagatta**

**Fortunella_hindsii_1** **atactattcaaaatcctcagtttgcgattatctaataaatcacttaatgaaagtagatta**

**Fortunella_hindsii_2** **atactattcaaaatcctcagtttgcgattatctaataaatcacttaatgaaagtagatta**

**Fortunella_hindsii_3** **atactattcaaaatcctcagtttgcgattatctaataaatcacttaatgaaagtagatta**

**Fortunella_japonica_1** **atactattcaaaatcctcagtttgcgattatctaataaatcacttaatgaaagtagatta**

**Fortunella_japonica_2** **atactattcaaaatcctcagtttgcgattatctaataaatcacttaatgaaagtagatta**

**Fortunella_japonica_3** **atactattcaaaatcctcagtttgcgattatctaataaatcacttaatgaaagtagatta**

**Fortunella_japonica_4** **atactattcaaaatcctcagtttgcgattatctaataaatcacttaatgaaagtagatta**

**Fortunella_japonica_5** **atactattcaaaatcctcagtttgcgattatctaataaatcacttaatgaaagtagatta**

**Fortunella_japonica_6** **atactattcaaaatcctcagtttgcgattatctaataaatcacttaatgaaagtagatta**

**Fortunella_japonica_7** **atactattcaaaatcctcagtttgcgattatctaataaatcacttaatgaaagtagatta**

**Fortunella_japonica_8** **atactattcaaaatcctcagtttgcgattatctaataaatcacttaatgaaagtagatta**

**Fortunella_japonica_9** **atactattcaaaatcctcagtttgcgattatctaataaatcacttaatgaaagtagatta**

**Fortunella_japonica_10** **atactattcaaaatcctcagtttgcgattatctaataaatcacttaatgaaagtagatta**

**Fortunella_japonica_11** **atactattcaaaatcctcagtttgcgattatctaataaatcacttaatgaaagtagatta**

**Fortunella_japonica_12** **atactattcaaaatcctcagtttgcgattatctaataaatcacttaatgaaagtagatta**

**Fortunella_margarita_1** **atactattcaaaatcctcagtttgcgattatctaataaatcacttaatgaaagtagatta**

**Fortunella_margarita_2** **atactattcaaaatcctcagtttgcgattatctaataaatcacttaatgaaagtagatta**

**Fortunella_venosa_1** **atactattcaaaatcctcagtttgcgattatctaataaatcacttaatgaaagtagatta**

**Fortunella_venosa_2** **atactattcaaaatcctcagtttgcgattatctaataaatcacttaatgaaagtagatta**

**Fortunella_venosa_3** **atactattcaaaatcctcagtttgcgattatctaataaatcacttaatgaaagtagatta**

13750	13760	13770	13780	13790	13800

....|....|....|....|....|....|....|....|....|....|....|....|

**Atalantia_kwangtungens** **tctttccattcatctcaaaacttcgatgatcccttcccgaaccaaacatgaatttttcga**

**Citrus_reticulata_1** **tctttccattcatctcaaaacttcgatgatcccttcccgaaccaaacatgaatttttcga**

**Citrus_reticulata_2** **tctttccattcatctcaaaacttcgatgatcccttcccgaaccaaacatgaatttttcga**

**Citrus_trifoliata_3** **tctttccattcatctcaaaactgcgatgatcccttcccgaaccaaacatgaatttttcga**

**Citrus_aurantiifolia** **tctttccattcatctcaaaacttcgatgatcccttcccgaaccaaacatgaatttttcga**

**Citrus_aurantium** **tctttccattcatctcaaaacttcgatgatcccttcccgaaccaaacatgaatttttcga**

**Citrus_cavaleriei** **tctttccatccatctcaaaacttcgatgatcccttcccgaaccaaacatgaatttttcga**

**Citrus_hongheensis** **tctttccattcatctcaaaacttcgatgatcccttcccgaaccaaacatgaatttttcga**

**Citrus_limon** **tctttccattcatctcaaaacttcgatgatcccttcccgaaccaaacatgaatttttcga**

**Citrus_maxima** **tctttccattcatctcaaaacttcgatgatcccttcccgaaccaaacatgaatttttcga**

**Citrus_medica** **tctttccattcatctcaaaacttcgatgatcccttcccgaaccaaacatgaatttttcga**

**Citrus_polytrifolia** **tctttccattcatctcaaaactgcgatgatcccttcccgaaccaaacatgaatttttcga**

**Citrus_reticulata** **tctttccattcatctcaaaacttcgatgatcccttcccgaaccaaacatgaatttttcga**

**Citrus_sinensis** **tctttccattcatctcaaaacttcgatgatcccttcccgaaccaaacatgaatttttcga**

**Fortunella_bawangica_1** **tctttccattcatctcaaaacttcgatgatcccttcccgaaccaaacatgaatttttcga**

**Fortunella_bawangica_2** **tctttccattcatctcaaaacttcgatgatcccttcccgaaccaaacatgaatttttcga**

**Fortunella_hindsii_1** **tctttccattcatctcaaaacttcgatgatcccttcccgaaccaaacatgaatttttcga**

**Fortunella_hindsii_2** **tctttccattcatctcaaaacttcgatgatcccttcccgaaccaaacatgaatttttcga**

**Fortunella_hindsii_3** **tctttccattcatctcaaaacttcgatgatcccttcccgaaccaaacatgaatttttcga**

**Fortunella_japonica_1** **tctttccattcatctcaaaacttcgatgatcccttcccgaaccaaacatgaatttttcga**

**Fortunella_japonica_2** **tctttccattcatctcaaaacttcgatgatcccttcccgaaccaaacatgaatttttcga**

**Fortunella_japonica_3** **tctttccattcatctcaaaacttcgatgatcccttcccgaaccaaacatgaatttttcga**

**Fortunella_japonica_4** **tctttccattcatctcaaaacttcgatgatcccttcccgaaccaaacatgaatttttcga**

**Fortunella_japonica_5** **tctttccattcatctcaaaacttcgatgatcccttcccgaaccaaacatgaatttttcga**

**Fortunella_japonica_6** **tctttccattcatctcaaaacttcgatgatcccttcccgaaccaaacatgaatttttcga**

**Fortunella_japonica_7** **tctttccattcatctcaaaacttcgatgatcccttcccgaaccaaacatgaatttttcga**

**Fortunella_japonica_8** **tctttccattcatctcaaaacttcgatgatcccttcccgaaccaaacatgaatttttcga**

**Fortunella_japonica_9** **tctttccattcatctcaaaacttcgatgatcccttcccgaaccaaacatgaatttttcga**

**Fortunella_japonica_10** **tctttccattcatctcaaaacttcgatgatcccttcccgaaccaaacatgaatttttcga**

**Fortunella_japonica_11** **tctttccattcatctcaaaacttcgatgatcccttcccgaaccaaacatgaatttttcga**

**Fortunella_japonica_12** **tctttccattcatctcaaaacttcgatgatcccttcccgaaccaaacatgaatttttcga**

**Fortunella_margarita_1** **tctttccattcatctcaaaacttcgatgatcccttcccgaaccaaacatgaatttttcga**

**Fortunella_margarita_2** **tctttccattcatctcaaaacttcgatgatcccttcccgaaccaaacatgaatttttcga**

**Fortunella_venosa_1** **tctttccattcatctcaaaacttcgatgatcccttcccgaaccaaacatgaatttttcga**

**Fortunella_venosa_2** **tctttccattcatctcaaaacttcgatgatcccttcccgaaccaaacatgaatttttcga**

**Fortunella_venosa_3** **tctttccattcatctcaaaacttcgatgatcccttcccgaaccaaacatgaatttttcga**

13810	13820	13830	13840	13850	13860

....|....|....|....|....|....|....|....|....|....|....|....|

**Atalantia_kwangtungens** **ttcatttggctctcccactcaattacgtcaactgtatg-----ggggggggttcccatat**

**Citrus_reticulata_1** **ttcatttggctctcccactcaattacgtcaactacttatgtatggggggggttcccatat**

**Citrus_reticulata_2** **ttcatttggctctcccactcaattacgtcaactacttatgtatggggggggttcccatat**

**Citrus_trifoliata_3** **ttcatttggctctcccactcaattacgtcaactacttatgtatggggggggttcccatat**

**Citrus_aurantiifolia** **ttcatttggctctcccactcaattacgtcaactacttatgtatggggggggttcccatat**

**Citrus_aurantium** **ttcatttggctctcccactcaattacgtcaactacttatgtatggggggggttcccatat**

**Citrus_cavaleriei** **ttcatttggctctcccactcaattacgtcaactacttatgtatggggggggttcccatat**

**Citrus_hongheensis** **ttcatttggctctcccactcaattacgtcaactacttatgtatggggggggttcccatat**

**Citrus_limon** **ttcatttggctctcccactcaattacgtcaactacttatgtatggggggggttcccatat**

**Citrus_maxima** **ttcatttggctctcccactcaattacgtcaactacttatgtatggggggggttcccatat**

**Citrus_medica** **ttcatttggctctcccactcaattacgtcaactacttatgtatggggggggttcccatat**

**Citrus_polytrifolia** **ttcatttggctctcccactcaattacgtcaactacttatgtatggggggggttcccatat**

**Citrus_reticulata** **ttcatttggctctcccactcaattacgtcaactacttatgtatggggggggttcccatat**

**Citrus_sinensis** **ttcatttggctctcccactcaattacgtcaactacttatgtatggggggggttcccatat**

**Fortunella_bawangica_1** **ttcatttggctctcccactcaattacgtcaactacttatgtatggggggggttcccatat**

**Fortunella_bawangica_2** **ttcatttggctctcccactcaattacgtcaactacttatgtatggggggggttcccatat**

**Fortunella_hindsii_1** **ttcatttggctctcccactcaattacgtcaactacttatgtatggggggggttcccatat**

**Fortunella_hindsii_2** **ttcatttggctctcccactcaattacgtcaactacttatgtatggggggggttcccatat**

**Fortunella_hindsii_3** **ttcatttggctctcccactcaattacgtcaactacttatgtatggggggggttcccatat**

**Fortunella_japonica_1** **ttcatttggctctcccactcaattacgtcaactacttatgtatggggggggttcccatat**

**Fortunella_japonica_2** **ttcatttggctctcccactcaattacgtcaactacttatgtatggggggggttcccatat**

**Fortunella_japonica_3** **ttcatttggctctcccactcaattacgtcaactacttatgtatggggggggttcccatat**

**Fortunella_japonica_4** **ttcatttggctctcccactcaattacgtcaactacttatgtatggggggggttcccatat**

**Fortunella_japonica_5** **ttcatttggctctcccactcaattacgtcaactacttatgtatggggggggttcccatat**

**Fortunella_japonica_6** **ttcatttggctctcccactcaattacgtcaactacttatgtatggggggggttcccatat**

**Fortunella_japonica_7** **ttcatttggctctcccactcaattacgtcaactacttatgtatggggggggttcccatat**

**Fortunella_japonica_8** **ttcatttggctctcccactcaattacgtcaactacttatgtatggggggggttcccatat**

**Fortunella_japonica_9** **ttcatttggctctcccactcaattacgtcaactacttatgtatggggggggttcccatat**

**Fortunella_japonica_10** **ttcatttggctctcccactcaattacgtcaactacttatgtatggggggggttcccatat**

**Fortunella_japonica_11** **ttcatttggctctcccactcaattacgtcaactacttatgtatggggggggttcccatat**

**Fortunella_japonica_12** **ttcatttggctctcccactcaattacgtcaactacttatgtatggggggggttcccatat**

**Fortunella_margarita_1** **ttcatttggctctcccactcaattacgtcaactacttatgtatggggggggttcccatat**

**Fortunella_margarita_2** **ttcatttggctctcccactcaattacgtcaactacttatgtatggggggggttcccatat**

**Fortunella_venosa_1** **ttcatttggctctcccactcaattacgtcaactacttatgtatggggggggttcccatat**

**Fortunella_venosa_2** **ttcatttggctctcccactcaattacgtcaactacttatgtatggggggggttcccatat**

**Fortunella_venosa_3** **ttcatttggctctcccactcaattacgtcaactacttatgtatggggggggttcccatat**

13870	13880	13890	13900	13910	13920

....|....|....|....|....|....|....|....|....|....|....|....|

**Atalantia_kwangtungens** **ctttttttaatgtaatgagcctatcctctccctctcttctctattcatattccaaaatga**

**Citrus_reticulata_1** **ctttttttaatgtaatgagcctatcctctccctctcttctctattcatattccaaaatga**

**Citrus_reticulata_2** **ctttttttaatgtaatgagcctatcctctccctctcttctctattcatattccaaaatga**

**Citrus_trifoliata_3** **ctttttttaatgtaatgagcctatcctctccctctcttctctattcatattccaaaatga**

**Citrus_aurantiifolia** **ctttttttaatgtaatgagcctatcctctccctctcttctctattcatattccaaaatga**

**Citrus_aurantium** **ctttttttaatgtaatgagcctatcctctccctctcttctctattcatattccaaaatga**

**Citrus_cavaleriei** **ctttttttaatgtaatgagcctatcctctccctctcttctctattcatattccaaaatga**

**Citrus_hongheensis** **ctttttttaatgtaatgagcctatcctctccctctcttctctattcatattccaaaatga**

**Citrus_limon** **ctttttttaatgtaatgagcctatcctctccctctcttctctattcatattccaaaatga**

**Citrus_maxima** **ctttttttaatgtaatgagcctatcctctccctctcttctctattcatattccaaaatga**

**Citrus_medica** **ctttttttaatgtaatgagcctatcctctccctctcttctctattcatattccaaaatga**

**Citrus_polytrifolia** **ctttttttaatgtaatgagcctatcctctccctctcttctctattcatattccaaaatga**

**Citrus_reticulata** **ctttttttaatgtaatgagcctatcctctccctctcttctctattcatattccaaaatga**

**Citrus_sinensis** **ctttttttaatgtaatgagcctatcctctccctctcttctctattcatattccaaaatga**

**Fortunella_bawangica_1** **ctttttttaatgtaatgagcctatcctctccctctcttctctattcatattccaaaatga**

**Fortunella_bawangica_2** **ctttttttaatgtaatgagcctatcctctccctctcttctctattcatattccaaaatga**

**Fortunella_hindsii_1** **ctttttttaatgtaatgagcctatcctctccctctcttctctattcatattccaaaatga**

**Fortunella_hindsii_2** **ctttttttaatgtaatgagcctatcctctccctctcttctctattcatattccaaaatga**

**Fortunella_hindsii_3** **ctttttttaatgtaatgagcctatcctctccctctcttctctattcatattccaaaatga**

**Fortunella_japonica_1** **ctttttttaatgtaatgagcctatcctctccctctcttctctattcatattccaaaatga**

**Fortunella_japonica_2** **ctttttttaatgtaatgagcctatcctctccctctcttctctattcatattccaaaatga**

**Fortunella_japonica_3** **ctttttttaatgtaatgagcctatcctctccctctcttctctattcatattccaaaatga**

**Fortunella_japonica_4** **ctttttttaatgtaatgagcctatcctctccctctcttctctattcatattccaaaatga**

**Fortunella_japonica_5** **ctttttttaatgtaatgagcctatcctctccctctcttctctattcatattccaaaatga**

**Fortunella_japonica_6** **ctttttttaatgtaatgagcctatcctctccctctcttctctattcatattccaaaatga**

**Fortunella_japonica_7** **ctttttttaatgtaatgagcctatcctctccctctcttctctattcatattccaaaatga**

**Fortunella_japonica_8** **ctttttttaatgtaatgagcctatcctctccctctcttctctattcatattccaaaatga**

**Fortunella_japonica_9** **ctttttttaatgtaatgagcctatcctctccctctcttctctattcatattccaaaatga**

**Fortunella_japonica_10** **ctttttttaatgtaatgagcctatcctctccctctcttctctattcatattccaaaatga**

**Fortunella_japonica_11** **ctttttttaatgtaatgagcctatcctctccctctcttctctattcatattccaaaatga**

**Fortunella_japonica_12** **ctttttttaatgtaatgagcctatcctctccctctcttctctattcatattccaaaatga**

**Fortunella_margarita_1** **ctttttttaatgtaatgagcctatcctctccctctcttctctattcatattccaaaatga**

**Fortunella_margarita_2** **ctttttttaatgtaatgagcctatcctctccctctcttctctattcatattccaaaatga**

**Fortunella_venosa_1** **ctttttttaatgtaatgagcctatcctctccctctcttctctattcatattccaaaatga**

**Fortunella_venosa_2** **ctttttttaatgtaatgagcctatcctctccctctcttctctattcatattccaaaatga**

**Fortunella_venosa_3** **ctttttttaatgtaatgagcctatcctctccctctcttctctattcatattccaaaatga**

13930	13940	13950	13960	13970	13980

....|....|....|....|....|....|....|....|....|....|....|....|

**Atalantia_kwangtungens** **atcttgcgactgatccctaatccaagaacagaatattcggaggactcttctgaccaaatc**

**Citrus_reticulata_1** **atcttgcgactgatccctaatccaagaacagaatattcggaggactcttctgaccaaatc**

**Citrus_reticulata_2** **atcttgcgactgatccctaatccaagaacagaatattcggaggactcttctgaccaaatc**

**Citrus_trifoliata_3** **atcttgcgactgatccctaatccaagaacagaatattcggaggactcttctgaccaaatc**

**Citrus_aurantiifolia** **atcttgcgactgatccctaatccaagaacagaatattcggaggactcttctgaccaaatc**

**Citrus_aurantium** **atcttgcgactgatccctaatccaagaacagaatattcggaggactcttctgaccaaatc**

**Citrus_cavaleriei** **atcttgcgactgatccctaatccaagaacagaatattcggaggactcttctgaccaaatc**

**Citrus_hongheensis** **atcttgcgactgatccctaatccaagaacagaatattcggaggactcttctgaccaaatc**

**Citrus_limon** **atcttgcgactgatccctaatccaagaacagaatattcggaggactcttctgaccaaatc**

**Citrus_maxima** **atcttgcgactgatccctaatccaagaacagaatattcggaggactcttctgaccaaatc**

**Citrus_medica** **atcttgcgactgatccctaatccaagaacagaatattcggaggactcttctgaccaaatc**

**Citrus_polytrifolia** **atcttgcgactgatccctaatccaagaacagaatattcggaggactcttctgaccaaatc**

**Citrus_reticulata** **atcttgcgactgatccctaatccaagaacagaatattcggaggactcttctgaccaaatc**

**Citrus_sinensis** **atcttgcgactgatccctaatccaagaacagaatattcggaggactcttctgaccaaatc**

**Fortunella_bawangica_1** **atcttgcgactgatccctaatccaagaacagaatattcggaggactcttctgatcaaatc**

**Fortunella_bawangica_2** **atcttgcgactgatccctaatccaagaacagaatattcggaggactcttctgatcaaatc**

**Fortunella_hindsii_1** **atcttgcgactgatccctaatccaagaacagaatattcggaggactcttctgatcaaatc**

**Fortunella_hindsii_2** **atcttgcgactgatccctaatccaagaacagaatattcggaggactcttctgatcaaatc**

**Fortunella_hindsii_3** **atcttgcgactgatccctaatccaagaacagaatattcggaggactcttctgatcaaatc**

**Fortunella_japonica_1** **atcttgcgactgatccctaatccaagaacagaatattcggaggactcttctgatcaaatc**

**Fortunella_japonica_2** **atcttgcgactgatccctaatccaagaacagaatattcggaggactcttctgatcaaatc**

**Fortunella_japonica_3** **atcttgcgactgatccctaatccaagaacagaatattcggaggactcttctgatcaaatc**

**Fortunella_japonica_4** **atcttgcgactgatccctaatccaagaacagaatattcggaggactcttctgatcaaatc**

**Fortunella_japonica_5** **atcttgcgactgatccctaatccaagaacagaatattcggaggactcttctgatcaaatc**

**Fortunella_japonica_6** **atcttgcgactgatccctaatccaagaacagaatattcggaggactcttctgatcaaatc**

**Fortunella_japonica_7** **atcttgcgactgatccctaatccaagaacagaatattcggaggactcttctgatcaaatc**

**Fortunella_japonica_8** **atcttgcgactgatccctaatccaagaacagaatattcggaggactcttctgatcaaatc**

**Fortunella_japonica_9** **atcttgcgactgatccctaatccaagaacagaatattcggaggactcttctgatcaaatc**

**Fortunella_japonica_10** **atcttgcgactgatccctaatccaagaacagaatattcggaggactcttctgatcaaatc**

**Fortunella_japonica_11** **atcttgcgactgatccctaatccaagaacagaatattcggaggactcttctgatcaaatc**

**Fortunella_japonica_12** **atcttgcgactgatccctaatccaagaacagaatattcggaggactcttctgatcaaatc**

**Fortunella_margarita_1** **atcttgcgactgatccctaatccaagaacagaatattcggaggactcttctgatcaaatc**

**Fortunella_margarita_2** **atcttgcgactgatccctaatccaagaacagaatattcggaggactcttctgatcaaatc**

**Fortunella_venosa_1** **atcttgcgactgatccctaatccaagaacagaatattcggaggactcttctgatcaaatc**

**Fortunella_venosa_2** **atcttgcgactgatccctaatccaagaacagaatattcggaggactcttctgatcaaatc**

**Fortunella_venosa_3** **atcttgcgactgatccctaatccaagaacagaatattcggaggactcttctgatcaaatc**

13990	14000	14010	14020	14030	14040

....|....|....|....|....|....|....|....|....|....|....|....|

**Atalantia_kwangtungens** **aaaatatataattgtcagcaaagttgtttctttttttcttcaaatccaaagaattattct**

**Citrus_reticulata_1** **aaaatatataattgtcagcaaagttgtttctttttttcttcaaatccaaagaattcttct**

**Citrus_reticulata_2** **aaaatatataattgtcagcaaagttgtttctttttttcttcaaatccaaagaattcttct**

**Citrus_trifoliata_3** **aaaatctataattgtcagcaaagttgtttctttttttcttcaaatccaaagaattcttct**

**Citrus_aurantiifolia** **aaaatatataattgtcaacaaagttgtttctttttttcttcaaatccaaagaattcttct**

**Citrus_aurantium** **aaaatatataattgtcagcaaagttgtttctttttttcttcaaatccaaagaattcttct**

**Citrus_cavaleriei** **aaaatatataattgtcagcaaagttgtttctttttttctgcaaatccaaagaattcttct**

**Citrus_hongheensis** **aaaatatataattgtcagcaaagttgtttctttttttcttcaaatccaaagaattcttct**

**Citrus_limon** **aaaatatataattgtcagcaaagttgtttctttttttcttcaaatccaaagaattcttct**

**Citrus_maxima** **aaaatatataattgtcagcaaagttgtttctttttttcttcaaatccaaagaattcttct**

**Citrus_medica** **aaaatatataattgtcagcaaagttgtttctttttttcttcaaatccaaagaattcttct**

**Citrus_polytrifolia** **aaaatctataattgtcagcaaagttgtttctttttttcttcaaatccaaagaattcttct**

**Citrus_reticulata** **aaaatatataattgtcagcaaagttgtttctttttttcttcaaatccaaagaattcttct**

**Citrus_sinensis** **aaaatatataattgtcagcaaagttgtttctttttttcttcaaatccaaagaattcttct**

**Fortunella_bawangica_1** **aaaatatataattgtcagcaaagttgtttctttttttcttcaaatccaaagaattcttct**

**Fortunella_bawangica_2** **aaaatatataattgtcagcaaagttgtttctttttttcttcaaatccaaagaattcttct**

**Fortunella_hindsii_1** **aaaatatataattgtcagcaaagttgtttctttttttcttcaaatccaaagaattcttct**

**Fortunella_hindsii_2** **aaaatatataattgtcagcaaagttgtttctttttttcttcaaatccaaagaattcttct**

**Fortunella_hindsii_3** **aaaatatataattgtcagcaaagttgtttctttttttcttcaaatccaaagaattcttct**

**Fortunella_japonica_1** **aaaatatataattgtcagcaaagttgtttctttttttcttcaaatccaaagaattcttct**

**Fortunella_japonica_2** **aaaatatataattgtcagcaaagttgtttctttttttcttcaaatccaaagaattcttct**

**Fortunella_japonica_3** **aaaatatataattgtcagcaaagttgtttctttttttcttcaaatccaaagaattcttct**

**Fortunella_japonica_4** **aaaatatataattgtcagcaaagttgtttctttttttcttcaaatccaaagaattcttct**

**Fortunella_japonica_5** **aaaatatataattgtcagcaaagttgtttctttttttcttcaaatccaaagaattcttct**

**Fortunella_japonica_6** **aaaatatataattgtcagcaaagttgtttctttttttcttcaaatccaaagaattcttct**

**Fortunella_japonica_7** **aaaatatataattgtcagcaaagttgtttctttttttcttcaaatccaaagaattcttct**

**Fortunella_japonica_8** **aaaatatataattgtcagcaaagttgtttctttttttcttcaaatccaaagaattcttct**

**Fortunella_japonica_9** **aaaatatataattgtcagcaaagttgtttctttttttcttcaaatccaaagaattcttct**

**Fortunella_japonica_10** **aaaatatataattgtcagcaaagttgtttctttttttcttcaaatccaaagaattcttct**

**Fortunella_japonica_11** **aaaatatataattgtcagcaaagttgtttctttttttcttcaaatccaaagaattcttct**

**Fortunella_japonica_12** **aaaatatataattgtcagcaaagttgtttctttttttcttcaaatccaaagaattcttct**

**Fortunella_margarita_1** **aaaatatataattgtcagcaaagttgtttctttttttcttcaaatccaaagaattcttct**

**Fortunella_margarita_2** **aaaatatataattgtcagcaaagttgtttctttttttcttcaaatccaaagaattcttct**

**Fortunella_venosa_1** **aaaatatataattgtcagcaaagttgtttctttttttcttcaaatccaaagaattcttct**

**Fortunella_venosa_2** **aaaatatataattgtcagcaaagttgtttctttttttcttcaaatccaaagaattcttct**

**Fortunella_venosa_3** **aaaatatataattgtcagcaaagttgtttctttttttcttcaaatccaaagaattcttct**

14050	14060	14070	14080	14090	14100

....|....|....|....|....|....|....|....|....|....|....|....|

**Atalantia_kwangtungens** **tactttctatggaggtcatcgattcagcataaataaggggttggttgaaatacctatttt**

**Citrus_reticulata_1** **tactttatatggaggtcatcgattcagcataaataa-gggttggttgaaatacctatttt**

**Citrus_reticulata_2** **tactttatatggaggtcatcgattcagcataaataa-gggttggttgaaatacctatttt**

**Citrus_trifoliata_3** **tactttatatggaggtcatcgattcagcataaataa-gggttggttgaaatacctatttt**

**Citrus_aurantiifolia** **tactttatatggaggtcatcgattcagcataaataa-gggttggttgaaatacctatttt**

**Citrus_aurantium** **tactttatatggaggtcatcgattcagcataaataa-gggttggttgaaatacctatttt**

**Citrus_cavaleriei** **tactttatatggaggtcatcgattcagcataaataa-gggttggttgaaatacctatttt**

**Citrus_hongheensis** **tactttatatggaggtcatcgattcagcataaataa-gggttggttgaaatacctatttt**

**Citrus_limon** **tactttatatggaggtcatcgattcagcataaataa-gggttggttgaaatacctatttt**

**Citrus_maxima** **tactttatatggaggtcatcgattcagcataaataa-gggttggttgaaatacctatttt**

**Citrus_medica** **tactttatatggaggtcatcgattcagcataaataa-gggttggttgaaatacctatttt**

**Citrus_polytrifolia** **tactttatatggaggtcatcgattcagcataaataa-gggttggttgaaatacctatttt**

**Citrus_reticulata** **tactttatatggaggtcatcgattcagcataaataa-gggttggttgaaatacctatttt**

**Citrus_sinensis** **tactttatatggaggtcatcgattcagcataaataa-gggttggttgaaatacctatttt**

**Fortunella_bawangica_1** **tactttatatggaggtcatcgattcagcataaataa-gggttggttgaaatacctatttt**

**Fortunella_bawangica_2** **tactttatatggaggtcatcgattcagcataaataa-gggttggttgaaatacctatttt**

**Fortunella_hindsii_1** **tactttatatggaggtcatcgattcagcataaataa-gggttggttgaaatacctatttt**

**Fortunella_hindsii_2** **tactttatatggaggtcatcgattcagcataaataa-gggttggttgaaatacctatttt**

**Fortunella_hindsii_3** **tactttatatggaggtcatcgattcagcataaataa-gggttggttgaaatacctatttt**

**Fortunella_japonica_1** **tactttatatggaggtcatcgattcagcataaataa-gggttggttgaaatacctatttt**

**Fortunella_japonica_2** **tactttatatggaggtcatcgattcagcataaataa-gggttggttgaaatacctatttt**

**Fortunella_japonica_3** **tactttatatggaggtcatcgattcagcataaataa-gggttggttgaaatacctatttt**

**Fortunella_japonica_4** **tactttatatggaggtcatcgattcagcataaataa-gggttggttgaaatacctatttt**

**Fortunella_japonica_5** **tactttatatggaggtcatcgattcagcataaataa-gggttggttgaaatacctatttt**

**Fortunella_japonica_6** **tactttatatggaggtcatcgattcagcataaataa-gggttggttgaaatacctatttt**

**Fortunella_japonica_7** **tactttatatggaggtcatcgattcagcataaataa-gggttggttgaaatacctatttt**

**Fortunella_japonica_8** **tactttatatggaggtcatcgattcagcataaataa-gggttggttgaaatacctatttt**

**Fortunella_japonica_9** **tactttatatggaggtcatcgattcagcataaataa-gggttggttgaaatacctatttt**

**Fortunella_japonica_10** **tactttatatggaggtcatcgattcagcataaataa-gggttggttgaaatacctatttt**

**Fortunella_japonica_11** **tactttatatggaggtcatcgattcagcataaataa-gggttggttgaaatacctatttt**

**Fortunella_japonica_12** **tactttatatggaggtcatcgattcagcataaataa-gggttggttgaaatacctatttt**

**Fortunella_margarita_1** **tactttatatggaggtcatcgattcagcataaataa-gggttggttgaaatacctatttt**

**Fortunella_margarita_2** **tactttatatggaggtcatcgattcagcataaataa-gggttggttgaaatacctatttt**

**Fortunella_venosa_1** **tactttatatggaggtcatcgattcagcataaataa-gggttggttgaaatacctatttt**

**Fortunella_venosa_2** **tactttatatggaggtcatcgattcagcataaataa-gggttggttgaaatacctatttt**

**Fortunella_venosa_3** **tactttatatggaggtcatcgattcagcataaataa-gggttggttgaaatacctatttt**

14110	14120	14130	14140	14150	14160

....|....|....|....|....|....|....|....|....|....|....|....|

**Atalantia_kwangtungens** **tccaatatttttattttccaataa-------aatttccactaccaataaaaggctcaaat**

**Citrus_reticulata_1** **tccaa------tattttccaataa-------aatttccaataccaataaaaggctcaaat**

**Citrus_reticulata_2** **tccaa------tattttccaataa-------aatttccaataccaataaaaggctcaaat**

**Citrus_trifoliata_3** **tccaa------tattttccaataa-------aatttccaataccaataaaaggctcaaat**

**Citrus_aurantiifolia** **tccaa------tattttccaataa-------aatttccaataccaataaaaggctcaaat**

**Citrus_aurantium** **tccaa------tattttccaatca-------aatttccaataccaataaaaggctcaaat**

**Citrus_cavaleriei** **tccaa------tattttccaataa-------aatttccaataccaataaaaggctcaaat**

**Citrus_hongheensis** **tccaa------tattttccaatca-------aatttccaataccaataaaaggctcaaat**

**Citrus_limon** **tccaa------tattttccaatca-------aatttccaataccaataaaaggctcaaat**

**Citrus_maxima** **tccaa------tattttccaatca-------aatttccaataccaataaaaggctcaaat**

**Citrus_medica** **tccaa------tattttccaataaaatttaaaatttccaataccaataaaaggctcaaat**

**Citrus_polytrifolia** **tccaa------tattttccaataa-------aatttccaataccaataaaaggctcaaat**

**Citrus_reticulata** **tccaa------tattttccaataa-------aatttccaataccaataaaaggctcaaat**

**Citrus_sinensis** **tccaa------tattttccaatca-------aatttccaataccaataaaaggctcaaat**

**Fortunella_bawangica_1** **tccaa------tattttccaataa-------aatttccaataccaataaaaggctcaaat**

**Fortunella_bawangica_2** **tccaa------tattttccaataa-------aatttccaataccaataaaaggctcaaat**

**Fortunella_hindsii_1** **tccaa------tattttccaataa-------aatttccaataccaataaaaggctcaaat**

**Fortunella_hindsii_2** **tccaa------tattttccaataa-------aatttccaataccaataaaaggctcaaat**

**Fortunella_hindsii_3** **tccaa------tattttccaataa-------aatttccaataccaataaaaggctcaaat**

**Fortunella_japonica_1** **tccaa------tattttccaataa-------aatttccaataccaataaaaggctcaaat**

**Fortunella_japonica_2** **tccaa------tattttccaataa-------aatttccaataccaataaaaggctcaaat**

**Fortunella_japonica_3** **tccaa------tattttccaataa-------aatttccaataccaataaaaggctcaaat**

**Fortunella_japonica_4** **tccaa------tattttccaataa-------aatttccaataccaataaaaggctcaaat**

**Fortunella_japonica_5** **tccaa------tattttccaataa-------aatttccaataccaataaaaggctcaaat**

**Fortunella_japonica_6** **tccaa------tattttccaataa-------aatttccaataccaataaaaggctcaaat**

**Fortunella_japonica_7** **tccaa------tattttccaataa-------aatttccaataccaataaaaggctcaaat**

**Fortunella_japonica_8** **tccaa------tattttccaataa-------aatttccaataccaataaaaggctcaaat**

**Fortunella_japonica_9** **tccaa------tattttccaataa-------aatttccaataccaataaaaggctcaaat**

**Fortunella_japonica_10** **tccaa------tattttccaataa-------aatttccaataccaataaaaggctcaaat**

**Fortunella_japonica_11** **tccaa------tattttccaataa-------aatttccaataccaataaaaggctcaaat**

**Fortunella_japonica_12** **tccaa------tattttccaataa-------aatttccaataccaataaaaggctcaaat**

**Fortunella_margarita_1** **tccaa------tattttccaataa-------aatttccaataccaataaaaggctcaaat**

**Fortunella_margarita_2** **tccaa------tattttccaataa-------aatttccaataccaataaaaggctcaaat**

**Fortunella_venosa_1** **tccaa------tattttccaataa-------aatttccaataccaataaaaggctcaaat**

**Fortunella_venosa_2** **tccaa------tattttccaataa-------aatttccaataccaataaaaggctcaaat**

**Fortunella_venosa_3** **tccaa------tattttccaataa-------aatttccaataccaataaaaggctcaaat**

14170	14180	14190	14200	14210	14220

....|....|....|....|....|....|....|....|....|....|....|....|

**Atalantia_kwangtungens** **ctttttatcaacatgagtgttttatatcg---aaaaaaaaaagttcaataatta------**

**Citrus_reticulata_1** **ctttttatcaacatgagtgttttatatcgaaaaaaaaaaaaagtccaataatta------**

**Citrus_reticulata_2** **ctttttatcaacatgagtgttttatatcgaaaaaaaaaaaaagtccaataatta------**

**Citrus_trifoliata_3** **ctttttatcaacatgagtgttttatatcg----aaaaaaaaagtccaataatta------**

**Citrus_aurantiifolia** **ctttttatcaacatgagtgttttatatcg----aaaaaaaaagtccaataattattatta**

**Citrus_aurantium** **ctttttatcaacatgagtgttttatatcg---aaaaaaaaaagtccaataattattatta**

**Citrus_cavaleriei** **ctttttatcaacatgagtgttttatatcg----aaaaaaaaagtccaataatta------**

**Citrus_hongheensis** **ctttttatcaacatgagtgttttatatcg----aaaaaaaaagtccaataattattatta**

**Citrus_limon** **ctttttatcaacatgagtgttttatatcg---aaaaaaaaaagtccaataattattatta**

**Citrus_maxima** **ctttttatcaacatgagtgttttatatcg---aaaaaaaaaagtccaataattattatta**

**Citrus_medica** **ctttttatcaacatgagtgttttatatcg--aaaaaaaaaaagtccaataatta------**

**Citrus_polytrifolia** **ctttttatcaacatgagtgttttatatcg-aaaaaaaaaaaagtccaataatta------**

**Citrus_reticulata** **ctttttatcaacatgagtgttttatatcgaaaaaaaaaaaaagtccaataatta------**

**Citrus_sinensis** **ctttttatcaacatgagtgttttatatcg--aaaaaaaaaaagtccaataattattatta**

**Fortunella_bawangica_1** **ctttttatcaacatgagtgttttatatcg----aaaaaaaaagtccaataatta------**

**Fortunella_bawangica_2** **ctttttatcaacatgagtgttttatatcg----aaaaaaaaagtccaataatta------**

**Fortunella_hindsii_1** **ctttttatcaacatgagtgttttatatcg----aaaaaaaaagtccaataatta------**

**Fortunella_hindsii_2** **ctttttatcaacatgagtgttttatatcg----aaaaaaaaagtccaataatta------**

**Fortunella_hindsii_3** **ctttttatcaacatgagtgttttatatcg----aaaaaaaaagtccaataatta------**

**Fortunella_japonica_1** **ctttttatcaacatgagtgttttatatcg----aaaaaaaaagtccaataatta------**

**Fortunella_japonica_2** **ctttttatcaacatgagtgttttatatcg----aaaaaaaaagtccaataatta------**

**Fortunella_japonica_3** **ctttttatcaacatgagtgttttatatcg----aaaaaaaaagtccaataatta------**

**Fortunella_japonica_4** **ctttttatcaacatgagtgttttatatcg----aaaaaaaaagtccaataatta------**

**Fortunella_japonica_5** **ctttttatcaacatgagtgttttatatcg----aaaaaaaaagtccaataatta------**

**Fortunella_japonica_6** **ctttttatcaacatgagtgttttatatcg----aaaaaaaaagtccaataatta------**

**Fortunella_japonica_7** **ctttttatcaacatgagtgttttatatcg----aaaaaaaaagtccaataatta------**

**Fortunella_japonica_8** **ctttttatcaacatgagtgttttatatcg----aaaaaaaaagtccaataatta------**

**Fortunella_japonica_9** **ctttttatcaacatgagtgttttatatcg----aaaaaaaaagtccaataatta------**

**Fortunella_japonica_10** **ctttttatcaacatgagtgttttatatcg----aaaaaaaaagtccaataatta------**

**Fortunella_japonica_11** **ctttttatcaacatgagtgttttatatcg----aaaaaaaaagtccaataatta------**

**Fortunella_japonica_12** **ctttttatcaacatgagtgttttatatcg----aaaaaaaaagtccaataatta------**

**Fortunella_margarita_1** **ctttttatcaacatgagtgttttatatcg----aaaaaaaaagtccaataatta------**

**Fortunella_margarita_2** **ctttttatcaacatgagtgttttatatcg----aaaaaaaaagtccaataatta------**

**Fortunella_venosa_1** **ctttttatcaacatgagtgttttatatcg----aaaaaaaaagtccaataatta------**

**Fortunella_venosa_2** **ctttttatcaacatgagtgttttatatcg----aaaaaaaaagtccaataatta------**

**Fortunella_venosa_3** **ctttttatcaacatgagtgttttatatcg----aaaaaaaaagtccaataatta------**

14230	14240	14250	14260	14270	14280

....|....|....|....|....|....|....|....|....|....|....|....|

**Atalantia_kwangtungens** **---------ttattaattattcattttgaaaccatt-------tctattctatagtaaaa**

**Citrus_reticulata_1** **---------ttattaattattcattttgaaaacatt-------tctattctatagtaaaa**

**Citrus_reticulata_2** **---------ttattaattattcattttgaaaacatt-------tctattctatagtaaaa**

**Citrus_trifoliata_3** **---------ttattaattattcattttgaaaacatt-------tctattctatagtaaaa**

**Citrus_aurantiifolia** **attattcatttattaattattcattttgaaaacatt-------tctattctatagtaaaa**

**Citrus_aurantium** **attattcatttattaattattcattttgaaaacatt-------tctattctatagtaaaa**

**Citrus_cavaleriei** **---------ttattaattattcattttgaaaacatt-------tctattctatagtaaaa**

**Citrus_hongheensis** **attattcatttattaattattcattttgaaaacatt-------tctattctatagtaaaa**

**Citrus_limon** **attattcatttattaattattcattttgaaaacatt-------tctattctatagtaaaa**

**Citrus_maxima** **attattcatttattaattattcattttgaaaacatt-------tctattctatagtaaaa**

**Citrus_medica** **---------ttattaattattcattttgaaaacatt-------tctattctatagtaaaa**

**Citrus_polytrifolia** **---------ttattaattattcattttgaaaacatt-------tctattctatagtaaaa**

**Citrus_reticulata** **---------ttattaattattcattttgaaaacatt-------tctattctatagtaaaa**

**Citrus_sinensis** **attattcatttattaattattcattttgaaaacatt-------tctattctatagtaaaa**

**Fortunella_bawangica_1** **---------ttattaattattcattttgaaaacatttctattctctattctatagtaaaa**

**Fortunella_bawangica_2** **---------ttattaattattcattttgaaaacatttctattctctattctatagtaaaa**

**Fortunella_hindsii_1** **---------ttattaattattcattttgaaaacatttctattctctattctatagtaaaa**

**Fortunella_hindsii_2** **---------ttattaattattcattttgaaaacatttctattctctattctatagtaaaa**

**Fortunella_hindsii_3** **---------ttattaattattcattttgaaaacatttctattctctattctatagtaaaa**

**Fortunella_japonica_1** **---------ttattaattattcattttgaaaacatttctattctctattctatagtaaaa**

**Fortunella_japonica_2** **---------ttattaattattcattttgaaaacatttctattctctattctatagtaaaa**

**Fortunella_japonica_3** **---------ttattaattattcattttgaaaacatttctattctctattctatagtaaaa**

**Fortunella_japonica_4** **---------ttattaattattcattttgaaaacatttctattctctattctatagtaaaa**

**Fortunella_japonica_5** **---------ttattaattattcattttgaaaacatttctattctctattctatagtaaaa**

**Fortunella_japonica_6** **---------ttattaattattcattttgaaaacatttctattctctattctatagtaaaa**

**Fortunella_japonica_7** **---------ttattaattattcattttgaaaacatttctattctctattctatagtaaaa**

**Fortunella_japonica_8** **---------ttattaattattcattttgaaaacatttctattctctattctatagtaaaa**

**Fortunella_japonica_9** **---------ttattaattattcattttgaaaacatttctattctctattctatagtaaaa**

**Fortunella_japonica_10** **---------ttattaattattcattttgaaaacatttctattctctattctatagtaaaa**

**Fortunella_japonica_11** **---------ttattaattattcattttgaaaacatttctattctctattctatagtaaaa**

**Fortunella_japonica_12** **---------ttattaattattcattttgaaaacatttctattctctattctatagtaaaa**

**Fortunella_margarita_1** **---------ttattaattattcattttgaaaacatttctattctctattctatagtaaaa**

**Fortunella_margarita_2** **---------ttattaattattcattttgaaaacatttctattctctattctatagtaaaa**

**Fortunella_venosa_1** **---------ttattaattattcattttgaaaacatttctattctctattctatagtaaaa**

**Fortunella_venosa_2** **---------ttattaattattcattttgaaaacatttctattctctattctatagtaaaa**

**Fortunella_venosa_3** **---------ttattaattattcattttgaaaacatttctattctctattctatagtaaaa**

14290	14300	14310	14320	14330	14340

....|....|....|....|....|....|....|....|....|....|....|....|

**Atalantia_kwangtungens** **catagtagtagaaagagtaccgtgctgtgtctggacttcaaactttagctttaaccatgt**

**Citrus_reticulata_1** **catagtagtagaaagagtaccgtgctgtgtctggacttcaaactttagctttaaccatgt**

**Citrus_reticulata_2** **catagtagtagaaagagtaccgtgctgtgtctggacttcaaactttagctttaaccatgt**

**Citrus_trifoliata_3** **catagtagtagaaagagtaccgtgctgtgtctggacttcaaactttagctttaaccatgt**

**Citrus_aurantiifolia** **catagtagtagaaagagtaccgtgctgtgtctggacttcaaactttagctttaaccatgt**

**Citrus_aurantium** **catagtagtagaaagagtaccgtgctgtgtctggacttcaaactttagctttaaccatgt**

**Citrus_cavaleriei** **catagtagtagaaagagtaccgtgctgtgtctggacttcaaactttagctttaaccatgt**

**Citrus_hongheensis** **catagtagtagaaagagtaccgtgctgtgtctggacttcaaactttagctttaaccatgt**

**Citrus_limon** **catagtagtagaaagagtaccgtgctgtgtctggacttcaaactttagctttaaccatgt**

**Citrus_maxima** **catagtagtagaaagagtaccgtgctgtgtctggacttcaaactttagctttaaccatgt**

**Citrus_medica** **catagtagtagaaagagtaccgggctgtgtctggacttcaaacgttagctttaaccatgt**

**Citrus_polytrifolia** **catagtagtagaaagagtaccgtgctgtgtctggacttcaaactttagctttaaccatgt**

**Citrus_reticulata** **catagtagtagaaagagtaccgtgctgtgtctggacttcaaactttagctttaaccatgt**

**Citrus_sinensis** **catagtagtagaaagagtaccgtgctgtgtctggacttcaaactttagctttaaccatgt**

**Fortunella_bawangica_1** **catagtagtagaaagagtaccgtgctgtgtctggacttcaaactttagctttaaccatgt**

**Fortunella_bawangica_2** **catagtagtagaaagagtaccgtgctgtgtctggacttcaaactttagctttaaccatgt**

**Fortunella_hindsii_1** **catagtagtagaaagagtaccgtgctgtgtctggacttcaaactttagctttaaccatgt**

**Fortunella_hindsii_2** **catagtagtagaaagagtaccgtgctgtgtctggacttcaaactttagctttaaccatgt**

**Fortunella_hindsii_3** **catagtagtagaaagagtaccgtgctgtgtctggacttcaaactttagctttaaccatgt**

**Fortunella_japonica_1** **catagtagtagaaagagtaccgtgctgtgtctggacttcaaactttagctttaaccatgt**

**Fortunella_japonica_2** **catagtagtagaaagagtaccgtgctgtgtctggacttcaaactttagctttaaccatgt**

**Fortunella_japonica_3** **catagtagtagaaagagtaccgtgctgtgtctggacttcaaactttagctttaaccatgt**

**Fortunella_japonica_4** **catagtagtagaaagagtaccgtgctgtgtctggacttcaaactttagctttaaccatgt**

**Fortunella_japonica_5** **catagtagtagaaagagtaccgtgctgtgtctggacttcaaactttagctttaaccatgt**

**Fortunella_japonica_6** **catagtagtagaaagagtaccgtgctgtgtctggacttcaaactttagctttaaccatgt**

**Fortunella_japonica_7** **catagtagtagaaagagtaccgtgctgtgtctggacttcaaactttagctttaaccatgt**

**Fortunella_japonica_8** **catagtagtagaaagagtaccgtgctgtgtctggacttcaaactttagctttaaccatgt**

**Fortunella_japonica_9** **catagtagtagaaagagtaccgtgctgtgtctggacttcaaactttagctttaaccatgt**

**Fortunella_japonica_10** **catagtagtagaaagagtaccgtgctgtgtctggacttcaaactttagctttaaccatgt**

**Fortunella_japonica_11** **catagtagtagaaagagtaccgtgctgtgtctggacttcaaactttagctttaaccatgt**

**Fortunella_japonica_12** **catagtagtagaaagagtaccgtgctgtgtctggacttcaaactttagctttaaccatgt**

**Fortunella_margarita_1** **catagtagtagaaagagtaccgtgctgtgtctggacttcaaactttagctttaaccatgt**

**Fortunella_margarita_2** **catagtagtagaaagagtaccgtgctgtgtctggacttcaaactttagctttaaccatgt**

**Fortunella_venosa_1** **catagtagtagaaagagtaccgtgctgtgtctggacttcaaactttagctttaaccatgt**

**Fortunella_venosa_2** **catagtagtagaaagagtaccgtgctgtgtctggacttcaaactttagctttaaccatgt**

**Fortunella_venosa_3** **catagtagtagaaagagtaccgtgctgtgtctggacttcaaactttagctttaaccatgt**

14350	14360	14370	14380	14390	14400

....|....|....|....|....|....|....|....|....|....|....|....|

**Atalantia_kwangtungens** **taatggtcccacattattggtttggttgatagagaatcaaaatagatttaccaacaaatc**

**Citrus_reticulata_1** **taatggtcccacattattggtttggttgatagagaatcaaaatagatttaccaacaaatc**

**Citrus_reticulata_2** **taatggtcccacattattggtttggttgatagagaatcaaaatagatttaccaacaaatc**

**Citrus_trifoliata_3** **taatggtcccacattattggtttggttgatagagaatcaaaatagatttaccaacaaatc**

**Citrus_aurantiifolia** **taatggtcccacattattggtttggttgatagagaatcaaaatggatttaccaacaaatc**

**Citrus_aurantium** **taatggtcccacattattggtttggttgatagagaatcaaaatagatttaccaacaaatc**

**Citrus_cavaleriei** **taatggtcccacattattggtttggttgatagagaatcaaaatagatttaccaacaaatc**

**Citrus_hongheensis** **taatggtcccacattattggtttggttgatagagaatcaaaatagatttaccaacaaatc**

**Citrus_limon** **taatggtcccacattattggtttggttgatagagaatcaaaatagatttaccaacaaatc**

**Citrus_maxima** **taatggtcccacattattggtttggttaatagagaatcaaaatagatttaccaacaaatc**

**Citrus_medica** **taatggtcccacattattggtttggttgatagagaatcaaaatagatttaccaacaaatc**

**Citrus_polytrifolia** **taatggtcccacattattggtttggttgatagagaatcaaaatagatttaccaacaaatc**

**Citrus_reticulata** **taatggtcccacattattggtttggttgatagagaatcaaaatagatttaccaacaaatc**

**Citrus_sinensis** **taatggtcccacattattggtttggttgatagagaatcaaaatagatttaccaacaaatc**

**Fortunella_bawangica_1** **taatggtcccacattattggtttggttgatagagaatcaaaatagatttaccaacaaatc**

**Fortunella_bawangica_2** **taatggtcccacattattggtttggttgatagagaatcaaaatagatttaccaacaaatc**

**Fortunella_hindsii_1** **taatggtcccacattattggtttggttgatagagaatcaaaatagatttaccaacaaatc**

**Fortunella_hindsii_2** **taatggtcccacattattggtttggttgatagagaatcaaaatagatttaccaacaaatc**

**Fortunella_hindsii_3** **taatggtcccacattattggtttggttgatagagaatcaaaatagatttaccaacaaatc**

**Fortunella_japonica_1** **taatgttcccacattattggtttggttgatagagaatcaaaatagatttaccaacaaatc**

**Fortunella_japonica_2** **taatgttcccacattattggtttggttgatagagaatcaaaatagatttaccaacaaatc**

**Fortunella_japonica_3** **taatgttcccacattattggtttggttgatagagaatcaaaatagatttaccaacaaatc**

**Fortunella_japonica_4** **taatgttcccacattattggtttggttgatagagaatcaaaatagatttaccaacaaatc**

**Fortunella_japonica_5** **taatgttcccacattattggtttggttgatagagaatcaaaatagatttaccaacaaatc**

**Fortunella_japonica_6** **taatgttcccacattattggtttggttgatagagaatcaaaatagatttaccaacaaatc**

**Fortunella_japonica_7** **taatgttcccacattattggtttggttgatagagaatcaaaatagatttaccaacaaatc**

**Fortunella_japonica_8** **taatgttcccacattattggtttggttgatagagaatcaaaatagatttaccaacaaatc**

**Fortunella_japonica_9** **taatgttcccacattattggtttggttgatagagaatcaaaatagatttaccaacaaatc**

**Fortunella_japonica_10** **taatggtcccacattattggtttggttgatagagaatcaaaatagatttaccaacaaatc**

**Fortunella_japonica_11** **taatgttcccacattattggtttggttgatagagaatcaaaatagatttaccaacaaatc**

**Fortunella_japonica_12** **taatgttcccacattattggtttggttgatagagaatcaaaatagatttaccaacaaatc**

**Fortunella_margarita_1** **taatggtcccacattattggtttggttgatagagaatcaaaatagatttaccaacaaatc**

**Fortunella_margarita_2** **taatggtcccacattattggtttggttgatagagaatcaaaatagatttaccaacaaatc**

**Fortunella_venosa_1** **taatggtcccacattattggtttggttgatagagaatcaaaatagatttaccaacaaatc**

**Fortunella_venosa_2** **taatggtcccacattattggtttggttgatagagaatcaaaatagatttaccaacaaatc**

**Fortunella_venosa_3** **taatggtcccacattattggtttggttgatagagaatcaaaatagatttaccaacaaatc**

14410	14420	14430	14440	14450	14460

....|....|....|....|....|....|....|....|....|....|....|....|

**Atalantia_kwangtungens** **acgaaatgctatggttcttaaatatgatttgaaatttattcagaagtaattcgcggaatc**

**Citrus_reticulata_1** **acgaaatgctatggttcttaaatatgatttgaaatttattcagaagtaattcgcggaatc**

**Citrus_reticulata_2** **acgaaatgctatggttcttaaatatgatttgaaatttattcagaagtaattcgcggaatc**

**Citrus_trifoliata_3** **acgaaatgctatggttcttaaatatgatttgcaatttattcagaagtaattcgcggaatc**

**Citrus_aurantiifolia** **acgaaatgctatggttcttaaatatgatttgaaatttattcagaagtaattcgcggaatc**

**Citrus_aurantium** **acgaaatgctatggttcttaaatatgatttgaaatttattcagaagtaattcgcggaatc**

**Citrus_cavaleriei** **acgaaatgctatggttcttaaatatgatttgaaatttattcagaagtaattcgcggaatc**

**Citrus_hongheensis** **acgaaatgctatggttcttaaatatgatttgaaatttattcagaagtaattcgcggaatc**

**Citrus_limon** **acgaaatgctatggttcttaaatatgatttgaaatttattcagaagtaattcgcggaatc**

**Citrus_maxima** **acgaaatgctatggttcttaaatatgatttgaaatttattcagaagtaattcgcggaatc**

**Citrus_medica** **acgaaatgctatggttcttaaatatgatttgaaatttattcagaagtaattcgcggaatc**

**Citrus_polytrifolia** **acgaaatgctatggttcttaaatatgatttgcaatttattcagaagtaattcgcggaatc**

**Citrus_reticulata** **acgaaatgctatggttcttaaatatgatttgaaatttattcagaagtaattcgcggaatc**

**Citrus_sinensis** **acgaaatgctatggttcttaaatatgatttgaaatttattcagaagtaattcgcggaatc**

**Fortunella_bawangica_1** **acgaaatgctatggttcttaaatatgatttgaaatttattcagaagtaattcgcggaatc**

**Fortunella_bawangica_2** **acgaaatgctatggttcttaaatatgatttgaaatttattcagaagtaattcgcggaatc**

**Fortunella_hindsii_1** **acgaaatgctatggttcttaaatatgatttgaaatttattcagaagtaattcgcggaatc**

**Fortunella_hindsii_2** **acgaaatgctatggttcttaaatatgatttgaaatttattcagaagtaattcgcggaatc**

**Fortunella_hindsii_3** **acgaaatgctatggttcttaaatatgatttgaaatttattcagaagtaattcgcggaatc**

**Fortunella_japonica_1** **acgaaatgctatggttcttaaatatgatttgaaatttattcagaagtaattcgcggaatc**

**Fortunella_japonica_2** **acgaaatgctatggttcttaaatatgatttgaaatttattcagaagtaattcgcggaatc**

**Fortunella_japonica_3** **acgaaatgctatggttcttaaatatgatttgaaatttattcagaagtaattcgcggaatc**

**Fortunella_japonica_4** **acgaaatgctatggttcttaaatatgatttgaaatttattcagaagtaattcgcggaatc**

**Fortunella_japonica_5** **acgaaatgctatggttcttaaatatgatttgaaatttattcagaagtaattcgcggaatc**

**Fortunella_japonica_6** **acgaaatgctatggttcttaaatatgatttgaaatttattcagaagtaattcgcggaatc**

**Fortunella_japonica_7** **acgaaatgctatggttcttaaatatgatttgaaatttattcagaagtaattcgcggaatc**

**Fortunella_japonica_8** **acgaaatgctatggttcttaaatatgatttgaaatttattcagaagtaattcgcggaatc**

**Fortunella_japonica_9** **acgaaatgctatggttcttaaatatgatttgaaatttattcagaagtaattcgcggaatc**

**Fortunella_japonica_10** **acgaaatgctatggttcttaaatatgatttgaaatttattcagaagtaattcgcggaatc**

**Fortunella_japonica_11** **acgaaatgctatggttcttaaatatgatttgaaatttattcagaagtaattcgcggaatc**

**Fortunella_japonica_12** **acgaaatgctatggttcttaaatatgatttgaaatttattcagaagtaattcgcggaatc**

**Fortunella_margarita_1** **acgaaatgctatggttcttaaatatgatttgaaatttattcagaagtaattcgcggaatc**

**Fortunella_margarita_2** **acgaaatgctatggttcttaaatatgatttgaaatttattcagaagtaattcgcggaatc**

**Fortunella_venosa_1** **acgaaatgctatggttcttaaatatgatttgaaatttattcagaagtaattcgcggaatc**

**Fortunella_venosa_2** **acgaaatgctatggttcttaaatatgatttgaaatttattcagaagtaattcgcggaatc**

**Fortunella_venosa_3** **acgaaatgctatggttcttaaatatgatttgaaatttattcagaagtaattcgcggaatc**

14470	14480	14490	14500	14510	14520

....|....|....|....|....|....|....|....|....|....|....|....|

**Atalantia_kwangtungens** **atgcacccttttccctttggtcctagttataacgaaaaaaagtgcagctggttgggtcca**

**Citrus_reticulata_1** **atgcacccttttccctttggtcctagttataacgaaaaaaagtgcagctggttgggtcca**

**Citrus_reticulata_2** **atgcacccttttccctttggtcctagttataacgaaaaaaagtgcagctggttgggtcca**

**Citrus_trifoliata_3** **atgcacccttttccctttggtcctagttataacgaaaaaaagtgcagctggttgggtcca**

**Citrus_aurantiifolia** **atgcacccttttccctttggtcctagttataacgaaaaaaagtgcagctggttgggtcca**

**Citrus_aurantium** **atgcacccttttccctttggtcctagttataacgaaaaaaagtgcagctggttgggtcca**

**Citrus_cavaleriei** **atgcacccttttccctttggtcctagttataacgaaaaaaagtgcagctggttgggtcca**

**Citrus_hongheensis** **atgcacccttttccctttggtcctagttataacgaaaaaaagtgcagctggttgggtcca**

**Citrus_limon** **atgcacccttttccctttggtcctagttataacgaaaaaaagtgcagctggttgggtcca**

**Citrus_maxima** **atgcacccttttccctttggtcctagttataacgaaaaaaagtgcagctggttgggtcca**

**Citrus_medica** **atgcacccttttccctttggtcctagttataacgaaaaaaagtgcagctggttgggtcca**

**Citrus_polytrifolia** **atgcacccttttccctttggtcctagttataacgaaaaaaagtgcagctggttgggtcca**

**Citrus_reticulata** **atgcacccttttccctttggtcctagttataacgaaaaaaagtgcagctggttgggtcca**

**Citrus_sinensis** **atgcacccttttccctttggtcctagttataacgaaaaaaagtgcagctggttgggtcca**

**Fortunella_bawangica_1** **atgcacccttttccctttggtcctagttataacgaaaaaaagtgcagctggttgggtcca**

**Fortunella_bawangica_2** **atgcacccttttccctttggtcctagttataacgaaaaaaagtgcagctggttgggtcca**

**Fortunella_hindsii_1** **atgcacccttttccctttggtcctagttataacgaaaaaaagtgcagctggttgggtcca**

**Fortunella_hindsii_2** **atgcacccttttccctttggtcctagttataacgaaaaaaagtgcagctggttgggtcca**

**Fortunella_hindsii_3** **atgcacccttttccctttggtcctagttataacgaaaaaaagtgcagctggttgggtcca**

**Fortunella_japonica_1** **atgcacccttttccctttggtcctagttataacgaaaaaaagtgcagctggttgggtcca**

**Fortunella_japonica_2** **atgcacccttttccctttggtcctagttataacgaaaaaaagtgcagctggttgggtcca**

**Fortunella_japonica_3** **atgcacccttttccctttggtcctagttataacgaaaaaaagtgcagctggttgggtcca**

**Fortunella_japonica_4** **atgcacccttttccctttggtcctagttataacgaaaaaaagtgcagctggttgggtcca**

**Fortunella_japonica_5** **atgcacccttttccctttggtcctagttataacgaaaaaaagtgcagctggttgggtcca**

**Fortunella_japonica_6** **atgcacccttttccctttggtcctagttataacgaaaaaaagtgcagctggttgggtcca**

**Fortunella_japonica_7** **atgcacccttttccctttggtcctagttataacgaaaaaaagtgcagctggttgggtcca**

**Fortunella_japonica_8** **atgcacccttttccctttggtcctagttataacgaaaaaaagtgcagctggttgggtcca**

**Fortunella_japonica_9** **atgcacccttttccctttggtcctagttataacgaaaaaaagtgcagctggttgggtcca**

**Fortunella_japonica_10** **atgcacccttttccctttggtcctagttataacgaaaaaaagtgcagctggttgggtcca**

**Fortunella_japonica_11** **atgcacccttttccctttggtcctagttataacgaaaaaaagtgcagctggttgggtcca**

**Fortunella_japonica_12** **atgcacccttttccctttggtcctagttataacgaaaaaaagtgcagctggttgggtcca**

**Fortunella_margarita_1** **atgcacccttttccctttggtcctagttataacgaaaaaaagtgcagctggttgggtcca**

**Fortunella_margarita_2** **atgcacccttttccctttggtcctagttataacgaaaaaaagtgcagctggttgggtcca**

**Fortunella_venosa_1** **atgcacccttttccctttggtcctagttataacgaaaaaaagtgcagctggttgggtcca**

**Fortunella_venosa_2** **atgcacccttttccctttggtcctagttataacgaaaaaaagtgcagctggttgggtcca**

**Fortunella_venosa_3** **atgcacccttttccctttggtcctagttataacgaaaaaaagtgcagctggttgggtcca**

14530	14540	14550	14560	14570	14580

....|....|....|....|....|....|....|....|....|....|....|....|

**Atalantia_kwangtungens** **gcctattcttgaaataaacaactcgcacacactccctttccaaaaaagatcaatacacca**

**Citrus_reticulata_1** **gcctattcttgaaataaacaactcgcacacactccctttccaaaaaagatcaatacacca**

**Citrus_reticulata_2** **gcctattcttgaaataaacaactcgcacacactccctttccaaaaaagatcaatacacca**

**Citrus_trifoliata_3** **gcctattcttgaaataaacaactcgcacacactccctttccaaaaaagatcaatacacca**

**Citrus_aurantiifolia** **gcctattcttgaaataaacaactcgcacacactccctttccaaaaaagatcaatacacca**

**Citrus_aurantium** **gcctattcttgaaataaacaactcgcacacactccctttccaaaaaagatcaatacacca**

**Citrus_cavaleriei** **gcctattcttgaaataaacaactcgcacacactccctttccaaaaaagatcaatacacca**

**Citrus_hongheensis** **gcctattcttgaaataaacaactcgcacacactccctttccaaaaaagatcaatacacca**

**Citrus_limon** **gcctattcttgaaataaacaactcgcacacactccctttccaaaaaagatcaatacacca**

**Citrus_maxima** **gcctattcttgaaataaacaactcgcacacactccctttccaaaaaagatcaatacacca**

**Citrus_medica** **gcctattcttgaaataaacaactcgcacacactccctttccaaaaaagatcaatacacca**

**Citrus_polytrifolia** **gcctattcttgaaataaacaactcgcacacactccctttccaaaaaagatcaatacacca**

**Citrus_reticulata** **gcctattcttgaaataaacaactcgcacacactccctttccaaaaaagatcaatacacca**

**Citrus_sinensis** **gcctattcttgaaataaacaactcgcacacactccctttccaaaaaagatcaatacacca**

**Fortunella_bawangica_1** **gcctattcttgaaataaacaactcgcacacactccctttccaaaaaagatcaatacacca**

**Fortunella_bawangica_2** **gcctattcttgaaataaacaactcgcacacactccctttccaaaaaagatcaatacacca**

**Fortunella_hindsii_1** **gcctattcttgaaataaacaactcgcacacactccctttccaaaaaagatcaatacacca**

**Fortunella_hindsii_2** **gcctattcttgaaataaacaactcgcacacactccctttccaaaaaagatcaatacacca**

**Fortunella_hindsii_3** **gcctattcttgaaataaacaactcgcacacactccctttccaaaaaagatcaatacacca**

**Fortunella_japonica_1** **gcctattcttgaaataaacaactcgcacacactccctttccaaaaaagatcaatacacca**

**Fortunella_japonica_2** **gcctattcttgaaataaacaactcgcacacactccctttccaaaaaagatcaatacacca**

**Fortunella_japonica_3** **gcctattcttgaaataaacaactcgcacacactccctttccaaaaaagatcaatacacca**

**Fortunella_japonica_4** **gcctattcttgaaataaacaactcgcacacactccctttccaaaaaagatcaatacacca**

**Fortunella_japonica_5** **gcctattcttgaaataaacaactcgcacacactccctttccaaaaaagatcaatacacca**

**Fortunella_japonica_6** **gcctattcttgaaataaacaactcgcacacactccctttccaaaaaagatcaatacacca**

**Fortunella_japonica_7** **gcctattcttgaaataaacaactcgcacacactccctttccaaaaaagatcaatacacca**

**Fortunella_japonica_8** **gcctattcttgaaataaacaactcgcacacactccctttccaaaaaagatcaatacacca**

**Fortunella_japonica_9** **gcctattcttgaaataaacaactcgcacacactccctttccaaaaaagatcaatacacca**

**Fortunella_japonica_10** **gcctattcttgaaataaacaactcgcacacactccctttccaaaaaagatcaatacacca**

**Fortunella_japonica_11** **gcctattcttgaaataaacaactcgcacacactccctttccaaaaaagatcaatacacca**

**Fortunella_japonica_12** **gcctattcttgaaataaacaactcgcacacactccctttccaaaaaagatcaatacacca**

**Fortunella_margarita_1** **gcctattcttgaaataaacaactcgcacacactccctttccaaaaaagatcaatacacca**

**Fortunella_margarita_2** **gcctattcttgaaataaacaactcgcacacactccctttccaaaaaagatcaatacacca**

**Fortunella_venosa_1** **gcctattcttgaaataaacaactcgcacacactccctttccaaaaaagatcaatacacca**

**Fortunella_venosa_2** **gcctattcttgaaataaacaactcgcacacactccctttccaaaaaagatcaatacacca**

**Fortunella_venosa_3** **gcctattcttgaaataaacaactcgcacacactccctttccaaaaaagatcaatacacca**

14590	14600	14610	14620	14630	14640

....|....|....|....|....|....|....|....|....|....|....|....|

**Atalantia_kwangtungens** **agcactacacttagatttattggatttgttgctaaaatatcggtattaagcccgaaactc**

**Citrus_reticulata_1** **agcactacacttagatttattggatttgttgctaaaatatcggtattaagcccgaaactc**

**Citrus_reticulata_2** **agcactacacttagatttattggatttgttgctaaaatatcggtattaagcccgaaactc**

**Citrus_trifoliata_3** **agcactacacttagatttattggatttgttgctaaaatatcggtattaagcccgaaactc**

**Citrus_aurantiifolia** **agcactacacttagatttattggatttgttgctaaaatatcggtattaagcccgaaactc**

**Citrus_aurantium** **agcactacacttagatttattggatttgttgctaaaatatcggtattaagcccgaaactc**

**Citrus_cavaleriei** **agcactacacttagatttattggatttgttgctaaaatatcggtattaagcccgaaactc**

**Citrus_hongheensis** **agcactacacttagatttattggatttgttgctaaaatatcggtattaagcccgaaactc**

**Citrus_limon** **agcactacacttagatttattggatttgttgctaaaatatcggtattaagcccgaaactc**

**Citrus_maxima** **agcactacacttagatttattggatttgttgctaaaatatcggtattaagcccgaaactc**

**Citrus_medica** **agtactacacttagatttattggatttgttgctaaaatatcggtattaagcccgaaactc**

**Citrus_polytrifolia** **agcactacacttagatttattggatttgttgctaaaatatcggtattaagcccgaaactc**

**Citrus_reticulata** **agcactacacttagatttattggatttgttgctaaaatatcggtattaagcccgaaactc**

**Citrus_sinensis** **agcactacacttagatttattggatttgttgctaaaatatcggtattaagcccgaaactc**

**Fortunella_bawangica_1** **agcactacacttagatttattggatttgttgctaaaatatcggtattaagcccgaaactc**

**Fortunella_bawangica_2** **agcactacacttagatttattggatttgttgctaaaatatcggtattaagcccgaaactc**

**Fortunella_hindsii_1** **agcactacacttagatttattggatttgttgctaaaatatcggtattaagcccgaaactc**

**Fortunella_hindsii_2** **agcactacacttagatttattggatttgttgctaaaatatcggtattaagcccgaaactc**

**Fortunella_hindsii_3** **agcactacacttagatttattggatttgttgctaaaatatcggtattaagcccgaaactc**

**Fortunella_japonica_1** **agcactacacttagatttattggatttgttgctaaaatatcggtattaagcccgaaactc**

**Fortunella_japonica_2** **agcactacacttagatttattggatttgttgctaaaatatcggtattaagcccgaaactc**

**Fortunella_japonica_3** **agcactacacttagatttattggatttgttgctaaaatatcggtattaagcccgaaactc**

**Fortunella_japonica_4** **agcactacacttagatttattggatttgttgctaaaatatcggtattaagcccgaaactc**

**Fortunella_japonica_5** **agcactacacttagatttattggatttgttgctaaaatatcggtattaagcccgaaactc**

**Fortunella_japonica_6** **agcactacacttagatttattggatttgttgctaaaatatcggtattaagcccgaaactc**

**Fortunella_japonica_7** **agcactacacttagatttattggatttgttgctaaaatatcggtattaagcccgaaactc**

**Fortunella_japonica_8** **agcactacacttagatttattggatttgttgctaaaatatcggtattaagcccgaaactc**

**Fortunella_japonica_9** **agcactacacttagatttattggatttgttgctaaaatatcggtattaagcccgaaactc**

**Fortunella_japonica_10** **agcactacacttagatttattggatttgttgctaaaatatcggtattaagcccgaaactc**

**Fortunella_japonica_11** **agcactacacttagatttattggatttgttgctaaaatatcggtattaagcccgaaactc**

**Fortunella_japonica_12** **agcactacacttagatttattggatttgttgctaaaatatcggtattaagcccgaaactc**

**Fortunella_margarita_1** **agcactacacttagatttattggatttgttgctaaaatatcggtattaagcccgaaactc**

**Fortunella_margarita_2** **agcactacacttagatttattggatttgttgctaaaatatcggtattaagcccgaaactc**

**Fortunella_venosa_1** **agcactacacttagatttattggatttgttgctaaaatatcggtattaagcccgaaactc**

**Fortunella_venosa_2** **agcactacacttagatttattggatttgttgctaaaatatcggtattaagcccgaaactc**

**Fortunella_venosa_3** **agcactacacttagatttattggatttgttgctaaaatatcggtattaagcccgaaactc**

14650	14660	14670	14680	14690	14700

....|....|....|....|....|....|....|....|....|....|....|....|

**Atalantia_kwangtungens** **ccggcgaatggccagttaaccaaagaaacgaaagaatcggttacatttttcatatgatct**

**Citrus_reticulata_1** **ccggcgaatggccagttaaccaaagaaacgaaagaatcggttacatttttcatatgatct**

**Citrus_reticulata_2** **ccggcgaatggccagttaaccaaagaaacgaaagaatcggttacatttttcatatgatct**

**Citrus_trifoliata_3** **ccggcgaatggccagttaaccaaagaaacgaaagaatcggttacatttttcatatgatct**

**Citrus_aurantiifolia** **ccggcgaatggccagttaaccaaagaaacgaaagaatcggttacatttttcatatgatct**

**Citrus_aurantium** **ccggcgaatggccagttaaccaaagaaacgaaagaatcggttacatttttcatatgatct**

**Citrus_cavaleriei** **ccggcgaatggccagttaaccaaagaaacgaaagaatcggttacatttttcatatgatct**

**Citrus_hongheensis** **ccggcgaatggccagttaaccaaagaaacgaaagaatcggttacatttttcatatgatct**

**Citrus_limon** **ccggcgaatggccagttaaccaaagaaacgaaagaatcggttacatttttcatatgatct**

**Citrus_maxima** **ccggcgaatggccagttaaccaaagaaacgaaagaatcggttacatttttcatatgatct**

**Citrus_medica** **ccggcgaatggccagttaaccaaagaaacgaaagaatcggttacatttttcatatgatct**

**Citrus_polytrifolia** **ccggcgaatggccagttaaccaaagaaacgaaagaatcggttacatttttcatatgatct**

**Citrus_reticulata** **ccggcgaatggccagttaaccaaagaaacgaaagaatcggttacatttttcatatgatct**

**Citrus_sinensis** **ccggcgaatggccagttaaccaaagaaacgaaagaatcggttacatttttcatatgatct**

**Fortunella_bawangica_1** **ccggcgaatggccagttaaccaaagaaacgaaagaatcggttacatttttcatatgatct**

**Fortunella_bawangica_2** **ccggcgaatggccagttaaccaaagaaacgaaagaatcggttacatttttcatatgatct**

**Fortunella_hindsii_1** **ccggcgaatggccagttaaccaaagaaacgaaagaatcggttacatttttcatatgatct**

**Fortunella_hindsii_2** **ccggcgaatggccagttaaccaaagaaacgaaagaatcggttacatttttcatatgatct**

**Fortunella_hindsii_3** **ccggcgaatggccagttaaccaaagaaacgaaagaatcggttacatttttcatatgatct**

**Fortunella_japonica_1** **ccggcgaatggccagttaaccaaagaaacgaaagaatcggttacatttttcatatgatct**

**Fortunella_japonica_2** **ccggcgaatggccagttaaccaaagaaacgaaagaatcggttacatttttcatatgatct**

**Fortunella_japonica_3** **ccggcgaatggccagttaaccaaagaaacgaaagaatcggttacatttttcatatgatct**

**Fortunella_japonica_4** **ccggcgaatggccagttaaccaaagaaacgaaagaatcggttacatttttcatatgatct**

**Fortunella_japonica_5** **ccggcgaatggccagttaaccaaagaaacgaaagaatcggttacatttttcatatgatct**

**Fortunella_japonica_6** **ccggcgaatggccagttaaccaaagaaacgaaagaatcggttacatttttcatatgatct**

**Fortunella_japonica_7** **ccggcgaatggccagttaaccaaagaaacgaaagaatcggttacatttttcatatgatct**

**Fortunella_japonica_8** **ccggcgaatggccagttaaccaaagaaacgaaagaatcggttacatttttcatatgatct**

**Fortunella_japonica_9** **ccggcgaatggccagttaaccaaagaaacgaaagaatcggttacatttttcatatgatct**

**Fortunella_japonica_10** **ccggcgaatggccagttaaccaaagaaacgaaagaatcggttacatttttcatatgatct**

**Fortunella_japonica_11** **ccggcgaatggccagttaaccaaagaaacgaaagaatcggttacatttttcatatgatct**

**Fortunella_japonica_12** **ccggcgaatggccagttaaccaaagaaacgaaagaatcggttacatttttcatatgatct**

**Fortunella_margarita_1** **ccggcgaatggccagttaaccaaagaaacgaaagaatcggttacatttttcatatgatct**

**Fortunella_margarita_2** **ccggcgaatggccagttaaccaaagaaacgaaagaatcggttacatttttcatatgatct**

**Fortunella_venosa_1** **ccggcgaatggccagttaaccaaagaaacgaaagaatcggttacatttttcatatgatct**

**Fortunella_venosa_2** **ccggcgaatggccagttaaccaaagaaacgaaagaatcggttacatttttcatatgatct**

**Fortunella_venosa_3** **ccggcgaatggccagttaaccaaagaaacgaaagaatcggttacatttttcatatgatct**

14710	14720	14730	14740	14750	14760

....|....|....|....|....|....|....|....|....|....|....|....|

**Atalantia_kwangtungens** **cctcttttagatagactaaa--aaaaaaaaaataagtaaatcgcccttcctatt------**

**Citrus_reticulata_1** **cctcttttagatagactaaa--aaaaaaaaatgaactgcgttctttttttttt--ttttt**

**Citrus_reticulata_2** **cctcttttagatagactaaa--aaaaaaaaatgaactgcgttctttttttttt--ttttt**

**Citrus_trifoliata_3** **cctcttttagatagactaaa--aaaaaaa--agaactgcgttctttttttttt--tt-tt**

**Citrus_aurantiifolia** **cctcttttag-------------------------------------------------t**

**Citrus_aurantium** **cctcttttagatagactaaa--aaaaaaaaaagaacgcagttctttttt------ttttt**

**Citrus_cavaleriei** **cctcttttagatagactaaa--aaaaaaaaatgaactgcgttc---ttttttt--ttttt**

**Citrus_hongheensis** **cctcttttagatagactaaaaaaaaaaaaaaagaacgcagttctttttttttttcttttt**

**Citrus_limon** **cctcttttagatagactaaa--aaaaaaaaaagaacgcagttctttttt------ttttt**

**Citrus_maxima** **cctcttttagatagactaaa--aaaaaaaaaagaacgcagttctttttt------ttttt**

**Citrus_medica** **cctcttttagatagactaaa--aaaaaa--------------------------------**

**Citrus_polytrifolia** **cctcttttagatagactaaa--aaaaaaa--agaactgcgttctttttttttt--ttagt**

**Citrus_reticulata** **cctcttttagatagactaaa--aaaaaaaaatgaactgcgttctttttttttt--ttttt**

**Citrus_sinensis** **cctcttttagatagactaaa--aaaaaaaaaagaacgcagttctttttt------ttttt**

**Fortunella_bawangica_1** **cctcttttagatagactaaa--aaaaaaa-------------------------------**

**Fortunella_bawangica_2** **cctcttttagatagactaaa--aaaaaaa-------------------------------**

**Fortunella_hindsii_1** **cctcttttagatagactaaa--aaaaaaa-------------------------------**

**Fortunella_hindsii_2** **cctcttttagatagactaaa--aaaaaaa-------------------------------**

**Fortunella_hindsii_3** **cctcttttagatagact-aa--aaaaaaa-------------------------------**

**Fortunella_japonica_1** **cctcttttagatagactaaa--aaaaaaa-------------------------------**

**Fortunella_japonica_2** **cctcttttagatagactaaa--aaaaaaa-------------------------------**

**Fortunella_japonica_3** **cctcttttagatagactaaa--aaaaaaa-------------------------------**

**Fortunella_japonica_4** **cctcttttagatagactaaa--aaaaaaa-------------------------------**

**Fortunella_japonica_5** **cctcttttagatagactaaa--aaaaaaa-------------------------------**

**Fortunella_japonica_6** **cctcttttagatagactaaa--aaaaaaa-------------------------------**

**Fortunella_japonica_7** **cctcttttagatagactaaa--aaaaaaa-------------------------------**

**Fortunella_japonica_8** **cctcttttagatagactaaa--aaaaaaa-------------------------------**

**Fortunella_japonica_9** **cctcttttagatagactaaa--aaaaaaa-------------------------------**

**Fortunella_japonica_10** **cctcttttagatagactaaa--aaaaaaa-------------------------------**

**Fortunella_japonica_11** **cctcttttagatagactaaa--aaaaaaa-------------------------------**

**Fortunella_japonica_12** **cctcttttagatagactaaa--aaaaaaa-------------------------------**

**Fortunella_margarita_1** **cctcttttagatagactaaa--aaaaaaa-------------------------------**

**Fortunella_margarita_2** **cctcttttagatagactaaa--aaaaaaa-------------------------------**

**Fortunella_venosa_1** **cctcttttagatagactaaa--aaaaaaa-------------------------------**

**Fortunella_venosa_2** **cctcttttagatagactaaa--aaaaaaa-------------------------------**

**Fortunella_venosa_3** **cctcttttagatagactaaa--aaaaaaa-------------------------------**

14770	14780	14790	14800	14810	14820

....|....|....|....|....|....|....|....|....|....|....|....|

**Atalantia_kwangtungens** **------------------------------------------------------------**

**Citrus_reticulata_1** **ctacgtaatatatattta-----------------tttaatttatttgtttttttagtaa**

**Citrus_reticulata_2** **ctacgtaatatatattta-----------------tttaatttatttgtttttttagtaa**

**Citrus_trifoliata_3** **ctacgtaatatatatttatttaattaatttgtttttttaattaatttgtttttttagtaa**

**Citrus_aurantiifolia** **ctacgtaatatatattta-----------------tttaatttatttgtttttttagtaa**

**Citrus_aurantium** **ctacgtaatatatattta-----------------tttaatttatttgtttttttagtaa**

**Citrus_cavaleriei** **ctacgtaatatatattta-----------------tttaatttatttgtttttttagtaa**

**Citrus_hongheensis** **ctacgtaatatatattta-----------------tttaatttatttgtttttttagtaa**

**Citrus_limon** **ctacgtaatatatattta-----------------tttaatttatttgtttttttagtaa**

**Citrus_maxima** **ctacgtaatatatattta-----------------tttaatttatttgtttttttagtaa**

**Citrus_medica** **------------------------------------------------------------**

**Citrus_polytrifolia** **ctacgtaatatatattta-----------------tttaattaatttgtttttttagtaa**

**Citrus_reticulata** **ctacgtaatatatattta-----------------tttaatttatttgtttttttagtaa**

**Citrus_sinensis** **ctacgtaatatatattta-----------------tttaatttatttgtttttttagtaa**

**Fortunella_bawangica_1** **------------------------------------------------------------**

**Fortunella_bawangica_2** **------------------------------------------------------------**

**Fortunella_hindsii_1** **------------------------------------------------------------**

**Fortunella_hindsii_2** **------------------------------------------------------------**

**Fortunella_hindsii_3** **------------------------------------------------------------**

**Fortunella_japonica_1** **------------------------------------------------------------**

**Fortunella_japonica_2** **------------------------------------------------------------**

**Fortunella_japonica_3** **------------------------------------------------------------**

**Fortunella_japonica_4** **------------------------------------------------------------**

**Fortunella_japonica_5** **------------------------------------------------------------**

**Fortunella_japonica_6** **------------------------------------------------------------**

**Fortunella_japonica_7** **------------------------------------------------------------**

**Fortunella_japonica_8** **------------------------------------------------------------**

**Fortunella_japonica_9** **------------------------------------------------------------**

**Fortunella_japonica_10** **------------------------------------------------------------**

**Fortunella_japonica_11** **------------------------------------------------------------**

**Fortunella_japonica_12** **------------------------------------------------------------**

**Fortunella_margarita_1** **------------------------------------------------------------**

**Fortunella_margarita_2** **------------------------------------------------------------**

**Fortunella_venosa_1** **------------------------------------------------------------**

**Fortunella_venosa_2** **------------------------------------------------------------**

**Fortunella_venosa_3** **------------------------------------------------------------**

14830	14840	14850	14860	14870	14880

....|....|....|....|....|....|....|....|....|....|....|....|

**Atalantia_kwangtungens** **--------tctaggattaaacaaaaggattcgattttgaaatcctttctttgaattggca**

**Citrus_reticulata_1** **tttacctatttcgaaacagggtcaaaaattcgatttcgaaatcctttctttgaattggca**

**Citrus_reticulata_2** **tttacctatttcgaaacagggtcaaaaattcgatttcgaaatcctttctttgaattggca**

**Citrus_trifoliata_3** **tttacctatttcgaaacagggtcaaaaattcgatttcgaaatcctttctttgaattgtca**

**Citrus_aurantiifolia** **tttacctatttcgaaacagggtcaaaaattcgatttcgaaatcctttctttgaattggca**

**Citrus_aurantium** **tttacctatttcgaaacagggtcaaaaattagatttcgaaatcctttctttgaattggca**

**Citrus_cavaleriei** **tttacctatttcgaaacagggtcaaaaattcgatttcgaaatcctttctttgaattggca**

**Citrus_hongheensis** **tttacctatttcgaaacagggtcaaaaattcgatttcgaaatcctttctttgaattggca**

**Citrus_limon** **tttacctatttcgaaacagggtcaaaaattagatttcgaaatcctttctttgaattggca**

**Citrus_maxima** **tttacctatttcgaaacagggtcaaaaattcgatttcgaaatcctttctttgaattggca**

**Citrus_medica** **------------------------------------------------------------**

**Citrus_polytrifolia** **tttacctatttcgaaacagggtcaaaaattcgatttcgaaatcctttctttgaattgtca**

**Citrus_reticulata** **tttacctatttcgaaacagggtcaaaaattcgatttcgaaatcctttctttgaattggca**

**Citrus_sinensis** **tttacctatttcgaaacagggtcaaaaattcgatttcgaaatcctttctttgaattggca**

**Fortunella_bawangica_1** **------------------------------------------------------------**

**Fortunella_bawangica_2** **------------------------------------------------------------**

**Fortunella_hindsii_1** **------------------------------------------------------------**

**Fortunella_hindsii_2** **------------------------------------------------------------**

**Fortunella_hindsii_3** **------------------------------------------------------------**

**Fortunella_japonica_1** **------------------------------------------------------------**

**Fortunella_japonica_2** **------------------------------------------------------------**

**Fortunella_japonica_3** **------------------------------------------------------------**

**Fortunella_japonica_4** **------------------------------------------------------------**

**Fortunella_japonica_5** **------------------------------------------------------------**

**Fortunella_japonica_6** **------------------------------------------------------------**

**Fortunella_japonica_7** **------------------------------------------------------------**

**Fortunella_japonica_8** **------------------------------------------------------------**

**Fortunella_japonica_9** **------------------------------------------------------------**

**Fortunella_japonica_10** **------------------------------------------------------------**

**Fortunella_japonica_11** **------------------------------------------------------------**

**Fortunella_japonica_12** **------------------------------------------------------------**

**Fortunella_margarita_1** **------------------------------------------------------------**

**Fortunella_margarita_2** **------------------------------------------------------------**

**Fortunella_venosa_1** **------------------------------------------------------------**

**Fortunella_venosa_2** **------------------------------------------------------------**

**Fortunella_venosa_3** **------------------------------------------------------------**

14890	14900	14910	14920	14930	14940

....|....|....|....|....|....|....|....|....|....|....|....|

**Atalantia_kwangtungens** **attgaggattcccgataagagagatacttattagtatcaagggccgggactcatgtcaat**

**Citrus_reticulata_1** **attggggattcccgataagagggatacttattagtattaggggccgggactcctgtcaat**

**Citrus_reticulata_2** **attggggattcccgataagagggatacttattagtattaggggccgggactcctgtcaat**

**Citrus_trifoliata_3** **attggggattcccgataagagggatacttattagtattaggggccgggactcctgtcaat**

**Citrus_aurantiifolia** **attggggattcccgataagagggatacttattagtattaggggccgggactcctgtccat**

**Citrus_aurantium** **attggggattcccgataagagggatacttattagtattaggggccgggactcctgtccat**

**Citrus_cavaleriei** **attggggattcccgataagagggatacttattagtattaggggccgggactcctgtcaat**

**Citrus_hongheensis** **attggggattcccgataagagggatacttattagtattaggggccgggactcctgtccat**

**Citrus_limon** **attggggattcccgataagagggatacttattagtattaggggccgggactcctgtccat**

**Citrus_maxima** **attggggattcccgataagagggatacttattagtattaggggccgggactcctgtccat**

**Citrus_medica** **------------------------------------------------------------**

**Citrus_polytrifolia** **attggggattcccgataagagggatacttattagtattaggggccgggactcctgtcaat**

**Citrus_reticulata** **attggggattcccgataagagggatacttattagtattaggggccgggactcctgtcaat**

**Citrus_sinensis** **attggggattcccgataagagggatacttattagtattaggggccgggactcctgtccat**

**Fortunella_bawangica_1** **------------------------------------------------------------**

**Fortunella_bawangica_2** **------------------------------------------------------------**

**Fortunella_hindsii_1** **------------------------------------------------------------**

**Fortunella_hindsii_2** **------------------------------------------------------------**

**Fortunella_hindsii_3** **------------------------------------------------------------**

**Fortunella_japonica_1** **------------------------------------------------------------**

**Fortunella_japonica_2** **------------------------------------------------------------**

**Fortunella_japonica_3** **------------------------------------------------------------**

**Fortunella_japonica_4** **------------------------------------------------------------**

**Fortunella_japonica_5** **------------------------------------------------------------**

**Fortunella_japonica_6** **------------------------------------------------------------**

**Fortunella_japonica_7** **------------------------------------------------------------**

**Fortunella_japonica_8** **------------------------------------------------------------**

**Fortunella_japonica_9** **------------------------------------------------------------**

**Fortunella_japonica_10** **------------------------------------------------------------**

**Fortunella_japonica_11** **------------------------------------------------------------**

**Fortunella_japonica_12** **------------------------------------------------------------**

**Fortunella_margarita_1** **------------------------------------------------------------**

**Fortunella_margarita_2** **------------------------------------------------------------**

**Fortunella_venosa_1** **------------------------------------------------------------**

**Fortunella_venosa_2** **------------------------------------------------------------**

**Fortunella_venosa_3** **------------------------------------------------------------**

14950	14960	14970	14980	14990	15000

....|....|....|....|....|....|....|....|....|....|....|....|

**Atalantia_kwangtungens** **tgcaaaacccctcgtttgttgcagcatcacttaaaaagagggtttcctttagactaagaa**

**Citrus_reticulata_1** **tgcaaaacccctcgtttgttgcagcatcacttaaaaagagggtttcctttagactaagaa**

**Citrus_reticulata_2** **tgcaaaacccctcgtttgttgcagcatcacttaaaaagagggtttcctttagactaagaa**

**Citrus_trifoliata_3** **tgcaaaacccctcgtttgttgcagcatcacttaaaaagagggtttccttgagactaagaa**

**Citrus_aurantiifolia** **tgcaaaacccctcgtttgttgcagcatcacttaaaaagagggtttcctttagactaagaa**

**Citrus_aurantium** **tgcaaaacccctcgtttgttgcagcatcacttaaaaagagggtttcctttagactaagaa**

**Citrus_cavaleriei** **tgcaaaacccctcgtttgttgcagcatcacttaaaaagagggtttcctttagactaagaa**

**Citrus_hongheensis** **tgcaaaacccctcgtttgttgcagcatcacttaaaaagagggtttcctttagactaagaa**

**Citrus_limon** **tgcaaaacccctcgtttgttgcagcatcacttaaaaagagggtttcctttagactaagaa**

**Citrus_maxima** **tgcaaaacccctcgtttgttgcagcatcacttaaaaagagggtttcctttagactaagaa**

**Citrus_medica** **------------------------------------------------------------**

**Citrus_polytrifolia** **tgcaaaacccctcgtttgttgcagcatcacttaaaaagagggtttccttgagactaagaa**

**Citrus_reticulata** **tgcaaaacccctcgtttgttgcagcatcacttaaaaagagggtttcctttagactaagaa**

**Citrus_sinensis** **tgcaaaacccctcgtttgttgcagcatcacttaaaaagagggtttcctttagactaagaa**

**Fortunella_bawangica_1** **------------------------------------------------------------**

**Fortunella_bawangica_2** **------------------------------------------------------------**

**Fortunella_hindsii_1** **------------------------------------------------------------**

**Fortunella_hindsii_2** **------------------------------------------------------------**

**Fortunella_hindsii_3** **------------------------------------------------------------**

**Fortunella_japonica_1** **------------------------------------------------------------**

**Fortunella_japonica_2** **------------------------------------------------------------**

**Fortunella_japonica_3** **------------------------------------------------------------**

**Fortunella_japonica_4** **------------------------------------------------------------**

**Fortunella_japonica_5** **------------------------------------------------------------**

**Fortunella_japonica_6** **------------------------------------------------------------**

**Fortunella_japonica_7** **------------------------------------------------------------**

**Fortunella_japonica_8** **------------------------------------------------------------**

**Fortunella_japonica_9** **------------------------------------------------------------**

**Fortunella_japonica_10** **------------------------------------------------------------**

**Fortunella_japonica_11** **------------------------------------------------------------**

**Fortunella_japonica_12** **------------------------------------------------------------**

**Fortunella_margarita_1** **------------------------------------------------------------**

**Fortunella_margarita_2** **------------------------------------------------------------**

**Fortunella_venosa_1** **------------------------------------------------------------**

**Fortunella_venosa_2** **------------------------------------------------------------**

**Fortunella_venosa_3** **------------------------------------------------------------**

15010	15020	15030	15040	15050	15060

....|....|....|....|....|....|....|....|....|....|....|....|

**Atalantia_kwangtungens** **agggaaagaaaagggcgaattggtatgcctatcctaattccccatcctcaaatcagtcct**

**Citrus_reticulata_1** **agggaaagaaaaaggcgaactggtatgcctatcctaattccccatcctcaaatcagtcct**

**Citrus_reticulata_2** **agggaaagaaaaaggcgaactggtatgcctatcctaattccccatcctcaaatcagtcct**

**Citrus_trifoliata_3** **agggaaagaaaaaggcgaactggtatgcctatcctaattccccatcctcaaatcagtcct**

**Citrus_aurantiifolia** **agggaaagaaaaaggcgaactggtatgcctatcctaattccccatcctcaaatcagtcct**

**Citrus_aurantium** **agggaaagaaaaaggcgaactggtatgcctatcctaattccccatcctcaaatcagtcct**

**Citrus_cavaleriei** **agggaaagaaaaaggcgaactggtatgcctatcctaattccccatcctcaaatcagtcct**

**Citrus_hongheensis** **agggaaagaaaaaggcgaactggtatgcctatcctaattccccatcctcaaatcagtcct**

**Citrus_limon** **agggaaagaaaaaggcgaactggtatgcctatcctaattccccatcctcaaatcagtcct**

**Citrus_maxima** **agggaaagaaaaaggcgaactggtatgcctatcctaattccccatcctcaaatcagtcct**

**Citrus_medica** **------------------------------------------------------------**

**Citrus_polytrifolia** **agggaaagaaaaaggcgaactggtatgcctatcctaattccccatcctcaaatcagtcct**

**Citrus_reticulata** **agggaaagaaaaaggcgaactggtatgcctatcctaattccccatcctcaaatcagtcct**

**Citrus_sinensis** **agggaaagaaaaaggcgaactggtatgcctatcctaattccccatcctcaaatcagtcct**

**Fortunella_bawangica_1** **------------------------------------------------------------**

**Fortunella_bawangica_2** **------------------------------------------------------------**

**Fortunella_hindsii_1** **------------------------------------------------------------**

**Fortunella_hindsii_2** **------------------------------------------------------------**

**Fortunella_hindsii_3** **------------------------------------------------------------**

**Fortunella_japonica_1** **------------------------------------------------------------**

**Fortunella_japonica_2** **------------------------------------------------------------**

**Fortunella_japonica_3** **------------------------------------------------------------**

**Fortunella_japonica_4** **------------------------------------------------------------**

**Fortunella_japonica_5** **------------------------------------------------------------**

**Fortunella_japonica_6** **------------------------------------------------------------**

**Fortunella_japonica_7** **------------------------------------------------------------**

**Fortunella_japonica_8** **------------------------------------------------------------**

**Fortunella_japonica_9** **------------------------------------------------------------**

**Fortunella_japonica_10** **------------------------------------------------------------**

**Fortunella_japonica_11** **------------------------------------------------------------**

**Fortunella_japonica_12** **------------------------------------------------------------**

**Fortunella_margarita_1** **------------------------------------------------------------**

**Fortunella_margarita_2** **------------------------------------------------------------**

**Fortunella_venosa_1** **------------------------------------------------------------**

**Fortunella_venosa_2** **------------------------------------------------------------**

**Fortunella_venosa_3** **------------------------------------------------------------**

15070	15080	15090	15100	15110	15120

....|....|....|....|....|....|....|....|....|....|....|....|

**Atalantia_kwangtungens** **tccaattaaaggagttaaatcttgatagaatttgaaaaagtcagtttcatatgatctcct**

**Citrus_reticulata_1** **tccaattggaggagttaaatcttgatagaattcaaaaaagccagaaacacaga-------**

**Citrus_reticulata_2** **tccaattggaggagttaaatcttgatagaattcaaaaaagccagaaacacaga-------**

**Citrus_trifoliata_3** **tccaattggaggagttaaatcttgatagaattcaaaaaagccagaaacacaga-------**

**Citrus_aurantiifolia** **tccaattggaggagttaaatcttgatagaattcaaaaaagccagaaacacgga-------**

**Citrus_aurantium** **tccaattggaggagttaaatcttgatagaattcaaaaaagccagaaacacgga-------**

**Citrus_cavaleriei** **tccaattggaggagttaaatcttgatagaattcaaaaaagccagaaacacaga-------**

**Citrus_hongheensis** **tccaattggaggagttaaatcttgatagaattcaaaaaagccagaaacacgga-------**

**Citrus_limon** **tccaattggaggagttaaatcttgatagaattcaaaaaagccagaaacacgga-------**

**Citrus_maxima** **tccaattggaggagttaaatcttgatagaattcaaaaaagccagaaacacgga-------**

**Citrus_medica** **------------------------------------------------------------**

**Citrus_polytrifolia** **tccaattggaggagttaaatcttgatagaattcaaaaaagccagaaacacaga-------**

**Citrus_reticulata** **tccaattggaggagttaaatcttgatagaattcaaaaaagccagaaacacaga-------**

**Citrus_sinensis** **tccaattggaggagttaaatcttgatagaattcaaaaaagccagaaacacgga-------**

**Fortunella_bawangica_1** **------------------------------------------------------------**

**Fortunella_bawangica_2** **------------------------------------------------------------**

**Fortunella_hindsii_1** **------------------------------------------------------------**

**Fortunella_hindsii_2** **------------------------------------------------------------**

**Fortunella_hindsii_3** **------------------------------------------------------------**

**Fortunella_japonica_1** **------------------------------------------------------------**

**Fortunella_japonica_2** **------------------------------------------------------------**

**Fortunella_japonica_3** **------------------------------------------------------------**

**Fortunella_japonica_4** **------------------------------------------------------------**

**Fortunella_japonica_5** **------------------------------------------------------------**

**Fortunella_japonica_6** **------------------------------------------------------------**

**Fortunella_japonica_7** **------------------------------------------------------------**

**Fortunella_japonica_8** **------------------------------------------------------------**

**Fortunella_japonica_9** **------------------------------------------------------------**

**Fortunella_japonica_10** **------------------------------------------------------------**

**Fortunella_japonica_11** **------------------------------------------------------------**

**Fortunella_japonica_12** **------------------------------------------------------------**

**Fortunella_margarita_1** **------------------------------------------------------------**

**Fortunella_margarita_2** **------------------------------------------------------------**

**Fortunella_venosa_1** **------------------------------------------------------------**

**Fortunella_venosa_2** **------------------------------------------------------------**

**Fortunella_venosa_3** **------------------------------------------------------------**

15130	15140	15150	15160	15170	15180

....|....|....|....|....|....|....|....|....|....|....|....|

**Atalantia_kwangtungens** **cttttagatagactaaaaaaaaaaaaata-------agtaaatcgcccttcctatttcta**

**Citrus_reticulata_1** **---tataataaataagagaaaaaaaaataagtaaattgtaaattgcccttcctatttcta**

**Citrus_reticulata_2** **---tataataaataagagaaaaaaaaataagtaaattgtaaattgcccttcctatttcta**

**Citrus_trifoliata_3** **---tataataaataagagaaaaaaaaaaa-------agtaaattgcccttcctatttcta**

**Citrus_aurantiifolia** **---tataataaataagagaaaaaaata---------agtaaattgcccttcctatttcta**

**Citrus_aurantium** **---tataataaataagagaaaaaaaata--------agtaaattgcccttcctatttcta**

**Citrus_cavaleriei** **---tataataaataagagaaaaaaaga---------agtaaattgcccttcctatttcta**

**Citrus_hongheensis** **---tataataaataagagaaaaaaaata--------agtaaattgcccttcctatttcta**

**Citrus_limon** **---tataataaataagagaaaaaaaata--------agtaaattgcccttcctatttcta**

**Citrus_maxima** **---tataataaataagagaaaaaaaata--------agtaaattgcccttcctatttcta**

**Citrus_medica** **------------------------------------agtaaattgcccttcctatttcta**

**Citrus_polytrifolia** **---tataataaataagag-aaaaaaaaaa-------agtaaattgcccttcctatttcta**

**Citrus_reticulata** **---tataataaataagagaaaaaaaaataagtaaattgtaaattgcccttcctatttcta**

**Citrus_sinensis** **---tataataaataagagaaaaaaaata--------agtaaattgcccttcctatttcta**

**Fortunella_bawangica_1** **------------------------------------agtaaattgcccttcctatttcta**

**Fortunella_bawangica_2** **------------------------------------agtaaattgcccttcctatttcta**

**Fortunella_hindsii_1** **------------------------------------agtaaattgcccttcctatttcta**

**Fortunella_hindsii_2** **------------------------------------agtaaattgcccttcctatttcta**

**Fortunella_hindsii_3** **------------------------------------agtaaattgcccttcctatttcta**

**Fortunella_japonica_1** **------------------------------------agtaaattgcccttcctatttcta**

**Fortunella_japonica_2** **------------------------------------agtaaattgcccttcctatttcta**

**Fortunella_japonica_3** **------------------------------------agtaaattgcccttcctatttcta**

**Fortunella_japonica_4** **------------------------------------agtaaattgcccttcctatttcta**

**Fortunella_japonica_5** **------------------------------------agtaaattgcccttcctatttcta**

**Fortunella_japonica_6** **------------------------------------agtaaattgcccttcctatttcta**

**Fortunella_japonica_7** **------------------------------------agtaaattgcccttcctatttcta**

**Fortunella_japonica_8** **------------------------------------agtaaattgcccttcctatttcta**

**Fortunella_japonica_9** **------------------------------------agtaaattgcccttcctatttcta**

**Fortunella_japonica_10** **------------------------------------agtaaattgcccttcctatttcta**

**Fortunella_japonica_11** **------------------------------------agtaaattgcccttcctatttcta**

**Fortunella_japonica_12** **------------------------------------agtaaattgcccttcctatttcta**

**Fortunella_margarita_1** **------------------------------------agtaaattgcccttcctatttcta**

**Fortunella_margarita_2** **------------------------------------agtaaattgcccttcctatttcta**

**Fortunella_venosa_1** **------------------------------------agtaaattgcccttcctatttcta**

**Fortunella_venosa_2** **------------------------------------agtaaattgcccttcctatttcta**

**Fortunella_venosa_3** **------------------------------------agtaaattgcccttcctatttcta**

15190	15200	15210	15220	15230	15240

....|....|....|....|....|....|....|....|....|....|....|....|

**Atalantia_kwangtungens** **ggattaaacaaaaggattcgcaaataaaagtgctaatgctacaaccagcccataaattgt**

**Citrus_reticulata_1** **ggattaaacaaaaggattcgcaaataaaagtgctaatgctacaaccagcccataaattgt**

**Citrus_reticulata_2** **ggattaaacaaaaggattcgcaaataaaagtgctaatgctacaaccagcccataaattgt**

**Citrus_trifoliata_3** **ggattaaacaaaaggattcgcaaataaaagtgctaatgctacaaccagcccataaattgt**

**Citrus_aurantiifolia** **ggattaaacaaaaggattcgcaaataaaagtgctaatgctacaaccagcccataaattgt**

**Citrus_aurantium** **ggattaaacaaaaggattcgcaaataaaagtgctaatgctacaaccagcccataaattgt**

**Citrus_cavaleriei** **ggattaaacaaaaggattcgcaaataaaagtgctaatgctacaaccagcccataaattgt**

**Citrus_hongheensis** **ggattaaacaaaaggattcgcaaataaaagtgctaatgctacaaccagcccataaattgt**

**Citrus_limon** **ggattaaacaaaaggattcgcaaataaaagtgctaatgctacaaccagcccataaattgt**

**Citrus_maxima** **ggattaaacaaaaggattcgcaaataaaagtgctaatgctacaaccagcccataaattgt**

**Citrus_medica** **ggattaaacaaaaggattcgcaaataaaagcgctaatgctacaaccagcccataaattgt**

**Citrus_polytrifolia** **ggattaaacaaaaggattcgcaaataaaagtgctaatgctacaaccagcccataaattgt**

**Citrus_reticulata** **ggattaaacaaaaggattcgcaaataaaagtgctaatgctacaaccagcccataaattgt**

**Citrus_sinensis** **ggattaaacaaaaggattcgcaaataaaagtgctaatgctacaaccagcccataaattgt**

**Fortunella_bawangica_1** **ggattaaacaaaaggattcgcaaataaaagtgctaatgctacaaccagcccataaattgt**

**Fortunella_bawangica_2** **ggattaaacaaaaggattcgcaaataaaagtgctaatgctacaaccagcccataaattgt**

**Fortunella_hindsii_1** **ggattaaacaaaaggattcgcaaataaaagtgctaatgctacaaccagcccataaattgt**

**Fortunella_hindsii_2** **ggattaaacaaaaggattcgcaaataaaagtgctaatgctacaaccagcccataaattgt**

**Fortunella_hindsii_3** **ggattaaacaaaaggattcgcaaataaaagtgctaatgctacaaccagcccataaattgt**

**Fortunella_japonica_1** **ggattaaacaaaaggattcgcaaataaaagtgctaatgctacaaccagcccataaattgt**

**Fortunella_japonica_2** **ggattaaacaaaaggattcgcaaataaaagtgctaatgctacaaccagcccataaattgt**

**Fortunella_japonica_3** **ggattaaacaaaaggattcgcaaataaaagtgctaatgctacaaccagcccataaattgt**

**Fortunella_japonica_4** **ggattaaacaaaaggattcgcaaataaaagtgctaatgctacaaccagcccataaattgt**

**Fortunella_japonica_5** **ggattaaacaaaaggattcgcaaataaaagtgctaatgctacaaccagcccataaattgt**

**Fortunella_japonica_6** **ggattaaacaaaaggattcgcaaataaaagtgctaatgctacaaccagcccataaattgt**

**Fortunella_japonica_7** **ggattaaacaaaaggattcgcaaataaaagtgctaatgctacaaccagcccataaattgt**

**Fortunella_japonica_8** **ggattaaacaaaaggattcgcaaataaaagtgctaatgctacaaccagcccataaattgt**

**Fortunella_japonica_9** **ggattaaacaaaaggattcgcaaataaaagtgctaatgctacaaccagcccataaattgt**

**Fortunella_japonica_10** **ggattaaacaaaaggattcgcaaataaaagtgctaatgctacaaccagcccataaattgt**

**Fortunella_japonica_11** **ggattaaacaaaaggattcgcaaataaaagtgctaatgctacaaccagcccataaattgt**

**Fortunella_japonica_12** **ggattaaacaaaaggattcgcaaataaaagtgctaatgctacaaccagcccataaattgt**

**Fortunella_margarita_1** **ggattaaacaaaaggattcgcaaataaaagtgctaatgctacaaccagcccataaattgt**

**Fortunella_margarita_2** **ggattaaacaaaaggattcgcaaataaaagtgctaatgctacaaccagcccataaattgt**

**Fortunella_venosa_1** **ggattaaacaaaaggattcgcaaataaaagtgctaatgctacaaccagcccataaattgt**

**Fortunella_venosa_2** **ggattaaacaaaaggattcgcaaataaaagtgctaatgctacaaccagcccataaattgt**

**Fortunella_venosa_3** **ggattaaacaaaaggattcgcaaataaaagtgctaatgctacaaccagcccataaattgt**

15250	15260	15270	15280	15290	15300

....|....|....|....|....|....|....|....|....|....|....|....|

**Atalantia_kwangtungens** **taaagcttccataaaagccagactaagcaataaagtacctcgtatttttccctccgcctc**

**Citrus_reticulata_1** **taaagcttccataaaagccagactaagcaataaagtacctcgtatttttccctccgcctc**

**Citrus_reticulata_2** **taaagcttccataaaagccagactaagcaataaagtacctcgtatttttccctccgcctc**

**Citrus_trifoliata_3** **taaagcttccataaaagccagactaagcaataaagtacctcgtatttttccctccgcctc**

**Citrus_aurantiifolia** **taaagcttccataaaagccagactaagcaataaagtacctcgtatttttccctccgcctc**

**Citrus_aurantium** **taaagcttccataaaagccagactaagcaataaagtacctcgtatttttccctctgcctc**

**Citrus_cavaleriei** **taaagcttccataaaagccagactaagcaataaagtacctcgtatttttccctccgcctc**

**Citrus_hongheensis** **taaagcttccataaaagccagactaagcaataaagtacctcgtatttttccctctgcctc**

**Citrus_limon** **taaagcttccataaaagccagactaagcaataaagtacctcgtatttttccctctgcctc**

**Citrus_maxima** **taaagcttccataaaagccagactaagcaataaagtacctcgtatttttccctctgcctc**

**Citrus_medica** **taaagcttccataaaagccagactaagcaataaagtacctcgtatttttccctccgcctc**

**Citrus_polytrifolia** **taaagcttccataaaagccagactaagcaataaagtacctcgtatttttccctccgcctc**

**Citrus_reticulata** **taaagcttccataaaagccagactaagcaataaagtacctcgtatttttccctccgcctc**

**Citrus_sinensis** **taaagcttccataaaagccagactaagcaataaagtacctcgtatttttccctctgcctc**

**Fortunella_bawangica_1** **taaagcttccataaaagccagactaagcaataaagtacctcgtatttttccctccgcctc**

**Fortunella_bawangica_2** **taaagcttccataaaagccagactaagcaataaagtacctcgtatttttccctccgcctc**

**Fortunella_hindsii_1** **taaagcttccataaaagccagactaagcaataaagtacctcgtatttttccctccgcctc**

**Fortunella_hindsii_2** **taaagcttccataaaagccagactaagcaataaagtacctcgtatttttccctccgcctc**

**Fortunella_hindsii_3** **taaagcttccataaaagccagactaagcaataaagtacctcgtatttttccctccgcctc**

**Fortunella_japonica_1** **taaagcttccataaaagccagactaagcaataaagtacctcgtatttttccctccgcctc**

**Fortunella_japonica_2** **taaagcttccataaaagccagactaagcaataaagtacctcgtatttttccctccgcctc**

**Fortunella_japonica_3** **taaagcttccataaaagccagactaagcaataaagtacctcgtatttttccctccgcctc**

**Fortunella_japonica_4** **taaagcttccataaaagccagactaagcaataaagtacctcgtatttttccctccgcctc**

**Fortunella_japonica_5** **taaagcttccataaaagccagactaagcaataaagtacctcgtatttttccctccgcctc**

**Fortunella_japonica_6** **taaagcttccataaaagccagactaagcaataaagtacctcgtatttttccctccgcctc**

**Fortunella_japonica_7** **taaagcttccataaaagccagactaagcaataaagtacctcgtatttttccctccgcctc**

**Fortunella_japonica_8** **taaagcttccataaaagccagactaagcaataaagtacctcgtatttttccctccgcctc**

**Fortunella_japonica_9** **taaagcttccataaaagccagactaagcaataaagtacctcgtatttttccctccgcctc**

**Fortunella_japonica_10** **taaagcttccataaaagccagactaagcaataaagtacctcgtatttttccctccgcctc**

**Fortunella_japonica_11** **taaagcttccataaaagccagactaagcaataaagtacctcgtatttttccctccgcctc**

**Fortunella_japonica_12** **taaagcttccataaaagccagactaagcaataaagtacctcgtatttttccctccgcctc**

**Fortunella_margarita_1** **taaagcttccataaaagccagactaagcaataaagtacctcgtatttttccctccgcctc**

**Fortunella_margarita_2** **taaagcttccataaaagccagactaagcaataaagtacctcgtatttttccctccgcctc**

**Fortunella_venosa_1** **taaagcttccataaaagccagactaagcaataaagtacctcgtatttttccctccgcctc**

**Fortunella_venosa_2** **taaagcttccataaaagccagactaagcaataaagtacctcgtatttttccctccgcctc**

**Fortunella_venosa_3** **taaagcttccataaaagccagactaagcaataaagtacctcgtatttttccctccgcctc**

15310	15320	15330	15340	15350	15360

....|....|....|....|....|....|....|....|....|....|....|....|

**Atalantia_kwangtungens** **gggctgtctcgcgatcccttctactgcctggcccgcagcagtaccttgaccaactccagg**

**Citrus_reticulata_1** **gggctgtctcgcgatcccttctactgcctggcccgcagcagtaccttgaccaactccagg**

**Citrus_reticulata_2** **gggctgtctcgcgatcccttctactgcctggcccgcagcagtaccttgaccaactccagg**

**Citrus_trifoliata_3** **gggctgtctcgcgatcccttctactgcctggcccgcagcagtaccttgaccaactccagg**

**Citrus_aurantiifolia** **gggctgtctcgcgatcccttctactgcctggcccgcagcagtaccttgaccaactccagg**

**Citrus_aurantium** **gggctgtctcgcgatcccttctactgcctggcccgcagcagtaccttgaccaactccagg**

**Citrus_cavaleriei** **gggctgtctcgcgatcccttctactgcctggcccgcagcagtaccttgaccaactccagg**

**Citrus_hongheensis** **gggctgtctcgcgatcccttctactgcctggcccgcagcagtaccttgaccaactccagg**

**Citrus_limon** **gggctgtctcgcgatcccttctactgcctggcccgcagcagtaccttgaccaactccagg**

**Citrus_maxima** **gggctgtctcgcgatcccttctactgcctggcccgcagcagtaccttgaccaactccagg**

**Citrus_medica** **gggctgtctcgcgatcccttctactgcctggcccgcagcagtaccttgaccaactccagg**

**Citrus_polytrifolia** **gggctgtctcgcgatcccttctactgcctggcccgcagcagtaccttgaccaactccagg**

**Citrus_reticulata** **gggctgtctcgcgatcccttctactgcctggcccgcagcagtaccttgaccaactccagg**

**Citrus_sinensis** **gggctgtctcgcgatcccttctactgcctggcccgcagcagtaccttgaccaactccagg**

**Fortunella_bawangica_1** **gggctgtctcgcgatcccttctactgcctggcccgcagcagtaccttgaccaactccagg**

**Fortunella_bawangica_2** **gggctgtctcgcgatcccttctactgcctggcccgcagcagtaccttgaccaactccagg**

**Fortunella_hindsii_1** **gggctgtctcgcgatcccttctactgcctggcccgcagcagtaccttgaccaactccagg**

**Fortunella_hindsii_2** **gggctgtctcgcgatcccttctactgcctggcccgcagcagtaccttgaccaactccagg**

**Fortunella_hindsii_3** **gggctgtctcgcgatcccttctactgcctggcccgcagcagtaccttgaccaactccagg**

**Fortunella_japonica_1** **gggctgtctcgcgatcccttctactgcctggcccgcagcagtaccttgaccaactccagg**

**Fortunella_japonica_2** **gggctgtctcgcgatcccttctactgcctggcccgcagcagtaccttgaccaactccagg**

**Fortunella_japonica_3** **gggctgtctcgcgatcccttctactgcctggcccgcagcagtaccttgaccaactccagg**

**Fortunella_japonica_4** **gggctgtctcgcgatcccttctactgcctggcccgcagcagtaccttgaccaactccagg**

**Fortunella_japonica_5** **gggctgtctcgcgatcccttctactgcctggcccgcagcagtaccttgaccaactccagg**

**Fortunella_japonica_6** **gggctgtctcgcgatcccttctactgcctggcccgcagcagtaccttgaccaactccagg**

**Fortunella_japonica_7** **gggctgtctcgcgatcccttctactgcctggcccgcagcagtaccttgaccaactccagg**

**Fortunella_japonica_8** **gggctgtctcgcgatcccttctactgcctggcccgcagcagtaccttgaccaactccagg**

**Fortunella_japonica_9** **gggctgtctcgcgatcccttctactgcctggcccgcagcagtaccttgaccaactccagg**

**Fortunella_japonica_10** **gggctgtctcgcgatcccttctactgcctggcccgcagcagtaccttgaccaactccagg**

**Fortunella_japonica_11** **gggctgtctcgcgatcccttctactgcctggcccgcagcagtaccttgaccaactccagg**

**Fortunella_japonica_12** **gggctgtctcgcgatcccttctactgcctggcccgcagcagtaccttgaccaactccagg**

**Fortunella_margarita_1** **gggctgtctcgcgatcccttctactgcctggcccgcagcagtaccttgaccaactccagg**

**Fortunella_margarita_2** **gggctgtctcgcgatcccttctactgcctggcccgcagcagtaccttgaccaactccagg**

**Fortunella_venosa_1** **gggctgtctcgcgatcccttctactgcctggcccgcagcagtaccttgaccaactccagg**

**Fortunella_venosa_2** **gggctgtctcgcgatcccttctactgcctggcccgcagcagtaccttgaccaactccagg**

**Fortunella_venosa_3** **gggctgtctcgcgatcccttctactgcctggcccgcagcagtaccttgaccaactccagg**

15370	15380	15390	15400	15410	15420

....|....|....|....|....|....|....|....|....|....|....|....|

**Atalantia_kwangtungens** **tccaatagaagcaagcccaacagccaacccagcagcaataacggaagcggcagaaatcag**

**Citrus_reticulata_1** **tccaatagaagcaagcccaacagccaacccagcagcaataacggaagcggcagaaatcag**

**Citrus_reticulata_2** **tccaatagaagcaagcccaacagccaacccagcagcaataacggaagcggcagaaatcag**

**Citrus_trifoliata_3** **tccaatagaagcaagcccaacagccaacccagcagcaataacggaagcggcagaaatcag**

**Citrus_aurantiifolia** **tccaatagaagcaagcccaacagccaacccagcagcaataacggaagcggcagaaatcag**

**Citrus_aurantium** **tccaatagaagcaagcccaacagccaacccagcagcaataacggaagcggcagaaatcag**

**Citrus_cavaleriei** **tccaatagaagcaagcccaacagccaacccagcagcaataacggaagcggcagaaatcag**

**Citrus_hongheensis** **tccaatagaagcaagcccaacagccaacccagcagcaataacggaagcggcagaaatcag**

**Citrus_limon** **tccaatagaagcaagcccaacagccaacccagcagcaataacggaagcggcagaaatcag**

**Citrus_maxima** **tccaatagaagcaagcccaacagccaacccagcagcaataacggaagcggcagaaatcag**

**Citrus_medica** **tccaatagaagcaagcccaacagccaacccagcagcaataacggaagcggcagaaatcag**

**Citrus_polytrifolia** **tccaatagaagcaagcccaacagccaacccagcagcaataacggaagcggcagaaatcag**

**Citrus_reticulata** **tccaatagaagcaagcccaacagccaacccagcagcaataacggaagcggcagaaatcag**

**Citrus_sinensis** **tccaatagaagcaagcccaacagccaacccagcagcaataacggaagcggcagaaatcag**

**Fortunella_bawangica_1** **tccaatagaagcaagcccaacagccaacccagcagcaataacggaagcggcagaaatcag**

**Fortunella_bawangica_2** **tccaatagaagcaagcccaacagccaacccagcagcaataacggaagcggcagaaatcag**

**Fortunella_hindsii_1** **tccaatagaagcaagcccaacagccaacccagcagcaataacggaagcggcagaaatcag**

**Fortunella_hindsii_2** **tccaatagaagcaagcccaacagccaacccagcagcaataacggaagcggcagaaatcag**

**Fortunella_hindsii_3** **tccaatagaagcaagcccaacagccaacccagcagcaataacggaagcggcagaaatcag**

**Fortunella_japonica_1** **tccaatagaagcaagcccaacagccaacccagcagcaataacggaagcggcagaaatcag**

**Fortunella_japonica_2** **tccaatagaagcaagcccaacagccaacccagcagcaataacggaagcggcagaaatcag**

**Fortunella_japonica_3** **tccaatagaagcaagcccaacagccaacccagcagcaataacggaagcggcagaaatcag**

**Fortunella_japonica_4** **tccaatagaagcaagcccaacagccaacccagcagcaataacggaagcggcagaaatcag**

**Fortunella_japonica_5** **tccaatagaagcaagcccaacagccaacccagcagcaataacggaagcggcagaaatcag**

**Fortunella_japonica_6** **tccaatagaagcaagcccaacagccaacccagcagcaataacggaagcggcagaaatcag**

**Fortunella_japonica_7** **tccaatagaagcaagcccaacagccaacccagcagcaataacggaagcggcagaaatcag**

**Fortunella_japonica_8** **tccaatagaagcaagcccaacagccaacccagcagcaataacggaagcggcagaaatcag**

**Fortunella_japonica_9** **tccaatagaagcaagcccaacagccaacccagcagcaataacggaagcggcagaaatcag**

**Fortunella_japonica_10** **tccaatagaagcaagcccaacagccaacccagcagcaataacggaagcggcagaaatcag**

**Fortunella_japonica_11** **tccaatagaagcaagcccaacagccaacccagcagcaataacggaagcggcagaaatcag**

**Fortunella_japonica_12** **tccaatagaagcaagcccaacagccaacccagcagcaataacggaagcggcagaaatcag**

**Fortunella_margarita_1** **tccaatagaagcaagcccaacagccaacccagcagcaataacggaagcggcagaaatcag**

**Fortunella_margarita_2** **tccaatagaagcaagcccaacagccaacccagcagcaataacggaagcggcagaaatcag**

**Fortunella_venosa_1** **tccaatagaagcaagcccaacagccaacccagcagcaataacggaagcggcagaaatcag**

**Fortunella_venosa_2** **tccaatagaagcaagcccaacagccaacccagcagcaataacggaagcggcagaaatcag**

**Fortunella_venosa_3** **tccaatagaagcaagcccaacagccaacccagcagcaataacggaagcggcagaaatcag**

15430	15440	15450	15460	15470	15480

....|....|....|....|....|....|....|....|....|....|....|....|

**Atalantia_kwangtungens** **tggattcatgataagtccctcgcactaaaataaagaaaaactaaagaaatcgttaatgat**

**Citrus_reticulata_1** **tggattcatgataagtccctcgcactaaaataaagaaaaactaaagaaatcgttaatgat**

**Citrus_reticulata_2** **tggattcatgataagtccctcgcactaaaataaagaaaaactaaagaaatcgttaatgat**

**Citrus_trifoliata_3** **tggattcatgataagtccctcgcactaaaataaagaaaaactaaagaaatcgttaatgat**

**Citrus_aurantiifolia** **tggattcatgataagtccctcgcactaaaataaagaaaaactaaagaaatcgttaatgat**

**Citrus_aurantium** **tggattcatgataagtccctcgcactaaaataaagaaaaactaaagaaatcgttaatgat**

**Citrus_cavaleriei** **tggattcatgataagtccctcgcactaaaataaagaaaaactaaagaaatcgttaatgat**

**Citrus_hongheensis** **tggattcatgataagtccctcgcactaaaataaagaaaaactaaagaaatcgttaatgat**

**Citrus_limon** **tggattcatgataagtccctcgcactaaaataaagaaaaactaaagaaatcgttaatgat**

**Citrus_maxima** **tggattcatgataagtccctcgcactaaaataaagaaaaactaaagaaatcgttaatgat**

**Citrus_medica** **tggattcatgataagtccctcgcactaaaataaagaaaaactaaagaaatcgttaatgat**

**Citrus_polytrifolia** **tggattcatgataagtccctcgcactaaaataaagaaaaactaaagaaatcgttaatgat**

**Citrus_reticulata** **tggattcatgataagtccctcgcactaaaataaagaaaaactaaagaaatcgttaatgat**

**Citrus_sinensis** **tggattcatgataagtccctcgcactaaaataaagaaaaactaaagaaatcgttaatgat**

**Fortunella_bawangica_1** **tggattcatgataagtccctcgcactaaaataaagaaaaactaaagaaatcgttaatgat**

**Fortunella_bawangica_2** **tggattcatgataagtccctcgcactaaaataaagaaaaactaaagaaatcgttaatgat**

**Fortunella_hindsii_1** **tggattcatgataagtccctcgcactaaaataaagaaaaactaaagaaatcgttaatgat**

**Fortunella_hindsii_2** **tggattcatgataagtccctcgcactaaaataaagaaaaactaaagaaatcgttaatgat**

**Fortunella_hindsii_3** **tggattcatgataagtccctcgcactaaaataaagaaaaactaaagaaatcgttaatgat**

**Fortunella_japonica_1** **tggattcatgataagtccctcgcactaaaataaagaaaaactaaagaaatcgttaatgat**

**Fortunella_japonica_2** **tggattcatgataagtccctcgcactaaaataaagaaaaactaaagaaatcgttaatgat**

**Fortunella_japonica_3** **tggattcatgataagtccctcgcactaaaataaagaaaaactaaagaaatcgttaatgat**

**Fortunella_japonica_4** **tggattcatgataagtccctcgcactaaaataaagaaaaactaaagaaatcgttaatgat**

**Fortunella_japonica_5** **tggattcatgataagtccctcgcactaaaataaagaaaaactaaagaaatcgttaatgat**

**Fortunella_japonica_6** **tggattcatgataagtccctcgcactaaaataaagaaaaactaaagaaatcgttaatgat**

**Fortunella_japonica_7** **tggattcatgataagtccctcgcactaaaataaagaaaaactaaagaaatcgttaatgat**

**Fortunella_japonica_8** **tggattcatgataagtccctcgcactaaaataaagaaaaactaaagaaatcgttaatgat**

**Fortunella_japonica_9** **tggattcatgataagtccctcgcactaaaataaagaaaaactaaagaaatcgttaatgat**

**Fortunella_japonica_10** **tggattcatgataagtccctcgcactaaaataaagaaaaactaaagaaatcgttaatgat**

**Fortunella_japonica_11** **tggattcatgataagtccctcgcactaaaataaagaaaaactaaagaaatcgttaatgat**

**Fortunella_japonica_12** **tggattcatgataagtccctcgcactaaaataaagaaaaactaaagaaatcgttaatgat**

**Fortunella_margarita_1** **tggattcatgataagtccctcgcactaaaataaagaaaaactaaagaaatcgttaatgat**

**Fortunella_margarita_2** **tggattcatgataagtccctcgcactaaaataaagaaaaactaaagaaatcgttaatgat**

**Fortunella_venosa_1** **tggattcatgataagtccctcgcactaaaataaagaaaaactaaagaaatcgttaatgat**

**Fortunella_venosa_2** **tggattcatgataagtccctcgcactaaaataaagaaaaactaaagaaatcgttaatgat**

**Fortunella_venosa_3** **tggattcatgataagtccctcgcactaaaataaagaaaaactaaagaaatcgttaatgat**

15490	15500	15510	15520	15530	15540

....|....|....|....|....|....|....|....|....|....|....|....|

**Atalantia_kwangtungens** **acaatcgtccaataaattatgacttaattattccgtcactaggattcgcccagccgaagt**

**Citrus_reticulata_1** **acaatcgtccaataaattatgacttaattattccgtcactaggattcgcccagccgaagt**

**Citrus_reticulata_2** **acaatcgtccaataaattatgacttaattattccgtcactaggattcgcccagccgaagt**

**Citrus_trifoliata_3** **acaatcgtccaataaattatgacttaattattccgtcactaggattcccccagccgaagt**

**Citrus_aurantiifolia** **acaatcgtccaataaattatgacttaattattccgtcactaggattcgcccagccgaagt**

**Citrus_aurantium** **acaatcgtccaataaattatgacttaattattccgtcactaggattcgcccagccgaagt**

**Citrus_cavaleriei** **acaatcgtccaataaattatgacttaattattccgtcactaggattcgcccagccgaagt**

**Citrus_hongheensis** **acaatcgtccaataaattatgacttaattattccgtcactaggattcgcccagccgaagt**

**Citrus_limon** **acaatcgtccaataaattatgacttaattattccgtcactaggattcgcccagccgaagt**

**Citrus_maxima** **acaatcgtccaataaattatgacttaattattccgtcactaggattcgcccagccgaagt**

**Citrus_medica** **acaatcgtccaataaattatgacttaattattccgtcactaggattcgcccagccaaagt**

**Citrus_polytrifolia** **acaatcgtccaataaattatgacttaattattccgtcactaggattcccccagccgaagt**

**Citrus_reticulata** **acaatcgtccaataaattatgacttaattattccgtcactaggattcgcccagccgaagt**

**Citrus_sinensis** **acaatcgtccaataaattatgacttaattattccgtcactaggattcgcccagccgaagt**

**Fortunella_bawangica_1** **acaatcgtccaataaattatgacttaattattccgtcactaggattcgcccagccgaagt**

**Fortunella_bawangica_2** **acaatcgtccaataaattatgacttaattattccgtcactaggattcgcccagccgaagt**

**Fortunella_hindsii_1** **acaatcgtccaataaattatgacttaattattccgtcactaggattcgcccagccgaagt**

**Fortunella_hindsii_2** **acaatcgtccaataaattatgacttaattattccgtcactaggattcgcccagccgaagt**

**Fortunella_hindsii_3** **acaatcgtccaataaattatgacttaattattccgtcactaggattcgcccagccgaagt**

**Fortunella_japonica_1** **acaatcgtccaataaattatgacttaattattccgtcactaggattcgcccagccgaagt**

**Fortunella_japonica_2** **acaatcgtccaataaattatgacttaattattccgtcactaggattcgcccagccgaagt**

**Fortunella_japonica_3** **acaatcgtccaataaattatgacttaattattccgtcactaggattcgcccagccgaagt**

**Fortunella_japonica_4** **acaatcgtccaataaattatgacttaattattccgtcactaggattcgcccagccgaagt**

**Fortunella_japonica_5** **acaatcgtccaataaattatgacttaattattccgtcactaggattcgcccagccgaagt**

**Fortunella_japonica_6** **acaatcgtccaataaattatgacttaattattccgtcactaggattcgcccagccgaagt**

**Fortunella_japonica_7** **acaatcgtccaataaattatgacttaattattccgtcactaggattcgcccagccgaagt**

**Fortunella_japonica_8** **acaatcgtccaataaattatgacttaattattccgtcactaggattcgcccagccgaagt**

**Fortunella_japonica_9** **acaatcgtccaataaattatgacttaattattccgtcactaggattcgcccagccgaagt**

**Fortunella_japonica_10** **acaatcgtccaataaattatgacttaattattccgtcactaggattcgcccagccgaagt**

**Fortunella_japonica_11** **acaatcgtccaataaattatgacttaattattccgtcactaggattcgcccagccgaagt**

**Fortunella_japonica_12** **acaatcgtccaataaattatgacttaattattccgtcactaggattcgcccagccgaagt**

**Fortunella_margarita_1** **acaatcgtccaataaattatgacttaattattccgtcactaggattcgcccagccgaagt**

**Fortunella_margarita_2** **acaatcgtccaataaattatgacttaattattccgtcactaggattcgcccagccgaagt**

**Fortunella_venosa_1** **acaatcgtccaataaattatgacttaattattccgtcactaggattcgcccagccgaagt**

**Fortunella_venosa_2** **acaatcgtccaataaattatgacttaattattccgtcactaggattcgcccagccgaagt**

**Fortunella_venosa_3** **acaatcgtccaataaattatgacttaattattccgtcactaggattcgcccagccgaagt**

15550	15560	15570	15580	15590	15600

....|....|....|....|....|....|....|....|....|....|....|....|

**Atalantia_kwangtungens** **aactaagaactcccaat--------tggcgtaataataatattattattgaaccatcaaa**

**Citrus_reticulata_1** **aactaagaactcccaat--------tggcgtaataataatattattattgaaccatcaaa**

**Citrus_reticulata_2** **aactaagaactcccaat--------tggcgtaataataatattattattgaaccatcaaa**

**Citrus_trifoliata_3** **aactaagaactcccaat--------tggcgtaataataatattattattgaaccatcaaa**

**Citrus_aurantiifolia** **aactaagaactcccaat--------tggcgtaataataatattattattgaaccatcaaa**

**Citrus_aurantium** **aactaagaactcccaat--------tggcgtaataataatattattattgaaccatcaaa**

**Citrus_cavaleriei** **aactaagaactcccaat--------tggcgtaataataatattattattgaaccatcaaa**

**Citrus_hongheensis** **aactaagaactcccaat--------tggcgtaataataatattattattgaaccatcaaa**

**Citrus_limon** **aactaagaactcccaat--------tggcgtaataataatattattattgaaccatcaaa**

**Citrus_maxima** **aactaagaactcccaat--------tggcgtaataataatattattattgaaccatcaaa**

**Citrus_medica** **aactaagaactcccaattggcgtaatggcgtaataataatattattattgaaccatcaaa**

**Citrus_polytrifolia** **aactaagaactcccaat--------tggcgtaataataatattattattgaaccatcaaa**

**Citrus_reticulata** **aactaagaactcccaat--------tggcgtaataataatattattattgaaccatcaaa**

**Citrus_sinensis** **aactaagaactcccaat--------tggcgtaataataatattattattgaaccatcaaa**

**Fortunella_bawangica_1** **aactaagaactcccaat--------tggcgtaataataatattattattgaaccatcaaa**

**Fortunella_bawangica_2** **aactaagaactcccaat--------tggcgtaataataatattattattgaaccatcaaa**

**Fortunella_hindsii_1** **aactaagaactcccaat--------tggcgtaataataatattattattgaaccatcaaa**

**Fortunella_hindsii_2** **aactaagaactcccaat--------tggcgtaataataatattattattgaaccatcaaa**

**Fortunella_hindsii_3** **aactaagaactcccaat--------tggcgtaataataatattattattgaaccatcaaa**

**Fortunella_japonica_1** **aactaagaactcccaat--------tggcgtaataataatattattattgaaccatcaaa**

**Fortunella_japonica_2** **aactaagaactcccaat--------tggcgtaataataatattattattgaaccatcaaa**

**Fortunella_japonica_3** **aactaagaactcccaat--------tggcgtaataataatattattattgaaccatcaaa**

**Fortunella_japonica_4** **aactaagaactcccaat--------tggcgtaataataatattattattgaaccatcaaa**

**Fortunella_japonica_5** **aactaagaactcccaat--------tggcgtaataataatattattattgaaccatcaaa**

**Fortunella_japonica_6** **aactaagaactcccaat--------tggcgtaataataatattattattgaaccatcaaa**

**Fortunella_japonica_7** **aactaagaactcccaat--------tggcgtaataataatattattattgaaccatcaaa**

**Fortunella_japonica_8** **aactaagaactcccaat--------tggcgtaataataatattattattgaaccatcaaa**

**Fortunella_japonica_9** **aactaagaactcccaat--------tggcgtaataataatattattattgaaccatcaaa**

**Fortunella_japonica_10** **aactaagaactcccaat--------tggcgtaataataatattattattgaaccatcaaa**

**Fortunella_japonica_11** **aactaagaactcccaat--------tggcgtaataataatattattattgaaccatcaaa**

**Fortunella_japonica_12** **aactaagaactcccaat--------tggcgtaataataatattattattgaaccatcaaa**

**Fortunella_margarita_1** **aactaagaactcccaat--------tggcgtaataataatattattattgaaccatcaaa**

**Fortunella_margarita_2** **aactaagaactcccaat--------tggcgtaataataatattattattgaaccatcaaa**

**Fortunella_venosa_1** **aactaagaactcccaat--------tggcgtaataataatattattattgaaccatcaaa**

**Fortunella_venosa_2** **aactaagaactcccaat--------tggcgtaataataatattattattgaaccatcaaa**

**Fortunella_venosa_3** **aactaagaactcccaat--------tggcgtaataataatattattattgaaccatcaaa**

15610	15620	15630	15640	15650	15660

....|....|....|....|....|....|....|....|....|....|....|....|

**Atalantia_kwangtungens** **actttttagatatctctgttttaggttttagtttcgatccacaggcacaacacaggattt**

**Citrus_reticulata_1** **actttttagatatctct-------tttttagtttcgatccacaggcacaacacaggattt**

**Citrus_reticulata_2** **actttttagatatctct-------tttttagtttcgatccacaggcacaacacaggattt**

**Citrus_trifoliata_3** **actttttagatatctct-------tttttagtttcgatccacaggcacaacacaggattt**

**Citrus_aurantiifolia** **actttttagatatctct-------tttttagtttcgatccacaggcacaacacaggattt**

**Citrus_aurantium** **actttttagatatctct-------tttttagtttcgatccacaggcacaacacaggattt**

**Citrus_cavaleriei** **actttttagatatctct-------tttttagtttcgatccacaggcacaacacaggattt**

**Citrus_hongheensis** **actttttagatatctct-------tttttagtttcgatccacaggcacaacacaggattt**

**Citrus_limon** **actttttagatatctct-------tttttagtttcgatccacaggcacaacacaggattt**

**Citrus_maxima** **actttttagatatctct-------tttttagtttcgatccacaggcacaacacaggattt**

**Citrus_medica** **actttttagatatctct-------tttttagtttcgatccacaggcacaacacaggattt**

**Citrus_polytrifolia** **actttttagatatctct-------tttttagtttcgatccacaggcacaacacaggattt**

**Citrus_reticulata** **actttttagatatctct-------tttttagtttcgatccacaggcacaacacaggattt**

**Citrus_sinensis** **actttttagatatctct-------tttttagtttcgatccacaggcacaacacaggattt**

**Fortunella_bawangica_1** **actttttagatatctct-------tttttagtttcgatccacaggcacaacacaggattt**

**Fortunella_bawangica_2** **actttttagatatctct-------tttttagtttcgatccacaggcacaacacaggattt**

**Fortunella_hindsii_1** **actttttagatatctct-------tttttagtttcgatccacaggcacaacacaggattt**

**Fortunella_hindsii_2** **actttttagatatctct-------tttttagtttcgatccacaggcacaacacaggattt**

**Fortunella_hindsii_3** **actttttagatatctct-------tttttagtttcgatccacaggcacaacacaggattt**

**Fortunella_japonica_1** **actttttagatatctct-------tttttagtttcgatccacaggcacaacacaggattt**

**Fortunella_japonica_2** **actttttagatatctct-------tttttagtttcgatccacaggcacaacacaggattt**

**Fortunella_japonica_3** **actttttagatatctct-------tttttagtttcgatccacaggcacaacacaggattt**

**Fortunella_japonica_4** **actttttagatatctct-------tttttagtttcgatccacaggcacaacacaggattt**

**Fortunella_japonica_5** **actttttagatatctct-------tttttagtttcgatccacaggcacaacacaggattt**

**Fortunella_japonica_6** **actttttagatatctct-------tttttagtttcgatccacaggcacaacacaggattt**

**Fortunella_japonica_7** **actttttagatatctct-------tttttagtttcgatccacaggcacaacacaggattt**

**Fortunella_japonica_8** **actttttagatatctct-------tttttagtttcgatccacaggcacaacacaggattt**

**Fortunella_japonica_9** **actttttagatatctct-------tttttagtttcgatccacaggcacaacacaggattt**

**Fortunella_japonica_10** **actttttagatatctct-------tttttagtttcgatccacaggcacaacacaggattt**

**Fortunella_japonica_11** **actttttagatatctct-------tttttagtttcgatccacaggcacaacacaggattt**

**Fortunella_japonica_12** **actttttagatatctct-------tttttagtttcgatccacaggcacaacacaggattt**

**Fortunella_margarita_1** **actttttagatatctct-------tttttagtttcgatccacaggcacaacacaggattt**

**Fortunella_margarita_2** **actttttagatatctct-------tttttagtttcgatccacaggcacaacacaggattt**

**Fortunella_venosa_1** **actttttagatatctct-------tttttagtttcgatccacaggcacaacacaggattt**

**Fortunella_venosa_2** **actttttagatatctct-------tttttagtttcgatccacaggcacaacacaggattt**

**Fortunella_venosa_3** **actttttagatatctct-------tttttagtttcgatccacaggcacaacacaggattt**

15670	15680	15690	15700	15710	15720

....|....|....|....|....|....|....|....|....|....|....|....|

**Atalantia_kwangtungens** **ttgtaaatccatacgacttttgttcttccatttcttttttttttttcttttttcggaacc**

**Citrus_reticulata_1** **ttgtaaatccatacgacttttgttcttcca-------------tttcttttttcggaacc**

**Citrus_reticulata_2** **ttgtaaatccatacgacttttgttcttcca-------------tttcttttttcggaacc**

**Citrus_trifoliata_3** **ttgtaaatccatacgacttttgttcttcca-------------tttcttttttcggaacc**

**Citrus_aurantiifolia** **ttgtaaatccatacgacttttgttcttcca-------------tttcttttttcggaacc**

**Citrus_aurantium** **ttgtaaatccatacgacttttgttcttcca-------------tttcttttttcggaacc**

**Citrus_cavaleriei** **ttgtaaatccatacgacttttgttcttcca-------------tttcttttttcggaacc**

**Citrus_hongheensis** **ttgtaaatccatacgacttttgttcttcca-------------tttcttttttcggaacc**

**Citrus_limon** **ttgtaaatccatacgacttttgttcttcca-------------tttcttttttcggaacc**

**Citrus_maxima** **ttgtaaatccatacgacttttgttcttcca-------------tttcttttttcggaacc**

**Citrus_medica** **ttgtaaatccatacgacttttgttcttcca-------------tttcttttttcggaacc**

**Citrus_polytrifolia** **ttgtaaatccatacgacttttgttcttcca-------------tttcttttttcggaacc**

**Citrus_reticulata** **ttgtaaatccatacgacttttgttcttcca-------------tttcttttttcggaacc**

**Citrus_sinensis** **ttgtaaatccatacgacttttgttcttcca-------------tttcttttttcggaacc**

**Fortunella_bawangica_1** **ttgtaaatccatacgactttggttcttcca-------------tttcttttttcggaacc**

**Fortunella_bawangica_2** **ttgtaaatccatacgactttggttcttcca-------------tttcttttttcggaacc**

**Fortunella_hindsii_1** **ttgtaaatccatacgactttggttcttcca-------------tttcttttttcggaacc**

**Fortunella_hindsii_2** **ttgtaaatccatacgactttggttcttcca-------------tttcttttttcggaacc**

**Fortunella_hindsii_3** **ttgtaaatccatacgactttggttcttcca-------------tttcttttttcggaacc**

**Fortunella_japonica_1** **ttgtaaatccatacgactttggttcttcca-------------tttcttttttcggaacc**

**Fortunella_japonica_2** **ttgtaaatccatacgactttggttcttcca-------------tttcttttttcggaacc**

**Fortunella_japonica_3** **ttgtaaatccatacgactttggttcttcca-------------tttcttttttcggaacc**

**Fortunella_japonica_4** **ttgtaaatccatacgactttggttcttcca-------------tttcttttttcggaacc**

**Fortunella_japonica_5** **ttgtaaatccatacgactttggttcttcca-------------tttcttttttcggaacc**

**Fortunella_japonica_6** **ttgtaaatccatacgactttggttcttcca-------------tttcttttttcggaacc**

**Fortunella_japonica_7** **ttgtaaatccatacgactttggttcttcca-------------tttcttttttcggaacc**

**Fortunella_japonica_8** **ttgtaaatccatacgactttggttcttcca-------------tttcttttttcggaacc**

**Fortunella_japonica_9** **ttgtaaatccatacgactttggttcttcca-------------tttcttttttcggaacc**

**Fortunella_japonica_10** **ttgtaaatccatacgactttggttcttcca-------------tttcttttttcggaacc**

**Fortunella_japonica_11** **ttgtaaatccatacgactttggttcttcca-------------tttcttttttcggaacc**

**Fortunella_japonica_12** **ttgtaaatccatacgactttggttcttcca-------------tttcttttttcggaacc**

**Fortunella_margarita_1** **ttgtaaatccatacgactttggttcttcca-------------tttcttttttcggaacc**

**Fortunella_margarita_2** **ttgtaaatccatacgactttggttcttcca-------------tttcttttttcggaacc**

**Fortunella_venosa_1** **ttgtaaatccatacgactttggttcttcca-------------tttcttttttcggaacc**

**Fortunella_venosa_2** **ttgtaaatccatacgactttggttcttcca-------------tttcttttttcggaacc**

**Fortunella_venosa_3** **ttgtaaatccatacgactttggttcttcca-------------tttcttttttcggaacc**

15730	15740	15750	15760	15770	15780

....|....|....|....|....|....|....|....|....|....|....|....|

**Atalantia_kwangtungens** **cttttttgaattcttcgttcgttttctttctttcatctctttagttttattgattcaatt**

**Citrus_reticulata_1** **cttttttgaattcttcgttcgttttctttctttcatctctttatttttattgattcaatt**

**Citrus_reticulata_2** **cttttttgaattcttcgttcgttttctttctttcatctctttatttttattgattcaatt**

**Citrus_trifoliata_3** **cttttttgaattcttcgttcgttttctttctttcatctctttatttttattgattcaatt**

**Citrus_aurantiifolia** **cttttttgaattcttcgttcgttttctttctttcatctctttatttttattgattcaatt**

**Citrus_aurantium** **cttttttgaattcttcgttcgttttctttctttcatctctttatttttattgattcaatt**

**Citrus_cavaleriei** **cttttttgagttcttcgttcgttttctttctttcatctctttatttttattgattcaatt**

**Citrus_hongheensis** **cttttttgaattcttcgttcgttttctttctttcatctctttatttttattgattcaatt**

**Citrus_limon** **cttttttgaattcttcgttcgttttctttctttcatctctttatttttattgattcaatt**

**Citrus_maxima** **cttttttgaattcttcgttcgttttctttctttcatctctttatttttattgattcaatt**

**Citrus_medica** **cttttttgaattcttcgttcgttttctttctttcatctctttatttttattgattcaatt**

**Citrus_polytrifolia** **cttttttgaattcttcgttcgttttctttctttcatctctttatttttattgattcaatt**

**Citrus_reticulata** **cttttttgaattcttcgttcgttttctttctttcatctctttatttttattgattcaatt**

**Citrus_sinensis** **cttttttgaattcttcgttcgttttctttctttcatctctttatttttattgattcaatt**

**Fortunella_bawangica_1** **cttttttgaattcttcgttcgttttctttctttcatctctttatttttattgattcaatt**

**Fortunella_bawangica_2** **cttttttgaattcttcgttcgttttctttctttcatctctttatttttattgattcaatt**

**Fortunella_hindsii_1** **cttttttgaattcttcgttcgttttctttctttcatctctttatttttattgattcaatt**

**Fortunella_hindsii_2** **cttttttgaattcttcgttcgttttctttctttcatctctttatttttattgattcaatt**

**Fortunella_hindsii_3** **ctttgttgaattcttcgttcgttttctttctttcatctctttatttttattgattcaatt**

**Fortunella_japonica_1** **cttttttgaattcttcgttcgttttctttctttcatctctttatttttattgattcaatt**

**Fortunella_japonica_2** **cttttttgaattcttcgttcgttttctttctttcatctctttatttttattgattcaatt**

**Fortunella_japonica_3** **cttttttgaattcttcgttcgttttctttctttcatctctttatttttattgattcaatt**

**Fortunella_japonica_4** **cttttttgaattcttcgttcgttttctttctttcatctctttatttttattgattcaatt**

**Fortunella_japonica_5** **cttttttgaattcttcgttcgttttctttctttcatctctttatttttattgattcaatt**

**Fortunella_japonica_6** **cttttttgaattcttcgttcgttttctttctttcatctctttatttttattgattcaatt**

**Fortunella_japonica_7** **cttttttgaattcttcgttcgttttctttctttcatctctttatttttattgattcaatt**

**Fortunella_japonica_8** **cttttttgaattcttcgttcgttttctttctttcatctctttatttttattgattcaatt**

**Fortunella_japonica_9** **cttttttgaattcttcgttcgttttctttctttcatctctttatttttattgattcaatt**

**Fortunella_japonica_10** **cttttttgaattcttcgttcgttttctttctttcatctctttatttttattgattcaatt**

**Fortunella_japonica_11** **cttttttgaattcttcgttcgttttctttctttcatctctttatttttattgattcaatt**

**Fortunella_japonica_12** **cttttttgaattcttcgttcgttttctttctttcatctctttatttttattgattcaatt**

**Fortunella_margarita_1** **cttttttgaattcttcgttcgttttctttctttcatctctttatttttattgattcaatt**

**Fortunella_margarita_2** **cttttttgaattcttcgttcgttttctttctttcatctctttatttttattgattcaatt**

**Fortunella_venosa_1** **cttttttgaattcttcgttcgttttctttctttcatctctttatttttattgattcaatt**

**Fortunella_venosa_2** **cttttttgaattcttcgttcgttttctttctttcatctctttatttttattgattcaatt**

**Fortunella_venosa_3** **cttttttgaattcttcgttcgttttctttctttcatctctttatttttattgattcaatt**

15790	15800	15810	15820	15830	15840

....|....|....|....|....|....|....|....|....|....|....|....|

**Atalantia_kwangtungens** **cccag--------tcaaagcaaatacgaaatcgaactattt--------ctattagaatc**

**Citrus_reticulata_1** **cccagtcaaagcatcaaagcaaagacgaaatcgaacaattt--------ctattagaatc**

**Citrus_reticulata_2** **cccagtcaaagcatcaaagcaaagacgaaatcgaacaattt--------ctattagaatc**

**Citrus_trifoliata_3** **cccagtcaaagcatcaaagcaaagacgaaatcgaacaattt--------cgattagaatc**

**Citrus_aurantiifolia** **cccagtcaaagcatcaaagcaaagacgaaatcgaacaatttctaaatttctattagaatc**

**Citrus_aurantium** **cccagtcaaagcatcaaagcaaagacgaaatcgaacaatttctaaatttctattagaatc**

**Citrus_cavaleriei** **cccagtcaaagcatcaaagcaaagacgaaatcgaacaattt--------ctattagaatc**

**Citrus_hongheensis** **cccagtcaaagcatcaaagcaaagacgaaatcgaacaatttctaaatttctattagaatc**

**Citrus_limon** **cccagtcaaagcatcaaagcaaagacgaaatcgaacaatttctaaatttctattagaatc**

**Citrus_maxima** **cccagtcaaagcatcaaagcaaagacgaaatcgaacaatttctaaatttctattagaatc**

**Citrus_medica** **cccagtcaaagcatcaaagcaaagacgaaatcgaacaattt--------ctattagaatc**

**Citrus_polytrifolia** **cccagtcaaagcatcaaagcaaagacgaaatcgaacaattt--------cgattagaatc**

**Citrus_reticulata** **cccagtcaaagcatcaaagcaaagacgaaatcgaacaattt--------ctattagaatc**

**Citrus_sinensis** **cccagtcaaagcatcaaagcaaagacgaaatcgaacaatttctaaatttctattagaatc**

**Fortunella_bawangica_1** **cccagtcaaagcatcaaagcaaagacgaaatcgaacaattt--------ctattagaatc**

**Fortunella_bawangica_2** **cccagtcaaagcatcaaagcaaagacgaaatcgaacaattt--------ctattagaatc**

**Fortunella_hindsii_1** **cccagtcaaagcatcaaagcaaagacgaaatcgaacaattt--------ctattagaatc**

**Fortunella_hindsii_2** **cccagtcaaagcatcaaagcaaagacgaaatcgaacaattt--------ctattagaatc**

**Fortunella_hindsii_3** **cccagtcaaagcatcaaagcaaagacgaaatcgaacaattt--------ctattagaatc**

**Fortunella_japonica_1** **cccagtcaaagcatcaaagcaaagacgaaatcgaacaattt--------ctattagaatc**

**Fortunella_japonica_2** **cccagtcaaagcatcaaagcaaagacgaaatcgaacaattt--------ctattagaatc**

**Fortunella_japonica_3** **cccagtcaaagcatcaaagcaaagacgaaatcgaacaattt--------ctattagaatc**

**Fortunella_japonica_4** **cccagtcaaagcatcaaagcaaagacgaaatcgaacaattt--------ctattagaatc**

**Fortunella_japonica_5** **cccagtcaaagcatcaaagcaaagacgaaatcgaacaattt--------ctattagaatc**

**Fortunella_japonica_6** **cccagtcaaagcatcaaagcaaagacgaaatcgaacaattt--------ctattagaatc**

**Fortunella_japonica_7** **cccagtcaaagcatcaaagcaaagacgaaatcgaacaattt--------ctattagaatc**

**Fortunella_japonica_8** **cccagtcaaagcatcaaagcaaagacgaaatcgaacaattt--------ctattagaatc**

**Fortunella_japonica_9** **cccagtcaaagcatcaaagcaaagacgaaatcgaacaattt--------ctattagaatc**

**Fortunella_japonica_10** **cccagtcaaagcatcaaagcaaagacgaaatcgaacaattt--------ctattagaatc**

**Fortunella_japonica_11** **cccagtcaaagcatcaaagcaaagacgaaatcgaacaattt--------ctattagaatc**

**Fortunella_japonica_12** **cccagtcaaagcatcaaagcaaagacgaaatcgaacaattt--------ctattagaatc**

**Fortunella_margarita_1** **cccagtcaaagcatcaaagcaaagacgaaatcgaacaattt--------ctattagaatc**

**Fortunella_margarita_2** **cccagtcaaagcatcaaagcaaagacgaaatcgaacaattt--------ctattagaatc**

**Fortunella_venosa_1** **cccagtcaaagcatcaaagcaaagacgaaatcgaacaattt--------ctattagaatc**

**Fortunella_venosa_2** **cccagtcaaagcatcaaagcaaagacgaaatcgaacaattt--------ctattagaatc**

**Fortunella_venosa_3** **cccagtcaaagcatcaaagcaaagacgaaatcgaacaattt--------ctattagaatc**

15850	15860	15870	15880	15890	15900

....|....|....|....|....|....|....|....|....|....|....|....|

**Atalantia_kwangtungens** **cctatcgaaattcacaataatcacaagagtagggtggattagatatatctttagttcata**

**Citrus_reticulata_1** **cctatcgaaattcacaataatcacaagagtagggtggattagatatatctttagttcata**

**Citrus_reticulata_2** **cctatcgaaattcacaataatcacaagagtagggtggattagatatatctttagttcata**

**Citrus_trifoliata_3** **cctatcgaaattcacaataatcacaagagtagggtggattagatatatctttagttcata**

**Citrus_aurantiifolia** **cctatcgaaattcacaataatcacaagagtagggtggattagatatatctttagttcata**

**Citrus_aurantium** **cctatcgaaattcacaataatcacaagagtagggtggattagatatatctttagttcata**

**Citrus_cavaleriei** **cctatcgaaattcacaataatcacaagagtagggtggattagatatatctttagttcata**

**Citrus_hongheensis** **cctatcgaaattcacaataatcacaagagtagggtggattagatatatctttagttcata**

**Citrus_limon** **cctatcgaaattcacaataatcacaagagtagggtggattagatatatctttagttcata**

**Citrus_maxima** **cctatcgaaattcacaataatcacaagagtagggtggattagatatatctttagttcata**

**Citrus_medica** **cctatcgaaattcacaataatcacaagagtagggtggattagatatatctttagttcata**

**Citrus_polytrifolia** **cctatcgaaattcacaataatcacaagagtagggtggattagatatatctttagttcata**

**Citrus_reticulata** **cctatcgaaattcacaataatcacaagagtagggtggattagatatatctttagttcata**

**Citrus_sinensis** **cctatcgaaattcacaataatcacaagagtagggtggattagatatatctttagttcata**

**Fortunella_bawangica_1** **cctatcgaaattcacaataatcacaagagtagggtggattagatatatctttagttcata**

**Fortunella_bawangica_2** **cctatcgaaattcacaataatcacaagagtagggtggattagatatatctttagttcata**

**Fortunella_hindsii_1** **cctatcgaaattcacaataatcacaagagtagggtggattagatatatctttagttcata**

**Fortunella_hindsii_2** **cctatcgaaattcacaataatcacaagagtagggtggattagatatatctttagttcata**

**Fortunella_hindsii_3** **cctatcgaaattcacaataatcacaagagtagggtggattagatatatctttagttcata**

**Fortunella_japonica_1** **cctatcgaaattcacaataatcacaagagtagggtggattagatatatctttagttcata**

**Fortunella_japonica_2** **cctatcgaaattcacaataatcacaagagtagggtggattagatatatctttagttcata**

**Fortunella_japonica_3** **cctatcgaaattcacaataatcacaagagtagggtggattagatatatctttagttcata**

**Fortunella_japonica_4** **cctatcgaaattcacaataatcacaagagtagggtggattagatatatctttagttcata**

**Fortunella_japonica_5** **cctatcgaaattcacaataatcacaagagtagggtggattagatatatctttagttcata**

**Fortunella_japonica_6** **cctatcgaaattcacaataatcacaagagtagggtggattagatatatctttagttcata**

**Fortunella_japonica_7** **cctatcgaaattcacaataatcacaagagtagggtggattagatatatctttagttcata**

**Fortunella_japonica_8** **cctatcgaaattcacaataatcacaagagtagggtggattagatatatctttagttcata**

**Fortunella_japonica_9** **cctatcgaaattcacaataatcacaagagtagggtggattagatatatctttagttcata**

**Fortunella_japonica_10** **cctatcgaaattcacaataatcacaagagtagggtggattagatatatctttagttcata**

**Fortunella_japonica_11** **cctatcgaaattcacaataatcacaagagtagggtggattagatatatctttagttcata**

**Fortunella_japonica_12** **cctatcgaaattcacaataatcacaagagtagggtggattagatatatctttagttcata**

**Fortunella_margarita_1** **cctatcgaaattcacaataatcacaagagtagggtggattagatatatctttagttcata**

**Fortunella_margarita_2** **cctatcgaaattcacaataatcacaagagtagggtggattagatatatctttagttcata**

**Fortunella_venosa_1** **cctatcgaaattcacaataatcacaagagtagggtggattagatatatctttagttcata**

**Fortunella_venosa_2** **cctatcgaaattcacaataatcacaagagtagggtggattagatatatctttagttcata**

**Fortunella_venosa_3** **cctatcgaaattcacaataatcacaagagtagggtggattagatatatctttagttcata**

15910	15920	15930	15940	15950	15960

....|....|....|....|....|....|....|....|....|....|....|....|

**Atalantia_kwangtungens** **tataactagcaatatctaatatcccatatacatgtctttcttacagaacgtaaaccaact**

**Citrus_reticulata_1** **tataactagcaatatctaatatcccatatacatgtctttcttacagaacgtaaaccaact**

**Citrus_reticulata_2** **tataactagcaatatctaatatcccatatacatgtctttcttacagaacgtaaaccaact**

**Citrus_trifoliata_3** **tataactagcaatatctaatatcccatatacatgtctttcttacagaacgtaaaccaact**

**Citrus_aurantiifolia** **tataactagcaatatctaatatcccatatacatgtctttcttacagaacgtaaaccaact**

**Citrus_aurantium** **tataactagcaatatctaatatcccatatacatgtctttcttacagaacgtaaaccaact**

**Citrus_cavaleriei** **tataactagcaatatctaatatcccatatacatgtctttcttacagaacgtaaaccaact**

**Citrus_hongheensis** **tataactagcaatatctaatatcccatatacatgtctttcttacagaacgtaaaccaact**

**Citrus_limon** **tataactagcaatatctaatatcccatatacatgtctttcttacagaacgtaaaccaact**

**Citrus_maxima** **tataactagcaatatctaatatcccatatacatgtctttcttacagaacgtaaaccaact**

**Citrus_medica** **tataactagcaatatctaatatcccatatacatgtctttcttacagaacgtaaaccaacg**

**Citrus_polytrifolia** **tataactagcaatatctaatatcccatatacatgtctttcttacagaacgtaaaccaact**

**Citrus_reticulata** **tataactagcaatatctaatatcccatatacatgtctttcttacagaacgtaaaccaact**

**Citrus_sinensis** **tataactagcaatatctaatatcccatatacatgtctttcttacagaacgtaaaccaact**

**Fortunella_bawangica_1** **tataactagcaatatctaatatcccatatacatgtctttcttacagaacgtaaaccaact**

**Fortunella_bawangica_2** **tataactagcaatatctaatatcccatatacatgtctttcttacagaacgtaaaccaact**

**Fortunella_hindsii_1** **tataactagcaatatctaatatcccatatacatgtctttcttacagaacgtaaaccaact**

**Fortunella_hindsii_2** **tataactagcaatatctaatatcccatatacatgtctttcttacagaacgtaaaccaact**

**Fortunella_hindsii_3** **tataactagcaatatctaatatcccatatacatgtctttcttacagaacgtaaaccaact**

**Fortunella_japonica_1** **tataactagcaatatctaatatcccatatacatgtctttcttacagaacgtaaaccaact**

**Fortunella_japonica_2** **tataactagcaatatctaatatcccatatacatgtctttcttacagaacgtaaaccaact**

**Fortunella_japonica_3** **tataactagcaatatctaatatcccatatacatgtctttcttacagaacgtaaaccaact**

**Fortunella_japonica_4** **tataactagcaatatctaatatcccatatacatgtctttcttacagaacgtaaaccaact**

**Fortunella_japonica_5** **tataactagcaatatctaatatcccatatacatgtctttcttacagaacgtaaaccaact**

**Fortunella_japonica_6** **tataactagcaatatctaatatcccatatacatgtctttcttacagaacgtaaaccaact**

**Fortunella_japonica_7** **tataactagcaatatctaatatcccatatacatgtctttcttacagaacgtaaaccaact**

**Fortunella_japonica_8** **tataactagcaatatctaatatcccatatacatgtctttcttacagaacgtaaaccaact**

**Fortunella_japonica_9** **tataactagcaatatctaatatcccatatacatgtctttcttacagaacgtaaaccaact**

**Fortunella_japonica_10** **tataactagcaatatctaatatcccatatacatgtctttcttacagaacgtaaaccaact**

**Fortunella_japonica_11** **tataactagcaatatctaatatcccatatacatgtctttcttacagaacgtaaaccaact**

**Fortunella_japonica_12** **tataactagcaatatctaatatcccatatacatgtctttcttacagaacgtaaaccaact**

**Fortunella_margarita_1** **tataactagcaatatctaatatcccatatacatgtctttcttacagaacgtaaaccaact**

**Fortunella_margarita_2** **tataactagcaatatctaatatcccatatacatgtctttcttacagaacgtaaaccaact**

**Fortunella_venosa_1** **tataactagcaatatctaatatcccatatacatgtctttcttacagaacgtaaaccaact**

**Fortunella_venosa_2** **tataactagcaatatctaatatcccatatacatgtctttcttacagaacgtaaaccaact**

**Fortunella_venosa_3** **tataactagcaatatctaatatcccatatacatgtctttcttacagaacgtaaaccaact**

15970	15980	15990	16000	16010	16020

....|....|....|....|....|....|....|....|....|....|....|....|

**Atalantia_kwangtungens** **attcatatcttagactccaagtatttgtatcttaaagtcaatcgtattctagaacccctt**

**Citrus_reticulata_1** **attaatatcttagactccaagtattcgtatcttaaagtcaatcgtattctcgaacccctt**

**Citrus_reticulata_2** **attaatatcttagactccaagtattcgtatcttaaagtcaatcgtattctcgaacccctt**

**Citrus_trifoliata_3** **attaatatcttagactccaagtattcgtatcttaaagtcaatcgtattctcgaacccctt**

**Citrus_aurantiifolia** **attaatatcttagactccaagtattcgtatcttaaagtcaatcgtattctcgaacccctt**

**Citrus_aurantium** **attaatatcttagactccaagtattcgtatcttaaagtcaatcgtattctcgaacccctt**

**Citrus_cavaleriei** **attaatatcttagactccaagtattcgtatcttaaagtcaatcgtattctcgaacccctt**

**Citrus_hongheensis** **attaatatcttagactccaagtattcgtatcttaaagtcaatcgtattctcgaacccctt**

**Citrus_limon** **attaatatcttagactccaagtattcgtatcttaaagtcaatcgtattctcgaacccctt**

**Citrus_maxima** **attaatatcttagactccaagtattcgtatcttaaagtcaatcgtattctcgaacccctt**

**Citrus_medica** **attaatatcttagactccaagtattcgtatcttaaagtcaatcgtattctagaa-ccctt**

**Citrus_polytrifolia** **attaatatcttagactccaagtattcgtatcttaaagtcaatcgtattctcgaacccctt**

**Citrus_reticulata** **attaatatcttagactccaagtattcgtatcttaaagtcaatcgtattctcgaacccctt**

**Citrus_sinensis** **attaatatcttagactccaagtattcgtatcttaaagtcaatcgtattctcgaacccctt**

**Fortunella_bawangica_1** **attaatatcttagactccaagtattcgtatcttaaagtcaatcgtattctcgaacccctt**

**Fortunella_bawangica_2** **attaatatcttagactccaagtattcgtatcttaaagtcaatcgtattctcgaacccctt**

**Fortunella_hindsii_1** **attaatatcttagactccaagtattcgtatcttaaagtcaatcgtattctcgaacccctt**

**Fortunella_hindsii_2** **attaatatcttagactccaagtattcgtatcttaaagtcaatcgtattctcgaacccctt**

**Fortunella_hindsii_3** **attaatatcttagactccaagtattcgtatcttaaagtcaatcgtattctcgaacccctt**

**Fortunella_japonica_1** **attaatatcttagactccaagtattcgtatcttaaagtcaatcgtattctcgaacccctt**

**Fortunella_japonica_2** **attaatatcttagactccaagtattcgtatcttaaagtcaatcgtattctcgaacccctt**

**Fortunella_japonica_3** **attaatatcttagactccaagtattcgtatcttaaagtcaatcgtattctcgaacccctt**

**Fortunella_japonica_4** **attaatatcttagactccaagtattcgtatcttaaagtcaatcgtattctcgaacccctt**

**Fortunella_japonica_5** **attaatatcttagactccaagtattcgtatcttaaagtcaatcgtattctcgaacccctt**

**Fortunella_japonica_6** **attaatatcttagactccaagtattcgtatcttaaagtcaatcgtattctcgaacccctt**

**Fortunella_japonica_7** **attaatatcttagactccaagtattcgtatcttaaagtcaatcgtattctcgaacccctt**

**Fortunella_japonica_8** **attaatatcttagactccaagtattcgtatcttaaagtcaatcgtattctcgaacccctt**

**Fortunella_japonica_9** **attaatatcttagactccaagtattcgtatcttaaagtcaatcgtattctcgaacccctt**

**Fortunella_japonica_10** **attaatatcttagactccaagtattcgtatcttaaagtcaatcgtattctcgaacccctt**

**Fortunella_japonica_11** **attaatatcttagactccaagtattcgtatcttaaagtcaatcgtattctcgaacccctt**

**Fortunella_japonica_12** **attaatatcttagactccaagtattcgtatcttaaagtcaatcgtattctcgaacccctt**

**Fortunella_margarita_1** **attaatatcttagactccaagtattcgtatcttaaagtcaatcgtattctcgaacccctt**

**Fortunella_margarita_2** **attaatatcttagactccaagtattcgtatcttaaagtcaatcgtattctcgaacccctt**

**Fortunella_venosa_1** **attaatatcttagactccaagtattcgtatcttaaagtcaatcgtattctcgaacccctt**

**Fortunella_venosa_2** **attaatatcttagactccaagtattcgtatcttaaagtcaatcgtattctcgaacccctt**

**Fortunella_venosa_3** **attaatatcttagactccaagtattcgtatcttaaagtcaatcgtattctcgaacccctt**

16030	16040	16050	16060	16070	16080

....|....|....|....|....|....|....|....|....|....|....|....|

**Atalantia_kwangtungens** **ttcatttcaaattcaaaaatacacaagagttggcatttgattccatatacctaattatgt**

**Citrus_reticulata_1** **ttaa----aattcaaaaaatacacaagagttggcatttgattccatatacctaattatgt**

**Citrus_reticulata_2** **ttaa----aattcaaaaaatacacaagagttggcatttgattccatatacctaattatgt**

**Citrus_trifoliata_3** **ttaa----aattcaaaaaatacacaagagttggcatttgattccatatacctaattatgt**

**Citrus_aurantiifolia** **ttaa----aactcaaaaaatacacaagagttggcatttgattccatatacctaattatgt**

**Citrus_aurantium** **ttaa----aactcaaaaaatacacaagagttggcatttgattccatatacctaattatgt**

**Citrus_cavaleriei** **ttaa----aattcaaaaaatacacaagagttggcatttgattccatatacctaattatgt**

**Citrus_hongheensis** **ttaa----aactcaaaaaatacacaagagttggcatttgattccatatacctaattatgt**

**Citrus_limon** **ttaa----aactcaaaaaatacacaagagttggcatttgattccatatacctaattatgt**

**Citrus_maxima** **ttaa----aactcaaaaaatacacaagagttggcatttgattccatatacctaattatgt**

**Citrus_medica** **ttaa----aattcaaaaaatacacaagagttggcatttgattccatatacctaattatgt**

**Citrus_polytrifolia** **ttaa----aattcaaaaaatacacaagagttggcatttgattccatatacctaattatgt**

**Citrus_reticulata** **ttaa----aattcaaaaaatacacaagagttggcatttgattccatatacctaattatgt**

**Citrus_sinensis** **ttaa----aactcaaaaaatacacaagagttggcatttgattccatatacctaattatgt**

**Fortunella_bawangica_1** **ttaa----aattcaaaaaatacacaagagttggcatttgattccatatacctaattatgt**

**Fortunella_bawangica_2** **ttaa----aattcaaaaaatacacaagagttggcatttgattccatatacctaattatgt**

**Fortunella_hindsii_1** **ttaa----aattcaaaaaatacacaagagttggcatttgattccatatacctaattatgt**

**Fortunella_hindsii_2** **ttaa----aattcaaaaaatacacaagagttggcatttgattccatatacctaattatgt**

**Fortunella_hindsii_3** **ttaa----aattcaaaaaatacacaagagttggcatttgattccatatacctaattatgt**

**Fortunella_japonica_1** **ttaa----aattcaaaaaatacacaagagttggcatttgattccatatacctaattatgt**

**Fortunella_japonica_2** **ttaa----aattcaaaaaatacacaagagttggcatttgattccatatacctaattatgt**

**Fortunella_japonica_3** **ttaa----aattcaaaaaatacacaagagttggcatttgattccatatacctaattatgt**

**Fortunella_japonica_4** **ttaa----aattcaaaaaatacacaagagttggcatttgattccatatacctaattatgt**

**Fortunella_japonica_5** **ttaa----aattcaaaaaatacacaagagttggcatttgattccatatacctaattatgt**

**Fortunella_japonica_6** **ttaa----aattcaaaaaatacacaagagttggcatttgattccatatacctaattatgt**

**Fortunella_japonica_7** **ttaa----aattcaaaaaatacacaagagttggcatttgattccatatacctaattatgt**

**Fortunella_japonica_8** **ttaa----aattcaaaaaatacacaagagttggcatttgattccatatacctaattatgt**

**Fortunella_japonica_9** **ttaa----aattcaaaaaatacacaagagttggcatttgattccatatacctaattatgt**

**Fortunella_japonica_10** **ttaa----aattcaaaaaatacacaagagttggcatttgattccatatacctaattatgt**

**Fortunella_japonica_11** **ttaa----aattcaaaaaatacacaagagttggcatttgattccatatacctaattatgt**

**Fortunella_japonica_12** **ttaa----aattcaaaaaatacacaagagttggcatttgattccatatacctaattatgt**

**Fortunella_margarita_1** **ttaa----aattcaaaaaatacacaagagttggcatttgattccatatacctaattatgt**

**Fortunella_margarita_2** **ttaa----aattcaaaaaatacacaagagttggcatttgattccatatacctaattatgt**

**Fortunella_venosa_1** **ttaa----aattcaaaaaatacacaagagttggcatttgattccatatacctaattatgt**

**Fortunella_venosa_2** **ttaa----aattcaaaaaatacacaagagttggcatttgattccatatacctaattatgt**

**Fortunella_venosa_3** **ttaa----aattcaaaaaatacacaagagttggcatttgattccatatacctaattatgt**

16090	16100	16110	16120	16130	16140

....|....|....|....|....|....|....|....|....|....|....|....|

**Atalantia_kwangtungens** **ccccctcgccgaatcaccccgttttttagaaatctc--ttttttttttttatttttttct**

**Citrus_reticulata_1** **acccctcgccgaatcaccccgttttttataaatctct-ttttttttttttttttttttct**

**Citrus_reticulata_2** **acccctcgccgaatcaccccgttttttataaatctct-ttttttttttttttttttttct**

**Citrus_trifoliata_3** **acccctcgccgaatcaccccgttttttataaatctc-----tttttttttttttttttct**

**Citrus_aurantiifolia** **acccctcgccgaatcaccccgttttttagaaatctc----------ttttttttttttct**

**Citrus_aurantium** **acccctcgccgaatcaccccgttttttataaatctc---------tttttttttttttct**

**Citrus_cavaleriei** **acccctcgccgaatcaccccgttttttataaatctc--ttttttttttttttttttttct**

**Citrus_hongheensis** **acccctcgccgaatcaccccgttttttataaatctc---------tttttttttttttct**

**Citrus_limon** **acccctcgccgaatcaccccgttttttataaatctc---------tttttttttttttct**

**Citrus_maxima** **acccctcgccgaatcaccccgttttttataaatctc----------ttttttttttttct**

**Citrus_medica** **acccctcgccgaatcaccccgttttttataaatctcttttttttttttttatttttttct**

**Citrus_polytrifolia** **acccctcgccgaatcaccccgttttttataaatctc----------ttttttttttttct**

**Citrus_reticulata** **acccctcgccgaatcaccccgttttttataaatctct-ttttttttttttttttttttct**

**Citrus_sinensis** **acccctcgccgaatcaccccgttttttataaatctc----------ttttttttttttct**

**Fortunella_bawangica_1** **acccctcgccgaatcaccccgttttttataaatctc--------ttttttttttt----t**

**Fortunella_bawangica_2** **acccctcgccgaatcaccccgttttttataaatctc--------ttttttttttt----t**

**Fortunella_hindsii_1** **acccctcgccgaatcaccccgttttttataaatctc--------ttttttttttttttat**

**Fortunella_hindsii_2** **acccctcgccgaatcaccccgttttttataaatctc--------ttttttttttttttat**

**Fortunella_hindsii_3** **acccctcgccgaatcaccccgttttttataaatctc--------ttttttttttttttat**

**Fortunella_japonica_1** **acccctcgccgaatcaccccgttttttataaatctc--------tttttttttttttttt**

**Fortunella_japonica_2** **acccctcgccgaatcaccccgttttttataaatctc--------tttttttttttttttt**

**Fortunella_japonica_3** **acccctcgccgaatcaccccgttttttataaatctc--------tttttttttttttttt**

**Fortunella_japonica_4** **acccctcgccgaatcaccccgttttttataaatctc--------tttttttttttttttt**

**Fortunella_japonica_5** **acccctcgccgaatcaccccgttttttataaatctc--------tttttttttttttttt**

**Fortunella_japonica_6** **acccctcgccgaatcaccccgttttttataaatctc--------tttttttttttttttt**

**Fortunella_japonica_7** **acccctcgccgaatcaccccgttttttataaatctc--------tttttttttttttttt**

**Fortunella_japonica_8** **acccctcgccgaatcaccccgttttttataaatctc--------tttttttttttttttt**

**Fortunella_japonica_9** **acccctcgccgaatcaccccgttttttataaatctc--------tttttttttttttttt**

**Fortunella_japonica_10** **acccctcgccgaatcaccccgttttttataaatctc--------ttttttttttt----t**

**Fortunella_japonica_11** **acccctcgccgaatcaccccgttttttagaaatctc--------tttttttttttttttt**

**Fortunella_japonica_12** **acccctcgccgaatcaccccgttttttataaatctc--------tttttttttttttttt**

**Fortunella_margarita_1** **acccctcgccgaatcaccccgttttttataaatctc--------ttttttttttt----t**

**Fortunella_margarita_2** **acccctcgccgaatcaccccgttttttataaatctc--------ttttttttttt----t**

**Fortunella_venosa_1** **acccctcgccgaatcaccccgttttttataaatctc--------ttttttttttttttat**

**Fortunella_venosa_2** **acccctcgccgaatcaccccgttttttataaatctc--------ttttttttttttttat**

**Fortunella_venosa_3** **acccctcgccgaatcaccccgttttttataaatctc--------ttttttttttttttat**

16150	16160	16170	16180	16190	16200

....|....|....|....|....|....|....|....|....|....|....|....|

**Atalantia_kwangtungens** **attccttttcgttatcattatccaccaggctacaatcgaaatagacgaattcattggggc**

**Citrus_reticulata_1** **attccttttcgttatcattatccaccaggctacaatcgaaatagacgaattcattggggc**

**Citrus_reticulata_2** **attccttttcgttatcattatccaccaggctacaatcgaaatagacgaattcattggggc**

**Citrus_trifoliata_3** **attccttttcgttatcgttatccaccaggctacaatcgaaatagacgaattcattggggc**

**Citrus_aurantiifolia** **attccttttcgttatcattatccaccaggctacaatcgaaatagacgaattcattggggc**

**Citrus_aurantium** **attccttttcgttatcattatccaccaggctacaatcgaaatagacgaattcattggggc**

**Citrus_cavaleriei** **attccttttcgttatcattatccaccaggctacaatcgaaatagacgaattcattggggc**

**Citrus_hongheensis** **attccttttcgttatcattatccaccaggctacaatcgaaatagacgaattcattggggc**

**Citrus_limon** **attccttttcgttatcattatccaccaggctacaatcgaaatagacgaattcattggggc**

**Citrus_maxima** **attccttttcgttatcattatccaccaggctacaatcgaaatagacgaattcattggggc**

**Citrus_medica** **attccttttcgttatcattatccaccaggctacaatcgaaatatacgaattcattggggc**

**Citrus_polytrifolia** **attccttttcgttatcgttatccaccaggctacaatcgaaatagacgaattcattggggc**

**Citrus_reticulata** **attccttttcgttatcattatccaccaggctacaatcgaaatagacgaattcattggggc**

**Citrus_sinensis** **attccttttcgttatcattatccaccaggctacaatcgaaatagacgaattcattggggc**

**Fortunella_bawangica_1** **attccttttcgttatcattatccaccaggctacaatcgaaatagacgaattcattggggc**

**Fortunella_bawangica_2** **attccttttcgttatcattatccaccaggctacaatcgaaatagacgaattcattggggc**

**Fortunella_hindsii_1** **attccttttcgttatcattatccaccaggctacaatcgaaatagacgaattcattggggc**

**Fortunella_hindsii_2** **attccttttcgttatcattatccaccaggctacaatcgaaatagacgaattcattggggc**

**Fortunella_hindsii_3** **attccttttcgttatcattatccaccaggctacaatcgaaatagacgaattcattggggc**

**Fortunella_japonica_1** **attccttttcgttatcattatccaccaggctacaatcgaaatagacgaattcattggggc**

**Fortunella_japonica_2** **attccttttcgttatcattatccaccaggctacaatcgaaatagacgaattcattggggc**

**Fortunella_japonica_3** **attccttttcgttatcattatccaccaggctacaatcgaaatagacgaattcattggggc**

**Fortunella_japonica_4** **attccttttcgttatcattatccaccaggctacaatcgaaatagacgaattcattggggc**

**Fortunella_japonica_5** **attccttttcgttatcattatccaccaggctacaatcgaaatagacgaattcattggggc**

**Fortunella_japonica_6** **attccttttcgttatcattatccaccaggctacaatcgaaatagacgaattcattggggc**

**Fortunella_japonica_7** **attccttttcgttatcattatccaccaggctacaatcgaaatagacgaattcattggggc**

**Fortunella_japonica_8** **attccttttcgttatcattatccaccaggctacaatcgaaatagacgaattcattggggc**

**Fortunella_japonica_9** **attccttttcgttatcattatccaccaggctacaatcgaaatagacgaattcattggggc**

**Fortunella_japonica_10** **attccttttcgttatcattatccaccaggctacaatcgaaatagacgaattcattggggc**

**Fortunella_japonica_11** **attccttttcgttatcattatccaccaggctacaatcgaaatagacgaattcattggggc**

**Fortunella_japonica_12** **attccttttcgttatcattatccaccaggctacaatcgaaatagacgaattcattggggc**

**Fortunella_margarita_1** **attccttttcgttatcattatccaccaggctacaatcgaaatagacgaattcattggggc**

**Fortunella_margarita_2** **attccttttcgttatcattatccaccaggctacaatcgaaatagacgaattcattggggc**

**Fortunella_venosa_1** **attccttttcgttatcattatccaccaggctacaatcgaaatagacgaattcattggggc**

**Fortunella_venosa_2** **attccttttcgttatcattatccaccaggctacaatcgaaatagacgaattcattggggc**

**Fortunella_venosa_3** **attccttttcgttatcattatccaccaggctacaatcgaaatagacgaattcattggggc**

16210	16220	16230	16240	16250	16260

....|....|....|....|....|....|....|....|....|....|....|....|

**Atalantia_kwangtungens** **gaatcattgcatagaactaaatataagtatttgattgaggtacggccatgcaatttatta**

**Citrus_reticulata_1** **gaatcattgcatagaactaaatatcagtatttgattgaggtacggccatgcaatttatta**

**Citrus_reticulata_2** **gaatcattgcatagaactaaatatcagtatttgattgaggtacggccatgcaatttatta**

**Citrus_trifoliata_3** **gaatcattgcatagaactaaatatcagtatttgattgaggtacggccatgcaatttatta**

**Citrus_aurantiifolia** **gaatcattgcatagaactaaatatcagtatttgattgaggtacggccatgcaatttatta**

**Citrus_aurantium** **gaatcattgcatagaactaaatatcagtatttgattgaggtacggccatgcaatttatta**

**Citrus_cavaleriei** **gaatcattgcatagaactaaatatcagtatttgattgaggtacggccatgcaatttatta**

**Citrus_hongheensis** **gaatcattgcatagaactaaatatcagtatttgattgaggtacggccatgcaatttatta**

**Citrus_limon** **gaatcattgcatagaactaaatatcagtatttgattgaggtacggccatgcaatttatta**

**Citrus_maxima** **gaatcattgcatagaactaaatatcagtatttgattgaggtacggccatgcaatttatta**

**Citrus_medica** **gaatcattgcatagaactaaatatcagtatttgattgaggtacggccatgcaatttatta**

**Citrus_polytrifolia** **gaatcattgcatagaactaaatatcagtatttgattgaggtacggccatgcaatttatta**

**Citrus_reticulata** **gaatcattgcatagaactaaatatcagtatttgattgaggtacggccatgcaatttatta**

**Citrus_sinensis** **gaatcattgcatagaactaaatatcagtatttgattgaggtacggccatgcaatttatta**

**Fortunella_bawangica_1** **gaatcattgcatagaactaaatatcagtatttgattgaggtacggccatgcaatttatta**

**Fortunella_bawangica_2** **gaatcattgcatagaactaaatatcagtatttgattgaggtacggccatgcaatttatta**

**Fortunella_hindsii_1** **gaatcattgcatagaactaaatatcagtatttgattgaggtacggccatgcaatttatta**

**Fortunella_hindsii_2** **gaatcattgcatagaactaaatatcagtatttgattgaggtacggccatgcaatttatta**

**Fortunella_hindsii_3** **gaatcattgcatagaactaaatatcagtatttgattgaggtacggccatgcaatttatta**

**Fortunella_japonica_1** **gaatcattgcatagaactaaatatcagtatttgattgaggtacggccatgcaatttatta**

**Fortunella_japonica_2** **gaatcattgcatagaactaaatatcagtatttgattgaggtacggccatgcaatttatta**

**Fortunella_japonica_3** **gaatcattgcatagaactaaatatcagtatttgattgaggtacggccatgcaatttatta**

**Fortunella_japonica_4** **gaatcattgcatagaactaaatatcagtatttgattgaggtacggccatgcaatttatta**

**Fortunella_japonica_5** **gaatcattgcatagaactaaatatcagtatttgattgaggtacggccatgcaatttatta**

**Fortunella_japonica_6** **gaatcattgcatagaactaaatatcagtatttgattgaggtacggccatgcaatttatta**

**Fortunella_japonica_7** **gaatcattgcatagaactaaatatcagtatttgattgaggtacggccatgcaatttatta**

**Fortunella_japonica_8** **gaatcattgcatagaactaaatatcagtatttgattgaggtacggccatgcaatttatta**

**Fortunella_japonica_9** **gaatcattgcatagaactaaatatcagtatttgattgaggtacggccatgcaatttatta**

**Fortunella_japonica_10** **gaatcattgcatagaactaaatatcagtatttgattgaggtacggccatgcaatttatta**

**Fortunella_japonica_11** **gaatcattgcatagaactaaatatcagtatttgattgaggtacggccatgcaatttatta**

**Fortunella_japonica_12** **gaatcattgcatagaactaaatatcagtatttgattgaggtacggccatgcaatttatta**

**Fortunella_margarita_1** **gaatcattgcatagaactaaatatcagtatttgattgaggtacggccatgcaatttatta**

**Fortunella_margarita_2** **gaatcattgcatagaactaaatatcagtatttgattgaggtacggccatgcaatttatta**

**Fortunella_venosa_1** **gaatcattgcatagaactaaatatcagtatttgattgaggtacggccatgcaatttatta**

**Fortunella_venosa_2** **gaatcattgcatagaactaaatatcagtatttgattgaggtacggccatgcaatttatta**

**Fortunella_venosa_3** **gaatcattgcatagaactaaatatcagtatttgattgaggtacggccatgcaatttatta**

16270	16280	16290	16300	16310	16320

....|....|....|....|....|....|....|....|....|....|....|....|

**Atalantia_kwangtungens** **tattaatattctcttttggtcaatcgttgaattgaagaattgactaaaatcaactaaaag**

**Citrus_reticulata_1** **tattaatattctcttttggtcaatcgttgaattgaagaattgactaaaatcaactaaaag**

**Citrus_reticulata_2** **tattaatattctcttttggtcaatcgttgaattgaagaattgactaaaatcaactaaaag**

**Citrus_trifoliata_3** **tattaatattctcttttggtcaatcgttgaattgaagaattgactaaaatcaactaaaag**

**Citrus_aurantiifolia** **tattaatattctcttttggtcaatcgttgaattgaagaattgactaaaatcaactaaaag**

**Citrus_aurantium** **tattaatattctcttttggtcaatcgttgaattgaagaattgactaaaatcaactaaaag**

**Citrus_cavaleriei** **tattaatattctcttttggtcaatcgttgaattgaagaattgactaaaatcaactaaaag**

**Citrus_hongheensis** **tattaatattctcttttggtcaatcgttgaattgaagaattgactaaaatcaactaaaag**

**Citrus_limon** **tattaatattctcttttggtcaatcgttgaattgaagaattgactaaaatcaactaaaag**

**Citrus_maxima** **tattaatattctcttttggtcaatcgttgaattgaagaattgactaaaagcaactaaaag**

**Citrus_medica** **tattaatattctcttttggtcaatcgttgaattgaagaattgactaaaatcaactaaaag**

**Citrus_polytrifolia** **tattaatattctcttttggtcaatcgttgaattgaagaattgactaaactcaactaaaag**

**Citrus_reticulata** **tattaatattctcttttggtcaatcgttgaattgaagaattgactaaaatcaactaaaag**

**Citrus_sinensis** **tattaatattctcttttggtcaatcgttgaattgaagaattgactaaaatcaactaaaag**

**Fortunella_bawangica_1** **tattaatattctcttttggtcaatcgttgaattgaagaattgactaaaatcaactaaaag**

**Fortunella_bawangica_2** **tattaatattctcttttggtcaatcgttgaattgaagaattgactaaaatcaactaaaag**

**Fortunella_hindsii_1** **tattaatattctcttttggtcaatcgttgaattgaagaattgactaaaatcaactaaaag**

**Fortunella_hindsii_2** **tattaatattctcttttggtcaatcgttgaattgaagaattgactaaaatcaactaaaag**

**Fortunella_hindsii_3** **tattaatattctcttttggtcaatcgttgaattgaagaattgactaaaatcaactaaaag**

**Fortunella_japonica_1** **tattaatattctcttttggtcaatcgttgaattgaagaattgactaaaatcaactaaaag**

**Fortunella_japonica_2** **tattaatattctcttttggtcaatcgttgaattgaagaattgactaaaatcaactaaaag**

**Fortunella_japonica_3** **tattaatattctcttttggtcaatcgttgaattgaagaattgactaaaatcaactaaaag**

**Fortunella_japonica_4** **tattaatattctcttttggtcaatcgttgaattgaagaattgactaaaatcaactaaaag**

**Fortunella_japonica_5** **tattaatattctcttttggtcaatcgttgaattgaagaattgactaaaatcaactaaaag**

**Fortunella_japonica_6** **tattaatattctcttttggtcaatcgttgaattgaagaattgactaaaatcaactaaaag**

**Fortunella_japonica_7** **tattaatattctcttttggtcaatcgttgaattgaagaattgactaaaatcaactaaaag**

**Fortunella_japonica_8** **tattaatattctcttttggtcaatcgttgaattgaagaattgactaaaatcaactaaaag**

**Fortunella_japonica_9** **tattaatattctcttttggtcaatcgttgaattgaagaattgactaaaatcaactaaaag**

**Fortunella_japonica_10** **tattaatattctcttttggtcaatcgttgaattgaagaattgactaaaatcaactaaaag**

**Fortunella_japonica_11** **tattaatattctcttttggtcaatcgttgaattgaagaattgactaaaatcaactaaaag**

**Fortunella_japonica_12** **tattaatattctcttttggtcaatcgttgaattgaagaattgactaaaatcaactaaaag**

**Fortunella_margarita_1** **tattaatattctcttttggtcaatcgttgaattgaagaattgactaaaatcaactaaaag**

**Fortunella_margarita_2** **tattaatattctcttttggtcaatcgttgaattgaagaattgactaaaatcaactaaaag**

**Fortunella_venosa_1** **tattaatattctcttttggtcaatcgttgaattgaagaattgactaaaatcaactaaaag**

**Fortunella_venosa_2** **tattaatattctcttttggtcaatcgttgaattgaagaattgactaaaatcaactaaaag**

**Fortunella_venosa_3** **tattaatattctcttttggtcaatcgttgaattgaagaattgactaaaatcaactaaaag**

16330	16340	16350	16360	16370	16380

....|....|....|....|....|....|....|....|....|....|....|....|

**Atalantia_kwangtungens** **gggaatcattttataaagcatctttcttttc-------------tttttttttttttaat**

**Citrus_reticulata_1** **gggaatcattttataaagcatctttct-----------------tttctttttttttaat**

**Citrus_reticulata_2** **gggaatcattttataaagcatctttct-----------------tttctttttttttaat**

**Citrus_trifoliata_3** **gggaatcattttataaagcatctttct-----------------tttctttttttttaat**

**Citrus_aurantiifolia** **gggaatcattttataaagcatctttct-----------------tttctttttttttaat**

**Citrus_aurantium** **gggaattattttataaagcatctttct-----------------tttctttttttttaat**

**Citrus_cavaleriei** **gggaatcattttataaagcatctttct-----------------tttctttttttttaat**

**Citrus_hongheensis** **gggaattattttataaagcatctttct-----------------tttctttttttttaat**

**Citrus_limon** **gggaattattttataaagcatctttct-----------------tttctttttttttaat**

**Citrus_maxima** **gggaattattttataaagcatctttct-----------------tttctttttttttaat**

**Citrus_medica** **gggaatctttttataaagcatctttctt----------------ttcttttttttttaat**

**Citrus_polytrifolia** **gggaatcattttataaagcatctttct-----------------tttctttttttttaat**

**Citrus_reticulata** **gggaatcattttataaagcatctttct-----------------tttctttttttttaat**

**Citrus_sinensis** **gggaattattttataaagcatctttct-----------------tttctttttttttaat**

**Fortunella_bawangica_1** **ggaaatcattttataaagcatctttctt----------------ttcttttttttttaat**

**Fortunella_bawangica_2** **ggaaatcattttataaagcatctttctt----------------ttcttttttttttaat**

**Fortunella_hindsii_1** **ggaaatcattttataaagcatctttcttt---------------tctttttttttttaat**

**Fortunella_hindsii_2** **ggaaatcattttataaagcatctttcttt---------------tctttttttttttaat**

**Fortunella_hindsii_3** **ggaaatcattttataaagcatctttcttt---------------tctttttttttttaat**

**Fortunella_japonica_1** **ggaaatcattttataaagcatctttctt----------------ttcttttttttttaat**

**Fortunella_japonica_2** **ggaaatcattttataaagcatctttctt----------------ttcttttttttttaat**

**Fortunella_japonica_3** **ggaaatcattttataaagcatctttctt----------------ttcttttttttttaat**

**Fortunella_japonica_4** **ggaaatcattttataaagcatctttctt----------------ttcttttttttttaat**

**Fortunella_japonica_5** **ggaaatcattttataaagcatctttctt----------------ttcttttttttttaat**

**Fortunella_japonica_6** **ggaaatcattttataaagcatctttctt----------------ttcttttttttttaat**

**Fortunella_japonica_7** **ggaaatcattttataaagcatctttctt----------------ttcttttttttttaat**

**Fortunella_japonica_8** **ggaaatcattttataaagcatctttctt----------------ttcttttttttttaat**

**Fortunella_japonica_9** **ggaaatcattttataaagcatctttctt----------------ttcttttttttttaat**

**Fortunella_japonica_10** **ggaaatcattttataaagcatctttctt----------------ttcttttttttttaat**

**Fortunella_japonica_11** **ggaaatcattttataaagcatctttctt----------------ttcttttttttttaat**

**Fortunella_japonica_12** **ggaaatcattttataaagcatctttctt----------------ttcttttttttttaat**

**Fortunella_margarita_1** **ggaaatcattttataaagcatctttctt----------------ttcttttttttttaat**

**Fortunella_margarita_2** **ggaaatcattttataaagcatctttctt----------------ttcttttttttttaat**

**Fortunella_venosa_1** **ggaaatcattttataaagcatctttcttttctttttttttttaatctttttttttttaat**

**Fortunella_venosa_2** **ggaaatcattttataaagcatctttcttt---------------tctttttttttttaat**

**Fortunella_venosa_3** **ggaaatcattttataaagcatctttcttt---------------tctttttttttttaat**

16390	16400	16410	16420	16430	16440

....|....|....|....|....|....|....|....|....|....|....|....|

**Atalantia_kwangtungens** **cacccgcctgtgagactataagaatttttattcaaattatcactcttggtatttgctatt**

**Citrus_reticulata_1** **cacccgcctgtgatactataagaatttttattcaacttatcactcttggtatttgctatt**

**Citrus_reticulata_2** **cacccgcctgtgatactataagaatttttattcaacttatcactcttggtatttgctatt**

**Citrus_trifoliata_3** **cacccgcctgtgatactataagaatttttattcaacttatcactcttggtatttgctatt**

**Citrus_aurantiifolia** **cacccgcctgtgatactataagaatttttattcaacttatcactcttggtatttgctatt**

**Citrus_aurantium** **cacccgcctgtgatactataagaatttttattcaacttatcactcttggtatttgctatt**

**Citrus_cavaleriei** **cacccgcctgtgatactataagaatttttattcaacttatcactcttggtatttgctatt**

**Citrus_hongheensis** **cacccgcctgtgatactataagaatttttattcaacttatcactcttggtatttgctatt**

**Citrus_limon** **cacccgcctgtgatactataagaatttttattcaacttatcactcttggtatttgctatt**

**Citrus_maxima** **cacccgcctgtgatactataagaatttttattcaacttatcactcttggtatttgctatt**

**Citrus_medica** **cacccgcctgtgatactataagaatttttattcaacttatcgctcttggtatttgctatt**

**Citrus_polytrifolia** **cacccgcctgtgatactataagaatttttattcaacttatcactcttggtatttgctatt**

**Citrus_reticulata** **cacccgcctgtgatactataagaatttttattcaacttatcactcttggtatttgctatt**

**Citrus_sinensis** **cacccgcctgtgatactataagaatttttattcaacttatcactcttggtatttgctatt**

**Fortunella_bawangica_1** **cacccgcctgtgatactataagcatttttattcaacttatccctcttggtatttgctatt**

**Fortunella_bawangica_2** **cacccgcctgtgatactataagcatttttattcaacttatccctcttggtatttgctatt**

**Fortunella_hindsii_1** **cacccgcctgtgatactataagcatttttattcaacttatccctcttggtatttgctatt**

**Fortunella_hindsii_2** **cacccgcctgtgatactataagcatttttattcaacttatccctcttggtatttgctatt**

**Fortunella_hindsii_3** **cacccgcctgtgatactataagcatttttattcaacttatccctcttggtatttgctatt**

**Fortunella_japonica_1** **cacccgcctgtgatactataagcatttttattcaacttatccctcttggtatttgctatt**

**Fortunella_japonica_2** **cacccgcctgtgatactataagcatttttattcaacttatccctcttggtatttgctatt**

**Fortunella_japonica_3** **cacccgcctgtgatactataagcatttttattcaacttatccctcttggtatttgctatt**

**Fortunella_japonica_4** **cacccgcctgtgatactataagcatttttattcaacttatccctcttggtatttgctatt**

**Fortunella_japonica_5** **cacccgcctgtgatactataagcatttttattcaacttatccctcttggtatttgctatt**

**Fortunella_japonica_6** **cacccgcctgtgatactataagcatttttattcaacttatccctcttggtatttgctatt**

**Fortunella_japonica_7** **cacccgcctgtgatactataagcatttttattcaacttatccctcttggtatttgctatt**

**Fortunella_japonica_8** **cacccgcctgtgatactataagcatttttattcaacttatccctcttggtatttgctatt**

**Fortunella_japonica_9** **cacccgcctgtgatactataagcatttttattcaacttatccctcttggtatttgctatt**

**Fortunella_japonica_10** **cacccgcctgtgatactataagcatttttattcaacttatccctcttggtatttgctatt**

**Fortunella_japonica_11** **cacccgcctgtgatactataagcatttttattcaacttatccctcttggtatttgctatt**

**Fortunella_japonica_12** **cacccgcctgtgatactataagcatttttattcaacttatccctcttggtatttgctatt**

**Fortunella_margarita_1** **cacccgcctgtgatactataagcatttttattcaacttatccctcttggtatttgctatt**

**Fortunella_margarita_2** **cacccgcctgtgatactataagcatttttattcaacttatccctcttggtatttgctatt**

**Fortunella_venosa_1** **cacccgcctgtgatactataagcatttttattcaacttatccctcttggtatttgctatt**

**Fortunella_venosa_2** **cacccgcctgtgatactataagcatttttattcaacttatccctcttggtatttgctatt**

**Fortunella_venosa_3** **cacccgcctgtgatactataagcatttttattcaacttatccctcttggtatttgctatt**

16450	16460	16470	16480	16490	16500

....|....|....|....|....|....|....|....|....|....|....|....|

**Atalantia_kwangtungens** **cgaattgaatgaacaaaaatgaacaaaacaatataaagaaaatattaattggcggggaga**

**Citrus_reticulata_1** **cgaattgaatgaacaaaaatgaacaaaacaatataaagaaaatattaattggcggggaga**

**Citrus_reticulata_2** **cgaattgaatgaacaaaaatgaacaaaacaatataaagaaaatattaattggcggggaga**

**Citrus_trifoliata_3** **cgaattgaatgaacaaaaatgaacaaaacaatataaagaaaatattaattggcggggcga**

**Citrus_aurantiifolia** **cgaattgaatgaacaaaaatgaacaaaacaatataaagaaaatattaattggcggggaga**

**Citrus_aurantium** **cgaattgaatgaacaaaaatgaacaaaacaatataaagaaaatattaattggcggggaga**

**Citrus_cavaleriei** **cgaattgaatgaacaaaaatgaacaaaacaatataaagaaaatattaattggcggggaga**

**Citrus_hongheensis** **cgaattgaatgaacaaaaatgaacaaaacaatataaagaaaatattaattggcggggaga**

**Citrus_limon** **cgaattgaatgaacaaaaatgaacaaaacaatataaagaaaatattaattggcggggaga**

**Citrus_maxima** **cgaattgaatgaacaaaaatgaacaaaacaatataaagaaaatattaattggcggggaga**

**Citrus_medica** **cgaattgaatgaacaaaaatgaacaaaacaatataaatcaaatattaattggcggggaga**

**Citrus_polytrifolia** **cgaattgaatgaacaaaaatgaacaaaacaatataaagaaaatattaattggcggggcga**

**Citrus_reticulata** **cgaattgaatgaacaaaaatgaacaaaacaatataaagaaaatattaattggcggggaga**

**Citrus_sinensis** **cgaattgaatgaacaaaaatgaacaaaacaatataaagaaaatattaattggcggggaga**

**Fortunella_bawangica_1** **cgaattgaatgaacaaaaatgaacaaaacaatataaagaaaatattaattggcggggaga**

**Fortunella_bawangica_2** **cgaattgaatgaacaaaaatgaacaaaacaatataaagaaaatattaattggcggggaga**

**Fortunella_hindsii_1** **cgaattgaatgaacaaaaatgaacaaaacaatataaagaaaatattaattggcggggaga**

**Fortunella_hindsii_2** **cgaattgaatgaacaaaaatgaacaaaacaatataaagaaaatattaattggcggggaga**

**Fortunella_hindsii_3** **cgaattgaatgaacaaaaatgaacaaaacaatataaagaaaatattaattggcggggaga**

**Fortunella_japonica_1** **cgaattgaatgaacaaaaatgaacaaaacaatataaagaaaatattaattggcggggaga**

**Fortunella_japonica_2** **cgaattgaatgaacaaaaatgaacaaaacaatataaagaaaatattaattggcggggaga**

**Fortunella_japonica_3** **cgaattgaatgaacaaaaatgaacaaaacaatataaagaaaatattaattggcggggaga**

**Fortunella_japonica_4** **cgaattgaatgaacaaaaatgaacaaaacaatataaagaaaatattaattggcggggaga**

**Fortunella_japonica_5** **cgaattgaatgaacaaaaatgaacaaaacaatataaagaaaatattaattggcggggaga**

**Fortunella_japonica_6** **cgaattgaatgaacaaaaatgaacaaaacaatataaagaaaatattaattggcggggaga**

**Fortunella_japonica_7** **cgaattgaatgaacaaaaatgaacaaaacaatataaagaaaatattaattggcggggaga**

**Fortunella_japonica_8** **cgaattgaatgaacaaaaatgaacaaaacaatataaagaaaatattaattggcggggaga**

**Fortunella_japonica_9** **cgaattgaatgaacaaaaatgaacaaaacaatataaagaaaatattaattggcggggaga**

**Fortunella_japonica_10** **cgaattgaatgaacaaaaatgaacaaaacaatataaagaaaatattaattggcggggaga**

**Fortunella_japonica_11** **cgaattgaatgaacaaaaatgaacaaaacaatataaagaaaatattaattggcggggaga**

**Fortunella_japonica_12** **cgaattgaatgaacaaaaatgaacaaaacaatataaagaaaatattaattggcggggaga**

**Fortunella_margarita_1** **cgaattgaatgaacaaaaatgaacaaaacaatataaagaaaatattaattggcggggaga**

**Fortunella_margarita_2** **cgaattgaatgaacaaaaatgaacaaaacaatataaagaaaatattaattggcggggaga**

**Fortunella_venosa_1** **cgaattgaatgaacaaaaatgaacaaaacaatataaagaaaatattaattggcggggaga**

**Fortunella_venosa_2** **cgaattgaatgaacaaaaatgaacaaaacaatataaagaaaatattaattggcggggaga**

**Fortunella_venosa_3** **cgaattgaatgaacaaaaatgaacaaaacaatataaagaaaatattaattggcggggaga**

16510	16520	16530	16540	16550	16560

....|....|....|....|....|....|....|....|....|....|....|....|

**Atalantia_kwangtungens** **ggggggggggatgatataataaggccaaagaggaatttgaggaaaaagatcctttagatc**

**Citrus_reticulata_1** **---gggggggatgatataataaggccaaagaggaatttgaggaaaaagatcctttagatc**

**Citrus_reticulata_2** **---gggggggatgatataataaggccaaagaggaatttgaggaaaaagatcctttagatc**

**Citrus_trifoliata_3** **---gggggggatgatataataaggccaaagaggaatttgaggaaaaagatcctttagatc**

**Citrus_aurantiifolia** **---gggggggatgatataataaggccaaagaggaatttgaggaaaaagatcctttagatc**

**Citrus_aurantium** **---gggggggatgatataataaggccaaagaggaatttgaggaaaaagatcctttagatc**

**Citrus_cavaleriei** **---gggggggatgatataataaggccaaagaggaatttgaggaaaaagatcctttagatc**

**Citrus_hongheensis** **---gggggggatgatataataaggccaaagaggaatttgaggaaaaagatcctttagatc**

**Citrus_limon** **---gggggggatgatataataaggccaaagaggaatttgaggaaaaagatcctttagatc**

**Citrus_maxima** **---gggggggatgatataataaggccaaagaggaatttgaggaaaaagatcctttagatc**

**Citrus_medica** **---gggggggatgatataataaggccaaagaggaatttgaggaaaaagatcctttagatc**

**Citrus_polytrifolia** **---gggggggatgatataataaggccaaagaggaatttgaggaaaaagatcctttagatc**

**Citrus_reticulata** **---gggggggatgatataataaggccaaagaggaatttgaggaaaaagatcctttagatc**

**Citrus_sinensis** **---gggggggatgatataataaggccaaagaggaatttgaggaaaaagatcctttagatc**

**Fortunella_bawangica_1** **--ggggggggatgatataataaggccaaagaggaatttgaggaaaaagatcctttagatc**

**Fortunella_bawangica_2** **--ggggggggatgatataataaggccaaagaggaatttgaggaaaaagatcctttagatc**

**Fortunella_hindsii_1** **--ggggggggatgatataataaggccaaagaggaatttgaggaaaaagatcctttagatc**

**Fortunella_hindsii_2** **--ggggggggatgatataataaggccaaagaggaatttgaggaaaaagatcctttagatc**

**Fortunella_hindsii_3** **--ggggggggatgatataataaggccaaagaggaatttgaggaaaaagatcctttagatc**

**Fortunella_japonica_1** **--ggggggggatgatataataaggccaaagaggaatttgaggaaaaagatcctttagatc**

**Fortunella_japonica_2** **--ggggggggatgatataataaggccaaagaggaatttgaggaaaaagatcctttagatc**

**Fortunella_japonica_3** **--ggggggggatgatataataaggccaaagaggaatttgaggaaaaagatcctttagatc**

**Fortunella_japonica_4** **--ggggggggatgatataataaggccaaagaggaatttgaggaaaaagatcctttagatc**

**Fortunella_japonica_5** **--ggggggggatgatataataaggccaaagaggaatttgaggaaaaagatcctttagatc**

**Fortunella_japonica_6** **--ggggggggatgatataataaggccaaagaggaatttgaggaaaaagatcctttagatc**

**Fortunella_japonica_7** **--ggggggggatgatataataaggccaaagaggaatttgaggaaaaagatcctttagatc**

**Fortunella_japonica_8** **--ggggggggatgatataataaggccaaagaggaatttgaggaaaaagatcctttagatc**

**Fortunella_japonica_9** **--ggggggggatgatataataaggccaaagaggaatttgaggaaaaagatcctttagatc**

**Fortunella_japonica_10** **--ggggggggatgatataataaggccaaagaggaatttgaggaaaaagatcctttagatc**

**Fortunella_japonica_11** **--ggggggggatgatataataaggccaaagaggaatttgaggaaaaagatcctttagatc**

**Fortunella_japonica_12** **--ggggggggatgatataataaggccaaagaggaatttgaggaaaaagatcctttagatc**

**Fortunella_margarita_1** **--ggggggggatgatataataaggccaaagaggaatttgaggaaaaagatcctttagatc**

**Fortunella_margarita_2** **--ggggggggatgatataataaggccaaagaggaatttgaggaaaaagatcctttagatc**

**Fortunella_venosa_1** **--ggggggggatgatataataaggccaaagaggaatttgaggaaaaagatcctttagatc**

**Fortunella_venosa_2** **--ggggggggatgatataataaggccaaagaggaatttgaggaaaaagatcctttagatc**

**Fortunella_venosa_3** **--ggggggggatgatataataaggccaaagaggaatttgaggaaaaagatcctttagatc**

16570	16580	16590	16600	16610	16620

....|....|....|....|....|....|....|....|....|....|....|....|

**Atalantia_kwangtungens** **tctttcctacaattccccaatatggtaattttttttc-----tacgactcttgatcgtat**

**Citrus_reticulata_1** **tctttcctacaattccccaatatcgtaattttttttc-----tacgactcttgatcgtat**

**Citrus_reticulata_2** **tctttcctacaattccccaatatcgtaattttttttc-----tacgactcttgatcgtat**

**Citrus_trifoliata_3** **tctttcctacaattccccaatatcgtaattttttttc-----tacgactcttgatcgtat**

**Citrus_aurantiifolia** **tctttcctacaattccccaatatcgtaattttttttc-----tacgactcttgatcgtat**

**Citrus_aurantium** **tctttcctacaattccccaatatcgtaattttttttc-----tacgactcttgatcgtat**

**Citrus_cavaleriei** **tctttcctacaattccccaatatcgtaattttttttc-----tacgactcttgatcgtat**

**Citrus_hongheensis** **tctttcctacaattccccaatatcgtaattttttttc-----tacgactcttgatcgtat**

**Citrus_limon** **tctttcctacaattccccaatatcgtaattttttttc-----tacgactcttgatcgtat**

**Citrus_maxima** **tctttcctacaattccccaatatcgtaattttttttc-----tacgactcttgatcgtat**

**Citrus_medica** **tctttcctacaattccccaatatcgtaatttttttttttttctacgactcttgatcgtat**

**Citrus_polytrifolia** **tctttcctacaattccccaatatcgtaattttttttc-----tacgactcttgatcgtat**

**Citrus_reticulata** **tctttcctacaattccccaatatcgtaattttttttc-----tacgactcttgatcgtat**

**Citrus_sinensis** **tctttcctacaattccccaatatcgtaattttttttc-----tacgactcttgatcgtat**

**Fortunella_bawangica_1** **tctttcctacaattccccaatatcgtaattttttttt----atacgactcttgatcgtat**

**Fortunella_bawangica_2** **tctttcctacaattccccaatatcgtaattttttttt----atacgactcttgatcgtat**

**Fortunella_hindsii_1** **tctttcctacaattccccaatatcgtaattttttttt----atacgactcttgatcgtat**

**Fortunella_hindsii_2** **tctttcctacaattccccaatatcgtaattttttttt----atacgactcttgatcgtat**

**Fortunella_hindsii_3** **tctttcctacaattccccaatatcgtaattttttttt----atacgactcttgatcgtat**

**Fortunella_japonica_1** **tctttcctacaattccccaatatcgtaattttttttt----atacgactcttgatcgtat**

**Fortunella_japonica_2** **tctttcctacaattccccaatatcgtaattttttttt----atacgactcttgatcgtat**

**Fortunella_japonica_3** **tctttcctacaattccccaatatcgtaattttttttt----atacgactcttgatcgtat**

**Fortunella_japonica_4** **tctttcctacaattccccaatatcgtaattttttttt----atacgactcttgatcgtat**

**Fortunella_japonica_5** **tctttcctacaattccccaatatcgtaattttttttt----atacgactcttgatcgtat**

**Fortunella_japonica_6** **tctttcctacaattccccaatatcgtaattttttttt----atacgactcttgatcgtat**

**Fortunella_japonica_7** **tctttcctacaattccccaatatcgtaattttttttt----atacgactcttgatcgtat**

**Fortunella_japonica_8** **tctttcctacaattccccaatatcgtaattttttttt----atacgactcttgatcgtat**

**Fortunella_japonica_9** **tctttcctacaattccccaatatcgtaattttttttt----atacgactcttgatcgtat**

**Fortunella_japonica_10** **tctttcctacaattccccaatatcgtaattttttttt----atacgactcttgatcgtat**

**Fortunella_japonica_11** **tctttcctacaattccccaatatcgtaattttttttt----atacgactcttgatcgtat**

**Fortunella_japonica_12** **tctttcctacaattccccaatatcgtaattttttttt----atacgactcttgatcgtat**

**Fortunella_margarita_1** **tctttcctacaattccccaatatcgtaattttttttt----atacgactcttgatcgtat**

**Fortunella_margarita_2** **tctttcctacaattccccaatatcgtaattttttttt----atacgactcttgatcgtat**

**Fortunella_venosa_1** **tctttcctacaattccccaatatcgtaattttttttt----atacgactcttgatcgtat**

**Fortunella_venosa_2** **tctttcctacaattccccaatatcgtaattttttttt----atacgactcttgatcgtat**

**Fortunella_venosa_3** **tctttcctacaattccccaatatcgtaattttttttt----atacgactcttgatcgtat**

16630	16640	16650	16660	16670	16680

....|....|....|....|....|....|....|....|....|....|....|....|

**Atalantia_kwangtungens** **atgctgtatttgtagatttatgtttggatatgtatttttcct------------aagtat**

**Citrus_reticulata_1** **atgcggtatttgtagatttatgtttggatatgtatttttcct------aagtagaagtat**

**Citrus_reticulata_2** **atgcggtatttgtagatttatgtttggatatgtatttttcct------aagtagaagtat**

**Citrus_trifoliata_3** **atgcggtatttgtagatttatgtttggatatgtatttttcct------aagtagaagtat**

**Citrus_aurantiifolia** **atgcggtatttgtagatttatgtttggatatgtatttttcct------aagtagaagtat**

**Citrus_aurantium** **atgcggtatttgtagatttatgtttggatatgtatttttcct------aagtagaagtat**

**Citrus_cavaleriei** **atgcggtatttgtagatttatgtttggatatgtatttttcct------aagtagaagtat**

**Citrus_hongheensis** **atgcggtatttgtagatttatgtttggatatgtatttttcct------aagtagaagtat**

**Citrus_limon** **atgcggtatttgtagatttatgtttggatatgtatttttcct------aagtagaagtat**

**Citrus_maxima** **atgcggtatttgtagatttatgtttggatatgtatttttcct------aagtagaagtat**

**Citrus_medica** **atgcggtatttgtagatttatgtttggatatgtatttttcct------aagtagaagtat**

**Citrus_polytrifolia** **atgcggtatttgtagatttatgtttggatatgtatttttcctaagtagaagtagaagtat**

**Citrus_reticulata** **atgcggtatttgtagatttatgtttggatatgtatttttcct------aagtagaagtat**

**Citrus_sinensis** **atgcggtatttgtagatttatgtttggatatgtatttttcct------aagtagaagtat**

**Fortunella_bawangica_1** **atgcggtatttgtagatttatgtttggatatgtatttttcct------aagtagaagtat**

**Fortunella_bawangica_2** **atgcggtatttgtagatttatgtttggatatgtatttttcct------aagtagaagtat**

**Fortunella_hindsii_1** **atgcggtatttgtagatttatgtttggatatgtatttttcct------aagtagaagtat**

**Fortunella_hindsii_2** **atgcggtatttgtagatttatgtttggatatgtatttttcct------aagtagaagtat**

**Fortunella_hindsii_3** **atgcggtatttgtagatttatgtttggatatgtatttttcct------aagtagaagtat**

**Fortunella_japonica_1** **atgcggtatttgtagatttatgtttggatatgtatttttcct------aagtagaagtat**

**Fortunella_japonica_2** **atgcggtatttgtagatttatgtttggatatgtatttttcct------aagtagaagtat**

**Fortunella_japonica_3** **atgcggtatttgtagatttatgtttggatatgtatttttcct------aagtagaagtat**

**Fortunella_japonica_4** **atgcggtatttgtagatttatgtttggatatgtatttttcct------aagtagaagtat**

**Fortunella_japonica_5** **atgcggtatttgtagatttatgtttggatatgtatttttcct------aagtagaagtat**

**Fortunella_japonica_6** **atgcggtatttgtagatttatgtttggatatgtatttttcct------aagtagaagtat**

**Fortunella_japonica_7** **atgcggtatttgtagatttatgtttggatatgtatttttcct------aagtagaagtat**

**Fortunella_japonica_8** **atgcggtatttgtagatttatgtttggatatgtatttttcct------aagtagaagtat**

**Fortunella_japonica_9** **atgcggtatttgtagatttatgtttggatatgtatttttcct------aagtagaagtat**

**Fortunella_japonica_10** **atgcggtatttgtagatttatgtttggatatgtatttttcct------aagtagaagtat**

**Fortunella_japonica_11** **atgcggtatttgtagatttatgtttggatatgtatttttcct------aagtagaagtat**

**Fortunella_japonica_12** **atgcggtatttgtagatttatgtttggatatgtatttttcct------aagtagaagtat**

**Fortunella_margarita_1** **atgcggtatttgtagatttatgtttggatatgtatttttcct------aagtagaagtat**

**Fortunella_margarita_2** **atgcggtatttgtagatttatgtttggatatgtatttttcct------aagtagaagtat**

**Fortunella_venosa_1** **atgcggtatttgtagatttatgtttggatatgtatttttcct------aagtagaagtat**

**Fortunella_venosa_2** **atgcggtatttgtagatttatgtttggatatgtatttttcct------aagtagaagtat**

**Fortunella_venosa_3** **atgcggtatttgtagatttatgtttggatatgtatttttcct------aagtagaagtat**

16690	16700	16710	16720	16730	16740

....|....|....|....|....|....|....|....|....|....|....|....|

**Atalantia_kwangtungens** **aagtagattatcttgagttacgcatacattgcctttgttctaagctac-aaaaaaagact**

**Citrus_reticulata_1** **aagtagattatcttgagttacgcatccattgcctttgttctaagctac-aaaaaaagact**

**Citrus_reticulata_2** **aagtagattatcttgagttacgcatccattgcctttgttctaagctac-aaaaaaagact**

**Citrus_trifoliata_3** **aagtagattatcttgagttacgcatccattgcctttgttctaagctacaaaaaaaagact**

**Citrus_aurantiifolia** **aagtagattatcttgagttacgcatccattgcctttgttctaagctac-aaaaaaagact**

**Citrus_aurantium** **aagtagattatcttgagttacgcatccattgcctttgttctaagctac-aaaaaaagact**

**Citrus_cavaleriei** **aagtagattatcttgagttacgcatccattgcctttgttctaagctac-aaaaaaagact**

**Citrus_hongheensis** **aagtagattatcttgagttacgcatccattgcctttgttctaagctac-aaaaaaagact**

**Citrus_limon** **aagtagattatcttgagttacgcatccattgcctttgttctaagctac-aaaaaaagact**

**Citrus_maxima** **aagtagattatcttgagttacgcatccattgcctttgttctaagctac-aaaaaaagact**

**Citrus_medica** **aagtagattatcttgagttacgcatccattgcttttgttctaagctac-aaaaaaagact**

**Citrus_polytrifolia** **aagtagattatcttgagttacgcatccattgcctttgttctaagctacaaaaaaaagact**

**Citrus_reticulata** **aagtagattatcttgagttacgcatccattgcctttgttctaagctac-aaaaaaagact**

**Citrus_sinensis** **aagtagattatcttgagttacgcatccattgcctttgttctaagctac-aaaaaaagact**

**Fortunella_bawangica_1** **aagtagattatcttgagttacgcatccattgcctttgttctaagctac-aaaaaaagact**

**Fortunella_bawangica_2** **aagtagattatcttgagttacgcatccattgcctttgttctaagctac-aaaaaaagact**

**Fortunella_hindsii_1** **aagtagattatcttgagttacgcatccattgcctttgttctaagctac-aaaaaaagact**

**Fortunella_hindsii_2** **aagtagattatcttgagttacgcatccattgcctttgttctaagctac-aaaaaaagact**

**Fortunella_hindsii_3** **aagtagattatcttgagttacgcatccattgcctttgttctaagctac-aaaaaaagact**

**Fortunella_japonica_1** **aagtagattatcttgagttacgcatccattgcctttgttctaagctac-aaaaaaagact**

**Fortunella_japonica_2** **aagtagattatcttgagttacgcatccattgcctttgttctaagctac-aaaaaaagact**

**Fortunella_japonica_3** **aagtagattatcttgagttacgcatccattgcctttgttctaagctac-aaaaaaagact**

**Fortunella_japonica_4** **aagtagattatcttgagttacgcatccattgcctttgttctaagctac-aaaaaaagact**

**Fortunella_japonica_5** **aagtagattatcttgagttacgcatccattgcctttgttctaagctac-aaaaaaagact**

**Fortunella_japonica_6** **aagtagattatcttgagttacgcatccattgcctttgttctaagctac-aaaaaaagact**

**Fortunella_japonica_7** **aagtagattatcttgagttacgcatccattgcctttgttctaagctac-aaaaaaagact**

**Fortunella_japonica_8** **aagtagattatcttgagttacgcatccattgcctttgttctaagctac-aaaaaaagact**

**Fortunella_japonica_9** **aagtagattatcttgagttacgcatccattgcctttgttctaagctac-aaaaaaagact**

**Fortunella_japonica_10** **aagtagattatcttgagttacgcatccattgcctttgttctaagctac-aaaaaaagact**

**Fortunella_japonica_11** **aagtagattatcttgagttacgcatccattgcctttgttctaagctac-aaaaaaagact**

**Fortunella_japonica_12** **aagtagattatcttgagttacgcatccattgcctttgttctaagctac-aaaaaaagact**

**Fortunella_margarita_1** **aagtagattatcttgagttacgcatccattgcctttgttctaagctac-aaaaaaagact**

**Fortunella_margarita_2** **aagtagattatcttgagttacgcatccattgcctttgttctaagctac-aaaaaaagact**

**Fortunella_venosa_1** **aagtagattatcttgagttacgcatccattgcctttgttctaagctac-aaaaaaagact**

**Fortunella_venosa_2** **aagtagattatcttgagttacgcatccattgcctttgttctaagctac-aaaaaaagact**

**Fortunella_venosa_3** **aagtagattatcttgagttacgcatccattgcctttgttctaagctac-aaaaaaagact**

16750	16760	16770	16780	16790	16800

....|....|....|....|....|....|....|....|....|....|....|....|

**Atalantia_kwangtungens** **atttcgaaaacgagtcaatgatgtccctccatagattcgcctatataagccgcagctaaa**

**Citrus_reticulata_1** **atttcgaaaacgagtcaatgatgtccctccatagattcgcctatataagccgcagctaaa**

**Citrus_reticulata_2** **atttcgaaaacgagtcaatgatgtccctccatagattcgcctatataagccgcagctaaa**

**Citrus_trifoliata_3** **atttcgaaaacgagtcaatgatgtccctccatagattcgcctatataagccgcagctaaa**

**Citrus_aurantiifolia** **atttcgaaaacgagtcaatgatgtccctccatagattcgcctatataagccgcagctaaa**

**Citrus_aurantium** **atttcgaaaacgagtcaatgatgtccctccatagattcgcctatataagccgcagctaaa**

**Citrus_cavaleriei** **atttcgaaaacgagtcaatgatgtccctccatagattcgcctatataagccgcagctaaa**

**Citrus_hongheensis** **atttcgaaaacgagtcaatgatgtccctccatagattcgcctatataagccgcagctaaa**

**Citrus_limon** **atttcgaaaacgagtcaatgatgtccctccatagattcgcctatataagccgcagctaaa**

**Citrus_maxima** **atttcgaaaacgagtcaatgatgtccctccatagattcgcctatataagccgcagctaaa**

**Citrus_medica** **atttcgaaaacgagtcaatgatgtccctccatagattcgcctatataagccgcagctaaa**

**Citrus_polytrifolia** **atttcgaaaacgagtcaatgatggccctccatagattcgcctatataagccgcagctaaa**

**Citrus_reticulata** **atttcgaaaacgagtcaatgatgtccctccatagattcgcctatataagccgcagctaaa**

**Citrus_sinensis** **atttcgaaaacgagtcaatgatgtccctccatagattcgcctatataagccgcagctaaa**

**Fortunella_bawangica_1** **atttcgaaaacgagtcaatgatgtccctccatagattcgcctatataagccgcagctaaa**

**Fortunella_bawangica_2** **atttcgaaaacgagtcaatgatgtccctccatagattcgcctatataagccgcagctaaa**

**Fortunella_hindsii_1** **atttcgaaaacgagtcaatgatgtccctccatagattcgcctatataagccgcagctaaa**

**Fortunella_hindsii_2** **atttcgaaaacgagtcaatgatgtccctccatagattcgcctatataagccgcagctaaa**

**Fortunella_hindsii_3** **atttcgaaaacgagtcaatgatgtccctccatagattcgcctatataagccgcagctaaa**

**Fortunella_japonica_1** **atttcgaaaacgagtcaatgatgtccctccatagattcgcctatataagccgcagctaaa**

**Fortunella_japonica_2** **atttcgaaaacgagtcaatgatgtccctccatagattcgcctatataagccgcagctaaa**

**Fortunella_japonica_3** **atttcgaaaacgagtcaatgatgtccctccatagattcgcctatataagccgcagctaaa**

**Fortunella_japonica_4** **atttcgaaaacgagtcaatgatgtccctccatagattcgcctatataagccgcagctaaa**

**Fortunella_japonica_5** **atttcgaaaacgagtcaatgatgtccctccatagattcgcctatataagccgcagctaaa**

**Fortunella_japonica_6** **atttcgaaaacgagtcaatgatgtccctccatagattcgcctatataagccgcagctaaa**

**Fortunella_japonica_7** **atttcgaaaacgagtcaatgatgtccctccatagattcgcctatataagccgcagctaaa**

**Fortunella_japonica_8** **atttcgaaaacgagtcaatgatgtccctccatagattcgcctatataagccgcagctaaa**

**Fortunella_japonica_9** **atttcgaaaacgagtcaatgatgtccctccatagattcgcctatataagccgcagctaaa**

**Fortunella_japonica_10** **atttcgaaaacgagtcaatgatgtccctccatagattcgcctatataagccgcagctaaa**

**Fortunella_japonica_11** **atttcgaaaacgagtcaatgatgtccctccatagattcgcctatataagccgcagctaaa**

**Fortunella_japonica_12** **atttcgaaaacgagtcaatgatgtccctccatagattcgcctatataagccgcagctaaa**

**Fortunella_margarita_1** **atttcgaaaacgagtcaatgatgtccctccatagattcgcctatataagccgcagctaaa**

**Fortunella_margarita_2** **atttcgaaaacgagtcaatgatgtccctccatagattcgcctatataagccgcagctaaa**

**Fortunella_venosa_1** **atttcgaaaacgagtcaatgatgtccctccatagattcgcctatataagccgcagctaaa**

**Fortunella_venosa_2** **atttcgaaaacgagtcaatgatgtccctccatagattcgcctatataagccgcagctaaa**

**Fortunella_venosa_3** **atttcgaaaacgagtcaatgatgtccctccatagattcgcctatataagccgcagctaaa**

16810	16820	16830	16840	16850	16860

....|....|....|....|....|....|....|....|....|....|....|....|

**Atalantia_kwangtungens** **gttgcaaaaataagagcttgaataccgcttgtaaataatccaaggaacatgacgggtata**

**Citrus_reticulata_1** **gttgcaaaaataagagcttgaataccgcttgtaaataatccaaggaacatgacgggtata**

**Citrus_reticulata_2** **gttgcaaaaataagagcttgaataccgcttgtaaataatccaaggaacatgacgggtata**

**Citrus_trifoliata_3** **gttgcaaaaataagagcttgaataccgcttgtaaataatccaaggaacatgacgggtata**

**Citrus_aurantiifolia** **gttgcaaaaataagagcttgaataccgcttgtaaataatccaaggaacatgacgggtata**

**Citrus_aurantium** **gttgcaaaaataagagcttgaataccgcttgtaaataatccaaggaacatgacgggtata**

**Citrus_cavaleriei** **gttgcaaaaataagagcttgaataccgcttgtaaataatccaaggaacatgacgggtata**

**Citrus_hongheensis** **gttgcaaaaataagagcttgaataccgcttgtaaataatccaaggaacatgacgggtata**

**Citrus_limon** **gttgcaaaaataagagcttgaataccgcttgtaaataatccaaggaacatgacgggtata**

**Citrus_maxima** **gttgcaaaaataagagcttgaataccgcttgtaaataatccaaggaacatgacgggtata**

**Citrus_medica** **gttgcaaaaataagagcttgaataccgcttgtaaataatccaaggaacatgacgggtata**

**Citrus_polytrifolia** **gttgcaaaaataagagcttgaataccgcttgtaaataatccaaggaacatgacgggtata**

**Citrus_reticulata** **gttgcaaaaataagagcttgaataccgcttgtaaataatccaaggaacatgacgggtata**

**Citrus_sinensis** **gttgcaaaaataagagcttgaataccgcttgtaaataatccaaggaacatgacgggtata**

**Fortunella_bawangica_1** **gttgcaaaaataagagcttgaataccgcttgtaaataatccaaggaacatgacgggtata**

**Fortunella_bawangica_2** **gttgcaaaaataagagcttgaataccgcttgtaaataatccaaggaacatgacgggtata**

**Fortunella_hindsii_1** **gttgcaaaaataagagcttgaataccgcttgtaaataatccaaggaacatgacgggtata**

**Fortunella_hindsii_2** **gttgcaaaaataagagcttgaataccgcttgtaaataatccaaggaacatgacgggtata**

**Fortunella_hindsii_3** **gttgcaaaaataagagcttgaataccgcttgtaaataatccaaggaacatgacgggtata**

**Fortunella_japonica_1** **gttgcaaaaataagagcttgaataccgcttgtaaataatccaaggaacatgacgggtata**

**Fortunella_japonica_2** **gttgcaaaaataagagcttgaataccgcttgtaaataatccaaggaacatgacgggtata**

**Fortunella_japonica_3** **gttgcaaaaataagagcttgaataccgcttgtaaataatccaaggaacatgacgggtata**

**Fortunella_japonica_4** **gttgcaaaaataagagcttgaataccgcttgtaaataatccaaggaacatgacgggtata**

**Fortunella_japonica_5** **gttgcaaaaataagagcttgaataccgcttgtaaataatccaaggaacatgacgggtata**

**Fortunella_japonica_6** **gttgcaaaaataagagcttgaataccgcttgtaaataatccaaggaacatgacgggtata**

**Fortunella_japonica_7** **gttgcaaaaataagagcttgaataccgcttgtaaataatccaaggaacatgacgggtata**

**Fortunella_japonica_8** **gttgcaaaaataagagcttgaataccgcttgtaaataatccaaggaacatgacgggtata**

**Fortunella_japonica_9** **gttgcaaaaataagagcttgaataccgcttgtaaataatccaaggaacatgacgggtata**

**Fortunella_japonica_10** **gttgcaaaaataagagcttgaataccgcttgtaaataatccaaggaacatgacgggtata**

**Fortunella_japonica_11** **gttgcaaaaataagagcttgaataccgcttgtaaataatccaaggaacatgacgggtata**

**Fortunella_japonica_12** **gttgcaaaaataagagcttgaataccgcttgtaaataatccaaggaacatgacgggtata**

**Fortunella_margarita_1** **gttgcaaaaataagagcttgaataccgcttgtaaataatccaaggaacatgacgggtata**

**Fortunella_margarita_2** **gttgcaaaaataagagcttgaataccgcttgtaaataatccaaggaacatgacgggtata**

**Fortunella_venosa_1** **gttgcaaaaataagagcttgaataccgcttgtaaataatccaaggaacatgacgggtata**

**Fortunella_venosa_2** **gttgcaaaaataagagcttgaataccgcttgtaaataatccaaggaacatgacgggtata**

**Fortunella_venosa_3** **gttgcaaaaataagagcttgaataccgcttgtaaataatccaaggaacatgacgggtata**

16870	16880	16890	16900	16910	16920

....|....|....|....|....|....|....|....|....|....|....|....|

**Atalantia_kwangtungens** **ggaaccactaaaggtactaaagaaacaagaacaacaactactaattcatcggctaatata**

**Citrus_reticulata_1** **ggaaccactaaaggtactaaagaaacaagaacaacaactactaattcatcggctaatata**

**Citrus_reticulata_2** **ggaaccactaaaggtactaaagaaacaagaacaacaactactaattcatcggctaatata**

**Citrus_trifoliata_3** **ggaaccactaaaggtactaaagaaacaagaacaacaactactaattcatcggctaatata**

**Citrus_aurantiifolia** **ggaaccactaaaggtactaaagaaacaagaacaacaactactaattcatcggctaatata**

**Citrus_aurantium** **ggaaccactaaaggtactaaagaaacaagaacaacaactactaattcatcggctaatata**

**Citrus_cavaleriei** **ggaaccactaaaggtactaaagaaacaagaacaacaactactaattcatcggctaatata**

**Citrus_hongheensis** **ggaaccactaaaggtactaaagaaacaagaacaacaactactaattcatcggctaatata**

**Citrus_limon** **ggaaccactaaaggtactaaagaaacaagaacaacaactactaattcatcggctaatata**

**Citrus_maxima** **ggaaccactaaaggtactaaagaaacaagaacaacaactactaattcatcggctaatata**

**Citrus_medica** **ggaaccactaaaggtactaaagaaacaagaacaacaactactaattcatcggctaatata**

**Citrus_polytrifolia** **ggaaccactaaaggtactaaagaaacaagaacaacaactactaattcatcggctaatata**

**Citrus_reticulata** **ggaaccactaaaggtactaaagaaacaagaacaacaactactaattcatcggctaatata**

**Citrus_sinensis** **ggaaccactaaaggtactaaagaaacaagaacaacaactactaattcatcggctaatata**

**Fortunella_bawangica_1** **ggaaccactaaaggtactaaagaaacaagaacaacaactactaattcatcggctaatata**

**Fortunella_bawangica_2** **ggaaccactaaaggtactaaagaaacaagaacaacaactactaattcatcggctaatata**

**Fortunella_hindsii_1** **ggaaccactaaaggtactaaagaaacaagaacaacaactactaattcatcggctaatata**

**Fortunella_hindsii_2** **ggaaccactaaaggtactaaagaaacaagaacaacaactactaattcatcggctaatata**

**Fortunella_hindsii_3** **ggaaccactaaaggtactaaagaaacaagaacaacaactactaattcatcggctaatata**

**Fortunella_japonica_1** **ggaaccactaaaggtactaaagaaacaagaacaacaactactaattcatcggctaatata**

**Fortunella_japonica_2** **ggaaccactaaaggtactaaagaaacaagaacaacaactactaattcatcggctaatata**

**Fortunella_japonica_3** **ggaaccactaaaggtactaaagaaacaagaacaacaactactaattcatcggctaatata**

**Fortunella_japonica_4** **ggaaccactaaaggtactaaagaaacaagaacaacaactactaattcatcggctaatata**

**Fortunella_japonica_5** **ggaaccactaaaggtactaaagaaacaagaacaacaactactaattcatcggctaatata**

**Fortunella_japonica_6** **ggaaccactaaaggtactaaagaaacaagaacaacaactactaattcatcggctaatata**

**Fortunella_japonica_7** **ggaaccactaaaggtactaaagaaacaagaacaacaactactaattcatcggctaatata**

**Fortunella_japonica_8** **ggaaccactaaaggtactaaagaaacaagaacaacaactactaattcatcggctaatata**

**Fortunella_japonica_9** **ggaaccactaaaggtactaaagaaacaagaacaacaactactaattcatcggctaatata**

**Fortunella_japonica_10** **ggaaccactaaaggtactaaagaaacaagaacaacaactactaattcatcggctaatata**

**Fortunella_japonica_11** **ggaaccactaaaggtactaaagaaacaagaacaacaactactaattcatcggctaatata**

**Fortunella_japonica_12** **ggaaccactaaaggtactaaagaaacaagaacaacaactactaattcatcggctaatata**

**Fortunella_margarita_1** **ggaaccactaaaggtactaaagaaacaagaacaacaactactaattcatcggctaatata**

**Fortunella_margarita_2** **ggaaccactaaaggtactaaagaaacaagaacaacaactactaattcatcggctaatata**

**Fortunella_venosa_1** **ggaaccactaaaggtactaaagaaacaagaacaacaactactaattcatcggctaatata**

**Fortunella_venosa_2** **ggaaccactaaaggtactaaagaaacaagaacaacaactactaattcatcggctaatata**

**Fortunella_venosa_3** **ggaaccactaaaggtactaaagaaacaagaacaacaactactaattcatcggctaatata**

16930	16940	16950	16960	16970	16980

....|....|....|....|....|....|....|....|....|....|....|....|

**Atalantia_kwangtungens** **ttcccaaaaagtcgaaaactaagtgatagaggttttgtgaaatcttctaaaatgttaatg**

**Citrus_reticulata_1** **ttcccaaaaagtcgaaaactaagtgatagaggttttgtgaaatcttctaaaatgttaatg**

**Citrus_reticulata_2** **ttcccaaaaagtcgaaaactaagtgatagaggttttgtgaaatcttctaaaatgttaatg**

**Citrus_trifoliata_3** **ttcccaaaaagtcgaaaactaagtgatagaggttttgtgaaatcttctaaaatgttaatg**

**Citrus_aurantiifolia** **ttcccaaaaagtcgaaaactaagtgatagaggttttgtgaaatcttctaaaatgttaatg**

**Citrus_aurantium** **ttcccaaaaagtcgaaaactaagtgatagaggttttgtgaaatcttctaaaatgttaatg**

**Citrus_cavaleriei** **ttcccaaaaagtcgaaaactaagtgatagaggttttgtgaaatcttctaaaatgttaatg**

**Citrus_hongheensis** **ttcccaaaaagtcgaaaactaagtgatagaggttttgtgaaatcttctaaaatgttaatg**

**Citrus_limon** **ttcccaaaaagtcgaaaactaagtgatagaggttttgtgaaatcttctaaaatgttaatg**

**Citrus_maxima** **ttcccaaaaagtcgaaaactaagtgatagaggttttgtgaaatcttctaaaatgttaatg**

**Citrus_medica** **ttcccaaaaagtcgaaaactaagtgatagaggttttgtgaaatcttctaaaatgttaatg**

**Citrus_polytrifolia** **ttcccaaaaagtcgaaaactaagtgatagaggttttgtgaaatcttctaaaatgttaatg**

**Citrus_reticulata** **ttcccaaaaagtcgaaaactaagtgatagaggttttgtgaaatcttctaaaatgttaatg**

**Citrus_sinensis** **ttcccaaaaagtcgaaaactaagtgatagaggttttgtgaaatcttctaaaatgttaatg**

**Fortunella_bawangica_1** **ttcccaaaaagtcgaaaactaagtgatagaggttttgtgaaatcttctaaaatgttaatg**

**Fortunella_bawangica_2** **ttcccaaaaagtcgaaaactaagtgatagaggttttgtgaaatcttctaaaatgttaatg**

**Fortunella_hindsii_1** **ttcccaaaaagtcgaaaactaagtgatagaggttttgtgaaatcttctaaaatgttaatg**

**Fortunella_hindsii_2** **ttcccaaaaagtcgaaaactaagtgatagaggttttgtgaaatcttctaaaatgttaatg**

**Fortunella_hindsii_3** **ttcccaaaaagtcgaaaactaagtgatagaggttttgtgaaatcttctaaaatgttaatg**

**Fortunella_japonica_1** **ttcccaaaaagtcgaaaactaagtgatagaggttttgtgaaatcttctaaaatgttaatg**

**Fortunella_japonica_2** **ttcccaaaaagtcgaaaactaagtgatagaggttttgtgaaatcttctaaaatgttaatg**

**Fortunella_japonica_3** **ttcccaaaaagtcgaaaactaagtgatagaggttttgtgaaatcttctaaaatgttaatg**

**Fortunella_japonica_4** **ttcccaaaaagtcgaaaactaagtgatagaggttttgtgaaatcttctaaaatgttaatg**

**Fortunella_japonica_5** **ttcccaaaaagtcgaaaactaagtgatagaggttttgtgaaatcttctaaaatgttaatg**

**Fortunella_japonica_6** **ttcccaaaaagtcgaaaactaagtgatagaggttttgtgaaatcttctaaaatgttaatg**

**Fortunella_japonica_7** **ttcccaaaaagtcgaaaactaagtgatagaggttttgtgaaatcttctaaaatgttaatg**

**Fortunella_japonica_8** **ttcccaaaaagtcgaaaactaagtgatagaggttttgtgaaatcttctaaaatgttaatg**

**Fortunella_japonica_9** **ttcccaaaaagtcgaaaactaagtgatagaggttttgtgaaatcttctaaaatgttaatg**

**Fortunella_japonica_10** **ttcccaaaaagtcgaaaactaagtgatagaggttttgtgaaatcttctaaaatgttaatg**

**Fortunella_japonica_11** **ttcccaaaaagtcgaaaactaagtgatagaggttttgtgaaatcttctaaaatgttaatg**

**Fortunella_japonica_12** **ttcccaaaaagtcgaaaactaagtgatagaggttttgtgaaatcttctaaaatgttaatg**

**Fortunella_margarita_1** **ttcccaaaaagtcgaaaactaagtgatagaggttttgtgaaatcttctaaaatgttaatg**

**Fortunella_margarita_2** **ttcccaaaaagtcgaaaactaagtgatagaggttttgtgaaatcttctaaaatgttaatg**

**Fortunella_venosa_1** **ttcccaaaaagtcgaaaactaagtgatagaggttttgtgaaatcttctaaaatgttaatg**

**Fortunella_venosa_2** **ttcccaaaaagtcgaaaactaagtgatagaggttttgtgaaatcttctaaaatgttaatg**

**Fortunella_venosa_3** **ttcccaaaaagtcgaaaactaagtgatagaggttttgtgaaatcttctaaaatgttaatg**

16990	17000	17010	17020	17030	17040

....|....|....|....|....|....|....|....|....|....|....|....|

**Atalantia_kwangtungens** **ggtaaaagaattggagtcggttgaatgtatttactgaaatagcctaatccctttttggaa**

**Citrus_reticulata_1** **ggtaaaagaattggagtcggttgaatgtatttactgaaatagcctaatccctttttggaa**

**Citrus_reticulata_2** **ggtaaaagaattggagtcggttgaatgtatttactgaaatagcctaatccctttttggaa**

**Citrus_trifoliata_3** **ggtaaaagaattggagtcggttgaatgtatttactgaaatagcctaatccctttttggaa**

**Citrus_aurantiifolia** **ggtaaaagaattggagtcggttgaatgtatttactgaaatagcctaatccctttttggaa**

**Citrus_aurantium** **ggtaaaagaattggagtcggttgaatgtatttactgaaatagcctaatccctttttggaa**

**Citrus_cavaleriei** **ggtaaaagaattggagtcggttgaatgtatttactgaaatagcctaatccctttttggaa**

**Citrus_hongheensis** **ggtaaaagaattggagtcggttgaatgtatttactgaaatagcctaatccctttttggaa**

**Citrus_limon** **ggtaaaagaattggagtcggttgaatgtatttactgaaatagcctaatccctttttggaa**

**Citrus_maxima** **ggtaaaagaattggagtcggttgaatgtatttactgaaatagcctaatccctttttggaa**

**Citrus_medica** **ggtaaaagaattggagtcggttgaatgtatttactgaaatagcctaatccctttttggaa**

**Citrus_polytrifolia** **ggtaaaagaattggagtcggttgaatgtatttactgaaatagcctaatccctttttggaa**

**Citrus_reticulata** **ggtaaaagaattggagtcggttgaatgtatttactgaaatagcctaatccctttttggaa**

**Citrus_sinensis** **ggtaaaagaattggagtcggttgaatgtatttactgaaatagcctaatccctttttggaa**

**Fortunella_bawangica_1** **ggtaaaagaattggagtcggttgaatgtatttactgaaatagcctaatccctttttggaa**

**Fortunella_bawangica_2** **ggtaaaagaattggagtcggttgaatgtatttactgaaatagcctaatccctttttggaa**

**Fortunella_hindsii_1** **ggtaaaagaattggagtcggttgaatgtatttactgaaatagcctaatccctttttggaa**

**Fortunella_hindsii_2** **ggtaaaagaattggagtcggttgaatgtatttactgaaatagcctaatccctttttggaa**

**Fortunella_hindsii_3** **ggtaaaagaattggagtcggttgaatgtatttactgaaatagcctaatccctttttggaa**

**Fortunella_japonica_1** **ggtaaaagaattggagtcggttgaatgtatttactgaaatagcctaatccctttttggaa**

**Fortunella_japonica_2** **ggtaaaagaattggagtcggttgaatgtatttactgaaatagcctaatccctttttggaa**

**Fortunella_japonica_3** **ggtaaaagaattggagtcggttgaatgtatttactgaaatagcctaatccctttttggaa**

**Fortunella_japonica_4** **ggtaaaagaattggagtcggttgaatgtatttactgaaatagcctaatccctttttggaa**

**Fortunella_japonica_5** **ggtaaaagaattggagtcggttgaatgtatttactgaaatagcctaatccctttttggaa**

**Fortunella_japonica_6** **ggtaaaagaattggagtcggttgaatgtatttactgaaatagcctaatccctttttggaa**

**Fortunella_japonica_7** **ggtaaaagaattggagtcggttgaatgtatttactgaaatagcctaatccctttttggaa**

**Fortunella_japonica_8** **ggtaaaagaattggagtcggttgaatgtatttactgaaatagcctaatccctttttggaa**

**Fortunella_japonica_9** **ggtaaaagaattggagtcggttgaatgtatttactgaaatagcctaatccctttttggaa**

**Fortunella_japonica_10** **ggtaaaagaattggagtcggttgaatgtatttactgaaatagcctaatccctttttggaa**

**Fortunella_japonica_11** **ggtaaaagaattggagtcggttgaatgtatttactgaaatagcctaatccctttttggaa**

**Fortunella_japonica_12** **ggtaaaagaattggagtcggttgaatgtatttactgaaatagcctaatccctttttggaa**

**Fortunella_margarita_1** **ggtaaaagaattggagtcggttgaatgtatttactgaaatagcctaatccctttttggaa**

**Fortunella_margarita_2** **ggtaaaagaattggagtcggttgaatgtatttactgaaatagcctaatccctttttggaa**

**Fortunella_venosa_1** **ggtaaaagaattggagtcggttgaatgtatttactgaaatagcctaatccctttttggaa**

**Fortunella_venosa_2** **ggtaaaagaattggagtcggttgaatgtatttactgaaatagcctaatccctttttggaa**

**Fortunella_venosa_3** **ggtaaaagaattggagtcggttgaatgtatttactgaaatagcctaatccctttttggaa**

17050	17060	17070	17080	17090	17100

....|....|....|....|....|....|....|....|....|....|....|....|

**Atalantia_kwangtungens** **agacccgcatagaagtatgctattgacgtgagcaaagctaaagcaacggtagtatttata**

**Citrus_reticulata_1** **agacccgcatagaagtatgctattgacgtgagcaaagctaaagcaacggtagtatttata**

**Citrus_reticulata_2** **agacccgcatagaagtatgctattgacgtgagcaaagctaaagcaacggtagtatttata**

**Citrus_trifoliata_3** **agacccgcatagaagtatgctattgacgtgagcaaagctaaagcaacggtagtatttata**

**Citrus_aurantiifolia** **agacccgcatagaagtatgctattgacgtgagcaaagctaaagcaacggtagtatttata**

**Citrus_aurantium** **agacccgcatagaagtatgctattgacgtgagcaaagctaaagcaacggtagtatttata**

**Citrus_cavaleriei** **agacccgcatagaagtatgctattgacgtgagcaaagctaaagcaacggtagtatttata**

**Citrus_hongheensis** **agacccgcatagaagtatgctattgacgtgagcaaagctaaagcaacggtagtatttata**

**Citrus_limon** **agacccgcatagaagtatgctattgacgtgagcaaagctaaagcaacggtagtatttata**

**Citrus_maxima** **agacccgcatagaagtatgctattgacgtgagcaaagctaaagcaacggtagtatttata**

**Citrus_medica** **agacccgcatagaagtatgctattgacgtgagcaaagctaaagcaacggtagtatttata**

**Citrus_polytrifolia** **agacccgcatagaagtatgctattgacgtgagcaaagctaaagcaacggtagtatttata**

**Citrus_reticulata** **agacccgcatagaagtatgctattgacgtgagcaaagctaaagcaacggtagtatttata**

**Citrus_sinensis** **agacccgcatagaagtatgctattgacgtgagcaaagctaaagcaacggtagtatttata**

**Fortunella_bawangica_1** **agacccgcatagaagtatgctattgacgtgagcaaagctaaagcaacggtagtatttata**

**Fortunella_bawangica_2** **agacccgcatagaagtatgctattgacgtgagcaaagctaaagcaacggtagtatttata**

**Fortunella_hindsii_1** **agacccgcatagaagtatgctattgacgtgagcaaagctaaagcaacggtagtatttata**

**Fortunella_hindsii_2** **agacccgcatagaagtatgctattgacgtgagcaaagctaaagcaacggtagtatttata**

**Fortunella_hindsii_3** **agacccgcatagaagtatgctattgacgtgagcaaagctaaagcaacggtagtatttata**

**Fortunella_japonica_1** **agacccgcatagaagtatgctattgacgtgagcaaagctaaagcaacggtagtatttata**

**Fortunella_japonica_2** **agacccgcatagaagtatgctattgacgtgagcaaagctaaagcaacggtagtatttata**

**Fortunella_japonica_3** **agacccgcatagaagtatgctattgacgtgagcaaagctaaagcaacggtagtatttata**

**Fortunella_japonica_4** **agacccgcatagaagtatgctattgacgtgagcaaagctaaagcaacggtagtatttata**

**Fortunella_japonica_5** **agacccgcatagaagtatgctattgacgtgagcaaagctaaagcaacggtagtatttata**

**Fortunella_japonica_6** **agacccgcatagaagtatgctattgacgtgagcaaagctaaagcaacggtagtatttata**

**Fortunella_japonica_7** **agacccgcatagaagtatgctattgacgtgagcaaagctaaagcaacggtagtatttata**

**Fortunella_japonica_8** **agacccgcatagaagtatgctattgacgtgagcaaagctaaagcaacggtagtatttata**

**Fortunella_japonica_9** **agacccgcatagaagtatgctattgacgtgagcaaagctaaagcaacggtagtatttata**

**Fortunella_japonica_10** **agacccgcatagaagtatgctattgacgtgagcaaagctaaagcaacggtagtatttata**

**Fortunella_japonica_11** **agacccgcatagaagtatgctattgacgtgagcaaagctaaagcaacggtagtatttata**

**Fortunella_japonica_12** **agacccgcatagaagtatgctattgacgtgagcaaagctaaagcaacggtagtatttata**

**Fortunella_margarita_1** **agacccgcatagaagtatgctattgacgtgagcaaagctaaagcaacggtagtatttata**

**Fortunella_margarita_2** **agacccgcatagaagtatgctattgacgtgagcaaagctaaagcaacggtagtatttata**

**Fortunella_venosa_1** **agacccgcatagaagtatgctattgacgtgagcaaagctaaagcaacggtagtatttata**

**Fortunella_venosa_2** **agacccgcatagaagtatgctattgacgtgagcaaagctaaagcaacggtagtatttata**

**Fortunella_venosa_3** **agacccgcatagaagtatgctattgacgtgagcaaagctaaagcaacggtagtatttata**

17110	17120	17130	17140	17150	17160

....|....|....|....|....|....|....|....|....|....|....|....|

**Atalantia_kwangtungens** **tcattcgtgggtgcggctaactccccatgaggtaactctatgattttccaaggtaaaaga**

**Citrus_reticulata_1** **tcattcgtgggtgcggctaactccccatgaggtaactctatgattttccaaggtaaaaga**

**Citrus_reticulata_2** **tcattcgtgggtgcggctaactccccatgaggtaactctatgattttccaaggtaaaaga**

**Citrus_trifoliata_3** **tcattcgtgggtgcggctaactccccatgaggtaactctatgattttccaaggtaaaaga**

**Citrus_aurantiifolia** **tcattcgtgggtgcggctaactccccatgaggtaactctatgattttccaaggtaaaaga**

**Citrus_aurantium** **tcattcgtgggtgcggctaactccccatgaggtaactctatgattttccaaggtaaaaga**

**Citrus_cavaleriei** **tcattcgtgggtgcggctaactccccatgaggtaactctatgattttccaaggtaaaaga**

**Citrus_hongheensis** **tcattcgtgggtgcggctaactccccatgaggtaactctatgattttccaaggtaaaaga**

**Citrus_limon** **tcattcgtgggtgcggctaactccccatgaggtaactctatgattttccaaggtaaaaga**

**Citrus_maxima** **tcattcgtgggtgcggctaactccccatgaggtaactctatgattttccaaggtaaaaga**

**Citrus_medica** **tcattcgtgggtgcggctaactccccatgaggtaactctatgattttccaaggtaaaaga**

**Citrus_polytrifolia** **tcattcgtgggtgcggctaactccccatgaggtaactctatgattttccaaggtaaaaga**

**Citrus_reticulata** **tcattcgtgggtgcggctaactccccatgaggtaactctatgattttccaaggtaaaaga**

**Citrus_sinensis** **tcattcgtgggtgcggctaactccccatgaggtaactctatgattttccaaggtaaaaga**

**Fortunella_bawangica_1** **tcattcgtgggtgcggctaactccccatgaggtaactctatgattttccaaggtaaaaga**

**Fortunella_bawangica_2** **tcattcgtgggtgcggctaactccccatgaggtaactctatgattttccaaggtaaaaga**

**Fortunella_hindsii_1** **tcattcgtgggtgcggctaactccccatgaggtaactctatgattttccaaggtaaaaga**

**Fortunella_hindsii_2** **tcattcgtgggtgcggctaactccccatgaggtaactctatgattttccaaggtaaaaga**

**Fortunella_hindsii_3** **tcattcgtgggtgcggctaactccccatgaggtaactctatgattttccaaggtaaaaga**

**Fortunella_japonica_1** **tcattcgtgggtgcggctaactccccatgaggtaactctatgattttccaaggtaaaaga**

**Fortunella_japonica_2** **tcattcgtgggtgcggctaactccccatgaggtaactctatgattttccaaggtaaaaga**

**Fortunella_japonica_3** **tcattcgtgggtgcggctaactccccatgaggtaactctatgattttccaaggtaaaaga**

**Fortunella_japonica_4** **tcattcgtgggtgcggctaactccccatgaggtaactctatgattttccaaggtaaaaga**

**Fortunella_japonica_5** **tcattcgtgggtgcggctaactccccatgaggtaactctatgattttccaaggtaaaaga**

**Fortunella_japonica_6** **tcattcgtgggtgcggctaactccccatgaggtaactctatgattttccaaggtaaaaga**

**Fortunella_japonica_7** **tcattcgtgggtgcggctaactccccatgaggtaactctatgattttccaaggtaaaaga**

**Fortunella_japonica_8** **tcattcgtgggtgcggctaactccccatgaggtaactctatgattttccaaggtaaaaga**

**Fortunella_japonica_9** **tcattcgtgggtgcggctaactccccatgaggtaactctatgattttccaaggtaaaaga**

**Fortunella_japonica_10** **tcattcgtgggtgcggctaactccccatgaggtaactctatgattttccaaggtaaaaga**

**Fortunella_japonica_11** **tcattcgtgggtgcggctaactccccatgaggtaactctatgattttccaaggtaaaaga**

**Fortunella_japonica_12** **tcattcgtgggtgcggctaactccccatgaggtaactctatgattttccaaggtaaaaga**

**Fortunella_margarita_1** **tcattcgtgggtgcggctaactccccatgaggtaactctatgattttccaaggtaaaaga**

**Fortunella_margarita_2** **tcattcgtgggtgcggctaactccccatgaggtaactctatgattttccaaggtaaaaga**

**Fortunella_venosa_1** **tcattcgtgggtgcggctaactccccatgaggtaactctatgattttccaaggtaaaaga**

**Fortunella_venosa_2** **tcattcgtgggtgcggctaactccccatgaggtaactctatgattttccaaggtaaaaga**

**Fortunella_venosa_3** **tcattcgtgggtgcggctaactccccatgaggtaactctatgattttccaaggtaaaaga**

17170	17180	17190	17200	17210	17220

....|....|....|....|....|....|....|....|....|....|....|....|

**Atalantia_kwangtungens** **gcacctgaccaattagaaacaaaaataaaaagaaacagagttccaataaagggaacccat**

**Citrus_reticulata_1** **gcacctgaccaattagaaacaaaaataaaaagaaacagagttccaataaagggaacccat**

**Citrus_reticulata_2** **gcacctgaccaattagaaacaaaaataaaaagaaacagagttccaataaagggaacccat**

**Citrus_trifoliata_3** **gcacctgaccaattagaaacaaaaataaaaagaaacagagttccaataaagggaacccat**

**Citrus_aurantiifolia** **gcacctgaccaattagaaacaaaaataaaaagaaacagagttccaataaagggaacccat**

**Citrus_aurantium** **gcacctgaccaattagaaacaaaaataaaaagaaacagagttccaataaagggaacccat**

**Citrus_cavaleriei** **gcacctgaccaattagaaacaaaaataaaaagaaacagagttccaataaagggaacccat**

**Citrus_hongheensis** **gcacctgaccaattagaaacaaaaataaaaagaaacagagttccaataaagggaacccat**

**Citrus_limon** **gcacctgaccaattagaaacaaaaataaaaagaaacagagttccaataaagggaacccat**

**Citrus_maxima** **gcacctgaccaattagaaacaaaaataaaaagaaacagagttccaataaagggaacccat**

**Citrus_medica** **gcacctgaccaattagaaacaaaaataaaaagaaacagagttccaataaagggaacccat**

**Citrus_polytrifolia** **gcacctgaccaattagaaacaaaaataaaaagaaacagagttccaataaagggaacccat**

**Citrus_reticulata** **gcacctgaccaattagaaacaaaaataaaaagaaacagagttccaataaagggaacccat**

**Citrus_sinensis** **gcacctgaccaattagaaacaaaaataaaaagaaacagagttccaataaagggaacccat**

**Fortunella_bawangica_1** **gcacctgaccaattagaaacaaaaataaaaagaaacagagttccaataaagggaacccat**

**Fortunella_bawangica_2** **gcacctgaccaattagaaacaaaaataaaaagaaacagagttccaataaagggaacccat**

**Fortunella_hindsii_1** **gcacctgaccaattagaaacaaaaataaaaagaaacagagttccaataaagggaacccat**

**Fortunella_hindsii_2** **gcacctgaccaattagaaacaaaaataaaaagaaacatagttccaataaagggaacccat**

**Fortunella_hindsii_3** **gcacctgaccaattagaaacaaaaataaaaagaaacagagttccaataaagggaacccat**

**Fortunella_japonica_1** **gcacctgaccaattagaaacaaaaataaaaagaaacagagttccaataaagggaacccat**

**Fortunella_japonica_2** **gcacctgaccaattagaaacaaaaataaaaagaaacagagttccaataaagggaacccat**

**Fortunella_japonica_3** **gcacctgaccaattagaaacaaaaataaaaagaaacagagttccaataaagggaacccat**

**Fortunella_japonica_4** **gcacctgaccaattagaaacaaaaataaaaagaaacagagttccaataaagggaacccat**

**Fortunella_japonica_5** **gcacctgaccaattagaaacaaaaataaaaagaaacagagttccaataaagggaacccat**

**Fortunella_japonica_6** **gcacctgaccaattagaaacaaaaataaaaagaaacagagttccaataaagggaacccat**

**Fortunella_japonica_7** **gcacctgaccaattagaaacaaaaataaaaagaaacagagttccaataaagggaacccat**

**Fortunella_japonica_8** **gcacctgaccaattagaaacaaaaataaaaagaaacagagttccaataaagggaacccat**

**Fortunella_japonica_9** **gcacctgaccaattagaaacaaaaataaaaagaaacagagttccaataaagggaacccat**

**Fortunella_japonica_10** **gcacctgaccaattagaaacaaaaataaaaagaaacagagttccaataaagggaacccat**

**Fortunella_japonica_11** **gcacctgaccaattagaaacaaaaataaaaagaaacagagttccaataaagggaacccat**

**Fortunella_japonica_12** **gcacctgaccaattagaaacaaaaataaaaagaaacagagttccaataaagggaacccat**

**Fortunella_margarita_1** **gcacctgaccaattagaaacaaaaataaaaagaaacagagttccaataaagggaacccat**

**Fortunella_margarita_2** **gcacctgaccaattagaaacaaaaataaaaagaaacagagttccaataaagggaacccat**

**Fortunella_venosa_1** **gcacctgaccaattagaaacaaaaataaaaagaaacagagttccaataaagggaacccat**

**Fortunella_venosa_2** **gcacctgaccaattagaaacaaaaataaaaagaaacagagttccaataaagggaacccat**

**Fortunella_venosa_3** **gcacctgaccaattagaaacaaaaataaaaagaaacagagttccaataaagggaacccat**

17230	17240	17250	17260	17270	17280

....|....|....|....|....|....|....|....|....|....|....|....|

**Atalantia_kwangtungens** **gggccatattcttctccaatctgagttttgctcacgtctcgaatgaattcaaggacatat**

**Citrus_reticulata_1** **gggccatattcttctccaatctgagttttgctcacgtctcgaatgaattcaaggacatat**

**Citrus_reticulata_2** **gggccatattcttctccaatctgagttttgctcacgtctcgaatgaattcaaggacatat**

**Citrus_trifoliata_3** **gggccatattcttctccaatctgagttttgctcacgtctcgaatgaattcaaggacatat**

**Citrus_aurantiifolia** **gggccatattcttctccaatctgagttttgctcacgtctcgaatgaattcaaggacatat**

**Citrus_aurantium** **gggccatattcttctccaatctgagttttgctcacgtctcgaatgaattcaaggacatat**

**Citrus_cavaleriei** **gggccatattcttctccaatctgagttttgctcacgtctcgaatgaattcaaggacatat**

**Citrus_hongheensis** **gggccatattcttctccaatctgagttttgctcacgtctcgaatgaattcaaggacatat**

**Citrus_limon** **gggccatattcttctccaatctgagttttgctcacgtctcgaatgaattcaaggacatat**

**Citrus_maxima** **gggccatattcttctccaatctgagttttgctcacgtctcgaatgaattcaaggacatat**

**Citrus_medica** **gggccatattcttctccaatctgagttttgctcacgtctcgaatgaattcaaggacatat**

**Citrus_polytrifolia** **gggccatattcttctccaatctgagttttgctcacgtctcgaatgaattcaaggacatat**

**Citrus_reticulata** **gggccatattcttctccaatctgagttttgctcacgtctcgaatgaattcaaggacatat**

**Citrus_sinensis** **gggccatattcttctccaatctgagttttgctcacgtctcgaatgaattcaaggacatat**

**Fortunella_bawangica_1** **gggccatattcttctccaatctgagttttgctcacgtctcgaatgaattcaaggacatat**

**Fortunella_bawangica_2** **gggccatattcttctccaatctgagttttgctcacgtctcgaatgaattcaaggacatat**

**Fortunella_hindsii_1** **gggccatattcttctccaatctgagttttgctcacgtctcgaatgaattcaaggacatat**

**Fortunella_hindsii_2** **gggccatattcttctccaatctgagttttgctcacgtctcgaatgaattcaaggacatat**

**Fortunella_hindsii_3** **gggccatattcttctccaatctgagttttgctcacgtctcgaatgaattcaaggacatat**

**Fortunella_japonica_1** **gggccatattcttctccaatctgagttttgctcacgtctcgaatgaattcaaggacatat**

**Fortunella_japonica_2** **gggccatattcttctccaatctgagttttgctcacgtctcgaatgaattcaaggacatat**

**Fortunella_japonica_3** **gggccatattcttctccaatctgagttttgctcacgtctcgaatgaattcaaggacatat**

**Fortunella_japonica_4** **gggccatattcttctccaatctgagttttgctcacgtctcgaatgaattcaaggacatat**

**Fortunella_japonica_5** **gggccatattcttctccaatctgagttttgctcacgtctcgaatgaattcaaggacatat**

**Fortunella_japonica_6** **gggccatattcttctccaatctgagttttgctcacgtctcgaatgaattcaaggacatat**

**Fortunella_japonica_7** **gggccatattcttctccaatctgagttttgctcacgtctcgaatgaattcaaggacatat**

**Fortunella_japonica_8** **gggccatattcttctccaatctgagttttgctcacgtctcgaatgaattcaaggacatat**

**Fortunella_japonica_9** **gggccatattcttctccaatctgagttttgctcacgtctcgaatgaattcaaggacatat**

**Fortunella_japonica_10** **gggccatattcttctccaatctgagttttgctcacgtctcgaatgaattcaaggacatat**

**Fortunella_japonica_11** **gggccatattcttctccaatctgagttttgctcacgtctcgaatgaattcaaggacatat**

**Fortunella_japonica_12** **gggccatattcttctccaatctgagttttgctcacgtctcgaatgaattcaaggacatat**

**Fortunella_margarita_1** **gggccatattcttctccaatctgagttttgctcacgtctcgaatgaattcaaggacatat**

**Fortunella_margarita_2** **gggccatattcttctccaatctgagttttgctcacgtctcgaatgaattcaaggacatat**

**Fortunella_venosa_1** **gggccatattcttctccaatctgagttttgctcacgtctcgaatgaattcaaggacatat**

**Fortunella_venosa_2** **gggccatattcttctccaatctgagttttgctcacgtctcgaatgaattcaaggacatat**

**Fortunella_venosa_3** **gggccatattcttctccaatctgagttttgctcacgtctcgaatgaattcaaggacatat**

17290	17300	17310	17320	17330	17340

....|....|....|....|....|....|....|....|....|....|....|....|

**Atalantia_kwangtungens** **tcgaagaaattttgagtggcagtcggaacggtttgtggattccgaacggctataagggct**

**Citrus_reticulata_1** **tcgaagaaattttgagtggcagtcggaacggtttgtggattccgaacggctataaaggct**

**Citrus_reticulata_2** **tcgaagaaattttgagtggcagtcggaacggtttgtggattccgaacggctataaaggct**

**Citrus_trifoliata_3** **tcgaagaaattttgagtggcagtcggaacggtttgtggattccgaacggctataaaggct**

**Citrus_aurantiifolia** **tcgaagaaattttgagtggcagtcggaacggtttgtggattccgaacggctataaaggct**

**Citrus_aurantium** **tcgaagaaattttgagtggcagtcggaacggtttgtggattccgaacggctataaaggct**

**Citrus_cavaleriei** **tcgaagaaattttgagtggcagtcggaacggtttgtggattccgaacggctataaaggct**

**Citrus_hongheensis** **tcgaagaaattttgagtggcagtcggaacggtttgtggattccgaacggctataaaggct**

**Citrus_limon** **tcgaagaaattttgagtggcagtcggaacggtttgtggattccgaacggctataaaggct**

**Citrus_maxima** **tcgaagaaattttgagtggcagtcggaacggtttgtggattccgaacggctataaaggct**

**Citrus_medica** **tcgaagaaattttgagtggcagtcggaacggtttgtggattccgaacggctataaaggct**

**Citrus_polytrifolia** **tcgaagaaattttgagtggcagtcggaacggtttgtggattccgaacggctataaaggct**

**Citrus_reticulata** **tcgaagaaattttgagtggcagtcggaacggtttgtggattccgaacggctataaaggct**

**Citrus_sinensis** **tcgaagaaattttgagtggcagtcggaacggtttgtggattccgaacggctataaaggct**

**Fortunella_bawangica_1** **tcgaagaaattttgagtggcagtcggaacggtttgtggattccgaacggctataaaggct**

**Fortunella_bawangica_2** **tcgaagaaattttgagtggcagtcggaacggtttgtggattccgaacggctataaaggct**

**Fortunella_hindsii_1** **tcgaagaaattttgagtggcagtcggaacggtttgtggattccgaacggctataaaggct**

**Fortunella_hindsii_2** **tcgaagaaattttgagtggcagtcggaacggtttgtggattccgaacggctataaaggct**

**Fortunella_hindsii_3** **tcgaagaaattttgagtggcagtcggaacggtttgtggattccgaacggctataaaggct**

**Fortunella_japonica_1** **tcgaagaaattttgagtggctgtcggaacggtttgtggattccgaacggctataaaggct**

**Fortunella_japonica_2** **tcgaagaaattttgagtggctgtcggaacggtttgtggattccgaacggctataaaggct**

**Fortunella_japonica_3** **tcgaagaaattttgagtggctgtcggaacggtttgtggattccgaacggctataaaggct**

**Fortunella_japonica_4** **tcgaagaaattttgagtggctgtcggaacggtttgtggattccgaacggctataaaggct**

**Fortunella_japonica_5** **tcgaagaaattttgagtggctgtcggaacggtttgtggattccgaacggctataaaggct**

**Fortunella_japonica_6** **tcgaagaaattttgagtggctgtcggaacggtttgtggattccgaacggctataaaggct**

**Fortunella_japonica_7** **tcgaagaaattttgagtggctgtcggaacggtttgtggattccgaacggctataaaggct**

**Fortunella_japonica_8** **tcgaagaaattttgagtggctgtcggaacggtttgtggattccgaacggctataaaggct**

**Fortunella_japonica_9** **tcgaagaaattttgagtggctgtcggaacggtttgtggattccgaacggctataaaggct**

**Fortunella_japonica_10** **tcgaagaaattttgagtggcagtcggaacggtttgtggattccgaacggctataaaggct**

**Fortunella_japonica_11** **tcgaagaaattttgagtggctgtcggaacggtttgtggattccgaacggctataaaggct**

**Fortunella_japonica_12** **tcgaagaaattttgagtggctgtcggaacggtttgtggattccgaacggctataaaggct**

**Fortunella_margarita_1** **tcgaagaaattttgagtggcagtcggaacggtttgtggattccgaacggctataaaggct**

**Fortunella_margarita_2** **tcgaagaaattttgagtggcagtcggaacggtttgtggattccgaacggctataaaggct**

**Fortunella_venosa_1** **tcgaagaaattttgagtggcagtcggaacggtttgtggattccgaacggctataaaggct**

**Fortunella_venosa_2** **tcgaagaaattttgagtggcagtcggaacggtttgtggattccgaacggctataaaggct**

**Fortunella_venosa_3** **tcgaagaaattttgagtggcagtcggaacggtttgtggattccgaacggctataaaggct**

17350	17360	17370	17380	17390	17400

....|....|....|....|....|....|....|....|....|....|....|....|

**Atalantia_kwangtungens** **gaacctaataagatagcaattacaacccaagaagtaataagtacttgggcatggacctgg**

**Citrus_reticulata_1** **gaacctaataagatagcaattacaacccaagaagtaataagtacttgggcatggacctgg**

**Citrus_reticulata_2** **gaacctaataagatagcaattacaacccaagaagtaataagtacttgggcatggacctgg**

**Citrus_trifoliata_3** **gaacctaataagatagcaattacaacccaagaagtaataagtacttgggcatggacctgg**

**Citrus_aurantiifolia** **gaacctaataagatagcaattacaacccaagaagtaataagtacttgggcatggacctgg**

**Citrus_aurantium** **gaacctaataagatagcaattacaacccaagaagtaataagtacttgggcatggacctgg**

**Citrus_cavaleriei** **gaacctaataagatagcaattacaacccaagaagtaataagtacttgggcatggacctgg**

**Citrus_hongheensis** **gaacctaataagatagcaattacaacccaagaagtaataagtacttgggcatggacctgg**

**Citrus_limon** **gaacctaataagatagcaattacaacccaagaagtaataagtacttgggcatggacctgg**

**Citrus_maxima** **gaacctaataagatagcaattacaacccaagaagtaataagtacttgggcatggacctgg**

**Citrus_medica** **gaacctaataagatagcaattacaacccaagaagtaataagtacttgggcatggacctgg**

**Citrus_polytrifolia** **gaacctaataagatagcaattacaacccaagaagtaataagtacttgggcatggacctgg**

**Citrus_reticulata** **gaacctaataagatagcaattacaacccaagaagtaataagtacttgggcatggacctgg**

**Citrus_sinensis** **gaacctaataagatagcaattacaacccaagaagtaataagtacttgggcatggacctgg**

**Fortunella_bawangica_1** **gaacctaataagatagcaattacaacccaagaagtaataagtacttgggcatggacctgg**

**Fortunella_bawangica_2** **gaacctaataagatagcaattacaacccaagaagtaataagtacttgggcatggacctgg**

**Fortunella_hindsii_1** **gaacctaataagatagcaattacaacccaagaagtaataagtacttgggcatggacctgg**

**Fortunella_hindsii_2** **gaacctaataagatagcaattacaacccaagaagtaataagtacttgggcatggacctgg**

**Fortunella_hindsii_3** **gaacctaataagatagcaattacaacccaagaagtaataagtacttgggcatggacctgg**

**Fortunella_japonica_1** **gaacctaataagatagcaattacaacccaagaagtaataagtacttgggcatggacctgg**

**Fortunella_japonica_2** **gaacctaataagatagcaattacaacccaagaagtaataagtacttgggcatggacctgg**

**Fortunella_japonica_3** **gaacctaataagatagcaattacaacccaagaagtaataagtacttgggcatggacctgg**

**Fortunella_japonica_4** **gaacctaataagatagcaattacaacccaagaagtaataagtacttgggcatggacctgg**

**Fortunella_japonica_5** **gaacctaataagatagcaattacaacccaagaagtaataagtacttgggcatggacctgg**

**Fortunella_japonica_6** **gaacctaataagatagcaattacaacccaagaagtaataagtacttgggcatggacctgg**

**Fortunella_japonica_7** **gaacctaataagatagcaattacaacccaagaagtaataagtacttgggcatggacctgg**

**Fortunella_japonica_8** **gaacctaataagatagcaattacaacccaagaagtaataagtacttgggcatggacctgg**

**Fortunella_japonica_9** **gaacctaataagatagcaattacaacccaagaagtaataagtacttgggcatggacctgg**

**Fortunella_japonica_10** **gaacctaataagatagcaattacaacccaagaagtaataagtacttgggcatggacctgg**

**Fortunella_japonica_11** **gaacctaataagatagcaattacaacccaagaagtaataagtacttgggcatggacctgg**

**Fortunella_japonica_12** **gaacctaataagatagcaattacaacccaagaagtaataagtacttgggcatggacctgg**

**Fortunella_margarita_1** **gaacctaataagatagcaattacaacccaagaagtaataagtacttgggcatggacctgg**

**Fortunella_margarita_2** **gaacctaataagatagcaattacaacccaagaagtaataagtacttgggcatggacctgg**

**Fortunella_venosa_1** **gaacctaataagatagcaattacaacccaagaagtaataagtacttgggcatggacctgg**

**Fortunella_venosa_2** **gaacctaataagatagcaattacaacccaagaagtaataagtacttgggcatggacctgg**

**Fortunella_venosa_3** **gaacctaataagatagcaattacaacccaagaagtaataagtacttgggcatggacctgg**

17410	17420	17430	17440	17450	17460

....|....|....|....|....|....|....|....|....|....|....|....|

**Atalantia_kwangtungens** **aacccacctatttgccaatagaaatgttggcctacttccacaccggatatatcgtataac**

**Citrus_reticulata_1** **aacccacctatttgccaatagaaatgttggcctacttccacaccggatatatcgtataac**

**Citrus_reticulata_2** **aacccacctatttgccaatagaaatgttggcctacttccacaccggatatatcgtataac**

**Citrus_trifoliata_3** **aacccccctatttgccaatagaaatgttggcctacttccacaccggatatatcgtataac**

**Citrus_aurantiifolia** **aacccacctatttgccaatagaaatgttggcctacttccacaccggatatatcgtataac**

**Citrus_aurantium** **aacccacctatttgccaatagaaatgttggcctacttccacaccggatatatcgtataac**

**Citrus_cavaleriei** **aacccacctatttgccaatagaaatgttggcctacttccacaccggatatctcgtataac**

**Citrus_hongheensis** **aacccacctatttgccaatagaaatgttggcctacttccacaccggatatatcgtataac**

**Citrus_limon** **aacccacctatttgccaatagaaatgttggcctacttccacaccggatatatcgtataac**

**Citrus_maxima** **aacccacctatttgccaatagaaatgttggcctacttccacaccggatatatcgtataac**

**Citrus_medica** **aacccacctatttgccaatagaaatgttgtcctacttccacaccggatatatcgtataac**

**Citrus_polytrifolia** **aacccccctatttgccaatagaaatgttggcctacttccacaccggatatatcgtataac**

**Citrus_reticulata** **aacccacctatttgccaatagaaatgttggcctacttccacaccggatatatcgtataac**

**Citrus_sinensis** **aacccacctatttgccaatagaaatgttggcctacttccacaccggatatatcgtataac**

**Fortunella_bawangica_1** **aacccacctatttgccaatagaaatgttggcctacttccacaccggatatatcgtataac**

**Fortunella_bawangica_2** **aacccacctatttgccaatagaaatgttggcctacttccacaccggatatatcgtataac**

**Fortunella_hindsii_1** **aacccacctatttgccaatagaaatgttggcctacttccacaccggatatatcgtataac**

**Fortunella_hindsii_2** **aacccacctatttgccaatagaaatgttggcctacttccacaccggatatatcgtataac**

**Fortunella_hindsii_3** **aacccacctatttgccaatagaaatgttggcctacttccacaccggatatatcgtataac**

**Fortunella_japonica_1** **aacccacctatttgccaatagaaatgttggcctacttccacaccggatatatcgtataac**

**Fortunella_japonica_2** **aacccacctatttgccaatagaaatgttggcctacttccacaccggatatatcgtataac**

**Fortunella_japonica_3** **aacccacctatttgccaatagaaatgttggcctacttccacaccggatatatcgtataac**

**Fortunella_japonica_4** **aacccacctatttgccaatagaaatgttggcctacttccacaccggatatatcgtataac**

**Fortunella_japonica_5** **aacccacctatttgccaatagaaatgttggcctacttccacaccggatatatcgtataac**

**Fortunella_japonica_6** **aacccacctatttgccaatagaaatgttggcctacttccacaccggatatatcgtataac**

**Fortunella_japonica_7** **aacccacctatttgccaatagaaatgttggcctacttccacaccggatatatcgtataac**

**Fortunella_japonica_8** **aacccacctatttgccaatagaaatgttggcctacttccacaccggatatatcgtataac**

**Fortunella_japonica_9** **aacccacctatttgccaatagaaatgttggcctacttccacaccggatatatcgtataac**

**Fortunella_japonica_10** **aacccacctatttgccaatagaaatgttggcctacttccacaccggatatatcgtataac**

**Fortunella_japonica_11** **aacccacctatttgccaatagaaatgttggcctacttccacaccggatatatcgtataac**

**Fortunella_japonica_12** **aacccacctatttgccaatagaaatgttggcctacttccacaccggatatatcgtataac**

**Fortunella_margarita_1** **aacccacctatttgccaatagaaatgttggcctacttccacaccggatatatcgtataac**

**Fortunella_margarita_2** **aacccacctatttgccaatagaaatgttggcctacttccacaccggatatatcgtataac**

**Fortunella_venosa_1** **aacccacctatttgccaatagaaatgttggcctacttccacaccggatatatcgtataac**

**Fortunella_venosa_2** **aacccacctatttgccaatagaaatgttggcctacttccacaccggatatatcgtataac**

**Fortunella_venosa_3** **aacccacctatttgccaatagaaatgttggcctacttccacaccggatatatcgtataac**

17470	17480	17490	17500	17510	17520

....|....|....|....|....|....|....|....|....|....|....|....|

**Atalantia_kwangtungens** **ccccttagtgtattcatggaacatgatagaacattcatattgccctctgaccgaaatata**

**Citrus_reticulata_1** **ccccttagtgtattcatggaacatgatagaacattcatatcgccctctgaccgaaataga**

**Citrus_reticulata_2** **ccccttagtgtattcatggaacatgatagaacattcatatcgccctctgaccgaaataga**

**Citrus_trifoliata_3** **ccccttagtgtattcatggaacatgatagaacattcatattgccctctgaccgaaataga**

**Citrus_aurantiifolia** **ccccttagtgtattcatggaacatgatagaacattcatattgccctctgaccgaaataga**

**Citrus_aurantium** **ccccttagtgtattcatggaacatgatagaacattcatattgccctctgaccgaaataga**

**Citrus_cavaleriei** **ccccttagtgtattcatggaacatgatagaacattcatatcgccctctgaccgaaataga**

**Citrus_hongheensis** **ccccttagtgtattcatggaacatgatagaacattcatattgccctctgaccgaaataga**

**Citrus_limon** **ccccttagtgtattcatggaacatgatagaacattcatattgccctctgaccgaaataga**

**Citrus_maxima** **ccccttagtgtattcatggaacatgatagaacattcatattgccctctgaccgaaataga**

**Citrus_medica** **ccccttagtgtattcatggaacatgatagaacattcatattgccctctgaccgaaataga**

**Citrus_polytrifolia** **ccccttagtgtattcatggaacatgatagaacattcatattgccctctgaccgaaataga**

**Citrus_reticulata** **ccccttagtgtattcatggaacatgatagaacattcatatcgccctctgaccgaaataga**

**Citrus_sinensis** **ccccttagtgtattcatggaacatgatagaacattcatattgccctctgaccgaaataga**

**Fortunella_bawangica_1** **ccccttagtgtattcatggaacatgatagaacattcatattgccctctgaccgaaataga**

**Fortunella_bawangica_2** **ccccttagtgtattcatggaacatgatagaacattcatattgccctctgaccgaaataga**

**Fortunella_hindsii_1** **ccccttagtgtattcatggaacatgatagaacattcatattgccctctgaccgaaataga**

**Fortunella_hindsii_2** **ccccttagtgtattcatggaacatgatagaacattcatattgccctctgaccgaaataga**

**Fortunella_hindsii_3** **ccccttagtgtattcatggaacatgatagaacattcatattgccctctgaccgaaataga**

**Fortunella_japonica_1** **ccccttagtgtattcatggaacatgatagaacattcatattgccctctgaccgaaataga**

**Fortunella_japonica_2** **ccccttagtgtattcatggaacatgatagaacattcatattgccctctgaccgaaatcga**

**Fortunella_japonica_3** **ccccttagtgtattcatggaacatgatagaacattcatattgccctctgaccgaaataga**

**Fortunella_japonica_4** **ccccttagtgtattcatggaacatgatagaacattcatattgccctctgaccgaaataga**

**Fortunella_japonica_5** **ccccttagtgtattcatggaacatgatagaacattcatattgccctctgaccgaaataga**

**Fortunella_japonica_6** **ccccttagtgtattcatggaacatgatagaacattcatattgccctctgaccgaaataga**

**Fortunella_japonica_7** **ccccttagtgtattcatggaacatgatagaacattcatattgccctctgaccgaaataga**

**Fortunella_japonica_8** **ccccttagtgtattcatggaacatgatagaacattcatattgccctctgaccgaaataga**

**Fortunella_japonica_9** **ccccttagtgtattcatggaacatgatagaacattcatattgccctctgaccgaaataga**

**Fortunella_japonica_10** **ccccttagtgtattcatggaacatgatagaacattcatattgccctctgaccgaaataga**

**Fortunella_japonica_11** **ccccttagtgtattcatggaacatgatagaacattcatattgccctctgaccgaaataga**

**Fortunella_japonica_12** **ccccttagtgtattcatggaacatgatagaacattcatattgccctctgaccgaaataga**

**Fortunella_margarita_1** **ccccttagtgtattcatggaacatgatagaacattcatattgccctctgaccgaaataga**

**Fortunella_margarita_2** **ccccttagtgtattcatggaacatgatagaacattcatattgccctctgaccgaaataga**

**Fortunella_venosa_1** **ccccttagtgtattcatggaacatgatagaacattcatattgccctctgaccgaaataga**

**Fortunella_venosa_2** **ccccttagtgtattcatggaacatgatagaacattcatattgccctctgaccgaaataga**

**Fortunella_venosa_3** **ccccttagtgtattcatggaacatgatagaacattcatattgccctctgaccgaaataga**

17530	17540	17550	17560	17570	17580

....|....|....|....|....|....|....|....|....|....|....|....|

**Atalantia_kwangtungens** **aatttaaaaagaattattttgattcaaccatcgtcttttcgacttgtctacttgaattgt**

**Citrus_reticulata_1** **aatttaaaaagaattattttgattcaaccatcttcttttcgacttgtctacttgaattgt**

**Citrus_reticulata_2** **aatttaaaaagaattattttgattcaaccatcttcttttcgacttgtctacttgaattgt**

**Citrus_trifoliata_3** **aatttaaaaagaattattttgattcaaccatcttcttttcgacttgtctacttgaattgt**

**Citrus_aurantiifolia** **aatttaaaaagaattattttgattcaaccatcttcttttcgacttgtctacttgaattgt**

**Citrus_aurantium** **aatttaaaaagaattattttgattcaaccatcttcttttcgacttgtctacttgaattgt**

**Citrus_cavaleriei** **aatttaaaaagaattattttgattcaaccatcttcttttcgacttgtctacttgaattgt**

**Citrus_hongheensis** **aatttaaaaagaattattttgattcaaccatcttcttttcgacttgtctacttgaattgt**

**Citrus_limon** **aatttaaaaagaattattttgattcaaccatcttcttttcgacttgtctacttgaattgt**

**Citrus_maxima** **aatttaaaaagaattattttgattcaaccatcttcttttcgacttgtctacttgaattgt**

**Citrus_medica** **aatttaaaaagaattattttgattcaaccatcttcttttcgacttgtctacttgaattgt**

**Citrus_polytrifolia** **aatttaaaaagaattattttgattcaaccatcttcttttcgacttgtctacttgaattgt**

**Citrus_reticulata** **aatttaaaaagaattattttgattcaaccatcttcttttcgacttgtctacttgaattgt**

**Citrus_sinensis** **aatttaaaaagaattattttgattcaaccatcttcttttcgacttgtctacttgaattgt**

**Fortunella_bawangica_1** **aatttaaaaagaattattttgattcaaccatcttcttttcgacttgtctacttgaattgt**

**Fortunella_bawangica_2** **aatttaaaaagaattattttgattcaaccatcttcttttcgacttgtctacttgaattgt**

**Fortunella_hindsii_1** **aatttaaaaagaattattttgattcaaccatcttcttttcgacttgtctacttgaattgt**

**Fortunella_hindsii_2** **aatttaaaaagaattattttgattcaaccatcttcttttcgacttgtctacttgaattgt**

**Fortunella_hindsii_3** **aatttaaaaagaattattttgattcaaccatcttcttttcgacttgtctacttgaattgt**

**Fortunella_japonica_1** **aatttaaaaagaattattttgattcaaccatcttcttttcgacttgtctacttgaattgt**

**Fortunella_japonica_2** **aatttaaaaagaattattttgattcaaccatcttcttttcgacttgtctacttgaattgt**

**Fortunella_japonica_3** **aatttaaaaagaattattttgattcaaccatcttcttttcgacttgtctacttgaattgt**

**Fortunella_japonica_4** **aatttaaaaagaattattttgattcaaccatcttcttttcgacttgtctacttgaattgt**

**Fortunella_japonica_5** **aatttaaaaagaattattttgattcaaccatcttcttttcgacttgtctacttgaattgt**

**Fortunella_japonica_6** **aatttaaaaagaattattttgattcaaccatcttcttttcgacttgtctacttgaattgt**

**Fortunella_japonica_7** **aatttaaaaagaattattttgattcaaccatcttcttttcgacttgtctacttgaattgt**

**Fortunella_japonica_8** **aatttaaaaagaattattttgattcaaccatcttcttttcgacttgtctacttgaattgt**

**Fortunella_japonica_9** **aatttaaaaagaattattttgattcaaccatcttcttttcgacttgtctacttgaattgt**

**Fortunella_japonica_10** **aatttaaaaagaattattttgattcaaccatcttcttttcgacttgtctacttgaattgt**

**Fortunella_japonica_11** **aatttaaaaagaattattttgattcaaccatcttcttttcgacttgtctacttgaattgt**

**Fortunella_japonica_12** **aatttaaaaagaattattttgattcaaccatcttcttttcgacttgtctacttgaattgt**

**Fortunella_margarita_1** **aatttaaaaagaattattttgattcaaccatcttcttttcgacttgtctacttgaattgt**

**Fortunella_margarita_2** **aatttaaaaagaattattttgattcaaccatcttcttttcgacttgtctacttgaattgt**

**Fortunella_venosa_1** **aatttaaaaagaattattttgattcaaccatcttcttttcgacttgtctacttgaattgt**

**Fortunella_venosa_2** **aatttaaaaagaattattttgattcaaccatcttcttttcgacttgtctacttgaattgt**

**Fortunella_venosa_3** **aatttaaaaagaattattttgattcaaccatcttcttttcgacttgtctacttgaattgt**

17590	17600	17610	17620	17630	17640

....|....|....|....|....|....|....|....|....|....|....|....|

**Atalantia_kwangtungens** **atattttgaatatctactaattgcataatatccaccaggtttggctcttttcttttgtgc**

**Citrus_reticulata_1** **atattttgaatatctgctaattgcataatatccaccaggtttgtctcttttcttttgtgc**

**Citrus_reticulata_2** **atattttgaatatctgctaattgcataatatccaccaggtttgtctcttttcttttgtgc**

**Citrus_trifoliata_3** **atattttgaatatctgctaattgcataatatccaccaggtttgtctcttttcttttgtgc**

**Citrus_aurantiifolia** **atattttgaatatctgctaattgcataatatccaccaggtttgtctcttttcttttgtgc**

**Citrus_aurantium** **atattttgaatatctgctaattgcataatatccaccaggtttgtctcttttcttttgtgc**

**Citrus_cavaleriei** **atattttgaatatctgctaattgcataatatccaccaggtttgtctcttttcttttgtgc**

**Citrus_hongheensis** **atattttgaatatctgctaattgcataatatccaccaggtttgtctcttttcttttgtgc**

**Citrus_limon** **atattttgaatatctgctaattgcataatatccaccaggtttgtctcttttcttttgtgc**

**Citrus_maxima** **atattttgaatatctgctaattgcataatatccaccaggtttgtctcttttcttttgtgc**

**Citrus_medica** **ctattttgaatatctgctaattgcataatatccaccaggtttgtctcttttcttttgtgc**

**Citrus_polytrifolia** **atattttgaatatctgctaattgcataatatccaccaggtttgtctcttttcttttgtgc**

**Citrus_reticulata** **atattttgaatatctgctaattgcataatatccaccaggtttgtctcttttcttttgtgc**

**Citrus_sinensis** **atattttgaatatctgctaattgcataatatccaccaggtttgtctcttttcttttgtgc**

**Fortunella_bawangica_1** **atattttgaatatctgctaattgcataatatccaccaggtttgtctcttttcttttgtgc**

**Fortunella_bawangica_2** **atattttgaatatctgctaattgcataatatccaccaggtttgtctcttttcttttgtgc**

**Fortunella_hindsii_1** **atattttgaatatctgctaattgcataatatccaccaggtttgtctcttttcttttgtgc**

**Fortunella_hindsii_2** **atattttgaatatctgctaattgcataatatccaccaggtttgtctcttttcttttgtgc**

**Fortunella_hindsii_3** **atattttgaatatctgctaattgcataatatccaccaggtttgtctcttttcttttgtgc**

**Fortunella_japonica_1** **atattttgaatatctgctaattgcataatatccaccaggtttgtctcttttcttttgtgc**

**Fortunella_japonica_2** **atattttgaatatctgctaattgcataatatccaccaggtttgtctcttttcttttgtgc**

**Fortunella_japonica_3** **atattttgaatatctgctaattgcataatatccaccaggtttgtctcttttcttttgtgc**

**Fortunella_japonica_4** **atattttgaatatctgctaattgcataatatccaccaggtttgtctcttttcttttgtgc**

**Fortunella_japonica_5** **atattttgaatatctgctaattgcataatatccaccaggtttgtctcttttcttttgtgc**

**Fortunella_japonica_6** **atattttgaatatctgctaattgcataatatccaccaggtttgtctcttttcttttgtgc**

**Fortunella_japonica_7** **atattttgaatatctgctaattgcataatatccaccaggtttgtctcttttcttttgtgc**

**Fortunella_japonica_8** **atattttgaatatctgctaattgcataatatccaccaggtttgtctcttttcttttgtgc**

**Fortunella_japonica_9** **atattttgaatatctgctaattgcataatatccaccaggtttgtctcttttcttttgtgc**

**Fortunella_japonica_10** **atattttgaatatctgctaattgcataatatccaccaggtttgtctcttttcttttgtgc**

**Fortunella_japonica_11** **atattttgaatatctgctaattgcataatatccaccaggtttgtctcttttcttttgtgc**

**Fortunella_japonica_12** **atattttgaatatctgctaattgcataatatccaccaggtttgtctcttttcttttgtgc**

**Fortunella_margarita_1** **atattttgaatatctgctaattgcataatatccaccaggtttgtctcttttcttttgtgc**

**Fortunella_margarita_2** **atattttgaatatctgctaattgcataatatccaccaggtttgtctcttttcttttgtgc**

**Fortunella_venosa_1** **atattttgaatatctgctaattgcataatatccaccaggtttgtctcttttcttttgtgc**

**Fortunella_venosa_2** **atattttgaatatctgctaattgcataatatccaccaggtttgtctcttttcttttgtgc**

**Fortunella_venosa_3** **atattttgaatatctgctaattgcataatatccaccaggtttgtctcttttcttttgtgc**

17650	17660	17670	17680	17690	17700

....|....|....|....|....|....|....|....|....|....|....|....|

**Atalantia_kwangtungens** **gattcgggaatagtacccaatccataaatctatggagttaccaaaataatttatttatct**

**Citrus_reticulata_1** **aattcaggaatagtacccaatccataaatctatggagttaccaaaataatttatttatct**

**Citrus_reticulata_2** **aattcaggaatagtacccaatccataaatctatggagttaccaaaataatttatttatct**

**Citrus_trifoliata_3** **aattcaggaatagtacccaatccataaatctatggagttaccaaaataatttatttatct**

**Citrus_aurantiifolia** **aattcaggaatagtacccaatccataaatctatggagttaccaaaataatttatttatct**

**Citrus_aurantium** **aattcaggaatagtacccaatccataaatctatggagttaccaaaataatttatttatct**

**Citrus_cavaleriei** **aattcaggaatagtacccaatccataaatctatggagttaccaaaataatttatttatct**

**Citrus_hongheensis** **aattcaggaatagtacccaatccataaatctatggagttaccaaaataatttatttatct**

**Citrus_limon** **aattcaggaatagtacccaatccataaatctatggagttaccaaaataatttatttatct**

**Citrus_maxima** **aattcaggaatagtacccaatccataaatctatggagttaccaaaataatttatttatct**

**Citrus_medica** **aattcaggaatagtacccaatccataaatctatggagttaccaaaataatttatttatct**

**Citrus_polytrifolia** **aattcaggaatagtacccaatccataaatctatggagttaccaaaataatttatttatct**

**Citrus_reticulata** **aattcaggaatagtacccaatccataaatctatggagttaccaaaataatttatttatct**

**Citrus_sinensis** **aattcaggaatagtacccaatccataaatctatggagttaccaaaataatttatttatct**

**Fortunella_bawangica_1** **aattcaggaatagtacccaatccataaatctatggagttaccaaaatactttatttatct**

**Fortunella_bawangica_2** **aattcaggaatagtacccaatccataaatctatggagttaccaaaatactttatttatct**

**Fortunella_hindsii_1** **aattcaggaatagtacccaatccataaatctatggagttaccaaaatactttatttatct**

**Fortunella_hindsii_2** **aattcaggaatagtacccaatccataaatctatggagttaccaaaatactttatttatct**

**Fortunella_hindsii_3** **aattcaggaatagtacccaatccataaatctatggagttaccaaaatactttatttatct**

**Fortunella_japonica_1** **aattcaggaatagtacccaatccataaatctatggagttaccaaaatacttgatttatct**

**Fortunella_japonica_2** **aattcaggaatagtacccaatccataaatctatggagttaccaaaatactttatttatct**

**Fortunella_japonica_3** **aattcaggaatagtacccaatccataaatctatggagttaccaaaatacttgatttatct**

**Fortunella_japonica_4** **aattcaggaatagtacccaatccataaatctatggagttaccaaaatacttgatttatct**

**Fortunella_japonica_5** **aattcaggaatagtacccaatccataaatctatggagttaccaaaatacttgatttatct**

**Fortunella_japonica_6** **aattcaggaatagtacccaatccataaatctatggagttaccaaaatacttgatttatct**

**Fortunella_japonica_7** **aattcaggaatagtacccaatccataaatctatggagttaccaaaatacttgatttatct**

**Fortunella_japonica_8** **aattcaggaatagtacccaatccataaatctatggagttaccaaaatacttgatttatct**

**Fortunella_japonica_9** **aattcaggaatagtacccaatccataaatctatggagttaccaaaatacttgatttatct**

**Fortunella_japonica_10** **aattcaggaatagtacccaatccataaatctatggagttaccaaaatactttatttatct**

**Fortunella_japonica_11** **aattcaggaatagtacccaatccataaatctatggagttaccaaaatactttatttatct**

**Fortunella_japonica_12** **aattcaggaatagtacccaatccataaatctatggagttaccaaaatacttgatttatct**

**Fortunella_margarita_1** **aattcaggaatagtacccaatccataaatctatggagttaccaaaatactttatttatct**

**Fortunella_margarita_2** **aattcaggaatagtacccaatccataaatctatggagttaccaaaatactttatttatct**

**Fortunella_venosa_1** **aattcaggaatagtacccaatccataaatctatggagttaccaaaatactttatttatct**

**Fortunella_venosa_2** **aattcaggaatagtacccaatccataaatctatggagttaccaaaatactttatttatct**

**Fortunella_venosa_3** **aattcaggaatagtacccaatccataaatctatggagttaccaaaatactttatttatct**

17710	17720	17730	17740	17750	17760

....|....|....|....|....|....|....|....|....|....|....|....|

**Atalantia_kwangtungens** **tattattaatcaacgatttcgtatatagctagagcgaccctcacaaattgcgaatactaa**

**Citrus_reticulata_1** **tattattaatcaaggatttcgtatatagctagagcgaccctcacaaattgcgaatactaa**

**Citrus_reticulata_2** **tattattaatcaaggatttcgtatatagctagagcgaccctcacaaattgcgaatactaa**

**Citrus_trifoliata_3** **tattattaatcaaggatttcgtatatagctagagcgaccctcacaaattgcgaatactaa**

**Citrus_aurantiifolia** **tattattaatcaaggatttcgtatatagctagagcgaccctcacaaattgcgaatactaa**

**Citrus_aurantium** **tattattaatcaaggatttcgtatatagctagagcgaccctcacaaattgcgaatactaa**

**Citrus_cavaleriei** **tattattaatcaaggatttcgtatatagctagagcgaccctcacaaattgcgcatactaa**

**Citrus_hongheensis** **tattattaatcaaggatttcgtatatagctagagcgaccctcacaaattgcgaatactaa**

**Citrus_limon** **tattattaatcaaggatttcgtatatagctagagcgaccctcacaaattgcgaatactaa**

**Citrus_maxima** **tattattaatcaaggatttcgtatatagctagagcgaccctcacaaattgcgaatactaa**

**Citrus_medica** **tattattaatcaaggatttcgtatatagctagagcgaccctcacaaattgcgaatactaa**

**Citrus_polytrifolia** **tattattaatcaaggatttcgtatatagctagagcgaccctcacaaattgcgaatactaa**

**Citrus_reticulata** **tattattaatcaaggatttcgtatatagctagagcgaccctcacaaattgcgaatactaa**

**Citrus_sinensis** **tattattaatcaaggatttcgtatatagctagagcgaccctcacaaattgcgaatactaa**

**Fortunella_bawangica_1** **tattattaatcaaggatttcgtatatagctagagcgaccctcacaaattgcgaatactaa**

**Fortunella_bawangica_2** **tattattaatcaaggatttcgtatatagctagagcgaccctcacaaattgcgaatactaa**

**Fortunella_hindsii_1** **tattattaatcaaggatttcgtatatagctagagcgaccctcacaaattgcgaatactaa**

**Fortunella_hindsii_2** **tattattaatcaaggatttcgtatatagctagagcgaccctcacaaattgcgaatactaa**

**Fortunella_hindsii_3** **tattattaatcaaggatttcgtatatagctagagcgaccctcacaaattgcgaatactaa**

**Fortunella_japonica_1** **tattattaatcaaggatttcgtatatagctagagcgaccctcacaaattgcgaatactaa**

**Fortunella_japonica_2** **tattattaatcaaggatttcgtatatagctagagcgaccctcacaaattgcgaatactaa**

**Fortunella_japonica_3** **tattattaatcaaggatttcgtatatagctagagcgaccctcacaaattgcgaatactaa**

**Fortunella_japonica_4** **tattattaatcaaggatttcgtatatagctagagcgaccctcacaaattgcgaatactaa**

**Fortunella_japonica_5** **tattattaatcaaggatttcgtatatagctagagcgaccctcacaaattgcgaatactaa**

**Fortunella_japonica_6** **tattattaatcaaggatttcgtatatagctagagcgaccctcacaaattgcgaatactaa**

**Fortunella_japonica_7** **tattattaatcaaggatttcgtatatagctagagcgaccctcacaaattgcgaatactaa**

**Fortunella_japonica_8** **tattattaatcaaggatttcgtatatagctagagcgaccctcacaaattgcgaatactaa**

**Fortunella_japonica_9** **tattattaatcaaggatttcgtatatagctagagcgaccctcacaaattgcgaatactaa**

**Fortunella_japonica_10** **tattattaatcaaggatttcgtatatagctagagcgaccctcacaaattgcgaatactaa**

**Fortunella_japonica_11** **tattattaatcaaggatttcgtatatagctagagcgaccctcacaaattgcgaatactaa**

**Fortunella_japonica_12** **tattattaatcaaggatttcgtatatagctagagcgaccctcacaaattgcgaatactaa**

**Fortunella_margarita_1** **tattattaatcaaggatttcgtatatagctagagcgaccctcacaaattgcgaatactaa**

**Fortunella_margarita_2** **tattattaatcaaggatttcgtatatagctagagcgaccctcacaaattgcgaatactaa**

**Fortunella_venosa_1** **tattattaatcaaggatttcgtatatagctagagcgaccctcacaaattgcgaatactaa**

**Fortunella_venosa_2** **tattattaatcaaggatttcgtatatagctagagcgaccctcacaaattgcgaatactaa**

**Fortunella_venosa_3** **tattattaatcaaggatttcgtatatagctagagcgaccctcacaaattgcgaatactaa**

17770	17780	17790	17800	17810	17820

....|....|....|....|....|....|....|....|....|....|....|....|

**Atalantia_kwangtungens** **tttgttaagaattaatcggattgaggctatagcgtcatcgtttgctggaatcgaaatatc**

**Citrus_reticulata_1** **tttgttaaggattaatcggattgaggctatagcgtcatcgtttgctggaatcgaaatatc**

**Citrus_reticulata_2** **tttgttaaggattaatcggattgaggctatagcgtcatcgtttgctggaatcgaaatatc**

**Citrus_trifoliata_3** **tttgttaagaattaatcggattgaggctatagcgtcatcgtttgctggaatcgaaatatc**

**Citrus_aurantiifolia** **tttgttaagaattaatcggattgaggctatagcgtcatcgtttgctggaatcgaaatatc**

**Citrus_aurantium** **tttgttaagaattaatcggattgaggctatagcgtcatcgtttgctggaatcgaaatatc**

**Citrus_cavaleriei** **tttgttaagaattaatcggattgaggctatagcgtcatcgtttgctggaatcgaaatatc**

**Citrus_hongheensis** **tttgttaagaattaatcggattgaggctatagcgtcatcgtttgctggaatcgaaatatc**

**Citrus_limon** **tttgttaagaattaatcggattgaggctatagcgtcatcgtttgctggaatcgaaatatc**

**Citrus_maxima** **tttgttaagaattaatcggattgaggctatagcgtcatcgtttgctggaatcgaaatatc**

**Citrus_medica** **tttgttaagaattaatcggattgaggctatagcgtcatcatttgctggaatcgaaatatc**

**Citrus_polytrifolia** **tttgttaagaattaatcggattgaggctatagcgtcatcgtttgctggaatcgaaatatc**

**Citrus_reticulata** **tttgttaaggattaatcggattgaggctatagcgtcatcgtttgctggaatcgaaatatc**

**Citrus_sinensis** **tttgttaagaattaatcggattgaggctatagcgtcatcgtttgctggaatcgaaatatc**

**Fortunella_bawangica_1** **tttgttaagaattaatcggattgaggctatagcgtcatcgtttgctggaatcgaaatatc**

**Fortunella_bawangica_2** **tttgttaagaattaatcggattgaggctatagcgtcatcgtttgctggaatcgaaatatc**

**Fortunella_hindsii_1** **tttgttaagaattaatcggattgaggctatagcgtcatcgtttgctggaatcgaaatatc**

**Fortunella_hindsii_2** **tttgttaagaattaatcggattgaggctatagcgtcatcgtttgctggaatcgaaatatc**

**Fortunella_hindsii_3** **tttgttaagaattaatcggattgaggctatagcgtcatcgtttgctggaatcgaaatatc**

**Fortunella_japonica_1** **tttgttaagaattaatcggattgaggctatagcgtcatcgtttgctggaatcgaaatatc**

**Fortunella_japonica_2** **tttgttaagaattaatcggattgaggctatagcgtcatcgtttgctggaatcgaaatatc**

**Fortunella_japonica_3** **tttgttaagaattaatcggattgaggctatagcgtcatcgtttgctggaatcgaaatatc**

**Fortunella_japonica_4** **tttgttaagaattaatcggattgaggctatagcgtcatcgtttgctggaatcgaaatatc**

**Fortunella_japonica_5** **tttgttaagaattaatcggattgaggctatagcgtcatcgtttgctggaatcgaaatatc**

**Fortunella_japonica_6** **tttgttaagaattaatcggattgaggctatagcgtcatcgtttgctggaatcgaaatatc**

**Fortunella_japonica_7** **tttgttaagaattaatcggattgaggctatagcgtcatcgtttgctggaatcgaaatatc**

**Fortunella_japonica_8** **tttgttaagaattaatcggattgaggctatagcgtcatcgtttgctggaatcgaaatatc**

**Fortunella_japonica_9** **tttgttaagaattaatcggattgaggctatagcgtcatcgtttgctggaatcgaaatatc**

**Fortunella_japonica_10** **tttgttaagaattaatcggattgaggctatagcgtcatcgtttgctggaatcgaaatatc**

**Fortunella_japonica_11** **tttgttaagaattaatcggattgaggctatagcgtcatcgtttgctggaatcgaaatatc**

**Fortunella_japonica_12** **tttgttaagaattaatcggattgaggctatagcgtcatcgtttgctggaatcgaaatatc**

**Fortunella_margarita_1** **tttgttaagaattaatcggattgaggctatagcgtcatcgtttgctggaatcgaaatatc**

**Fortunella_margarita_2** **tttgttaagaattaatcggattgaggctatagcgtcatcgtttgctggaatcgaaatatc**

**Fortunella_venosa_1** **tttgttaagaattaatcggattgaggctatagcgtcatcgtttgctggaatcgaaatatc**

**Fortunella_venosa_2** **tttgttaagaattaatcggattgaggctatagcgtcatcgtttgctggaatcgaaatatc**

**Fortunella_venosa_3** **tttgttaagaattaatcggattgaggctatagcgtcatcgtttgctggaatcgaaatatc**

17830	17840	17850	17860	17870	17880

....|....|....|....|....|....|....|....|....|....|....|....|

**Atalantia_kwangtungens** **tgtgagatcggggtcacaatttgtatcgattaaacaaattgttggaattcccaaagtgat**

**Citrus_reticulata_1** **tgcgagatcggggtcacaatttgtatcgattaaacaaattgttgaaattcccaaagtgat**

**Citrus_reticulata_2** **tgcgagatcggggtcacaatttgtatcgattaaacaaattgttgaaattcccaaagtgat**

**Citrus_trifoliata_3** **tgcgagatcggggtcacaatttgtatcgattaaacaaattgttggaattcccaaagtgat**

**Citrus_aurantiifolia** **tgcgagatcggggtcacaatttgtatcgattaaacaaattgttggaattcccaaagtgat**

**Citrus_aurantium** **tgcgagatcggggtcacaatttgtatcgattaaacaaattgttggaattcccaaagtgat**

**Citrus_cavaleriei** **tgcgagatcggggtcacaatttgtatcgattaaacaaattgttggaattcccaaagtgat**

**Citrus_hongheensis** **tgcgagatcggggtcacaatttgtatcgattaaacaaattgttggaattcccaaagtgat**

**Citrus_limon** **tgcgagatcggggtcacaatttgtatcgattaaacaaattgttggaattcccaaagtgat**

**Citrus_maxima** **tgcgagatcggggtcacaatttgtatcgattaaacaaattgttggaattcccaaagtgat**

**Citrus_medica** **tgcgagatcggggtcacaatttgtatcgattaaacaaattgttggaattcccaaagtgat**

**Citrus_polytrifolia** **tgcgagatcggggtcacaatttgtatcgattaaacaaattgttggaattcccaaagtgat**

**Citrus_reticulata** **tgcgagatcggggtcacaatttgtatcgattaaacaaattgttgaaattcccaaagtgat**

**Citrus_sinensis** **tgcgagatcggggtcacaatttgtatcgattaaacaaattgttggaattcccaaagtgat**

**Fortunella_bawangica_1** **tgcgagatcggggtcacaatttgtatcgattaaacaaattgttggaattcccaaagtgat**

**Fortunella_bawangica_2** **tgcgagatcggggtcacaatttgtatcgattaaacaaattgttggaattcccaaagtgat**

**Fortunella_hindsii_1** **tgcgagatcggggtcacaatttgtatcgattaaacaaattgttggaattcccaaagtgat**

**Fortunella_hindsii_2** **tgcgagatcggggtcacaatttgtatcgattaaacaaattgttggaattcccaaagtgat**

**Fortunella_hindsii_3** **tgcgagatcggggtcacaatttgtatcgattaaacaaattgttggaattcccaaagtgat**

**Fortunella_japonica_1** **tgcgagatcggggtcacaatttgtatcgattaaacaaattgttggaattcccaaagtgat**

**Fortunella_japonica_2** **tgcgagatcggggtcacaatttgtatcgattaaacaaattgttggaattcccaaagtgat**

**Fortunella_japonica_3** **tgcgagatcggggtcacaatttgtatcgattaaacaaattgttggaattcccaaagtgat**

**Fortunella_japonica_4** **tgcgagatcggggtcacaatttgtatcgattaaacaaattgttggaattcccaaagtgat**

**Fortunella_japonica_5** **tgcgagatcggggtcacaatttgtatcgattaaacaaattgttggaattcccaaagtgat**

**Fortunella_japonica_6** **tgcgagatcggggtcacaatttgtatcgattaaacaaattgttggaattcccaaagtgat**

**Fortunella_japonica_7** **tgcgagatcggggtcacaatttgtatcgattaaacaaattgttggaattcccaaagtgat**

**Fortunella_japonica_8** **tgcgagatcggggtcacaatttgtatcgattaaacaaattgttggaattcccaaagtgat**

**Fortunella_japonica_9** **tgcgagatcggggtcacaatttgtatcgattaaacaaattgttggaattcccaaagtgat**

**Fortunella_japonica_10** **tgcgagatcggggtcacaatttgtatcgattaaacaaattgttggaattcccaaagtgat**

**Fortunella_japonica_11** **tgcgagatcggggtcacaatttgtatcgattaaacaaattgttggaattcccaaagtgat**

**Fortunella_japonica_12** **tgcgagatcggggtcacaatttgtatcgattaaacaaattgttggaattcccaaagtgat**

**Fortunella_margarita_1** **tgcgagatcggggtcacaatttgtatcgattaaacaaattgttggaattcccaaagtgat**

**Fortunella_margarita_2** **tgcgagatcggggtcacaatttgtatcgattaaacaaattgttggaattcccaaagtgat**

**Fortunella_venosa_1** **tgcgagatcggggtcacaatttgtatcgattaaacaaattgttggaattcccaaagtgat**

**Fortunella_venosa_2** **tgcgagatcggggtcacaatttgtatcgattaaacaaattgttggaattcccaaagtgat**

**Fortunella_venosa_3** **tgcgagatcggggtcacaatttgtatcgattaaacaaattgttggaattcccaaagtgat**

17890	17900	17910	17920	17930	17940

....|....|....|....|....|....|....|....|....|....|....|....|

**Atalantia_kwangtungens** **acattctcgaagagccgtatattcttcttgctgatcaacgacgattacaatatcgggtac**

**Citrus_reticulata_1** **acattctcgaagagccgtatattcttcttgctgatcaacgacgattacaatatcgggtac**

**Citrus_reticulata_2** **acattctcgaagagccgtatattcttcttgctgatcaacgacgattacaatatcgggtac**

**Citrus_trifoliata_3** **acattctcgaagagccgtatattcttcttgctgatcaacgacgattacaatatcgggtac**

**Citrus_aurantiifolia** **acattctcgaagagccgtatattcttcttgctgatcaacgacgattacaatatcgggtac**

**Citrus_aurantium** **acattctcgaagagccgtatattcttcttgctgatcaacgacgattacaatatcgggtac**

**Citrus_cavaleriei** **acattctcgaagagccgtatattcttcttgctgatcaacgacgattacaatatcgggtac**

**Citrus_hongheensis** **acattctcgaagagccgtatattcttcttgctgatcaacgacgattacaatatcgggtac**

**Citrus_limon** **acattctcgaagagccgtatattcttcttgctgatcaacgacgattacaatatcgggtac**

**Citrus_maxima** **acattctcgaagagccgtatattcttcttgctgatcaacgacgattacaatatcgggtac**

**Citrus_medica** **acattctcgaagagccgtatattcttcttgctgatcaacgacgattacaatatcgggtac**

**Citrus_polytrifolia** **acattctcgaagagccgtatattcttcttgctgatcaacgacgattacaatatcgggtac**

**Citrus_reticulata** **acattctcgaagagccgtatattcttcttgctgatcaacgacgattacaatatcgggtac**

**Citrus_sinensis** **acattctcgaagagccgtatattcttcttgctgatcaacgacgattacaatatcgggtac**

**Fortunella_bawangica_1** **acattctcgaagagccgtatattcttcttgctgatcaacgacgattacaatatcgggtac**

**Fortunella_bawangica_2** **acattctcgaagagccgtatattcttcttgctgatcaacgacgattacaatatcgggtac**

**Fortunella_hindsii_1** **acattctcgaagagccgtatattcttcttgctgatcaacgacgattacaatatcgggtac**

**Fortunella_hindsii_2** **acattctcgaagagccgtatattcttcttgctgatcaacgacgattacaatatcgggtac**

**Fortunella_hindsii_3** **acattctcgaagagccgtatattcttcttgctgatcaacgacgattacaatatcgggtac**

**Fortunella_japonica_1** **acattctcgaagagccgtatattcttcttgctgatcaacgacgattacaatatcgggtac**

**Fortunella_japonica_2** **acattctcgaagagccgtatattcttcttgctgatcaacgacgattacaatatcgggtac**

**Fortunella_japonica_3** **acattctcgaagagccgtatattcttcttgctgatcaacgacgattacaatatcgggtac**

**Fortunella_japonica_4** **acattctcgaagagccgtatattcttcttgctgatcaacgacgattacaatatcgggtac**

**Fortunella_japonica_5** **acattctcgaagagccgtatattcttcttgctgatcaacgacgattacaatatcgggtac**

**Fortunella_japonica_6** **acattctcgaagagccgtatattcttcttgctgatcaacgacgattacaatatcgggtac**

**Fortunella_japonica_7** **acattctcgaagagccgtatattcttcttgctgatcaacgacgattacaatatcgggtac**

**Fortunella_japonica_8** **acattctcgaagagccgtatattcttcttgctgatcaacgacgattacaatatcgggtac**

**Fortunella_japonica_9** **acattctcgaagagccgtatattcttcttgctgatcaacgacgattacaatatcgggtac**

**Fortunella_japonica_10** **acattctcgaagagccgtatattcttcttgctgatcaacgacgattacaatatcgggtac**

**Fortunella_japonica_11** **acattctcgaagagccgtatattcttcttgctgatcaacgacgattacaatatcgggtac**

**Fortunella_japonica_12** **acattctcgaagagccgtatattcttcttgctgatcaacgacgattacaatatcgggtac**

**Fortunella_margarita_1** **acattctcgaagagccgtatattcttcttgctgatcaacgacgattacaatatcgggtac**

**Fortunella_margarita_2** **acattctcgaagagccgtatattcttcttgctgatcaacgacgattacaatatcgggtac**

**Fortunella_venosa_1** **acattctcgaagagccgtatattcttcttgctgatcaacgacgattacaatatcgggtac**

**Fortunella_venosa_2** **acattctcgaagagccgtatattcttcttgctgatcaacgacgattacaatatcgggtac**

**Fortunella_venosa_3** **acattctcgaagagccgtatattcttcttgctgatcaacgacgattacaatatcgggtac**

17950	17960	17970	17980	17990	18000

....|....|....|....|....|....|....|....|....|....|....|....|

**Atalantia_kwangtungens** **cctcgtcatatatttaatcccgcccagatacgtttgcagacgcgataattgtctcttcaa**

**Citrus_reticulata_1** **cctcgtcatatatttaattccgcccagatacgtttgcagacgcgataattgtctcttcaa**

**Citrus_reticulata_2** **cctcgtcatatatttaattccgcccagatacgtttgcagacgcgataattgtctcttcaa**

**Citrus_trifoliata_3** **cctcgtcatatatttaatcccgcccagatacgtttgcagacgcgataattgtctcttcaa**

**Citrus_aurantiifolia** **cctcgtcatatatttaatcccgcccagatacgtttgcagacgcgataattgtctcttcaa**

**Citrus_aurantium** **cctcgtcatatatttaatcccgcccagatacgtttgcagacgcgataattgtctcttcaa**

**Citrus_cavaleriei** **cctcgtcatatatttaattccgcccagatacgtttgcagacgcgataattgtctcttcaa**

**Citrus_hongheensis** **cctcgtcatatatttaatcccgcccagatacgtttgcagacgcgataattgtctcttcaa**

**Citrus_limon** **cctcgtcatatatttaatcccgcccagatacgtttgcagacgcgataattgtctcttcaa**

**Citrus_maxima** **cctcgtcatatatttaatcccgcccagatacgtttgcagacgcgataattgtctcttcaa**

**Citrus_medica** **cctcgtcatatatttaatcccgcccagatacgtttgcagacgcgataattgtctcttcaa**

**Citrus_polytrifolia** **cctcgtcatatatttaatcccgcccagatacgtttgcagacgcgataattgtctcttcaa**

**Citrus_reticulata** **cctcgtcatatatttaattccgcccagatacgtttgcagacgcgataattgtctcttcaa**

**Citrus_sinensis** **cctcgtcatatatttaatcccgcccagatacgtttgcagacgcgataattgtctcttcaa**

**Fortunella_bawangica_1** **cctcgtcatatatttaatcccgcccagatacgtttgcagacgcgataattgtctcttcaa**

**Fortunella_bawangica_2** **cctcgtcatatatttaatcccgcccagatacgtttgcagacgcgataattgtctcttcaa**

**Fortunella_hindsii_1** **cctcgtcatatatttaatcccgcccagatacgtttgcagacgcgataattgtctcttcaa**

**Fortunella_hindsii_2** **cctcgtcatatatttaatcccgcccagatacgtttgcagacgcgataattgtctcttcaa**

**Fortunella_hindsii_3** **cctcgtcatatatttaatcccgcccagatacgtttgcagacgcgataattgtctcttcaa**

**Fortunella_japonica_1** **cctcgtcatatatttaatcccgcccagatacgtttgcagacgcgataattgtctcttcaa**

**Fortunella_japonica_2** **cctcgtcatatatttaatcccgcccagatacgtttgcagacgcgataattgtctcttcaa**

**Fortunella_japonica_3** **cctcgtcatatatttaatcccgcccagatacgtttgcagacgcgataattgtctcttcaa**

**Fortunella_japonica_4** **cctcgtcatatatttaatcccgcccagatacgtttgcagacgcgataattgtctcttcaa**

**Fortunella_japonica_5** **cctcgtcatatatttaatcccgcccagatacgtttgcagacgcgataattgtctcttcaa**

**Fortunella_japonica_6** **cctcgtcatatatttaatcccgcccagatacgtttgcagacgcgataattgtctcttcaa**

**Fortunella_japonica_7** **cctcgtcatatatttaatcccgcccagatacgtttgcagacgcgataattgtctcttcaa**

**Fortunella_japonica_8** **cctcgtcatatatttaatcccgcccagatacgtttgcagacgcgataattgtctcttcaa**

**Fortunella_japonica_9** **cctcgtcatatatttaatcccgcccagatacgtttgcagacgcgataattgtctcttcaa**

**Fortunella_japonica_10** **cctcgtcatatatttaatcccgcccagatacgtttgcagacgcgataattgtctcttcaa**

**Fortunella_japonica_11** **cctcgtcatatatttaatcccgcccagatacgtttgcagacgcgataattgtctcttcaa**

**Fortunella_japonica_12** **cctcgtcatatatttaatcccgcccagatacgtttgcagacgcgataattgtctcttcaa**

**Fortunella_margarita_1** **cctcgtcatatatttaatcccgcccagatacgtttgcagacgcgataattgtctcttcaa**

**Fortunella_margarita_2** **cctcgtcatatatttaatcccgcccagatacgtttgcagacgcgataattgtctcttcaa**

**Fortunella_venosa_1** **cctcgtcatatatttaatcccgcccagatacgtttgcagacgcgataattgtctcttcaa**

**Fortunella_venosa_2** **cctcgtcatatatttaatcccgcccagatacgtttgcagacgcgataattgtctcttcaa**

**Fortunella_venosa_3** **cctcgtcatatatttaatcccgcccagatacgtttgcagacgcgataattgtctcttcaa**

18010	18020	18030	18040	18050	18060

....|....|....|....|....|....|....|....|....|....|....|....|

**Atalantia_kwangtungens** **aatagcggcatcccttttgggaagactgtcgagtctcccccctttttgttccgttctcaa**

**Citrus_reticulata_1** **aatagcggcatcccttttgggaagactgtcgagtctcccccctttttgttccgttctcaa**

**Citrus_reticulata_2** **aatagcggcatcccttttgggaagactgtcgagtctcccccctttttgttccgttctcaa**

**Citrus_trifoliata_3** **aatagcggcatcccttttgggaagactgtcgagtctcccccctttttgttccgttctcaa**

**Citrus_aurantiifolia** **aatagcggcatcccttttgggaagactgtcgagtctcccccctttttgttccgttctcaa**

**Citrus_aurantium** **aatagcggcatcccttttgggaagactgtcgagtctcccccctttttgttccgttctcaa**

**Citrus_cavaleriei** **aatagcggcatcccttttgggaagactgtcgagtctcccccctttttgttccgttctcaa**

**Citrus_hongheensis** **aatagcggcatcccttttgggaagactgtcgagtctcccccctttttgttccgttctcaa**

**Citrus_limon** **aatagcggcatcccttttgggaagactgtcgagtctcccccctttttgttccgttctcaa**

**Citrus_maxima** **aatagcggcatcccttttgggaagactgtcgagtctcccccctttttgttccgttctcaa**

**Citrus_medica** **aatagcggcatcccttttgggaagactgttgagtctcccccctttttgttccgttctcaa**

**Citrus_polytrifolia** **aatagcggcatcccttttgggaagactgtcgagtctcccccctttttgttccgttctcaa**

**Citrus_reticulata** **aatagcggcatcccttttgggaagactgtcgagtctcccccctttttgttccgttctcaa**

**Citrus_sinensis** **aatagcggcatcccttttgggaagactgtcgagtctcccccctttttgttccgttctcaa**

**Fortunella_bawangica_1** **aatagcggcatcccttttgggaagactgtcgagtctcccccctttttgttccgttctcaa**

**Fortunella_bawangica_2** **aatagcggcatcccttttgggaagactgtcgagtctcccccctttttgttccgttctcaa**

**Fortunella_hindsii_1** **aatagcggcatcccttttgggaagactgtcgagtctcccccctttttgttccgttctcaa**

**Fortunella_hindsii_2** **aatagcggcatcccttttgggaagactgtcgagtctcccccctttttgttccgttctcaa**

**Fortunella_hindsii_3** **aatagcggcatcccttttgggaagactgtcgagtctcccccctttttgttccgttctcaa**

**Fortunella_japonica_1** **aatagcggcatcccttttgggaagactgtcgagtctcccccctttttgttccgttctcaa**

**Fortunella_japonica_2** **aatagcggcatcccttttgggaagactgtcgagtctcccccctttttgttccgttctcaa**

**Fortunella_japonica_3** **aatagcggcatcccttttgggaagactgtcgagtctcccccctttttgttccgttctcaa**

**Fortunella_japonica_4** **aatagcggcatcccttttgggaagactgtcgagtctcccccctttttgttccgttctcaa**

**Fortunella_japonica_5** **aatagcggcatcccttttgggaagactgtcgagtctcccccctttttgttccgttctcaa**

**Fortunella_japonica_6** **aatagcggcatcccttttgggaagactgtcgagtctcccccctttttgttccgttctcaa**

**Fortunella_japonica_7** **aatagcggcatcccttttgggaagactgtcgagtctcccccctttttgttccgttctcaa**

**Fortunella_japonica_8** **aatagcggcatcccttttgggaagactgtcgagtctcccccctttttgttccgttctcaa**

**Fortunella_japonica_9** **aatagcggcatcccttttgggaagactgtcgagtctcccccctttttgttccgttctcaa**

**Fortunella_japonica_10** **aatagcggcatcccttttgggaagactgtcgagtctcccccctttttgttccgttctcaa**

**Fortunella_japonica_11** **aatagcggcatcccttttgggaagactgtcgagtctcccccctttttgttccgttctcaa**

**Fortunella_japonica_12** **aatagcggcatcccttttgggaagactgtcgagtctcccccctttttgttccgttctcaa**

**Fortunella_margarita_1** **aatagcggcatcccttttgggaagactgtcgagtctcccccctttttgttccgttctcaa**

**Fortunella_margarita_2** **aatagcggcatcccttttgggaagactgtcgagtctcccccctttttgttccgttctcaa**

**Fortunella_venosa_1** **aatagcggcatcccttttgggaagactgtcgagtctcccccctttttgttccgttctcaa**

**Fortunella_venosa_2** **aatagcggcatcccttttgggaagactgtcgagtctcccccctttttgttccgttctcaa**

**Fortunella_venosa_3** **aatagcggcatcccttttgggaagactgtcgagtctcccccctttttgttccgttctcaa**

18070	18080	18090	18100	18110	18120

....|....|....|....|....|....|....|....|....|....|....|....|

**Atalantia_kwangtungens** **atccctgaacttgtgaagtcgcgtttctgtagtggaccaattggttaacataccgccgag**

**Citrus_reticulata_1** **atccctgaacttgtgaagtcgcgtttctgtagtggaccaattggttaacataccgccgag**

**Citrus_reticulata_2** **atccctgaacttgtgaagtcgcgtttctgtagtggaccaattggttaacataccgccgag**

**Citrus_trifoliata_3** **atccctgaacttgtgaagtcgcgtttctgtagtggaccaattggttaacataccgccgag**

**Citrus_aurantiifolia** **atccctgaacttgtgaagtcgcgtttctgtagtggaccaattggttaacataccgccgag**

**Citrus_aurantium** **atccctgaacttgtgaagtcgcgtttctgtagtggaccaattggttaacataccgccgag**

**Citrus_cavaleriei** **atccctgaacttgtgaagtcgcgtttctgtagtggaccaattggttaacataccgccgag**

**Citrus_hongheensis** **atccctgaacttgtgaagtcgcgtttctgtagtggaccaattggttaacataccgccgag**

**Citrus_limon** **atccctgaacttgtgaagtcgcgtttctgtagtggaccaattggttaacataccgccgag**

**Citrus_maxima** **atccctgaacttgtgaagtcgcgtttctgtagtggaccaattggttaacataccgccgag**

**Citrus_medica** **atccctgaacttgtgaagtcgcgtttctgtagtggaccaattggttaacataccgccgag**

**Citrus_polytrifolia** **atccctgaacttgtgaagtcgcgtttctgtagtggaccaattggttaacataccgccgag**

**Citrus_reticulata** **atccctgaacttgtgaagtcgcgtttctgtagtggaccaattggttaacataccgccgag**

**Citrus_sinensis** **atccctgaacttgtgaagtcgcgtttctgtagtggaccaattggttaacataccgccgag**

**Fortunella_bawangica_1** **atccctgaacttgtgaagtcgcgtttctgtagtggaccaattggttaacataccgccgag**

**Fortunella_bawangica_2** **atccctgaacttgtgaagtcgcgtttctgtagtggaccaattggttaacataccgccgag**

**Fortunella_hindsii_1** **atccctgaacttgtgaagtcgcgtttctgtagtggaccaattggttaacataccgccgag**

**Fortunella_hindsii_2** **atccctgaacttgtgaagtcgcgtttctgtagtggaccaattggttaacataccgccgag**

**Fortunella_hindsii_3** **atccctgaacttgtgaagtcgcgtttctgtagtggaccaattggttaacataccgccgag**

**Fortunella_japonica_1** **atccctgaacttgtgaagtcgcgtttctgtagtggaccaattggttaacataccgccgag**

**Fortunella_japonica_2** **atccctgaacttgtgaagtcgcgtttctgtagtggaccaattggttaacataccgccgag**

**Fortunella_japonica_3** **atccctgaacttgtgaagtcgcgtttctgtagtggaccaattggttaacataccgccgag**

**Fortunella_japonica_4** **atccctgaacttgtgaagtcgcgtttctgtagtggaccaattggttaacataccgccgag**

**Fortunella_japonica_5** **atccctgaacttgtgaagtcgcgtttctgtagtggaccaattggttaacataccgccgag**

**Fortunella_japonica_6** **atccctgaacttgtgaagtcgcgtttctgtagtggaccaattggttaacataccgccgag**

**Fortunella_japonica_7** **atccctgaacttgtgaagtcgcgtttctgtagtggaccaattggttaacataccgccgag**

**Fortunella_japonica_8** **atccctgaacttgtgaagtcgcgtttctgtagtggaccaattggttaacataccgccgag**

**Fortunella_japonica_9** **atccctgaacttgtgaagtcgcgtttctgtagtggaccaattggttaacataccgccgag**

**Fortunella_japonica_10** **atccctgaacttgtgaagtcgcgtttctgtagtggaccaattggttaacataccgccgag**

**Fortunella_japonica_11** **atccctgaacttgtgaagtcgcgtttctgtagtggaccaattggttaacataccgccgag**

**Fortunella_japonica_12** **atccctgaacttgtgaagtcgcgtttctgtagtggaccaattggttaacataccgccgag**

**Fortunella_margarita_1** **atccctgaacttgtgaagtcgcgtttctgtagtggaccaattggttaacataccgccgag**

**Fortunella_margarita_2** **atccctgaacttgtgaagtcgcgtttctgtagtggaccaattggttaacataccgccgag**

**Fortunella_venosa_1** **atccctgaacttgtgaagtcgcgtttctgtagtggaccaattggttaacataccgccgag**

**Fortunella_venosa_2** **atccctgaacttgtgaagtcgcgtttctgtagtggaccaattggttaacataccgccgag**

**Fortunella_venosa_3** **atccctgaacttgtgaagtcgcgtttctgtagtggaccaattggttaacataccgccgag**

18130	18140	18150	18160	18170	18180

....|....|....|....|....|....|....|....|....|....|....|....|

**Atalantia_kwangtungens** **ccatttttgattaacataatggcaccgagcccttattgcagctcgcgcgactaaatcagc**

**Citrus_reticulata_1** **ccatttttgattaacataatggcaccgagcccttattgcagctcgcgcgactaaatcagc**

**Citrus_reticulata_2** **ccatttttgattaacataatggcaccgagcccttattgcagctcgcgcgactaaatcagc**

**Citrus_trifoliata_3** **ccatttttgattaacataatggcaccgagcccttattgcagctcgcgcgactaaatcagc**

**Citrus_aurantiifolia** **ccatttttgattaacataatggcaccgagcccttattgcagctcgcgcgactaaatcagc**

**Citrus_aurantium** **ccatttttgattaacataatggcaccgagcccttattgcagctcgcgcgactaaatcagc**

**Citrus_cavaleriei** **ccatttttgattaacataatggcaccgagcccttattgcagctcgcgcgactaaatcagc**

**Citrus_hongheensis** **ccatttttgattaacataatggcaccgagcccttattgcagctcgcgcgactaaatcagc**

**Citrus_limon** **ccatttttgattaacataatggcaccgagcccttattgcagctcgcgcgactaaatcagc**

**Citrus_maxima** **ccatttttgattaacataatggcaccgagcccttattgcagctcgcgcgactaaatcagc**

**Citrus_medica** **ccatttttgattaacataatggcaccgagcccttattgcagctcgcgcgactaaatcagc**

**Citrus_polytrifolia** **ccatttttgattaacataatggcaccgagcccttattgcagctcgcgcgactaaatcagc**

**Citrus_reticulata** **ccatttttgattaacataatggcaccgagcccttattgcagctcgcgcgactaaatcagc**

**Citrus_sinensis** **ccatttttgattaacataatggcaccgagcccttattgcagctcgcgcgactaaatcagc**

**Fortunella_bawangica_1** **ccatttttgattaacataatggcaccgagcccttattgcagctcgcgcgactaaatcagc**

**Fortunella_bawangica_2** **ccatttttgattaacataatggcaccgagcccttattgcagctcgcgcgactaaatcagc**

**Fortunella_hindsii_1** **ccatttttgattaacataatggcaccgagcccttattgcagctcgcgcgactaaatcagc**

**Fortunella_hindsii_2** **ccatttttgattaacataatggcaccgagcccttattgcagctcgcgcgactaaatcagc**

**Fortunella_hindsii_3** **ccatttttgattaacataatggcaccgagcccttattgcagctcgcgcgactaaatcagc**

**Fortunella_japonica_1** **ccatttttgattaacataatggcaccgagcccttattgcagctcgcgcgactaaatcagc**

**Fortunella_japonica_2** **ccatttttgattaacataatggcaccgagcccttattgcagctcgcgcgactaaatcagc**

**Fortunella_japonica_3** **ccatttttgattaacataatggcaccgagcccttattgcagctcgcgcgactaaatcagc**

**Fortunella_japonica_4** **ccatttttgattaacataatggcaccgagcccttattgcagctcgcgcgactaaatcagc**

**Fortunella_japonica_5** **ccatttttgattaacataatggcaccgagcccttattgcagctcgcgcgactaaatcagc**

**Fortunella_japonica_6** **ccatttttgattaacataatggcaccgagcccttattgcagctcgcgcgactaaatcagc**

**Fortunella_japonica_7** **ccatttttgattaacataatggcaccgagcccttattgcagctcgcgcgactaaatcagc**

**Fortunella_japonica_8** **ccatttttgattaacataatggcaccgagcccttattgcagctcgcgcgactaaatcagc**

**Fortunella_japonica_9** **ccatttttgattaacataatggcaccgagcccttattgcagctcgcgcgactaaatcagc**

**Fortunella_japonica_10** **ccatttttgattaacataatggcaccgagcccttattgcagctcgcgcgactaaatcagc**

**Fortunella_japonica_11** **ccatttttgattaacataatggcaccgagcccttattgcagctcgcgcgactaaatcagc**

**Fortunella_japonica_12** **ccatttttgattaacataatggcaccgagcccttattgcagctcgcgcgactaaatcagc**

**Fortunella_margarita_1** **ccatttttgattaacataatggcaccgagcccttattgcagctcgcgcgactaaatcagc**

**Fortunella_margarita_2** **ccatttttgattaacataatggcaccgagcccttattgcagctcgcgcgactaaatcagc**

**Fortunella_venosa_1** **ccatttttgattaacataatggcaccgagcccttattgcagctcgcgcgactaaatcagc**

**Fortunella_venosa_2** **ccatttttgattaacataatggcaccgagcccttattgcagctcgcgcgactaaatcagc**

**Fortunella_venosa_3** **ccatttttgattaacataatggcaccgagcccttattgcagctcgcgcgactaaatcagc**

18190	18200	18210	18220	18230	18240

....|....|....|....|....|....|....|....|....|....|....|....|

**Atalantia_kwangtungens** **tgctttatttttagtaccaacaattaagaattgttttcccctacttgctgcatcaaaaac**

**Citrus_reticulata_1** **tgctttatttttagtaccaacaattaagaattgttttcccctacttgctgcatcaaaaac**

**Citrus_reticulata_2** **tgctttatttttagtaccaacaattaagaattgttttcccctacttgctgcatcaaaaac**

**Citrus_trifoliata_3** **tgctttatttttagtaccaacaattaagaattgttttcccctacttgctgcatcaaaaac**

**Citrus_aurantiifolia** **tgctttatttttagtaccaacaattaagaattgttttcccctacttgctgcatcaaaaac**

**Citrus_aurantium** **tgctttatttttagtaccaacaattaagaattgttttcccctacttgctgcatcaaaaac**

**Citrus_cavaleriei** **tgctttatttttagtaccaacaattaagaattgttttcccctacttgctgcatcaaaaac**

**Citrus_hongheensis** **tgctttatttttagtaccaacaattaagaattgttttcccctacttgctgcatcaaaaac**

**Citrus_limon** **tgctttatttttagtaccaacaattaagaattgttttcccctacttgctgcatcaaaaac**

**Citrus_maxima** **tgctttatttttagtaccaacaattaagaattgttttcccctacttgctgcatcaaaaac**

**Citrus_medica** **tgctttatttttagtaccaacaattaagaattgttttcccctacttgctgcatcaaaaac**

**Citrus_polytrifolia** **tgctttatttttagtaccaacaattaagaattgttttcccctacttgctgcatcaaaaac**

**Citrus_reticulata** **tgctttatttttagtaccaacaattaagaattgttttcccctacttgctgcatcaaaaac**

**Citrus_sinensis** **tgctttatttttagtaccaacaattaagaattgttttcccctacttgctgcatcaaaaac**

**Fortunella_bawangica_1** **tgctttatttttagtaccaacaattaagaattgttttcccctacttgctgcatcaaaaac**

**Fortunella_bawangica_2** **tgctttatttttagtaccaacaattaagaattgttttcccctacttgctgcatcaaaaac**

**Fortunella_hindsii_1** **tgctttatttttagtaccaacaattaagaattgttttcccctacttgctgcatcaaaaac**

**Fortunella_hindsii_2** **tgctttatttttagtaccaacaattaagaattgttttcccctacttgctgcatcaaaaac**

**Fortunella_hindsii_3** **tgctttatttttagtaccaacaattaagaattgttttcccctacttgctgcatcaaaaac**

**Fortunella_japonica_1** **tgctttatttttagtaccaacaattaagaattgttttcccctacttgctgcatcaaaaac**

**Fortunella_japonica_2** **tgctttatttttagtaccaacaattaagaattgttttcccctacttgctgcatcaaaaac**

**Fortunella_japonica_3** **tgctttatttttagtaccaacaattaagaattgttttcccctacttgctgcatcaaaaac**

**Fortunella_japonica_4** **tgctttatttttagtaccaacaattaagaattgttttcccctacttgctgcatcaaaaac**

**Fortunella_japonica_5** **tgctttatttttagtaccaacaattaagaattgttttcccctacttgctgcatcaaaaac**

**Fortunella_japonica_6** **tgctttatttttagtaccaacaattaagaattgttttcccctacttgctgcatcaaaaac**

**Fortunella_japonica_7** **tgctttatttttagtaccaacaattaagaattgttttcccctacttgctgcatcaaaaac**

**Fortunella_japonica_8** **tgctttatttttagtaccaacaattaagaattgttttcccctacttgctgcatcaaaaac**

**Fortunella_japonica_9** **tgctttatttttagtaccaacaattaagaattgttttcccctacttgctgcatcaaaaac**

**Fortunella_japonica_10** **tgctttatttttagtaccaacaattaagaattgttttcccctacttgctgcatcaaaaac**

**Fortunella_japonica_11** **tgctttatttttagtaccaacaattaagaattgttttcccctacttgctgcatcaaaaac**

**Fortunella_japonica_12** **tgctttatttttagtaccaacaattaagaattgttttcccctacttgctgcatcaaaaac**

**Fortunella_margarita_1** **tgctttatttttagtaccaacaattaagaattgttttcccctacttgctgcatcaaaaac**

**Fortunella_margarita_2** **tgctttatttttagtaccaacaattaagaattgttttcccctacttgctgcatcaaaaac**

**Fortunella_venosa_1** **tgctttatttttagtaccaacaattaagaattgttttcccctacttgctgcatcaaaaac**

**Fortunella_venosa_2** **tgctttatttttagtaccaacaattaagaattgttttcccctacttgctgcatcaaaaac**

**Fortunella_venosa_3** **tgctttatttttagtaccaacaattaagaattgttttcccctacttgctgcatcaaaaac**

18250	18260	18270	18280	18290	18300

....|....|....|....|....|....|....|....|....|....|....|....|

**Atalantia_kwangtungens** **taaatcacaagcttctgataaaaaccgagcagttcgagtcagatttgtaatatgaatacc**

**Citrus_reticulata_1** **taaatcacaagcttctgataaaaaccgagcagttcgagtcagatttgtaatatgaatacc**

**Citrus_reticulata_2** **taaatcacaagcttctgataaaaaccgagcagttcgagtcagatttgtaatatgaatacc**

**Citrus_trifoliata_3** **taaatcacaagcttctgataaaaaccgagcagttcgagtcagatttgtaatatgaatacc**

**Citrus_aurantiifolia** **taaatcacaagcttctgataaaaaccgagcagttcgagtcagatttgtaatatgaatacc**

**Citrus_aurantium** **taaatcacaagcttctgataaaaaccgagcagttcgagtcagatttgtaatatgaatacc**

**Citrus_cavaleriei** **taaatcacaagcttctgataaaaaccgagcagttcgagtcagatttgtaatatgaatacc**

**Citrus_hongheensis** **taaatcacaagcttctgataaaaaccgagcagttcgagtcagatttgtaatatgaatacc**

**Citrus_limon** **taaatcacaagcttctgataaaaaccgagcagttcgagtcagatttgtaatatgaatacc**

**Citrus_maxima** **taaatcacaagcttctgataaaaaccgagcagttcgagtcagatttgtaatatgaatacc**

**Citrus_medica** **taaatcacaagcttctgataaaaaccgagcagttcgagtcagatttgtaatatgaatacc**

**Citrus_polytrifolia** **taaatcacaagcttctgataaaaaccgagcagttcgagtcagatttgtaatatgaatacc**

**Citrus_reticulata** **taaatcacaagcttctgataaaaaccgagcagttcgagtcagatttgtaatatgaatacc**

**Citrus_sinensis** **taaatcacaagcttctgataaaaaccgagcagttcgagtcagatttgtaatatgaatacc**

**Fortunella_bawangica_1** **taaatcacaagcttctgataaaaaccgagcagttcgagtcagatttgtaatatgaatacc**

**Fortunella_bawangica_2** **taaatcacaagcttctgataaaaaccgagcagttcgagtcagatttgtaatatgaatacc**

**Fortunella_hindsii_1** **taaatcacaagcttctgataaaaaccgagcagttcgagtcagatttgtaatatgaatacc**

**Fortunella_hindsii_2** **taaatcacaagcttctgataaaaaccgagcagttcgagtcagatttgtaatatgaatacc**

**Fortunella_hindsii_3** **taaatcacaagcttctgataaaaaccgagcagttcgagtcagatttgtaatatgaatacc**

**Fortunella_japonica_1** **taaatcacaagcttctgataaaaaccgagcagttcgagtcagatttgtaatatgaatacc**

**Fortunella_japonica_2** **taaatcacaagcttctgataaaaaccgagcagttcgagtcagatttgtaatatgaatacc**

**Fortunella_japonica_3** **taaatcacaagcttctgataaaaaccgagcagttcgagtcagatttgtaatatgaatacc**

**Fortunella_japonica_4** **taaatcacaagcttctgataaaaaccgagcagttcgagtcagatttgtaatatgaatacc**

**Fortunella_japonica_5** **taaatcacaagcttctgataaaaaccgagcagttcgagtcagatttgtaatatgaatacc**

**Fortunella_japonica_6** **taaatcacaagcttctgataaaaaccgagcagttcgagtcagatttgtaatatgaatacc**

**Fortunella_japonica_7** **taaatcacaagcttctgataaaaaccgagcagttcgagtcagatttgtaatatgaatacc**

**Fortunella_japonica_8** **taaatcacaagcttctgataaaaaccgagcagttcgagtcagatttgtaatatgaatacc**

**Fortunella_japonica_9** **taaatcacaagcttctgataaaaaccgagcagttcgagtcagatttgtaatatgaatacc**

**Fortunella_japonica_10** **taaatcacaagcttctgataaaaaccgagcagttcgagtcagatttgtaatatgaatacc**

**Fortunella_japonica_11** **taaatcacaagcttctgataaaaaccgagcagttcgagtcagatttgtaatatgaatacc**

**Fortunella_japonica_12** **taaatcacaagcttctgataaaaaccgagcagttcgagtcagatttgtaatatgaatacc**

**Fortunella_margarita_1** **taaatcacaagcttctgataaaaaccgagcagttcgagtcagatttgtaatatgaatacc**

**Fortunella_margarita_2** **taaatcacaagcttctgataaaaaccgagcagttcgagtcagatttgtaatatgaatacc**

**Fortunella_venosa_1** **taaatcacaagcttctgataaaaaccgagcagttcgagtcagatttgtaatatgaatacc**

**Fortunella_venosa_2** **taaatcacaagcttctgataaaaaccgagcagttcgagtcagatttgtaatatgaatacc**

**Fortunella_venosa_3** **taaatcacaagcttctgataaaaaccgagcagttcgagtcagatttgtaatatgaatacc**

18310	18320	18330	18340	18350	18360

....|....|....|....|....|....|....|....|....|....|....|....|

**Atalantia_kwangtungens** **tttatgttttgcagatatataaggtgccattctaggattccatttccgagtaccatgacc**

**Citrus_reticulata_1** **tttatgttttgcagatatataaggtgccattctaggattccatttccgagtaccatgacc**

**Citrus_reticulata_2** **tttatgttttgcagatatataaggtgccattctaggattccatttccgagtaccatgacc**

**Citrus_trifoliata_3** **tttatgttttgcagatatataaggtgccattctaggattccatttccgagtaccatgacc**

**Citrus_aurantiifolia** **tttatgttttgcagatatataaggtgccattctaggattccatttccgagtaccatgacc**

**Citrus_aurantium** **tttatgttttgcagatatataaggtgccattctaggattccatttccgagtaccatgacc**

**Citrus_cavaleriei** **tttatgttttgcagatatataaggtgccattctaggattccatttccgagtaccatgacc**

**Citrus_hongheensis** **tttatgttttgcagatatataaggtgccattctaggattccatttccgagtaccatgacc**

**Citrus_limon** **tttatgttttgcagatatataaggtgccattctaggattccatttccgagtaccatgacc**

**Citrus_maxima** **tttatgttttgcagatatataaggtgccattctaggattccatttccgagtaccatgacc**

**Citrus_medica** **tttatgttttgcagatatataaggtgccattctaggattccatttccgagtaccatgacc**

**Citrus_polytrifolia** **tttatgttttgcagatatataaggtgccattctaggattccatttccgagtaccatgacc**

**Citrus_reticulata** **tttatgttttgcagatatataaggtgccattctaggattccatttccgagtaccatgacc**

**Citrus_sinensis** **tttatgttttgcagatatataaggtgccattctaggattccatttccgagtaccatgacc**

**Fortunella_bawangica_1** **tttatgttttgcagatatataaggtgccattctaggattccatttccgagtaccatgacc**

**Fortunella_bawangica_2** **tttatgttttgcagatatataaggtgccattctaggattccatttccgagtaccatgacc**

**Fortunella_hindsii_1** **tttatgttttgcagatatataaggtgccattctaggattccatttccgagtaccatgacc**

**Fortunella_hindsii_2** **tttatgttttgcagatatataaggtgccattctaggattccatttccgagtaccatgacc**

**Fortunella_hindsii_3** **tttatgttttgcagatatataaggtgccattctaggattccatttccgagtaccatgacc**

**Fortunella_japonica_1** **tttatgttttgcagatatataaggtgccattctaggattccatttccgagtaccatgacc**

**Fortunella_japonica_2** **tttatgttttgcagatatataaggtgccattctaggattccatttccgagtaccatgacc**

**Fortunella_japonica_3** **tttatgttttgcagatatataaggtgccattctaggattccatttccgagtaccatgacc**

**Fortunella_japonica_4** **tttatgttttgcagatatataaggtgccattctaggattccatttccgagtaccatgacc**

**Fortunella_japonica_5** **tttatgttttgcagatatataaggtgccattctaggattccatttccgagtaccatgacc**

**Fortunella_japonica_6** **tttatgttttgcagatatataaggtgccattctaggattccatttccgagtaccatgacc**

**Fortunella_japonica_7** **tttatgttttgcagatatataaggtgccattctaggattccatttccgagtaccatgacc**

**Fortunella_japonica_8** **tttatgttttgcagatatataaggtgccattctaggattccatttccgagtaccatgacc**

**Fortunella_japonica_9** **tttatgttttgcagatatataaggtgccattctaggattccatttccgagtaccatgacc**

**Fortunella_japonica_10** **tttatgttttgcagatatataaggtgccattctaggattccatttccgagtaccatgacc**

**Fortunella_japonica_11** **tttatgttttgcagatatataaggtgccattctaggattccatttccgagtaccatgacc**

**Fortunella_japonica_12** **tttatgttttgcagatatataaggtgccattctaggattccatttccgagtaccatgacc**

**Fortunella_margarita_1** **tttatgttttgcagatatataaggtgccattctaggattccatttccgagtaccatgacc**

**Fortunella_margarita_2** **tttatgttttgcagatatataaggtgccattctaggattccatttccgagtaccatgacc**

**Fortunella_venosa_1** **tttatgttttgcagatatataaggtgccattctaggattccatttccgagtaccatgacc**

**Fortunella_venosa_2** **tttatgttttgcagatatataaggtgccattctaggattccatttccgagtaccatgacc**

**Fortunella_venosa_3** **tttatgttttgcagatatataaggtgccattctaggattccatttccgagtaccatgacc**

18370	18380	18390	18400	18410	18420

....|....|....|....|....|....|....|....|....|....|....|....|

**Atalantia_kwangtungens** **aaaatgaattcctgcttccatcatctcttccaaatggatgttccaataccttcttgccat**

**Citrus_reticulata_1** **aaaatgaattcctgcttccatcatctcttccaaatggatgttccaataccttcttgccat**

**Citrus_reticulata_2** **aaaatgaattcctgcttccatcatctcttccaaatggatgttccaataccttcttgccat**

**Citrus_trifoliata_3** **aaaatgaattcctgcttccatcatctcttccaaatggatgttccaataccttcttgccat**

**Citrus_aurantiifolia** **aaaatgaattcctgcttccatcatctcttccaaatggatgttccaataccttcttgccat**

**Citrus_aurantium** **aaaatgaattcccgcttccatcatctcttccaaatggatgttccaataccttcttgccat**

**Citrus_cavaleriei** **aaaatgaattcctgcttccatcatctcttccaaatggatgttccaataccttcttgccat**

**Citrus_hongheensis** **aaaatgaattcctgcttccatcatctcttccaaatggatgttccaataccttcttgccat**

**Citrus_limon** **aaaatgaattcccgcttccatcatctcttccaaatggatgttccaataccttcttgccat**

**Citrus_maxima** **aaaatgaattcctgcttccatcatctcttccaaatggatgttccaataccttcttgccat**

**Citrus_medica** **aaaatgaattcctgcttccatcatctcttccaaatggatgttccaataccttcttgccat**

**Citrus_polytrifolia** **aaaatgaattcctgcttccatcatctcttccaaatggatgttccaataccttcttgccat**

**Citrus_reticulata** **aaaatgaattcctgcttccatcatctcttccaaatggatgttccaataccttcttgccat**

**Citrus_sinensis** **aaaatgaattcctgcttccatcatctcttccaaatggatgttccaataccttcttgccat**

**Fortunella_bawangica_1** **aaaatgaattcctgcttccatcatctcttccaaatggatgttccaataccttcttgccat**

**Fortunella_bawangica_2** **aaaatgaattcctgcttccatcatctcttccaaatggatgttccaataccttcttgccat**

**Fortunella_hindsii_1** **aaaatgaattcctgcttccatcatctcttccaaatggatgttccaataccttcttgccat**

**Fortunella_hindsii_2** **aaaatgaattcctgcttccatcatctcttccaaatggatgttccaataccttcttgccat**

**Fortunella_hindsii_3** **aaaatgaattcctgcttccatcatctcttccaaatggatgttccaataccttcttgccat**

**Fortunella_japonica_1** **aaaatgaattcctgcttccatcatctcttccaaatggatgttccaataccttcttgccat**

**Fortunella_japonica_2** **aaaatgaattcctgcttccatcatctcttccaaatggatgttccaataccttcttgccat**

**Fortunella_japonica_3** **aaaatgaattcctgcttccatcatctcttccaaatggatgttccaataccttcttgccat**

**Fortunella_japonica_4** **aaaatgaattcctgcttccatcatctcttccaaatggatgttccaataccttcttgccat**

**Fortunella_japonica_5** **aaaatgaattcctgcttccatcatctcttccaaatggatgttccaataccttcttgccat**

**Fortunella_japonica_6** **aaaatgaattcctgcttccatcatctcttccaaatggatgttccaataccttcttgccat**

**Fortunella_japonica_7** **aaaatgaattcctgcttccatcatctcttccaaatggatgttccaataccttcttgccat**

**Fortunella_japonica_8** **aaaatgaattcctgcttccatcatctcttccaaatggatgttccaataccttcttgccat**

**Fortunella_japonica_9** **aaaatgaattcctgcttccatcatctcttccaaatggatgttccaataccttcttgccat**

**Fortunella_japonica_10** **aaaatgaattcctgcttccatcatctcttccaaatggatgttccaataccttcttgccat**

**Fortunella_japonica_11** **aaaatgaattcctgcttccatcatctcttccaaatggatgttccaataccttcttgccat**

**Fortunella_japonica_12** **aaaatgaattcctgcttccatcatctcttccaaatggatgttccaataccttcttgccat**

**Fortunella_margarita_1** **aaaatgaattcctgcttccatcatctcttccaaatggatgttccaataccttcttgccat**

**Fortunella_margarita_2** **aaaatgaattcctgcttccatcatctcttccaaatggatgttccaataccttcttgccat**

**Fortunella_venosa_1** **aaaatgaattcctgcttccatcatctcttccaaatggatgttccaataccttcttgccat**

**Fortunella_venosa_2** **aaaatgaattcctgcttccatcatctcttccaaatggatgttccaataccttcttgccat**

**Fortunella_venosa_3** **aaaatgaattcctgcttccatcatctcttccaaatggatgttccaataccttcttgccat**

18430	18440	18450	18460	18470	18480

....|....|....|....|....|....|....|....|....|....|....|....|

**Atalantia_kwangtungens** **ttctcccccacttcctc--tt---ttttttttaagagacgaggcgccctgaaataaataa**

**Citrus_reticulata_1** **ttctcccccacttcctc--tt---ttttttttaagagacgaggcgccctgaaataaataa**

**Citrus_reticulata_2** **ttctcccccacttcctc--tt---ttttttttaagagacgaggcgccctgaaataaataa**

**Citrus_trifoliata_3** **ttctcccccacttcctc--tt---ttttttttaagagacgaggcgtcctgaaataaataa**

**Citrus_aurantiifolia** **ttctcccccacttcctc--ttt--ttttttttaagagacgaggcgccctgaaataaataa**

**Citrus_aurantium** **ttctcccccacttc--------------ttttaagagacgaggcgccctgaaataaataa**

**Citrus_cavaleriei** **ttctcccccacttcctc--tt----tttttttaagagacgaggcgccctgaaataaataa**

**Citrus_hongheensis** **ttctcccccacttcctc--tt---ttttttttaagagacgaggcgccctgaaataaataa**

**Citrus_limon** **ttctcccccacttc--------------ttttaagagacgaggcgccctgaaataaataa**

**Citrus_maxima** **ttctcccccacttcctc--tt---ttttttttaagagacgaggcgccctgaaataaataa**

**Citrus_medica** **ttctcccccacttcctctttttttttttttttaagagacgaggcgccctgaaataaataa**

**Citrus_polytrifolia** **ttctcccccacttcctc--tt---ttttttttaagagacgaggcgccctgaaataaataa**

**Citrus_reticulata** **ttctcccccacttcctc--tt---ttttttttaagagacgaggcgccctgaaataaataa**

**Citrus_sinensis** **ttctcccccacttcctc--tt---ttttttttaagagacgaggcgccctgaaataaataa**

**Fortunella_bawangica_1** **ttctcccccacttcctcttttt-tttttttttaagagacgaggcgccctgaaataaataa**

**Fortunella_bawangica_2** **ttctcccccacttcctcttttt-tttttttttaagagacgaggcgccctgaaataaataa**

**Fortunella_hindsii_1** **ttctcccccacttcctc--tttttttttttttaagagacgaggcgccctgaaataaataa**

**Fortunella_hindsii_2** **ttctcccccacttcctc--tttttttttttttaagagacgaggcgccctgaaataaataa**

**Fortunella_hindsii_3** **ttctcccccacttcctc--tttttttttttttaagagacgaggcgccctgaaataaataa**

**Fortunella_japonica_1** **ttctcccccacttcctc--ttt-tttttttttaagagacgaggcgccctgaaataaataa**

**Fortunella_japonica_2** **ttctcccccacttcctc--tttttttttttttaagagacgaggcgccctgaaataaataa**

**Fortunella_japonica_3** **ttctcccccacttcctc--ttt-tttttttttaagagacgaggcgccctgaaataaataa**

**Fortunella_japonica_4** **ttctcccccacttcctc--ttt-tttttttttaagagacgaggcgccctgaaataaataa**

**Fortunella_japonica_5** **ttctcccccacttcctc--ttt-tttttttttaagagacgaggcgccctgaaataaataa**

**Fortunella_japonica_6** **ttctcccccacttcctc--ttt-tttttttttaagagacgaggcgccctgaaataaataa**

**Fortunella_japonica_7** **ttctcccccacttcctc--ttt-tttttttttaagagacgaggcgccctgaaataaataa**

**Fortunella_japonica_8** **ttctcccccacttcctc--ttt-tttttttttaagagacgaggcgccctgaaataaataa**

**Fortunella_japonica_9** **ttctcccccacttcctc--ttt-tttttttttaagagacgaggcgccctgaaataaataa**

**Fortunella_japonica_10** **ttctcccccacttcctctttttttttttttttaagagacgaggcgccctgaaataaataa**

**Fortunella_japonica_11** **ttctcccccacttcctc--ttt-tttttttttaagagacgaggcgccctgaaataaataa**

**Fortunella_japonica_12** **ttctcccccacttcctc--ttt-tttttttttaagagacgaggcgccctgaaataaataa**

**Fortunella_margarita_1** **ttctcccccacttcctctttttttttttttttaagagacgaggcgccctgaaataaataa**

**Fortunella_margarita_2** **ttctcccccacttcctctttttttttttttttaagagacgaggcgccctgaaataaataa**

**Fortunella_venosa_1** **ttctcccccacttcctc-ttttttttttttttaagagacgaggcgccctgaaataaataa**

**Fortunella_venosa_2** **ttctcccccacttcctc--tttttttttttttaagagacgaggcgccctgaaataaataa**

**Fortunella_venosa_3** **ttctcccccacttcctctttttttttttttttaagagacgaggcgccctgaaataaataa**

18490	18500	18510	18520	18530	18540

....|....|....|....|....|....|....|....|....|....|....|....|

**Atalantia_kwangtungens** **ttgttccgaaggaaccttctcttctaccagagagtgaccattgatacacgacccaggcca**

**Citrus_reticulata_1** **ttgttccgagggaaccttctcttctaccagagagtgaccattgatacacgacccaggcca**

**Citrus_reticulata_2** **ttgttccgagggaaccttctcttctaccagagagtgaccattgatacacgacccaggcca**

**Citrus_trifoliata_3** **ttgttccgagggaaccttctcttctaccagagagtgaccattgatacacgacccaggcca**

**Citrus_aurantiifolia** **ttgttccgagggaaccttctcttctaccagagagtgaccattgatacacgacccaggcca**

**Citrus_aurantium** **ttgttccgagggaaccttctcttctaccagagagtgaccattgatacacgacccaggcca**

**Citrus_cavaleriei** **ttgttccgagggaaccttctcttctaccagagagtgaccattgatacacgacccaggcca**

**Citrus_hongheensis** **ttgttccgagggaaccttctcttctaccagagagtgaccattgatacacgacccaggcca**

**Citrus_limon** **ttgttccgagggaaccttctcttctaccagagagtgaccattgatacacgacccaggcca**

**Citrus_maxima** **ttgttccgagggaaccttctcttctaccagagagtgaccattgatacacgacccaggcca**

**Citrus_medica** **ttgttccgagggaaccttctcttctaccaaagagtgaccattgatacacgacccaggcca**

**Citrus_polytrifolia** **ttgttccgagggaaccttctcttctaccagagagtgaccattgatacacgacccaggcca**

**Citrus_reticulata** **ttgttccgagggaaccttctcttctaccagagagtgaccattgatacacgacccaggcca**

**Citrus_sinensis** **ttgttccgagggaaccttctcttctaccagagagtgaccattgatacacgacccaggcca**

**Fortunella_bawangica_1** **ttgttccgagggaaccttctcttctaccagagagtgaccattgatacacgacccaggcca**

**Fortunella_bawangica_2** **ttgttccgagggaaccttctcttctaccagagagtgaccattgatacacgacccaggcca**

**Fortunella_hindsii_1** **ttgttccgagggaaccttctcttctaccagagagtgaccattgatacacgacccaggcca**

**Fortunella_hindsii_2** **ttgttccgagggaaccttctcttctaccagagagtgaccattgatacacgacccaggcca**

**Fortunella_hindsii_3** **ttgttccgagggaaccttctcttctaccagagagtgaccattgatacacgacccaggcca**

**Fortunella_japonica_1** **ttgttccgagggaaccttctcttctaccagagagtgaccattgatacacgacccaggcca**

**Fortunella_japonica_2** **ttgttccgagggaaccttctcttctaccagagagtgaccattgatacacgacccaggcca**

**Fortunella_japonica_3** **ttgttccgagggaaccttctcttctaccagagagtgaccattgatacacgacccaggcca**

**Fortunella_japonica_4** **ttgttccgagggaaccttctcttctaccagagagtgaccattgatacacgacccaggcca**

**Fortunella_japonica_5** **ttgttccgagggaaccttctcttctaccagagagtgaccattgatacacgacccaggcca**

**Fortunella_japonica_6** **ttgttccgagggaaccttctcttctaccagagagtgaccattgatacacgacccaggcca**

**Fortunella_japonica_7** **ttgttccgagggaaccttctcttctaccagagagtgaccattgatacacgacccaggcca**

**Fortunella_japonica_8** **ttgttccgagggaaccttctcttctaccagagagtgaccattgatacacgacccaggcca**

**Fortunella_japonica_9** **ttgttccgagggaaccttctcttctaccagagagtgaccattgatacacgacccaggcca**

**Fortunella_japonica_10** **ttgttccgagggaaccttctcttctaccagagagtgaccattgatacacgacccaggcca**

**Fortunella_japonica_11** **ttgttccgagggaaccttctcttctaccagagagtgaccattgatacacgacccaggcca**

**Fortunella_japonica_12** **ttgttccgagggaaccttctcttctaccagagagtgaccattgatacacgacccaggcca**

**Fortunella_margarita_1** **ttgttccgagggaaccttctcttctaccagagagtgaccattgatacacgacccaggcca**

**Fortunella_margarita_2** **ttgttccgagggaaccttctcttctaccagagagtgaccattgatacacgacccaggcca**

**Fortunella_venosa_1** **ttgttccgagggaaccttctcttctaccagagagtgaccattgatacacgacccaggcca**

**Fortunella_venosa_2** **ttgttccgagggaaccttctcttctaccagagagtgaccattgatacacgacccaggcca**

**Fortunella_venosa_3** **ttgttccgagggaaccttctcttctaccagagagtgaccattgatacacgacccaggcca**

18550	18560	18570	18580	18590	18600

....|....|....|....|....|....|....|....|....|....|....|....|

**Atalantia_kwangtungens** **ttcattactttctattcattattatccttcgttctattcattattatctttcttttctta**

**Citrus_reticulata_1** **ttcattactttctattcattattatccttcgttctattcattattatctttcttttcgta**

**Citrus_reticulata_2** **ttcattactttctattcattattatccttcgttctattcattattatctttcttttcgta**

**Citrus_trifoliata_3** **ttcattacttcctattcattattatccttcgttctattcattattatctttcttttcgta**

**Citrus_aurantiifolia** **ttcattactttctattcattattatccttcgttctattcattattatctttcttttcgta**

**Citrus_aurantium** **ttcattactttctattcattattatccttcgttctattcattattatctttcttttcgta**

**Citrus_cavaleriei** **ttcattact----------------------ttctattcattattatctttcttttcgta**

**Citrus_hongheensis** **ttcattactttctattcattattatccttcgttctattcattattatctttcttttcgta**

**Citrus_limon** **ttcattactttctattcattattatccttcgttctattcattattatctttcttttcgta**

**Citrus_maxima** **ttcattactttctattcattattatccttcgttctattcattattatctttcttttcgta**

**Citrus_medica** **ttcattactttctattcattattatccttcgttctattcattattatctttcttttcgta**

**Citrus_polytrifolia** **ttcattacttcctattcattattatccttcgttctattcattattatctttcttttcgta**

**Citrus_reticulata** **ttcattactttctattcattattatccttcgttctattcattattatctttcttttcgta**

**Citrus_sinensis** **ttcattactttctattcattattatccttcgttctattcattattatctttcttttcgta**

**Fortunella_bawangica_1** **ttcattactttctattcattattatccttcgttctattcattattatctttcttttcgta**

**Fortunella_bawangica_2** **ttcattactttctattcattattatccttcgttctattcattattatctttcttttcgta**

**Fortunella_hindsii_1** **ttcattactttctattcattattatccttcgttctattcattattatctttcttttcgta**

**Fortunella_hindsii_2** **ttcattactttctattcattattatccttcgttctattcattattatctttcttttcgta**

**Fortunella_hindsii_3** **ttcattactttctattcattattatccttcgttctattcattattatctttcttttcgta**

**Fortunella_japonica_1** **ttcattactttctattcattattatccttcgttctattcattattatctttcttttcgta**

**Fortunella_japonica_2** **ttcattactttctattcattattatccttcgttctattcattattatctttcttttcgta**

**Fortunella_japonica_3** **ttcattactttctattcattattatccttcgttctattcattattatctttcttttcgta**

**Fortunella_japonica_4** **ttcattactttctattcattattatccttcgttctattcattattatctttcttttcgta**

**Fortunella_japonica_5** **ttcattactttctattcattattatccttcgttctattcattattatctttcttttcgta**

**Fortunella_japonica_6** **ttcattactttctattcattattatccttcgttctattcattattatctttcttttcgta**

**Fortunella_japonica_7** **ttcattactttctattcattattatccttcgttctattcattattatctttcttttcgta**

**Fortunella_japonica_8** **ttcattactttctattcattattatccttcgttctattcattattatctttcttttcgta**

**Fortunella_japonica_9** **ttcattactttctattcattattatccttcgttctattcattattatctttcttttcgta**

**Fortunella_japonica_10** **ttcattactttctattcattattatccttcgttctattcattattatctttcttttcgta**

**Fortunella_japonica_11** **ttcattactttctattcattattatccttcgttctattcattattatctttcttttcgta**

**Fortunella_japonica_12** **ttcattactttctattcattattatccttcgttctattcattattatctttcttttcgta**

**Fortunella_margarita_1** **ttcattactttctattcattattatccttcgttctattcattattatctttcttttcgta**

**Fortunella_margarita_2** **ttcattactttctattcattattatccttcgttctattcattattatctttcttttcgta**

**Fortunella_venosa_1** **ttcattactttctattcattattatccttcgttctattcattattatctttcttttcgta**

**Fortunella_venosa_2** **ttcattactttctattcattattatccttcgttctattcattattatctttcttttcgta**

**Fortunella_venosa_3** **ttcattactttctattcattattatccttcgttctattcattattatctttcttttcgta**

18610	18620	18630	18640	18650	18660

....|....|....|....|....|....|....|....|....|....|....|....|

**Atalantia_kwangtungens** **ccattaagaaatc------------aaatgatcacaaatagttaaaagaattcgctccta**

**Citrus_reticulata_1** **ccattaaaaaatc------------aaatgatcacaaatagttaaaagaattcgctccta**

**Citrus_reticulata_2** **ccattaaaaaatc------------aaatgatcacaaatagttaaaagaattcgctccta**

**Citrus_trifoliata_3** **ccattaaaaaatc------------aaatgatcacaaatagttaaaagaattcgctccta**

**Citrus_aurantiifolia** **ccattaaaaaatc------------aaatgatcacaaatagttaaaagaattcgctccta**

**Citrus_aurantium** **ccattaaaaaatcaaatgatcacaaaaatgatcacaaatagttaaaagaattcgctccta**

**Citrus_cavaleriei** **ccattaaaaaatc------------aaatgatcacaaatagttaaaagaattcgctccta**

**Citrus_hongheensis** **ccattaaaaaatc------------aaatgatcacaaatagttaaaagaattcgctccta**

**Citrus_limon** **ccattaaaaaatcaaatgatcacaaaaatgatcacaaatagttaaaagaattcgctccta**

**Citrus_maxima** **ccattaaaaaatc------------aaatgatcacaaatagttaaaagaattcgctccta**

**Citrus_medica** **ccattaaaaaatc------------aaatgatcacaaatagttaaaagaattcgctccta**

**Citrus_polytrifolia** **ccattaaaaaatc------------aaatgatcacaaatagttaaaagaattcgctccta**

**Citrus_reticulata** **ccattaaaaaatc------------aaatgatcacaaatagttaaaagaattcgctccta**

**Citrus_sinensis** **ccattaaaaaatc------------aaatgatcacaaatagttaaaagaattcgctccta**

**Fortunella_bawangica_1** **ccattaaaaaatc------------aaatgatcacaaatagttaaaagaattcgctccta**

**Fortunella_bawangica_2** **ccattaaaaaatc------------aaatgatcacaaatagttaaaagaattcgctccta**

**Fortunella_hindsii_1** **ccattaaaaaatc------------aaatgatcacaaatagttaaaagaattcgctccta**

**Fortunella_hindsii_2** **ccattaaaaaatc------------aaatgatcacaaatagttaaaagaattcgctccta**

**Fortunella_hindsii_3** **ccattaaaaaatc------------aaatgatcacaaatagttaaaagaattcgctccta**

**Fortunella_japonica_1** **ccattaaaaaatc------------aaatgatcacaaatagttaaaagaattcgctccta**

**Fortunella_japonica_2** **ccattaaaaaatc------------aaatgatcacaaatagttaaaagaattcgctccta**

**Fortunella_japonica_3** **ccattaaaaaatc------------aaatgatcacaaatagttaaaagaattcgctccta**

**Fortunella_japonica_4** **ccattaaaaaatc------------aaatgatcacaaatagttaaaagaattcgctccta**

**Fortunella_japonica_5** **ccattaaaaaatc------------aaatgatcacaaatagttaaaagaattcgctccta**

**Fortunella_japonica_6** **ccattaaaaaatc------------aaatgatcacaaatagttaaaagaattcgctccta**

**Fortunella_japonica_7** **ccattaaaaaatc------------aaatgatcacaaatagttaaaagaattcgctccta**

**Fortunella_japonica_8** **ccattaaaaaatc------------aaatgatcacaaatagttaaaagaattcgctccta**

**Fortunella_japonica_9** **ccattaaaaaatc------------aaatgatcacaaatagttaaaagaattcgctccta**

**Fortunella_japonica_10** **ccattaaaaaatc------------aaatgatcacaaatagttaaaagaattcgctccta**

**Fortunella_japonica_11** **ccattaaaaaatc------------aaatgatcacaaatagttaaaagaattcgctccta**

**Fortunella_japonica_12** **ccattaaaaaatc------------aaatgatcacaaatagttaaaagaattcgctccta**

**Fortunella_margarita_1** **ccattaaaaaatc------------aaatgatcacaaatagttaaaagaattcgctccta**

**Fortunella_margarita_2** **ccattaaaaaatc------------aaatgatcacaaatagttaaaagaattcgctccta**

**Fortunella_venosa_1** **ccattaaaaaatc------------aaatgatcacaaatagttaaaagaattcgctccta**

**Fortunella_venosa_2** **ccattaaaaaatc------------aaatgatcacaaatagttaaaagaattcgctccta**

**Fortunella_venosa_3** **ccattaaaaaatc------------aaatgatcacaaatagttaaaagaattcgctccta**

18670	18680	18690	18700	18710	18720

....|....|....|....|....|....|....|....|....|....|....|....|

**Atalantia_kwangtungens** **ggactcattaaaccctctaagcaattgtgctctgatgcatcatg------gaaagtcgtt**

**Citrus_reticulata_1** **ggactcattaaaccctctaagcaattgtgctctgatgtatcatg------gaaagttgtt**

**Citrus_reticulata_2** **ggactcattaaaccctctaagcaattgtgctctgatgtatcatg------gaaagttgtt**

**Citrus_trifoliata_3** **ggactcattaaaccctctaagcaattgtgctctgatgtatcatg------gaaagtcgtt**

**Citrus_aurantiifolia** **ggactcattaaaccctctaagcaattgtgctctgatgtatcatg------gaaagtcgtt**

**Citrus_aurantium** **ggactcattaaaccctctaagcaattgtgctctgatgtatcatg------gaaagtcgtt**

**Citrus_cavaleriei** **ggactcattaaaccctctaagcaattgtgctctgatgtatcatg------gaaagttgtt**

**Citrus_hongheensis** **ggactcattaaaccctctaagcaattgtgctctgatgtatcatg------gaaagtcgtt**

**Citrus_limon** **ggactcattaaaccctctaagcaattgtgctctgatgtatcatg------gaaagtcgtt**

**Citrus_maxima** **ggactcattaaaccctctaagcaattgtgctctgatgtatcatg------gaaagtcgtt**

**Citrus_medica** **ggactcattaaaccctctaagcaattgtgctctgatgtatcatggaaagtgaaagtcgtt**

**Citrus_polytrifolia** **ggactcattaaaccctctaagcaattgtgctctgatgtatcatg------gaaagtcgtt**

**Citrus_reticulata** **ggactcattaaaccctctaagcaattgtgctctgatgtatcatg------gaaagttgtt**

**Citrus_sinensis** **ggactcattaaaccctctaagcaattgtgctctgatgtatcatg------gaaagtcgtt**

**Fortunella_bawangica_1** **ggactcattaaaccctctaagcaattgtgctctgatgtatcatg------gaaagtcgtt**

**Fortunella_bawangica_2** **ggactcattaaaccctctaagcaattgtgctctgatgtatcatg------gaaagtcgtt**

**Fortunella_hindsii_1** **ggactcattaaaccctctaagcaattgtgctctgatgtatcatg------gaaagtcgtt**

**Fortunella_hindsii_2** **ggactcattaaaccctctaagcaattgtgctctgatgtatcatg------gaaagtcgtt**

**Fortunella_hindsii_3** **ggactcattaaaccctctaagcaattgtgctctgatgtatcatg------gaaagtcgtt**

**Fortunella_japonica_1** **ggactcattaaaccctctaagcaattgtgctctgatgtatcatg------gaaagtcgtt**

**Fortunella_japonica_2** **ggactcattaaaccctctaagcaattgtgctctgatgtatcatg------gaaagtcgtt**

**Fortunella_japonica_3** **ggactcattaaaccctctaagcaattgtgctctgatgtatcatg------gaaagtcgtt**

**Fortunella_japonica_4** **ggactcattaaaccctctaagcaattgtgctctgatgtatcatg------gaaagtcgtt**

**Fortunella_japonica_5** **ggactcattaaaccctctaagcaattgtgctctgatgtatcatg------gaaagtcgtt**

**Fortunella_japonica_6** **ggactcattaaaccctctaagcaattgtgctctgatgtatcatg------gaaagtcgtt**

**Fortunella_japonica_7** **ggactcattaaaccctctaagcaattgtgctctgatgtatcatg------gaaagtcgtt**

**Fortunella_japonica_8** **ggactcattaaaccctctaagcaattgtgctctgatgtatcatg------gaaagtcgtt**

**Fortunella_japonica_9** **ggactcattaaaccctctaagcaattgtgctctgatgtatcatg------gaaagtcgtt**

**Fortunella_japonica_10** **ggactcattaaaccctctaagcaattgtgctctgatgtatcatg------gaaagtcgtt**

**Fortunella_japonica_11** **ggactcattaaaccctctaagcaattgtgctctgatgtatcatg------gaaagtcgtt**

**Fortunella_japonica_12** **ggactcattaaaccctctaagcaattgtgctctgatgtatcatg------gaaagtcgtt**

**Fortunella_margarita_1** **ggactcattaaaccctctaagcaattgtgctctgatgtatcatg------gaaagtcgtt**

**Fortunella_margarita_2** **ggactcattaaaccctctaagcaattgtgctctgatgtatcatg------gaaagtcgtt**

**Fortunella_venosa_1** **ggactcattaaaccctctaagcaattgtgctctgatgtatcatg------gaaagtcgtt**

**Fortunella_venosa_2** **ggactcattaaaccctctaagcaattgtgctctgatgtatcatg------gaaagtcgtt**

**Fortunella_venosa_3** **ggactcattaaaccctctaagcaattgtgctctgatgtatcatg------gaaagtcgtt**

18730	18740	18750	18760	18770	18780

....|....|....|....|....|....|....|....|....|....|....|....|

**Atalantia_kwangtungens** **gaaatgcacgagtcaaataattctctgtggtgtaagaaaatatctctcatttcccccccg**

**Citrus_reticulata_1** **gaaatgcacgagtcaaataattctctgtggtgtaagaaaatatctctcatttcccccccg**

**Citrus_reticulata_2** **gaaatgcacgagtcaaataattctctgtggtgtaagaaaatatctctcatttcccccccg**

**Citrus_trifoliata_3** **gaaatgcacgagtcaaataattctctgtggtgtaagaaaatatctctcatttcccccccg**

**Citrus_aurantiifolia** **gaaatgcacgagtcaaataattctctgtggtgtaagaaaatatctctcatttcccccccg**

**Citrus_aurantium** **gaaatgcacgagtcaaataattctctgtggtgtaagaaaatatctctcatttcccccccg**

**Citrus_cavaleriei** **gaaatgcacgagtcaaataattctctgtggtgtaagaaaatatctctcatttcccccccg**

**Citrus_hongheensis** **gaaatgcacgagtcaaataattctctgtggtgtaagaaaatatctctcatttcccccccg**

**Citrus_limon** **gaaatgcacgagtcaaataattctctgtggtgtaagaaaatatctctcatttcccccccg**

**Citrus_maxima** **gaaatgcacgagtcaaataattctctgtggtgtaagaaaatatctctcatttcccccccg**

**Citrus_medica** **gaaatgcacgagtcaaataattctctgtggtgtaagaaaatatctctcatttcccccccg**

**Citrus_polytrifolia** **gaaatgcacgagtcaaataattctctgtggtgtaagaaaatatctctcatttcccccccg**

**Citrus_reticulata** **gaaatgcacgagtcaaataattctctgtggtgtaagaaaatatctctcatttcccccccg**

**Citrus_sinensis** **gaaatgcacgagtcaaataattctctgtggtgtaagaaaatatctctcatttcccccccg**

**Fortunella_bawangica_1** **gaaatgcacgagtcaaataattctctgtggtgtaagaaaatatctctcatttcccccccg**

**Fortunella_bawangica_2** **gaaatgcacgagtcaaataattctctgtggtgtaagaaaatatctctcatttcccccccg**

**Fortunella_hindsii_1** **gaaatgcacgagtcaaataattctctgtggtgtaagaaaatatctctcatttcccccccg**

**Fortunella_hindsii_2** **gaaatgcacgagtcaaataattctctgtggtgtaagaaaatatctctcatttcccccccg**

**Fortunella_hindsii_3** **gaaatgcacgagtcaaataattctctgtggtgtaagaaaatatctctcatttcccccccg**

**Fortunella_japonica_1** **gaaatgcacgagtcaaataattctctgtggtgtaagaaaatatctctcatttcccccccg**

**Fortunella_japonica_2** **gaaatgcacgagtcaaataattctctgtggtgtaagaaaatatctctcatttcccccccg**

**Fortunella_japonica_3** **gaaatgcacgagtcaaataattctctgtggtgtaagaaaatatctctcatttcccccccg**

**Fortunella_japonica_4** **gaaatgcacgagtcaaataattctctgtggtgtaagaaaatatctctcatttcccccccg**

**Fortunella_japonica_5** **gaaatgcacgagtcaaataattctctgtggtgtaagaaaatatctctcatttcccccccg**

**Fortunella_japonica_6** **gaaatgcacgagtcaaataattctctgtggtgtaagaaaatatctctcatttcccccccg**

**Fortunella_japonica_7** **gaaatgcacgagtcaaataattctctgtggtgtaagaaaatatctctcatttcccccccg**

**Fortunella_japonica_8** **gaaatgcacgagtcaaataattctctgtggtgtaagaaaatatctctcatttcccccccg**

**Fortunella_japonica_9** **gaaatgcacgagtcaaataattctctgtggtgtaagaaaatatctctcatttcccccccg**

**Fortunella_japonica_10** **gaaatgcacgagtcaaataattctctgtggtgtaagaaaatatctctcatttcccccccg**

**Fortunella_japonica_11** **gaaatgcacgagtcaaataattctctgtggtgtaagaaaatatctctcatttcccccccg**

**Fortunella_japonica_12** **gaaatgcacgagtcaaataattctctgtggtgtaagaaaatatctctcatttcccccccg**

**Fortunella_margarita_1** **gaaatgcacgagtcaaataattctctgtggtgtaagaaaatatctctcatttcccccccg**

**Fortunella_margarita_2** **gaaatgcacgagtcaaataattctctgtggtgtaagaaaatatctctcatttcccccccg**

**Fortunella_venosa_1** **gaaatgcacgagtcaaataattctctgtggtgtaagaaaatatctctcatttcccccccg**

**Fortunella_venosa_2** **gaaatgcacgagtcaaataattctctgtggtgtaagaaaatatctctcatttcccccccg**

**Fortunella_venosa_3** **gaaatgcacgagtcaaataattctctgtggtgtaagaaaatatctctcatttcccccccg**

18790	18800	18810	18820	18830	18840

....|....|....|....|....|....|....|....|....|....|....|....|

**Atalantia_kwangtungens** **aataaattccctttttgtctttccaaaagaatgttgttatgctgccttgaacagtggact**

**Citrus_reticulata_1** **aataaattccctttttgtctttccaaaagaatgttgttatgctgccttgaacagtggact**

**Citrus_reticulata_2** **aataaattccctttttgtctttccaaaagaatgttgttatgctgccttgaacagtggact**

**Citrus_trifoliata_3** **aataaattccctttttgtctttccaaaagaatgttgttatgctgccttgaacagtggact**

**Citrus_aurantiifolia** **aataaattccctttttgtctttccaaaagaatgttgttatgctgccttgaacagtggact**

**Citrus_aurantium** **aataaattccctttttgtctttccaaaagaatgttgttatgctgccttgaacagtggact**

**Citrus_cavaleriei** **aataaattccctttttggctttccaaaaaaatgttgttatgctgccttgaacagtggact**

**Citrus_hongheensis** **aataaattccctttttgtctttccaaaagaatgttgttatgctgccttgaacagtggact**

**Citrus_limon** **aataaattccctttttgtctttccaaaagaatgttgttatgctgccttgaacagtggact**

**Citrus_maxima** **aataaattccctttttgtctttccaaaagaatgttgttatgctgccttgaacagtggact**

**Citrus_medica** **aataaattccctttttttctttccaaaagaatgttgttatgctgccttgaacagtggact**

**Citrus_polytrifolia** **aataaattccctttttgtctttccaaaagaatgttgttatgctgccttgaacagtggact**

**Citrus_reticulata** **aataaattccctttttgtctttccaaaagaatgttgttatgctgccttgaacagtggact**

**Citrus_sinensis** **aataaattccctttttgtctttccaaaagaatgttgttatgctgccttgaacagtggact**

**Fortunella_bawangica_1** **aataaattccctttttgtctttccaaaagaatgttgttatgctgccttgaacagtggact**

**Fortunella_bawangica_2** **aataaattccctttttgtctttccaaaagaatgttgttatgctgccttgaacagtggact**

**Fortunella_hindsii_1** **aataaattccctttttgtctttccaaaagaatgttgttatgctgccttgaacagtggact**

**Fortunella_hindsii_2** **aataaattccctttttgtctttccaaaagaatgttgttatgctgccttgaacagtggact**

**Fortunella_hindsii_3** **aataaattccctttttgtctttccaaaagaatgttgttatgctgccttgaacagtggact**

**Fortunella_japonica_1** **aataaattccctttttgtctttccaaaagaatgttgttatgctgccttgaacagtggact**

**Fortunella_japonica_2** **aataaattccctttttgtctttccaaaagaatgttgttatgctgccttgaacagtggact**

**Fortunella_japonica_3** **aataaattccctttttgtctttccaaaagaatgttgttatgctgccttgaacagtggact**

**Fortunella_japonica_4** **aataaattccctttttgtctttccaaaagaatgttgttatgctgccttgaacagtggact**

**Fortunella_japonica_5** **aataaattccctttttgtctttccaaaagaatgttgttatgctgccttgaacagtggact**

**Fortunella_japonica_6** **aataaattccctttttgtctttccaaaagaatgttgttatgctgccttgaacagtggact**

**Fortunella_japonica_7** **aataaattccctttttgtctttccaaaagaatgttgttatgctgccttgaacagtggact**

**Fortunella_japonica_8** **aataaattccctttttgtctttccaaaagaatgttgttatgctgccttgaacagtggact**

**Fortunella_japonica_9** **aataaattccctttttgtctttccaaaagaatgttgttatgctgccttgaacagtggact**

**Fortunella_japonica_10** **aataaattccctttttgtctttccaaaagaatgttgttatgctgccttgaacagtggact**

**Fortunella_japonica_11** **aataaattccctttttgtctttccaaaagaatgttgttatgctgccttgaacagtggact**

**Fortunella_japonica_12** **aataaattccctttttgtctttccaaaagaatgttgttatgctgccttgaacagtggact**

**Fortunella_margarita_1** **aataaattccctttttgtctttccaaaagaatgttgttatgctgccttgaacagtggact**

**Fortunella_margarita_2** **aataaattccctttttgtctttccaaaagaatgttgttatgctgccttgaacagtggact**

**Fortunella_venosa_1** **aataaattccctttttgtctttccaaaagaatgttgttatgctgccttgaacagtggact**

**Fortunella_venosa_2** **aataaattccctttttgtctttccaaaagaatgttgttatgctgccttgaacagtggact**

**Fortunella_venosa_3** **aataaattccctttttgtctttccaaaagaatgttgttatgctgccttgaacagtggact**

18850	18860	18870	18880	18890	18900

....|....|....|....|....|....|....|....|....|....|....|....|

**Atalantia_kwangtungens** **aatcctttgaatccggtaccgactggtattatcccccccagaacaacgttttctttcagg**

**Citrus_reticulata_1** **aatcctttgaatccggtaccgactggtattatcccccccagaacaacgttttctttcagg**

**Citrus_reticulata_2** **aatcctttgaatccggtaccgactggtattatcccccccagaacaacgttttctttcagg**

**Citrus_trifoliata_3** **aatcctttgaatccggtaccgactggtattatcccccccagaacaacgttttctttcagg**

**Citrus_aurantiifolia** **aatcctttgaatccggtaccgactggtattatcccccccagaacaacgttttctttcagg**

**Citrus_aurantium** **aatcctttgaatccggtaccgactggtattatcccccccagaacaacgttttctttcagg**

**Citrus_cavaleriei** **aatcctttgaatccggtaccgactggtattatcccccccagaacaacgttttctttcagg**

**Citrus_hongheensis** **aatcctttgaatccggtaccgactggtattatcccccccagaacaacgttttctttcagg**

**Citrus_limon** **aatcctttgaatccggtaccgactggtattatcccccccagaacaacgttttctttcagg**

**Citrus_maxima** **aatcctttgaatccggtaccgactggtattatcccccccagaacaacgttttctttcagg**

**Citrus_medica** **aatcctttgaatccggtaccgactggtattatcccccccagaacaacgttttctttcagg**

**Citrus_polytrifolia** **aatcctttgaatccggtaccgactggtattatcccccccagaacaacgttttctttcagg**

**Citrus_reticulata** **aatcctttgaatccggtaccgactggtattatcccccccagaacaacgttttctttcagg**

**Citrus_sinensis** **aatcctttgaatccggtaccgactggtattatcccccccagaacaacgttttctttcagg**

**Fortunella_bawangica_1** **aatcctttgaatccggtaccgactggtattatcccccccagaacaacgttttctttcagg**

**Fortunella_bawangica_2** **aatcctttgaatccggtaccgactggtattatcccccccagaacaacgttttctttcagg**

**Fortunella_hindsii_1** **aatcctttgaatccggtaccgactggtattatcccccccagaacaacgttttctttcagg**

**Fortunella_hindsii_2** **aatcctttgaatccggtaccgactggtattatcccccccagaacaacgttttctttcagg**

**Fortunella_hindsii_3** **aatcctttgaatccggtaccgactggtattatcccccccagaacaacgttttctttcagg**

**Fortunella_japonica_1** **aatcctttgaatccggtaccgactggtattatcccccccagaacaacgttttctttcagg**

**Fortunella_japonica_2** **aatcctttgaatccggtaccgactggtattatcccccccagaacaacgttttctttcagg**

**Fortunella_japonica_3** **aatcctttgaatccggtaccgactggtattatcccccccagaacaacgttttctttcagg**

**Fortunella_japonica_4** **aatcctttgaatccggtaccgactggtattatcccccccagaacaacgttttctttcagg**

**Fortunella_japonica_5** **aatcctttgaatccggtaccgactggtattatcccccccagaacaacgttttctttcagg**

**Fortunella_japonica_6** **aatcctttgaatccggtaccgactggtattatcccccccagaacaacgttttctttcagg**

**Fortunella_japonica_7** **aatcctttgaatccggtaccgactggtattatcccccccagaacaacgttttctttcagg**

**Fortunella_japonica_8** **aatcctttgaatccggtaccgactggtattatcccccccagaacaacgttttctttcagg**

**Fortunella_japonica_9** **aatcctttgaatccggtaccgactggtattatcccccccagaacaacgttttctttcagg**

**Fortunella_japonica_10** **aatcctttgaatccggtaccgactggtattatcccccccagaacaacgttttctttcagg**

**Fortunella_japonica_11** **aatcctttgaatccggtaccgactggtattatcccccccagaacaacgttttctttcagg**

**Fortunella_japonica_12** **aatcctttgaatccggtaccgactggtattatcccccccagaacaacgttttctttcagg**

**Fortunella_margarita_1** **aatcctttgaatccggtaccgactggtattatcccccccagaacaacgttttctttcagg**

**Fortunella_margarita_2** **aatcctttgaatccggtaccgactggtattatcccccccagaacaacgttttctttcagg**

**Fortunella_venosa_1** **aatcctttgaatccggtaccgactggtattatcccccccagaacaacgttttctttcagg**

**Fortunella_venosa_2** **aatcctttgaatccggtaccgactggtattatcccccccagaacaacgttttctttcagg**

**Fortunella_venosa_3** **aatcctttgaatccggtaccgactggtattatcccccccagaacaacgttttctttcagg**

18910	18920	18930	18940	18950	18960

....|....|....|....|....|....|....|....|....|....|....|....|

**Atalantia_kwangtungens** **cctttcaaccaatcgatacgacctcggagagcagcttttgctaaaactcgcgtggtttct**

**Citrus_reticulata_1** **cctttcaaccaatcgatacgacctcggagagcagcttttgctaaaactcgcgtggtttct**

**Citrus_reticulata_2** **cctttcaaccaatcgatacgacctcggagagcagcttttgctaaaactcgcgtggtttct**

**Citrus_trifoliata_3** **cctttcaaccaatcgatacgacctcggagagcagcttttgctaaaacgcgcgtggtttct**

**Citrus_aurantiifolia** **cctttcaaccaatcgatacgacctcggagagcagcttttgctaaaactcgcgtggtttct**

**Citrus_aurantium** **cctttcaaccaatcgatacgacctcggagagcagcttttgctaaaactcgcgtggtttct**

**Citrus_cavaleriei** **cctttcaaccaatcgatacgacctcggagagcagcttttgctaaaactcgcgtggtttct**

**Citrus_hongheensis** **cctttcaaccaatcgatacgacctcggagagcagcttttgctaaaactcgcgtggtttct**

**Citrus_limon** **cctttcaaccaatcgatacgacctcggagagcagcttttgctaaaactcgcgtggtttct**

**Citrus_maxima** **cctttcaaccaatcgatacgacctcggagagcagcttttgctaaaactcgcgtggtttct**

**Citrus_medica** **cctttcaaccaatcgatacgacctcggagagcagcttttgctaaaactcgcgtggtttct**

**Citrus_polytrifolia** **cctttcaaccaatcgatacgacctcggagagcagcttttgctaaaacgcgcgtggtttct**

**Citrus_reticulata** **cctttcaaccaatcgatacgacctcggagagcagcttttgctaaaactcgcgtggtttct**

**Citrus_sinensis** **cctttcaaccaatcgatacgacctcggagagcagcttttgctaaaactcgcgtggtttct**

**Fortunella_bawangica_1** **cctttcaaccaatcgatacgacctcggagagcagcttttgctaaaactcgcgtggtttct**

**Fortunella_bawangica_2** **cctttcaaccaatcgatacgacctcggagagcagcttttgctaaaactcgcgtggtttct**

**Fortunella_hindsii_1** **cctttcaaccaatcgatacgacctcggagagcagcttttgctaaaactcgcgtggtttct**

**Fortunella_hindsii_2** **cctttcaaccaatcgatacgacctcggagagcagcttttgctaaaactcgcgtggtttct**

**Fortunella_hindsii_3** **cctttcaaccaatcgatacgacctcggagagcagcttttgctaaaactcgcgtggtttct**

**Fortunella_japonica_1** **cctttcaaccaatcgatacgacctcggagagcagcttttgctaaaactcgcgtggtttct**

**Fortunella_japonica_2** **cctttcaaccaatcgatacgacctcggagagcagcttttgctaaaactcgcgtggtttct**

**Fortunella_japonica_3** **cctttcaaccaatcgatacgacctcggagagcagcttttgctaaaactcgcgtggtttct**

**Fortunella_japonica_4** **cctttcaaccaatcgatacgacctcggagagcagcttttgctaaaactcgcgtggtttct**

**Fortunella_japonica_5** **cctttcaaccaatcgatacgacctcggagagcagcttttgctaaaactcgcgtggtttct**

**Fortunella_japonica_6** **cctttcaaccaatcgatacgacctcggagagcagcttttgctaaaactcgcgtggtttct**

**Fortunella_japonica_7** **cctttcaaccaatcgatacgacctcggagagcagcttttgctaaaactcgcgtggtttct**

**Fortunella_japonica_8** **cctttcaaccaatcgatacgacctcggagagcagcttttgctaaaactcgcgtggtttct**

**Fortunella_japonica_9** **cctttcaaccaatcgatacgacctcggagagcagcttttgctaaaactcgcgtggtttct**

**Fortunella_japonica_10** **cctttcaaccaatcgatacgacctcggagagcagcttttgctaaaactcgcgtggtttct**

**Fortunella_japonica_11** **cctttcaaccaatcgatacgacctcggagagcagcttttgctaaaactcgcgtggtttct**

**Fortunella_japonica_12** **cctttcaaccaatcgatacgacctcggagagcagcttttgctaaaactcgcgtggtttct**

**Fortunella_margarita_1** **cctttcaaccaatcgatacgacctcggagagcagcttttgctaaaactcgcgtggtttct**

**Fortunella_margarita_2** **cctttcaaccaatcgatacgacctcggagagcagcttttgctaaaactcgcgtggtttct**

**Fortunella_venosa_1** **cctttcaaccaatcgatacgacctcggagagcagcttttgctaaaactcgcgtggtttct**

**Fortunella_venosa_2** **cctttcaaccaatcgatacgacctcggagagcagcttttgctaaaactcgcgtggtttct**

**Fortunella_venosa_3** **cctttcaaccaatcgatacgacctcggagagcagcttttgctaaaactcgcgtggtttct**

18970	18980	18990	19000	19010	19020

....|....|....|....|....|....|....|....|....|....|....|....|

**Atalantia_kwangtungens** **tgaaaactcgcttcggatataaaactttgagtattcagagacgctctcgttattcccaat**

**Citrus_reticulata_1** **tgaaaacttgcttcggatataaaactttgagtattcagagacgctctcgttattcccaat**

**Citrus_reticulata_2** **tgaaaacttgcttcggatataaaactttgagtattcagagacgctctcgttattcccaat**

**Citrus_trifoliata_3** **tgaaaacttgcttcggatataaaactttgagtattcagagacgctctcgttattcccaat**

**Citrus_aurantiifolia** **tgaaaacttgcttcggatataaaactttgagtattcagagacgctctcgttattcccaat**

**Citrus_aurantium** **tgaaaacttgcttcggatataaaactttgagtattcagagacgctctcgttattcccaat**

**Citrus_cavaleriei** **tgaaaacttgcttcggatataaaactttgagtattcagagacgctctcgttattcccaat**

**Citrus_hongheensis** **tgaaaacttgcttcggatataaaactttgagtattcagagacgctctcgttattcccaat**

**Citrus_limon** **tgaaaacttgcttcggatataaaactttgagtattcagagacgctctcgttattcccaat**

**Citrus_maxima** **tgaaaacttgcttcggatataaaactttgagtattcagagacgctctcgttattcccaat**

**Citrus_medica** **tgaaaacttgcttcggatataaaactttgagtattcagagacgctctcgttattcccaat**

**Citrus_polytrifolia** **tgaaaacttgcttcggatataaaactttgagtattcagagacgctctcgttattcccaat**

**Citrus_reticulata** **tgaaaacttgcttcggatataaaactttgagtattcagagacgctctcgttattcccaat**

**Citrus_sinensis** **tgaaaacttgcttcggatataaaactttgagtattcagagacgctctcgttattcccaat**

**Fortunella_bawangica_1** **tgaaaacttgcttcggatataaaactttgagtattcagagacgctctagttattcccaat**

**Fortunella_bawangica_2** **tgaaaacttgcttcggatataaaactttgagtattcagagacgctctagttattcccaat**

**Fortunella_hindsii_1** **tgaaaacttgcttcggatataaaactttgagtattcagagacgctctagttattcccaat**

**Fortunella_hindsii_2** **tgaaaacttgcttcggatataaaactttgagtattcagagacgctctagttattcccaat**

**Fortunella_hindsii_3** **tgaaaacttgcttcggatataaaactttgagtattcagagacgctctagttattcccaat**

**Fortunella_japonica_1** **tgaaaacttgcttcggatataaaactttgagtattcagagacgctctagttattcccaat**

**Fortunella_japonica_2** **tgaaaacttgcttcggatataaaactttgagtattcagagacgctctagttattcccaat**

**Fortunella_japonica_3** **tgaaaacttgcttcggatataaaactttgagtattcagagacgctctagttattcccaat**

**Fortunella_japonica_4** **tgaaaacttgcttcggatataaaactttgagtattcagagacgctctagttattcccaat**

**Fortunella_japonica_5** **tgaaaacttgcttcggatataaaactttgagtattcagagacgctctagttattcccaat**

**Fortunella_japonica_6** **tgaaaacttgcttcggatataaaactttgagtattcagagacgctctagttattcccaat**

**Fortunella_japonica_7** **tgaaaacttgcttcggatataaaactttgagtattcagagacgctctagttattcccaat**

**Fortunella_japonica_8** **tgaaaacttgcttcggatataaaactttgagtattcagagacgctctagttattcccaat**

**Fortunella_japonica_9** **tgaaaacttgcttcggatataaaactttgagtattcagagacgctctagttattcccaat**

**Fortunella_japonica_10** **tgaaaacttgcttcggatataaaactttgagtattcagagacgctctagttattcccaat**

**Fortunella_japonica_11** **tgaaaacttgcttcggatataaaactttgagtattcagagacgctctagttattcccaat**

**Fortunella_japonica_12** **tgaaaacttgcttcggatataaaactttgagtattcagagacgctctagttattcccaat**

**Fortunella_margarita_1** **tgaaaacttgcttcggatataaaactttgagtattcagagacgctctagttattcccaat**

**Fortunella_margarita_2** **tgaaaacttgcttcggatataaaactttgagtattcagagacgctctagttattcccaat**

**Fortunella_venosa_1** **tgaaaacttgcttcggatataaaactttgagtattcagagacgctctagttattcccaat**

**Fortunella_venosa_2** **tgaaaacttgcttcggatataaaactttgagtattcagagacgctctagttattcccaat**

**Fortunella_venosa_3** **tgaaaacttgcttcggatataaaactttgagtattcagagacgctctagttattcccaat**

19030	19040	19050	19060	19070	19080

....|....|....|....|....|....|....|....|....|....|....|....|

**Atalantia_kwangtungens** **aagatagctcgataacagatcacttcttccaaagcgcgccccattcgttccgctcgtaac**

**Citrus_reticulata_1** **aagctagctcgataacagatcacttcttccaaagcgcgccccattcgttccgctcgtaac**

**Citrus_reticulata_2** **aagctagctcgataacagatcacttcttccaaagcgcgccccattcgttccgctcgtaac**

**Citrus_trifoliata_3** **aagatagctcgataacagatcacttcttccaaagcgcgccccattcgttccgctcgtaac**

**Citrus_aurantiifolia** **aagatagctcgataacagatcacttcttccaaagcgcgccccattcgttccgctcgtaac**

**Citrus_aurantium** **aagatagctcgataacagatcacttcttccaaagcgcgccccattcgttccgctcgtaac**

**Citrus_cavaleriei** **aagatagctcgataacagatcacttcttccaaagcgcgccccattcgttccgctcgtaac**

**Citrus_hongheensis** **aagatagctcgataacagatcacttcttccaaagcgcgccccattcgttccgctcgtaac**

**Citrus_limon** **aagatagctcgataacagatcacttcttccaaagcgcgccccattcgttccgctcgtaac**

**Citrus_maxima** **aagatagctcgataacagatcacttcttccaaagcgcgccccattcgttccgctcgtaac**

**Citrus_medica** **aagatagctcgataacagatcacttcttccaaagcgcgccccattcgttccgctcgtaac**

**Citrus_polytrifolia** **aagatagctcgataacagatcacttcttccaaagcgcgccccattcgttccgctcgtaac**

**Citrus_reticulata** **aagctagctcgataacagatcacttcttccaaagcgcgccccattcgttccgctcgtaac**

**Citrus_sinensis** **aagatagctcgataacagatcacttcttccaaagcgcgccccattcgttccgctcgtaac**

**Fortunella_bawangica_1** **aagatagctcgataacagatcacttcttccaaagcgcgccccattcgttccgctcgtaac**

**Fortunella_bawangica_2** **aagatagctcgataacagatcacttcttccaaagcgcgccccattcgttccgctcgtaac**

**Fortunella_hindsii_1** **aagatagctcgataacagatcacttcttccaaagcgcgccccattcgttccgctcgtaac**

**Fortunella_hindsii_2** **aagatagctcgataacagatcacttcttccaaagcgcgccccattcgttccgctcgtaac**

**Fortunella_hindsii_3** **aagatagctcgataacagatcacttcttccaaagcgcgccccattcgttccgctcgtaac**

**Fortunella_japonica_1** **aagatagctcgataacagatcacttcttccaaagcgcgccccattcgttccgctcgtaac**

**Fortunella_japonica_2** **aagatagctcgataacagatcacttcttccaaagcgcgccccattcgttccgctcgtaac**

**Fortunella_japonica_3** **aagatagctcgataacagatcacttcttccaaagcgcgccccattcgttccgctcgtaac**

**Fortunella_japonica_4** **aagatagctcgataacagatcacttcttccaaagcgcgccccattcgttccgctcgtaac**

**Fortunella_japonica_5** **aagatagctcgataacagatcacttcttccaaagcgcgccccattcgttccgctcgtaac**

**Fortunella_japonica_6** **aagatagctcgataacagatcacttcttccaaagcgcgccccattcgttccgctcgtaac**

**Fortunella_japonica_7** **aagatagctcgataacagatcacttcttccaaagcgcgccccattcgttccgctcgtaac**

**Fortunella_japonica_8** **aagatagctcgataacagatcacttcttccaaagcgcgccccattcgttccgctcgtaac**

**Fortunella_japonica_9** **aagatagctcgataacagatcacttcttccaaagcgcgccccattcgttccgctcgtaac**

**Fortunella_japonica_10** **aagatagctcgataacagatcacttcttccaaagcgcgccccattcgttccgctcgtaac**

**Fortunella_japonica_11** **aagatagctcgataacagatcacttcttccaaagcgcgccccattcgttccgctcgtaac**

**Fortunella_japonica_12** **aagatagctcgataacagatcacttcttccaaagcgcgccccattcgttccgctcgtaac**

**Fortunella_margarita_1** **aagatagctcgataacagatcacttcttccaaagcgcgccccattcgttccgctcgtaac**

**Fortunella_margarita_2** **aagatagctcgataacagatcacttcttccaaagcgcgccccattcgttccgctcgtaac**

**Fortunella_venosa_1** **aagatagctcgataacagatcacttcttccaaagcgcgccccattcgttccgctcgtaac**

**Fortunella_venosa_2** **aagatagctcgataacagatcacttcttccaaagcgcgccccattcgttccgctcgtaac**

**Fortunella_venosa_3** **aagatagctcgataacagatcacttcttccaaagcgcgccccattcgttccgctcgtaac**

19090	19100	19110	19120	19130	19140

....|....|....|....|....|....|....|....|....|....|....|....|

**Atalantia_kwangtungens** **aatccaatcagttcgccgggtaaaaaaatattagacattccatcttcggaaactaaaact**

**Citrus_reticulata_1** **aatccaatcagttcgccgggtaaaaaaatattagacattccatcttcggaaactaaaact**

**Citrus_reticulata_2** **aatccaatcagttcgccgggtaaaaaaatattagacattccatcttcggaaactaaaact**

**Citrus_trifoliata_3** **aatccaataagttcgccgggtaaaaaaatattagacattccatcttcggaaactaaaact**

**Citrus_aurantiifolia** **aatccaatcagttcgccgggtaaaaaaatattagacattccatcttcggaaactaaaact**

**Citrus_aurantium** **aatccaatcagttcgccgggtaaaaaaatattagacattccatcttcggaaactaaaact**

**Citrus_cavaleriei** **aatccaatcagttcgccgggtaaaaaaatattagacattccatcttcggaaactaaaact**

**Citrus_hongheensis** **aatccaatcagttcgccgggtaaaaaaagattagacattccatcttcggaaactaaaact**

**Citrus_limon** **aatccaatcagttcgccgggtaaaaaaatattagacattccatcttcggaaactaaaact**

**Citrus_maxima** **aatccaatcagttcgccgggtaaaaaaatattagacattccatcttcggaaactaaaact**

**Citrus_medica** **aatccaatcagttcgccgggtaaaaaaatattagacattccatcttcggaaacgaaaact**

**Citrus_polytrifolia** **aatccaataagttcgccgggtaaaaaaatattagacattccatcttcggaaactaaaact**

**Citrus_reticulata** **aatccaatcagttcgccgggtaaaaaaatattagacattccatcttcggaaactaaaact**

**Citrus_sinensis** **aatccaatcagttcgccgggtaaaaaaatattagacattccatcttcggaaactaaaact**

**Fortunella_bawangica_1** **aatccaatcagttcgccgggtaaaaaaatattagacattccatcttcggaaactaaaact**

**Fortunella_bawangica_2** **aatccaatcagttcgccgggtaaaaaaatattagacattccatcttcggaaactaaaact**

**Fortunella_hindsii_1** **aatccaatcagttcgccgggtaaaaaaatattagacattccatcttcggaaactaaaact**

**Fortunella_hindsii_2** **aatccaatcagttcgccgggtaaaaaaatattagacattccatcttcggaaactaaaact**

**Fortunella_hindsii_3** **aatccaatcagttcgccgggtaaaaaaatattagacattccatcttcggaaactaaaact**

**Fortunella_japonica_1** **aatccaatcagttcgccgggtaaaaaaatattagacattccatcttcggaaactaaaact**

**Fortunella_japonica_2** **aatccaatcagttcgccgggtaaaaaaatattagacattccatcttcggaaactaaaact**

**Fortunella_japonica_3** **aatccaatcagttcgccgggtaaaaaaatattagacattccatcttcggaaactaaaact**

**Fortunella_japonica_4** **aatccaatcagttcgccgggtaaaaaaatattagacattccatcttcggaaactaaaact**

**Fortunella_japonica_5** **aatccaatcagttcgccgggtaaaaaaatattagacattccatcttcggaaactaaaact**

**Fortunella_japonica_6** **aatccaatcagttcgccgggtaaaaaaatattagacattccatcttcggaaactaaaact**

**Fortunella_japonica_7** **aatccaatcagttcgccgggtaaaaaaatattagacattccatcttcggaaactaaaact**

**Fortunella_japonica_8** **aatccaatcagttcgccgggtaaaaaaatattagacattccatcttcggaaactaaaact**

**Fortunella_japonica_9** **aatccaatcagttcgccgggtaaaaaaatattagacattccatcttcggaaactaaaact**

**Fortunella_japonica_10** **aatccaatcagttcgccgggtaaaaaaatattagacattccatcttcggaaactaaaact**

**Fortunella_japonica_11** **aatccaatcagttcgccgggtaaaaaaatattagacattccatcttcggaaactaaaact**

**Fortunella_japonica_12** **aatccaatcagttcgccgggtaaaaaaatattagacattccatcttcggaaactaaaact**

**Fortunella_margarita_1** **aatccaatcagttcgccgggtaaaaaaatattagacattccatcttcggaaactaaaact**

**Fortunella_margarita_2** **aatccaatcagttcgccgggtaaaaaaatattagacattccatcttcggaaactaaaact**

**Fortunella_venosa_1** **aatccaatcagttcgccgggtaaaaaaatattagacattccatcttcggaaactaaaact**

**Fortunella_venosa_2** **aatccaatcagttcgccgggtaaaaaaatattagacattccatcttcggaaactaaaact**

**Fortunella_venosa_3** **aatccaatcagttcgccgggtaaaaaaatattagacattccatcttcggaaactaaaact**

19150	19160	19170	19180	19190	19200

....|....|....|....|....|....|....|....|....|....|....|....|

**Atalantia_kwangtungens** **tttgatgttatttgacgtacaataatttctatgtgcctattatggatctgcaccccctgc**

**Citrus_reticulata_1** **tttgatgttatttgacgtacaataatttctatatgcctattatggatctgcaccccctgc**

**Citrus_reticulata_2** **tttgatgttatttgacgtacaataatttctatatgcctattatggatctgcaccccctgc**

**Citrus_trifoliata_3** **tttgatgttatttgacgtacaataatttctatatgcctattatggatctgcaccccctgc**

**Citrus_aurantiifolia** **tttgatgttatttgacgtacaataatttctatatgcctattatggatctgcaccccctgc**

**Citrus_aurantium** **tttgatgttatttgacgtacaataatttctatatgcctattatggatctgcaccccctgc**

**Citrus_cavaleriei** **tttgatgttatttgacgtacaataatttctatatgcctattatggatctgcacgccctgc**

**Citrus_hongheensis** **tttgatgttatttgacgtacaataatttctatatgcctattatggatctgcaccccctgc**

**Citrus_limon** **tttgatgttatttgacgtacaataatttctatatgcctattatggatctgcaccccctgc**

**Citrus_maxima** **tttgatgttatttgacgtacaataatttctatatgcctattatggatctgcaccccctgc**

**Citrus_medica** **tttgatgttatttgacgtacaataatttctatatgcctattatggatctgcaccccctgc**

**Citrus_polytrifolia** **tttgatgttatttgacgtacaataatttctatatgcctattatggatctgcaccccctgc**

**Citrus_reticulata** **tttgatgttatttgacgtacaataatttctatatgcctattatggatctgcaccccctgc**

**Citrus_sinensis** **tttgatgttatttgacgtacaataatttctatatgcctattatggatctgcaccccctgc**

**Fortunella_bawangica_1** **tttgatgttatttgacgtacaataatttctatatgcctattatggatctgcaccccctgc**

**Fortunella_bawangica_2** **tttgatgttatttgacgtacaataatttctatatgcctattatggatctgcaccccctgc**

**Fortunella_hindsii_1** **tttgatgttatttgacgtacaataatttctatatgcctattatggatctgcaccccctgc**

**Fortunella_hindsii_2** **tttgatgttatttgacgtacaataatttctatatgcctattatggatctgcaccccctgc**

**Fortunella_hindsii_3** **tttgatgttatttgacgtacaataatttctatatgcctattatggatctgcaccccctgc**

**Fortunella_japonica_1** **tttgatgttatttgacgtacaataatttctatatgcctattatggatctgcaccccctgc**

**Fortunella_japonica_2** **tttgatgttatttgacgtacaataatttctatatgcctattatggatctgcaccccctgc**

**Fortunella_japonica_3** **tttgatgttatttgacgtacaataatttctatatgcctattatggatctgcaccccctgc**

**Fortunella_japonica_4** **tttgatgttatttgacgtacaataatttctatatgcctattatggatctgcaccccctgc**

**Fortunella_japonica_5** **tttgatgttatttgacgtacaataatttctatatgcctattatggatctgcaccccctgc**

**Fortunella_japonica_6** **tttgatgttatttgacgtacaataatttctatatgcctattatggatctgcaccccctgc**

**Fortunella_japonica_7** **tttgatgttatttgacgtacaataatttctatatgcctattatggatctgcaccccctgc**

**Fortunella_japonica_8** **tttgatgttatttgacgtacaataatttctatatgcctattatggatctgcaccccctgc**

**Fortunella_japonica_9** **tttgatgttatttgacgtacaataatttctatatgcctattatggatctgcaccccctgc**

**Fortunella_japonica_10** **tttgatgttatttgacgtacaataatttctatatgcctattatggatctgcaccccctgc**

**Fortunella_japonica_11** **tttgatgttatttgacgtacaataatttctatatgcctattatggatctgcaccccctgc**

**Fortunella_japonica_12** **tttgatgttatttgacgtacaataatttctatatgcctattatggatctgcaccccctgc**

**Fortunella_margarita_1** **tttgatgttatttgacgtacaataatttctatatgcctattatggatctgcaccccctgc**

**Fortunella_margarita_2** **tttgatgttatttgacgtacaataatttctatatgcctattatggatctgcaccccctgc**

**Fortunella_venosa_1** **tttgatgttatttgacgtacaataatttctatatgcctattatggatctgcaccccctgc**

**Fortunella_venosa_2** **tttgatgttatttgacgtacaataatttctatatgcctattatggatctgcaccccctgc**

**Fortunella_venosa_3** **tttgatgttatttgacgtacaataatttctatatgcctattatggatctgcaccccctgc**

19210	19220	19230	19240	19250	19260

....|....|....|....|....|....|....|....|....|....|....|....|

**Atalantia_kwangtungens** **gagcgataaaccttttggatcttattaaccaaagagatacgactttgcactatagttagc**

**Citrus_reticulata_1** **gatcgataaaccttttggatcttattaaccaaagagatacgactttgcactatagttagc**

**Citrus_reticulata_2** **gatcgataaaccttttggatcttattaaccaaagagatacgactttgcactatagttagc**

**Citrus_trifoliata_3** **gatcgataaaccttttggatcttattaaccaaagagatacgactttgcactatagttagc**

**Citrus_aurantiifolia** **gatcgataaaccttttggatcttattaaccaaagagatacgactttgcactatagttagc**

**Citrus_aurantium** **gatcgataaaccttttggatcttattaaccaaagagatacgactttgcactatagttagc**

**Citrus_cavaleriei** **gatcgataaaccttttggatcttattaaccaaagagatacgactttgcactatagttagc**

**Citrus_hongheensis** **gatcgataaaccttttggatcttattaaccaaagagatacgactttgcactatagttagc**

**Citrus_limon** **gatcgataaaccttttggatcttattaaccaaagagatacgactttgcactatagttagc**

**Citrus_maxima** **gatcgataaaccttttggatcttattaaccaaagagatacgactttgcactatagttagc**

**Citrus_medica** **gatcgataaaccttttggatcttattaaccaaagagatacgactttgcactatagttagc**

**Citrus_polytrifolia** **gatcgataaaccttttggatcttattaaccaaagagatacgactttgcactatagttagc**

**Citrus_reticulata** **gatcgataaaccttttggatcttattaaccaaagagatacgactttgcactatagttagc**

**Citrus_sinensis** **gatcgataaaccttttggatcttattaaccaaagagatacgactttgcactatagttagc**

**Fortunella_bawangica_1** **gatcgataaaccttttggatcttattaaccaaagagatacgactttgcactatagttagc**

**Fortunella_bawangica_2** **gatcgataaaccttttggatcttattaaccaaagagatacgactttgcactatagttagc**

**Fortunella_hindsii_1** **gatcgataaaccttttggatcttattaaccaaagagatacgactttgcactatagttagc**

**Fortunella_hindsii_2** **gatcgataaaccttttggatcttattaaccaaagagatacgactttgcactatagttagc**

**Fortunella_hindsii_3** **gatcgataaaccttttggatcttattaaccaaagagatacgactttgcactatagttagc**

**Fortunella_japonica_1** **gatcgataaaccttttggatcttattaaccaaagagatacgactttgcactatagttagc**

**Fortunella_japonica_2** **gatcgataaaccttttggatcttattaaccaaagagatacgactttgcactatagttagc**

**Fortunella_japonica_3** **gatcgataaaccttttggatcttattaaccaaagagatacgactttgcactatagttagc**

**Fortunella_japonica_4** **gatcgataaaccttttggatcttattaaccaaagagatacgactttgcactatagttagc**

**Fortunella_japonica_5** **gatcgataaaccttttggatcttattaaccaaagagatacgactttgcactatagttagc**

**Fortunella_japonica_6** **gatcgataaaccttttggatcttattaaccaaagagatacgactttgcactatagttagc**

**Fortunella_japonica_7** **gatcgataaaccttttggatcttattaaccaaagagatacgactttgcactatagttagc**

**Fortunella_japonica_8** **gatcgataaaccttttggatcttattaaccaaagagatacgactttgcactatagttagc**

**Fortunella_japonica_9** **gatcgataaaccttttggatcttattaaccaaagagatacgactttgcactatagttagc**

**Fortunella_japonica_10** **gatcgataaaccttttggatcttattaaccaaagagatacgactttgcactatagttagc**

**Fortunella_japonica_11** **gatcgataaaccttttggatcttattaaccaaagagatacgactttgcactatagttagc**

**Fortunella_japonica_12** **gatcgataaaccttttggatcttattaaccaaagagatacgactttgcactatagttagc**

**Fortunella_margarita_1** **gatcgataaaccttttggatcttattaaccaaagagatacgactttgcactatagttagc**

**Fortunella_margarita_2** **gatcgataaaccttttggatcttattaaccaaagagatacgactttgcactatagttagc**

**Fortunella_venosa_1** **gatcgataaaccttttggatcttattaaccaaagagatacgactttgcactatagttagc**

**Fortunella_venosa_2** **gatcgataaaccttttggatcttattaaccaaagagatacgactttgcactatagttagc**

**Fortunella_venosa_3** **gatcgataaaccttttggatcttattaaccaaagagatacgactttgcactatagttagc**

19270	19280	19290	19300	19310	19320

....|....|....|....|....|....|....|....|....|....|....|....|

**Atalantia_kwangtungens** **tcagcaccaatcaataatccccagggaatcccaagaattcttgttatactcgcgttccaa**

**Citrus_reticulata_1** **tcagcaccaatcaagaatccccagggaatcccaagaattcttgttatactcgcgttccaa**

**Citrus_reticulata_2** **tcagcaccaatcaagaatccccagggaatcccaagaattcttgttatactcgcgttccaa**

**Citrus_trifoliata_3** **tcagcaccaatcaagaatccccagggaatcccaagaattcttgttatactcgcgttccaa**

**Citrus_aurantiifolia** **tcagcaccaatcaagaatccccagggaatcccaagaattcttgttatactcgcgttccaa**

**Citrus_aurantium** **tcagcaccaatcaagaatccccagggaatcccaagaattcttgttatactcgcgttccaa**

**Citrus_cavaleriei** **tcagcaccaatcaagaatccccagggaatcccaagaattcttgttatactcgcgttccaa**

**Citrus_hongheensis** **tcagcaccaatcaagaatccccagggaatcccaagaattcttgttatactcgcgttccaa**

**Citrus_limon** **tcagcaccaatcaagaatccccagggaatcccaagaattcttgttatactcgcgttccaa**

**Citrus_maxima** **tcagcaccaatcaagaatccccagggaatcccaagaattcttgttatactcgcgttccaa**

**Citrus_medica** **tcagcaccaatcaagaatccccagggaatcccaagaattcttgttatactcgcgttccaa**

**Citrus_polytrifolia** **tcagcaccaatcaagaatccccagggaatcccaagaattcttgttatactcgcgttccaa**

**Citrus_reticulata** **tcagcaccaatcaagaatccccagggaatcccaagaattcttgttatactcgcgttccaa**

**Citrus_sinensis** **tcagcaccaatcaagaatccccagggaatcccaagaattcttgttatactcgcgttccaa**

**Fortunella_bawangica_1** **tcagcaccaatcaagaatccccagggaatcccaagaattcttgttatactcgcgttccaa**

**Fortunella_bawangica_2** **tcagcaccaatcaagaatccccagggaatcccaagaattcttgttatactcgcgttccaa**

**Fortunella_hindsii_1** **tcagcaccaatcaagaatccccagggaatcccaagaattcttgttatactcgcgttccaa**

**Fortunella_hindsii_2** **tcagcaccaatcaagaatccccagggaatcccaagaattcttgttatactcgcgttccaa**

**Fortunella_hindsii_3** **tcagcaccaatcaagaatccccagggaatcccaagaattcttgttatactcgcgttccaa**

**Fortunella_japonica_1** **tcagcaccaatcaagaatccccagggaatcccaagaattcttgttatactcgcattccaa**

**Fortunella_japonica_2** **tcagcaccaatcaagaatccccagggaatcccaagaattcttgttatactcgcattccaa**

**Fortunella_japonica_3** **tcagcaccaatcaagaatccccagggaatcccaagaattcttgttatactcgcattccaa**

**Fortunella_japonica_4** **tcagcaccaatcaagaatccccagggaatcccaagaattcttgttatactcgcattccaa**

**Fortunella_japonica_5** **tcagcaccaatcaagaatccccagggaatcccaagaattcttgttatactcgcattccaa**

**Fortunella_japonica_6** **tcagcaccaatcaagaatccccagggaatcccaagaattcttgttatactcgcattccaa**

**Fortunella_japonica_7** **tcagcaccaatcaagaatccccagggaatcccaagaattcttgttatactcgcattccaa**

**Fortunella_japonica_8** **tcagcaccaatcaagaatccccagggaatcccaagaattcttgttatactcgcattccaa**

**Fortunella_japonica_9** **tcagcaccaatcaagaatccccagggaatcccaagaattcttgttatactcgcattccaa**

**Fortunella_japonica_10** **tcagcaccaatcaagaatccccagggaatcccaagaattcttgttatactcgcgttccaa**

**Fortunella_japonica_11** **tcagcaccaatcaagaatccccagggaatcccaagaattcttgttatactcgcattccaa**

**Fortunella_japonica_12** **tcagcaccaatcaagaatccccagggaatcccaagaattcttgttatactcgcattccaa**

**Fortunella_margarita_1** **tcagcaccaatcaagaatccccagggaatcccaagaattcttgttatactcgcgttccaa**

**Fortunella_margarita_2** **tcagcaccaatcaagaatccccagggaatcccaagaattcttgttatactcgcgttccaa**

**Fortunella_venosa_1** **tcagcaccaatcaagaatccccagggaatcccaagaattcttgttatactcgcgttccaa**

**Fortunella_venosa_2** **tcagcaccaatcaagaatccccagggaatcccaagaattcttgttatactcgcgttccaa**

**Fortunella_venosa_3** **tcagcaccaatcaagaatccccagggaatcccaagaattcttgttatactcgcgttccaa**

19330	19340	19350	19360	19370	19380

....|....|....|....|....|....|....|....|....|....|....|....|

**Atalantia_kwangtungens** **ccctcaactctcttttctaggttcatcgatattgaatcaagcgaacgcacttctaacacc**

**Citrus_reticulata_1** **ccctcaactctcttttctaggttcatcgatattgaatcaagcgaacgcacttctaacacc**

**Citrus_reticulata_2** **ccctcaactctcttttctaggttcatcgatattgaatcaagcgaacgcacttctaacacc**

**Citrus_trifoliata_3** **ccctcaactctcttttctaggttcatcgatattgaatcaagcgaacgcacttctaacacc**

**Citrus_aurantiifolia** **ccctcaactctcttttctaggttcatcgatattgaatcaagcgaacgcacttctaacacc**

**Citrus_aurantium** **ccctcaactctcttttctaggttcatcgatattgaatcaagcgaacgcacttctaacacc**

**Citrus_cavaleriei** **ccctcaactctcttttctaggttcatcgatattgaatcaagcgaacgcacttctaacacc**

**Citrus_hongheensis** **ccctcaactctcttttctaggttcatcgatattgaatcaagcgaacgcacttctaacacc**

**Citrus_limon** **ccctcaactctcttttctaggttcatcgatattgaatcaagcgaacgcacttctaacacc**

**Citrus_maxima** **ccctcaactctcttttctaggttcatcgatattgaatcaagcgaacgcacttctaacacc**

**Citrus_medica** **ccctcaactctcttttctaggttcatcgatattgaatcaagcgaacgcacttctaacacc**

**Citrus_polytrifolia** **ccctcaactctcttttctaggttcatcgatattgaatcaagcgaacgcacttctaacacc**

**Citrus_reticulata** **ccctcaactctcttttctaggttcatcgatattgaatcaagcgaacgcacttctaacacc**

**Citrus_sinensis** **ccctcaactctcttttctaggttcatcgatattgaatcaagcgaacgcacttctaacacc**

**Fortunella_bawangica_1** **ccctcaactctcttttctaggttcatcgatattgaatcaagcgaacgcacttctaacacc**

**Fortunella_bawangica_2** **ccctcaactctcttttctaggttcatcgatattgaatcaagcgaacgcacttctaacacc**

**Fortunella_hindsii_1** **ccctcaactctcttttctaggttcatcgatattgaatcaagcgaacgcacttctaacacc**

**Fortunella_hindsii_2** **ccctcaactctcttttctaggttcatcgatattgaatcaagcgaacgcacttctaacacc**

**Fortunella_hindsii_3** **ccctcaactctcttttctaggttcatcgatattgaatcaagcgaacgcacttctaacacc**

**Fortunella_japonica_1** **ccctcaactctcttttctaggttcatcgatattgaatcaagcgaacgcacttctaacacc**

**Fortunella_japonica_2** **ccctcaactctcttttctaggttcatcgatattgaatcaagcgaacgcacttctaacacc**

**Fortunella_japonica_3** **ccctcaactctcttttctaggttcatcgatattgaatcaagcgaacgcacttctaacacc**

**Fortunella_japonica_4** **ccctcaactctcttttctaggttcatcgatattgaatcaagcgaacgcacttctaacacc**

**Fortunella_japonica_5** **ccctcaactctcttttctaggttcatcgatattgaatcaagcgaacgcacttctaacacc**

**Fortunella_japonica_6** **ccctcaactctcttttctaggttcatcgatattgaatcaagcgaacgcacttctaacacc**

**Fortunella_japonica_7** **ccctcaactctcttttctaggttcatcgatattgaatcaagcgaacgcacttctaacacc**

**Fortunella_japonica_8** **ccctcaactctcttttctaggttcatcgatattgaatcaagcgaacgcacttctaacacc**

**Fortunella_japonica_9** **ccctcaactctcttttctaggttcatcgatattgaatcaagcgaacgcacttctaacacc**

**Fortunella_japonica_10** **ccctcaactctcttttctaggttcatcgatattgaatcaagcgaacgcacttctaacacc**

**Fortunella_japonica_11** **ccctcaactctcttttctaggttcatcgatattgaatcaagcgaacgcacttctaacacc**

**Fortunella_japonica_12** **ccctcaactctcttttctaggttcatcgatattgaatcaagcgaacgcacttctaacacc**

**Fortunella_margarita_1** **ccctcaactctcttttctaggttcatcgatattgaatcaagcgaacgcacttctaacacc**

**Fortunella_margarita_2** **ccctcaactctcttttctaggttcatcgatattgaatcaagcgaacgcacttctaacacc**

**Fortunella_venosa_1** **ccctcaactctcttttctaggttcatcgatattgaatcaagcgaacgcacttctaacacc**

**Fortunella_venosa_2** **ccctcaactctcttttctaggttcatcgatattgaatcaagcgaacgcacttctaacacc**

**Fortunella_venosa_3** **ccctcaactctcttttctaggttcatcgatattgaatcaagcgaacgcacttctaacacc**

19390	19400	19410	19420	19430	19440

....|....|....|....|....|....|....|....|....|....|....|....|

**Atalantia_kwangtungens** **tgttctacttttggaagaccctgcgttatatcaccagatctcgatttttcatatataaat**

**Citrus_reticulata_1** **tgttctacttttggaagaccctgcgttatatcaccagatctcgatttttcatatataaat**

**Citrus_reticulata_2** **tgttctacttttggaagaccctgcgttatatcaccagatctcgatttttcatatataaat**

**Citrus_trifoliata_3** **tgttctacttttggaagaccctgcgttatatcaccagatctcgatttttcatatataaat**

**Citrus_aurantiifolia** **tgttctacttttggaagaccctgcgttatatcaccagatctcgatttttcatatataaat**

**Citrus_aurantium** **tgttctacttttggaagaccctgcgttatatcaccagatctcgatttttcatatataaat**

**Citrus_cavaleriei** **tgttctacttttggaagaccctgcgttatatcaacagatctcgatttttcatatataaat**

**Citrus_hongheensis** **tgttctacttttggaagaccctgcgttatatcaccagatctcgatttttcatatataaat**

**Citrus_limon** **tgttctacttttggaagaccctgcgttatatcaccagatctcgatttttcatatataaat**

**Citrus_maxima** **tgttctacttttggaagaccctgcgttatatcaccagatctcgatttttcatatataaat**

**Citrus_medica** **tgttctacttttggaagaccctgcgttatatcaccagatctcgatttttcatatataaat**

**Citrus_polytrifolia** **tgttctacttttggaagaccctgcgttatatcaccagatctcgatttttcatatataaat**

**Citrus_reticulata** **tgttctacttttggaagaccctgcgttatatcaccagatctcgatttttcatatataaat**

**Citrus_sinensis** **tgttctacttttggaagaccctgcgttatatcaccagatctcgatttttcatatataaat**

**Fortunella_bawangica_1** **tgttctacttttggaagaccctgcgttatatcaccagatctcgatttttcatatataaat**

**Fortunella_bawangica_2** **tgttctacttttggaagaccctgcgttatatcaccagatctcgatttttcatatataaat**

**Fortunella_hindsii_1** **tgttctacttttggaagaccctgcgttatatcaccagatctcgatttttcatatataaat**

**Fortunella_hindsii_2** **tgttctacttttggaagaccctgcgttatatcaccagatctcgatttttcatatataaat**

**Fortunella_hindsii_3** **tgttctacttttggaagaccctgcgttatatcaccagatctcgatttttcatatataaat**

**Fortunella_japonica_1** **tgttctacttttggaagaccctgcgttatatcaccagatctcgatttttcatatataaat**

**Fortunella_japonica_2** **tgttctacttttggaagaccctgcgttatatcaccagatctcgatttttcatatataaat**

**Fortunella_japonica_3** **tgttctacttttggaagaccctgcgttatatcaccagatctcgatttttcatatataaat**

**Fortunella_japonica_4** **tgttctacttttggaagaccctgcgttatatcaccagatctcgatttttcatatataaat**

**Fortunella_japonica_5** **tgttctacttttggaagaccctgcgttatatcaccagatctcgatttttcatatataaat**

**Fortunella_japonica_6** **tgttctacttttggaagaccctgcgttatatcaccagatctcgatttttcatatataaat**

**Fortunella_japonica_7** **tgttctacttttggaagaccctgcgttatatcaccagatctcgatttttcatatataaat**

**Fortunella_japonica_8** **tgttctacttttggaagaccctgcgttatatcaccagatctcgatttttcatatataaat**

**Fortunella_japonica_9** **tgttctacttttggaagaccctgcgttatatcaccagatctcgatttttcatatataaat**

**Fortunella_japonica_10** **tgttctacttttggaagaccctgcgttatatcaccagatctcgatttttcatatataaat**

**Fortunella_japonica_11** **tgttctacttttggaagaccctgcgttatatcaccagatctcgatttttcatatataaat**

**Fortunella_japonica_12** **tgttctacttttggaagaccctgcgttatatcaccagatctcgatttttcatatataaat**

**Fortunella_margarita_1** **tgttctacttttggaagaccctgcgttatatcaccagatctcgatttttcatatataaat**

**Fortunella_margarita_2** **tgttctacttttggaagaccctgcgttatatcaccagatctcgatttttcatatataaat**

**Fortunella_venosa_1** **tgttctacttttggaagaccctgcgttatatcaccagatctcgatttttcatatataaat**

**Fortunella_venosa_2** **tgttctacttttggaagaccctgcgttatatcaccagatctcgatttttcatatataaat**

**Fortunella_venosa_3** **tgttctacttttggaagaccctgcgttatatcaccagatctcgatttttcatatataaat**

19450	19460	19470	19480	19490	19500

....|....|....|....|....|....|....|....|....|....|....|....|

**Atalantia_kwangtungens** **gtaactaatgtatccccttcgtaaagaattgatccataatgcccatgaacagttgctcca**

**Citrus_reticulata_1** **gtaactaatgtatccccttcggaaaggattgctccataatgcccatgaacagttgctcca**

**Citrus_reticulata_2** **gtaactaatgtatccccttcggaaaggattgctccataatgcccatgaacagttgctcca**

**Citrus_trifoliata_3** **gtaactaatgtatccccttcgtaaaggattgctccataatgcccatgaacagttgctcca**

**Citrus_aurantiifolia** **gtaactaatgtatccccttcgtaaaggattgctccataatgcccatgaacagttgctcca**

**Citrus_aurantium** **gtaactaatgtatccccttcgtaaaggattgctccataatgcccatgaacagttgctcca**

**Citrus_cavaleriei** **gtaactaatgtatccccttcggaaaggattgctccataatgcccatgaacagttgctcca**

**Citrus_hongheensis** **gtaactaatgtatccccttcgtaaaggattgctccataatgcccatgaacagttgctcca**

**Citrus_limon** **gtaactaatgtatccccttcgtaaaggattgctccataatgcccatgaacagttgctcca**

**Citrus_maxima** **gtaactaatgtatccccttcgtaaaggattgctccataatgcccatgaacagttgctcca**

**Citrus_medica** **gtaactaatgtatccccttcgtaaaggattgctccataatgcccatgaacagttgctcca**

**Citrus_polytrifolia** **gtaactaatgtatccccttcgtaaaggattgctccataatgcccatgaacagttgctcca**

**Citrus_reticulata** **gtaactaatgtatccccttcggaaaggattgctccataatgcccatgaacagttgctcca**

**Citrus_sinensis** **gtaactaatgtatccccttcgtaaaggattgctccataatgcccatgaacagttgctcca**

**Fortunella_bawangica_1** **gtaactaatgtatccccttcgtaaaggattgctccataatgcccatgaacagttgctcca**

**Fortunella_bawangica_2** **gtaactaatgtatccccttcgtaaaggattgctccataatgcccatgaacagttgctcca**

**Fortunella_hindsii_1** **gtaactaatgtatccccttcgtaaaggattgctccataatgcccatgaacagttgctcca**

**Fortunella_hindsii_2** **gtaactaatgtatccccttcgtaaaggattgctccataatgcccatgaacagttgctcca**

**Fortunella_hindsii_3** **gtaactaatgtatccccttcgtaaaggattgctccataatgcccatgaacagttgctcca**

**Fortunella_japonica_1** **gtaactaatgtatccccttcgtaaaggattgctccataatgcccatgaacagttgctcca**

**Fortunella_japonica_2** **gtaactaatgtatccccttcgtaaaggattgctccataatgcccatgaacagttgctcca**

**Fortunella_japonica_3** **gtaactaatgtatccccttcgtaaaggattgctccataatgcccatgaacagttgctcca**

**Fortunella_japonica_4** **gtaactaatgtatccccttcgtaaaggattgctccataatgcccatgaacagttgctcca**

**Fortunella_japonica_5** **gtaactaatgtatccccttcgtaaaggattgctccataatgcccatgaacagttgctcca**

**Fortunella_japonica_6** **gtaactaatgtatccccttcgtaaaggattgctccataatgcccatgaacagttgctcca**

**Fortunella_japonica_7** **gtaactaatgtatccccttcgtaaaggattgctccataatgcccatgaacagttgctcca**

**Fortunella_japonica_8** **gtaactaatgtatccccttcgtaaaggattgctccataatgcccatgaacagttgctcca**

**Fortunella_japonica_9** **gtaactaatgtatccccttcgtaaaggattgctccataatgcccatgaacagttgctcca**

**Fortunella_japonica_10** **gtaactaatgtatccccttcgtaaaggattgctccataatgcccatgaacagttgctcca**

**Fortunella_japonica_11** **gtaactaatgtatccccttcgtaaaggattgctccataatgcccatgaacagttgctcca**

**Fortunella_japonica_12** **gtaactaatgtatccccttcgtaaaggattgctccataatgcccatgaacagttgctcca**

**Fortunella_margarita_1** **gtaactaatgtatccccttcgtaaaggattgctccataatgcccatgaacagttgctcca**

**Fortunella_margarita_2** **gtaactaatgtatccccttcgtaaaggattgctccataatgcccatgaacagttgctcca**

**Fortunella_venosa_1** **gtaactaatgtatccccttcgtaaaggattgctccataatgcccatgaacagttgctcca**

**Fortunella_venosa_2** **gtaactaatgtatccccttcgtaaaggattgctccataatgcccatgaacagttgctcca**

**Fortunella_venosa_3** **gtaactaatgtatccccttcgtaaaggattgctccataatgcccatgaacagttgctcca**

19510	19520	19530	19540	19550	19560

....|....|....|....|....|....|....|....|....|....|....|....|

**Atalantia_kwangtungens** **ggagtagccaaataaggcttagctgatcgtattactacagagttaacttgaacaattaaa**

**Citrus_reticulata_1** **ggagtagccaaataaggcttagctgatcgtattactacagagttaacttgaacaattaaa**

**Citrus_reticulata_2** **ggagtagccaaataaggcttagctgatcgtattactacagagttaacttgaacaattaaa**

**Citrus_trifoliata_3** **ggagtagccaaataaggcttagctgatcgtattactacagagttaacttgaacaattaaa**

**Citrus_aurantiifolia** **ggagtagccaaataaggcttagctgatcgtattactacagagttaacttgaacaattaaa**

**Citrus_aurantium** **ggagtagccaaataaggcttagctgatcgtattactacagagttaacttgaacaattaaa**

**Citrus_cavaleriei** **ggagtagccaaataaggcttagctgatcgtattactacagagttaacttgaacaattaaa**

**Citrus_hongheensis** **ggagtagccaaataaggcttagctgatcgtattactacagagttaacttgaacaattaaa**

**Citrus_limon** **ggagtagccaaataaggcttagctgatcgtattactacagagttaacttgaacaattaaa**

**Citrus_maxima** **ggagtagccaaataaggcttagctgatcgtattactacagagttaacttgaacaattaaa**

**Citrus_medica** **ggagtagccaaataaggcttagctgatcgtattactacagagttaacttgaacaattaaa**

**Citrus_polytrifolia** **ggagtagccaaataaggcttagctgatcgtattactacagagttaacttgaacaattaaa**

**Citrus_reticulata** **ggagtagccaaataaggcttagctgatcgtattactacagagttaacttgaacaattaaa**

**Citrus_sinensis** **ggagtagccaaataaggcttagctgatcgtattactacagagttaacttgaacaattaaa**

**Fortunella_bawangica_1** **ggagtagccaaataaggcttagctgatcgtattactacagagttaacttgaacaattaaa**

**Fortunella_bawangica_2** **ggagtagccaaataaggcttagctgatcgtattactacagagttaacttgaacaattaaa**

**Fortunella_hindsii_1** **ggagtagccaaataaggcttagctgatcgtattactacagagttaacttgaacaattaaa**

**Fortunella_hindsii_2** **ggagtagccaaataaggcttagctgatcgtattactacagagttaacttgaacaattaaa**

**Fortunella_hindsii_3** **ggagtagccaaataaggcttagctgatcgtattactacagagttaacttgaacaattaaa**

**Fortunella_japonica_1** **ggagtagccaaataaggcttagctgatcgtattactacagagttaacttgaacaattaaa**

**Fortunella_japonica_2** **ggagtagccaaataaggcttagctgatcgtattactacagagttaacttgaacaattaaa**

**Fortunella_japonica_3** **ggagtagccaaataaggcttagctgatcgtattactacagagttaacttgaacaattaaa**

**Fortunella_japonica_4** **ggagtagccaaataaggcttagctgatcgtattactacagagttaacttgaacaattaaa**

**Fortunella_japonica_5** **ggagtagccaaataaggcttagctgatcgtattactacagagttaacttgaacaattaaa**

**Fortunella_japonica_6** **ggagtagccaaataaggcttagctgatcgtattactacagagttaacttgaacaattaaa**

**Fortunella_japonica_7** **ggagtagccaaataaggcttagctgatcgtattactacagagttaacttgaacaattaaa**

**Fortunella_japonica_8** **ggagtagccaaataaggcttagctgatcgtattactacagagttaacttgaacaattaaa**

**Fortunella_japonica_9** **ggagtagccaaataaggcttagctgatcgtattactacagagttaacttgaacaattaaa**

**Fortunella_japonica_10** **ggagtagccaaataaggcttagctgatcgtattactacagagttaacttgaacaattaaa**

**Fortunella_japonica_11** **ggagtagccaaataaggcttagctgatcgtattactacagagttaacttgaacaattaaa**

**Fortunella_japonica_12** **ggagtagccaaataaggcttagctgatcgtattactacagagttaacttgaacaattaaa**

**Fortunella_margarita_1** **ggagtagccaaataaggcttagctgatcgtattactacagagttaacttgaacaattaaa**

**Fortunella_margarita_2** **ggagtagccaaataaggcttagctgatcgtattactacagagttaacttgaacaattaaa**

**Fortunella_venosa_1** **ggagtagccaaataaggcttagctgatcgtattactacagagttaacttgaacaattaaa**

**Fortunella_venosa_2** **ggagtagccaaataaggcttagctgatcgtattactacagagttaacttgaacaattaaa**

**Fortunella_venosa_3** **ggagtagccaaataaggcttagctgatcgtattactacagagttaacttgaacaattaaa**

19570	19580	19590	19600	19610	19620

....|....|....|....|....|....|....|....|....|....|....|....|

**Atalantia_kwangtungens** **acttgaccggattttagggatggtccccttttggttatacgtacattttcacaaagaaac**

**Citrus_reticulata_1** **acttgaccggattttaggtatggtccccttttggttatacgtacattttcacaaagaaac**

**Citrus_reticulata_2** **acttgaccggattttaggtatggtccccttttggttatacgtacattttcacaaagaaac**

**Citrus_trifoliata_3** **acttgaccggattttaggtatggtccccttttggttatacctacattttcacaaagaaac**

**Citrus_aurantiifolia** **acttgaccggattttaggtatggtccccttttggttatacgtacattttcacaaagaaac**

**Citrus_aurantium** **acttgaccggattttaggtatggtccccttttggttatacgtacattttcacaaagaaac**

**Citrus_cavaleriei** **acttgaccggattttaggtatggtccccttttggttatacgtacattttcacaaagaaac**

**Citrus_hongheensis** **acttgaccggattttaggtatggtccccttttggttatacgtacattttcacaaagaaac**

**Citrus_limon** **acttgaccggattttaggtatggtccccttttggttatacgtacattttcacaaagaaac**

**Citrus_maxima** **acttgaccggattttaggtatggtccccttttggttatacgtacattttcacaaagaaac**

**Citrus_medica** **acttgaccggattttaggtatggtccccttttggttatacgtacattttcacaaagaaac**

**Citrus_polytrifolia** **acttgaccggattttaggtatggtccccttttggttatacctacattttcacaaagaaac**

**Citrus_reticulata** **acttgaccggattttaggtatggtccccttttggttatacgtacattttcacaaagaaac**

**Citrus_sinensis** **acttgaccggattttaggtatggtccccttttggttatacgtacattttcacaaagaaac**

**Fortunella_bawangica_1** **acttgaccggattttaggtatggtccccttttggttatacgtacattttcacaaagaaac**

**Fortunella_bawangica_2** **acttgaccggattttaggtatggtccccttttggttatacgtacattttcacaaagaaac**

**Fortunella_hindsii_1** **acttgaccggattttaggtatggtccccttttggttatacgtacattttcacaaagaaac**

**Fortunella_hindsii_2** **acttgaccggattttaggtatggtccccttttggttatacgtacattttcacaaagaaac**

**Fortunella_hindsii_3** **acttgaccggattttaggtatggtccccttttggttatacgtacattttcacaaagaaac**

**Fortunella_japonica_1** **acttgaccggattttaggtatggtccccttttggttatacgtacattttcacaaagaaac**

**Fortunella_japonica_2** **acttgaccggattttaggtatggtccccttttggttatacgtacattttcacaaagaaac**

**Fortunella_japonica_3** **acttgaccggattttaggtatggtccccttttggttatacgtacattttcacaaagaaac**

**Fortunella_japonica_4** **acttgaccggattttaggtatggtccccttttggttatacgtacattttcacaaagaaac**

**Fortunella_japonica_5** **acttgaccggattttaggtatggtccccttttggttatacgtacattttcacaaagaaac**

**Fortunella_japonica_6** **acttgaccggattttaggtatggtccccttttggttatacgtacattttcacaaagaaac**

**Fortunella_japonica_7** **acttgaccggattttaggtatggtccccttttggttatacgtacattttcacaaagaaac**

**Fortunella_japonica_8** **acttgaccggattttaggtatggtccccttttggttatacgtacattttcacaaagaaac**

**Fortunella_japonica_9** **acttgaccggattttaggtatggtccccttttggttatacgtacattttcacaaagaaac**

**Fortunella_japonica_10** **acttgaccggattttaggtatggtccccttttggttatacgtacattttcacaaagaaac**

**Fortunella_japonica_11** **acttgaccggattttaggtatggtccccttttggttatacgtacattttcacaaagaaac**

**Fortunella_japonica_12** **acttgaccggattttaggtatggtccccttttggttatacgtacattttcacaaagaaac**

**Fortunella_margarita_1** **acttgaccggattttaggtatggtccccttttggttatacgtacattttcacaaagaaac**

**Fortunella_margarita_2** **acttgaccggattttaggtatggtccccttttggttatacgtacattttcacaaagaaac**

**Fortunella_venosa_1** **acttgaccggattttaggtatggtccccttttggttatacgtacattttcacaaagaaac**

**Fortunella_venosa_2** **acttgaccggattttaggtatggtccccttttggttatacgtacattttcacaaagaaac**

**Fortunella_venosa_3** **acttgaccggattttaggtatggtccccttttggttatacgtacattttcacaaagaaac**

19630	19640	19650	19660	19670	19680

....|....|....|....|....|....|....|....|....|....|....|....|

**Atalantia_kwangtungens** **tgcccaagactaattatcggggacatttcctcacaataattaggctggagaaaataccaa**

**Citrus_reticulata_1** **tgcccaagactaattatcggggacatttcctcacaataattaggctggagaaaataccaa**

**Citrus_reticulata_2** **tgcccaagactaattatcggggacatttcctcacaataattaggctggagaaaataccaa**

**Citrus_trifoliata_3** **tgcccaagactaattatcggggacatttcctcacaataattaggctggagaaaataccaa**

**Citrus_aurantiifolia** **tgcccaagactaattatcggggacatttcctcacaataattaggctggagaaaataccaa**

**Citrus_aurantium** **tgcccaagactaattatcggggacatttcctcacaataattaggctggagaaaataccaa**

**Citrus_cavaleriei** **tgcccaagactaattatcggggacatttcctcacaataattaggctggagaaaataccaa**

**Citrus_hongheensis** **tgcccaagactaattatcggggacatttcctcacaataattaggctggagaaaataccaa**

**Citrus_limon** **tgcccaagactaattatcggggacatttcctcacaataattaggctggagaaaataccaa**

**Citrus_maxima** **tgcccaagactaattatcggggacatttcctcacaataattaggctggagaaaataccaa**

**Citrus_medica** **tgcccaagactaattatcggggacatttcctcacaataattaggctggagaaaataccaa**

**Citrus_polytrifolia** **tgcccaagactaattatcggggacatttcctcacaataattaggctggagaaaataccaa**

**Citrus_reticulata** **tgcccaagactaattatcggggacatttcctcacaataattaggctggagaaaataccaa**

**Citrus_sinensis** **tgcccaagactaattatcggggacatttcctcacaataattaggctggagaaaataccaa**

**Fortunella_bawangica_1** **tgcccaagactaattatcggggacatttcctcacaataattaggctggagaaaataccaa**

**Fortunella_bawangica_2** **tgcccaagactaattatcggggacatttcctcacaataattaggctggagaaaataccaa**

**Fortunella_hindsii_1** **tgcccaagactaattatcggggacatttcctcacaataattaggctggagaaaataccaa**

**Fortunella_hindsii_2** **tgcccaagactaattatcggggacatttcctcacaataattaggctggagaaaataccaa**

**Fortunella_hindsii_3** **tgcccaagactaattatcggggacatttcctcacaataattaggctggagaaaataccaa**

**Fortunella_japonica_1** **tgcccaagactaattatcggggacatttcctcacaataattaggctggagaaaataccaa**

**Fortunella_japonica_2** **tgcccaagactaattatcggggacatttcctcacaataattaggctggagaaaataccaa**

**Fortunella_japonica_3** **tgcccaagactaattatcggggacatttcctcacaataattaggctggagaaaataccaa**

**Fortunella_japonica_4** **tgcccaagactaattatcggggacatttcctcacaataattaggctggagaaaataccaa**

**Fortunella_japonica_5** **tgcccaagactaattatcggggacatttcctcacaataattaggctggagaaaataccaa**

**Fortunella_japonica_6** **tgcccaagactaattatcggggacatttcctcacaataattaggctggagaaaataccaa**

**Fortunella_japonica_7** **tgcccaagactaattatcggggacatttcctcacaataattaggctggagaaaataccaa**

**Fortunella_japonica_8** **tgcccaagactaattatcggggacatttcctcacaataattaggctggagaaaataccaa**

**Fortunella_japonica_9** **tgcccaagactaattatcggggacatttcctcacaataattaggctggagaaaataccaa**

**Fortunella_japonica_10** **tgcccaagactaattatcggggacatttcctcacaataattaggctggagaaaataccaa**

**Fortunella_japonica_11** **tgcccaagactaattatcggggacatttcctcacaataattaggctggagaaaataccaa**

**Fortunella_japonica_12** **tgcccaagactaattatcggggacatttcctcacaataattaggctggagaaaataccaa**

**Fortunella_margarita_1** **tgcccaagactaattatcggggacatttcctcacaataattaggctggagaaaataccaa**

**Fortunella_margarita_2** **tgcccaagactaattatcggggacatttcctcacaataattaggctggagaaaataccaa**

**Fortunella_venosa_1** **tgcccaagactaattatcggggacatttcctcacaataattaggctggagaaaataccaa**

**Fortunella_venosa_2** **tgcccaagactaattatcggggacatttcctcacaataattaggctggagaaaataccaa**

**Fortunella_venosa_3** **tgcccaagactaattatcggggacatttcctcacaataattaggctggagaaaataccaa**

19690	19700	19710	19720	19730	19740

....|....|....|....|....|....|....|....|....|....|....|....|

**Atalantia_kwangtungens** **ttcaaattaaatggattcaaaaggatcttactatctgtatcaggattataaatttccccg**

**Citrus_reticulata_1** **ttcaaattaaatggattcaaaaggatcttactatctgtatcaggattataaatttccccg**

**Citrus_reticulata_2** **ttcaaattaaatggattcaaaaggatcttactatctgtatcaggattataaatttccccg**

**Citrus_trifoliata_3** **ttcaaattaaatggattcaaaaggatcttactatctgtatcaggattataaatttccccg**

**Citrus_aurantiifolia** **ttcaaattaaatggattcaaaaggatcttactatctgtatcaggattataaatttccccg**

**Citrus_aurantium** **ttcaaattaaatggattcaaaaggatcttactatctgtatcaggattataaatttccccg**

**Citrus_cavaleriei** **ttcaaattaaatggattcaaaaggatcttactatctgtatcaggattataaatttccccg**

**Citrus_hongheensis** **ttcaaattaaatggattcaaaaggatcttactatctgtatcaggattataaatttccccg**

**Citrus_limon** **ttcaaattaaatggattcaaaaggatcttactatctgtatcaggattataaatttccccg**

**Citrus_maxima** **ttcaaattaaatggattcaaaaggatcttactatctgtatcaggattataaatttccccg**

**Citrus_medica** **ttcaaattaaatggattcaaaaggatcttactatctgtatcaggattataaatttccccg**

**Citrus_polytrifolia** **ttcaaattaaatggattcaaaaggatcttactatctgtatcaggattataaatttccccg**

**Citrus_reticulata** **ttcaaattaaatggattcaaaaggatcttactatctgtatcaggattataaatttccccg**

**Citrus_sinensis** **ttcaaattaaatggattcaaaaggatcttactatctgtatcaggattataaatttccccg**

**Fortunella_bawangica_1** **ttcaaattaaatggattcaaaaggatcttactatctgtatcaggattataaatttccccg**

**Fortunella_bawangica_2** **ttcaaattaaatggattcaaaaggatcttactatctgtatcaggattataaatttccccg**

**Fortunella_hindsii_1** **ttcaaattaaatggattcaaaaggatcttactatctgtatcaggattataaatttccccg**

**Fortunella_hindsii_2** **ttcaaattaaatggattcaaaaggatcttactatctgtatcaggattataaatttccccg**

**Fortunella_hindsii_3** **ttcaaattaaatggattcaaaaggatcttactatctgtatcaggattataaatttccccg**

**Fortunella_japonica_1** **ttcaaattaaatggattcaaaaggatcttactatctgtatcaggattataaatttccccg**

**Fortunella_japonica_2** **ttcaaattaaatggattcaaaaggatcttactatctgtatcaggattataaatttccccg**

**Fortunella_japonica_3** **ttcaaattaaatggattcaaaaggatcttactatctgtatcaggattataaatttccccg**

**Fortunella_japonica_4** **ttcaaattaaatggattcaaaaggatcttactatctgtatcaggattataaatttccccg**

**Fortunella_japonica_5** **ttcaaattaaatggattcaaaaggatcttactatctgtatcaggattataaatttccccg**

**Fortunella_japonica_6** **ttcaaattaaatggattcaaaaggatcttactatctgtatcaggattataaatttccccg**

**Fortunella_japonica_7** **ttcaaattaaatggattcaaaaggatcttactatctgtatcaggattataaatttccccg**

**Fortunella_japonica_8** **ttcaaattaaatggattcaaaaggatcttactatctgtatcaggattataaatttccccg**

**Fortunella_japonica_9** **ttcaaattaaatggattcaaaaggatcttactatctgtatcaggattataaatttccccg**

**Fortunella_japonica_10** **ttcaaattaaatggattcaaaaggatcttactatctgtatcaggattataaatttccccg**

**Fortunella_japonica_11** **ttcaaattaaatggattcaaaaggatcttactatctgtatcaggattataaatttccccg**

**Fortunella_japonica_12** **ttcaaattaaatggattcaaaaggatcttactatctgtatcaggattataaatttccccg**

**Fortunella_margarita_1** **ttcaaattaaatggattcaaaaggatcttactatctgtatcaggattataaatttccccg**

**Fortunella_margarita_2** **ttcaaattaaatggattcaaaaggatcttactatctgtatcaggattataaatttccccg**

**Fortunella_venosa_1** **ttcaaattaaatggattcaaaaggatcttactatctgtatcaggattataaatttccccg**

**Fortunella_venosa_2** **ttcaaattaaatggattcaaaaggatcttactatctgtatcaggattataaatttccccg**

**Fortunella_venosa_3** **ttcaaattaaatggattcaaaaggatcttactatctgtatcaggattataaatttccccg**

19750	19760	19770	19780	19790	19800

....|....|....|....|....|....|....|....|....|....|....|....|

**Atalantia_kwangtungens** **ttttcgtccattaaataatattgaagtacttgaaaaggctgttttaaattgtcaagttgc**

**Citrus_reticulata_1** **gtttcgtccattaaataatatttaagtacttgaaaaggctgttttaaattgtcaagtttc**

**Citrus_reticulata_2** **gtttcgtccattaaataatatttaagtacttgaaaaggctgttttaaattgtcaagtttc**

**Citrus_trifoliata_3** **gtttcgtccattaaataatatttaagtacttgaaaaggctgttttaaattgtcaagtttc**

**Citrus_aurantiifolia** **gtttcgtccattaaataatatttaagtacttgaaaaggctgttttaaattgtcaagtttc**

**Citrus_aurantium** **gtttcgtccattaaataatatttaagtacttgaaaaggctgttttaaattgtcaagtttc**

**Citrus_cavaleriei** **gtttcgtccattaaataatatttaagtacttgaaaaggctgttttaaattgtcaagtttc**

**Citrus_hongheensis** **gtttcgtccattaaataatatttaagtacttgaaaaggctgttttaaattgtcaagtttc**

**Citrus_limon** **gtttcgtccattaaataatatttaagtacttgaaaaggctgttttaaattgtcaagtttc**

**Citrus_maxima** **gtttcgtccattaaataatatttaagtacttgaaaaggctgttttaaattgtcaagtttc**

**Citrus_medica** **gtttcgtccattaaataatatttaagtacttgaaaaggctgttttaaattgtcaagtttc**

**Citrus_polytrifolia** **gtttcgtccattaaataatatttaagtacttgaaaaggctgttttaaattgtcaagtttc**

**Citrus_reticulata** **gtttcgtccattaaataatatttaagtacttgaaaaggctgttttaaattgtcaagtttc**

**Citrus_sinensis** **gtttcgtccattaaataatatttaagtacttgaaaaggctgttttaaattgtcaagtttc**

**Fortunella_bawangica_1** **gtttcgtccattaaataatatttaagtacttgaaaaggctgttttaaattgtcaagtttc**

**Fortunella_bawangica_2** **gtttcgtccattaaataatatttaagtacttgaaaaggctgttttaaattgtcaagtttc**

**Fortunella_hindsii_1** **gtttcgtccattaaataatatttaagtacttgaaaaggctgttttaaattgtcaagtttc**

**Fortunella_hindsii_2** **gtttcgtccattaaataatatttaagtacttgaaaaggctgttttaaattgtcaagtttc**

**Fortunella_hindsii_3** **gtttcgtccattaaataatatttaagtacttgaaaaggctgttttaaattgtcaagtttc**

**Fortunella_japonica_1** **gtttcgtccattaaataatatttaagtacttgaaaaggctgttttaaattgtcaagtttc**

**Fortunella_japonica_2** **gtttcgtccattaaataatatttaagtacttgaaaaggctgttttaaattgtcaagtttc**

**Fortunella_japonica_3** **gtttcgtccattaaataatatttaagtacttgaaaaggctgttttaaattgtcaagtttc**

**Fortunella_japonica_4** **gtttcgtccattaaataatatttaagtacttgaaaaggctgttttaaattgtcaagtttc**

**Fortunella_japonica_5** **gtttcgtccattaaataatatttaagtacttgaaaaggctgttttaaattgtcaagtttc**

**Fortunella_japonica_6** **gtttcgtccattaaataatatttaagtacttgaaaaggctgttttaaattgtcaagtttc**

**Fortunella_japonica_7** **gtttcgtccattaaataatatttaagtacttgaaaaggctgttttaaattgtcaagtttc**

**Fortunella_japonica_8** **gtttcgtccattaaataatatttaagtacttgaaaaggctgttttaaattgtcaagtttc**

**Fortunella_japonica_9** **gtttcgtccattaaataatatttaagtacttgaaaaggctgttttaaattgtcaagtttc**

**Fortunella_japonica_10** **gtttcgtccattaaataatatttaagtacttgaaaaggctgttttaaattgtcaagtttc**

**Fortunella_japonica_11** **gtttcgtccattaaataatatttaagtacttgaaaaggctgttttaaattgtcaagtttc**

**Fortunella_japonica_12** **gtttcgtccattaaataatatttaagtacttgaaaaggctgttttaaattgtcaagtttc**

**Fortunella_margarita_1** **gtttcgtccattaaataatatttaagtacttgaaaaggctgttttaaattgtcaagtttc**

**Fortunella_margarita_2** **gtttcgtccattaaataatatttaagtacttgaaaaggctgttttaaattgtcaagtttc**

**Fortunella_venosa_1** **gtttcgtccattaaataatatttaagtacttgaaaaggctgttttaaattgtcaagtttc**

**Fortunella_venosa_2** **gtttcgtccattaaataatatttaagtacttgaaaaggctgttttaaattgtcaagtttc**

**Fortunella_venosa_3** **gtttcgtccattaaataatatttaagtacttgaaaaggctgttttaaattgtcaagtttc**

19810	19820	19830	19840	19850	19860

....|....|....|....|....|....|....|....|....|....|....|....|

**Atalantia_kwangtungens** **aaatatttagttaccgagatatgattatgagttattaaatagtaaaatgaataaaaattc**

**Citrus_reticulata_1** **aaatatttagttaccgagatatgattatgagttattaaatagtaaaatgaataaaaattc**

**Citrus_reticulata_2** **aaatatttagttaccgagatatgattatgagttattaaatagtaaaatgaataaaaattc**

**Citrus_trifoliata_3** **aaatatttagttaccgagatatgattatgagttattaaatagtaaaatgaataaaaattc**

**Citrus_aurantiifolia** **aaatatttagttaccgagatatgattatgagttattaaatagtaaaatgaataaaaattc**

**Citrus_aurantium** **aaatatttagttaccgagatatgattatgagttattaaatagtaaaatgaataaaaattc**

**Citrus_cavaleriei** **aaatatttagttaccgagatatgattatgagttattaaatagtaaaatgaataaaaattc**

**Citrus_hongheensis** **aaatatttagttaccgagatatgattatgagttattaaatagtaaaatgaataaaaattc**

**Citrus_limon** **aaatatttagttaccgagatatgattatgagttattaaatagtaaaatgaataaaaattc**

**Citrus_maxima** **aaatatttagttaccgagatatgattatgagttattaaatagtaaaatgaataaaaattc**

**Citrus_medica** **aaatatttagttaccgagatatgattatgagttattaaatagtaaaatgaataaaaattc**

**Citrus_polytrifolia** **aaatatttagttaccgatatatgattatgagttattaaatagtaaaatgaataaaaattc**

**Citrus_reticulata** **aaatatttagttaccgagatatgattatgagttattaaatagtaaaatgaataaaaattc**

**Citrus_sinensis** **aaatatttagttaccgagatatgattatgagttattaaatagtaaaatgaataaaaattc**

**Fortunella_bawangica_1** **aaatatttagttaccgagatatgattatgagttattaaatagtaaaatgaataaaaattc**

**Fortunella_bawangica_2** **aaatatttagttaccgagatatgattatgagttattaaatagtaaaatgaataaaaattc**

**Fortunella_hindsii_1** **aaatatttagttaccgagatatgattatgagttattaaatagtaaaatgaataaaaattc**

**Fortunella_hindsii_2** **aaatatttagttaccgagatatgattatgagttattaaatagtaaaatgaataaaaattc**

**Fortunella_hindsii_3** **aaatatttagttaccgagatatgattatgagttattaaatagtaaaatgaataaaaattc**

**Fortunella_japonica_1** **aaatatttagttaccgagatatgattatgagttattaaatagtaaaatgaataaaaattc**

**Fortunella_japonica_2** **aaatatttagttaccgagatatgattatgagttattaaatagtaaaatgaataaaaattc**

**Fortunella_japonica_3** **aaatatttagttaccgagatatgattatgagttattaaatagtaaaatgaataaaaattc**

**Fortunella_japonica_4** **aaatatttagttaccgagatatgattatgagttattaaatagtaaaatgaataaaaattc**

**Fortunella_japonica_5** **aaatatttagttaccgagatatgattatgagttattaaatagtaaaatgaataaaaattc**

**Fortunella_japonica_6** **aaatatttagttaccgagatatgattatgagttattaaatagtaaaatgaataaaaattc**

**Fortunella_japonica_7** **aaatatttagttaccgagatatgattatgagttattaaatagtaaaatgaataaaaattc**

**Fortunella_japonica_8** **aaatatttagttaccgagatatgattatgagttattaaatagtaaaatgaataaaaattc**

**Fortunella_japonica_9** **aaatatttagttaccgagatatgattatgagttattaaatagtaaaatgaataaaaattc**

**Fortunella_japonica_10** **aaatatttagttaccgagatatgattatgagttattaaatagtaaaatgaataaaaattc**

**Fortunella_japonica_11** **aaatatttagttaccgagatatgattatgagttattaaatagtaaaatgaataaaaattc**

**Fortunella_japonica_12** **aaatatttagttaccgagatatgattatgagttattaaatagtaaaatgaataaaaattc**

**Fortunella_margarita_1** **aaatatttagttaccgagatatgattatgagttattaaatagtaaaatgaataaaaattc**

**Fortunella_margarita_2** **aaatatttagttaccgagatatgattatgagttattaaatagtaaaatgaataaaaattc**

**Fortunella_venosa_1** **aaatatttagttaccgagatatgattatgagttattaaatagtaaaatgaataaaaattc**

**Fortunella_venosa_2** **aaatatttagttaccgagatatgattatgagttattaaatagtaaaatgaataaaaattc**

**Fortunella_venosa_3** **aaatatttagttaccgagatatgattatgagttattaaatagtaaaatgaataaaaattc**

19870	19880	19890	19900	19910	19920

....|....|....|....|....|....|....|....|....|....|....|....|

**Atalantia_kwangtungens** **gcaatttgaaggaatgttcctaagggtcccaacgaagtcttaattagagttagcgaatct**

**Citrus_reticulata_1** **gcaatttgaaggaatgttcctaagggtcccaacgaagtcttaattggagttagcgaatct**

**Citrus_reticulata_2** **gcaatttgaaggaatgttcctaagggtcccaacgaagtcttaattggagttagcgaatct**

**Citrus_trifoliata_3** **gcaatttgaaggaatgttcctaagggtcccaacgaagtcttaattggagttagcgaatct**

**Citrus_aurantiifolia** **gcaatttgaaggaatgttcctaagggtcccaacgaagtcttaattggagttagcgaatct**

**Citrus_aurantium** **gcaatttgaaggaatgttcctaagggtcccaacgaagtcttaattggagttagcgaatct**

**Citrus_cavaleriei** **gcaatttgaaggaatgttcctaagggtcccaacgaagtcttaattggagttagcgaatct**

**Citrus_hongheensis** **gcaatttgaaggaatgttcctaagggtcccaacgaagtcttaattggagttagcgaatct**

**Citrus_limon** **gcaatttgaaggaatgttcctaagggtcccaacgaagtcttaattggagttagcgaatct**

**Citrus_maxima** **gcaatttgaaggaatgttcctaagggtcccaacgaagtcttaattggagttagcgaatct**

**Citrus_medica** **gcaatttgaaggaatgttcctaagggtcccaacgaagtcttaattggagttagcgaatct**

**Citrus_polytrifolia** **gcaatttgaaggaatgttcctaagggtcccaacgaagtcttaattggagttagcgaatct**

**Citrus_reticulata** **gcaatttgaaggaatgttcctaagggtcccaacgaagtcttaattggagttagcgaatct**

**Citrus_sinensis** **gcaatttgaaggaatgttcctaagggtcccaacgaagtcttaattggagttagcgaatct**

**Fortunella_bawangica_1** **gcaatttgaaggaatgttcctaagggtcccaacgaagtcttaattggagttagcgaatct**

**Fortunella_bawangica_2** **gcaatttgaaggaatgttcctaagggtcccaacgaagtcttaattggagttagcgaatct**

**Fortunella_hindsii_1** **gcaatttgaaggaatgttcctaagggtcccaacgaagtcttaattggagttagcgaatct**

**Fortunella_hindsii_2** **gcaatttgaaggaatgttcctaagggtcccaacgaagtcttaattggagttagcgaatct**

**Fortunella_hindsii_3** **gcaatttgaaggaatgttcctaagggtcccaacgaagtcttaattggagttagcgaatct**

**Fortunella_japonica_1** **gcaatttgaaggaatgttcctaagggtcccaacgaagtcttaattggagttagcgaatct**

**Fortunella_japonica_2** **gcaatttgaaggaatgttcctaagggtcccaacgaagtcttaattggagttagcgaatct**

**Fortunella_japonica_3** **gcaatttgaaggaatgttcctaagggtcccaacgaagtcttaattggagttagcgaatct**

**Fortunella_japonica_4** **gcaatttgaaggaatgttcctaagggtcccaacgaagtcttaattggagttagcgaatct**

**Fortunella_japonica_5** **gcaatttgaaggaatgttcctaagggtcccaacgaagtcttaattggagttagcgaatct**

**Fortunella_japonica_6** **gcaatttgaaggaatgttcctaagggtcccaacgaagtcttaattggagttagcgaatct**

**Fortunella_japonica_7** **gcaatttgaaggaatgttcctaagggtcccaacgaagtcttaattggagttagcgaatct**

**Fortunella_japonica_8** **gcaatttgaaggaatgttcctaagggtcccaacgaagtcttaattggagttagcgaatct**

**Fortunella_japonica_9** **gcaatttgaaggaatgttcctaagggtcccaacgaagtcttaattggagttagcgaatct**

**Fortunella_japonica_10** **gcaatttgaaggaatgttcctaagggtcccaacgaagtcttaattggagttagcgaatct**

**Fortunella_japonica_11** **gcaatttgaaggaatgttcctaagggtcccaacgaagtcttaattggagttagcgaatct**

**Fortunella_japonica_12** **gcaatttgaaggaatgttcctaagggtcccaacgaagtcttaattggagttagcgaatct**

**Fortunella_margarita_1** **gcaatttgaaggaatgttcctaagggtcccaacgaagtcttaattggagttagcgaatct**

**Fortunella_margarita_2** **gcaatttgaaggaatgttcctaagggtcccaacgaagtcttaattggagttagcgaatct**

**Fortunella_venosa_1** **gcaatttgaaggaatgttcctaagggtcccaacgaagtcttaattggagttagcgaatct**

**Fortunella_venosa_2** **gcaatttgaaggaatgttcctaagggtcccaacgaagtcttaattggagttagcgaatct**

**Fortunella_venosa_3** **gcaatttgaaggaatgttcctaagggtcccaacgaagtcttaattggagttagcgaatct**

19930	19940	19950	19960	19970	19980

....|....|....|....|....|....|....|....|....|....|....|....|

**Atalantia_kwangtungens** **ttttgaattggaattgattgttttatcacattgtgatattttacatcgttcaatggaccc**

**Citrus_reticulata_1** **tttttaattggaattgattgttttatcacattgtgatattttacatcgttcaatggaccc**

**Citrus_reticulata_2** **tttttaattggaattgattgttttatcacattgtgatattttacatcgttcaatggaccc**

**Citrus_trifoliata_3** **ttttgaattggaattgattgttttatcacattgtgatatttgacatcgttcaatggaccc**

**Citrus_aurantiifolia** **ttttgaattggaattgattgttttatcacattgtgatattttacatcgttcaatggaccc**

**Citrus_aurantium** **ttttgaattggaattgattgttttatcacattgtgatattttacatcgttcaatggaccc**

**Citrus_cavaleriei** **ttttgaattggaattgattgttttatcacattgtgatattttacatcgttcaatggaccc**

**Citrus_hongheensis** **ttttgaattggaattgattgttttatcacattgtgatattttacatcgttcaatggaccc**

**Citrus_limon** **ttttgaattggaattgattgttttatcacattgtgatattttacatcgttcaatggaccc**

**Citrus_maxima** **ttttgaattggaattgattgttttatcacattgtgatattttacatcgttcaatggaccc**

**Citrus_medica** **ttttgaattggaattgattgttttatcacattgtgatattttacatcgttcaatggaccc**

**Citrus_polytrifolia** **ttttgaattggaattgattgttttatcacattgtgatatttgacatcgttcaatggaccc**

**Citrus_reticulata** **tttttaattggaattgattgttttatcacattgtgatattttacatcgttcaatggaccc**

**Citrus_sinensis** **ttttgaattggaattgattgttttatcacattgtgatattttacatcgttcaatggaccc**

**Fortunella_bawangica_1** **ttttgaattggaattgattgttttatcacattgtgatattttacatcgttcaatggaccc**

**Fortunella_bawangica_2** **ttttgaattggaattgattgttttatcacattgtgatattttacatcgttcaatggaccc**

**Fortunella_hindsii_1** **ttttgaattggaattgattgttttatcacattgtgatattttacatcgttcaatggaccc**

**Fortunella_hindsii_2** **ttttgaattggaattgattgttttatcacattgtgatattttacatcgttcaatggaccc**

**Fortunella_hindsii_3** **ttttgaattggaattgattgttttatcacattgtgatattttacatcgttcaatggaccc**

**Fortunella_japonica_1** **ttttgaattggaattgattgttttatcacattgtgatattttacatcgttcaatggaccc**

**Fortunella_japonica_2** **ttttgaattggaattgattgttttatcacattgtgatattttacatcgttcaatggaccc**

**Fortunella_japonica_3** **ttttgaattggaattgattgttttatcacattgtgatattttacatcgttcaatggaccc**

**Fortunella_japonica_4** **ttttgaattggaattgattgttttatcacattgtgatattttacatcgttcaatggaccc**

**Fortunella_japonica_5** **ttttgaattggaattgattgttttatcacattgtgatattttacatcgttcaatggaccc**

**Fortunella_japonica_6** **ttttgaattggaattgattgttttatcacattgtgatattttacatcgttcaatggaccc**

**Fortunella_japonica_7** **ttttgaattggaattgattgttttatcacattgtgatattttacatcgttcaatggaccc**

**Fortunella_japonica_8** **ttttgaattggaattgattgttttatcacattgtgatattttacatcgttcaatggaccc**

**Fortunella_japonica_9** **ttttgaattggaattgattgttttatcacattgtgatattttacatcgttcaatggaccc**

**Fortunella_japonica_10** **ttttgaattggaattgattgttttatcacattgtgatattttacatcgttcaatggaccc**

**Fortunella_japonica_11** **ttttgaattggaattgattgttttatcacattgtgatattttacatcgttcaatggaccc**

**Fortunella_japonica_12** **ttttgaattggaattgattgttttatcacattgtgatattttacatcgttcaatggaccc**

**Fortunella_margarita_1** **ttttgaattggaattgattgttttatcacattgtgatattttacatcgttcaatggaccc**

**Fortunella_margarita_2** **ttttgaattggaattgattgttttatcacattgtgatattttacatcgttcaatggaccc**

**Fortunella_venosa_1** **ttttgaattggaattgattgttttatcacattgtgatattttacatcgttcaatggaccc**

**Fortunella_venosa_2** **ttttgaattggaattgattgttttatcacattgtgatattttacatcgttcaatggaccc**

**Fortunella_venosa_3** **ttttgaattggaattgattgttttatcacattgtgatattttacatcgttcaatggaccc**

19990	20000	20010	20020	20030	20040

....|....|....|....|....|....|....|....|....|....|....|....|

**Atalantia_kwangtungens** **attcgaaaataattagatgatgataaaattatcaaagattggcattccttatttttattc**

**Citrus_reticulata_1** **attcgaaaataattagatgatgataaaattatcaaaacttggcattccttatttttattc**

**Citrus_reticulata_2** **attcgaaaataattagatgatgataaaattatcaaaacttggcattccttatttttattc**

**Citrus_trifoliata_3** **attcgaaaataattagatgatgataaaattatcaaaacttggcattccttatttttattc**

**Citrus_aurantiifolia** **attcgaaaataattagatgatgataaaattatcaaaacttggcattccttatttttattc**

**Citrus_aurantium** **attcgaaaataattagatgatgataaaattatcaaaacttggcattccttatttttattc**

**Citrus_cavaleriei** **attcgaaaataattagatgatgataaaattatcaaaacttggcattccttatttttattc**

**Citrus_hongheensis** **attcgaaaataattagatgatgataaaattatcaaaacttggcattccttatttttattc**

**Citrus_limon** **attcgaaaataattagatgatgataaaattatcaaaacttggcattccttatttttattc**

**Citrus_maxima** **attcgaaaataattagatgatgataaaattatcaaaacttggcattccttatttttattc**

**Citrus_medica** **attcgaaaataattagatgatgataaaattatcaaaacttggcattccttatttttattc**

**Citrus_polytrifolia** **attcgaaaataattagatgatgataaaattatcaaaacttggcattccttatttttattc**

**Citrus_reticulata** **attcgaaaataattagatgatgataaaattatcaaaacttggcattccttatttttattc**

**Citrus_sinensis** **attcgaaaataattagatgatgataaaattatcaaaacttggcattccttatttttattc**

**Fortunella_bawangica_1** **attcgaaaataattagatgatgataaaattatcaaaacttggcattccttatttttattc**

**Fortunella_bawangica_2** **attcgaaaataattagatgatgataaaattatcaaaacttggcattccttatttttattc**

**Fortunella_hindsii_1** **attcgaaaataattagatgatgataaaattatcaaaacttggcattccttatttttattc**

**Fortunella_hindsii_2** **attcgaaaataattagatgatgataaaattatcaaaacttggcattccttatttttattc**

**Fortunella_hindsii_3** **attcgaaaataattagatgatgataaaattatcaaaacttggcattccttatttttattc**

**Fortunella_japonica_1** **attcgaaaataattagatgatgataaaattatcaaaacttggcattccttatttttattc**

**Fortunella_japonica_2** **attcgaaaataattagatgatgataaaattatcaaaacttggcattccttatttttattc**

**Fortunella_japonica_3** **attcgaaaataattagatgatgataaaattatcaaaacttggcattccttatttttattc**

**Fortunella_japonica_4** **attcgaaaataattagatgatgataaaattatcaaaacttggcattccttatttttattc**

**Fortunella_japonica_5** **attcgaaaataattagatgatgataaaattatcaaaacttggcattccttatttttattc**

**Fortunella_japonica_6** **attcgaaaataattagatgatgataaaattatcaaaacttggcattccttatttttattc**

**Fortunella_japonica_7** **attcgaaaataattagatgatgataaaattatcaaaacttggcattccttatttttattc**

**Fortunella_japonica_8** **attcgaaaataattagatgatgataaaattatcaaaacttggcattccttatttttattc**

**Fortunella_japonica_9** **attcgaaaataattagatgatgataaaattatcaaaacttggcattccttatttttattc**

**Fortunella_japonica_10** **attcgaaaataattagatgatgataaaattatcaaaacttggcattccttatttttattc**

**Fortunella_japonica_11** **attcgaaaataattagatgatgataaaattatcaaaacttggcattccttatttttattc**

**Fortunella_japonica_12** **attcgaaaataattagatgatgataaaattatcaaaacttggcattccttatttttattc**

**Fortunella_margarita_1** **attcgaaaataattagatgatgataaaattatcaaaacttggcattccttatttttattc**

**Fortunella_margarita_2** **attcgaaaataattagatgatgataaaattatcaaaacttggcattccttatttttattc**

**Fortunella_venosa_1** **attcgaaaataattagatgatgataaaattatcaaaacttggcattccttatttttattc**

**Fortunella_venosa_2** **attcgaaaataattagatgatgataaaattatcaaaacttggcattccttatttttattc**

**Fortunella_venosa_3** **attcgaaaataattagatgatgataaaattatcaaaacttggcattccttatttttattc**

20050	20060	20070	20080	20090	20100

....|....|....|....|....|....|....|....|....|....|....|....|

**Atalantia_kwangtungens** **aacaacgtacgaatagttccttgattttgtctaagcgattgttcaacccctgccttgcca**

**Citrus_reticulata_1** **aacaacgtacgaatagttccttgattttgtctaagcgattgttcaacccctgccttgcca**

**Citrus_reticulata_2** **aacaacgtacgaatagttccttgattttgtctaagcgattgttcaacccctgccttgcca**

**Citrus_trifoliata_3** **aacaacgtacgaatagttccttgattttgtctaagcgattgttcaacccctgccttgcca**

**Citrus_aurantiifolia** **aacaacgtacgaatagttccttgattttgtctaagcgattgttcaacccctgccttacca**

**Citrus_aurantium** **aacaacgtacgaatagttccttgattttgtctaagcgattgttcaacccctgccttgcca**

**Citrus_cavaleriei** **aacaacgtacgaatagttccttgattttgtctaagcgattgttcaacccctgccttgcca**

**Citrus_hongheensis** **aacaacgtacgaatagttccttgattttgtctaagcgattgttcaacccctgccttgcca**

**Citrus_limon** **aacaacgtacgaatagttccttgattttgtctaagcgattgttcaacccctgccttgcca**

**Citrus_maxima** **aacaacgtacgaatagttccttgattttgtctaagcgattgttcaacccctgccttgcca**

**Citrus_medica** **aacaacgtacgaatagttccttgattttgtctaagcgattgttcaacccctgccttgcca**

**Citrus_polytrifolia** **aacaacgtacgaatagttccttgattttgtctaagcgattgttcaacccctgccttgcca**

**Citrus_reticulata** **aacaacgtacgaatagttccttgattttgtctaagcgattgttcaacccctgccttgcca**

**Citrus_sinensis** **aacaacgtacgaatagttccttgattttgtctaagcgattgttcaacccctgccttgcca**

**Fortunella_bawangica_1** **aacaacgtacgaatagttccttgattttgtctaagcgattgttcaacccctgccttgcca**

**Fortunella_bawangica_2** **aacaacgtacgaatagttccttgattttgtctaagcgattgttcaacccctgccttgcca**

**Fortunella_hindsii_1** **aacaacgtacgaatagttccttgattttgtctaagcgattgttcaacccctgccttgcca**

**Fortunella_hindsii_2** **aacaacgtacgaatagttccttgattttgtctaagcgattgttcaacccctgccttgcca**

**Fortunella_hindsii_3** **aacaacgtacgaatagttccttgattttgtctaagcgattgttcaacccctgccttgcca**

**Fortunella_japonica_1** **aacaacgtacgaatagttccttgattttgtctaagcgattgttcaacccctgccttgcca**

**Fortunella_japonica_2** **aacaacgtacgaatagttccttgattttgtctaagcgattgttcaacccctgccttgcca**

**Fortunella_japonica_3** **aacaacgtacgaatagttccttgattttgtctaagcgattgttcaacccctgccttgcca**

**Fortunella_japonica_4** **aacaacgtacgaatagttccttgattttgtctaagcgattgttcaacccctgccttgcca**

**Fortunella_japonica_5** **aacaacgtacgaatagttccttgattttgtctaagcgattgttcaacccctgccttgcca**

**Fortunella_japonica_6** **aacaacgtacgaatagttccttgattttgtctaagcgattgttcaacccctgccttgcca**

**Fortunella_japonica_7** **aacaacgtacgaatagttccttgattttgtctaagcgattgttcaacccctgccttgcca**

**Fortunella_japonica_8** **aacaacgtacgaatagttccttgattttgtctaagcgattgttcaacccctgccttgcca**

**Fortunella_japonica_9** **aacaacgtacgaatagttccttgattttgtctaagcgattgttcaacccctgccttgcca**

**Fortunella_japonica_10** **aacaacgtacgaatagttccttgattttgtctaagcgattgttcaacccctgccttgcca**

**Fortunella_japonica_11** **aacaacgtacgaatagttccttgattttgtctaagcgattgttcaacccctgccttgcca**

**Fortunella_japonica_12** **aacaacgtacgaatagttccttgattttgtctaagcgattgttcaacccctgccttgcca**

**Fortunella_margarita_1** **aacaacgtacgaatagttccttgattttgtctaagcgattgttcaacccctgccttgcca**

**Fortunella_margarita_2** **aacaacgtacgaatagttccttgattttgtctaagcgattgttcaacccctgccttgcca**

**Fortunella_venosa_1** **aacaacgtacgaatagttccttgattttgtctaagcgattgttcaacccctgccttgcca**

**Fortunella_venosa_2** **aacaacgtacgaatagttccttgattttgtctaagcgattgttcaacccctgccttgcca**

**Fortunella_venosa_3** **aacaacgtacgaatagttccttgattttgtctaagcgattgttcaacccctgccttgcca**

20110	20120	20130	20140	20150	20160

....|....|....|....|....|....|....|....|....|....|....|....|

**Atalantia_kwangtungens** **aaaacggaataaaacggattgctattggtgcgagctgaaccattatcagaaattaatcct**

**Citrus_reticulata_1** **aacacggaataaaacggattgctattggtgcgagctgaaccattatcagaaattaatcct**

**Citrus_reticulata_2** **aacacggaataaaacggattgctattggtgcgagctgaaccattatcagaaattaatcct**

**Citrus_trifoliata_3** **aacacggaataaaacggattgctattggtgcgagctgaaccattatcagaaattaatcct**

**Citrus_aurantiifolia** **aacacggaataaaacggattgctattggtgcgagctgaaccattatcagaaattaatcct**

**Citrus_aurantium** **aacacggaataaaacggattgctattggtgcgagctgaaccattatcagaaattaatcct**

**Citrus_cavaleriei** **aacacggaataaaacggattgctattggtgcgagctgaaccattatcagaaattaatcct**

**Citrus_hongheensis** **aacacggaataaaacggattgctattggtgcgagctgaaccattatcagaaattaatcct**

**Citrus_limon** **aacacggaataaaacggattgctattggtgcgagctgaaccattatcagaaattaatcct**

**Citrus_maxima** **aacacggaataaaacggattgctattggtgcgagctgaaccattatcagaaattaatcct**

**Citrus_medica** **aaaacggaataaaacggattgctattggtgcgagctgaaccattatcagaaattaatcct**

**Citrus_polytrifolia** **aacacggaataaaacggattgctattggtgcgagctgaaccattatcagaaattaatcct**

**Citrus_reticulata** **aacacggaataaaacggattgctattggtgcgagctgaaccattatcagaaattaatcct**

**Citrus_sinensis** **aacacggaataaaacggattgctattggtgcgagctgaaccattatcagaaattaatcct**

**Fortunella_bawangica_1** **aacacggaataaaacggattgctattgatgcgagctgaaccattatcagaaattaatcct**

**Fortunella_bawangica_2** **aacacggaataaaacggattgctattgatgcgagctgaaccattatcagaaattaatcct**

**Fortunella_hindsii_1** **aacacggaataaaacggattgctattgatgcgagctgaaccattatcagaaattaatcct**

**Fortunella_hindsii_2** **aacacggaataaaacggattgctattgatgcgagctgaaccattatcagaaattaatcct**

**Fortunella_hindsii_3** **aacacggaataaaacggattgctattgatgcgagctgaaccattatcagaaattaatcct**

**Fortunella_japonica_1** **aacacggaataaaacggattgctattgatgcgagctgaaccattatcagaaattaatcct**

**Fortunella_japonica_2** **aacacggaataaaacggattgctattgatgcgagctgaaccattatcagaaattaatcct**

**Fortunella_japonica_3** **aacacggaataaaacggattgctattgatgcgagctgaaccattatcagaaattaatcct**

**Fortunella_japonica_4** **aacacggaataaaacggattgctattgatgcgagctgaaccattatcagaaattaatcct**

**Fortunella_japonica_5** **aacacggaataaaacggattgctattgatgcgagctgaaccattatcagaaattaatcct**

**Fortunella_japonica_6** **aacacggaataaaacggattgctattgatgcgagctgaaccattatcagaaattaatcct**

**Fortunella_japonica_7** **aacacggaataaaacggattgctattgatgcgagctgaaccattatcagaaattaatcct**

**Fortunella_japonica_8** **aacacggaataaaacggattgctattgatgcgagctgaaccattatcagaaattaatcct**

**Fortunella_japonica_9** **aacacggaataaaacggattgctattgatgcgagctgaaccattatcagaaattaatcct**

**Fortunella_japonica_10** **aacacggaataaaacggattgctattgatgcgagctgaaccattatcagaaattaatcct**

**Fortunella_japonica_11** **aacacggaataaaacggattgctattgatgcgagctgaaccattatcagaaattaatcct**

**Fortunella_japonica_12** **aacacggaataaaacggattgctattgatgcgagctgaaccattatcagaaattaatcct**

**Fortunella_margarita_1** **aacacggaataaaacggattgctattgatgcgagctgaaccattatcagaaattaatcct**

**Fortunella_margarita_2** **aacacggaataaaacggattgctattgatgcgagctgaaccattatcagaaattaatcct**

**Fortunella_venosa_1** **aacacggaataaaacggattgctattgatgcgagctgaaccattatcagaaattaatcct**

**Fortunella_venosa_2** **aacacggaataaaacggattgctattgatgcgagctgaaccattatcagaaattaatcct**

**Fortunella_venosa_3** **aacacggaataaaacggattgctattgatgcgagctgaaccattatcagaaattaatcct**

20170	20180	20190	20200	20210	20220

....|....|....|....|....|....|....|....|....|....|....|....|

**Atalantia_kwangtungens** **gaacccgacggatgatccctttttctgagatacgaaatattggatttcactaagttgatt**

**Citrus_reticulata_1** **gaacccgacggatgatccctttttctgagatacgaaatattggatttcactaagttgatt**

**Citrus_reticulata_2** **gaacccgacggatgatccctttttctgagatacgaaatattggatttcactaagttgatt**

**Citrus_trifoliata_3** **gaacccgacggatgatccctttttctgagatacgaaatattggatttcactaagttgatt**

**Citrus_aurantiifolia** **gaacccgacggatgatccctttttctgagatacgaaatattggatttcactaagtggatt**

**Citrus_aurantium** **gaacccgacggatgatccctttttctgagatacgaaatattggatttcactaagttgatt**

**Citrus_cavaleriei** **gaacccgacggatgatccctttttctgagatacgaaatattggatttcactaagttgatt**

**Citrus_hongheensis** **gaacccgacggatgatccctttttctgagatacgaaatattggatttcactaagttgatt**

**Citrus_limon** **gaacccgacggatgatccctttttctgagatacgaaatattggatttcactaagttgatt**

**Citrus_maxima** **gaacccgacggatgatccctttttctgagatacgaaatattggatttcactaagttgatt**

**Citrus_medica** **gaacccgacggatgatccctttttctgagatacgaaatattggatttcactaagttgatt**

**Citrus_polytrifolia** **gaacccgacggatgatccctttttctgagatacgaaatattggatttcactaagttgatt**

**Citrus_reticulata** **gaacccgacggatgatccctttttctgagatacgaaatattggatttcactaagttgatt**

**Citrus_sinensis** **gaacccgacggatgatccctttttctgagatacgaaatattggatttcactaagttgatt**

**Fortunella_bawangica_1** **gaacccgacggatgatccctttttctgagatacgaaatattggatttcactaagttgatt**

**Fortunella_bawangica_2** **gaacccgacggatgatccctttttctgagatacgaaatattggatttcactaagttgatt**

**Fortunella_hindsii_1** **gaacccgacggatgatccctttttctgagatacgaaatattggatttcactaagttgatt**

**Fortunella_hindsii_2** **gaacccgacggatgatccctttttctgagatacgaaatattggatttcactaagttgatt**

**Fortunella_hindsii_3** **gaacccgacggatgatccctttttctgagatacgaaatattggatttcactaagttgatt**

**Fortunella_japonica_1** **gaacccgacggatgatccctttttctgagatacgaaatattggatttcactaagttgatt**

**Fortunella_japonica_2** **gaacccgacggatgatccctttttctgagatacgaaatattggatttcactaagttgatt**

**Fortunella_japonica_3** **gaacccgacggatgatccctttttctgagatacgaaatattggatttcactaagttgatt**

**Fortunella_japonica_4** **gaacccgacggatgatccctttttctgagatacgaaatattggatttcactaagttgatt**

**Fortunella_japonica_5** **gaacccgacggatgatccctttttctgagatacgaaatattggatttcactaagttgatt**

**Fortunella_japonica_6** **gaacccgacggatgatccctttttctgagatacgaaatattggatttcactaagttgatt**

**Fortunella_japonica_7** **gaacccgacggatgatccctttttctgagatacgaaatattggatttcactaagttgatt**

**Fortunella_japonica_8** **gaacccgacggatgatccctttttctgagatacgaaatattggatttcactaagttgatt**

**Fortunella_japonica_9** **gaacccgacggatgatccctttttctgagatacgaaatattggatttcactaagttgatt**

**Fortunella_japonica_10** **gaacccgacggatgatccctttttctgagatacgaaatattggatttcactaagttgatt**

**Fortunella_japonica_11** **gaacccgacggatgatccctttttctgagatacgaaatattggatttcactaagttgatt**

**Fortunella_japonica_12** **gaacccgacggatgatccctttttctgagatacgaaatattggatttcactaagttgatt**

**Fortunella_margarita_1** **gaacccgacggatgatccctttttctgagatacgaaatattggatttcactaagttgatt**

**Fortunella_margarita_2** **gaacccgacggatgatccctttttctgagatacgaaatattggatttcactaagttgatt**

**Fortunella_venosa_1** **gaacccgacggatgatccctttttctgagatacgaaatattggatttcactaagttgatt**

**Fortunella_venosa_2** **gaacccgacggatgatccctttttctgagatacgaaatattggatttcactaagttgatt**

**Fortunella_venosa_3** **gaacccgacggatgatccctttttctgagatacgaaatattggatttcactaagttgatt**

20230	20240	20250	20260	20270	20280

....|....|....|....|....|....|....|....|....|....|....|....|

**Atalantia_kwangtungens** **cttaggaaatctcgaatcagaccatttgtacgtacttcaacaaaggaagcccaaacctct**

**Citrus_reticulata_1** **cttaggaaatctcgaatcagaccatttgtacgtacttcaacaaaggaagcacaaacctct**

**Citrus_reticulata_2** **cttaggaaatctcgaatcagaccatttgtacgtacttcaacaaaggaagcacaaacctct**

**Citrus_trifoliata_3** **cttaggaaatctcgaatcagaccatttgtacgtacttcaacaaaggaagcacaaacctct**

**Citrus_aurantiifolia** **cttaggaaatctcgaatcagaccatttgtacgtacttcaacaaaggaagcacaaacctct**

**Citrus_aurantium** **cttaggaaatttcgaatcagaccatttgtacgtacttcaacaaaggaagcacaaacctct**

**Citrus_cavaleriei** **cttaggaaatctcgaatcagaccatttgtacgtacttcaacaaaggaagcacaaacctct**

**Citrus_hongheensis** **cttaggaaatttcgaatcagaccatttgtacgtacttcaacaaaggaagcacaaacctct**

**Citrus_limon** **cttaggaaatttcgaatcagaccatttgtacgtacttcaacaaaggaagcacaaacctct**

**Citrus_maxima** **cttaggaaatttcgaatcagaccatttgtacgtacttcaacaaaggaagcacaaacctct**

**Citrus_medica** **cttaggaaatctcgaatcagaccatttgtacgtacttcaacaaaggaagcacaaacctct**

**Citrus_polytrifolia** **cttaggaaatctcgaatcagaccatttgtacgtacttcaacaaaggaagcacaaacctct**

**Citrus_reticulata** **cttaggaaatctcgaatcagaccatttgtacgtacttcaacaaaggaagcacaaacctct**

**Citrus_sinensis** **cttaggaaatttcgaatcagaccatttgtacgtacttcaacaaaggaagcacaaacctct**

**Fortunella_bawangica_1** **cttaggaaatctcgaatcagaccatttgtacgtacttcaacaaaggaagcacaaacctct**

**Fortunella_bawangica_2** **cttaggaaatctcgaatcagaccatttgtacgtacttcaacaaaggaagcacaaacctct**

**Fortunella_hindsii_1** **cttaggaaatctcgaatcagaccatttgtacgtacttcaacaaaggaagcacaaacctct**

**Fortunella_hindsii_2** **cttaggaaatctcgaatcagaccatttgtacgtacttcaacaaaggaagcacaaacctct**

**Fortunella_hindsii_3** **cttaggaaatctcgaatcagaccatttgtacgtacttcaacaaaggaagcacaaacctct**

**Fortunella_japonica_1** **cttaggaaatctcgaatcagaccatttgtacgtacttcaacaaaggaagcacaaacctct**

**Fortunella_japonica_2** **cttaggaaatctcgaatcagaccatttgtacgtacttcaacaaaggaagcacaaacctct**

**Fortunella_japonica_3** **cttaggaaatctcgaatcagaccatttgtacgtacttcaacaaaggaagcacaaacctct**

**Fortunella_japonica_4** **cttaggaaatctcgaatcagaccatttgtacgtacttcaacaaaggaagcacaaacctct**

**Fortunella_japonica_5** **cttaggaaatctcgaatcagaccatttgtacgtacttcaacaaaggaagcacaaacctct**

**Fortunella_japonica_6** **cttaggaaatctcgaatcagaccatttgtacgtacttcaacaaaggaagcacaaacctct**

**Fortunella_japonica_7** **cttaggaaatctcgaatcagaccatttgtacgtacttcaacaaaggaagcacaaacctct**

**Fortunella_japonica_8** **cttaggaaatctcgaatcagaccatttgtacgtacttcaacaaaggaagcacaaacctct**

**Fortunella_japonica_9** **cttaggaaatctcgaatcagaccatttgtacgtacttcaacaaaggaagcacaaacctct**

**Fortunella_japonica_10** **cttaggaaatctcgaatcagaccatttgtacgtacttcaacaaaggaagcacaaacctct**

**Fortunella_japonica_11** **cttaggaaatctcgaatcagaccatttgtacgtacttcaacaaaggaagcacaaacctct**

**Fortunella_japonica_12** **cttaggaaatctcgaatcagaccatttgtacgtacttcaacaaaggaagcacaaacctct**

**Fortunella_margarita_1** **cttaggaaatctcgaatcagaccatttgtacgtacttcaacaaaggaagcacaaacctct**

**Fortunella_margarita_2** **cttaggaaatctcgaatcagaccatttgtacgtacttcaacaaaggaagcacaaacctct**

**Fortunella_venosa_1** **cttaggaaatctcgaatcagaccatttgtacgtacttcaacaaaggaagcacaaacctct**

**Fortunella_venosa_2** **cttaggaaatctcgaatcagaccatttgtacgtacttcaacaaaggaagcacaaacctct**

**Fortunella_venosa_3** **cttaggaaatctcgaatcagaccatttgtacgtacttcaacaaaggaagcacaaacctct**

20290	20300	20310	20320	20330	20340

....|....|....|....|....|....|....|....|....|....|....|....|

**Atalantia_kwangtungens** **tcgacggaagaacttttgttgtcttggtcccaattcaacactaaacacgtccgaactaat**

**Citrus_reticulata_1** **tcgacggaagaacttttgttgtcttggtcccaattcaacactaaacacgtccgaactaat**

**Citrus_reticulata_2** **tcgacggaagaacttttgttgtcttggtcccaattcaacactaaacacgtccgaactaat**

**Citrus_trifoliata_3** **tcgacggaagaacttttgttgtcttggtcccaattcaacactaaacacgtccgaactaat**

**Citrus_aurantiifolia** **tcgacggaagaacttttgttgtcttggtcccaattcaacactaaacacgtccgaactaat**

**Citrus_aurantium** **tcgacggaagaacttttgttgtcttggtcccaattcaacactaaacacgtccgaactaat**

**Citrus_cavaleriei** **tcgacggaagaacttttgttgtcttggtcccaattcaacactaaacacgtccgaactaat**

**Citrus_hongheensis** **tcgacggaagaacttttgttgtcttggtcccaattcaacactaaacacgtccgaactaat**

**Citrus_limon** **tcgacggaagaacttttgttgtcttggtcccaattcaacactaaacacgtccgaactaat**

**Citrus_maxima** **tcgacggaagaacttttgttgtcttggtcccaattcaacactaaacacgtccgaactaat**

**Citrus_medica** **tcgacggaagaacttttgttgtcttggtcccaattcaacactaaacacgtccgaactaat**

**Citrus_polytrifolia** **tcgacggaagaacttttgttgtcttggtcccaattcaacactaaacacgtccgaactaat**

**Citrus_reticulata** **tcgacggaagaacttttgttgtcttggtcccaattcaacactaaacacgtccgaactaat**

**Citrus_sinensis** **tcgacggaagaacttttgttgtcttggtcccaattcaacactaaacacgtccgaactaat**

**Fortunella_bawangica_1** **tcgacggaagaacttttgttgtcttggtcccaattcaacactaaacacgtccgaactaat**

**Fortunella_bawangica_2** **tcgacggaagaacttttgttgtcttggtcccaattcaacactaaacacgtccgaactaat**

**Fortunella_hindsii_1** **tcgacggaagaacttttgttgtcttggtcccaattcaacactaaacacgtccgaactaat**

**Fortunella_hindsii_2** **tcgacggaagaacttttgttgtcttggtcccaattcaacactaaacacgtccgaactaat**

**Fortunella_hindsii_3** **tcgacggaagaacttttgttgtcttggtcccaattcaacactaaacacgtccgaactaat**

**Fortunella_japonica_1** **tcgacggaagaacttttgttgtcttggtcccaattcaacactaaacacgtccgaactaat**

**Fortunella_japonica_2** **tcgacggaagaacttttgttgtcttggtcccaattcaacactaaacacgtccgaactaat**

**Fortunella_japonica_3** **tcgacggaagaacttttgttgtcttggtcccaattcaacactaaacacgtccgaactaat**

**Fortunella_japonica_4** **tcgacggaagaacttttgttgtcttggtcccaattcaacactaaacacgtccgaactaat**

**Fortunella_japonica_5** **tcgacggaagaacttttgttgtcttggtcccaattcaacactaaacacgtccgaactaat**

**Fortunella_japonica_6** **tcgacggaagaacttttgttgtcttggtcccaattcaacactaaacacgtccgaactaat**

**Fortunella_japonica_7** **tcgacggaagaacttttgttgtcttggtcccaattcaacactaaacacgtccgaactaat**

**Fortunella_japonica_8** **tcgacggaagaacttttgttgtcttggtcccaattcaacactaaacacgtccgaactaat**

**Fortunella_japonica_9** **tcgacggaagaacttttgttgtcttggtcccaattcaacactaaacacgtccgaactaat**

**Fortunella_japonica_10** **tcgacggaagaacttttgttgtcttggtcccaattcaacactaaacacgtccgaactaat**

**Fortunella_japonica_11** **tcgacggaagaacttttgttgtcttggtcccaattcaacactaaacacgtccgaactaat**

**Fortunella_japonica_12** **tcgacggaagaacttttgttgtcttggtcccaattcaacactaaacacgtccgaactaat**

**Fortunella_margarita_1** **tcgacggaagaacttttgttgtcttggtcccaattcaacactaaacacgtccgaactaat**

**Fortunella_margarita_2** **tcgacggaagaacttttgttgtcttggtcccaattcaacactaaacacgtccgaactaat**

**Fortunella_venosa_1** **tcgacggaagaacttttgttgtcttggtcccaattcaacactaaacacgtccgaactaat**

**Fortunella_venosa_2** **tcgacggaagaacttttgttgtcttggtcccaattcaacactaaacacgtccgaactaat**

**Fortunella_venosa_3** **tcgacggaagaacttttgttgtcttggtcccaattcaacactaaacacgtccgaactaat**

20350	20360	20370	20380	20390	20400

....|....|....|....|....|....|....|....|....|....|....|....|

**Atalantia_kwangtungens** **tgaagacttgtatcagaaattcccccagcggctttgcccttcccataaaggacataattg**

**Citrus_reticulata_1** **tgaagacttgtatcagaaattcccccagcggctttgcccttcccataaaggacataattg**

**Citrus_reticulata_2** **tgaagacttgtatcagaaattcccccagcggctttgcccttcccataaaggacataattg**

**Citrus_trifoliata_3** **tgaagacttgtatcagaaattcccccagcggctttgcccttcccataaaggacataattg**

**Citrus_aurantiifolia** **tgaagacttgtatcagaaattcccccagcggctttgcccttcccataaaggacataattg**

**Citrus_aurantium** **tgaagacttgtatcagaaattcccccagcggctttgcccttcccataaaggacataattg**

**Citrus_cavaleriei** **tgaagacttgtatcagaaattcccccagcggctttgcccttcccataaaggacataattg**

**Citrus_hongheensis** **tgaagacttgtatcagaaattcccccagcggctttgcccttcccataaaggacataattg**

**Citrus_limon** **tgaagacttgtatcagaaattcccccagcggctttgcccttcccataaaggacataattg**

**Citrus_maxima** **tgaagacttgtatcagaaattcccccagcggctttgcccttcccataaaggacataattg**

**Citrus_medica** **tgaagacttgtatcagaaattcccccagcggctttgcccttcccataaaggacataattg**

**Citrus_polytrifolia** **tgaagacttgtatcagaaattcccccagcggctttgcccttcccataaaggacataattg**

**Citrus_reticulata** **tgaagacttgtatcagaaattcccccagcggctttgcccttcccataaaggacataattg**

**Citrus_sinensis** **tgaagacttgtatcagaaattcccccagcggctttgcccttcccataaaggacataattg**

**Fortunella_bawangica_1** **tgaagacttgtatcagaaattcccccagcggctttgcccttcccataaaggacataattg**

**Fortunella_bawangica_2** **tgaagacttgtatcagaaattcccccagcggctttgcccttcccataaaggacataattg**

**Fortunella_hindsii_1** **tgaagacttgtatcagaaattcccccagcggctttgcccttcccataaaggacataattg**

**Fortunella_hindsii_2** **tgaagacttgtatcagaaattcccccagcggctttgcccttcccataaaggacataattg**

**Fortunella_hindsii_3** **tgaagacttgtatcagaaattcccccagcggctttgcccttcccataaaggacataattg**

**Fortunella_japonica_1** **tgaagacttgtatcagaaattcccccagcggctttgcccttcccataaaggacataattg**

**Fortunella_japonica_2** **tgaagacttgtatcagaaattcccccagcggctttgcccttcccataaaggacataattg**

**Fortunella_japonica_3** **tgaagacttgtatcagaaattcccccagcggctttgcccttcccataaaggacataattg**

**Fortunella_japonica_4** **tgaagacttgtatcagaaattcccccagcggctttgcccttcccataaaggacataattg**

**Fortunella_japonica_5** **tgaagacttgtatcagaaattcccccagcggctttgcccttcccataaaggacataattg**

**Fortunella_japonica_6** **tgaagacttgtatcagaaattcccccagcggctttgcccttcccataaaggacataattg**

**Fortunella_japonica_7** **tgaagacttgtatcagaaattcccccagcggctttgcccttcccataaaggacataattg**

**Fortunella_japonica_8** **tgaagacttgtatcagaaattcccccagcggctttgcccttcccataaaggacataattg**

**Fortunella_japonica_9** **tgaagacttgtatcagaaattcccccagcggctttgcccttcccataaaggacataattg**

**Fortunella_japonica_10** **tgaagacttgtatcagaaattcccccagcggctttgcccttcccataaaggacataattg**

**Fortunella_japonica_11** **tgaagacttgtatcagaaattcccccagcggctttgcccttcccataaaggacataattg**

**Fortunella_japonica_12** **tgaagacttgtatcagaaattcccccagcggctttgcccttcccataaaggacataattg**

**Fortunella_margarita_1** **tgaagacttgtatcagaaattcccccagcggctttgcccttcccataaaggacataattg**

**Fortunella_margarita_2** **tgaagacttgtatcagaaattcccccagcggctttgcccttcccataaaggacataattg**

**Fortunella_venosa_1** **tgaagacttgtatcagaaattcccccagcggctttgcccttcccataaaggacataattg**

**Fortunella_venosa_2** **tgaagacttgtatcagaaattcccccagcggctttgcccttcccataaaggacataattg**

**Fortunella_venosa_3** **tgaagacttgtatcagaaattcccccagcggctttgcccttcccataaaggacataattg**

20410	20420	20430	20440	20450	20460

....|....|....|....|....|....|....|....|....|....|....|....|

**Atalantia_kwangtungens** **acaactcgaaattgcatattatccttttcccgcagcggatcctgggggaagagtgttgct**

**Citrus_reticulata_1** **acaactcgaaattgcatattatccttttcccgcagcggatcctggggaaagagtgttgct**

**Citrus_reticulata_2** **acaactcgaaattgcatattatccttttcccgcagcggatcctggggaaagagtgttgct**

**Citrus_trifoliata_3** **acaactcgaaattgcatattatccttttcccgcagcggatcctggggaaagagtgttgct**

**Citrus_aurantiifolia** **acaactcgaaattgcatattatccttttcccgcagcggatcctggggaaagagtgttgct**

**Citrus_aurantium** **acaactcgaaattgcatattatccttttcccgcagcggatcctggggaaagagtgttgct**

**Citrus_cavaleriei** **acaactcgaaattgcatattatccttttcccgcagcggatcctggggaaagagtgttgct**

**Citrus_hongheensis** **acaactcgaaattgcatattatccttttcccgcagcggatcctggggaaagagtgttgct**

**Citrus_limon** **acaactcgaaattgcatattatccttttcccgcagcggatcctggggaaagagtgttgct**

**Citrus_maxima** **acaactcgaaattgcatattatccttttcccgcagcggatcctggggaaagagtgttgct**

**Citrus_medica** **acaactcgaaattgcatattatccttttcccgcagcggatcctggggaaagagtgttgct**

**Citrus_polytrifolia** **acaactcgaaattgcatattatccttttcccgcagcggatcctggggaaagagtgttgct**

**Citrus_reticulata** **acaactcgaaattgcatattatccttttcccgcagcggatcctggggaaagagtgttgct**

**Citrus_sinensis** **acaactcgaaattgcatattatccttttcccgcagcggatcctggggaaagagtgttgct**

**Fortunella_bawangica_1** **acaactcgaaattgcatattatccttttcccgcagcggatcctggggaaagagtgttgct**

**Fortunella_bawangica_2** **acaactcgaaattgcatattatccttttcccgcagcggatcctggggaaagagtgttgct**

**Fortunella_hindsii_1** **acaactcgaaattgcatattatccttttcccgcagcggatcctggggaaagagtgttgct**

**Fortunella_hindsii_2** **acaactcgaaattgcatattatccttttcccgcagcggatcctggggaaagagtgttgct**

**Fortunella_hindsii_3** **acaactcgaaattgcatattatccttttcccgcagcggatcctggggaaagagtgttgct**

**Fortunella_japonica_1** **acaactcgaaattgcatattatccttttcccgcagcggatcctggggaaagagtgttgct**

**Fortunella_japonica_2** **acaactcgaaattgcatattatccttttcccgcagcggatcctggggaaagagtgttgct**

**Fortunella_japonica_3** **acaactcgaaattgcatattatccttttcccgcagcggatcctggggaaagagtgttgct**

**Fortunella_japonica_4** **acaactcgaaattgcatattatccttttcccgcagcggatcctggggaaagagtgttgct**

**Fortunella_japonica_5** **acaactcgaaattgcatattatccttttcccgcagcggatcctggggaaagagtgttgct**

**Fortunella_japonica_6** **acaactcgaaattgcatattatccttttcccgcagcggatcctggggaaagagtgttgct**

**Fortunella_japonica_7** **acaactcgaaattgcatattatccttttcccgcagcggatcctggggaaagagtgttgct**

**Fortunella_japonica_8** **acaactcgaaattgcatattatccttttcccgcagcggatcctggggaaagagtgttgct**

**Fortunella_japonica_9** **acaactcgaaattgcatattatccttttcccgcagcggatcctggggaaagagtgttgct**

**Fortunella_japonica_10** **acaactcgaaattgcatattatccttttcccgcagcggatcctggggaaagagtgttgct**

**Fortunella_japonica_11** **acaactcgaaattgcatattatccttttcccgcagcggatcctggggaaagagtgttgct**

**Fortunella_japonica_12** **acaactcgaaattgcatattatccttttcccgcagcggatcctggggaaagagtgttgct**

**Fortunella_margarita_1** **acaactcgaaattgcatattatccttttcccgcagcggatcctggggaaagagtgttgct**

**Fortunella_margarita_2** **acaactcgaaattgcatattatccttttcccgcagcggatcctggggaaagagtgttgct**

**Fortunella_venosa_1** **acaactcgaaattgcatattatccttttcccgcagcggatcctggggaaagagtgttgct**

**Fortunella_venosa_2** **acaactcgaaattgcatattatccttttcccgcagcggatcctggggaaagagtgttgct**

**Fortunella_venosa_3** **acaactcgaaattgcatattatccttttcccgcagcggatcctggggaaagagtgttgct**

20470	20480	20490	20500	20510	20520

....|....|....|....|....|....|....|....|....|....|....|....|

**Atalantia_kwangtungens** **aaatttataccgtccgctatttcatatgtgactacgggtcgaaccaaaacaaaagacttt**

**Citrus_reticulata_1** **aaatttataccgttcgctatttcatatgtgactacgggtcgaaccaaaacaaaagacttt**

**Citrus_reticulata_2** **aaatttataccgttcgctatttcatatgtgactacgggtcgaaccaaaacaaaagacttt**

**Citrus_trifoliata_3** **aaatttataccgtccgctatttcatatgtgactacgggtcgaaccaaaacaaaagacttt**

**Citrus_aurantiifolia** **aaatttataccgttcgctatttcatatgtgactacgggtcgaaccaaaacaaaagacttt**

**Citrus_aurantium** **aaatttataccgttcgctatttcatatgtgactacgggtcgaaccaaaacaaaagacttt**

**Citrus_cavaleriei** **aaatttataccgttcgctatttcatatgtgactacgggtcgaaccaaaacaaaagacttt**

**Citrus_hongheensis** **aaatttataccgttcgctatttcatatgtgactacgggtcgaaccaaaacaaaagacttt**

**Citrus_limon** **aaatttataccgttcgctatttcatatgtgactacgggtcgaaccaaaacaaaagacttt**

**Citrus_maxima** **aaatttataccgttcgctatttcatatgtgactacgggtcgaaccaaaacaaaagacttt**

**Citrus_medica** **aaatttataccgtccgctatttcatatgtgactacgggtcgaaccaaaacaaaagacttt**

**Citrus_polytrifolia** **aaatttataccgtccgctatttcatatgtgactacgggtcgaaccaaaacaaaagacttt**

**Citrus_reticulata** **aaatttataccgttcgctatttcatatgtgactacgggtcgaaccaaaacaaaagacttt**

**Citrus_sinensis** **aaatttataccgttcgctatttcatatgtgactacgggtcgaaccaaaacaaaagacttt**

**Fortunella_bawangica_1** **aaatttataccgtccgctatttcatatgtgactacgggtcgaaccaaaacaaaagacttt**

**Fortunella_bawangica_2** **aaatttataccgtccgctatttcatatgtgactacgggtcgaaccaaaacaaaagacttt**

**Fortunella_hindsii_1** **aaatttataccgtccgctatttcatatgtgactacgggtcgaaccaaaacaaaagacttt**

**Fortunella_hindsii_2** **aaatttataccgtccgctatttcatatgtgactacgggtcgaaccaaaacaaaagacttt**

**Fortunella_hindsii_3** **aaatttataccgtccgctatttcatatgtgactacgggtcgaaccaaaacaaaagacttt**

**Fortunella_japonica_1** **aaatttataccgtccgctatttcatatgtgactacgggtcgaaccaaaacaaaagacttt**

**Fortunella_japonica_2** **aaatttataccgtccgctatttcatatgtgactacgggtcgaaccaaaacaaaagacttt**

**Fortunella_japonica_3** **aaatttataccgtccgctatttcatatgtgactacgggtcgaaccaaaacaaaagacttt**

**Fortunella_japonica_4** **aaatttataccgtccgctatttcatatgtgactacgggtcgaaccaaaacaaaagacttt**

**Fortunella_japonica_5** **aaatttataccgtccgctatttcatatgtgactacgggtcgaaccaaaacaaaagacttt**

**Fortunella_japonica_6** **aaatttataccgtccgctatttcatatgtgactacgggtcgaaccaaaacaaaagacttt**

**Fortunella_japonica_7** **aaatttataccgtccgctatttcatatgtgactacgggtcgaaccaaaacaaaagacttt**

**Fortunella_japonica_8** **aaatttataccgtccgctatttcatatgtgactacgggtcgaaccaaaacaaaagacttt**

**Fortunella_japonica_9** **aaatttataccgtccgctatttcatatgtgactacgggtcgaaccaaaacaaaagacttt**

**Fortunella_japonica_10** **aaatttataccgtccgctatttcatatgtgactacgggtcgaaccaaaacaaaagacttt**

**Fortunella_japonica_11** **aaatttataccgtccgctatttcatatgtgactacgggtcgaaccaaaacaaaagacttt**

**Fortunella_japonica_12** **aaatttataccgtccgctatttcatatgtgactacgggtcgaaccaaaacaaaagacttt**

**Fortunella_margarita_1** **aaatttataccgtccgctatttcatatgtgactacgggtcgaaccaaaacaaaagacttt**

**Fortunella_margarita_2** **aaatttataccgtccgctatttcatatgtgactacgggtcgaaccaaaacaaaagacttt**

**Fortunella_venosa_1** **aaatttataccgtccgctatttcatatgtgactacgggtcgaaccaaaacaaaagacttt**

**Fortunella_venosa_2** **aaatttataccgtccgctatttcatatgtgactacgggtcgaaccaaaacaaaagacttt**

**Fortunella_venosa_3** **aaatttataccgtccgctatttcatatgtgactacgggtcgaaccaaaacaaaagacttt**

20530	20540	20550	20560	20570	20580

....|....|....|....|....|....|....|....|....|....|....|....|

**Atalantia_kwangtungens** **ttcttggtaggtgtgatccgttgaacatagatccactttttcaatttttttgattcctta**

**Citrus_reticulata_1** **ttctttgtaggtgtgatccgttgaacatagatccactttttcaatttttttgattcctta**

**Citrus_reticulata_2** **ttctttgtaggtgtgatccgttgaacatagatccactttttcaatttttttgattcctta**

**Citrus_trifoliata_3** **ttctttgtaggtgtgatccgttgaacatagatccactttttcaatttttttgattcctta**

**Citrus_aurantiifolia** **ttctttgtaggtgtgatccgttgaacatagatccactttttcaatttttttgattcctta**

**Citrus_aurantium** **ttctttgtaggtgtgatccgttgaacatagatccactttttcaatttttttgattcctta**

**Citrus_cavaleriei** **ttctttgtaggtgtgatccgttgaacatagatccactttttcaatttttttgattcctta**

**Citrus_hongheensis** **ttctttgtaggtgtgatccgttgaacatagatccactttttcaatttttttgattcctta**

**Citrus_limon** **ttctttgtaggtgtgatccgttgaacatagatccactttttcaatttttttgattcctta**

**Citrus_maxima** **ttctttgtaggtgtgatccgttgaacatagatccactttttcaatttttttgattcctta**

**Citrus_medica** **ttctttgtaggtgtgatccgttgaacatagatccactttttcaatttttttgattcctta**

**Citrus_polytrifolia** **ttctttgtaggtgtgatccgttgaacatagatccactttttcaatttttttgattcctta**

**Citrus_reticulata** **ttctttgtaggtgtgatccgttgaacatagatccactttttcaatttttttgattcctta**

**Citrus_sinensis** **ttctttgtaggtgtgatccgttgaacatagatccactttttcaatttttttgattcctta**

**Fortunella_bawangica_1** **ttctttgtaggtgtgatccgttgaacatagatccactttttcaatttttttgattcctta**

**Fortunella_bawangica_2** **ttctttgtaggtgtgatccgttgaacatagatccactttttcaatttttttgattcctta**

**Fortunella_hindsii_1** **ttctttgtaggtgtgatccgttgaacatagatccactttttcaatttttttgattcctta**

**Fortunella_hindsii_2** **ttctttgtaggtgtgatccgttgaacatagatccactttttcaatttttttgattcctta**

**Fortunella_hindsii_3** **ttctttgtaggtgtgatccgttgaacatagatccactttttcaatttttttgattcctta**

**Fortunella_japonica_1** **ttctttgtaggtgtgatccgttgaacatagatccactttttccatttttttgattcctta**

**Fortunella_japonica_2** **ttctttgtaggtgtgatccgttgaacatagatccactttttcaatttttttgattcctta**

**Fortunella_japonica_3** **ttctttgtaggtgtgatccgttgaacatagatccactttttccatttttttgattcctta**

**Fortunella_japonica_4** **ttctttgtaggtgtgatccgttgaacatagatccactttttccatttttttgattcctta**

**Fortunella_japonica_5** **ttctttgtaggtgtgatccgttgaacatagatccactttttccatttttttgattcctta**

**Fortunella_japonica_6** **ttctttgtaggtgtgatccgttgaacatagatccactttttccatttttttgattcctta**

**Fortunella_japonica_7** **ttctttgtaggtgtgatccgttgaacatagatccactttttccatttttttgattcctta**

**Fortunella_japonica_8** **ttctttgtaggtgtgatccgttgaacatagatccactttttccatttttttgattcctta**

**Fortunella_japonica_9** **ttctttgtaggtgtgatccgttgaacatagatccactttttccatttttttgattcctta**

**Fortunella_japonica_10** **ttctttgtaggtgtgatccgttgaacatagatccactttttcaatttttttgattcctta**

**Fortunella_japonica_11** **ttctttgtaggtgtgatccgttgaacatagatccactttttccatttttttgattcctta**

**Fortunella_japonica_12** **ttctttgtaggtgtgatccgttgaacatagatccactttttccatttttttgattcctta**

**Fortunella_margarita_1** **ttctttgtaggtgtgatccgttgaacatagatccactttttcaatttttttgattcctta**

**Fortunella_margarita_2** **ttctttgtaggtgtgatccgttgaacatagatccactttttcaatttttttgattcctta**

**Fortunella_venosa_1** **ttctttgtaggtgtgatccgttgaacatagatccactttttcaatttttttgattcctta**

**Fortunella_venosa_2** **ttctttgtaggtgtgatccgttgaacatagatccactttttcaatttttttgattcctta**

**Fortunella_venosa_3** **ttctttgtaggtgtgatccgttgaacatagatccactttttcaatttttttgattcctta**

20590	20600	20610	20620	20630	20640

....|....|....|....|....|....|....|....|....|....|....|....|

**Atalantia_kwangtungens** **gtggcttccttaagtttttttttttcactttctggcggtatcaggatgccactgtgtcgg**

**Citrus_reticulata_1** **gtggcttccttaagtttttttttttcactttctggcggtatcaggatgccactgtgtcgg**

**Citrus_reticulata_2** **gtggcttccttaagtttttttttttcactttctggcggtatcaggatgccactgtgtcgg**

**Citrus_trifoliata_3** **gtggcttccttaagtttttttttttcactttctggcggtatcaggatgccactgtgtcgg**

**Citrus_aurantiifolia** **gtggcttccttaagttttgttttttcactttctggcggtatcaggatgccactgtgtcgg**

**Citrus_aurantium** **gtggcttccttaagttttgttttttcactttctggcggtatcaggatgccactgtgtcgg**

**Citrus_cavaleriei** **gtggcttccttaagtttttttttttcactttctggcggtatcaggatgccactgtgtcgg**

**Citrus_hongheensis** **gtggcttccttaagttttgttttttcactttctggcggtatcaggatgccactgtgtcgg**

**Citrus_limon** **gtggcttccttaagttttgttttttcactttctggcggtatcaggatgccactgtgtcgg**

**Citrus_maxima** **gtggcttccttaagttttgttttttcactttctggcggtatcaggatgccactgtgtcgg**

**Citrus_medica** **gtggcttccttaagtttttttttttcactttctggcggtatcaggatgccactgtgtcgg**

**Citrus_polytrifolia** **gtggcttccttaagtttttttttttcactttctggcggtatcaggatgccactgtgtcgg**

**Citrus_reticulata** **gtggcttccttaagtttttttttttcactttctggcggtatcaggatgccactgtgtcgg**

**Citrus_sinensis** **gtggcttccttaagttttgttttttcactttctggcggtatcaggatgccactgtgtcgg**

**Fortunella_bawangica_1** **gtggcttccttaagtttttttttttcactttctggcggtatcaggatgccacggtgtcgg**

**Fortunella_bawangica_2** **gtggcttccttaagtttttttttttcactttctggcggtatcaggatgccacggtgtcgg**

**Fortunella_hindsii_1** **gtggcttccttaagtttttttttttcactttctggcggtatcaggatgccacggtgtcgg**

**Fortunella_hindsii_2** **gtggcttccttaagtttttttttttcactttctggcggtatcaggatgccacggtgtcgg**

**Fortunella_hindsii_3** **gtggcttccttaagtttttttttttcactttctggcggtatcaggatgccacggtgtcgg**

**Fortunella_japonica_1** **gtggcttccttaagtttttttttttcactttctggcggtatcaggatgccacggtgtcgg**

**Fortunella_japonica_2** **gtggcttccttaagtttttttttttcactttctggcggtatcaggatgccacggtgtcgg**

**Fortunella_japonica_3** **gtggcttccttaagtttttttttttcactttctggcggtatcaggatgccacggtgtcgg**

**Fortunella_japonica_4** **gtggcttccttaagtttttttttttcactttctggcggtatcaggatgccacggtgtcgg**

**Fortunella_japonica_5** **gtggcttccttaagtttttttttttcactttctggcggtatcaggatgccacggtgtcgg**

**Fortunella_japonica_6** **gtggcttccttaagtttttttttttcactttctggcggtatcaggatgccacggtgtcgg**

**Fortunella_japonica_7** **gtggcttccttaagtttttttttttcactttctggcggtatcaggatgccacggtgtcgg**

**Fortunella_japonica_8** **gtggcttccttaagtttttttttttcactttctggcggtatcaggatgccacggtgtcgg**

**Fortunella_japonica_9** **gtggcttccttaagtttttttttttcactttctggcggtatcaggatgccacggtgtcgg**

**Fortunella_japonica_10** **gtggcttccttaagtttttttttttcactttctggcggtatcaggatgccacggtgtcgg**

**Fortunella_japonica_11** **gtggcttccttaagtttttttttttcactttctggcggtatcaggatgccacggtgtcgg**

**Fortunella_japonica_12** **gtggcttccttaagtttttttttttcactttctggcggtatcaggatgccacggtgtcgg**

**Fortunella_margarita_1** **gtggcttccttaagtttttttttttcactttctggcggtatcaggatgccacggtgtcgg**

**Fortunella_margarita_2** **gtggcttccttaagtttttttttttcactttctggcggtatcaggatgccacggtgtcgg**

**Fortunella_venosa_1** **gtggcttccttaagtttttttttttcactttctggcggtatcaggatgccacggtgtcgg**

**Fortunella_venosa_2** **gtggcttccttaagtttttttttttcactttctggcggtatcaggatgccacggtgtcgg**

**Fortunella_venosa_3** **gtggcttccttaagtttttttttttcactttctggcggtatcaggatgccacggtgtcgg**

20650	20660	20670	20680	20690	20700

....|....|....|....|....|....|....|....|....|....|....|....|

**Atalantia_kwangtungens** **gatatcttatctatctctccgggaaaatggatatctcccgaaaatatttttagttcaacc**

**Citrus_reticulata_1** **gatatcttatctatctctccgggaaaatggatatctcccgaaaatatttttagttcaacc**

**Citrus_reticulata_2** **gatatcttatctatctctccgggaaaatggatatctcccgaaaatatttttagttcaacc**

**Citrus_trifoliata_3** **gatatcttatctatctctccgggaaaatggatatctcccgaaaatatttttagttcaacc**

**Citrus_aurantiifolia** **gatatcttatctatctctccgggaaaatggatatctcccgaaaatatttttagttcaacc**

**Citrus_aurantium** **gatatcttatctatctctccgggaaaatggatatctcccgaaaatatttttagttcaacc**

**Citrus_cavaleriei** **gatatcttatctatctctccgggaaaatggatatctcccgaaaatattttgagttcaacc**

**Citrus_hongheensis** **gatatcttatctatctctccgggaaaatggatatctcccgaaaatatttttagttcaacc**

**Citrus_limon** **gatatcttatctatctctccgggaaaatggatatctcccgaaaatatttttagttcaacc**

**Citrus_maxima** **gatatcttatctatctctccgggaaaatggatatctcccgaaaatatttttagttcaacc**

**Citrus_medica** **gatatcttatctatctctccgggaaaatggatatctcccgaaaatatttttagttcaacc**

**Citrus_polytrifolia** **gatatcttatctatctctccgggaaaatggatatctcccgaaaatatttttagttcaacc**

**Citrus_reticulata** **gatatcttatctatctctccgggaaaatggatatctcccgaaaatatttttagttcaacc**

**Citrus_sinensis** **gatatcttatctatctctccgggaaaatggatatctcccgaaaatatttttagttcaacc**

**Fortunella_bawangica_1** **gatatcttatctatctctccgggaaaatggatatctcccgaaaatattttgagttcaacc**

**Fortunella_bawangica_2** **gatatcttatctatctctccgggaaaatggatatctcccgaaaatattttgagttcaacc**

**Fortunella_hindsii_1** **gatatcttatctatctctccgggaaaatggatatctcccgaaaatattttgagttcaacc**

**Fortunella_hindsii_2** **gatatcttatctatctctccgggaaaatggatatctcccgaaaatattttgagttcaacc**

**Fortunella_hindsii_3** **gatatcttatctatctctccgggaaaatggatatctcccgaaaatattttgagttcaacc**

**Fortunella_japonica_1** **gatatcttatctatctctccgggaaaatggatatctcccgaaaatattttgagttcaacc**

**Fortunella_japonica_2** **gatatcttatctatctctccgggaaaatggatatctcccgaaaatattttgagttcaacc**

**Fortunella_japonica_3** **gatatcttatctatctctccgggaaaatggatatctcccgaaaatattttgagttcaacc**

**Fortunella_japonica_4** **gatatcttatctatctctccgggaaaatggatatctcccgaaaatattttgagttcaacc**

**Fortunella_japonica_5** **gatatcttatctatctctccgggaaaatggatatctcccgaaaatattttgagttcaacc**

**Fortunella_japonica_6** **gatatcttatctatctctccgggaaaatggatatctcccgaaaatattttgagttcaacc**

**Fortunella_japonica_7** **gatatcttatctatctctccgggaaaatggatatctcccgaaaatattttgagttcaacc**

**Fortunella_japonica_8** **gatatcttatctatctctccgggaaaatggatatctcccgaaaatattttgagttcaacc**

**Fortunella_japonica_9** **gatatcttatctatctctccgggaaaatggatatctcccgaaaatattttgagttcaacc**

**Fortunella_japonica_10** **gatatcttatctatctctccgggaaaatggatatctcccgaaaatattttgagttcaacc**

**Fortunella_japonica_11** **gatatcttatctatctctccgggaaaatggatatctcccgaaaatattttgagttcaacc**

**Fortunella_japonica_12** **gatatcttatctatctctccgggaaaatggatatctcccgaaaatattttgagttcaacc**

**Fortunella_margarita_1** **gatatcttatctatctctccgggaaaatggatatctcccgaaaatattttgagttcaacc**

**Fortunella_margarita_2** **gatatcttatctatctctccgggaaaatggatatctcccgaaaatattttgagttcaacc**

**Fortunella_venosa_1** **gatatcttatctatctctccgggaaaatggatatctcccgaaaatattttgagttcaacc**

**Fortunella_venosa_2** **gatatcttatctatctctccgggaaaatggatatctcccgaaaatattttgagttcaacc**

**Fortunella_venosa_3** **gatatcttatctatctctccgggaaaatggatatctcccgaaaatattttgagttcaacc**

20710	20720	20730	20740	20750	20760

....|....|....|....|....|....|....|....|....|....|....|....|

**Atalantia_kwangtungens** **cctcccctttttctctccattcggaccaatccgcccacccggcttcgtatatttaaagtg**

**Citrus_reticulata_1** **cctcccctttttctctccattcggaccaatccgcccacccggcttcgtatatttaaagtg**

**Citrus_reticulata_2** **cctcccctttttctctccattcggaccaatccgcccacccggcttcgtatatttaaagtg**

**Citrus_trifoliata_3** **cctcccctttttctctccattcggaccaatccgcccacccggcttcgtatatttaaagtg**

**Citrus_aurantiifolia** **ccccccctttttctctccattcggaccaatccgcccacccggcttcgtatatttaaagtg**

**Citrus_aurantium** **cctcccctttttctctccattcggaccaatccgcccacccggcttcgtatatttaaagtg**

**Citrus_cavaleriei** **cctcccctttttctctccattcggaccaatccgcccacccggcttcgtatatttaaagtg**

**Citrus_hongheensis** **cctcccctttttctctccattcggaccaatccgcccacccggcttcgtatatttaaagtg**

**Citrus_limon** **cctcccctttttctctccattcggaccaatccgcccacccggcttcgtatatttaaagtg**

**Citrus_maxima** **cctcccctttttctctccattcggaccaatccgcccacccggcttcgtatatttaaagtg**

**Citrus_medica** **cctcccctttttctctccattcggaccaatccgcccacccggcttcgtatatttaaagtg**

**Citrus_polytrifolia** **cctcccctttttctctccattcggaccaatccgcccacccggcttcgtatatttaaagtg**

**Citrus_reticulata** **cctcccctttttctctccattcggaccaatccgcccacccggcttcgtatatttaaagtg**

**Citrus_sinensis** **cctcccctttttctctccattcggaccaatccgcccacccggcttcgtatatttaaagtg**

**Fortunella_bawangica_1** **cctcccctttttctctccattcggaccaatccgcccacccggcttcgtatatttaaagtg**

**Fortunella_bawangica_2** **cctcccctttttctctccattcggaccaatccgcccacccggcttcgtatatttaaagtg**

**Fortunella_hindsii_1** **cctcccctttttctctccattcggaccaatccgcccacccggcttcgtatatttaaagtg**

**Fortunella_hindsii_2** **cctcccctttttctctccattcggaccaatccgcccacccggcttcgtatatttaaagtg**

**Fortunella_hindsii_3** **cctcccctttttctctccattcggaccaatccgcccacccggcttcgtatatttaaagtg**

**Fortunella_japonica_1** **cctcccctttttctctccattcggaccaatccgcccacccggcttcgtatatttaaagtg**

**Fortunella_japonica_2** **cctcccctttttctctccattcggaccaatccgcccacccggcttcgtatatttaaagtg**

**Fortunella_japonica_3** **cctcccctttttctctccattcggaccaatccgcccacccggcttcgtatatttaaagtg**

**Fortunella_japonica_4** **cctcccctttttctctccattcggaccaatccgcccacccggcttcgtatatttaaagtg**

**Fortunella_japonica_5** **cctcccctttttctctccattcggaccaatccgcccacccggcttcgtatatttaaagtg**

**Fortunella_japonica_6** **cctcccctttttctctccattcggaccaatccgcccacccggcttcgtatatttaaagtg**

**Fortunella_japonica_7** **cctcccctttttctctccattcggaccaatccgcccacccggcttcgtatatttaaagtg**

**Fortunella_japonica_8** **cctcccctttttctctccattcggaccaatccgcccacccggcttcgtatatttaaagtg**

**Fortunella_japonica_9** **cctcccctttttctctccattcggaccaatccgcccacccggcttcgtatatttaaagtg**

**Fortunella_japonica_10** **cctcccctttttctctccattcggaccaatccgcccacccggcttcgtatatttaaagtg**

**Fortunella_japonica_11** **cctcccctttttctctccattcggaccaatccgcccacccggcttcgtatatttaaagtg**

**Fortunella_japonica_12** **cctcccctttttctctccattcggaccaatccgcccacccggcttcgtatatttaaagtg**

**Fortunella_margarita_1** **cctcccctttttctctccattcggaccaatccgcccacccggcttcgtatatttaaagtg**

**Fortunella_margarita_2** **cctcccctttttctctccattcggaccaatccgcccacccggcttcgtatatttaaagtg**

**Fortunella_venosa_1** **cctcccctttttctctccattcggaccaatccgcccacccggcttcgtatatttaaagtg**

**Fortunella_venosa_2** **cctcccctttttctctccattcggaccaatccgcccacccggcttcgtatatttaaagtg**

**Fortunella_venosa_3** **cctcccctttttctctccattcggaccaatccgcccacccggcttcgtatatttaaagtg**

20770	20780	20790	20800	20810	20820

....|....|....|....|....|....|....|....|....|....|....|....|

**Atalantia_kwangtungens** **atttgtgtgtctactccaatgatactattattccgtaccattaggtaagaagattcggga**

**Citrus_reticulata_1** **atttgtgtgtctactccaatgatactattattccgtaccattaggtaagaagattcggga**

**Citrus_reticulata_2** **atttgtgtgtctactccaatgatactattattccgtaccattaggtaagaagattcggga**

**Citrus_trifoliata_3** **atttgtgtgtctactccaatgatactattattccgtaccattaggtaagaagattcggga**

**Citrus_aurantiifolia** **atttgtgtgtctactccaatgatactattattccgtaccattaggtaagaagattcggga**

**Citrus_aurantium** **atttgtgtgtctactccaatgatactattattccgtaccattaggtaagaagattcggga**

**Citrus_cavaleriei** **atttgtgtgtctactccaatgatactattattccgtaccattaggtaagaagattcggga**

**Citrus_hongheensis** **atttgtgtgtctactccaatgatactattattccgtaccattaggtaagaagattcggga**

**Citrus_limon** **atttgtgtgtctactccaatgatactattattccgtaccattaggtaagaagattcggga**

**Citrus_maxima** **atttgtgtgtctactccaatgatactattattccgtaccattaggtaagaagattcggga**

**Citrus_medica** **atttgtgtgtctactccaacgatactattattccgtaccattaggtaagaagattcggga**

**Citrus_polytrifolia** **atttgtgtgtctactccaatgatactattattccgtaccattaggtaagaagattcggga**

**Citrus_reticulata** **atttgtgtgtctactccaatgatactattattccgtaccattaggtaagaagattcggga**

**Citrus_sinensis** **atttgtgtgtctactccaatgatactattattccgtaccattaggtaagaagattcggga**

**Fortunella_bawangica_1** **atttgtgtgtctactccaatgatactattattccgtaccattaggtaagaagattcggga**

**Fortunella_bawangica_2** **atttgtgtgtctactccaatgatactattattccgtaccattaggtaagaagattcggga**

**Fortunella_hindsii_1** **atttgtgtgtctactccaatgatactattattccgtaccattaggtaagaagattcggga**

**Fortunella_hindsii_2** **atttgtgtgtctactccaatgatactattattccgtaccattaggtaagaagattcggga**

**Fortunella_hindsii_3** **atttgtgtgtctactccaatgatactattattccgtaccattaggtaagaagattcggga**

**Fortunella_japonica_1** **atttgtgtgtctactccaatgatactattattccgtaccattaggtaagaagattcggga**

**Fortunella_japonica_2** **atttgtgtgtctactccaatgatactattattccgtaccattaggtaagaagattcggga**

**Fortunella_japonica_3** **atttgtgtgtctactccaatgatactattattccgtaccattaggtaagaagattcggga**

**Fortunella_japonica_4** **atttgtgtgtctactccaatgatactattattccgtaccattaggtaagaagattcggga**

**Fortunella_japonica_5** **atttgtgtgtctactccaatgatactattattccgtaccattaggtaagaagattcggga**

**Fortunella_japonica_6** **atttgtgtgtctactccaatgatactattattccgtaccattaggtaagaagattcggga**

**Fortunella_japonica_7** **atttgtgtgtctactccaatgatactattattccgtaccattaggtaagaagattcggga**

**Fortunella_japonica_8** **atttgtgtgtctactccaatgatactattattccgtaccattaggtaagaagattcggga**

**Fortunella_japonica_9** **atttgtgtgtctactccaatgatactattattccgtaccattaggtaagaagattcggga**

**Fortunella_japonica_10** **atttgtgtgtctactccaatgatactattattccgtaccattaggtaagaagattcggga**

**Fortunella_japonica_11** **atttgtgtgtctactccaatgatactattattccgtaccattaggtaagaagattcggga**

**Fortunella_japonica_12** **atttgtgtgtctactccaatgatactattattccgtaccattaggtaagaagattcggga**

**Fortunella_margarita_1** **atttgtgtgtctactccaatgatactattattccgtaccattaggtaagaagattcggga**

**Fortunella_margarita_2** **atttgtgtgtctactccaatgatactattattccgtaccattaggtaagaagattcggga**

**Fortunella_venosa_1** **atttgtgtgtctactccaatgatactattattccgtaccattaggtaagaagattcggga**

**Fortunella_venosa_2** **atttgtgtgtctactccaatgatactattattccgtaccattaggtaagaagattcggga**

**Fortunella_venosa_3** **atttgtgtgtctactccaatgatactattattccgtaccattaggtaagaagattcggga**

20830	20840	20850	20860	20870	20880

....|....|....|....|....|....|....|....|....|....|....|....|

**Atalantia_kwangtungens** **aaaatatgcactttctccggaatgaaaaaaaggcgatctattttcatttggtattttggc**

**Citrus_reticulata_1** **aaaatatgcacttcctccggaatgaaaaaaaggcgatctattttcatttggtattttggc**

**Citrus_reticulata_2** **aaaatatgcacttcctccggaatgaaaaaaaggcgatctattttcatttggtattttggc**

**Citrus_trifoliata_3** **aaaatatgcacttcctccggaatgaaaaaaaggcgatctattttcatttggtattttggc**

**Citrus_aurantiifolia** **aaaatatgcacttcctccggaatgaaaaaaaggcgatctattttcatttggtattttggc**

**Citrus_aurantium** **aaaatatgcacttcctccggaatgaaaaaaaggcgatctattttcatttggtattttggc**

**Citrus_cavaleriei** **aaaatatgcacttcctccggaatgaaaaaaaggcgatctattttcatttggtattttggc**

**Citrus_hongheensis** **aaaatatgcacttcctccggaatgaaaaaaaggcgatctattttcatttggtattttggc**

**Citrus_limon** **aaaatatgcacttcctccggaatgaaaaaaaggcgatctattttcatttggtattttggc**

**Citrus_maxima** **aaaatatgcacttcctccggaatgaaaaaaaggcgatctattttcatttggtattttggc**

**Citrus_medica** **aaaatatgcacttcctccggaatgaaaaaaaggcgatctattttcatttggtattttggc**

**Citrus_polytrifolia** **aaaatatgcacttcctccggaatgaaaaaaaggcgatctattttcatttggtattttggc**

**Citrus_reticulata** **aaaatatgcacttcctccggaatgaaaaaaaggcgatctattttcatttggtattttggc**

**Citrus_sinensis** **aaaatatgcacttcctccggaatgaaaaaaaggcgatctattttcatttggtattttggc**

**Fortunella_bawangica_1** **aaaatatgcacttcctccggaatgaaaaaaaggcgatctattttcatttggtattttggc**

**Fortunella_bawangica_2** **aaaatatgcacttcctccggaatgaaaaaaaggcgatctattttcatttggtattttggc**

**Fortunella_hindsii_1** **aaaatatgcacttcctccggaatgaaaaaaaggcgatctattttcatttggtattttggc**

**Fortunella_hindsii_2** **aaaatatgcacttcctccggaatgaaaaaaaggcgatctattttcatttggtattttggc**

**Fortunella_hindsii_3** **aaaatatgcacttcctccggaatgaaaaaaaggcgatctattttcatttggtattttggc**

**Fortunella_japonica_1** **aaaatatgcacttcctccggaatgaaaaaaaggcgatctattttcatttggtattttggc**

**Fortunella_japonica_2** **aaaatatgcacttcctccggaatgaaaaaaaggcgatctattttcatttggtattttggc**

**Fortunella_japonica_3** **aaaatatgcacttcctccggaatgaaaaaaaggcgatctattttcatttggtattttggc**

**Fortunella_japonica_4** **aaaatatgcacttcctccggaatgaaaaaaaggcgatctattttcatttggtattttggc**

**Fortunella_japonica_5** **aaaatatgcacttcctccggaatgaaaaaaaggcgatctattttcatttggtattttggc**

**Fortunella_japonica_6** **aaaatatgcacttcctccggaatgaaaaaaaggcgatctattttcatttggtattttggc**

**Fortunella_japonica_7** **aaaatatgcacttcctccggaatgaaaaaaaggcgatctattttcatttggtattttggc**

**Fortunella_japonica_8** **aaaatatgcacttcctccggaatgaaaaaaaggcgatctattttcatttggtattttggc**

**Fortunella_japonica_9** **aaaatatgcacttcctccggaatgaaaaaaaggcgatctattttcatttggtattttggc**

**Fortunella_japonica_10** **aaaatatgcacttcctccggaatgaaaaaaaggcgatctattttcatttggtattttggc**

**Fortunella_japonica_11** **aaaatatgcacttcctccggaatgaaaaaaaggcgatctattttcatttggtattttggc**

**Fortunella_japonica_12** **aaaatatgcacttcctccggaatgaaaaaaaggcgatctattttcatttggtattttggc**

**Fortunella_margarita_1** **aaaatatgcacttcctccggaatgaaaaaaaggcgatctattttcatttggtattttggc**

**Fortunella_margarita_2** **aaaatatgcacttcctccggaatgaaaaaaaggcgatctattttcatttggtattttggc**

**Fortunella_venosa_1** **aaaatatgcacttcctccggaatgaaaaaaaggcgatctattttcatttggtattttggc**

**Fortunella_venosa_2** **aaaatatgcacttcctccggaatgaaaaaaaggcgatctattttcatttggtattttggc**

**Fortunella_venosa_3** **aaaatatgcacttcctccggaatgaaaaaaaggcgatctattttcatttggtattttggc**

20890	20900	20910	20920	20930	20940

....|....|....|....|....|....|....|....|....|....|....|....|

**Atalantia_kwangtungens** **ttaatttttttgagtcctcgatactcaatcaagtcctcttttttaacgattgaatgcgct**

**Citrus_reticulata_1** **ttaatttttttgagtcctcgatactcaatcaagtcctcttttttaacgattgaatgcgcc**

**Citrus_reticulata_2** **ttaatttttttgagtcctcgatactcaatcaagtcctcttttttaacgattgaatgcgcc**

**Citrus_trifoliata_3** **ttaatttttttgagtcctcgatactcaatcaagtcctcttttttaacgattgaatgcgcc**

**Citrus_aurantiifolia** **ttaatttttttgagtcctcgatactcaatcaagtcctcttttttaacgattgaatgcgcc**

**Citrus_aurantium** **ttaatttttttgagtcctcgatactcaatcaagtcctcttttttaacgattgaatgcgcc**

**Citrus_cavaleriei** **ttaatttttttgagtcctcgatactcaatcaagtcctcttttttaacgattgaatgcgcc**

**Citrus_hongheensis** **ttaatttttttgagtcctcgatactcaatcaagtcctcttttttaacgattgaatgcgcc**

**Citrus_limon** **ttaatttttttgagtcctcgatactcaatcaagtcctcttttttaacgattgaatgcgcc**

**Citrus_maxima** **ttaatttttttgagtcctcgatactcaatcaagtcctcttttttaacgattgaatgcgcc**

**Citrus_medica** **ttaatttttttgagtcctcgatactcaatcaagtcctcttttttaacgattgaatgcgcc**

**Citrus_polytrifolia** **ttaatttttttgagtcctcgatactcaatcaagtcctcttttttaacgattgaatgcgcc**

**Citrus_reticulata** **ttaatttttttgagtcctcgatactcaatcaagtcctcttttttaacgattgaatgcgcc**

**Citrus_sinensis** **ttaatttttttgagtcctcgatactcaatcaagtcctcttttttaacgattgaatgcgcc**

**Fortunella_bawangica_1** **ttaatttttttgagtcctcgatactcaatcaagtcctcttttttaacgattgaatgcgcc**

**Fortunella_bawangica_2** **ttaatttttttgagtcctcgatactcaatcaagtcctcttttttaacgattgaatgcgcc**

**Fortunella_hindsii_1** **ttaatttttttgagtcctcgatactcaatcaagtcctcttttttaacgattgaatgcgcc**

**Fortunella_hindsii_2** **ttaatttttttgagtcctcgatactcaatcaagtcctcttttttaacgattgaatgcgcc**

**Fortunella_hindsii_3** **ttaatttttttgagtcctcgatactcaatcaagtcctcttttttaacgattgaatgcgcc**

**Fortunella_japonica_1** **ttaatttttttgagtcctcgatactcaatcaagtcctcttttttaacgattgaatgcgcc**

**Fortunella_japonica_2** **ttaatttttttgagtcctcgatactcaatcaagtcctcttttttaacgattgaatgcgcc**

**Fortunella_japonica_3** **ttaatttttttgagtcctcgatactcaatcaagtcctcttttttaacgattgaatgcgcc**

**Fortunella_japonica_4** **ttaatttttttgagtcctcgatactcaatcaagtcctcttttttaacgattgaatgcgcc**

**Fortunella_japonica_5** **ttaatttttttgagtcctcgatactcaatcaagtcctcttttttaacgattgaatgcgcc**

**Fortunella_japonica_6** **ttaatttttttgagtcctcgatactcaatcaagtcctcttttttaacgattgaatgcgcc**

**Fortunella_japonica_7** **ttaatttttttgagtcctcgatactcaatcaagtcctcttttttaacgattgaatgcgcc**

**Fortunella_japonica_8** **ttaatttttttgagtcctcgatactcaatcaagtcctcttttttaacgattgaatgcgcc**

**Fortunella_japonica_9** **ttaatttttttgagtcctcgatactcaatcaagtcctcttttttaacgattgaatgcgcc**

**Fortunella_japonica_10** **ttaatttttttgagtcctcgatactcaatcaagtcctcttttttaacgattgaatgcgcc**

**Fortunella_japonica_11** **ttaatttttttgagtcctcgatactcaatcaagtcctcttttttaacgattgaatgcgcc**

**Fortunella_japonica_12** **ttaatttttttgagtcctcgatactcaatcaagtcctcttttttaacgattgaatgcgcc**

**Fortunella_margarita_1** **ttaatttttttgagtcctcgatactcaatcaagtcctcttttttaacgattgaatgcgcc**

**Fortunella_margarita_2** **ttaatttttttgagtcctcgatactcaatcaagtcctcttttttaacgattgaatgcgcc**

**Fortunella_venosa_1** **ttaatttttttgagtcctcgatactcaatcaagtcctcttttttaacgattgaatgcgcc**

**Fortunella_venosa_2** **ttaatttttttgagtcctcgatactcaatcaagtcctcttttttaacgattgaatgcgcc**

**Fortunella_venosa_3** **ttaatttttttgagtcctcgatactcaatcaagtcctcttttttaacgattgaatgcgcc**

20950	20960	20970	20980	20990	21000

....|....|....|....|....|....|....|....|....|....|....|....|

**Atalantia_kwangtungens** **cccaaagtcccccatttagtaattccggaactctttcttctgtatcgaggatcgtcgaaa**

**Citrus_reticulata_1** **cccagagccccccatttagtaattccggaactctttcttctgtatcgaggatcgtcgaaa**

**Citrus_reticulata_2** **cccagagccccccatttagtaattccggaactctttcttctgtatcgaggatcgtcgaaa**

**Citrus_trifoliata_3** **cccagagtcccccatttagtaattccggaactctttcttctgtatcgaggatcgtcgaaa**

**Citrus_aurantiifolia** **cccagagtcccccatttagtaattccggaactctttcttctgtatcgaggatcgtcgaaa**

**Citrus_aurantium** **cccagagtcccccatttagtaattccggaactctttcttctgtatcgaggatcgtcgaaa**

**Citrus_cavaleriei** **cccagagccccccatttagtaattccggaactctttcttctgtatcgaggatcgtcgaaa**

**Citrus_hongheensis** **cccagagtcccccatttagtaattccggaactctttcttctgtatcgaggatcgtcgaaa**

**Citrus_limon** **cccagagtcccccatttagtaattccggaactctttcttctgtatcgaggatcgtcgaaa**

**Citrus_maxima** **cccagagtcccccatttagtaattccggaactctttcttctgtatcgaggatcgtcgaaa**

**Citrus_medica** **cccagagtcccccatttagtaattccggaactctttcttctgtatcgaggatcgtcgaaa**

**Citrus_polytrifolia** **cccagagtcccccatttagtaattccggaactctttcttctgtatcgaggatcgtcgaaa**

**Citrus_reticulata** **cccagagccccccatttagtaattccggaactctttcttctgtatcgaggatcgtcgaaa**

**Citrus_sinensis** **cccagagtcccccatttagtaattccggaactctttcttctgtatcgaggatcgtcgaaa**

**Fortunella_bawangica_1** **cccagagtcccccatttagtaattccggaactctttcttctgtatcgaggatcgtcgaaa**

**Fortunella_bawangica_2** **cccagagtcccccatttagtaattccggaactctttcttctgtatcgaggatcgtcgaaa**

**Fortunella_hindsii_1** **cccagagtcccccatttagtaattccggaactctttcttctgtatcgaggatcgtcgaaa**

**Fortunella_hindsii_2** **cccagagtcccccatttagtaattccggaactctttcttctgtatcgaggatcgtcgaaa**

**Fortunella_hindsii_3** **cccagagtcccccatttagtaattccggaactctttcttctgtatcgaggatcgtcgaaa**

**Fortunella_japonica_1** **cccagagtcccccatttagtaattccggaactctttcttctgtatcgaggatcgtcgaaa**

**Fortunella_japonica_2** **cccagagtcccccatttagtaattccggaactctttcttctgtatcgaggatcgtcgaaa**

**Fortunella_japonica_3** **cccagagtcccccatttagtaattccggaactctttcttctgtatcgaggatcgtcgaaa**

**Fortunella_japonica_4** **cccagagtcccccatttagtaattccggaactctttcttctgtatcgaggatcgtcgaaa**

**Fortunella_japonica_5** **cccagagtcccccatttagtaattccggaactctttcttctgtatcgaggatcgtcgaaa**

**Fortunella_japonica_6** **cccagagtcccccatttagtaattccggaactctttcttctgtatcgaggatcgtcgaaa**

**Fortunella_japonica_7** **cccagagtcccccatttagtaattccggaactctttcttctgtatcgaggatcgtcgaaa**

**Fortunella_japonica_8** **cccagagtcccccatttagtaattccggaactctttcttctgtatcgaggatcgtcgaaa**

**Fortunella_japonica_9** **cccagagtcccccatttagtaattccggaactctttcttctgtatcgaggatcgtcgaaa**

**Fortunella_japonica_10** **cccagagtcccccatttagtaattccggaactctttcttctgtatcgaggatcgtcgaaa**

**Fortunella_japonica_11** **cccagagtcccccatttagtaattccggaactctttcttctgtatcgaggatcgtcgaaa**

**Fortunella_japonica_12** **cccagagtcccccatttagtaattccggaactctttcttctgtatcgaggatcgtcgaaa**

**Fortunella_margarita_1** **cccagagtcccccatttagtaattccggaactctttcttctgtatcgaggatcgtcgaaa**

**Fortunella_margarita_2** **cccagagtcccccatttagtaattccggaactctttcttctgtatcgaggatcgtcgaaa**

**Fortunella_venosa_1** **cccagagtcccccatttagtaattccggaactctttcttctgtatcgaggatcgtcgaaa**

**Fortunella_venosa_2** **cccagagtcccccatttagtaattccggaactctttcttctgtatcgaggatcgtcgaaa**

**Fortunella_venosa_3** **cccagagtcccccatttagtaattccggaactctttcttctgtatcgaggatcgtcgaaa**

21010	21020	21030	21040	21050	21060

....|....|....|....|....|....|....|....|....|....|....|....|

**Atalantia_kwangtungens** **taagcaagaatactatttctacggaaaataccccttacgggtatttcaatcgagataccc**

**Citrus_reticulata_1** **taagcaagaatactatttctacggaaaataccccttacgggtatttcaatcgagatacct**

**Citrus_reticulata_2** **taagcaagaatactatttctacggaaaataccccttacgggtatttcaatcgagatacct**

**Citrus_trifoliata_3** **taagcaagaatactatttctacggaaaataccccttacgggtatttcactcgagatacct**

**Citrus_aurantiifolia** **taagcaagaatactatttctacggaaaataccccttacgggtatttcaatcgagatacct**

**Citrus_aurantium** **taagcaagaatactatttctacggaaaataccccttacgggtatttcaatcgagatacct**

**Citrus_cavaleriei** **taagcaagaatactatttctacggaaaataccccttacgggtatttcaatcgagatacct**

**Citrus_hongheensis** **taagcaagaatactatttctacggaaaataccccttacgggtatttcaatcgagatacct**

**Citrus_limon** **taagcaagaatactatttctacggaaaataccccttacgggtatttcaatcgagatacct**

**Citrus_maxima** **taagcaagaatactatttctacggaaaataccccttacgggtatttcaatcgagatacct**

**Citrus_medica** **taagcaagaatactatttctacggaaaataccccttacgggtatttcaatcgagatacct**

**Citrus_polytrifolia** **taagcaagaatactatttctacggaaaataccccttacgggtatttcactcgagatacct**

**Citrus_reticulata** **taagcaagaatactatttctacggaaaataccccttacgggtatttcaatcgagatacct**

**Citrus_sinensis** **taagcaagaatactatttctacggaaaataccccttacgggtatttcaatcgagatacct**

**Fortunella_bawangica_1** **taagcaagaatactatttctacggaaaataccccttacgggtatttcaatcgagatacct**

**Fortunella_bawangica_2** **taagcaagaatactatttctacggaaaataccccttacgggtatttcaatcgagatacct**

**Fortunella_hindsii_1** **taagcaagaatactatttctacggaaaataccccttacgggtatttcaatcgagatacct**

**Fortunella_hindsii_2** **taagcaagaatactatttctacggaaaataccccttacgggtatttcaatcgagatacct**

**Fortunella_hindsii_3** **taagcaagaatactatttctacggaaaataccccttacgggtatttcaatcgagatacct**

**Fortunella_japonica_1** **taagcaagaatactatttctacggaaaataccccttacgggtatttcaatcgagatacct**

**Fortunella_japonica_2** **taagcaagaatactatttctacggaaaataccccttacgggtatttcaatcgagatacct**

**Fortunella_japonica_3** **taagcaagaatactatttctacggaaaataccccttacgggtatttcaatcgagatacct**

**Fortunella_japonica_4** **taagcaagaatactatttctacggaaaataccccttacgggtatttcaatcgagatacct**

**Fortunella_japonica_5** **taagcaagaatactatttctacggaaaataccccttacgggtatttcaatcgagatacct**

**Fortunella_japonica_6** **taagcaagaatactatttctacggaaaataccccttacgggtatttcaatcgagatacct**

**Fortunella_japonica_7** **taagcaagaatactatttctacggaaaataccccttacgggtatttcaatcgagatacct**

**Fortunella_japonica_8** **taagcaagaatactatttctacggaaaataccccttacgggtatttcaatcgagatacct**

**Fortunella_japonica_9** **taagcaagaatactatttctacggaaaataccccttacgggtatttcaatcgagatacct**

**Fortunella_japonica_10** **taagcaagaatactatttctacggaaaataccccttacgggtatttcaatcgagatacct**

**Fortunella_japonica_11** **taagcaagaatactatttctacggaaaataccccttacgggtatttcaatcgagatacct**

**Fortunella_japonica_12** **taagcaagaatactatttctacggaaaataccccttacgggtatttcaatcgagatacct**

**Fortunella_margarita_1** **taagcaagaatactatttctacggaaaataccccttacgggtatttcaatcgagatacct**

**Fortunella_margarita_2** **taagcaagaatactatttctacggaaaataccccttacgggtatttcaatcgagatacct**

**Fortunella_venosa_1** **taagcaagaatactatttctacggaaaataccccttacgggtatttcaatcgagatacct**

**Fortunella_venosa_2** **taagcaagaatactatttctacggaaaataccccttacgggtatttcaatcgagatacct**

**Fortunella_venosa_3** **taagcaagaatactatttctacggaaaataccccttacgggtatttcaatcgagatacct**

21070	21080	21090	21100	21110	21120

....|....|....|....|....|....|....|....|....|....|....|....|

**Atalantia_kwangtungens** **gaatggggcattagctctttctcttgctcttgaatcgagtggaatggaataagaaatcca**

**Citrus_reticulata_1** **gaatggggcattagctctttctcttgctcttgaatcgagtggaatggaataagaaatcca**

**Citrus_reticulata_2** **gaatggggcattagctctttctcttgctcttgaatcgagtggaatggaataagaaatcca**

**Citrus_trifoliata_3** **gaatggggcattagctctttctcttgctcttgaatcgagtggaatggaataagaaatcca**

**Citrus_aurantiifolia** **gaatggggcattagctctttctcttgctcttgaatcgagtggaatggaataagaaatcca**

**Citrus_aurantium** **gaatggggcattagctctttctcttgctcttgaatcgagtggaatggaataagaaatcca**

**Citrus_cavaleriei** **gaatggggcgttagctctttctcttgctcttgaatcgagtggaatggaataataaatcca**

**Citrus_hongheensis** **gaatggggcattagctctttctcttgctcttgaatcgagtggaatggaataagaaatcca**

**Citrus_limon** **gaatggggcattagctctttctcttgctcttgaatcgagtggaatggaataagaaatcca**

**Citrus_maxima** **gaatggggcattagctctttctcttgctcttgaatcgagtggaatggaataagaaatcca**

**Citrus_medica** **gaatggggcattagctctttctcttgcgcttgaatcgagtggaatggaataagaaatcca**

**Citrus_polytrifolia** **gaatggggcattagctctttctcttgctcttgaatcgagtggaatggaataagaaatcca**

**Citrus_reticulata** **gaatggggcattagctctttctcttgctcttgaatcgagtggaatggaataagaaatcca**

**Citrus_sinensis** **gaatggggcattagctctttctcttgctcttgaatcgagtggaatggaataagaaatcca**

**Fortunella_bawangica_1** **gaatggggcattagctctttctcttgctcttgaatcgagtggaatggaataagaaatcca**

**Fortunella_bawangica_2** **gaatggggcattagctctttctcttgctcttgaatcgagtggaatggaataagaaatcca**

**Fortunella_hindsii_1** **gaatggggcattagctctttctcttgctcttgaatcgagtggaatggaataagaaatcca**

**Fortunella_hindsii_2** **gaatggggcattagctctttctcttgctcttgaatcgagtggaatggaataagaaatcca**

**Fortunella_hindsii_3** **gaatggggcattagctctttctcttgctcttgaatcgagtggaatggaataagaaatcca**

**Fortunella_japonica_1** **gaatggggcattagctctttctcttgctcttgaatcgagtggaatggaataagaaatcca**

**Fortunella_japonica_2** **gaatggggcattagctctttctcttgctcttgaatcgagtggaatggaataagaaatcca**

**Fortunella_japonica_3** **gaatggggcattagctctttctcttgctcttgaatcgagtggaatggaataagaaatcca**

**Fortunella_japonica_4** **gaatggggcattagctctttctcttgctcttgaatcgagtggaatggaataagaaatcca**

**Fortunella_japonica_5** **gaatggggcattagctctttctcttgctcttgaatcgagtggaatggaataagaaatcca**

**Fortunella_japonica_6** **gaatggggcattagctctttctcttgctcttgaatcgagtggaatggaataagaaatcca**

**Fortunella_japonica_7** **gaatggggcattagctctttctcttgctcttgaatcgagtggaatggaataagaaatcca**

**Fortunella_japonica_8** **gaatggggcattagctctttctcttgctcttgaatcgagtggaatggaataagaaatcca**

**Fortunella_japonica_9** **gaatggggcattagctctttctcttgctcttgaatcgagtggaatggaataagaaatcca**

**Fortunella_japonica_10** **gaatggggcattagctctttctcttgctcttgaatcgagtggaatggaataagaaatcca**

**Fortunella_japonica_11** **gaatggggcattagctctttctcttgctcttgaatcgagtggaatggaataagaaatcca**

**Fortunella_japonica_12** **gaatggggcattagctctttctcttgctcttgaatcgagtggaatggaataagaaatcca**

**Fortunella_margarita_1** **gaatggggcattagctctttctcttgctcttgaatcgagtggaatggaataagaaatcca**

**Fortunella_margarita_2** **gaatggggcattagctctttctcttgctcttgaatcgagtggaatggaataagaaatcca**

**Fortunella_venosa_1** **gaatggggcattagctctttctcttgctcttgaatcgagtggaatggaataagaaatcca**

**Fortunella_venosa_2** **gaatggggcattagctctttctcttgctcttgaatcgagtggaatggaataagaaatcca**

**Fortunella_venosa_3** **gaatggggcattagctctttctcttgctcttgaatcgagtggaatggaataagaaatcca**

21130	21140	21150	21160	21170	21180

....|....|....|....|....|....|....|....|....|....|....|....|

**Atalantia_kwangtungens** **tttctttgcctttttgccaataaatccgaatttgcgtggagaattgcaggatgtctgaga**

**Citrus_reticulata_1** **tttctttgcctttttgccaataaatccgaatttgcgtggagaattgcaggatggatgaga**

**Citrus_reticulata_2** **tttctttgcctttttgccaataaatccgaatttgcgtggagaattgcaggatggatgaga**

**Citrus_trifoliata_3** **tttctttgcctttttgccaataaatccgaatttgcgtggagaattgcaggatggatgaga**

**Citrus_aurantiifolia** **tttctttgcctttttgccaataaatccgaatttgcgtggagaattgcaggatggatgaga**

**Citrus_aurantium** **tttctttgcctttttgccaataaatccgaatttgcgtggagaattgcaggatggatgaga**

**Citrus_cavaleriei** **tttctttgcctttttgccaataaatccgaatttgcgtggagaattgcaggatggatgaga**

**Citrus_hongheensis** **tttctttgcctttttgccaataaatccgaatttgcgtggagaattgcaggatggatgaga**

**Citrus_limon** **tttctttgcctttttgccaataaatccgaatttgcgtggagaattgcaggatggatgaga**

**Citrus_maxima** **tttctttgcctttttgccaataaatccgaatttgcgtggagaattgcaggatggatgaga**

**Citrus_medica** **tttctttgcctttttgccaataaatccgaatttgcgtggagaattgcaggatggatgaga**

**Citrus_polytrifolia** **tttctttgcctttttgccaataaatccgaatttgcgtggagaattgcaggatggatgaga**

**Citrus_reticulata** **tttctttgcctttttgccaataaatccgaatttgcgtggagaattgcaggatggatgaga**

**Citrus_sinensis** **tttctttgcctttttgccaataaatccgaatttgcgtggagaattgcaggatggatgaga**

**Fortunella_bawangica_1** **tttctttgcctttttgccaataaatccgaatttgcgtggagaattgcaggatggatgaga**

**Fortunella_bawangica_2** **tttctttgcctttttgccaataaatccgaatttgcgtggagaattgcaggatggatgaga**

**Fortunella_hindsii_1** **tttctttgcctttttgccaataaatccgaatttgcgtggagaattgcaggatggatgaga**

**Fortunella_hindsii_2** **tttctttgcctttttgccaataaatccgaatttgcgtggagaattgcaggatggatgaga**

**Fortunella_hindsii_3** **tttctttgcctttttgccaataaatccgaatttgcgtggagaattgcaggatggatgaga**

**Fortunella_japonica_1** **tttctttgcctttttgccaataaatccgaatttgcgtggagaattgcaggatggatgaga**

**Fortunella_japonica_2** **tttctttgcctttttgccaataaatccgaatttgcgtggagaattgcaggatggatgaga**

**Fortunella_japonica_3** **tttctttgcctttttgccaataaatccgaatttgcgtggagaattgcaggatggatgaga**

**Fortunella_japonica_4** **tttctttgcctttttgccaataaatccgaatttgcgtggagaattgcaggatggatgaga**

**Fortunella_japonica_5** **tttctttgcctttttgccaataaatccgaatttgcgtggagaattgcaggatggatgaga**

**Fortunella_japonica_6** **tttctttgcctttttgccaataaatccgaatttgcgtggagaattgcaggatggatgaga**

**Fortunella_japonica_7** **tttctttgcctttttgccaataaatccgaatttgcgtggagaattgcaggatggatgaga**

**Fortunella_japonica_8** **tttctttgcctttttgccaataaatccgaatttgcgtggagaattgcaggatggatgaga**

**Fortunella_japonica_9** **tttctttgcctttttgccaataaatccgaatttgcgtggagaattgcaggatggatgaga**

**Fortunella_japonica_10** **tttctttgcctttttgccaataaatccgaatttgcgtggagaattgcaggatggatgaga**

**Fortunella_japonica_11** **tttctttgcctttttgccaataaatccgaatttgcgtggagaattgcaggatggatgaga**

**Fortunella_japonica_12** **tttctttgcctttttgccaataaatccgaatttgcgtggagaattgcaggatggatgaga**

**Fortunella_margarita_1** **tttctttgcctttttgccaataaatccgaatttgcgtggagaattgcaggatggatgaga**

**Fortunella_margarita_2** **tttctttgcctttttgccaataaatccgaatttgcgtggagaattgcaggatggatgaga**

**Fortunella_venosa_1** **tttctttgcctttttgccaataaatccgaatttgcgtggagaattgcaggatggatgaga**

**Fortunella_venosa_2** **tttctttgcctttttgccaataaatccgaatttgcgtggagaattgcaggatggatgaga**

**Fortunella_venosa_3** **tttctttgcctttttgccaataaatccgaatttgcgtggagaattgcaggatggatgaga**

21190	21200	21210	21220	21230	21240

....|....|....|....|....|....|....|....|....|....|....|....|

**Atalantia_kwangtungens** **ttacaatgaccagtacctatgattctattaaatcccgaataatcagacatctcaagaatt**

**Citrus_reticulata_1** **ttacaatgaccagtacctatgattctattaaatcccgaataatcagacatctcaagaatt**

**Citrus_reticulata_2** **ttacaatgaccagtacctatgattctattaaatcccgaataatcagacatctcaagaatt**

**Citrus_trifoliata_3** **ttacaatgaccagtacctatgattctattaaatcccgaataatcagacatctcaagaatt**

**Citrus_aurantiifolia** **ttacaatgaccagtacctatgattctattaaatcccgaataatcagacatctcaagaatt**

**Citrus_aurantium** **ttacaatgaccagtacttatgattctattaaatcccgaataatcagacatctcaagaatt**

**Citrus_cavaleriei** **ttacaatgaccagtacctatgattctattaaatcccgaataatcagacatctcaagaatt**

**Citrus_hongheensis** **ttacaatgaccagtacttatgattctattaaatcccgaataatcagacatctcaagaatt**

**Citrus_limon** **ttacaatgaccagtacttatgattctattaaatcccgaataatcagacatctcaagaatt**

**Citrus_maxima** **ttacaatgaccagtacttatgattctattaaatcccgaataatcagacatctcaagaatt**

**Citrus_medica** **ttacaatgaccagtacctatgattctattaaatcccgaataatcagacatctcaagaatt**

**Citrus_polytrifolia** **ttacaatgaccagtacctatgattctattaaatcccgaataatcagacatctcaagaatt**

**Citrus_reticulata** **ttacaatgaccagtacctatgattctattaaatcccgaataatcagacatctcaagaatt**

**Citrus_sinensis** **ttacaatgaccagtacttatgattctattaaatcccgaataatcagacatctcaagaatt**

**Fortunella_bawangica_1** **ttacaatgaccagtacctatgattctattaaatcccgaataatcagacatctcaagaatt**

**Fortunella_bawangica_2** **ttacaatgaccagtacctatgattctattaaatcccgaataatcagacatctcaagaatt**

**Fortunella_hindsii_1** **ttacaatgaccagtacctatgattctattaaatcccgaataatcagacatctcaagaatt**

**Fortunella_hindsii_2** **ttacaatgaccagtacctatgattctattaaatcccgaataatcagacatctcaagaatt**

**Fortunella_hindsii_3** **ttacaatgaccagtacctatgattctattaaatcccgaataatcagacatctcaagaatt**

**Fortunella_japonica_1** **ttacaatgaccagtacctatgattctattaaatcccgaataatcagacatctcaagaatt**

**Fortunella_japonica_2** **ttacaatgaccagtacctatgattctattaaatcccgaataatcagacatctcaagaatt**

**Fortunella_japonica_3** **ttacaatgaccagtacctatgattctattaaatcccgaataatcagacatctcaagaatt**

**Fortunella_japonica_4** **ttacaatgaccagtacctatgattctattaaatcccgaataatcagacatctcaagaatt**

**Fortunella_japonica_5** **ttacaatgaccagtacctatgattctattaaatcccgaataatcagacatctcaagaatt**

**Fortunella_japonica_6** **ttacaatgaccagtacctatgattctattaaatcccgaataatcagacatctcaagaatt**

**Fortunella_japonica_7** **ttacaatgaccagtacctatgattctattaaatcccgaataatcagacatctcaagaatt**

**Fortunella_japonica_8** **ttacaatgaccagtacctatgattctattaaatcccgaataatcagacatctcaagaatt**

**Fortunella_japonica_9** **ttacaatgaccagtacctatgattctattaaatcccgaataatcagacatctcaagaatt**

**Fortunella_japonica_10** **ttacaatgaccagtacctatgattctattaaatcccgaataatcagacatctcaagaatt**

**Fortunella_japonica_11** **ttacaatgaccagtacctatgattctattaaatcccgaataatcagacatctcaagaatt**

**Fortunella_japonica_12** **ttacaatgaccagtacctatgattctattaaatcccgaataatcagacatctcaagaatt**

**Fortunella_margarita_1** **ttacaatgaccagtacctatgattctattaaatcccgaataatcagacatctcaagaatt**

**Fortunella_margarita_2** **ttacaatgaccagtacctatgattctattaaatcccgaataatcagacatctcaagaatt**

**Fortunella_venosa_1** **ttacaatgaccagtacctatgattctattaaatcccgaataatcagacatctcaagaatt**

**Fortunella_venosa_2** **ttacaatgaccagtacctatgattctattaaatcccgaataatcagacatctcaagaatt**

**Fortunella_venosa_3** **ttacaatgaccagtacctatgattctattaaatcccgaataatcagacatctcaagaatt**

21250	21260	21270	21280	21290	21300

....|....|....|....|....|....|....|....|....|....|....|....|

**Atalantia_kwangtungens** **ctacctttttttttagcagaaaaatcagaactaaaaaattggtggcccgcttgatcatta**

**Citrus_reticulata_1** **cgacctttttttttagcagaaaaatcagaactaaaaaatttgtgtcccgtttgatcatta**

**Citrus_reticulata_2** **cgacctttttttttagcagaaaaatcagaactaaaaaatttgtgtcccgtttgatcatta**

**Citrus_trifoliata_3** **cgacctttttttttagcagaaaaatcagaactaaaaaatttgtgtcccgtttgatcatta**

**Citrus_aurantiifolia** **cgacttttttttttagcagaaaaatcagaactaaaaaatttgtgtcccgtttgatcatta**

**Citrus_aurantium** **cgacctttttttttagcagaaaaatcagaactaaaaaatttgtgtcccgtttgatcatta**

**Citrus_cavaleriei** **cgacctttttttttagcagaaaaatcagaactaaaaaatttgtgtcccgtttgatcatta**

**Citrus_hongheensis** **cgacctttttttttagcagaaaaatcagaactaaaaaatttgtgtcccgtttgatcatta**

**Citrus_limon** **cgacctttttttttagcagaaaaatcagaactaaaaaatttgtgtcccgtttgatcatta**

**Citrus_maxima** **cgacctttttttttagcagaaaaatcagaactaaaaaatttgtgtcccgtttgatcatta**

**Citrus_medica** **cgacctttttttttagcagaaaaatcagaactaaaaaatttgtgtcccgtttgatcatta**

**Citrus_polytrifolia** **cgacctttttttttagcagaaaaatcagaactaaaaaatttgtgtcccgtttgatcatta**

**Citrus_reticulata** **cgacctttttttttagcagaaaaatcagaactaaaaaatttgtgtcccgtttgatcatta**

**Citrus_sinensis** **cgacctttttttttagcagaaaaatcagaactaaaaaatttgtgtcccgtttgatcatta**

**Fortunella_bawangica_1** **cgacctttttttttagcagaaaaatcagaactaaaaaatttgtgtcccgtttgatcatta**

**Fortunella_bawangica_2** **cgacctttttttttagcagaaaaatcagaactaaaaaatttgtgtcccgtttgatcatta**

**Fortunella_hindsii_1** **cgacctttttttttagcagaaaaatcagaactaaaaaatttgtgtcccgtttgatcatta**

**Fortunella_hindsii_2** **cgacctttttttttagcagaaaaatcagaactaaaaaatttgtgtcccgtttgatcatta**

**Fortunella_hindsii_3** **cgacctttttttttagcagaaaaatcagaactaaaaaatttgtgtcccgtttgatcatta**

**Fortunella_japonica_1** **cgacctttttttttagcagaaaaatcagaactaaaaaatttgtgtcccgtttgatcatta**

**Fortunella_japonica_2** **cgacctttttttttagcagaaaaatcagaactaaaaaatttgtgtcccgtttgatcatta**

**Fortunella_japonica_3** **cgacctttttttttagcagaaaaatcagaactaaaaaatttgtgtcccgtttgatcatta**

**Fortunella_japonica_4** **cgacctttttttttagcagaaaaatcagaactaaaaaatttgtgtcccgtttgatcatta**

**Fortunella_japonica_5** **cgacctttttttttagcagaaaaatcagaactaaaaaatttgtgtcccgtttgatcatta**

**Fortunella_japonica_6** **cgacctttttttttagcagaaaaatcagaactaaaaaatttgtgtcccgtttgatcatta**

**Fortunella_japonica_7** **cgacctttttttttagcagaaaaatcagaactaaaaaatttgtgtcccgtttgatcatta**

**Fortunella_japonica_8** **cgacctttttttttagcagaaaaatcagaactaaaaaatttgtgtcccgtttgatcatta**

**Fortunella_japonica_9** **cgacctttttttttagcagaaaaatcagaactaaaaaatttgtgtcccgtttgatcatta**

**Fortunella_japonica_10** **cgacctttttttttagcagaaaaatcagaactaaaaaatttgtgtcccgtttgatcatta**

**Fortunella_japonica_11** **cgacctttttttttagcagaaaaatcagaactaaaaaatttgtgtcccgtttgatcatta**

**Fortunella_japonica_12** **cgacctttttttttagcagaaaaatcagaactaaaaaatttgtgtcccgtttgatcatta**

**Fortunella_margarita_1** **cgacctttttttttagcagaaaaatcagaactaaaaaatttgtgtcccgtttgatcatta**

**Fortunella_margarita_2** **cgacctttttttttagcagaaaaatcagaactaaaaaatttgtgtcccgtttgatcatta**

**Fortunella_venosa_1** **cgacctttttttttagcagaaaaatcagaactaaaaaatttgtgtcccgtttgatcatta**

**Fortunella_venosa_2** **cgacctttttttttagcagaaaaatcagaactaaaaaatttgtgtcccgtttgatcatta**

**Fortunella_venosa_3** **cgacctttttttttagcagaaaaatcagaactaaaaaatttgtgtcccgtttgatcatta**

21310	21320	21330	21340	21350	21360

....|....|....|....|....|....|....|....|....|....|....|....|

**Atalantia_kwangtungens** **ttcactgagagcgagcggctagaaatctctcttcgttcgacagaaagagaatgaatattc**

**Citrus_reticulata_1** **ttcactgagagcgagaggctcgaaatctctcttcgttcgacagaaagagaatgaatattc**

**Citrus_reticulata_2** **ttcactgagagcgagaggctcgaaatctctcttcgttcgacagaaagagaatgaatattc**

**Citrus_trifoliata_3** **ttcactgagagcgagaggctagaaatctctcttcgttcgacagaaagagaatgaatattc**

**Citrus_aurantiifolia** **ttcactgagagcgagaggctagaaatctctcttcgttcgacagaaagagaatgaatattc**

**Citrus_aurantium** **ttcactgagagcgagaggctagaaatctctcttcgttcgacagaaagagaatgaatattc**

**Citrus_cavaleriei** **ttcactgagagcgagaggctcgaaatctctcttcgttcgacagaaagagaatgaatattc**

**Citrus_hongheensis** **ttcactgagagcgagaggctagaaatctctcttcgttcgacagaaagagaatgaatattc**

**Citrus_limon** **ttcactgagagcgagaggctagaaatctctcttcgttcgacagaaagagaatgaatattc**

**Citrus_maxima** **ttcactgagagcgagaggctagaaatctctcttcgttcgacagaaagagaatgaatattc**

**Citrus_medica** **ttcactgagagcgagaggctagaaatctctcttcgttcgacagaaagagaatgaatattc**

**Citrus_polytrifolia** **ttcactgagagcgagaggctagaaatctctcttcgttcgacagaaagagaatgaatattc**

**Citrus_reticulata** **ttcactgagagcgagaggctcgaaatctctcttcgttcgacagaaagagaatgaatattc**

**Citrus_sinensis** **ttcactgagagcgagaggctagaaatctctcttcgttcgacagaaagagaatgaatattc**

**Fortunella_bawangica_1** **ttcactgagagcgagaggctagaaatctctcttcgttcgacagaaagagaatgaatattc**

**Fortunella_bawangica_2** **ttcactgagagcgagaggctagaaatctctcttcgttcgacagaaagagaatgaatattc**

**Fortunella_hindsii_1** **ttcactgagagcgagaggctagaaatctctcttcgttcgacagaaagagaatgaatattc**

**Fortunella_hindsii_2** **ttcactgagagcgagaggctagaaatctctcttcgttcgacagaaagagaatgaatattc**

**Fortunella_hindsii_3** **ttcactgagagcgagaggctagaaatctctcttcgttcgacagaaagagaatgaatattc**

**Fortunella_japonica_1** **ttcactgagagcgagaggctagaaatctctcttcgttcgacagaaagagaatgaatattc**

**Fortunella_japonica_2** **ttcactgagagcgagaggctagaaatctctcttcgttcgacagaaagagaatgaatattc**

**Fortunella_japonica_3** **ttcactgagagcgagaggctagaaatctctcttcgttcgacagaaagagaatgaatattc**

**Fortunella_japonica_4** **ttcactgagagcgagaggctagaaatctctcttcgttcgacagaaagagaatgaatattc**

**Fortunella_japonica_5** **ttcactgagagcgagaggctagaaatctctcttcgttcgacagaaagagaatgaatattc**

**Fortunella_japonica_6** **ttcactgagagcgagaggctagaaatctctcttcgttcgacagaaagagaatgaatattc**

**Fortunella_japonica_7** **ttcactgagagcgagaggctagaaatctctcttcgttcgacagaaagagaatgaatattc**

**Fortunella_japonica_8** **ttcactgagagcgagaggctagaaatctctcttcgttcgacagaaagagaatgaatattc**

**Fortunella_japonica_9** **ttcactgagagcgagaggctagaaatctctcttcgttcgacagaaagagaatgaatattc**

**Fortunella_japonica_10** **ttcactgagagcgagaggctagaaatctctcttcgttcgacagaaagagaatgaatattc**

**Fortunella_japonica_11** **ttcactgagagcgagaggctagaaatctctcttcgttcgacagaaagagaatgaatattc**

**Fortunella_japonica_12** **ttcactgagagcgagaggctagaaatctctcttcgttcgacagaaagagaatgaatattc**

**Fortunella_margarita_1** **ttcactgagagcgagaggctagaaatctctcttcgttcgacagaaagagaatgaatattc**

**Fortunella_margarita_2** **ttcactgagagcgagaggctagaaatctctcttcgttcgacagaaagagaatgaatattc**

**Fortunella_venosa_1** **ttcactgagagcgagaggctagaaatctctcttcgttcgacagaaagagaatgaatattc**

**Fortunella_venosa_2** **ttcactgagagcgagaggctagaaatctctcttcgttcgacagaaagagaatgaatattc**

**Fortunella_venosa_3** **ttcactgagagcgagaggctagaaatctctcttcgttcgacagaaagagaatgaatattc**

21370	21380	21390	21400	21410	21420

....|....|....|....|....|....|....|....|....|....|....|....|

**Atalantia_kwangtungens** **atttgatcttgatccttgtggagtgaaaaagaaactacactagatctgcgtgaacccccc**

**Citrus_reticulata_1** **atttgatcttgatccttgtggagtgaaaaagaaactacactagatctgcgtgaacccccc**

**Citrus_reticulata_2** **atttgatcttgatccttgtggagtgaaaaagaaactacactagatctgcgtgaacccccc**

**Citrus_trifoliata_3** **atttgatcttgatccttgtggagtgaaaaagaaactacactagatctgcgtgaacccccc**

**Citrus_aurantiifolia** **atttgatcttgatccttgtggagtgaaaaagaaactacactagatctgcgtgaacccccc**

**Citrus_aurantium** **atttgatcttgatccttgtggagtgaaaaagaaactacactagatctgcgtgaacccccc**

**Citrus_cavaleriei** **atttgatcttgatccttgtggagtgaaaaagaaactacactagatctgcgtgaacccccc**

**Citrus_hongheensis** **atttgatcttgatccttgtggagtgaaaaagaaactacactagatctgcgtgaacccccc**

**Citrus_limon** **atttgatcttgatccttgtggagtgaaaaagaaactacactagatctgcgtgaacccccc**

**Citrus_maxima** **atttgatcttgatccttgtggagtgaaaaagaaactacactagatctgcgtgaacccccc**

**Citrus_medica** **atttgatcttgatccttgtggagtgaaaaagaaactacactagatctgcgtgaacccccc**

**Citrus_polytrifolia** **atttgatcttgatccttgtggagtgaaaaagaaactacactagatctgcgtgaacccccc**

**Citrus_reticulata** **atttgatcttgatccttgtggagtgaaaaagaaactacactagatctgcgtgaacccccc**

**Citrus_sinensis** **atttgatcttgatccttgtggagtgaaaaagaaactacactagatctgcgtgaacccccc**

**Fortunella_bawangica_1** **atttgatcttgatccttgtggagtgaaaaagaaactacactagatctgcgtgaacccccc**

**Fortunella_bawangica_2** **atttgatcttgatccttgtggagtgaaaaagaaactacactagatctgcgtgaacccccc**

**Fortunella_hindsii_1** **atttgatcttgatccttgtggagtgaaaaagaaactacactagatctgcgtgaacccccc**

**Fortunella_hindsii_2** **atttgatcttgatccttgtggagtgaaaaagaaactacactagatctgcgtgaacccccc**

**Fortunella_hindsii_3** **atttgatcttgatccttgtggagtgaaaaagaaactacactagatctgcgtgaacccccc**

**Fortunella_japonica_1** **atttgatcttgatccttgtggagtgaaaaagaaactacactagatctgcgtgaacccccc**

**Fortunella_japonica_2** **atttgatcttgatccttgtggagtgaaaaagaaactacactagatctgcgtgaacccccc**

**Fortunella_japonica_3** **atttgatcttgatccttgtggagtgaaaaagaaactacactagatctgcgtgaacccccc**

**Fortunella_japonica_4** **atttgatcttgatccttgtggagtgaaaaagaaactacactagatctgcgtgaacccccc**

**Fortunella_japonica_5** **atttgatcttgatccttgtggagtgaaaaagaaactacactagatctgcgtgaacccccc**

**Fortunella_japonica_6** **atttgatcttgatccttgtggagtgaaaaagaaactacactagatctgcgtgaacccccc**

**Fortunella_japonica_7** **atttgatcttgatccttgtggagtgaaaaagaaactacactagatctgcgtgaacccccc**

**Fortunella_japonica_8** **atttgatcttgatccttgtggagtgaaaaagaaactacactagatctgcgtgaacccccc**

**Fortunella_japonica_9** **atttgatcttgatccttgtggagtgaaaaagaaactacactagatctgcgtgaacccccc**

**Fortunella_japonica_10** **atttgatcttgatccttgtggagtgaaaaagaaactacactagatctgcgtgaacccccc**

**Fortunella_japonica_11** **atttgatcttgatccttgtggagtgaaaaagaaactacactagatctgcgtgaacccccc**

**Fortunella_japonica_12** **atttgatcttgatccttgtggagtgaaaaagaaactacactagatctgcgtgaacccccc**

**Fortunella_margarita_1** **atttgatcttgatccttgtggagtgaaaaagaaactacactagatctgcgtgaacccccc**

**Fortunella_margarita_2** **atttgatcttgatccttgtggagtgaaaaagaaactacactagatctgcgtgaacccccc**

**Fortunella_venosa_1** **atttgatcttgatccttgtggagtgaaaaagaaactacactagatctgcgtgaacccccc**

**Fortunella_venosa_2** **atttgatcttgatccttgtggagtgaaaaagaaactacactagatctgcgtgaacccccc**

**Fortunella_venosa_3** **atttgatcttgatccttgtggagtgaaaaagaaactacactagatctgcgtgaacccccc**

21430	21440	21450	21460	21470	21480

....|....|....|....|....|....|....|....|....|....|....|....|

**Atalantia_kwangtungens** **gacaatatccataaatggcttgtttttggcaagaggtggacattactatatgtaaattcg**

**Citrus_reticulata_1** **gacaatatccataaatggcttgtttttggcaagaggtggacattactatatgtaaattcg**

**Citrus_reticulata_2** **gacaatatccataaatggcttgtttttggcaagaggtggacattactatatgtaaattcg**

**Citrus_trifoliata_3** **gacaatatccataaatggcttgtttttggcaagaggtggacattactatatgtaaattcg**

**Citrus_aurantiifolia** **gacaatatccataaatggcttgtttttggcaagaggtggacattactatatgtaaattcg**

**Citrus_aurantium** **gacaatatccataaatggcttgtttttggcaagaggtggacattactatatgtaaattcg**

**Citrus_cavaleriei** **gacaatatccataaatggcttgtttttggcaagaggtggacattactatatgtaaattcg**

**Citrus_hongheensis** **gacaatatccataaatggcttgtttttggcaagaggtggacattactatatgtaaattcg**

**Citrus_limon** **gacaatatccataaatggcttgtttttggcaagaggtggacattactatatgtaaattcg**

**Citrus_maxima** **gacaatatccataaatggcttgtttttggcaagaggtggacattactatatgtaaattcg**

**Citrus_medica** **gacaatatccataaatggcttgtttttggcaagaggtggacattactatatgtaaattcg**

**Citrus_polytrifolia** **gacaatatccataaatggcttgtttttggcaagaggtggacattactatatgtaaattcg**

**Citrus_reticulata** **gacaatatccataaatggcttgtttttggcaagaggtggacattactatatgtaaattcg**

**Citrus_sinensis** **gacaatatccataaatggcttgtttttggcaagaggtggacattactatatgtaaattcg**

**Fortunella_bawangica_1** **gacaatatccataaatggcttgtttttggcaagaggtggacattactatatgtaaattcg**

**Fortunella_bawangica_2** **gacaatatccataaatggcttgtttttggcaagaggtggacattactatatgtaaattcg**

**Fortunella_hindsii_1** **gacaatatccataaatggcttgtttttggcaagaggtggacattactatatataaattcg**

**Fortunella_hindsii_2** **gacaatatccataaatggcttgtttttggcaagaggtggacattactatatataaattcg**

**Fortunella_hindsii_3** **gacaatatccataaatggcttgtttttggcaagaggtggacattactatatataaattcg**

**Fortunella_japonica_1** **gacaatatccataaatggcttgtttttggcaagaggtggacattactatatgtaaattcg**

**Fortunella_japonica_2** **gacaatatccataaatggcttgtttttggcaagaggtggacattactatatgtaaattcg**

**Fortunella_japonica_3** **gacaatatccataaatggcttgtttttggcaagaggtggacattactatatgtaaattcg**

**Fortunella_japonica_4** **gacaatatccataaatggcttgtttttggcaagaggtggacattactatatgtaaattcg**

**Fortunella_japonica_5** **gacaatatccataaatggcttgtttttggcaagaggtggacattactatatgtaaattcg**

**Fortunella_japonica_6** **gacaatatccataaatggcttgtttttggcaagaggtggacattactatatgtaaattcg**

**Fortunella_japonica_7** **gacaatatccataaatggcttgtttttggcaagaggtggacattactatatgtaaattcg**

**Fortunella_japonica_8** **gacaatatccataaatggcttgtttttggcaagaggtggacattactatatgtaaattcg**

**Fortunella_japonica_9** **gacaatatccataaatggcttgtttttggcaagaggtggacattactatatgtaaattcg**

**Fortunella_japonica_10** **gacaatatccataaatggcttgtttttggcaagaggtggacattactatatgtaaattcg**

**Fortunella_japonica_11** **gacaatatccataaatggcttgtttttggcaagaggtggacattactatatgtaaattcg**

**Fortunella_japonica_12** **gacaatatccataaatggcttgtttttggcaagaggtggacattactatatgtaaattcg**

**Fortunella_margarita_1** **gacaatatccataaatggcttgtttttggcaagaggtggacattactatatgtaaattcg**

**Fortunella_margarita_2** **gacaatatccataaatggcttgtttttggcaagaggtggacattactatatgtaaattcg**

**Fortunella_venosa_1** **gacaatatccataaatggcttgtttttggcaagaggtggacattactatatataaattcg**

**Fortunella_venosa_2** **gacaatatccataaatggcttgtttttggcaagaggtggacattactatatataaattcg**

**Fortunella_venosa_3** **gacaatatccataaatggcttgtttttggcaagaggtggacattactatatataaattcg**

21490	21500	21510	21520	21530	21540

....|....|....|....|....|....|....|....|....|....|....|....|

**Atalantia_kwangtungens** **ggtgcatggtacacatcagtactccagtgcatttctccctctgaatcagaataaatatgt**

**Citrus_reticulata_1** **ggtgcatggtacacatcagtactccagtgcatttctccctctgaatcagaataaatatgt**

**Citrus_reticulata_2** **ggtgcatggtacacatcagtactccagtgcatttctccctctgaatcagaataaatatgt**

**Citrus_trifoliata_3** **ggtgcatggtacacatcagtactccagtgcatttctccctctgaatcagaataaatatgt**

**Citrus_aurantiifolia** **ggtgcatggtacacatcagtactccagtgcatttctccctctgaatcagaataaatatgt**

**Citrus_aurantium** **ggtgcatggtacacatcagtactccagtgcatttctccctctgaatcagaataaatatgt**

**Citrus_cavaleriei** **ggtgcatggtacacatcagtactccagtgcatttctccctctgaatcagaataaatatgt**

**Citrus_hongheensis** **ggtgcatggtacacatcagtactccagtgcatttctccctctgaatcagaataaatatgt**

**Citrus_limon** **ggtgcatggtacacatcagtactccagtgcatttctccctctgaatcagaataaatatgt**

**Citrus_maxima** **ggtgcatggtacacatcagtactccagtgcatttctccctctgaatcagaataaatatgt**

**Citrus_medica** **ggtgcatggtacacatcagtactccagtgcatttctccctctgaatcagaataaatatgt**

**Citrus_polytrifolia** **ggtgcatggtacacatcagtactccagtgcatttctccctctgaatcagaataaatatgt**

**Citrus_reticulata** **ggtgcatggtacacatcagtactccagtgcatttctccctctgaatcagaataaatatgt**

**Citrus_sinensis** **ggtgcatggtacacatcagtactccagtgcatttctccctctgaatcagaataaatatgt**

**Fortunella_bawangica_1** **ggtgcatggtacacatcagtactccagtgcatttctccctctgaatcagaataaatatgt**

**Fortunella_bawangica_2** **ggtgcatggtacacatcagtactccagtgcatttctccctctgaatcagaataaatatgt**

**Fortunella_hindsii_1** **ggtgcatggtacacatcagtactccagtgcatttctccctctgaatcagaataaatatgt**

**Fortunella_hindsii_2** **ggtgcatggtacacatcagtactccagtgcatttctccctctgaatcagaataaatatgt**

**Fortunella_hindsii_3** **ggtgcatggtacacatcagtactccagtgcatttctccctctgaatcagaataaatatgt**

**Fortunella_japonica_1** **ggtgcatggtacacatcagtactccagtgcatttctccctctgaatcagaataaatatgt**

**Fortunella_japonica_2** **ggtgcatggtacacatcagtactccagtgcatttctccctctgaatcagaataaatatgt**

**Fortunella_japonica_3** **ggtgcatggtacacatcagtactccagtgcatttctccctctgaatcagaataaatatgt**

**Fortunella_japonica_4** **ggtgcatggtacacatcagtactccagtgcatttctccctctgaatcagaataaatatgt**

**Fortunella_japonica_5** **ggtgcatggtacacatcagtactccagtgcatttctccctctgaatcagaataaatatgt**

**Fortunella_japonica_6** **ggtgcatggtacacatcagtactccagtgcatttctccctctgaatcagaataaatatgt**

**Fortunella_japonica_7** **ggtgcatggtacacatcagtactccagtgcatttctccctctgaatcagaataaatatgt**

**Fortunella_japonica_8** **ggtgcatggtacacatcagtactccagtgcatttctccctctgaatcagaataaatatgt**

**Fortunella_japonica_9** **ggtgcatggtacacatcagtactccagtgcatttctccctctgaatcagaataaatatgt**

**Fortunella_japonica_10** **ggtgcatggtacacatcagtactccagtgcatttctccctctgaatcagaataaatatgt**

**Fortunella_japonica_11** **ggtgcatggtacacatcagtactccagtgcatttctccctctgaatcagaataaatatgt**

**Fortunella_japonica_12** **ggtgcatggtacacatcagtactccagtgcatttctccctctgaatcagaataaatatgt**

**Fortunella_margarita_1** **ggtgcatggtacacatcagtactccagtgcatttctccctctgaatcagaataaatatgt**

**Fortunella_margarita_2** **ggtgcatggtacacatcagtactccagtgcatttctccctctgaatcagaataaatatgt**

**Fortunella_venosa_1** **ggtgcatggtacacatcagtactccagtgcatttctccctctgaatcagaataaatatgt**

**Fortunella_venosa_2** **ggtgcatggtacacatcagtactccagtgcatttctccctctgaatcagaataaatatgt**

**Fortunella_venosa_3** **ggtgcatggtacacatcagtactccagtgcatttctccctctgaatcagaataaatatgt**

21550	21560	21570	21580	21590	21600

....|....|....|....|....|....|....|....|....|....|....|....|

**Atalantia_kwangtungens** **tttcgaaccctctctttaaaattcaaagtgtatgctcccgcccgaatctcagcaatcact**

**Citrus_reticulata_1** **tttcgaaccctctctttaaaattcaaagtgtatgctcccgcccgaatctcagcaatcact**

**Citrus_reticulata_2** **tttcgaaccctctctttaaaattcaaagtgtatgctcccgcccgaatctcagcaatcact**

**Citrus_trifoliata_3** **tttcgaaccctctctttaaaattcaaagtgtatgctcccgcccgaatctcagcaatcact**

**Citrus_aurantiifolia** **tttcgaaccctctctttaaaattcaaagtgtatgctcccgcccgaatctcagcaatcact**

**Citrus_aurantium** **tttcgaaccctctctttaaaattcaaagtgtatgctcccgcccgaatctcagcaatcact**

**Citrus_cavaleriei** **tttcgaaccctctctttaaaattcaaagtgtatgctcccgcccgaatctcagcaatcact**

**Citrus_hongheensis** **tttcgaaccctctctttaaaattcaaagtgtatgctcccgcccgaatctcagcaatcact**

**Citrus_limon** **tttcgaaccctctctttaaaattcaaagtgtatgctcccgcccgaatctcagcaatcact**

**Citrus_maxima** **tttcgaaccctctctttaaaattcaaagtgtatgctcccgcccgaatctcagcaatcact**

**Citrus_medica** **tttcgaaccctctctttaaaattcaaagtgtatgctcccgcccgaatctcagcaatcact**

**Citrus_polytrifolia** **tttcgaaccctctctttaaaattcaaagtgtatgctcccgcccgaatctcagcaatcact**

**Citrus_reticulata** **tttcgaaccctctctttaaaattcaaagtgtatgctcccgcccgaatctcagcaatcact**

**Citrus_sinensis** **tttcgaaccctctctttaaaattcaaagtgtatgctcccgcccgaatctcagcaatcact**

**Fortunella_bawangica_1** **tttcgaaccctctctttaaaattcaaagtgtatgctcccgcccgaatctcagcaatcact**

**Fortunella_bawangica_2** **tttcgaaccctctctttaaaattcaaagtgtatgctcccgcccgaatctcagcaatcact**

**Fortunella_hindsii_1** **tttcgaaccctctctttaaaattcaaagtgtatgctcccgcccgaatctcagcaatcact**

**Fortunella_hindsii_2** **tttcgaaccctctctttaaaattcaaagtgtatgctcccgcccgaatctcagcaatcact**

**Fortunella_hindsii_3** **tttcgaaccctctctttaaaattcaaagtgtatgctcccgcccgaatctcagcaatcact**

**Fortunella_japonica_1** **tttcgaaccctctctttaaaattcaaagtgtatgctcccgcccgaatctcagcaatcact**

**Fortunella_japonica_2** **tttcgaaccctctctttaaaattcaaagtgtatgctcccgcccgaatctcagcaatcact**

**Fortunella_japonica_3** **tttcgaaccctctctttaaaattcaaagtgtatgctcccgcccgaatctcagcaatcact**

**Fortunella_japonica_4** **tttcgaaccctctctttaaaattcaaagtgtatgctcccgcccgaatctcagcaatcact**

**Fortunella_japonica_5** **tttcgaaccctctctttaaaattcaaagtgtatgctcccgcccgaatctcagcaatcact**

**Fortunella_japonica_6** **tttcgaaccctctctttaaaattcaaagtgtatgctcccgcccgaatctcagcaatcact**

**Fortunella_japonica_7** **tttcgaaccctctctttaaaattcaaagtgtatgctcccgcccgaatctcagcaatcact**

**Fortunella_japonica_8** **tttcgaaccctctctttaaaattcaaagtgtatgctcccgcccgaatctcagcaatcact**

**Fortunella_japonica_9** **tttcgaaccctctctttaaaattcaaagtgtatgctcccgcccgaatctcagcaatcact**

**Fortunella_japonica_10** **tttcgaaccctctctttaaaattcaaagtgtatgctcccgcccgaatctcagcaatcact**

**Fortunella_japonica_11** **tttcgaaccctctctttaaaattcaaagtgtatgctcccgcccgaatctcagcaatcact**

**Fortunella_japonica_12** **tttcgaaccctctctttaaaattcaaagtgtatgctcccgcccgaatctcagcaatcact**

**Fortunella_margarita_1** **tttcgaaccctctctttaaaattcaaagtgtatgctcccgcccgaatctcagcaatcact**

**Fortunella_margarita_2** **tttcgaaccctctctttaaaattcaaagtgtatgctcccgcccgaatctcagcaatcact**

**Fortunella_venosa_1** **tttcgaaccctctctttaaaattcaaagtgtatgctcccgcccgaatctcagcaatcact**

**Fortunella_venosa_2** **tttcgaaccctctctttaaaattcaaagtgtatgctcccgcccgaatctcagcaatcact**

**Fortunella_venosa_3** **tttcgaaccctctctttaaaattcaaagtgtatgctcccgcccgaatctcagcaatcact**

21610	21620	21630	21640	21650	21660

....|....|....|....|....|....|....|....|....|....|....|....|

**Atalantia_kwangtungens** **tgttctgattcgacatattgatcattttgaactaaaagaaaacttttaggtggaatagtc**

**Citrus_reticulata_1** **tgttctgattcgacatattgatcattttgaactaaaagaaaactttttggtggaatagtc**

**Citrus_reticulata_2** **tgttctgattcgacatattgatcattttgaactaaaagaaaactttttggtggaatagtc**

**Citrus_trifoliata_3** **tgttctgattcgacatattgatcattttgaactaaaagaaaactttttggtggaatagtc**

**Citrus_aurantiifolia** **tgttctgattcgacatattgatcattttgaactaaaagaaaactttttggtggaatagtc**

**Citrus_aurantium** **tgttctgattcgacatattgatcattttgaactaaaagaaaactttttggtggaatagtc**

**Citrus_cavaleriei** **tgttctgattcgacatattgatcattttgaactaaaagaaaactttttggtggaatagtc**

**Citrus_hongheensis** **tgttctgattcgacatattgatcattttgaactaaaagaaaactttttggtggaatagtc**

**Citrus_limon** **tgttctgattcgacatattgatcattttgaactaaaagaaaactttttggtggaatagtc**

**Citrus_maxima** **tgttctgattcgacatattgatcattttgaactaaaagaaaactttttggtggaatagtc**

**Citrus_medica** **tgttctgattcgacatattgatcattttgaactaaaagaaaactttttggtggaatagtc**

**Citrus_polytrifolia** **tgttctgattcgacatattgatcattttgaactaaaagaaaactttttggtggaatagtc**

**Citrus_reticulata** **tgttctgattcgacatattgatcattttgaactaaaagaaaactttttggtggaatagtc**

**Citrus_sinensis** **tgttctgattcgacatattgatcattttgaactaaaagaaaactttttggtggaatagtc**

**Fortunella_bawangica_1** **tgttctgattcgacatattgatcattttgaactaaaagaaaactttttggtggaatagtc**

**Fortunella_bawangica_2** **tgttctgattcgacatattgatcattttgaactaaaagaaaactttttggtggaatagtc**

**Fortunella_hindsii_1** **tgttctgattcgacatattgatcattttgaactaaaagaaaactttttggtggaatagtc**

**Fortunella_hindsii_2** **tgttctgattcgacatattgatcattttgaactaaaagaaaactttttggtggaatagtc**

**Fortunella_hindsii_3** **tgttctgattcgacatattgatcattttgaactaaaagaaaactttttggtggaatagtc**

**Fortunella_japonica_1** **tgttctgattcgacatattgatcattttgaactaaaagaaaactttttggtggaatagtc**

**Fortunella_japonica_2** **tgttctgattcgacatattgatcattttgaactaaaagaaaactttttggtggaatagtc**

**Fortunella_japonica_3** **tgttctgattcgacatattgatcattttgaactaaaagaaaactttttggtggaatagtc**

**Fortunella_japonica_4** **tgttctgattcgacatattgatcattttgaactaaaagaaaactttttggtggaatagtc**

**Fortunella_japonica_5** **tgttctgattcgacatattgatcattttgaactaaaagaaaactttttggtggaatagtc**

**Fortunella_japonica_6** **tgttctgattcgacatattgatcattttgaactaaaagaaaactttttggtggaatagtc**

**Fortunella_japonica_7** **tgttctgattcgacatattgatcattttgaactaaaagaaaactttttggtggaatagtc**

**Fortunella_japonica_8** **tgttctgattcgacatattgatcattttgaactaaaagaaaactttttggtggaatagtc**

**Fortunella_japonica_9** **tgttctgattcgacatattgatcattttgaactaaaagaaaactttttggtggaatagtc**

**Fortunella_japonica_10** **tgttctgattcgacatattgatcattttgaactaaaagaaaactttttggtggaatagtc**

**Fortunella_japonica_11** **tgttctgattcgacatattgatcattttgaactaaaagaaaactttttggtggaatagtc**

**Fortunella_japonica_12** **tgttctgattcgacatattgatcattttgaactaaaagaaaactttttggtggaatagtc**

**Fortunella_margarita_1** **tgttctgattcgacatattgatcattttgaactaaaagaaaactttttggtggaatagtc**

**Fortunella_margarita_2** **tgttctgattcgacatattgatcattttgaactaaaagaaaactttttggtggaatagtc**

**Fortunella_venosa_1** **tgttctgattcgacatattgatcattttgaactaaaagaaaactttttggtggaatagtc**

**Fortunella_venosa_2** **tgttctgattcgacatattgatcattttgaactaaaagaaaactttttggtggaatagtc**

**Fortunella_venosa_3** **tgttctgattcgacatattgatcattttgaactaaaagaaaactttttggtggaatagtc**

21670	21680	21690	21700	21710	21720

....|....|....|....|....|....|....|....|....|....|....|....|

**Atalantia_kwangtungens** **acattgtgcataatatcttcaccctcaataattacatccaaatctatagaacatagaaaa**

**Citrus_reticulata_1** **acattgtgcataatatcttcaccctcaataattacatccaaatctatagaacatagaaaa**

**Citrus_reticulata_2** **acattgtgcataatatcttcaccctcaataattacatccaaatctatagaacatagaaaa**

**Citrus_trifoliata_3** **acattgtgcataatatcttcaccctcaataattacatccaaatctatagaacatagaaaa**

**Citrus_aurantiifolia** **acattgtgcataatatcttcaccctcaataattacatccaaatctatagaacatagaaaa**

**Citrus_aurantium** **acattgtgcataatatcttcaccctcaataattacatccaaatctatagaacatagaaaa**

**Citrus_cavaleriei** **acattgtgcataatatcttcaccctcaataattacatccaaatctatagaacatagaaaa**

**Citrus_hongheensis** **acattgtgcataatatcttcaccctcaataattacatccaaatctatagaacatagaaaa**

**Citrus_limon** **acattgtgcataatatcttcaccctcaataattacatccaaatctatagaacatagaaaa**

**Citrus_maxima** **acattgtgcataatatcttcaccctcaataattacatccaaatctatagaacatagaaaa**

**Citrus_medica** **acattgtgcataatatcttcaccctcaataattacatccaaatctatagaacatagaaaa**

**Citrus_polytrifolia** **acattgtgcataatatcttcaccctcaataattacatccaaatctatagaacatagaaaa**

**Citrus_reticulata** **acattgtgcataatatcttcaccctcaataattacatccaaatctatagaacatagaaaa**

**Citrus_sinensis** **acattgtgcataatatcttcaccctcaataattacatccaaatctatagaacatagaaaa**

**Fortunella_bawangica_1** **acattgtgcataatatcttcaccctcaataattacatccaaatctatagaacatagaaaa**

**Fortunella_bawangica_2** **acattgtgcataatatcttcaccctcaataattacatccaaatctatagaacatagaaaa**

**Fortunella_hindsii_1** **acattgtgcataatatcttcaccctcaataattacatccaaatctatagaacatagaaaa**

**Fortunella_hindsii_2** **acattgtgcataatatcttcaccctcaataattacatccaaatctatagaacatagaaaa**

**Fortunella_hindsii_3** **acattgtgcataatatcttcaccctcaataattacatccaaatctatagaacatagaaaa**

**Fortunella_japonica_1** **acattgtgcataatatcttcaccctcaataattacatccaaatctatagaacatagaaaa**

**Fortunella_japonica_2** **acattgtgcataatatcttcaccctcaataattacatccaaatctatagaacatagaaaa**

**Fortunella_japonica_3** **acattgtgcataatatcttcaccctcaataattacatccaaatctatagaacatagaaaa**

**Fortunella_japonica_4** **acattgtgcataatatcttcaccctcaataattacatccaaatctatagaacatagaaaa**

**Fortunella_japonica_5** **acattgtgcataatatcttcaccctcaataattacatccaaatctatagaacatagaaaa**

**Fortunella_japonica_6** **acattgtgcataatatcttcaccctcaataattacatccaaatctatagaacatagaaaa**

**Fortunella_japonica_7** **acattgtgcataatatcttcaccctcaataattacatccaaatctatagaacatagaaaa**

**Fortunella_japonica_8** **acattgtgcataatatcttcaccctcaataattacatccaaatctatagaacatagaaaa**

**Fortunella_japonica_9** **acattgtgcataatatcttcaccctcaataattacatccaaatctatagaacatagaaaa**

**Fortunella_japonica_10** **acattgtgcataatatcttcaccctcaataattacatccaaatctatagaacatagaaaa**

**Fortunella_japonica_11** **acattgtgcataatatcttcaccctcaataattacatccaaatctatagaacatagaaaa**

**Fortunella_japonica_12** **acattgtgcataatatcttcaccctcaataattacatccaaatctatagaacatagaaaa**

**Fortunella_margarita_1** **acattgtgcataatatcttcaccctcaataattacatccaaatctatagaacatagaaaa**

**Fortunella_margarita_2** **acattgtgcataatatcttcaccctcaataattacatccaaatctatagaacatagaaaa**

**Fortunella_venosa_1** **acattgtgcataatatcttcaccctcaataattacatccaaatctatagaacatagaaaa**

**Fortunella_venosa_2** **acattgtgcataatatcttcaccctcaataattacatccaaatctatagaacatagaaaa**

**Fortunella_venosa_3** **acattgtgcataatatcttcaccctcaataattacatccaaatctatagaacatagaaaa**

21730	21740	21750	21760	21770	21780

....|....|....|....|....|....|....|....|....|....|....|....|

**Atalantia_kwangtungens** **gccggatgcccgtgacgtgtgcgcgtgggatgaaccaaatcctcattgaatttgatttta**

**Citrus_reticulata_1** **gccggatgcccgtgacgtgtgcgcgtgggatgaaccaaatcctcattgaatttgatttta**

**Citrus_reticulata_2** **gccggatgcccgtgacgtgtgcgcgtgggatgaaccaaatcctcattgaatttgatttta**

**Citrus_trifoliata_3** **gccggatgcccgtgacgtgtgcgcgtgggatgaaccaaatcctcattgaatttgatttta**

**Citrus_aurantiifolia** **gccggatgcccgtgacgtgtgcgcgtgggatgaaccaaatcctcattgaatttgatttta**

**Citrus_aurantium** **gccggatgcccgtgacgtgtgcgcgtgggatgaaccaaatcctcattgaatttgatttta**

**Citrus_cavaleriei** **gccggatgcccgtgacgtgtgcgcgtgggatgaaccaaatcctcattgaatttgatttta**

**Citrus_hongheensis** **gccggatgcccgtgacgtgtgcgcgtgggatgaaccaaatcctcattgaatttgatttta**

**Citrus_limon** **gccggatgcccgtgacgtgtgcgcgtgggatgaaccaaatcctcattgaatttgatttta**

**Citrus_maxima** **gccggatgcccgtgacgtgtgcgcgtgggatgaaccaaatcctcattgaatttgatttta**

**Citrus_medica** **gccggatgcccgtgacgtgtgcgcgtgggatgaaccaaatcctcattgaattttatttta**

**Citrus_polytrifolia** **gccggatgcccgtgacgtgtgcgcgtgggatgaaccaaatcctcattgaatttgatttta**

**Citrus_reticulata** **gccggatgcccgtgacgtgtgcgcgtgggatgaaccaaatcctcattgaatttgatttta**

**Citrus_sinensis** **gccggatgcccgtgacgtgtgcgcgtgggatgaaccaaatcctcattgaatttgatttta**

**Fortunella_bawangica_1** **gccggatgcccgtgacgtgtgcgcgtgggatgaaccaaatcctcattgaatttgatttta**

**Fortunella_bawangica_2** **gccggatgcccgtgacgtgtgcgcgtgggatgaaccaaatcctcattgaatttgatttta**

**Fortunella_hindsii_1** **gccggatgcccgtgacgtgtgcgcgtgggatgaaccaaatcctcattgaatttgatttta**

**Fortunella_hindsii_2** **gccggatgcccgtgacgtgtgcgcgtgggatgaaccaaatcctcattgaatttgatttta**

**Fortunella_hindsii_3** **gccggatgcccgtgacgtgtgcgcgtgggatgaaccaaatcctcattgaatttgatttta**

**Fortunella_japonica_1** **gccggatgcccgtgacgtgtgcgcgtgggatgaaccaaatcctcattgaatttgatttta**

**Fortunella_japonica_2** **gccggatgcccgtgacgtgtgcgcgtgggatgaaccaaatcctcattgaatttgatttta**

**Fortunella_japonica_3** **gccggatgcccgtgacgtgtgcgcgtgggatgaaccaaatcctcattgaatttgatttta**

**Fortunella_japonica_4** **gccggatgcccgtgacgtgtgcgcgtgggatgaaccaaatcctcattgaatttgatttta**

**Fortunella_japonica_5** **gccggatgcccgtgacgtgtgcgcgtgggatgaaccaaatcctcattgaatttgatttta**

**Fortunella_japonica_6** **gccggatgcccgtgacgtgtgcgcgtgggatgaaccaaatcctcattgaatttgatttta**

**Fortunella_japonica_7** **gccggatgcccgtgacgtgtgcgcgtgggatgaaccaaatcctcattgaatttgatttta**

**Fortunella_japonica_8** **gccggatgcccgtgacgtgtgcgcgtgggatgaaccaaatcctcattgaatttgatttta**

**Fortunella_japonica_9** **gccggatgcccgtgacgtgtgcgcgtgggatgaaccaaatcctcattgaatttgatttta**

**Fortunella_japonica_10** **gccggatgcccgtgacgtgtgcgcgtgggatgaaccaaatcctcattgaatttgatttta**

**Fortunella_japonica_11** **gccggatgcccgtgacgtgtgcgcgtgggatgaaccaaatcctcattgaatttgatttta**

**Fortunella_japonica_12** **gccggatgcccgtgacgtgtgcgcgtgggatgaaccaaatcctcattgaatttgatttta**

**Fortunella_margarita_1** **gccggatgcccgtgacgtgtgcgcgtgggatgaaccaaatcctcattgaatttgatttta**

**Fortunella_margarita_2** **gccggatgcccgtgacgtgtgcgcgtgggatgaaccaaatcctcattgaatttgatttta**

**Fortunella_venosa_1** **gccggatgcccgtgacgtgtgcgcgtgggatgaaccaaatcctcattgaatttgatttta**

**Fortunella_venosa_2** **gccggatgcccgtgacgtgtgcgcgtgggatgaaccaaatcctcattgaatttgatttta**

**Fortunella_venosa_3** **gccggatgcccgtgacgtgtgcgcgtgggatgaaccaaatcctcattgaatttgatttta**

21790	21800	21810	21820	21830	21840

....|....|....|....|....|....|....|....|....|....|....|....|

**Atalantia_kwangtungens** **ccattaaaaggggctcgtacatgttctgcagtgccccctgtaaatacgccgccggtatga**

**Citrus_reticulata_1** **ccattagaaggggctcgtacatgttctgcagtgccccccgtaaatacgccgccggtatga**

**Citrus_reticulata_2** **ccattagaaggggctcgtacatgttctgcagtgccccccgtaaatacgccgccggtatga**

**Citrus_trifoliata_3** **ccattagaaggggctcgtacatgttctgcagtgccccccgtaaatacgccgccggtatga**

**Citrus_aurantiifolia** **ccattagaaggggctcgtacatgttctgcagtgccccccgtaaatacgccgccggtatga**

**Citrus_aurantium** **ccattagaaggggctcgtacatgttctgcagtgccccccgtaaatacgccgccggtatga**

**Citrus_cavaleriei** **ccattagaaggggctcgtacatgttctgcagtgccccccgtaaatacgccgccggtatga**

**Citrus_hongheensis** **ccattagaaggggctcgtacatgttctgcagtgccccccgtaaatacgccgccggtatga**

**Citrus_limon** **ccattagaaggggctcgtacatgttctgcagtgccccccgtaaatacgccgccggtatga**

**Citrus_maxima** **ccattagaaggggctcgtacatgttctgcagtgccccccgtaaatacgccgccggtatga**

**Citrus_medica** **ccattagaaggggctcgtacatgttctgcagtgccccccgtaaatacgccgccggtatga**

**Citrus_polytrifolia** **ccattagaaggggctcgtacatgttctgcagtgccccccgtaaatacgccgccggtatga**

**Citrus_reticulata** **ccattagaaggggctcgtacatgttctgcagtgccccccgtaaatacgccgccggtatga**

**Citrus_sinensis** **ccattagaaggggctcgtacatgttctgcagtgccccccgtaaatacgccgccggtatga**

**Fortunella_bawangica_1** **ccattagaaggggctcgtacatgttctgcagtgccccccgtaaatacgccgccggtatga**

**Fortunella_bawangica_2** **ccattagaaggggctcgtacatgttctgcagtgccccccgtaaatacgccgccggtatga**

**Fortunella_hindsii_1** **ccattagaaggggctcgtacatgttctgcagtgccccccgtaaatacgccgccggtatga**

**Fortunella_hindsii_2** **ccattagaaggggctcgtacatgttctgcagtgccccccgtaaatacgccgccggtatga**

**Fortunella_hindsii_3** **ccattagaaggggctcgtacatgttctgcagtgccccccgtaaatacgccgccggtatga**

**Fortunella_japonica_1** **ccattagaaggggctcgtacatgttctgcagtgccccccgtaaatacgccgccggtatga**

**Fortunella_japonica_2** **ccattagaaggggctcgtacatgttctgcagtgccccccgtaaatacgccgccggtatga**

**Fortunella_japonica_3** **ccattagaaggggctcgtacatgttctgcagtgccccccgtaaatacgccgccggtatga**

**Fortunella_japonica_4** **ccattagaaggggctcgtacatgttctgcagtgccccccgtaaatacgccgccggtatga**

**Fortunella_japonica_5** **ccattagaaggggctcgtacatgttctgcagtgccccccgtaaatacgccgccggtatga**

**Fortunella_japonica_6** **ccattagaaggggctcgtacatgttctgcagtgccccccgtaaatacgccgccggtatga**

**Fortunella_japonica_7** **ccattagaaggggctcgtacatgttctgcagtgccccccgtaaatacgccgccggtatga**

**Fortunella_japonica_8** **ccattagaaggggctcgtacatgttctgcagtgccccccgtaaatacgccgccggtatga**

**Fortunella_japonica_9** **ccattagaaggggctcgtacatgttctgcagtgccccccgtaaatacgccgccggtatga**

**Fortunella_japonica_10** **ccattagaaggggctcgtacatgttctgcagtgccccccgtaaatacgccgccggtatga**

**Fortunella_japonica_11** **ccattagaaggggctcgtacatgttctgcagtgccccccgtaaatacgccgccggtatga**

**Fortunella_japonica_12** **ccattagaaggggctcgtacatgttctgcagtgccccccgtaaatacgccgccggtatga**

**Fortunella_margarita_1** **ccattagaaggggctcgtacatgttctgcagtgccccccgtaaatacgccgccggtatga**

**Fortunella_margarita_2** **ccattagaaggggctcgtacatgttctgcagtgccccccgtaaatacgccgccggtatga**

**Fortunella_venosa_1** **ccattagaaggggctcgtacatgttctgcagtgccccccgtaaatacgccgccggtatga**

**Fortunella_venosa_2** **ccattagaaggggctcgtacatgttctgcagtgccccccgtaaatacgccgccggtatga**

**Fortunella_venosa_3** **ccattagaaggggctcgtacatgttctgcagtgccccccgtaaatacgccgccggtatga**

21850	21860	21870	21880	21890	21900

....|....|....|....|....|....|....|....|....|....|....|....|

**Atalantia_kwangtungens** **aaagttcttaatgttagttgagtccctggttctccaatagattgacccgcaataatacct**

**Citrus_reticulata_1** **aaagttcttaatgttagttgagtccctggttctccaatagattgacccgcaataatacct**

**Citrus_reticulata_2** **aaagttcttaatgttagttgagtccctggttctccaatagattgacccgcaataatacct**

**Citrus_trifoliata_3** **aaagttcttaatgttagttgagtccctggttctccaatagattgacccgcaataatacct**

**Citrus_aurantiifolia** **aaagttcttaatgttagttgagtccctggttctccaatagattgacccgcaataatacct**

**Citrus_aurantium** **aaagttcttaatgttagttgagtccctggttctccaatagattgacccgcaataatacct**

**Citrus_cavaleriei** **aaagttcttaatgttagttgagtccctggttctccaatagattgacccgcaataatacct**

**Citrus_hongheensis** **aaagttcttaatgttagttgagtccctggttctccaatagattgacccgcaataatacct**

**Citrus_limon** **aaagttcttaatgttagttgagtccctggttctccaatagattgacccgcaataatacct**

**Citrus_maxima** **aaagttcttaatgttagttgagtccctggttctccaatagattgacccgcaataatacct**

**Citrus_medica** **aaagttcttaatgttagttgagtccctggttctccaatagattgacccgcaataatacct**

**Citrus_polytrifolia** **aaagttcttaatgttagttgagtccctggttctccaatagattgacccgcaataatacct**

**Citrus_reticulata** **aaagttcttaatgttagttgagtccctggttctccaatagattgacccgcaataatacct**

**Citrus_sinensis** **aaagttcttaatgttagttgagtccctggttctccaatagattgacccgcaataatacct**

**Fortunella_bawangica_1** **aaagttcttaatgttagttgagtccctggttctccaatagattgacccgcaataatacct**

**Fortunella_bawangica_2** **aaagttcttaatgttagttgagtccctggttctccaatagattgacccgcaataatacct**

**Fortunella_hindsii_1** **aaagttcttaatgttagttgagtccctggttctccaatagattgacccgcaataatacct**

**Fortunella_hindsii_2** **aaagttcttaatgttagttgagtccctggttctccaatagattgacccgcaataatacct**

**Fortunella_hindsii_3** **aaagttcttaatgttagttgagtccctggttctccaatagattgacccgcaataatacct**

**Fortunella_japonica_1** **aaagttcttaatgttagttgagtccctggttctccaatagattgacccgcaataatacct**

**Fortunella_japonica_2** **aaagttcttaatgttagttgagtccctggttctccaatagattgacccgcaataatacct**

**Fortunella_japonica_3** **aaagttcttaatgttagttgagtccctggttctccaatagattgacccgcaataatacct**

**Fortunella_japonica_4** **aaagttcttaatgttagttgagtccctggttctccaatagattgacccgcaataatacct**

**Fortunella_japonica_5** **aaagttcttaatgttagttgagtccctggttctccaatagattgacccgcaataatacct**

**Fortunella_japonica_6** **aaagttcttaatgttagttgagtccctggttctccaatagattgacccgcaataatacct**

**Fortunella_japonica_7** **aaagttcttaatgttagttgagtccctggttctccaatagattgacccgcaataatacct**

**Fortunella_japonica_8** **aaagttcttaatgttagttgagtccctggttctccaatagattgacccgcaataatacct**

**Fortunella_japonica_9** **aaagttcttaatgttagttgagtccctggttctccaatagattgacccgcaataatacct**

**Fortunella_japonica_10** **aaagttcttaatgttagttgagtccctggttctccaatagattgacccgcaataatacct**

**Fortunella_japonica_11** **aaagttcttaatgttagttgagtccctggttctccaatagattgacccgcaataatacct**

**Fortunella_japonica_12** **aaagttcttaatgttagttgagtccctggttctccaatagattgacccgcaataatacct**

**Fortunella_margarita_1** **aaagttcttaatgttagttgagtccctggttctccaatagattgacccgcaataatacct**

**Fortunella_margarita_2** **aaagttcttaatgttagttgagtccctggttctccaatagattgacccgcaataatacct**

**Fortunella_venosa_1** **aaagttcttaatgttagttgagtccctggttctccaatagattgacccgcaataatacct**

**Fortunella_venosa_2** **aaagttcttaatgttagttgagtccctggttctccaatagattgacccgcaataatacct**

**Fortunella_venosa_3** **aaagttcttaatgttagttgagtccctggttctccaatagattgacccgcaataatacct**

21910	21920	21930	21940	21950	21960

....|....|....|....|....|....|....|....|....|....|....|....|

**Atalantia_kwangtungens** **acggcttcccccaattcaaccaggtcgccatgagtcggactccgaccatagcataatcga**

**Citrus_reticulata_1** **acggcttcccccaattcaaccaggtcaccatgagtcggactccgaccataacataatcga**

**Citrus_reticulata_2** **acggcttcccccaattcaaccaggtcaccatgagtcggactccgaccataacataatcga**

**Citrus_trifoliata_3** **acggcttcccccaattcaaccaggtcaccatgagtcggactccgaccataacataatcga**

**Citrus_aurantiifolia** **acggcttcccccaattcaaccaggtcaccatgagtcggactccgaccataacataatcga**

**Citrus_aurantium** **acggcttcccccaattcaaccaggtcaccatgagtcggactccgaccataacataatcga**

**Citrus_cavaleriei** **acggcttcccccaattcaaccaggtcaccatgagtcggactccgaccataacataatcga**

**Citrus_hongheensis** **acggcttcccccaattcaaccaggtcaccatgagtcggactccgaccataacataatcga**

**Citrus_limon** **acggcttcccccaattcaaccaggtcaccatgagtcggactccgaccataacataatcga**

**Citrus_maxima** **acggcttcccccaattcaaccaggtcaccatgagtcggactccgaccataacataatcga**

**Citrus_medica** **acggcttcccccaattcaaccaggtcaccatgagtcggactccgaccataacataatcga**

**Citrus_polytrifolia** **acggcttcccccaattcaaccaggtcaccatgagtcggactccgaccataacataatcga**

**Citrus_reticulata** **acggcttcccccaattcaaccaggtcaccatgagtcggactccgaccataacataatcga**

**Citrus_sinensis** **acggcttcccccaattcaaccaggtcaccatgagtcggactccgaccataacataatcga**

**Fortunella_bawangica_1** **acggcttcccccaattcaaccaggtcaccatgagtcggactccgaccataacataatcga**

**Fortunella_bawangica_2** **acggcttcccccaattcaaccaggtcaccatgagtcggactccgaccataacataatcga**

**Fortunella_hindsii_1** **acggcttcccccaattcaaccaggtcaccatgagtcggactccgaccataacataatcga**

**Fortunella_hindsii_2** **acggcttcccccaattcaaccaggtcaccatgagtcggactccgaccataacataatcga**

**Fortunella_hindsii_3** **acggcttcccccaattcaaccaggtcaccatgagtcggactccgaccataacataatcga**

**Fortunella_japonica_1** **acggcttcccccaattcaaccaggtcaccatgagtcggactccgaccataacataatcga**

**Fortunella_japonica_2** **acggcttcccccaattcaaccaggtcaccatgagtcggactccgaccataacataatcga**

**Fortunella_japonica_3** **acggcttcccccaattcaaccaggtcaccatgagtcggactccgaccataacataatcga**

**Fortunella_japonica_4** **acggcttcccccaattcaaccaggtcaccatgagtcggactccgaccataacataatcga**

**Fortunella_japonica_5** **acggcttcccccaattcaaccaggtcaccatgagtcggactccgaccataacataatcga**

**Fortunella_japonica_6** **acggcttcccccaattcaaccaggtcaccatgagtcggactccgaccataacataatcga**

**Fortunella_japonica_7** **acggcttcccccaattcaaccaggtcaccatgagtcggactccgaccataacataatcga**

**Fortunella_japonica_8** **acggcttcccccaattcaaccaggtcaccatgagtcggactccgaccataacataatcga**

**Fortunella_japonica_9** **acggcttcccccaattcaaccaggtcaccatgagtcggactccgaccataacataatcga**

**Fortunella_japonica_10** **acggcttcccccaattcaaccaggtcaccatgagtcggactccgaccataacataatcga**

**Fortunella_japonica_11** **acggcttcccccaattcaaccaggtcaccatgagtcggactccgaccataacataatcga**

**Fortunella_japonica_12** **acggcttcccccaattcaaccaggtcaccatgagtcggactccgaccataacataatcga**

**Fortunella_margarita_1** **acggcttcccccaattcaaccaggtcaccatgagtcggactccgaccataacataatcga**

**Fortunella_margarita_2** **acggcttcccccaattcaaccaggtcaccatgagtcggactccgaccataacataatcga**

**Fortunella_venosa_1** **acggcttcccccaattcaaccaggtcaccatgagtcggactccgaccataacataatcga**

**Fortunella_venosa_2** **acggcttcccccaattcaaccaggtcaccatgagtcggactccgaccataacataatcga**

**Fortunella_venosa_3** **acggcttcccccaattcaaccaggtcaccatgagtcggactccgaccataacataatcga**

21970	21980	21990	22000	22010	22020

....|....|....|....|....|....|....|....|....|....|....|....|

**Atalantia_kwangtungens** **cagatccacgatgtactcctacaagtaaagggggttcgaatagatattgcttgtgttcga**

**Citrus_reticulata_1** **cagatccacgatgtactcctacaagtaaagggggttcgaatagatattgcttgtgttcga**

**Citrus_reticulata_2** **cagatccacgatgtactcctacaagtaaagggggttcgaatagatattgcttgtgttcga**

**Citrus_trifoliata_3** **cagatccacgatgtactcctacaagtaaagggggttcgaatagatattgcttgtgttcga**

**Citrus_aurantiifolia** **cagatccacgatgtactcctacaagtaaagggggttcgaatagatattgcttgtgttcga**

**Citrus_aurantium** **cagatccacgatgtactcctacaagtaaagggggttcgaatagatattgcttgtgttcga**

**Citrus_cavaleriei** **cagatccacgatgtactcctacaagtaaagggggttcgaatagatattgcttgtgttcga**

**Citrus_hongheensis** **cagatccacgatgtactcctacaagtaaagggggttcgaatagatattgcttgtgttcga**

**Citrus_limon** **cagatccacgatgtactcctacaagtaaagggggttcgaatagatattgcttgtgttcga**

**Citrus_maxima** **cagatccacgatgtactcctacaagtaaagggggttcgaatagatattgcttgtgttcga**

**Citrus_medica** **cagatccacgatgtactcctacaagtaaagggggttcgaatagatattgcttgtgttcga**

**Citrus_polytrifolia** **cagatccacgatgtactcctacaagtaaagggggttcgaatagatattgcttgtgttcga**

**Citrus_reticulata** **cagatccacgatgtactcctacaagtaaagggggttcgaatagatattgcttgtgttcga**

**Citrus_sinensis** **cagatccacgatgtactcctacaagtaaagggggttcgaatagatattgcttgtgttcga**

**Fortunella_bawangica_1** **cagatccacgatgtactcctacaagtaaagggggttcgaatagatattgcttgtgttcga**

**Fortunella_bawangica_2** **cagatccacgatgtactcctacaagtaaagggggttcgaatagatattgcttgtgttcga**

**Fortunella_hindsii_1** **cagatccacgatgtactcctacaagtaaagggggttcgaatagatattgcttgtgttcga**

**Fortunella_hindsii_2** **cagatccacgatgtactcctacaagtaaagggggttcgaatagatattgcttgtgttcga**

**Fortunella_hindsii_3** **cagatccacgatgtactcctacaagtaaagggggttcgaatagatattgcttgtgttcga**

**Fortunella_japonica_1** **cagatccacgatgtactcctacaagtaaagggggttcgaatagatattgcttgtgttcga**

**Fortunella_japonica_2** **cagatccacgatgtactcctacaagtaaagggggttcgaatagatattgcttgtgttcga**

**Fortunella_japonica_3** **cagatccacgatgtactcctacaagtaaagggggttcgaatagatattgcttgtgttcga**

**Fortunella_japonica_4** **cagatccacgatgtactcctacaagtaaagggggttcgaatagatattgcttgtgttcga**

**Fortunella_japonica_5** **cagatccacgatgtactcctacaagtaaagggggttcgaatagatattgcttgtgttcga**

**Fortunella_japonica_6** **cagatccacgatgtactcctacaagtaaagggggttcgaatagatattgcttgtgttcga**

**Fortunella_japonica_7** **cagatccacgatgtactcctacaagtaaagggggttcgaatagatattgcttgtgttcga**

**Fortunella_japonica_8** **cagatccacgatgtactcctacaagtaaagggggttcgaatagatattgcttgtgttcga**

**Fortunella_japonica_9** **cagatccacgatgtactcctacaagtaaagggggttcgaatagatattgcttgtgttcga**

**Fortunella_japonica_10** **cagatccacgatgtactcctacaagtaaagggggttcgaatagatattgcttgtgttcga**

**Fortunella_japonica_11** **cagatccacgatgtactcctacaagtaaagggggttcgaatagatattgcttgtgttcga**

**Fortunella_japonica_12** **cagatccacgatgtactcctacaagtaaagggggttcgaatagatattgcttgtgttcga**

**Fortunella_margarita_1** **cagatccacgatgtactcctacaagtaaagggggttcgaatagatattgcttgtgttcga**

**Fortunella_margarita_2** **cagatccacgatgtactcctacaagtaaagggggttcgaatagatattgcttgtgttcga**

**Fortunella_venosa_1** **cagatccacgatgtactcctacaagtaaagggggttcgaatagatattgcttgtgttcga**

**Fortunella_venosa_2** **cagatccacgatgtactcctacaagtaaagggggttcgaatagatattgcttgtgttcga**

**Fortunella_venosa_3** **cagatccacgatgtactcctacaagtaaagggggttcgaatagatattgcttgtgttcga**

22030	22040	22050	22060	22070	22080

....|....|....|....|....|....|....|....|....|....|....|....|

**Atalantia_kwangtungens** **agggttatgagtcgattgacaagtccaatcccaatatcttgatttctaatggcgatggat**

**Citrus_reticulata_1** **agggttatgagtcgattgacaagtccaatcccaatatcttgatttctaatggcaatggat**

**Citrus_reticulata_2** **agggttatgagtcgattgacaagtccaatcccaatatcttgatttctaatggcaatggat**

**Citrus_trifoliata_3** **agggttatgagtcgattgacaagtccaatcccaatatcttgatttctaatggcaatggat**

**Citrus_aurantiifolia** **agggttatgagtcgattgacaagtccaatcccaatatcttgatttctaatggcaatggat**

**Citrus_aurantium** **agggttatgagtcgattgacaagtccaatcccaatatcttgatttctaatggcaatggat**

**Citrus_cavaleriei** **agggttatgagtcgattgacaagtccaatcccaatatcttgatttctaatggcaatggat**

**Citrus_hongheensis** **agggttatgagtcgattgacaagtccaatcccaatatcttgatttctaatggcaatggat**

**Citrus_limon** **agggttatgagtcgattgacaagtccaatcccaatatcttgatttctaatggcaatggat**

**Citrus_maxima** **agggttatgagtcgattgacaagtccaatcccaatatcttgatttctaatggcaatggat**

**Citrus_medica** **agggttatgagtcgattgacaagtccaatcccaatatcttgatttctaatggcaatggat**

**Citrus_polytrifolia** **agggttatgagtcgattgacaagtccaatcccaatatcttgatttctaatggcaatggat**

**Citrus_reticulata** **agggttatgagtcgattgacaagtccaatcccaatatcttgatttctaatggcaatggat**

**Citrus_sinensis** **agggttatgagtcgattgacaagtccaatcccaatatcttgatttctaatggcaatggat**

**Fortunella_bawangica_1** **agggttatgagtcgattgacaagtccaatcccaatatcttgatttctaatggcaatggat**

**Fortunella_bawangica_2** **agggttatgagtcgattgacaagtccaatcccaatatcttgatttctaatggcaatggat**

**Fortunella_hindsii_1** **agggttatgagtcgattgacaagtccaatcccaatatcttgatttctaatggcaatggat**

**Fortunella_hindsii_2** **agggttatgagtcgattgacaagtccaatcccaatatcttgatttctaatggcaatggat**

**Fortunella_hindsii_3** **agggttatgagtcgattgacaagtccaatcccaatatcttgatttctaatggcaatggat**

**Fortunella_japonica_1** **agggttatgagtcgattgacaagtccaatcccaatatcttgatttctaatggcaatggat**

**Fortunella_japonica_2** **agggttatgagtcgattgacaagtccaatcccaatatcttgatttctaatggcaatggat**

**Fortunella_japonica_3** **agggttatgagtcgattgacaagtccaatcccaatatcttgatttctaatggcaatggat**

**Fortunella_japonica_4** **agggttatgagtcgattgacaagtccaatcccaatatcttgatttctaatggcaatggat**

**Fortunella_japonica_5** **agggttatgagtcgattgacaagtccaatcccaatatcttgatttctaatggcaatggat**

**Fortunella_japonica_6** **agggttatgagtcgattgacaagtccaatcccaatatcttgatttctaatggcaatggat**

**Fortunella_japonica_7** **agggttatgagtcgattgacaagtccaatcccaatatcttgatttctaatggcaatggat**

**Fortunella_japonica_8** **agggttatgagtcgattgacaagtccaatcccaatatcttgatttctaatggcaatggat**

**Fortunella_japonica_9** **agggttatgagtcgattgacaagtccaatcccaatatcttgatttctaatggcaatggat**

**Fortunella_japonica_10** **agggttatgagtcgattgacaagtccaatcccaatatcttgatttctaatggcaatggat**

**Fortunella_japonica_11** **agggttatgagtcgattgacaagtccaatcccaatatcttgatttctaatggcaatggat**

**Fortunella_japonica_12** **agggttatgagtcgattgacaagtccaatcccaatatcttgatttctaatggcaatggat**

**Fortunella_margarita_1** **agggttatgagtcgattgacaagtccaatcccaatatcttgatttctaatggcaatggat**

**Fortunella_margarita_2** **agggttatgagtcgattgacaagtccaatcccaatatcttgatttctaatggcaatggat**

**Fortunella_venosa_1** **agggttatgagtcgattgacaagtccaatcccaatatcttgatttctaatggcaatggat**

**Fortunella_venosa_2** **agggttatgagtcgattgacaagtccaatcccaatatcttgatttctaatggcaatggat**

**Fortunella_venosa_3** **agggttatgagtcgattgacaagtccaatcccaatatcttgatttctaatggcaatggat**

22090	22100	22110	22120	22130	22140

....|....|....|....|....|....|....|....|....|....|....|....|

**Atalantia_kwangtungens** **cgctggcccatatatatatcgtctgctaatacacgaccaattaatgtttggctaaaaacc**

**Citrus_reticulata_1** **cgctggcccatatatatatcgtccgctaatacacgaccaattaatgtttggctaaaaacc**

**Citrus_reticulata_2** **cgctggcccatatatatatcgtccgctaatacacgaccaattaatgtttggctaaaaacc**

**Citrus_trifoliata_3** **cgctggcccatatatatatcgtccgctaatacacgaccaattaatgtttggctaaaaacc**

**Citrus_aurantiifolia** **cgctggcccatatatatatcgtccgctaatacacgaccaattaatgtttggctaaaaacc**

**Citrus_aurantium** **cgctggcccatatatatatcgtccgctaatacacgaccaattaatgtttggctaaaaacc**

**Citrus_cavaleriei** **cgctggcccatatatatatcgtccgctaatacacgaccaattaatgtttggctaaaaacc**

**Citrus_hongheensis** **cgctggcccatatatatatcgtccgctaatacacgaccaattaatgtttggctaaaaacc**

**Citrus_limon** **cgctggcccatatatatatcgtccgctaatacacgaccaattaatgtttggctaaaaacc**

**Citrus_maxima** **cgctggcccatatatatatcgtccgctaatacacgaccaattaatgtttggctaaaaacc**

**Citrus_medica** **cgctggcccatatatatatcgtccgctaatacacgaccaattaatgtttggctaaaaacc**

**Citrus_polytrifolia** **cgctggcccatatatatatcgtccgctaatacacgaccaattaatgtttggctaaaaacc**

**Citrus_reticulata** **cgctggcccatatatatatcgtccgctaatacacgaccaattaatgtttggctaaaaacc**

**Citrus_sinensis** **cgctggcccatatatatatcgtccgctaatacacgaccaattaatgtttggctaaaaacc**

**Fortunella_bawangica_1** **cgctggcccatatatatatcgtccgctaatacacgaccaattaatgtttggctaaaaacc**

**Fortunella_bawangica_2** **cgctggcccatatatatatcgtccgctaatacacgaccaattaatgtttggctaaaaacc**

**Fortunella_hindsii_1** **cgctggcccatatatatatcgtccgctaatacacgaccaattaatgtttggctaaaaacc**

**Fortunella_hindsii_2** **cgctggcccatatatatatcgtccgctaatacacgaccaattaatgtttggctaaaaacc**

**Fortunella_hindsii_3** **cgctggcccatatatatatcgtccgctaatacacgaccaattaatgtttggctaaaaacc**

**Fortunella_japonica_1** **cgctggcccatatatatatcgtccgctaatacacgaccaattaatgtttggctaaaaacc**

**Fortunella_japonica_2** **cgctggcccatatatatatcgtccgctaatacacgaccaattaatgtttggctaaaaacc**

**Fortunella_japonica_3** **cgctggcccatatatatatcgtccgctaatacacgaccaattaatgtttggctaaaaacc**

**Fortunella_japonica_4** **cgctggcccatatatatatcgtccgctaatacacgaccaattaatgtttggctaaaaacc**

**Fortunella_japonica_5** **cgctggcccatatatatatcgtccgctaatacacgaccaattaatgtttggctaaaaacc**

**Fortunella_japonica_6** **cgctggcccatatatatatcgtccgctaatacacgaccaattaatgtttggctaaaaacc**

**Fortunella_japonica_7** **cgctggcccatatatatatcgtccgctaatacacgaccaattaatgtttggctaaaaacc**

**Fortunella_japonica_8** **cgctggcccatatatatatcgtccgctaatacacgaccaattaatgtttggctaaaaacc**

**Fortunella_japonica_9** **cgctggcccatatatatatcgtccgctaatacacgaccaattaatgtttggctaaaaacc**

**Fortunella_japonica_10** **cgctggcccatatatatatcgtccgctaatacacgaccaattaatgtttggctaaaaacc**

**Fortunella_japonica_11** **cgctggcccatatatatatcgtccgctaatacacgaccaattaatgtttggctaaaaacc**

**Fortunella_japonica_12** **cgctggcccatatatatatcgtccgctaatacacgaccaattaatgtttggctaaaaacc**

**Fortunella_margarita_1** **cgctggcccatatatatatcgtccgctaatacacgaccaattaatgtttggctaaaaacc**

**Fortunella_margarita_2** **cgctggcccatatatatatcgtccgctaatacacgaccaattaatgtttggctaaaaacc**

**Fortunella_venosa_1** **cgctggcccatatatatatcgtccgctaatacacgaccaattaatgtttggctaaaaacc**

**Fortunella_venosa_2** **cgctggcccatatatatatcgtccgctaatacacgaccaattaatgtttggctaaaaacc**

**Fortunella_venosa_3** **cgctggcccatatatatatcgtccgctaatacacgaccaattaatgtttggctaaaaacc**

22150	22160	22170	22180	22190	22200

....|....|....|....|....|....|....|....|....|....|....|....|

**Atalantia_kwangtungens** **ctttccgacatcatcctattttgattttgaggactcacagaaattcctcggacagtgcca**

**Citrus_reticulata_1** **ctttccgacatcatcctattttgattttgaggactcacagaaattcctcggacagtgcca**

**Citrus_reticulata_2** **ctttccgacatcatcctattttgattttgaggactcacagaaattcctcggacagtgcca**

**Citrus_trifoliata_3** **ctttccgacatcatcctattttgattttgaggactcacagaaattcctcggacagtgcca**

**Citrus_aurantiifolia** **ctttccgacatcatcctattttgattttgaggactcacagaaattcctcggacagtgcca**

**Citrus_aurantium** **ctttccgacatcatcctattttgattttgaggactcacagaaattcctcggacagtgcca**

**Citrus_cavaleriei** **ctttccgacatcatcctattttgattttgaggactcacagaaattcctcggacagtgcca**

**Citrus_hongheensis** **ctttccgacatcatcctattttgattttgaggactcacagaaattcctcggacagtgcca**

**Citrus_limon** **ctttccgacatcatcctattttgattttgaggactcacagaaattcctcggacagtgcca**

**Citrus_maxima** **ctttccgacatcatcctattttgattttgaggactcacagaaattcctcggacagtgcca**

**Citrus_medica** **ctttccgacatcatcctattttgattttgaggactcacagaaattcctcggacagtgcca**

**Citrus_polytrifolia** **ctttccgacatcatcctattttgattttgaggactcacagaaattcctcggacagtgcca**

**Citrus_reticulata** **ctttccgacatcatcctattttgattttgaggactcacagaaattcctcggacagtgcca**

**Citrus_sinensis** **ctttccgacatcatcctattttgattttgaggactcacagaaattcctcggacagtgcca**

**Fortunella_bawangica_1** **ctttccgacatcatcctattttgattttgaggactcacagaaattcctcggacagtgcca**

**Fortunella_bawangica_2** **ctttccgacatcatcctattttgattttgaggactcacagaaattcctcggacagtgcca**

**Fortunella_hindsii_1** **ctttccgacatcatcctattttgattttgaggactcacagaaattcctcggacagtgcca**

**Fortunella_hindsii_2** **ctttccgacatcatcctattttgattttgaggactcacagaaattcctcggacagtgcca**

**Fortunella_hindsii_3** **ctttccgacatcatcctattttgattttgaggactcacagaaattcctcggacagtgcca**

**Fortunella_japonica_1** **ctttccgacatcatcctattttgattttgaggactcacagaaattcctcggacagtgcca**

**Fortunella_japonica_2** **ctttccgacatcatcctattttgattttgaggactcacagaaattcctcggacagtgcca**

**Fortunella_japonica_3** **ctttccgacatcatcctattttgattttgaggactcacagaaattcctcggacagtgcca**

**Fortunella_japonica_4** **ctttccgacatcatcctattttgattttgaggactcacagaaattcctcggacagtgcca**

**Fortunella_japonica_5** **ctttccgacatcatcctattttgattttgaggactcacagaaattcctcggacagtgcca**

**Fortunella_japonica_6** **ctttccgacatcatcctattttgattttgaggactcacagaaattcctcggacagtgcca**

**Fortunella_japonica_7** **ctttccgacatcatcctattttgattttgaggactcacagaaattcctcggacagtgcca**

**Fortunella_japonica_8** **ctttccgacatcatcctattttgattttgaggactcacagaaattcctcggacagtgcca**

**Fortunella_japonica_9** **ctttccgacatcatcctattttgattttgaggactcacagaaattcctcggacagtgcca**

**Fortunella_japonica_10** **ctttccgacatcatcctattttgattttgaggactcacagaaattcctcggacagtgcca**

**Fortunella_japonica_11** **ctttccgacatcatcctattttgattttgaggactcacagaaattcctcggacagtgcca**

**Fortunella_japonica_12** **ctttccgacatcatcctattttgattttgaggactcacagaaattcctcggacagtgcca**

**Fortunella_margarita_1** **ctttccgacatcatcctattttgattttgaggactcacagaaattcctcggacagtgcca**

**Fortunella_margarita_2** **ctttccgacatcatcctattttgattttgaggactcacagaaattcctcggacagtgcca**

**Fortunella_venosa_1** **ctttccgacatcatcctattttgattttgaggactcacagaaattcctcggacagtgcca**

**Fortunella_venosa_2** **ctttccgacatcatcctattttgattttgaggactcacagaaattcctcggacagtgcca**

**Fortunella_venosa_3** **ctttccgacatcatcctattttgattttgaggactcacagaaattcctcggacagtgcca**

22210	22220	22230	22240	22250	22260

....|....|....|....|....|....|....|....|....|....|....|....|

**Atalantia_kwangtungens** **caatctgttcgacgtacaataatgtgttgaactacttcaacaagtctgcgcgtaagatat**

**Citrus_reticulata_1** **caatctgttctacgtacaataatgtgttgaactacttcaacaagtctgcgcgtaagatat**

**Citrus_reticulata_2** **caatctgttctacgtacaataatgtgttgaactacttcaacaagtctgcgcgtaagatat**

**Citrus_trifoliata_3** **caatctgttctacgtacaataatgtgttgaactacttcaacaagtctgcgcgtaagatat**

**Citrus_aurantiifolia** **caatctgttctacgtacaataatgtgttgaactacttcaacaagtctgcgcgtaagatat**

**Citrus_aurantium** **caatctgttctacgtacaataatgtgttgaactacttcaacaagtctgcgcgtaagatat**

**Citrus_cavaleriei** **caatctgttctacgtacaataatgtgttgaactacttcaacaagtctgcgcgtaagatat**

**Citrus_hongheensis** **caatctgttctacgtacaataatgtgttgaactacttcaacaagtctgcgcgtaagatat**

**Citrus_limon** **caatctgttctacgtacaataatgtgttgaactacttcaacaagtctgcgcgtaagatat**

**Citrus_maxima** **caatctgttctacgtacaataatgtgttgaactacttcaacaagtctgcgcgtaagatat**

**Citrus_medica** **caatctgttctacgtacaataatgtgttgaactacttcaacaagtctgcgcgtaagatat**

**Citrus_polytrifolia** **caatctgttctacgtacaataatgtgttgaactacttcaacaagtctgcgcgtaagatat**

**Citrus_reticulata** **caatctgttctacgtacaataatgtgttgaactacttcaacaagtctgcgcgtaagatat**

**Citrus_sinensis** **caatctgttctacgtacaataatgtgttgaactacttcaacaagtctgcgcgtaagatat**

**Fortunella_bawangica_1** **caatctgttctacgtacaataatgtgttgaactacttcaacaagtctgcgcgtaagatat**

**Fortunella_bawangica_2** **caatctgttctacgtacaataatgtgttgaactacttcaacaagtctgcgcgtaagatat**

**Fortunella_hindsii_1** **caatctgttctacgtacaataatgtgttgaactacttcaacaagtctgcgcgtaagatat**

**Fortunella_hindsii_2** **caatctgttctacgtacaataatgtgttgaactacttcaacaagtctgcgcgtaagatat**

**Fortunella_hindsii_3** **caatctgttctacgtacaataatgtgttgaactacttcaacaagtctgcgcgtaagatat**

**Fortunella_japonica_1** **caatctgttctacgtacaataatgtgttgaactacttcaacaagtctgcgcgtaagatat**

**Fortunella_japonica_2** **caatctgttctacgtacaataatgtgttgaactacttcaacaagtctgcgcgtaagatat**

**Fortunella_japonica_3** **caatctgttctacgtacaataatgtgttgaactacttcaacaagtctgcgcgtaagatat**

**Fortunella_japonica_4** **caatctgttctacgtacaataatgtgttgaactacttcaacaagtctgcgcgtaagatat**

**Fortunella_japonica_5** **caatctgttctacgtacaataatgtgttgaactacttcaacaagtctgcgcgtaagatat**

**Fortunella_japonica_6** **caatctgttctacgtacaataatgtgttgaactacttcaacaagtctgcgcgtaagatat**

**Fortunella_japonica_7** **caatctgttctacgtacaataatgtgttgaactacttcaacaagtctgcgcgtaagatat**

**Fortunella_japonica_8** **caatctgttctacgtacaataatgtgttgaactacttcaacaagtctgcgcgtaagatat**

**Fortunella_japonica_9** **caatctgttctacgtacaataatgtgttgaactacttcaacaagtctgcgcgtaagatat**

**Fortunella_japonica_10** **caatctgttctacgtacaataatgtgttgaactacttcaacaagtctgcgcgtaagatat**

**Fortunella_japonica_11** **caatctgttctacgtacaataatgtgttgaactacttcaacaagtctgcgcgtaagatat**

**Fortunella_japonica_12** **caatctgttctacgtacaataatgtgttgaactacttcaacaagtctgcgcgtaagatat**

**Fortunella_margarita_1** **caatctgttctacgtacaataatgtgttgaactacttcaacaagtctgcgcgtaagatat**

**Fortunella_margarita_2** **caatctgttctacgtacaataatgtgttgaactacttcaacaagtctgcgcgtaagatat**

**Fortunella_venosa_1** **caatctgttctacgtacaataatgtgttgaactacttcaacaagtctgcgcgtaagatat**

**Fortunella_venosa_2** **caatctgttctacgtacaataatgtgttgaactacttcaacaagtctgcgcgtaagatat**

**Fortunella_venosa_3** **caatctgttctacgtacaataatgtgttgaactacttcaacaagtctgcgcgtaagatat**

22270	22280	22290	22300	22310	22320

....|....|....|....|....|....|....|....|....|....|....|....|

**Atalantia_kwangtungens** **ccagcatctgatgttcggacagcggtatcgacaacccccttgcgggctccatagcaagaa**

**Citrus_reticulata_1** **ccagcatctgatgttcggacagcggtatcgacaacccccttgcgggctccatagcaagaa**

**Citrus_reticulata_2** **ccagcatctgatgttcggacagcggtatcgacaacccccttgcgggctccatagcaagaa**

**Citrus_trifoliata_3** **ccagcatctgatgttcggacagcggtatcgacaacccccttgcgggctccatagcaagaa**

**Citrus_aurantiifolia** **ccagcatctgatgttcggacagcggtatcgacaacccccttgcgggctccatagcaagaa**

**Citrus_aurantium** **ccagcatctgatgttcggacagcggtatcgacaacccccttgcgggctccatagcaagaa**

**Citrus_cavaleriei** **ccagcatctgatgttcggacagcggtatcgacaacccccttgcgggctccatagcaagaa**

**Citrus_hongheensis** **ccagcatctgatgttcggacagcggtatcgacaacccccttgcgggctccatagcaagaa**

**Citrus_limon** **ccagcatctgatgttcggacagcggtatcgacaacccccttgcgggctccatagcaagaa**

**Citrus_maxima** **ccagcatctgatgttcggacagcggtatcgacaacccccttgcgggctccatagcaagaa**

**Citrus_medica** **ccagcatctgatgttcggacagcggtatcgacaacccccttgcgggctccatagcaagaa**

**Citrus_polytrifolia** **ccagcatctgatgttcggacagcggtatcgacaacccccttgcgggctccatagcaagaa**

**Citrus_reticulata** **ccagcatctgatgttcggacagcggtatcgacaacccccttgcgggctccatagcaagaa**

**Citrus_sinensis** **ccagcatctgatgttcggacagcggtatcgacaacccccttgcgggctccatagcaagaa**

**Fortunella_bawangica_1** **ccagcatctgatgttcggacagcggtatcgacaacccccttgcgggctccatagcaagaa**

**Fortunella_bawangica_2** **ccagcatctgatgttcggacagcggtatcgacaacccccttgcgggctccatagcaagaa**

**Fortunella_hindsii_1** **ccagcatctgatgttcggacagcggtatcgacaacccccttgcgggctccatagcaagaa**

**Fortunella_hindsii_2** **ccagcatctgatgttcggacagcggtatcgacaacccccttgcgggctccatagcaagaa**

**Fortunella_hindsii_3** **ccagcatctgatgttcggacagcggtatcgacaacccccttgcgggctccatagcaagaa**

**Fortunella_japonica_1** **ccagcatctgatgttcggacagcggtatcgacaacccccttgcgggctccatagcaagaa**

**Fortunella_japonica_2** **ccagcatctgatgttcggacagcggtatcgacaacccccttgcgggctccatagcaagaa**

**Fortunella_japonica_3** **ccagcatctgatgttcggacagcggtatcgacaacccccttgcgggctccatagcaagaa**

**Fortunella_japonica_4** **ccagcatctgatgttcggacagcggtatcgacaacccccttgcgggctccatagcaagaa**

**Fortunella_japonica_5** **ccagcatctgatgttcggacagcggtatcgacaacccccttgcgggctccatagcaagaa**

**Fortunella_japonica_6** **ccagcatctgatgttcggacagcggtatcgacaacccccttgcgggctccatagcaagaa**

**Fortunella_japonica_7** **ccagcatctgatgttcggacagcggtatcgacaacccccttgcgggctccatagcaagaa**

**Fortunella_japonica_8** **ccagcatctgatgttcggacagcggtatcgacaacccccttgcgggctccatagcaagaa**

**Fortunella_japonica_9** **ccagcatctgatgttcggacagcggtatcgacaacccccttgcgggctccatagcaagaa**

**Fortunella_japonica_10** **ccagcatctgatgttcggacagcggtatcgacaacccccttgcgggctccatagcaagaa**

**Fortunella_japonica_11** **ccagcatctgatgttcggacagcggtatcgacaacccccttgcgggctccatagcaagaa**

**Fortunella_japonica_12** **ccagcatctgatgttcggacagcggtatcgacaacccccttgcgggctccatagcaagaa**

**Fortunella_margarita_1** **ccagcatctgatgttcggacagcggtatcgacaacccccttgcgggctccatagcaagaa**

**Fortunella_margarita_2** **ccagcatctgatgttcggacagcggtatcgacaacccccttgcgggctccatagcaagaa**

**Fortunella_venosa_1** **ccagcatctgatgttcggacagcggtatcgacaacccccttgcgggctccatagcaagaa**

**Fortunella_venosa_2** **ccagcatctgatgttcggacagcggtatcgacaacccccttgcgggctccatagcaagaa**

**Fortunella_venosa_3** **ccagcatctgatgttcggacagcggtatcgacaacccccttgcgggctccatagcaagaa**

22330	22340	22350	22360	22370	22380

....|....|....|....|....|....|....|....|....|....|....|....|

**Atalantia_kwangtungens** **atgatatattctgttaaagaaagtccttcgcgtaaattgctttgaatgggtaaatcaatc**

**Citrus_reticulata_1** **atgatatattctgttaaagaaagcccttcgcgtaaattactttgaatgggtaaatcaatc**

**Citrus_reticulata_2** **atgatatattctgttaaagaaagcccttcgcgtaaattactttgaatgggtaaatcaatc**

**Citrus_trifoliata_3** **atgatatattctgttaaagaaagcccttcgcgtaaattactttgaatgggtaaatcaatc**

**Citrus_aurantiifolia** **atgatatattctgttaaagaaagcccttcgcgtaaattactttgaatgggtaaatcaatc**

**Citrus_aurantium** **atgatatattctgttaaagaaagcccttcgcgtaaattactttgaatgggtaaatcaatc**

**Citrus_cavaleriei** **atgatatattctgttaaagaaagcccttcgcgtaaattactttgaatgggtaaatcaatc**

**Citrus_hongheensis** **atgatatattctgttaaagaaagcccttcgcgtaaattactttgaatgggtaaatcaatc**

**Citrus_limon** **atgatatattctgttaaagaaagcccttcgcgtaaattactttgaatgggtaaatcaatc**

**Citrus_maxima** **atgatatattctgttaaagaaagcccttcgcgtaaattactttgaatgggtaaatcaatc**

**Citrus_medica** **atgatatattctgttaaagaaagcccttcgcgtaaattactttgaatgggtaaatcaatc**

**Citrus_polytrifolia** **atgatatattctgttaaagaaagcccttcgcgtaaattactttgaatgggtaaatcaatc**

**Citrus_reticulata** **atgatatattctgttaaagaaagcccttcgcgtaaattactttgaatgggtaaatcaatc**

**Citrus_sinensis** **atgatatattctgttaaagaaagcccttcgcgtaaattactttgaatgggtaaatcaatc**

**Fortunella_bawangica_1** **atgatatattctgttaaagaaagcccttcgcgtaaattactttgaatgggtaaatcaatc**

**Fortunella_bawangica_2** **atgatatattctgttaaagaaagcccttcgcgtaaattactttgaatgggtaaatcaatc**

**Fortunella_hindsii_1** **atgatatattctgttaaagaaagcccttcgcgtaaattactttgaatgggtaaatcaatc**

**Fortunella_hindsii_2** **atgatatattctgttaaagaaagcccttcgcgtaaattactttgaatgggtaaatcaatc**

**Fortunella_hindsii_3** **atgatatattctgttaaagaaagcccttcgcgtaaattactttgaatgggtaaatcaatc**

**Fortunella_japonica_1** **atgatatattctgttaaagaaagcccttcgcgtaaattactttgaatgggtaaatcaatc**

**Fortunella_japonica_2** **atgatatattctgttaaagaaagcccttcgcgtaaattactttgaatgggtaaatcaatc**

**Fortunella_japonica_3** **atgatatattctgttaaagaaagcccttcgcgtaaattactttgaatgggtaaatcaatc**

**Fortunella_japonica_4** **atgatatattctgttaaagaaagcccttcgcgtaaattactttgaatgggtaaatcaatc**

**Fortunella_japonica_5** **atgatatattctgttaaagaaagcccttcgcgtaaattactttgaatgggtaaatcaatc**

**Fortunella_japonica_6** **atgatatattctgttaaagaaagcccttcgcgtaaattactttgaatgggtaaatcaatc**

**Fortunella_japonica_7** **atgatatattctgttaaagaaagcccttcgcgtaaattactttgaatgggtaaatcaatc**

**Fortunella_japonica_8** **atgatatattctgttaaagaaagcccttcgcgtaaattactttgaatgggtaaatcaatc**

**Fortunella_japonica_9** **atgatatattctgttaaagaaagcccttcgcgtaaattactttgaatgggtaaatcaatc**

**Fortunella_japonica_10** **atgatatattctgttaaagaaagcccttcgcgtaaattactttgaatgggtaaatcaatc**

**Fortunella_japonica_11** **atgatatattctgttaaagaaagcccttcgcgtaaattactttgaatgggtaaatcaatc**

**Fortunella_japonica_12** **atgatatattctgttaaagaaagcccttcgcgtaaattactttgaatgggtaaatcaatc**

**Fortunella_margarita_1** **atgatatattctgttaaagaaagcccttcgcgtaaattactttgaatgggtaaatcaatc**

**Fortunella_margarita_2** **atgatatattctgttaaagaaagcccttcgcgtaaattactttgaatgggtaaatcaatc**

**Fortunella_venosa_1** **atgatatattctgttaaagaaagcccttcgcgtaaattactttgaatgggtaaatcaatc**

**Fortunella_venosa_2** **atgatatattctgttaaagaaagcccttcgcgtaaattactttgaatgggtaaatcaatc**

**Fortunella_venosa_3** **atgatatattctgttaaagaaagcccttcgcgtaaattactttgaatgggtaaatcaatc**

22390	22400	22410	22420	22430	22440

....|....|....|....|....|....|....|....|....|....|....|....|

**Atalantia_kwangtungens** **atttgcccttggggatcagacattaatcctctcatacccaccaattggtgtacttgagat**

**Citrus_reticulata_1** **atttgcccttggggatcagacattaatcctctcatacccaccaattggtgtacttgagat**

**Citrus_reticulata_2** **atttgcccttggggatcagacattaatcctctcatacccaccaattggtgtacttgagat**

**Citrus_trifoliata_3** **atttgcccttggggatcagacattaatcctctcatacccaccaattggtgtacttgagat**

**Citrus_aurantiifolia** **atttgcccttggggatcagacattaatcctctcatacccaccaattggtgtacttgagat**

**Citrus_aurantium** **atttgcccttggggatcagacattaatcctctcatacccaccaattggtgtacttgagat**

**Citrus_cavaleriei** **atttgcccttggggatcagacattaatcctctcatacccaccaattggtgtacttgagat**

**Citrus_hongheensis** **atttgcccttggggatcagacattaatcctctcatacccaccaattggtgtacttgagat**

**Citrus_limon** **atttgcccttggggatcagacattaatcctctcatacccaccaattggtgtacttgagat**

**Citrus_maxima** **atttgcccttggggatcagacattaatcctctcatacccaccaattggtgtacttgagat**

**Citrus_medica** **atttgcccttggggatcagacattaatcctctcatacccaccaattggtgtacttgagat**

**Citrus_polytrifolia** **atttgcccttggggatcagacattaatcctctcatacccaccaattggtgtacttgagat**

**Citrus_reticulata** **atttgcccttggggatcagacattaatcctctcatacccaccaattggtgtacttgagat**

**Citrus_sinensis** **atttgcccttggggatcagacattaatcctctcatacccaccaattggtgtacttgagat**

**Fortunella_bawangica_1** **atttgcccttggggatcagacattaatcctctcatacccaccaattggtgtacttgagat**

**Fortunella_bawangica_2** **atttgcccttggggatcagacattaatcctctcatacccaccaattggtgtacttgagat**

**Fortunella_hindsii_1** **atttgcccttggggatcagacattaatcctctcatacccaccaattggtgtacttgagat**

**Fortunella_hindsii_2** **atttgcccttggggatcagacattaatcctctcatacccaccaattggtgtacttgagat**

**Fortunella_hindsii_3** **atttgcccttggggatcagacattaatcctctcatacccaccaattggtgtacttgagat**

**Fortunella_japonica_1** **atttgcccttggggatcagacattaatcctctcatacccaccaattggtgtacttgagat**

**Fortunella_japonica_2** **atttgcccttggggatcagacattaatcctctcatacccaccaattggtgtacttgagat**

**Fortunella_japonica_3** **atttgcccttggggatcagacattaatcctctcatacccaccaattggtgtacttgagat**

**Fortunella_japonica_4** **atttgcccttggggatcagacattaatcctctcatacccaccaattggtgtacttgagat**

**Fortunella_japonica_5** **atttgcccttggggatcagacattaatcctctcatacccaccaattggtgtacttgagat**

**Fortunella_japonica_6** **atttgcccttggggatcagacattaatcctctcatacccaccaattggtgtacttgagat**

**Fortunella_japonica_7** **atttgcccttggggatcagacattaatcctctcatacccaccaattggtgtacttgagat**

**Fortunella_japonica_8** **atttgcccttggggatcagacattaatcctctcatacccaccaattggtgtacttgagat**

**Fortunella_japonica_9** **atttgcccttggggatcagacattaatcctctcatacccaccaattggtgtacttgagat**

**Fortunella_japonica_10** **atttgcccttggggatcagacattaatcctctcatacccaccaattggtgtacttgagat**

**Fortunella_japonica_11** **atttgcccttggggatcagacattaatcctctcatacccaccaattggtgtacttgagat**

**Fortunella_japonica_12** **atttgcccttggggatcagacattaatcctctcatacccaccaattggtgtacttgagat**

**Fortunella_margarita_1** **atttgcccttggggatcagacattaatcctctcatacccaccaattggtgtacttgagat**

**Fortunella_margarita_2** **atttgcccttggggatcagacattaatcctctcatacccaccaattggtgtacttgagat**

**Fortunella_venosa_1** **atttgcccttggggatcagacattaatcctctcatacccaccaattggtgtacttgagat**

**Fortunella_venosa_2** **atttgcccttggggatcagacattaatcctctcatacccaccaattggtgtacttgagat**

**Fortunella_venosa_3** **atttgcccttggggatcagacattaatcctctcatacccaccaattggtgtacttgagat**

22450	22460	22470	22480	22490	22500

....|....|....|....|....|....|....|....|....|....|....|....|

**Atalantia_kwangtungens** **gcatttcctctagcccccgaaaaagacattatatggactggattaaaaggctcagtcatc**

**Citrus_reticulata_1** **gcatttcctctagcccccgaaaaagacattatatggactggattaaaaggctcagtcatc**

**Citrus_reticulata_2** **gcatttcctctagcccccgaaaaagacattatatggactggattaaaaggctcagtcatc**

**Citrus_trifoliata_3** **gcatttcctctagcccccgaaaaagacattatatggactggattaaaaggctcagtcatc**

**Citrus_aurantiifolia** **gcatttcctctagcccccgaaaaagacattatatggactggattaaaaggctcagtcatc**

**Citrus_aurantium** **gcatttcctctagcccccgaaaaagacattatatggactggattaaaaggctcagtcatc**

**Citrus_cavaleriei** **gcatttcctctagcccccgaaaaagacattatatggactggattaaaaggctcagtcatc**

**Citrus_hongheensis** **gcatttcctctagcccccgaaaaagacattatatggactggattaaaaggctcagtcatc**

**Citrus_limon** **gcatttcctctagcccccgaaaaagacattatatggactggattaaaaggctcagtcatc**

**Citrus_maxima** **gcatttcctctagcccccgaaaaagacattatatggactggattaaaaggctcagtcatc**

**Citrus_medica** **gcatttcctctagcccccgaaaaagacattatatggactggattaaaaggctcagtcatc**

**Citrus_polytrifolia** **gcatttcctctagcccccgaaaaagacattatatggactggattaaaaggctcagtcatc**

**Citrus_reticulata** **gcatttcctctagcccccgaaaaagacattatatggactggattaaaaggctcagtcatc**

**Citrus_sinensis** **gcatttcctctagcccccgaaaaagacattatatggactggattaaaaggctcagtcatc**

**Fortunella_bawangica_1** **gcatttcctctagcccccgaaaaagacattatatggactggattaaaaggctcagtcatc**

**Fortunella_bawangica_2** **gcatttcctctagcccccgaaaaagacattatatggactggattaaaaggctcagtcatc**

**Fortunella_hindsii_1** **gcatttcctctagcccccgaaaaagacattatatggactggattaaaaggctcagtcatc**

**Fortunella_hindsii_2** **gcatttcctctagcccccgaaaaagacattatatggactggattaaaaggctcagtcatc**

**Fortunella_hindsii_3** **gcatttcctctagcccccgaaaaagacattatatggactggattaaaaggctcagtcatc**

**Fortunella_japonica_1** **gcatttcctctagcccccgaaaaagacattatatggactggattaaaaggctcagtcatc**

**Fortunella_japonica_2** **gcatttcctctagcccccgaaaaagacattatatggactggattaaaaggctcagtcatc**

**Fortunella_japonica_3** **gcatttcctctagcccccgaaaaagacattatatggactggattaaaaggctcagtcatc**

**Fortunella_japonica_4** **gcatttcctctagcccccgaaaaagacattatatggactggattaaaaggctcagtcatc**

**Fortunella_japonica_5** **gcatttcctctagcccccgaaaaagacattatatggactggattaaaaggctcagtcatc**

**Fortunella_japonica_6** **gcatttcctctagcccccgaaaaagacattatatggactggattaaaaggctcagtcatc**

**Fortunella_japonica_7** **gcatttcctctagcccccgaaaaagacattatatggactggattaaaaggctcagtcatc**

**Fortunella_japonica_8** **gcatttcctctagcccccgaaaaagacattatatggactggattaaaaggctcagtcatc**

**Fortunella_japonica_9** **gcatttcctctagcccccgaaaaagacattatatggactggattaaaaggctcagtcatc**

**Fortunella_japonica_10** **gcatttcctctagcccccgaaaaagacattatatggactggattaaaaggctcagtcatc**

**Fortunella_japonica_11** **gcatttcctctagcccccgaaaaagacattatatggactggattaaaaggctcagtcatc**

**Fortunella_japonica_12** **gcatttcctctagcccccgaaaaagacattatatggactggattaaaaggctcagtcatc**

**Fortunella_margarita_1** **gcatttcctctagcccccgaaaaagacattatatggactggattaaaaggctcagtcatc**

**Fortunella_margarita_2** **gcatttcctctagcccccgaaaaagacattatatggactggattaaaaggctcagtcatc**

**Fortunella_venosa_1** **gcatttcctctagcccccgaaaaagacattatatggactggattaaaaggctcagtcatc**

**Fortunella_venosa_2** **gcatttcctctagcccccgaaaaagacattatatggactggattaaaaggctcagtcatc**

**Fortunella_venosa_3** **gcatttcctctagcccccgaaaaagacattatatggactggattaaaaggctcagtcatc**

22510	22520	22530	22540	22550	22560

....|....|....|....|....|....|....|....|....|....|....|....|

**Atalantia_kwangtungens** **ctaaaattaggattcatttcttgtcgcaaatattcacttgtagcataccatatctcaatg**

**Citrus_reticulata_1** **ctaaaattaggattcatttcttgtcgcaaatattcacttgtagcataccatatctcaatg**

**Citrus_reticulata_2** **ctaaaattaggattcatttcttgtcgcaaatattcacttgtagcataccatatctcaatg**

**Citrus_trifoliata_3** **ctaaaattaggattcatttcttgtcgcaaatattcacttgtagcataccatatctcaatg**

**Citrus_aurantiifolia** **ctaaaattaggattcatttcttgtcgcaaatattcacttgtagcataccatatctcaatg**

**Citrus_aurantium** **ctaaaattaggattcatttcttgtcgcaaatattcacttgtagcataccatatctcaatg**

**Citrus_cavaleriei** **ctaaaattaggattcatttcttgtcgcaaatattcacttgtagcataccatatctcaatg**

**Citrus_hongheensis** **ctaaaattaggattcatttcttgtcgcaaatattcacttgtagcataccatatctcaatg**

**Citrus_limon** **ctaaaattaggattcatttcttgtcgcaaatattcacttgtagcataccatatctcaatg**

**Citrus_maxima** **ctaaaattaggattcatttcttgtcgcaaatattcacttgtagcataccatatctcaatg**

**Citrus_medica** **ctaaaattaggattcatttcttgtcgcaaatattcacttgtagcataccatatctcaatg**

**Citrus_polytrifolia** **ctaaaattaggattcatttcttgtcgcaaatattcacttgtagcataccatatctcaatg**

**Citrus_reticulata** **ctaaaattaggattcatttcttgtcgcaaatattcacttgtagcataccatatctcaatg**

**Citrus_sinensis** **ctaaaattaggattcatttcttgtcgcaaatattcacttgtagcataccatatctcaatg**

**Fortunella_bawangica_1** **ctaaaattaggattcatttcttgtcgcaaatattcacttgtagcataccatatctcaatg**

**Fortunella_bawangica_2** **ctaaaattaggattcatttcttgtcgcaaatattcacttgtagcataccatatctcaatg**

**Fortunella_hindsii_1** **ctaaaattaggattcatttcttgtcgcaaatattcacttgtagcataccatatctcaatg**

**Fortunella_hindsii_2** **ctaaaattaggattcatttcttgtcgcaaatattcacttgtagcataccatatctcaatg**

**Fortunella_hindsii_3** **ctaaaattaggattcatttcttgtcgcaaatattcacttgtagcataccatatctcaatg**

**Fortunella_japonica_1** **ctaaaattaggattcatttcttgtcgcaaatattcacttgtagcataccatatctcaatg**

**Fortunella_japonica_2** **ctaaaattaggattcatttcttgtcgcaaatattcacttgtagcataccatatctcaatg**

**Fortunella_japonica_3** **ctaaaattaggattcatttcttgtcgcaaatattcacttgtagcataccatatctcaatg**

**Fortunella_japonica_4** **ctaaaattaggattcatttcttgtcgcaaatattcacttgtagcataccatatctcaatg**

**Fortunella_japonica_5** **ctaaaattaggattcatttcttgtcgcaaatattcacttgtagcataccatatctcaatg**

**Fortunella_japonica_6** **ctaaaattaggattcatttcttgtcgcaaatattcacttgtagcataccatatctcaatg**

**Fortunella_japonica_7** **ctaaaattaggattcatttcttgtcgcaaatattcacttgtagcataccatatctcaatg**

**Fortunella_japonica_8** **ctaaaattaggattcatttcttgtcgcaaatattcacttgtagcataccatatctcaatg**

**Fortunella_japonica_9** **ctaaaattaggattcatttcttgtcgcaaatattcacttgtagcataccatatctcaatg**

**Fortunella_japonica_10** **ctaaaattaggattcatttcttgtcgcaaatattcacttgtagcataccatatctcaatg**

**Fortunella_japonica_11** **ctaaaattaggattcatttcttgtcgcaaatattcacttgtagcataccatatctcaatg**

**Fortunella_japonica_12** **ctaaaattaggattcatttcttgtcgcaaatattcacttgtagcataccatatctcaatg**

**Fortunella_margarita_1** **ctaaaattaggattcatttcttgtcgcaaatattcacttgtagcataccatatctcaatg**

**Fortunella_margarita_2** **ctaaaattaggattcatttcttgtcgcaaatattcacttgtagcataccatatctcaatg**

**Fortunella_venosa_1** **ctaaaattaggattcatttcttgtcgcaaatattcacttgtagcataccatatctcaatg**

**Fortunella_venosa_2** **ctaaaattaggattcatttcttgtcgcaaatattcacttgtagcataccatatctcaatg**

**Fortunella_venosa_3** **ctaaaattaggattcatttcttgtcgcaaatattcacttgtagcataccatatctcaatg**

22570	22580	22590	22600	22610	22620

....|....|....|....|....|....|....|....|....|....|....|....|

**Atalantia_kwangtungens** **gattgccgtagtttttctatcgcgtgtacattcccataatgatagtgtttttccaaaata**

**Citrus_reticulata_1** **gattgtcgtagtttttctatcgcgtgtacattcccataatgatagtgtttttccaaaata**

**Citrus_reticulata_2** **gattgtcgtagtttttctatcgcgtgtacattcccataatgatagtgtttttccaaaata**

**Citrus_trifoliata_3** **gattgtcgtagtttttctatcgcgtgtacattcccataatgatagtgtttttccaaaata**

**Citrus_aurantiifolia** **gattgtcgtagtttttctatcgcgtgtacattcccataatgatagtgtttttccaaaata**

**Citrus_aurantium** **gattgtcgtagtttttctatcgcgtgtacattcccataatgatagtgtttttccaaaata**

**Citrus_cavaleriei** **gattgtcgtagtttttctatcgcgtgtacattcccataatgatagtgtttttccaaaata**

**Citrus_hongheensis** **gattgtcgtagtttttctatcgcgtgtacattcccataatgatagtgtttttccaaaata**

**Citrus_limon** **gattgtcgtagtttttctatcgcgtgtacattcccataatgatagtgtttttccaaaata**

**Citrus_maxima** **gattgtcgtagtttttctatcgcgtgtacattcccataatgatagtgtttttccaaaata**

**Citrus_medica** **gattgtcgtagtttttctatcgcgtgtacattcccataatgatagtgtttttccaaaata**

**Citrus_polytrifolia** **gattgtcgtagtttttctatcgcgtgtacattcccataatgatagtgtttttccaaaata**

**Citrus_reticulata** **gattgtcgtagtttttctatcgcgtgtacattcccataatgatagtgtttttccaaaata**

**Citrus_sinensis** **gattgtcgtagtttttctatcgcgtgtacattcccataatgatagtgtttttccaaaata**

**Fortunella_bawangica_1** **gattgtcgtagtttttctatcgcgtgtacattcccataatgatagtgtttttccaaaata**

**Fortunella_bawangica_2** **gattgtcgtagtttttctatcgcgtgtacattcccataatgatagtgtttttccaaaata**

**Fortunella_hindsii_1** **gattgtcgtagtttttctatcgcgtgtacattcccataatgatagtgtttttccaaaata**

**Fortunella_hindsii_2** **gattgtcgtagtttttctatcgcgtgtacattcccataatgatagtgtttttccaaaata**

**Fortunella_hindsii_3** **gattgtcgtagtttttctatcgcgtgtacattcccataatgatagtgtttttccaaaata**

**Fortunella_japonica_1** **gattgtcgtagtttttctatcgcgtgtacattcccataatgatagtgtttttccaaaata**

**Fortunella_japonica_2** **gattgtcgtagtttttctatcgcgtgtacattcccataatgatagtgtttttccaaaata**

**Fortunella_japonica_3** **gattgtcgtagtttttctatcgcgtgtacattcccataatgatagtgtttttccaaaata**

**Fortunella_japonica_4** **gattgtcgtagtttttctatcgcgtgtacattcccataatgatagtgtttttccaaaata**

**Fortunella_japonica_5** **gattgtcgtagtttttctatcgcgtgtacattcccataatgatagtgtttttccaaaata**

**Fortunella_japonica_6** **gattgtcgtagtttttctatcgcgtgtacattcccataatgatagtgtttttccaaaata**

**Fortunella_japonica_7** **gattgtcgtagtttttctatcgcgtgtacattcccataatgatagtgtttttccaaaata**

**Fortunella_japonica_8** **gattgtcgtagtttttctatcgcgtgtacattcccataatgatagtgtttttccaaaata**

**Fortunella_japonica_9** **gattgtcgtagtttttctatcgcgtgtacattcccataatgatagtgtttttccaaaata**

**Fortunella_japonica_10** **gattgtcgtagtttttctatcgcgtgtacattcccataatgatagtgtttttccaaaata**

**Fortunella_japonica_11** **gattgtcgtagtttttctatcgcgtgtacattcccataatgatagtgtttttccaaaata**

**Fortunella_japonica_12** **gattgtcgtagtttttctatcgcgtgtacattcccataatgatagtgtttttccaaaata**

**Fortunella_margarita_1** **gattgtcgtagtttttctatcgcgtgtacattcccataatgatagtgtttttccaaaata**

**Fortunella_margarita_2** **gattgtcgtagtttttctatcgcgtgtacattcccataatgatagtgtttttccaaaata**

**Fortunella_venosa_1** **gattgtcgtagtttttctatcgcgtgtacattcccataatgatagtgtttttccaaaata**

**Fortunella_venosa_2** **gattgtcgtagtttttctatcgcgtgtacattcccataatgatagtgtttttccaaaata**

**Fortunella_venosa_3** **gattgtcgtagtttttctatcgcgtgtacattcccataatgatagtgtttttccaaaata**

22630	22640	22650	22660	22670	22680

....|....|....|....|....|....|....|....|....|....|....|....|

**Atalantia_kwangtungens** **aaactttgttgttcagcatcttggactagccatcgcttagaaggtatcgttaaaagatca**

**Citrus_reticulata_1** **aaactttgttgttcagcatcttggactagccatcgcttagaaggtatcgttaaaagatca**

**Citrus_reticulata_2** **aaactttgttgttcagcatcttggactagccatcgcttagaaggtatcgttaaaagatca**

**Citrus_trifoliata_3** **aaactttgttgttcagcatcttggactagccatcgcttagaaggtatcgttaaaagatca**

**Citrus_aurantiifolia** **aaactttgttgttcagcatcttggactagccatcgcttagaaggtatcgttaaaagatca**

**Citrus_aurantium** **aaactttgttgttcagcatcttggactagccatcgcttagaaggtatcgttaaaagatca**

**Citrus_cavaleriei** **aaactttgttgttcagcatcttggactagccatcgcttagaaggtatcgttaaaagatca**

**Citrus_hongheensis** **aaactttgttgttcagcatcttggactagccatcgcttagaaggtatcgttaaaagatca**

**Citrus_limon** **aaactttgttgttcagcatcttggactagccatcgcttagaaggtatcgttaaaagatca**

**Citrus_maxima** **aaactttgttgttcagcatcttggactagccatcgcttagaaggtatcgttaaaagatca**

**Citrus_medica** **aaactttgttgttcagcatcttggactagccatcgcttagaaggtatcgttaaaagatca**

**Citrus_polytrifolia** **aaactttgttgttcagcatcttggactagccatcgcttagaaggtatcgttaaaagatca**

**Citrus_reticulata** **aaactttgttgttcagcatcttggactagccatcgcttagaaggtatcgttaaaagatca**

**Citrus_sinensis** **aaactttgttgttcagcatcttggactagccatcgcttagaaggtatcgttaaaagatca**

**Fortunella_bawangica_1** **aaactttgttgttcagcatcttggactagccatcgcttagaaggtatcgttaaaagatca**

**Fortunella_bawangica_2** **aaactttgttgttcagcatcttggactagccatcgcttagaaggtatcgttaaaagatca**

**Fortunella_hindsii_1** **aaactttgttgttcagcatcttggactagccatcgcttagaaggtatcgttaaaagatca**

**Fortunella_hindsii_2** **aaactttgttgttcagcatcttggactagccatcgcttagaaggtatcgttaaaagatca**

**Fortunella_hindsii_3** **aaactttgttgttcagcatcttggactagccatcgcttagaaggtatcgttaaaagatca**

**Fortunella_japonica_1** **aaactttgttgttcagcatcttggactagccatcgcttagaaggtatcgttaaaagatca**

**Fortunella_japonica_2** **aaactttgttgttcagcatcttggactagccatcgcttagaaggtatcgttaaaagatca**

**Fortunella_japonica_3** **aaactttgttgttcagcatcttggactagccatcgcttagaaggtatcgttaaaagatca**

**Fortunella_japonica_4** **aaactttgttgttcagcatcttggactagccatcgcttagaaggtatcgttaaaagatca**

**Fortunella_japonica_5** **aaactttgttgttcagcatcttggactagccatcgcttagaaggtatcgttaaaagatca**

**Fortunella_japonica_6** **aaactttgttgttcagcatcttggactagccatcgcttagaaggtatcgttaaaagatca**

**Fortunella_japonica_7** **aaactttgttgttcagcatcttggactagccatcgcttagaaggtatcgttaaaagatca**

**Fortunella_japonica_8** **aaactttgttgttcagcatcttggactagccatcgcttagaaggtatcgttaaaagatca**

**Fortunella_japonica_9** **aaactttgttgttcagcatcttggactagccatcgcttagaaggtatcgttaaaagatca**

**Fortunella_japonica_10** **aaactttgttgttcagcatcttggactagccatcgcttagaaggtatcgttaaaagatca**

**Fortunella_japonica_11** **aaactttgttgttcagcatcttggactagccatcgcttagaaggtatcgttaaaagatca**

**Fortunella_japonica_12** **aaactttgttgttcagcatcttggactagccatcgcttagaaggtatcgttaaaagatca**

**Fortunella_margarita_1** **aaactttgttgttcagcatcttggactagccatcgcttagaaggtatcgttaaaagatca**

**Fortunella_margarita_2** **aaactttgttgttcagcatcttggactagccatcgcttagaaggtatcgttaaaagatca**

**Fortunella_venosa_1** **aaactttgttgttcagcatcttggactagccatcgcttagaaggtatcgttaaaagatca**

**Fortunella_venosa_2** **aaactttgttgttcagcatcttggactagccatcgcttagaaggtatcgttaaaagatca**

**Fortunella_venosa_3** **aaactttgttgttcagcatcttggactagccatcgcttagaaggtatcgttaaaagatca**

22690	22700	22710	22720	22730	22740

....|....|....|....|....|....|....|....|....|....|....|....|

**Atalantia_kwangtungens** **tcaatgcctaatgaaatagatgtagcagtggcttgctggaaccccagggtctttacttga**

**Citrus_reticulata_1** **tcaatgcctaatgaaatagatgtagcagtggcttgctggaaccccagggtctttacttga**

**Citrus_reticulata_2** **tcaatgcctaatgaaatagatgtagcagtggcttgctggaaccccagggtctttacttga**

**Citrus_trifoliata_3** **tcaatgcctaatgaaatagatgtagcagtggcttgctggaaccccagggtctttacttga**

**Citrus_aurantiifolia** **tcaatgcctaatgaaatagatgtagcagtggcttgctggaaccccagggtctttacttga**

**Citrus_aurantium** **tcaatgcctaatgaaatagatgtagcagtggcttgctggaaccccagggtctttacttga**

**Citrus_cavaleriei** **tcaatgcctaatgaaatagatgtagcagtggcttgctggaaccccagggtctttacttga**

**Citrus_hongheensis** **tcaatgcctaatgaaatagatgtagcagtggcttgctggaaccccagggtctttacttga**

**Citrus_limon** **tcaatgcctaatgaaatagatgtagcagtggcttgctggaaccccagggtctttacttga**

**Citrus_maxima** **tcaatgcctaatgaaatagatgtagcagtggcttgctggaaccccagggtctttacttga**

**Citrus_medica** **tcaatgcctaatgaaatagatgtagcagtggcttgctggaaccccagggtctttacttga**

**Citrus_polytrifolia** **tcaatgcctaatgaaatagatgtagcagtggcttgctggaaccccagggtctttacttga**

**Citrus_reticulata** **tcaatgcctaatgaaatagatgtagcagtggcttgctggaaccccagggtctttacttga**

**Citrus_sinensis** **tcaatgcctaatgaaatagatgtagcagtggcttgctggaaccccagggtctttacttga**

**Fortunella_bawangica_1** **tcaatgcctaatgaaatagatgtagcagtggcttgctggaaccccagggtctttacttga**

**Fortunella_bawangica_2** **tcaatgcctaatgaaatagatgtagcagtggcttgctggaaccccagggtctttacttga**

**Fortunella_hindsii_1** **tcaatgcctaatgaaatagatgtagcagtggcttgctggaaccccagggtctttacttga**

**Fortunella_hindsii_2** **tcaatgcctaatgaaatagatgtagcagtggcttgctggaaccccagggtctttacttga**

**Fortunella_hindsii_3** **tcaatgcctaatgaaatagatgtagcagtggcttgctggaaccccagggtctttacttga**

**Fortunella_japonica_1** **tcaatgcctaatgaaatagatgtagcagtggcttgctggaaccccagggtctttacttga**

**Fortunella_japonica_2** **tcaatgcctaatgaaatagatgtagcagtggcttgctggaaccccagggtctttacttga**

**Fortunella_japonica_3** **tcaatgcctaatgaaatagatgtagcagtggcttgctggaaccccagggtctttacttga**

**Fortunella_japonica_4** **tcaatgcctaatgaaatagatgtagcagtggcttgctggaaccccagggtctttacttga**

**Fortunella_japonica_5** **tcaatgcctaatgaaatagatgtagcagtggcttgctggaaccccagggtctttacttga**

**Fortunella_japonica_6** **tcaatgcctaatgaaatagatgtagcagtggcttgctggaaccccagggtctttacttga**

**Fortunella_japonica_7** **tcaatgcctaatgaaatagatgtagcagtggcttgctggaaccccagggtctttacttga**

**Fortunella_japonica_8** **tcaatgcctaatgaaatagatgtagcagtggcttgctggaaccccagggtctttacttga**

**Fortunella_japonica_9** **tcaatgcctaatgaaatagatgtagcagtggcttgctggaaccccagggtctttacttga**

**Fortunella_japonica_10** **tcaatgcctaatgaaatagatgtagcagtggcttgctggaaccccagggtctttacttga**

**Fortunella_japonica_11** **tcaatgcctaatgaaatagatgtagcagtggcttgctggaaccccagggtctttacttga**

**Fortunella_japonica_12** **tcaatgcctaatgaaatagatgtagcagtggcttgctggaaccccagggtctttacttga**

**Fortunella_margarita_1** **tcaatgcctaatgaaatagatgtagcagtggcttgctggaaccccagggtctttacttga**

**Fortunella_margarita_2** **tcaatgcctaatgaaatagatgtagcagtggcttgctggaaccccagggtctttacttga**

**Fortunella_venosa_1** **tcaatgcctaatgaaatagatgtagcagtggcttgctggaaccccagggtctttacttga**

**Fortunella_venosa_2** **tcaatgcctaatgaaatagatgtagcagtggcttgctggaaccccagggtctttacttga**

**Fortunella_venosa_3** **tcaatgcctaatgaaatagatgtagcagtggcttgctggaaccccagggtctttacttga**

22750	22760	22770	22780	22790	22800

....|....|....|....|....|....|....|....|....|....|....|....|

**Atalantia_kwangtungens** **tccaggatgtgtgatgtatatgccattccgaagtgatctattaacctgctaataagtcgt**

**Citrus_reticulata_1** **tccaggatgtgggatgtatatgccattccgaagtgatctattaacctgctaataagtcgt**

**Citrus_reticulata_2** **tccaggatgtgggatgtatatgccattccgaagtgatctattaacctgctaataagtcgt**

**Citrus_trifoliata_3** **tccaggatgtgtgatgtatatgccattccgaagtgatctattaacctgctaataagtcgt**

**Citrus_aurantiifolia** **tccaggatgtgggatgtatatgccattccgaagtgatctattaacctgctaataagtcgt**

**Citrus_aurantium** **tccaggatgtgggatgtatatgccattccgaagtgatctattaacctgctaataagtcgt**

**Citrus_cavaleriei** **tccaggatgtgggatgtatatgccattccgaagtgatctattaacctgctaataagtcgt**

**Citrus_hongheensis** **tccaggatgtgggatgtatatgccattccgaagtgatctattaacctgctaataagtcgt**

**Citrus_limon** **tccaggatgtgggatgtatatgccattccgaagtgatctattaacctgctaataagtcgt**

**Citrus_maxima** **tccaggatgtgggatgtatatgccattccgaagtgatctattaacctgctaataagtcgt**

**Citrus_medica** **tccaggatgtgtgatgtatatgccattccgaagtgatctattaacctgctaataagtcgt**

**Citrus_polytrifolia** **tccaggatgtgtgatgtatatgccattccgaagtgatctattaacctgctaataagtcgt**

**Citrus_reticulata** **tccaggatgtgggatgtatatgccattccgaagtgatctattaacctgctaataagtcgt**

**Citrus_sinensis** **tccaggatgtgggatgtatatgccattccgaagtgatctattaacctgctaataagtcgt**

**Fortunella_bawangica_1** **tccaggatgtgtgatgtatatgccattccgaagtgatctattaacctgctaataagtcgt**

**Fortunella_bawangica_2** **tccaggatgtgtgatgtatatgccattccgaagtgatctattaacctgctaataagtcgt**

**Fortunella_hindsii_1** **tccaggatgtgtgatgtatatgccattccgaagtgatctattaacctgctaataagtcgt**

**Fortunella_hindsii_2** **tccaggatgtgtgatgtatatgccattccgaagtgatctattaacctgctaataagtcgt**

**Fortunella_hindsii_3** **tccaggatgtgtgatgtatatgccattccgaagtgatctattaacctgctaataagtcgt**

**Fortunella_japonica_1** **tccaggatgtgtgatgtatatgccattccgaagtgatctattaacctgctaataagtcgt**

**Fortunella_japonica_2** **tccaggatgtgtgatgtatatgccattccgaagtgatctattaacctgctaataagtcgt**

**Fortunella_japonica_3** **tccaggatgtgtgatgtatatgccattccgaagtgatctattaacctgctaataagtcgt**

**Fortunella_japonica_4** **tccaggatgtgtgatgtatatgccattccgaagtgatctattaacctgctaataagtcgt**

**Fortunella_japonica_5** **tccaggatgtgtgatgtatatgccattccgaagtgatctattaacctgctaataagtcgt**

**Fortunella_japonica_6** **tccaggatgtgtgatgtatatgccattccgaagtgatctattaacctgctaataagtcgt**

**Fortunella_japonica_7** **tccaggatgtgtgatgtatatgccattccgaagtgatctattaacctgctaataagtcgt**

**Fortunella_japonica_8** **tccaggatgtgtgatgtatatgccattccgaagtgatctattaacctgctaataagtcgt**

**Fortunella_japonica_9** **tccaggatgtgtgatgtatatgccattccgaagtgatctattaacctgctaataagtcgt**

**Fortunella_japonica_10** **tccaggatgtgtgatgtatatgccattccgaagtgatctattaacctgctaataagtcgt**

**Fortunella_japonica_11** **tccaggatgtgtgatgtatatgccattccgaagtgatctattaacctgctaataagtcgt**

**Fortunella_japonica_12** **tccaggatgtgtgatgtatatgccattccgaagtgatctattaacctgctaataagtcgt**

**Fortunella_margarita_1** **tccaggatgtgtgatgtatatgccattccgaagtgatctattaacctgctaataagtcgt**

**Fortunella_margarita_2** **tccaggatgtgtgatgtatatgccattccgaagtgatctattaacctgctaataagtcgt**

**Fortunella_venosa_1** **tccaggatgtgtgatgtatatgccattccgaagtgatctattaacctgctaataagtcgt**

**Fortunella_venosa_2** **tccaggatgtgtgatgtatatgccattccgaagtgatctattaacctgctaataagtcgt**

**Fortunella_venosa_3** **tccaggatgtgtgatgtatatgccattccgaagtgatctattaacctgctaataagtcgt**

22810	22820	22830	22840	22850	22860

....|....|....|....|....|....|....|....|....|....|....|....|

**Atalantia_kwangtungens** **ttaatggcagttccatctatcattttattgtgaaagaccagactcacccgttctgccata**

**Citrus_reticulata_1** **ttaatggcagttccatctatcattttattgtgaaagaccagactcacccgttctgccata**

**Citrus_reticulata_2** **ttaatggcagttccatctatcattttattgtgaaagaccagactcacccgttctgccata**

**Citrus_trifoliata_3** **ttaatggcagttccatctatcattttattgtgaaagaccagactccctcgttctgccata**

**Citrus_aurantiifolia** **ttaatggcagttccatctatcattttattgtgaaagaccagactcacccgttctgccata**

**Citrus_aurantium** **ttaatggcagttccatctatcattttattgtgaaagaccagactcacccgttctgccata**

**Citrus_cavaleriei** **ttaatggcagttccatctatcattttattgtgaaagaccagactcacccgttctgccata**

**Citrus_hongheensis** **ttaatggcagttccatctatcattttattgtgaaagaccagactcacccgttctgccata**

**Citrus_limon** **ttaatggcagttccatctatcattttattgtgaaagaccagactcacccgttctgccata**

**Citrus_maxima** **ttaatggcagttccatctatcattttattgtgaaagaccagactcacccgttctgccata**

**Citrus_medica** **ttaatggcagttccatctatcattttattgtgaaagaccagactcacccgttctgccata**

**Citrus_polytrifolia** **ttaatggcagttccatctatcattttattgtgaaagaccagactccctcgttctgccata**

**Citrus_reticulata** **ttaatggcagttccatctatcattttattgtgaaagaccagactcacccgttctgccata**

**Citrus_sinensis** **ttaatggcagttccatctatcattttattgtgaaagaccagactcacccgttctgccata**

**Fortunella_bawangica_1** **ttaatggcagttccatctatcattttattgtgaaagaccagactcacccgttctgccata**

**Fortunella_bawangica_2** **ttaatggcagttccatctatcattttattgtgaaagaccagactcacccgttctgccata**

**Fortunella_hindsii_1** **ttaatggcagttccatctatcattttattgtgaaagaccagactcacccgttctgccata**

**Fortunella_hindsii_2** **ttaatggcagttccatctatcattttattgtgaaagaccagactcacccgttctgccata**

**Fortunella_hindsii_3** **ttaatggcagttccatctatcattttattgtgaaagaccagactcacccgttctgccata**

**Fortunella_japonica_1** **ttaatggcagttccatctatcattttattgtgaaagaccagactcacccgttctaccata**

**Fortunella_japonica_2** **ttaatggcagttccatctatcattttattgtgaaagaccagactcacccgttctgccata**

**Fortunella_japonica_3** **ttaatggcagttccatctatcattttattgtgaaagaccagactcacccgttctaccata**

**Fortunella_japonica_4** **ttaatggcagttccatctatcattttattgtgaaagaccagactcacccgttctaccata**

**Fortunella_japonica_5** **ttaatggcagttccatctatcattttattgtgaaagaccagactcacccgttctaccata**

**Fortunella_japonica_6** **ttaatggcagttccatctatcattttattgtgaaagaccagactcacccgttctaccata**

**Fortunella_japonica_7** **ttaatggcagttccatctatcattttattgtgaaagaccagactcacccgttctaccata**

**Fortunella_japonica_8** **ttaatggcagttccatctatcattttattgtgaaagaccagactcacccgttctaccata**

**Fortunella_japonica_9** **ttaatggcagttccatctatcattttattgtgaaagaccagactcacccgttctaccata**

**Fortunella_japonica_10** **ttaatggcagttccatctatcattttattgtgaaagaccagactcacccgttctgccata**

**Fortunella_japonica_11** **ttaatggcagttccatctatcattttattgtgaaagaccagactcacccgttctgccata**

**Fortunella_japonica_12** **ttaatggcagttccatctatcattttattgtgaaagaccagactcacccgttctaccata**

**Fortunella_margarita_1** **ttaatggcagttccatctatcattttattgtgaaagaccagactcacccgttctgccata**

**Fortunella_margarita_2** **ttaatggcagttccatctatcattttattgtgaaagaccagactcacccgttctgccata**

**Fortunella_venosa_1** **ttaatggcagttccatctatcattttattgtgaaagaccagactcacccgttctgccata**

**Fortunella_venosa_2** **ttaatggcagttccatctatcattttattgtgaaagaccagactcacccgttctgccata**

**Fortunella_venosa_3** **ttaatggcagttccatctatcattttattgtgaaagaccagactcacccgttctgccata**

22870	22880	22890	22900	22910	22920

....|....|....|....|....|....|....|....|....|....|....|....|

**Atalantia_kwangtungens** **agtacctccgtattccgctgagtaggattcgccaatggattttagtcaatgagtggaaaa**

**Citrus_reticulata_1** **agtacctccgtattccgctgagtaggattcgccaatggattttagtcaatgagtggaaaa**

**Citrus_reticulata_2** **agtacctccgtattccgctgagtaggattcgccaatggattttagtcaatgagtggaaaa**

**Citrus_trifoliata_3** **agtacctccgtattccgctgagtaggattcgccaatggattttagtcaatgagtggaaaa**

**Citrus_aurantiifolia** **agtacctccgtattccgctgagtaggattcgccaatggattttagtcaatgagtggaaaa**

**Citrus_aurantium** **agtacctccgtattccgctgagtaggattcgccaatggattttagtcaatgagtggaaaa**

**Citrus_cavaleriei** **agtacctccgtattccgctgagtaggattcgccaatggattttagtcaatgagtggaaaa**

**Citrus_hongheensis** **agtacctccgtattccgctgagtaggattcgccaatggattttagtcaatgagtggaaaa**

**Citrus_limon** **agtacctccgtattccgctgagtaggattcgccaatggattttagtcaatgagtggaaaa**

**Citrus_maxima** **agtacctccgtattccgctgagtaggattcgccaatggattttagtcaatgagtggaaaa**

**Citrus_medica** **agtacctccgtattccgctgagtaggattcgccaatggattttagtcaatgagtggaaaa**

**Citrus_polytrifolia** **agtacctccgtattccgctgagtaggattcaccaatggattttagtcaatgagtggaaaa**

**Citrus_reticulata** **agtacctccgtattccgctgagtaggattcgccaatggattttagtcaatgagtggaaaa**

**Citrus_sinensis** **agtacctccgtattccgctgagtaggattcgccaatggattttagtcaatgagtggaaaa**

**Fortunella_bawangica_1** **agtacctccgtattccgctgagtaggattcgccaatggattttagtcaatgagtggaaaa**

**Fortunella_bawangica_2** **agtacctccgtattccgctgagtaggattcgccaatggattttagtcaatgagtggaaaa**

**Fortunella_hindsii_1** **agtacctccgtattccgctgagtaggattcgccaatggattttagtcaatgagtggaaaa**

**Fortunella_hindsii_2** **agtacctccgtattccgctgagtaggattcgccaatggattttagtcaatgagtggaaaa**

**Fortunella_hindsii_3** **agtacctccgtattccgctgagtaggattcgccaatggattttagtcaatgagtggaaaa**

**Fortunella_japonica_1** **agtacctccgtattccgctgagtaggattcgccaatggattttagtcaatgagtggaaaa**

**Fortunella_japonica_2** **agtacctccgtattccgctgagtaggattcgccaatggattttagtcaatgagtggaaaa**

**Fortunella_japonica_3** **agtacctccgtattccgctgagtaggattcgccaatggattttagtcaatgagtggaaaa**

**Fortunella_japonica_4** **agtacctccgtattccgctgagtaggattcgccaatggattttagtcaatgagtggaaaa**

**Fortunella_japonica_5** **agtacctccgtattccgctgagtaggattcgccaatggattttagtcaatgagtggaaaa**

**Fortunella_japonica_6** **agtacctccgtattccgctgagtaggattcgccaatggattttagtcaatgagtggaaaa**

**Fortunella_japonica_7** **agtacctccgtattccgctgagtaggattcgccaatggattttagtcaatgagtggaaaa**

**Fortunella_japonica_8** **agtacctccgtattccgctgagtaggattcgccaatggattttagtcaatgagtggaaaa**

**Fortunella_japonica_9** **agtacctccgtattccgctgagtaggattcgccaatggattttagtcaatgagtggaaaa**

**Fortunella_japonica_10** **agtacctccgtattccgctgagtaggattcgccaatggattttagtcaatgagtggaaaa**

**Fortunella_japonica_11** **agtacctccgtattccgctgagtaggattcgccaatggattttagtcaatgagtggaaaa**

**Fortunella_japonica_12** **agtacctccgtattccgctgagtaggattcgccaatggattttagtcaatgagtggaaaa**

**Fortunella_margarita_1** **agtacctccgtattccgctgagtaggattcgccaatggattttagtcaatgagtggaaaa**

**Fortunella_margarita_2** **agtacctccgtattccgctgagtaggattcgccaatggattttagtcaatgagtggaaaa**

**Fortunella_venosa_1** **agtacctccgtattccgctgagtaggattcgccaatggattttagtcaatgagtggaaaa**

**Fortunella_venosa_2** **agtacctccgtattccgctgagtaggattcgccaatggattttagtcaatgagtggaaaa**

**Fortunella_venosa_3** **agtacctccgtattccgctgagtaggattcgccaatggattttagtcaatgagtggaaaa**

22930	22940	22950	22960	22970	22980

....|....|....|....|....|....|....|....|....|....|....|....|

**Atalantia_kwangtungens** **cttccttttctcgatcttgattcgcgtagaaaggtcagcaatggtggtcatacttgaacc**

**Citrus_reticulata_1** **cttccttttctcgatcttgattcgcgtagaaaggtcagcaatggtggtcatacttgaacc**

**Citrus_reticulata_2** **cttccttttctcgatcttgattcgcgtagaaaggtcagcaatggtggtcatacttgaacc**

**Citrus_trifoliata_3** **cttccttttctcgatcttgattcgcgtagaaaggtcagcaatggtggtcatacttgaacc**

**Citrus_aurantiifolia** **cttccttttctcgatcttgattcgcgtagaaaggtcagcaatggtggtcatacttgaacc**

**Citrus_aurantium** **cttccttttctcgatcttgattcgcgtagaaaggtcagcaatggtggtcatacttgaacc**

**Citrus_cavaleriei** **cttccttttctcgatcttgattcgcgtagaaaggtcagcaatggtggtcatacttgaacc**

**Citrus_hongheensis** **cttccttttctcgatcttgattcgcgtagaaaggtcagcaatggtggtcatacttgaacc**

**Citrus_limon** **cttccttttctcgatcttgattcgcgtagaaaggtcagcaatggtggtcatacttgaacc**

**Citrus_maxima** **cttccttttctcgatcttgattcgcgtagaaaggtcagcaatggtggtcatacttgaacc**

**Citrus_medica** **cttccttttctcgatcttgattcgcgtagaaaggtcagcaatggtggtcatacttgaacc**

**Citrus_polytrifolia** **cttccttttctcgatcttgattcgcgtagaaaggtcagcaatggtggtcatacttgaacc**

**Citrus_reticulata** **cttccttttctcgatcttgattcgcgtagaaaggtcagcaatggtggtcatacttgaacc**

**Citrus_sinensis** **cttccttttctcgatcttgattcgcgtagaaaggtcagcaatggtggtcatacttgaacc**

**Fortunella_bawangica_1** **cttccttttctcgatcttgattcgcgtagaaaggtcagcaatggtggtcatacttgaacc**

**Fortunella_bawangica_2** **cttccttttctcgatcttgattcgcgtagaaaggtcagcaatggtggtcatacttgaacc**

**Fortunella_hindsii_1** **cttccttttctcgatcttgattcgcgtagaaaggtcagcaatggtggtcatacttgaacc**

**Fortunella_hindsii_2** **cttccttttctcgatcttgattcgcgtagaaaggtcagcaatggtggtcatacttgaacc**

**Fortunella_hindsii_3** **cttccttttctcgatcttgattcgcgtagaaaggtcagcaatggtggtcatacttgaacc**

**Fortunella_japonica_1** **cttccttttctcgatcttgattcgcgtagaaaggtcagcaatggtggtcatacttgaacc**

**Fortunella_japonica_2** **cttccttttctcgatcttgattcgcgtagaaaggtcagcaatggtggtcatacttgaacc**

**Fortunella_japonica_3** **cttccttttctcgatcttgattcgcgtagaaaggtcagcaatggtggtcatacttgaacc**

**Fortunella_japonica_4** **cttccttttctcgatcttgattcgcgtagaaaggtcagcaatggtggtcatacttgaacc**

**Fortunella_japonica_5** **cttccttttctcgatcttgattcgcgtagaaaggtcagcaatggtggtcatacttgaacc**

**Fortunella_japonica_6** **cttccttttctcgatcttgattcgcgtagaaaggtcagcaatggtggtcatacttgaacc**

**Fortunella_japonica_7** **cttccttttctcgatcttgattcgcgtagaaaggtcagcaatggtggtcatacttgaacc**

**Fortunella_japonica_8** **cttccttttctcgatcttgattcgcgtagaaaggtcagcaatggtggtcatacttgaacc**

**Fortunella_japonica_9** **cttccttttctcgatcttgattcgcgtagaaaggtcagcaatggtggtcatacttgaacc**

**Fortunella_japonica_10** **cttccttttctcgatcttgattcgcgtagaaaggtcagcaatggtggtcatacttgaacc**

**Fortunella_japonica_11** **cttccttttctcgatcttgattcgcgtagaaaggtccgcaatggtggtcatacttgaacc**

**Fortunella_japonica_12** **cttccttttctcgatcttgattcgcgtagaaaggtcagcaatggtggtcatacttgaacc**

**Fortunella_margarita_1** **cttccttttctcgatcttgattcgcgtagaaaggtcagcaatggtggtcatacttgaacc**

**Fortunella_margarita_2** **cttccttttctcgatcttgattcgcgtagaaaggtcagcaatggtggtcatacttgaacc**

**Fortunella_venosa_1** **cttccttttctcgatcttgattcgcgtagaaaggtcagcaatggtggtcatacttgaacc**

**Fortunella_venosa_2** **cttccttttctcgatcttgattcgcgtagaaaggtcagcaatggtggtcatacttgaacc**

**Fortunella_venosa_3** **cttccttttctcgatcttgattcgcgtagaaaggtcagcaatggtggtcatacttgaacc**

22990	23000	23010	23020	23030	23040

....|....|....|....|....|....|....|....|....|....|....|....|

**Atalantia_kwangtungens** **ggaaaggctcaaggagtcgtagaattacttagtttagacaccagatgattaggtaccata**

**Citrus_reticulata_1** **ggaaaggcccaaggagtcgtagaattacttagtttagacaccagatgattaggtaccata**

**Citrus_reticulata_2** **ggaaaggcccaaggagtcgtagaattacttagtttagacaccagatgattaggtaccata**

**Citrus_trifoliata_3** **ggaaaggcccacggagtcgtagaattacttagtttagacaccagatgattaggtaccata**

**Citrus_aurantiifolia** **ggaaaggcccaaggagtcgtagaattacttagtttagacaccagatgattaggtaccata**

**Citrus_aurantium** **ggaaaggcccaaggagtcgtagaattacttagtttagacaccagatgattaggtaccata**

**Citrus_cavaleriei** **ggaaaggcccaaggagtcgtagaattacttagtttagacaccagatgattaggtaccata**

**Citrus_hongheensis** **ggaaaggcccaaggagtcgtagaattacttagtttagacaccagatgattaggtaccata**

**Citrus_limon** **ggaaaggcccaaggagtcgtagaattacttagtttagacaccagatgattaggtaccata**

**Citrus_maxima** **ggaaaggcccaaggagtcgtagaattacttagtttagacaccagatgattaggtaccata**

**Citrus_medica** **ggaaaagcccaaggagtcgtagaattacttagtttagacaccagatgattaggtaccata**

**Citrus_polytrifolia** **ggaaaggcccacggagtcgtagaattacttagtttagacaccagatgattaggtaccata**

**Citrus_reticulata** **ggaaaggcccaaggagtcgtagaattacttagtttagacaccagatgattaggtaccata**

**Citrus_sinensis** **ggaaaggcccaaggagtcgtagaattacttagtttagacaccagatgattaggtaccata**

**Fortunella_bawangica_1** **ggaaaggcccaaggagtcgtagaattacttagtttagacaccagatgattaggtaccata**

**Fortunella_bawangica_2** **ggaaaggcccaaggagtcgtagaattacttagtttagacaccagatgattaggtaccata**

**Fortunella_hindsii_1** **ggaaaggcccaaggagtcgtagaattacttagtttagacaccagatgattaggtaccata**

**Fortunella_hindsii_2** **ggaaaggcccaaggagtcgtagaattacttagtttagacaccagatgattaggtaccata**

**Fortunella_hindsii_3** **ggaaaggcccaaggagtcgtagaattacttagtttagacaccagatgattaggtaccata**

**Fortunella_japonica_1** **ggaaaggcccaaggagtcgtagaattacttagtttagacaccagatgattaggtaccata**

**Fortunella_japonica_2** **ggaaaggcccaaggagtcgtagaattacttagtttagacaccagatgattaggtaccata**

**Fortunella_japonica_3** **ggaaaggcccaaggagtcgtagaattacttagtttagacaccagatgattaggtaccata**

**Fortunella_japonica_4** **ggaaaggcccaaggagtcgtagaattacttagtttagacaccagatgattaggtaccata**

**Fortunella_japonica_5** **ggaaaggcccaaggagtcgtagaattacttagtttagacaccagatgattaggtaccata**

**Fortunella_japonica_6** **ggaaaggcccaaggagtcgtagaattacttagtttagacaccagatgattaggtaccata**

**Fortunella_japonica_7** **ggaaaggcccaaggagtcgtagaattacttagtttagacaccagatgattaggtaccata**

**Fortunella_japonica_8** **ggaaaggcccaaggagtcgtagaattacttagtttagacaccagatgattaggtaccata**

**Fortunella_japonica_9** **ggaaaggcccaaggagtcgtagaattacttagtttagacaccagatgattaggtaccata**

**Fortunella_japonica_10** **ggaaaggcccaaggagtcgtagaattacttagtttagacaccagatgattaggtaccata**

**Fortunella_japonica_11** **ggaaaggcccaaggagtcgtagaattacttagtttagacaccagatgattaggtaccata**

**Fortunella_japonica_12** **ggaaaggcccaaggagtcgtagaattacttagtttagacaccagatgattaggtaccata**

**Fortunella_margarita_1** **ggaaaggcccaaggagtcgtagaattacttagtttagacaccagatgattaggtaccata**

**Fortunella_margarita_2** **ggaaaggcccaaggagtcgtagaattacttagtttagacaccagatgattaggtaccata**

**Fortunella_venosa_1** **ggaaaggcccaaggagtcgtagaattacttagtttagacaccagatgattaggtaccata**

**Fortunella_venosa_2** **ggaaaggcccaaggagtcgtagaattacttagtttagacaccagatgattaggtaccata**

**Fortunella_venosa_3** **ggaaaggcccaaggagtcgtagaattacttagtttagacaccagatgattaggtaccata**

23050	23060	23070	23080	23090	23100

....|....|....|....|....|....|....|....|....|....|....|....|

**Atalantia_kwangtungens** **tgagcaggcccggcaaaacccttgtatagcttcttcgatttctcgataaagagaaatatg**

**Citrus_reticulata_1** **tgagcaggcccggcaaaacccttgtatagcttcttcgatttctcgataaagagaaatatg**

**Citrus_reticulata_2** **tgagcaggcccggcaaaacccttgtatagcttcttcgatttctcgataaagagaaatatg**

**Citrus_trifoliata_3** **tgagcaggcccggcaaaacccttgtatagcttcttcgatttctcgataaagagaaatatg**

**Citrus_aurantiifolia** **tgagcaggcccggcaaaacccttgtatagcttcttcgatttctcgataaagagaaatatg**

**Citrus_aurantium** **tgagcaggcccggcaaaacccttgtatagcttcttcgatttctcgataaagagaaatatg**

**Citrus_cavaleriei** **tgagcaggcccggcaaaacccttgtatagcttcttcgatttctcgataaagagaaatatg**

**Citrus_hongheensis** **tgagcaggcccggcaaaacccttgtatagcttcttcgatttctcgataaagagaaatatg**

**Citrus_limon** **tgagcaggcccggcaaaacccttgtatagcttcttcgatttctcgataaagagaaatatg**

**Citrus_maxima** **tgagcaggcccggcaaaacccttgtatagcttcttcgatttctcgataaagagaaatatg**

**Citrus_medica** **tgagcaggcccggcaaaacccttgtatagcttcttcgatttctcgataaagagaaatatg**

**Citrus_polytrifolia** **tgagcaggcccggcaaaacccttgtatagcttcttcgatttctcgataaagagaaatatg**

**Citrus_reticulata** **tgagcaggcccggcaaaacccttgtatagcttcttcgatttctcgataaagagaaatatg**

**Citrus_sinensis** **tgagcaggcccggcaaaacccttgtatagcttcttcgatttctcgataaagagaaatatg**

**Fortunella_bawangica_1** **tgagcaggcccggcaaaacccttgtatagcttcttcgatttctcgataaagagaaatatg**

**Fortunella_bawangica_2** **tgagcaggcccggcaaaacccttgtatagcttcttcgatttctcgataaagagaaatatg**

**Fortunella_hindsii_1** **tgagcaggcccggcaaaacccttgtatagcttcttcgatttctcgataaagagaaatatg**

**Fortunella_hindsii_2** **tgagcaggcccggcaaaacccttgtatagcttcttcgatttctcgataaagagaaatatg**

**Fortunella_hindsii_3** **tgagcaggcccggcaaaacccttgtatagcttcttcgatttctcgataaagagaaatatg**

**Fortunella_japonica_1** **tgagcaggcccggcaaaacccttgtatagcttcttcgatttctcgataaagagaaatatg**

**Fortunella_japonica_2** **tgagcaggcccggcaaaacccttgtatagcttcttcgatttctcgataaagagaaatatg**

**Fortunella_japonica_3** **tgagcaggcccggcaaaacccttgtatagcttcttcgatttctcgataaagagaaatatg**

**Fortunella_japonica_4** **tgagcaggcccggcaaaacccttgtatagcttcttcgatttctcgataaagagaaatatg**

**Fortunella_japonica_5** **tgagcaggcccggcaaaacccttgtatagcttcttcgatttctcgataaagagaaatatg**

**Fortunella_japonica_6** **tgagcaggcccggcaaaacccttgtatagcttcttcgatttctcgataaagagaaatatg**

**Fortunella_japonica_7** **tgagcaggcccggcaaaacccttgtatagcttcttcgatttctcgataaagagaaatatg**

**Fortunella_japonica_8** **tgagcaggcccggcaaaacccttgtatagcttcttcgatttctcgataaagagaaatatg**

**Fortunella_japonica_9** **tgagcaggcccggcaaaacccttgtatagcttcttcgatttctcgataaagagaaatatg**

**Fortunella_japonica_10** **tgagcaggcccggcaaaacccttgtatagcttcttcgatttctcgataaagagaaatatg**

**Fortunella_japonica_11** **tgagcaggcccggcaaaacccttgtatagcttcttcgatttctcgataaagagaaatatg**

**Fortunella_japonica_12** **tgagcaggcccggcaaaacccttgtatagcttcttcgatttctcgataaagagaaatatg**

**Fortunella_margarita_1** **tgagcaggcccggcaaaacccttgtatagcttcttcgatttctcgataaagagaaatatg**

**Fortunella_margarita_2** **tgagcaggcccggcaaaacccttgtatagcttcttcgatttctcgataaagagaaatatg**

**Fortunella_venosa_1** **tgagcaggcccggcaaaacccttgtatagcttcttcgatttctcgataaagagaaatatg**

**Fortunella_venosa_2** **tgagcaggcccggcaaaacccttgtatagcttcttcgatttctcgataaagagaaatatg**

**Fortunella_venosa_3** **tgagcaggcccggcaaaacccttgtatagcttcttcgatttctcgataaagagaaatatg**

23110	23120	23130	23140	23150	23160

....|....|....|....|....|....|....|....|....|....|....|....|

**Atalantia_kwangtungens** **gcccacggtggttcgaatgtatatagaaagaatttctttttttacacttcgtactattag**

**Citrus_reticulata_1** **gcccacggtggttcgaatgtatatagaaagaatttctttttttacacttcgtactattag**

**Citrus_reticulata_2** **gcccacggtggttcgaatgtatatagaaagaatttctttttttacacttcgtactattag**

**Citrus_trifoliata_3** **gcccacggtggttcgaatgtatatagaaagaatttctttttttacacttcgtactattag**

**Citrus_aurantiifolia** **gcccacggtggttcgaatgtatatagaaagaatttctttttttacacttcgtactattag**

**Citrus_aurantium** **gcccacggtggttcgaatgtatatagaaagaatttctttttttacacttcgtactattag**

**Citrus_cavaleriei** **gcccacggtggttcgaatgtatatagaaagaatttctttttttacacttcgtactattag**

**Citrus_hongheensis** **gcccacggtggttcgaatgtatatagaaagaatttctttttttacacttcgtactattag**

**Citrus_limon** **gcccacggtggttcgaatgtatatagaaagaatttctttttttacacttcgtactattag**

**Citrus_maxima** **gcccacggtggttcgaatgtatatagaaagaatttctttttttacacttcgtactattag**

**Citrus_medica** **gcccacggtggttcgaatgtatatagaaagaatttctttttttacacttcgtactattag**

**Citrus_polytrifolia** **gcccacggtggttcgaatgtatatagaaagaatttctttttttacacttcgtactattag**

**Citrus_reticulata** **gcccacggtggttcgaatgtatatagaaagaatttctttttttacacttcgtactattag**

**Citrus_sinensis** **gcccacggtggttcgaatgtatatagaaagaatttctttttttacacttcgtactattag**

**Fortunella_bawangica_1** **gcccacggtggttcgaatgtatatagaaagaatttctttttttacacttcgtactattag**

**Fortunella_bawangica_2** **gcccacggtggttcgaatgtatatagaaagaatttctttttttacacttcgtactattag**

**Fortunella_hindsii_1** **gcccacggtggttcgaatgtatatagaaagaatttctttttttacacttcgtactattag**

**Fortunella_hindsii_2** **gcccacggtggttcgaatgtatatagaaagaatttctttttttacacttcgtactattag**

**Fortunella_hindsii_3** **gcccacggtggttcgaatgtatatagaaagaatttctttttttacacttcgtactattag**

**Fortunella_japonica_1** **gcccacggtggttcgaatgtatatagaaagaatttctttttttacacttcgtactattag**

**Fortunella_japonica_2** **gcccacggtggttcgaatgtatatagaaagaatttctttttttacacttcgtactattag**

**Fortunella_japonica_3** **gcccacggtggttcgaatgtatatagaaagaatttctttttttacacttcgtactattag**

**Fortunella_japonica_4** **gcccacggtggttcgaatgtatatagaaagaatttctttttttacacttcgtactattag**

**Fortunella_japonica_5** **gcccacggtggttcgaatgtatatagaaagaatttctttttttacacttcgtactattag**

**Fortunella_japonica_6** **gcccacggtggttcgaatgtatatagaaagaatttctttttttacacttcgtactattag**

**Fortunella_japonica_7** **gcccacggtggttcgaatgtatatagaaagaatttctttttttacacttcgtactattag**

**Fortunella_japonica_8** **gcccacggtggttcgaatgtatatagaaagaatttctttttttacacttcgtactattag**

**Fortunella_japonica_9** **gcccacggtggttcgaatgtatatagaaagaatttctttttttacacttcgtactattag**

**Fortunella_japonica_10** **gcccacggtggttcgaatgtatatagaaagaatttctttttttacacttcgtactattag**

**Fortunella_japonica_11** **gcccacggtggttcgaatgtatatagaaagaatttctttttttacacttcgtactattag**

**Fortunella_japonica_12** **gcccacggtggttcgaatgtatatagaaagaatttctttttttacacttcgtactattag**

**Fortunella_margarita_1** **gcccacggtggttcgaatgtatatagaaagaatttctttttttacacttcgtactattag**

**Fortunella_margarita_2** **gcccacggtggttcgaatgtatatagaaagaatttctttttttacacttcgtactattag**

**Fortunella_venosa_1** **gcccacggtggttcgaatgtatatagaaagaatttctttttttacacttcgtactattag**

**Fortunella_venosa_2** **gcccacggtggttcgaatgtatatagaaagaatttctttttttacacttcgtactattag**

**Fortunella_venosa_3** **gcccacggtggttcgaatgtatatagaaagaatttctttttttacacttcgtactattag**

23170	23180	23190	23200	23210	23220

....|....|....|....|....|....|....|....|....|....|....|....|

**Atalantia_kwangtungens** **ataatgcccataaatctcatgagaggtacccaaagattcatagtgaacttcgatgggagc**

**Citrus_reticulata_1** **ataatgcccataaatctcatgagaggtacccaaagattcatagtgaacttcgatgggagc**

**Citrus_reticulata_2** **ataatgcccataaatctcatgagaggtacccaaagattcatagtgaacttcgatgggagc**

**Citrus_trifoliata_3** **ataatgcccataaatctcatgcgaggtacccaaagattcatagtgaacttcgatgggagc**

**Citrus_aurantiifolia** **ataatgcccataaatctcatgagaggtacccaaagattcatagtgaacttcgatgggagc**

**Citrus_aurantium** **ataatgcccataaatctcatgagaggtacccaaagattcatagtgaacttcgatgggagc**

**Citrus_cavaleriei** **ataatgcccataaatctcatgagaggtacccaaagattcatagtgaacttcgatgggagc**

**Citrus_hongheensis** **ataatgcccataaatctcatgagaggtacccaaagattcatagtgaacttcgatgggagc**

**Citrus_limon** **ataatgcccataaatctcatgagaggtacccaaagattcatagtgaacttcgatgggagc**

**Citrus_maxima** **ataatgcccataaatctcatgagaggtacccaaagattcatagtgaacttcgatgggagc**

**Citrus_medica** **ataatgcccataaatctcatgagaggtacccaaagattcatagtgaacttcgatgggagc**

**Citrus_polytrifolia** **ataatgcccataaatctcatgcgaggtacccaaagattcatagtgaacttcgatgggagc**

**Citrus_reticulata** **ataatgcccataaatctcatgagaggtacccaaagattcatagtgaacttcgatgggagc**

**Citrus_sinensis** **ataatgcccataaatctcatgagaggtacccaaagattcatagtgaacttcgatgggagc**

**Fortunella_bawangica_1** **ataatgcccataaatctcatgagaggtacccaaagattcatagtgaacttcgatgggagc**

**Fortunella_bawangica_2** **ataatgcccataaatctcatgagaggtacccaaagattcatagtgaacttcgatgggagc**

**Fortunella_hindsii_1** **ataatgcccataaatctcatgagaggtacccaaagattcatagtgaacttcgatgggagc**

**Fortunella_hindsii_2** **ataatgcccataaatctcatgagaggtacccaaagattcatagtgaacttcgatgggagc**

**Fortunella_hindsii_3** **ataatgcccataaatctcatgagaggtacccaaagattcatagtgaacttcgatgggagc**

**Fortunella_japonica_1** **ataatgcccataaatctcatgagaggtacccaaagattcatagtgaacttcgatgggagc**

**Fortunella_japonica_2** **ataatgcccataaatctcatgagaggtacccaaagattcatagtgaacttcgatgggagc**

**Fortunella_japonica_3** **ataatgcccataaatctcatgagaggtacccaaagattcatagtgaacttcgatgggagc**

**Fortunella_japonica_4** **ataatgcccataaatctcatgagaggtacccaaagattcatagtgaacttcgatgggagc**

**Fortunella_japonica_5** **ataatgcccataaatctcatgagaggtacccaaagattcatagtgaacttcgatgggagc**

**Fortunella_japonica_6** **ataatgcccataaatctcatgagaggtacccaaagattcatagtgaacttcgatgggagc**

**Fortunella_japonica_7** **ataatgcccataaatctcatgagaggtacccaaagattcatagtgaacttcgatgggagc**

**Fortunella_japonica_8** **ataatgcccataaatctcatgagaggtacccaaagattcatagtgaacttcgatgggagc**

**Fortunella_japonica_9** **ataatgcccataaatctcatgagaggtacccaaagattcatagtgaacttcgatgggagc**

**Fortunella_japonica_10** **ataatgcccataaatctcatgagaggtacccaaagattcatagtgaacttcgatgggagc**

**Fortunella_japonica_11** **ataatgcccataaatctcatgagaggtacccaaagattcatagtgaacttcgatgggagc**

**Fortunella_japonica_12** **ataatgcccataaatctcatgagaggtacccaaagattcatagtgaacttcgatgggagc**

**Fortunella_margarita_1** **ataatgcccataaatctcatgagaggtacccaaagattcatagtgaacttcgatgggagc**

**Fortunella_margarita_2** **ataatgcccataaatctcatgagaggtacccaaagattcatagtgaacttcgatgggagc**

**Fortunella_venosa_1** **ataatgcccataaatctcatgagaggtacccaaagattcatagtgaacttcgatgggagc**

**Fortunella_venosa_2** **ataatgcccataaatctcatgagaggtacccaaagattcatagtgaacttcgatgggagc**

**Fortunella_venosa_3** **ataatgcccataaatctcatgagaggtacccaaagattcatagtgaacttcgatgggagc**

23230	23240	23250	23260	23270	23280

....|....|....|....|....|....|....|....|....|....|....|....|

**Atalantia_kwangtungens** **ttcccttgaagcaataagtcgttgatctaatcgccaccgaagccacaacggactatctaa**

**Citrus_reticulata_1** **ttcccttgaagcaataaggcgttgatctaatcgccaccgaagccacaacggactatctaa**

**Citrus_reticulata_2** **ttcccttgaagcaataaggcgttgatctaatcgccaccgaagccacaacggactatctaa**

**Citrus_trifoliata_3** **ttcccttgaagcaataaggcgttgatctaatcgccaccgaagccacaacggactatctaa**

**Citrus_aurantiifolia** **ttcccttgaagcaataaggcgttgatctaatcgccaccgaagccacaacggactatctaa**

**Citrus_aurantium** **ttcccttgaagcaataaggcgttgatctaatcgccaccgaagccacaacggactatctaa**

**Citrus_cavaleriei** **ttcccttgaagcaataaggcgttgatctaatcgccaccgaagccacaacggactatctaa**

**Citrus_hongheensis** **ttcccttgaagcaataaggcgttgatctaatcgccaccgaagccacaacggactatctaa**

**Citrus_limon** **ttcccttgaagcaataaggcgttgatctaatcgccaccgaagccacaacggactatctaa**

**Citrus_maxima** **ttcccttgaagcaataaggcgttgatctaatcgccaccgaagccacaacggactatctaa**

**Citrus_medica** **ttcccttgaagcaataaggcgttgatctaatcgccaccgaagccacaacggactatctaa**

**Citrus_polytrifolia** **ttcccttgaagcaataaggcgttgatctaatcgccaccgaagccacaacggactatctaa**

**Citrus_reticulata** **ttcccttgaagcaataaggcgttgatctaatcgccaccgaagccacaacggactatctaa**

**Citrus_sinensis** **ttcccttgaagcaataaggcgttgatctaatcgccaccgaagccacaacggactatctaa**

**Fortunella_bawangica_1** **ttcccttgaagcaataaggcgttgatctaatcgccaccgaagccacaacggactatctaa**

**Fortunella_bawangica_2** **ttcccttgaagcaataaggcgttgatctaatcgccaccgaagccacaacggactatctaa**

**Fortunella_hindsii_1** **ttcccttgaagcaataaggcgttgatctaatcgccaccgaagccacaacggactatctaa**

**Fortunella_hindsii_2** **ttcccttgaagcaataaggcgttgatctaatcgccaccgaagccacaacggactatctaa**

**Fortunella_hindsii_3** **ttcccttgaagcaataaggcgttgatctaatcgccaccgaagccacaacggactatctaa**

**Fortunella_japonica_1** **ttcccttgaagcaataaggcgttgatctaatcgccaccgaagccacaacggactatctaa**

**Fortunella_japonica_2** **ttcccttgaagcaataaggcgttgatctaatcgccaccgaagccacaacggactatctaa**

**Fortunella_japonica_3** **ttcccttgaagcaataaggcgttgatctaatcgccaccgaagccacaacggactatctaa**

**Fortunella_japonica_4** **ttcccttgaagcaataaggcgttgatctaatcgccaccgaagccacaacggactatctaa**

**Fortunella_japonica_5** **ttcccttgaagcaataaggcgttgatctaatcgccaccgaagccacaacggactatctaa**

**Fortunella_japonica_6** **ttcccttgaagcaataaggcgttgatctaatcgccaccgaagccacaacggactatctaa**

**Fortunella_japonica_7** **ttcccttgaagcaataaggcgttgatctaatcgccaccgaagccacaacggactatctaa**

**Fortunella_japonica_8** **ttcccttgaagcaataaggcgttgatctaatcgccaccgaagccacaacggactatctaa**

**Fortunella_japonica_9** **ttcccttgaagcaataaggcgttgatctaatcgccaccgaagccacaacggactatctaa**

**Fortunella_japonica_10** **ttcccttgaagcaataaggcgttgatctaatcgccaccgaagccacaacggactatctaa**

**Fortunella_japonica_11** **ttcccttgaagcaataaggcgttgatctaatcgccaccgaagccacaacggactatctaa**

**Fortunella_japonica_12** **ttcccttgaagcaataaggcgttgatctaatcgccaccgaagccacaacggactatctaa**

**Fortunella_margarita_1** **ttcccttgaagcaataaggcgttgatctaatcgccaccgaagccacaacggactatctaa**

**Fortunella_margarita_2** **ttcccttgaagcaataaggcgttgatctaatcgccaccgaagccacaacggactatctaa**

**Fortunella_venosa_1** **ttcccttgaagcaataaggcgttgatctaatcgccaccgaagccacaacggactatctaa**

**Fortunella_venosa_2** **ttcccttgaagcaataaggcgttgatctaatcgccaccgaagccacaacggactatctaa**

**Fortunella_venosa_3** **ttcccttgaagcaataaggcgttgatctaatcgccaccgaagccacaacggactatctaa**

23290	23300	23310	23320	23330	23340

....|....|....|....|....|....|....|....|....|....|....|....|

**Atalantia_kwangtungens** **attgattcttttctgccgataagctccaattgcatcataggaattacaaaaaaggggttc**

**Citrus_reticulata_1** **attgattcttttctgccgataagctccaattgcatcataggaattacaaaaaaggggttc**

**Citrus_reticulata_2** **attgattcttttctgccgataagctccaattgcatcataggaattacaaaaaaggggttc**

**Citrus_trifoliata_3** **attgattcttttctgccgataagctccaattgcatcataggaattacaaaaaaggggttc**

**Citrus_aurantiifolia** **attgattcttttctgccgataagctccaattgcatcataggaattacaaaaaaggggttc**

**Citrus_aurantium** **attgattcttttctgccgataagctccaattgcatcataggaattacaaaaaaggggttc**

**Citrus_cavaleriei** **attgattcttttctgccgataagctccaattgcatcataggaattacaaaaaaggggttc**

**Citrus_hongheensis** **attgattcttttctgccgataagctccaattgcatcataggaattacaaaaaaggggttc**

**Citrus_limon** **attgattcttttctgccgataagctccaattgcatcataggaattacaaaaaaggggttc**

**Citrus_maxima** **attgattcttttctgccgataagctccaattgcatcataggaattacaaaaaaggggttc**

**Citrus_medica** **attgattcttttctgccgataagctccaattgcatcataggaattacaaaaaaggggttc**

**Citrus_polytrifolia** **attgattcttttctgccgataagctccaattgcatcataggaattacaaaaaaggggttc**

**Citrus_reticulata** **attgattcttttctgccgataagctccaattgcatcataggaattacaaaaaaggggttc**

**Citrus_sinensis** **attgattcttttctgccgataagctccaattgcatcataggaattacaaaaaaggggttc**

**Fortunella_bawangica_1** **attgattcttttctgccgataagctccaattgcatcataggaattacaaaaaaggggttc**

**Fortunella_bawangica_2** **attgattcttttctgccgataagctccaattgcatcataggaattacaaaaaaggggttc**

**Fortunella_hindsii_1** **attgattcttttctgccgataagctccaattgcatcataggaattacaaaaaaggggttc**

**Fortunella_hindsii_2** **attgattcttttctgccgataagctccaattgcatcataggaattacaaaaaaggggttc**

**Fortunella_hindsii_3** **attgattcttttctgccgataagctccaattgcatcataggaattacaaaaaaggggttc**

**Fortunella_japonica_1** **attgattcttttctgccgataagctccaattgcatcataggaattacaaaaaaggggttc**

**Fortunella_japonica_2** **attgattcttttctgccgataagctccaattgcatcataggaattacaaaaaaggggttc**

**Fortunella_japonica_3** **attgattcttttctgccgataagctccaattgcatcataggaattacaaaaaaggggttc**

**Fortunella_japonica_4** **attgattcttttctgccgataagctccaattgcatcataggaattacaaaaaaggggttc**

**Fortunella_japonica_5** **attgattcttttctgccgataagctccaattgcatcataggaattacaaaaaaggggttc**

**Fortunella_japonica_6** **attgattcttttctgccgataagctccaattgcatcataggaattacaaaaaaggggttc**

**Fortunella_japonica_7** **attgattcttttctgccgataagctccaattgcatcataggaattacaaaaaaggggttc**

**Fortunella_japonica_8** **attgattcttttctgccgataagctccaattgcatcataggaattacaaaaaaggggttc**

**Fortunella_japonica_9** **attgattcttttctgccgataagctccaattgcatcataggaattacaaaaaaggggttc**

**Fortunella_japonica_10** **attgattcttttctgccgataagctccaattgcatcataggaattacaaaaaaggggttc**

**Fortunella_japonica_11** **attgattcttttctgccgataagctccaattgcatcataggaattacaaaaaaggggttc**

**Fortunella_japonica_12** **attgattcttttctgccgataagctccaattgcatcataggaattacaaaaaaggggttc**

**Fortunella_margarita_1** **attgattcttttctgccgataagctccaattgcatcataggaattacaaaaaaggggttc**

**Fortunella_margarita_2** **attgattcttttctgccgataagctccaattgcatcataggaattacaaaaaaggggttc**

**Fortunella_venosa_1** **attgattcttttctgccgataagctccaattgcatcataggaattacaaaaaaggggttc**

**Fortunella_venosa_2** **attgattcttttctgccgataagctccaattgcatcataggaattacaaaaaaggggttc**

**Fortunella_venosa_3** **attgattcttttctgccgataagctccaattgcatcataggaattacaaaaaaggggttc**

23350	23360	23370	23380	23390	23400

....|....|....|....|....|....|....|....|....|....|....|....|

**Atalantia_kwangtungens** **tttcgtatacttatagctattatcgtcaattctttcatctggataatttcttcgattaca**

**Citrus_reticulata_1** **tttcgtatacttatagctattatcgtcaattctttcatctggataatttcttcgattcca**

**Citrus_reticulata_2** **tttcgtatacttatagctattatcgtcaattctttcatctggataatttcttcgattcca**

**Citrus_trifoliata_3** **tttcgtatacttatagctattatcgtcaattctttcatctggataatttattcgattcca**

**Citrus_aurantiifolia** **tttcgtatacgtatagctattatcgtcaattctttcatctggataatttcttcgattcca**

**Citrus_aurantium** **tttcgtatacgtatagctattatcgtcaattctttcatctggataatttcttcgattcca**

**Citrus_cavaleriei** **tttcgtatacttatagctattatcgtcaattctttcatctggataatttcttcgattcca**

**Citrus_hongheensis** **tttcgtatacgtatagctattatcgtcaattctttcatctggataatttcttcgattcca**

**Citrus_limon** **tttcgtatacgtatagctattatcgtcaattctttcatctggataatttcttcgattcca**

**Citrus_maxima** **tttcgtatacgtatagctattatcgtcaattctttcatctggataatttcttcgattcca**

**Citrus_medica** **tttcgtatacttatagctattatcgtcaattctttcatctggataatttcttcgattcca**

**Citrus_polytrifolia** **tttcgtatacttatagctattatcgtcaattctttcatctggataatttattcgattcca**

**Citrus_reticulata** **tttcgtatacttatagctattatcgtcaattctttcatctggataatttcttcgattcca**

**Citrus_sinensis** **tttcgtatacgtatagctattatcgtcaattctttcatctggataatttcttcgattcca**

**Fortunella_bawangica_1** **tttcgtatacttatagctattatcgtcaattctttcatctggataatttcttcgattcca**

**Fortunella_bawangica_2** **tttcgtatacttatagctattatcgtcaattctttcatctggataatttcttcgattcca**

**Fortunella_hindsii_1** **tttcgtatacttatagctattatcgtcaattctttcatctggataatttcttcgattcca**

**Fortunella_hindsii_2** **tttcgtatacttatagctattatcgtcaattctttcatctggataatttcttcgattcca**

**Fortunella_hindsii_3** **tttcgtatacttatagctattatcgtcaattctttcatctggataatttcttcgattcca**

**Fortunella_japonica_1** **tttcgtatacttatagctattatcgtcaattctttcatctggataatttcttcgattcca**

**Fortunella_japonica_2** **tttcgtatacttatagctattatcgtcaattctttcatctggataatttcttcgattcca**

**Fortunella_japonica_3** **tttcgtatacttatagctattatcgtcaattctttcatctggataatttcttcgattcca**

**Fortunella_japonica_4** **tttcgtatacttatagctattatcgtcaattctttcatctggataatttcttcgattcca**

**Fortunella_japonica_5** **tttcgtatacttatagctattatcgtcaattctttcatctggataatttcttcgattcca**

**Fortunella_japonica_6** **tttcgtatacttatagctattatcgtcaattctttcatctggataatttcttcgattcca**

**Fortunella_japonica_7** **tttcgtatacttatagctattatcgtcaattctttcatctggataatttcttcgattcca**

**Fortunella_japonica_8** **tttcgtatacttatagctattatcgtcaattctttcatctggataatttcttcgattcca**

**Fortunella_japonica_9** **tttcgtatacttatagctattatcgtcaattctttcatctggataatttcttcgattcca**

**Fortunella_japonica_10** **tttcgtatacttatagctattatcgtcaattctttcatctggataatttcttcgattcca**

**Fortunella_japonica_11** **tttcgtatacttatagctattatcgtcaattctttcatctggataatttcttcgattcca**

**Fortunella_japonica_12** **tttcgtatacttatagctattatcgtcaattctttcatctggataatttcttcgattcca**

**Fortunella_margarita_1** **tttcgtatacttatagctattatcgtcaattctttcatctggataatttcttcgattcca**

**Fortunella_margarita_2** **tttcgtatacttatagctattatcgtcaattctttcatctggataatttcttcgattcca**

**Fortunella_venosa_1** **tttcgtatacttatagctattatcgtcaattctttcatctggataatttcttcgattcca**

**Fortunella_venosa_2** **tttcgtatacttatagctattatcgtcaattctttcatctggataatttcttcgattcca**

**Fortunella_venosa_3** **tttcgtatacttatagctattatcgtcaattctttcatctggataatttcttcgattcca**

23410	23420	23430	23440	23450	23460

....|....|....|....|....|....|....|....|....|....|....|....|

**Atalantia_kwangtungens** **tgtattatacctatttgcacaaatacctcgacgattcccgctcgttaatacatagagccc**

**Citrus_reticulata_1** **tgtattatacctatttgcacaaatacctcgacgattcccgctcgttaatacatagagccc**

**Citrus_reticulata_2** **tgtattatacctatttgcacaaatacctcgacgattcccgctcgttaatacatagagccc**

**Citrus_trifoliata_3** **tgtattatacctatttgcacaaatacctcgacgattcccgctcgttaatacatagagccc**

**Citrus_aurantiifolia** **tgtattatacctatttgcacaaatacctcgacgattcccgctcgttaatacatagagccc**

**Citrus_aurantium** **tgtattatacctatttgcacaaatacctcgacgattcccgctcgttaatacatagagccc**

**Citrus_cavaleriei** **tgtattatacctatttgcacaaatacctcgacgattcccgctcgttaatacatagagccc**

**Citrus_hongheensis** **tgtattatacctatttgcacaaatacctcgacgattcccgctcgttaatacatagagccc**

**Citrus_limon** **tgtattatacctatttgcacaaatacctcgacgattcccgctcgttaatacatagagccc**

**Citrus_maxima** **tgtattatacctatttgcacaaatacctcgacgattcccgctcgttaatacatagagccc**

**Citrus_medica** **tgtattatacctatttgcacaaatacctcgacgattcccgctcgttaatacatagagccc**

**Citrus_polytrifolia** **tgtattatacctatttgcacaaatacctcgacgattcccgctcgttaatacatagagccc**

**Citrus_reticulata** **tgtattatacctatttgcacaaatacctcgacgattcccgctcgttaatacatagagccc**

**Citrus_sinensis** **tgtattatacctatttgcacaaatacctcgacgattcccgctcgttaatacatagagccc**

**Fortunella_bawangica_1** **tgtattatacctatttgcacaaatacctcgacgattcccgctcgttaatacatagagccc**

**Fortunella_bawangica_2** **tgtattatacctatttgcacaaatacctcgacgattcccgctcgttaatacatagagccc**

**Fortunella_hindsii_1** **tgtattatacctatttgcacaaatacctcgacgattcccgctcgttaatacatagagccc**

**Fortunella_hindsii_2** **tgtattatacctatttgcacaaatacctcgacgattcccgctcgttaatacatagagccc**

**Fortunella_hindsii_3** **tgtattatacctatttgcacaaatacctcgacgattcccgctcgttaatacatagagccc**

**Fortunella_japonica_1** **tgtattatacctatttgcacaaatacctcgacgattcccgctcgttaatacatagagccc**

**Fortunella_japonica_2** **tgtattatacctatttgcacaaatacctcgacgattcccgctcgttaatacatagagccc**

**Fortunella_japonica_3** **tgtattatacctatttgcacaaatacctcgacgattcccgctcgttaatacatagagccc**

**Fortunella_japonica_4** **tgtattatacctatttgcacaaatacctcgacgattcccgctcgttaatacatagagccc**

**Fortunella_japonica_5** **tgtattatacctatttgcacaaatacctcgacgattcccgctcgttaatacatagagccc**

**Fortunella_japonica_6** **tgtattatacctatttgcacaaatacctcgacgattcccgctcgttaatacatagagccc**

**Fortunella_japonica_7** **tgtattatacctatttgcacaaatacctcgacgattcccgctcgttaatacatagagccc**

**Fortunella_japonica_8** **tgtattatacctatttgcacaaatacctcgacgattcccgctcgttaatacatagagccc**

**Fortunella_japonica_9** **tgtattatacctatttgcacaaatacctcgacgattcccgctcgttaatacatagagccc**

**Fortunella_japonica_10** **tgtattatacctatttgcacaaatacctcgacgattcccgctcgttaatacatagagccc**

**Fortunella_japonica_11** **tgtattatacctatttgcacaaatacctcgacgattcccgctcgttaatacatagagccc**

**Fortunella_japonica_12** **tgtattatacctatttgcacaaatacctcgacgattcccgctcgttaatacatagagccc**

**Fortunella_margarita_1** **tgtattatacctatttgcacaaatacctcgacgattcccgctcgttaatacatagagccc**

**Fortunella_margarita_2** **tgtattatacctatttgcacaaatacctcgacgattcccgctcgttaatacatagagccc**

**Fortunella_venosa_1** **tgtattatacctatttgcacaaatacctcgacgattcccgctcgttaatacatagagccc**

**Fortunella_venosa_2** **tgtattatacctatttgcacaaatacctcgacgattcccgctcgttaatacatagagccc**

**Fortunella_venosa_3** **tgtattatacctatttgcacaaatacctcgacgattcccgctcgttaatacatagagccc**

23470	23480	23490	23500	23510	23520

....|....|....|....|....|....|....|....|....|....|....|....|

**Atalantia_kwangtungens** **aataagcatatcttgagtcggtacggaaatgggatctccaatagtaggagacaagagatt**

**Citrus_reticulata_1** **aataagcatatcttgagtcggtatggaaatgggatctccaatagtaggagacaagagatt**

**Citrus_reticulata_2** **aataagcatatcttgagtcggtatggaaatgggatctccaatagtaggagacaagagatt**

**Citrus_trifoliata_3** **aataagcatatcttgagtcggtatggaaatgggatctccaatagtaggagacaagagatt**

**Citrus_aurantiifolia** **aataagcatatcttgagtcggtatggaaatgggatctccaatagtaggagacaagagatt**

**Citrus_aurantium** **aataagcatatcttgagtcggtatggaaatgggatctccaatagtaggagacaagagatt**

**Citrus_cavaleriei** **aataagcatatcttgagtcggtatggaaatgggatctccaatagtaggagacaagagatt**

**Citrus_hongheensis** **aataagcatatcttgagtcggtatggaaatgggatctccaatagtaggagacaagagatt**

**Citrus_limon** **aataagcatatcttgagtcggtatggaaatgggatctccaatagtaggagacaagagatt**

**Citrus_maxima** **aataagcatatcttgagtcggtatggaaatgggatctccaatagtaggagacaagagatt**

**Citrus_medica** **aataagcatatcttgagtcggtatggaaatgggatctccaatagtaggagacaagagatt**

**Citrus_polytrifolia** **aataagcatatcttgagtcggtatggaaatgggatctccaatagtaggagacaagagatt**

**Citrus_reticulata** **aataagcatatcttgagtcggtatggaaatgggatctccaatagtaggagacaagagatt**

**Citrus_sinensis** **aataagcatatcttgagtcggtatggaaatgggatctccaatagtaggagacaagagatt**

**Fortunella_bawangica_1** **aataagcatatcttgagtcggtatggaaatgggatctccaatagtaggagacaagagatt**

**Fortunella_bawangica_2** **aataagcatatcttgagtcggtatggaaatgggatctccaatagtaggagacaagagatt**

**Fortunella_hindsii_1** **aataagcatatcttgagtcggtatggaaatgggatctccaatagtaggagacaagagatt**

**Fortunella_hindsii_2** **aataagcatatcttgagtcggtatggaaatgggatctccaatagtaggagacaagagatt**

**Fortunella_hindsii_3** **aataagcatatcttgagtcggtatggaaatgggatctccaatagtaggagacaagagatt**

**Fortunella_japonica_1** **aataagcatatcttgagtcggtatggaaatgggatctccaatagtaggagacaagagatt**

**Fortunella_japonica_2** **aataagcatatcttgagtcggtatggaaatgggatctccaatagtaggagacaagagatt**

**Fortunella_japonica_3** **aataagcatatcttgagtcggtatggaaatgggatctccaatagtaggagacaagagatt**

**Fortunella_japonica_4** **aataagcatatcttgagtcggtatggaaatgggatctccaatagtaggagacaagagatt**

**Fortunella_japonica_5** **aataagcatatcttgagtcggtatggaaatgggatctccaatagtaggagacaagagatt**

**Fortunella_japonica_6** **aataagcatatcttgagtcggtatggaaatgggatctccaatagtaggagacaagagatt**

**Fortunella_japonica_7** **aataagcatatcttgagtcggtatggaaatgggatctccaatagtaggagacaagagatt**

**Fortunella_japonica_8** **aataagcatatcttgagtcggtatggaaatgggatctccaatagtaggagacaagagatt**

**Fortunella_japonica_9** **aataagcatatcttgagtcggtatggaaatgggatctccaatagtaggagacaagagatt**

**Fortunella_japonica_10** **aataagcatatcttgagtcggtatggaaatgggatctccaatagtaggagacaagagatt**

**Fortunella_japonica_11** **aataagcatatcttgagtcggtatggaaatgggatctccaatagtaggagacaagagatt**

**Fortunella_japonica_12** **aataagcatatcttgagtcggtatggaaatgggatctccaatagtaggagacaagagatt**

**Fortunella_margarita_1** **aataagcatatcttgagtcggtatggaaatgggatctccaatagtaggagacaagagatt**

**Fortunella_margarita_2** **aataagcatatcttgagtcggtatggaaatgggatctccaatagtaggagacaagagatt**

**Fortunella_venosa_1** **aataagcatatcttgagtcggtatggaaatgggatctccaatagtaggagacaagagatt**

**Fortunella_venosa_2** **aataagcatatcttgagtcggtatggaaatgggatctccaatagtaggagacaagagatt**

**Fortunella_venosa_3** **aataagcatatcttgagtcggtatggaaatgggatctccaatagtaggagacaagagatt**

23530	23540	23550	23560	23570	23580

....|....|....|....|....|....|....|....|....|....|....|....|

**Atalantia_kwangtungens** **catatgagaaaacataagtaaacgagcctccgcttgagcctctaaagataagggtacatg**

**Citrus_reticulata_1** **catatgagaaaacataagtaaacgagcctctgcttgagcctctaaagataagggtacatg**

**Citrus_reticulata_2** **catatgagaaaacataagtaaacgagcctctgcttgagcctctaaagataagggtacatg**

**Citrus_trifoliata_3** **catatgagaaaacataagtaaacgagcctctgcttgagcctctaaagataagggtacatg**

**Citrus_aurantiifolia** **catatgagaaaacataagtaaacgagcctctgcttgagcctctaaagataagggtacatg**

**Citrus_aurantium** **catatgagaaaacataagtaaccgagcctctgcttgagcctctaaagataagggtacatg**

**Citrus_cavaleriei** **catatgagaaaacataagtaaacgagcctctgcttgagcctctaaagataagggtacatg**

**Citrus_hongheensis** **catatgagaaaacataagtaaccgagcctctgcttgagcctctaaagataagggtacatg**

**Citrus_limon** **catatgagaaaacataagtaaccgagcctctgcttgagcctctaaagataagggtacatg**

**Citrus_maxima** **catatgagaaaacataagtaaccgagcctctgcttgagcctctaaagataagggtacatg**

**Citrus_medica** **catatgagaaaacataagtaaacgagcctctgcttgagcctctaaagataagggtacatg**

**Citrus_polytrifolia** **catatgagaaaacataagtaaacgagcctctgcttgagcctctaaagataagggtacatg**

**Citrus_reticulata** **catatgagaaaacataagtaaacgagcctctgcttgagcctctaaagataagggtacatg**

**Citrus_sinensis** **catatgagaaaacataagtaaccgagcctctgcttgagcctctaaagataagggtacatg**

**Fortunella_bawangica_1** **catatgagaaaacataagtaaacgagcctctgcttgagcctctaaagataagggtacatg**

**Fortunella_bawangica_2** **catatgagaaaacataagtaaacgagcctctgcttgagcctctaaagataagggtacatg**

**Fortunella_hindsii_1** **catatgagaaaacataagtaaacgagcctctgcttgagcctctaaagataagggtacatg**

**Fortunella_hindsii_2** **catatgagaaaacataagtaaacgagcctctgcttgagcctctaaagataagggtacatg**

**Fortunella_hindsii_3** **catatgagaaaacataagtaaacgagcctctgcttgagcctctaaagataagggtacatg**

**Fortunella_japonica_1** **catatgagaaaacataagtaaacgagcctctgcttgagcctctaaagataagggtacatg**

**Fortunella_japonica_2** **catatgagaaaacataagtaaacgagcctctgcttgagcctctaaagataagggtacatg**

**Fortunella_japonica_3** **catatgagaaaacataagtaaacgagcctctgcttgagcctctaaagataagggtacatg**

**Fortunella_japonica_4** **catatgagaaaacataagtaaacgagcctctgcttgagcctctaaagataagggtacatg**

**Fortunella_japonica_5** **catatgagaaaacataagtaaacgagcctctgcttgagcctctaaagataagggtacatg**

**Fortunella_japonica_6** **catatgagaaaacataagtaaacgagcctctgcttgagcctctaaagataagggtacatg**

**Fortunella_japonica_7** **catatgagaaaacataagtaaacgagcctctgcttgagcctctaaagataagggtacatg**

**Fortunella_japonica_8** **catatgagaaaacataagtaaacgagcctctgcttgagcctctaaagataagggtacatg**

**Fortunella_japonica_9** **catatgagaaaacataagtaaacgagcctctgcttgagcctctaaagataagggtacatg**

**Fortunella_japonica_10** **catatgagaaaacataagtaaacgagcctctgcttgagcctctaaagataagggtacatg**

**Fortunella_japonica_11** **catatgagaaaacataagtaaacgagcctctgcttgagcctctaaagataagggtacatg**

**Fortunella_japonica_12** **catatgagaaaacataagtaaacgagcctctgcttgagcctctaaagataagggtacatg**

**Fortunella_margarita_1** **catatgagaaaacataagtaaacgagcctctgcttgagcctctaaagataagggtacatg**

**Fortunella_margarita_2** **catatgagaaaacataagtaaacgagcctctgcttgagcctctaaagataagggtacatg**

**Fortunella_venosa_1** **catatgagaaaacataagtaaacgagcctctgcttgagcctctaaagataagggtacatg**

**Fortunella_venosa_2** **catatgagaaaacataagtaaacgagcctctgcttgagcctctaaagataagggtacatg**

**Fortunella_venosa_3** **catatgagaaaacataagtaaacgagcctctgcttgagcctctaaagataagggtacatg**

23590	23600	23610	23620	23630	23640

....|....|....|....|....|....|....|....|....|....|....|....|

**Atalantia_kwangtungens** **aacagccatttgatccccatcaaagtctgcgttgaatcccttacaaactaatggatgtaa**

**Citrus_reticulata_1** **aacagccatttgatccccatcaaagtctgcgttgaatcccttacaaactaatggatgtaa**

**Citrus_reticulata_2** **aacagccatttgatccccatcaaagtctgcgttgaatcccttacaaactaatggatgtaa**

**Citrus_trifoliata_3** **aacagccatttgatccccatcaaagtctgcgttgaatcccttacaaactaatggatgtaa**

**Citrus_aurantiifolia** **aacagccatttgatccccatcaaagtctgcgttgaatcccttacaaactaatggatgtaa**

**Citrus_aurantium** **aacagccatttgatccccatcaaagtctgcgttgaatcccttacaaactaatggatgtaa**

**Citrus_cavaleriei** **aacagccatttgatccccatcaaagtctgcgttgaatcccttacaaactaatggatgtaa**

**Citrus_hongheensis** **aacagccatttgatccccatcaaagtctgcgttgaatcccttacaaactaatggatgtaa**

**Citrus_limon** **aacagccatttgatccccatcaaagtctgcgttgaatcccttacaaactaatggatgtaa**

**Citrus_maxima** **aacagccatttgatccccatcaaagtctgcgttgaatcccttacaaactaatggatgtaa**

**Citrus_medica** **aacagccatttgatccccatcaaagtctgcgttgaatcccttacaaactaatggatgtaa**

**Citrus_polytrifolia** **aacagccatttgatccccatcaaagtctgcgttgaatcccttacaaactaatggatgtaa**

**Citrus_reticulata** **aacagccatttgatccccatcaaagtctgcgttgaatcccttacaaactaatggatgtaa**

**Citrus_sinensis** **aacagccatttgatccccatcaaagtctgcgttgaatcccttacaaactaatggatgtaa**

**Fortunella_bawangica_1** **aacagccatttgatccccatcaaagtctgcgttgaatcccttacaaactaatggatgtaa**

**Fortunella_bawangica_2** **aacagccatttgatccccatcaaagtctgcgttgaatcccttacaaactaatggatgtaa**

**Fortunella_hindsii_1** **aacagccatttgatccccatcaaagtctgcgttgaatcccttacaaactaatggatgtaa**

**Fortunella_hindsii_2** **aacagccatttgatccccatcaaagtctgcgttgaatcccttacaaactaatggatgtaa**

**Fortunella_hindsii_3** **aacagccatttgatccccatcaaagtctgcgttgaatcccttacaaactaatggatgtaa**

**Fortunella_japonica_1** **aacagccatttgatccccatcaaagtctgcgttgaatcccttacaaactaatggatgtaa**

**Fortunella_japonica_2** **aacagccatttgatccccatcaaagtctgcgttgaatcccttacaaactaatggatgtaa**

**Fortunella_japonica_3** **aacagccatttgatccccatcaaagtctgcgttgaatcccttacaaactaatggatgtaa**

**Fortunella_japonica_4** **aacagccatttgatccccatcaaagtctgcgttgaatcccttacaaactaatggatgtaa**

**Fortunella_japonica_5** **aacagccatttgatccccatcaaagtctgcgttgaatcccttacaaactaatggatgtaa**

**Fortunella_japonica_6** **aacagccatttgatccccatcaaagtctgcgttgaatcccttacaaactaatggatgtaa**

**Fortunella_japonica_7** **aacagccatttgatccccatcaaagtctgcgttgaatcccttacaaactaatggatgtaa**

**Fortunella_japonica_8** **aacagccatttgatccccatcaaagtctgcgttgaatcccttacaaactaatggatgtaa**

**Fortunella_japonica_9** **aacagccatttgatccccatcaaagtctgcgttgaatcccttacaaactaatggatgtaa**

**Fortunella_japonica_10** **aacagccatttgatccccatcaaagtctgcgttgaatcccttacaaactaatggatgtaa**

**Fortunella_japonica_11** **aacagccatttgatccccatcaaagtctgcgttgaatcccttacaaactaatggatgtaa**

**Fortunella_japonica_12** **aacagccatttgatccccatcaaagtctgcgttgaatcccttacaaactaatggatgtaa**

**Fortunella_margarita_1** **aacagccatttgatccccatcaaagtctgcgttgaatcccttacaaactaatggatgtaa**

**Fortunella_margarita_2** **aacagccatttgatccccatcaaagtctgcgttgaatcccttacaaactaatggatgtaa**

**Fortunella_venosa_1** **aacagccatttgatccccatcaaagtctgcgttgaatcccttacaaactaatggatgtaa**

**Fortunella_venosa_2** **aacagccatttgatccccatcaaagtctgcgttgaatcccttacaaactaatggatgtaa**

**Fortunella_venosa_3** **aacagccatttgatccccatcaaagtctgcgttgaatcccttacaaactaatggatgtaa**

23650	23660	23670	23680	23690	23700

....|....|....|....|....|....|....|....|....|....|....|....|

**Atalantia_kwangtungens** **acaaatagcgcgcccttccactaaaatgggctggaatgcctgtacgcccaatctatgcag**

**Citrus_reticulata_1** **acaaatagcgcgcccttccactaaaatgggctggaatgcctgtacgcccaatctatgcag**

**Citrus_reticulata_2** **acaaatagcgcgcccttccactaaaatgggctggaatgcctgtacgcccaatctatgcag**

**Citrus_trifoliata_3** **acaaatagcgcgcccttccactaaaatgggctggaatgcctgtacgcccaatctatgcag**

**Citrus_aurantiifolia** **acaaatagcgcgcccttccactaaaatgggctggaatgcctgtacgcccaatctatgcag**

**Citrus_aurantium** **acaaatagcgcgcccttccactaaaatgggctggaatgcctgtacgcccaatctatgcag**

**Citrus_cavaleriei** **acaaatagcgcgcccttccactaaaatgggctggaatgcctgtacgcccaatctatgcag**

**Citrus_hongheensis** **acaaatagcgcgcccttccactaaaatgggctggaatgcctgtacgcccaatctatgcag**

**Citrus_limon** **acaaatagcgcgcccttccactaaaatgggctggaatgcctgtacgcccaatctatgcag**

**Citrus_maxima** **acaaatagcgcgcccttccactaaaatgggctggaatgcctgtacgcccaatctatgcag**

**Citrus_medica** **acaaatagcgcgcccttccactaaaatgggctggaatgcctgtacgcccaatctatgcag**

**Citrus_polytrifolia** **acaaatagcgcgcccttccactaaaatgggctggaatgcctgtacgcccaatctatgcag**

**Citrus_reticulata** **acaaatagcgcgcccttccactaaaatgggctggaatgcctgtacgcccaatctatgcag**

**Citrus_sinensis** **acaaatagcgcgcccttccactaaaatgggctggaatgcctgtacgcccaatctatgcag**

**Fortunella_bawangica_1** **acaaatagcgcgcccttccactaaaatgggctggaatgcctgtacgcccaatctatgcag**

**Fortunella_bawangica_2** **acaaatagcgcgcccttccactaaaatgggctggaatgcctgtacgcccaatctatgcag**

**Fortunella_hindsii_1** **acaaatagcgcgcccttccactaaaatgggctggaatgcctgtacgcccaatctatgcag**

**Fortunella_hindsii_2** **acaaatagcgcgcccttccactaaaatgggctggaatgcctgtacgcccaatctatgcag**

**Fortunella_hindsii_3** **acaaatagcgcgcccttccactaaaatgggctggaatgcctgtacgcccaatctatgcag**

**Fortunella_japonica_1** **acaaatagcgcgcccttccactaaaatgggctggaatgcctgtacgcccaatctatgcag**

**Fortunella_japonica_2** **acaaatagcgcgcccttccactaaaatgggctggaatgcctgtacgcccaatctatgcag**

**Fortunella_japonica_3** **acaaatagcgcgcccttccactaaaatgggctggaatgcctgtacgcccaatctatgcag**

**Fortunella_japonica_4** **acaaatagcgcgcccttccactaaaatgggctggaatgcctgtacgcccaatctatgcag**

**Fortunella_japonica_5** **acaaatagcgcgcccttccactaaaatgggctggaatgcctgtacgcccaatctatgcag**

**Fortunella_japonica_6** **acaaatagcgcgcccttccactaaaatgggctggaatgcctgtacgcccaatctatgcag**

**Fortunella_japonica_7** **acaaatagcgcgcccttccactaaaatgggctggaatgcctgtacgcccaatctatgcag**

**Fortunella_japonica_8** **acaaatagcgcgcccttccactaaaatgggctggaatgcctgtacgcccaatctatgcag**

**Fortunella_japonica_9** **acaaatagcgcgcccttccactaaaatgggctggaatgcctgtacgcccaatctatgcag**

**Fortunella_japonica_10** **acaaatagcgcgcccttccactaaaatgggctggaatgcctgtacgcccaatctatgcag**

**Fortunella_japonica_11** **acaaatagcgcgcccttccactaaaatgggctggaatgcctgtacgcccaatctatgcag**

**Fortunella_japonica_12** **acaaatagcgcgcccttccactaaaatgggctggaatgcctgtacgcccaatctatgcag**

**Fortunella_margarita_1** **acaaatagcgcgcccttccactaaaatgggctggaatgcctgtacgcccaatctatgcag**

**Fortunella_margarita_2** **acaaatagcgcgcccttccactaaaatgggctggaatgcctgtacgcccaatctatgcag**

**Fortunella_venosa_1** **acaaatagcgcgcccttccactaaaatgggctggaatgcctgtacgcccaatctatgcag**

**Fortunella_venosa_2** **acaaatagcgcgcccttccactaaaatgggctggaatgcctgtacgcccaatctatgcag**

**Fortunella_venosa_3** **acaaatagcgcgcccttccactaaaatgggctggaatgcctgtacgcccaatctatgcag**

23710	23720	23730	23740	23750	23760

....|....|....|....|....|....|....|....|....|....|....|....|

**Atalantia_kwangtungens** **agtaggcgctctattcagcaatactggatgtccctgcataacttcctgcagtatttccca**

**Citrus_reticulata_1** **agtaggcgctctattcagcaatactggatgtccctgcataacttcctgcagtatttccca**

**Citrus_reticulata_2** **agtaggcgctctattcagcaatactggatgtccctgcataacttcctgcagtatttccca**

**Citrus_trifoliata_3** **agtaggcgctctattcagcaatactggatgtccctgcataacttcctgcagtatttccca**

**Citrus_aurantiifolia** **agtaggcgctctattcagcaatactggatgtccctgcataacttcctgcagtatttccca**

**Citrus_aurantium** **agtaggcgctctattcagcaatactggatgtccctgcataacttcctgcagtatttccca**

**Citrus_cavaleriei** **agtaggcgctctattcagcaatactggatgtccctgcataacttcctgcagtatttccca**

**Citrus_hongheensis** **agtaggcgctctattcagcaatactggatgtccctgcataacttcctgcagtatttccca**

**Citrus_limon** **agtaggcgctctattcagcaatactggatgtccctgcataacttcctgcagtatttccca**

**Citrus_maxima** **agtaggcgctctattcagcaatactggatgtccctgcataacttcctgcagtatttccca**

**Citrus_medica** **agtaggcgctctattcagcaatactggatgtccctgcataacttcctgcagtatttccca**

**Citrus_polytrifolia** **agtaggcgctctattcagcaatactggatgtccctgcataacttcctgcagtatttccca**

**Citrus_reticulata** **agtaggcgctctattcagcaatactggatgtccctgcataacttcctgcagtatttccca**

**Citrus_sinensis** **agtaggcgctctattcagcaatactggatgtccctgcataacttcctgcagtatttccca**

**Fortunella_bawangica_1** **agtaggcgctctattcagcaatactggatgtccctgcataacttcctgcagtatttccca**

**Fortunella_bawangica_2** **agtaggcgctctattcagcaatactggatgtccctgcataacttcctgcagtatttccca**

**Fortunella_hindsii_1** **agtaggcgctctattcagcaatactggatgtccctgcataacttcctgcagtatttccca**

**Fortunella_hindsii_2** **agtaggcgctctattcagcaatactggatgtccctgcataacttcctgcagtatttccca**

**Fortunella_hindsii_3** **agtaggcgctctattcagcaatactggatgtccctgcataacttcctgcagtatttccca**

**Fortunella_japonica_1** **agtaggcgctctattcagcaatactggatgtccctgcataacttcctgcagtatttccca**

**Fortunella_japonica_2** **agtaggcgctctattcagcaatactggatgtccctgcataacttcctgcagtatttccca**

**Fortunella_japonica_3** **agtaggcgctctattcagcaatactggatgtccctgcataacttcctgcagtatttccca**

**Fortunella_japonica_4** **agtaggcgctctattcagcaatactggatgtccctgcataacttcctgcagtatttccca**

**Fortunella_japonica_5** **agtaggcgctctattcagcaatactggatgtccctgcataacttcctgcagtatttccca**

**Fortunella_japonica_6** **agtaggcgctctattcagcaatactggatgtccctgcataacttcctgcagtatttccca**

**Fortunella_japonica_7** **agtaggcgctctattcagcaatactggatgtccctgcataacttcctgcagtatttccca**

**Fortunella_japonica_8** **agtaggcgctctattcagcaatactggatgtccctgcataacttcctgcagtatttccca**

**Fortunella_japonica_9** **agtaggcgctctattcagcaatactggatgtccctgcataacttcctgcagtatttccca**

**Fortunella_japonica_10** **agtaggcgctctattcagcaatactggatgtccctgcataacttcctgcagtatttccca**

**Fortunella_japonica_11** **agtaggcgctctattcagcaatactggatgtccctgcataacttcctgcagtatttccca**

**Fortunella_japonica_12** **agtaggcgctctattcagcaatactggatgtccctgcataacttcctgcagtatttccca**

**Fortunella_margarita_1** **agtaggcgctctattcagcaatactggatgtccctgcataacttcctgcagtatttccca**

**Fortunella_margarita_2** **agtaggcgctctattcagcaatactggatgtccctgcataacttcctgcagtatttccca**

**Fortunella_venosa_1** **agtaggcgctctattcagcaatactggatgtccctgcataacttcctgcagtatttccca**

**Fortunella_venosa_2** **agtaggcgctctattcagcaatactggatgtccctgcataacttcctgcagtatttccca**

**Fortunella_venosa_3** **agtaggcgctctattcagcaatactggatgtccctgcataacttcctgcagtatttccca**

23770	23780	23790	23800	23810	23820

....|....|....|....|....|....|....|....|....|....|....|....|

**Atalantia_kwangtungens** **gataatcggccctttttcccgaattttactcttagcaactcctatgttcgaagcaaggtg**

**Citrus_reticulata_1** **gataatcggccctttttcccgaattttactcttagcaactcctatgttcgaagcaaggtg**

**Citrus_reticulata_2** **gataatcggccctttttcccgaattttactcttagcaactcctatgttcgaagcaaggtg**

**Citrus_trifoliata_3** **gataatcggccctttttcccgaattttactcttagcaactcctatgttcgaagcaaggtg**

**Citrus_aurantiifolia** **gataatcggccctttttcccgaattttactcttagcaactcctatgttcgaagcaaggtg**

**Citrus_aurantium** **gataatcggccctttttcccgaattttactcttagcaactcctatgttcgaagcaaggtg**

**Citrus_cavaleriei** **gataatcggccctttttcccgaattttactcttagcaactcctatgttcgaagcaaggtg**

**Citrus_hongheensis** **gataatcggccctttttcccgaattttactcttagcaactcctatgttcgaagcaaggtg**

**Citrus_limon** **gataatcggccctttttcccgaattttactcttagcaactcctatgttcgaagcaaggtg**

**Citrus_maxima** **gataatcggccctttttcccgaattttactcttagcaactcctatgttcgaagcaaggtg**

**Citrus_medica** **gataatcggccctttttcccgaattttactcttagcaactcctatgttcgaagcaaggtg**

**Citrus_polytrifolia** **gataatcggccctttttcccgaattttactcttagcaactcctatgttcgaagcaaggtg**

**Citrus_reticulata** **gataatcggccctttttcccgaattttactcttagcaactcctatgttcgaagcaaggtg**

**Citrus_sinensis** **gataatcggccctttttcccgaattttactcttagcaactcctatgttcgaagcaaggtg**

**Fortunella_bawangica_1** **gataatcggccctttttcccgaattttactcttagcaactcctatgttcgaagcaaggtg**

**Fortunella_bawangica_2** **gataatcggccctttttcccgaattttactcttagcaactcctatgttcgaagcaaggtg**

**Fortunella_hindsii_1** **gataatcggccctttttcccgaattttactcttagcaactcctatgttcgaagcaaggtg**

**Fortunella_hindsii_2** **gataatcggccctttttcccgaattttactcttagcaactcctatgttcgaagcaaggtg**

**Fortunella_hindsii_3** **gataatcggccctttttcccgaattttactcttagcaactcctatgttcgaagcaaggtg**

**Fortunella_japonica_1** **gataatcggccctttttcccgaattttactcttagcaactcctatgttcgaagcaaggtg**

**Fortunella_japonica_2** **gataatcggccctttttcccgaattttactcttagcaactcctatgttcgaagcaaggtg**

**Fortunella_japonica_3** **gataatcggccctttttcccgaattttactcttagcaactcctatgttcgaagcaaggtg**

**Fortunella_japonica_4** **gataatcggccctttttcccgaattttactcttagcaactcctatgttcgaagcaaggtg**

**Fortunella_japonica_5** **gataatcggccctttttcccgaattttactcttagcaactcctatgttcgaagcaaggtg**

**Fortunella_japonica_6** **gataatcggccctttttcccgaattttactcttagcaactcctatgttcgaagcaaggtg**

**Fortunella_japonica_7** **gataatcggccctttttcccgaattttactcttagcaactcctatgttcgaagcaaggtg**

**Fortunella_japonica_8** **gataatcggccctttttcccgaattttactcttagcaactcctatgttcgaagcaaggtg**

**Fortunella_japonica_9** **gataatcggccctttttcccgaattttactcttagcaactcctatgttcgaagcaaggtg**

**Fortunella_japonica_10** **gataatcggccctttttcccgaattttactcttagcaactcctatgttcgaagcaaggtg**

**Fortunella_japonica_11** **gataatcggccctttttcccgaattttactcttagcaactcctatgttcgaagcaaggtg**

**Fortunella_japonica_12** **gataatcggccctttttcccgaattttactcttagcaactcctatgttcgaagcaaggtg**

**Fortunella_margarita_1** **gataatcggccctttttcccgaattttactcttagcaactcctatgttcgaagcaaggtg**

**Fortunella_margarita_2** **gataatcggccctttttcccgaattttactcttagcaactcctatgttcgaagcaaggtg**

**Fortunella_venosa_1** **gataatcggccctttttcccgaattttactcttagcaactcctatgttcgaagcaaggtg**

**Fortunella_venosa_2** **gataatcggccctttttcccgaattttactcttagcaactcctatgttcgaagcaaggtg**

**Fortunella_venosa_3** **gataatcggccctttttcccgaattttactcttagcaactcctatgttcgaagcaaggtg**

23830	23840	23850	23860	23870	23880

....|....|....|....|....|....|....|....|....|....|....|....|

**Atalantia_kwangtungens** **ttgtctaattagcccacaaattacaaaagtctggaaaagctctattgcgatttcgcgagg**

**Citrus_reticulata_1** **ttgtctaattagcccacaaattacaaaagtctggaaaagctctattgcgatttcgcgagg**

**Citrus_reticulata_2** **ttgtctaattagcccacaaattacaaaagtctggaaaagctctattgcgatttcgcgagg**

**Citrus_trifoliata_3** **ttgtctaattagcccacaaattacaaaagtctggaaaagctctattgcgatttcgcgagg**

**Citrus_aurantiifolia** **ttgtctaattagcccacaaattacaaaagtctggaaaagctctagtgcgatttcgcgagg**

**Citrus_aurantium** **ttgtctaattagcccacaaattacaaaagtctggaaaagctctattgcgatttcgcgagg**

**Citrus_cavaleriei** **ttgtctaattagcccacaaattacaaaagtctggaaaagctctattgcaatttcgcgagg**

**Citrus_hongheensis** **ttgtctaattagcccacaaattacaaaagtctggaaaagctctattgcgatttcgcgagg**

**Citrus_limon** **ttgtctaattagcccacaaattacaaaagtctggaaaagctctattgcgatttcgcgagg**

**Citrus_maxima** **ttgtctaattagcccacaaattacaaaagtctggaaaagctctattgcgatttcgcgagg**

**Citrus_medica** **ttgtctaattagcccacaaattacaaaagtctggaaaagctctattgcgatttcgcgagg**

**Citrus_polytrifolia** **ttgtctaattagcccacaaattacaaaagtctggaaaagctctattgcgatttcgcgagg**

**Citrus_reticulata** **ttgtctaattagcccacaaattacaaaagtctggaaaagctctattgcgatttcgcgagg**

**Citrus_sinensis** **ttgtctaattagcccacaaattacaaaagtctggaaaagctctattgcgatttcgcgagg**

**Fortunella_bawangica_1** **ttgtctaattagcccacaaattacaaaagtctggaaaagctctattgcgatttcgcgagg**

**Fortunella_bawangica_2** **ttgtctaattagcccacaaattacaaaagtctggaaaagctctattgcgatttcgcgagg**

**Fortunella_hindsii_1** **ttgtctaattagcccacaaattacaaaagtctggaaaagctctattgcgatttcgcgagg**

**Fortunella_hindsii_2** **ttgtctaattagcccacaaattacaaaagtctggaaaagctctattgcgatttcgcgagg**

**Fortunella_hindsii_3** **ttgtctaattagcccacaaattacaaaagtctggaaaagctctattgcgatttcgcgagg**

**Fortunella_japonica_1** **ttgtctaattagcccacaaattacaaaagtctggaaaagctctattgcgatttcgcgagg**

**Fortunella_japonica_2** **ttgtctaattagcccacaaattacaaaagtctggaaaagctctattgcgatttcgcgagg**

**Fortunella_japonica_3** **ttgtctaattagcccacaaattacaaaagtctggaaaagctctattgcgatttcgcgagg**

**Fortunella_japonica_4** **ttgtctaattagcccacaaattacaaaagtctggaaaagctctattgcgatttcgcgagg**

**Fortunella_japonica_5** **ttgtctaattagcccacaaattacaaaagtctggaaaagctctattgcgatttcgcgagg**

**Fortunella_japonica_6** **ttgtctaattagcccacaaattacaaaagtctggaaaagctctattgcgatttcgcgagg**

**Fortunella_japonica_7** **ttgtctaattagcccacaaattacaaaagtctggaaaagctctattgcgatttcgcgagg**

**Fortunella_japonica_8** **ttgtctaattagcccacaaattacaaaagtctggaaaagctctattgcgatttcgcgagg**

**Fortunella_japonica_9** **ttgtctaattagcccacaaattacaaaagtctggaaaagctctattgcgatttcgcgagg**

**Fortunella_japonica_10** **ttgtctaattagcccacaaattacaaaagtctggaaaagctctattgcgatttcgcgagg**

**Fortunella_japonica_11** **ttgtctaattagcccacaaattacaaaagtctggaaaagctctattgcgatttcgcgagg**

**Fortunella_japonica_12** **ttgtctaattagcccacaaattacaaaagtctggaaaagctctattgcgatttcgcgagg**

**Fortunella_margarita_1** **ttgtctaattagcccacaaattacaaaagtctggaaaagctctattgcgatttcgcgagg**

**Fortunella_margarita_2** **ttgtctaattagcccacaaattacaaaagtctggaaaagctctattgcgatttcgcgagg**

**Fortunella_venosa_1** **ttgtctaattagcccacaaattacaaaagtctggaaaagctctattgcgatttcgcgagg**

**Fortunella_venosa_2** **ttgtctaattagcccacaaattacaaaagtctggaaaagctctattgcgatttcgcgagg**

**Fortunella_venosa_3** **ttgtctaattagcccacaaattacaaaagtctggaaaagctctattgcgatttcgcgagg**

23890	23900	23910	23920	23930	23940

....|....|....|....|....|....|....|....|....|....|....|....|

**Atalantia_kwangtungens** **caatccacattgatgtaatgaaagtgaggggcccacaacaatgacagaccgccccgaata**

**Citrus_reticulata_1** **caatccacattgatgtaatgaaagtgaggggcccacaacaatgacagaccgccccgaata**

**Citrus_reticulata_2** **caatccacattgatgtaatgaaagtgaggggcccacaacaatgacagaccgccccgaata**

**Citrus_trifoliata_3** **caatccacattgatgtaatgaaagtgaggggcccacaacaatgacagaccgccccgaata**

**Citrus_aurantiifolia** **caatccacattgatgtaatgaaagtgaggggcccacaacaatgacagaccgccccgaata**

**Citrus_aurantium** **caatccacattgatgtaatgaaagtgaggggcccacaacaatgacagaccgccccgaata**

**Citrus_cavaleriei** **caatccacattgatgtaatgaaagtgaggggcccacaacaatgacagaccgccccgaata**

**Citrus_hongheensis** **caatccacattgatgtaatgaaagtgaggggcccacaacaatgacagaccgccccgaata**

**Citrus_limon** **caatccacattgatgtaatgaaagtgaggggcccacaacaatgacagaccgccccgaata**

**Citrus_maxima** **caatccacattgatgtaatgaaagtgaggggcccacaacaatgacagaccgccccgaata**

**Citrus_medica** **caatccacattgatgtaatgaaagtgaggggcccacaacaatgacagaccgccccgaata**

**Citrus_polytrifolia** **caatccacattgatgtaatgaaagtgaggggcccacaacaatgacagaccgccccgaata**

**Citrus_reticulata** **caatccacattgatgtaatgaaagtgaggggcccacaacaatgacagaccgccccgaata**

**Citrus_sinensis** **caatccacattgatgtaatgaaagtgaggggcccacaacaatgacagaccgccccgaata**

**Fortunella_bawangica_1** **caatccacattgatgtaatgaaagtgaggggcccacaacaatgacagaccgccccgaata**

**Fortunella_bawangica_2** **caatccacattgatgtaatgaaagtgaggggcccacaacaatgacagaccgccccgaata**

**Fortunella_hindsii_1** **caatccacattgatgtaatgaaagtgaggggcccacaacaatgacagaccgccccgaata**

**Fortunella_hindsii_2** **caatccacattgatgtaatgaaagtgaggggcccacaacaatgacagaccgccccgaata**

**Fortunella_hindsii_3** **caatccacattgatgtaatgaaagtgaggggcccacaacaatgacagaccgccccgaata**

**Fortunella_japonica_1** **caatccacattgatgtaatgaaagtgaggggcccacaacaatgacagaccgccccgaata**

**Fortunella_japonica_2** **caatccacattgatgtaatgaaagtgaggggcccacaacaatgacagaccgccccgaata**

**Fortunella_japonica_3** **caatccacattgatgtaatgaaagtgaggggcccacaacaatgacagaccgccccgaata**

**Fortunella_japonica_4** **caatccacattgatgtaatgaaagtgaggggcccacaacaatgacagaccgccccgaata**

**Fortunella_japonica_5** **caatccacattgatgtaatgaaagtgaggggcccacaacaatgacagaccgccccgaata**

**Fortunella_japonica_6** **caatccacattgatgtaatgaaagtgaggggcccacaacaatgacagaccgccccgaata**

**Fortunella_japonica_7** **caatccacattgatgtaatgaaagtgaggggcccacaacaatgacagaccgccccgaata**

**Fortunella_japonica_8** **caatccacattgatgtaatgaaagtgaggggcccacaacaatgacagaccgccccgaata**

**Fortunella_japonica_9** **caatccacattgatgtaatgaaagtgaggggcccacaacaatgacagaccgccccgaata**

**Fortunella_japonica_10** **caatccacattgatgtaatgaaagtgaggggcccacaacaatgacagaccgccccgaata**

**Fortunella_japonica_11** **caatccacattgatgtaatgaaagtgaggggcccacaacaatgacagaccgccccgaata**

**Fortunella_japonica_12** **caatccacattgatgtaatgaaagtgaggggcccacaacaatgacagaccgccccgaata**

**Fortunella_margarita_1** **caatccacattgatgtaatgaaagtgaggggcccacaacaatgacagaccgccccgaata**

**Fortunella_margarita_2** **caatccacattgatgtaatgaaagtgaggggcccacaacaatgacagaccgccccgaata**

**Fortunella_venosa_1** **caatccacattgatgtaatgaaagtgaggggcccacaacaatgacagaccgccccgaata**

**Fortunella_venosa_2** **caatccacattgatgtaatgaaagtgaggggcccacaacaatgacagaccgccccgaata**

**Fortunella_venosa_3** **caatccacattgatgtaatgaaagtgaggggcccacaacaatgacagaccgccccgaata**

23950	23960	23970	23980	23990	24000

....|....|....|....|....|....|....|....|....|....|....|....|

**Atalantia_kwangtungens** **atcgacccgtttgccaagcagagtctcacgaaatcttccctcttttccttcaattacatc**

**Citrus_reticulata_1** **atcgacccgtttgccaagcagagtctcacgaaatcttccctcttttccttcaattacatc**

**Citrus_reticulata_2** **atcgacccgtttgccaagcagagtctcacgaaatcttccctcttttccttcaattacatc**

**Citrus_trifoliata_3** **atcgacccgtttgccaagcagagtctcacgaaatcttccctcttttccttcaattacatc**

**Citrus_aurantiifolia** **atcgacccgtttgccaagcagagtctcacgaaatcttccctcttttccttcaattacatc**

**Citrus_aurantium** **atcgacccgtttgccaagcagagtctcacgaaatcttccctcttttccttcaattacatc**

**Citrus_cavaleriei** **atcgacccgtttgccaagcagagtctcacgaaatcttccctcttttccttcaattacatc**

**Citrus_hongheensis** **atcgacccgtttgccaagcagagtctcacgaaatcttccctcttttccttcaattacatc**

**Citrus_limon** **atcgacccgtttgccaagcagagtctcacgaaatcttccctcttttccttcaattacatc**

**Citrus_maxima** **atcgacccgtttgccaagcagagtctcacgaaatcttccctcttttccttcaattacatc**

**Citrus_medica** **atcgacccgtttgccaagcagagtctcacgaaatcttccctcttttccttcaattacatc**

**Citrus_polytrifolia** **atcgacccgtttgccaagcagagtctcacgaaatcttccctcttttccttcaattacatc**

**Citrus_reticulata** **atcgacccgtttgccaagcagagtctcacgaaatcttccctcttttccttcaattacatc**

**Citrus_sinensis** **atcgacccgtttgccaagcagagtctcacgaaatcttccctcttttccttcaattacatc**

**Fortunella_bawangica_1** **atcgacccgtttgccaagcagagtctcacgaaatcttccctcttttccttcaattacatc**

**Fortunella_bawangica_2** **atcgacccgtttgccaagcagagtctcacgaaatcttccctcttttccttcaattacatc**

**Fortunella_hindsii_1** **atcgacccgtttgccaagcagagtctcacgaaatcttccctcttttccttcaattacatc**

**Fortunella_hindsii_2** **atcgacccgtttgccaagcagagtctcacgaaatcttccctcttttccttcaattacatc**

**Fortunella_hindsii_3** **atcgacccgtttgccaagcagagtctcacgaaatcttccctcttttccttcaattacatc**

**Fortunella_japonica_1** **atcgacccgtttgccaagcagagtctcacgaaatcttccctcttttccttcaattacatc**

**Fortunella_japonica_2** **atcgacccgtttgccaagcagagtctcacgaaatcttccctcttttccttcaattacatc**

**Fortunella_japonica_3** **atcgacccgtttgccaagcagagtctcacgaaatcttccctcttttccttcaattacatc**

**Fortunella_japonica_4** **atcgacccgtttgccaagcagagtctcacgaaatcttccctcttttccttcaattacatc**

**Fortunella_japonica_5** **atcgacccgtttgccaagcagagtctcacgaaatcttccctcttttccttcaattacatc**

**Fortunella_japonica_6** **atcgacccgtttgccaagcagagtctcacgaaatcttccctcttttccttcaattacatc**

**Fortunella_japonica_7** **atcgacccgtttgccaagcagagtctcacgaaatcttccctcttttccttcaattacatc**

**Fortunella_japonica_8** **atcgacccgtttgccaagcagagtctcacgaaatcttccctcttttccttcaattacatc**

**Fortunella_japonica_9** **atcgacccgtttgccaagcagagtctcacgaaatcttccctcttttccttcaattacatc**

**Fortunella_japonica_10** **atcgacccgtttgccaagcagagtctcacgaaatcttccctcttttccttcaattacatc**

**Fortunella_japonica_11** **atcgacccgtttgccaagcagagtctcacgaaatcttccctcttttccttcaattacatc**

**Fortunella_japonica_12** **atcgacccgtttgccaagcagagtctcacgaaatcttccctcttttccttcaattacatc**

**Fortunella_margarita_1** **atcgacccgtttgccaagcagagtctcacgaaatcttccctcttttccttcaattacatc**

**Fortunella_margarita_2** **atcgacccgtttgccaagcagagtctcacgaaatcttccctcttttccttcaattacatc**

**Fortunella_venosa_1** **atcgacccgtttgccaagcagagtctcacgaaatcttccctcttttccttcaattacatc**

**Fortunella_venosa_2** **atcgacccgtttgccaagcagagtctcacgaaatcttccctcttttccttcaattacatc**

**Fortunella_venosa_3** **atcgacccgtttgccaagcagagtctcacgaaatcttccctcttttccttcaattacatc**

24010	24020	24030	24040	24050	24060

....|....|....|....|....|....|....|....|....|....|....|....|

**Atalantia_kwangtungens** **tgaaaacgacttataaatcttattatgaccatccctcattggctgtccacggattccatt**

**Citrus_reticulata_1** **tgaaaacgacttataaatcttattatgaccatccctcattggctgtccacggattccatt**

**Citrus_reticulata_2** **tgaaaacgacttataaatcttattatgaccatccctcattggctgtccacggattccatt**

**Citrus_trifoliata_3** **tgaaaacgacttataaatcttattatgaccatccctcattggctgtccacggattccatt**

**Citrus_aurantiifolia** **tgaaaacgacttataaatcttattatgaccatccctcattggctgtccacggattccatt**

**Citrus_aurantium** **tgaaaacgacttataaatcttattatgaccatccctcattggctgtccacggattccatt**

**Citrus_cavaleriei** **tgaaaacgacttataaatcttattatgaccatccctcattggctgtccacggattccatt**

**Citrus_hongheensis** **tgaaaacgacttataaatcttattatgaccatccctcattggctgtccacggattccatt**

**Citrus_limon** **tgaaaacgacttataaatcttattatgaccatccctcattggctgtccacggattccatt**

**Citrus_maxima** **tgaaaacgacttataaatcttattatgaccatccctcattggctgtccacggattccatt**

**Citrus_medica** **tgaaaacgacttataaatcttattatgaccatccctcattggctgtccacggattccatt**

**Citrus_polytrifolia** **tgaaaacgacttataaatcttattatgaccatccctcattggctgtccacggattccatt**

**Citrus_reticulata** **tgaaaacgacttataaatcttattatgaccatccctcattggctgtccacggattccatt**

**Citrus_sinensis** **tgaaaacgacttataaatcttattatgaccatccctcattggctgtccacggattccatt**

**Fortunella_bawangica_1** **tgaaaacgacttataaatcttattatgaccatccctcattggctgtccacggattccatt**

**Fortunella_bawangica_2** **tgaaaacgacttataaatcttattatgaccatccctcattggctgtccacggattccatt**

**Fortunella_hindsii_1** **tgaaaacgacttataaatcttattatgaccatccctcattggctgtccacggattccatt**

**Fortunella_hindsii_2** **tgaaaacgacttataaatcttattatgaccatccctcattggctgtccacggattccatt**

**Fortunella_hindsii_3** **tgaaaacgacttataaatcttattatgaccatccctcattggctgtccacggattccatt**

**Fortunella_japonica_1** **tgaaaacgacttataaatcttattatgaccatccctcattggctgtccacggattccatt**

**Fortunella_japonica_2** **tgaaaacgacttataaatcttattatgaccatccctcattggctgtccacggattccatt**

**Fortunella_japonica_3** **tgaaaacgacttataaatcttattatgaccatccctcattggctgtccacggattccatt**

**Fortunella_japonica_4** **tgaaaacgacttataaatcttattatgaccatccctcattggctgtccacggattccatt**

**Fortunella_japonica_5** **tgaaaacgacttataaatcttattatgaccatccctcattggctgtccacggattccatt**

**Fortunella_japonica_6** **tgaaaacgacttataaatcttattatgaccatccctcattggctgtccacggattccatt**

**Fortunella_japonica_7** **tgaaaacgacttataaatcttattatgaccatccctcattggctgtccacggattccatt**

**Fortunella_japonica_8** **tgaaaacgacttataaatcttattatgaccatccctcattggctgtccacggattccatt**

**Fortunella_japonica_9** **tgaaaacgacttataaatcttattatgaccatccctcattggctgtccacggattccatt**

**Fortunella_japonica_10** **tgaaaacgacttataaatcttattatgaccatccctcattggctgtccacggattccatt**

**Fortunella_japonica_11** **tgaaaacgacttataaatcttattatgaccatccctcattggctgtccacggattccatt**

**Fortunella_japonica_12** **tgaaaacgacttataaatcttattatgaccatccctcattggctgtccacggattccatt**

**Fortunella_margarita_1** **tgaaaacgacttataaatcttattatgaccatccctcattggctgtccacggattccatt**

**Fortunella_margarita_2** **tgaaaacgacttataaatcttattatgaccatccctcattggctgtccacggattccatt**

**Fortunella_venosa_1** **tgaaaacgacttataaatcttattatgaccatccctcattggctgtccacggattccatt**

**Fortunella_venosa_2** **tgaaaacgacttataaatcttattatgaccatccctcattggctgtccacggattccatt**

**Fortunella_venosa_3** **tgaaaacgacttataaatcttattatgaccatccctcattggctgtccacggattccatt**

24070	24080	24090	24100	24110	24120

....|....|....|....|....|....|....|....|....|....|....|....|

**Atalantia_kwangtungens** **atcaagaagcgtatccacggcttcttgtaccaatttctcctgacacattactaattcccc**

**Citrus_reticulata_1** **atcaagaagcgtatccacggcttcttgtaccaatttctcctgacacattactaattcccc**

**Citrus_reticulata_2** **atcaagaagcgtatccacggcttcttgtaccaatttctcctgacacattactaattcccc**

**Citrus_trifoliata_3** **atcaagaagcgtatccacggcttcttgtaccaatttctcctgacacattactaattcccc**

**Citrus_aurantiifolia** **atcaagaagcgtatccacggcttcttgtaccaatttctcctgacacattactaattcccc**

**Citrus_aurantium** **atcaagaagcgtatccacggcttcttgtaccaatttctcctgacacattaccaattcccc**

**Citrus_cavaleriei** **atcaagaagcgtatccacggcttcttgtaccaatttctcctgacacattactaattcccc**

**Citrus_hongheensis** **atcaagaagcgtatccacggcttcttgtaccaatttctcctgacacattaccaattcccc**

**Citrus_limon** **atcaagaagcgtatccacggcttcttgtaccaatttctcctgacacattaccaattcccc**

**Citrus_maxima** **atcaagaagcgtatccacggcttcttgtaccaatttctcctgacacattaccaattcccc**

**Citrus_medica** **atcaagaagcgtatccacggcttcttgtaccaatttctcctgacacattactaattcccc**

**Citrus_polytrifolia** **atcaagaagcgtatccacggcttcttgtaccaatttctcctgacacattactaattcccc**

**Citrus_reticulata** **atcaagaagcgtatccacggcttcttgtaccaatttctcctgacacattactaattcccc**

**Citrus_sinensis** **atcaagaagcgtatccacggcttcttgtaccaatttctcctgacacattaccaattcccc**

**Fortunella_bawangica_1** **atcaagaagcgtatccacggcttcttgtaccaatttctcctgacacattactaattcccc**

**Fortunella_bawangica_2** **atcaagaagcgtatccacggcttcttgtaccaatttctcctgacacattactaattcccc**

**Fortunella_hindsii_1** **atcaagaagcgtatccacggcttcttgtaccaatttctcctgacacattactaattcccc**

**Fortunella_hindsii_2** **atcaagaagcgtatccacggcttcttgtaccaatttctcctgacacattactaattcccc**

**Fortunella_hindsii_3** **atcaagaagcgtatccacggcttcttgtaccaatttctcctgacacattactaattcccc**

**Fortunella_japonica_1** **atcaagaagcgtatccacggcttcttgtaccaatttctcctgacacattactaattcccc**

**Fortunella_japonica_2** **atcaagaagcgtatccacggcttcttgtaccaatttctcctgacacattactaattcccc**

**Fortunella_japonica_3** **atcaagaagcgtatccacggcttcttgtaccaatttctcctgacacattactaattcccc**

**Fortunella_japonica_4** **atcaagaagcgtatccacggcttcttgtaccaatttctcctgacacattactaattcccc**

**Fortunella_japonica_5** **atcaagaagcgtatccacggcttcttgtaccaatttctcctgacacattactaattcccc**

**Fortunella_japonica_6** **atcaagaagcgtatccacggcttcttgtaccaatttctcctgacacattactaattcccc**

**Fortunella_japonica_7** **atcaagaagcgtatccacggcttcttgtaccaatttctcctgacacattactaattcccc**

**Fortunella_japonica_8** **atcaagaagcgtatccacggcttcttgtaccaatttctcctgacacattactaattcccc**

**Fortunella_japonica_9** **atcaagaagcgtatccacggcttcttgtaccaatttctcctgacacattactaattcccc**

**Fortunella_japonica_10** **atcaagaagcgtatccacggcttcttgtaccaatttctcctgacacattactaattcccc**

**Fortunella_japonica_11** **atcaagaagcgtatccacggcttcttgtaccaatttctcctgacacattactaattcccc**

**Fortunella_japonica_12** **atcaagaagcgtatccacggcttcttgtaccaatttctcctgacacattactaattcccc**

**Fortunella_margarita_1** **atcaagaagcgtatccacggcttcttgtaccaatttctcctgacacattactaattcccc**

**Fortunella_margarita_2** **atcaagaagcgtatccacggcttcttgtaccaatttctcctgacacattactaattcccc**

**Fortunella_venosa_1** **atcaagaagcgtatccacggcttcttgtaccaatttctcctgacacattactaattcccc**

**Fortunella_venosa_2** **atcaagaagcgtatccacggcttcttgtaccaatttctcctgacacattactaattcccc**

**Fortunella_venosa_3** **atcaagaagcgtatccacggcttcttgtaccaatttctcctgacacattactaattcccc**

24130	24140	24150	24160	24170	24180

....|....|....|....|....|....|....|....|....|....|....|....|

**Atalantia_kwangtungens** **tggcgtagatctacttgttgttaatagatcgataagagtattgttccgatagataactct**

**Citrus_reticulata_1** **tggcgtagatctacttgttgttaataggtcgataagagtattgttccgatagataactct**

**Citrus_reticulata_2** **tggcgtagatctacttgttgttaataggtcgataagagtattgttccgatagataactct**

**Citrus_trifoliata_3** **tggcgtagatctacttgttgttaataggtcgataagagtattgttccgatagataactct**

**Citrus_aurantiifolia** **tggcgtagatctacttgttgttaataggtcgataagagtattgttccgatagataactct**

**Citrus_aurantium** **tggcgtagatctacttgttgttaataggtcgataagagtattgttccgatagataactct**

**Citrus_cavaleriei** **tggcgtagatctacttgttgttaataggtcgataagagtattgttccgatagataactct**

**Citrus_hongheensis** **tggcgtagatctacttgttgttaataggtcgataagagtattgttccgatagataactct**

**Citrus_limon** **tggcgtagatctacttgttgttaataggtcgataagagtattgttccgatagataactct**

**Citrus_maxima** **tggcgtagatctacttgttgttaataggtcgataagagtattgttccgatagataactct**

**Citrus_medica** **tggcgtagatctacttgttgttaataggtcgataagagtattgttccgatagataactct**

**Citrus_polytrifolia** **tggcgtagatctacttgttgttaataggtcgataagagtattgttccgatagataactct**

**Citrus_reticulata** **tggcgtagatctacttgttgttaataggtcgataagagtattgttccgatagataactct**

**Citrus_sinensis** **tggcgtagatctacttgttgttaataggtcgataagagtattgttccgatagataactct**

**Fortunella_bawangica_1** **tggcgtagatctacttgttgttactaggtcgataagagtattgttccgatagataactct**

**Fortunella_bawangica_2** **tggcgtagatctacttgttgttactaggtcgataagagtattgttccgatagataactct**

**Fortunella_hindsii_1** **tggcgtagatctacttgttgttactaggtcgataagagtattgttccgatagataactct**

**Fortunella_hindsii_2** **tggcgtagatctacttgttgttactaggtcgataagagtattgttccgatagataactct**

**Fortunella_hindsii_3** **tggcgtagatctacttgttgttactaggtcgataagagtattgttccgatagataactct**

**Fortunella_japonica_1** **tggcgtagatctacttgttgttactaggtcgataagagtattgttccgatagataactct**

**Fortunella_japonica_2** **tggcgtagatctacttgttgttactaggtcgataagagtattgttccgatagataactct**

**Fortunella_japonica_3** **tggcgtagatctacttgttgttactaggtcgataagagtattgttccgatagataactct**

**Fortunella_japonica_4** **tggcgtagatctacttgttgttactaggtcgataagagtattgttccgatagataactct**

**Fortunella_japonica_5** **tggcgtagatctacttgttgttactaggtcgataagagtattgttccgatagataactct**

**Fortunella_japonica_6** **tggcgtagatctacttgttgttactaggtcgataagagtattgttccgatagataactct**

**Fortunella_japonica_7** **tggcgtagatctacttgttgttactaggtcgataagagtattgttccgatagataactct**

**Fortunella_japonica_8** **tggcgtagatctacttgttgttactaggtcgataagagtattgttccgatagataactct**

**Fortunella_japonica_9** **tggcgtagatctacttgttgttactaggtcgataagagtattgttccgatagataactct**

**Fortunella_japonica_10** **tggcgtagatctacttgttgttactaggtcgataagagtattgttccgatagataactct**

**Fortunella_japonica_11** **tggcgtagatctacttgttgttactaggtcgataagagtattgttccgatagataactct**

**Fortunella_japonica_12** **tggcgtagatctacttgttgttactaggtcgataagagtattgttccgatagataactct**

**Fortunella_margarita_1** **tggcgtagatctacttgttgttactaggtcgataagagtattgttccgatagataactct**

**Fortunella_margarita_2** **tggcgtagatctacttgttgttactaggtcgataagagtattgttccgatagataactct**

**Fortunella_venosa_1** **tggcgtagatctacttgttgttactaggtcgataagagtattgttccgatagataactct**

**Fortunella_venosa_2** **tggcgtagatctacttgttgttactaggtcgataagagtattgttccgatagataactct**

**Fortunella_venosa_3** **tggcgtagatctacttgttgttactaggtcgataagagtattgttccgatagataactct**

24190	24200	24210	24220	24230	24240

....|....|....|....|....|....|....|....|....|....|....|....|

**Atalantia_kwangtungens** **tctatagagttcattaatatctgaactcattagtttacctccatctatctgaatgatagg**

**Citrus_reticulata_1** **tctatagagttcattaatatctgaactcattagtttacctccatctatttgaatgatagg**

**Citrus_reticulata_2** **tctatagagttcattaatatctgaactcattagtttacctccatctatttgaatgatagg**

**Citrus_trifoliata_3** **tctatagagttcattaatatctgaactcattagtttacctccatctacttgaatgatagg**

**Citrus_aurantiifolia** **tctatagagttcattaatatctgaactcattagtttacctccatctatttgaatgatagg**

**Citrus_aurantium** **tcgatagagttcattaatatctgaactcattagtttacctccatctatttgaatgatagg**

**Citrus_cavaleriei** **tctatagagttcattaatatctgaactcattagtttacctccatctatttgaatgatagg**

**Citrus_hongheensis** **tctatagagttcattaatatctgaactcattagtttacctccatctatttgaatgatagg**

**Citrus_limon** **tcgatagagttcattaatatctgaactcattagtttacctccatctatttgaatgatagg**

**Citrus_maxima** **tctatagagttcattaatatctgaactcattagtttacctccatctatttgaatgatagg**

**Citrus_medica** **tctatagagttcattaatatctgaactcattagtttacctccatctatttgaatgatagg**

**Citrus_polytrifolia** **tctatagagttcattaatatctgaactcattagtttacctccatctacttgaatgatagg**

**Citrus_reticulata** **tctatagagttcattaatatctgaactcattagtttacctccatctatttgaatgatagg**

**Citrus_sinensis** **tctatagagttcattaatatctgaactcattagtttacctccatctatttgaatgatagg**

**Fortunella_bawangica_1** **tctatagagttcattaatatctgaactcattagtttacctccatctatttgaatgatagg**

**Fortunella_bawangica_2** **tctatagagttcattaatatctgaactcattagtttacctccatctatttgaatgatagg**

**Fortunella_hindsii_1** **tctatagagttcattaatatctgaactcattagtttacctccatctatttgaatgatagg**

**Fortunella_hindsii_2** **tctatagagttcattaatatctgaactcattagtttacctccatctatttgaatgatagg**

**Fortunella_hindsii_3** **tctatagagttcattaatatctgaactcattagtttacctccatctatttgaatgatagg**

**Fortunella_japonica_1** **tctatagagttcattaatatctgaactcattagtttacctccatctatttgaatgatagg**

**Fortunella_japonica_2** **tctatagagttcattaatatctgaactcattagtttacctccatctatttgaatgatagg**

**Fortunella_japonica_3** **tctatagagttcattaatatctgaactcattagtttacctccatctatttgaatgatagg**

**Fortunella_japonica_4** **tctatagagttcattaatatctgaactcattagtttacctccatctatttgaatgatagg**

**Fortunella_japonica_5** **tctatagagttcattaatatctgaactcattagtttacctccatctatttgaatgatagg**

**Fortunella_japonica_6** **tctatagagttcattaatatctgaactcattagtttacctccatctatttgaatgatagg**

**Fortunella_japonica_7** **tctatagagttcattaatatctgaactcattagtttacctccatctatttgaatgatagg**

**Fortunella_japonica_8** **tctatagagttcattaatatctgaactcattagtttacctccatctatttgaatgatagg**

**Fortunella_japonica_9** **tctatagagttcattaatatctgaactcattagtttacctccatctatttgaatgatagg**

**Fortunella_japonica_10** **tctatagagttcattaatatctgaactcattagtttacctccatctatttgaatgatagg**

**Fortunella_japonica_11** **tctatagagttcattaatatctgaactcattagtttacctccatctatttgaatgatagg**

**Fortunella_japonica_12** **tctatagagttcattaatatctgaactcattagtttacctccatctatttgaatgatagg**

**Fortunella_margarita_1** **tctatagagttcattaatatctgaactcattagtttacctccatctatttgaatgatagg**

**Fortunella_margarita_2** **tctatagagttcattaatatctgaactcattagtttacctccatctatttgaatgatagg**

**Fortunella_venosa_1** **tctatagagttcattaatatctgaactcattagtttacctccatctatttgaatgatagg**

**Fortunella_venosa_2** **tctatagagttcattaatatctgaactcattagtttacctccatctatttgaatgatagg**

**Fortunella_venosa_3** **tctatagagttcattaatatctgaactcattagtttacctccatctatttgaatgatagg**

24250	24260	24270	24280	24290	24300

....|....|....|....|....|....|....|....|....|....|....|....|

**Atalantia_kwangtungens** **cctcaactcgggaggaagaacaggtaatagacataaaaccatccactccggttctatatt**

**Citrus_reticulata_1** **cctcaactcgggaggaagaacaggtaatagacataaaaccatccactccggttctatatt**

**Citrus_reticulata_2** **cctcaactcgggaggaagaacaggtaatagacataaaaccatccactccggttctatatt**

**Citrus_trifoliata_3** **cctcaactcgggaggaagaacaggtaatagacataaaaccatccactccggttctatatt**

**Citrus_aurantiifolia** **cctcaactcgggaggaagaacaggtaatagacataaaaccatccactccggttctatatt**

**Citrus_aurantium** **cctcaactcgggaggaagaacaggtaatagacataaaaccatccactccggttctatatt**

**Citrus_cavaleriei** **cctcaactcgggaggaagaacaggtaatagacataaaaccatccactccggttctatatt**

**Citrus_hongheensis** **cctcaactcgggaggaagaacaggtaatagacataaaaccatccactccggttctatatt**

**Citrus_limon** **cctcaactcgggaggaagaacaggtaatagacataaaaccatccactccggttctatatt**

**Citrus_maxima** **cctcaactcgggaggaagaacaggtaatagacataaaaccatccactctggttctatatt**

**Citrus_medica** **cctcaactcgggaggaagaacaggtaatagacataaaaccatccactccggttctatatt**

**Citrus_polytrifolia** **cctcaactcgggaggaagaacaggtaatagacataaaaccatccactccggttctatatt**

**Citrus_reticulata** **cctcaactcgggaggaagaacaggtaatagacataaaaccatccactccggttctatatt**

**Citrus_sinensis** **cctcaactcgggaggaagaacaggtaatagacataaaaccatccactctggttctatatt**

**Fortunella_bawangica_1** **cctcaactcgggaggaagaacaggtaatagacataaaaccatccactccggttctatatt**

**Fortunella_bawangica_2** **cctcaactcgggaggaagaacaggtaatagacataaaaccatccactccggttctatatt**

**Fortunella_hindsii_1** **cctcaactcgggaggaagaacaggtaatagacataaaaccatccactccggttctatatt**

**Fortunella_hindsii_2** **cctcaactcgggaggaagaacaggtaatagacataaaaccatccactccggttctatatt**

**Fortunella_hindsii_3** **cctcaactcgggaggaagaacaggtaatagacataaaaccatccactccggttctatatt**

**Fortunella_japonica_1** **cctcaactcgggaggaagaacaggtaatagacataaaaccatccactccggttctatatt**

**Fortunella_japonica_2** **cctcaactcgggaggaagaacaggtaatagacataaaaccatccactccggttctatatt**

**Fortunella_japonica_3** **cctcaactcgggaggaagaacaggtaatagacataaaaccatccactccggttctatatt**

**Fortunella_japonica_4** **cctcaactcgggaggaagaacaggtaatagacataaaaccatccactccggttctatatt**

**Fortunella_japonica_5** **cctcaactcgggaggaagaacaggtaatagacataaaaccatccactccggttctatatt**

**Fortunella_japonica_6** **cctcaactcgggaggaagaacaggtaatagacataaaaccatccactccggttctatatt**

**Fortunella_japonica_7** **cctcaactcgggaggaagaacaggtaatagacataaaaccatccactccggttctatatt**

**Fortunella_japonica_8** **cctcaactcgggaggaagaacaggtaatagacataaaaccatccactccggttctatatt**

**Fortunella_japonica_9** **cctcaactcgggaggaagaacaggtaatagacataaaaccatccactccggttctatatt**

**Fortunella_japonica_10** **cctcaactcgggaggaagaacaggtaatagacataaaaccatccactccggttctatatt**

**Fortunella_japonica_11** **cctcaactcgggaggaagaacaggtaatagacataaaaccatccactccggttctatatt**

**Fortunella_japonica_12** **cctcaactcgggaggaagaacaggtaatagacataaaaccatccactccggttctatatt**

**Fortunella_margarita_1** **cctcaactcgggaggaagaacaggtaatagacataaaaccatccactccggttctatatt**

**Fortunella_margarita_2** **cctcaactcgggaggaagaacaggtaatagacataaaaccatccactccggttctatatt**

**Fortunella_venosa_1** **cctcaactcgggaggaagaacaggtaatagacataaaaccatccactccggttctatatt**

**Fortunella_venosa_2** **cctcaactcgggaggaagaacaggtaatagacataaaaccatccactccggttctatatt**

**Fortunella_venosa_3** **cctcaactcgggaggaagaacaggtaatagacataaaaccatccactccggttctatatt**

24310	24320	24330	24340	24350	24360

....|....|....|....|....|....|....|....|....|....|....|....|

**Atalantia_kwangtungens** **tgttcgaagaaaatgcttagctaattccatgcgtctaaccagaaaatcccttcttcgtcc**

**Citrus_reticulata_1** **tgttcgaagaaaatgcttagctaattccatgcgtctaaccaaaaaatcccttcttcgtcc**

**Citrus_reticulata_2** **tgttcgaagaaaatgcttagctaattccatgcgtctaaccaaaaaatcccttcttcgtcc**

**Citrus_trifoliata_3** **tgttcgaagaaaatgcttagctaattccatgcgtctaaccaaaaaatcccttcttcgtcc**

**Citrus_aurantiifolia** **tgttcgaagaaaatgcttagctaattccatgcgtctaaccaaaaaatcccttcttcgtcc**

**Citrus_aurantium** **tgttcgaagaaaatgcttagctaattccatgcgtctaaccaaaaaatcccttcttcgtcc**

**Citrus_cavaleriei** **tgttcgaagaaaatgcttagctaattccatgcgtctaaccaaaaaatcccttcttcgtcc**

**Citrus_hongheensis** **tgttcgaagaaaatgcttagctaattccatgcgtctaaccaaaaaatcccttcttcgtcc**

**Citrus_limon** **tgttcgaagaaaatgcttagctaattccatgcgtctaaccaaaaaatcccttcttcgtcc**

**Citrus_maxima** **tgttcgaagaaaatgcttagctaattccatgcgtctaaccaaaaaatcccttcttcgtcc**

**Citrus_medica** **tgttcgaagaaaatgcttagctaattccatgcgtctaaccaaaaaatcccttcttcgtcc**

**Citrus_polytrifolia** **tgttcgaagaaaatgcttagctaattccatgcgtctaaccaaaaaatcccttcttcgtcc**

**Citrus_reticulata** **tgttcgaagaaaatgcttagctaattccatgcgtctaaccaaaaaatcccttcttcgtcc**

**Citrus_sinensis** **tgttcgaagaaaatgcttagctaattccatgcgtctaaccaaaaaatcccttcttcgtcc**

**Fortunella_bawangica_1** **tgttcgaagaaaatgcttagctaattccatgcgtctaaccaaaaaatcccttcttcgtcc**

**Fortunella_bawangica_2** **tgttcgaagaaaatgcttagctaattccatgcgtctaaccaaaaaatcccttcttcgtcc**

**Fortunella_hindsii_1** **tgttcgaagaaaatgcttagctaattccatgcgtctaaccaaaaaatcccttcttcgtcc**

**Fortunella_hindsii_2** **tgttcgaagaaaatgcttagctaattccatgcgtctaaccaaaaaatcccttcttcgtcc**

**Fortunella_hindsii_3** **tgttcgaagaaaatgcttagctaattccatgcgtctaaccaaaaaatcccttcttcgtcc**

**Fortunella_japonica_1** **tgttcgaagaaaatgcttagctaattccatgcgtctaaccaaaaaatcccttcttcgtcc**

**Fortunella_japonica_2** **tgttcgaagaaaatgcttagctaattccatgcgtctaaccaaaaaatcccttcttcgtcc**

**Fortunella_japonica_3** **tgttcgaagaaaatgcttagctaattccatgcgtctaaccaaaaaatcccttcttcgtcc**

**Fortunella_japonica_4** **tgttcgaagaaaatgcttagctaattccatgcgtctaaccaaaaaatcccttcttcgtcc**

**Fortunella_japonica_5** **tgttcgaagaaaatgcttagctaattccatgcgtctaaccaaaaaatcccttcttcgtcc**

**Fortunella_japonica_6** **tgttcgaagaaaatgcttagctaattccatgcgtctaaccaaaaaatcccttcttcgtcc**

**Fortunella_japonica_7** **tgttcgaagaaaatgcttagctaattccatgcgtctaaccaaaaaatcccttcttcgtcc**

**Fortunella_japonica_8** **tgttcgaagaaaatgcttagctaattccatgcgtctaaccaaaaaatcccttcttcgtcc**

**Fortunella_japonica_9** **tgttcgaagaaaatgcttagctaattccatgcgtctaaccaaaaaatcccttcttcgtcc**

**Fortunella_japonica_10** **tgttcgaagaaaatgcttagctaattccatgcgtctaaccaaaaaatcccttcttcgtcc**

**Fortunella_japonica_11** **tgttcgaagaaaatgcttagctaattccatgcgtctaaccaaaaaatcccttcttcgtcc**

**Fortunella_japonica_12** **tgttcgaagaaaatgcttagctaattccatgcgtctaaccaaaaaatcccttcttcgtcc**

**Fortunella_margarita_1** **tgttcgaagaaaatgcttagctaattccatgcgtctaaccaaaaaatcccttcttcgtcc**

**Fortunella_margarita_2** **tgttcgaagaaaatgcttagctaattccatgcgtctaaccaaaaaatcccttcttcgtcc**

**Fortunella_venosa_1** **tgttcgaagaaaatgcttagctaattccatgcgtctaaccaaaaaatcccttcttcgtcc**

**Fortunella_venosa_2** **tgttcgaagaaaatgcttagctaattccatgcgtctaaccaaaaaatcccttcttcgtcc**

**Fortunella_venosa_3** **tgttcgaagaaaatgcttagctaattccatgcgtctaaccaaaaaatcccttcttcgtcc**

24370	24380	24390	24400	24410	24420

....|....|....|....|....|....|....|....|....|....|....|....|

**Atalantia_kwangtungens** **aatttttcgctcttcccagtcattaccggcgggcccttcttcccctaattctttccattc**

**Citrus_reticulata_1** **aatttttcgatcttcccagtcattaccggcgggcccttcttccactaattctttccattc**

**Citrus_reticulata_2** **aatttttcgatcttcccagtcattaccggcgggcccttcttccactaattctttccattc**

**Citrus_trifoliata_3** **aatttttcgatcttcccagtcattaccggcgggcccttcttcccctaattctttccattc**

**Citrus_aurantiifolia** **aatttttcgatcttcccagtcattaccggcgggcccttcttcccctaattctttccattc**

**Citrus_aurantium** **aatttttcgatcttcccagtcattaccggcgggcccttcttcccctaattctttccattc**

**Citrus_cavaleriei** **aatttttcgatcttcccagtcattaccggcgggcccttcttcccctaattctttccattc**

**Citrus_hongheensis** **aatttttcgatcttcccagtcattaccggcgggcccttcttcccctaattctttccattc**

**Citrus_limon** **aatttttcgatcttcccagtcattaccggcgggcccttcttcccctaattctttccattc**

**Citrus_maxima** **aatttttcgatcttcccagtcattaccggcgggcccttcttcccctaattctttccattc**

**Citrus_medica** **aatttttcgatcttcccagtcattaccggcgggcccttcttcccctaattctttccattc**

**Citrus_polytrifolia** **aatttttcgatcttcccagtcattaccggcgggcccttcttcccctaattctttccattc**

**Citrus_reticulata** **aatttttcgatcttcccagtcattaccggcgggcccttcttccactaattctttccattc**

**Citrus_sinensis** **aatttttcgatcttcccagtcattaccggcgggcccttcttcccctaattctttccattc**

**Fortunella_bawangica_1** **aatttttcgatcttcccagtcattaccggcgggcccttcttcccctaattctttccattc**

**Fortunella_bawangica_2** **aatttttcgatcttcccagtcattaccggcgggcccttcttcccctaattctttccattc**

**Fortunella_hindsii_1** **aatttttcgatcttcccagtcattaccggcgggcccttcttcccctaattctttccattc**

**Fortunella_hindsii_2** **aatttttcgatcttcccagtcattaccggcgggcccttcttcccctaattctttccattc**

**Fortunella_hindsii_3** **aatttttcgatcttcccagtcattaccggcgggcccttcttcccctaattctttccattc**

**Fortunella_japonica_1** **aatttttcgatcttcccagtcattaccggcgggcccttcttcccctaattctttccattc**

**Fortunella_japonica_2** **aatttttcgatcttcccagtcattaccggcgggcccttcttcccctaattctttccattc**

**Fortunella_japonica_3** **aatttttcgatcttcccagtcattaccggcgggcccttcttcccctaattctttccattc**

**Fortunella_japonica_4** **aatttttcgatcttcccagtcattaccggcgggcccttcttcccctaattctttccattc**

**Fortunella_japonica_5** **aatttttcgatcttcccagtcattaccggcgggcccttcttcccctaattctttccattc**

**Fortunella_japonica_6** **aatttttcgatcttcccagtcattaccggcgggcccttcttcccctaattctttccattc**

**Fortunella_japonica_7** **aatttttcgatcttcccagtcattaccggcgggcccttcttcccctaattctttccattc**

**Fortunella_japonica_8** **aatttttcgatcttcccagtcattaccggcgggcccttcttcccctaattctttccattc**

**Fortunella_japonica_9** **aatttttcgatcttcccagtcattaccggcgggcccttcttcccctaattctttccattc**

**Fortunella_japonica_10** **aatttttcgatcttcccagtcattaccggcgggcccttcttcccctaattctttccattc**

**Fortunella_japonica_11** **aatttttcgatcttcccagtcattaccggcgggcccttcttcccctaattctttccattc**

**Fortunella_japonica_12** **aatttttcgatcttcccagtcattaccggcgggcccttcttcccctaattctttccattc**

**Fortunella_margarita_1** **aatttttcgatcttcccagtcattaccggcgggcccttcttcccctaattctttccattc**

**Fortunella_margarita_2** **aatttttcgatcttcccagtcattaccggcgggcccttcttcccctaattctttccattc**

**Fortunella_venosa_1** **aatttttcgatcttcccagtcattaccggcgggcccttcttcccctaattctttccattc**

**Fortunella_venosa_2** **aatttttcgatcttcccagtcattaccggcgggcccttcttcccctaattctttccattc**

**Fortunella_venosa_3** **aatttttcgatcttcccagtcattaccggcgggcccttcttcccctaattctttccattc**

24430	24440	24450	24460	24470	24480

....|....|....|....|....|....|....|....|....|....|....|....|

**Atalantia_kwangtungens** **taacaaggaattatctagaataattcgcaaatctagatcggctaattgttcccggatagc**

**Citrus_reticulata_1** **taacaaggaattatctagaataattcgcaaatctagatcggctaattgttcccggatagc**

**Citrus_reticulata_2** **taacaaggaattatctagaataattcgcaaatctagatcggctaattgttcccggatagc**

**Citrus_trifoliata_3** **taacaaggaattatctagaataattcgcaaatctagatcggctaattgttcccggatagc**

**Citrus_aurantiifolia** **taacaaggaattatctagaataattcgcaaatctagatcggctaattgttcccggatagc**

**Citrus_aurantium** **taacaaggaattatctagaataattcgcaaatctagatcggctaattgttcccggatagc**

**Citrus_cavaleriei** **taacaaggaattatctagaataattcgcaaatctagatcggctaattgttcccggatagc**

**Citrus_hongheensis** **taacaaggaattatctagaataattcgcaaatctagatcggctaattgttcccggatagc**

**Citrus_limon** **taacaaggaattatctagaataattcgcaaatctagatcggctaattgttcccggatagc**

**Citrus_maxima** **taacaaggaattatctagaataattcgcaaatctagatcggctaattgttcccggatagc**

**Citrus_medica** **taacaaggaattatctagaataattcgcaaatctagatcggctaattgttcccggatagc**

**Citrus_polytrifolia** **taacaaggaattatctagaataattcgcaaatctagatcggctaattgttcccggatagc**

**Citrus_reticulata** **taacaaggaattatctagaataattcgcaaatctagatcggctaattgttcccggatagc**

**Citrus_sinensis** **taacaaggaattatctagaataattcgcaaatctagatcggctaattgttcccggatagc**

**Fortunella_bawangica_1** **taacaaggaattatctagaataattcgcaaatctagatcggctaattgttcccggatagc**

**Fortunella_bawangica_2** **taacaaggaattatctagaataattcgcaaatctagatcggctaattgttcccggatagc**

**Fortunella_hindsii_1** **taacaaggaattatctagaataattcgcaaatctagatcggctaattgttcccggatagc**

**Fortunella_hindsii_2** **taacaaggaattatctagaataattcgcaaatctagatcggctaattgttcccggatagc**

**Fortunella_hindsii_3** **taacaaggaattatctagaataattcgcaaatctagatcggctaattgttcccggatagc**

**Fortunella_japonica_1** **taacaaggaattatctagaataattcgcaaatctagatcggctaattgttcccggatagc**

**Fortunella_japonica_2** **taacaaggaattatctagaataattcgcaaatctagatcggctaattgttcccggatagc**

**Fortunella_japonica_3** **taacaaggaattatctagaataattcgcaaatctagatcggctaattgttcccggatagc**

**Fortunella_japonica_4** **taacaaggaattatctagaataattcgcaaatctagatcggctaattgttcccggatagc**

**Fortunella_japonica_5** **taacaaggaattatctagaataattcgcaaatctagatcggctaattgttcccggatagc**

**Fortunella_japonica_6** **taacaaggaattatctagaataattcgcaaatctagatcggctaattgttcccggatagc**

**Fortunella_japonica_7** **taacaaggaattatctagaataattcgcaaatctagatcggctaattgttcccggatagc**

**Fortunella_japonica_8** **taacaaggaattatctagaataattcgcaaatctagatcggctaattgttcccggatagc**

**Fortunella_japonica_9** **taacaaggaattatctagaataattcgcaaatctagatcggctaattgttcccggatagc**

**Fortunella_japonica_10** **taacaaggaattatctagaataattcgcaaatctagatcggctaattgttcccggatagc**

**Fortunella_japonica_11** **taacaaggaattatctagaataattcgcaaatctagatcggctaattgttcccggatagc**

**Fortunella_japonica_12** **taacaaggaattatctagaataattcgcaaatctagatcggctaattgttcccggatagc**

**Fortunella_margarita_1** **taacaaggaattatctagaataattcgcaaatctagatcggctaattgttcccggatagc**

**Fortunella_margarita_2** **taacaaggaattatctagaataattcgcaaatctagatcggctaattgttcccggatagc**

**Fortunella_venosa_1** **taacaaggaattatctagaataattcgcaaatctagatcggctaattgttcccggatagc**

**Fortunella_venosa_2** **taacaaggaattatctagaataattcgcaaatctagatcggctaattgttcccggatagc**

**Fortunella_venosa_3** **taacaaggaattatctagaataattcgcaaatctagatcggctaattgttcccggatagc**

24490	24500	24510	24520	24530	24540

....|....|....|....|....|....|....|....|....|....|....|....|

**Atalantia_kwangtungens** **aactgctccagtagaaatttctcggttacgaaatttatcgaaaccctgggtagtaaaaaa**

**Citrus_reticulata_1** **aactgctccagtagaaatttctcggttacgaaatttatcgaaaccttgggtagtaaaaaa**

**Citrus_reticulata_2** **aactgctccagtagaaatttctcggttacgaaatttatcgaaaccttgggtagtaaaaaa**

**Citrus_trifoliata_3** **aactgctccagtagaaatttctcggttacgaaatttatcgaaaccttgggtagtaaaaaa**

**Citrus_aurantiifolia** **aactgctccagtagaaatttctcggttacgaaatttatcgaaaccttgggtagtaaaaaa**

**Citrus_aurantium** **aactgctccagtagaaatttctcggttacgaaatttatcgaaaccttgggtagtaaaaaa**

**Citrus_cavaleriei** **aactgctccagtagaaatttctcggttacgaaatttatcgaaaccttgggtagtaaaaaa**

**Citrus_hongheensis** **aactgctccagtagaaatttctcggttacgaaatttatcgaaaccttgggtagtaaaaaa**

**Citrus_limon** **aactgctccagtagaaatttctcggttacgaaatttatcgaaaccttgggtagtaaaaaa**

**Citrus_maxima** **aactgctccagtagaaatttctcggttacgaaatttatcgaaaccttgggtagtaaaaaa**

**Citrus_medica** **aactgctccagtagaaatttctcggttacgaaatttatcgaaaccttgggtagtaaaaaa**

**Citrus_polytrifolia** **aactgctccagtagaaatttctcggttacgaaatttatcgaaaccttgggtagtaaaaaa**

**Citrus_reticulata** **aactgctccagtagaaatttctcggttacgaaatttatcgaaaccttgggtagtaaaaaa**

**Citrus_sinensis** **aactgctccagtagaaatttctcggttacgaaatttatcgaaaccttgggtagtaaaaaa**

**Fortunella_bawangica_1** **aactgctccagtagaaatttctcggttacgaaatttatcgaaaccttgggtagtaaaaaa**

**Fortunella_bawangica_2** **aactgctccagtagaaatttctcggttacgaaatttatcgaaaccttgggtagtaaaaaa**

**Fortunella_hindsii_1** **aactgctccagtagaaatttctcggttacgaaatttatcgaaaccttgggtagtaaaaaa**

**Fortunella_hindsii_2** **aactgctccagtagaaatttctcggttacgaaatttatcgaaaccttgggtagtaaaaaa**

**Fortunella_hindsii_3** **aactgctccagtagaaatttctcggttacgaaatttatcgaaaccttgggtagtaaaaaa**

**Fortunella_japonica_1** **aactgctccagtagaaatttctcggttacgaaatttatcgaaaccttgggtagtaaaaaa**

**Fortunella_japonica_2** **aactgctccagtagaaatttctcggttacgaaatttatcgaaaccttgggtagtaaaaaa**

**Fortunella_japonica_3** **aactgctccagtagaaatttctcggttacgaaatttatcgaaaccttgggtagtaaaaaa**

**Fortunella_japonica_4** **aactgctccagtagaaatttctcggttacgaaatttatcgaaaccttgggtagtaaaaaa**

**Fortunella_japonica_5** **aactgctccagtagaaatttctcggttacgaaatttatcgaaaccttgggtagtaaaaaa**

**Fortunella_japonica_6** **aactgctccagtagaaatttctcggttacgaaatttatcgaaaccttgggtagtaaaaaa**

**Fortunella_japonica_7** **aactgctccagtagaaatttctcggttacgaaatttatcgaaaccttgggtagtaaaaaa**

**Fortunella_japonica_8** **aactgctccagtagaaatttctcggttacgaaatttatcgaaaccttgggtagtaaaaaa**

**Fortunella_japonica_9** **aactgctccagtagaaatttctcggttacgaaatttatcgaaaccttgggtagtaaaaaa**

**Fortunella_japonica_10** **aactgctccagtagaaatttctcggttacgaaatttatcgaaaccttgggtagtaaaaaa**

**Fortunella_japonica_11** **aactgctccagtagaaatttctcggttacgaaatttatcgaaaccttgggtagtaaaaaa**

**Fortunella_japonica_12** **aactgctccagtagaaatttctcggttacgaaatttatcgaaaccttgggtagtaaaaaa**

**Fortunella_margarita_1** **aactgctccagtagaaatttctcggttacgaaatttatcgaaaccttgggtagtaaaaaa**

**Fortunella_margarita_2** **aactgctccagtagaaatttctcggttacgaaatttatcgaaaccttgggtagtaaaaaa**

**Fortunella_venosa_1** **aactgctccagtagaaatttctcggttacgaaatttatcgaaaccttgggtagtaaaaaa**

**Fortunella_venosa_2** **aactgctccagtagaaatttctcggttacgaaatttatcgaaaccttgggtagtaaaaaa**

**Fortunella_venosa_3** **aactgctccagtagaaatttctcggttacgaaatttatcgaaaccttgggtagtaaaaaa**

24550	24560	24570	24580	24590	24600

....|....|....|....|....|....|....|....|....|....|....|....|

**Atalantia_kwangtungens** **aagtgggatgctgtatttccaggattgtatttcatattcgaatgaaccgcgtaatcgtaa**

**Citrus_reticulata_1** **aagtgggatgctgtatttccaggattgtatttcatattcgaatgaaccgcgtaatcgtaa**

**Citrus_reticulata_2** **aagtgggatgctgtatttccaggattgtatttcatattcgaatgaaccgcgtaatcgtaa**

**Citrus_trifoliata_3** **aagtgggatgctgtatttccaggattgtatttcatattcgaatgaaccgcgtaatcgtaa**

**Citrus_aurantiifolia** **aagtgggatgctgtatttccaggattgtatttcatattcgaatgaaccgcgtaatcgtaa**

**Citrus_aurantium** **aagtgggatgctgtatttccaggattgtatttcatattcgaatgaaccgcgtaatcgtaa**

**Citrus_cavaleriei** **aagtgggatgctgtatttccaggattgtatttcatattcgaatgaaccgcgtaatcgtaa**

**Citrus_hongheensis** **aagtgggatgctgtatttccaggattgtatttcatattcgaatgaaccgcgtaatcgtaa**

**Citrus_limon** **aagtgggatgctgtatttccaggattgtatttcatattcgaatgaaccgcgtaatcgtaa**

**Citrus_maxima** **aagtgggatgctgtatttccaggattgtatttcatattcgaatgaaccgcgtaatcgtaa**

**Citrus_medica** **aagtgggatgctgtatttccaggattgtatttcatattcgaatgaaccgcgtaatcgtaa**

**Citrus_polytrifolia** **aagtgggatgctgtatttccaggattgtatttcatattcgaatgaaccgcgtaatcgtaa**

**Citrus_reticulata** **aagtgggatgctgtatttccaggattgtatttcatattcgaatgaaccgcgtaatcgtaa**

**Citrus_sinensis** **aagtgggatgctgtatttccaggattgtatttcatattcgaatgaaccgcgtaatcgtaa**

**Fortunella_bawangica_1** **aagtgggatgctgtatttccaggattgtatttcatattcgaatgaaccgcgtaatcgtaa**

**Fortunella_bawangica_2** **aagtgggatgctgtatttccaggattgtatttcatattcgaatgaaccgcgtaatcgtaa**

**Fortunella_hindsii_1** **aagtgggatgctgtatttccaggattgtatttcatattcgaatgaaccgcgtaatcgtaa**

**Fortunella_hindsii_2** **aagtgggatgctgtatttccaggattgtatttcatattcgaatgaaccgcgtaatcgtaa**

**Fortunella_hindsii_3** **aagtgggatgctgtatttccaggattgtatttcatattcgaatgaaccgcgtaatcgtaa**

**Fortunella_japonica_1** **aagtgggatgctgtatttccaggattgtatttcatattcgaatgaaccgcgtaatcgtaa**

**Fortunella_japonica_2** **aagtgggatgctgtatttccaggattgtatttcatattcgaatgaaccgcgtaatcgtaa**

**Fortunella_japonica_3** **aagtgggatgctgtatttccaggattgtatttcatattcgaatgaaccgcgtaatcgtaa**

**Fortunella_japonica_4** **aagtgggatgctgtatttccaggattgtatttcatattcgaatgaaccgcgtaatcgtaa**

**Fortunella_japonica_5** **aagtgggatgctgtatttccaggattgtatttcatattcgaatgaaccgcgtaatcgtaa**

**Fortunella_japonica_6** **aagtgggatgctgtatttccaggattgtatttcatattcgaatgaaccgcgtaatcgtaa**

**Fortunella_japonica_7** **aagtgggatgctgtatttccaggattgtatttcatattcgaatgaaccgcgtaatcgtaa**

**Fortunella_japonica_8** **aagtgggatgctgtatttccaggattgtatttcatattcgaatgaaccgcgtaatcgtaa**

**Fortunella_japonica_9** **aagtgggatgctgtatttccaggattgtatttcatattcgaatgaaccgcgtaatcgtaa**

**Fortunella_japonica_10** **aagtgggatgctgtatttccaggattgtatttcatattcgaatgaaccgcgtaatcgtaa**

**Fortunella_japonica_11** **aagtgggatgctgtatttccaggattgtatttcatattcgaatgaaccgcgtaatcgtaa**

**Fortunella_japonica_12** **aagtgggatgctgtatttccaggattgtatttcatattcgaatgaaccgcgtaatcgtaa**

**Fortunella_margarita_1** **aagtgggatgctgtatttccaggattgtatttcatattcgaatgaaccgcgtaatcgtaa**

**Fortunella_margarita_2** **aagtgggatgctgtatttccaggattgtatttcatattcgaatgaaccgcgtaatcgtaa**

**Fortunella_venosa_1** **aagtgggatgctgtatttccaggattgtatttcatattcgaatgaaccgcgtaatcgtaa**

**Fortunella_venosa_2** **aagtgggatgctgtatttccaggattgtatttcatattcgaatgaaccgcgtaatcgtaa**

**Fortunella_venosa_3** **aagtgggatgctgtatttccaggattgtatttcatattcgaatgaaccgcgtaatcgtaa**

24610	24620	24630	24640	24650	24660

....|....|....|....|....|....|....|....|....|....|....|....|

**Atalantia_kwangtungens** **aaaagtcggttttttcgctataggcctagcaaaagaaaaattgggataggaccctatcct**

**Citrus_reticulata_1** **aaaagtcggttttttcgctataggcctagcaaaagaaaaattgggataggaccctatcct**

**Citrus_reticulata_2** **aaaagtcggttttttcgctataggcctagcaaaagaaaaattgggataggaccctatcct**

**Citrus_trifoliata_3** **aaaagtaggttttttcgctataggcctagcaaaagaaaaattgggataggaccctatcct**

**Citrus_aurantiifolia** **aaaagtcggttttttcgctataggcctagcaaaagaaaaattgggataggaccctatcct**

**Citrus_aurantium** **aaaagtcggttttttcgctataggcctagcaaaagaaaaattgggataggaccctatcct**

**Citrus_cavaleriei** **aaaagtcggttttttcgctataggcctagcaaaagaaaaattgggataggaccctatcct**

**Citrus_hongheensis** **aaaagtcggttttttcgctataggcctagcaaaagaaaaattgggataggaccctatcct**

**Citrus_limon** **aaaagtcggttttttcgctataggcctagcaaaagaaaaattgggataggaccctatcct**

**Citrus_maxima** **aaaagtcggttttttcgctataggcctagcaaaagaaaaattgggataggaccctatcct**

**Citrus_medica** **aaaagtcggttttttcgctataggcctagcaaaagaaaaattgggataggaccctatcct**

**Citrus_polytrifolia** **aaaagtaggttttttcgctataggcctagcaaaagaaaaattgggataggaccctatcct**

**Citrus_reticulata** **aaaagtcggttttttcgctataggcctagcaaaagaaaaattgggataggaccctatcct**

**Citrus_sinensis** **aaaagtcggttttttcgctataggcctagcaaaagaaaaattgggataggaccctatcct**

**Fortunella_bawangica_1** **aaaagtcggttttttcgctataggcctagcaaaagaaaaattgggataggaccctatcct**

**Fortunella_bawangica_2** **aaaagtcggttttttcgctataggcctagcaaaagaaaaattgggataggaccctatcct**

**Fortunella_hindsii_1** **aaaagtcggttttttcgctataggcctagcaaaagaaaaattgggataggaccctatcct**

**Fortunella_hindsii_2** **aaaagtcggttttttcgctataggcctagcaaaagaaaaattgggataggaccctatcct**

**Fortunella_hindsii_3** **aaaagtcggttttttcgctataggcctagcaaaagaaaaattgggataggaccctatcct**

**Fortunella_japonica_1** **aaaagtcggttttttcgctataggcctagcaaaagaaaaattgggataggaccctatcct**

**Fortunella_japonica_2** **aaaagtcggttttttcgctataggcctagcaaaagaaaaattgggataggaccctatcct**

**Fortunella_japonica_3** **aaaagtcggttttttcgctataggcctagcaaaagaaaaattgggataggaccctatcct**

**Fortunella_japonica_4** **aaaagtcggttttttcgctataggcctagcaaaagaaaaattgggataggaccctatcct**

**Fortunella_japonica_5** **aaaagtcggttttttcgctataggcctagcaaaagaaaaattgggataggaccctatcct**

**Fortunella_japonica_6** **aaaagtcggttttttcgctataggcctagcaaaagaaaaattgggataggaccctatcct**

**Fortunella_japonica_7** **aaaagtcggttttttcgctataggcctagcaaaagaaaaattgggataggaccctatcct**

**Fortunella_japonica_8** **aaaagtcggttttttcgctataggcctagcaaaagaaaaattgggataggaccctatcct**

**Fortunella_japonica_9** **aaaagtcggttttttcgctataggcctagcaaaagaaaaattgggataggaccctatcct**

**Fortunella_japonica_10** **aaaagtcggttttttcgctataggcctagcaaaagaaaaattgggataggaccctatcct**

**Fortunella_japonica_11** **aaaagtcggttttttcgctataggcctagcaaaagaaaaattgggataggaccctatcct**

**Fortunella_japonica_12** **aaaagtcggttttttcgctataggcctagcaaaagaaaaattgggataggaccctatcct**

**Fortunella_margarita_1** **aaaagtcggttttttcgctataggcctagcaaaagaaaaattgggataggaccctatcct**

**Fortunella_margarita_2** **aaaagtcggttttttcgctataggcctagcaaaagaaaaattgggataggaccctatcct**

**Fortunella_venosa_1** **aaaagtcggttttttcgctataggcctagcaaaagaaaaattgggataggaccctatcct**

**Fortunella_venosa_2** **aaaagtcggttttttcgctataggcctagcaaaagaaaaattgggataggaccctatcct**

**Fortunella_venosa_3** **aaaagtcggttttttcgctataggcctagcaaaagaaaaattgggataggaccctatcct**

24670	24680	24690	24700	24710	24720

....|....|....|....|....|....|....|....|....|....|....|....|

**Atalantia_kwangtungens** **atgggatct-cccccccatcaaaatcggacatgagcgtttcttctcatccggctcaagta**

**Citrus_reticulata_1** **atgggatct-cccccccatcaaaatcggacatgagcgtttcttctcatccggctcaagta**

**Citrus_reticulata_2** **atgggatct-cccccccatcaaaatcggacatgagcgtttcttctcatccggctcaagta**

**Citrus_trifoliata_3** **atgggatct-cccccccatcaaaatcggacatgagcgtttcttctcatccggctcaagta**

**Citrus_aurantiifolia** **atgggatct-cccccccatcaaaatcggacatgagcgtttcttctcatccggctcaagta**

**Citrus_aurantium** **atgggatct-cccccccatcaaaatcggacatgagcgtttcttctcatccggctcaagta**

**Citrus_cavaleriei** **atgggatct-cccccccatcaaaatcggacatgagcgtttcttctcatccggctcaagta**

**Citrus_hongheensis** **atgggatct-cccccccatcaaaatcggacatgagcgtttcttctcatccggctcaagta**

**Citrus_limon** **atgggatct-cccccccatcaaaatcggacatgagcgtttcttctcatccggctcaagta**

**Citrus_maxima** **atgggatct-cccccccatcaaaatcggacatgagcgtttcttctcatccggctcaagta**

**Citrus_medica** **atgggatctccccccccatcaaaatcggacatgagcgtttcttctcatccggctcaagta**

**Citrus_polytrifolia** **atgggatct-cccccccatcaaaatcggacatgagcgtttcttctcatccggctcaagta**

**Citrus_reticulata** **atgggatct-cccccccatcaaaatcggacatgagcgtttcttctcatccggctcaagta**

**Citrus_sinensis** **atgggatct-cccccccatcaaaatcggacatgagcgtttcttctcatccggctcaagta**

**Fortunella_bawangica_1** **atgggatct-cccccccatcaaaatcggacatgagcgtttcttctcatccggctcaagta**

**Fortunella_bawangica_2** **atgggatct-cccccccatcaaaatcggacatgagcgtttcttctcatccggctcaagta**

**Fortunella_hindsii_1** **atgggatct-cccccccatcaaaatcggacatgagcgtttcttctcatccggctcaagta**

**Fortunella_hindsii_2** **atgggatct-cccccccatcaaaatcggacatgagcgtttcttctcatccggctcaagta**

**Fortunella_hindsii_3** **atgggatct-cccccccatcaaaatcggacatgagcgtttcttctcatccggctcaagta**

**Fortunella_japonica_1** **atgggatct-cccccccatcaaaatcggacatgagcgtttcttctcatccggctcaagta**

**Fortunella_japonica_2** **atgggatct-cccccccatcaaaatcggacatgagcgtttcttctcatccggctcaagta**

**Fortunella_japonica_3** **atgggatct-cccccccatcaaaatcggacatgagcgtttcttctcatccggctcaagta**

**Fortunella_japonica_4** **atgggatct-cccccccatcaaaatcggacatgagcgtttcttctcatccggctcaagta**

**Fortunella_japonica_5** **atgggatct-cccccccatcaaaatcggacatgagcgtttcttctcatccggctcaagta**

**Fortunella_japonica_6** **atgggatct-cccccccatcaaaatcggacatgagcgtttcttctcatccggctcaagta**

**Fortunella_japonica_7** **atgggatct-cccccccatcaaaatcggacatgagcgtttcttctcatccggctcaagta**

**Fortunella_japonica_8** **atgggatct-cccccccatcaaaatcggacatgagcgtttcttctcatccggctcaagta**

**Fortunella_japonica_9** **atgggatct-cccccccatcaaaatcggacatgagcgtttcttctcatccggctcaagta**

**Fortunella_japonica_10** **atgggatct-cccccccatcaaaatcggacatgagcgtttcttctcatccggctcaagta**

**Fortunella_japonica_11** **atgggatct-cccccccatcaaaatcggacatgagcgtttcttctcatccggctcaagta**

**Fortunella_japonica_12** **atgggatct-cccccccatcaaaatcggacatgagcgtttcttctcatccggctcaagta**

**Fortunella_margarita_1** **atgggatct-cccccccatcaaaatcggacatgagcgtttcttctcatccggctcaagta**

**Fortunella_margarita_2** **atgggatct-cccccccatcaaaatcggacatgagcgtttcttctcatccggctcaagta**

**Fortunella_venosa_1** **atgggatct-cccccccatcaaaatcggacatgagcgtttcttctcatccggctcaagta**

**Fortunella_venosa_2** **atgggatct-cccccccatcaaaatcggacatgagcgtttcttctcatccggctcaagta**

**Fortunella_venosa_3** **atgggatct-cccccccatcaaaatcggacatgagcgtttcttctcatccggctcaagta**

24730	24740	24750	24760	24770	24780

....|....|....|....|....|....|....|....|....|....|....|....|

**Atalantia_kwangtungens** **gttacgccaaataaggataaagaaagggattctcgctttcaaattctataaaaaacccca**

**Citrus_reticulata_1** **gttacgccaaataaggataaagaaagggattctcgctttcaaattctataaaaaacccca**

**Citrus_reticulata_2** **gttacgccaaataaggataaagaaagggattctcgctttcaaattctataaaaaacccca**

**Citrus_trifoliata_3** **gttacgccaaataaggataaagaaagggattctcgctttcaaattctataaaaaacccca**

**Citrus_aurantiifolia** **gttacgccaaataaggataaagaaagggattctcgctttcaaattctataaaaaacccca**

**Citrus_aurantium** **gttacgccaaataaggataaagaaagggattctcgctttcaaattctataaaaaacccca**

**Citrus_cavaleriei** **gttacgccaaataaggataaagaaagggattctcgctttcaaattctataaaaaacccca**

**Citrus_hongheensis** **gttacgccaaataaggataaagaaagggattctcgctttcaaattctataaaaaacccca**

**Citrus_limon** **gttacgccaaataaggataaagaaagggattctcgctttcaaattctataaaaaacccca**

**Citrus_maxima** **gttacgccaaataaggataaagaaagggattctcgctttcaaattctataaaaaacccca**

**Citrus_medica** **gttacgccaaataaggataaagaaagggattctcgctttcaaattctataaaaaacccca**

**Citrus_polytrifolia** **gttacgccaaataaggataaagaaagggattctcgctttcaaattctataaaaaacccca**

**Citrus_reticulata** **gttacgccaaataaggataaagaaagggattctcgctttcaaattctataaaaaacccca**

**Citrus_sinensis** **gttacgccaaataaggataaagaaagggattctcgctttcaaattctataaaaaacccca**

**Fortunella_bawangica_1** **gttacgccaaataaggataaagaaagggattctcgctttcaaattctataaaaaacccca**

**Fortunella_bawangica_2** **gttacgccaaataaggataaagaaagggattctcgctttcaaattctataaaaaacccca**

**Fortunella_hindsii_1** **gttacgccaaataaggataaagaaagggattctcgctttcaaattctataaaaaacccca**

**Fortunella_hindsii_2** **gttacgccaaataaggataaagaaagggattctcgctttcaaattctataaaaaacccca**

**Fortunella_hindsii_3** **gttacgccaaataaggataaagaaagggattctcgctttcaaattctataaaaaacccca**

**Fortunella_japonica_1** **gttacgccaaataaggataaagaaagggattctcgctttcaaattctataaaaaacccca**

**Fortunella_japonica_2** **gttacgccaaataaggataaagaaagggattctcgctttcaaattctataaaaaacccca**

**Fortunella_japonica_3** **gttacgccaaataaggataaagaaagggattctcgctttcaaattctataaaaaacccca**

**Fortunella_japonica_4** **gttacgccaaataaggataaagaaagggattctcgctttcaaattctataaaaaacccca**

**Fortunella_japonica_5** **gttacgccaaataaggataaagaaagggattctcgctttcaaattctataaaaaacccca**

**Fortunella_japonica_6** **gttacgccaaataaggataaagaaagggattctcgctttcaaattctataaaaaacccca**

**Fortunella_japonica_7** **gttacgccaaataaggataaagaaagggattctcgctttcaaattctataaaaaacccca**

**Fortunella_japonica_8** **gttacgccaaataaggataaagaaagggattctcgctttcaaattctataaaaaacccca**

**Fortunella_japonica_9** **gttacgccaaataaggataaagaaagggattctcgctttcaaattctataaaaaacccca**

**Fortunella_japonica_10** **gttacgccaaataaggataaagaaagggattctcgctttcaaattctataaaaaacccca**

**Fortunella_japonica_11** **gttacgccaaataaggataaagaaagggattctcgctttcaaattctataaaaaacccca**

**Fortunella_japonica_12** **gttacgccaaataaggataaagaaagggattctcgctttcaaattctataaaaaacccca**

**Fortunella_margarita_1** **gttacgccaaataaggataaagaaagggattctcgctttcaaattctataaaaaacccca**

**Fortunella_margarita_2** **gttacgccaaataaggataaagaaagggattctcgctttcaaattctataaaaaacccca**

**Fortunella_venosa_1** **gttacgccaaataaggataaagaaagggattctcgctttcaaattctataaaaaacccca**

**Fortunella_venosa_2** **gttacgccaaataaggataaagaaagggattctcgctttcaaattctataaaaaacccca**

**Fortunella_venosa_3** **gttacgccaaataaggataaagaaagggattctcgctttcaaattctataaaaaacccca**

24790	24800	24810	24820	24830	24840

....|....|....|....|....|....|....|....|....|....|....|....|

**Atalantia_kwangtungens** **ga-ccaaaacaactactccttactcaagttcccagtaaagaccaagcaacatttcattga**

**Citrus_reticulata_1** **tacccaaaacaactactccttactcaagttcccagtaaagaccaagcaacatttcattga**

**Citrus_reticulata_2** **tacccaaaacaactactccttactcaagttcccagtaaagaccaagcaacatttcattga**

**Citrus_trifoliata_3** **tacccaaaacaactactccttactcaagttcccagtaaagaccaagcaacatttcattga**

**Citrus_aurantiifolia** **tacccaaaacaactactccttactcaagttcccagtaaagaccaagcaacatttcattga**

**Citrus_aurantium** **tacccaaaacaactactccttactcaagttcccagtaaagaccaagcaacatttcattga**

**Citrus_cavaleriei** **tacccaaaacaactactccttactcaagttcccagtaaagaccaagcaacatttcattga**

**Citrus_hongheensis** **tacccaaaacaactactccttactcaagttcccagtaaagaccaagcaacatttcattga**

**Citrus_limon** **tacccaaaacaactactccttactcaagttcccagtaaagaccaagcaacatttcattga**

**Citrus_maxima** **tacccaaaacaactactccttactcaagttcccagtaaagaccaagcaacatttcattga**

**Citrus_medica** **tacccaaaacaactactccttactcaagttcccagtaaagaccaagcaacatttcattga**

**Citrus_polytrifolia** **tacccaaaacaactactccttactcaagttcccagtaaagaccaagcaacatttcattga**

**Citrus_reticulata** **tacccaaaacaactactccttactcaagttcccagtaaagaccaagcaacatttcattga**

**Citrus_sinensis** **tacccaaaacaactactccttactcaagttcccagtaaagaccaagcaacatttcattga**

**Fortunella_bawangica_1** **tacccaaaacaactactccttactcaagttcccagtaaagaccaagcaacatttcattga**

**Fortunella_bawangica_2** **tacccaaaacaactactccttactcaagttcccagtaaagaccaagcaacatttcattga**

**Fortunella_hindsii_1** **tacccaaaacaactactccttactcaagttcccagtaaagaccaagcaacatttcattga**

**Fortunella_hindsii_2** **tacccaaaacaactactccttactcaagttcccagtaaagaccaagcaacatttcattga**

**Fortunella_hindsii_3** **tacccaaaacaactactccttactcaagttcccagtaaagaccaagcaacatttcattga**

**Fortunella_japonica_1** **tacccaaaacaactactccttactcaagttcccagtaaagaccaagcaacatttcattga**

**Fortunella_japonica_2** **tacccaaaacaactactccttactcaagttcccagtaaagaccaagcaacatttcattga**

**Fortunella_japonica_3** **tacccaaaacaactactccttactcaagttcccagtaaagaccaagcaacatttcattga**

**Fortunella_japonica_4** **tacccaaaacaactactccttactcaagttcccagtaaagaccaagcaacatttcattga**

**Fortunella_japonica_5** **tacccaaaacaactactccttactcaagttcccagtaaagaccaagcaacatttcattga**

**Fortunella_japonica_6** **tacccaaaacaactactccttactcaagttcccagtaaagaccaagcaacatttcattga**

**Fortunella_japonica_7** **tacccaaaacaactactccttactcaagttcccagtaaagaccaagcaacatttcattga**

**Fortunella_japonica_8** **tacccaaaacaactactccttactcaagttcccagtaaagaccaagcaacatttcattga**

**Fortunella_japonica_9** **tacccaaaacaactactccttactcaagttcccagtaaagaccaagcaacatttcattga**

**Fortunella_japonica_10** **tacccaaaacaactactccttactcaagttcccagtaaagaccaagcaacatttcattga**

**Fortunella_japonica_11** **tacccaaaacaactactccttactcaagttcccagtaaagaccaagcaacatttcattga**

**Fortunella_japonica_12** **tacccaaaacaactactccttactcaagttcccagtaaagaccaagcaacatttcattga**

**Fortunella_margarita_1** **tacccaaaacaactactccttactcaagttcccagtaaagaccaagcaacatttcattga**

**Fortunella_margarita_2** **tacccaaaacaactactccttactcaagttcccagtaaagaccaagcaacatttcattga**

**Fortunella_venosa_1** **tacccaaaacaactactccttactcaagttcccagtaaagaccaagcaacatttcattga**

**Fortunella_venosa_2** **tacccaaaacaactactccttactcaagttcccagtaaagaccaagcaacatttcattga**

**Fortunella_venosa_3** **tacccaaaacaactactccttactcaagttcccagtaaagaccaagcaacatttcattga**

24850	24860	24870	24880	24890	24900

....|....|....|....|....|....|....|....|....|....|....|....|

**Atalantia_kwangtungens** **ttcattcttcttttccttttattgagattttcgaaattctttattcaattataaattacg**

**Citrus_reticulata_1** **ttcattcttcttttccttttattgagattttcgaaattctttattcaattataaattacg**

**Citrus_reticulata_2** **ttcattcttcttttccttttattgagattttcgaaattctttattcaattataaattacg**

**Citrus_trifoliata_3** **ttcattcttcttttccttttattgagattttcgaaattctttattcaattataaattacg**

**Citrus_aurantiifolia** **ttcattcttcttttccttttattgagattttcgaaattctttattcaattataaattacg**

**Citrus_aurantium** **ttcattcttcttttccttttattgagattttcgaaattctttattcaattataaattacg**

**Citrus_cavaleriei** **ttcattcttcttttccttttattgagattttcgaaattctttattcaattataaattacg**

**Citrus_hongheensis** **ttcattcttcttttccttttattgagattttcgaaattctttattcaattataaattacg**

**Citrus_limon** **ttcattcttcttttccttttattgagattttcgaaattctttattcaattataaattacg**

**Citrus_maxima** **ttcattcttcttttccttttattgagattttcgaaattctttattcaattataaattacg**

**Citrus_medica** **ttcattcttcttttccttttattgagattttcgaaattctttattcaattataaattacg**

**Citrus_polytrifolia** **ttcattcttcttttccttttattgagattttcgaaattctttattcaattataaattacg**

**Citrus_reticulata** **ttcattcttcttttccttttattgagattttcgaaattctttattcaattataaattacg**

**Citrus_sinensis** **ttcattcttcttttccttttattgagattttcgaaattctttattcaattataaattacg**

**Fortunella_bawangica_1** **ttcattcttcttttccttttattgagattttcgaaattctttattcaattataaattacg**

**Fortunella_bawangica_2** **ttcattcttcttttccttttattgagattttcgaaattctttattcaattataaattacg**

**Fortunella_hindsii_1** **ttcattcttcttttccttttattgagattttcgaaattctttattcaattataaattacg**

**Fortunella_hindsii_2** **ttcattcttcttttccttttattgagattttcgaaattctttattcaattataaattacg**

**Fortunella_hindsii_3** **ttcattcttcttttccttttattgagattttcgaaattctttattcaattataaattacg**

**Fortunella_japonica_1** **ttcattcttcttttccttttattgagattttcgaaattctttattcaattataaattacg**

**Fortunella_japonica_2** **ttcattcttcttttccttttattgagattttcgaaattctttattcaattataaattacg**

**Fortunella_japonica_3** **ttcattcttcttttccttttattgagattttcgaaattctttattcaattataaattacg**

**Fortunella_japonica_4** **ttcattcttcttttccttttattgagattttcgaaattctttattcaattataaattacg**

**Fortunella_japonica_5** **ttcattcttcttttccttttattgagattttcgaaattctttattcaattataaattacg**

**Fortunella_japonica_6** **ttcattcttcttttccttttattgagattttcgaaattctttattcaattataaattacg**

**Fortunella_japonica_7** **ttcattcttcttttccttttattgagattttcgaaattctttattcaattataaattacg**

**Fortunella_japonica_8** **ttcattcttcttttccttttattgagattttcgaaattctttattcaattataaattacg**

**Fortunella_japonica_9** **ttcattcttcttttccttttattgagattttcgaaattctttattcaattataaattacg**

**Fortunella_japonica_10** **ttcattcttcttttccttttattgagattttcgaaattctttattcaattataaattacg**

**Fortunella_japonica_11** **ttcattcttcttttccttttattgagattttcgaaattctttattcaattataaattacg**

**Fortunella_japonica_12** **ttcattcttcttttccttttattgagattttcgaaattctttattcaattataaattacg**

**Fortunella_margarita_1** **ttcattcttcttttccttttattgagattttcgaaattctttattcaattataaattacg**

**Fortunella_margarita_2** **ttcattcttcttttccttttattgagattttcgaaattctttattcaattataaattacg**

**Fortunella_venosa_1** **ttcattcttcttttccttttattgagattttcgaaattctttattcaattataaattacg**

**Fortunella_venosa_2** **ttcattcttcttttccttttattgagattttcgaaattctttattcaattataaattacg**

**Fortunella_venosa_3** **ttcattcttcttttccttttattgagattttcgaaattctttattcaattataaattacg**

24910	24920	24930	24940	24950	24960

....|....|....|....|....|....|....|....|....|....|....|....|

**Atalantia_kwangtungens** **aaactgaaatgtgaaattcttgagtagtctacttccccccgaatgacaaatccccttacc**

**Citrus_reticulata_1** **aaactgaaatgtgaaattcttgagtagtctacttccccccgaatgacaaatccccttacc**

**Citrus_reticulata_2** **aaactgaaatgtgaaattcttgagtagtctacttccccccgaatgacaaatccccttacc**

**Citrus_trifoliata_3** **aaactgaaatgtgaaattcttgagtagtctacttccccccgaatgacaaatccccttacc**

**Citrus_aurantiifolia** **aaactgaaatgtgaaattcttgagtagtctacttccccccgaatgacaaatccccttacc**

**Citrus_aurantium** **aaactgaaatgtgaaattcttgagtagtctacttccccccgaatgacaaatccccttacc**

**Citrus_cavaleriei** **aaactgaaatgtgaaattcttgagtagtctacttccccccgaatgacaaatccccttacc**

**Citrus_hongheensis** **aaactgaaatgtgaaattcttgagtagtctacttccccccgaatgacaaatccccttacc**

**Citrus_limon** **aaactgaaatgtgaaattcttgagtagtctacttccccccgaatgacaaatccccttacc**

**Citrus_maxima** **aaactgaaatgtgaaattcttgagtagtctacttccccccgaatgacaaatccccttacc**

**Citrus_medica** **aaactgaaatgtgaaattcttgagtagtctacttccccccgaatgacaaatccccttacc**

**Citrus_polytrifolia** **aaactgaaatgtgaaattcttgagtagtctacttccccccgaatgacaaatccccttacc**

**Citrus_reticulata** **aaactgaaatgtgaaattcttgagtagtctacttccccccgaatgacaaatccccttacc**

**Citrus_sinensis** **aaactgaaatgtgaaattcttgagtagtctacttccccccgaatgacaaatccccttacc**

**Fortunella_bawangica_1** **aaactgaaatgtgaaattcttgagtagtctacttccccccgaatgacaaatccccttacc**

**Fortunella_bawangica_2** **aaactgaaatgtgaaattcttgagtagtctacttccccccgaatgacaaatccccttacc**

**Fortunella_hindsii_1** **aaactgaaatgtgaaattcttgagtagtctacttccccccgaatgacaaatccccttacc**

**Fortunella_hindsii_2** **aaactgaaatgtgaaattcttgagtagtctacttccccccgaatgacaaatccccttacc**

**Fortunella_hindsii_3** **aaactgaaatgtgaaattcttgagtagtctacttccccccgaatgacaaatccccttacc**

**Fortunella_japonica_1** **aaactgaaatgtgaaattcttgagtagtctacttccccccgaatgacaaatccccttacc**

**Fortunella_japonica_2** **aaactgaaatgtgaaattcttgagtagtctacttccccccgaatgacaaatccccttacc**

**Fortunella_japonica_3** **aaactgaaatgtgaaattcttgagtagtctacttccccccgaatgacaaatccccttacc**

**Fortunella_japonica_4** **aaactgaaatgtgaaattcttgagtagtctacttccccccgaatgacaaatccccttacc**

**Fortunella_japonica_5** **aaactgaaatgtgaaattcttgagtagtctacttccccccgaatgacaaatccccttacc**

**Fortunella_japonica_6** **aaactgaaatgtgaaattcttgagtagtctacttccccccgaatgacaaatccccttacc**

**Fortunella_japonica_7** **aaactgaaatgtgaaattcttgagtagtctacttccccccgaatgacaaatccccttacc**

**Fortunella_japonica_8** **aaactgaaatgtgaaattcttgagtagtctacttccccccgaatgacaaatccccttacc**

**Fortunella_japonica_9** **aaactgaaatgtgaaattcttgagtagtctacttccccccgaatgacaaatccccttacc**

**Fortunella_japonica_10** **aaactgaaatgtgaaattcttgagtagtctacttccccccgaatgacaaatccccttacc**

**Fortunella_japonica_11** **aaactgaaatgtgaaattcttgagtagtctacttccccccgaatgacaaatccccttacc**

**Fortunella_japonica_12** **aaactgaaatgtgaaattcttgagtagtctacttccccccgaatgacaaatccccttacc**

**Fortunella_margarita_1** **aaactgaaatgtgaaattcttgagtagtctacttccccccgaatgacaaatccccttacc**

**Fortunella_margarita_2** **aaactgaaatgtgaaattcttgagtagtctacttccccccgaatgacaaatccccttacc**

**Fortunella_venosa_1** **aaactgaaatgtgaaattcttgagtagtctacttccccccgaatgacaaatccccttacc**

**Fortunella_venosa_2** **aaactgaaatgtgaaattcttgagtagtctacttccccccgaatgacaaatccccttacc**

**Fortunella_venosa_3** **aaactgaaatgtgaaattcttgagtagtctacttccccccgaatgacaaatccccttacc**

24970	24980	24990	25000	25010	25020

....|....|....|....|....|....|....|....|....|....|....|....|

**Atalantia_kwangtungens** **ttaaattatt----aaattaaataaattaaaggaaagaagtgccttggaattcataaggg**

**Citrus_reticulata_1** **tt------------aaattaaataaattaaaggaaagaagtgccttggaattcataaggg**

**Citrus_reticulata_2** **tt------------aaattaaataaattaaaggaaagaagtgccttggaattcataaggg**

**Citrus_trifoliata_3** **tt------------aaattaaataaattaaaggaaagaagtgccttggaattcataaggg**

**Citrus_aurantiifolia** **tt------------aaattaaataaattaaaggaaagaagtgccttggaattcataaggg**

**Citrus_aurantium** **tt------------aaattaaataaattaaaggaaagaagtgccttggaattcataaggg**

**Citrus_cavaleriei** **tt------------aaattaaataaattaaaggaaagaagtgccttggaattcataaggg**

**Citrus_hongheensis** **ttaaattaaataaaaaattaaataaattaaaggaaagaagtgccttggaattcataaggg**

**Citrus_limon** **tt------------aaattaaataaattaaaggaaagaagtgccttggaattcataaggg**

**Citrus_maxima** **tt------------aaattaaataaattaaaggaaagaagtgccttggaattcataaggg**

**Citrus_medica** **ttaaattatt----aaatttaaaaaattaaaggaaagaagtgccttggaattcataaggg**

**Citrus_polytrifolia** **tt------------aaattaaataaattaaaggaaagaagtgccttggaattcataaggg**

**Citrus_reticulata** **tt------------aaattaaataaattaaaggaaagaagtgccttggaattcataaggg**

**Citrus_sinensis** **tt------------aaattaaataaattaaaggaaagaagtgccttggaattcataaggg**

**Fortunella_bawangica_1** **tt------------aaattaaagaaataaaaggaaagaagtgccttggaattcataaggg**

**Fortunella_bawangica_2** **tt------------aaattaaagaaataaaaggaaagaagtgccttggaattcataaggg**

**Fortunella_hindsii_1** **tt------------aaattaaagaaataaaaggaaagaagtgccttggaattcataaggg**

**Fortunella_hindsii_2** **tt------------aaattaaagaaataaaaggaaagaagtgccttggaattcataaggg**

**Fortunella_hindsii_3** **tt------------aaattaaagaaataaaaggaaagaagtgccttggaattcataaggg**

**Fortunella_japonica_1** **tt------------aaattaaagaaataaaaggaaagaagtgccttggaattcataaggg**

**Fortunella_japonica_2** **tt------------aaattaaagaaataaaaggaaagaagtgccttggaattcataaggg**

**Fortunella_japonica_3** **tt------------aaattaaagaaataaaaggaaagaagtgccttggaattcataaggg**

**Fortunella_japonica_4** **tt------------aaattaaagaaataaaaggaaagaagtgccttggaattcataaggg**

**Fortunella_japonica_5** **tt------------aaattaaagaaataaaaggaaagaagtgccttggaattcataaggg**

**Fortunella_japonica_6** **tt------------aaattaaagaaataaaaggaaagaagtgccttggaattcataaggg**

**Fortunella_japonica_7** **tt------------aaattaaagaaataaaaggaaagaagtgccttggaattcataaggg**

**Fortunella_japonica_8** **tt------------aaattaaagaaataaaaggaaagaagtgccttggaattcataaggg**

**Fortunella_japonica_9** **tt------------aaattaaagaaataaaaggaaagaagtgccttggaattcataaggg**

**Fortunella_japonica_10** **tt------------aaattaaagaaataaaaggaaagaagtgccttggaattcataaggg**

**Fortunella_japonica_11** **tt------------aaattaaagaaataaaaggaaagaagtgccttggaattcataaggg**

**Fortunella_japonica_12** **tt------------aaattaaagaaataaaaggaaagaagtgccttggaattcataaggg**

**Fortunella_margarita_1** **tt------------aaattaaagaaataaaaggaaagaagtgccttggaattcataaggg**

**Fortunella_margarita_2** **tt------------aaattaaagaaataaaaggaaagaagtgccttggaattcataaggg**

**Fortunella_venosa_1** **tt------------aaattaaagaaataaaaggaaagaagtgccttggaattcataaggg**

**Fortunella_venosa_2** **tt------------aaattaaagaaataaaaggaaagaagtgccttggaattcataaggg**

**Fortunella_venosa_3** **tt------------aaattaaagaaataaaaggaaagaagtgccttggaattcataaggg**

25030	25040	25050	25060	25070	25080

....|....|....|....|....|....|....|....|....|....|....|....|

**Atalantia_kwangtungens** **atttttttgtctatgtatcgttcctttcgatcttttaggtcctgcttcacctcgacggtt**

**Citrus_reticulata_1** **atttttttgtctatgtatcgttcctttcgatcttttaggtcctgcttcacctcgacggtt**

**Citrus_reticulata_2** **atttttttgtctatgtatcgttcctttcgatcttttaggtcctgcttcacctcgacggtt**

**Citrus_trifoliata_3** **atttttttgtctatgtatcgttcctttcgatcttttaggtcctgcttcacctcgacggtt**

**Citrus_aurantiifolia** **atttttttgtctatgtatcgttcctttcgatcttttaggtcctgcttcacctcgacggtt**

**Citrus_aurantium** **atttttttgtctatgtatcgttcctttcgatcttttaggtcctgcttcacctcgacggtt**

**Citrus_cavaleriei** **atttttttgtctatgtatcgttcctttcgatcttttaggtcctgcttcacctcgacggtt**

**Citrus_hongheensis** **atttttttgtctatgtatcgttcctttcgatcttttaggtcctgcttcacctcgacggtt**

**Citrus_limon** **atttttttgtctatgtatcgttcctttcgatcttttaggtcctgcttcacctcgacggtt**

**Citrus_maxima** **atttttttgtctatgtatcgttcctttcgatcttttaggtcctgcttcacctcgacggtt**

**Citrus_medica** **atttttttgtctatgtatcgttcctttcgatcttttaggtcctgcttcacctcgacggtt**

**Citrus_polytrifolia** **atttttttgtctatgtatcgttcctttcgatcttttaggtcctgcttcacctcgacggtt**

**Citrus_reticulata** **atttttttgtctatgtatcgttcctttcgatcttttaggtcctgcttcacctcgacggtt**

**Citrus_sinensis** **atttttttgtctatgtatcgttcctttcgatcttttaggtcctgcttcacctcgacggtt**

**Fortunella_bawangica_1** **atttttttgtctatgtatcgttcctttcgatcttttaggtcctgcttcacctcgacggtt**

**Fortunella_bawangica_2** **atttttttgtctatgtatcgttcctttcgatcttttaggtcctgcttcacctcgacggtt**

**Fortunella_hindsii_1** **atttttttgtctatgtatcgttcctttcgatcttttaggtcctgcttcacctcgacggtt**

**Fortunella_hindsii_2** **atttttttgtctatgtatcgttcctttcgatcttttaggtcctgcttcacctcgacggtt**

**Fortunella_hindsii_3** **atttttttgtctatgtatcgttcctttcgatcttttaggtcctgcttcacctcgacggtt**

**Fortunella_japonica_1** **atttttttgtctatgtatcgttcctttcgatcttttaggtcctgcttcacctcgacggtt**

**Fortunella_japonica_2** **atttttttgtctatgtatcgttcctttcgatcttttaggtcctgcttcacctcgacggtt**

**Fortunella_japonica_3** **atttttttgtctatgtatcgttcctttcgatcttttaggtcctgcttcacctcgacggtt**

**Fortunella_japonica_4** **atttttttgtctatgtatcgttcctttcgatcttttaggtcctgcttcacctcgacggtt**

**Fortunella_japonica_5** **atttttttgtctatgtatcgttcctttcgatcttttaggtcctgcttcacctcgacggtt**

**Fortunella_japonica_6** **atttttttgtctatgtatcgttcctttcgatcttttaggtcctgcttcacctcgacggtt**

**Fortunella_japonica_7** **atttttttgtctatgtatcgttcctttcgatcttttaggtcctgcttcacctcgacggtt**

**Fortunella_japonica_8** **atttttttgtctatgtatcgttcctttcgatcttttaggtcctgcttcacctcgacggtt**

**Fortunella_japonica_9** **atttttttgtctatgtatcgttcctttcgatcttttaggtcctgcttcacctcgacggtt**

**Fortunella_japonica_10** **atttttttgtctatgtatcgttcctttcgatcttttaggtcctgcttcacctcgacggtt**

**Fortunella_japonica_11** **atttttttgtctatgtatcgttcctttcgatcttttaggtcctgcttcacctcgacggtt**

**Fortunella_japonica_12** **atttttttgtctatgtatcgttcctttcgatcttttaggtcctgcttcacctcgacggtt**

**Fortunella_margarita_1** **atttttttgtctatgtatcgttcctttcgatcttttaggtcctgcttcacctcgacggtt**

**Fortunella_margarita_2** **atttttttgtctatgtatcgttcctttcgatcttttaggtcctgcttcacctcgacggtt**

**Fortunella_venosa_1** **atttttttgtctatgtatcgttcctttcgatcttttaggtcctgcttcacctcgacggtt**

**Fortunella_venosa_2** **atttttttgtctatgtatcgttcctttcgatcttttaggtcctgcttcacctcgacggtt**

**Fortunella_venosa_3** **atttttttgtctatgtatcgttcctttcgatcttttaggtcctgcttcacctcgacggtt**

25090	25100	25110	25120	25130	25140

....|....|....|....|....|....|....|....|....|....|....|....|

**Atalantia_kwangtungens** **atgccacgatatacttaaagcctatatgcgatgtatagactcccccaaccatgacatatt**

**Citrus_reticulata_1** **atgccacgatatacttaaagcctatatgcgatgtatagactcccccaaccatgacatatt**

**Citrus_reticulata_2** **atgccacgatatacttaaagcctatatgcgatgtatagactcccccaaccatgacatatt**

**Citrus_trifoliata_3** **atgccacgatatacttaaagcctatatgcgatgtatagactcccccaaccatgacatatt**

**Citrus_aurantiifolia** **atgccacgatatacttaaagcctatatgcgatgtatagactcccccaaccatgacatatt**

**Citrus_aurantium** **atgccacgatatacttaaagcctatatgcgatgtatagactcccccaaccatgacatatt**

**Citrus_cavaleriei** **atgccacgatatacttaaagcctatatgcgatgtatagactcccccaaccatgacatatt**

**Citrus_hongheensis** **atgccacgatatacttaaagcctatatgcgatgtatagactcccccaaccatgacatatt**

**Citrus_limon** **atgccacgatatacttaaagcctatatgcgatgtatagactcccccaaccatgacatatt**

**Citrus_maxima** **atgccacgatatacttaaagcctatatgcgatgtatagactcccccaaccatgacatatt**

**Citrus_medica** **atgccacgatatacttaaagcctatatgcgatgtatagactcccccaaccatgacatatt**

**Citrus_polytrifolia** **atgccacgatatacttaaagcctatatgcgatgtatagactcccccaaccatgacatatt**

**Citrus_reticulata** **atgccacgatatacttaaagcctatatgcgatgtatagactcccccaaccatgacatatt**

**Citrus_sinensis** **atgccacgatatacttaaagcctatatgcgatgtatagactcccccaaccatgacatatt**

**Fortunella_bawangica_1** **atgccacgatatacttaaagcctatatgcgatgtatagactcccccaaccatgacatatt**

**Fortunella_bawangica_2** **atgccacgatatacttaaagcctatatgcgatgtatagactcccccaaccatgacatatt**

**Fortunella_hindsii_1** **atgccacgatatacttaaagcctatatgcgatgtatagactcccccaaccatgacatatt**

**Fortunella_hindsii_2** **atgccacgatatacttaaagcctatatgcgatgtatagactcccccaaccatgacatatt**

**Fortunella_hindsii_3** **atgccacgatatacttaaagcctatatgcgatgtatagactcccccaaccatgacatatt**

**Fortunella_japonica_1** **atgccacgatatacttaaagcctatatgcgatgtatagactcccccaaccatgacatatt**

**Fortunella_japonica_2** **atgccacgatatacttaaagcctatatgcgatgtatagactcccccaaccatgacatatt**

**Fortunella_japonica_3** **atgccacgatatacttaaagcctatatgcgatgtatagactcccccaaccatgacatatt**

**Fortunella_japonica_4** **atgccacgatatacttaaagcctatatgcgatgtatagactcccccaaccatgacatatt**

**Fortunella_japonica_5** **atgccacgatatacttaaagcctatatgcgatgtatagactcccccaaccatgacatatt**

**Fortunella_japonica_6** **atgccacgatatacttaaagcctatatgcgatgtatagactcccccaaccatgacatatt**

**Fortunella_japonica_7** **atgccacgatatacttaaagcctatatgcgatgtatagactcccccaaccatgacatatt**

**Fortunella_japonica_8** **atgccacgatatacttaaagcctatatgcgatgtatagactcccccaaccatgacatatt**

**Fortunella_japonica_9** **atgccacgatatacttaaagcctatatgcgatgtatagactcccccaaccatgacatatt**

**Fortunella_japonica_10** **atgccacgatatacttaaagcctatatgcgatgtatagactcccccaaccatgacatatt**

**Fortunella_japonica_11** **atgccacgatatacttaaagcctatatgcgatgtatagactcccccaaccatgacatatt**

**Fortunella_japonica_12** **atgccacgatatacttaaagcctatatgcgatgtatagactcccccaaccatgacatatt**

**Fortunella_margarita_1** **atgccacgatatacttaaagcctatatgcgatgtatagactcccccaaccatgacatatt**

**Fortunella_margarita_2** **atgccacgatatacttaaagcctatatgcgatgtatagactcccccaaccatgacatatt**

**Fortunella_venosa_1** **atgccacgatatacttaaagcctatatgcgatgtatagactcccccaaccatgacatatt**

**Fortunella_venosa_2** **atgccacgatatacttaaagcctatatgcgatgtatagactcccccaaccatgacatatt**

**Fortunella_venosa_3** **atgccacgatatacttaaagcctatatgcgatgtatagactcccccaaccatgacatatt**

25150	25160	25170	25180	25190	25200

....|....|....|....|....|....|....|....|....|....|....|....|

**Atalantia_kwangtungens** **tgcttacttgag-gaacagaatttcattttttttttttcacgaggtacaactagaaattc**

**Citrus_reticulata_1** **tgcttacttgaggaaacagaatttcat---------------------------------**

**Citrus_reticulata_2** **tgcttacttgaggaaacagaatttcat---------------------------------**

**Citrus_trifoliata_3** **tgcttacttgag-gaacagaatttcat---------------------------------**

**Citrus_aurantiifolia** **tgcttacttgag-gaacagaatttcatttttttt--------------------------**

**Citrus_aurantium** **tgcttacttgag-gaacagaatttca----------------------------------**

**Citrus_cavaleriei** **tgc----------gaacagaatttca----------------------------------**

**Citrus_hongheensis** **tgcttacttgag-gaacagaatttca----------------------------------**

**Citrus_limon** **tgcttacttgag-gaacagaatttca----------------------------------**

**Citrus_maxima** **tgcttacttgag-gaacagaatttca----------------------------------**

**Citrus_medica** **tgcttac------taacagaatttca----------------------------------**

**Citrus_polytrifolia** **tgcttacttgag-gaacagaatttca----------------------------------**

**Citrus_reticulata** **tgcttacttgaggaaacagaatttcat---------------------------------**

**Citrus_sinensis** **tgcttacttgag-gaacagaatttca----------------------------------**

**Fortunella_bawangica_1** **tgcttacttgag-gaacagaatttcatt--------------------------------**

**Fortunella_bawangica_2** **tgcttacttgag-gaacagaatttcatt--------------------------------**

**Fortunella_hindsii_1** **tgcttacttgag-gaacagaatttcatt--------------------------------**

**Fortunella_hindsii_2** **tgcttacttgag-gaacagaatttcatt--------------------------------**

**Fortunella_hindsii_3** **tgcttacttgag-gaacagaatttcatt--------------------------------**

**Fortunella_japonica_1** **tgcttacttgag-gaacagaatttcat---------------------------------**

**Fortunella_japonica_2** **tgcttacttgag-gaacagaatttcat---------------------------------**

**Fortunella_japonica_3** **tgcttacttgag-gaacagaatttcat---------------------------------**

**Fortunella_japonica_4** **tgcttacttgag-gaacagaatttcat---------------------------------**

**Fortunella_japonica_5** **tgcttacttgag-gaacagaatttcat---------------------------------**

**Fortunella_japonica_6** **tgcttacttgag-gaacagaatttcat---------------------------------**

**Fortunella_japonica_7** **tgcttacttgag-gaacagaatttcat---------------------------------**

**Fortunella_japonica_8** **tgcttacttgag-gaacagaatttcat---------------------------------**

**Fortunella_japonica_9** **tgcttacttgag-gaacagaatttcat---------------------------------**

**Fortunella_japonica_10** **tgcttacttgag-gaacagaatttca-t--------------------------------**

**Fortunella_japonica_11** **tgcttacttgag-gaacagaatttcat---------------------------------**

**Fortunella_japonica_12** **tgcttacttgag-gaacagaatttcat---------------------------------**

**Fortunella_margarita_1** **tgcttacttgag-gaacagaatttcatt--------------------------------**

**Fortunella_margarita_2** **tgcttacttgag-gaacagaatttcatt--------------------------------**

**Fortunella_venosa_1** **tgcttacttgag-gaacagaatttcat---------------------------------**

**Fortunella_venosa_2** **tgcttacttgag-gaacagaatttcat---------------------------------**

**Fortunella_venosa_3** **tgcttacttgag-gaacagaatttcat---------------------------------**

25210	25220	25230	25240	25250	25260

....|....|....|....|....|....|....|....|....|....|....|....|

**Atalantia_kwangtungens** **ttatttatttttgactgaacagaatttcattttttttttttcacgaggtacaactagaaa**

**Citrus_reticulata_1** **----------------------------tttttttttttttcacgaggtacaactagaaa**

**Citrus_reticulata_2** **----------------------------tttttttttttttcacgaggtacaactagaaa**

**Citrus_trifoliata_3** **-----------------------------ttttttttttttcacgaggtacaactagaaa**

**Citrus_aurantiifolia** **--------------------------tttttttttttttttcacgaggtacaactagaaa**

**Citrus_aurantium** **------------------------------tttttttttttcacgaggtacaactagaaa**

**Citrus_cavaleriei** **------------------------------tttttttttttcacgaggtacaactagaaa**

**Citrus_hongheensis** **------------------------------tttttttttttcacgaggtacaactagaaa**

**Citrus_limon** **------------------------------tttttttttttcacgaggtacaactagaaa**

**Citrus_maxima** **------------------------------tttttttttttcacgaggtacaactagaaa**

**Citrus_medica** **------------------------------tttttttttttcacgaggtacaactagaaa**

**Citrus_polytrifolia** **------------------------------tttttttttttcacgaggtacaactagaaa**

**Citrus_reticulata** **----------------------------tttttttttttttcacgaggtacaactagaaa**

**Citrus_sinensis** **------------------------------tttttttttttcacgaggtacaactagaaa**

**Fortunella_bawangica_1** **---------------------------ttttttttttttttcacgaggtacaactagaaa**

**Fortunella_bawangica_2** **---------------------------ttttttttttttttcacgaggtacaactagaaa**

**Fortunella_hindsii_1** **---------------------------ttttttttttttttcacgaggtacaactagaaa**

**Fortunella_hindsii_2** **---------------------------ttttttttttttttcacgaggtacaactagaaa**

**Fortunella_hindsii_3** **---------------------------ttttttttttttttcacgaggtacaactagaaa**

**Fortunella_japonica_1** **----------------------------tttttttttttttcacgaggtacaactagaaa**

**Fortunella_japonica_2** **----------------------------tttttttttttttcacgaggtacaactagaaa**

**Fortunella_japonica_3** **----------------------------tttttttttttttcacgaggtacaactagaaa**

**Fortunella_japonica_4** **----------------------------tttttttttttttcacgaggtacaactagaaa**

**Fortunella_japonica_5** **----------------------------tttttttttttttcacgaggtacaactagaaa**

**Fortunella_japonica_6** **----------------------------tttttttttttttcacgaggtacaactagaaa**

**Fortunella_japonica_7** **----------------------------tttttttttttttcacgaggtacaactagaaa**

**Fortunella_japonica_8** **----------------------------tttttttttttttcacgaggtacaactagaaa**

**Fortunella_japonica_9** **----------------------------tttttttttttttcacgaggtacaactagaaa**

**Fortunella_japonica_10** **---------------------------ttttttttttttttcacgaggtacaactagaaa**

**Fortunella_japonica_11** **----------------------------tttttttttttttcacgaggtacaactagaaa**

**Fortunella_japonica_12** **----------------------------tttttttttttttcacgaggtacaactagaaa**

**Fortunella_margarita_1** **---------------------------ttttttttttttttcacgaggtacaactagaaa**

**Fortunella_margarita_2** **---------------------------ttttttttttttttcacgaggtacaactagaaa**

**Fortunella_venosa_1** **----------------------------tttttttttttttcacgaggtacaactagaaa**

**Fortunella_venosa_2** **----------------------------tttttttttttttcacgaggtacaactagaaa**

**Fortunella_venosa_3** **----------------------------tttttttttttttcacgaggtacaactagaaa**

25270	25280	25290	25300	25310	25320

....|....|....|....|....|....|....|....|....|....|....|....|

**Atalantia_kwangtungens** **ttcttatttatttttgactgaatcgaccatagaccaattcccctttttc-ttttttgggg**

**Citrus_reticulata_1** **ttcttatttattttttactgaatcgaccatagaccaattcccctttttcgtttttggggg**

**Citrus_reticulata_2** **ttcttatttattttttactgaatcgaccatagaccaattcccctttttcgtttttggggg**

**Citrus_trifoliata_3** **ttcttatttattttttactgaatcgaccatagaccaattcccctttttcgtttttggggg**

**Citrus_aurantiifolia** **ttcttatttattttttactgaatcgaccatagaccaattccccttttccgtttttggggg**

**Citrus_aurantium** **ttcttatttattttttactgaatcgaccatagaccaattcccctttttcgtttttggggg**

**Citrus_cavaleriei** **ttcttatttattttttactgaatcgaccatagaccaattcccctttttcgtttttggggg**

**Citrus_hongheensis** **ttcttatttattttttactgaatcgaccatagaccaattcccctttttcgtttttggggg**

**Citrus_limon** **ttcttatttattttttactgaatcgaccatagaccaattcccctttttcgtttttggggg**

**Citrus_maxima** **ttcttatttattttttactgaatcgaccatagaccaattcccctttttcgtttttggggg**

**Citrus_medica** **ttcttatttattttttactgaatcgaccatagaccaattcccctttttcgtttttggggg**

**Citrus_polytrifolia** **ttcttatttattttttactgaatcgaccatagaccaattcccctttttcgtttttggggg**

**Citrus_reticulata** **ttcttatttattttttactgaatcgaccatagaccaattcccctttttcgtttttggggg**

**Citrus_sinensis** **ttcttatttattttttactgaatcgaccatagaccaattcccctttttcgtttttggggg**

**Fortunella_bawangica_1** **ttcttatttattttttactgaatcgaccatagaccaattcccctttttcgtttttggggg**

**Fortunella_bawangica_2** **ttcttatttattttttactgaatcgaccatagaccaattcccctttttcgtttttggggg**

**Fortunella_hindsii_1** **ttcttatttattttttactgaatcgaccatagaccaattcccctttttcgtttttggggg**

**Fortunella_hindsii_2** **ttcttatttattttttactgaatcgaccatagaccaattcccctttttcgtttttggggg**

**Fortunella_hindsii_3** **ttcttatttattttttactgaatcgaccatagaccaattcccctttttcgtttttggggg**

**Fortunella_japonica_1** **ttcttatttattttttactgaatcgaccatagaccaattcccctttttcgtttttggggg**

**Fortunella_japonica_2** **ttcttatttattttttactgaatcgaccatagaccaattcccctttttcgtttttggggg**

**Fortunella_japonica_3** **ttcttatttattttttactgaatcgaccatagaccaattcccctttttcgtttttggggg**

**Fortunella_japonica_4** **ttcttatttattttttactgaatcgaccatagaccaattcccctttttcgtttttggggg**

**Fortunella_japonica_5** **ttcttatttattttttactgaatcgaccatagaccaattcccctttttcgtttttggggg**

**Fortunella_japonica_6** **ttcttatttattttttactgaatcgaccatagaccaattcccctttttcgtttttggggg**

**Fortunella_japonica_7** **ttcttatttattttttactgaatcgaccatagaccaattcccctttttcgtttttggggg**

**Fortunella_japonica_8** **ttcttatttattttttactgaatcgaccatagaccaattcccctttttcgtttttggggg**

**Fortunella_japonica_9** **ttcttatttattttttactgaatcgaccatagaccaattcccctttttcgtttttggggg**

**Fortunella_japonica_10** **ttcttatttattttttactgaatcgaccatagaccaattcccctttttcgtttttggggg**

**Fortunella_japonica_11** **ttcttatttattttttactgaatcgaccatagaccaattcccctttttcgtttttggggg**

**Fortunella_japonica_12** **ttcttatttattttttactgaatcgaccatagaccaattcccctttttcgtttttggggg**

**Fortunella_margarita_1** **ttcttatttattttttactgaatcgaccatagaccaattcccctttttcgtttttggggg**

**Fortunella_margarita_2** **ttcttatttattttttactgaatcgaccatagaccaattcccctttttcgtttttggggg**

**Fortunella_venosa_1** **ttcttatttattttttactgaatcgaccatagaccaattcccctttttcgtttttggggg**

**Fortunella_venosa_2** **ttcttatttattttttactgaatcgaccatagaccaattcccctttttcgtttttggggg**

**Fortunella_venosa_3** **ttcttatttattttttactgaatcgaccatagaccaattcccctttttcgtttttggggg**

25330	25340	25350	25360	25370	25380

....|....|....|....|....|....|....|....|....|....|....|....|

**Atalantia_kwangtungens** **gagtattgaatacacccatcggtctgagcttcatgtgactcctcccaagagacatgtcag**

**Citrus_reticulata_1** **gagtattgaatacacccatcggtctgagcttcatgtgactcctccaaagagacatgtcag**

**Citrus_reticulata_2** **gagtattgaatacacccatcggtctgagcttcatgtgactcctccaaagagacatgtcag**

**Citrus_trifoliata_3** **gagtattgaatacacccatcggtctgagcttcatgtgactcctccaaagagacatgtcag**

**Citrus_aurantiifolia** **gagtattgaatacacccatcggtctgagcttcatgtgactcctccaaagagacatgtcag**

**Citrus_aurantium** **gagtattgaatacacccatcggtctgagcttcatgtgactcctccaaagagacatgtcag**

**Citrus_cavaleriei** **gagtattgaatacacccatcggtctgagcttcatgtgactcctccaaagagacatgtcag**

**Citrus_hongheensis** **gagtattgaatacacccatcggtctgagcttcatgtgactcctccaaagagacatgtcag**

**Citrus_limon** **gagtattgaatacacccatcggtctgagcttcatgtgactcctccaaagagacatgtcag**

**Citrus_maxima** **gagtattgaatacacccatcggtctgagcttcatgtgactcctccaaagagacatgtcag**

**Citrus_medica** **gagtattgaatacacccatcggtctgagcttcatgtgactcctccaaagagacatgtcag**

**Citrus_polytrifolia** **gagtattgaatacacccatcggtctgagcttcatgtgactcctccaaagagacatgtcag**

**Citrus_reticulata** **gagtattgaatacacccatcggtctgagcttcatgtgactcctccaaagagacatgtcag**

**Citrus_sinensis** **gagtattgaatacacccatcggtctgagcttcatgtgactcctccaaagagacatgtcag**

**Fortunella_bawangica_1** **gagtattgaatacacccatcggtctgagcttcatgtgactcctccaaagagacatgtcag**

**Fortunella_bawangica_2** **gagtattgaatacacccatcggtctgagcttcatgtgactcctccaaagagacatgtcag**

**Fortunella_hindsii_1** **gagtattgaatacacccatcggtctgagcttcatgtgactcctccaaagagacatgtcag**

**Fortunella_hindsii_2** **gagtattgaatacacccatcggtctgagcttcatgtgactcctccaaagagacatgtcag**

**Fortunella_hindsii_3** **gagtattgaatacacccatcggtctgagcttcatgtgactcctccaaagagacatgtcag**

**Fortunella_japonica_1** **gagtattgaatacacccatcggtctgagcttcatgtgactcctccaaagagacatgtcag**

**Fortunella_japonica_2** **gagtattgaatacacccatcggtctgagcttcatgtgactcctccaaagagacatgtcag**

**Fortunella_japonica_3** **gagtattgaatacacccatcggtctgagcttcatgtgactcctccaaagagacatgtcag**

**Fortunella_japonica_4** **gagtattgaatacacccatcggtctgagcttcatgtgactcctccaaagagacatgtcag**

**Fortunella_japonica_5** **gagtattgaatacacccatcggtctgagcttcatgtgactcctccaaagagacatgtcag**

**Fortunella_japonica_6** **gagtattgaatacacccatcggtctgagcttcatgtgactcctccaaagagacatgtcag**

**Fortunella_japonica_7** **gagtattgaatacacccatcggtctgagcttcatgtgactcctccaaagagacatgtcag**

**Fortunella_japonica_8** **gagtattgaatacacccatcggtctgagcttcatgtgactcctccaaagagacatgtcag**

**Fortunella_japonica_9** **gagtattgaatacacccatcggtctgagcttcatgtgactcctccaaagagacatgtcag**

**Fortunella_japonica_10** **gagtattgaatacacccatcggtctgagcttcatgtgactcctccaaagagacatgtcag**

**Fortunella_japonica_11** **gagtattgaatacacccatcggtctgagcttcatgtgactcctccaaagagacatgtcag**

**Fortunella_japonica_12** **gagtattgaatacacccatcggtctgagcttcatgtgactcctccaaagagacatgtcag**

**Fortunella_margarita_1** **gagtattgaatacacccatcggtctgagcttcatgtgactcctccaaagagacatgtcag**

**Fortunella_margarita_2** **gagtattgaatacacccatcggtctgagcttcatgtgactcctccaaagagacatgtcag**

**Fortunella_venosa_1** **gagtattgaatacacccatcggtctgagcttcatgtgactcctccaaagagacatgtcag**

**Fortunella_venosa_2** **gagtattgaatacacccatcggtctgagcttcatgtgactcctccaaagagacatgtcag**

**Fortunella_venosa_3** **gagtattgaatacacccatcggtctgagcttcatgtgactcctccaaagagacatgtcag**

25390	25400	25410	25420	25430	25440

....|....|....|....|....|....|....|....|....|....|....|....|

**Atalantia_kwangtungens** **gttcagggcatcccaattcgattgcctgggatgacagtttcatatttctaatctgtaaaa**

**Citrus_reticulata_1** **gttcagggcatcccaattcgattgcctgggatgacagtttcatatttctaatctgtaaaa**

**Citrus_reticulata_2** **gttcagggcatcccaattcgattgcctgggatgacagtttcatatttctaatctgtaaaa**

**Citrus_trifoliata_3** **gttcagggcatcccaattcgattgcctgggatgacagtttcatatttctaatctgtaaaa**

**Citrus_aurantiifolia** **gttcagggcatcccaattcgattgcctgggatgacagtttcatatttctaatctgtaaaa**

**Citrus_aurantium** **gttcagggcatcccaattcgattgcctgggatgacagtttcatatttcgaatctgtaaaa**

**Citrus_cavaleriei** **gttcagggcatcccaattcgattgcctgggatgacagtttcatatttcgaatctgtaaaa**

**Citrus_hongheensis** **gttcagggcatcccaattcgattgcctgggatgacagtttcatatttcgaatctgtaaaa**

**Citrus_limon** **gttcagggcatcccaattcgattgcctgggatgacagtttcatatttcgaatctgtaaaa**

**Citrus_maxima** **gttcagggcatcccaattcgattgcctgggatgacagtttcatatttcgaatctgtaaaa**

**Citrus_medica** **gttcagggcatcccaattcgattgcctgggatgacagtttcatatttctaatctgtaaaa**

**Citrus_polytrifolia** **gttcagggcatcccaattcgattgcctgggatgacagtttcatatttctaatctgtaaaa**

**Citrus_reticulata** **gttcagggcatcccaattcgattgcctgggatgacagtttcatatttctaatctgtaaaa**

**Citrus_sinensis** **gttcagggcatcccaattcgattgcctgggatgacagtttcatatttcgaatctgtaaaa**

**Fortunella_bawangica_1** **gttcagggcatcccaattcgattgcctgggatgacagtttcatatttctaatctgtaaaa**

**Fortunella_bawangica_2** **gttcagggcatcccaattcgattgcctgggatgacagtttcatatttctaatctgtaaaa**

**Fortunella_hindsii_1** **gttcagggcatcccaattcgattgcctgggatgacagtttcatatttctaatctgtaaaa**

**Fortunella_hindsii_2** **gttcagggcatcccaattcgattgcctgggatgacagtttcatatttctaatctgtaaaa**

**Fortunella_hindsii_3** **gttcagggcatcccaattcgattgcctgggatgacagtttcatatttctaatctgtaaaa**

**Fortunella_japonica_1** **gttcagggcatcccaattcgattgcctgggatgacagtttcatatttctaatctgtaaaa**

**Fortunella_japonica_2** **gttcagggcatcccaattcgattgcctgggatgacagtttcatatttctaatctgtaaaa**

**Fortunella_japonica_3** **gttcagggcatcccaattcgattgcctgggatgacagtttcatatttctaatctgtaaaa**

**Fortunella_japonica_4** **gttcagggcatcccaattcgattgcctgggatgacagtttcatatttctaatctgtaaaa**

**Fortunella_japonica_5** **gttcagggcatcccaattcgattgcctgggatgacagtttcatatttctaatctgtaaaa**

**Fortunella_japonica_6** **gttcagggcatcccaattcgattgcctgggatgacagtttcatatttctaatctgtaaaa**

**Fortunella_japonica_7** **gttcagggcatcccaattcgattgcctgggatgacagtttcatatttctaatctgtaaaa**

**Fortunella_japonica_8** **gttcagggcatcccaattcgattgcctgggatgacagtttcatatttctaatctgtaaaa**

**Fortunella_japonica_9** **gttcagggcatcccaattcgattgcctgggatgacagtttcatatttctaatctgtaaaa**

**Fortunella_japonica_10** **gttcagggcatcccaattcgattgcctgggatgacagtttcatatttctaatctgtaaaa**

**Fortunella_japonica_11** **gttcagggcatcccaattcgattgcctgggatgacagtttcatatttctaatctgtaaaa**

**Fortunella_japonica_12** **gttcagggcatcccaattcgattgcctgggatgacagtttcatatttctaatctgtaaaa**

**Fortunella_margarita_1** **gttcagggcatcccaattcgattgcctgggatgacagtttcatatttctaatctgtaaaa**

**Fortunella_margarita_2** **gttcagggcatcccaattcgattgcctgggatgacagtttcatatttctaatctgtaaaa**

**Fortunella_venosa_1** **gttcagggcatcccaattcgattgcctgggatgacagtttcatatttctaatctgtaaaa**

**Fortunella_venosa_2** **gttcagggcatcccaattcgattgcctgggatgacagtttcatatttctaatctgtaaaa**

**Fortunella_venosa_3** **gttcagggcatcccaattcgattgcctgggatgacagtttcatatttctaatctgtaaaa**

25450	25460	25470	25480	25490	25500

....|....|....|....|....|....|....|....|....|....|....|....|

**Atalantia_kwangtungens** **tcaaaatttcgatcaaatcacacatcgcaatatactaggccttctaattctttaagaggt**

**Citrus_reticulata_1** **tcaaaatttcgatcaaatcacacatcgcaatatactaggccttctaattctttaagaggt**

**Citrus_reticulata_2** **tcaaaatttcgatcaaatcacacatcgcaatatactaggccttctaattctttaagaggt**

**Citrus_trifoliata_3** **tcaaaatttcgatcaaatcacacatcgcaatatactaggccttctaattctttaagaggt**

**Citrus_aurantiifolia** **tcaaaatttcgatcaaatcacacatcgcaatatactaggccttctaattctttaagaggt**

**Citrus_aurantium** **tcaaaatttcgatcaaatcacacatcgcaatatactaggccttctaattctttaagaggt**

**Citrus_cavaleriei** **tcaaaatttcgatcaaatcacacatcgcaatatactaggccttctaattctttaagaggt**

**Citrus_hongheensis** **tcaaaatttcgatcaaatcacacatcgcaatatactaggccttctaattctttaagaggt**

**Citrus_limon** **tcaaaatttcgatcaaatcacacatcgcaatatactaggccttctaattctttaagaggt**

**Citrus_maxima** **tcaaaatttcgatcaaatcacacatcgcaatatactaggccttctaattctttaagaggt**

**Citrus_medica** **tcaaaatttcgatcaaatcacacatcgcaatatactaggccttctaattctttaagaggt**

**Citrus_polytrifolia** **tcaaaatttcgatcaaatcacacatcgcaatatactaggccttctaattctttaagaggt**

**Citrus_reticulata** **tcaaaatttcgatcaaatcacacatcgcaatatactaggccttctaattctttaagaggt**

**Citrus_sinensis** **tcaaaatttcgatcaaatcacacatcgcaatatactaggccttctaattctttaagaggt**

**Fortunella_bawangica_1** **tcaaaatttcgatcaaatcacacatcgcaatatactaggccttctaattctttaagaggt**

**Fortunella_bawangica_2** **tcaaaatttcgatcaaatcacacatcgcaatatactaggccttctaattctttaagaggt**

**Fortunella_hindsii_1** **tcaaaatttcgatcaaatcacacatcgcaatatactaggccttctaattctttaagaggt**

**Fortunella_hindsii_2** **tcaaaatttcgatcaaatcacacatcgcaatatactaggccttctaattctttaagaggt**

**Fortunella_hindsii_3** **tcaaaatttcgatcaaatcacacatcgcaatatactaggccttctaattctttaagaggt**

**Fortunella_japonica_1** **tcaaaatttcgatcaaatcacacatcgcaatatactaggccttctaattctttaagaggt**

**Fortunella_japonica_2** **tcaaaatttcgatcaaatcacacatcgcaatatactaggccttctaattctttaagaggt**

**Fortunella_japonica_3** **tcaaaatttcgatcaaatcacacatcgcaatatactaggccttctaattctttaagaggt**

**Fortunella_japonica_4** **tcaaaatttcgatcaaatcacacatcgcaatatactaggccttctaattctttaagaggt**

**Fortunella_japonica_5** **tcaaaatttcgatcaaatcacacatcgcaatatactaggccttctaattctttaagaggt**

**Fortunella_japonica_6** **tcaaaatttcgatcaaatcacacatcgcaatatactaggccttctaattctttaagaggt**

**Fortunella_japonica_7** **tcaaaatttcgatcaaatcacacatcgcaatatactaggccttctaattctttaagaggt**

**Fortunella_japonica_8** **tcaaaatttcgatcaaatcacacatcgcaatatactaggccttctaattctttaagaggt**

**Fortunella_japonica_9** **tcaaaatttcgatcaaatcacacatcgcaatatactaggccttctaattctttaagaggt**

**Fortunella_japonica_10** **tcaaaatttcgatcaaatcacacatcgcaatatactaggccttctaattctttaagaggt**

**Fortunella_japonica_11** **tcaaaatttcgatcaaatcacacatcgcaatatactaggccttctaattctttaagaggt**

**Fortunella_japonica_12** **tcaaaatttcgatcaaatcacacatcgcaatatactaggccttctaattctttaagaggt**

**Fortunella_margarita_1** **tcaaaatttcgatcaaatcacacatcgcaatatactaggccttctaattctttaagaggt**

**Fortunella_margarita_2** **tcaaaatttcgatcaaatcacacatcgcaatatactaggccttctaattctttaagaggt**

**Fortunella_venosa_1** **tcaaaatttcgatcaaatcacacatcgcaatatactaggccttctaattctttaagaggt**

**Fortunella_venosa_2** **tcaaaatttcgatcaaatcacacatcgcaatatactaggccttctaattctttaagaggt**

**Fortunella_venosa_3** **tcaaaatttcgatcaaatcacacatcgcaatatactaggccttctaattctttaagaggt**

25510	25520	25530	25540	25550	25560

....|....|....|....|....|....|....|....|....|....|....|....|

**Atalantia_kwangtungens** **ttatctaaaagattcgcgatataactaggaagacgtttcaaataccacacgtgggttact**

**Citrus_reticulata_1** **ttatctaaaagattcgcgatataactaggaagacgtttcaaataccacacgtgggttact**

**Citrus_reticulata_2** **ttatctaaaagattcgcgatataactaggaagacgtttcaaataccacacgtgggttact**

**Citrus_trifoliata_3** **ttatctaaaagattcgcgatataactaggaagacgtttcaaataccacacgtgggttact**

**Citrus_aurantiifolia** **ttatctaaaagattcgcgatataactaggaagacgtttcaaataccacacgtgggttact**

**Citrus_aurantium** **ttatctaaaagattcgcgatataactaggaagacgtttcaaataccacacgtgggttact**

**Citrus_cavaleriei** **ttatctaaaagattcgcgatataactaggaagacgtttcaaataccacacgtgggttact**

**Citrus_hongheensis** **ttatctaaaagattcgcgatataactaggaagacgtttcaaataccacacgtgggttact**

**Citrus_limon** **ttatctaaaagattcgcgatataactaggaagacgtttcaaataccacacgtgggttact**

**Citrus_maxima** **ttatctaaaagattcgcgatataactaggaagacgtttcaaataccacacgtgggttact**

**Citrus_medica** **ttatctaaaagattcgcgatataactaggaagacgtttcaaataccacacgtgggttact**

**Citrus_polytrifolia** **ttatctaaaagattcgcgatataactaggaagacgtttcaaataccacacgtgggttact**

**Citrus_reticulata** **ttatctaaaagattcgcgatataactaggaagacgtttcaaataccacacgtgggttact**

**Citrus_sinensis** **ttatctaaaagattcgcgatataactaggaagacgtttcaaataccacacgtgggttact**

**Fortunella_bawangica_1** **ttatctaaaagattcgcgatataactaggaagacgtttcaaataccacacgtgggttact**

**Fortunella_bawangica_2** **ttatctaaaagattcgcgatataactaggaagacgtttcaaataccacacgtgggttact**

**Fortunella_hindsii_1** **ttatctaaaagattcgcgatataactaggaagacgtttcaaataccacacgtgggttact**

**Fortunella_hindsii_2** **ttatctaaaagattcgcgatataactaggaagacgtttcaaataccacacgtgggttact**

**Fortunella_hindsii_3** **ttatctaaaagattcgcgatataactaggaagacgtttcaaataccacacgtgggttact**

**Fortunella_japonica_1** **ttatctaaaagattcgcgatataactaggaagacgtttcaaataccacacgtgggttact**

**Fortunella_japonica_2** **ttatctaaaagattcgcgatataactaggaagacgtttcaaataccacacgtgggttact**

**Fortunella_japonica_3** **ttatctaaaagattcgcgatataactaggaagacgtttcaaataccacacgtgggttact**

**Fortunella_japonica_4** **ttatctaaaagattcgcgatataactaggaagacgtttcaaataccacacgtgggttact**

**Fortunella_japonica_5** **ttatctaaaagattcgcgatataactaggaagacgtttcaaataccacacgtgggttact**

**Fortunella_japonica_6** **ttatctaaaagattcgcgatataactaggaagacgtttcaaataccacacgtgggttact**

**Fortunella_japonica_7** **ttatctaaaagattcgcgatataactaggaagacgtttcaaataccacacgtgggttact**

**Fortunella_japonica_8** **ttatctaaaagattcgcgatataactaggaagacgtttcaaataccacacgtgggttact**

**Fortunella_japonica_9** **ttatctaaaagattcgcgatataactaggaagacgtttcaaataccacacgtgggttact**

**Fortunella_japonica_10** **ttatctaaaagattcgcgatataactaggaagacgtttcaaataccacacgtgggttact**

**Fortunella_japonica_11** **ttatctaaaagattcgcgatataactaggaagacgtttcaaataccacacgtgggttact**

**Fortunella_japonica_12** **ttatctaaaagattcgcgatataactaggaagacgtttcaaataccacacgtgggttact**

**Fortunella_margarita_1** **ttatctaaaagattcgcgatataactaggaagacgtttcaaataccacacgtgggttact**

**Fortunella_margarita_2** **ttatctaaaagattcgcgatataactaggaagacgtttcaaataccacacgtgggttact**

**Fortunella_venosa_1** **ttatctaaaagattcgcgatataactaggaagacgtttcaaataccacacgtgggttact**

**Fortunella_venosa_2** **ttatctaaaagattcgcgatataactaggaagacgtttcaaataccacacgtgggttact**

**Fortunella_venosa_3** **ttatctaaaagattcgcgatataactaggaagacgtttcaaataccacacgtgggttact**

25570	25580	25590	25600	25610	25620

....|....|....|....|....|....|....|....|....|....|....|....|

**Atalantia_kwangtungens** **gggcatcccagttttatgtatcccatttgatatcttcgtatccgagaatcaacaaactca**

**Citrus_reticulata_1** **gggcatcccagttttatgtatcccatttgatatcttcgtatccgagaatcaacaaattca**

**Citrus_reticulata_2** **gggcatcccagttttatgtatcccatttgatatcttcgtatccgagaatcaacaaattca**

**Citrus_trifoliata_3** **gggcatcccagttttatgtatcccatttgatatcttcgtatccgagaatcaacaaattca**

**Citrus_aurantiifolia** **gggcatcccagttttatgtatcccatttgatatcttcgtatccgagaatcaacaaattca**

**Citrus_aurantium** **gggcatcccagttttatgtatcccatttgatatcttcgtatccgagaatcaacaaattca**

**Citrus_cavaleriei** **gggcatcccagttttatgtatcccatttgatatcttcgtatccgagaatcaacaaattca**

**Citrus_hongheensis** **gggcatcccagttttatgtatcccatttgatatcttcgtatccgagaatcaacaaattca**

**Citrus_limon** **gggcatcccagttttatgtatcccatttgatatcttcgtatccgagaatcaacaaattca**

**Citrus_maxima** **gggcatcccagttttatgtatcccatttgatatcttcgtatccgagaatcaacaaattca**

**Citrus_medica** **gggcatcccagttttatgtatcccatttgatatcttcgtatccgagaatcaacaaattca**

**Citrus_polytrifolia** **gggcatcccagttttatgtatcccatttgatatcttcgtatccgagaatcaacaaattca**

**Citrus_reticulata** **gggcatcccagttttatgtatcccatttgatatcttcgtatccgagaatcaacaaattca**

**Citrus_sinensis** **gggcatcccagttttatgtatcccatttgatatcttcgtatccgagaatcaacaaattca**

**Fortunella_bawangica_1** **gggcatcccagttttatgtatcccatttgatatcttcgtatccgagaatcaacaaattca**

**Fortunella_bawangica_2** **gggcatcccagttttatgtatcccatttgatatcttcgtatccgagaatcaacaaattca**

**Fortunella_hindsii_1** **gggcatcccagttttatgtatcccatttgatatcttcgtatccgagaatcaacaaattca**

**Fortunella_hindsii_2** **gggcatcccagttttatgtatcccatttgatatcttcgtatccgagaatcaacaaattca**

**Fortunella_hindsii_3** **gggcatcccagttttatgtatcccatttgatatcttcgtatccgagaatcaacaaattca**

**Fortunella_japonica_1** **gggcatcccagttttatgtatcccatttgatatcttcgtatccgagaatcaacaaattca**

**Fortunella_japonica_2** **gggcatcccagttttatgtatcccatttgatatcttcgtatccgagaatcaacaaattca**

**Fortunella_japonica_3** **gggcatcccagttttatgtatcccatttgatatcttcgtatccgagaatcaacaaattca**

**Fortunella_japonica_4** **gggcatcccagttttatgtatcccatttgatatcttcgtatccgagaatcaacaaattca**

**Fortunella_japonica_5** **gggcatcccagttttatgtatcccatttgatatcttcgtatccgagaatcaacaaattca**

**Fortunella_japonica_6** **gggcatcccagttttatgtatcccatttgatatcttcgtatccgagaatcaacaaattca**

**Fortunella_japonica_7** **gggcatcccagttttatgtatcccatttgatatcttcgtatccgagaatcaacaaattca**

**Fortunella_japonica_8** **gggcatcccagttttatgtatcccatttgatatcttcgtatccgagaatcaacaaattca**

**Fortunella_japonica_9** **gggcatcccagttttatgtatcccatttgatatcttcgtatccgagaatcaacaaattca**

**Fortunella_japonica_10** **gggcatcccagttttatgtatcccatttgatatcttcgtatccgagaatcaacaaattca**

**Fortunella_japonica_11** **gggcatcccagttttatgtatcccatttgatatcttcgtatccgagaatcaacaaattca**

**Fortunella_japonica_12** **gggcatcccagttttatgtatcccatttgatatcttcgtatccgagaatcaacaaattca**

**Fortunella_margarita_1** **gggcatcccagttttatgtatcccatttgatatcttcgtatccgagaatcaacaaattca**

**Fortunella_margarita_2** **gggcatcccagttttatgtatcccatttgatatcttcgtatccgagaatcaacaaattca**

**Fortunella_venosa_1** **gggcatcccagttttatgtatcccatttgatatcttcgtatccgagaatcaacaaattca**

**Fortunella_venosa_2** **gggcatcccagttttatgtatcccatttgatatcttcgtatccgagaatcaacaaattca**

**Fortunella_venosa_3** **gggcatcccagttttatgtatcccatttgatatcttcgtatccgagaatcaacaaattca**

25630	25640	25650	25660	25670	25680

....|....|....|....|....|....|....|....|....|....|....|....|

**Atalantia_kwangtungens** **accccacattgttcacaaaattgcgggtcttctttttcatctccgattattcgataattt**

**Citrus_reticulata_1** **accccacattgttcacaaaattgcgggtcttctttttcatctccgattattcgataattt**

**Citrus_reticulata_2** **accccacattgttcacaaaattgcgggtcttctttttcatctccgattattcgataattt**

**Citrus_trifoliata_3** **accccacattgttcacaaaattgcgggtcttctttttcatctccgattattcgataattt**

**Citrus_aurantiifolia** **accccacattgttcacaaaattgcgggtcttctttttcatctccgattattcgataattt**

**Citrus_aurantium** **accccacattgttcacaaaattgcgggtcttctttttcatctccgattattcgataattt**

**Citrus_cavaleriei** **accccacattgttcacaaaattgcgggtcttctttttcatctccgattattcgataattt**

**Citrus_hongheensis** **accccacattgttcacaaaattgcgggtcttctttttcatctccgattattcgataattt**

**Citrus_limon** **accccacattgttcacaaaattgcgggtcttctttttcatctccgattattcgataattt**

**Citrus_maxima** **accccacattgttcacaaaattgcgggtcttctttttcatctccgattattcgataattt**

**Citrus_medica** **accccacattgttcacaaaattgcgggtcttctttttcatctccgattattcgataattt**

**Citrus_polytrifolia** **accccacattgttcacaaaattgcgggtcttctttttcatctccgattattcgataattt**

**Citrus_reticulata** **accccacattgttcacaaaattgcgggtcttctttttcatctccgattattcgataattt**

**Citrus_sinensis** **accccacattgttcacaaaattgcgggtcttctttttcatctccgattattcgataattt**

**Fortunella_bawangica_1** **accccacattgttcacaaaattgcgggtcttctttttcatctccgattattcgataattt**

**Fortunella_bawangica_2** **accccacattgttcacaaaattgcgggtcttctttttcatctccgattattcgataattt**

**Fortunella_hindsii_1** **accccacattgttcacaaaattgcgggtcttctttttcatctccgattattcgataattt**

**Fortunella_hindsii_2** **accccacattgttcacaaaattgcgggtcttctttttcatctccgattattcgataattt**

**Fortunella_hindsii_3** **accccacattgttcacaaaattgcgggtcttctttttcatctccgattattcgataattt**

**Fortunella_japonica_1** **accccacattgttcacaaaattgcgggtcttctttttcatctccgattattcgataattt**

**Fortunella_japonica_2** **accccgcattgttcacaaaattgcgggtcttctttttcatctccgattattcgataattt**

**Fortunella_japonica_3** **accccacattgttcacaaaattgcgggtcttctttttcatctccgattattcgataattt**

**Fortunella_japonica_4** **accccacattgttcacaaaattgcgggtcttctttttcatctccgattattcgataattt**

**Fortunella_japonica_5** **accccacattgttcacaaaattgcgggtcttctttttcatctccgattattcgataattt**

**Fortunella_japonica_6** **accccacattgttcacaaaattgcgggtcttctttttcatctccgattattcgataattt**

**Fortunella_japonica_7** **accccacattgttcacaaaattgcgggtcttctttttcatctccgattattcgataattt**

**Fortunella_japonica_8** **accccacattgttcacaaaattgcgggtcttctttttcatctccgattattcgataattt**

**Fortunella_japonica_9** **accccacattgttcacaaaattgcgggtcttctttttcatctccgattattcgataattt**

**Fortunella_japonica_10** **accccacattgttcacaaaattgcgggtcttctttttcatctccgattattcgataattt**

**Fortunella_japonica_11** **accccacattgttcacaaaattgcgggtcttctttttcatctccgattattcgataattt**

**Fortunella_japonica_12** **accccacattgttcacaaaattgcgggtcttctttttcatctccgattattcgataattt**

**Fortunella_margarita_1** **accccacattgttcacaaaattgcgggtcttctttttcatctccgattattcgataattt**

**Fortunella_margarita_2** **accccacattgttcacaaaattgcgggtcttctttttcatctccgattattcgataattt**

**Fortunella_venosa_1** **accccacattgttcacaaaattgcgggtcttctttttcatctccgattattcgataattt**

**Fortunella_venosa_2** **accccacattgttcacaaaattgcgggtcttctttttcatctccgattattcgataattt**

**Fortunella_venosa_3** **accccacattgttcacaaaattgcgggtcttctttttcatctccgattattcgataattt**

25690	25700	25710	25720	25730	25740

....|....|....|....|....|....|....|....|....|....|....|....|

**Atalantia_kwangtungens** **ccacaagcacaaattccacttttaataggcccaaaaattctttcacaaaataatccatct**

**Citrus_reticulata_1** **ccacaagcacaaattccacttttaataggcccaaaaattctttcacaaaataatccatct**

**Citrus_reticulata_2** **ccacaagcacaaattccacttttaataggcccaaaaattctttcacaaaataatccatct**

**Citrus_trifoliata_3** **ccacaagcacaaattccacttttaataggcccaaaaattctttcacaaaataatccatct**

**Citrus_aurantiifolia** **ccacaagcacaaattccacttttaataggcccaaaaattctttcacaaaataatccatct**

**Citrus_aurantium** **ccacaagcacaaattccacttttaataggcccaaaaattctttcacaaaataatccatct**

**Citrus_cavaleriei** **ccacaagcacaaattccacttttaataggcccaaaaattctttcacaaaataatccatct**

**Citrus_hongheensis** **ccacaagcacaaattccacttttaataggcccaaaaattctttcacaaaataatccatct**

**Citrus_limon** **ccacaagcacaaattccacttttaataggcccaaaaattctttcacaaaataatccatct**

**Citrus_maxima** **ccacaagcacaaattccacttttaataggcccaaaaattctttcacaaaataatccatct**

**Citrus_medica** **ccacaagcacaaattccacttttaataggcccaaaaattctttcacaaaataatccatct**

**Citrus_polytrifolia** **ccacaagcacaaattccacttttaataggcccaaaaattctttcacaaaataatccatct**

**Citrus_reticulata** **ccacaagcacaaattccacttttaataggcccaaaaattctttcacaaaataatccatct**

**Citrus_sinensis** **ccacaagcacaaattccacttttaataggcccaaaaattctttcacaaaataatccatct**

**Fortunella_bawangica_1** **ccacaagcacaaattccacttttaataggcccaaaaattctttcacaaaataatccatct**

**Fortunella_bawangica_2** **ccacaagcacaaattccacttttaataggcccaaaaattctttcacaaaataatccatct**

**Fortunella_hindsii_1** **ccacaagcacaaattccacttttaataggcccaaaaattctttcacaaaataatccatct**

**Fortunella_hindsii_2** **ccacaagcacaaattccacttttaataggcccaaaaattctttcacaaaataatccatct**

**Fortunella_hindsii_3** **ccacaagcacaaattccacttttaataggcccaaaaattctttcacaaaataatccatct**

**Fortunella_japonica_1** **ccacaagcacaaattccacttttaataggcccaaaaattctttcacaaaataatccatct**

**Fortunella_japonica_2** **ccacaagcacaaattccacttttaataggcccaaaaattctttcacaaaataatccatct**

**Fortunella_japonica_3** **ccacaagcacaaattccacttttaataggcccaaaaattctttcacaaaataatccatct**

**Fortunella_japonica_4** **ccacaagcacaaattccacttttaataggcccaaaaattctttcacaaaataatccatct**

**Fortunella_japonica_5** **ccacaagcacaaattccacttttaataggcccaaaaattctttcacaaaataatccatct**

**Fortunella_japonica_6** **ccacaagcacaaattccacttttaataggcccaaaaattctttcacaaaataatccatct**

**Fortunella_japonica_7** **ccacaagcacaaattccacttttaataggcccaaaaattctttcacaaaataatccatct**

**Fortunella_japonica_8** **ccacaagcacaaattccacttttaataggcccaaaaattctttcacaaaataatccatct**

**Fortunella_japonica_9** **ccacaagcacaaattccacttttaataggcccaaaaattctttcacaaaataatccatct**

**Fortunella_japonica_10** **ccacaagcacaaattccacttttaataggcccaaaaattctttcacaaaataatccatct**

**Fortunella_japonica_11** **ccacaagcacaaattccacttttaataggcccaaaaattctttcacaaaataatccatct**

**Fortunella_japonica_12** **ccacaagcacaaattccacttttaataggcccaaaaattctttcacaaaataatccatct**

**Fortunella_margarita_1** **ccacaagcacaaattccacttttaataggcccaaaaattctttcacaaaataatccatct**

**Fortunella_margarita_2** **ccacaagcacaaattccacttttaataggcccaaaaattctttcacaaaataatccatct**

**Fortunella_venosa_1** **ccacaagcacaaattccacttttaataggcccaaaaattctttcacaaaataatccatct**

**Fortunella_venosa_2** **ccacaagcacaaattccacttttaataggcccaaaaattctttcacaaaataatccatct**

**Fortunella_venosa_3** **ccacaagcacaaattccacttttaataggcccaaaaattctttcacaaaataatccatct**

25750	25760	25770	25780	25790	25800

....|....|....|....|....|....|....|....|....|....|....|....|

**Atalantia_kwangtungens** **ttttccggtttattggttttgtaatgaaaagtatagggttttgtcacttctccaattatc**

**Citrus_reticulata_1** **ttttccggtttattggttttgtaatgaaaagtatagggttttgtcacttctccaattatc**

**Citrus_reticulata_2** **ttttccggtttattggttttgtaatgaaaagtatagggttttgtcacttctccaattatc**

**Citrus_trifoliata_3** **ttttccggtttattggttttgtaatgaaaagtatagggttttgtcacttctccaattatc**

**Citrus_aurantiifolia** **ttttccggtttattggttttgtaatgaaaagtatagggttttgtcacttctccaattatc**

**Citrus_aurantium** **ttttccggtttattggttttgtaatgaaaagtatagggttttgtcacttctccaattatc**

**Citrus_cavaleriei** **ttttccggtttattggttttgtaatgaaaagtatagggttttgtcacttctccaattatc**

**Citrus_hongheensis** **ttttccggtttattggttttgtaatgaaaagtatagggttttgtcacttctccaattatc**

**Citrus_limon** **ttttccggtttattggttttgtaatgaaaagtatagggttttgtcacttctccaattatc**

**Citrus_maxima** **ttttccggtttattggttttgtaatgaaaagtatagggttttgtcacttctccaattatc**

**Citrus_medica** **ttttccggtttattggttttgtaatgaaaagtatagggttttgtcacttctccaattatc**

**Citrus_polytrifolia** **ttttccggtttattggttttgtaatgaaaagtatagggttttgtcacttctccaattatc**

**Citrus_reticulata** **ttttccggtttattggttttgtaatgaaaagtatagggttttgtcacttctccaattatc**

**Citrus_sinensis** **ttttccggtttattggttttgtaatgaaaagtatagggttttgtcacttctccaattatc**

**Fortunella_bawangica_1** **ttttccggtttattggttttgtaatgaaaagtatagggttttgtcacttctccaattatc**

**Fortunella_bawangica_2** **ttttccggtttattggttttgtaatgaaaagtatagggttttgtcacttctccaattatc**

**Fortunella_hindsii_1** **ttttccggtttattggttttgtaatgaaaagtatagggttttgtcacttctccaattatc**

**Fortunella_hindsii_2** **ttttccggtttattggttttgtaatgaaaagtatagggttttgtcacttctccaattatc**

**Fortunella_hindsii_3** **ttttccggtttattggttttgtaatgaaaagtatagggttttgtcacttctccaattatc**

**Fortunella_japonica_1** **ttttccggtttattggttttgtaatgaaaagtatagggttttgtcacttctccaattatc**

**Fortunella_japonica_2** **ttttccggtttattggttttgtaatgaaaagtatagggttttgtcacttctccaattatc**

**Fortunella_japonica_3** **ttttccggtttattggttttgtaatgaaaagtatagggttttgtcacttctccaattatc**

**Fortunella_japonica_4** **ttttccggtttattggttttgtaatgaaaagtatagggttttgtcacttctccaattatc**

**Fortunella_japonica_5** **ttttccggtttattggttttgtaatgaaaagtatagggttttgtcacttctccaattatc**

**Fortunella_japonica_6** **ttttccggtttattggttttgtaatgaaaagtatagggttttgtcacttctccaattatc**

**Fortunella_japonica_7** **ttttccggtttattggttttgtaatgaaaagtatagggttttgtcacttctccaattatc**

**Fortunella_japonica_8** **ttttccggtttattggttttgtaatgaaaagtatagggttttgtcacttctccaattatc**

**Fortunella_japonica_9** **ttttccggtttattggttttgtaatgaaaagtatagggttttgtcacttctccaattatc**

**Fortunella_japonica_10** **ttttccggtttattggttttgtaatgaaaagtatagggttttgtcacttctccaattatc**

**Fortunella_japonica_11** **ttttccggtttattggttttgtaatgaaaagtatagggttttgtcacttctccaattatc**

**Fortunella_japonica_12** **ttttccggtttattggttttgtaatgaaaagtatagggttttgtcacttctccaattatc**

**Fortunella_margarita_1** **ttttccggtttattggttttgtaatgaaaagtatagggttttgtcacttctccaattatc**

**Fortunella_margarita_2** **ttttccggtttattggttttgtaatgaaaagtatagggttttgtcacttctccaattatc**

**Fortunella_venosa_1** **ttttccggtttattggttttgtaatgaaaagtatagggttttgtcacttctccaattatc**

**Fortunella_venosa_2** **ttttccggtttattggttttgtaatgaaaagtatagggttttgtcacttctccaattatc**

**Fortunella_venosa_3** **ttttccggtttattggttttgtaatgaaaagtatagggttttgtcacttctccaattatc**

25810	25820	25830	25840	25850	25860

....|....|....|....|....|....|....|....|....|....|....|....|

**Atalantia_kwangtungens** **tctccattaggtaggattttattggcccaagcgcgtatttgttgaggagaaactgatcca**

**Citrus_reticulata_1** **tctccattaggtaggattttattggcccaagcgcgtatttgttgaggagaaactgatcca**

**Citrus_reticulata_2** **tctccattaggtaggattttattggcccaagcgcgtatttgttgaggagaaactgatcca**

**Citrus_trifoliata_3** **tctccattaggtaggattttattggcccaagcacgtatttgttgaggagaaactgatcca**

**Citrus_aurantiifolia** **tctccattaggtaggattttattggcccaagcgcgtatttgttgaggagaaactgatcca**

**Citrus_aurantium** **tctccattaggtaggattttattggcccaagcgcgtatttgttgaggagaaactgatcca**

**Citrus_cavaleriei** **tctccattaggtaggattttattggcccaagcgcgtatttgttgaggagaaactgatcca**

**Citrus_hongheensis** **tctccattaggtaggattttattggcccaagcgcgtatttgttgaggagaaactgatcca**

**Citrus_limon** **tctccattaggtaggattttattggcccaagcgcgtatttgttgaggagaaactgatcca**

**Citrus_maxima** **tctccattaggtaggattttattggcccaagcgcgtatttgttgaggagaaactgatcca**

**Citrus_medica** **tctccattaggtaggattttattggcccaagcgcgtatttgttgaggagaaactgatcca**

**Citrus_polytrifolia** **tctccattaggtaggattttattggcccaagcacgtatttgttgaggagaaactgatcca**

**Citrus_reticulata** **tctccattaggtaggattttattggcccaagcgcgtatttgttgaggagaaactgatcca**

**Citrus_sinensis** **tctccattaggtaggattttattggcccaagcgcgtatttgttgaggagaaactgatcca**

**Fortunella_bawangica_1** **tctccattaggtaggattttattggcccaagcgcgtatttgttgaggagaaactgatcca**

**Fortunella_bawangica_2** **tctccattaggtaggattttattggcccaagcgcgtatttgttgaggagaaactgatcca**

**Fortunella_hindsii_1** **tctccattaggtaggattttattggcccaagcgcgtatttgttgaggagaaactgatcca**

**Fortunella_hindsii_2** **tctccattaggtaggattttattggcccaagcgcgtatttgttgaggagaaactgatcca**

**Fortunella_hindsii_3** **tctccattaggtaggattttattggcccaagcgcgtatttgttgaggagaaactgatcca**

**Fortunella_japonica_1** **tctccattaggtaggattttattggcccaagcgcgtatttgttgaggagaaactgatcca**

**Fortunella_japonica_2** **tctccattaggtaggattttattggcccaagcgcgtatttgttgaggagaaactgatcca**

**Fortunella_japonica_3** **tctccattaggtaggattttattggcccaagcgcgtatttgttgaggagaaactgatcca**

**Fortunella_japonica_4** **tctccattaggtaggattttattggcccaagcgcgtatttgttgaggagaaactgatcca**

**Fortunella_japonica_5** **tctccattaggtaggattttattggcccaagcgcgtatttgttgaggagaaactgatcca**

**Fortunella_japonica_6** **tctccattaggtaggattttattggcccaagcgcgtatttgttgaggagaaactgatcca**

**Fortunella_japonica_7** **tctccattaggtaggattttattggcccaagcgcgtatttgttgaggagaaactgatcca**

**Fortunella_japonica_8** **tctccattaggtaggattttattggcccaagcgcgtatttgttgaggagaaactgatcca**

**Fortunella_japonica_9** **tctccattaggtaggattttattggcccaagcgcgtatttgttgaggagaaactgatcca**

**Fortunella_japonica_10** **tctccattaggtaggattttattggcccaagcgcgtatttgttgaggagaaactgatcca**

**Fortunella_japonica_11** **tctccattaggtaggattttattggcccaagcgcgtatttgttgaggagaaactgatcca**

**Fortunella_japonica_12** **tctccattaggtaggattttattggcccaagcgcgtatttgttgaggagaaactgatcca**

**Fortunella_margarita_1** **tctccattaggtaggattttattggcccaagcgcgtatttgttgaggagaaactgatcca**

**Fortunella_margarita_2** **tctccattaggtaggattttattggcccaagcgcgtatttgttgaggagaaactgatcca**

**Fortunella_venosa_1** **tctccattaggtaggattttattggcccaagcgcgtatttgttgaggagaaactgatcca**

**Fortunella_venosa_2** **tctccattaggtaggattttattggcccaagcgcgtatttgttgaggagaaactgatcca**

**Fortunella_venosa_3** **tctccattaggtaggattttattggcccaagcgcgtatttgttgaggagaaactgatcca**

25870	25880	25890	25900	25910	25920

....|....|....|....|....|....|....|....|....|....|....|....|

**Atalantia_kwangtungens** **attcggagttgttgatgtttatatcgatcaatcatagaagaaaatttttgattcattccg**

**Citrus_reticulata_1** **attcggagttgttgatgtttatatcgatcaatcatagaagaaaatttttgattcattccg**

**Citrus_reticulata_2** **attcggagttgttgatgtttatatcgatcaatcatagaagaaaatttttgattcattccg**

**Citrus_trifoliata_3** **attcggagttgttgatgtttatatcgatcaatcatagaagaaaatttttgattcattccg**

**Citrus_aurantiifolia** **attcggagttgttgatgtttatatcgatcaatcatagaagaaaatttttgattcattccg**

**Citrus_aurantium** **attcggagttgttgatgtttatatcgatcaatcatagaagaaaatttttgattcattccg**

**Citrus_cavaleriei** **attcggagttgttgatgtttatatcgatcaatcatagaagaaaatttttgattcattccg**

**Citrus_hongheensis** **attcggagttgttgatgtttatatcgatcaatcatagaagaaaatttttgattcattccg**

**Citrus_limon** **attcggagttgttgatgtttatatcgatcaatcatagaagaaaatttttgattcattccg**

**Citrus_maxima** **attcggagttgttgatgtttatatcgatcaatcatagaagaaaatttttgattcattccg**

**Citrus_medica** **attcggagttgttgatgtttatatcgatcaatcatagaagaaaatttttgattcattccg**

**Citrus_polytrifolia** **attcggagttgttgatgtttatatcgatcaatcatagaagaaaatttttgattcattccg**

**Citrus_reticulata** **attcggagttgttgatgtttatatcgatcaatcatagaagaaaatttttgattcattccg**

**Citrus_sinensis** **attcggagttgttgatgtttatatcgatcaatcatagaagaaaatttttgattcattccg**

**Fortunella_bawangica_1** **attcggagttgttgatgtttatatcgatcaatcatagaagaaaatttttgattcattccg**

**Fortunella_bawangica_2** **attcggagttgttgatgtttatatcgatcaatcatagaagaaaatttttgattcattccg**

**Fortunella_hindsii_1** **attcggagttgttgatgtttatatcgatcaatcatagaagaaaatttttgattcattccg**

**Fortunella_hindsii_2** **attcggagttgttgatgtttatatcgatcaatcatagaagaaaatttttgattcattccg**

**Fortunella_hindsii_3** **attcggagttgttgatgtttatatcgatcaatcatagaagaaaatttttgattcattccg**

**Fortunella_japonica_1** **attcggagttgttgatgtttatatcgatcaatcatagaagaaaatttttgattcattccg**

**Fortunella_japonica_2** **attcggagttgttgatgtttatatcgatcaatcatagaagaaaatttttgattcattccg**

**Fortunella_japonica_3** **attcggagttgttgatgtttatatcgatcaatcatagaagaaaatttttgattcattccg**

**Fortunella_japonica_4** **attcggagttgttgatgtttatatcgatcaatcatagaagaaaatttttgattcattccg**

**Fortunella_japonica_5** **attcggagttgttgatgtttatatcgatcaatcatagaagaaaatttttgattcattccg**

**Fortunella_japonica_6** **attcggagttgttgatgtttatatcgatcaatcatagaagaaaatttttgattcattccg**

**Fortunella_japonica_7** **attcggagttgttgatgtttatatcgatcaatcatagaagaaaatttttgattcattccg**

**Fortunella_japonica_8** **attcggagttgttgatgtttatatcgatcaatcatagaagaaaatttttgattcattccg**

**Fortunella_japonica_9** **attcggagttgttgatgtttatatcgatcaatcatagaagaaaatttttgattcattccg**

**Fortunella_japonica_10** **attcggagttgttgatgtttatatcgatcaatcatagaagaaaatttttgattcattccg**

**Fortunella_japonica_11** **attcggagttgttgatgtttatatcgatcaatcatagaagaaaatttttgattcattccg**

**Fortunella_japonica_12** **attcggagttgttgatgtttatatcgatcaatcatagaagaaaatttttgattcattccg**

**Fortunella_margarita_1** **attcggagttgttgatgtttatatcgatcaatcatagaagaaaatttttgattcattccg**

**Fortunella_margarita_2** **attcggagttgttgatgtttatatcgatcaatcatagaagaaaatttttgattcattccg**

**Fortunella_venosa_1** **attcggagttgttgatgtttatatcgatcaatcatagaagaaaatttttgattcattccg**

**Fortunella_venosa_2** **attcggagttgttgatgtttatatcgatcaatcatagaagaaaatttttgattcattccg**

**Fortunella_venosa_3** **attcggagttgttgatgtttatatcgatcaatcatagaagaaaatttttgattcattccg**

25930	25940	25950	25960	25970	25980

....|....|....|....|....|....|....|....|....|....|....|....|

**Atalantia_kwangtungens** **attaag-----------cttccttcttattaatctggaagtttttctcagatacaagaaa**

**Citrus_reticulata_1** **attaag-----------cttccttcttattaatctggaagtttttctcagatacaagaaa**

**Citrus_reticulata_2** **attaag-----------cttccttcttattaatctggaagtttttctcagatacaagaaa**

**Citrus_trifoliata_3** **attaag-----------cttccttcttattaatctggaagtttttctcagatacaagaaa**

**Citrus_aurantiifolia** **attaag-----------cttccttcttattaatctggaagtttttctcagatacaacaaa**

**Citrus_aurantium** **attaag-----------cttccttcttattaatctggaagtttttctcagatacaacaaa**

**Citrus_cavaleriei** **attaag-----------cttccttcttattaatctggaagtttttctcagatacaagaaa**

**Citrus_hongheensis** **attaag-----------cttccttcttattaatctggaagtttttctcagatacaacaaa**

**Citrus_limon** **attaag-----------cttccttcttattaatctggaagtttttctcagatacaacaaa**

**Citrus_maxima** **attaag-----------cttccttcttattaatctggaagtttttctcagatacaacaaa**

**Citrus_medica** **attaag-----------cttccttcttattaatctggaagtttttctcagatacaagaaa**

**Citrus_polytrifolia** **attaag-----------cttccttcttattaatctggaagtttttctcagatacaagaaa**

**Citrus_reticulata** **attaag-----------cttccttcttattaatctggaagtttttctcagatacaagaaa**

**Citrus_sinensis** **attaag-----------cttccttcttattaatctggaagtttttctcagatacaacaaa**

**Fortunella_bawangica_1** **attaag-----------cttccttcttattaatctggaagtttttctcagatacaagaaa**

**Fortunella_bawangica_2** **attaag-----------cttccttcttattaatctggaagtttttctcagatacaagaaa**

**Fortunella_hindsii_1** **attaag-----------cttccttcttattaatctggaagtttttctcagatacaagaaa**

**Fortunella_hindsii_2** **attaag-----------cttccttcttattaatctggaagtttttctcagatacaagaaa**

**Fortunella_hindsii_3** **attaag-----------cttccttcttattaatctggaagtttttctcagatacaagaaa**

**Fortunella_japonica_1** **attaag-----------cttccttcttattaatctggaagtttttctcagatacaagaaa**

**Fortunella_japonica_2** **attaag-----------cttccttcttattaatctggaagtttttctcagatacaagaaa**

**Fortunella_japonica_3** **attaag-----------cttccttcttattaatctggaagtttttctcagatacaagaaa**

**Fortunella_japonica_4** **attaag-----------cttccttcttattaatctggaagtttttctcagatacaagaaa**

**Fortunella_japonica_5** **attaag-----------cttccttcttattaatctggaagtttttctcagatacaagaaa**

**Fortunella_japonica_6** **attaag-----------cttccttcttattaatctggaagtttttctcagatacaagaaa**

**Fortunella_japonica_7** **attaag-----------cttccttcttattaatctggaagtttttctcagatacaagaaa**

**Fortunella_japonica_8** **attaag-----------cttccttcttattaatctggaagtttttctcagatacaagaaa**

**Fortunella_japonica_9** **attaag-----------cttccttcttattaatctggaagtttttctcagatacaagaaa**

**Fortunella_japonica_10** **attaag-----------cttccttcttattaatctggaagtttttctcagatacaagaaa**

**Fortunella_japonica_11** **attaag-----------cttccttcttattaatctggaagtttttctcagatacaagaaa**

**Fortunella_japonica_12** **attaag-----------cttccttcttattaatctggaagtttttctcagatacaagaaa**

**Fortunella_margarita_1** **attaag-----------cttccttcttattaatctggaagtttttctcagatacaagaaa**

**Fortunella_margarita_2** **attaag-----------cttccttcttattaatctggaagtttttctcagatacaagaaa**

**Fortunella_venosa_1** **attaagcttccttcttacttccttcttattaatctggaagtttttctcagatacaagaaa**

**Fortunella_venosa_2** **attaag-----------cttccttcttattaatctggaagtttttctcagatacaagaaa**

**Fortunella_venosa_3** **attaag-----------cttccttcttattaatctggaagtttttctcagatacaagaaa**

25990	26000	26010	26020	26030	26040

....|....|....|....|....|....|....|....|....|....|....|....|

**Atalantia_kwangtungens** **atgattcagttccaaagccaaagatcgtagttctcgaacgagcaatcgaaaagattctgg**

**Citrus_reticulata_1** **atgattcagttccaaagccaaagatcgtagttctcgaacgagcaatcgaaaagattctgg**

**Citrus_reticulata_2** **atgattcagttccaaagccaaagatcgtagttctcgaacgagcaatcgaaaagattctgg**

**Citrus_trifoliata_3** **atgattcagttccaaagccaaagatcgtagttctcgaacgagcaatcgaaaagattctgg**

**Citrus_aurantiifolia** **atgattcagttccaaagccaaagatcgtagttctcgaacgagcaatcgaaaagattctgg**

**Citrus_aurantium** **atgattcagttccaaagccaaagatcgtagttctcgaacgagcaatcgaaaagattctgg**

**Citrus_cavaleriei** **atgattcagttccaaagccaaagatcgtagttctcgaacgagcaatcgaaaagattctgg**

**Citrus_hongheensis** **atgattcagttccaaagccaaagatcgtagttctcgaacgagcaatcgaaaagattctgg**

**Citrus_limon** **atgattcagttccaaagccaaagatcgtagttctcgaacgagcaatcgaaaagattctgg**

**Citrus_maxima** **atgattcagttccaaagccaaagatcgtagttctcgaacgagcaatcgaaaagattctgg**

**Citrus_medica** **atgattcagttccaaagccaaagatcgtagttctcgaacgagcaatcgaaaagattctgg**

**Citrus_polytrifolia** **atgattcagttccaaagccaaagatcgtagttctcgaacgagcaatcgaaaagattctgg**

**Citrus_reticulata** **atgattcagttccaaagccaaagatcgtagttctcgaacgagcaatcgaaaagattctgg**

**Citrus_sinensis** **atgattcagttccaaagccaaagatcgtagttctcgaacgagcaatcgaaaagattctgg**

**Fortunella_bawangica_1** **atgattcagttccaaagccaaagatcgtagttctcgaacgagcaatcgaaaagattctgg**

**Fortunella_bawangica_2** **atgattcagttccaaagccaaagatcgtagttctcgaacgagcaatcgaaaagattctgg**

**Fortunella_hindsii_1** **atgattcagttccaaagccaaagatcgtagttctcgaacgagcaatcgaaaagattctgg**

**Fortunella_hindsii_2** **atgattcagttccaaagccaaagatcgtagttctcgaacgagcaatcgaaaagattctgg**

**Fortunella_hindsii_3** **atgattcagttccaaagccaaagatcgtagttctcgaacgagcaatcgaaaagattctgg**

**Fortunella_japonica_1** **atgattcagttccaaagccaaagatcgtagttctcgaacgagcaatcgaaaagattctgg**

**Fortunella_japonica_2** **atgattcagttccaaagccaaagatcgtagttctcgaacgagcaatcgaaaagattctgg**

**Fortunella_japonica_3** **atgattcagttccaaagccaaagatcgtagttctcgaacgagcaatcgaaaagattctgg**

**Fortunella_japonica_4** **atgattcagttccaaagccaaagatcgtagttctcgaacgagcaatcgaaaagattctgg**

**Fortunella_japonica_5** **atgattcagttccaaagccaaagatcgtagttctcgaacgagcaatcgaaaagattctgg**

**Fortunella_japonica_6** **atgattcagttccaaagccaaagatcgtagttctcgaacgagcaatcgaaaagattctgg**

**Fortunella_japonica_7** **atgattcagttccaaagccaaagatcgtagttctcgaacgagcaatcgaaaagattctgg**

**Fortunella_japonica_8** **atgattcagttccaaagccaaagatcgtagttctcgaacgagcaatcgaaaagattctgg**

**Fortunella_japonica_9** **atgattcagttccaaagccaaagatcgtagttctcgaacgagcaatcgaaaagattctgg**

**Fortunella_japonica_10** **atgattcagttccaaagccaaagatcgtagttctcgaacgagcaatcgaaaagattctgg**

**Fortunella_japonica_11** **atgattcagttccaaagccaaagatcgtagttctcgaacgagcaatcgaaaagattctgg**

**Fortunella_japonica_12** **atgattcagttccaaagccaaagatcgtagttctcgaacgagcaatcgaaaagattctgg**

**Fortunella_margarita_1** **atgattcagttccaaagccaaagatcgtagttctcgaacgagcaatcgaaaagattctgg**

**Fortunella_margarita_2** **atgattcagttccaaagccaaagatcgtagttctcgaacgagcaatcgaaaagattctgg**

**Fortunella_venosa_1** **atgattcagttccaaagccaaagatcgtagttctcgaacgagcaatcgaaaagattctgg**

**Fortunella_venosa_2** **atgattcagttccaaagccaaagatcgtagttctcgaacgagcaatcgaaaagattctgg**

**Fortunella_venosa_3** **atgattcagttccaaagccaaagatcgtagttctcgaacgagcaatcgaaaagattctgg**

26050	26060	26070	26080	26090	26100

....|....|....|....|....|....|....|....|....|....|....|....|

**Atalantia_kwangtungens** **agcatcttccgggttaggtattgtttctccaatgattgtagtaccaagtacttcttggcg**

**Citrus_reticulata_1** **agcatcttccgggttaggtattgtttctccaatgattgtagtaccaagtacttcttggcg**

**Citrus_reticulata_2** **agcatcttccgggttaggtattgtttctccaatgattgtagtaccaagtacttcttggcg**

**Citrus_trifoliata_3** **agcatcttccgggttaggtattgtttctccaatgattgtagtaccaagtacttcttggcg**

**Citrus_aurantiifolia** **agcatcttccgggttaggtattgtttctccaatgattgtagtaccaagtacttcttggcg**

**Citrus_aurantium** **agcatcttccgggttaggtattgtttctccaatgattgtagtaccaagtacttcttggcg**

**Citrus_cavaleriei** **agcatcttccgggttaggtattgtttctccaatgattgtagtaccaagtacttcttggcg**

**Citrus_hongheensis** **agcatcttccgggttaggtattgtttctccaatgattgtagtagcaagtacttcttggcg**

**Citrus_limon** **agcatcttccgggttaggtattgtttctccaatgattgtagtaccaagtacttcttggcg**

**Citrus_maxima** **agcatcttccgggttaggtattgtttctccaatgattgtagtaccaagtacttcttggcg**

**Citrus_medica** **agcatcttccgggttaggtattgtttctccaatgattgtagtaccaagtacttcttggcg**

**Citrus_polytrifolia** **agcatcttccgggttaggtattgtttctccaatgattgtagtaccaagtacttcttggcg**

**Citrus_reticulata** **agcatcttccgggttaggtattgtttctccaatgattgtagtaccaagtacttcttggcg**

**Citrus_sinensis** **agcatcttccgggttaggtattgtttctccaatgattgtagtaccaagtacttcttggcg**

**Fortunella_bawangica_1** **agcatcttccgggttaggtattgtttctccaatgattgtagtaccaagtacttcttggcg**

**Fortunella_bawangica_2** **agcatcttccgggttaggtattgtttctccaatgattgtagtaccaagtacttcttggcg**

**Fortunella_hindsii_1** **agcatcttccgggttaggtattgtttctccaatgattgtagtaccaagtacttcttggcg**

**Fortunella_hindsii_2** **agcatcttccgggttaggtattgtttctccaatgattgtagtaccaagtacttcttggcg**

**Fortunella_hindsii_3** **agcatcttccgggttaggtattgtttctccaatgattgtagtaccaagtacttcttggcg**

**Fortunella_japonica_1** **agcatcttccgggttaggtattgtttctccaatgattgtagtaccaagtacttcttggcg**

**Fortunella_japonica_2** **agcatcttccgggttaggtattgtttctccaatgattgtagtaccaagtacttcttggcg**

**Fortunella_japonica_3** **agcatcttccgggttaggtattgtttctccaatgattgtagtaccaagtacttcttggcg**

**Fortunella_japonica_4** **agcatcttccgggttaggtattgtttctccaatgattgtagtaccaagtacttcttggcg**

**Fortunella_japonica_5** **agcatcttccgggttaggtattgtttctccaatgattgtagtaccaagtacttcttggcg**

**Fortunella_japonica_6** **agcatcttccgggttaggtattgtttctccaatgattgtagtaccaagtacttcttggcg**

**Fortunella_japonica_7** **agcatcttccgggttaggtattgtttctccaatgattgtagtaccaagtacttcttggcg**

**Fortunella_japonica_8** **agcatcttccgggttaggtattgtttctccaatgattgtagtaccaagtacttcttggcg**

**Fortunella_japonica_9** **agcatcttccgggttaggtattgtttctccaatgattgtagtaccaagtacttcttggcg**

**Fortunella_japonica_10** **agcatcttccgggttaggtattgtttctccaatgattgtagtaccaagtacttcttggcg**

**Fortunella_japonica_11** **agcatcttccgggttaggtattgtttctccaatgattgtagtaccaagtacttcttggcg**

**Fortunella_japonica_12** **agcatcttccgggttaggtattgtttctccaatgattgtagtaccaagtacttcttggcg**

**Fortunella_margarita_1** **agcatcttccgggttaggtattgtttctccaatgattgtagtaccaagtacttcttggcg**

**Fortunella_margarita_2** **agcatcttccgggttaggtattgtttctccaatgattgtagtaccaagtacttcttggcg**

**Fortunella_venosa_1** **agcatcttccgggttaggtattgtttctccaatgattgtagtaccaagtacttcttggcg**

**Fortunella_venosa_2** **agcatcttccgggttaggtattgtttctccaatgattgtagtaccaagtacttcttggcg**

**Fortunella_venosa_3** **agcatcttccgggttaggtattgtttctccaatgattgtagtaccaagtacttcttggcg**

26110	26120	26130	26140	26150	26160

....|....|....|....|....|....|....|....|....|....|....|....|

**Atalantia_kwangtungens** **cgctcgaatatgatcagatttataagtaagcatctcttgtaaaatatgagcaacaccaaa**

**Citrus_reticulata_1** **cgctctaatatgatcagatttataagtaagcatctcttgtaaaatatgagcaacaccaaa**

**Citrus_reticulata_2** **cgctctaatatgatcagatttataagtaagcatctcttgtaaaatatgagcaacaccaaa**

**Citrus_trifoliata_3** **cgctctaatatgatcagatttataagtaagcatctcttgtaaaatatgagcaacaccaaa**

**Citrus_aurantiifolia** **cgctctaatatgatcagatttataagtaagcatctcttgtaaaatatgagcaacaccaaa**

**Citrus_aurantium** **cgctctaatatgatcagatttataagtaagcatctcttgtaaaatatgagcaacaccaaa**

**Citrus_cavaleriei** **cgctctaatatgatcagatttataagtaagcatctcttgtaaaatatgagcaacaccaaa**

**Citrus_hongheensis** **cgctctaatatgatcagatttataagtaagcatctcttgtaaaatatgagcaacaccaaa**

**Citrus_limon** **cgctctaatatgatcagatttataagtaagcatctcttgtaaaatatgagcaacaccaaa**

**Citrus_maxima** **cgctctaatatgatcagatttataagtaagcatctcttgtaaaatatgagcaacaccaaa**

**Citrus_medica** **cgctctaatatgatcagatttataagtaagcatctcttgtaaaatatgagcaacaccaaa**

**Citrus_polytrifolia** **cgctctaatatgatcagatttataagtaagcatctcttgtaaaatatgagcaacaccaaa**

**Citrus_reticulata** **cgctctaatatgatcagatttataagtaagcatctcttgtaaaatatgagcaacaccaaa**

**Citrus_sinensis** **cgctctaatatgatcagatttataagtaagcatctcttgtaaaatatgagcaacaccaaa**

**Fortunella_bawangica_1** **cgctctaatatgatcagatttataagtaagcatctcttgtaaaatatgagcaacaccaaa**

**Fortunella_bawangica_2** **cgctctaatatgatcagatttataagtaagcatctcttgtaaaatatgagcaacaccaaa**

**Fortunella_hindsii_1** **cgctctaatatgatcagatttataagtaagcatctcttgtaaaatatgagcaacaccaaa**

**Fortunella_hindsii_2** **cgctctaatatgatcagatttataagtaagcatctcttgtaaaatatgagcaacaccaaa**

**Fortunella_hindsii_3** **cgctctaatatgatcagatttataagtaagcatctcttgtaaaatatgagcaacaccaaa**

**Fortunella_japonica_1** **cgctctaatatgatcagatttataagtaagcatctcttgtaaaatatgagcaacaccaaa**

**Fortunella_japonica_2** **cgctctaatatgatcagatttataagtaagcatctcttgtaaaatatgagcaacaccaaa**

**Fortunella_japonica_3** **cgctctaatatgatcagatttataagtaagcatctcttgtaaaatatgagcaacaccaaa**

**Fortunella_japonica_4** **cgctctaatatgatcagatttataagtaagcatctcttgtaaaatatgagcaacaccaaa**

**Fortunella_japonica_5** **cgctctaatatgatcagatttataagtaagcatctcttgtaaaatatgagcaacaccaaa**

**Fortunella_japonica_6** **cgctctaatatgatcagatttataagtaagcatctcttgtaaaatatgagcaacaccaaa**

**Fortunella_japonica_7** **cgctctaatatgatcagatttataagtaagcatctcttgtaaaatatgagcaacaccaaa**

**Fortunella_japonica_8** **cgctctaatatgatcagatttataagtaagcatctcttgtaaaatatgagcaacaccaaa**

**Fortunella_japonica_9** **cgctctaatatgatcagatttataagtaagcatctcttgtaaaatatgagcaacaccaaa**

**Fortunella_japonica_10** **cgctctaatatgatcagatttataagtaagcatctcttgtaaaatatgagcaacaccaaa**

**Fortunella_japonica_11** **cgctctaatatgatcagatttataagtaagcatctcttgtaaaatatgagcaacaccaaa**

**Fortunella_japonica_12** **cgctctaatatgatcagatttataagtaagcatctcttgtaaaatatgagcaacaccaaa**

**Fortunella_margarita_1** **cgctctaatatgatcagatttataagtaagcatctcttgtaaaatatgagcaacaccaaa**

**Fortunella_margarita_2** **cgctctaatatgatcagatttataagtaagcatctcttgtaaaatatgagcaacaccaaa**

**Fortunella_venosa_1** **cgctctaatatgatcagatttataagtaagcatctcttgtaaaatatgagcaacaccaaa**

**Fortunella_venosa_2** **cgctctaatatgatcagatttataagtaagcatctcttgtaaaatatgagcaacaccaaa**

**Fortunella_venosa_3** **cgctctaatatgatcagatttataagtaagcatctcttgtaaaatatgagcaacaccaaa**

26170	26180	26190	26200	26210	26220

....|....|....|....|....|....|....|....|....|....|....|....|

**Atalantia_kwangtungens** **gccctctagagcccaaacctccatttctcctacccgttgtcccccttgtttggaccttcc**

**Citrus_reticulata_1** **gccctctagagcccaaacctccatttctcctacccgttgtcccccttgtttggaccttcc**

**Citrus_reticulata_2** **gccctctagagcccaaacctccatttctcctacccgttgtcccccttgtttggaccttcc**

**Citrus_trifoliata_3** **gccctctagagcccaaacctccatttcgcctacccgttgtcccccttgtttggaccttcc**

**Citrus_aurantiifolia** **gccctctagagcccaaacctccatttctcctacccgttgtcccccttgtttggaccttcc**

**Citrus_aurantium** **gccctctagagcccaaacctccatttctcctacccgttgtcccccttgtttggaccttcc**

**Citrus_cavaleriei** **gccctctagagcccaaacctccatttctcctacccgttgccccccttgtttggaccttcc**

**Citrus_hongheensis** **gccctctagagcccaaacctccatttctcctacccgttgccccccttgtttggaccttcc**

**Citrus_limon** **gccctctagagcccaaacctccatttctcctacccgttgtcccccttgtttggaccttcc**

**Citrus_maxima** **gccctctagagcccaaacctccatttctcctacccgttgtcccccttgtttggaccttcc**

**Citrus_medica** **gccctctagagcccaaacctccatttctcctacccgttgtcccccttgtttggaccttcc**

**Citrus_polytrifolia** **gccctctagagcccaaacctccatttcgcctacccgttgtcccccttgtttggaccttcc**

**Citrus_reticulata** **gccctctagagcccaaacctccatttctcctacccgttgtcccccttgtttggaccttcc**

**Citrus_sinensis** **gccctctagagcccaaacctccatttctcctacccgttgtcccccttgtttggaccttcc**

**Fortunella_bawangica_1** **gccctctagagcccaaacctccatttctcctacccgttgtcccccttgtttggaccttcc**

**Fortunella_bawangica_2** **gccctctagagcccaaacctccatttctcctacccgttgtcccccttgtttggaccttcc**

**Fortunella_hindsii_1** **gccctctagagcccaaacctccatttctcctacccgttgtcccccttgtttggaccttcc**

**Fortunella_hindsii_2** **gccctctagagcccaaacctccatttctcctacccgttgtcccccttgtttggaccttcc**

**Fortunella_hindsii_3** **gccctctagagcccaaacctccatttctcctacccgttgtcccccttgtttggaccttcc**

**Fortunella_japonica_1** **gccctctagagcccaaacctccatttctcctacccgttgtcccccttgtttggaccttcc**

**Fortunella_japonica_2** **gccctctagagcccaaacctccatttctcctacccgttgtcccccttgtttggaccttcc**

**Fortunella_japonica_3** **gccctctagagcccaaacctccatttctcctacccgttgtcccccttgtttggaccttcc**

**Fortunella_japonica_4** **gccctctagagcccaaacctccatttctcctacccgttgtcccccttgtttggaccttcc**

**Fortunella_japonica_5** **gccctctagagcccaaacctccatttctcctacccgttgtcccccttgtttggaccttcc**

**Fortunella_japonica_6** **gccctctagagcccaaacctccatttctcctacccgttgtcccccttgtttggaccttcc**

**Fortunella_japonica_7** **gccctctagagcccaaacctccatttctcctacccgttgtcccccttgtttggaccttcc**

**Fortunella_japonica_8** **gccctctagagcccaaacctccatttctcctacccgttgtcccccttgtttggaccttcc**

**Fortunella_japonica_9** **gccctctagagcccaaacctccatttctcctacccgttgtcccccttgtttggaccttcc**

**Fortunella_japonica_10** **gccctctagagcccaaacctccatttctcctacccgttgtcccccttgtttggaccttcc**

**Fortunella_japonica_11** **gccctctagagcccaaacctccatttctcctacccgttgtcccccttgtttggaccttcc**

**Fortunella_japonica_12** **gccctctagagcccaaacctccatttctcctacccgttgtcccccttgtttggaccttcc**

**Fortunella_margarita_1** **gccctctagagcccaaacctccatttctcctacccgttgtcccccttgtttggaccttcc**

**Fortunella_margarita_2** **gccctctagagcccaaacctccatttctcctacccgttgtcccccttgtttggaccttcc**

**Fortunella_venosa_1** **gccctctagagcccaaacctccatttctcctacccgttgtcccccttgtttggaccttcc**

**Fortunella_venosa_2** **gccctctagagcccaaacctccatttctcctacccgttgtcccccttgtttggaccttcc**

**Fortunella_venosa_3** **gccctctagagcccaaacctccatttctcctacccgttgtcccccttgtttggaccttcc**

26230	26240	26250	26260	26270	26280

....|....|....|....|....|....|....|....|....|....|....|....|

**Atalantia_kwangtungens** **tctaaggggttgttgggtaacaagtgcataatgcccactggaacgtccgtgtactttatc**

**Citrus_reticulata_1** **tctaaggggttgttgggtaacaagtgcataatgcccactggaacgtccgtgtactttatc**

**Citrus_reticulata_2** **tctaaggggttgttgggtaacaagtgcataatgcccactggaacgtccgtgtactttatc**

**Citrus_trifoliata_3** **tctaaggggttgttgggtaacaagtgcataatgcccactggaacgtccgtgtactttatc**

**Citrus_aurantiifolia** **tctaaggggttgttgggtaacaagtgcataatgcccactggaacgtccgtgtactttatc**

**Citrus_aurantium** **tctaaggggttgttgggtaacaagtgcataatgcccactggaacgtccgtgtactttatc**

**Citrus_cavaleriei** **tctaaggggttgttgggtaacaagtgcataatgcccactggaacgtccgtgtactttatc**

**Citrus_hongheensis** **tctaaggggttgttgggtaacaagtgcataatgcccactggaacgtccgtgtactttatc**

**Citrus_limon** **tctaaggggttgttgggtaacaagtgcataatgcccactggaacgtccgtgtactttatc**

**Citrus_maxima** **tctaaggggttgttgggtaacaagtgcataatgcccactggaacgtccgtgtactttatc**

**Citrus_medica** **tctaaggggttgttgggtaacaagtgcataatgcccactggaacgtccgtgtactttatc**

**Citrus_polytrifolia** **tctaaggggttgttgggtaacaagtgcataatgcccactggaacgtccgtgtactttatc**

**Citrus_reticulata** **tctaaggggttgttgggtaacaagtgcataatgcccactggaacgtccgtgtactttatc**

**Citrus_sinensis** **tctaaggggttgttgggtaacaagtgcataatgcccactggaacgtccgtgtactttatc**

**Fortunella_bawangica_1** **tctaaggggttgttgggtaacaagtgcataatgcccactggaacgtccgtgtactttatc**

**Fortunella_bawangica_2** **tctaaggggttgttgggtaacaagtgcataatgcccactggaacgtccgtgtactttatc**

**Fortunella_hindsii_1** **tctaaggggttgttgggtaacaagtgcataatgcccactggaacgtccgtgtactttatc**

**Fortunella_hindsii_2** **tctaaggggttgttgggtaacaagtgcataatgcccactggaacgtccgtgtactttatc**

**Fortunella_hindsii_3** **tctaaggggttgttgggtaacaagtgcataatgcccactggaacgtccgtgtactttatc**

**Fortunella_japonica_1** **tctaaggggttgttgggtaacaagtgcataatgcccactggaacgtccgtgtactttatc**

**Fortunella_japonica_2** **tctaaggggttgttgggtaacaagtgcataatgcccactggaacgtccgtgtactttatc**

**Fortunella_japonica_3** **tctaaggggttgttgggtaacaagtgcataatgcccactggaacgtccgtgtactttatc**

**Fortunella_japonica_4** **tctaaggggttgttgggtaacaagtgcataatgcccactggaacgtccgtgtactttatc**

**Fortunella_japonica_5** **tctaaggggttgttgggtaacaagtgcataatgcccactggaacgtccgtgtactttatc**

**Fortunella_japonica_6** **tctaaggggttgttgggtaacaagtgcataatgcccactggaacgtccgtgtactttatc**

**Fortunella_japonica_7** **tctaaggggttgttgggtaacaagtgcataatgcccactggaacgtccgtgtactttatc**

**Fortunella_japonica_8** **tctaaggggttgttgggtaacaagtgcataatgcccactggaacgtccgtgtactttatc**

**Fortunella_japonica_9** **tctaaggggttgttgggtaacaagtgcataatgcccactggaacgtccgtgtactttatc**

**Fortunella_japonica_10** **tctaaggggttgttgggtaacaagtgcataatgcccactggaacgtccgtgtactttatc**

**Fortunella_japonica_11** **tctaaggggttgttgggtaacaagtgcataatgcccactggaacgtccgtgtactttatc**

**Fortunella_japonica_12** **tctaaggggttgttgggtaacaagtgcataatgcccactggaacgtccgtgtactttatc**

**Fortunella_margarita_1** **tctaaggggttgttgggtaacaagtgcataatgcccactggaacgtccgtgtactttatc**

**Fortunella_margarita_2** **tctaaggggttgttgggtaacaagtgcataatgcccactggaacgtccgtgtactttatc**

**Fortunella_venosa_1** **tctaaggggttgttgggtaacaagtgcataatgcccactggaacgtccgtgtactttatc**

**Fortunella_venosa_2** **tctaaggggttgttgggtaacaagtgcataatgcccactggaacgtccgtgtactttatc**

**Fortunella_venosa_3** **tctaaggggttgttgggtaacaagtgcataatgcccactggaacgtccgtgtactttatc**

26290	26300	26310	26320	26330	26340

....|....|....|....|....|....|....|....|....|....|....|....|

**Atalantia_kwangtungens** **atcaacttgatgaattaatttcaagatataaggctttcctattagaacaggttcttcaaa**

**Citrus_reticulata_1** **atcaacttgatgaattaatttcaagatataaggctttcctattagaacaggttcttcaaa**

**Citrus_reticulata_2** **atcaacttgatgaattaatttcaagatataaggctttcctattagaacaggttcttcaaa**

**Citrus_trifoliata_3** **atcaacttgatgaattaatttcaagatataaggctttcctattagaacaggttcttcaaa**

**Citrus_aurantiifolia** **atcaacttgatgaattaatttcaagatataaggctttcctattagaacaggttcttcaaa**

**Citrus_aurantium** **atcaacttgatgaattaatttcaagatataaggctttcctattagaacaggttcttcaaa**

**Citrus_cavaleriei** **atcaacttgatgaattaatttcaagatataaggctttcctattagaacaggttcttcaaa**

**Citrus_hongheensis** **atcaacttgatgaattaatttcaagatataaggctttcctattagaacaggttcttcaaa**

**Citrus_limon** **atcaacttgatgaattaatttcaagatataaggctttcctattagaacaggttcttcaaa**

**Citrus_maxima** **atcaacttgatgaattaatttcaagatataaggctttcctattagaacaggttcttcaaa**

**Citrus_medica** **atcaacttgatgaattaatttcaagatataaggctttcctattagaacaggttcttcaaa**

**Citrus_polytrifolia** **atcaacttgatgaattaatttcaagatataaggctttcctattagaacaggttcttcaaa**

**Citrus_reticulata** **atcaacttgatgaattaatttcaagatataaggctttcctattagaacaggttcttcaaa**

**Citrus_sinensis** **atcaacttgatgaattaatttcaagatataaggctttcctattagaacaggttcttcaaa**

**Fortunella_bawangica_1** **atcaacttgatgaattaatttcaagatataaggctttcctattagaacaggttcttcaaa**

**Fortunella_bawangica_2** **atcaacttgatgaattaatttcaagatataaggctttcctattagaacaggttcttcaaa**

**Fortunella_hindsii_1** **atcaacttgatgaattaatttcaagatataaggctttcctattagaacaggttcttcaaa**

**Fortunella_hindsii_2** **atcaacttgatgaattaatttcaagatataaggctttcctattagaacaggttcttcaaa**

**Fortunella_hindsii_3** **atcaacttgatgaattaatttcaagatataaggctttcctattagaacaggttcttcaaa**

**Fortunella_japonica_1** **atcaacttgatgaattaatttcaagatataaggctttcctattagaacaggttcttcaaa**

**Fortunella_japonica_2** **atcaacttgatgaattaatttcaagatataaggctttcctattagaacaggttcttcaaa**

**Fortunella_japonica_3** **atcaacttgatgaattaatttcaagatataaggctttcctattagaacaggttcttcaaa**

**Fortunella_japonica_4** **atcaacttgatgaattaatttcaagatataaggctttcctattagaacaggttcttcaaa**

**Fortunella_japonica_5** **atcaacttgatgaattaatttcaagatataaggctttcctattagaacaggttcttcaaa**

**Fortunella_japonica_6** **atcaacttgatgaattaatttcaagatataaggctttcctattagaacaggttcttcaaa**

**Fortunella_japonica_7** **atcaacttgatgaattaatttcaagatataaggctttcctattagaacaggttcttcaaa**

**Fortunella_japonica_8** **atcaacttgatgaattaatttcaagatataaggctttcctattagaacaggttcttcaaa**

**Fortunella_japonica_9** **atcaacttgatgaattaatttcaagatataaggctttcctattagaacaggttcttcaaa**

**Fortunella_japonica_10** **atcaacttgatgaattaatttcaagatataaggctttcctattagaacaggttcttcaaa**

**Fortunella_japonica_11** **atcaacttgatgaattaatttcaagatataaggctttcctattagaacaggttcttcaaa**

**Fortunella_japonica_12** **atcaacttgatgaattaatttcaagatataaggctttcctattagaacaggttcttcaaa**

**Fortunella_margarita_1** **atcaacttgatgaattaatttcaagatataaggctttcctattagaacaggttcttcaaa**

**Fortunella_margarita_2** **atcaacttgatgaattaatttcaagatataaggctttcctattagaacaggttcttcaaa**

**Fortunella_venosa_1** **atcaacttgatgaattaatttcaagatataaggctttcctattagaacaggttcttcaaa**

**Fortunella_venosa_2** **atcaacttgatgaattaatttcaagatataaggctttcctattagaacaggttcttcaaa**

**Fortunella_venosa_3** **atcaacttgatgaattaatttcaagatataaggctttcctattagaacaggttcttcaaa**

26350	26360	26370	26380	26390	26400

....|....|....|....|....|....|....|....|....|....|....|....|

**Atalantia_kwangtungens** **aggatctcccgttcgtccatcaaatattctgctttttcccgggtattccggctcaaatac**

**Citrus_reticulata_1** **aggatctcccgttcgtccatcaaatattctgctttttcccgggtattccggctcaaatac**

**Citrus_reticulata_2** **aggatctcccgttcgtccatcaaatattctgctttttcccgggtattccggctcaaatac**

**Citrus_trifoliata_3** **aggatctcccgttcgtccatcaaatattctgctttttcccgggtattccggctcaaatac**

**Citrus_aurantiifolia** **aggatctcccgttcgtccatcaaatattctgctttttcccgggtattccggctcaaatac**

**Citrus_aurantium** **aggatctcccgttcgtccatcaaatattctgctttttcccgggtattccggctcaaatac**

**Citrus_cavaleriei** **aggatctcccgttcgtccatcaaatattctgctttttcccgggtattccggctcaaatac**

**Citrus_hongheensis** **aggatctcccgttcgtccatcaaatattctgctttttcccgggtattccggctcaaatac**

**Citrus_limon** **aggatctcccgttcgtccatcaaatattctgctttttcccgggtattccggctcaaatac**

**Citrus_maxima** **aggatctcccgttcgtccatcaaatattctgctttttcccgggtattccggctcaaatac**

**Citrus_medica** **aggatctcccgttcgtccatcaaatattctgctttttcccgggtattccggctcaaatac**

**Citrus_polytrifolia** **aggatctcccgttcgtccatcaaatattctgctttttcccgggtattccggctcaaatac**

**Citrus_reticulata** **aggatctcccgttcgtccatcaaatattctgctttttcccgggtattccggctcaaatac**

**Citrus_sinensis** **aggatctcccgttcgtccatcaaatattctgctttttcccgggtattccggctcaaatac**

**Fortunella_bawangica_1** **aggatctcccgttcgtccatcaaatattctgctttttcccgggtattccggctcaaatac**

**Fortunella_bawangica_2** **aggatctcccgttcgtccatcaaatattctgctttttcccgggtattccggctcaaatac**

**Fortunella_hindsii_1** **aggatctcccgttcgtccatcaaatattctgctttttcccgggtattccggctcaaatac**

**Fortunella_hindsii_2** **aggatctcccgttcgtccatcaaatattctgctttttcccgggtattccggctcaaatac**

**Fortunella_hindsii_3** **aggatctcccgttcgtccatcaaatattctgctttttcccgggtattccggctcaaatac**

**Fortunella_japonica_1** **aggatctcccgttcgtccatcaaatattctgctttttcccgggtattccggctcaaatac**

**Fortunella_japonica_2** **aggatctcccgttcgtccatcaaatattctgctttttcccgggtattccggctcaaatac**

**Fortunella_japonica_3** **aggatctcccgttcgtccatcaaatattctgctttttcccgggtattccggctcaaatac**

**Fortunella_japonica_4** **aggatctcccgttcgtccatcaaatattctgctttttcccgggtattccggctcaaatac**

**Fortunella_japonica_5** **aggatctcccgttcgtccatcaaatattctgctttttcccgggtattccggctcaaatac**

**Fortunella_japonica_6** **aggatctcccgttcgtccatcaaatattctgctttttcccgggtattccggctcaaatac**

**Fortunella_japonica_7** **aggatctcccgttcgtccatcaaatattctgctttttcccgggtattccggctcaaatac**

**Fortunella_japonica_8** **aggatctcccgttcgtccatcaaatattctgctttttcccgggtattccggctcaaatac**

**Fortunella_japonica_9** **aggatctcccgttcgtccatcaaatattctgctttttcccgggtattccggctcaaatac**

**Fortunella_japonica_10** **aggatctcccgttcgtccatcaaatattctgctttttcccgggtattccggctcaaatac**

**Fortunella_japonica_11** **aggatctcccgttcgtccatcaaatattctgctttttcccgggtattccggctcaaatac**

**Fortunella_japonica_12** **aggatctcccgttcgtccatcaaatattctgctttttcccgggtattccggctcaaatac**

**Fortunella_margarita_1** **aggatctcccgttcgtccatcaaatattctgctttttcccgggtattccggctcaaatac**

**Fortunella_margarita_2** **aggatctcccgttcgtccatcaaatattctgctttttcccgggtattccggctcaaatac**

**Fortunella_venosa_1** **aggatctcccgttcgtccatcaaatattctgctttttcccgggtattccggctcaaatac**

**Fortunella_venosa_2** **aggatctcccgttcgtccatcaaatattctgctttttcccgggtattccggctcaaatac**

**Fortunella_venosa_3** **aggatctcccgttcgtccatcaaatattctgctttttcccgggtattccggctcaaatac**

26410	26420	26430	26440	26450	26460

....|....|....|....|....|....|....|....|....|....|....|....|

**Atalantia_kwangtungens** **ccatggattcgcggtttgcttactggcttcatataattcagaaaacacgagttttctcga**

**Citrus_reticulata_1** **ccatggattcgcggtttgcttactggcttcatataattcagaaaacacgagttttctcga**

**Citrus_reticulata_2** **ccatggattcgcggtttgcttactggcttcatataattcagaaaacacgagttttctcga**

**Citrus_trifoliata_3** **ccatggattcgcggtttgcttactggcttcatataattcagaaaacacgagttttctcga**

**Citrus_aurantiifolia** **ccatggattcgcggtttgcttactggcttcatataattcagaaaacacgagttttctcga**

**Citrus_aurantium** **ccatggattcgcggtttgcttactggcttcatataattcagaaaacacgagttttctcga**

**Citrus_cavaleriei** **ccatggattcgcggtttgcttactggcttcatataattcagaaaacacgagttttctcga**

**Citrus_hongheensis** **ccatggattcgcggtttgcttactggcttcatataattcagaaaacacgagttttctcga**

**Citrus_limon** **ccatggattcgcggtttgcttactggcttcatataattcagaaaacacgagttttctcga**

**Citrus_maxima** **ccatggattcgcggtttgcttactggcttcatataattcagaaaacacgagttttctcga**

**Citrus_medica** **ccatggattcgcggtttgcttacaggcttcatataattcagaaaacacgagttttctcga**

**Citrus_polytrifolia** **ccatggattcgcggtttgcttactggcttcatataattcagaaaacacgagttttctcga**

**Citrus_reticulata** **ccatggattcgcggtttgcttactggcttcatataattcagaaaacacgagttttctcga**

**Citrus_sinensis** **ccatggattcgcggtttgcttactggcttcatataattcagaaaacacgagttttctcga**

**Fortunella_bawangica_1** **ccatggattcgcggtttgcttactggcttcatataattcagaaaacacgagttttctcga**

**Fortunella_bawangica_2** **ccatggattcgcggtttgcttactggcttcatataattcagaaaacacgagttttctcga**

**Fortunella_hindsii_1** **ccatggattcgcggtttgcttactggcttcatataattcagaaaacacgagttttctcga**

**Fortunella_hindsii_2** **ccatggattcgcggtttgcttactggcttcatataattcagaaaacacgagttttctcga**

**Fortunella_hindsii_3** **ccatggattcgcggtttgcttactggcttcatataattcagaaaacacgagttttctcga**

**Fortunella_japonica_1** **ccatggattcgcggtttgcttactggcttcatataattcagaaaacacgagttttctcga**

**Fortunella_japonica_2** **ccatggattcgcggtttgcttactggcttcatataattcagaaaacacgagttttctcga**

**Fortunella_japonica_3** **ccatggattcgcggtttgcttactggcttcatataattcagaaaacacgagttttctcga**

**Fortunella_japonica_4** **ccatggattcgcggtttgcttactggcttcatataattcagaaaacacgagttttctcga**

**Fortunella_japonica_5** **ccatggattcgcggtttgcttactggcttcatataattcagaaaacacgagttttctcga**

**Fortunella_japonica_6** **ccatggattcgcggtttgcttactggcttcatataattcagaaaacacgagttttctcga**

**Fortunella_japonica_7** **ccatggattcgcggtttgcttactggcttcatataattcagaaaacacgagttttctcga**

**Fortunella_japonica_8** **ccatggattcgcggtttgcttactggcttcatataattcagaaaacacgagttttctcga**

**Fortunella_japonica_9** **ccatggattcgcggtttgcttactggcttcatataattcagaaaacacgagttttctcga**

**Fortunella_japonica_10** **ccatggattcgcggtttgcttactggcttcatataattcagaaaacacgagttttctcga**

**Fortunella_japonica_11** **ccatggattcgcggtttgcttactggcttcatataattcagaaaacacgagttttctcga**

**Fortunella_japonica_12** **ccatggattcgcggtttgcttactggcttcatataattcagaaaacacgagttttctcga**

**Fortunella_margarita_1** **ccatggattcgcggtttgcttactggcttcatataattcagaaaacacgagttttctcga**

**Fortunella_margarita_2** **ccatggattcgcggtttgcttactggcttcatataattcagaaaacacgagttttctcga**

**Fortunella_venosa_1** **ccatggattcgcggtttgcttactggcttcatataattcagaaaacacgagttttctcga**

**Fortunella_venosa_2** **ccatggattcgcggtttgcttactggcttcatataattcagaaaacacgagttttctcga**

**Fortunella_venosa_3** **ccatggattcgcggtttgcttactggcttcatataattcagaaaacacgagttttctcga**

26470	26480	26490	26500	26510	26520

....|....|....|....|....|....|....|....|....|....|....|....|

**Atalantia_kwangtungens** **agcctcttgttcatatctctcatcaaaaggtgctattcgataatgtcgatttagcagacc**

**Citrus_reticulata_1** **agcctcttgttcatatctctcatcaaaaggtgctattcgataatgtcgatttagcagacc**

**Citrus_reticulata_2** **agcctcttgttcatatctctcatcaaaaggtgctattcgataatgtcgatttagcagacc**

**Citrus_trifoliata_3** **agcctcttgttcatatctctcatcaaaaggtgctattcgataatgtcgatttagcagacc**

**Citrus_aurantiifolia** **agcctcttgttcatatctctcatcaaaaggtgctattcgataatgtcgatttagcagacc**

**Citrus_aurantium** **agcctcttgttcatatctctcatcaaaaggtgctattcgataatgtcgatttagcagacc**

**Citrus_cavaleriei** **agcctcttgttcatatctctcatcaaaaggtgctattcgataatgtcgatttagcagacc**

**Citrus_hongheensis** **agcctcttgttcatatctctcatcaaaaggtgctattcgataatgtcgatttagcagacc**

**Citrus_limon** **agcctcttgttcatatctctcatcaaaaggtgctattcgataatgtcgatttagcagacc**

**Citrus_maxima** **agcctcttgttcatatctctcatcaaaaggtgctattcgataatgtcgatttagcagacc**

**Citrus_medica** **agcctcttgttcatatctctcatcaaaaggtgctattcgataatgtcgatttagcagacc**

**Citrus_polytrifolia** **agcctcttgttcatatctctcatcaaaaggtgctattcgataatgtcgatttagcagacc**

**Citrus_reticulata** **agcctcttgttcatatctctcatcaaaaggtgctattcgataatgtcgatttagcagacc**

**Citrus_sinensis** **agcctcttgttcatatctctcatcaaaaggtgctattcgataatgtcgatttagcagacc**

**Fortunella_bawangica_1** **agcctcttgttcatatctctcatcaaaaggtgctattcgataatgtcgatttagcagacc**

**Fortunella_bawangica_2** **agcctcttgttcatatctctcatcaaaaggtgctattcgataatgtcgatttagcagacc**

**Fortunella_hindsii_1** **agcctcttgttcatatctctcatcaaaaggtgctattcgataatgtcgatttagcagacc**

**Fortunella_hindsii_2** **agcctcttgttcatatctctcatcaaaaggtgctattcgataatgtcgatttagcagacc**

**Fortunella_hindsii_3** **agcctcttgttcatatctctcatcaaaaggtgctattcgataatgtcgatttagcagacc**

**Fortunella_japonica_1** **agcctcttgttcatatctctcatcaaaaggtgctattcgataatgtcgatttagcagacc**

**Fortunella_japonica_2** **agcctcttgttcatatctctcatcaaaaggtgctattcgataatgtcgatttagcagacc**

**Fortunella_japonica_3** **agcctcttgttcatatctctcatcaaaaggtgctattcgataatgtcgatttagcagacc**

**Fortunella_japonica_4** **agcctcttgttcatatctctcatcaaaaggtgctattcgataatgtcgatttagcagacc**

**Fortunella_japonica_5** **agcctcttgttcatatctctcatcaaaaggtgctattcgataatgtcgatttagcagacc**

**Fortunella_japonica_6** **agcctcttgttcatatctctcatcaaaaggtgctattcgataatgtcgatttagcagacc**

**Fortunella_japonica_7** **agcctcttgttcatatctctcatcaaaaggtgctattcgataatgtcgatttagcagacc**

**Fortunella_japonica_8** **agcctcttgttcatatctctcatcaaaaggtgctattcgataatgtcgatttagcagacc**

**Fortunella_japonica_9** **agcctcttgttcatatctctcatcaaaaggtgctattcgataatgtcgatttagcagacc**

**Fortunella_japonica_10** **agcctcttgttcatatctctcatcaaaaggtgctattcgataatgtcgatttagcagacc**

**Fortunella_japonica_11** **agcctcttgttcatatctctcatcaaaaggtgctattcgataatgtcgatttagcagacc**

**Fortunella_japonica_12** **agcctcttgttcatatctctcatcaaaaggtgctattcgataatgtcgatttagcagacc**

**Fortunella_margarita_1** **agcctcttgttcatatctctcatcaaaaggtgctattcgataatgtcgatttagcagacc**

**Fortunella_margarita_2** **agcctcttgttcatatctctcatcaaaaggtgctattcgataatgtcgatttagcagacc**

**Fortunella_venosa_1** **agcctcttgttcatatctctcatcaaaaggtgctattcgataatgtcgatttagcagacc**

**Fortunella_venosa_2** **agcctcttgttcatatctctcatcaaaaggtgctattcgataatgtcgatttagcagacc**

**Fortunella_venosa_3** **agcctcttgttcatatctctcatcaaaaggtgctattcgataatgtcgatttagaagacc**

26530	26540	26550	26560	26570	26580

....|....|....|....|....|....|....|....|....|....|....|....|

**Atalantia_kwangtungens** **ccccgctaacccgagcgagcattcaaatatctgtcctacattcattcgcgagggtactcc**

**Citrus_reticulata_1** **ccccgctaacccgagcgagcattcaaatatctgtcctacattcattcgcgagggtactcc**

**Citrus_reticulata_2** **ccccgctaacccgagcgagcattcaaatatctgtcctacattcattcgcgagggtactcc**

**Citrus_trifoliata_3** **ccccgctaacccgagcgagcattcaaatatctgtcctacattcattcgcgagggtactcc**

**Citrus_aurantiifolia** **ccccgctaacccgagcgagcattcaaatatctgtcctacattcattcgcgagggtactcc**

**Citrus_aurantium** **ccccgctaacccgagcgagcattcaaatatctgtcctacattcattcgcgagggtactcc**

**Citrus_cavaleriei** **ccccgctaacccgagcgagcattcaaatatctgtcctacattcattcgcgagggtactcc**

**Citrus_hongheensis** **ccccgctaacccgagcgagcattcaaatatctgtcctacattcattcgcgagggtactcc**

**Citrus_limon** **ccccgctaacccgagcgagcattcaaatatctgtcctacattcattcgcgagggtactcc**

**Citrus_maxima** **ccccgctaacccgagcgagcattcaaatatctgtcctacattcattcgcgagggtactcc**

**Citrus_medica** **ccccgctaacccgagcgagcattcaaatatctgtcctacattcattcgcgagggtactcc**

**Citrus_polytrifolia** **ccccgctaacccgagcgagcattcaaatatctgtcctacattcattcgcgagggtactcc**

**Citrus_reticulata** **ccccgctaacccgagcgagcattcaaatatctgtcctacattcattcgcgagggtactcc**

**Citrus_sinensis** **ccccgctaacccgagcgagcattcaaatatctgtcctacattcattcgcgagggtactcc**

**Fortunella_bawangica_1** **ccccgctaacccgagcgagcattcaaatatctgtcctacattcattcgcgagggtactcc**

**Fortunella_bawangica_2** **ccccgctaacccgagcgagcattcaaatatctgtcctacattcattcgcgagggtactcc**

**Fortunella_hindsii_1** **ccccgctaacccgagcgagcattcaaatatctgtcctacattcattcgcgagggtactcc**

**Fortunella_hindsii_2** **ccccgctaacccgagcgagcattcaaatatctgtcctacattcattcgcgagggtactcc**

**Fortunella_hindsii_3** **ccccgctaacccgagcgagcattcaaatatctgtcctacattcattcgcgagggtactcc**

**Fortunella_japonica_1** **ccccgctaacccgagcgagcattcaaatatctgtcctacattcattcgcgagggtactcc**

**Fortunella_japonica_2** **ccccgctaacccgagcgagcattcaaatatctgtcctacattcattcgcgagggtactcc**

**Fortunella_japonica_3** **ccccgctaacccgagcgagcattcaaatatctgtcctacattcattcgcgagggtactcc**

**Fortunella_japonica_4** **ccccgctaacccgagcgagcattcaaatatctgtcctacattcattcgcgagggtactcc**

**Fortunella_japonica_5** **ccccgctaacccgagcgagcattcaaatatctgtcctacattcattcgcgagggtactcc**

**Fortunella_japonica_6** **ccccgctaacccgagcgagcattcaaatatctgtcctacattcattcgcgagggtactcc**

**Fortunella_japonica_7** **ccccgctaacccgagcgagcattcaaatatctgtcctacattcattcgcgagggtactcc**

**Fortunella_japonica_8** **ccccgctaacccgagcgagcattcaaatatctgtcctacattcattcgcgagggtactcc**

**Fortunella_japonica_9** **ccccgctaacccgagcgagcattcaaatatctgtcctacattcattcgcgagggtactcc**

**Fortunella_japonica_10** **ccccgctaacccgagcgagcattcaaatatctgtcctacattcattcgcgagggtactcc**

**Fortunella_japonica_11** **ccccgctaacccgagcgagcattcaaatatctgtcctacattcattcgcgagggtactcc**

**Fortunella_japonica_12** **ccccgctaacccgagcgagcattcaaatatctgtcctacattcattcgcgagggtactcc**

**Fortunella_margarita_1** **ccccgctaacccgagcgagcattcaaatatctgtcctacattcattcgcgagggtactcc**

**Fortunella_margarita_2** **ccccgctaacccgagcgagcattcaaatatctgtcctacattcattcgcgagggtactcc**

**Fortunella_venosa_1** **ccccgctaacccgagcgagcattcaaatatctgtcctacattcattcgcgagggtactcc**

**Fortunella_venosa_2** **ccccgctaacccgagcgagcattcaaatatctgtcctacattcattcgcgagggtactcc**

**Fortunella_venosa_3** **ccccgctaacccgagcgagcattcaaatatctgtcctacattcattcgcgagggtactcc**

26590	26600	26610	26620	26630	26640

....|....|....|....|....|....|....|....|....|....|....|....|

**Atalantia_kwangtungens** **tagtgggttgaaaaccatatcaacaggccttccatcttgcaaataaggcatatcctgtct**

**Citrus_reticulata_1** **tagtgggttgaaaaccatatcaacaggccttccatcttgcaaataaggcatatcctgtct**

**Citrus_reticulata_2** **tagtgggttgaaaaccatatcaacaggccttccatcttgcaaataaggcatatcctgtct**

**Citrus_trifoliata_3** **tagtgggttgaaaaccatatcaacaggccttccatcttgcaaataaggcatatcctgtct**

**Citrus_aurantiifolia** **tagtgggttgaaaaccatatcaacaggccttccatcttgcaaataaggcatatcctgtct**

**Citrus_aurantium** **tagtgggttgaaaaccatatcaacaggccttccatcttgcaaataaggcatatcctgtct**

**Citrus_cavaleriei** **tagtgggttgaaaaccatatcaacaggccttccatcttgcaaataaggcatatcctgtct**

**Citrus_hongheensis** **tagtgggttgaaaaccatatcaacaggccttccatcttgcaaataaggcatatcctgtct**

**Citrus_limon** **tagtgggttgaaaaccatatcaacaggccttccatcttgcaaataaggcatatcctgtct**

**Citrus_maxima** **tagtgggttgaaaaccatatcaacaggccttccatcttgcaaataaggcatatcctgtct**

**Citrus_medica** **tagtgggttgaaaaccatatcaacaggccttccatcttgcaaataaggcatatcctgtct**

**Citrus_polytrifolia** **tagtgggttgaaaaccatatcaacaggccttccatcttgcaaataaggcatatcctgtct**

**Citrus_reticulata** **tagtgggttgaaaaccatatcaacaggccttccatcttgcaaataaggcatatcctgtct**

**Citrus_sinensis** **tagtgggttgaaaaccatatcaacaggccttccatcttgcaaataaggcatatcctgtct**

**Fortunella_bawangica_1** **tagtgggttgaaaaccatatcaacaggccttccatcttgcaaataaggcatatcctgtct**

**Fortunella_bawangica_2** **tagtgggttgaaaaccatatcaacaggccttccatcttgcaaataaggcatatcctgtct**

**Fortunella_hindsii_1** **tagtgggttgaaaaccatatcaacaggccttccatcttgcaaataaggcatatcctgtct**

**Fortunella_hindsii_2** **tagtgggttgaaaaccatatcaacaggccttccatcttgcaaataaggcatatcctgtct**

**Fortunella_hindsii_3** **tagtgggttgaaaaccatatcaacaggccttccatcttgcaaataaggcatatcctgtct**

**Fortunella_japonica_1** **tagtgggttgaaaaccatatcaacaggccttccatcttgcaaataaggcatatcctgtct**

**Fortunella_japonica_2** **tagtgggttgaaaaccatatcaacaggccttccatcttgcaaataaggcatatcctgtct**

**Fortunella_japonica_3** **tagtgggttgaaaaccatatcaacaggccttccatcttgcaaataaggcatatcctgtct**

**Fortunella_japonica_4** **tagtgggttgaaaaccatatcaacaggccttccatcttgcaaataaggcatatcctgtct**

**Fortunella_japonica_5** **tagtgggttgaaaaccatatcaacaggccttccatcttgcaaataaggcatatcctgtct**

**Fortunella_japonica_6** **tagtgggttgaaaaccatatcaacaggccttccatcttgcaaataaggcatatcctgtct**

**Fortunella_japonica_7** **tagtgggttgaaaaccatatcaacaggccttccatcttgcaaataaggcatatcctgtct**

**Fortunella_japonica_8** **tagtgggttgaaaaccatatcaacaggccttccatcttgcaaataaggcatatcctgtct**

**Fortunella_japonica_9** **tagtgggttgaaaaccatatcaacaggccttccatcttgcaaataaggcatatcctgtct**

**Fortunella_japonica_10** **tagtgggttgaaaaccatatcaacaggccttccatcttgcaaataaggcatatcctgtct**

**Fortunella_japonica_11** **tagtgggttgaaaaccatatcaacaggccttccatcttgcaaataaggcatatcctgtct**

**Fortunella_japonica_12** **tagtgggttgaaaaccatatcaacaggccttccatcttgcaaataaggcatatcctgtct**

**Fortunella_margarita_1** **tagtgggttgaaaaccatatcaacaggccttccatcttgcaaataaggcatatcctgtct**

**Fortunella_margarita_2** **tagtgggttgaaaaccatatcaacaggccttccatcttgcaaataaggcatatcctgtct**

**Fortunella_venosa_1** **tagtgggttgaaaaccatatcaacaggccttccatcttgcaaataaggcatatcctgtct**

**Fortunella_venosa_2** **tagtgggttgaaaaccatatcaacaggccttccatcttgcaaataaggcatatcctgtct**

**Fortunella_venosa_3** **tagtgggttgaaaaccatatcaacaggccttccatcttgcaaataaggcatatcctgtct**

26650	26660	26670	26680	26690	26700

....|....|....|....|....|....|....|....|....|....|....|....|

**Atalantia_kwangtungens** **aggcaaaatttttgaaatgatacccttatttccatgtcttccagctactttatcacctac**

**Citrus_reticulata_1** **aggcaaaatttttgaaacgatacccttatttccatgtcttccagctactttatcacctac**

**Citrus_reticulata_2** **aggcaaaatttttgaaacgatacccttatttccatgtcttccagctactttatcacctac**

**Citrus_trifoliata_3** **aggcaaaatttttgaaacgatacccttatttccatgtcttccagctactttatcacctac**

**Citrus_aurantiifolia** **aggcaaaatttttgaaacgatacccttatttccatgtcttccagctactttatcacctac**

**Citrus_aurantium** **aggcaaaatttttgaaacgatacccttatttccatgtcttccagctactttatcacctac**

**Citrus_cavaleriei** **aggcaaaatttttgaaacgatacccttatttccatgtcttccagctactttatcacctac**

**Citrus_hongheensis** **aggcaaaatttttgaaacgatacccttatttccatgtcttccagctactttatcacctac**

**Citrus_limon** **aggcaaaatttttgaaacgatacccttatttccatgtcttccagctactttatcacctac**

**Citrus_maxima** **aggcaaaatttttgaaacgatacccttatttccatgtcttccagctactttatcacctac**

**Citrus_medica** **agccaaaatttttgaaacgatacccttatttccatgtcttccagctactttatcacctac**

**Citrus_polytrifolia** **aggcaaaatttttgaaacgatacccttatttccatgtcttccagctactttatcacctac**

**Citrus_reticulata** **aggcaaaatttttgaaacgatacccttatttccatgtcttccagctactttatcacctac**

**Citrus_sinensis** **aggcaaaatttttgaaacgatacccttatttccatgtcttccagctactttatcacctac**

**Fortunella_bawangica_1** **aggcaaaatttttgaaacgatacccttatttccatgtcttccagctactttatcacctac**

**Fortunella_bawangica_2** **aggcaaaatttttgaaacgatacccttatttccatgtcttccagctactttatcacctac**

**Fortunella_hindsii_1** **aggcaaaatttttgaaacgatacccttatttccatgtcttccagctactttatcacctac**

**Fortunella_hindsii_2** **aggcaaaatttttgaaacgatacccttatttccatgtcttccagctactttatcacctac**

**Fortunella_hindsii_3** **aggcaaaatttttgaaacgatacccttatttccatgtcttccagctactttatcacctac**

**Fortunella_japonica_1** **aggcaaaatttttgaaacgatacccttatttccatgtcttccagctactttatcacctac**

**Fortunella_japonica_2** **aggcaaaatttttgaaacgatacccttatttccatgtcttccagctactttatcacctac**

**Fortunella_japonica_3** **aggcaaaatttttgaaacgatacccttatttccatgtcttccagctactttatcacctac**

**Fortunella_japonica_4** **aggcaaaatttttgaaacgatacccttatttccatgtcttccagctactttatcacctac**

**Fortunella_japonica_5** **aggcaaaatttttgaaacgatacccttatttccatgtcttccagctactttatcacctac**

**Fortunella_japonica_6** **aggcaaaatttttgaaacgatacccttatttccatgtcttccagctactttatcacctac**

**Fortunella_japonica_7** **aggcaaaatttttgaaacgatacccttatttccatgtcttccagctactttatcacctac**

**Fortunella_japonica_8** **aggcaaaatttttgaaacgatacccttatttccatgtcttccagctactttatcacctac**

**Fortunella_japonica_9** **aggcaaaatttttgaaacgatacccttatttccatgtcttccagctactttatcacctac**

**Fortunella_japonica_10** **aggcaaaatttttgaaacgatacccttatttccatgtcttccagctactttatcacctac**

**Fortunella_japonica_11** **aggcaaaatttttgaaacgatacccttatttccatgtcttccagctactttatcacctac**

**Fortunella_japonica_12** **aggcaaaatttttgaaacgatacccttatttccatgtcttccagctactttatcacctac**

**Fortunella_margarita_1** **aggcaaaatttttgaaacgatacccttatttccatgtcttccagctactttatcacctac**

**Fortunella_margarita_2** **aggcaaaatttttgaaacgatacccttatttccatgtcttccagctactttatcacctac**

**Fortunella_venosa_1** **aggcaaaatttttgaaacgatacccttatttccatgtcttccagctactttatcacctac**

**Fortunella_venosa_2** **aggcaaaatttttgaaacgatacccttatttccatgtcttccagctactttatcacctac**

**Fortunella_venosa_3** **aggcaaaatttttgaaacgatacccttatttccatgtcttccagctactttatcacctac**

26710	26720	26730	26740	26750	26760

....|....|....|....|....|....|....|....|....|....|....|....|

**Atalantia_kwangtungens** **tttgatttcacgtttctgtgaaatatatacacaaattgtttctggattataactagaacc**

**Citrus_reticulata_1** **tttgatttcacgtttctgtgaaatatatacacaaatcgtttctggattataactagaacc**

**Citrus_reticulata_2** **tttgatttcacgtttctgtgaaatatatacacaaatcgtttctggattataactagaacc**

**Citrus_trifoliata_3** **tttgatttcacgtttctgtgaaatatatacacaaattgtttctggattataactagaacc**

**Citrus_aurantiifolia** **tttgatttcacgtttctgtgaaatatatacacaaatcctttctggattataactagaacc**

**Citrus_aurantium** **tttgatttcacgtttctgtgaaatatatacacaaatcgtttctggattataactagaacc**

**Citrus_cavaleriei** **tttgatttcacgtttctgtgaaatatatacacaaatcgtttctggattataactagaacc**

**Citrus_hongheensis** **tttgatttcacgtttctgtgaaatatatacacaaatcgtttctggattataactagaacc**

**Citrus_limon** **tttgatttcacgtttctgtgaaatatatacacaaatcgtttctggattataactagaacc**

**Citrus_maxima** **tttgatttcacgtttctgtgaaatatatacacaaatcgtttctggattataactagaacc**

**Citrus_medica** **tttgatttcacgtttctgtgaaatatatacacaaatcgtttctggattataactagaacc**

**Citrus_polytrifolia** **tttgatttcacgtttctgtgaaatatatacacaaatcgtttctggattataactagaacc**

**Citrus_reticulata** **tttgatttcacgtttctgtgaaatatatacacaaatcgtttctggattataactagaacc**

**Citrus_sinensis** **tttgatttcacgtttctgtgaaatatatacacaaatcgtttctggattataactagaacc**

**Fortunella_bawangica_1** **tttgatttcacgtttctgtgaaatatatacacaaatcgtttctggattataactagaacc**

**Fortunella_bawangica_2** **tttgatttcacgtttctgtgaaatatatacacaaatcgtttctggattataactagaacc**

**Fortunella_hindsii_1** **tttgatttcacgtttctgtgaaatatatacacaaatcgtttctggattataactagaacc**

**Fortunella_hindsii_2** **tttgatttcacgtttctgtgaaatatatacacaaatcgtttctggattataactagaacc**

**Fortunella_hindsii_3** **tttgatttcacgtttctgtgaaatatatacacaaatcgtttctggattataactagaacc**

**Fortunella_japonica_1** **tttgatttcacgtttctgtgaaatatatacacaaatcgtttctggattataactagaacc**

**Fortunella_japonica_2** **tttgatttcacgtttctgtgaaatatatacacaaatcgtttctggattataactagaacc**

**Fortunella_japonica_3** **tttgatttcacgtttctgtgaaatatatacacaaatcgtttctggattataactagaacc**

**Fortunella_japonica_4** **tttgatttcacgtttctgtgaaatatatacacaaatcgtttctggattataactagaacc**

**Fortunella_japonica_5** **tttgatttcacgtttctgtgaaatatatacacaaatcgtttctggattataactagaacc**

**Fortunella_japonica_6** **tttgatttcacgtttctgtgaaatatatacacaaatcgtttctggattataactagaacc**

**Fortunella_japonica_7** **tttgatttcacgtttctgtgaaatatatacacaaatcgtttctggattataactagaacc**

**Fortunella_japonica_8** **tttgatttcacgtttctgtgaaatatatacacaaatcgtttctggattataactagaacc**

**Fortunella_japonica_9** **tttgatttcacgtttctgtgaaatatatacacaaatcgtttctggattataactagaacc**

**Fortunella_japonica_10** **tttgatttcacgtttctgtgaaatatatacacaaatcgtttctggattataactagaacc**

**Fortunella_japonica_11** **tttgatttcacgtttctgtgaaatatatacacaaatcgtttctggattataactagaacc**

**Fortunella_japonica_12** **tttgatttcacgtttctgtgaaatatatacacaaatcgtttctggattataactagaacc**

**Fortunella_margarita_1** **tttgatttcacgtttctgtgaaatatatacacaaatcgtttctggattataactagaacc**

**Fortunella_margarita_2** **tttgatttcacgtttctgtgaaatatatacacaaatcgtttctggattataactagaacc**

**Fortunella_venosa_1** **tttgatttcacgtttctgtgaaatatatacacaaatcgtttctggattataactagaacc**

**Fortunella_venosa_2** **tttgatttcacgtttctgtgaaatatatacacaaatcgtttctggattataactagaacc**

**Fortunella_venosa_3** **tttgatttcacgtttctgtgaaatatatacacaaatcgtttctggattataactagaacc**

26770	26780	26790	26800	26810	26820

....|....|....|....|....|....|....|....|....|....|....|....|

**Atalantia_kwangtungens** **cccctttttctggacccatctcacatcaataacgcgacccctaccacctataggtagttt**

**Citrus_reticulata_1** **cccctttttctggacccatctcacatcaataacgcgacccctaccacctataggtagttt**

**Citrus_reticulata_2** **cccctttttctggacccatctcacatcaataacgcgacccctaccacctataggtagttt**

**Citrus_trifoliata_3** **cccctttttctggacccatctcacatcaataacgcgacccctaccacctataggtagttt**

**Citrus_aurantiifolia** **cccctttttctggacccatctcacatcaataacgcgacccctaccacctataggtagttt**

**Citrus_aurantium** **cccctttttctggacccatctcacatcaataacgcgacccctaccacctataggtagttt**

**Citrus_cavaleriei** **cccctttttctggacccatctcacatcaataacgcgacccctaccacctataggtagttt**

**Citrus_hongheensis** **cccctttttctggacccatctcacatcaataacgcgacccctaccacctataggtagttt**

**Citrus_limon** **cccctttttctggacccatctcacatcaataacgcgacccctaccacctataggtagttt**

**Citrus_maxima** **cccctttttctggacccatctcacatcaataacgcgacccctaccacctataggtagttt**

**Citrus_medica** **cccctttttctggacccatctcacatcaataacgcgacccctaccacctataggtagttt**

**Citrus_polytrifolia** **cccctttttctggacccatctcacatcaataacgcgacccctaccacctataggtagttt**

**Citrus_reticulata** **cccctttttctggacccatctcacatcaataacgcgacccctaccacctataggtagttt**

**Citrus_sinensis** **cccctttttctggacccatctcacatcaataacgcgacccctaccacctataggtagttt**

**Fortunella_bawangica_1** **cccctttttctggacccatctcacatcaataacgcgacccctaccacctataggtagttt**

**Fortunella_bawangica_2** **cccctttttctggacccatctcacatcaataacgcgacccctaccacctataggtagttt**

**Fortunella_hindsii_1** **cccctttttctggacccatctcacatcaataacgcgacccctaccacctataggtagttt**

**Fortunella_hindsii_2** **cccctttttctggacccatctcacatcaataacgcgacccctaccacctataggtagttt**

**Fortunella_hindsii_3** **cccctttttctggacccatctcacatcaataacgcgacccctaccacctataggtagttt**

**Fortunella_japonica_1** **cccctttttctggacccatctcacatcaataacgcgacccctaccacctataggtagttt**

**Fortunella_japonica_2** **cccctttttctggacccatctcacatcaataacgcgacccctaccacctataggtagttt**

**Fortunella_japonica_3** **cccctttttctggacccatctcacatcaataacgcgacccctaccacctataggtagttt**

**Fortunella_japonica_4** **cccctttttctggacccatctcacatcaataacgcgacccctaccacctataggtagttt**

**Fortunella_japonica_5** **cccctttttctggacccatctcacatcaataacgcgacccctaccacctataggtagttt**

**Fortunella_japonica_6** **cccctttttctggacccatctcacatcaataacgcgacccctaccacctataggtagttt**

**Fortunella_japonica_7** **cccctttttctggacccatctcacatcaataacgcgacccctaccacctataggtagttt**

**Fortunella_japonica_8** **cccctttttctggacccatctcacatcaataacgcgacccctaccacctataggtagttt**

**Fortunella_japonica_9** **cccctttttctggacccatctcacatcaataacgcgacccctaccacctataggtagttt**

**Fortunella_japonica_10** **cccctttttctggacccatctcacatcaataacgcgacccctaccacctataggtagttt**

**Fortunella_japonica_11** **cccctttttctggacccatctcacatcaataacgcgacccctaccacctataggtagttt**

**Fortunella_japonica_12** **cccctttttctggacccatctcacatcaataacgcgacccctaccacctataggtagttt**

**Fortunella_margarita_1** **cccctttttctggacccatctcacatcaataacgcgacccctaccacctataggtagttt**

**Fortunella_margarita_2** **cccctttttctggacccatctcacatcaataacgcgacccctaccacctataggtagttt**

**Fortunella_venosa_1** **cccctttttctggacccatctcacatcaataacgcgacccctaccacctataggtagttt**

**Fortunella_venosa_2** **cccctttttctggacccatctcacatcaataacgcgacccctaccacctataggtagttt**

**Fortunella_venosa_3** **cccctttttctggacccatctcacatcaataacgcgacccctaccacctataggtagttt**

26830	26840	26850	26860	26870	26880

....|....|....|....|....|....|....|....|....|....|....|....|

**Atalantia_kwangtungens** **taggcaagtttcctttgaagtggatacctgaatgccaagtatggcccgtaataatctatc**

**Citrus_reticulata_1** **taggcaagtttcctttgaagtggatacctgaatgccaagtatggcccgtaataatctatc**

**Citrus_reticulata_2** **taggcaagtttcctttgaagtggatacctgaatgccaagtatggcccgtaataatctatc**

**Citrus_trifoliata_3** **taggcaagtttcctttgaagtggatacctgaatgccaagtatggcccgtaataatctatc**

**Citrus_aurantiifolia** **taggcaagtttcctttgaagtggatacctgaatgccaagtatggcccgtaataatctatc**

**Citrus_aurantium** **taggcaagtttcctttgaagtggatacctgaatgccaagtatggcccgtaataatctatc**

**Citrus_cavaleriei** **taggcaagtttcctttgaagtggatacctgaatgccaagtatggcccgtaataatctatc**

**Citrus_hongheensis** **taggcaagtttcctttgaagtggatacctgaatgccaagtatggcccgtaataatctatc**

**Citrus_limon** **taggcaagtttcctttgaagtggatacctgaatgccaagtatggcccgtaataatctatc**

**Citrus_maxima** **taggcaagtttcctttgaagtggatacctgaatgccaagtatggcccgtaataatctatc**

**Citrus_medica** **taggcaagtttcctttgaagtggatacctgaatgccaagtatggcccgtaataatctatc**

**Citrus_polytrifolia** **taggcaagtttcctttgaagtggatacctgaatgccaagtatggcccgtaataatctatc**

**Citrus_reticulata** **taggcaagtttcctttgaagtggatacctgaatgccaagtatggcccgtaataatctatc**

**Citrus_sinensis** **taggcaagtttcctttgaagtggatacctgaatgccaagtatggcccgtaataatctatc**

**Fortunella_bawangica_1** **taggcaagtttcctttgaagtggatacctgaatgccaagtatggcccgtaataatctatc**

**Fortunella_bawangica_2** **taggcaagtttcctttgaagtggatacctgaatgccaagtatggcccgtaataatctatc**

**Fortunella_hindsii_1** **taggcaagtttcctttgaagtggatacctgaatgccaagtatggcccgtaataatctatc**

**Fortunella_hindsii_2** **taggcaagtttcctttgaagtggatacctgaatgccaagtatggcccgtaataatctatc**

**Fortunella_hindsii_3** **taggcaagtttcctttgaagtggatacctgaatgccaagtatggcccgtaataatctatc**

**Fortunella_japonica_1** **taggcaagtttcctttgaagtggatacctgaatgccaagtatggcccgtaataatctatc**

**Fortunella_japonica_2** **taggcaagtttcctttgaagtggatacctgaatgccaagtatggcccgtaataatctatc**

**Fortunella_japonica_3** **taggcaagtttcctttgaagtggatacctgaatgccaagtatggcccgtaataatctatc**

**Fortunella_japonica_4** **taggcaagtttcctttgaagtggatacctgaatgccaagtatggcccgtaataatctatc**

**Fortunella_japonica_5** **taggcaagtttcctttgaagtggatacctgaatgccaagtatggcccgtaataatctatc**

**Fortunella_japonica_6** **taggcaagtttcctttgaagtggatacctgaatgccaagtatggcccgtaataatctatc**

**Fortunella_japonica_7** **taggcaagtttcctttgaagtggatacctgaatgccaagtatggcccgtaataatctatc**

**Fortunella_japonica_8** **taggcaagtttcctttgaagtggatacctgaatgccaagtatggcccgtaataatctatc**

**Fortunella_japonica_9** **taggcaagtttcctttgaagtggatacctgaatgccaagtatggcccgtaataatctatc**

**Fortunella_japonica_10** **taggcaagtttcctttgaagtggatacctgaatgccaagtatggcccgtaataatctatc**

**Fortunella_japonica_11** **taggcaagtttcctttgaagtggatacctgaatgccaagtatggcccgtaataatctatc**

**Fortunella_japonica_12** **taggcaagtttcctttgaagtggatacctgaatgccaagtatggcccgtaataatctatc**

**Fortunella_margarita_1** **taggcaagtttcctttgaagtggatacctgaatgccaagtatggcccgtaataatctatc**

**Fortunella_margarita_2** **taggcaagtttcctttgaagtggatacctgaatgccaagtatggcccgtaataatctatc**

**Fortunella_venosa_1** **taggcaagtttcctttgaagtggatacctgaatgccaagtatggcccgtaataatctatc**

**Fortunella_venosa_2** **taggcaagtttcctttgaagtggatacctgaatgccaagtatggcccgtaataatctatc**

**Fortunella_venosa_3** **taggcaagtttcctttgaagtggatacctgaatgccaagtatggcccgtaataatctatc**

26890	26900	26910	26920	26930	26940

....|....|....|....|....|....|....|....|....|....|....|....|

**Atalantia_kwangtungens** **ttccggagcatacgatgattctttcgccgcctgaggcgttaatttacctactaaaatatc**

**Citrus_reticulata_1** **ttccggagcatacgatgattctttcgccgcctgaggcgttaatttacctactaaaatatc**

**Citrus_reticulata_2** **ttccggagcatacgatgattctttcgccgcctgaggcgttaatttacctactaaaatatc**

**Citrus_trifoliata_3** **ttccggagcatacgatgattctttcgccgcctgaggcgttaatttacctactaaaatatc**

**Citrus_aurantiifolia** **ttccggagcatacgatgattctttcgccgcctgaggcgttaatttacctactaaaatatc**

**Citrus_aurantium** **ttccggagcatacgatgattctttcgccgcctgaggcgttaatttacctactaaaatatc**

**Citrus_cavaleriei** **ttccggagcatacgatgattctttcgccgcctgaggcgttaatttacctactaaaatatc**

**Citrus_hongheensis** **ttccggagcatacgatgattctttcgccgcctgaggcgttaatttacctactaaaatatc**

**Citrus_limon** **ttccggagcatacgatgattctttcgccgcctgaggcgttaatttacctactaaaatatc**

**Citrus_maxima** **ttccggagcatacgatgattctttcgccgcctgaggcgttaatttacctactaaaatatc**

**Citrus_medica** **ttccggagcatacgatgattctttcgccgcctgaggcgttaatttacctactaaaatatc**

**Citrus_polytrifolia** **ttccggagcatacgatgattctttcgccgcctgaggcgttaatttacctactaaaatatc**

**Citrus_reticulata** **ttccggagcatacgatgattctttcgccgcctgaggcgttaatttacctactaaaatatc**

**Citrus_sinensis** **ttccggagcatacgatgattctttcgccgcctgaggcgttaatttacctactaaaatatc**

**Fortunella_bawangica_1** **ttccggagcatacgatgattctttcgccgcctgaggcgttaatttacctactaaaatatc**

**Fortunella_bawangica_2** **ttccggagcatacgatgattctttcgccgcctgaggcgttaatttacctactaaaatatc**

**Fortunella_hindsii_1** **ttccggagcatacgatgattctttcgccgcctgaggcgttaatttacctactaaaatatc**

**Fortunella_hindsii_2** **ttccggagcatacgatgattctttcgccgcctgaggcgttaatttacctactaaaatatc**

**Fortunella_hindsii_3** **ttccggagcatacgatgattctttcgccgcctgaggcgttaatttacctactaaaatatc**

**Fortunella_japonica_1** **ttccggagcatacgatgattctttcgccgcctgaggcgttaatttacctactaaaatatc**

**Fortunella_japonica_2** **ttccggagcatacgatgattctttcgccgcctgaggcgttaatttacctactaaaatatc**

**Fortunella_japonica_3** **ttccggagcatacgatgattctttcgccgcctgaggcgttaatttacctactaaaatatc**

**Fortunella_japonica_4** **ttccggagcatacgatgattctttcgccgcctgaggcgttaatttacctactaaaatatc**

**Fortunella_japonica_5** **ttccggagcatacgatgattctttcgccgcctgaggcgttaatttacctactaaaatatc**

**Fortunella_japonica_6** **ttccggagcatacgatgattctttcgccgcctgaggcgttaatttacctactaaaatatc**

**Fortunella_japonica_7** **ttccggagcatacgatgattctttcgccgcctgaggcgttaatttacctactaaaatatc**

**Fortunella_japonica_8** **ttccggagcatacgatgattctttcgccgcctgaggcgttaatttacctactaaaatatc**

**Fortunella_japonica_9** **ttccggagcatacgatgattctttcgccgcctgaggcgttaatttacctactaaaatatc**

**Fortunella_japonica_10** **ttccggagcatacgatgattctttcgccgcctgaggcgttaatttacctactaaaatatc**

**Fortunella_japonica_11** **ttccggagcatacgatgattctttcgccgcctgaggcgttaatttacctactaaaatatc**

**Fortunella_japonica_12** **ttccggagcatacgatgattctttcgccgcctgaggcgttaatttacctactaaaatatc**

**Fortunella_margarita_1** **ttccggagcatacgatgattctttcgccgcctgaggcgttaatttacctactaaaatatc**

**Fortunella_margarita_2** **ttccggagcatacgatgattctttcgccgcctgaggcgttaatttacctactaaaatatc**

**Fortunella_venosa_1** **ttccggagcatacgatgattctttcgccgcctgaggcgttaatttacctactaaaatatc**

**Fortunella_venosa_2** **ttccggagcatacgatgattctttcgccgcctgaggcgttaatttacctactaaaatatc**

**Fortunella_venosa_3** **ttccggagcatacgatgattctttcgccgcctgaggcgttaatttacctactaaaatatc**

26950	26960	26970	26980	26990	27000

....|....|....|....|....|....|....|....|....|....|....|....|

**Atalantia_kwangtungens** **gccggcctctacccaagatcccagcatcacaattccatttttgtctaaattgcggagtaa**

**Citrus_reticulata_1** **gccggtctctacccaagatcccagcatcacaattccatttttgtctaaattgcggagtaa**

**Citrus_reticulata_2** **gccggtctctacccaagatcccagcatcacaattccatttttgtctaaattgcggagtaa**

**Citrus_trifoliata_3** **gccggtctctacccaagatcccagcatcacaattccatttttgtctaaattgcggagtaa**

**Citrus_aurantiifolia** **gccggtctctacccaagatcccagcatcacaattccatttttgtctaaattgcggagtaa**

**Citrus_aurantium** **gccggtctctacccaagatcccagcatcacaattccatttttgtctaaattgcggagtaa**

**Citrus_cavaleriei** **gccggtctctacccaagatcccagcatcacaattccatttttgtctaaattgcggagtaa**

**Citrus_hongheensis** **gccggtctctacccaagatcccagcatcacaattccatttttgtctaaattgcggagtaa**

**Citrus_limon** **gccggtctctacccaagatcccagcatcacaattccatttttgtctaaattgcggagtaa**

**Citrus_maxima** **gccggtctctacccaagatcccagcatcacaattccatttttgtctaaattgcggagtaa**

**Citrus_medica** **gccggtctctacccaagatcccagcatcacaattccatttttgtctaaattgcggagtaa**

**Citrus_polytrifolia** **gccggtctctacccaagatcccagcatcacaattccatttttgtctaaattgcggagtaa**

**Citrus_reticulata** **gccggtctctacccaagatcccagcatcacaattccatttttgtctaaattgcggagtaa**

**Citrus_sinensis** **gccggtctctacccaagatcccagcatcacaattccatttttgtctaaattgcggagtaa**

**Fortunella_bawangica_1** **gccggtctctacccaagatcccagcatcacaattccatttttgtctaaattgcggagtaa**

**Fortunella_bawangica_2** **gccggtctctacccaagatcccagcatcacaattccatttttgtctaaattgcggagtaa**

**Fortunella_hindsii_1** **gccggtctctacccaagatcccagcatcacaattccatttttgtctaaattgcggagtaa**

**Fortunella_hindsii_2** **gccggtctctacccaagatcccagcatcacaattccatttttgtctaaattgcggagtaa**

**Fortunella_hindsii_3** **gccggtctctacccaagatcccagcatcacaattccatttttgtctaaattgcggagtaa**

**Fortunella_japonica_1** **gccggtctctacccaagatcccagcatcacaattccatttttgtctaaattgcggagtaa**

**Fortunella_japonica_2** **gccggtctctacccaagatcccagcatcacaattccatttttgtctaaattgcggagtaa**

**Fortunella_japonica_3** **gccggtctctacccaagatcccagcatcacaattccatttttgtctaaattgcggagtaa**

**Fortunella_japonica_4** **gccggtctctacccaagatcccagcatcacaattccatttttgtctaaattgcggagtaa**

**Fortunella_japonica_5** **gccggtctctacccaagatcccagcatcacaattccatttttgtctaaattgcggagtaa**

**Fortunella_japonica_6** **gccggtctctacccaagatcccagcatcacaattccatttttgtctaaattgcggagtaa**

**Fortunella_japonica_7** **gccggtctctacccaagatcccagcatcacaattccatttttgtctaaattgcggagtaa**

**Fortunella_japonica_8** **gccggtctctacccaagatcccagcatcacaattccatttttgtctaaattgcggagtaa**

**Fortunella_japonica_9** **gccggtctctacccaagatcccagcatcacaattccatttttgtctaaattgcggagtaa**

**Fortunella_japonica_10** **gccggtctctacccaagatcccagcatcacaattccatttttgtctaaattgcggagtaa**

**Fortunella_japonica_11** **gccggtctctacccaagatcccagcatcacaattccatttttgtctaaattgcggagtaa**

**Fortunella_japonica_12** **gccggtctctacccaagatcccagcatcacaattccatttttgtctaaattgcggagtaa**

**Fortunella_margarita_1** **gccggtctctacccaagatcccagcatcacaattccatttttgtctaaattgcggagtaa**

**Fortunella_margarita_2** **gccggtctctacccaagatcccagcatcacaattccatttttgtctaaattgcggagtaa**

**Fortunella_venosa_1** **gccggtctctacccaagatcccagcatcacaattccatttttgtctaaattgcggagtaa**

**Fortunella_venosa_2** **gccggtctctacccaagatcccagcatcacaattccatttttgtctaaattgcggagtaa**

**Fortunella_venosa_3** **gccggtctctacccaagatcccagcatcacaattccatttttgtctaaattgcggagtaa**

27010	27020	27030	27040	27050	27060

....|....|....|....|....|....|....|....|....|....|....|....|

**Atalantia_kwangtungens** **ccgggcttctagatgtggtatttcattagtgatcctttcgggaccttgacttgtcacatg**

**Citrus_reticulata_1** **ccgggcttctagatgtggtatttcattagtgatcctttcgggaccttgacttgtcacatg**

**Citrus_reticulata_2** **ccgggcttctagatgtggtatttcattagtgatcctttcgggaccttgacttgtcacatg**

**Citrus_trifoliata_3** **ccgggcttctagatgtggtatttcattagtgatcctttcgggaccttgacttgtcacatg**

**Citrus_aurantiifolia** **ccgggcttctagatgtggtatttcattagtgatcctttcgggaccttgacttgtcacatg**

**Citrus_aurantium** **ccgggcttctagatgtggtatttcattagtgatcctttcgggaccttgacttgtcacatg**

**Citrus_cavaleriei** **ccgggcttctagatgtggtatttcattagtgatcctttcgggaccttgacttgtcacatg**

**Citrus_hongheensis** **ccgggcttctagatgtggtatttcattagtgatcctttcgggaccttgacttgtcacatg**

**Citrus_limon** **ccgggcttctagatgtggtatttcattagtgatcctttcgggaccttgacttgtcacatg**

**Citrus_maxima** **ccgggcttctagatgtggtatttcattagtgatcctttcgggaccttgacttgtcacatg**

**Citrus_medica** **ccgggcttctagatgtggtatttcattagtgatcctttcgggaccttgacttgtcacatg**

**Citrus_polytrifolia** **ccgggcttctagatgtggtatttcattagtgatcctttcgggaccttgacttgtcacatg**

**Citrus_reticulata** **ccgggcttctagatgtggtatttcattagtgatcctttcgggaccttgacttgtcacatg**

**Citrus_sinensis** **ccgggcttctagatgtggtatttcattagtgatcctttcgggaccttgacttgtcacatg**

**Fortunella_bawangica_1** **ccgggcttctagatgtggtatttcattagtgatcctttcgggaccttgacttgtcacatg**

**Fortunella_bawangica_2** **ccgggcttctagatgtggtatttcattagtgatcctttcgggaccttgacttgtcacatg**

**Fortunella_hindsii_1** **ccgggcttctagatgtggtatttcattagtgatcctttcgggaccttgacttgtcacatg**

**Fortunella_hindsii_2** **ccgggcttctagatgtggtatttcattagtgatcctttcgggaccttgacttgtcacatg**

**Fortunella_hindsii_3** **ccgggcttctagatgtggtatttcattagtgatcctttcgggaccttgacttgtcacatg**

**Fortunella_japonica_1** **ccgggcttctagatgtggtatttcattagtgatcctttcgggaccttgacttgtcacatg**

**Fortunella_japonica_2** **ccgggcttctagatgtggtatttcattagtgatcctttcgggaccttgacttgtcacatg**

**Fortunella_japonica_3** **ccgggcttctagatgtggtatttcattagtgatcctttcgggaccttgacttgtcacatg**

**Fortunella_japonica_4** **ccgggcttctagatgtggtatttcattagtgatcctttcgggaccttgacttgtcacatg**

**Fortunella_japonica_5** **ccgggcttctagatgtggtatttcattagtgatcctttcgggaccttgacttgtcacatg**

**Fortunella_japonica_6** **ccgggcttctagatgtggtatttcattagtgatcctttcgggaccttgacttgtcacatg**

**Fortunella_japonica_7** **ccgggcttctagatgtggtatttcattagtgatcctttcgggaccttgacttgtcacatg**

**Fortunella_japonica_8** **ccgggcttctagatgtggtatttcattagtgatcctttcgggaccttgacttgtcacatg**

**Fortunella_japonica_9** **ccgggcttctagatgtggtatttcattagtgatcctttcgggaccttgacttgtcacatg**

**Fortunella_japonica_10** **ccgggcttctagatgtggtatttcattagtgatcctttcgggaccttgacttgtcacatg**

**Fortunella_japonica_11** **ccgggcttctagatgtggtatttcattagtgatcctttcgggaccttgacttgtcacatg**

**Fortunella_japonica_12** **ccgggcttctagatgtggtatttcattagtgatcctttcgggaccttgacttgtcacatg**

**Fortunella_margarita_1** **ccgggcttctagatgtggtatttcattagtgatcctttcgggaccttgacttgtcacatg**

**Fortunella_margarita_2** **ccgggcttctagatgtggtatttcattagtgatcctttcgggaccttgacttgtcacatg**

**Fortunella_venosa_1** **ccgggcttctagatgtggtatttcattagtgatcctttcgggaccttgacttgtcacatg**

**Fortunella_venosa_2** **ccgggcttctagatgtggtatttcattagtgatcctttcgggaccttgacttgtcacatg**

**Fortunella_venosa_3** **ccgggcttctagatgtggtatttcattagtgatcctttcgggaccttgacttgtcacatg**

27070	27080	27090	27100	27110	27120

....|....|....|....|....|....|....|....|....|....|....|....|

**Atalantia_kwangtungens** **agtctgaatttcgtatttccgtatgtgaaaagaagtataaatatctctatatatcagacg**

**Citrus_reticulata_1** **agtctgaatttcgtatttccgtatgtgaaaagaagtataaatatctctatatatcagacg**

**Citrus_reticulata_2** **agtctgaatttcgtatttccgtatgtgaaaagaagtataaatatctctatatatcagacg**

**Citrus_trifoliata_3** **agtctgaatttcgtatttccgtatgtgaaaagaagtataaatatctctatatatcagacg**

**Citrus_aurantiifolia** **agtctgaatttcgtatttccgtatgtgaaaagaagtataaatatctctatatatcagacg**

**Citrus_aurantium** **agtctgaatttcgtatttccgtatgtgaaaagaagtataaatatctctatatatcagacg**

**Citrus_cavaleriei** **agtctgaatttcgtatttccgtatgtgaaaagaagtataaatatctctatatatcagacg**

**Citrus_hongheensis** **agtctgaatttcgtatttccgtatgtgaaaagaagtataaatatctctatatatcagacg**

**Citrus_limon** **agtctgaatttcgtatttccgtatgtgaaaagaagtataaatatctctatatatcagacg**

**Citrus_maxima** **agtctgaatttcgtatttccgtatgtgaaaagaagtataaatatctctatatatcagacg**

**Citrus_medica** **agtctgaatttcgtatttccgtatgtgaaaagaagtataaatatctctatatatcagacg**

**Citrus_polytrifolia** **agtctgaatttcgtatttccgtatgtgaaaagaagtataaatatctctatatatcagacg**

**Citrus_reticulata** **agtctgaatttcgtatttccgtatgtgaaaagaagtataaatatctctatatatcagacg**

**Citrus_sinensis** **agtctgaatttcgtatttccgtatgtgaaaagaagtataaatatctctatatatcagacg**

**Fortunella_bawangica_1** **agtctgaatttcgtatttccgtatgtgaaaagaagtataaatatctctatatatcagacg**

**Fortunella_bawangica_2** **agtctgaatttcgtatttccgtatgtgaaaagaagtataaatatctctatatatcagacg**

**Fortunella_hindsii_1** **agtctgaatttcgtatttccgtatgtgaaaagaagtataaatatctctatatatcagacg**

**Fortunella_hindsii_2** **agtctgaatttcgtatttccgtatgtgaaaagaagtataaatatctctatatatcagacg**

**Fortunella_hindsii_3** **agtctgaatttcgtatttccgtatgtgaaaagaagtataaatatctctatatatcagacg**

**Fortunella_japonica_1** **agtctgaatttcgtatttccgtatgtgaaaagaagtataaatatctctatatatcagacg**

**Fortunella_japonica_2** **agtctgaatttcgtatttccgtatgtgaaaagaagtataaatatctctatatatcagacg**

**Fortunella_japonica_3** **agtctgaatttcgtatttccgtatgtgaaaagaagtataaatatctctatatatcagacg**

**Fortunella_japonica_4** **agtctgaatttcgtatttccgtatgtgaaaagaagtataaatatctctatatatcagacg**

**Fortunella_japonica_5** **agtctgaatttcgtatttccgtatgtgaaaagaagtataaatatctctatatatcagacg**

**Fortunella_japonica_6** **agtctgaatttcgtatttccgtatgtgaaaagaagtataaatatctctatatatcagacg**

**Fortunella_japonica_7** **agtctgaatttcgtatttccgtatgtgaaaagaagtataaatatctctatatatcagacg**

**Fortunella_japonica_8** **agtctgaatttcgtatttccgtatgtgaaaagaagtataaatatctctatatatcagacg**

**Fortunella_japonica_9** **agtctgaatttcgtatttccgtatgtgaaaagaagtataaatatctctatatatcagacg**

**Fortunella_japonica_10** **agtctgaatttcgtatttccgtatgtgaaaagaagtataaatatctctatatatcagacg**

**Fortunella_japonica_11** **agtctgaatttcgtatttccgtatgtgaaaagaagtataaatatctctatatatcagacg**

**Fortunella_japonica_12** **agtctgaatttcgtatttccgtatgtgaaaagaagtataaatatctctatatatcagacg**

**Fortunella_margarita_1** **agtctgaatttcgtatttccgtatgtgaaaagaagtataaatatctctatatatcagacg**

**Fortunella_margarita_2** **agtctgaatttcgtatttccgtatgtgaaaagaagtataaatatctctatatatcagacg**

**Fortunella_venosa_1** **agtctgaatttcgtatttccgtatgtgaaaagaagtataaatatctctatatatcagacg**

**Fortunella_venosa_2** **agtctgaatttcgtatttccgtatgtgaaaagaagtataaatatctctatatatcagacg**

**Fortunella_venosa_3** **agtctgaatttcgtatttccgtatgtgaaaagaagtataaatatctctatatatcagacg**

27130	27140	27150	27160	27170	27180

....|....|....|....|....|....|....|....|....|....|....|....|

**Atalantia_kwangtungens** **ttcgctaataagtaccgcatcctcaaaattgtagccttcccatggcatataagctactaa**

**Citrus_reticulata_1** **ttcgctaataagtaccgcatcctcaaaattgtagccttcccatggcatataagctactaa**

**Citrus_reticulata_2** **ttcgctaataagtaccgcatcctcaaaattgtagccttcccatggcatataagctactaa**

**Citrus_trifoliata_3** **ttcgctaataagtaccgcatcctcaaaattgtagccttcccatggcatataagctactaa**

**Citrus_aurantiifolia** **ttcgctaataagtaccgcatcctcaaaattgtagccttcccatggcatataagctactaa**

**Citrus_aurantium** **ttcgctaataagtaccgcatcctcaaaattgtagccttcccatggcatataagctactaa**

**Citrus_cavaleriei** **ttcgctaataagtaccgcatcctcaaaattgtagccttcccatggcatataagctactaa**

**Citrus_hongheensis** **ttcgctaataagtaccgcatcctcaaaattgtagccttcccatggcatataagctactaa**

**Citrus_limon** **ttcgctaataagtaccgcatcctcaaaattgtagccttcccatggcatataagctactaa**

**Citrus_maxima** **ttcgctaataagtaccgcatcctcaaaattgtagccttcccatggcatataagctactaa**

**Citrus_medica** **ttcgctaataagtaccgcatcctcaaaattgtagccttcccatggcatataagctactaa**

**Citrus_polytrifolia** **ttcgctaataagtaccgcatcctcaaaattgtagccttcccatggcatataagctactaa**

**Citrus_reticulata** **ttcgctaataagtaccgcatcctcaaaattgtagccttcccatggcatataagctactaa**

**Citrus_sinensis** **ttcgctaataagtaccgcatcctcaaaattgtagccttcccatggcatataagctactaa**

**Fortunella_bawangica_1** **ttcgctaataagtaccgcatcctcaaaattgtagccttcccatggcatataagctactaa**

**Fortunella_bawangica_2** **ttcgctaataagtaccgcatcctcaaaattgtagccttcccatggcatataagctactaa**

**Fortunella_hindsii_1** **ttcgctaataagtaccgcatcctcaaaattgtagccttcccatggcatataagctactaa**

**Fortunella_hindsii_2** **ttcgctaataagtaccgcatcctcaaaattgtagccttcccatggcatataagctactaa**

**Fortunella_hindsii_3** **ttcgctaataagtaccgcatcctcaaaattgtagccttcccatggcatataagctactaa**

**Fortunella_japonica_1** **ttcgctaataagtaccgcatcctcaaaattgtagccttcccatggcatataagctactaa**

**Fortunella_japonica_2** **ttcgctaataagtaccgcatcctcaaaattgtagccttcccatggcatataagctactaa**

**Fortunella_japonica_3** **ttcgctaataagtaccgcatcctcaaaattgtagccttcccatggcatataagctactaa**

**Fortunella_japonica_4** **ttcgctaataagtaccgcatcctcaaaattgtagccttcccatggcatataagctactaa**

**Fortunella_japonica_5** **ttcgctaataagtaccgcatcctcaaaattgtagccttcccatggcatataagctactaa**

**Fortunella_japonica_6** **ttcgctaataagtaccgcatcctcaaaattgtagccttcccatggcatataagctactaa**

**Fortunella_japonica_7** **ttcgctaataagtaccgcatcctcaaaattgtagccttcccatggcatataagctactaa**

**Fortunella_japonica_8** **ttcgctaataagtaccgcatcctcaaaattgtagccttcccatggcatataagctactaa**

**Fortunella_japonica_9** **ttcgctaataagtaccgcatcctcaaaattgtagccttcccatggcatataagctactaa**

**Fortunella_japonica_10** **ttcgctaataagtaccgcatcctcaaaattgtagccttcccatggcatataagctactaa**

**Fortunella_japonica_11** **ttcgctaataagtaccgcatcctcaaaattgtagccttcccatggcatataagctactaa**

**Fortunella_japonica_12** **ttcgctaataagtaccgcatcctcaaaattgtagccttcccatggcatataagctactaa**

**Fortunella_margarita_1** **ttcgctaataagtaccgcatcctcaaaattgtagccttcccatggcatataagctactaa**

**Fortunella_margarita_2** **ttcgctaataagtaccgcatcctcaaaattgtagccttcccatggcatataagctactaa**

**Fortunella_venosa_1** **ttcgctaataagtaccgcatcctcaaaattgtagccttcccatggcatataagctactaa**

**Fortunella_venosa_2** **ttcgctaataagtaccgcatcctcaaaattgtagccttcccatggcatataagctactaa**

**Fortunella_venosa_3** **ttcgctaataagtaccgcatcctcaaaattgtagccttcccatggcatataagctactaa**

27190	27200	27210	27220	27230	27240

....|....|....|....|....|....|....|....|....|....|....|....|

**Atalantia_kwangtungens** **gatgtttttccctaaagcgagttcgccgccaactgtagcggcaccatccgctaaaacttg**

**Citrus_reticulata_1** **tatgtttttccctaaagcgagttcgccgccaactgtagcggcaccatccgctaaaacttg**

**Citrus_reticulata_2** **tatgtttttccctaaagcgagttcgccgccaactgtagcggcaccatccgctaaaacttg**

**Citrus_trifoliata_3** **tatgtttttccctaaagcgagttcgccgccaactgtagcggcaccatccgctaaaacttg**

**Citrus_aurantiifolia** **tatgtttttccctaaagcgagttcgccgccaactgtagcggcaccatccgctaaaacttg**

**Citrus_aurantium** **tatgtttttccctaaagcgagttcgccgccaactgtagcggcaccatccgctaaaacttg**

**Citrus_cavaleriei** **tatgtttttccctaaagcgagttcgccgccaactgtagcggcaccatccgctaaaacttg**

**Citrus_hongheensis** **tatgtttttccctaaagcgagttcgccgccaactgtagcggcaccatccgctaaaacttg**

**Citrus_limon** **tatgtttttccctaaagcgagttcgccgccaactgtagcggcaccatccgctaaaacttg**

**Citrus_maxima** **tatgtttttccctaaagcgagttcgccgccaactgtagcggcaccatccgctaaaacttg**

**Citrus_medica** **tatgtttttccctaaagcgagttcgccgccaactgtagcggcaccatccgctaaaacttg**

**Citrus_polytrifolia** **tatgtttttccctaaagcgagttcgccgccaactgtagcggcaccatccgctaaaacttg**

**Citrus_reticulata** **tatgtttttccctaaagcgagttcgccgccaactgtagcggcaccatccgctaaaacttg**

**Citrus_sinensis** **tatgtttttccctaaagcgagttcgccgccaactgtagcggcaccatccgctaaaacttg**

**Fortunella_bawangica_1** **tatgtttttccctaaagcgagttcgccgccaactgtagcggcaccatccgctaaaacttg**

**Fortunella_bawangica_2** **tatgtttttccctaaagcgagttcgccgccaactgtagcggcaccatccgctaaaacttg**

**Fortunella_hindsii_1** **tatgtttttccctaaagcgagttcgccgccaactgtagcggcaccatccgctaaaacttg**

**Fortunella_hindsii_2** **tatgtttttccctaaagcgagttcgccgccaactgtagcggcaccatccgctaaaacttg**

**Fortunella_hindsii_3** **tatgtttttccctaaagcgagttcgccgccaactgtagcggcaccatccgctaaaacttg**

**Fortunella_japonica_1** **tatgtttttccctaaagcgagttcgccgccaactgtagcggcaccatccgctaaaacttg**

**Fortunella_japonica_2** **tatgtttttccctaaagcgagttcgccgccaactgtagcggcaccatccgctaaaacttg**

**Fortunella_japonica_3** **tatgtttttccctaaagcgagttcgccgccaactgtagcggcaccatccgctaaaacttg**

**Fortunella_japonica_4** **tatgtttttccctaaagcgagttcgccgccaactgtagcggcaccatccgctaaaacttg**

**Fortunella_japonica_5** **tatgtttttccctaaagcgagttcgccgccaactgtagcggcaccatccgctaaaacttg**

**Fortunella_japonica_6** **tatgtttttccctaaagcgagttcgccgccaactgtagcggcaccatccgctaaaacttg**

**Fortunella_japonica_7** **tatgtttttccctaaagcgagttcgccgccaactgtagcggcaccatccgctaaaacttg**

**Fortunella_japonica_8** **tatgtttttccctaaagcgagttcgccgccaactgtagcggcaccatccgctaaaacttg**

**Fortunella_japonica_9** **tatgtttttccctaaagcgagttcgccgccaactgtagcggcaccatccgctaaaacttg**

**Fortunella_japonica_10** **tatgtttttccctaaagcgagttcgccgccaactgtagcggcaccatccgctaaaacttg**

**Fortunella_japonica_11** **tatgtttttccctaaagcgagttcgccgccaactgtagcggcaccatccgctaaaacttg**

**Fortunella_japonica_12** **tatgtttttccctaaagcgagttcgccgccaactgtagcggcaccatccgctaaaacttg**

**Fortunella_margarita_1** **tatgtttttccctaaagcgagttcgccgccaactgtagcggcaccatccgctaaaacttg**

**Fortunella_margarita_2** **tatgtttttccctaaagcgagttcgccgccaactgtagcggcaccatccgctaaaacttg**

**Fortunella_venosa_1** **tatgtttttccctaaagcgagttcgccgccaactgtagcggcaccatccgctaaaacttg**

**Fortunella_venosa_2** **tatgtttttccctaaagcgagttcgccgccaactgtagcggcaccatccgctaaaacttg**

**Fortunella_venosa_3** **tatgtttttccctaaagcgagttcgccgccaactgtagcggcaccatccgctaaaacttg**

27250	27260	27270	27280	27290	27300

....|....|....|....|....|....|....|....|....|....|....|....|

**Atalantia_kwangtungens** **gccctttttaatgcatttaccccgcccaacctggggtttttgatgcatacaagtattttt**

**Citrus_reticulata_1** **gccctttttaatgcatttaccccgcccaacctggggtttttgatgcatacaagtattttt**

**Citrus_reticulata_2** **gccctttttaatgcatttaccccgcccaacctggggtttttgatgcatacaagtattttt**

**Citrus_trifoliata_3** **gccctttttaatgcatttaccccgcccaacctggggtttttgatgcatacaagtattttt**

**Citrus_aurantiifolia** **gccctttttaatgcatttaccccgcccaacctggggtttttgatgcatacaagtattttt**

**Citrus_aurantium** **gccctttttaatgcatttaccccgcccaacctggggtttttgatgcatacaagtattttt**

**Citrus_cavaleriei** **gccctttttaatgcatttaccccgcccaacctggggtttttgatgcatacaagtattttt**

**Citrus_hongheensis** **gccctttttaatgcatttaccccgcccaacctggggtttttgatgcatacaagtattttt**

**Citrus_limon** **gccctttttaatgcatttaccccgcccaacctggggtttttgatgcatacaagtattttt**

**Citrus_maxima** **gccctttttaatgcatttaccccgcccaacctggggtttttgatgcatacaagtattttt**

**Citrus_medica** **gccctttttaatgcatttaccccgcccaacctggggtttttgatgcatacaagtattttt**

**Citrus_polytrifolia** **gccctttttaatgcatttaccccgcccaacctggggtttttgatgcatacaagtattttt**

**Citrus_reticulata** **gccctttttaatgcatttaccccgcccaacctggggtttttgatgcatacaagtattttt**

**Citrus_sinensis** **gccctttttaatgcatttaccccgcccaacctggggtttttgatgcatacaagtattttt**

**Fortunella_bawangica_1** **gccctttttaatgcatttaccccgcccaacctggggtttttgatgcatacaagtattttt**

**Fortunella_bawangica_2** **gccctttttaatgcatttaccccgcccaacctggggtttttgatgcatacaagtattttt**

**Fortunella_hindsii_1** **gccctttttaatgcatttaccccgcccaacctggggtttttgatgcatacaagtattttt**

**Fortunella_hindsii_2** **gccctttttaatgcatttaccccgcccaacctggggtttttgatgcatacaagtattttt**

**Fortunella_hindsii_3** **gccctttttaatgcatttaccccgcccaacctggggtttttgatgcatacaagtattttt**

**Fortunella_japonica_1** **gccctttttaatgcatttaccccgcccaacctggggtttttgatgcatacaagtattttt**

**Fortunella_japonica_2** **gccctttttaatgcatttaccccgcccaacctggggtttttgatgcatacaagtattttt**

**Fortunella_japonica_3** **gccctttttaatgcatttaccccgcccaacctggggtttttgatgcatacaagtattttt**

**Fortunella_japonica_4** **gccctttttaatgcatttaccccgcccaacctggggtttttgatgcatacaagtattttt**

**Fortunella_japonica_5** **gccctttttaatgcatttaccccgcccaacctggggtttttgatgcatacaagtattttt**

**Fortunella_japonica_6** **gccctttttaatgcatttaccccgcccaacctggggtttttgatgcatacaagtattttt**

**Fortunella_japonica_7** **gccctttttaatgcatttaccccgcccaacctggggtttttgatgcatacaagtattttt**

**Fortunella_japonica_8** **gccctttttaatgcatttaccccgcccaacctggggtttttgatgcatacaagtattttt**

**Fortunella_japonica_9** **gccctttttaatgcatttaccccgcccaacctggggtttttgatgcatacaagtattttt**

**Fortunella_japonica_10** **gccctttttaatgcatttaccccgcccaacctggggtttttgatgcatacaagtattttt**

**Fortunella_japonica_11** **gccctttttaatgcatttaccccgcccaacctggggtttttgatgcatacaagtattttt**

**Fortunella_japonica_12** **gccctttttaatgcatttaccccgcccaacctggggtttttgatgcatacaagtattttt**

**Fortunella_margarita_1** **gccctttttaatgcatttaccccgcccaacctggggtttttgatgcatacaagtattttt**

**Fortunella_margarita_2** **gccctttttaatgcatttaccccgcccaacctggggtttttgatgcatacaagtattttt**

**Fortunella_venosa_1** **gccctttttaatgcatttaccccgcccaacctggggtttttgatgcatacaagtattttt**

**Fortunella_venosa_2** **gccctttttaatgcatttaccccgcccaacctggggtttttgatgcatacaagtattttt**

**Fortunella_venosa_3** **gccctttttaatgcatttaccccgcccaacctggggtttttgatgcatacaagtattttt**

27310	27320	27330	27340	27350	27360

....|....|....|....|....|....|....|....|....|....|....|....|

**Atalantia_kwangtungens** **gtttgaacgttgatacataattaatgggatactatatgtattaccgttgcccgataaaac**

**Citrus_reticulata_1** **gtttgaacgttgatacataattaatggaatacaatatgtattcccgtttcccgataaaac**

**Citrus_reticulata_2** **gtttgaacgttgatacataattaatggaatacaatatgtattcccgtttcccgataaaac**

**Citrus_trifoliata_3** **gtttgaacgttgatacataattaatgggatactatatgtattcccgtttcccgataaaac**

**Citrus_aurantiifolia** **gtttgaacgttgatacataattaatgggatactatatgtattaccgtttcccgataaaac**

**Citrus_aurantium** **gtttgaacgttgatacataattaatgggatactatatgtattaccgtttcccgataaaac**

**Citrus_cavaleriei** **gtttgaacgttgatacataattaatgggatactatatgtattaccgtttcccgataaaac**

**Citrus_hongheensis** **gtttgaacgttgatacataattaatgggatactatatgtattaccgtttcccgataaaac**

**Citrus_limon** **gtttgaacgttgatacataattaatgggatactatatgtattaccgtttcccgataaaac**

**Citrus_maxima** **gtttgaacgttgatacataattaatgggatactatatgtattaccgtttcccgataaaac**

**Citrus_medica** **gtttgaacgttgatacataatgaatgggatactatatgtattaccgtttcccgataaaac**

**Citrus_polytrifolia** **gtttgaacgttgatacataattaatgggatactatatgtattcccgtttcccgataaaac**

**Citrus_reticulata** **gtttgaacgttgatacataattaatggaatacaatatgtattcccgtttcccgataaaac**

**Citrus_sinensis** **gtttgaacgttgatacataattaatgggatactatatgtattaccgtttcccgataaaac**

**Fortunella_bawangica_1** **gtttgaacgttgatacataattaatgggatactatatgtattcccgtttcccgataaaac**

**Fortunella_bawangica_2** **gtttgaacgttgatacataattaatgggatactatatgtattcccgtttcccgataaaac**

**Fortunella_hindsii_1** **gtttgaacgttgatacataattaatgggatactatatgtattcccgtttcccgataaaac**

**Fortunella_hindsii_2** **gtttgaacgttgatacataattaatgggatactatatgtattcccgtttcccgataaaac**

**Fortunella_hindsii_3** **gtttgaacgttgatacataattaatgggatactatatgtattcccgtttcccgataaaac**

**Fortunella_japonica_1** **gtttgaacgttgatacataattaatgggatactatatgtattcccgtttcccgataaaac**

**Fortunella_japonica_2** **gtttgaacgttgatacataattaatgggatactatatgtattcccgtttcccgataaaac**

**Fortunella_japonica_3** **gtttgaacgttgatacataattaatgggatactatatgtattcccgtttcccgataaaac**

**Fortunella_japonica_4** **gtttgaacgttgatacataattaatgggatactatatgtattcccgtttcccgataaaac**

**Fortunella_japonica_5** **gtttgaacgttgatacataattaatgggatactatatgtattcccgtttcccgataaaac**

**Fortunella_japonica_6** **gtttgaacgttgatacataattaatgggatactatatgtattcccgtttcccgataaaac**

**Fortunella_japonica_7** **gtttgaacgttgatacataattaatgggatactatatgtattcccgtttcccgataaaac**

**Fortunella_japonica_8** **gtttgaacgttgatacataattaatgggatactatatgtattcccgtttcccgataaaac**

**Fortunella_japonica_9** **gtttgaacgttgatacataattaatgggatactatatgtattcccgtttcccgataaaac**

**Fortunella_japonica_10** **gtttgaacgttgatacataattaatgggatactatatgtattcccgtttcccgataaaac**

**Fortunella_japonica_11** **gtttgaacgttgatacataattaatgggatactatatgtattcccgtttcccgataaaac**

**Fortunella_japonica_12** **gtttgaacgttgatacataattaatgggatactatatgtattcccgtttcccgataaaac**

**Fortunella_margarita_1** **gtttgaacgttgatacataattaatgggatactatatgtattcccgtttcccgataaaac**

**Fortunella_margarita_2** **gtttgaacgttgatacataattaatgggatactatatgtattcccgtttcccgataaaac**

**Fortunella_venosa_1** **gtttgaacgttgatacataattaatgggatactatatgtattcccgtttcccgataaaac**

**Fortunella_venosa_2** **gtttgaacgttgatacataattaatggaatactatatgtattcccgtttcccgataaaac**

**Fortunella_venosa_3** **gtttgaacgttgatacataattaatggaatactatatgtattcccgtttcccgataaaac**

27370	27380	27390	27400	27410	27420

....|....|....|....|....|....|....|....|....|....|....|....|

**Atalantia_kwangtungens** **aatcttgtcaatatcggtataaatgatctttccctcgtgttcggctatagcgggaacccc**

**Citrus_reticulata_1** **aatcttgtcaatatcggtataaatgatctttccctcgtgttcggctatagcgggaacccc**

**Citrus_reticulata_2** **aatcttgtcaatatcggtataaatgatctttccctcgtgttcggctatagcgggaacccc**

**Citrus_trifoliata_3** **aatcttgtcaatatcggtataaatgatctttccctcgtgttcggctatagcgggaacccc**

**Citrus_aurantiifolia** **aatcttgtcaatatcggtataaatgatctttccctcgtgttcggctatagcgggaacccc**

**Citrus_aurantium** **aatcttgtcaatatcggtataaatgatctttccctcgtgttcggctatagcgggaacccc**

**Citrus_cavaleriei** **aatcttgtcaatatcggtataaatgatctttccctcgtgttcggctatagcgggaacccc**

**Citrus_hongheensis** **aatcttgtcaatatcggtataaatgatctttccctcgtgttcggctatagcgggaacccc**

**Citrus_limon** **aatcttgtcaatatcggtataaatgatctttccctcgtgttcggctatagcgggaacccc**

**Citrus_maxima** **aatcttgtcaatatcggtataaatgatctttccctcgtgttcggctatagcgggaacccc**

**Citrus_medica** **aatcttgtcaatatcggtataaatgatctttccctcgtgttcggctatagcgggaacccc**

**Citrus_polytrifolia** **aatcttgtcaatatcggtataaatgatctttccctcgtgttcggctatagcgggaacccc**

**Citrus_reticulata** **aatcttgtcaatatcggtataaatgatctttccctcgtgttcggctatagcgggaacccc**

**Citrus_sinensis** **aatcttgtcaatatcggtataaatgatctttccctcgtgttcggctatagcgggaacccc**

**Fortunella_bawangica_1** **aatcttgtcaatatcggtataaatgatctttccctcgtgttcggctatagcgggaacccc**

**Fortunella_bawangica_2** **aatcttgtcaatatcggtataaatgatctttccctcgtgttcggctatagcgggaacccc**

**Fortunella_hindsii_1** **aatcttgtcaatatcggtataaatgatctttccctcgtgttcggctatagcgggaacccc**

**Fortunella_hindsii_2** **aatcttgtcaatatcggtataaatgatctttccctcgtgttcggctatagcgggaacccc**

**Fortunella_hindsii_3** **aatcttgtcaatatcggtataaatgatctttccctcgtgttcggctatagcgggaacccc**

**Fortunella_japonica_1** **aatcttgtcaatatcggtataaatgatctttccctcgtgttcggctatagcgggaacccc**

**Fortunella_japonica_2** **aatcttgtcaatatcggtataaatgatctttccctcgtgttcggctatagcgggaacccc**

**Fortunella_japonica_3** **aatcttgtcaatatcggtataaatgatctttccctcgtgttcggctatagcgggaacccc**

**Fortunella_japonica_4** **aatcttgtcaatatcggtataaatgatctttccctcgtgttcggctatagcgggaacccc**

**Fortunella_japonica_5** **aatcttgtcaatatcggtataaatgatctttccctcgtgttcggctatagcgggaacccc**

**Fortunella_japonica_6** **aatcttgtcaatatcggtataaatgatctttccctcgtgttcggctatagcgggaacccc**

**Fortunella_japonica_7** **aatcttgtcaatatcggtataaatgatctttccctcgtgttcggctatagcgggaacccc**

**Fortunella_japonica_8** **aatcttgtcaatatcggtataaatgatctttccctcgtgttcggctatagcgggaacccc**

**Fortunella_japonica_9** **aatcttgtcaatatcggtataaatgatctttccctcgtgttcggctatagcgggaacccc**

**Fortunella_japonica_10** **aatcttgtcaatatcggtataaatgatctttccctcgtgttcggctatagcgggaacccc**

**Fortunella_japonica_11** **aatcttgtcaatatcggtataaatgatctttccctcgtgttcggctatagcgggaacccc**

**Fortunella_japonica_12** **aatcttgtcaatatcggtataaatgatctttccctcgtgttcggctatagcgggaacccc**

**Fortunella_margarita_1** **aatcttgtcaatatcggtataaatgatctttccctcgtgttcggctatagcgggaacccc**

**Fortunella_margarita_2** **aatcttgtcaatatcggtataaatgatctttccctcgtgttcggctatagcgggaacccc**

**Fortunella_venosa_1** **aatcttgtcaatatcggtataaatgatctttccctcgtgttcggctatagcgggaacccc**

**Fortunella_venosa_2** **aatcttgtcaatatcggtataaatgatctttccctcgtgttcggctatagcgggaacccc**

**Fortunella_venosa_3** **aatcttgtcaatatcggtataaatgatctttccctcgtgttcggctatagcgggaacccc**

27430	27440	27450	27460	27470	27480

....|....|....|....|....|....|....|....|....|....|....|....|

**Atalantia_kwangtungens** **tgaatctagagccacttgacgttccaacccagttccaacaatgcacttctcggaccgaac**

**Citrus_reticulata_1** **tgaatctagagccacttgaggttccaacccagttccaacaatgcacttctcggaccgaac**

**Citrus_reticulata_2** **tgaatctagagccacttgaggttccaacccagttccaacaatgcacttctcggaccgaac**

**Citrus_trifoliata_3** **tgaatctagagccacttgaggttccaacccagttccaacaatgcacttctcggaccgaac**

**Citrus_aurantiifolia** **tgaatctagagccacttgaggttccaacccagttccaacaatgcacttctcggaccgaac**

**Citrus_aurantium** **tgaatctagagccacttgaggttccaacccagttccaacaatgcacttctcggaccgaac**

**Citrus_cavaleriei** **tgaatctagagccacttgaggttccaacccagttccaacaatgcacttctcggaccgaac**

**Citrus_hongheensis** **tgaatctagagccacttgaggttccaacccagttccaacaatgcacttctcggaccgaac**

**Citrus_limon** **tgaatctagagccacttgaggttccaacccagttccaacaatgcacttctcggaccgaac**

**Citrus_maxima** **tgaatctagagccacttgaggttccaacccagttccaacaatgcacttctcggaccgaac**

**Citrus_medica** **tgaatctagagccacttgaggttccaacccagttccaacaatgcacttctcggaccgaac**

**Citrus_polytrifolia** **tgaatctagagccacttgaggttccaacccagttccaacaatgcacttctcggaccgaac**

**Citrus_reticulata** **tgaatctagagccacttgaggttccaacccagttccaacaatgcacttctcggaccgaac**

**Citrus_sinensis** **tgaatctagagccacttgaggttccaacccagttccaacaatgcacttctcggaccgaac**

**Fortunella_bawangica_1** **tgaatctagagccacttgaggttccaacccagttccaacaatgcacttctcggaccgaac**

**Fortunella_bawangica_2** **tgaatctagagccacttgaggttccaacccagttccaacaatgcacttctcggaccgaac**

**Fortunella_hindsii_1** **tgaatctagagccacttgaggttccaacccagttccaacaatgcacttctcggaccgaac**

**Fortunella_hindsii_2** **tgaatctagagccacttgaggttccaacccagttccaacaatgcacttctcggaccgaac**

**Fortunella_hindsii_3** **tgaatctagagccacttgaggttccaacccagttccaacaatgcacttctcggaccgaac**

**Fortunella_japonica_1** **tgaatctagagccacttgaggttccaacccagttccaacaatgcacttctcggaccgaac**

**Fortunella_japonica_2** **tgaatctagagccacttgaggttccaacccagttccaacaatgcacttctcggaccgaac**

**Fortunella_japonica_3** **tgaatctagagccacttgaggttccaacccagttccaacaatgcacttctcggaccgaac**

**Fortunella_japonica_4** **tgaatctagagccacttgaggttccaacccagttccaacaatgcacttctcggaccgaac**

**Fortunella_japonica_5** **tgaatctagagccacttgaggttccaacccagttccaacaatgcacttctcggaccgaac**

**Fortunella_japonica_6** **tgaatctagagccacttgaggttccaacccagttccaacaatgcacttctcggaccgaac**

**Fortunella_japonica_7** **tgaatctagagccacttgaggttccaacccagttccaacaatgcacttctcggaccgaac**

**Fortunella_japonica_8** **tgaatctagagccacttgaggttccaacccagttccaacaatgcacttctcggaccgaac**

**Fortunella_japonica_9** **tgaatctagagccacttgaggttccaacccagttccaacaatgcacttctcggaccgaac**

**Fortunella_japonica_10** **tgaatctagagccacttgaggttccaacccagttccaacaatgcacttctcggaccgaac**

**Fortunella_japonica_11** **tgaatctagagccacttgaggttccaacccagttccaacaatgcacttctcggaccgaac**

**Fortunella_japonica_12** **tgaatctagagccacttgaggttccaacccagttccaacaatgcacttctcggaccgaac**

**Fortunella_margarita_1** **tgaatctagagccacttgaggttccaacccagttccaacaatgcacttctcggaccgaac**

**Fortunella_margarita_2** **tgaatctagagccacttgaggttccaacccagttccaacaatgcacttctcggaccgaac**

**Fortunella_venosa_1** **tgaatctagagccacttgaggttccaacccagttccaacaatgcacttctcggaccgaac**

**Fortunella_venosa_2** **tgaatctagagccacttgaggttccaacccagttccaacaatgcacttctcggaccgaac**

**Fortunella_venosa_3** **tgaatctagagccacttgaggttccaacccagttccaacaatgcacttctcggaccgaac**

27490	27500	27510	27520	27530	27540

....|....|....|....|....|....|....|....|....|....|....|....|

**Atalantia_kwangtungens** **gagcggaactgcttggcgttgcatattagaactcattaaagcccgattcgcatcattatg**

**Citrus_reticulata_1** **gagcggaactgcttggcgttgcatattagaactcattaaagcccgattcgcatcattatg**

**Citrus_reticulata_2** **gagcggaactgcttggcgttgcatattagaactcattaaagcccgattcgcatcattatg**

**Citrus_trifoliata_3** **gagcggaactgcttggcgttgcatattagaactcattaaagcccgattcgcatcattatg**

**Citrus_aurantiifolia** **gagcggaactgcttggcgttgcatattagaactcattaaagcccgattcgcatcattatg**

**Citrus_aurantium** **gagcggaactgcttggcgttgcatattagaactcattaaagcccgattcgcatcattatg**

**Citrus_cavaleriei** **gagcggaactgcttggcgttgcatattagaactcattaaagcccgattcgcatcattatg**

**Citrus_hongheensis** **gagcggaactgcttggcgttgcatattagaactcattaaagcccgattcgcatcattatg**

**Citrus_limon** **gagcggaactgcttggcgttgcatattagaactcattaaagcccgattcgcatcattatg**

**Citrus_maxima** **gagcggaactgcttggcgttgcatattagaactcattaaagcccgattcgcatcattatg**

**Citrus_medica** **gagcggaactgcttggcgttgcatattagaactcattaaagcccgattcgcatcattatg**

**Citrus_polytrifolia** **gagcggaactgcttggcgttgcatattagaactcattaaagcccgattcgcatcattatg**

**Citrus_reticulata** **gagcggaactgcttggcgttgcatattagaactcattaaagcccgattcgcatcattatg**

**Citrus_sinensis** **gagcggaactgcttggcgttgcatattagaactcattaaagcccgattcgcatcattatg**

**Fortunella_bawangica_1** **gagcggaactgcttggcgttgcatattagaactcattaaagcccgattcgcatcattatg**

**Fortunella_bawangica_2** **gagcggaactgcttggcgttgcatattagaactcattaaagcccgattcgcatcattatg**

**Fortunella_hindsii_1** **gagcggaactgcttggcgttgcatattagaactcattaaagcccgattcgcatcattatg**

**Fortunella_hindsii_2** **gagcggaactgcttggcgttgcatattagaactcattaaagcccgattcgcatcattatg**

**Fortunella_hindsii_3** **gagcggaactgcttggcgttgcatattagaactcattaaagcccgattcgcatcattatg**

**Fortunella_japonica_1** **gagcggaactgcttggcgttgcatattagaactcattaaagcccgattcgcatcattatg**

**Fortunella_japonica_2** **gagcggaactgcttggcgttgcatattagaactcattaaagcccgattcgcatcattatg**

**Fortunella_japonica_3** **gagcggaactgcttggcgttgcatattagaactcattaaagcccgattcgcatcattatg**

**Fortunella_japonica_4** **gagcggaactgcttggcgttgcatattagaactcattaaagcccgattcgcatcattatg**

**Fortunella_japonica_5** **gagcggaactgcttggcgttgcatattagaactcattaaagcccgattcgcatcattatg**

**Fortunella_japonica_6** **gagcggaactgcttggcgttgcatattagaactcattaaagcccgattcgcatcattatg**

**Fortunella_japonica_7** **gagcggaactgcttggcgttgcatattagaactcattaaagcccgattcgcatcattatg**

**Fortunella_japonica_8** **gagcggaactgcttggcgttgcatattagaactcattaaagcccgattcgcatcattatg**

**Fortunella_japonica_9** **gagcggaactgcttggcgttgcatattagaactcattaaagcccgattcgcatcattatg**

**Fortunella_japonica_10** **gagcggaactgcttggcgttgcatattagaactcattaaagcccgattcgcatcattatg**

**Fortunella_japonica_11** **gagcggaactgcttggcgttgcatattagaactcattaaagcccgattcgcatcattatg**

**Fortunella_japonica_12** **gagcggaactgcttggcgttgcatattagaactcattaaagcccgattcgcatcattatg**

**Fortunella_margarita_1** **gagcggaactgcttggcgttgcatattagaactcattaaagcccgattcgcatcattatg**

**Fortunella_margarita_2** **gagcggaactgcttggcgttgcatattagaactcattaaagcccgattcgcatcattatg**

**Fortunella_venosa_1** **gagcggaactgcttggcgttgcatattagaactcattaaagcccgattcgcatcattatg**

**Fortunella_venosa_2** **gagcggaactgcttggcgttgcatattagaactcattaaagcccgattcgcatcattatg**

**Fortunella_venosa_3** **gagcggaactgcttggcgttgcatattagaactcattaaagcccgattcgcatcattatg**

27550	27560	27570	27580	27590	27600

....|....|....|....|....|....|....|....|....|....|....|....|

**Atalantia_kwangtungens** **ctcgataaaaggaatgagggaagccccaatagaaaaatattggaagggaaaaatacttcg**

**Citrus_reticulata_1** **ctcgataaaaggaatgagggaagccccaatagaaaaatattggaagggaaaaatacttcg**

**Citrus_reticulata_2** **ctcgataaaaggaatgagggaagccccaatagaaaaatattggaagggaaaaatacttcg**

**Citrus_trifoliata_3** **ctcgataaaaggaatgagggaagccccaatagaaaaatattggaagggaaaaatacttcg**

**Citrus_aurantiifolia** **ctcgataaaaggaatgagggaagccccaatagaaaaatattggaagggaaaaatacttcg**

**Citrus_aurantium** **ctcgataaaaggaatgagggaagccccaatagaaaaatattggaagggaaaaatacttcg**

**Citrus_cavaleriei** **ctcgataaaaggaatgagggaagccccaatagaaaaatattggaagggaaaaatacttcg**

**Citrus_hongheensis** **ctcgataaaaggaatgagggaagccccaatagaaaaatattggaagggaaaaatacttcg**

**Citrus_limon** **ctcgataaaaggaatgagggaagccccaatagaaaaatattggaagggaaaaatacttcg**

**Citrus_maxima** **ctcgataaaaggaatgagggaagccccaatagaaaaatattggaagggaaaaatacttcg**

**Citrus_medica** **ctcgataaaaggaatgagggaagccccaatagaaaaatattggaagggaaaaatacttcg**

**Citrus_polytrifolia** **ctcgataaaaggaatgagggaagccccaatagaaaaatattggaagggaaaaatacttcg**

**Citrus_reticulata** **ctcgataaaaggaatgagggaagccccaatagaaaaatattggaagggaaaaatacttcg**

**Citrus_sinensis** **ctcgataaaaggaatgagggaagccccaatagaaaaatattggaagggaaaaatacttcg**

**Fortunella_bawangica_1** **ctcgataaaaggaatgagggaagccccaatagaaaaatattggaagggaaaaatacttcg**

**Fortunella_bawangica_2** **ctcgataaaaggaatgagggaagccccaatagaaaaatattggaagggaaaaatacttcg**

**Fortunella_hindsii_1** **ctcgataaaaggaatgagggaagccccaatagaaaaatattggaagggaaaaatacttcg**

**Fortunella_hindsii_2** **ctcgataaaaggaatgagggaagccccaatagaaaaatattggaagggaaaaatacttcg**

**Fortunella_hindsii_3** **ctcgataaaaggaatgagggaagccccaatagaaaaatattggaagggaaaaatacttcg**

**Fortunella_japonica_1** **ctcgataaaaggaatgagggaagccccaatagaaaaatattggaagggaaaaatacttcg**

**Fortunella_japonica_2** **ctcgataaaaggaatgagggaagccccaatagaaaaatattggaagggaaaaatacttcg**

**Fortunella_japonica_3** **ctcgataaaaggaatgagggaagccccaatagaaaaatattggaagggaaaaatacttcg**

**Fortunella_japonica_4** **ctcgataaaaggaatgagggaagccccaatagaaaaatattggaagggaaaaatacttcg**

**Fortunella_japonica_5** **ctcgataaaaggaatgagggaagccccaatagaaaaatattggaagggaaaaatacttcg**

**Fortunella_japonica_6** **ctcgataaaaggaatgagggaagccccaatagaaaaatattggaagggaaaaatacttcg**

**Fortunella_japonica_7** **ctcgataaaaggaatgagggaagccccaatagaaaaatattggaagggaaaaatacttcg**

**Fortunella_japonica_8** **ctcgataaaaggaatgagggaagccccaatagaaaaatattggaagggaaaaatacttcg**

**Fortunella_japonica_9** **ctcgataaaaggaatgagggaagccccaatagaaaaatattggaagggaaaaatacttcg**

**Fortunella_japonica_10** **ctcgataaaaggaatgagggaagccccaatagaaaaatattggaagggaaaaatacttcg**

**Fortunella_japonica_11** **ctcgataaaaggaatgagggaagccccaatagaaaaatattggaagggaaaaatacttcg**

**Fortunella_japonica_12** **ctcgataaaaggaatgagggaagccccaatagaaaaatattggaagggaaaaatacttcg**

**Fortunella_margarita_1** **ctcgataaaaggaatgagggaagccccaatagaaaaatattggaagggaaaaatacttcg**

**Fortunella_margarita_2** **ctcgataaaaggaatgagggaagccccaatagaaaaatattggaagggaaaaatacttcg**

**Fortunella_venosa_1** **ctcgataaaaggaatgagggaagccccaatagaaaaatattggaagggaaaaatacttcg**

**Fortunella_venosa_2** **ctcgataaaaggaatgagggaagccccaatagaaaaatattggaagggaaaaatacttcg**

**Fortunella_venosa_3** **ctcgataaaaggaatgagggaagccccaatagaaaaatattggaagggaaaaatacttcg**

27610	27620	27630	27640	27650	27660

....|....|....|....|....|....|....|....|....|....|....|....|

**Atalantia_kwangtungens** **aagatgaacctgttcccacgcaatagtcaggaactcttgacggtatcgagttggaacaac**

**Citrus_reticulata_1** **aagatgaacctgttcccacgcaatagtcaggaactcttgacggtatcgagtcggaacaac**

**Citrus_reticulata_2** **aagatgaacctgttcccacgcaatagtcaggaactcttgacggtatcgagtcggaacaac**

**Citrus_trifoliata_3** **aagatgaacctgttcccacgcaatagtcaggaactcttgacggtatcgagtcggaacaac**

**Citrus_aurantiifolia** **aagatgaacctgttcccacgcaatagtcaggaactcttgacggtatcgagtcggaacaac**

**Citrus_aurantium** **aagatgaacctgttcccacgcaatagtcaggaactcttgacggtatcgagtcggaacaac**

**Citrus_cavaleriei** **aagatgaacctgttcccacgcaatagtcaggaactcttgacggtatcgagtcggaacaac**

**Citrus_hongheensis** **aagatgaacctgttcccacgcaatagtcaggaactcttgacggtatcgagtcggaacaac**

**Citrus_limon** **aagatgaacctgttcccacgcaatagtcaggaactcttgacggtatcgagtcggaacaac**

**Citrus_maxima** **aagatgaacctgttcccacgcaatagtcaggaactcttgacggtatcgagtcggaacaac**

**Citrus_medica** **aagatgaacctgttcccacgcaatagtcaggaactcttgacggtatcgagtcggaacaac**

**Citrus_polytrifolia** **aagatgaacctgttcccacgcaatagtcaggaactcttgacggtatcgagtcggaacaac**

**Citrus_reticulata** **aagatgaacctgttcccacgcaatagtcaggaactcttgacggtatcgagtcggaacaac**

**Citrus_sinensis** **aagatgaacctgttcccacgcaatagtcaggaactcttgacggtatcgagtcggaacaac**

**Fortunella_bawangica_1** **aagatgaacctgttcccacgcaatagtcaggaactcttgacggtatcgagtcggaacaac**

**Fortunella_bawangica_2** **aagatgaacctgttcccacgcaatagtcaggaactcttgacggtatcgagtcggaacaac**

**Fortunella_hindsii_1** **aagatgaacctgttcccacgcaatagtcaggaactcttgacggtatcgagtcggaacaac**

**Fortunella_hindsii_2** **aagatgaacctgttcccacgcaatagtcaggaactcttgacggtatcgagtcggaacaac**

**Fortunella_hindsii_3** **aagatgaacctgttcccacgcaatagtcaggaactcttgacggtatcgagtcggaacaac**

**Fortunella_japonica_1** **aagatgaacctgttcccacgcaatagtcaggaactcttgacggtatcgagtcggaacaac**

**Fortunella_japonica_2** **aagatgaacctgttcccacgcaatagtcaggaactcttgacggtatcgagtcggaacaac**

**Fortunella_japonica_3** **aagatgaacctgttcccacgcaatagtcaggaactcttgacggtatcgagtcggaacaac**

**Fortunella_japonica_4** **aagatgaacctgttcccacgcaatagtcaggaactcttgacggtatcgagtcggaacaac**

**Fortunella_japonica_5** **aagatgaacctgttcccacgcaatagtcaggaactcttgacggtatcgagtcggaacaac**

**Fortunella_japonica_6** **aagatgaacctgttcccacgcaatagtcaggaactcttgacggtatcgagtcggaacaac**

**Fortunella_japonica_7** **aagatgaacctgttcccacgcaatagtcaggaactcttgacggtatcgagtcggaacaac**

**Fortunella_japonica_8** **aagatgaacctgttcccacgcaatagtcaggaactcttgacggtatcgagtcggaacaac**

**Fortunella_japonica_9** **aagatgaacctgttcccacgcaatagtcaggaactcttgacggtatcgagtcggaacaac**

**Fortunella_japonica_10** **aagatgaacctgttcccacgcaatagtcaggaactcttgacggtatcgagtcggaacaac**

**Fortunella_japonica_11** **aagatgaacctgttcccacgcaatagtcaggaactcttgacggtatcgagtcggaacaac**

**Fortunella_japonica_12** **aagatgaacctgttcccacgcaatagtcaggaactcttgacggtatcgagtcggaacaac**

**Fortunella_margarita_1** **aagatgaacctgttcccacgcaatagtcaggaactcttgacggtatcgagtcggaacaac**

**Fortunella_margarita_2** **aagatgaacctgttcccacgcaatagtcaggaactcttgacggtatcgagtcggaacaac**

**Fortunella_venosa_1** **aagatgaacctgttcccacgcaatagtcaggaactcttgacggtatcgagtcggaacaac**

**Fortunella_venosa_2** **aagatgaacctgttcccacgcaatagtcaggaactcttgacggtatcgagtcggaacaac**

**Fortunella_venosa_3** **aagatgaacctgttcccacgcaatagtcaggaactcttgacggtatcgagtcggaacaac**

27670	27680	27690	27700	27710	27720

....|....|....|....|....|....|....|....|....|....|....|....|

**Atalantia_kwangtungens** **ctgttcttccggactaccctgattcagtgccaaagaatttcctgccgctaccatatagta**

**Citrus_reticulata_1** **ctgttcttccggactaccctgattcagtgccaaagaatttcctgccgctaccatatagta**

**Citrus_reticulata_2** **ctgttcttccggactaccctgattcagtgccaaagaatttcctgccgctaccatatagta**

**Citrus_trifoliata_3** **ctgttcttccggactaccctgattcagtgccaaagaatttcctgccgctaccatatagta**

**Citrus_aurantiifolia** **ctgttcttccggactaccctgattcagtgccaaagaatttcctgccgctaccatatagta**

**Citrus_aurantium** **ctgttcttccggactaccctgattcagtgccaaagaatttcctgccgctaccatatagta**

**Citrus_cavaleriei** **ctgttcttccggactaccctgattcagtgccaacgaatttcctgccgctaccatatagta**

**Citrus_hongheensis** **ctgttcttccggactaccctgattcagtgccaaagaatttcctgccgctaccatatagta**

**Citrus_limon** **ctgttcttccggactaccctgattcagtgccaaagaatttcctgccgctaccatatagta**

**Citrus_maxima** **ctgttcttccggactaccctgattcagtgccaaagaatttcctgccgctaccatatagta**

**Citrus_medica** **ctgttcttccggactaccctgattcagtgccaaagaatttcctgccgctaccatatagta**

**Citrus_polytrifolia** **ctgttcttccggactaccctgattcagtgccaaagaatttcctgccgctaccatatagta**

**Citrus_reticulata** **ctgttcttccggactaccctgattcagtgccaaagaatttcctgccgctaccatatagta**

**Citrus_sinensis** **ctgttcttccggactaccctgattcagtgccaaagaatttcctgccgctaccatatagta**

**Fortunella_bawangica_1** **ctgttcttccggactaccctgattcagtgccaaagaatttcctgccgctaccatatagta**

**Fortunella_bawangica_2** **ctgttcttccggactaccctgattcagtgccaaagaatttcctgccgctaccatatagta**

**Fortunella_hindsii_1** **ctgttcttccggactaccctgattcagtgccaaagaatttcctgccgctaccatatagta**

**Fortunella_hindsii_2** **ctgttcttccggactaccctgattcagtgccaaagaatttcctgccgctaccatatagta**

**Fortunella_hindsii_3** **ctgttcttccggactaccctgattcagtgccaaagaatttcctgccgctaccatatagta**

**Fortunella_japonica_1** **ctgttcttccggactaccctgattcagtgccaaagaatttcctgccgctaccatatagta**

**Fortunella_japonica_2** **ctgttcttccggactaccctgattcagtgccaaagaatttcctgccgctaccatatagta**

**Fortunella_japonica_3** **ctgttcttccggactaccctgattcagtgccaaagaatttcctgccgctaccatatagta**

**Fortunella_japonica_4** **ctgttcttccggactaccctgattcagtgccaaagaatttcctgccgctaccatatagta**

**Fortunella_japonica_5** **ctgttcttccggactaccctgattcagtgccaaagaatttcctgccgctaccatatagta**

**Fortunella_japonica_6** **ctgttcttccggactaccctgattcagtgccaaagaatttcctgccgctaccatatagta**

**Fortunella_japonica_7** **ctgttcttccggactaccctgattcagtgccaaagaatttcctgccgctaccatatagta**

**Fortunella_japonica_8** **ctgttcttccggactaccctgattcagtgccaaagaatttcctgccgctaccatatagta**

**Fortunella_japonica_9** **ctgttcttccggactaccctgattcagtgccaaagaatttcctgccgctaccatatagta**

**Fortunella_japonica_10** **ctgttcttccggactaccctgattcagtgccaaagaatttcctgccgctaccatatagta**

**Fortunella_japonica_11** **ctgttcttccggactaccctgattcagtgccaaagaatttcctgccgctaccatatagta**

**Fortunella_japonica_12** **ctgttcttccggactaccctgattcagtgccaaagaatttcctgccgctaccatatagta**

**Fortunella_margarita_1** **ctgttcttccggactaccctgattcagtgccaaagaatttcctgccgctaccatatagta**

**Fortunella_margarita_2** **ctgttcttccggactaccctgattcagtgccaaagaatttcctgccgctaccatatagta**

**Fortunella_venosa_1** **ctgttcttccggactaccctgattcagtgccaaagaatttcctgccgctaccatatagta**

**Fortunella_venosa_2** **ctgttcttccggactaccctgattcagtgccaaagaatttcctgccgctaccatatagta**

**Fortunella_venosa_3** **ctgttcttccggactaccctgattcagtgccaaagaatttcctgccgctaccatatagta**

27730	27740	27750	27760	27770	27780

....|....|....|....|....|....|....|....|....|....|....|....|

**Atalantia_kwangtungens** **ttcatctctacttggtgataaataaagcatccgtactttttttgatttttcaaagatttc**

**Citrus_reticulata_1** **ttcatctctacttggtgataaataaagcatccgtactttttttgatttttcaaagatttc**

**Citrus_reticulata_2** **ttcatctctacttggtgataaataaagcatccgtactttttttgatttttcaaagatttc**

**Citrus_trifoliata_3** **ttcatctctacttggtgataaataaagcatccgtactttttttgatttttcaaagatttc**

**Citrus_aurantiifolia** **ttcatctctacttggtgataaataaagcatccgtactttttttgatttttcaaagatttc**

**Citrus_aurantium** **ttcatctctacttggtgataaataaagcatccgtactttttttgatttttcaaagatttc**

**Citrus_cavaleriei** **ttcatctctacttggtgataaataaagcatccgtactttttttgatttttcaaagatttc**

**Citrus_hongheensis** **ttcatctctacttggtgataaataaagcatccgtactttttttgatttttcaaagatttc**

**Citrus_limon** **ttcatctctacttggtgataaataaagcatccgtactttttttgatttttcaaagatttc**

**Citrus_maxima** **ttcatctctacttggtgataaataaagcatccgtactttttttgatttttcaaagatttc**

**Citrus_medica** **ttcatctctacttggtgataaataaagcatccgtactgtttttgatttttcaaagatttc**

**Citrus_polytrifolia** **ttcatctctacttggtgataaataaagcatccgtactttttttgatttttcaaagatttc**

**Citrus_reticulata** **ttcatctctacttggtgataaataaagcatccgtactttttttgatttttcaaagatttc**

**Citrus_sinensis** **ttcatctctacttggtgataaataaagcatccgtactttttttgatttttcaaagatttc**

**Fortunella_bawangica_1** **ttcatctctacttggtgataaataaagcatccgtactttttttgatttttcaaagatttc**

**Fortunella_bawangica_2** **ttcatctctacttggtgataaataaagcatccgtactttttttgatttttcaaagatttc**

**Fortunella_hindsii_1** **ttcatctctacttggtgataaataaagcatccgtactttttttgatttttcaaagatttc**

**Fortunella_hindsii_2** **ttcatctctacttggtgataaataaagcatccgtactttttttgatttttcaaagatttc**

**Fortunella_hindsii_3** **ttcatctctacttggtgataaataaagcatccgtactttttttgatttttcaaagatttc**

**Fortunella_japonica_1** **ttcatctctacttggtgataaataaagcatccgtactttttttgatttttcaaagatttc**

**Fortunella_japonica_2** **ttcatctctacttggtgataaataaagcatccgtactttttttgatttttcaaagatttc**

**Fortunella_japonica_3** **ttcatctctacttggtgataaataaagcatccgtactttttttgatttttcaaagatttc**

**Fortunella_japonica_4** **ttcatctctacttggtgataaataaagcatccgtactttttttgatttttcaaagatttc**

**Fortunella_japonica_5** **ttcatctctacttggtgataaataaagcatccgtactttttttgatttttcaaagatttc**

**Fortunella_japonica_6** **ttcatctctacttggtgataaataaagcatccgtactttttttgatttttcaaagatttc**

**Fortunella_japonica_7** **ttcatctctacttggtgataaataaagcatccgtactttttttgatttttcaaagatttc**

**Fortunella_japonica_8** **ttcatctctacttggtgataaataaagcatccgtactttttttgatttttcaaagatttc**

**Fortunella_japonica_9** **ttcatctctacttggtgataaataaagcatccgtactttttttgatttttcaaagatttc**

**Fortunella_japonica_10** **ttcatctctacttggtgataaataaagcatccgtactttttttgatttttcaaagatttc**

**Fortunella_japonica_11** **ttcatctctacttggtgataaataaagcatccgtactttttttgatttttcaaagatttc**

**Fortunella_japonica_12** **ttcatctctacttggtgataaataaagcatccgtactttttttgatttttcaaagatttc**

**Fortunella_margarita_1** **ttcatctctacttggtgataaataaagcatccgtactttttttgatttttcaaagatttc**

**Fortunella_margarita_2** **ttcatctctacttggtgataaataaagcatccgtactttttttgatttttcaaagatttc**

**Fortunella_venosa_1** **ttcatctctacttggtgataaataaagcatccgtactttttttgatttttcaaagatttc**

**Fortunella_venosa_2** **ttcatctctacttggtgataaataaagcatccgtactttttttgatttttcaaagatttc**

**Fortunella_venosa_3** **ttcatctctacttggtgataaataaagcatccgtactttttttgatttttcaaagatttc**

27790	27800	27810	27820	27830	27840

....|....|....|....|....|....|....|....|....|....|....|....|

**Atalantia_kwangtungens** **ataaaacgggctttctagagacccccaataaccaattctcgcatgaattgctaaggatcc**

**Citrus_reticulata_1** **ataaaacgggctttctagagacccccaataaccaattctcgcatgaattgctaaggatcc**

**Citrus_reticulata_2** **ataaaacgggctttctagagacccccaataaccaattctcgcatgaattgctaaggatcc**

**Citrus_trifoliata_3** **ataaaacgggctttctagagacccccaataaccaattctcgcatgaattgctaaggatcc**

**Citrus_aurantiifolia** **ataaaacgggctttctagagacccccaataaccaattctcgcatgaattgctaaggatcc**

**Citrus_aurantium** **ataaaacgggctttctagagacccccaataaccaattctcgcatgaattgctaaggatcc**

**Citrus_cavaleriei** **ataaaacgggctttctagagacccccaataaccaattctcgcatgaattgctaaggatcc**

**Citrus_hongheensis** **ataaaacgggctttctagagacccccaataaccaattctcgcatgaattgctaaggatcc**

**Citrus_limon** **ataaaacgggctttctagagacccccaataaccaattctcgcatgaattgctaaggatcc**

**Citrus_maxima** **ataaaacgggctttctagagacccccaataaccaattctcgcatgaattgctaaggatcc**

**Citrus_medica** **ataaaacgggctttctagagacccccaataaccaattctcgcatgaattgctaaggatcc**

**Citrus_polytrifolia** **ataaaacgggctttctagagacccccaataaccaattctcgcatgaattgctaaggatcc**

**Citrus_reticulata** **ataaaacgggctttctagagacccccaataaccaattctcgcatgaattgctaaggatcc**

**Citrus_sinensis** **ataaaacgggctttctagagacccccaataaccaattctcgcatgaattgctaaggatcc**

**Fortunella_bawangica_1** **ataaaacgggctttctagagacccccaataaccaattctcgcatgaattgctaaggatcc**

**Fortunella_bawangica_2** **ataaaacgggctttctagagacccccaataaccaattctcgcatgaattgctaaggatcc**

**Fortunella_hindsii_1** **ataaaacgggctttctagagacccccaataaccaattctcgcatgaattgctaaggatcc**

**Fortunella_hindsii_2** **ataaaacgggctttctagagacccccaataaccaattctcgcatgaattgctaaggatcc**

**Fortunella_hindsii_3** **ataaaacgggctttctagagacccccaataaccaattctcgcatgaattgctaaggatcc**

**Fortunella_japonica_1** **ataaaacgggctttctagagacccccaataaccaattctcgcatgaattgctaaggatcc**

**Fortunella_japonica_2** **ataaaacgggctttctagagacccccaataaccaattctcgcatgaattgctaaggatcc**

**Fortunella_japonica_3** **ataaaacgggctttctagagacccccaataaccaattctcgcatgaattgctaaggatcc**

**Fortunella_japonica_4** **ataaaacgggctttctagagacccccaataaccaattctcgcatgaattgctaaggatcc**

**Fortunella_japonica_5** **ataaaacgggctttctagagacccccaataaccaattctcgcatgaattgctaaggatcc**

**Fortunella_japonica_6** **ataaaacgggctttctagagacccccaataaccaattctcgcatgaattgctaaggatcc**

**Fortunella_japonica_7** **ataaaacgggctttctagagacccccaataaccaattctcgcatgaattgctaaggatcc**

**Fortunella_japonica_8** **ataaaacgggctttctagagacccccaataaccaattctcgcatgaattgctaaggatcc**

**Fortunella_japonica_9** **ataaaacgggctttctagagacccccaataaccaattctcgcatgaattgctaaggatcc**

**Fortunella_japonica_10** **ataaaacgggctttctagagacccccaataaccaattctcgcatgaattgctaaggatcc**

**Fortunella_japonica_11** **ataaaacgggctttctagagacccccaataaccaattctcgcatgaattgctaaggatcc**

**Fortunella_japonica_12** **ataaaacgggctttctagagacccccaataaccaattctcgcatgaattgctaaggatcc**

**Fortunella_margarita_1** **ataaaacgggctttctagagacccccaataaccaattctcgcatgaattgctaaggatcc**

**Fortunella_margarita_2** **ataaaacgggctttctagagacccccaataaccaattctcgcatgaattgctaaggatcc**

**Fortunella_venosa_1** **ataaaacgggctttctagagacccccaataaccaattctcgcatgaattgctaaggatcc**

**Fortunella_venosa_2** **ataaaacgggctttctagagacccccaataaccaattctcgcatgaattgctaaggatcc**

**Fortunella_venosa_3** **ataaaacgggctttctagagacccccaataaccaattctcgcatgaattgctaaggatcc**

27850	27860	27870	27880	27890	27900

....|....|....|....|....|....|....|....|....|....|....|....|

**Atalantia_kwangtungens** **aataagtccaacattgattccttcggacgtgtcaattggacaaatgcggccatagtgact**

**Citrus_reticulata_1** **aataagtccaacattgattccttcggacgtgtcaattggacaaatgcggccatagtgact**

**Citrus_reticulata_2** **aataagtccaacattgattccttcggacgtgtcaattggacaaatgcggccatagtgact**

**Citrus_trifoliata_3** **aataagtccaacattgattccttcggacgtgtcaattggacaaatgcggccatagtgact**

**Citrus_aurantiifolia** **aataagtccaacattgattccttcggacgtgtcaattggacaaatgcggccatagtgact**

**Citrus_aurantium** **aataagtccaacattgattccttcggacgtgtcaattggacaaatgcggccatagtgact**

**Citrus_cavaleriei** **aataagtccaacattgattccttcggacgtgtcaattggacaaatgcggccatagtgact**

**Citrus_hongheensis** **aataagtccaacattgattccttcggacgtgtcaattggacaaatgcggccatagtgact**

**Citrus_limon** **aataagtccaacattgattccttcggacgtgtcaattggacaaatgcggccatagtgact**

**Citrus_maxima** **aataagtccaacattgattccttcggacgtgtcaattggacaaatgcggccatagtgact**

**Citrus_medica** **aataagtccaacattgattccttcggacgtgtcaattggacaaatgcggccatagtgact**

**Citrus_polytrifolia** **aataagtccaacattgattccttcggacgtgtcaattggacaaatgcggccatagtgact**

**Citrus_reticulata** **aataagtccaacattgattccttcggacgtgtcaattggacaaatgcggccatagtgact**

**Citrus_sinensis** **aataagtccaacattgattccttcggacgtgtcaattggacaaatgcggccatagtgact**

**Fortunella_bawangica_1** **aataagtccaacattgattccttcggacgtgtcaattggacaaatgcgtccatagtgact**

**Fortunella_bawangica_2** **aataagtccaacattgattccttcggacgtgtcaattggacaaatgcgtccatagtgact**

**Fortunella_hindsii_1** **aataagtccaacattgattccttcggacgtgtcaattggacaaatgcggccatagtgact**

**Fortunella_hindsii_2** **aataagtccaacattgattccttcggacgtgtcaattggacaaatgcggccatagtgact**

**Fortunella_hindsii_3** **aataagtccaacattgattccttcggacgtgtcaattggacaaatgcggccatagtgact**

**Fortunella_japonica_1** **aataagtccaacattgattccttcggacgtgtcaattggacaaatgcggccatagtgact**

**Fortunella_japonica_2** **aataagtccaacattgattccttcggacgtgtcaattggacaaatgcggccatagtgact**

**Fortunella_japonica_3** **aataagtccaacattgattccttcggacgtgtcaattggacaaatgcggccatagtgact**

**Fortunella_japonica_4** **aataagtccaacattgattccttcggacgtgtcaattggacaaatgcggccatagtgact**

**Fortunella_japonica_5** **aataagtccaacattgattccttcggacgtgtcaattggacaaatgcggccatagtgact**

**Fortunella_japonica_6** **aataagtccaacattgattccttcggacgtgtcaattggacaaatgcggccatagtgact**

**Fortunella_japonica_7** **aataagtccaacattgattccttcggacgtgtcaattggacaaatgcggccatagtgact**

**Fortunella_japonica_8** **aataagtccaacattgattccttcggacgtgtcaattggacaaatgcggccatagtgact**

**Fortunella_japonica_9** **aataagtccaacattgattccttcggacgtgtcaattggacaaatgcggccatagtgact**

**Fortunella_japonica_10** **aataagtccaacattgattccttcggacgtgtcaattggacaaatgcgtccatagtgact**

**Fortunella_japonica_11** **aataagtccaacattgattccttcggacgtgtcaattggacaaatgcggccatagtgact**

**Fortunella_japonica_12** **aataagtccaacattgattccttcggacgtgtcaattggacaaatgcggccatagtgact**

**Fortunella_margarita_1** **aataagtccaacattgattccttcggacgtgtcaattggacaaatgcgtccatagtgact**

**Fortunella_margarita_2** **aataagtccaacattgattccttcggacgtgtcaattggacaaatgcgtccatagtgact**

**Fortunella_venosa_1** **aataagtccaacattgattccttcggacgtgtcaattggacaaatgcggccatagtgact**

**Fortunella_venosa_2** **aataagtccaacattgattccttcggacgtgtcaattggacaaatgcggccatagtgact**

**Fortunella_venosa_3** **aataagtccaacattgattccttcggacgtgtcaattggacaaatgcggccatagtgact**

27910	27920	27930	27940	27950	27960

....|....|....|....|....|....|....|....|....|....|....|....|

**Atalantia_kwangtungens** **aggatggatatctcgtacccgaaaactggcagttcgccctgtcaatcctccggggcccaa**

**Citrus_reticulata_1** **aggatggatatctcgtacccgaaaactcgcagttcgccctgtcaatcctccggggcccaa**

**Citrus_reticulata_2** **aggatggatatctcgtacccgaaaactcgcagttcgccctgtcaatcctccggggcccaa**

**Citrus_trifoliata_3** **aggatggatatctcgtacccgaaaactcgcagttcgccctgtcaatcctccggggcccaa**

**Citrus_aurantiifolia** **aggatggatatctcgtacccgaaaactcgcagttcgccctgtcaatcctccggggcccaa**

**Citrus_aurantium** **aggatggatatctcgtacccgaaaactcgcagttcgccctgtcaatcctccggggcccaa**

**Citrus_cavaleriei** **aggatggatatctcgtacccgaaaactcgcagttcgccctgtcaatcctccggggcccaa**

**Citrus_hongheensis** **aggatggatatctcgtacccgaaaactcgcagttcgccctgtcaatcctccggggcccaa**

**Citrus_limon** **aggatggatatctcgtacccgaaaactcgcagttcgccctgtcaatcctccggggcccaa**

**Citrus_maxima** **aggatggatatctcgtacccgaaaactcgcagttcgccctgtcaatcctccggggcccaa**

**Citrus_medica** **aggatggatatctcgtacccgaaaactcgcagttcgccctgtcaatcctccggggcccaa**

**Citrus_polytrifolia** **aggatggatatctcgtacccgaaaactcgcagttcgccctgtcaatcctccggggcccaa**

**Citrus_reticulata** **aggatggatatctcgtacccgaaaactcgcagttcgccctgtcaatcctccggggcccaa**

**Citrus_sinensis** **aggatggatatctcgtacccgaaaactcgcagttcgccctgtcaatcctccggggcccaa**

**Fortunella_bawangica_1** **aggatggatatctcgtacccgaaaactcgcagttcgccctgtcaatcctccggggcccaa**

**Fortunella_bawangica_2** **aggatggatatctcgtacccgaaaactcgcagttcgccctgtcaatcctccggggcccaa**

**Fortunella_hindsii_1** **aggatggatatctcgtacccgaaaactcgcagttcgccctgtcaatcctccggggcccaa**

**Fortunella_hindsii_2** **aggatggatatctcgtacccgaaaactcgcagttcgccctgtcaatcctccggggcccaa**

**Fortunella_hindsii_3** **aggatggatatctcgtacccgaaaactcgcagttcgccctgtcaatcctccggggcccaa**

**Fortunella_japonica_1** **aggatggatatctcggacccgaaaactcgcagttcgccctgtcaatcctccggggcccaa**

**Fortunella_japonica_2** **aggatggatatctcgtacccgaaaactcgcagttcgccctgtcaatcctccggggcccaa**

**Fortunella_japonica_3** **aggatggatatctcggacccgaaaactcgcagttcgccctgtcaatcctccggggcccaa**

**Fortunella_japonica_4** **aggatggatatctcggacccgaaaactcgcagttcgccctgtcaatcctccggggcccaa**

**Fortunella_japonica_5** **aggatggatatctcggacccgaaaactcgcagttcgccctgtcaatcctccggggcccaa**

**Fortunella_japonica_6** **aggatggatatctcggacccgaaaactcgcagttcgccctgtcaatcctccggggcccaa**

**Fortunella_japonica_7** **aggatggatatctcggacccgaaaactcgcagttcgccctgtcaatcctccggggcccaa**

**Fortunella_japonica_8** **aggatggatatctcggacccgaaaactcgcagttcgccctgtcaatcctccggggcccaa**

**Fortunella_japonica_9** **aggatggatatctcggacccgaaaactcgcagttcgccctgtcaatcctccggggcccaa**

**Fortunella_japonica_10** **aggatggatatctcgtacccgaaaactcgcagttcgccctgtcaatcctccggggcccaa**

**Fortunella_japonica_11** **aggatggatatctcgtacccgaaaactcgcagttcgccctgtcaatcctccggggcccaa**

**Fortunella_japonica_12** **aggatggatatctcggacccgaaaactcgcagttcgccctgtcaatcctccggggcccaa**

**Fortunella_margarita_1** **aggatggatatctcgtacccgaaaactcgcagttcgccctgtcaatcctccggggcccaa**

**Fortunella_margarita_2** **aggatggatatctcgtacccgaaaactcgcagttcgccctgtcaatcctccggggcccaa**

**Fortunella_venosa_1** **aggatggatatctcgtacccgaaaactcgcagttcgccctgtcaatcctccggggcccaa**

**Fortunella_venosa_2** **aggatggatatctcgtacccgaaaactcgcagttcgccctgtcaatcctccggggcccaa**

**Fortunella_venosa_3** **aggatggatatctcgtacccgaaaactcgcagttcgccctgtcaatcctccggggcccaa**

27970	27980	27990	28000	28010	28020

....|....|....|....|....|....|....|....|....|....|....|....|

**Atalantia_kwangtungens** **ataactcaattttctcccatgaactatttgtgtcaatggattagttcgatccaaaacttg**

**Citrus_reticulata_1** **ataactcaattttctcccatgaactatttgtgtcaatggattagttcgatccaaaacttg**

**Citrus_reticulata_2** **ataactcaattttctcccatgaactatttgtgtcaatggattagttcgatccaaaacttg**

**Citrus_trifoliata_3** **ataactcaattttctcccatgaactatttgtgtcaatggattagttcgatccaaaacttg**

**Citrus_aurantiifolia** **ataactcaattttctcccatgaactatttgtgtcaatggattagttcgatccaaaacttg**

**Citrus_aurantium** **ataactcaattttctcccatgaactatttgtgtcaatgggttagttcgatccaaaacttg**

**Citrus_cavaleriei** **ataactcaattttctcccatgaactatttgtgtcaatggattagttcgatccaaaacttg**

**Citrus_hongheensis** **ataactcaattttctcccatgaactatttgtgtcaatgggttagttcgatccaaaacttg**

**Citrus_limon** **ataactcaattttctcccatgaactatttgtgtcaatgggttagttcgatccaaaacttg**

**Citrus_maxima** **ataactcaattttctcccatgaactatttgtgtcaatgggttagttcgatccaaaacttg**

**Citrus_medica** **ataactcaattttctcccatgaactatttgtgtcaatggattagttcgatccaaaacttg**

**Citrus_polytrifolia** **ataactcaattttctcccatgaactatttgtgtcaatggattagttcgatccaaaacttg**

**Citrus_reticulata** **ataactcaattttctcccatgaactatttgtgtcaatggattagttcgatccaaaacttg**

**Citrus_sinensis** **ataactcaattttctcccatgaactatttgtgtcaatgggttagttcgatccaaaacttg**

**Fortunella_bawangica_1** **ataactcaattttctcccatgaactatttgtgtcaatggattagttcgatccaaaacttg**

**Fortunella_bawangica_2** **ataactcaattttctcccatgaactatttgtgtcaatggattagttcgatccaaaacttg**

**Fortunella_hindsii_1** **ataactcaattttctcccatgaactatttgtgtcaatggattagttcgatccaaaacttg**

**Fortunella_hindsii_2** **ataactcaattttctcccatgaactatttgtgtcaatggattagttcgatccaaaacttg**

**Fortunella_hindsii_3** **ataactcaattttctcccatgaactatttgtgtcaatggattagttcgatccaaaacttg**

**Fortunella_japonica_1** **ataactcaattttctcccatgaactatttgtgtcaatggattagttcgatccaaaacttg**

**Fortunella_japonica_2** **ataactcaattttctcccatgaactatttgtgtcaatggattagttcgatccaaaacttg**

**Fortunella_japonica_3** **ataactcaattttctcccatgaactatttgtgtcaatggattagttcgatccaaaacttg**

**Fortunella_japonica_4** **ataactcaattttctcccatgaactatttgtgtcaatggattagttcgatccaaaacttg**

**Fortunella_japonica_5** **ataactcaattttctcccatgaactatttgtgtcaatggattagttcgatccaaaacttg**

**Fortunella_japonica_6** **ataactcaattttctcccatgaactatttgtgtcaatggattagttcgatccaaaacttg**

**Fortunella_japonica_7** **ataactcaattttctcccatgaactatttgtgtcaatggattagttcgatccaaaacttg**

**Fortunella_japonica_8** **ataactcaattttctcccatgaactatttgtgtcaatggattagttcgatccaaaacttg**

**Fortunella_japonica_9** **ataactcaattttctcccatgaactatttgtgtcaatggattagttcgatccaaaacttg**

**Fortunella_japonica_10** **ataactcaattttctcccatgaactatttgtgtcaatggattagttcgatccaaaacttg**

**Fortunella_japonica_11** **ataactcaattttctcccatgaactatttgtgtcaatggattagttcgatccaaaacttg**

**Fortunella_japonica_12** **ataactcaattttctcccatgaactatttgtgtcaatggattagttcgatccaaaacttg**

**Fortunella_margarita_1** **ataactcaattttctcccatgaactatttgtgtcaatggattagttcgatccaaaacttg**

**Fortunella_margarita_2** **ataactcaattttctcccatgaactatttgtgtcaatggattagttcgatccaaaacttg**

**Fortunella_venosa_1** **ataactcaattttctcccatgaactatttgtgtcaatggattagttcgatccaaaacttg**

**Fortunella_venosa_2** **ataactcaattttctcccatgaactatttgtgtcaatggattagttcgatccaaaacttg**

**Fortunella_venosa_3** **ataactcaattttctcccatgaactatttgtgtcaatggattagttcgatccaaaacttg**

28030	28040	28050	28060	28070	28080

....|....|....|....|....|....|....|....|....|....|....|....|

**Atalantia_kwangtungens** **agataatggatgtaatccgaaaaaagaatcataagtggttgttaagggagttgaagttac**

**Citrus_reticulata_1** **agataatggatgtaatccgaaaaaagaatcataagtggttgttaagggagttgaagttac**

**Citrus_reticulata_2** **agataatggatgtaatccgaaaaaagaatcataagtggttgttaagggagttgaagttac**

**Citrus_trifoliata_3** **agataatggatgtaatccgaaaaaagaatcataagtggttgttaagggagttgaagttac**

**Citrus_aurantiifolia** **agataatggatgtaatccgaaaaaagaatcataagtggttgttaagggagttgaagttac**

**Citrus_aurantium** **agataatggatgtaatccgaaaaaagaatcataagtggttgttaagggagttgaagttac**

**Citrus_cavaleriei** **agataatggatgtaatccgaaaaaagaatcataagtggttgttaagggagttgaagttac**

**Citrus_hongheensis** **agataatggatgtaatccgaaaaaagaatcataagtggttgttaagggagttgaagttac**

**Citrus_limon** **agataatggatgtaatccgaaaaaagaatcataagtggttgttaagggagttgaagttac**

**Citrus_maxima** **agataatggatgtaatccgaaaaaagaatcataagtggttgttaagggagttgaagttac**

**Citrus_medica** **agataatggatgtaatccgaaaaaagaatcataagtggttgttaagggagttgaagttac**

**Citrus_polytrifolia** **agataatggatgtaatccgaaaaaagaatcataagtggttgttaagggagttgaagttac**

**Citrus_reticulata** **agataatggatgtaatccgaaaaaagaatcataagtggttgttaagggagttgaagttac**

**Citrus_sinensis** **agataatggatgtaatccgaaaaaagaatcataagtggttgttaagggagttgaagttac**

**Fortunella_bawangica_1** **agataatggatgtaatccgaaaaaagaatcataagtggttgttaagggagttgaagttac**

**Fortunella_bawangica_2** **agataatggatgtaatccgaaaaaagaatcataagtggttgttaagggagttgaagttac**

**Fortunella_hindsii_1** **agataatggatgtaatccgaaaaaagaatcataagtggttgttaagggagttgaagttac**

**Fortunella_hindsii_2** **agataatggatgtaatccgaaaaaagaatcataagtggttgttaagggagttgaagttac**

**Fortunella_hindsii_3** **agataatggatgtaatccgaaaaaagaatcataagtggttgttaagggagttgaagttac**

**Fortunella_japonica_1** **agataatggatgtaatccgaaaaaagaatcataagtggttgttaagggagttgaagttac**

**Fortunella_japonica_2** **agataatggatgtaatccgaaaaaagaatcataagtggttgttaagggagttgaagttac**

**Fortunella_japonica_3** **agataatggatgtaatccgaaaaaagaatcataagtggttgttaagggagttgaagttac**

**Fortunella_japonica_4** **agataatggatgtaatccgaaaaaagaatcataagtggttgttaagggagttgaagttac**

**Fortunella_japonica_5** **agataatggatgtaatccgaaaaaagaatcataagtggttgttaagggagttgaagttac**

**Fortunella_japonica_6** **agataatggatgtaatccgaaaaaagaatcataagtggttgttaagggagttgaagttac**

**Fortunella_japonica_7** **agataatggatgtaatccgaaaaaagaatcataagtggttgttaagggagttgaagttac**

**Fortunella_japonica_8** **agataatggatgtaatccgaaaaaagaatcataagtggttgttaagggagttgaagttac**

**Fortunella_japonica_9** **agataatggatgtaatccgaaaaaagaatcataagtggttgttaagggagttgaagttac**

**Fortunella_japonica_10** **agataatggatgtaatccgaaaaaagaatcataagtggttgttaagggagttgaagttac**

**Fortunella_japonica_11** **agataatggatgtaatccgaaaaaagaatcataagtggttgttaagggagttgaagttac**

**Fortunella_japonica_12** **agataatggatgtaatccgaaaaaagaatcataagtggttgttaagggagttgaagttac**

**Fortunella_margarita_1** **agataatggatgtaatccgaaaaaagaatcataagtggttgttaagggagttgaagttac**

**Fortunella_margarita_2** **agataatggatgtaatccgaaaaaagaatcataagtggttgttaagggagttgaagttac**

**Fortunella_venosa_1** **agataatggatgtaatccgaaaaaagaatcataagtggttgttaagggagttgaagttac**

**Fortunella_venosa_2** **agataatggatgtaatccgaaaaaagaatcataagtggttgttaagggagttgaagttac**

**Fortunella_venosa_3** **agataatggatgtaatccgaaaaaagaatcataagtggttgttaagggagttgaagttac**

28090	28100	28110	28120	28130	28140

....|....|....|....|....|....|....|....|....|....|....|....|

**Atalantia_kwangtungens** **caaattttgaggagttggcatcaatttatgcctaattgctccacatatagttcctctaat**

**Citrus_reticulata_1** **caaattttgaggagttggcatcaatttatgccgaattgctccacatatagttcctctaat**

**Citrus_reticulata_2** **caaattttgaggagttggcatcaatttatgccgaattgctccacatatagttcctctaat**

**Citrus_trifoliata_3** **caaattttgaggagttggcatcaatttatgcctaattgctccacatatagttcctctaat**

**Citrus_aurantiifolia** **caaattttgaggagttggcatcaatttatgcctaattgctccacatatagttcctctaat**

**Citrus_aurantium** **caaattttgaggagttggcatcaatttatgcctaattgctccacatatagttcctctaat**

**Citrus_cavaleriei** **caaattttgaggagttggcatcaatttatgccgaattgctccacatatagttcctctaat**

**Citrus_hongheensis** **caaattttgaggagttggcatcaatttatgcctaattgctccacatatagttcctctaat**

**Citrus_limon** **caaattttgaggagttggcatcaatttatgcctaattgctccacatatagttcctctaat**

**Citrus_maxima** **caaattttgaggagttggcatcaatttatgcctaattgctccacatatagttcctctaat**

**Citrus_medica** **caaattttgaggagttggcatcaatttatgcctaattgctccacatatagttcctctaat**

**Citrus_polytrifolia** **caaattttgaggagttggcatcaatttatgcctaattgctccacatatagttcctctaat**

**Citrus_reticulata** **caaattttgaggagttggcatcaatttatgccgaattgctccacatatagttcctctaat**

**Citrus_sinensis** **caaattttgaggagttggcatcaatttatgcctaattgctccacatatagttcctctaat**

**Fortunella_bawangica_1** **caaattttgaggagttggcatcaatttatgcctaattgctccacatatagttcctctaat**

**Fortunella_bawangica_2** **caaattttgaggagttggcatcaatttatgcctaattgctccacatatagttcctctaat**

**Fortunella_hindsii_1** **caaattttgaggagttggcatcaatttatgcctaattgctccacatatagttcctctaat**

**Fortunella_hindsii_2** **caaattttgaggagttggcatcaatttatgcctaattgctccacatatagttcctctaat**

**Fortunella_hindsii_3** **caaattttgaggagttggcatcaatttatgcctaattgctccacatatagttcctctaat**

**Fortunella_japonica_1** **caaattttgaggagttggcatcaatttatgcctaattgctccacatatagttcctctaat**

**Fortunella_japonica_2** **caaattttgaggagttggcatcaatttatgcctaattgctccacatatagttcctctaat**

**Fortunella_japonica_3** **caaattttgaggagttggcatcaatttatgcctaattgctccacatatagttcctctaat**

**Fortunella_japonica_4** **caaattttgaggagttggcatcaatttatgcctaattgctccacatatagttcctctaat**

**Fortunella_japonica_5** **caaattttgaggagttggcatcaatttatgcctaattgctccacatatagttcctctaat**

**Fortunella_japonica_6** **caaattttgaggagttggcatcaatttatgcctaattgctccacatatagttcctctaat**

**Fortunella_japonica_7** **caaattttgaggagttggcatcaatttatgcctaattgctccacatatagttcctctaat**

**Fortunella_japonica_8** **caaattttgaggagttggcatcaatttatgcctaattgctccacatatagttcctctaat**

**Fortunella_japonica_9** **caaattttgaggagttggcatcaatttatgcctaattgctccacatatagttcctctaat**

**Fortunella_japonica_10** **caaattttgaggagttggcatcaatttatgcctaattgctccacatatagttcctctaat**

**Fortunella_japonica_11** **caaattttgaggagttggcatcaatttatgcctaattgctccacatatagttcctctaat**

**Fortunella_japonica_12** **caaattttgaggagttggcatcaatttatgcctaattgctccacatatagttcctctaat**

**Fortunella_margarita_1** **caaattttgaggagttggcatcaatttatgcctaattgctccacatatagttcctctaat**

**Fortunella_margarita_2** **caaattttgaggagttggcatcaatttatgcctaattgctccacatatagttcctctaat**

**Fortunella_venosa_1** **caaattttgaggagttggcatcaatttatgcctaattgctccacatatagttcctctaat**

**Fortunella_venosa_2** **caaattttgaggagttggcatcaatttatgcctaattgctccacatatagttcctctaat**

**Fortunella_venosa_3** **caaattttgaggagttggcatcaatttatgcctaattgctccacatatagttcctctaat**

28150	28160	28170	28180	28190	28200

....|....|....|....|....|....|....|....|....|....|....|....|

**Atalantia_kwangtungens** **cacattttctaaccgaatcagggccaatccgaattgatcttgtaaaagattcgcgacaga**

**Citrus_reticulata_1** **cacattttctaaccgaaccagggccaatccgaattgatcttgtaaaagattcgcgacaga**

**Citrus_reticulata_2** **cacattttctaaccgaaccagggccaatccgaattgatcttgtaaaagattcgcgacaga**

**Citrus_trifoliata_3** **cacattttctaaccgaaccagggccaatccgaattgatcttgtaaaagattcgcgacaga**

**Citrus_aurantiifolia** **cacattttctaaccgaaccagggccaatccgaattgatcttgtaaaagattcgcgacaga**

**Citrus_aurantium** **cacattttctaaccgaaccagggccaatccgaattgatcttgtaaaagattcgcgacaga**

**Citrus_cavaleriei** **cacattttctaaccgaaccagggccaatccgaattgatcttgtaaaagattcgcgacaga**

**Citrus_hongheensis** **cacattttctaaccgaaccagggccaatccgaattgatcttgtaaaagattcgcgacaga**

**Citrus_limon** **cacattttctaaccgaaccagggccaatccgaattgatcttgtaaaagattcgcgacaga**

**Citrus_maxima** **cacattttctaaccgaaccagggccaatccgaattgatcttgtaaaagattcgcgacaga**

**Citrus_medica** **cacattttctaaccgaaccagggccaatccgaattgatcttgtaaaagattcgcgacaga**

**Citrus_polytrifolia** **cacattttctaaccgaaccagggccaatccgaattgatcttgtaaaagattcgcgacaga**

**Citrus_reticulata** **cacattttctaaccgaaccagggccaatccgaattgatcttgtaaaagattcgcgacaga**

**Citrus_sinensis** **cacattttctaaccgaaccagggccaatccgaattgatcttgtaaaagattcgcgacaga**

**Fortunella_bawangica_1** **cacattttctaaccgaaccagggccaatccgaattgatcttgtaaaagattcgcgacaga**

**Fortunella_bawangica_2** **cacattttctaaccgaaccagggccaatccgaattgatcttgtaaaagattcgcgacaga**

**Fortunella_hindsii_1** **cacattttctaaccgaaccagggccaatccgaattgatcttgtaaaagattcgcgacaga**

**Fortunella_hindsii_2** **cacattttctaaccgaaccagggccaatccgaattgatcttgtaaaagattcgcgacaga**

**Fortunella_hindsii_3** **cacattttctaaccgaaccagggccaatccgaattgatcttgtaaaagattcgcgacaga**

**Fortunella_japonica_1** **cacattttctaaccgaaccagggccaatccgaattgatcttgtaaaagattcgcgacaga**

**Fortunella_japonica_2** **cacattttctaaccgaaccagggccaatccgaattgatcttgtaaaagattcgcgacaga**

**Fortunella_japonica_3** **cacattttctaaccgaaccagggccaatccgaattgatcttgtaaaagattcgcgacaga**

**Fortunella_japonica_4** **cacattttctaaccgaaccagggccaatccgaattgatcttgtaaaagattcgcgacaga**

**Fortunella_japonica_5** **cacattttctaaccgaaccagggccaatccgaattgatcttgtaaaagattcgcgacaga**

**Fortunella_japonica_6** **cacattttctaaccgaaccagggccaatccgaattgatcttgtaaaagattcgcgacaga**

**Fortunella_japonica_7** **cacattttctaaccgaaccagggccaatccgaattgatcttgtaaaagattcgcgacaga**

**Fortunella_japonica_8** **cacattttctaaccgaaccagggccaatccgaattgatcttgtaaaagattcgcgacaga**

**Fortunella_japonica_9** **cacattttctaaccgaaccagggccaatccgaattgatcttgtaaaagattcgcgacaga**

**Fortunella_japonica_10** **cacattttctaaccgaaccagggccaatccgaattgatcttgtaaaagattcgcgacaga**

**Fortunella_japonica_11** **cacattttctaaccgaaccagggccaatccgaattgatcttgtaaaagattcgcgacaga**

**Fortunella_japonica_12** **cacattttctaaccgaaccagggccaatccgaattgatcttgtaaaagattcgcgacaga**

**Fortunella_margarita_1** **cacattttctaaccgaaccagggccaatccgaattgatcttgtaaaagattcgcgacaga**

**Fortunella_margarita_2** **cacattttctaaccgaaccagggccaatccgaattgatcttgtaaaagattcgcgacaga**

**Fortunella_venosa_1** **cacattttctaaccgaaccagggccaatccgaattgatcttgtaaaagattcgcgacaga**

**Fortunella_venosa_2** **cacattttctaaccgaaccagggccaatccgaattgatcttgtaaaagattcgcgacaga**

**Fortunella_venosa_3** **cacattttctaaccgaaccagggccaatccgaattgatcttgtaaaagattcgcgacaga**

28210	28220	28230	28240	28250	28260

....|....|....|....|....|....|....|....|....|....|....|....|

**Atalantia_kwangtungens** **ccgaatacgtttatttttcaaatgattcatatcgtcaagtgtacccattccaaatttcag**

**Citrus_reticulata_1** **ccgaatacgtttatttttcaaatgattcatatcgtcaagtgtacccattccaaatttcag**

**Citrus_reticulata_2** **ccgaatacgtttatttttcaaatgattcatatcgtcaagtgtacccattccaaatttcag**

**Citrus_trifoliata_3** **ccgaatacgtttatttttcaaatgattcatatcgtcaagtgtacccattccaaatttcag**

**Citrus_aurantiifolia** **ccgaatacgtttatttttcaaatgattcatatcgtcaagtgtacccattccaaatttcag**

**Citrus_aurantium** **ccgaatacgtttatttttcaaatgattcatatcgtcaagtgtacccattccaaatttaag**

**Citrus_cavaleriei** **ccgaatacgtttatttttcaaatgattcatatcgtcaagtgtacccattccaaatttcag**

**Citrus_hongheensis** **ccgaatacgtttatttttcaaatgattcatatcgtcaagtgtacccattccaaatttcag**

**Citrus_limon** **ccgaatacgtttatttttcaaatgattcatatcgtcaagtgtacccattccaaatttaag**

**Citrus_maxima** **ccgaatacgtttatttttcaaatgattcatatcgtcaagtgtacccattccaaatttcag**

**Citrus_medica** **ccgaatacgtttatttttcaaatgattcatatcgtcaagtgtacccattccaaatttcag**

**Citrus_polytrifolia** **ccgaatacgtttatttttcaaatgattcatatcgtcaagtgtacccattccaaatttcag**

**Citrus_reticulata** **ccgaatacgtttatttttcaaatgattcatatcgtcaagtgtacccattccaaatttcag**

**Citrus_sinensis** **ccgaatacgtttatttttcaaatgattcatatcgtcaagtgtacccattccaaatttcag**

**Fortunella_bawangica_1** **tcgaatacgtttatttttcaaatgattcatatcgtcaagtgtacccattccaaatttcag**

**Fortunella_bawangica_2** **tcgaatacgtttatttttcaaatgattcatatcgtcaagtgtacccattccaaatttcag**

**Fortunella_hindsii_1** **tcgaatacgtttatttttcaaatgattcatatcgtcaagtgtacccattccaaatttcag**

**Fortunella_hindsii_2** **tcgaatacgtttatttttcaaatgattcatatcgtcaagtgtacccattccaaatttcag**

**Fortunella_hindsii_3** **tcgaatacgtttatttttcaaatgattcatatcgtcaagtgtacccattccaaatttcag**

**Fortunella_japonica_1** **tcgaatacgtttatttttcaaatgattcatatcgtcaagtgtacccattccaaatttcag**

**Fortunella_japonica_2** **tcgaatacgtttatttttcaaatgattcatatcgtcaagtgtacccattccaaatttcag**

**Fortunella_japonica_3** **tcgaatacgtttatttttcaaatgattcatatcgtcaagtgtacccattccaaatttcag**

**Fortunella_japonica_4** **tcgaatacgtttatttttcaaatgattcatatcgtcaagtgtacccattccaaatttcag**

**Fortunella_japonica_5** **tcgaatacgtttatttttcaaatgattcatatcgtcaagtgtacccattccaaatttcag**

**Fortunella_japonica_6** **tcgaatacgtttatttttcaaatgattcatatcgtcaagtgtacccattccaaatttcag**

**Fortunella_japonica_7** **tcgaatacgtttatttttcaaatgattcatatcgtcaagtgtacccattccaaatttcag**

**Fortunella_japonica_8** **tcgaatacgtttatttttcaaatgattcatatcgtcaagtgtacccattccaaatttcag**

**Fortunella_japonica_9** **tcgaatacgtttatttttcaaatgattcatatcgtcaagtgtacccattccaaatttcag**

**Fortunella_japonica_10** **tcgaatacgtttatttttcaaatgattcatatcgtcaagtgtacccattccaaatttcag**

**Fortunella_japonica_11** **tcgaatacgtttatttttcaaatgattcatatcgtcaagtgtacccattccaaatttcag**

**Fortunella_japonica_12** **tcgaatacgtttatttttcaaatgattcatatcgtcaagtgtacccattccaaatttcag**

**Fortunella_margarita_1** **tcgaatacgtttatttttcaaatgattcatatcgtcaagtgtacccattccaaatttcag**

**Fortunella_margarita_2** **tcgaatacgtttatttttcaaatgattcatatcgtcaagtgtacccattccaaatttcag**

**Fortunella_venosa_1** **tcgaatacgtttatttttcaaatgattcatatcgtcaagtgtacccattccaaatttcag**

**Fortunella_venosa_2** **tcgaatacgtttatttttcaaatgattcatatcgtcaagtgtacccattccaaatttcag**

**Fortunella_venosa_3** **tcgaatacgtttatttttcaaatgattcatatcgtcaagtgtacccattccaaatttcag**

28270	28280	28290	28300	28310	28320

....|....|....|....|....|....|....|....|....|....|....|....|

**Atalantia_kwangtungens** **tccaatcaaatgatcgacagctgccaatacatctcgtggtaacaaaaatgtattgttctg**

**Citrus_reticulata_1** **tccaatcaaatgatcgacagctgccaatacatctcgtggtaacaaaaatgtattgttctg**

**Citrus_reticulata_2** **tccaatcaaatgatcgacagctgccaatacatctcgtggtaacaaaaatgtattgttctg**

**Citrus_trifoliata_3** **tccaatcaaatgatcgacagctgccaatacatctcgtggtaacaaaaatgtattgttctg**

**Citrus_aurantiifolia** **tccaatcaaatgatcgacagctgccaatacatctcgtggtaacaaaaatgtattgttctg**

**Citrus_aurantium** **tccaatcaaatgatcgacagctgccaatacatctcgtggtaacaaaaatgtattgttctg**

**Citrus_cavaleriei** **tccaatcaaatgatcgacagctgccaatacatctcgtggtaacaaaaatgtattgttctg**

**Citrus_hongheensis** **tccaatcaaatgatcgacagctgccaatacatctcgtggtaacaaaaatgtattgttctg**

**Citrus_limon** **tccaatcaaatgatcgacagctgccaatacatctcgtggtaacaaaaatgtattgttctg**

**Citrus_maxima** **tccaatcaaatgatcgacagctgccaatacatctcgtggtaacaaaaatgtattgttctg**

**Citrus_medica** **tccaatcaaatgatcgacagctgccaatacatctcgtggtagcaaaaatgtattgttctg**

**Citrus_polytrifolia** **tccaatcaaatgatcgacagctgccaatacatctcgtggtaacaaaaatgtattgttctg**

**Citrus_reticulata** **tccaatcaaatgatcgacagctgccaatacatctcgtggtaacaaaaatgtattgttctg**

**Citrus_sinensis** **tccaatcaaatgatcgacagctgccaatacatctcgtggtaacaaaaatgtattgttctg**

**Fortunella_bawangica_1** **tccaatcaaatgatcgacagctgccaatacatctcgtggtaacaaaaatgtattgttctg**

**Fortunella_bawangica_2** **tccaatcaaatgatcgacagctgccaatacatctcgtggtaacaaaaatgtattgttctg**

**Fortunella_hindsii_1** **tccaatcaaatgatcgacagctgccaatacatctcgtggtaacaaaaatgtattgttctg**

**Fortunella_hindsii_2** **tccaatcaaatgatcgacagctgccaatacatctcgtggtaacaaaaatgtattgttctg**

**Fortunella_hindsii_3** **tccaatcaaatgatcgacagctgccaatacatctcgtggtaacaaaaatgtattgttctg**

**Fortunella_japonica_1** **tccaatcaaatgatcgacagctgccaatacatctcgtggtaacaaaaatgtattgttctg**

**Fortunella_japonica_2** **tccaatcaaatgatcgacagctgccaatacatctcgtggtaacaaaaatgtattgttctg**

**Fortunella_japonica_3** **tccaatcaaatgatcgacagctgccaatacatctcgtggtaacaaaaatgtattgttctg**

**Fortunella_japonica_4** **tccaatcaaatgatcgacagctgccaatacatctcgtggtaacaaaaatgtattgttctg**

**Fortunella_japonica_5** **tccaatcaaatgatcgacagctgccaatacatctcgtggtaacaaaaatgtattgttctg**

**Fortunella_japonica_6** **tccaatcaaatgatcgacagctgccaatacatctcgtggtaacaaaaatgtattgttctg**

**Fortunella_japonica_7** **tccaatcaaatgatcgacagctgccaatacatctcgtggtaacaaaaatgtattgttctg**

**Fortunella_japonica_8** **tccaatcaaatgatcgacagctgccaatacatctcgtggtaacaaaaatgtattgttctg**

**Fortunella_japonica_9** **tccaatcaaatgatcgacagctgccaatacatctcgtggtaacaaaaatgtattgttctg**

**Fortunella_japonica_10** **tccaatcaaatgatcgacagctgccaatacatctcgtggtaacaaaaatgtattgttctg**

**Fortunella_japonica_11** **tccaatcaaatgatcgacagctgccaatacatctcgtggtaacaaaaatgtattgttctg**

**Fortunella_japonica_12** **tccaatcaaatgatcgacagctgccaatacatctcgtggtaacaaaaatgtattgttctg**

**Fortunella_margarita_1** **tccaatcaaatgatcgacagctgccaatacatctcgtggtaacaaaaatgtattgttctg**

**Fortunella_margarita_2** **tccaatcaaatgatcgacagctgccaatacatctcgtggtaacaaaaatgtattgttctg**

**Fortunella_venosa_1** **tccaatcaaatgatcgacagctgccaatacatctcgtggtaacaaaaatgtattgttctg**

**Fortunella_venosa_2** **tccaatcaaatgatcgacagctgccaatacatctcgtggtaacaaaaatgtattgttctg**

**Fortunella_venosa_3** **tccaatcaaatgatcgacagctgccaatacatctcgtggtaacaaaaatgtattgttctg**

28330	28340	28350	28360	28370	28380

....|....|....|....|....|....|....|....|....|....|....|....|

**Atalantia_kwangtungens** **aggtatattaagattcagtctctggttcatatttcgtctaccaatctttcctaattcaca**

**Citrus_reticulata_1** **aggtatattcagattcagtctctggttcatatttcgtctaccaatctttcctaattcaca**

**Citrus_reticulata_2** **aggtatattcagattcagtctctggttcatatttcgtctaccaatctttcctaattcaca**

**Citrus_trifoliata_3** **aggtatattcagattcagtctctggttcatatttcgtctaccaatctttcctaattcaca**

**Citrus_aurantiifolia** **aggtatattcagattcagtctctggttcatatttcgtctaccaatctttcctaattcaca**

**Citrus_aurantium** **aggtatattcagattcagtctctggttcatatttcgtctaccaatctttcctaattcaca**

**Citrus_cavaleriei** **aggtatattcagattcagtctctggttcatatttcgtctaccaatctttcctaattcaca**

**Citrus_hongheensis** **aggtatattcagattcagtctctggttcatatttcgtctaccaatctttcctaattcaca**

**Citrus_limon** **aggtatattcagattcagtctctggttcatatttcgtctaccaatctttcctaattcaca**

**Citrus_maxima** **aggtatattcagattcagtctctggttcatatttcgtctaccaatctttcctaattcaca**

**Citrus_medica** **aggtatattcagattcagtctctggttcatatttcgtctaccaatctttcctaattcaca**

**Citrus_polytrifolia** **aggtatattcagattcagtctctggttcatatttcgtctaccaatctttcctaattcaca**

**Citrus_reticulata** **aggtatattcagattcagtctctggttcatatttcgtctaccaatctttcctaattcaca**

**Citrus_sinensis** **aggtatattcagattcagtctctggttcatatttcgtctaccaatctttcctaattcaca**

**Fortunella_bawangica_1** **aggtatattcagattcagtctctggttcatatttcgtctaccaatctttcctaattcaca**

**Fortunella_bawangica_2** **aggtatattcagattcagtctctggttcatatttcgtctaccaatctttcctaattcaca**

**Fortunella_hindsii_1** **aggtatattcagattcagtctctggttcatatttcgtctaccaatctttcctaattcaca**

**Fortunella_hindsii_2** **aggtatattcagattcagtctctggttcatatttcgtctaccaatctttcctaattcaca**

**Fortunella_hindsii_3** **aggtatattcagattcagtctctggttcatatttcgtctaccaatctttcctaattcaca**

**Fortunella_japonica_1** **aggtatattcagattcagtctctggttcatatttcgtctaccaatctttcctaattcaca**

**Fortunella_japonica_2** **aggtatattcagattcagtctctggttcatatttcgtctaccaatctttcctaattcaca**

**Fortunella_japonica_3** **aggtatattcagattcagtctctggttcatatttcgtctaccaatctttcctaattcaca**

**Fortunella_japonica_4** **aggtatattcagattcagtctctggttcatatttcgtctaccaatctttcctaattcaca**

**Fortunella_japonica_5** **aggtatattcagattcagtctctggttcatatttcgtctaccaatctttcctaattcaca**

**Fortunella_japonica_6** **aggtatattcagattcagtctctggttcatatttcgtctaccaatctttcctaattcaca**

**Fortunella_japonica_7** **aggtatattcagattcagtctctggttcatatttcgtctaccaatctttcctaattcaca**

**Fortunella_japonica_8** **aggtatattcagattcagtctctggttcatatttcgtctaccaatctttcctaattcaca**

**Fortunella_japonica_9** **aggtatattcagattcagtctctggttcatatttcgtctaccaatctttcctaattcaca**

**Fortunella_japonica_10** **aggtatattcagattcagtctctggttcatatttcgtctaccaatctttcctaattcaca**

**Fortunella_japonica_11** **aggtatattcagattcagtctctggttcatatttcgtctaccaatctttcctaattcaca**

**Fortunella_japonica_12** **aggtatattcagattcagtctctggttcatatttcgtctaccaatctttcctaattcaca**

**Fortunella_margarita_1** **aggtatattcagattcagtctctggttcatatttcgtctaccaatctttcctaattcaca**

**Fortunella_margarita_2** **aggtatattcagattcagtctctggttcatatttcgtctaccaatctttcctaattcaca**

**Fortunella_venosa_1** **aggtatattcagattcagtctctggttcatatttcgtctaccaatctttcctaattcaca**

**Fortunella_venosa_2** **aggtatattcagattcagtctctggttcatatttcgtctaccaatctttcctaattcaca**

**Fortunella_venosa_3** **aggtatattcagattcagtctctggttcatatttcgtctaccaatctttcctaattcaca**

28390	28400	28410	28420	28430	28440

....|....|....|....|....|....|....|....|....|....|....|....|

**Atalantia_kwangtungens** **tctttggtgaaaaaatttcttttgtaattccttacataaggattccgaaaataccggatc**

**Citrus_reticulata_1** **tctttggtgaaaaaatttcttttgtaattccttacacaaggattccgaaaataccggatc**

**Citrus_reticulata_2** **tctttggtgaaaaaatttcttttgtaattccttacacaaggattccgaaaataccggatc**

**Citrus_trifoliata_3** **tctttggtgaaaaaatttcttttgtaattccttacataaggattccgaaaataccggatc**

**Citrus_aurantiifolia** **tctttggtgaaaaaatttcttttgtaattccttacataaggattccgaaaataccggatc**

**Citrus_aurantium** **tctttggtgaaaaaatttcttttgtaattccttacataaggattccgaaaataccggatc**

**Citrus_cavaleriei** **tctttggtgaaaaaatttcttttgtaattccttacataaggattccgaaaataccggatc**

**Citrus_hongheensis** **tctttggtgaaaaaatttcttttgtaattccttacataaggattccgaaaataccggatc**

**Citrus_limon** **tctttggtgaaaaaatttcttttgtaattccttacataaggattccgaaaataccggatc**

**Citrus_maxima** **tctttggtgaaaaaatttcttttgtaattccttacataaggattccgaaaataccggatc**

**Citrus_medica** **tctttggtgaaaaaatttcttttgtaattccttacataaggattccgaaaataccggatc**

**Citrus_polytrifolia** **tctttggtgaaaaaatttcttttgtaattccttacataaggattccgaaaataccggatc**

**Citrus_reticulata** **tctttggtgaaaaaatttcttttgtaattccttacacaaggattccgaaaataccggatc**

**Citrus_sinensis** **tctttggtgaaaaaatttcttttgtaattccttacataaggattccgaaaataccggatc**

**Fortunella_bawangica_1** **tctttggtgaaaaaatttcttttgtaattccttacataaggattccgaaaataccggatc**

**Fortunella_bawangica_2** **tctttggtgaaaaaatttcttttgtaattccttacataaggattccgaaaataccggatc**

**Fortunella_hindsii_1** **tctttggtgaaaaaatttcttttgtaattccttacataaggattccgaaaataccggatc**

**Fortunella_hindsii_2** **tctttggtgaaaaaatttcttttgtaattccttacataaggattccgaaaataccggatc**

**Fortunella_hindsii_3** **tctttggtgaaaaaatttcttttgtaattccttacataaggattccgaaaataccggatc**

**Fortunella_japonica_1** **tctttggtgaaaaaatttcttttgtaattccttacataaggattccgaaaataccggatc**

**Fortunella_japonica_2** **tctttggtgaaaaaatttcttttgtaattccttacataaggattccgaaaataccggatc**

**Fortunella_japonica_3** **tctttggtgaaaaaatttcttttgtaattccttacataaggattccgaaaataccggatc**

**Fortunella_japonica_4** **tctttggtgaaaaaatttcttttgtaattccttacataaggattccgaaaataccggatc**

**Fortunella_japonica_5** **tctttggtgaaaaaatttcttttgtaattccttacataaggattccgaaaataccggatc**

**Fortunella_japonica_6** **tctttggtgaaaaaatttcttttgtaattccttacataaggattccgaaaataccggatc**

**Fortunella_japonica_7** **tctttggtgaaaaaatttcttttgtaattccttacataaggattccgaaaataccggatc**

**Fortunella_japonica_8** **tctttggtgaaaaaatttcttttgtaattccttacataaggattccgaaaataccggatc**

**Fortunella_japonica_9** **tctttggtgaaaaaatttcttttgtaattccttacataaggattccgaaaataccggatc**

**Fortunella_japonica_10** **tctttggtgaaaaaatttcttttgtaattccttacataaggattccgaaaataccggatc**

**Fortunella_japonica_11** **tctttggtgaaaaaatttcttttgtaattccttacataaggattccgaaaataccggatc**

**Fortunella_japonica_12** **tctttggtgaaaaaatttcttttgtaattccttacataaggattccgaaaataccggatc**

**Fortunella_margarita_1** **tctttggtgaaaaaatttcttttgtaattccttacataaggattccgaaaataccggatc**

**Fortunella_margarita_2** **tctttggtgaaaaaatttcttttgtaattccttacataaggattccgaaaataccggatc**

**Fortunella_venosa_1** **tctttggtgaaaaaatttcttttgtaattccttacataaggattccgaaaataccggatc**

**Fortunella_venosa_2** **tctttggtgaaaaaatttcttttgtaattccttacataaggattccgaaaataccggatc**

**Fortunella_venosa_3** **tctttggtgaaaaaatttcttttgtaattccttacataaggattccgaaaataccggatc**

28450	28460	28470	28480	28490	28500

....|....|....|....|....|....|....|....|....|....|....|....|

**Atalantia_kwangtungens** **tccgcctacacaagcaaattgttgataaaactccaaaatggcattttcttttgatccaat**

**Citrus_reticulata_1** **tccgcctacacaagcaaattgttgataaaactccaaaatggcattttcttttgatccaat**

**Citrus_reticulata_2** **tccgcctacacaagcaaattgttgataaaactccaaaatggcattttcttttgatccaat**

**Citrus_trifoliata_3** **tccgcctacacaagcaaattgttgataaaactccaaaatggcattttcttttgatccaat**

**Citrus_aurantiifolia** **tccgcctacacaagcaaattgttgataaaactccaaaatggcattttcttttgatccaat**

**Citrus_aurantium** **tccgcctacacaagcaaattgttgataaaactccaaaatggcattttcttttgatccaat**

**Citrus_cavaleriei** **tccgcctacacaagcaaattgttgataaaactccaaaatggcattttcttttgatccaat**

**Citrus_hongheensis** **tccgcctacacaagcaaattgttgataaaactccaaaatggcattttcttttgatccaat**

**Citrus_limon** **tccgcctacacaagcaaattgttgataaaactccaaaatggcattttcttttgatccaat**

**Citrus_maxima** **tccgcctacacaagcaaattgttgataaaactccaaaatggcattttcttttgatccaat**

**Citrus_medica** **tccgcctacacaagcaaattgttgataaaactccaaaatggcattttcttttgatccaat**

**Citrus_polytrifolia** **tccgcctacacaagcaaattgttgataaaactccaaaatggcattttcttttgatccaat**

**Citrus_reticulata** **tccgcctacacaagcaaattgttgataaaactccaaaatggcattttcttttgatccaat**

**Citrus_sinensis** **tccgcctacacaagcaaattgttgataaaactccaaaatggcattttcttttgatccaat**

**Fortunella_bawangica_1** **tccgcctacacaagcaaattgttgataaaactccaaaatggcattttcttttgatccaat**

**Fortunella_bawangica_2** **tccgcctacacaagcaaattgttgataaaactccaaaatggcattttcttttgatccaat**

**Fortunella_hindsii_1** **tccgcctacacaagcaaattgttgataaaactccaaaatggcattttcttttgatccaat**

**Fortunella_hindsii_2** **tccgcctacacaagcaaattgttgataaaactccaaaatggcattttcttttgatccaat**

**Fortunella_hindsii_3** **tccgcctacacaagcaaattgttgataaaactccaaaatggcattttcttttgatccaat**

**Fortunella_japonica_1** **tccgcctacacaagcaaattgttgataaaactccaaaatggcattttcttttgatccaat**

**Fortunella_japonica_2** **tccgcctacacaagcaaattgttgataaaactccaaaatggcattttcttttgatccaat**

**Fortunella_japonica_3** **tccgcctacacaagcaaattgttgataaaactccaaaatggcattttcttttgatccaat**

**Fortunella_japonica_4** **tccgcctacacaagcaaattgttgataaaactccaaaatggcattttcttttgatccaat**

**Fortunella_japonica_5** **tccgcctacacaagcaaattgttgataaaactccaaaatggcattttcttttgatccaat**

**Fortunella_japonica_6** **tccgcctacacaagcaaattgttgataaaactccaaaatggcattttcttttgatccaat**

**Fortunella_japonica_7** **tccgcctacacaagcaaattgttgataaaactccaaaatggcattttcttttgatccaat**

**Fortunella_japonica_8** **tccgcctacacaagcaaattgttgataaaactccaaaatggcattttcttttgatccaat**

**Fortunella_japonica_9** **tccgcctacacaagcaaattgttgataaaactccaaaatggcattttcttttgatccaat**

**Fortunella_japonica_10** **tccgcctacacaagcaaattgttgataaaactccaaaatggcattttcttttgatccaat**

**Fortunella_japonica_11** **tccgcctacacaagcaaattgttgataaaactccaaaatggcattttcttttgatccaat**

**Fortunella_japonica_12** **tccgcctacacaagcaaattgttgataaaactccaaaatggcattttcttttgatccaat**

**Fortunella_margarita_1** **tccgcctacacaagcaaattgttgataaaactccaaaatggcattttcttttgatccaat**

**Fortunella_margarita_2** **tccgcctacacaagcaaattgttgataaaactccaaaatggcattttcttttgatccaat**

**Fortunella_venosa_1** **tccgcctacacaagcaaattgttgataaaactccaaaatggcattttcttttgatccaat**

**Fortunella_venosa_2** **tccgcctacacaagcaaattgttgataaaactccaaaatggcattttcttttgatccaat**

**Fortunella_venosa_3** **tccgcctacacaagcaaattgttgataaaactccaaaatggcattttcttttgatccaat**

28510	28520	28530	28540	28550	28560

....|....|....|....|....|....|....|....|....|....|....|....|

**Atalantia_kwangtungens** **tttttttttctccttattcgtcaggaaagacaagaaaatttcagggtagcaaatattctc**

**Citrus_reticulata_1** **tttttttttctccttattcgtcaggaaagacaagaaaatttcagggtagcaaatattctc**

**Citrus_reticulata_2** **tttttttttctccttattcgtcaggaaagacaagaaaatttcagggtagcaaatattctc**

**Citrus_trifoliata_3** **tttttttttctccttattcgtcaggaaagacaagaaaatttcagggtagcaaatattctc**

**Citrus_aurantiifolia** **tttttttttctccttattcgtcaggaaagacaagaaaatttcagggtagcaaatattctc**

**Citrus_aurantium** **tttttttttctccttattcgtcaggaaagacaagaaaatttcagggtagcaaatattctc**

**Citrus_cavaleriei** **tttttttttctccttattcgtcaggaaagacaagaaaatttcagggtagcaaatattctc**

**Citrus_hongheensis** **tttttttttctccttattcgtcaggaaagacaagaaaatttcagggtagcaaatattctc**

**Citrus_limon** **tttttttttctccttattcgtcaggaaagacaagaaaatttcagggtagcaaatattctc**

**Citrus_maxima** **tttttttttctccttattcgtcaggaaagacaagaaaatttcagggtagcaaatattctc**

**Citrus_medica** **tttttttttatccttattcgtcaggaaagacaagaaaatttcagggtagcaaatattttc**

**Citrus_polytrifolia** **tttttttttctccttattcgtcaggaaagacaagaaaatttcagggtagcaaatattctc**

**Citrus_reticulata** **tttttttttctccttattcgtcaggaaagacaagaaaatttcagggtagcaaatattctc**

**Citrus_sinensis** **tttttttttctccttattcgtcaggaaagacaagaaaatttcagggtagcaaatattctc**

**Fortunella_bawangica_1** **tttttttttctccttattcgtcaggaaagacaagaaaatttcagggtagcaaatattctc**

**Fortunella_bawangica_2** **tttttttttctccttattcgtcaggaaagacaagaaaatttcagggtagcaaatattctc**

**Fortunella_hindsii_1** **tttttttttctccttattcgtcaggaaagacaagaaaatttcagggtagcaaatattctc**

**Fortunella_hindsii_2** **tttttttttctccttattcgtcaggaaagacaagaaaatttcagggtagcaaatattctc**

**Fortunella_hindsii_3** **tttttttttctccttattcgtcaggaaagacaagaaaatttcagggtagcaaatattctc**

**Fortunella_japonica_1** **tttttttttctccttattcgtcaggaaagacaagaaaatttcagggtagcaaatattctc**

**Fortunella_japonica_2** **tttttttttctccttattcgtcaggaaagacaagaaaatttcagggtagcaaatattctc**

**Fortunella_japonica_3** **tttttttttctccttattcgtcaggaaagacaagaaaatttcagggtagcaaatattctc**

**Fortunella_japonica_4** **tttttttttctccttattcgtcaggaaagacaagaaaatttcagggtagcaaatattctc**

**Fortunella_japonica_5** **tttttttttctccttattcgtcaggaaagacaagaaaatttcagggtagcaaatattctc**

**Fortunella_japonica_6** **tttttttttctccttattcgtcaggaaagacaagaaaatttcagggtagcaaatattctc**

**Fortunella_japonica_7** **tttttttttctccttattcgtcaggaaagacaagaaaatttcagggtagcaaatattctc**

**Fortunella_japonica_8** **tttttttttctccttattcgtcaggaaagacaagaaaatttcagggtagcaaatattctc**

**Fortunella_japonica_9** **tttttttttctccttattcgtcaggaaagacaagaaaatttcagggtagcaaatattctc**

**Fortunella_japonica_10** **tttttttttctccttattcgtcaggaaagacaagaaaatttcagggtagcaaatattctc**

**Fortunella_japonica_11** **tttttttttctccttattcgtcaggaaagacaagaaaatttcagggtagcaaatattctc**

**Fortunella_japonica_12** **tttttttttctccttattcgtcaggaaagacaagaaaatttcagggtagcaaatattctc**

**Fortunella_margarita_1** **tttttttttctccttattcgtcaggaaagacaagaaaatttcagggtagcaaatattctc**

**Fortunella_margarita_2** **tttttttttctccttattcgtcaggaaagacaagaaaatttcagggtagcaaatattctc**

**Fortunella_venosa_1** **tttttttttctccttattcgtcaggaaagacaagaaaatttcagggtagcaaatattctc**

**Fortunella_venosa_2** **tttttttttctccttattcgtcaggaaagacaagaaaatttcagggtagcaaatattctc**

**Fortunella_venosa_3** **tttttttttctccttattcgtcaggaaagacaagaaaatttcagggtagcaaatattctc**

28570	28580	28590	28600	28610	28620

....|....|....|....|....|....|....|....|....|....|....|....|

**Atalantia_kwangtungens** **tagaatttctcgtagattcgaacccatagctgctgatagaactagaatagatactttctg**

**Citrus_reticulata_1** **tagaatttctcgtagattcgaacccatagctgctgatagaactagaatagatactttctg**

**Citrus_reticulata_2** **tagaatttctcgtagattcgaacccatagctgctgatagaactagaatagatactttctg**

**Citrus_trifoliata_3** **tagaatttctcgtagattcgaacccatagctgctgatagaactagaatagatactttctg**

**Citrus_aurantiifolia** **tagaatttctcgtagattcgaacccatagctgctgatagaactagaatagatactttctg**

**Citrus_aurantium** **tagaatttctcgtagattcgaacccatagctgctgatagaactagaatagatactttctg**

**Citrus_cavaleriei** **tagaatttctcgtagattcgaacccatagctgctgatagaactagaatagatactttctg**

**Citrus_hongheensis** **tagaatttctcgtagattcgaacccatagctgctgatagaactagaatagatactttctg**

**Citrus_limon** **tagaatttctcgtagattcgaacccatagctgctgatagaactagaatagatactttctg**

**Citrus_maxima** **tagaatttctcgtagattcgaacccatagctgctgatagaactagaatagatactttctg**

**Citrus_medica** **tagaatttctcgtagattcgaacccatagctgctgatagaactagaatagatactttctg**

**Citrus_polytrifolia** **tagaatttctcgtagattcgaacccatagctgctgatagaactagaatagatactttctg**

**Citrus_reticulata** **tagaatttctcgtagattcgaacccatagctgctgatagaactagaatagatactttctg**

**Citrus_sinensis** **tagaatttctcgtagattcgaacccatagctgctgatagaactagaatagatactttctg**

**Fortunella_bawangica_1** **tagaatttctcgtagattcgaacccatagctgctgatagaactagaatagatactttctg**

**Fortunella_bawangica_2** **tagaatttctcgtagattcgaacccatagctgctgatagaactagaatagatactttctg**

**Fortunella_hindsii_1** **tagaatttctcgtagattcgaacccatagctgctgatagaactagaatagatactttctg**

**Fortunella_hindsii_2** **tagaatttctcgtagattcgaacccatagctgctgatagaactagaatagatactttctg**

**Fortunella_hindsii_3** **tagaatttctcgtagattcgaacccatagctgctgatagaactagaatagatactttctg**

**Fortunella_japonica_1** **tagaatttctcgtagattcgaacccatagctgctgatagaactagaatagatactttctg**

**Fortunella_japonica_2** **tagaatttctcgtagattcgaacccatagctgctgatagaactagaatagatactttctg**

**Fortunella_japonica_3** **tagaatttctcgtagattcgaacccatagctgctgatagaactagaatagatactttctg**

**Fortunella_japonica_4** **tagaatttctcgtagattcgaacccatagctgctgatagaactagaatagatactttctg**

**Fortunella_japonica_5** **tagaatttctcgtagattcgaacccatagctgctgatagaactagaatagatactttctg**

**Fortunella_japonica_6** **tagaatttctcgtagattcgaacccatagctgctgatagaactagaatagatactttctg**

**Fortunella_japonica_7** **tagaatttctcgtagattcgaacccatagctgctgatagaactagaatagatactttctg**

**Fortunella_japonica_8** **tagaatttctcgtagattcgaacccatagctgctgatagaactagaatagatactttctg**

**Fortunella_japonica_9** **tagaatttctcgtagattcgaacccatagctgctgatagaactagaatagatactttctg**

**Fortunella_japonica_10** **tagaatttctcgtagattcgaacccatagctgctgatagaactagaatagatactttctg**

**Fortunella_japonica_11** **tagaatttctcgtagattcgaacccatagctgctgatagaactagaatagatactttctg**

**Fortunella_japonica_12** **tagaatttctcgtagattcgaacccatagctgctgatagaactagaatagatactttctg**

**Fortunella_margarita_1** **tagaatttctcgtagattcgaacccatagctgctgatagaactagaatagatactttctg**

**Fortunella_margarita_2** **tagaatttctcgtagattcgaacccatagctgctgatagaactagaatagatactttctg**

**Fortunella_venosa_1** **tagaatttctcgtagattcgaacccatagctgctgatagaactagaatagatactttctg**

**Fortunella_venosa_2** **tagaatttctcgtagattcgaacccatagctgctgatagaactagaatagatactttctg**

**Fortunella_venosa_3** **tagaatttctcgtagattcgaacccatagctgctgatagaactagaatagatactttctg**

28630	28640	28650	28660	28670	28680

....|....|....|....|....|....|....|....|....|....|....|....|

**Atalantia_kwangtungens** **tttcctactcacacgagcccatatccttgcttttctatcaatctctaattctaatcttcc**

**Citrus_reticulata_1** **tttcctactcacacgagcccatatccttgcttttctatcaatctctaattcgaatcttcc**

**Citrus_reticulata_2** **tttcctactcacacgagcccatatccttgcttttctatcaatctctaattcgaatcttcc**

**Citrus_trifoliata_3** **tttcctactcacacgagcccatatccttgcttttctatcaatctctaattctaatcttcc**

**Citrus_aurantiifolia** **tttcctactcacacgagcccatatccttgcttttctatcaatctctaattcgaatcttcc**

**Citrus_aurantium** **tttcctactcacacgagcccatatccttgcttttctatcaatctctaattcgaatcttcc**

**Citrus_cavaleriei** **tttcctactcacacgagcccatatccttgcttttctatcaatctctaattcgaatcttcc**

**Citrus_hongheensis** **tttcctactcacacgagcccatatccttgcttttctatcaatctctaattcgaatcttcc**

**Citrus_limon** **tttcctactcacacgagcccatatccttgcttttctatcaatctctaattcgaatcttcc**

**Citrus_maxima** **tttcctactcacacgagcccatatccttgcttttctatcaatctctaattcgaatcttcc**

**Citrus_medica** **tttcctactcacacgagcccatatccttgcttttctatcaatctctaattctaatcttcc**

**Citrus_polytrifolia** **tttcctactcacacgagcccatatccttgcttttctatcaatctctaattctaatcttcc**

**Citrus_reticulata** **tttcctactcacacgagcccatatccttgcttttctatcaatctctaattcgaatcttcc**

**Citrus_sinensis** **tttcctactcacacgagcccatatccttgcttttctatcaatctctaattcgaatcttcc**

**Fortunella_bawangica_1** **tttcctactcacacgagcccatatccttgcttttctatcaatctctaattcgaatcttcc**

**Fortunella_bawangica_2** **tttcctactcacacgagcccatatccttgcttttctatcaatctctaattcgaatcttcc**

**Fortunella_hindsii_1** **tttcctactcacacgagcccatatccttgcttttctatcaatctctaattcgaatcttcc**

**Fortunella_hindsii_2** **tttcctactcacacgagcccatatccttgcttttctatcaatctctaattcgaatcttcc**

**Fortunella_hindsii_3** **tttcctactcacacgagcccatatccttgcttttctatcaatctctaattcgaatcttcc**

**Fortunella_japonica_1** **tttcctactcacacgagcccatatccttgcttttctatcaatctctaattcgaatcttcc**

**Fortunella_japonica_2** **tttcctactcacacgagcccatatccttgcttttctatcaatctctaattcgaatcttcc**

**Fortunella_japonica_3** **tttcctactcacacgagcccatatccttgcttttctatcaatctctaattcgaatcttcc**

**Fortunella_japonica_4** **tttcctactcacacgagcccatatccttgcttttctatcaatctctaattcgaatcttcc**

**Fortunella_japonica_5** **tttcctactcacacgagcccatatccttgcttttctatcaatctctaattcgaatcttcc**

**Fortunella_japonica_6** **tttcctactcacacgagcccatatccttgcttttctatcaatctctaattcgaatcttcc**

**Fortunella_japonica_7** **tttcctactcacacgagcccatatccttgcttttctatcaatctctaattcgaatcttcc**

**Fortunella_japonica_8** **tttcctactcacacgagcccatatccttgcttttctatcaatctctaattcgaatcttcc**

**Fortunella_japonica_9** **tttcctactcacacgagcccatatccttgcttttctatcaatctctaattcgaatcttcc**

**Fortunella_japonica_10** **tttcctactcacacgagcccatatccttgcttttctatcaatctctaattcgaatcttcc**

**Fortunella_japonica_11** **tttcctactcacacgagcccatatccttgcttttctatcaatctctaattcgaatcttcc**

**Fortunella_japonica_12** **tttcctactcacacgagcccatatccttgcttttctatcaatctctaattcgaatcttcc**

**Fortunella_margarita_1** **tttcctactcacacgagcccatatccttgcttttctatcaatctctaattcgaatcttcc**

**Fortunella_margarita_2** **tttcctactcacacgagcccatatccttgcttttctatcaatctctaattcgaatcttcc**

**Fortunella_venosa_1** **tttcctactcacacgagcccatatccttgcttttctatcaatctctaattcgaatcttcc**

**Fortunella_venosa_2** **tttcctactcacacgagcccatatccttgcttttctatcaatctctaattcgaatcttcc**

**Fortunella_venosa_3** **tttcctactcacacgagcccatatccttgcttttctatcaatctctaattcgaatcttcc**

28690	28700	28710	28720	28730	28740

....|....|....|....|....|....|....|....|....|....|....|....|

**Atalantia_kwangtungens** **cccccaatcagatattatggtgcctgtatagaccgaaattccgttatggcccaattctga**

**Citrus_reticulata_1** **cccccaatcagatattatggtgcctgtatagaccgaaattccgttatggcccaattctga**

**Citrus_reticulata_2** **cccccaatcagatattatggtgcctgtatagaccgaaattccgttatggcccaattctga**

**Citrus_trifoliata_3** **cccccaatcagatattatggtgcctgtatagaccgaaattccgttatggcccaattctga**

**Citrus_aurantiifolia** **cccccaatcagatattatggtgcctgtatagaccgaaattccgttatggcccaattctga**

**Citrus_aurantium** **cccccaatcagatattatggtgcctgtatagaccgaaattccgttatggcccaattctga**

**Citrus_cavaleriei** **cccccaatcagatattatggtgcctgtatagaccgaaattccgttatggcccaattctga**

**Citrus_hongheensis** **cccccaatcagatattatggtgcctgtatagaccgaaattccgttatggcccaattctga**

**Citrus_limon** **cccccaatcagatattatggtgcctgtatagaccgaaattccgttatggcccaattctga**

**Citrus_maxima** **cccccaatcagatattatggtgcctgtatagaccgaaattccgttatggcccaattctga**

**Citrus_medica** **cccccaatcagatattatggtgcctgtatagaccgaaattccgttatggcccaattctga**

**Citrus_polytrifolia** **cccccaatcagatattatggtgcctgtatagaccgaaattccgttatggcccaattctga**

**Citrus_reticulata** **cccccaatcagatattatggtgcctgtatagaccgaaattccgttatggcccaattctga**

**Citrus_sinensis** **cccccaatcagatattatggtgcctgtatagaccgaaattccgttatggcccaattctga**

**Fortunella_bawangica_1** **cccccaatcagatattatggtgcctgtatagaccgaaattccgttatggcccaattctga**

**Fortunella_bawangica_2** **cccccaatcagatattatggtgcctgtatagaccgaaattccgttatggcccaattctga**

**Fortunella_hindsii_1** **cccccaatcagatattatggtgcctgtatagaccgaaattccgttatggcccaattctga**

**Fortunella_hindsii_2** **cccccaatcagatattatggtgcctgtatagaccgaaattccgttatggcccaattctga**

**Fortunella_hindsii_3** **cccccaatcagatattatggtgcctgtatagaccgaaattccgttatggcccaattctga**

**Fortunella_japonica_1** **cccccaatcagatattatggtgcctgtatagaccgaaattccgttatggcccaattctga**

**Fortunella_japonica_2** **cccccaatcagatattatggtgcctgtatagaccgaaattccgttatggcccaattctga**

**Fortunella_japonica_3** **cccccaatcagatattatggtgcctgtatagaccgaaattccgttatggcccaattctga**

**Fortunella_japonica_4** **cccccaatcagatattatggtgcctgtatagaccgaaattccgttatggcccaattctga**

**Fortunella_japonica_5** **cccccaatcagatattatggtgcctgtatagaccgaaattccgttatggcccaattctga**

**Fortunella_japonica_6** **cccccaatcagatattatggtgcctgtatagaccgaaattccgttatggcccaattctga**

**Fortunella_japonica_7** **cccccaatcagatattatggtgcctgtatagaccgaaattccgttatggcccaattctga**

**Fortunella_japonica_8** **cccccaatcagatattatggtgcctgtatagaccgaaattccgttatggcccaattctga**

**Fortunella_japonica_9** **cccccaatcagatattatggtgcctgtatagaccgaaattccgttatggcccaattctga**

**Fortunella_japonica_10** **cccccaatcagatattatggtgcctgtatagaccgaaattccgttatggcccaattctga**

**Fortunella_japonica_11** **cccccaatcagatattatggtgcctgtatagaccgaaattccgttatggcccaattctga**

**Fortunella_japonica_12** **cccccaatcagatattatggtgcctgtatagaccgaaattccgttatggcccaattctga**

**Fortunella_margarita_1** **cccccaatcagatattatggtgcctgtatagaccgaaattccgttatggcccaattctga**

**Fortunella_margarita_2** **cccccaatcagatattatggtgcctgtatagaccgaaattccgttatggcccaattctga**

**Fortunella_venosa_1** **cccccaatcagatattatggtgcctgtatagaccgaaattccgttatggcccaattctga**

**Fortunella_venosa_2** **cccccaatcagatattatggtgcctgtatagaccgaaattccgttatggcccaattctga**

**Fortunella_venosa_3** **cccccaatcagatattatggtgcctgtatagaccgaaattccgttatggcccaattctga**

28750	28760	28770	28780	28790	28800

....|....|....|....|....|....|....|....|....|....|....|....|

**Atalantia_kwangtungens** **ccgataatagataccgggactttgcaatatttgatttatgacaattctgtatattccgtt**

**Citrus_reticulata_1** **ccgataatagataccgggactttgcaatatttgattgatgacaattctgtatattccgtt**

**Citrus_reticulata_2** **ccgataatagataccgggactttgcaatatttgattgatgacaattctgtatattccgtt**

**Citrus_trifoliata_3** **ccgataatagataccgggactttgcaatatttgattgatgacaattctgtatattccgtt**

**Citrus_aurantiifolia** **ccgataatagataccgggactttgcaatatttgattgatgacaattctgtatattccgtt**

**Citrus_aurantium** **ccgataatagataccgggactttgcaatatttgattgatgacaattctgtatattccgtt**

**Citrus_cavaleriei** **ccgataatagataccgggactttgcaatatttgattgatgacaattctgtatattccgtt**

**Citrus_hongheensis** **ccgataatagataccgggactttgcaatatttgattgatgacaattctgtatattccgtt**

**Citrus_limon** **ccgataatagataccgggactttgcaatatttgattgatgacaattctgtatattccgtt**

**Citrus_maxima** **ccgataatagataccgggactttgcaatatttgattgatgacaattctgtatattccgtt**

**Citrus_medica** **ccgataatagataccgggactttgcaatatttgattgatgacaattctgtatattccgtt**

**Citrus_polytrifolia** **ccgataatagataccgggactttgcaatatttgattgatgacaattctgtatattccgtt**

**Citrus_reticulata** **ccgataatagataccgggactttgcaatatttgattgatgacaattctgtatattccgtt**

**Citrus_sinensis** **ccgataatagataccgggactttgcaatatttgattgatgacaattctgtatattccgtt**

**Fortunella_bawangica_1** **ccgataatagataccgggactttgcaatatttgattgatgacaattctgtatattccgtt**

**Fortunella_bawangica_2** **ccgataatagataccgggactttgcaatatttgattgatgacaattctgtatattccgtt**

**Fortunella_hindsii_1** **ccgataatagataccgggactttgcaatatttgattgatgacaattctgtatattccgtt**

**Fortunella_hindsii_2** **ccgataatagataccgggactttgcaatatttgattgatgacaattctgtatattccgtt**

**Fortunella_hindsii_3** **ccgataatagataccgggactttgcaatatttgattgatgacaattctgtatattccgtt**

**Fortunella_japonica_1** **ccgataatagataccgggactttgcaatatttgattgatgacaattctgtatattccgtt**

**Fortunella_japonica_2** **ccgataatagataccgggactttgcaatatttgattgatgacaattctgtatattccgtt**

**Fortunella_japonica_3** **ccgataatagataccgggactttgcaatatttgattgatgacaattctgtatattccgtt**

**Fortunella_japonica_4** **ccgataatagataccgggactttgcaatatttgattgatgacaattctgtatattccgtt**

**Fortunella_japonica_5** **ccgataatagataccgggactttgcaatatttgattgatgacaattctgtatattccgtt**

**Fortunella_japonica_6** **ccgataatagataccgggactttgcaatatttgattgatgacaattctgtatattccgtt**

**Fortunella_japonica_7** **ccgataatagataccgggactttgcaatatttgattgatgacaattctgtatattccgtt**

**Fortunella_japonica_8** **ccgataatagataccgggactttgcaatatttgattgatgacaattctgtatattccgtt**

**Fortunella_japonica_9** **ccgataatagataccgggactttgcaatatttgattgatgacaattctgtatattccgtt**

**Fortunella_japonica_10** **ccgataatagataccgggactttgcaatatttgattgatgacaattctgtatattccgtt**

**Fortunella_japonica_11** **ccgataatagataccgggactttgcaatatttgattgatgacaattctgtatattccgtt**

**Fortunella_japonica_12** **ccgataatagataccgggactttgcaatatttgattgatgacaattctgtatattccgtt**

**Fortunella_margarita_1** **ccgataatagataccgggactttgcaatatttgattgatgacaattctgtatattccgtt**

**Fortunella_margarita_2** **ccgataatagataccgggactttgcaatatttgattgatgacaattctgtatattccgtt**

**Fortunella_venosa_1** **ccgataatagataccgggactttgcaatatttgattgatgacaattctgtatattccgtt**

**Fortunella_venosa_2** **ccgataatagataccgggactttgcaatatttgattgatgacaattctgtatattccgtt**

**Fortunella_venosa_3** **ccgataatagataccgggactttgcaatatttgattgatgacaattctgtatattccgtt**

28810	28820	28830	28840	28850	28860

....|....|....|....|....|....|....|....|....|....|....|....|

**Atalantia_kwangtungens** **tactatagaagttcccaaagaattcattagaggaatgtttccaataaaaatagtttgttc**

**Citrus_reticulata_1** **tactatagaagttcccaaagaattcattagaggaatgtttccaataaaaatagtttgttc**

**Citrus_reticulata_2** **tactatagaagttcccaaagaattcattagaggaatgtttccaataaaaatagtttgttc**

**Citrus_trifoliata_3** **tactatagaagttcccaaagaattcattagaggaatgtttccaataaaaatagtttgttc**

**Citrus_aurantiifolia** **tactatagaagttcccaaagaattcattagaggaatgtttccaataaaaatagtttgttc**

**Citrus_aurantium** **tactatagaagttcccaaagaattcattagaggaatgtttccaataaaaatagtttgttc**

**Citrus_cavaleriei** **tactatagaagttcccaaagaattcattagaggaatgtttccaataaaaatagtttgttc**

**Citrus_hongheensis** **tactatagaagttcccaaagaattcattagaggaatgtttccaataaaaatagtttgttc**

**Citrus_limon** **tactatagaagttcccaaagaattcattagaggaatgtttccaataaaaatagtttgttc**

**Citrus_maxima** **tactatagaagttcccaaagaattcattagaggaatgtttccaataaaaatagtttgttc**

**Citrus_medica** **tactatagaagttcccaaagaattcattagaggaatgtttccaataaaaatagtttgttc**

**Citrus_polytrifolia** **tactatagaagttcccaaagaattcattagaggaatgtttccaataaaaatagtttgttc**

**Citrus_reticulata** **tactatagaagttcccaaagaattcattagaggaatgtttccaataaaaatagtttgttc**

**Citrus_sinensis** **tactatagaagttcccaaagaattcattagaggaatgtttccaataaaaatagtttgttc**

**Fortunella_bawangica_1** **tactatagaagttcccaaagaattcattagaggaatgtttccaataaaaatagtttgttc**

**Fortunella_bawangica_2** **tactatagaagttcccaaagaattcattagaggaatgtttccaataaaaatagtttgttc**

**Fortunella_hindsii_1** **tactatagaagttcccaaagaattcattagaggaatgtttccaataaaaatagtttgttc**

**Fortunella_hindsii_2** **tactatagaagttcccaaagaattcattagaggaatgtttccaataaaaatagtttgttc**

**Fortunella_hindsii_3** **tactatagaagttcccaaagaattcattagaggaatgtttccaataaaaatagtttgttc**

**Fortunella_japonica_1** **tactatagaagttcccaaagaattcattagaggaatgtttccaataaaaatagtttgttc**

**Fortunella_japonica_2** **tactatagaagttcccaaagaattcattagaggaatgtttccaataaaaatagtttgttc**

**Fortunella_japonica_3** **tactatagaagttcccaaagaattcattagaggaatgtttccaataaaaatagtttgttc**

**Fortunella_japonica_4** **tactatagaagttcccaaagaattcattagaggaatgtttccaataaaaatagtttgttc**

**Fortunella_japonica_5** **tactatagaagttcccaaagaattcattagaggaatgtttccaataaaaatagtttgttc**

**Fortunella_japonica_6** **tactatagaagttcccaaagaattcattagaggaatgtttccaataaaaatagtttgttc**

**Fortunella_japonica_7** **tactatagaagttcccaaagaattcattagaggaatgtttccaataaaaatagtttgttc**

**Fortunella_japonica_8** **tactatagaagttcccaaagaattcattagaggaatgtttccaataaaaatagtttgttc**

**Fortunella_japonica_9** **tactatagaagttcccaaagaattcattagaggaatgtttccaataaaaatagtttgttc**

**Fortunella_japonica_10** **tactatagaagttcccaaagaattcattagaggaatgtttccaataaaaatagtttgttc**

**Fortunella_japonica_11** **tactatagaagttcccaaagaattcattagaggaatgtttccaataaaaatagtttgttc**

**Fortunella_japonica_12** **tactatagaagttcccaaagaattcattagaggaatgtttccaataaaaatagtttgttc**

**Fortunella_margarita_1** **tactatagaagttcccaaagaattcattagaggaatgtttccaataaaaatagtttgttc**

**Fortunella_margarita_2** **tactatagaagttcccaaagaattcattagaggaatgtttccaataaaaatagtttgttc**

**Fortunella_venosa_1** **tactatagaagttcccaaagaattcattagaggaatgtttccaataaaaatagtttgttc**

**Fortunella_venosa_2** **tactatagaagttcccaaagaattcattagaggaatgtttccaataaaaatagtttgttc**

**Fortunella_venosa_3** **tactatagaagttcccaaagaattcattagaggaatgtttccaataaaaatagtttgttc**

28870	28880	28890	28900	28910	28920

....|....|....|....|....|....|....|....|....|....|....|....|

**Atalantia_kwangtungens** **ctgcatgtcccctcggcttttccaaattaatccggcggatacatataattcagaagaata**

**Citrus_reticulata_1** **ctgcatgtcccctcggcttttccaaattaatcctgcggatacatataattcagaagaata**

**Citrus_reticulata_2** **ctgcatgtcccctcggcttttccaaattaatcctgcggatacatataattcagaagaata**

**Citrus_trifoliata_3** **ctgcatgtcccctcggcttttccaaattaatcctgcggatacatataattcagaagaata**

**Citrus_aurantiifolia** **ctgcatgtcccctcggcttttccaaattaatcctgcggatacatataattcagaagaata**

**Citrus_aurantium** **ctgcatgtcccctcggcttttccaaattaatcctgcggatacatataattcagaagaata**

**Citrus_cavaleriei** **ctgcatgtcccctcggcttttccaaattaatcctgcggatacatataattcagaagaata**

**Citrus_hongheensis** **ctgcatgtcccctcggcttttccaaattaatcctgcggatacatataattcagaagaata**

**Citrus_limon** **ctgcatgtcccctcggcttttccaaattaatcctgcggatacatataattcagaagaata**

**Citrus_maxima** **ctgcatgtcccctcggcttttccaaattaatcctgcggatacatataattcagaagaata**

**Citrus_medica** **ctgcatgtcccctcggcttttccaaattaatcctgcggatacatataattcagaagaata**

**Citrus_polytrifolia** **ctgcatgtcccctcggcttttccaaattaatcctgcggatacatataattcagaagaata**

**Citrus_reticulata** **ctgcatgtcccctcggcttttccaaattaatcctgcggatacatataattcagaagaata**

**Citrus_sinensis** **ctgcatgtcccctcggcttttccaaattaatcctgcggatacatataattcagaagaata**

**Fortunella_bawangica_1** **ctgcatgtcccctcggcttttccaaattaatcctgcggatacatataattcagaagaata**

**Fortunella_bawangica_2** **ctgcatgtcccctcggcttttccaaattaatcctgcggatacatataattcagaagaata**

**Fortunella_hindsii_1** **ctgcatgtcccctcggcttttccaaattaatcctgcggatacatataattcagaagaata**

**Fortunella_hindsii_2** **ctgcatgtcccctcggcttttccaaattaatcctgcggatacatataattcagaagaata**

**Fortunella_hindsii_3** **ctgcatgtcccctcggcttttccaaattaatcctgcggatacatataattcagaagaata**

**Fortunella_japonica_1** **ctgcatgtcccctcggcttttccaaattaatcctgcggatacatataattcagaagaata**

**Fortunella_japonica_2** **ctgcatgtcccctcggcttttccaaattaatcctgcggatacatataattcagaagaata**

**Fortunella_japonica_3** **ctgcatgtcccctcggcttttccaaattaatcctgcggatacatataattcagaagaata**

**Fortunella_japonica_4** **ctgcatgtcccctcggcttttccaaattaatcctgcggatacatataattcagaagaata**

**Fortunella_japonica_5** **ctgcatgtcccctcggcttttccaaattaatcctgcggatacatataattcagaagaata**

**Fortunella_japonica_6** **ctgcatgtcccctcggcttttccaaattaatcctgcggatacatataattcagaagaata**

**Fortunella_japonica_7** **ctgcatgtcccctcggcttttccaaattaatcctgcggatacatataattcagaagaata**

**Fortunella_japonica_8** **ctgcatgtcccctcggcttttccaaattaatcctgcggatacatataattcagaagaata**

**Fortunella_japonica_9** **ctgcatgtcccctcggcttttccaaattaatcctgcggatacatataattcagaagaata**

**Fortunella_japonica_10** **ctgcatgtcccctcggcttttccaaattaatcctgcggatacatataattcagaagaata**

**Fortunella_japonica_11** **ctgcatgtcccctcggcttttccaaattaatcctgcggatacatataattcagaagaata**

**Fortunella_japonica_12** **ctgcatgtcccctcggcttttccaaattaatcctgcggatacatataattcagaagaata**

**Fortunella_margarita_1** **ctgcatgtcccctcggcttttccaaattaatcctgcggatacatataattcagaagaata**

**Fortunella_margarita_2** **ctgcatgtcccctcggcttttccaaattaatcctgcggatacatataattcagaagaata**

**Fortunella_venosa_1** **ctgcatgtcccctcggcttttccaaattaatcctgcggatacatataattcagaagaata**

**Fortunella_venosa_2** **ctgcatgtcccctcggcttttccaaattaatcctgcggatacatataattcagaagaata**

**Fortunella_venosa_3** **ctgcatgtcccctcggcttttccaaattaatcctgcggatacatataattcagaagaata**

28930	28940	28950	28960	28970	28980

....|....|....|....|....|....|....|....|....|....|....|....|

**Atalantia_kwangtungens** **cgtgaatgattcatatacagcatctctttcttgtagcaagggttctaccaattgatatgt**

**Citrus_reticulata_1** **cgtgaatgattcatatacagcatctctttcttttagcaagggttctaccaattgatatgt**

**Citrus_reticulata_2** **cgtgaatgattcatatacagcatctctttcttttagcaagggttctaccaattgatatgt**

**Citrus_trifoliata_3** **cgtgaatgattcatatacagcatctctttcttttagcaagggttctaccaattgatatgt**

**Citrus_aurantiifolia** **cgtgaatgattcatatacagcatctctttcttttagcaagggttctaccaattgatatgt**

**Citrus_aurantium** **cgtgaatgattcatatacagcatctctttcttttagcaagggttctaccaattgatatgt**

**Citrus_cavaleriei** **cgtgaatgattcatatacagcatctctttcttttagcaagggttctaccaattgatatgt**

**Citrus_hongheensis** **cgtgaatgattcatatacagcatctctttcttttagcaagggttctaccaattgatatgt**

**Citrus_limon** **cgtgaatgattcatatacagcatctctttcttttagcaagggttctaccaattgatatgt**

**Citrus_maxima** **cgtgaatgattcatatacagcatctctttcttttagcaagggttctaccaattgatatgt**

**Citrus_medica** **cgtgaatgattcatatacagcatctctttcttttagcaagggttctaccaattgatatgt**

**Citrus_polytrifolia** **cgtgaatgattcatatacagcatctctttcttttagcaagggttctaccaattgatatgt**

**Citrus_reticulata** **cgtgaatgattcatatacagcatctctttcttttagcaagggttctaccaattgatatgt**

**Citrus_sinensis** **cgtgaatgattcatatacagcatctctttcttttagcaagggttctaccaattgatatgt**

**Fortunella_bawangica_1** **cgtgaatgattcatatacagcatctctttcttttagcaagggttctaccaattgatatgt**

**Fortunella_bawangica_2** **cgtgaatgattcatatacagcatctctttcttttagcaagggttctaccaattgatatgt**

**Fortunella_hindsii_1** **cgtgaatgattcatatacagcatctctttcttttagcaagggttctaccaattgatatgt**

**Fortunella_hindsii_2** **cgtgaatgattcatatacagcatctctttcttttagcaagggttctaccaattgatatgt**

**Fortunella_hindsii_3** **cgtgaatgattcatatacagcatctctttcttttagcaagggttctaccaattgatatgt**

**Fortunella_japonica_1** **cgtgaatgattcatatacagcatctctttcttttagcaagggttctaccaattgatatgt**

**Fortunella_japonica_2** **cgtgaatgattcatatacagcatctctttcttttagcaagggttctaccaattgatatgt**

**Fortunella_japonica_3** **cgtgaatgattcatatacagcatctctttcttttagcaagggttctaccaattgatatgt**

**Fortunella_japonica_4** **cgtgaatgattcatatacagcatctctttcttttagcaagggttctaccaattgatatgt**

**Fortunella_japonica_5** **cgtgaatgattcatatacagcatctctttcttttagcaagggttctaccaattgatatgt**

**Fortunella_japonica_6** **cgtgaatgattcatatacagcatctctttcttttagcaagggttctaccaattgatatgt**

**Fortunella_japonica_7** **cgtgaatgattcatatacagcatctctttcttttagcaagggttctaccaattgatatgt**

**Fortunella_japonica_8** **cgtgaatgattcatatacagcatctctttcttttagcaagggttctaccaattgatatgt**

**Fortunella_japonica_9** **cgtgaatgattcatatacagcatctctttcttttagcaagggttctaccaattgatatgt**

**Fortunella_japonica_10** **cgtgaatgattcatatacagcatctctttcttttagcaagggttctaccaattgatatgt**

**Fortunella_japonica_11** **cgtgaatgattcatatacagcatctctttcttttagcaagggttctaccaattgatatgt**

**Fortunella_japonica_12** **cgtgaatgattcatatacagcatctctttcttttagcaagggttctaccaattgatatgt**

**Fortunella_margarita_1** **cgtgaatgattcatatacagcatctctttcttttagcaagggttctaccaattgatatgt**

**Fortunella_margarita_2** **cgtgaatgattcatatacagcatctctttcttttagcaagggttctaccaattgatatgt**

**Fortunella_venosa_1** **cgtgaatgattcatatacagcatctctttcttttagcaagggttctaccaattgatatgt**

**Fortunella_venosa_2** **cgtgaatgattcatatacagcatctctttcttttagcaagggttctaccaattgatatgt**

**Fortunella_venosa_3** **cgtgaatgattcatatacagcatctctttcttttagcaagggttctaccaattgatatgt**

28990	29000	29010	29020	29030	29040

....|....|....|....|....|....|....|....|....|....|....|....|

**Atalantia_kwangtungens** **ttccacaaataattgaaattcaatttcttgatctgtatcttcaatttttggaaacttata**

**Citrus_reticulata_1** **ttccacaaataattgaaattcaatttcttgatctgtatcttcaatttttggaaacttata**

**Citrus_reticulata_2** **ttccacaaataattgaaattcaatttcttgatctgtatcttcaatttttggaaacttata**

**Citrus_trifoliata_3** **ttccacaaataattgaaattcaatttcttgatctgtatcttcaatttttggaaacttata**

**Citrus_aurantiifolia** **ttccacaaataattgaaattcaatttcttgatctgtatcttcaatttttggaaacttata**

**Citrus_aurantium** **ttccacaaataattgaaattcaatttcttgatctgtatcttcaatttttggaaacttata**

**Citrus_cavaleriei** **ttccacaaataattgaaattcaatttcttgatctgtatcttcaatttttggaaacttata**

**Citrus_hongheensis** **ttccacaaataattgaaattcaatttcttgatctgtatcttcaatttttggaaacttata**

**Citrus_limon** **ttccacaaataattgaaattcaatttcttgatctgtatcttcaatttttggaaacttata**

**Citrus_maxima** **ttccacaaataattgaaattcaatttcttgatctgtatcttcaatttttggaaacttata**

**Citrus_medica** **ttccacaaataattgaaattcaatttcttgatctgtatcttcaatttttggaaacttata**

**Citrus_polytrifolia** **ttccacaaataattgaaattcaatttcttgatctgtatcttcaatttttggaaacttata**

**Citrus_reticulata** **ttccacaaataattgaaattcaatttcttgatctgtatcttcaatttttggaaacttata**

**Citrus_sinensis** **ttccacaaataattgaaattcaatttcttgatctgtatcttcaatttttggaaacttata**

**Fortunella_bawangica_1** **ttccacaaataattgaaattcaatttcttgatctgtatcttcaatttttggaaacttata**

**Fortunella_bawangica_2** **ttccacaaataattgaaattcaatttcttgatctgtatcttcaatttttggaaacttata**

**Fortunella_hindsii_1** **ttccacaaataattgaaattcaatttcttgatctgtatcttcaatttttggaaacttata**

**Fortunella_hindsii_2** **ttccacaaataattgaaattcaatttcttgatctgtatcttcaatttttggaaacttata**

**Fortunella_hindsii_3** **ttccacaaataattgaaattcaatttcttgatctgtatcttcaatttttggaaacttata**

**Fortunella_japonica_1** **ttccacaaataattgaaattcaatttcttgatctgtatcttcaatttttggaaacttata**

**Fortunella_japonica_2** **ttccacaaataattgaaattcaatttcttgatctgtatcttcaatttttggaaacttata**

**Fortunella_japonica_3** **ttccacaaataattgaaattcaatttcttgatctgtatcttcaatttttggaaacttata**

**Fortunella_japonica_4** **ttccacaaataattgaaattcaatttcttgatctgtatcttcaatttttggaaacttata**

**Fortunella_japonica_5** **ttccacaaataattgaaattcaatttcttgatctgtatcttcaatttttggaaacttata**

**Fortunella_japonica_6** **ttccacaaataattgaaattcaatttcttgatctgtatcttcaatttttggaaacttata**

**Fortunella_japonica_7** **ttccacaaataattgaaattcaatttcttgatctgtatcttcaatttttggaaacttata**

**Fortunella_japonica_8** **ttccacaaataattgaaattcaatttcttgatctgtatcttcaatttttggaaacttata**

**Fortunella_japonica_9** **ttccacaaataattgaaattcaatttcttgatctgtatcttcaatttttggaaacttata**

**Fortunella_japonica_10** **ttccacaaataattgaaattcaatttcttgatctgtatcttcaatttttggaaacttata**

**Fortunella_japonica_11** **ttccacaaataattgaaattcaatttcttgatctgtatcttcaatttttggaaacttata**

**Fortunella_japonica_12** **ttccacaaataattgaaattcaatttcttgatctgtatcttcaatttttggaaacttata**

**Fortunella_margarita_1** **ttccacaaataattgaaattcaatttcttgatctgtatcttcaatttttggaaacttata**

**Fortunella_margarita_2** **ttccacaaataattgaaattcaatttcttgatctgtatcttcaatttttggaaacttata**

**Fortunella_venosa_1** **ttccacaaataattgaaattcaatttcttgatctgtatcttcaatttttggaaacttata**

**Fortunella_venosa_2** **ttccacaaataattgaaattcaatttcttgatctgtatcttcaatttttggaaacttata**

**Fortunella_venosa_3** **ttccacaaataattgaaattcaatttcttgatctgtatcttcaatttttggaaacttata**

29050	29060	29070	29080	29090	29100

....|....|....|....|....|....|....|....|....|....|....|....|

**Atalantia_kwangtungens** **aagctcttctgttaagccctgatcaatgaacctacaaaatccttcaaattggatctgatt**

**Citrus_reticulata_1** **aagctcttctgttaagccctgatcaatgaacctacaaaatccttcaaattgtatctgatt**

**Citrus_reticulata_2** **aagctcttctgttaagccctgatcaatgaacctacaaaatccttcaaattgtatctgatt**

**Citrus_trifoliata_3** **aagctcttctgttaagccctgatcaatgaacctacaaaatccttcaaattgtatctgatt**

**Citrus_aurantiifolia** **aagctcttctgttaagccctgatcaatgaacctacaaaatccttcaaattgtatctgatt**

**Citrus_aurantium** **aagctcttctgttaagccctgatcaatgaacctacaaaatccttcaaattgtatctgatt**

**Citrus_cavaleriei** **aagctcttctgttaagccctgatcaatgaacctacaaaatccttcaaattgtatctgatt**

**Citrus_hongheensis** **aagctcttctgttaagccctgatcaatgaacctacaaaatccttcaaattgtatctgatt**

**Citrus_limon** **aagctcttctgttaagccctgatcaatgaacctacaaaatccttcaaattgtatctgatt**

**Citrus_maxima** **aagctcttctgttaagccctgatcaatgaacctacaaaatccttcaaattgtatctgatt**

**Citrus_medica** **aagctcttctgttaagccctgatcaatgaacctacaaaatccttcaaattgtatctgatt**

**Citrus_polytrifolia** **aagctcttctgttaagccctgatcaatgaacctacaaaatccttcaaattgtatctgatt**

**Citrus_reticulata** **aagctcttctgttaagccctgatcaatgaacctacaaaatccttcaaattgtatctgatt**

**Citrus_sinensis** **aagctcttctgttaagccctgatcaatgaacctacaaaatccttcaaattgtatctgatt**

**Fortunella_bawangica_1** **aagctcttctgttaagccctgatcaatgaacctacaaaatccttcaaattgtatctgatt**

**Fortunella_bawangica_2** **aagctcttctgttaagccctgatcaatgaacctacaaaatccttcaaattgtatctgatt**

**Fortunella_hindsii_1** **aagctcttctgttaagccctgatcaatgaacctacaaaatccttcaaattgtatctgatt**

**Fortunella_hindsii_2** **aagctcttctgttaagccctgatcaatgaacctacaaaatccttcaaattgtatctgatt**

**Fortunella_hindsii_3** **aagctcttctgttaagccctgatcaatgaacctacaaaatccttcaaattgtatctgatt**

**Fortunella_japonica_1** **aagctcttctgttaagccctgatcaatgaacctacaaaatccttcaaattgtatctgatt**

**Fortunella_japonica_2** **aagctcttctgttaagccctgatcaatgaacctacaaaatccttcaaattgtatctgatt**

**Fortunella_japonica_3** **aagctcttctgttaagccctgatcaatgaacctacaaaatccttcaaattgtatctgatt**

**Fortunella_japonica_4** **aagctcttctgttaagccctgatcaatgaacctacaaaatccttcaaattgtatctgatt**

**Fortunella_japonica_5** **aagctcttctgttaagccctgatcaatgaacctacaaaatccttcaaattgtatctgatt**

**Fortunella_japonica_6** **aagctcttctgttaagccctgatcaatgaacctacaaaatccttcaaattgtatctgatt**

**Fortunella_japonica_7** **aagctcttctgttaagccctgatcaatgaacctacaaaatccttcaaattgtatctgatt**

**Fortunella_japonica_8** **aagctcttctgttaagccctgatcaatgaacctacaaaatccttcaaattgtatctgatt**

**Fortunella_japonica_9** **aagctcttctgttaagccctgatcaatgaacctacaaaatccttcaaattgtatctgatt**

**Fortunella_japonica_10** **aagctcttctgttaagccctgatcaatgaacctacaaaatccttcaaattgtatctgatt**

**Fortunella_japonica_11** **aagctcttctgttaagccctgatcaatgaacctacaaaatccttcaaattgtatctgatt**

**Fortunella_japonica_12** **aagctcttctgttaagccctgatcaatgaacctacaaaatccttcaaattgtatctgatt**

**Fortunella_margarita_1** **aagctcttctgttaagccctgatcaatgaacctacaaaatccttcaaattgtatctgatt**

**Fortunella_margarita_2** **aagctcttctgttaagccctgatcaatgaacctacaaaatccttcaaattgtatctgatt**

**Fortunella_venosa_1** **aagctcttctgttaagccctgatcaatgaacctacaaaatccttcaaattgtatctgatt**

**Fortunella_venosa_2** **aagctcttctgttaagccctgatcaatgaacctacaaaatccttcaaattgtatctgatt**

**Fortunella_venosa_3** **aagctcttctgttaagccctgatcaatgaacctacaaaatccttcaaattgtatctgatt**

29110	29120	29130	29140	29150	29160

....|....|....|....|....|....|....|....|....|....|....|....|

**Atalantia_kwangtungens** **caacccaggtattgtagacattcccgcatttccatctccgagcattttgaatttcccatt**

**Citrus_reticulata_1** **caacccaggtattgtagacattcctgcatttccatctccgagcattttgaatttcccatt**

**Citrus_reticulata_2** **caacccaggtattgtagacattcctgcatttccatctccgagcattttgaatttcccatt**

**Citrus_trifoliata_3** **caacccaggtattgtagacattcctgcatttccatctccgagcattttgaatttcccatt**

**Citrus_aurantiifolia** **caacccaggtattgtagacattcctgcatttccatctccgagcattttgaatttcccatt**

**Citrus_aurantium** **caacccaggtattgtagacattcctgcatttccatctccgagcattttgaatttcccatt**

**Citrus_cavaleriei** **caacccaggtattgtagacattcctgcatttccatctccgagcattttgaatttcccatt**

**Citrus_hongheensis** **caacccaggtattgtagacattcctgcatttccatctccgagcattttgaatttcccatt**

**Citrus_limon** **caacccaggtattgtagacattcctgcatttccatctccgagcattttgaatttcccatt**

**Citrus_maxima** **caacccaggtattgtagacattcctgcatttccatctccgagcattttgaatttcccatt**

**Citrus_medica** **caacccaggtattgtagacattcctgcatttccatctccgagcattttgaatttcccatt**

**Citrus_polytrifolia** **caacccaggtattgtagacattcctgcatttccatctccgagcattttgaatttcccatt**

**Citrus_reticulata** **caacccaggtattgtagacattcctgcatttccatctccgagcattttgaatttcccatt**

**Citrus_sinensis** **caacccaggtattgtagacattcctgcatttccatctccgagcattttgaatttcccatt**

**Fortunella_bawangica_1** **caacccaggtattgtagacattcctgcatttccatctccgagcattttgaatttcccatt**

**Fortunella_bawangica_2** **caacccaggtattgtagacattcctgcatttccatctccgagcattttgaatttcccatt**

**Fortunella_hindsii_1** **caacccaggtattgtagacattcctgcatttccatctccgagcattttgaatttcccatt**

**Fortunella_hindsii_2** **caacccaggtattgtagacattcctgcatttccatctccgagcattttgaatttcccatt**

**Fortunella_hindsii_3** **caacccaggtattgtagacattcctgcatttccatctccgagcattttgaatttcccatt**

**Fortunella_japonica_1** **caacccaggtattgtagacattcctgcatttccatctccgagcattttgaatttcccatt**

**Fortunella_japonica_2** **caacccaggtattgtagacattcctgcatttccatctccgagcattttgaatttcccatt**

**Fortunella_japonica_3** **caacccaggtattgtagacattcctgcatttccatctccgagcattttgaatttcccatt**

**Fortunella_japonica_4** **caacccaggtattgtagacattcctgcatttccatctccgagcattttgaatttcccatt**

**Fortunella_japonica_5** **caacccaggtattgtagacattcctgcatttccatctccgagcattttgaatttcccatt**

**Fortunella_japonica_6** **caacccaggtattgtagacattcctgcatttccatctccgagcattttgaatttcccatt**

**Fortunella_japonica_7** **caacccaggtattgtagacattcctgcatttccatctccgagcattttgaatttcccatt**

**Fortunella_japonica_8** **caacccaggtattgtagacattcctgcatttccatctccgagcattttgaatttcccatt**

**Fortunella_japonica_9** **caacccaggtattgtagacattcctgcatttccatctccgagcattttgaatttcccatt**

**Fortunella_japonica_10** **caacccaggtattgtagacattcctgcatttccatctccgagcattttgaatttcccatt**

**Fortunella_japonica_11** **caacccaggtattgtagacattcctgcatttccatctccgagcattttgaatttcccatt**

**Fortunella_japonica_12** **caacccaggtattgtagacattcctgcatttccatctccgagcattttgaatttcccatt**

**Fortunella_margarita_1** **caacccaggtattgtagacattcctgcatttccatctccgagcattttgaatttcccatt**

**Fortunella_margarita_2** **caacccaggtattgtagacattcctgcatttccatctccgagcattttgaatttcccatt**

**Fortunella_venosa_1** **caacccaggtattgtagacattcctgcatttccatctccgagcattttgaatttcccatt**

**Fortunella_venosa_2** **caacccaggtattgtagacattcctgcatttccatctccgagcattttgaatttcccatt**

**Fortunella_venosa_3** **caacccaggtattgtagacattcctgcatttccatctccgagcattttgaatttcccatt**

29170	29180	29190	29200	29210	29220

....|....|....|....|....|....|....|....|....|....|....|....|

**Atalantia_kwangtungens** **tatcaaaaaatcccattcgtttctcattctgcatcgattcatatgaatcgatgcagaatg**

**Citrus_reticulata_1** **tatcaaaaaatcccattcgtttctcattctgcatcgattcatatgattcgatgcagaatg**

**Citrus_reticulata_2** **tatcaaaaaatcccattcgtttctcattctgcatcgattcatatgattcgatgcagaatg**

**Citrus_trifoliata_3** **tatcaaaaaatcccatttgtttctcattctgcatcgattcatatgattcgatgcagaatg**

**Citrus_aurantiifolia** **tatcaaaaaatcccattcgtttctcattctgcatcgattcatatgattcgatgcagaatg**

**Citrus_aurantium** **tatcaaaaaatcccattcgtttctcattctgcatcgattcatatgattcgatgcagaatg**

**Citrus_cavaleriei** **tatcaaaaaatcccattcgtttctcattctgcatcgattcatatgattcgatgcagaatg**

**Citrus_hongheensis** **tatcaaaaaatcccattcgtttctcattctgcatcgattcatatgattcgatgcagaatg**

**Citrus_limon** **tatcaaaaaatcccattcgtttctcattctgcatcgattcatatgattcgatgcagaatg**

**Citrus_maxima** **tatcaaaaaatcccattcgtttctcattctgcatcgattcatatgattcgatgcagaatg**

**Citrus_medica** **tatcaaaaaatcccattcgtttctcattctgcatcgattcatatgaatcgatgcagaatg**

**Citrus_polytrifolia** **tatcaaaaaatcccatttgtttctcattctgcatcgaatcatatgattcgatgcagaatg**

**Citrus_reticulata** **tatcaaaaaatcccattcgtttctcattctgcatcgattcatatgattcgatgcagaatg**

**Citrus_sinensis** **tatcaaaaaatcccattcgtttctcattctgcatcgattcatatgattcgatgcagaatg**

**Fortunella_bawangica_1** **tatcaaaaaatcccattcgtttctcattctgcatcgattcatatgaatcgatgcagaatg**

**Fortunella_bawangica_2** **tatcaaaaaatcccattcgtttctcattctgcatcgattcatatgaatcgatgcagaatg**

**Fortunella_hindsii_1** **tatcaaaaaatcccattcgtttctcattctgcatcgattcatatgaatcgatgcagaatg**

**Fortunella_hindsii_2** **tatcaaaaaatcccattcgtttctcattctgcatcgattcatatgaatcgatgcagaatg**

**Fortunella_hindsii_3** **tatcaaaaaatcccattcgtttctcattctgcatcgattcatatgaatcgatgcagaatg**

**Fortunella_japonica_1** **tatcaaaaaatcccattcgtttctcattctgcatcgattcatatgaatcgatgcagaatg**

**Fortunella_japonica_2** **tatcaaaaaatcccattcgtttctcattctgcatcgattcatatgaatcgatgcagaatg**

**Fortunella_japonica_3** **tatcaaaaaatcccattcgtttctcattctgcatcgattcatatgaatcgatgcagaatg**

**Fortunella_japonica_4** **tatcaaaaaatcccattcgtttctcattctgcatcgattcatatgaatcgatgcagaatg**

**Fortunella_japonica_5** **tatcaaaaaatcccattcgtttctcattctgcatcgattcatatgaatcgatgcagaatg**

**Fortunella_japonica_6** **tatcaaaaaatcccattcgtttctcattctgcatcgattcatatgaatcgatgcagaatg**

**Fortunella_japonica_7** **tatcaaaaaatcccattcgtttctcattctgcatcgattcatatgaatcgatgcagaatg**

**Fortunella_japonica_8** **tatcaaaaaatcccattcgtttctcattctgcatcgattcatatgaatcgatgcagaatg**

**Fortunella_japonica_9** **tatcaaaaaatcccattcgtttctcattctgcatcgattcatatgaatcgatgcagaatg**

**Fortunella_japonica_10** **tatcaaaaaatcccattcgtttctcattctgcatcgattcatatgaatcgatgcagaatg**

**Fortunella_japonica_11** **tatcaaaaaatcccattcgtttctcattctgcatcgattcatatgaatcgatgcagaatg**

**Fortunella_japonica_12** **tatcaaaaaatcccattcgtttctcattctgcatcgattcatatgaatcgatgcagaatg**

**Fortunella_margarita_1** **tatcaaaaaatcccattcgtttctcattctgcatcgattcatatgaatcgatgcagaatg**

**Fortunella_margarita_2** **tatcaaaaaatcccattcgtttctcattctgcatcgattcatatgaatcgatgcagaatg**

**Fortunella_venosa_1** **tatcaaaaaatcccattcgtttctcattctgcatcgattcatatgaatcgatgcagaatg**

**Fortunella_venosa_2** **tatcaaaaaatcccattcgtttctcattctgcatcgattcatatgaatcgatgcagaatg**

**Fortunella_venosa_3** **tatcaaaaaatcccattcgtttctcattctgcatcgattcatatgaatcgatgcagaatg**

29230	29240	29250	29260	29270	29280

....|....|....|....|....|....|....|....|....|....|....|....|

**Atalantia_kwangtungens** **ctggaatttagattctgtttactgaatcgcatgaaattttacccaactccatatagatgg**

**Citrus_reticulata_1** **ctggaatttagattctgtttactgaatcgcatgaaattttacccaactccatatagatgg**

**Citrus_reticulata_2** **ctggaatttagattctgtttactgaatcgcatgaaattttacccaactccatatagatgg**

**Citrus_trifoliata_3** **ctggaatttagattctgtttactgaatcgcatgaaattttacccaactccatatagatgg**

**Citrus_aurantiifolia** **ctggaatttagattctgtttactgaatcgcatgaaattttacccaactccatatagatgg**

**Citrus_aurantium** **ctggaatttagattctgtttactgaatcgcatgaaattttacccaactccatatagatgg**

**Citrus_cavaleriei** **ctggaatttagattctgtttactgaatcgcatgaaattttacccaactccatatagatgg**

**Citrus_hongheensis** **ctggaatttagattctgtttactgaatcgcatgaaattttacccaactccatatagatgg**

**Citrus_limon** **ctggaatttagattctgtttactgaatcgcatgaaattttacccaactccatatagatgg**

**Citrus_maxima** **ctggaatttagattctgtttactgaatcgcatgaaattttacccaactccatatagatgg**

**Citrus_medica** **ctggaatttagattctgtttactgaatcgcatgaaattttacccaactccatatagatgg**

**Citrus_polytrifolia** **ctggaatttagattctgtttactgaatcgcatgaaattttacccaactccatatagatgg**

**Citrus_reticulata** **ctggaatttagattctgtttactgaatcgcatgaaattttacccaactccatatagatgg**

**Citrus_sinensis** **ctggaatttagattctgtttactgaatcgcatgaaattttacccaactccatatagatgg**

**Fortunella_bawangica_1** **ctggaatttagattctgtttactgaatcgcatgaaattttacccaactccatatagatgg**

**Fortunella_bawangica_2** **ctggaatttagattctgtttactgaatcgcatgaaattttacccaactccatatagatgg**

**Fortunella_hindsii_1** **ctggaatttagattctgtttactgaatcgcatgaaattttacccaactccatatagatgg**

**Fortunella_hindsii_2** **ctggaatttagattctgtttactgaatcgcatgaaattttacccaactccatatagatgg**

**Fortunella_hindsii_3** **ctggaatttagattctgtttactgaatcgcatgaaattttacccaactccatatagatgg**

**Fortunella_japonica_1** **ctggaatttagattctgtttactgaatcgcatgaaattttacccaactccatatagatgg**

**Fortunella_japonica_2** **ctggaatttagattctgtttactgaatcgcatgaaattttacccaactccatatagatgg**

**Fortunella_japonica_3** **ctggaatttagattctgtttactgaatcgcatgaaattttacccaactccatatagatgg**

**Fortunella_japonica_4** **ctggaatttagattctgtttactgaatcgcatgaaattttacccaactccatatagatgg**

**Fortunella_japonica_5** **ctggaatttagattctgtttactgaatcgcatgaaattttacccaactccatatagatgg**

**Fortunella_japonica_6** **ctggaatttagattctgtttactgaatcgcatgaaattttacccaactccatatagatgg**

**Fortunella_japonica_7** **ctggaatttagattctgtttactgaatcgcatgaaattttacccaactccatatagatgg**

**Fortunella_japonica_8** **ctggaatttagattctgtttactgaatcgcatgaaattttacccaactccatatagatgg**

**Fortunella_japonica_9** **ctggaatttagattctgtttactgaatcgcatgaaattttacccaactccatatagatgg**

**Fortunella_japonica_10** **ctggaatttagattctgtttactgaatcgcatgaaattttacccaactccatatagatgg**

**Fortunella_japonica_11** **ctggaatttagattctgtttactgaatcgcatgaaattttacccaactccatatagatgg**

**Fortunella_japonica_12** **ctggaatttagattctgtttactgaatcgcatgaaattttacccaactccatatagatgg**

**Fortunella_margarita_1** **ctggaatttagattctgtttactgaatcgcatgaaattttacccaactccatatagatgg**

**Fortunella_margarita_2** **ctggaatttagattctgtttactgaatcgcatgaaattttacccaactccatatagatgg**

**Fortunella_venosa_1** **ctggaatttagattctgtttactgaatcgcatgaaattttacccaactccatatagatgg**

**Fortunella_venosa_2** **ctggaatttagattctgtttactgaatcgcatgaaattttacccaactccatatagatgg**

**Fortunella_venosa_3** **ctggaatttagattctgtttactgaatcgcatgaaattttatccaactccatatagatgg**

29290	29300	29310	29320	29330	29340

....|....|....|....|....|....|....|....|....|....|....|....|

**Atalantia_kwangtungens** **aatgtatgaaatacggatgaacgggggaaaagggatgattttatacttaataaaattgga**

**Citrus_reticulata_1** **aatgtatgaaatacgtatgaacgggggaaaaaggatgattttatacttaataaaattgga**

**Citrus_reticulata_2** **aatgtatgaaatacgtatgaacgggggaaaaaggatgattttatacttaataaaattgga**

**Citrus_trifoliata_3** **aatgtatgaaatacgtatgaac-ggggaaaaaggatgattttatacttaataaaattgga**

**Citrus_aurantiifolia** **aatgtatgaaatacgtatgaacgggggaaaaaggatgattttatacttaataaaattgga**

**Citrus_aurantium** **aatgtatgaaatacgtatgaacgggggaaaaaggatgattttatacttaataaaattgga**

**Citrus_cavaleriei** **aatgtatgaaatacgtatgaacgggggaaaaaggatgattttatacttaataaaattgga**

**Citrus_hongheensis** **aatgtatgaaatacgtatgaacgggggaaaaaggatgattttatacttaataaaattgga**

**Citrus_limon** **aatgtatgaaatacgtatgaacgggggaaaaaggatgattttatacttaataaaattgga**

**Citrus_maxima** **aatgtatgaaatacgtatgaacgggggaaaaaggatgattttatacttaataaaattgga**

**Citrus_medica** **gatgtatgaaatacgtatgaacgggggaaaaaggatgattttatacttaataaaattgga**

**Citrus_polytrifolia** **aatgtatgaaatacgtatgaac-ggggaaaagggatgattttatacttaataaaattgga**

**Citrus_reticulata** **aatgtatgaaatacgtatgaacgggggaaaaaggatgattttatacttaataaaattgga**

**Citrus_sinensis** **aatgtatgaaatacgtatgaacgggggaaaaaggatgattttatacttaataaaattgga**

**Fortunella_bawangica_1** **aatgtatgaaatacgtatgaacgggggaaaaaggatgattttatacttaataaaattgga**

**Fortunella_bawangica_2** **aatgtatgaaatacgtatgaacgggggaaaaaggatgattttatacttaataaaattgga**

**Fortunella_hindsii_1** **aatgtatgaaatacgtatgaacgggggaaaaaggatgattttatacttaataaaattgga**

**Fortunella_hindsii_2** **aatgtatgaaatacgtatgaacgggggaaaaaggatgattttatacttaataaaattgga**

**Fortunella_hindsii_3** **aatgtatgaaatacgtatgaacgggggaaaaaggatgattttatacttaataaaattgga**

**Fortunella_japonica_1** **aatgtatgaaatacgtatgaacgggggaaaaaggatgattttatacttaataaaattgga**

**Fortunella_japonica_2** **aatgtatgaaatacgtatgaacgggggaaaaaggatgattttatacttaataaaattgga**

**Fortunella_japonica_3** **aatgtatgaaatacgtatgaacgggggaaaaaggatgattttatacttaataaaattgga**

**Fortunella_japonica_4** **aatgtatgaaatacgtatgaacgggggaaaaaggatgattttatacttaataaaattgga**

**Fortunella_japonica_5** **aatgtatgaaatacgtatgaacgggggaaaaaggatgattttatacttaataaaattgga**

**Fortunella_japonica_6** **aatgtatgaaatacgtatgaacgggggaaaaaggatgattttatacttaataaaattgga**

**Fortunella_japonica_7** **aatgtatgaaatacgtatgaacgggggaaaaaggatgattttatacttaataaaattgga**

**Fortunella_japonica_8** **aatgtatgaaatacgtatgaacgggggaaaaaggatgattttatacttaataaaattgga**

**Fortunella_japonica_9** **aatgtatgaaatacgtatgaacgggggaaaaaggatgattttatacttaataaaattgga**

**Fortunella_japonica_10** **aatgtatgaaatacgtatgaacgggggaaaaaggatgattttatacttaataaaattgga**

**Fortunella_japonica_11** **aatgtatgaaatacgtatgaacgggggaaaaaggatgattttatacttaataaaattgga**

**Fortunella_japonica_12** **aatgtatgaaatacgtatgaacgggggaaaaaggatgattttatacttaataaaattgga**

**Fortunella_margarita_1** **aatgtatgaaatacgtatgaacgggggaaaaaggatgattttatacttaataaaattgga**

**Fortunella_margarita_2** **aatgtatgaaatacgtatgaacgggggaaaaaggatgattttatacttaataaaattgga**

**Fortunella_venosa_1** **aatgtatgaaatacgtatgaacgggggaaaaaggatgattttatacttaataaaattgga**

**Fortunella_venosa_2** **aatgtatgaaatacgtatgaacgggggaaaaaggatgattttatacttaataaaattgga**

**Fortunella_venosa_3** **aatgtatgaaatacgtatgaacgggggaaaaaggatgattttatacttaataaaattgga**

29350	29360	29370	29380	29390	29400

....|....|....|....|....|....|....|....|....|....|....|....|

**Atalantia_kwangtungens** **atttctaagagacagatccaaatggaaaggaagcgataaattccacttagacttgcggag**

**Citrus_reticulata_1** **atttctaagagacagatccaaatggaaaggaagcgataaattccacttagacttacggag**

**Citrus_reticulata_2** **atttctaagagacagatccaaatggaaaggaagcgataaattccacttagacttacggag**

**Citrus_trifoliata_3** **atttctaagagacagatccaaatggaaaggaagcgataaatcacacttagacttacggag**

**Citrus_aurantiifolia** **atttctaagagacagatccaaatggaaaggaagcgataaattccacttagacttacggag**

**Citrus_aurantium** **atttctaagagacagatccaaatggaaaggaagcgataaattccacttagacttacggag**

**Citrus_cavaleriei** **atttctaagagacagatccaaatggaaaggaagcgataaattccacttagacttacggag**

**Citrus_hongheensis** **atttctaagagacagatccaaatggaaaggaagcgataaattccacttagacttacggag**

**Citrus_limon** **atttctaagagacagatccaaatggaaaggaagcgataaattccacttagacttacggag**

**Citrus_maxima** **atttctaagagacagatccaaatggaaaggaagcgataaattccacttagacttacggag**

**Citrus_medica** **atttctaagagacagatccaaatggaaaggaagcgataaattccacttagacttacggag**

**Citrus_polytrifolia** **atttctaagagacagatccaaatggaaaggaagcgataaattacacttagacttacggag**

**Citrus_reticulata** **atttctaagagacagatccaaatggaaaggaagcgataaattccacttagacttacggag**

**Citrus_sinensis** **atttctaagagacagatccaaatggaaaggaagcgataaattccacttagacttacggag**

**Fortunella_bawangica_1** **atttctaagagacagatccaaatggaaaggaagcgataaattccacttagacttacggag**

**Fortunella_bawangica_2** **atttctaagagacagatccaaatggaaaggaagcgataaattccacttagacttacggag**

**Fortunella_hindsii_1** **atttctaagagacagatccaaatggaaaggaagcgataaattccacttagacttacggag**

**Fortunella_hindsii_2** **atttctaagagacagatccaaatggaaaggaagcgataaattccacttagacttacggag**

**Fortunella_hindsii_3** **atttctaagagacagatccaaatggaaaggaagcgataaattccacttagacttacggag**

**Fortunella_japonica_1** **atttctaagagacagatccaaatggaaaggaagcgataaattccacttagacttacggag**

**Fortunella_japonica_2** **atttctaagagacagatccaaatggaaaggaagcgataaattccacttagacttacggag**

**Fortunella_japonica_3** **atttctaagagacagatccaaatggaaaggaagcgataaattccacttagacttacggag**

**Fortunella_japonica_4** **atttctaagagacagatccaaatggaaaggaagcgataaattccacttagacttacggag**

**Fortunella_japonica_5** **atttctaagagacagatccaaatggaaaggaagcgataaattccacttagacttacggag**

**Fortunella_japonica_6** **atttctaagagacagatccaaatggaaaggaagcgataaattccacttagacttacggag**

**Fortunella_japonica_7** **atttctaagagacagatccaaatggaaaggaagcgataaattccacttagacttacggag**

**Fortunella_japonica_8** **atttctaagagacagatccaaatggaaaggaagcgataaattccacttagacttacggag**

**Fortunella_japonica_9** **atttctaagagacagatccaaatggaaaggaagcgataaattccacttagacttacggag**

**Fortunella_japonica_10** **atttctaagagacagatccaaatggaaaggaagcgataaattccacttagacttacggag**

**Fortunella_japonica_11** **atttctaagagacagatccaaatggaaaggaagcgataaattccacttagacttacggag**

**Fortunella_japonica_12** **atttctaagagacagatccaaatggaaaggaagcgataaattccacttagacttacggag**

**Fortunella_margarita_1** **atttctaagagacagatccaaatggaaaggaagcgataaattccacttagacttacggag**

**Fortunella_margarita_2** **atttctaagagacagatccaaatggaaaggaagcgataaattccacttagacttacggag**

**Fortunella_venosa_1** **atttctaagagacagatccaaatggaaaggaagcgataaattccacttagacttacggag**

**Fortunella_venosa_2** **atttctaagagacagatccaaatggaaaggaagcgataaattccacttagacttacggag**

**Fortunella_venosa_3** **atttctaagagacagatccaaatggaaaggaagcgataaattccacttagacttacggag**

29410	29420	29430	29440	29450	29460

....|....|....|....|....|....|....|....|....|....|....|....|

**Atalantia_kwangtungens** **ttttgtatagaatc-aaaaaataattcaatttataccattattatgatattccaatccgt**

**Citrus_reticulata_1** **ttttgtatagaatcaaaaaaataattcaatttataccattattatgatattccaatccgt**

**Citrus_reticulata_2** **ttttgtatagaatcaaaaaaataattcaatttataccattattatgatattccaatccgt**

**Citrus_trifoliata_3** **ttttgtatagaatcaaaaaaataattcaatttataccattattatgatattccaatccgt**

**Citrus_aurantiifolia** **ttttgtatagaatcaaaaaaataattcaatttataccattattatgatattccaatccgt**

**Citrus_aurantium** **ttttgtatagaatcaaaaaaataattcaatttataccattattatgatattccaatccgt**

**Citrus_cavaleriei** **ttttgtatagaatcaaaaaaataattcaatttataccattattatgatattccaatccgt**

**Citrus_hongheensis** **ttttgtatagaatcaaaaaaataattcaatttataccattattatgatattccaatccgt**

**Citrus_limon** **ttttgtatagaatcaaaaaaataattcaatttataccattattatgatattccaatccgt**

**Citrus_maxima** **ttttgtatagaatcaaaaaaataattcaatttataccattattatgatattccaatccgt**

**Citrus_medica** **ttttgtatagaatcaaaaaaataattcaatttataccattattatgatattccaatccgt**

**Citrus_polytrifolia** **ttttgtatagaatcaaaaaaataattcaatttataccattattatgatattccaatccgt**

**Citrus_reticulata** **ttttgtatagaatcaaaaaaataattcaatttataccattattatgatattccaatccgt**

**Citrus_sinensis** **ttttgtatagaatcaaaaaaataattcaatttataccattattatgatattccaatccgt**

**Fortunella_bawangica_1** **ttttgtatagaatcaaaaaaataattcaatttataccattattatgatattccaatccgt**

**Fortunella_bawangica_2** **ttttgtatagaatcaaaaaaataattcaatttataccattattatgatattccaatccgt**

**Fortunella_hindsii_1** **ttttgtatagaatcaaaaaaataattcaatttataccattattatgatattccaatccgt**

**Fortunella_hindsii_2** **ttttgtatagaatcaaaaaaataattcaatttataccattattatgatattccaatccgt**

**Fortunella_hindsii_3** **ttttgtatagaatcaaaaaaataattcaatttataccattattatgatattccaatccgt**

**Fortunella_japonica_1** **ttttgtatagaatcaaaaaaataattcaatttataccattattatgatattccaatccgt**

**Fortunella_japonica_2** **ttttgtatagaatcaaaaaaataattcaatttataccattattatgatattccaatccgt**

**Fortunella_japonica_3** **ttttgtatagaatcaaaaaaataattcaatttataccattattatgatattccaatccgt**

**Fortunella_japonica_4** **ttttgtatagaatcaaaaaaataattcaatttataccattattatgatattccaatccgt**

**Fortunella_japonica_5** **ttttgtatagaatcaaaaaaataattcaatttataccattattatgatattccaatccgt**

**Fortunella_japonica_6** **ttttgtatagaatcaaaaaaataattcaatttataccattattatgatattccaatccgt**

**Fortunella_japonica_7** **ttttgtatagaatcaaaaaaataattcaatttataccattattatgatattccaatccgt**

**Fortunella_japonica_8** **ttttgtatagaatcaaaaaaataattcaatttataccattattatgatattccaatccgt**

**Fortunella_japonica_9** **ttttgtatagaatcaaaaaaataattcaatttataccattattatgatattccaatccgt**

**Fortunella_japonica_10** **ttttgtatagaatcaaaaaaataattcaatttataccattattatgatattccaatccgt**

**Fortunella_japonica_11** **ttttgtatagaatcaaaaaaataattcaatttataccattattatgatattccaatccgt**

**Fortunella_japonica_12** **ttttgtatagaatcaaaaaaataattcaatttataccattattatgatattccaatccgt**

**Fortunella_margarita_1** **ttttgtatagaatcaaaaaaataattcaatttataccattattatgatattccaatccgt**

**Fortunella_margarita_2** **ttttgtatagaatcaaaaaaataattcaatttataccattattatgatattccaatccgt**

**Fortunella_venosa_1** **ttttgtatagaatcaaaaaaataattcaatttataccattattatgatattccaatccgt**

**Fortunella_venosa_2** **ttttgtatagaatcaaaaaaataattcaatttataccattattatgatattccaatccgt**

**Fortunella_venosa_3** **ttttgtatagaatcaaaaaaataattcaatttataccattattatgatattccaatccgt**

29470	29480	29490	29500	29510	29520

....|....|....|....|....|....|....|....|....|....|....|....|

**Atalantia_kwangtungens** **taggataccagaaaaataaacgtcgtgatttgatctattcgctaagataaagacataaga**

**Citrus_reticulata_1** **taggataccagaaaaataaacgtcgtgatttgatctattcgctaagataaagacataaga**

**Citrus_reticulata_2** **taggataccagaaaaataaacgtcgtgatttgatctattcgctaagataaagacataaga**

**Citrus_trifoliata_3** **taggataccagaaaaataaacgtcgtgatttgatctattcgctaagataaagacataaga**

**Citrus_aurantiifolia** **taggataccagaaaaataaacgtcgtgatttgatctattcgctaagataaagacataaga**

**Citrus_aurantium** **taggataccagaaaaataaacgtcgtgatttgatctattcgctaagataaagacataaga**

**Citrus_cavaleriei** **taggataccagaaaaataaacgtcgtgatttgatctattcgctaagataaagacataaga**

**Citrus_hongheensis** **taggataccagaaaaataaacgtcgtgatttgatctattcgctaagataaagacataaga**

**Citrus_limon** **taggataccagaaaaataaacgtcgtgatttgatctattcgctaagataaagacataaga**

**Citrus_maxima** **taggataccagaaaaataaacgtcgtgatttgatctattcgctaagataaagacataaga**

**Citrus_medica** **taggataccagaaaaataaacgtcgtgatttgatctattcgctaagataaagacataaga**

**Citrus_polytrifolia** **taggataccagaaaaataaacgtcgtgatttgatctattcgctaagataaagacataaga**

**Citrus_reticulata** **taggataccagaaaaataaacgtcgtgatttgatctattcgctaagataaagacataaga**

**Citrus_sinensis** **taggataccagaaaaataaacgtcgtgatttgatctattcgctaagataaagacataaga**

**Fortunella_bawangica_1** **taggataccagaaaaataaacgtcgtgatttgatctattcgctaagataaagacataaga**

**Fortunella_bawangica_2** **taggataccagaaaaataaacgtcgtgatttgatctattcgctaagataaagacataaga**

**Fortunella_hindsii_1** **taggataccagaaaaataaacgtcgtgatttgatctattcgctaagataaagacataaga**

**Fortunella_hindsii_2** **taggataccagaaaaataaacgtcgtgatttgatctattcgctaagataaagacataaga**

**Fortunella_hindsii_3** **taggataccagaaaaataaacgtcgtgatttgatctattcgctaagataaagacataaga**

**Fortunella_japonica_1** **taggataccagaaaaataaacgtcgtgatttgatctattcgctaagataaagacataaga**

**Fortunella_japonica_2** **taggataccagaaaaataaacgtcgtgatttgatctattcgctaagataaagacataaga**

**Fortunella_japonica_3** **taggataccagaaaaataaacgtcgtgatttgatctattcgctaagataaagacataaga**

**Fortunella_japonica_4** **taggataccagaaaaataaacgtcgtgatttgatctattcgctaagataaagacataaga**

**Fortunella_japonica_5** **taggataccagaaaaataaacgtcgtgatttgatctattcgctaagataaagacataaga**

**Fortunella_japonica_6** **taggataccagaaaaataaacgtcgtgatttgatctattcgctaagataaagacataaga**

**Fortunella_japonica_7** **taggataccagaaaaataaacgtcgtgatttgatctattcgctaagataaagacataaga**

**Fortunella_japonica_8** **taggataccagaaaaataaacgtcgtgatttgatctattcgctaagataaagacataaga**

**Fortunella_japonica_9** **taggataccagaaaaataaacgtcgtgatttgatctattcgctaagataaagacataaga**

**Fortunella_japonica_10** **taggataccagaaaaataaacgtcgtgatttgatctattcgctaagataaagacataaga**

**Fortunella_japonica_11** **taggataccagaaaaataaacgtcgtgatttgatctattcgctaagataaagacataaga**

**Fortunella_japonica_12** **taggataccagaaaaataaacgtcgtgatttgatctattcgctaagataaagacataaga**

**Fortunella_margarita_1** **taggataccagaaaaataaacgtcgtgatttgatctattcgctaagataaagacataaga**

**Fortunella_margarita_2** **taggataccagaaaaataaacgtcgtgatttgatctattcgctaagataaagacataaga**

**Fortunella_venosa_1** **taggataccagaaaaataaacgtcgtgatttgatctattcgctaagataaagacataaga**

**Fortunella_venosa_2** **taggataccagaaaaataaacgtcgtgatttgatctattcgctaagataaagacataaga**

**Fortunella_venosa_3** **taggataccagaaaaataaacgtcgtgatttgatctattcgctaagataaagacataaga**

29530	29540	29550	29560	29570	29580

....|....|....|....|....|....|....|....|....|....|....|....|

**Atalantia_kwangtungens** **atcagacacaatataacagcgtaaagctcatttttttaacacttaacctttaaccttttc**

**Citrus_reticulata_1** **atcagacacaatataccagcgtaaagctcatttttttagcac-------ttacctttttc**

**Citrus_reticulata_2** **atcagacacaatataccagcgtaaagctcatttttttagcac-------ttacctttttc**

**Citrus_trifoliata_3** **atcagacacaatataccagcgtaaagctcatttttttagcac-------ttacctttttc**

**Citrus_aurantiifolia** **atcagacacaatataccagcgtaaagctcatttttttagcac-------ttacctttttc**

**Citrus_aurantium** **atcagacacaatataccagcgtaaagctcatttttttagcac-------ttacctttttc**

**Citrus_cavaleriei** **atcagacacaatataccagcgtaaagctcatttttttagcac-------ttacctttttc**

**Citrus_hongheensis** **atcagacacaatataccagcgtaaagctcatttttttagcac-------ttacctttttc**

**Citrus_limon** **atcagacacaatataccagcgtaaagctcatttttttagcac-------ttacctttttc**

**Citrus_maxima** **atcagacacaatataccagcgtaaagctcatttttttagcac-------ttacctttttc**

**Citrus_medica** **atcagacacaatataccagcgtaaagctcatttttttagcac-------ttacctttttc**

**Citrus_polytrifolia** **atcagacacaatataccagcgtaaagctcatttttttagcac-------ttacctttttc**

**Citrus_reticulata** **atcagacacaatataccagcgtaaagctcatttttttagcac-------ttacctttttc**

**Citrus_sinensis** **atcagacacaatataccagcgtaaagctcatttttttagcac-------ttacctttttc**

**Fortunella_bawangica_1** **atcagacacaatataccagcgtaaagctcatttttttagcac-------ttacctttttc**

**Fortunella_bawangica_2** **atcagacacaatataccagcgtaaagctcatttttttagcac-------ttacctttttc**

**Fortunella_hindsii_1** **atcagacacaatataccagcgtaaagctcatttttttagcac-------ttacctttttc**

**Fortunella_hindsii_2** **atcagacacaatataccagcgtaaagctcatttttttagcac-------ttacctttttc**

**Fortunella_hindsii_3** **atcagacacaatataccagcgtaaagctcatttttttagcac-------ttacctttttc**

**Fortunella_japonica_1** **atcagacacaatataccagcgtaaagctcatttttttagcac-------ttacctttttc**

**Fortunella_japonica_2** **atcagacacaatataccagcgtaaagctcatttttttagcac-------ttacctttttc**

**Fortunella_japonica_3** **atcagacacaatataccagcgtaaagctcatttttttagcac-------ttacctttttc**

**Fortunella_japonica_4** **atcagacacaatataccagcgtaaagctcatttttttagcac-------ttacctttttc**

**Fortunella_japonica_5** **atcagacacaatataccagcgtaaagctcatttttttagcac-------ttacctttttc**

**Fortunella_japonica_6** **atcagacacaatataccagcgtaaagctcatttttttagcac-------ttacctttttc**

**Fortunella_japonica_7** **atcagacacaatataccagcgtaaagctcatttttttagcac-------ttacctttttc**

**Fortunella_japonica_8** **atcagacacaatataccagcgtaaagctcatttttttagcac-------ttacctttttc**

**Fortunella_japonica_9** **atcagacacaatataccagcgtaaagctcatttttttagcac-------ttacctttttc**

**Fortunella_japonica_10** **atcagacacaatataccagcgtaaagctcatttttttagcac-------ttacctttttc**

**Fortunella_japonica_11** **atcagacacaatataccagcgtaaagctcatttttttagcac-------ttacctttttc**

**Fortunella_japonica_12** **atcagacacaatataccagcgtaaagctcatttttttagcac-------ttacctttttc**

**Fortunella_margarita_1** **atcagacacaatataccagcgtaaagctcatttttttagcac-------ttacctttttc**

**Fortunella_margarita_2** **atcagacacaatataccagcgtaaagctcatttttttagcac-------ttacctttttc**

**Fortunella_venosa_1** **atcagacacaatataccagcgtaaagctcatttttttagcac-------ttacctttttc**

**Fortunella_venosa_2** **atcagacacaatataccagcgtaaagctcatttttttagcac-------ttacctttttc**

**Fortunella_venosa_3** **atcagacacaatataccagcgtaaagctcatttttttagcac-------ttacctttttc**

29590	29600	29610	29620	29630	29640

....|....|....|....|....|....|....|....|....|....|....|....|

**Atalantia_kwangtungens** **gtggattccactgttcaaaaaatgatttgcggagaaccccctttttctttttttagtaga**

**Citrus_reticulata_1** **gtggattccactgttcaaaaaatgatttgcgaagaaccccctttttctttttttagtcga**

**Citrus_reticulata_2** **gtggattccactgttcaaaaaatgatttgcgaagaaccccctttttctttttttagtcga**

**Citrus_trifoliata_3** **gtggattccactgttcaaaaaatgatttgcgaagaaccccctttttctttttttagtaga**

**Citrus_aurantiifolia** **gtggattccactgttcaaaaaatgatttgcgaagaaccccctttttatttttttagtaga**

**Citrus_aurantium** **gtggattccactgttcaaaaaatgatttgcgaagaaccccctttttatttttttagtcga**

**Citrus_cavaleriei** **gtggattccactgttcaaaaaatgatttgcgaagaaccccctttttctttttttagtcga**

**Citrus_hongheensis** **gtggattccactgttcaaaaaatgatttgcgaagaaccccctttttatttttttagtcga**

**Citrus_limon** **gtggattccactgttcaaaaaatgatttgcgaagaaccccctttttatttttttagtcga**

**Citrus_maxima** **gtggattccactgttcaaaaaatgatttgcgaagaaccccctttttatttttttagtcga**

**Citrus_medica** **gtggattccactgttcaaaaaatgatttgcgaagaaccccctttttctttttttagtcga**

**Citrus_polytrifolia** **gtggattccactgttcaaaaaatgatttgcgaagaaccccctttttctttttttagtaga**

**Citrus_reticulata** **gtggattccactgttcaaaaaatgatttgcgaagaaccccctttttctttttttagtcga**

**Citrus_sinensis** **gtggattccactgttcaaaaaatgatttgcgaagaaccccctttttatttttttagtcga**

**Fortunella_bawangica_1** **gtggattccactgttcaaaaaatgatttgcgaagaaccccctttttctttttttagtaga**

**Fortunella_bawangica_2** **gtggattccactgttcaaaaaatgatttgcgaagaaccccctttttctttttttagtaga**

**Fortunella_hindsii_1** **gtggattccactgttcaaaaaatgatttgcgaagaaccccctttttctttttttagtaga**

**Fortunella_hindsii_2** **gtggattccactgttcaaaaaatgatttgcgaagaaccccctttttctttttttagtaga**

**Fortunella_hindsii_3** **gtggattccactgttcaaaaaatgatttgcgaagaaccccctttttctttttttagtaga**

**Fortunella_japonica_1** **gtggattccactgttcaaaaaatgatttgcgaagaaccccctttttctttttttagtaga**

**Fortunella_japonica_2** **gtggattccactgttcaaaaaatgatttgcgaagaaccccctttttctttttttagtaga**

**Fortunella_japonica_3** **gtggattccactgttcaaaaaatgatttgcgaagaaccccctttttctttttttagtaga**

**Fortunella_japonica_4** **gtggattccactgttcaaaaaatgatttgcgaagaaccccctttttctttttttagtaga**

**Fortunella_japonica_5** **gtggattccactgttcaaaaaatgatttgcgaagaaccccctttttctttttttagtaga**

**Fortunella_japonica_6** **gtggattccactgttcaaaaaatgatttgcgaagaaccccctttttctttttttagtaga**

**Fortunella_japonica_7** **gtggattccactgttcaaaaaatgatttgcgaagaaccccctttttctttttttagtaga**

**Fortunella_japonica_8** **gtggattccactgttcaaaaaatgatttgcgaagaaccccctttttctttttttagtaga**

**Fortunella_japonica_9** **gtggattccactgttcaaaaaatgatttgcgaagaaccccctttttctttttttagtaga**

**Fortunella_japonica_10** **gtggattccactgttcaaaaaatgatttgcgaagaaccccctttttctttttttagtaga**

**Fortunella_japonica_11** **gtggattccactgttcaaaaaatgatttgcgaagaaccccctttttctttttttagtaga**

**Fortunella_japonica_12** **gtggattccactgttcaaaaaatgatttgcgaagaaccccctttttctttttttagtaga**

**Fortunella_margarita_1** **gtggattccactgttcaaaaaatgatttgcgaagaaccccctttttctttttttagtaga**

**Fortunella_margarita_2** **gtggattccactgttcaaaaaatgatttgcgaagaaccccctttttctttttttagtaga**

**Fortunella_venosa_1** **gtggattccactgttcaaaaaatgatttgcgaagaaccccctttttctttttttagtaga**

**Fortunella_venosa_2** **gtggattccactgttcaaaaaatgatttgcgaagaaccccctttttctttttttagtaga**

**Fortunella_venosa_3** **gtggattccactgttcaaaaaatgatttgcgaagaaccccctttttctttttttagtaga**

29650	29660	29670	29680	29690	29700

....|....|....|....|....|....|....|....|....|....|....|....|

**Atalantia_kwangtungens** **atttttagaataggattgaagtgcgtaagacggcttgtatttagtaaacccgtgccgata**

**Citrus_reticulata_1** **atttttagaataggattgaagtgcgtaagacggcttgtatttagtaaacccgtgccgata**

**Citrus_reticulata_2** **atttttagaataggattgaagtgcgtaagacggcttgtatttagtaaacccgtgccgata**

**Citrus_trifoliata_3** **atttttagaataggattgaagtgcgtaagacggcttgtatttagtaaacccgtgccgata**

**Citrus_aurantiifolia** **atttttagaataggattgaagtgcgtaagacggcttgtatttagtaaacccgtgccgata**

**Citrus_aurantium** **atttttagaataggattgaagtgcgtaagacggcttgtatttagtaaacccgtgccgata**

**Citrus_cavaleriei** **atttttagaataggattgaagtgcgtaagacggcttgtatttagtaaacccgtgccgata**

**Citrus_hongheensis** **atttttagaataggattgaagtgcgtaagacggcttgtatttagtaaacccgtgccgata**

**Citrus_limon** **atttttagaataggattgaagtgcgtaagacggcttgtatttagtaaacccgtgccgata**

**Citrus_maxima** **atttttagaataggattgaagtgcgtaagacggcttgtatttagtaaacccgtgccgata**

**Citrus_medica** **attttttgaataggattgaagtgcgtaagacggcttgtatttagtaaacccgtgccgata**

**Citrus_polytrifolia** **atttttagaataggattgaagtgcgtaagacggcttgtatttcgtaaacccgtgccgata**

**Citrus_reticulata** **atttttagaataggattgaagtgcgtaagacggcttgtatttagtaaacccgtgccgata**

**Citrus_sinensis** **atttttagaataggattgaagtgcgtaagacggcttgtatttagtaaacccgtgccgata**

**Fortunella_bawangica_1** **atttttagaataggattgaagtgcgtaagacggcttgtatttagtaaacccgtgccgata**

**Fortunella_bawangica_2** **atttttagaataggattgaagtgcgtaagacggcttgtatttagtaaacccgtgccgata**

**Fortunella_hindsii_1** **atttttagaataggattgaagtgcgtaagacggcttgtatttagtaaacccgtgccgata**

**Fortunella_hindsii_2** **atttttagaataggattgaagtgcgtaagacggcttgtatttagtaaacccgtgccgata**

**Fortunella_hindsii_3** **atttttagaataggattgaagtgcgtaagacggcttgtatttagtaaacccgtgccgata**

**Fortunella_japonica_1** **atttttagaataggattgaagtgcgtaagacggcttgtatttagtaaacccgtgccgata**

**Fortunella_japonica_2** **atttttagaataggattgaagtgcgtaagacggcttgtatttagtaaacccgtgccgata**

**Fortunella_japonica_3** **atttttagaataggattgaagtgcgtaagacggcttgtatttagtaaacccgtgccgata**

**Fortunella_japonica_4** **atttttagaataggattgaagtgcgtaagacggcttgtatttagtaaacccgtgccgata**

**Fortunella_japonica_5** **atttttagaataggattgaagtgcgtaagacggcttgtatttagtaaacccgtgccgata**

**Fortunella_japonica_6** **atttttagaataggattgaagtgcgtaagacggcttgtatttagtaaacccgtgccgata**

**Fortunella_japonica_7** **atttttagaataggattgaagtgcgtaagacggcttgtatttagtaaacccgtgccgata**

**Fortunella_japonica_8** **atttttagaataggattgaagtgcgtaagacggcttgtatttagtaaacccgtgccgata**

**Fortunella_japonica_9** **atttttagaataggattgaagtgcgtaagacggcttgtatttagtaaacccgtgccgata**

**Fortunella_japonica_10** **atttttagaataggattgaagtgcgtaagacggcttgtatttagtaaacccgtgccgata**

**Fortunella_japonica_11** **atttttagaataggattgaagtgcgtaagacggcttgtatttagtaaacctgtgccgata**

**Fortunella_japonica_12** **atttttagaataggattgaagtgcgtaagacggcttgtatttagtaaacccgtgccgata**

**Fortunella_margarita_1** **atttttagaataggattgaagtgcgtaagacggcttgtatttagtaaacccgtgccgata**

**Fortunella_margarita_2** **atttttagaataggattgaagtgcgtaagacggcttgtatttagtaaacccgtgccgata**

**Fortunella_venosa_1** **atttttagaataggattgaagtgcgtaagacggcttgtatttagtaaacccgtgccgata**

**Fortunella_venosa_2** **atttttagaataggattgaagtgcgtaagacggcttgtatttagtaaacccgtgccgata**

**Fortunella_venosa_3** **atttttagaataggattgaagtgcgtaagacggcttgtatttagtaaacccgtgccgata**

29710	29720	29730	29740	29750	29760

....|....|....|....|....|....|....|....|....|....|....|....|

**Atalantia_kwangtungens** **tagctatctatatatacatcgcccccctttattgcatagttcgcttcgggacagcacatg**

**Citrus_reticulata_1** **tagctatctatatatacatcgcccccctttattgcatagttcgcttcgggacagcgcatg**

**Citrus_reticulata_2** **tagctatctatatatacatcgcccccctttattgcatagttcgcttcgggacagcgcatg**

**Citrus_trifoliata_3** **tagctatctatatatacatcgcccccctttattgcacagttcgcttcgggacagcgcatg**

**Citrus_aurantiifolia** **tagctatctatatatacatcgcccccctttattgcatagttcgcttcgggacagcgcatg**

**Citrus_aurantium** **tagctatctatatatacatcgcccccctttattgcatagttcgcttcgggacagcgcatg**

**Citrus_cavaleriei** **tagctatctatatatacatcgcccccctttattgcatagttcgcttcgggacagcgcatg**

**Citrus_hongheensis** **tagctatctatatatacatcgcccccctttattgcatagttcgcttcgggacagcgcatg**

**Citrus_limon** **tagctatctatatatacatcgcccccctttattgcatagttcgcttcgggacagcgcatg**

**Citrus_maxima** **tagctatctatatatacatcgcccccctttattgcatagttcgcttcgggacagcgcatg**

**Citrus_medica** **tag------atatatacatcgcccccc------gcatagttcgcttcgggacagcgcatg**

**Citrus_polytrifolia** **tagctatctatatatacatcgcccccctttattgcacagttcgcttcgggacagcgcatg**

**Citrus_reticulata** **tagctatctatatatacatcgcccccctttattgcatagttcgcttcgggacagcgcatg**

**Citrus_sinensis** **tagctatctatatatacatcgcccccctttattgcatagttcgcttcgggacagcgcatg**

**Fortunella_bawangica_1** **tagctatctatatatacatcgcccccctttattgcatagttcgcttcgggacagcgcatg**

**Fortunella_bawangica_2** **tagctatctatatatacatcgcccccctttattgcatagttcgcttcgggacagcgcatg**

**Fortunella_hindsii_1** **tagctatctatatatacatcgcccccctttattgcatagttcgcttcgggacagcgcatg**

**Fortunella_hindsii_2** **tagctatctatatatacatcgcccccctttattgcatagttcgcttcgggacagcgcatg**

**Fortunella_hindsii_3** **tagctatctatatatacatcgcccccctttattgcatagttcgcttcgggacagcgcatg**

**Fortunella_japonica_1** **tagctatctatatatacatcgcccccctttattgcatagttcgcttcgggacagcgcatg**

**Fortunella_japonica_2** **tagctatctatatatacatcgcccccctttattgcatagttcgcttcgggacagcgcatg**

**Fortunella_japonica_3** **tagctatctatatatacatcgcccccctttattgcatagttcgcttcgggacagcgcatg**

**Fortunella_japonica_4** **tagctatctatatatacatcgcccccctttattgcatagttcgcttcgggacagcgcatg**

**Fortunella_japonica_5** **tagctatctatatatacatcgcccccctttattgcatagttcgcttcgggacagcgcatg**

**Fortunella_japonica_6** **tagctatctatatatacatcgcccccctttattgcatagttcgcttcgggacagcgcatg**

**Fortunella_japonica_7** **tagctatctatatatacatcgcccccctttattgcatagttcgcttcgggacagcgcatg**

**Fortunella_japonica_8** **tagctatctatatatacatcgcccccctttattgcatagttcgcttcgggacagcgcatg**

**Fortunella_japonica_9** **tagctatctatatatacatcgcccccctttattgcatagttcgcttcgggacagcgcatg**

**Fortunella_japonica_10** **tagctatctatatatacatcgcccccctttattgcatagttcgcttcgggacagcgcatg**

**Fortunella_japonica_11** **tagctatctatatatacatcgcccccctttattgcatagttcgcttcgggacagcgcatg**

**Fortunella_japonica_12** **tagctatctatatatacatcgcccccctttattgcatagttcgcttcgggacagcgcatg**

**Fortunella_margarita_1** **tagctatctatatatacatcgcccccctttattgcatagttcgcttcgggacagcgcatg**

**Fortunella_margarita_2** **tagctatctatatatacatcgcccccctttattgcatagttcgcttcgggacagcgcatg**

**Fortunella_venosa_1** **tagctatctatatatacatcgcccccctttattgcatagttcgcttcgggacagcgcatg**

**Fortunella_venosa_2** **tagctatctatatatacatcgcccccctttattgcatagttcgcttcgggacagcgcatg**

**Fortunella_venosa_3** **tagctatctatatatacatcgcccccctttattgcatagttcgcttcgggacagcgcatg**

29770	29780	29790	29800	29810	29820

....|....|....|....|....|....|....|....|....|....|....|....|

**Atalantia_kwangtungens** **tcgtgctctatcaaaatttcgattttctcttcaatcta------aattgcagtacgacaa**

**Citrus_reticulata_1** **tcgtgctctatcaaaatttcgattttctcttcaatcta------aattgcagtacgacaa**

**Citrus_reticulata_2** **tcgtgctctatcaaaatttcgattttctcttcaatcta------aattgcagtacgacaa**

**Citrus_trifoliata_3** **tcgtgctctatcaaaattttgattttctcttcaatcta------aattgcagtacgacaa**

**Citrus_aurantiifolia** **tcgtgctctatcaaaatttcgattttctcttcaatcta------aattgcagtacgacaa**

**Citrus_aurantium** **tcgtgctctatcaaaatttcgattttctcttcaatcta------aattgcagtacgacaa**

**Citrus_cavaleriei** **tcgtgctctatcaaaatttcgattttctcttcaatcta------aattgcagtacgacaa**

**Citrus_hongheensis** **tcgtgctctatcaaaatttcgattttctcttcaatcta------aattgcagtacgacaa**

**Citrus_limon** **tcgtgctctatcaaaatttcgattttctcttcaatcta------aattgcagtacgacaa**

**Citrus_maxima** **tcgtgctctatcaaaatttcgattttctcttcaatcta------aattgcagtacgacaa**

**Citrus_medica** **tcgtgctctatcaaaatttcgattttctcttcaatcta------aattgcagtacgacaa**

**Citrus_polytrifolia** **tcgtgctctatcaaaattttgattttctcttcaatcta------aattgcagtacgacaa**

**Citrus_reticulata** **tcgtgctctatcaaaatttcgattttctcttcaatcta------aattgcagtacgacaa**

**Citrus_sinensis** **tcgtgctctatcaaaatttcgattttctcttcaatcta------aattgcagtacgacaa**

**Fortunella_bawangica_1** **tcgtgctctatcaaaatttcgattttctcttcaatctaaattgcaattgcagtacgacaa**

**Fortunella_bawangica_2** **tcgtgctctatcaaaatttcgattttctcttcaatctaaattgcaattgcagtacgacaa**

**Fortunella_hindsii_1** **tcgtgctctatcaaaatttcgattttctcttcaatctaaattgcaattgcagtacgacaa**

**Fortunella_hindsii_2** **tcgtgctctatcaaaatttcgattttctcttcaatctaaattgcaattgcagtacgacaa**

**Fortunella_hindsii_3** **tcgtgctctatcaaaatttcgattttctcttcaatctaaattgcaattgcagtacgacaa**

**Fortunella_japonica_1** **tcgtgctctatcaaaatttcgattttctcttcaatctaaattgcaattgcagtacgacaa**

**Fortunella_japonica_2** **tcgtgctctatcaaaatttcgattttctcttcaatctaaattgcaattgcagtacgacaa**

**Fortunella_japonica_3** **tcgtgctctatcaaaatttcgattttctcttcaatctaaattgcaattgcagtacgacaa**

**Fortunella_japonica_4** **tcgtgctctatcaaaatttcgattttctcttcaatctaaattgcaattgcagtacgacaa**

**Fortunella_japonica_5** **tcgtgctctatcaaaatttcgattttctcttcaatctaaattgcaattgcagtacgacaa**

**Fortunella_japonica_6** **tcgtgctctatcaaaatttcgattttctcttcaatctaaattgcaattgcagtacgacaa**

**Fortunella_japonica_7** **tcgtgctctatcaaaatttcgattttctcttcaatctaaattgcaattgcagtacgacaa**

**Fortunella_japonica_8** **tcgtgctctatcaaaatttcgattttctcttcaatctaaattgcaattgcagtacgacaa**

**Fortunella_japonica_9** **tcgtgctctatcaaaatttcgattttctcttcaatctaaattgcaattgcagtacgacaa**

**Fortunella_japonica_10** **tcgtgctctatcaaaatttcgattttctcttcaatctaaattgcaattgcagtacgacaa**

**Fortunella_japonica_11** **tcgtgctctatcaaaatttcgattttctcttcaatctaaattgcaattgcagtacgacaa**

**Fortunella_japonica_12** **tcgtgctctatcaaaatttcgattttctcttcaatctaaattgcaattgcagtacgacaa**

**Fortunella_margarita_1** **tcgtgctctatcaaaatttcgattttctcttcaatctaaattgcaattgcagtacgacaa**

**Fortunella_margarita_2** **tcgtgctctatcaaaatttcgattttctcttcaatctaaattgcaattgcagtacgacaa**

**Fortunella_venosa_1** **tcgtgctctatcaaaatttcgattttctcttcaatctaaattgcaattgcagtacgacaa**

**Fortunella_venosa_2** **tcgtgctctatcaaaatttcgattttctcttcaatctaaattgcaattgcagtacgacaa**

**Fortunella_venosa_3** **tcgtgctctatcaaaatttcgattttctcttcaatctaaattgcaattgcagtacgacaa**

29830	29840	29850	29860	29870	29880

....|....|....|....|....|....|....|....|....|....|....|....|

**Atalantia_kwangtungens** **ttttcggcaaattccctatagagaggcatcagacacagataagataaccaataagaataa**

**Citrus_reticulata_1** **ttttcggaaaattccctatagagaggcatcagacacagataagataacc------aataa**

**Citrus_reticulata_2** **ttttcggaaaattccctatagagaggcatcagacacagataagataacc------aataa**

**Citrus_trifoliata_3** **ttttcggcaaattccctatagagaggcatcagacacagataagatcacc------aataa**

**Citrus_aurantiifolia** **ttttcggcaaattccctatagagaggcatcagacacagataagataacc------aataa**

**Citrus_aurantium** **ttttcggcaaattccctatagagaggcatcagacacagataagataacc------aataa**

**Citrus_cavaleriei** **ttttcggcaaattccctatagagaggcatcagacacagataagataacc------aataa**

**Citrus_hongheensis** **ttttcggcaaattccctatagagaggcatcagacacagataagataacc------aataa**

**Citrus_limon** **ttttcggcaaattccctatagagaggcatcagacacagataagataacc------aataa**

**Citrus_maxima** **ttttcggcaaattccctatagagaggcatcagacacagataagataacc------aataa**

**Citrus_medica** **ttttcggcaaattccctatagagaggcatcagacacagataagataacc------aataa**

**Citrus_polytrifolia** **ttttcggcaaattccctatagagaggcatcagacacagataagatcacc------aataa**

**Citrus_reticulata** **ttttcggaaaattccctatagagaggcatcagacacagataagataacc------aataa**

**Citrus_sinensis** **ttttcggcaaattccctatagagaggcatcagacacagataagataacc------aataa**

**Fortunella_bawangica_1** **ttttcggcaaattccctatagagaggcatcagacacagataagataacc------aataa**

**Fortunella_bawangica_2** **ttttcggcaaattccctatagagaggcatcagacacagataagataacc------aataa**

**Fortunella_hindsii_1** **ttttcggcaaattccctatagagaggcatcagacacagataagataacc------aataa**

**Fortunella_hindsii_2** **ttttcggcaaattccctatagagaggcatcagacacagataagataacc------aataa**

**Fortunella_hindsii_3** **ttttcggcaaattccctatagagaggcatcagacacagataagataacc------aataa**

**Fortunella_japonica_1** **ttttcggcaaattccctatagagaggcatcagacacagataagataacc------aataa**

**Fortunella_japonica_2** **ttttcggcaaattccctatagagaggcatcagacacagataagataacc------aataa**

**Fortunella_japonica_3** **ttttcggcaaattccctatagagaggcatcagacacagataagataacc------aataa**

**Fortunella_japonica_4** **ttttcggcaaattccctatagagaggcatcagacacagataagataacc------aataa**

**Fortunella_japonica_5** **ttttcggcaaattccctatagagaggcatcagacacagataagataacc------aataa**

**Fortunella_japonica_6** **ttttcggcaaattccctatagagaggcatcagacacagataagataacc------aataa**

**Fortunella_japonica_7** **ttttcggcaaattccctatagagaggcatcagacacagataagataacc------aataa**

**Fortunella_japonica_8** **ttttcggcaaattccctatagagaggcatcagacacagataagataacc------aataa**

**Fortunella_japonica_9** **ttttcggcaaattccctatagagaggcatcagacacagataagataacc------aataa**

**Fortunella_japonica_10** **ttttcggcaaattccctatagagaggcatcagacacagataagataacc------aataa**

**Fortunella_japonica_11** **ttttcggcaaattccctatagagaggcatcagacacagataagataacc------aataa**

**Fortunella_japonica_12** **ttttcggcaaattccctatagagaggcatcagacacagataagataacc------aataa**

**Fortunella_margarita_1** **ttttcggcaaattccctatagagaggcatcagacacagataagataacc------aataa**

**Fortunella_margarita_2** **ttttcggcaaattccctatagagaggcatcagacacagataagataacc------aataa**

**Fortunella_venosa_1** **ttttcggcaaattccctatagagaggcatcagacacagataagataacc------aataa**

**Fortunella_venosa_2** **ttttcggcaaattccctatagagaggcatcagacacagataagataacc------aataa**

**Fortunella_venosa_3** **ttttcggcaaattccctatagagaggcatcagacacagataagataacc------aataa**

29890	29900	29910	29920	29930	29940

....|....|....|....|....|....|....|....|....|....|....|....|

**Atalantia_kwangtungens** **gctagtcgcgaaacgatttctgttttttatgtttggggtttacatatactcataattgct**

**Citrus_reticulata_1** **gctagccgcgaaacga---------tttatgtttggggtttacatatactcataattgct**

**Citrus_reticulata_2** **gctagccgcgaaacga---------tttatgtttggggtttacatatactcataattgct**

**Citrus_trifoliata_3** **gctagccgcgaaacga---------tttatgtttggggtttacatatactcataattgct**

**Citrus_aurantiifolia** **gctagccgcgaaacga---------tttatgtttggggtttacatatactcataattgct**

**Citrus_aurantium** **gctagccgcgaaacga---------tttatgtttggggtttacatatactcataattgct**

**Citrus_cavaleriei** **gctagccgcgaaacga---------tttatgtttggggtttacatatactcataattgct**

**Citrus_hongheensis** **gctagccgcgaaacga---------tttatgtttggggtttacatatactcataattgct**

**Citrus_limon** **gctagccgcgaaacga---------tttatgtttggggtttacatatactcataattgct**

**Citrus_maxima** **gctagccgcgaaacga---------tttatgtttggggtttacatatactcataattgct**

**Citrus_medica** **gctagccgcgaaacga---------tttatgtttggggtttacatatactcataattgct**

**Citrus_polytrifolia** **gctagccgcgaaacga---------tttatgtttggggtttacatatactcataattgct**

**Citrus_reticulata** **gctagccgcgaaacga---------tttatgtttggggtttacatatactcataattgct**

**Citrus_sinensis** **gctagccgcgaaacga---------tttatgtttggggtttacatatactcataattgct**

**Fortunella_bawangica_1** **gctagccgcgaaacga---------tttatgtttggggtttacatatactcataattgct**

**Fortunella_bawangica_2** **gctagccgcgaaacga---------tttatgtttggggtttacatatactcataattgct**

**Fortunella_hindsii_1** **gctagccgcgaaacga---------tttatgtttggggtttacatatactcataattgct**

**Fortunella_hindsii_2** **gctagccgcgaaacga---------tttatgtttggggtttacatatactcataattgct**

**Fortunella_hindsii_3** **gctagccgcgaaacga---------tttatgtttggggtttacatatactcataattgct**

**Fortunella_japonica_1** **gctagccgcgaaacga---------tttatgtttggggtttacatatactcataattgct**

**Fortunella_japonica_2** **gctagccgcgaaacga---------tttatgtttggggtttacatatactcataattgct**

**Fortunella_japonica_3** **gctagccgcgaaacga---------tttatgtttggggtttacatatactcataattgct**

**Fortunella_japonica_4** **gctagccgcgaaacga---------tttatgtttggggtttacatatactcataattgct**

**Fortunella_japonica_5** **gctagccgcgaaacga---------tttatgtttggggtttacatatactcataattgct**

**Fortunella_japonica_6** **gctagccgcgaaacga---------tttatgtttggggtttacatatactcataattgct**

**Fortunella_japonica_7** **gctagccgcgaaacga---------tttatgtttggggtttacatatactcataattgct**

**Fortunella_japonica_8** **gctagccgcgaaacga---------tttatgtttggggtttacatatactcataattgct**

**Fortunella_japonica_9** **gctagccgcgaaacga---------tttatgtttggggtttacatatactcataattgct**

**Fortunella_japonica_10** **gctagccgcgaaacga---------tttatgtttggggtttacatatactcataattgct**

**Fortunella_japonica_11** **gctagccgcgaaacga---------tttatgtttggggtttacatatactcataattgct**

**Fortunella_japonica_12** **gctagccgcgaaacga---------tttatgtttggggtttacatatactcataattgct**

**Fortunella_margarita_1** **gctagccgcgaaacga---------tttatgtttggggtttacatatactcataattgct**

**Fortunella_margarita_2** **gctagccgcgaaacga---------tttatgtttggggtttacatatactcataattgct**

**Fortunella_venosa_1** **gctagccgcgaaacga---------tttatgtttggggtttacatatactcataattgct**

**Fortunella_venosa_2** **gctagccgcgaaacga---------tttatgtttggggtttacatatactcataattgct**

**Fortunella_venosa_3** **gctagccgcgaaacga---------tttatgtttggggtttacatatactcataattgct**

29950	29960	29970	29980	29990	30000

....|....|....|....|....|....|....|....|....|....|....|....|

**Atalantia_kwangtungens** **gttataattgaaatggagaaggctttt------gttatagaaaaaaccaatattgattag**

**Citrus_reticulata_1** **gttataattgaaatggagaaggctttt------tttatagaaaaaaccaatattgattag**

**Citrus_reticulata_2** **gttataattgaaatggagaaggctttt------tttatagaaaaaaccaatattgattag**

**Citrus_trifoliata_3** **gttataattgaaatggagaaggctttttttatatttatagaaaaaaccaatattgattag**

**Citrus_aurantiifolia** **gttataattgaaatggagaaggctttt------tttatagaaaaaaccaatattgattag**

**Citrus_aurantium** **gttataattgaaatggagaaggctttt------tttatagaaaaaaccaatattgattag**

**Citrus_cavaleriei** **gttataattgaaatggagaaggctttt------tttatagaaaaaaccaatattgattag**

**Citrus_hongheensis** **gttataattgaaatggagaaggctttt------tttatagaaaaaaccaatattgattag**

**Citrus_limon** **gttataattgaaatggagaaggctttt------tttatagaaaaaaccaatattgattag**

**Citrus_maxima** **gttataattgaaatggagaaggctttt------tttatagaaaaaaccaatattgattag**

**Citrus_medica** **gttataattgaaatggagaaggctttt------tttatagaaaaaaccaatattgattag**

**Citrus_polytrifolia** **gttataattgaaatggagaaggctttttttatatttatagaaaaaaccaatattgattag**

**Citrus_reticulata** **gttataattgaaatggagaaggctttt------tttatagaaaaaaccaatattgattag**

**Citrus_sinensis** **gttataattgaaatggagaaggctttt------tttatagaaaaaaccaatattgattag**

**Fortunella_bawangica_1** **gttataattgaaatggagaaggctttt------tttatagaaaaaaccaatattgattag**

**Fortunella_bawangica_2** **gttataattgaaatggagaaggctttt------tttatagaaaaaaccaatattgattag**

**Fortunella_hindsii_1** **gttataattgaaatggagaaggctttt------tttatagaaaaaaccaatattgattag**

**Fortunella_hindsii_2** **gttataattgaaatggagaaggctttt------tttatagaaaaaaccaatattgattag**

**Fortunella_hindsii_3** **gttataattgaaatggagaaggctttt------tttatagaaaaaaccaatattgattag**

**Fortunella_japonica_1** **gttataattgaaatggagaaggctttt------tttatagaaaaaaccaatattgattag**

**Fortunella_japonica_2** **gttataattgaaatggagaaggctttt------tttatagaaaaaaccaatattgattag**

**Fortunella_japonica_3** **gttataattgaaatggagaaggctttt------tttatagaaaaaaccaatattgattag**

**Fortunella_japonica_4** **gttataattgaaatggagaaggctttt------tttatagaaaaaaccaatattgattag**

**Fortunella_japonica_5** **gttataattgaaatggagaaggctttt------tttatagaaaaaaccaatattgattag**

**Fortunella_japonica_6** **gttataattgaaatggagaaggctttt------tttatagaaaaaaccaatattgattag**

**Fortunella_japonica_7** **gttataattgaaatggagaaggctttt------tttatagaaaaaaccaatattgattag**

**Fortunella_japonica_8** **gttataattgaaatggagaaggctttt------tttatagaaaaaaccaatattgattag**

**Fortunella_japonica_9** **gttataattgaaatggagaaggctttt------tttatagaaaaaaccaatattgattag**

**Fortunella_japonica_10** **gttataattgaaatggagaaggctttt------tttatagaaaaaaccaatattgattag**

**Fortunella_japonica_11** **gttataattgaaatggagaaggctttt------tttatagaaaaaaccaatattgattag**

**Fortunella_japonica_12** **gttataattgaaatggagaaggctttt------tttatagaaaaaaccaatattgattag**

**Fortunella_margarita_1** **gttataattgaaatggagaaggctttt------tttatagaaaaaaccaatattgattag**

**Fortunella_margarita_2** **gttataattgaaatggagaaggctttt------tttatagaaaaaaccaatattgattag**

**Fortunella_venosa_1** **gttataattgaaatggagaaggctttt------tttatagaaaaaaccaatattgattag**

**Fortunella_venosa_2** **gttataattgaaatggagaaggctttt------tttatagaaaaaaccaatattgattag**

**Fortunella_venosa_3** **gttataattgaaatggagaaggctttt------tttatagaaaaaaccaatattgattag**

30010	30020	30030	30040	30050	30060

....|....|....|....|....|....|....|....|....|....|....|....|

**Atalantia_kwangtungens** **gtaggtatcaa-tttgattttcttctatcatcaggaaccacaatttc-------aattga**

**Citrus_reticulata_1** **gtaggtatcaattttgattttcttctatcattaggaaacaaaattgacaatttcaatttc**

**Citrus_reticulata_2** **gtaggtatcaattttgattttcttctatcattaggaaacaaaattgacaatttcaatttc**

**Citrus_trifoliata_3** **gtaggtatcaattttgattttcttctatcattaggaaacacaatttacaatttcaattta**

**Citrus_aurantiifolia** **gtaggtatcaattttgattttcttctatcattaggaaacacaatttacaatttcaattta**

**Citrus_aurantium** **gtaggtatcaattttgattttcttctatcattaggaaacacaatttacaattaaaattta**

**Citrus_cavaleriei** **gtaggtatcaattttgattttcttctatcattaggaaacaaaatttacaatttcaattta**

**Citrus_hongheensis** **gtaggtatcaattttgattttcttctatcattaggaaacacaatttacaattaaaattta**

**Citrus_limon** **gtaggtatcaattttgattttcttctatcattaggaaacacaatttacaattaaaattta**

**Citrus_maxima** **gtaggtatcaattttgattttcttctatcattaggaaacacaatttacaattaaaattta**

**Citrus_medica** **gtaggtatcaattttgattttcttctatcattaggaaacacaattta-------aattta**

**Citrus_polytrifolia** **gtaggtatcaattttgattttcttctatcattaggaaacacaatttacaatttcaattta**

**Citrus_reticulata** **gtaggtatcaattttgattttcttctatcattaggaaacaaaattgacaatttcaatttc**

**Citrus_sinensis** **gtaggtatcaattttgattttcttctatcattaggaaacacaatttacaattaaaattta**

**Fortunella_bawangica_1** **gtaggtatcaattttgattttcttctatcattaggaaacacaatttacaatttaaattta**

**Fortunella_bawangica_2** **gtaggtatcaattttgattttcttctatcattaggaaacacaatttacaatttaaattta**

**Fortunella_hindsii_1** **gtaggtatcaattttgattttcttctatcattaggaaacacaatttacaatttaaattta**

**Fortunella_hindsii_2** **gtaggtatcaattttgattttcttctatcattaggaaacacaatttacaatttaaattta**

**Fortunella_hindsii_3** **gtaggtatcaattttgattttcttctatcattaggaaacacaatttacaatttaaattta**

**Fortunella_japonica_1** **gtaggtatcaattttgattttcttctatcattaggaaacacaatttacaatttaaattta**

**Fortunella_japonica_2** **gtaggtatcaattttgattttcttctatcattaggaaacacaatttacaatttaaattta**

**Fortunella_japonica_3** **gtaggtatcaattttgattttcttctatcattaggaaacacaatttacaatttaaattta**

**Fortunella_japonica_4** **gtaggtatcaattttgattttcttctatcattaggaaacacaatttacaatttaaattta**

**Fortunella_japonica_5** **gtaggtatcaattttgattttcttctatcattaggaaacacaatttacaatttaaattta**

**Fortunella_japonica_6** **gtaggtatcaattttgattttcttctatcattaggaaacacaatttacaatttaaattta**

**Fortunella_japonica_7** **gtaggtatcaattttgattttcttctatcattaggaaacacaatttacaatttaaattta**

**Fortunella_japonica_8** **gtaggtatcaattttgattttcttctatcattaggaaacacaatttacaatttaaattta**

**Fortunella_japonica_9** **gtaggtatcaattttgattttcttctatcattaggaaacacaatttacaatttaaattta**

**Fortunella_japonica_10** **gtaggtatcaattttgattttcttctatcattaggaaacacaatttacaatttaaattta**

**Fortunella_japonica_11** **gtaggtatcaattttgattttcttctatcattaggaaacacaatttacaatttaaattta**

**Fortunella_japonica_12** **gtaggtatcaattttgattttcttctatcattaggaaacacaatttacaatttaaattta**

**Fortunella_margarita_1** **gtaggtatcaattttgattttcttctatcattaggaaacacaatttacaatttaaattta**

**Fortunella_margarita_2** **gtaggtatcaattttgattttcttctatcattaggaaacacaatttacaatttaaattta**

**Fortunella_venosa_1** **gtaggtatcaattttgattttcttctatcattaggaaacacaatttacaatttaaattta**

**Fortunella_venosa_2** **gtaggtatcaattttgattttcttctatcattaggaaacacaatttacaatttaaattta**

**Fortunella_venosa_3** **gtaggtatcaattttgattttcttctatcattaggaaacacaatttacaatttaaattta**

30070	30080	30090	30100	30110	30120

....|....|....|....|....|....|....|....|....|....|....|....|

**Atalantia_kwangtungens** **aatccaagagttggtcacgaatttacagtcactagttaatggttccaatttgtccttaat**

**Citrus_reticulata_1** **aatccaagagttggtcacgaattttcagtcactagttaatggttccaatttgtccttaat**

**Citrus_reticulata_2** **aatccaagagttggtcacgaattttcagtcactagttaatggttccaatttgtccttaat**

**Citrus_trifoliata_3** **aatccaagagttggtcacgaatttacagtcactagttaatggttccaatttgtccttaat**

**Citrus_aurantiifolia** **aatccaagagttggtcacgaatttacagtcactagttaatggttccaatttgtccttaat**

**Citrus_aurantium** **aatccaagagttggtcacgaatttacagtcactagttaatggttccaatttgtccttaat**

**Citrus_cavaleriei** **aatccaagagttggtcacgaatttacagtcactagttaatggttccaatttgtccttaat**

**Citrus_hongheensis** **aatccaagagttggtcacgaatttacagtcactagttaatggttccaatttgtccttaat**

**Citrus_limon** **aatccaagagttggtcacgaatttacagtcactagttaatggttccaatttgtccttaat**

**Citrus_maxima** **aatccaagagttggtcacgaatttacagtcactagttaatggttccaatttgtccttaat**

**Citrus_medica** **aatccaagagttggtcacgaatttacagtcactagttaatggttccaatttgtccttaat**

**Citrus_polytrifolia** **aatccaagagttggtcacgaatttacagtcactagttaatggttccaatttgtccttaat**

**Citrus_reticulata** **aatccaagagttggtcacgaattttcagtcactagttaatggttccaatttgtccttaat**

**Citrus_sinensis** **aatccaagagttggtcacgaatttacagtcactagttaatggttccaatttgtccttaat**

**Fortunella_bawangica_1** **aatccaagagttggtcacgaatttacagtcactagttaatggttccaatttgtccttaat**

**Fortunella_bawangica_2** **aatccaagagttggtcacgaatttacagtcactagttaatggttccaatttgtccttaat**

**Fortunella_hindsii_1** **aatccaagagttggtcacgaatttacagtcactagttaatggttccaatttgtccttaat**

**Fortunella_hindsii_2** **aatccaagagttggtcacgaatttacagtcactagttaatggttccaatttgtccttaat**

**Fortunella_hindsii_3** **aatccaagagttggtcacgaatttacagtcactagttaatggttccaatttgtccttaat**

**Fortunella_japonica_1** **aatccaagagttggtcacgaatttacagtcactagttaatggttccaatttgtccttaat**

**Fortunella_japonica_2** **aatccaagagttggtcacgaatttacagtcactagttaatggttccaatttgtccttaat**

**Fortunella_japonica_3** **aatccaagagttggtcacgaatttacagtcactagttaatggttccaatttgtccttaat**

**Fortunella_japonica_4** **aatccaagagttggtcacgaatttacagtcactagttaatggttccaatttgtccttaat**

**Fortunella_japonica_5** **aatccaagagttggtcacgaatttacagtcactagttaatggttccaatttgtccttaat**

**Fortunella_japonica_6** **aatccaagagttggtcacgaatttacagtcactagttaatggttccaatttgtccttaat**

**Fortunella_japonica_7** **aatccaagagttggtcacgaatttacagtcactagttaatggttccaatttgtccttaat**

**Fortunella_japonica_8** **aatccaagagttggtcacgaatttacagtcactagttaatggttccaatttgtccttaat**

**Fortunella_japonica_9** **aatccaagagttggtcacgaatttacagtcactagttaatggttccaatttgtccttaat**

**Fortunella_japonica_10** **aatccaagagttggtcacgaatttacagtcactagttaatggttccaatttgtccttaat**

**Fortunella_japonica_11** **aatccaagagttggtcacgaatttacagtcactagttaatggttccaatttgtccttaat**

**Fortunella_japonica_12** **aatccaagagttggtcacgaatttacagtcactagttaatggttccaatttgtccttaat**

**Fortunella_margarita_1** **aatccaagagttggtcacgaatttacagtcactagttaatggttccaatttgtccttaat**

**Fortunella_margarita_2** **aatccaagagttggtcacgaatttacagtcactagttaatggttccaatttgtccttaat**

**Fortunella_venosa_1** **aatccaagagttggtcacgaatttacagtcactagttaatggttccaatttgtccttaat**

**Fortunella_venosa_2** **aatccaagagttggtcacgaatttacagtcactagttaatggttccaatttgtccttaat**

**Fortunella_venosa_3** **aatccaagagttggtcacgaatttacagtcactagttaatggttccaatttgtccttaat**

30130	30140	30150	30160	30170	30180

....|....|....|....|....|....|....|....|....|....|....|....|

**Atalantia_kwangtungens** **tttggcttttgtgactgaaaatgcacatttcttttttcatttttcaatagaaaaaaagaa**

**Citrus_reticulata_1** **tttggcttttgtgactgaaaatgcacatttcttttttcatttttcaatagaaaaaaagaa**

**Citrus_reticulata_2** **tttggcttttgtgactgaaaatgcacatttcttttttcatttttcaatagaaaaaaagaa**

**Citrus_trifoliata_3** **tttggcttttgtgactgaaaatgcacatttcttttttcatttttcaatagaaaaaaagaa**

**Citrus_aurantiifolia** **tttggcttttgtgactgaaaatgcacatttcttttttcatttttcaatagaaaaaaagaa**

**Citrus_aurantium** **tttggcttttgtgactgaaaatgcacatttcttttttcatttttcaatagaaaaaaagaa**

**Citrus_cavaleriei** **tttggcttttgtgactgaaaatgcacatttcttttttcatttttcaatagaaaaaaagaa**

**Citrus_hongheensis** **tttggcttttgtgactgaaaatgcacatttcttttttcatttttcaatagaaaaaaagaa**

**Citrus_limon** **tttggcttttgtgactgaaaatgcacatttcttttttcatttttcaatagaaaaaaagaa**

**Citrus_maxima** **tttggcttttgtgactgaaaatgcacatttcttttttcatttttcaatagaaaaaaagaa**

**Citrus_medica** **tttggcttttgtgactgaaaatgcacatttcttttttcatttttcaatagaaaaaaagaa**

**Citrus_polytrifolia** **tttggcttttgtgactgaaaatgcacatttcttttttcatttttcaatagaaaaaaagaa**

**Citrus_reticulata** **tttggcttttgtgactgaaaatgcacatttcttttttcatttttcaatagaaaaaaagaa**

**Citrus_sinensis** **tttggcttttgtgactgaaaatgcacatttcttttttcatttttcaatagaaaaaaagaa**

**Fortunella_bawangica_1** **tttggcttttgtgactgaaaatgcacatttcttttttcatttttcaatagaaaaaaagaa**

**Fortunella_bawangica_2** **tttggcttttgtgactgaaaatgcacatttcttttttcatttttcaatagaaaaaaagaa**

**Fortunella_hindsii_1** **tttggcttttgtgactgaaaatgcacatttcttttttcatttttcaatagaaaaaaagaa**

**Fortunella_hindsii_2** **tttggcttttgtgactgaaaatgcacatttcttttttcatttttcaatagaaaaaaagaa**

**Fortunella_hindsii_3** **tttggcttttgtgactgaaaatgcacatttcttttttcatttttcaatagaaaaaaagaa**

**Fortunella_japonica_1** **tttggcttttgtgactgaaaatgcacatttcttttttcatttttcaatagaaaaaaagaa**

**Fortunella_japonica_2** **tttggcttttgtgactgaaaatgcacatttcttttttcatttttcaatagaaaaaaagaa**

**Fortunella_japonica_3** **tttggcttttgtgactgaaaatgcacatttcttttttcatttttcaatagaaaaaaagaa**

**Fortunella_japonica_4** **tttggcttttgtgactgaaaatgcacatttcttttttcatttttcaatagaaaaaaagaa**

**Fortunella_japonica_5** **tttggcttttgtgactgaaaatgcacatttcttttttcatttttcaatagaaaaaaagaa**

**Fortunella_japonica_6** **tttggcttttgtgactgaaaatgcacatttcttttttcatttttcaatagaaaaaaagaa**

**Fortunella_japonica_7** **tttggcttttgtgactgaaaatgcacatttcttttttcatttttcaatagaaaaaaagaa**

**Fortunella_japonica_8** **tttggcttttgtgactgaaaatgcacatttcttttttcatttttcaatagaaaaaaagaa**

**Fortunella_japonica_9** **tttggcttttgtgactgaaaatgcacatttcttttttcatttttcaatagaaaaaaagaa**

**Fortunella_japonica_10** **tttggcttttgtgactgaaaatgcacatttcttttttcatttttcaatagaaaaaaagaa**

**Fortunella_japonica_11** **tttggcttttgtgactgaaaatgcacatttcttttttcatttttcaatagaaaaaaagaa**

**Fortunella_japonica_12** **tttggcttttgtgactgaaaatgcacatttcttttttcatttttcaatagaaaaaaagaa**

**Fortunella_margarita_1** **tttggcttttgtgactgaaaatgcacatttcttttttcatttttcaatagaaaaaaagaa**

**Fortunella_margarita_2** **tttggcttttgtgactgaaaatgcacatttcttttttcatttttcaatagaaaaaaagaa**

**Fortunella_venosa_1** **tttggcttttgtgactgaaaatgcacatttcttttttcatttttcaatagaaaaaaagaa**

**Fortunella_venosa_2** **tttggcttttgtgactgaaaatgcacatttcttttttcatttttcaatagaaaaaaagaa**

**Fortunella_venosa_3** **tttggcttttgtgactgaaaatgcacatttcttttttcatttttcaatagaaaaaaagaa**

30190	30200	30210	30220	30230	30240

....|....|....|....|....|....|....|....|....|....|....|....|

**Atalantia_kwangtungens** **agagaaaagagagggattaagatgttgtgtgagatactataaaatcaattgaagaaccag**

**Citrus_reticulata_1** **agagaaaagagaggtattaagatgttgtgtgagatactataaaatcaattgaagaaccgg**

**Citrus_reticulata_2** **agagaaaagagaggtattaagatgttgtgtgagatactataaaatcaattgaagaaccgg**

**Citrus_trifoliata_3** **agagaaaagagagggattaagatgttgtgtgagatactataaaatcaattgaagaaccgg**

**Citrus_aurantiifolia** **agagaaaagagagggattaagatgttgtgtgagatactataaaatcaattgaagaaccgg**

**Citrus_aurantium** **agagaaaagagagggattaagatgttgtgtgagatactataaaatcaattgaagaaccgg**

**Citrus_cavaleriei** **agagaaaagagagggattaagatgttgtgtgagatactataaaatcaattgaagaaccgg**

**Citrus_hongheensis** **agagaaaagagagggattaagatgttgtgtgagatactataaaatcaattgaagaaccgg**

**Citrus_limon** **agagaaaagagagggattaagatgttgtgtgagatactataaaatcaattgaagaaccgg**

**Citrus_maxima** **agagaaaagagagggattaagatgttgtgtgagatactataaaatcaattgaagaaccgg**

**Citrus_medica** **agagaaaagagagggattaagatgttgtgtgagatactataaaatcaattgaagaaccgg**

**Citrus_polytrifolia** **agagaaaagagagggattaagatgttgtgtgagatactataaaatcaattgaagaaccgg**

**Citrus_reticulata** **agagaaaagagaggtattaagatgttgtgtgagatactataaaatcaattgaagaaccgg**

**Citrus_sinensis** **agagaaaagagagggattaagatgttgtgtgagatactataaaatcaattgaagaaccgg**

**Fortunella_bawangica_1** **agagaaaagatagggattaagatgttgtgtgagatactataaaatcaattgaagaaccgg**

**Fortunella_bawangica_2** **agagaaaagatagggattaagatgttgtgtgagatactataaaatcaattgaagaaccgg**

**Fortunella_hindsii_1** **agagaaaagatagggattaagatgttgtgtgagatactataaaatcaattgaagaaccgg**

**Fortunella_hindsii_2** **agagaaaagatagggattaagatgttgtgtgagatactataaaatcaattgaagaaccgg**

**Fortunella_hindsii_3** **agagaaaagatagggattaagatgttgtgtgagatactataaaatcaattgaagaaccgg**

**Fortunella_japonica_1** **agagaaaagatagggattaagatgttgtgtgagatactataaaatcaattgaagaaccgg**

**Fortunella_japonica_2** **agagaaaagatagggattaagatgttgtgtgagatactataaaatcaattgaagaaccgg**

**Fortunella_japonica_3** **agagaaaagatagggattaagatgttgtgtgagatactataaaatcaattgaagaaccgg**

**Fortunella_japonica_4** **agagaaaagatagggattaagatgttgtgtgagatactataaaatcaattgaagaaccgg**

**Fortunella_japonica_5** **agagaaaagatagggattaagatgttgtgtgagatactataaaatcaattgaagaaccgg**

**Fortunella_japonica_6** **agagaaaagatagggattaagatgttgtgtgagatactataaaatcaattgaagaaccgg**

**Fortunella_japonica_7** **agagaaaagatagggattaagatgttgtgtgagatactataaaatcaattgaagaaccgg**

**Fortunella_japonica_8** **agagaaaagatagggattaagatgttgtgtgagatactataaaatcaattgaagaaccgg**

**Fortunella_japonica_9** **agagaaaagatagggattaagatgttgtgtgagatactataaaatcaattgaagaaccgg**

**Fortunella_japonica_10** **agagaaaagatagggattaagatgttgtgtgagatactataaaatcaattgaagaaccgg**

**Fortunella_japonica_11** **agagaaaagatagggattaagatgttgtgtgagatactataaaatcaattgaagaaccgg**

**Fortunella_japonica_12** **agagaaaagatagggattaagatgttgtgtgagatactataaaatcaattgaagaaccgg**

**Fortunella_margarita_1** **agagaaaagatagggattaagatgttgtgtgagatactataaaatcaattgaagaaccgg**

**Fortunella_margarita_2** **agagaaaagatagggattaagatgttgtgtgagatactataaaatcaattgaagaaccgg**

**Fortunella_venosa_1** **agagaaaagatagggattaagatgttgtgtgagatactataaaatcaattgaagaaccgg**

**Fortunella_venosa_2** **agagaaaagatagggattaagatgttgtgtgagatactataaaatcaattgaagaaccgg**

**Fortunella_venosa_3** **agagaaaagatagggattaagatgttgtgtgagatactataaaatcaattgaagaaccgg**

30250	30260	30270	30280	30290	30300

....|....|....|....|....|....|....|....|....|....|....|....|

**Atalantia_kwangtungens** **agccgatccaaaaaaaggacagattttttttaggcttttaggcaaggaattaagaaaaac**

**Citrus_reticulata_1** **agccgatccaaaaaaaggacatatttt--------ttttaggcaaggaattaagaaaaac**

**Citrus_reticulata_2** **agccgatccaaaaaaaggacatatttt--------ttttaggcaaggaattaagaaaaac**

**Citrus_trifoliata_3** **agccgatccaaaaaaaggacatatttt--------ttttaggcaaggaattaagaaaaac**

**Citrus_aurantiifolia** **agccgatccaaaaaaaggacatatttt--------ttttaggcaaggaattaagaaaaac**

**Citrus_aurantium** **agccgatccaaaaaaaggacatatttt--------ttttaggcaaggaattaagaaaaac**

**Citrus_cavaleriei** **agccgatccaaaaaaaggacatatttt--------ttttaggcaaggaattaagaaaaac**

**Citrus_hongheensis** **agccgatccaaaaaaaggacatatttt--------ttttaggcaaggaattaagaaaaac**

**Citrus_limon** **agccgatccaaaaaaaggacatatttt--------ttttaggcaaggaattaagaaaaac**

**Citrus_maxima** **agccgatccaaaaaaaggacatatttt--------ttttaggcaaggaattaagaaaaac**

**Citrus_medica** **agccgatccaaaaaaaggacatatttt--------ttttaggcaaggaattaagaaaaac**

**Citrus_polytrifolia** **agccgatccaaaaaaaggacatatttt--------ttttaggcaaggaattaagaaaaac**

**Citrus_reticulata** **agccgatccaaaaaaaggacatatttt--------ttttaggcaaggaattaagaaaaac**

**Citrus_sinensis** **agccgatccaaaaaaaggacatatttt--------ttttaggcaaggaattaagaaaaac**

**Fortunella_bawangica_1** **agccgatccaaaaaaaggacatatttt--------ttttaggcaaggaattaagaaaaac**

**Fortunella_bawangica_2** **agccgatccaaaaaaaggacatatttt--------ttttaggcaaggaattaagaaaaac**

**Fortunella_hindsii_1** **agccgatccaaaaaaaggacatatttt--------ttttaggcaaggaattaagaaaaac**

**Fortunella_hindsii_2** **agccgatccaaaaaaaggacatatttt--------ttttaggcaaggaattaagaaaaac**

**Fortunella_hindsii_3** **agccgatccaaaaaaaggacatatttt--------ttttaggcaaggaattaagaaaaac**

**Fortunella_japonica_1** **agccgatccaaaaaaaggacatatttt--------ttttaggcaaggaattaagaaaaac**

**Fortunella_japonica_2** **agccgatccaaaaaaaggacatatttt--------ttttaggcaaggaattaagaaaaac**

**Fortunella_japonica_3** **agccgatccaaaaaaaggacatatttt--------ttttaggcaaggaattaagaaaaac**

**Fortunella_japonica_4** **agccgatccaaaaaaaggacatatttt--------ttttaggcaaggaattaagaaaaac**

**Fortunella_japonica_5** **agccgatccaaaaaaaggacatatttt--------ttttaggcaaggaattaagaaaaac**

**Fortunella_japonica_6** **agccgatccaaaaaaaggacatatttt--------ttttaggcaaggaattaagaaaaac**

**Fortunella_japonica_7** **agccgatccaaaaaaaggacatatttt--------ttttaggcaaggaattaagaaaaac**

**Fortunella_japonica_8** **agccgatccaaaaaaaggacatatttt--------ttttaggcaaggaattaagaaaaac**

**Fortunella_japonica_9** **agccgatccaaaaaaaggacatatttt--------ttttaggcaaggaattaagaaaaac**

**Fortunella_japonica_10** **agccgatccaaaaaaaggacatatttt--------ttttaggcaaggaattaagaaaaac**

**Fortunella_japonica_11** **agccgatccaaaaaaaggacatatttt--------ttttaggcaaggaattaagaaaaac**

**Fortunella_japonica_12** **agccgatccaaaaaaaggacatatttt--------ttttaggcaaggaattaagaaaaac**

**Fortunella_margarita_1** **agccgatccaaaaaaaggacatatttt--------ttttaggcaaggaattaagaaaaac**

**Fortunella_margarita_2** **agccgatccaaaaaaaggacatatttt--------ttttaggcaaggaattaagaaaaac**

**Fortunella_venosa_1** **agccgatccaaaaaaaggacatatttt--------ttttaggcaaggaattaagaaaaac**

**Fortunella_venosa_2** **agccgatccaaaaaaaggacatatttt--------ttttaggcaaggaattaagaaaaac**

**Fortunella_venosa_3** **agccgatccaaaaaaaggacatatttt--------ttttaggcaaggaattaagaaaaac**

30310	30320	30330	30340	30350	30360

....|....|....|....|....|....|....|....|....|....|....|....|

**Atalantia_kwangtungens** **gaaatttcagatggacgtacacaaaaaaaagaaaagacattgaata---cccccccaaac**

**Citrus_reticulata_1** **gagattttatatggacgtacacaaaaaaaagaaaagacattgagtaccccccccccaaac**

**Citrus_reticulata_2** **gagattttatatggacgtacacaaaaaaaagaaaagacattgagtaccccccccccaaac**

**Citrus_trifoliata_3** **gagattttatatggacgtacacaaaaaaaagaaaagacattgagta---cccccccaaac**

**Citrus_aurantiifolia** **gagattttatatggacgtacacaaaaaaaagaaaagacattgagtaccccccccccaaac**

**Citrus_aurantium** **gagattttatatggacgtacacaaaaaaaagaaaagacattgagta-cccccccccaaac**

**Citrus_cavaleriei** **gagattttatatggacgtacacaaaaaaaagaaaagacattgagta-cccccccccaaac**

**Citrus_hongheensis** **gagattttatatggacgtacacaaaaaaaagaaaagacattgagta-cccccccccaaac**

**Citrus_limon** **gagattttatatggacgtacacaaaaaaaagaaaagacattgagta-cccccccccaaac**

**Citrus_maxima** **gagattttatatggacgtacacaaaaaaaagaaaagacattgagtaccccccccccaaac**

**Citrus_medica** **gagattttatatggacgtacacaaaaaaaagaaaagacattgagta-cccccccccaaac**

**Citrus_polytrifolia** **gagattttatatggacgtacacaaaaaaaagaaaagacattgagta---cccccccaaac**

**Citrus_reticulata** **gagattttatatggacgtacacaaaaaaaagaaaagacattgagtaccccccccccaaac**

**Citrus_sinensis** **gagattttatatggacgtacacaaaaaaaagaaaagacattgagtaccccccccccaaac**

**Fortunella_bawangica_1** **gagattttatatggacgtacacaaaaaaaagaaaagacattgagta--ccccccccaaac**

**Fortunella_bawangica_2** **gagattttatatggacgtacacaaaaaaaagaaaagacattgagta--ccccccccaaac**

**Fortunella_hindsii_1** **gagattttatatggacgtacacaaaaaaaagaaaagacattgagta--ccccccccaaac**

**Fortunella_hindsii_2** **gagattttatatggacgtacacaaaaaaaagaaaagacattgagta--ccccccccaaac**

**Fortunella_hindsii_3** **gagattttatatggacgtacacaaaaaaaagaaaagacattgagta--ccccccccaaac**

**Fortunella_japonica_1** **gagattttatatggacgtacacaaaaaaaagaaaagacattgagta--ccccccccaaac**

**Fortunella_japonica_2** **gagattttatatggacgtacacaaaaaaaagaaaagacattgagta--ccccccccaaac**

**Fortunella_japonica_3** **gagattttatatggacgtacacaaaaaaaagaaaagacattgagta--ccccccccaaac**

**Fortunella_japonica_4** **gagattttatatggacgtacacaaaaaaaagaaaagacattgagta--ccccccccaaac**

**Fortunella_japonica_5** **gagattttatatggacgtacacaaaaaaaagaaaagacattgagta--ccccccccaaac**

**Fortunella_japonica_6** **gagattttatatggacgtacacaaaaaaaagaaaagacattgagta--ccccccccaaac**

**Fortunella_japonica_7** **gagattttatatggacgtacacaaaaaaaagaaaagacattgagta--ccccccccaaac**

**Fortunella_japonica_8** **gagattttatatggacgtacacaaaaaaaagaaaagacattgagta--ccccccccaaac**

**Fortunella_japonica_9** **gagattttatatggacgtacacaaaaaaaagaaaagacattgagta--ccccccccaaac**

**Fortunella_japonica_10** **gagattttatatggacgtacacaaaaaaaagaaaagacattgagta--ccccccccaaac**

**Fortunella_japonica_11** **gagattttatatggacgtacacaaaaaaaagaaaagacattgagta--ccccccccaaac**

**Fortunella_japonica_12** **gagattttatatggacgtacacaaaaaaaagaaaagacattgagta--ccccccccaaac**

**Fortunella_margarita_1** **gagattttatatggacgtacacaaaaaaaagaaaagacattgagta--ccccccccaaac**

**Fortunella_margarita_2** **gagattttatatggacgtacacaaaaaaaagaaaagacattgagta--ccccccccaaac**

**Fortunella_venosa_1** **gagattttatatggacgtacacaaaaaaaagaaaagacattgagta--ccccccccaaac**

**Fortunella_venosa_2** **gagattttatatggacgtacacaaaaaaaagaaaagacattgagta--ccccccccaaac**

**Fortunella_venosa_3** **gagattttatatggacgtacacaaaaaaaagaaaagacattgagta--ccccccccaaac**

30370	30380	30390	30400	30410	30420

....|....|....|....|....|....|....|....|....|....|....|....|

**Atalantia_kwangtungens** **aaaacaagcaaagggggaattttttcatctattttgtatcgttttggcggcatggccgag**

**Citrus_reticulata_1** **aaaacaagcaaagggggaattttttaatctattttgtatcgttttggcggcatggccgag**

**Citrus_reticulata_2** **aaaacaagcaaagggggaattttttaatctattttgtatcgttttggcggcatggccgag**

**Citrus_trifoliata_3** **aaaacaagcaaagggggaattttttcatctattttgtatcgttttggcggcatggccgag**

**Citrus_aurantiifolia** **aaaacaagcaaagggggaattttttcatctattttgtatcgttttggcggcatggccgag**

**Citrus_aurantium** **aaaacaagcaaagggggaattttttcatctattttgtatcgttttggcggcatggccgag**

**Citrus_cavaleriei** **aaaacaagcaaagggggaattttttcatctattttgtatcgttttggcggcatggccgag**

**Citrus_hongheensis** **aaaacaagcaaagggggaattttttcatctattttgtatcgttttggcggcatggccgag**

**Citrus_limon** **aaaacaagcaaagggggaattttttcatctattttgtatcgttttggcggcatggccgag**

**Citrus_maxima** **aaaacaagcaaagggggaattttttcatctattttgtatcgttttggcggcatggccgag**

**Citrus_medica** **aaaacaagcaaagggggaattttttcatctattttgtatcgttttggcggcatggccgag**

**Citrus_polytrifolia** **aaaacaagcaaagggggaattttttcatctattttgtatcgttttggcggcatggccgag**

**Citrus_reticulata** **aaaacaagcaaagggggaattttttaatctattttgtatcgttttggcggcatggccgag**

**Citrus_sinensis** **aaaacaagcaaagggggaattttttcatctattttgtatcgttttggcggcatggccgag**

**Fortunella_bawangica_1** **aaaacaagcaaagggggaattttttcatctattttgtatcgttttggcggcatggccgag**

**Fortunella_bawangica_2** **aaaacaagcaaagggggaattttttcatctattttgtatcgttttggcggcatggccgag**

**Fortunella_hindsii_1** **aaaacaagcaaagggggaattttttcatctattttgtatcgttttggcggcatggccgag**

**Fortunella_hindsii_2** **aaaacaagcaaagggggaattttttcatctattttgtatcgttttggcggcatggccgag**

**Fortunella_hindsii_3** **aaaacaagcaaagggggaattttttcatctattttgtatcgttttggcggcatggccgag**

**Fortunella_japonica_1** **aaaacaagcaaagggggaattttttcatctattttgtatcgttttggcggcatggccgag**

**Fortunella_japonica_2** **aaaacaagcaaagggggaattttttcatctattttgtatcgttttggcggcatggccgag**

**Fortunella_japonica_3** **aaaacaagcaaagggggaattttttcatctattttgtatcgttttggcggcatggccgag**

**Fortunella_japonica_4** **aaaacaagcaaagggggaattttttcatctattttgtatcgttttggcggcatggccgag**

**Fortunella_japonica_5** **aaaacaagcaaagggggaattttttcatctattttgtatcgttttggcggcatggccgag**

**Fortunella_japonica_6** **aaaacaagcaaagggggaattttttcatctattttgtatcgttttggcggcatggccgag**

**Fortunella_japonica_7** **aaaacaagcaaagggggaattttttcatctattttgtatcgttttggcggcatggccgag**

**Fortunella_japonica_8** **aaaacaagcaaagggggaattttttcatctattttgtatcgttttggcggcatggccgag**

**Fortunella_japonica_9** **aaaacaagcaaagggggaattttttcatctattttgtatcgttttggcggcatggccgag**

**Fortunella_japonica_10** **aaaacaagcaaagggggaattttttcatctattttgtatcgttttggcggcatggccgag**

**Fortunella_japonica_11** **aaaacaagcaaagggggaattttttcatctattttgtatcgttttggcggcatggccgag**

**Fortunella_japonica_12** **aaaacaagcaaagggggaattttttcatctattttgtatcgttttggcggcatggccgag**

**Fortunella_margarita_1** **aaaacaagcaaagggggaattttttcatctattttgtatcgttttggcggcatggccgag**

**Fortunella_margarita_2** **aaaacaagcaaagggggaattttttcatctattttgtatcgttttggcggcatggccgag**

**Fortunella_venosa_1** **aaaacaagcaaagggggaattttttcatctattttgtatcgttttggcggcatggccgag**

**Fortunella_venosa_2** **aaaacaagcaaagggggaattttttcatctattttgtatcgttttggcggcatggccgag**

**Fortunella_venosa_3** **aaaacaagcaaagggggaattttttcatctattttgtatcgttttggcggcatggccgag**

30430	30440	30450	30460	30470	30480

....|....|....|....|....|....|....|....|....|....|....|....|

**Atalantia_kwangtungens** **cggtaaggcgggggactgcaaatcctttttccccagttcaaatctgggtgtcgcctgatc**

**Citrus_reticulata_1** **cggtaaggcgggggactgcaaatcctttttccccagttcaaatctgggtgtcgcctgatc**

**Citrus_reticulata_2** **cggtaaggcgggggactgcaaatcctttttccccagttcaaatctgggtgtcgcctgatc**

**Citrus_trifoliata_3** **cggtaaggcgggggactgcaaatcctttttccccagttcaaatctgggtgtcgcctgatc**

**Citrus_aurantiifolia** **cggtaaggcgggggactgcaaatcctttttccccagttcaaatctgggtgtcgcctgatc**

**Citrus_aurantium** **cggtaaggcgggggactgcaaatcctttttccccagttcaaatctgggtgtcgcctgatc**

**Citrus_cavaleriei** **cggtaaggcgggggactgcaaatcctttttccccagttcaaatctgggtgtcgcctgatc**

**Citrus_hongheensis** **cggtaaggcgggggactgcaaatcctttttccccagttcaaatctgggtgtcgcctgatc**

**Citrus_limon** **cggtaaggcgggggactgcaaatcctttttccccagttcaaatctgggtgtcgcctgatc**

**Citrus_maxima** **cggtaaggcgggggactgcaaatcctttttccccagttcaaatctgggtgtcgcctgatc**

**Citrus_medica** **cggtaaggcgggggactgcaaatcctttttccccagttcaaatctgggtgtcgcctgatc**

**Citrus_polytrifolia** **cggtaaggcgggggactgcaaatcctttttccccagttcaaatctgggtgtcgcctgatc**

**Citrus_reticulata** **cggtaaggcgggggactgcaaatcctttttccccagttcaaatctgggtgtcgcctgatc**

**Citrus_sinensis** **cggtaaggcgggggactgcaaatcctttttccccagttcaaatctgggtgtcgcctgatc**

**Fortunella_bawangica_1** **cggtaaggcgggggactgcaaatcctttttccccagttcaaatctgggtgtcgcctgatc**

**Fortunella_bawangica_2** **cggtaaggcgggggactgcaaatcctttttccccagttcaaatctgggtgtcgcctgatc**

**Fortunella_hindsii_1** **cggtaaggcgggggactgcaaatcctttttccccagttcaaatctgggtgtcgcctgatc**

**Fortunella_hindsii_2** **cggtaaggcgggggactgcaaatcctttttccccagttcaaatctgggtgtcgcctgatc**

**Fortunella_hindsii_3** **cggtaaggcgggggactgcaaatcctttttccccagttcaaatctgggtgtcgcctgatc**

**Fortunella_japonica_1** **cggtaaggcgggggactgcaaatcctttttccccagttcaaatctgggtgtcgcctgatc**

**Fortunella_japonica_2** **cggtaaggcgggggactgcaaatcctttttccccagttcaaatctgggtgtcgcctgatc**

**Fortunella_japonica_3** **cggtaaggcgggggactgcaaatcctttttccccagttcaaatctgggtgtcgcctgatc**

**Fortunella_japonica_4** **cggtaaggcgggggactgcaaatcctttttccccagttcaaatctgggtgtcgcctgatc**

**Fortunella_japonica_5** **cggtaaggcgggggactgcaaatcctttttccccagttcaaatctgggtgtcgcctgatc**

**Fortunella_japonica_6** **cggtaaggcgggggactgcaaatcctttttccccagttcaaatctgggtgtcgcctgatc**

**Fortunella_japonica_7** **cggtaaggcgggggactgcaaatcctttttccccagttcaaatctgggtgtcgcctgatc**

**Fortunella_japonica_8** **cggtaaggcgggggactgcaaatcctttttccccagttcaaatctgggtgtcgcctgatc**

**Fortunella_japonica_9** **cggtaaggcgggggactgcaaatcctttttccccagttcaaatctgggtgtcgcctgatc**

**Fortunella_japonica_10** **cggtaaggcgggggactgcaaatcctttttccccagttcaaatctgggtgtcgcctgatc**

**Fortunella_japonica_11** **cggtaaggcgggggactgcaaatcctttttccccagttcaaatctgggtgtcgcctgatc**

**Fortunella_japonica_12** **cggtaaggcgggggactgcaaatcctttttccccagttcaaatctgggtgtcgcctgatc**

**Fortunella_margarita_1** **cggtaaggcgggggactgcaaatcctttttccccagttcaaatctgggtgtcgcctgatc**

**Fortunella_margarita_2** **cggtaaggcgggggactgcaaatcctttttccccagttcaaatctgggtgtcgcctgatc**

**Fortunella_venosa_1** **cggtaaggcgggggactgcaaatcctttttccccagttcaaatctgggtgtcgcctgatc**

**Fortunella_venosa_2** **cggtaaggcgggggactgcaaatcctttttccccagttcaaatctgggtgtcgcctgatc**

**Fortunella_venosa_3** **cggtaaggcgggggactgcaaatcctttttccccagttcaaatctgggtgtcgcctgatc**

30490	30500	30510	30520	30530	30540

....|....|....|....|....|....|....|....|....|....|....|....|

**Atalantia_kwangtungens** **aacaaacgataagactcgcaatcccttattctgcttggctggtctaactcgccgaatc-t**

**Citrus_reticulata_1** **aacaaacgataagactcgcaatcccttattctgcttggctggtctaactcgccgaatctt**

**Citrus_reticulata_2** **aacaaacgataagactcgcaatcccttattctgcttggctggtctaactcgccgaatctt**

**Citrus_trifoliata_3** **aacaaacgataagactcgcaatcccttattctgcttggctggtctaactcgccgaatctt**

**Citrus_aurantiifolia** **aacaaacgataagactcgcaatcccttattctgcttggctggtctaactcgccgaatctt**

**Citrus_aurantium** **aacaaacgataagactcgcaatcccttattctgcttggctggtctaactcgccgaatctt**

**Citrus_cavaleriei** **aacaaacgataagactcgcaatcccttattctgcttggctggtctaactcgccgaatctt**

**Citrus_hongheensis** **aacaaacgataagactcgcaatcccttattctgcttggctggtctaactcgccgaatctt**

**Citrus_limon** **aacaaacgataagactcgcaatcccttattctgcttggctggtctaactcgccgaatctt**

**Citrus_maxima** **aacaaacgataagactcgcaatcccttattctgcttggctggtctaactcgccgaatctt**

**Citrus_medica** **aacaaacgataagactcgcaatcccttattctgcttggctggtctaactcgccgaatctt**

**Citrus_polytrifolia** **aacaaacgataagactcgcaatcccttattctgcttggctggtctaactcgccgaatctt**

**Citrus_reticulata** **aacaaacgataagactcgcaatcccttattctgcttggctggtctaactcgccgaatctt**

**Citrus_sinensis** **aacaaacgataagactcgcaatcccttattctgcttggctggtctaactcgccgaatctt**

**Fortunella_bawangica_1** **aacaaacgataagactcgcaatcccttattctgcttggctgggctaactcgccgaatctt**

**Fortunella_bawangica_2** **aacaaacgataagactcgcaatcccttattctgcttggctgggctaactcgccgaatctt**

**Fortunella_hindsii_1** **aacaaacgataagactcgcaatcccttattctgcttggctgggctaactcgccgaatctt**

**Fortunella_hindsii_2** **aacaaacgataagactcgcaatcccttattctgcttggctgggctaactcgccgaatctt**

**Fortunella_hindsii_3** **aacaaacgataagactcgcaatcccttattctgcttggctgggctaactcgccgaatctt**

**Fortunella_japonica_1** **aacaaacgataagactcgcaatcccttattctgcttggctgggctaactcgccgaatctt**

**Fortunella_japonica_2** **aacaaacgataagactcgcaatcccttattctgcttggctgggctaactcgccgaatctt**

**Fortunella_japonica_3** **aacaaacgataagactcgcaatcccttattctgcttggctgggctaactcgccgaatctt**

**Fortunella_japonica_4** **aacaaacgataagactcgcaatcccttattctgcttggctgggctaactcgccgaatctt**

**Fortunella_japonica_5** **aacaaacgataagactcgcaatcccttattctgcttggctgggctaactcgccgaatctt**

**Fortunella_japonica_6** **aacaaacgataagactcgcaatcccttattctgcttggctgggctaactcgccgaatctt**

**Fortunella_japonica_7** **aacaaacgataagactcgcaatcccttattctgcttggctgggctaactcgccgaatctt**

**Fortunella_japonica_8** **aacaaacgataagactcgcaatcccttattctgcttggctgggctaactcgccgaatctt**

**Fortunella_japonica_9** **aacaaacgataagactcgcaatcccttattctgcttggctgggctaactcgccgaatctt**

**Fortunella_japonica_10** **aacaaacgataagactcgcaatcccttattctgcttggctgggctaactcgccgaatctt**

**Fortunella_japonica_11** **aacaaacgataagactcgcaatcccttattctgcttggctgggctaactcgccgaatctt**

**Fortunella_japonica_12** **aacaaacgataagactcgcaatcccttattctgcttggctgggctaactcgccgaatctt**

**Fortunella_margarita_1** **aacaaacgataagactcgcaatcccttattctgcttggctgggctaactcgccgaatctt**

**Fortunella_margarita_2** **aacaaacgataagactcgcaatcccttattctgcttggctgggctaactcgccgaatctt**

**Fortunella_venosa_1** **aacaaacgataagactcgcaatcccttattctgcttggctgggctaactcgccgaatctt**

**Fortunella_venosa_2** **aacaaacgataagactcgcaatcccttattctgcttggctgggctaactcgccgaatctt**

**Fortunella_venosa_3** **aacaaacgataagactcgcaatcccttattctgcttggctgggctaactcgccgaatctt**

30550	30560	30570	30580	30590	30600

....|....|....|....|....|....|....|....|....|....|....|....|

**Atalantia_kwangtungens** **ttccagcaaagtacgcgactcttgacactttcgtgattcgaaatagctggggtttctgtt**

**Citrus_reticulata_1** **ttccagcaaagtacgcgactcttgacattttcgtgattcgaaatagctggggtttctgtt**

**Citrus_reticulata_2** **ttccagcaaagtacgcgactcttgacattttcgtgattcgaaatagctggggtttctgtt**

**Citrus_trifoliata_3** **ttacagcaaagtacgcgactcttgacattttcgtgattcgaaatagctggggtttctgtt**

**Citrus_aurantiifolia** **ttccagcaaagtacgcgactcttgacattttcgtgattcgaaatagctggggtttctgtt**

**Citrus_aurantium** **ttccagcaaagtacgcgactcttgacattttcgtgattcgaaatagctggggtttctgtt**

**Citrus_cavaleriei** **ttccagcaaagtacgcgactcttgacattttcgtgattcgaaatagctggggtttctgtt**

**Citrus_hongheensis** **ttccagcaaagtacgcgactcttgacattttcgtgattcgaaatagctggggtttctgtt**

**Citrus_limon** **ttccagcaaagtacgcgactcttgacattttcgtgattcgaaatagctggggtttctgtt**

**Citrus_maxima** **ttccagcaaagtacgcgactcttgacattttcgtgattcgaaatagctggggtttctgtt**

**Citrus_medica** **ttccagcaaagtacgcgactcttgaaattttcgtgattcgaaatagctggggtttctgtt**

**Citrus_polytrifolia** **ttacagcaaagtacgcgactcttgacattttcgtgattcgaaatagctggggtttctgtt**

**Citrus_reticulata** **ttccagcaaagtacgcgactcttgacattttcgtgattcgaaatagctggggtttctgtt**

**Citrus_sinensis** **ttccagcaaagtacgcgactcttgacattttcgtgattcgaaatagctggggtttctgtt**

**Fortunella_bawangica_1** **ttccagcaaagtacgcgactcttgacattttcgtgattcgaaatagctggggtttctgtt**

**Fortunella_bawangica_2** **ttccagcaaagtacgcgactcttgacattttcgtgattcgaaatagctggggtttctgtt**

**Fortunella_hindsii_1** **ttccagcaaagtacgcgactcttgacattttcgtgattcgaaatagctggggtttctgtt**

**Fortunella_hindsii_2** **ttccagcaaagtacgcgactcttgacattttcgtgattcgaaatagctggggtttctgtt**

**Fortunella_hindsii_3** **ttccagcaaagtacgcgactcttgacattttcgtgattcgaaatagctggggtttctgtt**

**Fortunella_japonica_1** **ttccagcaaagtacgcgactcttgacattttcgtgattcgaaatagctggggtttctgtt**

**Fortunella_japonica_2** **ttccagcaaagtacgcgactcttgacattttcgtgattcgaaatagctggggtttctgtt**

**Fortunella_japonica_3** **ttccagcaaagtacgcgactcttgacattttcgtgattcgaaatagctggggtttctgtt**

**Fortunella_japonica_4** **ttccagcaaagtacgcgactcttgacattttcgtgattcgaaatagctggggtttctgtt**

**Fortunella_japonica_5** **ttccagcaaagtacgcgactcttgacattttcgtgattcgaaatagctggggtttctgtt**

**Fortunella_japonica_6** **ttccagcaaagtacgcgactcttgacattttcgtgattcgaaatagctggggtttctgtt**

**Fortunella_japonica_7** **ttccagcaaagtacgcgactcttgacattttcgtgattcgaaatagctggggtttctgtt**

**Fortunella_japonica_8** **ttccagcaaagtacgcgactcttgacattttcgtgattcgaaatagctggggtttctgtt**

**Fortunella_japonica_9** **ttccagcaaagtacgcgactcttgacattttcgtgattcgaaatagctggggtttctgtt**

**Fortunella_japonica_10** **ttccagcaaagtacgcgactcttgacattttcgtgattcgaaatagctggggtttctgtt**

**Fortunella_japonica_11** **ttccagcaaagtacgcgactcttgacattttcgtgattcgaaatagctggggtttctgtt**

**Fortunella_japonica_12** **ttccagcaaagtacgcgactcttgacattttcgtgattcgaaatagctggggtttctgtt**

**Fortunella_margarita_1** **ttccagcaaagtacgcgactcttgacattttcgtgattcgaaatagctggggtttctgtt**

**Fortunella_margarita_2** **ttccagcaaagtacgcgactcttgacattttcgtgattcgaaatagctggggtttctgtt**

**Fortunella_venosa_1** **ttccagcaaagtacgcgactcttgacattttcgtgattcgaaatagctggggtttctgtt**

**Fortunella_venosa_2** **ttccagcaaagtacgcgactcttgacattttcgtgattcgaaatagctggggtttctgtt**

**Fortunella_venosa_3** **ttccagcaaagtacgcgactcttgacattttcgtgattcgaaatagctggggtttctgtt**

30610	30620	30630	30640	30650	30660

....|....|....|....|....|....|....|....|....|....|....|....|

**Atalantia_kwangtungens** **c----------ttttttctgttccttaaacttaa------gttaatccttgcatttagga**

**Citrus_reticulata_1** **ct----tttttttttttctgttccttaaacttaaagtgctgttaatccttgcatttagga**

**Citrus_reticulata_2** **ct----tttttttttttctgttccttaaacttaaagtgctgttaatccttgcatttagga**

**Citrus_trifoliata_3** **c------ttttttt-ttctgttccttaaacttaaagtgctgttaatccttgcatttagga**

**Citrus_aurantiifolia** **ct----tttttttttttctgttccttaaacttaaagtgctgttaatccttgcatttagga**

**Citrus_aurantium** **c-----tttttttttttctgttccttaaacttaaagtgctgttaatccttgcatttagga**

**Citrus_cavaleriei** **ct----tttttttttttctgttccttaaacttaaagtgctgttaatccttgcatttagga**

**Citrus_hongheensis** **c-----tttttttttttctgttccttaaacttaaagtgctgttaatccttgcatttagga**

**Citrus_limon** **c-----tttttttttttctgttccttaaacttaaagtgctgttaatccttgcatttagga**

**Citrus_maxima** **c------ttttttttttctgttccttaaacttaaagtgctgttaatccttgcatttagga**

**Citrus_medica** **ctttttttttttattttctgttccttaaacttaaagtgctgttaatccttgcatttagga**

**Citrus_polytrifolia** **c------ttttttttttctgttccttaaacttaaagtgctgttaatccttgcatttagga**

**Citrus_reticulata** **ct----tttttttttttctgttccttaaacttaaagtgctgttaatccttgcatttagga**

**Citrus_sinensis** **c------ttttttttttctgttccttaaacttaaagtgctgttaatccttgcatttagga**

**Fortunella_bawangica_1** **c-------tttttttttctgttccttaaacttaaagtgctgttaatccttgcatttagga**

**Fortunella_bawangica_2** **c-------tttttttttctgttccttaaacttaaagtgctgttaatccttgcatttagga**

**Fortunella_hindsii_1** **c-------tttttttttctgttccttaaacttaaagtgctgttaatccttgcatttagga**

**Fortunella_hindsii_2** **c-------tttttttttctgttccttaaacttaaagtgctgttaatccttgcatttagga**

**Fortunella_hindsii_3** **c-------tttttttttctgttccttaaacttaaagtgctgttaatccttgcatttagga**

**Fortunella_japonica_1** **c-------tttttttttctgttccttaaacttaaagtgctgttaatccttgcatttagga**

**Fortunella_japonica_2** **c-------tttttttttctgttccttaaacttaaagtgctgttaatccttgcatttagga**

**Fortunella_japonica_3** **c-------tttttttttctgttccttaaacttaaagtgctgttaatccttgcatttagga**

**Fortunella_japonica_4** **c-------tttttttttctgttccttaaacttaaagtgctgttaatccttgcatttagga**

**Fortunella_japonica_5** **c-------tttttttttctgttccttaaacttaaagtgctgttaatccttgcatttagga**

**Fortunella_japonica_6** **c-------tttttttttctgttccttaaacttaaagtgctgttaatccttgcatttagga**

**Fortunella_japonica_7** **c-------tttttttttctgttccttaaacttaaagtgctgttaatccttgcatttagga**

**Fortunella_japonica_8** **c-------tttttttttctgttccttaaacttaaagtgctgttaatccttgcatttagga**

**Fortunella_japonica_9** **c-------tttttttttctgttccttaaacttaaagtgctgttaatccttgcatttagga**

**Fortunella_japonica_10** **c-------tttttttttctgttccttaaacttaaagtgctgttaatccttgcatttagga**

**Fortunella_japonica_11** **c-------tttttttttctgttccttaaacttaaagtgctgttaatccttgcatttagga**

**Fortunella_japonica_12** **c-------tttttttttctgttccttaaacttaaagtgctgttaatccttgcatttagga**

**Fortunella_margarita_1** **c-------tttttttttctgttccttaaacttaaagtgctgttaatccttgcatttagga**

**Fortunella_margarita_2** **c-------tttttttttctgttccttaaacttaaagtgctgttaatccttgcatttagga**

**Fortunella_venosa_1** **c-------tttttttttctgttccttaaacttaaagtgctgttaatccttgcatttagga**

**Fortunella_venosa_2** **c-------tttttttttctgttccttaaacttaaagtgctgttaatccttgcatttagga**

**Fortunella_venosa_3** **c-------tttttttttctgttccttaaacttaaagtgctgttaatccttgcatttagga**

30670	30680	30690	30700	30710	30720

....|....|....|....|....|....|....|....|....|....|....|....|

**Atalantia_kwangtungens** **ttcacag-------aaagatta---tagtagaagcgctttcatatcaaatcaagagttac**

**Citrus_reticulata_1** **ttcacgg-------aaagattatagtagtagaagcgctttcatatcaaatcaagagttac**

**Citrus_reticulata_2** **ttcacgg-------aaagattatagtagtagaagcgctttcatatcaaatcaagagttac**

**Citrus_trifoliata_3** **ttcacgg-------aaagattatagtagtagaagcgctttcatatcaaatcaagagttat**

**Citrus_aurantiifolia** **ttcacggaaagattaaagattatagtagtagaagcgctttcatatcaaatcaagagttac**

**Citrus_aurantium** **ttcacgg-------aaagattatagtagtagaagcgctttcatatcaaatcaagagttac**

**Citrus_cavaleriei** **ttcacgg-------aaagattatagtagtagaagcgctttcatatcaaatcaagagttac**

**Citrus_hongheensis** **ttcacgg-------aaagattctagtagtagaagcgctttcatatcaaatcaagagttac**

**Citrus_limon** **ttcacgg-------aaagattatagtagtagaagcgctttcatatcaaatcaagagttac**

**Citrus_maxima** **ttcacgg-------aaagattatagtagtagaagcgctttcatatcaaatcaagagttac**

**Citrus_medica** **ttcacgg-------aaagattatagtagtagaagcgctttcatatcaaatcaagagttac**

**Citrus_polytrifolia** **ttcacgg-------aaagattatagtagtagaagcgctttcatatcaaatcaagagttac**

**Citrus_reticulata** **ttcacgg-------aaagattatagtagtagaagcgctttcatatcaaatcaagagttac**

**Citrus_sinensis** **ttcacgg-------aaagattatagtagtagaagcgctttcatatcaaatcaagagttac**

**Fortunella_bawangica_1** **ttcacgg-------aaagattatagtagtagaagcgctttcatatcaaatcaagagttac**

**Fortunella_bawangica_2** **ttcacgg-------aaagattatagtagtagaagcgctttcatatcaaatcaagagttac**

**Fortunella_hindsii_1** **ttcacgg-------aaagattatagtagtagaagcgctttcatatcaaatcaagagttac**

**Fortunella_hindsii_2** **ttcacgg-------aaagattatagtagtagaagcgctttcatatcaaatcaagagttac**

**Fortunella_hindsii_3** **ttcacgg-------aaagattatagtagtagaagcgctttcatatcaaatcaagagttac**

**Fortunella_japonica_1** **ttcacgg-------aaagattatagtagtagaagcgctttcatatcaaatcaagagttac**

**Fortunella_japonica_2** **ttcacgg-------aaagattatagtagtagaagcgctttcatatcaaatcaagagttac**

**Fortunella_japonica_3** **ttcacgg-------aaagattatagtagtagaagcgctttcatatcaaatcaagagttac**

**Fortunella_japonica_4** **ttcacgg-------aaagattatagtagtagaagcgctttcatatcaaatcaagagttac**

**Fortunella_japonica_5** **ttcacgg-------aaagattatagtagtagaagcgctttcatatcaaatcaagagttac**

**Fortunella_japonica_6** **ttcacgg-------aaagattatagtagtagaagcgctttcatatcaaatcaagagttac**

**Fortunella_japonica_7** **ttcacgg-------aaagattatagtagtagaagcgctttcatatcaaatcaagagttac**

**Fortunella_japonica_8** **ttcacgg-------aaagattatagtagtagaagcgctttcatatcaaatcaagagttac**

**Fortunella_japonica_9** **ttcacgg-------aaagattatagtagtagaagcgctttcatatcaaatcaagagttac**

**Fortunella_japonica_10** **ttcacgg-------aaagattatagtagtagaagcgctttcatatcaaatcaagagttac**

**Fortunella_japonica_11** **ttcacgg-------aaagattatagtagtagaagcgctttcatatcaaatcaagagttac**

**Fortunella_japonica_12** **ttcacgg-------aaagattatagtagtagaagcgctttcatatcaaatcaagagttac**

**Fortunella_margarita_1** **ttcacgg-------aaagattatagtagtagaagcgctttcatatcaaatcaagagttac**

**Fortunella_margarita_2** **ttcacgg-------aaagattatagtagtagaagcgctttcatatcaaatcaagagttac**

**Fortunella_venosa_1** **ttcacgg-------aaagattatagtagtagaagcgctttcatatcaaatcaagagttac**

**Fortunella_venosa_2** **ttcacgg-------aaagattatagtagtagaagcgctttcatatcaaatcaagagttac**

**Fortunella_venosa_3** **ttcacgg-------aaagattatagtagtagaagcgctttcatatcaaatcaagagttac**

30730	30740	30750	30760	30770	30780

....|....|....|....|....|....|....|....|....|....|....|....|

**Atalantia_kwangtungens** **cgatttatttccactgttaattgctaatgcagggtctactgaccgggtcttgaattagat**

**Citrus_reticulata_1** **cgatttatttccactgctaattgctaatgcagggtctactgaccgggtcttgaattagat**

**Citrus_reticulata_2** **cgatttatttccactgctaattgctaatgcagggtctactgaccgggtcttgaattagat**

**Citrus_trifoliata_3** **cgatttatttccactgctaattgctaatgcagggtctactgaccgggtcttgaattagat**

**Citrus_aurantiifolia** **cgatttatttccactgctaattgctaatgcagggtctactgaccgggtcttgaattagat**

**Citrus_aurantium** **cgatttatttccactgctaattgctaatgcagggtctactgaccgggtcttgaattagat**

**Citrus_cavaleriei** **cgatttatttccactgctaattgctaatgcagggtctactgaccgggtcttgaattagat**

**Citrus_hongheensis** **cgatttatttccactgctaattgctaatgcagggtctactgaccgtgtcttgaattagat**

**Citrus_limon** **cgatttatttccactgctaattgctaatgcagggtctactgaccgggtcttgaattagat**

**Citrus_maxima** **cgatttatttccactgctaattgctaatgcagggtctactgaccgggtcttgaattagat**

**Citrus_medica** **cgatttatttccactgctaattgctaatgcagggtctactgaccgggtcttgaattagat**

**Citrus_polytrifolia** **cgatttatttccactgctaattgctaatgcagggtctactgaccgggtcttgaattagat**

**Citrus_reticulata** **cgatttatttccactgctaattgctaatgcagggtctactgaccgggtcttgaattagat**

**Citrus_sinensis** **cgatttatttccactgctaattgctaatgcagggtctactgaccgggtcttgaattagat**

**Fortunella_bawangica_1** **cgatttatttccactgctaattgctaatgcagggtctactgaccgggtcttgaattagat**

**Fortunella_bawangica_2** **cgatttatttccactgctaattgctaatgcagggtctactgaccgggtcttgaattagat**

**Fortunella_hindsii_1** **cgatttatttccactgctaattgctaatgcagggtctactgaccgggtcttgaattagat**

**Fortunella_hindsii_2** **cgatttatttccactgctaattgctaatgcagggtctactgaccgggtcttgaattagat**

**Fortunella_hindsii_3** **cgatttatttccactgctaattgctaatgcagggtctactgaccgggtcttgaattagat**

**Fortunella_japonica_1** **cgatttatttccactgctaattgctaatgcagggtctactgaccgggtcttgaattagat**

**Fortunella_japonica_2** **cgatttatttccactgctaattgctaatgcagggtctactgaccgggtcttgaattagat**

**Fortunella_japonica_3** **cgatttatttccactgctaattgctaatgcagggtctactgaccgggtcttgaattagat**

**Fortunella_japonica_4** **cgatttatttccactgctaattgctaatgcagggtctactgaccgggtcttgaattagat**

**Fortunella_japonica_5** **cgatttatttccactgctaattgctaatgcagggtctactgaccgggtcttgaattagat**

**Fortunella_japonica_6** **cgatttatttccactgctaattgctaatgcagggtctactgaccgggtcttgaattagat**

**Fortunella_japonica_7** **cgatttatttccactgctaattgctaatgcagggtctactgaccgggtcttgaattagat**

**Fortunella_japonica_8** **cgatttatttccactgctaattgctaatgcagggtctactgaccgggtcttgaattagat**

**Fortunella_japonica_9** **cgatttatttccactgctaattgctaatgcagggtctactgaccgggtcttgaattagat**

**Fortunella_japonica_10** **cgatttatttccactgctaattgctaatgcagggtctactgaccgggtcttgaattagat**

**Fortunella_japonica_11** **cgatttatttccactgctaattgctaatgcagggtctactgaccgggtcttgaattagat**

**Fortunella_japonica_12** **cgatttatttccactgctaattgctaatgcagggtctactgaccgggtcttgaattagat**

**Fortunella_margarita_1** **cgatttatttccactgctaattgctaatgcagggtctactgaccgggtcttgaattagat**

**Fortunella_margarita_2** **cgatttatttccactgctaattgctaatgcagggtctactgaccgggtcttgaattagat**

**Fortunella_venosa_1** **cgatttatttccactgctaattgctaatgcagggtctactgaccgggtcttgaattagat**

**Fortunella_venosa_2** **cgatttatttccactgctaattgctaatgcagggtctactgaccgggtcttgaattagat**

**Fortunella_venosa_3** **cgatttatttccactgctaattgctaatgcagggtctactgaccgggtcttgaattagat**

30790	30800	30810	30820	30830	30840

....|....|....|....|....|....|....|....|....|....|....|....|

**Atalantia_kwangtungens** **tgaaaagcccga--gggagaatcgaattaatcgttatggaaggggcgggagagactttgt**

**Citrus_reticulata_1** **tgaaaagcccga--gggagaatcgaattaatcgttatggaaggggcgggagatactttgt**

**Citrus_reticulata_2** **tgaaaagcccga--gggagaatcgaattaatcgttatggaaggggcgggagatactttgt**

**Citrus_trifoliata_3** **tgaaaagcccgaagggaagaatcgaattaatcgttatggaaggggcgggagatactttgt**

**Citrus_aurantiifolia** **tgaaaagcccga--gggagaatcgaattaatcgttatggaaggggcgggagatactttgt**

**Citrus_aurantium** **tgaaaagcccga--gggagaatcgaattaatcgttatggaaggggcgggagatactttgt**

**Citrus_cavaleriei** **tgaaaagcccga--gggagaatcgaattaatcgttatggaaggggcgggagatactttgt**

**Citrus_hongheensis** **tgaaaagcccga--gggagaatcgaattaatcgttatggaaggggcgggagatactttgt**

**Citrus_limon** **tgaaaagcccga--gggagaatcgaattaatcgttatggaaggggcgggagatactttgt**

**Citrus_maxima** **tgaaaagcccga--gggagaatcgaattaatcgttatggaaggggcgggagatactttgt**

**Citrus_medica** **tgaaaagcccga--gggagaatcgaattaatcgttatggaaggggcgggagatactttgt**

**Citrus_polytrifolia** **tgaaaagcccgaagggaagaatcgaattaatcgttatggaaggggcgggagatactttgt**

**Citrus_reticulata** **tgaaaagcccga--gggagaatcgaattaatcgttatggaaggggcgggagatactttgt**

**Citrus_sinensis** **tgaaaagcccga--gggagaatcgaattaatcgttatggaaggggcgggagatactttgt**

**Fortunella_bawangica_1** **tgaaaagcccga--gggagaatcgaattaatcgttatggaaggggcgggagatactttgt**

**Fortunella_bawangica_2** **tgaaaagcccga--gggagaatcgaattaatcgttatggaaggggcgggagatactttgt**

**Fortunella_hindsii_1** **tgaaaagcccga--gggagaatcgaattaatcgttatggaaggggcgggagatactttgt**

**Fortunella_hindsii_2** **tgaaaagcccga--gggagaatcgaattaatcgttatggaaggggcgggagatactttgt**

**Fortunella_hindsii_3** **tgaaaagcccga--gggagaatcgaattaatcgttatggaaggggcgggagatactttgt**

**Fortunella_japonica_1** **tgaaaagcccga--gggagaatcgaattaatcgttatggaaggggcgggagatactttgt**

**Fortunella_japonica_2** **tgaaaagcccga--gggagaatcgaattaatcgttatggaaggggcgggagatactttgt**

**Fortunella_japonica_3** **tgaaaagcccga--gggagaatcgaattaatcgttatggaaggggcgggagatactttgt**

**Fortunella_japonica_4** **tgaaaagcccga--gggagaatcgaattaatcgttatggaaggggcgggagatactttgt**

**Fortunella_japonica_5** **tgaaaagcccga--gggagaatcgaattaatcgttatggaaggggcgggagatactttgt**

**Fortunella_japonica_6** **tgaaaagcccga--gggagaatcgaattaatcgttatggaaggggcgggagatactttgt**

**Fortunella_japonica_7** **tgaaaagcccga--gggagaatcgaattaatcgttatggaaggggcgggagatactttgt**

**Fortunella_japonica_8** **tgaaaagcccga--gggagaatcgaattaatcgttatggaaggggcgggagatactttgt**

**Fortunella_japonica_9** **tgaaaagcccga--gggagaatcgaattaatcgttatggaaggggcgggagatactttgt**

**Fortunella_japonica_10** **tgaaaagcccga--gggagaatcgaattaatcgttatggaaggggcgggagatactttgt**

**Fortunella_japonica_11** **tgaaaagcccga--gggagaatcgaattaatcgttatggaaggggcgggagatactttgt**

**Fortunella_japonica_12** **tgaaaagcccga--gggagaatcgaattaatcgttatggaaggggcgggagatactttgt**

**Fortunella_margarita_1** **tgaaaagcccga--gggagaatcgaattaatcgttatggaaggggcgggagatactttgt**

**Fortunella_margarita_2** **tgaaaagcccga--gggagaatcgaattaatcgttatggaaggggcgggagatactttgt**

**Fortunella_venosa_1** **tgaaaagcccga--gggagaatcgaattaatcgttatggaaggggcgggagatactttgt**

**Fortunella_venosa_2** **tgaaaagcccga--gggagaatcgaattaatcgttatggaaggggcgggagatactttgt**

**Fortunella_venosa_3** **tgaaaagcccga--gggagaatcgaattaatcgttatggaaggggcgggagatactttgt**

30850	30860	30870	30880	30890	30900

....|....|....|....|....|....|....|....|....|....|....|....|

**Atalantia_kwangtungens** **atacagtatacagactcataagagtctctgagtgcacgggcattcaatcaatattcgatt**

**Citrus_reticulata_1** **atgcagtatacagactcataagagtctatgaatgcacgggcattcaatcaatattcgatt**

**Citrus_reticulata_2** **atgcagtatacagactcataagagtctatgaatgcacgggcattcaatcaatattcgatt**

**Citrus_trifoliata_3** **atgcagtatacagactcataagagtctctgaatgcacgggcattcaatcaatattcgatt**

**Citrus_aurantiifolia** **atgcagtatacagactcataagagtctctgaatgcacgggcattcaatcaatattcgatt**

**Citrus_aurantium** **atgcagtatacagactcataagagtctctgaatgcacgggcattcaatcaatattcgatt**

**Citrus_cavaleriei** **atgcagtatacagactcataagagtctctgaatgcacgggcattcaatcaatattcgatt**

**Citrus_hongheensis** **atgcagtatacagactcataagagtctctgaatgcacgggcattcaatcaatattcgatt**

**Citrus_limon** **atgcagtatacagactcataagagtctctgaatgcacgggcattcaatcaatattcgatt**

**Citrus_maxima** **atgcagtatacagactcataagagtctctgaatgcacgggcattcaatcaatattcgatt**

**Citrus_medica** **atgcagtatacagactcataagagtctctgaatgcacgggcattcaatcaatattcgatt**

**Citrus_polytrifolia** **atgcagtatacagactcataagagtctctgaatgcacgggcattcaatcaatattcgatt**

**Citrus_reticulata** **atgcagtatacagactcataagagtctatgaatgcacgggcattcaatcaatattcgatt**

**Citrus_sinensis** **atgcagtatacagactcataagagtctctgaatgcacgggcattcaatcaatattcgatt**

**Fortunella_bawangica_1** **atgcagtatacagactcataagagtctctgaatgcacgggcattcaatcaatattcgatt**

**Fortunella_bawangica_2** **atgcagtatacagactcataagagtctctgaatgcacgggcattcaatcaatattcgatt**

**Fortunella_hindsii_1** **atgcagtatacagactcataagagtctctgaatgcacgggcattcaatcaatattcgatt**

**Fortunella_hindsii_2** **atgcagtatacagactcataagagtctctgaatgcacgggcagtcaatcaatattcgatt**

**Fortunella_hindsii_3** **atgcagtatacagactcataagagtctctgaatgcacgggcattcaatcaatattcgatt**

**Fortunella_japonica_1** **atgcagtatacagactcataagagtctctgaatgcacgggcattcaatcaatattcgatt**

**Fortunella_japonica_2** **atgcagtatacagactcataagagtctctgaatgcacgggcattcaatcaatattcgatt**

**Fortunella_japonica_3** **atgcagtatacagactcataagagtctctgaatgcacgggcattcaatcaatattcgatt**

**Fortunella_japonica_4** **atgcagtatacagactcataagagtctctgaatgcacgggcattcaatcaatattcgatt**

**Fortunella_japonica_5** **atgcagtatacagactcataagagtctctgaatgcacgggcattcaatcaatattcgatt**

**Fortunella_japonica_6** **atgcagtatacagactcataagagtctctgaatgcacgggcattcaatcaatattcgatt**

**Fortunella_japonica_7** **atgcagtatacagactcataagagtctctgaatgcacgggcattcaatcaatattcgatt**

**Fortunella_japonica_8** **atgcagtatacagactcataagagtctctgaatgcacgggcattcaatcaatattcgatt**

**Fortunella_japonica_9** **atgcagtatacagactcataagagtctctgaatgcacgggcattcaatcaatattcgatt**

**Fortunella_japonica_10** **atgcagtatacagactcataagagtctctgaatgcacgggcattcaatcaatattcgatt**

**Fortunella_japonica_11** **atgcagtatacagactcataagagtctctgaatgcacgggcattcaatcaatattcgatt**

**Fortunella_japonica_12** **atgcagtatacagactcataagagtctctgaatgcacgggcattcaatcaatattcgatt**

**Fortunella_margarita_1** **atgcagtatacagactcataagagtctctgaatgcacgggcattcaatcaatattcgatt**

**Fortunella_margarita_2** **atgcagtatacagactcataagagtctctgaatgcacgggcattcaatcaatattcgatt**

**Fortunella_venosa_1** **atgcagtatacagactcataagagtctctgaatgcacgggcattcaatcaatattcgatt**

**Fortunella_venosa_2** **atgcagtatacagactcataagagtctctgaatgcacgggcattcaatcaatattcgatt**

**Fortunella_venosa_3** **atgcagtatacagactcataagagtctctgaatgcacgggcattcaatcaatattcgatt**

30910	30920	30930	30940	30950	30960

....|....|....|....|....|....|....|....|....|....|....|....|

**Atalantia_kwangtungens** **ggacggataattggcttttactt-----------aat-------gataataaagggcaac**

**Citrus_reticulata_1** **ggacggataattggcttttactt-----------aat-------gataataaagggcaac**

**Citrus_reticulata_2** **ggacggataattggcttttactt-----------aat-------gataataaagggcaac**

**Citrus_trifoliata_3** **ggacggataattggcttttactt-----------aatgataatagataataaagggcaac**

**Citrus_aurantiifolia** **ggacggataattggcttttactt-----------aat-------gataataaagggcaac**

**Citrus_aurantium** **ggacggataattggcttttactt-----------aat-------gataataaagggcaac**

**Citrus_cavaleriei** **ggacggataattggcttttacttaatgataataaaat-------gataataaagggcaac**

**Citrus_hongheensis** **ggacggataattggcttttactt-----------aat-------gataataaagggcaac**

**Citrus_limon** **ggacggataattggcttttactt-----------aat-------gataataaagggcaac**

**Citrus_maxima** **ggacggataattggcttttactt-----------aat-------gataataaagggcaac**

**Citrus_medica** **ggacggataattggcttttactt-----------aat-------gataataaagggcaac**

**Citrus_polytrifolia** **ggacggataattggcttttactt-----------aat-------gataataaagggcaac**

**Citrus_reticulata** **ggacggataattggcttttactt-----------aat-------gataataaagggcaac**

**Citrus_sinensis** **ggacggataattggcttttactt-----------aat-------gataataaagggcaac**

**Fortunella_bawangica_1** **ggacggataattggcttttactt-----------aat-------gataataaagggcaac**

**Fortunella_bawangica_2** **ggacggataattggcttttactt-----------aat-------gataataaagggcaac**

**Fortunella_hindsii_1** **ggacggataattggcttttactt-----------aat-------gataataaagggcaac**

**Fortunella_hindsii_2** **ggacggataattggcttttactt-----------aat-------gataataaagggcaac**

**Fortunella_hindsii_3** **ggacggataattggcttttactt-----------aat-------gataataaagggcaac**

**Fortunella_japonica_1** **ggacggataattggcttttactt-----------aat-------gataataaagggcaac**

**Fortunella_japonica_2** **ggacggataattggcttttactt-----------aat-------gataataaagggcaac**

**Fortunella_japonica_3** **ggacggataattggcttttactt-----------aat-------gataataaagggcaac**

**Fortunella_japonica_4** **ggacggataattggcttttactt-----------aat-------gataataaagggcaac**

**Fortunella_japonica_5** **ggacggataattggcttttactt-----------aat-------gataataaagggcaac**

**Fortunella_japonica_6** **ggacggataattggcttttactt-----------aat-------gataataaagggcaac**

**Fortunella_japonica_7** **ggacggataattggcttttactt-----------aat-------gataataaagggcaac**

**Fortunella_japonica_8** **ggacggataattggcttttactt-----------aat-------gataataaagggcaac**

**Fortunella_japonica_9** **ggacggataattggcttttactt-----------aat-------gataataaagggcaac**

**Fortunella_japonica_10** **ggacggataattggcttttactt-----------aat-------gataataaagggcaac**

**Fortunella_japonica_11** **ggacggataattggcttttactt-----------aat-------gataataaagggcaac**

**Fortunella_japonica_12** **ggacggataattggcttttactt-----------aat-------gataataaagggcaac**

**Fortunella_margarita_1** **ggacggataattggcttttactt-----------aat-------gataataaagggcaac**

**Fortunella_margarita_2** **ggacggataattggcttttactt-----------aat-------gataataaagggcaac**

**Fortunella_venosa_1** **ggacggataattggcttttactt-----------aat-------gataataaagggcaac**

**Fortunella_venosa_2** **ggacggataattggcttttactt-----------aat-------gataataaagggcaac**

**Fortunella_venosa_3** **ggacggataattggcttttactt-----------aat-------gataataaagggcaac**

30970	30980	30990	31000	31010	31020

....|....|....|....|....|....|....|....|....|....|....|....|

**Atalantia_kwangtungens** **aaaaaaaaaacgaggagttcttattattactacatattaggtaacactccctttgatact**

**Citrus_reticulata_1** **--aaaaaaaacgaggagttcttattattactacagattaggtaacactccctttgatact**

**Citrus_reticulata_2** **--aaaaaaaacgaggagttcttattattactacagattaggtaacactccctttgatact**

**Citrus_trifoliata_3** **--aaaaaaaacgaggagttcttattattactacagattaggtaacactccctttgatact**

**Citrus_aurantiifolia** **--aaaaaaaacgaggagttcttattattactacagattaggtaacactccctttgatact**

**Citrus_aurantium** **--aaaaaaaacgaggagttcttattattactacagattaggtaacactccctttgatact**

**Citrus_cavaleriei** **--aaaaaaaacgaggagttcttattattactacagattaggtaacactccctttgatact**

**Citrus_hongheensis** **--aaaaaaaacgaggagttcttattattactacagattaggtaacactccctttgatact**

**Citrus_limon** **--aaaaaaaacgaggagttcttattattactacagattaggtaacactccctttgatact**

**Citrus_maxima** **--aaaaaaaacgaggagttcttattattactacagattaggtaacactccctttgatact**

**Citrus_medica** **-aaaaaaaaacgaggagttcttattattactacagattaggtaacactccctttgatact**

**Citrus_polytrifolia** **--aaaaaaaacgaggagttcttattattactacagattaggtaacactccctttgatact**

**Citrus_reticulata** **--aaaaaaaacgaggagttcttattattactacagattaggtaacactccctttgatact**

**Citrus_sinensis** **--aaaaaaaacgaggagttcttattattactacagattaggtaacactccctttgatact**

**Fortunella_bawangica_1** **--aaaaaaaacgaggagttcttattattactacagattaggtaacactccctttgatact**

**Fortunella_bawangica_2** **--aaaaaaaacgaggagttcttattattactacagattaggtaacactccctttgatact**

**Fortunella_hindsii_1** **--aaaaaaaacgaggagttcttattattactacagattaggtaacactccctttgatact**

**Fortunella_hindsii_2** **--aaaaaaaacgaggagttcttattattactacagattaggtaacactccctttgatact**

**Fortunella_hindsii_3** **--aaaaaaaacgaggagttcttattattactacagattaggtaacactccctttgatact**

**Fortunella_japonica_1** **--aaaaaaaacgaggagttcttattattactacagattaggtaacactccctttgatact**

**Fortunella_japonica_2** **--aaaaaaaacgaggagttcttattattactacagattaggtaacactccctttgatact**

**Fortunella_japonica_3** **--aaaaaaaacgaggagttcttattattactacagattaggtaacactccctttgatact**

**Fortunella_japonica_4** **--aaaaaaaacgaggagttcttattattactacagattaggtaacactccctttgatact**

**Fortunella_japonica_5** **--aaaaaaaacgaggagttcttattattactacagattaggtaacactccctttgatact**

**Fortunella_japonica_6** **--aaaaaaaacgaggagttcttattattactacagattaggtaacactccctttgatact**

**Fortunella_japonica_7** **--aaaaaaaacgaggagttcttattattactacagattaggtaacactccctttgatact**

**Fortunella_japonica_8** **--aaaaaaaacgaggagttcttattattactacagattaggtaacactccctttgatact**

**Fortunella_japonica_9** **--aaaaaaaacgaggagttcttattattactacagattaggtaacactccctttgatact**

**Fortunella_japonica_10** **--aaaaaaaacgaggagttcttattattactacagattaggtaacactccctttgatact**

**Fortunella_japonica_11** **--aaaaaaaacgaggagttcttattattactacagattaggtaacactccctttgatact**

**Fortunella_japonica_12** **--aaaaaaaacgaggagttcttattattactacagattaggtaacactccctttgatact**

**Fortunella_margarita_1** **--aaaaaaaacgaggagttcttattattactacagattaggtaacactccctttgatact**

**Fortunella_margarita_2** **--aaaaaaaacgaggagttcttattattactacagattaggtaacactccctttgatact**

**Fortunella_venosa_1** **--aaaaaaaacgaggagttcttattattactacagattaggtaacactccctttgatact**

**Fortunella_venosa_2** **--aaaaaaaacgaggagttcttattattactacagattaggtaacactccctttgatact**

**Fortunella_venosa_3** **--aaaaaaaacgaggagttcttattattactacagattaggtaacactccctttgatact**

31030	31040	31050	31060	31070	31080

....|....|....|....|....|....|....|....|....|....|....|....|

**Atalantia_kwangtungens** **tccaaagaaaagtgcgactgtgtattttgtttca-ttttccggattctactagaaatcat**

**Citrus_reticulata_1** **tccaaagaaaagtgcgactgtgtattttgtttcatttttccggattctactagaaatcat**

**Citrus_reticulata_2** **tccaaagaaaagtgcgactgtgtattttgtttcatttttccggattctactagaaatcat**

**Citrus_trifoliata_3** **tccaaagaaaagtgcgactgtgtattttgtttcatttttccggattctactcgaaatcat**

**Citrus_aurantiifolia** **tccaaagaaaagtgcgactgtgtattttgtttcatttttccggattctactagaaatcat**

**Citrus_aurantium** **tccaaagaaaagtgcgactgtgtattttgtttcatttttccggattctactagaaatcat**

**Citrus_cavaleriei** **tccaaagaaaagtgcgactgtgtattttgtttcatttttccggattctactagaaatcat**

**Citrus_hongheensis** **tccaaagaaaagtgcgactgtgtattttgtttcatttttccggattctactagaaatcat**

**Citrus_limon** **tccaaagaaaagtgcgactgtgtattttgtttcatttttccggattctactagaaatcat**

**Citrus_maxima** **tccaaagaaaagtgcgactgtgtattttgtttcatttttccggattctactagaaatcat**

**Citrus_medica** **tccaaagaaaagtgcgactgtgtattttgtttcatttttccggattctactagaaatcat**

**Citrus_polytrifolia** **tccaaagaaaagtgcgactgtgtattttgtttcatttttccggattctactcgaaatcat**

**Citrus_reticulata** **tccaaagaaaagtgcgactgtgtattttgtttcatttttccggattctactagaaatcat**

**Citrus_sinensis** **tccaaagaaaagtgcgactgtgtattttgtttcatttttccggattctactagaaatcat**

**Fortunella_bawangica_1** **tccaaagaaaagtgcgactgtggattttgtttcatttttccggattctactagaaatcat**

**Fortunella_bawangica_2** **tccaaagaaaagtgcgactgtggattttgtttcatttttccggattctactagaaatcat**

**Fortunella_hindsii_1** **tccaaagaaaagtgcgactgtggattttgtttcatttttccggattctactagaaatcat**

**Fortunella_hindsii_2** **tccaaagaaaagtgcgactgtggattttgtttcatttttccggattctactagaaatcat**

**Fortunella_hindsii_3** **tccaaagaaaagtgcgactgtggattttgtttcatttttccggattctactagaaatcat**

**Fortunella_japonica_1** **tccaaagaaaagtgcgactgtggattttgtttcatttttccggattctactagaaatcat**

**Fortunella_japonica_2** **tccaaagaaaagtgcgactgtggattttgtttcatttttccggattctactagaaatcat**

**Fortunella_japonica_3** **tccaaagaaaagtgcgactgtggattttgtttcatttttccggattctactagaaatcat**

**Fortunella_japonica_4** **tccaaagaaaagtgcgactgtggattttgtttcatttttccggattctactagaaatcat**

**Fortunella_japonica_5** **tccaaagaaaagtgcgactgtggattttgtttcatttttccggattctactagaaatcat**

**Fortunella_japonica_6** **tccaaagaaaagtgcgactgtggattttgtttcatttttccggattctactagaaatcat**

**Fortunella_japonica_7** **tccaaagaaaagtgcgactgtggattttgtttcatttttccggattctactagaaatcat**

**Fortunella_japonica_8** **tccaaagaaaagtgcgactgtggattttgtttcatttttccggattctactagaaatcat**

**Fortunella_japonica_9** **tccaaagaaaagtgcgactgtggattttgtttcatttttccggattctactagaaatcat**

**Fortunella_japonica_10** **tccaaagaaaagtgcgactgtggattttgtttcatttttccggattctactagaaatcat**

**Fortunella_japonica_11** **tccaaagaaaagtgcgactgtggattttgtttcatttttccggattctactagaaatcat**

**Fortunella_japonica_12** **tccaaagaaaagtgcgactgtggattttgtttcatttttccggattctactagaaatcat**

**Fortunella_margarita_1** **tccaaagaaaagtgcgactgtggattttgtttcatttttccggattctactagaaatcat**

**Fortunella_margarita_2** **tccaaagaaaagtgcgactgtggattttgtttcatttttccggattctactagaaatcat**

**Fortunella_venosa_1** **tccaaagaaaagtgcgactgtggattttgtttcatttttccggattctactagaaatcat**

**Fortunella_venosa_2** **tccaaagaaaagtgcgactgtggattttgtttcatttttccggattctactagaaatcat**

**Fortunella_venosa_3** **tccaaagaaaagtgcgactgtggattttgtttcatttttccggattctactagaaatcat**

31090	31100	31110	31120	31130	31140

....|....|....|....|....|....|....|....|....|....|....|....|

**Atalantia_kwangtungens** **atacgaacttgttctaagtggatttattggagcgtttcatatttagtggagtctttcatc**

**Citrus_reticulata_1** **atacgaacttgttctaagtggatttattggagcgtttcatatttagtggagtctttcatc**

**Citrus_reticulata_2** **atacgaacttgttctaagtggatttattggagcgtttcatatttagtggagtctttcatc**

**Citrus_trifoliata_3** **atacgaacttgttctaagtggagttattggagcgtttcatatttagtggagtctttcatc**

**Citrus_aurantiifolia** **atacgaacttgttctaagtggatttattggagcgtttcatatttagtggagtctttcatc**

**Citrus_aurantium** **atacgaacttgttctaagtggatttattggagcgtttcatatttagtggagtctttcatc**

**Citrus_cavaleriei** **atacgaacttgttctaagtggatttattggagcgtttcatatttagtggagtctttcatc**

**Citrus_hongheensis** **atacgaacttgttctaagtggatttattggagcgtttcatatttagtggagtctttcatc**

**Citrus_limon** **atacgaacttgttctaagtggatttattggagcgtttcatatttagtggagtctttcatc**

**Citrus_maxima** **atacgaacttgttctaagtggatttattggagcgtttcatatttagtggagtctttcatc**

**Citrus_medica** **atacgaacttgttctaagtggatttattggagcgtttcatatttagtggagtctttcatc**

**Citrus_polytrifolia** **atacgaacttgttctaagtggagttattggagcgtttcatatttagtggagtctttcatc**

**Citrus_reticulata** **atacgaacttgttctaagtggatttattggagcgtttcatatttagtggagtctttcatc**

**Citrus_sinensis** **atacgaacttgttctaagtggatttattggagcgtttcatatttagtggagtctttcatc**

**Fortunella_bawangica_1** **atacgaacttgttctaagtggatttattggagcgtttcatatttagtggagtctttcatc**

**Fortunella_bawangica_2** **atacgaacttgttctaagtggatttattggagcgtttcatatttagtggagtctttcatc**

**Fortunella_hindsii_1** **atacgaacttgttctaagtggatttattggagcgtttcatatttagtggagtctttcatc**

**Fortunella_hindsii_2** **atacgaacttgttctaagtggatttattggagcgtttcatatttagtggagtctttcatc**

**Fortunella_hindsii_3** **atacgaacttgttctaagtggatttattggagcgtttcatatttagtggagtctttcatc**

**Fortunella_japonica_1** **atacgaacttgttctaagtggatttattggagcgtttcatatttagtggagtctttcatc**

**Fortunella_japonica_2** **atacgaacttgttctaagtggatttattggagcgtttcatatttagtggagtctttcatc**

**Fortunella_japonica_3** **atacgaacttgttctaagtggatttattggagcgtttcatatttagtggagtctttcatc**

**Fortunella_japonica_4** **atacgaacttgttctaagtggatttattggagcgtttcatatttagtggagtctttcatc**

**Fortunella_japonica_5** **atacgaacttgttctaagtggatttattggagcgtttcatatttagtggagtctttcatc**

**Fortunella_japonica_6** **atacgaacttgttctaagtggatttattggagcgtttcatatttagtggagtctttcatc**

**Fortunella_japonica_7** **atacgaacttgttctaagtggatttattggagcgtttcatatttagtggagtctttcatc**

**Fortunella_japonica_8** **atacgaacttgttctaagtggatttattggagcgtttcatatttagtggagtctttcatc**

**Fortunella_japonica_9** **atacgaacttgttctaagtggatttattggagcgtttcatatttagtggagtctttcatc**

**Fortunella_japonica_10** **atacgaacttgttctaagtggatttattggagcgtttcatatttagtggagtctttcatc**

**Fortunella_japonica_11** **atacgaacttgttctaagtggatttattggagcgtttcatatttagtggagtctttcatc**

**Fortunella_japonica_12** **atacgaacttgttctaagtggatttattggagcgtttcatatttagtggagtctttcatc**

**Fortunella_margarita_1** **atacgaacttgttctaagtggatttattggagcgtttcatatttagtggagtctttcatc**

**Fortunella_margarita_2** **atacgaacttgttctaagtggatttattggagcgtttcatatttagtggagtctttcatc**

**Fortunella_venosa_1** **atacgaacttgttctaagtggatttattggagcgtttcatatttagtggagtctttcatc**

**Fortunella_venosa_2** **atacgaacttgttctaagtggatttattggagcgtttcatatttagtggagtctttcatc**

**Fortunella_venosa_3** **atacgaacttgttctaagtggatttattggagcgtttcatatttagtggagtctttcatc**

31150	31160	31170	31180	31190	31200

....|....|....|....|....|....|....|....|....|....|....|....|

**Atalantia_kwangtungens** **aagggcgttgggtcagaatccctttttgactctgcgccagtgattccactattattagga**

**Citrus_reticulata_1** **aagggcgttgggccagaatccctttttgactctgcgccagtgattccactattattagga**

**Citrus_reticulata_2** **aagggcgttgggccagaatccctttttgactctgcgccagtgattccactattattagga**

**Citrus_trifoliata_3** **aagggcgttgggccagaatccctttttgactctgcgccagtgattccactattattagga**

**Citrus_aurantiifolia** **aagggcgttgggccagaatccctttttgactctgcgccagtgattccactattattagga**

**Citrus_aurantium** **aagggcgttgggccagaatccctttttgactctgcgccagtgattccactattattagga**

**Citrus_cavaleriei** **aagggcgttgggccagaatccctttttgactctgcgccagtgattccactattattagga**

**Citrus_hongheensis** **aagggcgttgggccagaatccctttttgactctgcgccagtgattccactattattagga**

**Citrus_limon** **aagggcgttgggccagaatccctttttgactctgcgccagtgattccactattattagga**

**Citrus_maxima** **aagggcgttgggccagaatccctttttgactctgcgccagtgattccactattattagga**

**Citrus_medica** **aagggcgttgggccagaatccctttttgactctgcgccagtgattccactattattagga**

**Citrus_polytrifolia** **aagggcgttgggccagaatccctttttgactctgcgccagtgattccactattattagga**

**Citrus_reticulata** **aagggcgttgggccagaatccctttttgactctgcgccagtgattccactattattagga**

**Citrus_sinensis** **aagggcgttgggccagaatccctttttgactctgcgccagtgattccactattattagga**

**Fortunella_bawangica_1** **aagggcgttgggccagaatccctttttgactctgcgccagtgattccactattattagga**

**Fortunella_bawangica_2** **aagggcgttgggccagaatccctttttgactctgcgccagtgattccactattattagga**

**Fortunella_hindsii_1** **aagggcgttgggccagaatccctttttgactctgcgccagtgattccactattattagga**

**Fortunella_hindsii_2** **aagggcgttgggccagaatccctttttgactctgcgccagtgattccactattattagga**

**Fortunella_hindsii_3** **aagggcgttgggccagaatccctttttgactctgcgccagtgattccactattattagga**

**Fortunella_japonica_1** **aagggcgttgggccagaatccctttttgactctgcgccagtgattccactattattagga**

**Fortunella_japonica_2** **aagggcgttgggccagaatccctttttgactctgcgccagtgattccactattattagga**

**Fortunella_japonica_3** **aagggcgttgggccagaatccctttttgactctgcgccagtgattccactattattagga**

**Fortunella_japonica_4** **aagggcgttgggccagaatccctttttgactctgcgccagtgattccactattattagga**

**Fortunella_japonica_5** **aagggcgttgggccagaatccctttttgactctgcgccagtgattccactattattagga**

**Fortunella_japonica_6** **aagggcgttgggccagaatccctttttgactctgcgccagtgattccactattattagga**

**Fortunella_japonica_7** **aagggcgttgggccagaatccctttttgactctgcgccagtgattccactattattagga**

**Fortunella_japonica_8** **aagggcgttgggccagaatccctttttgactctgcgccagtgattccactattattagga**

**Fortunella_japonica_9** **aagggcgttgggccagaatccctttttgactctgcgccagtgattccactattattagga**

**Fortunella_japonica_10** **aagggcgttgggccagaatccctttttgactctgcgccagtgattccactattattagga**

**Fortunella_japonica_11** **aagggcgttgggccagaatccctttttgactctgcgccagtgattccactattattagga**

**Fortunella_japonica_12** **aagggcgttgggccagaatccctttttgactctgcgccagtgattccactattattagga**

**Fortunella_margarita_1** **aagggcgttgggccagaatccctttttgactctgcgccagtgattccactattattagga**

**Fortunella_margarita_2** **aagggcgttgggccagaatccctttttgactctgcgccagtgattccactattattagga**

**Fortunella_venosa_1** **aagggcgttgggccagaatccctttttgactctgcgccagtgattccactattattagga**

**Fortunella_venosa_2** **aagggcgttgggccagaatccctttttgactctgcgccagtgattccactattattagga**

**Fortunella_venosa_3** **aagggcgttgggccagaatccctttttgactctgcgccagtgattccactattattagga**

31210	31220	31230	31240	31250	31260

....|....|....|....|....|....|....|....|....|....|....|....|

**Atalantia_kwangtungens** **aacaataatggaataaattcttcatattcatagagataggggacataattcacatggata**

**Citrus_reticulata_1** **aacaataatggaataaattcttcatattcatagagataggggacataattcacatggata**

**Citrus_reticulata_2** **aacaataatggaataaattcttcatattcatagagataggggacataattcacatggata**

**Citrus_trifoliata_3** **aacaataatggaagaaattcttcatattcatagagataggggacatcattcacatggata**

**Citrus_aurantiifolia** **aacaataatggaataaattcttcatattcatagagataggggacataattcacatggata**

**Citrus_aurantium** **aacaataatagaataaattcttcatattcatagagataggggacataattcacatggata**

**Citrus_cavaleriei** **aacaataatggaatcaattcttcatattcatagagataggggacataattcacatggata**

**Citrus_hongheensis** **aacaataatagaataaattcttcatattcatagagataggggacataattcacatggata**

**Citrus_limon** **aacaataatagaataaattcttcatattcatagagataggggacataattcacatggata**

**Citrus_maxima** **aacaataatagaataaattcttcatattcatagagataggggacataattcacatggata**

**Citrus_medica** **aacaataatggaatcaattcttcatattcatagagataggggacataattcacatggata**

**Citrus_polytrifolia** **aacaataatggaagaaattcttcatattcatagagataggggacatcattcacatggata**

**Citrus_reticulata** **aacaataatggaataaattcttcatattcatagagataggggacataattcacatggata**

**Citrus_sinensis** **aacaataatagaataaattcttcatattcatagagataggggacataattcacatggata**

**Fortunella_bawangica_1** **aacaataatggaataaattcttcatattcatagagataggggacataattcacatggata**

**Fortunella_bawangica_2** **aacaataatggaataaattcttcatattcatagagataggggacataattcacatggata**

**Fortunella_hindsii_1** **aacaataatggaataaattcttcatattcatagagataggggacataattcacatggata**

**Fortunella_hindsii_2** **aacaataatggaataaattcttcatattcatagagataggggacataattcacatggata**

**Fortunella_hindsii_3** **aacaataatggaataaattcttcatattcatagagataggggacataattcacatggata**

**Fortunella_japonica_1** **aacaataatggaataaattcttcatattcatagagataggggacataattcacatggata**

**Fortunella_japonica_2** **aacaataatggaataaattcttcatattcatagagataggggacataattcacatggata**

**Fortunella_japonica_3** **aacaataatggaataaattcttcatattcatagagataggggacataattcacatggata**

**Fortunella_japonica_4** **aacaataatggaataaattcttcatattcatagagataggggacataattcacatggata**

**Fortunella_japonica_5** **aacaataatggaataaattcttcatattcatagagataggggacataattcacatggata**

**Fortunella_japonica_6** **aacaataatggaataaattcttcatattcatagagataggggacataattcacatggata**

**Fortunella_japonica_7** **aacaataatggaataaattcttcatattcatagagataggggacataattcacatggata**

**Fortunella_japonica_8** **aacaataatggaataaattcttcatattcatagagataggggacataattcacatggata**

**Fortunella_japonica_9** **aacaataatggaataaattcttcatattcatagagataggggacataattcacatggata**

**Fortunella_japonica_10** **aacaataatggaataaattcttcatattcatagagataggggacataattcacatggata**

**Fortunella_japonica_11** **aacaataatggaataaattcttcatattcatagagataggggacataattcacatggata**

**Fortunella_japonica_12** **aacaataatggaataaattcttcatattcatagagataggggacataattcacatggata**

**Fortunella_margarita_1** **aacaataatggaataaattcttcatattcatagagataggggacataattcacatggata**

**Fortunella_margarita_2** **aacaataatggaataaattcttcatattcatagagataggggacataattcacatggata**

**Fortunella_venosa_1** **aacaataatggaataaattcttcatattcatagagataggggacataattcacatggata**

**Fortunella_venosa_2** **aacaataatggaataaattcttcatattcatagagataggggacataattcacatggata**

**Fortunella_venosa_3** **aacaataatggaataaattcttcatattcatagagataggggacataattcacatggata**

31270	31280	31290	31300	31310	31320

....|....|....|....|....|....|....|....|....|....|....|....|

**Atalantia_kwangtungens** **tagtaagtctcgcttgggctgctttaatggtagtctttacattttccctttcgctcgtag**

**Citrus_reticulata_1** **tagtaagtctcgcttgggctgctttaatggtagtctttacattttccctttcgctcgtag**

**Citrus_reticulata_2** **tagtaagtctcgcttgggctgctttaatggtagtctttacattttccctttcgctcgtag**

**Citrus_trifoliata_3** **tagtaagtctcgcttgggctgctttaatggtagtctttacattttccctttcgctcgtag**

**Citrus_aurantiifolia** **tagtaagtctcgcttgggctgctttaatggtagtctttacattttccctttcgctcgtag**

**Citrus_aurantium** **tagtaagtctcgcttgggctgctttaatggtagtctttacattttccctttcgctcgtag**

**Citrus_cavaleriei** **tagtaagtctcgcttgggctgctttaatggtagtctttacattttccctttcgctcgtag**

**Citrus_hongheensis** **tagtaagtctcgcttgggctgctttaatggtagtctttacattttccctttcgctcgtag**

**Citrus_limon** **tagtaagtctcgcttgggctgctttaatggtagtctttacattttccctttcgctcgtag**

**Citrus_maxima** **tagtaagtctcgcttgggctgctttaatggtagtctttacattttccctttcgctcgtag**

**Citrus_medica** **tagtaagtctcgcttgggctgctttaatggtagtctttacattttccctttcgctcgtag**

**Citrus_polytrifolia** **tagtaagtctcgcttgggctgctttaatggtagtctttacattttccctttcgctcgtag**

**Citrus_reticulata** **tagtaagtctcgcttgggctgctttaatggtagtctttacattttccctttcgctcgtag**

**Citrus_sinensis** **tagtaagtctcgcttgggctgctttaatggtagtctttacattttccctttcgctcgtag**

**Fortunella_bawangica_1** **tagtaagtctcgcttgggctgctttaatggtagtctttacattttccctttcgctcgtag**

**Fortunella_bawangica_2** **tagtaagtctcgcttgggctgctttaatggtagtctttacattttccctttcgctcgtag**

**Fortunella_hindsii_1** **tagtaagtctcgcttgggctgctttaatggtagtctttacattttccctttcgctcgtag**

**Fortunella_hindsii_2** **tagtaagtctcgcttgggctgctttaatggtagtctttacattttccctttcgctcgtag**

**Fortunella_hindsii_3** **tagtaagtctcgcttgggctgctttaatggtagtctttacattttccctttcgctcgtag**

**Fortunella_japonica_1** **tagtaagtctcgcttgggctgctttaatggtagtctttacattttccctttcgctcgtag**

**Fortunella_japonica_2** **tagtaagtctcgcttgggctgctttaatggtagtctttacattttccctttcgctcgtag**

**Fortunella_japonica_3** **tagtaagtctcgcttgggctgctttaatggtagtctttacattttccctttcgctcgtag**

**Fortunella_japonica_4** **tagtaagtctcgcttgggctgctttaatggtagtctttacattttccctttcgctcgtag**

**Fortunella_japonica_5** **tagtaagtctcgcttgggctgctttaatggtagtctttacattttccctttcgctcgtag**

**Fortunella_japonica_6** **tagtaagtctcgcttgggctgctttaatggtagtctttacattttccctttcgctcgtag**

**Fortunella_japonica_7** **tagtaagtctcgcttgggctgctttaatggtagtctttacattttccctttcgctcgtag**

**Fortunella_japonica_8** **tagtaagtctcgcttgggctgctttaatggtagtctttacattttccctttcgctcgtag**

**Fortunella_japonica_9** **tagtaagtctcgcttgggctgctttaatggtagtctttacattttccctttcgctcgtag**

**Fortunella_japonica_10** **tagtaagtctcgcttgggctgctttaatggtagtctttacattttccctttcgctcgtag**

**Fortunella_japonica_11** **tagtaagtctcgcttgggctgctttaatggtagtctttacattttccctttcgctcgtag**

**Fortunella_japonica_12** **tagtaagtctcgcttgggctgctttaatggtagtctttacattttccctttcgctcgtag**

**Fortunella_margarita_1** **tagtaagtctcgcttgggctgctttaatggtagtctttacattttccctttcgctcgtag**

**Fortunella_margarita_2** **tagtaagtctcgcttgggctgctttaatggtagtctttacattttccctttcgctcgtag**

**Fortunella_venosa_1** **tagtaagtctcgcttgggctgctttaatggtagtctttacattttccctttcgctcgtag**

**Fortunella_venosa_2** **tagtaagtctcgcttgggctgctttaatggtagtctttacattttccctttcgctcgtag**

**Fortunella_venosa_3** **tagtaagtctcgcttgggctgctttaatggtagtctttacattttccctttcgctcgtag**

31330	31340	31350	31360	31370	31380

....|....|....|....|....|....|....|....|....|....|....|....|

**Atalantia_kwangtungens** **tatggggaagaagtggactctagagatactactaattgagtt-gggaatcaaactgtatc**

**Citrus_reticulata_1** **tatggggaagaagtggactctagagatactactaattgagttggggaatcaaactgtatc**

**Citrus_reticulata_2** **tatggggaagaagtggactctagagatactactaattgagttggggaatcaaactgtatc**

**Citrus_trifoliata_3** **tatggggaagaagtggactctagagatactactaattgagttggggaatcaaactgtatc**

**Citrus_aurantiifolia** **tatggggaagaagtggactctagagatactactaattgagttggggaatcaaactgtatc**

**Citrus_aurantium** **tatggggaagaagtggactctagagatactactaattgagttggggaatcaaactgtatc**

**Citrus_cavaleriei** **tatggggaagaagtggactctagagatactactaattgagttggggaatcaaactgtatc**

**Citrus_hongheensis** **tatggggaagaagtggactctagagatactactaattgagttggggaatcaaactgtatc**

**Citrus_limon** **tatggggaagaagtggactctagagatactactaattgagttggggaatcaaactgtatc**

**Citrus_maxima** **tatggggaagaagtggactctagagatactactaattgagttggggaatcaaactgtatc**

**Citrus_medica** **tatggggaagaagtggactctagagatactactaattgagttggggaatcaaactgtatc**

**Citrus_polytrifolia** **tatggggaagaagtggactctagagatactactaattgagttggggaatcaaactgtatc**

**Citrus_reticulata** **tatggggaagaagtggactctagagatactactaattgagttggggaatcaaactgtatc**

**Citrus_sinensis** **tatggggaagaagtggactctagagatactactaattgagttggggaatcaaactgtatc**

**Fortunella_bawangica_1** **tatggggaagaagtggactctagagatactactaattgagttggggaatcaaactgtatc**

**Fortunella_bawangica_2** **tatggggaagaagtggactctagagatactactaattgagttggggaatcaaactgtatc**

**Fortunella_hindsii_1** **tatggggaagaagtggactctagagatactactaattgagttggggaatcaaactgtatc**

**Fortunella_hindsii_2** **tatggggaagaagtggactctagagatactactaattgagttggggaatcaaactgtatc**

**Fortunella_hindsii_3** **tatggggaagaagtggactctagagatactactaattgagttggggaatcaaactgtatc**

**Fortunella_japonica_1** **tatggggaagaagtggactctagagatactactaattgagttggggaatcaaactgtatc**

**Fortunella_japonica_2** **tatggggaagaagtggactctagagatactactaattgagttggggaatcaaactgtatc**

**Fortunella_japonica_3** **tatggggaagaagtggactctagagatactactaattgagttggggaatcaaactgtatc**

**Fortunella_japonica_4** **tatggggaagaagtggactctagagatactactaattgagttggggaatcaaactgtatc**

**Fortunella_japonica_5** **tatggggaagaagtggactctagagatactactaattgagttggggaatcaaactgtatc**

**Fortunella_japonica_6** **tatggggaagaagtggactctagagatactactaattgagttggggaatcaaactgtatc**

**Fortunella_japonica_7** **tatggggaagaagtggactctagagatactactaattgagttggggaatcaaactgtatc**

**Fortunella_japonica_8** **tatggggaagaagtggactctagagatactactaattgagttggggaatcaaactgtatc**

**Fortunella_japonica_9** **tatggggaagaagtggactctagagatactactaattgagttggggaatcaaactgtatc**

**Fortunella_japonica_10** **tatggggaagaagtggactctagagatactactaattgagttggggaatcaaactgtatc**

**Fortunella_japonica_11** **tatggggaagaagtggactctagagatactactaattgagttggggaatcaaactgtatc**

**Fortunella_japonica_12** **tatggggaagaagtggactctagagatactactaattgagttggggaatcaaactgtatc**

**Fortunella_margarita_1** **tatggggaagaagtggactctagagatactactaattgagttggggaatcaaactgtatc**

**Fortunella_margarita_2** **tatggggaagaagtggactctagagatactactaattgagttggggaatcaaactgtatc**

**Fortunella_venosa_1** **tatggggaagaagtggactctagagatactactaattgagttggggaatcaaactgtatc**

**Fortunella_venosa_2** **tatggggaagaagtggactctagagatactactaattgagttggggaatcaaactgtatc**

**Fortunella_venosa_3** **tatggggaagaagtggactctagagatactactaattgagttggggaatcaaactgtatc**

31390	31400	31410	31420	31430	31440

....|....|....|....|....|....|....|....|....|....|....|....|

**Atalantia_kwangtungens** **actttttttatagatcgttctgcaaagccttt---------ttaattatattaattattt**

**Citrus_reticulata_1** **actttttttatagatcgttctgcaaagccttt---------ttaattctattaattatt-**

**Citrus_reticulata_2** **actttttttatagatcgttctgcaaagccttt---------ttaattctattaattatt-**

**Citrus_trifoliata_3** **actttttttatagatcgttctgcaaagccttt---------ttaattatattaattattt**

**Citrus_aurantiifolia** **actttttttatagatcgttctgcaaagccttt---------ttaattatattaattatt-**

**Citrus_aurantium** **actttttttatagatcgttctgcaaagccttt---------ttaattatattaattatt-**

**Citrus_cavaleriei** **actttttttatagatcgttctgcaaagccttt---------ttaattctattaattatt-**

**Citrus_hongheensis** **actttttttatagatcgttctgcaaagccttt---------ttaattatattaattatt-**

**Citrus_limon** **actttttttatagatcgttctgcaaagccttt---------ttaattatattaattatt-**

**Citrus_maxima** **actttttttatagatcgttctgcaaagccttt---------ttaattatattaattatt-**

**Citrus_medica** **actttttttatagatcgttctgcaaagccttt---------ttaattatattaattatt-**

**Citrus_polytrifolia** **actttttttatagatcgttctgcaaagcctttttaattatattaattatattaattattt**

**Citrus_reticulata** **actttttttatagatcgttctgcaaagccttt---------ttaattctattaattatt-**

**Citrus_sinensis** **actttttttatagatcgttctgcaaagccttt---------ttaattatattaattatt-**

**Fortunella_bawangica_1** **actttttttatagatcgttctgcaaagccttt---------ttaattatattaattatt-**

**Fortunella_bawangica_2** **actttttttatagatcgttctgcaaagccttt---------ttaattatattaattatt-**

**Fortunella_hindsii_1** **actttttttatagatcgttctgcaaagccttt---------ttaattatattaattatt-**

**Fortunella_hindsii_2** **actttttttatagatcgttctgcaaagccttt---------ttaattatattaattatt-**

**Fortunella_hindsii_3** **actttttttatagatcgttctgcaaagccttt---------ttaattatattaattatt-**

**Fortunella_japonica_1** **actttttttatagatcgttctgcaaagccttt---------ttaattatattaattatt-**

**Fortunella_japonica_2** **actttttttatagatcgttctgcaaagccttt---------ttaattatattaattatt-**

**Fortunella_japonica_3** **actttttttatagatcgttctgcaaagccttt---------ttaattatattaattatt-**

**Fortunella_japonica_4** **actttttttatagatcgttctgcaaagccttt---------ttaattatattaattatt-**

**Fortunella_japonica_5** **actttttttatagatcgttctgcaaagccttt---------ttaattatattaattatt-**

**Fortunella_japonica_6** **actttttttatagatcgttctgcaaagccttt---------ttaattatattaattatt-**

**Fortunella_japonica_7** **actttttttatagatcgttctgcaaagccttt---------ttaattatattaattatt-**

**Fortunella_japonica_8** **actttttttatagatcgttctgcaaagccttt---------ttaattatattaattatt-**

**Fortunella_japonica_9** **actttttttatagatcgttctgcaaagccttt---------ttaattatattaattatt-**

**Fortunella_japonica_10** **actttttttatagatcgttctgcaaagccttt---------ttaattatattaattatt-**

**Fortunella_japonica_11** **actttttttatagatcgttctgcaaagccttt---------ttaattatattaattatt-**

**Fortunella_japonica_12** **actttttttatagatcgttctgcaaagccttt---------ttaattatattaattatt-**

**Fortunella_margarita_1** **actttttttatagatcgttctgcaaagccttt---------ttaattatattaattatt-**

**Fortunella_margarita_2** **actttttttatagatcgttctgcaaagccttt---------ttaattatattaattatt-**

**Fortunella_venosa_1** **actttttttatagatcgttctgcaaagccttt---------ttaattatattaattatt-**

**Fortunella_venosa_2** **actttttttatagatcgttctgcaaagccttt---------ttaattatattaattatt-**

**Fortunella_venosa_3** **actttttttatagatcgttctgcaaagccttt---------ttaattatattaattatt-**

31450	31460	31470	31480	31490	31500

....|....|....|....|....|....|....|....|....|....|....|....|

**Atalantia_kwangtungens** **aataattaatataataattaaattcagtaatttaattccatttagaatttcgaaattgaa**

**Citrus_reticulata_1** **-----------aaataa-------------------------------------------**

**Citrus_reticulata_2** **-----------aaataa-------------------------------------------**

**Citrus_trifoliata_3** **-----ttaatataataattaaattcagtaatttaat------------------------**

**Citrus_aurantiifolia** **-----------taataa-------------------------------------------**

**Citrus_aurantium** **-----------taataa-------------------------------------------**

**Citrus_cavaleriei** **-----------aaataa-------------------------------------------**

**Citrus_hongheensis** **-----------taataa-------------------------------------------**

**Citrus_limon** **-----------taataa-------------------------------------------**

**Citrus_maxima** **-----------taagaa-------------------------------------------**

**Citrus_medica** **-----------taataa-------------------------------------------**

**Citrus_polytrifolia** **-----ttaatataataattaaattcagtaatttaat------------------------**

**Citrus_reticulata** **-----------aaataa-------------------------------------------**

**Citrus_sinensis** **-----------taataa-------------------------------------------**

**Fortunella_bawangica_1** **-----------aaataa-------------------------------------------**

**Fortunella_bawangica_2** **-----------aaataa-------------------------------------------**

**Fortunella_hindsii_1** **-----------aaataa-------------------------------------------**

**Fortunella_hindsii_2** **-----------aaataa-------------------------------------------**

**Fortunella_hindsii_3** **-----------aaataa-------------------------------------------**

**Fortunella_japonica_1** **-----------aaataa-------------------------------------------**

**Fortunella_japonica_2** **-----------aaataa-------------------------------------------**

**Fortunella_japonica_3** **-----------aaataa-------------------------------------------**

**Fortunella_japonica_4** **-----------aaataa-------------------------------------------**

**Fortunella_japonica_5** **-----------aaataa-------------------------------------------**

**Fortunella_japonica_6** **-----------aaataa-------------------------------------------**

**Fortunella_japonica_7** **-----------aaataa-------------------------------------------**

**Fortunella_japonica_8** **-----------aaataa-------------------------------------------**

**Fortunella_japonica_9** **-----------aaataa-------------------------------------------**

**Fortunella_japonica_10** **-----------aaataa-------------------------------------------**

**Fortunella_japonica_11** **-----------aaataa-------------------------------------------**

**Fortunella_japonica_12** **-----------aaataa-------------------------------------------**

**Fortunella_margarita_1** **-----------aaataa-------------------------------------------**

**Fortunella_margarita_2** **-----------aaataa-------------------------------------------**

**Fortunella_venosa_1** **-----------aaataa-------------------------------------------**

**Fortunella_venosa_2** **-----------aaataa-------------------------------------------**

**Fortunella_venosa_3** **-----------aaataa-------------------------------------------**

31510	31520	31530	31540	31550	31560

....|....|....|....|....|....|....|....|....|....|....|....|

**Atalantia_kwangtungens** **ttaatcaaaagatcgttctgcaaagcctttttaattatattaattatttaataattaata**

**Citrus_reticulata_1** **------------------------------------------------------ttaata**

**Citrus_reticulata_2** **------------------------------------------------------ttaata**

**Citrus_trifoliata_3** **-----------------------------------------------------tttaata**

**Citrus_aurantiifolia** **------------------------------------------------------ttaata**

**Citrus_aurantium** **------------------------------------------------------ttaata**

**Citrus_cavaleriei** **------------------------------------------------------ttaata**

**Citrus_hongheensis** **------------------------------------------------------ttaata**

**Citrus_limon** **------------------------------------------------------ttaata**

**Citrus_maxima** **------------------------------------------------------ttaata**

**Citrus_medica** **------------------------------------------------------ttaata**

**Citrus_polytrifolia** **-----------------------------------------------------tttaata**

**Citrus_reticulata** **------------------------------------------------------ttaata**

**Citrus_sinensis** **------------------------------------------------------ttaata**

**Fortunella_bawangica_1** **------------------------------------------------------ttaata**

**Fortunella_bawangica_2** **------------------------------------------------------ttaata**

**Fortunella_hindsii_1** **------------------------------------------------------ttaata**

**Fortunella_hindsii_2** **------------------------------------------------------ttaata**

**Fortunella_hindsii_3** **------------------------------------------------------ttaata**

**Fortunella_japonica_1** **------------------------------------------------------ttaata**

**Fortunella_japonica_2** **------------------------------------------------------ttaata**

**Fortunella_japonica_3** **------------------------------------------------------ttaata**

**Fortunella_japonica_4** **------------------------------------------------------ttaata**

**Fortunella_japonica_5** **------------------------------------------------------ttaata**

**Fortunella_japonica_6** **------------------------------------------------------ttaata**

**Fortunella_japonica_7** **------------------------------------------------------ttaata**

**Fortunella_japonica_8** **------------------------------------------------------ttaata**

**Fortunella_japonica_9** **------------------------------------------------------ttaata**

**Fortunella_japonica_10** **------------------------------------------------------ttaata**

**Fortunella_japonica_11** **------------------------------------------------------ttaata**

**Fortunella_japonica_12** **------------------------------------------------------ttaata**

**Fortunella_margarita_1** **------------------------------------------------------ttaata**

**Fortunella_margarita_2** **------------------------------------------------------ttaata**

**Fortunella_venosa_1** **------------------------------------------------------ttaata**

**Fortunella_venosa_2** **------------------------------------------------------ttaata**

**Fortunella_venosa_3** **------------------------------------------------------ttaata**

31570	31580	31590	31600	31610	31620

....|....|....|....|....|....|....|....|....|....|....|....|

**Atalantia_kwangtungens** **taataattaaattcagtaatttaattccatttagaatttcgaaattgaattaatcaaaat**

**Citrus_reticulata_1** **gaataattaaattcagtaatttaattccatttagaatttcgaaattgaattaatcaaaat**

**Citrus_reticulata_2** **gaataattaaattcagtaatttaattccatttagaatttcgaaattgaattaatcaaaat**

**Citrus_trifoliata_3** **taataattaaattcagtaatttaattccatttagaatttcgaaattgaattaatcaaaat**

**Citrus_aurantiifolia** **taataattaaattcagtaatttaattccatttagaatttcgaaattgaattaatcaaaat**

**Citrus_aurantium** **taataattaaattcagtaattaaattccatttagaatttcgaaattgaattaatcaaaat**

**Citrus_cavaleriei** **gaataattaaattcagtaatttaattccatttcgaatttcgaaattgaattaatcaaaat**

**Citrus_hongheensis** **taataattaaattcagtaattaaattccatttagaatttcgaaattgaattaatcaaaat**

**Citrus_limon** **taataattaaattcagtaattaaattccatttagaatttcgaaattgaattaatcaaaat**

**Citrus_maxima** **taataattaaattcagtaattaaattccatttagaatttcgaaattgaattaatcaaaat**

**Citrus_medica** **taataattaaattcagtaatttaattccatttagaatttcgaaattgaattaatcaaaat**

**Citrus_polytrifolia** **taataattaaattcagtaatttaattccatttagaatttcgaaattgaattaatcaaaat**

**Citrus_reticulata** **gaataattaaattcagtaatttaattccatttagaatttcgaaattgaattaatcaaaat**

**Citrus_sinensis** **taataattaaattcagtaattaaattccatttagaatttcgaaattgaattaatcaaaat**

**Fortunella_bawangica_1** **taataattaaattcagtaatttaattccatttagaatttcgaaattgaattaatcaaaat**

**Fortunella_bawangica_2** **taataattaaattcagtaatttaattccatttagaatttcgaaattgaattaatcaaaat**

**Fortunella_hindsii_1** **taataattaaattcagtaatttaattccatttagaatttcgaaattgaattaatcaaaat**

**Fortunella_hindsii_2** **taataattaaattcagtaatttaattccatttagaatttcgaaattgaattaatcaaaat**

**Fortunella_hindsii_3** **taataattaaattcagtaatttaattccatttagaatttcgaaattgaattaatcaaaat**

**Fortunella_japonica_1** **taataattaaattcagtaatttaattccatttagaatttcgaaattgaattaatcaaaat**

**Fortunella_japonica_2** **taataattaaattcagtaatttaattccatttagaatttcgaaattgaattaatcaaaat**

**Fortunella_japonica_3** **taataattaaattcagtaatttaattccatttagaatttcgaaattgaattaatcaaaat**

**Fortunella_japonica_4** **taataattaaattcagtaatttaattccatttagaatttcgaaattgaattaatcaaaat**

**Fortunella_japonica_5** **taataattaaattcagtaatttaattccatttagaatttcgaaattgaattaatcaaaat**

**Fortunella_japonica_6** **taataattaaattcagtaatttaattccatttagaatttcgaaattgaattaatcaaaat**

**Fortunella_japonica_7** **taataattaaattcagtaatttaattccatttagaatttcgaaattgaattaatcaaaat**

**Fortunella_japonica_8** **taataattaaattcagtaatttaattccatttagaatttcgaaattgaattaatcaaaat**

**Fortunella_japonica_9** **taataattaaattcagtaatttaattccatttagaatttcgaaattgaattaatcaaaat**

**Fortunella_japonica_10** **taataattaaattcagtaatttaattccatttagaatttcgaaattgaattaatcaaaat**

**Fortunella_japonica_11** **taataattaaattcagtaatttaattccatttagaatttcgaaattgaattaatcaaaat**

**Fortunella_japonica_12** **taataattaaattcagtaatttaattccatttagaatttcgaaattgaattaatcaaaat**

**Fortunella_margarita_1** **taataattaaattcagtaatttaattccatttagaatttcgaaattgaattaatcaaaat**

**Fortunella_margarita_2** **taataattaaattcagtaatttaattccatttagaatttcgaaattgaattaatcaaaat**

**Fortunella_venosa_1** **taataattaaattcagtaatttaattccatttagaatttcgaaattgaattaatcaaaat**

**Fortunella_venosa_2** **taataattaaattcagtaatttaattccatttagaatttcgaaattgaattaatcaaaat**

**Fortunella_venosa_3** **taataattaaattcagtaatttaattccatttagaatttcgaaattgaattaatcaaaat**

31630	31640	31650	31660	31670	31680

....|....|....|....|....|....|....|....|....|....|....|....|

**Atalantia_kwangtungens** **atcttcatttcggaattctattggcttgtattctggcgaggtaattgtattcgattctat**

**Citrus_reticulata_1** **accttcatttcggaattctattggcttggattctggcgaggtaattgtattcgattctat**

**Citrus_reticulata_2** **accttcatttcggaattctattggcttggattctggcgaggtaattgtattcgattctat**

**Citrus_trifoliata_3** **accttcatttcggaattctattggcttggattctggcgaggtaattgtattcgattctat**

**Citrus_aurantiifolia** **accttcatttcggaattctattggcttggattctggcgaggtaattgtattcgattctat**

**Citrus_aurantium** **accttcatttcggaattctattggcttggattctggcgaggtaattgtattcgattctat**

**Citrus_cavaleriei** **accttcatttcggaattctattggcttggattctggcgaggtaattgtattcgattctat**

**Citrus_hongheensis** **accttcatttcggaattctattggcttggattctggcgaggtaattgtattcgattctat**

**Citrus_limon** **accttcatttcggaattctattggcttggattctggcgaggtaattgtattcgattctat**

**Citrus_maxima** **accttcatttcggaattctattggcttggattctggcgaggtaattgtattcgattctat**

**Citrus_medica** **accttcatttcggaattctattggcttggattctggcgaggtaattgtattcgattctat**

**Citrus_polytrifolia** **accttcatttcggaattctattggcttggattctggcgaggtaattgtattcgattctat**

**Citrus_reticulata** **accttcatttcggaattctattggcttggattctggcgaggtaattgtattcgattctat**

**Citrus_sinensis** **accttcatttcggaattctattggcttggattctggcgaggtaattgtattcgattctat**

**Fortunella_bawangica_1** **accttcatttcggaattctattggcttggattctggcgaggtaattgtattcgattctat**

**Fortunella_bawangica_2** **accttcatttcggaattctattggcttggattctggcgaggtaattgtattcgattctat**

**Fortunella_hindsii_1** **accttcatttcggaattctattggcttggattctggcgaggtaattgtattcgattctat**

**Fortunella_hindsii_2** **accttcatttcggaattctattggcttggattctggcgaggtaattgtattcgattctat**

**Fortunella_hindsii_3** **accttcatttcggaattctattggcttggattctggcgaggtaattgtattcgattctat**

**Fortunella_japonica_1** **accttcatttcggaattctattggcttggattctggcgaggtaattgtattcgattctat**

**Fortunella_japonica_2** **accttcatttcggaattctattggcttggattctggcgaggtaattgtattcgattctat**

**Fortunella_japonica_3** **accttcatttcggaattctattggcttggattctggcgaggtaattgtattcgattctat**

**Fortunella_japonica_4** **accttcatttcggaattctattggcttggattctggcgaggtaattgtattcgattctat**

**Fortunella_japonica_5** **accttcatttcggaattctattggcttggattctggcgaggtaattgtattcgattctat**

**Fortunella_japonica_6** **accttcatttcggaattctattggcttggattctggcgaggtaattgtattcgattctat**

**Fortunella_japonica_7** **accttcatttcggaattctattggcttggattctggcgaggtaattgtattcgattctat**

**Fortunella_japonica_8** **accttcatttcggaattctattggcttggattctggcgaggtaattgtattcgattctat**

**Fortunella_japonica_9** **accttcatttcggaattctattggcttggattctggcgaggtaattgtattcgattctat**

**Fortunella_japonica_10** **accttcatttcggaattctattggcttggattctggcgaggtaattgtattcgattctat**

**Fortunella_japonica_11** **accttcatttcggaattctattggcttggattctggcgaggtaattgtattcgattctat**

**Fortunella_japonica_12** **accttcatttcggaattctattggcttggattctggcgaggtaattgtattcgattctat**

**Fortunella_margarita_1** **accttcatttcggaattctattggcttggattctggcgaggtaattgtattcgattctat**

**Fortunella_margarita_2** **accttcatttcggaattctattggcttggattctggcgaggtaattgtattcgattctat**

**Fortunella_venosa_1** **accttcatttcggaattctattggcttggattctggcgaggtaattgtattcgattctat**

**Fortunella_venosa_2** **accttcatttcggaattctattggcttggattctggcgaggtaattgtattcgattctat**

**Fortunella_venosa_3** **accttcatttcggaattctattggcttggattctggcgaggtaattgtattcgattctat**

31690	31700	31710	31720	31730	31740

....|....|....|....|....|....|....|....|....|....|....|....|

**Atalantia_kwangtungens** **tatgaatagaatacaattcataatatatatgaataatgctctttcagaatccaaatcaaa**

**Citrus_reticulata_1** **tatgaatagaatacaattcataatatatatgaataatgctctttcagaatccaaatcaaa**

**Citrus_reticulata_2** **tatgaatagaatacaattcataatatatatgaataatgctctttcagaatccaaatcaaa**

**Citrus_trifoliata_3** **tatgaatagaatacaattcataatatatatgaataatgctctttcagaatccaaatcaaa**

**Citrus_aurantiifolia** **tatgaatagaatacaattcataatatatatgaataatgctctttcagaatccaaatcaaa**

**Citrus_aurantium** **tatgaatagaatacaattcataatatatatgaataatgctctttcagaatccaaatcaaa**

**Citrus_cavaleriei** **tatgaatagaatacaattcataatatatatgaataatgctctttcagaatccaaatcaaa**

**Citrus_hongheensis** **tatgaatagaatacaattcataatatatatgaataatgctctttcagaatccaaatcaaa**

**Citrus_limon** **tatgaatagaatacaattcataatatatatgaataatgctctttcagaatccaaatcaaa**

**Citrus_maxima** **tatgaatagaatacaattcataatatatatgaataatgctctttccgaatccaaatcaaa**

**Citrus_medica** **tatgactcgaatacaattcataatatatatgaataatgctctttcagaatccaaatcaaa**

**Citrus_polytrifolia** **tatgaatagaatacaattcataatatatatgaataatgctctttcagaatccaaatcaaa**

**Citrus_reticulata** **tatgaatagaatacaattcataatatatatgaataatgctctttcagaatccaaatcaaa**

**Citrus_sinensis** **tatgaatagaatacaattcataatatatatgaataatgctctttcagaatccaaatcaaa**

**Fortunella_bawangica_1** **tatgaatagaatacaattcataatatatatgaataatgctctttcagaatccaaatcaaa**

**Fortunella_bawangica_2** **tatgaatagaatacaattcataatatatatgaataatgctctttcagaatccaaatcaaa**

**Fortunella_hindsii_1** **tatgaatagaatacaattcataatatatatgaataatgctctttcagaatccaaatcaaa**

**Fortunella_hindsii_2** **tatgaatagaatacaattcataatatatatgaataatgctctttcagaatccaaatcaaa**

**Fortunella_hindsii_3** **tatgaatagaatacaattcataatatatatgaataatgctctttcagaatccaaatcaaa**

**Fortunella_japonica_1** **tatgaatagaatacaattcataatatatatgaataatgctctttcataatccaaatcaaa**

**Fortunella_japonica_2** **tatgaatagaatacaattcataatatatatgaataatgctctttcataatccaaatcaaa**

**Fortunella_japonica_3** **tatgaatagaatacaattcataatatatatgaataatgctctttcataatccaaatcaaa**

**Fortunella_japonica_4** **tatgaatagaatacaattcataatatatatgaataatgctctttcataatccaaatcaaa**

**Fortunella_japonica_5** **tatgaatagaatacaattcataatatatatgaataatgctctttcataatccaaatcaaa**

**Fortunella_japonica_6** **tatgaatagaatacaattcataatatatatgaataatgctctttcataatccaaatcaaa**

**Fortunella_japonica_7** **tatgaatagaatacaattcataatatatatgaataatgctctttcataatccaaatcaaa**

**Fortunella_japonica_8** **tatgaatagaatacaattcataatatatatgaataatgctctttcataatccaaatcaaa**

**Fortunella_japonica_9** **tatgaatagaatacaattcataatatatatgaataatgctctttcataatccaaatcaaa**

**Fortunella_japonica_10** **tatgaatagaatacaattcataatatatatgaataatgctctttcagaatccaaatcaaa**

**Fortunella_japonica_11** **tatgaatagaatacaattcataatatatatgaataatgctctttcataatccaaatcaaa**

**Fortunella_japonica_12** **tatgaatagaatacaattcataatatatatgaataatgctctttcataatccaaatcaaa**

**Fortunella_margarita_1** **tatgaatagaatacaattcataatatatatgaataatgctctttcagaatccaaatcaaa**

**Fortunella_margarita_2** **tatgaatagaatacaattcataatatatatgaataatgctctttcagaatccaaatcaaa**

**Fortunella_venosa_1** **tatgaatagaatacaattcataatatatatgaataatgctctttcagaatccaaatcaaa**

**Fortunella_venosa_2** **tatgaatagaatacaattcataatatatatgaataatgctctttcagaatccaaatcaaa**

**Fortunella_venosa_3** **tatgaatagaatacaattcataatatatatgaataatgctctttcagaatccaaatcaaa**

31750	31760	31770	31780	31790	31800

....|....|....|....|....|....|....|....|....|....|....|....|

**Atalantia_kwangtungens** **ccgatatttccaaaaagcccctatttctattttgaaaggaagggggatacagatcatagg**

**Citrus_reticulata_1** **cctatatttccaaaattcccctctttctattttgaaaggaagggggatacagatcatagg**

**Citrus_reticulata_2** **cctatatttccaaaattcccctctttctattttgaaaggaagggggatacagatcatagg**

**Citrus_trifoliata_3** **ccgctatttccaaaattcccctatttctattttgaaaggaagggggatacagatcatagg**

**Citrus_aurantiifolia** **ccgatatttccaaaattcccctatttctattttgaaaggaagggggatacagatcatagg**

**Citrus_aurantium** **ccgatatttccaaaattcccctatttctattttgaaaggaagggggatacagatcatagg**

**Citrus_cavaleriei** **ccgatatttccaaaattcccctatttctattttgaaaggaagggggatacagatcatagg**

**Citrus_hongheensis** **ccgatatttccaaaattcccctatttctattttgaaaggaagggggatacagatcatagg**

**Citrus_limon** **ccgatatttccaaaattcccctatttctattttgaaaggaagggggatacagatcatagg**

**Citrus_maxima** **ccgatatttccaaaattcccctatttctattttgaaaggaagggggatacagatcatagg**

**Citrus_medica** **ccgatatttccaaaaatcccctatttctattttgaaaggaagggggatacagatcatagg**

**Citrus_polytrifolia** **ccgctatttccaaaattcccctatttctattttgaaaggaagggggatacagatcatagg**

**Citrus_reticulata** **cctatatttccaaaattcccctctttctattttgaaaggaagggggatacagatcatagg**

**Citrus_sinensis** **ccgatatttccaaaattcccctatttctattttgaaaggaagggggatacagatcatagg**

**Fortunella_bawangica_1** **ccgatatttccaaaattcc-----ttctattttgaaaggaagggggatacagatcatagg**

**Fortunella_bawangica_2** **ccgatatttccaaaattcc-----ttctattttgaaaggaagggggatacagatcatagg**

**Fortunella_hindsii_1** **ccgatatttccaaaattcc-----ttctattttgaaaggaagggggatacagatcatagg**

**Fortunella_hindsii_2** **ccgatatttccaaaattcc-----ttctattttgaaaggaagggggatacagatcatagg**

**Fortunella_hindsii_3** **ccgatatttccaaaattcc-----ttctattttgaaaggaagggggatacagatcatagg**

**Fortunella_japonica_1** **ccgatatttccaaaattcc-----ttctattttgaaaggaagggggatacagatcatagg**

**Fortunella_japonica_2** **ccgatatttccaaaattcc-----ttctattttgaaaggaagggggatacagatcatagg**

**Fortunella_japonica_3** **ccgatatttccaaaattcc-----ttctattttgaaaggaagggggatacagatcatagg**

**Fortunella_japonica_4** **ccgatatttccaaaattcc-----ttctattttgaaaggaagggggatacagatcatagg**

**Fortunella_japonica_5** **ccgatatttccaaaattcc-----ttctattttgaaaggaagggggatacagatcatagg**

**Fortunella_japonica_6** **ccgatatttccaaaattcc-----ttctattttgaaaggaagggggatacagatcatagg**

**Fortunella_japonica_7** **ccgatatttccaaaattcc-----ttctattttgaaaggaagggggatacagatcatagg**

**Fortunella_japonica_8** **ccgatatttccaaaattcc-----ttctattttgaaaggaagggggatacagatcatagg**

**Fortunella_japonica_9** **ccgatatttccaaaattcc-----ttctattttgaaaggaagggggatacagatcatagg**

**Fortunella_japonica_10** **ccgatatttccaaaattcc-----ttctattttgaaaggaagggggatacagatcatagg**

**Fortunella_japonica_11** **ccgatatttccaaaattcc-----ttctattttgaaaggaagggggatacagatcatagg**

**Fortunella_japonica_12** **ccgatatttccaaaattcc-----ttctattttgaaaggaagggggatacagatcatagg**

**Fortunella_margarita_1** **ccgatatttccaaaattcc-----ttctattttgaaaggaagggggatacagatcatagg**

**Fortunella_margarita_2** **ccgatatttccaaaattcc-----ttctattttgaaaggaagggggatacagatcatagg**

**Fortunella_venosa_1** **ccgatatttccaaaattcc-----ttctattttgaaaggaagggggatacagatcatagg**

**Fortunella_venosa_2** **ccgatatttccaaaattcc-----ttctattttgaaaggaagggggatacagatcatagg**

**Fortunella_venosa_3** **ccgatatttccaaaattcc-----ttctattttgaaaggaagggggatacagatcatagg**

31810	31820	31830	31840	31850	31860

....|....|....|....|....|....|....|....|....|....|....|....|

**Atalantia_kwangtungens** **aattt---------cgaagtcttccgaaattttctatttggtttttctgaaccggccctt**

**Citrus_reticulata_1** **aattttattccaaccgaagtcttcctagattttctatttggtttttctgaaccggccctt**

**Citrus_reticulata_2** **aattttattccaaccgaagtcttcctagattttctatttggtttttctgaaccggccctt**

**Citrus_trifoliata_3** **aattttattccaaccgaagtcttcctagattttctatttggtttttctgaaccggccctt**

**Citrus_aurantiifolia** **aattttattccaaccgaagtcttcctagattttctatttggtttttctgaaccggccctt**

**Citrus_aurantium** **aattttattccaaccgaagtcttcctagattttctatttggtttttctgaaccggccctt**

**Citrus_cavaleriei** **aattttattccaaccgaagtcttcctagattttctatttggtttttctgaaccggccctt**

**Citrus_hongheensis** **aattttattctaaccgaagtcttcctagattttctatttggtttttctgaaccggccctt**

**Citrus_limon** **aattttattccaaccgaagtcttcctagattttctatttggtttttctgaaccggccctt**

**Citrus_maxima** **aattttattccaaccgaagtcttcctagattttctatttggtttttctgaaccggccctt**

**Citrus_medica** **aattttattccaaccgaagtcttcctagattttctatttggtttttctgaaccggccctt**

**Citrus_polytrifolia** **aattttattccaaccgaagtcttcctagattttctatttggtttttctgaaccggccctt**

**Citrus_reticulata** **aattttattccaaccgaagtcttcctagattttctatttggtttttctgaaccggccctt**

**Citrus_sinensis** **aattttattccaaccgaagtcttcctagattttctatttggtttttctgaaccggccctt**

**Fortunella_bawangica_1** **aattttattccaaccgaagtcttcctagattttctatttggtttttctgaaccggccctt**

**Fortunella_bawangica_2** **aattttattccaaccgaagtcttcctagattttctatttggtttttctgaaccggccctt**

**Fortunella_hindsii_1** **aattttattccaaccgaagtcttcctagattttctatttggtttttctgaaccggccctt**

**Fortunella_hindsii_2** **aattttattccaaccgaagtcttcctagattttctatttggtttttctgaaccggccctt**

**Fortunella_hindsii_3** **aattttattccaaccgaagtcttcctagattttctatttggtttttctgaaccggccctt**

**Fortunella_japonica_1** **aattttattccaaccgaagtcttcctagattttctatttggtttttctgaaccggccctt**

**Fortunella_japonica_2** **aattttattccaaccgaagtcttcctagattttctatttggtttttctgaaccggccctt**

**Fortunella_japonica_3** **aattttattccaaccgaagtcttcctagattttctatttggtttttctgaaccggccctt**

**Fortunella_japonica_4** **aattttattccaaccgaagtcttcctagattttctatttggtttttctgaaccggccctt**

**Fortunella_japonica_5** **aattttattccaaccgaagtcttcctagattttctatttggtttttctgaaccggccctt**

**Fortunella_japonica_6** **aattttattccaaccgaagtcttcctagattttctatttggtttttctgaaccggccctt**

**Fortunella_japonica_7** **aattttattccaaccgaagtcttcctagattttctatttggtttttctgaaccggccctt**

**Fortunella_japonica_8** **aattttattccaaccgaagtcttcctagattttctatttggtttttctgaaccggccctt**

**Fortunella_japonica_9** **aattttattccaaccgaagtcttcctagattttctatttggtttttctgaaccggccctt**

**Fortunella_japonica_10** **aattttattccaaccgaagtcttcctagattttctatttggtttttctgaaccggccctt**

**Fortunella_japonica_11** **aattttattccaaccgaagtcttcctagattttctatttggtttttctgaaccggccctt**

**Fortunella_japonica_12** **aattttattccaaccgaagtcttcctagattttctatttggtttttctgaaccggccctt**

**Fortunella_margarita_1** **aattttattccaaccgaagtcttcctagattttctatttggtttttctgaaccggccctt**

**Fortunella_margarita_2** **aattttattccaaccgaagtcttcctagattttctatttggtttttctgaaccggccctt**

**Fortunella_venosa_1** **aattttattccaaccgaagtcttcctagattttctatttggtttttctgaaccggccctt**

**Fortunella_venosa_2** **aattttattccaaccgaagtcttcctagattttctatttggtttttctgaaccggccctt**

**Fortunella_venosa_3** **aattttattccaaccgaagtcttcctagattttctatttggtttttctgaaccggccctt**

31870	31880	31890	31900	31910	31920

....|....|....|....|....|....|....|....|....|....|....|....|

**Atalantia_kwangtungens** **gccagagttctctatggacaatggggaagaacgcggaaaaccggacccc---tttttttt**

**Citrus_reticulata_1** **gccagagttctctatggacaatggggaagaacgcggaaaaccggacccccc-tttttttt**

**Citrus_reticulata_2** **gccagagttctctatggacaatggggaagaacgcggaaaaccggacccccc-tttttttt**

**Citrus_trifoliata_3** **gccagagttctctatggacaatggggaagaacgcggaaaaccggacccc-cttttttttt**

**Citrus_aurantiifolia** **gccagagttctctatggacaatggggaagaacgcgtaaaaccggacccccc-tttttttt**

**Citrus_aurantium** **gccagagttctctatggacaatggggaagaacgcggaaaaccggacccccc-tttttttt**

**Citrus_cavaleriei** **gccagagttctctatggacaatggggaagaacgcggaaaaccggaccccccttttttttt**

**Citrus_hongheensis** **gccagagttctctatggacaatggggaagaacgcggaaaaccggaccccccttttttttt**

**Citrus_limon** **gccagagttctctatggacaatggggaagaacgcggaaaaccggacccccc-tttttttt**

**Citrus_maxima** **gccagagttctctatggacaatggggaagaacgcggaaaaccggacccccc-tttttttt**

**Citrus_medica** **gccagagttctcaatggacaatggggaagaacgcggaaaaccggaccccc--tttttttt**

**Citrus_polytrifolia** **gccagagttctctatggacaatggggaagaacgcggaaaaccggaccccttttttttttt**

**Citrus_reticulata** **gccagagttctctatggacaatggggaagaacgcggaaaaccggacccccc-tttttttt**

**Citrus_sinensis** **gccagagttctctatggacaatggggaagaacgcggaaaaccggacccccc-tttttttt**

**Fortunella_bawangica_1** **gccagagttctctatggacaatgggtaagaacgcggaaaaccggacccccc-tttttttt**

**Fortunella_bawangica_2** **gccagagttctctatggacaatgggtaagaacgcggaaaaccggacccccc-tttttttt**

**Fortunella_hindsii_1** **gccagagttctctatggacaatgggtaagaacgcggaaaaccggacccccc-tttttttt**

**Fortunella_hindsii_2** **gccagagttctctatggacaatgggtaagaacgcggaaaaccggacccccc-tttttttt**

**Fortunella_hindsii_3** **gccagagttctctatggacaatgggtaagaacgcggaaaaccggacccccc-tttttttt**

**Fortunella_japonica_1** **gccagagttctctatggacaatgggtaagaacgcggaaaaccggacccccc-tttttttt**

**Fortunella_japonica_2** **gccagagttctctatggacaatgggtaagaacgcggaaaaccggacccccc-tttttttt**

**Fortunella_japonica_3** **gccagagttctctatggacaatgggtaagaacgcggaaaaccggacccccc-tttttttt**

**Fortunella_japonica_4** **gccagagttctctatggacaatgggtaagaacgcggaaaaccggacccccc-tttttttt**

**Fortunella_japonica_5** **gccagagttctctatggacaatgggtaagaacgcggaaaaccggacccccc-tttttttt**

**Fortunella_japonica_6** **gccagagttctctatggacaatgggtaagaacgcggaaaaccggacccccc-tttttttt**

**Fortunella_japonica_7** **gccagagttctctatggacaatgggtaagaacgcggaaaaccggacccccc-tttttttt**

**Fortunella_japonica_8** **gccagagttctctatggacaatgggtaagaacgcggaaaaccggacccccc-tttttttt**

**Fortunella_japonica_9** **gccagagttctctatggacaatgggtaagaacgcggaaaaccggacccccc-tttttttt**

**Fortunella_japonica_10** **gccagagttctctatggacaatgggtaagaacgcggaaaaccggaccccccttttttttt**

**Fortunella_japonica_11** **gccagagttctctatggacaatgggtaagaacgcggaaaaccggacccccc-tttttttt**

**Fortunella_japonica_12** **gccagagttctctatggacaatgggtaagaacgcggaaaaccggacccccc-tttttttt**

**Fortunella_margarita_1** **gccagagttctctatggacaatgggtaagaacgcggaaaaccggacccccc-tttttttt**

**Fortunella_margarita_2** **gccagagttctctatggacaatgggtaagaacgcggaaaaccggacccccc-tttttttt**

**Fortunella_venosa_1** **gccagagttctctatggacaatgggtaagaacgcggaaaaccggacccccc-tttttttt**

**Fortunella_venosa_2** **gccagagttctctatggacaatgggtaagaacgcggaaaaccggacccccc-tttttttt**

**Fortunella_venosa_3** **gccagagttctctatggacaatgggtaagaacgcggaaaaccggacccccc-tttttttt**

31930	31940	31950	31960	31970	31980

....|....|....|....|....|....|....|....|....|....|....|....|

**Atalantia_kwangtungens** **tattggcctgttcaaagagaaaagaaaggggttcacg-----------------------**

**Citrus_reticulata_1** **tattggactgttcaaagagaaaagaaa-gggttcacg-----------------------**

**Citrus_reticulata_2** **tattggactgttcaaagagaaaagaaa-gggttcacg-----------------------**

**Citrus_trifoliata_3** **tattggactgttcaaagagaaaagaaa-gggttcacg-----------------------**

**Citrus_aurantiifolia** **tattggactgttcaaagagaaaagaaa-gggttcacg-----------------------**

**Citrus_aurantium** **tattggactgttcaaagagaaaagaaa-gggttcacg-----------------------**

**Citrus_cavaleriei** **tattggactgttcaaagagaaaagaaa-gggttcacg-----------------------**

**Citrus_hongheensis** **tattggactgttcaaagagaaaagaaa-gggttcacg-----------------------**

**Citrus_limon** **tattggactgttcaaagagaaaagaaa-gggttcacg-----------------------**

**Citrus_maxima** **tattggactgttcaaagagaaaagaaa-gggttcacg-----------------------**

**Citrus_medica** **tattggactgttcaaagagaaaagaaa-gggttcacg-----------------------**

**Citrus_polytrifolia** **tattggactgttcaaagagaaaagaaa-gggttcacg-----------------------**

**Citrus_reticulata** **tattggactgttcaaagagaaaagaaa-gggttcacg-----------------------**

**Citrus_sinensis** **tattggactgttcaaagagaaaagaaa-gggttcacg-----------------------**

**Fortunella_bawangica_1** **tattggactgttcaaagagaaaagaaa-gggttcacg-----------------------**

**Fortunella_bawangica_2** **tattggactgttcaaagagaaaagaaa-gggttcacg-----------------------**

**Fortunella_hindsii_1** **tattggactgttcaaagagaaaagaaa-gggttcacgatttaatttgaatagttaataat**

**Fortunella_hindsii_2** **tattggactgttcaaagagaaaagaaa-gggttcacgatttaatttgaatagttaataat**

**Fortunella_hindsii_3** **tattggactgttcaaagagaaaagaaa-gggttcacgatttaatttgaatagttaataat**

**Fortunella_japonica_1** **tattggactgttcaaagagaaaagaaa-gggttcacg-----------------------**

**Fortunella_japonica_2** **tattggactgttcaaagagaaaagaaa-gggttcacg-----------------------**

**Fortunella_japonica_3** **tattggactgttcaaagagaaaagaaa-gggttcacg-----------------------**

**Fortunella_japonica_4** **tattggactgttcaaagagaaaagaaa-gggttcacg-----------------------**

**Fortunella_japonica_5** **tattggactgttcaaagagaaaagaaa-gggttcacg-----------------------**

**Fortunella_japonica_6** **tattggactgttcaaagagaaaagaaa-gggttcacg-----------------------**

**Fortunella_japonica_7** **tattggactgttcaaagagaaaagaaa-gggttcacg-----------------------**

**Fortunella_japonica_8** **tattggactgttcaaagagaaaagaaa-gggttcacg-----------------------**

**Fortunella_japonica_9** **tattggactgttcaaagagaaaagaaa-gggttcacg-----------------------**

**Fortunella_japonica_10** **tattggactgttcaaagagaaaagaaa-gggttcacg-----------------------**

**Fortunella_japonica_11** **tattggactgttcaaagagaaaagaaa-gggttcacg-----------------------**

**Fortunella_japonica_12** **tattggactgttcaaagagaaaagaaa-gggttcacg-----------------------**

**Fortunella_margarita_1** **tattggactgttcaaagagaaaagaaa-gggttcacg-----------------------**

**Fortunella_margarita_2** **tattggactgttcaaagagaaaagaaa-gggttcacg-----------------------**

**Fortunella_venosa_1** **tattggactgttcaaagagaaaagaaa-gggttcacg-----------------------**

**Fortunella_venosa_2** **tattggactgttcaaagagaaaagaaa-gggttcacg-----------------------**

**Fortunella_venosa_3** **tattggactgttcaaagagaaaagaaa-gggttcacg-----------------------**

31990	32000	32010	32020	32030	32040

....|....|....|....|....|....|....|....|....|....|....|....|

**Atalantia_kwangtungens** **-atttaatttgaaagagaaa----------------------agaaaggggttcacgatt**

**Citrus_reticulata_1** **-atttaattt--------------------------------------------------**

**Citrus_reticulata_2** **-atttaattt--------------------------------------------------**

**Citrus_trifoliata_3** **-atttaattt--------------------------------------------------**

**Citrus_aurantiifolia** **-atttaattt--------------------------------------------------**

**Citrus_aurantium** **-atttaattt--------------------------------------------------**

**Citrus_cavaleriei** **-atttaattt--------------------------------------------------**

**Citrus_hongheensis** **-atttaattt--------------------------------------------------**

**Citrus_limon** **-atttaattt--------------------------------------------------**

**Citrus_maxima** **-atttaattt--------------------------------------------------**

**Citrus_medica** **-atttaattt--------------------------------------------------**

**Citrus_polytrifolia** **-atttaattt--------------------------------------------------**

**Citrus_reticulata** **-atttaattt--------------------------------------------------**

**Citrus_sinensis** **-atttaattt--------------------------------------------------**

**Fortunella_bawangica_1** **-atttaatttgaatagttaataataatttaatttgaatatttgaatagttaataataatt**

**Fortunella_bawangica_2** **-atttaatttgaatagttaataataatttaatttgaatatttgaatagttaataataatt**

**Fortunella_hindsii_1** **aatttaatttgaatagttaataataatttaatttga--------atagttaataataatt**

**Fortunella_hindsii_2** **aatttaatttgaatagttaataataatttaatttga--------atagttaataataatt**

**Fortunella_hindsii_3** **aatttaatttgaatagttaataataatttaatttga--------atagttaataataatt**

**Fortunella_japonica_1** **-atttaatttgaatagttaataataatttaatttga--------atagttaataataatt**

**Fortunella_japonica_2** **-atttaatttgaatagttaataataatttaatttga--------atagttaataataat-**

**Fortunella_japonica_3** **-atttaatttgaatagttaataataatttaatttga--------atagttaataataatt**

**Fortunella_japonica_4** **-atttaatttgaatagttaataataatttaatttga--------atagttaataataatt**

**Fortunella_japonica_5** **-atttaatttgaatagttaataataatttaatttga--------atagttaataataatt**

**Fortunella_japonica_6** **-atttaatttgaatagttaataataatttaatttga--------atagttaataataatt**

**Fortunella_japonica_7** **-atttaatttgaatagttaataataatttaatttga--------atagttaataataatt**

**Fortunella_japonica_8** **-atttaatttgaatagttaataataatttaatttga--------atagttaataataatt**

**Fortunella_japonica_9** **-atttaatttgaatagttaataataatttaatttga--------atagttaataataatt**

**Fortunella_japonica_10** **-atttaatttgaatagttaataataatttaatttgaatatttgaatagttaataataatt**

**Fortunella_japonica_11** **-atttaatttgaatagttaataataatttaatttga--------atagttaataataatt**

**Fortunella_japonica_12** **-atttaatttgaatagttaataataatttaatttga--------atagttaataataatt**

**Fortunella_margarita_1** **-atttaatttgaatagttaataataatttaatttgaatatttgaatagttaataataatt**

**Fortunella_margarita_2** **-atttaatttgaatagttaataataatttaatttgaatatttgaatagttaataataatt**

**Fortunella_venosa_1** **-atttaatttgaatagttaataataatttaatttga--------atagttaataataatt**

**Fortunella_venosa_2** **-atttaatttgaatagttaataataatttaatttga--------atagttaataataatt**

**Fortunella_venosa_3** **-atttaatttgaatagttaataataataaaatttga--------atagttaataataatt**

32050	32060	32070	32080	32090	32100

....|....|....|....|....|....|....|....|....|....|....|....|

**Atalantia_kwangtungens** **taattt--------------------gaatagttaataataataagattgtgccttggtc**

**Citrus_reticulata_1** **--------------------------gaatagttaataataataagattgtgccttggtc**

**Citrus_reticulata_2** **--------------------------gaatagttaataataataagattgtgccttggtc**

**Citrus_trifoliata_3** **--------------------------gaatagttaataataataagattgtgccttggtc**

**Citrus_aurantiifolia** **--------------------------gaatagttaataataataagattgtgccttggtc**

**Citrus_aurantium** **--------------------------gaatagttaataataataagattgtgctttggtc**

**Citrus_cavaleriei** **--------------------------gaatagttaataataataagattgtgccttggtc**

**Citrus_hongheensis** **--------------------------gaatagttaataataataagattgtgccttggtc**

**Citrus_limon** **--------------------------gaatagttaataataataagattgtgctttggtc**

**Citrus_maxima** **--------------------------gaatagttaataataataagattgtgctttggtc**

**Citrus_medica** **--------------------------gaatagttaataataataagattgtgccttggtc**

**Citrus_polytrifolia** **--------------------------gaatagttaataataataagattgtgccttggtc**

**Citrus_reticulata** **--------------------------gaatagttaataataataagattgtgccttggtc**

**Citrus_sinensis** **--------------------------gaatagttaataataataagattgtgctttggtc**

**Fortunella_bawangica_1** **taatttgaatagttaataataataaggaatagttaataataataagattgtgccttggtc**

**Fortunella_bawangica_2** **taatttgaatagttaataataataaggaatagttaataataataagattgtgccttggtc**

**Fortunella_hindsii_1** **taatttgaatagttaataataataaggaatagttaataataataagattgtgccttggtc**

**Fortunella_hindsii_2** **taatttgaatagttaataataataaggaatagttaataataataagattgtgccttggtc**

**Fortunella_hindsii_3** **taatttgaatagttaataataataaggaatagttaataataataagattgtgccttggtc**

**Fortunella_japonica_1** **taatttgaatagttaataataataaggaatagttaataataataagattgtgccttggtc**

**Fortunella_japonica_2** **-----------------------aaggaatagttaataataataagattgtgccttggtc**

**Fortunella_japonica_3** **taatttgaatagttaataataataaggaatagttaataataataagattgtgccttggtc**

**Fortunella_japonica_4** **taatttgaatagttaataataataaggaatagttaataataataagattgtgccttggtc**

**Fortunella_japonica_5** **taatttgaatagttaataataataaggaatagttaataataataagattgtgccttggtc**

**Fortunella_japonica_6** **taatttgaatagttaataataataaggaatagttaataataataagattgtgccttggtc**

**Fortunella_japonica_7** **taatttgaatagttaataataataaggaatagttaataataataagattgtgccttggtc**

**Fortunella_japonica_8** **taatttgaatagttaataataataaggaatagttaataataataagattgtgccttggtc**

**Fortunella_japonica_9** **taatttgaatagttaataataataaggaatagttaataataataagattgtgccttggtc**

**Fortunella_japonica_10** **taatttgaatagttaataataataaggaatagttaataataataagattgtgccttggtc**

**Fortunella_japonica_11** **taatttgaatagttaataataataaggaatagttaataataataagattgtgccttggtc**

**Fortunella_japonica_12** **taatttgaatagttaataataataaggaatagttaataataataagattgtgccttggtc**

**Fortunella_margarita_1** **taatttgaatagttaataataataaggaatagttaataataataagattgtgccttggtc**

**Fortunella_margarita_2** **taatttgaatagttaataataataaggaatagttaataataataagattgtgccttggtc**

**Fortunella_venosa_1** **taatttgaatagttaataataataaggaatagttaataataataagattgtgccttggtc**

**Fortunella_venosa_2** **taatttgaatagttaataataataaggaatagttaataataataagattgtgccttggtc**

**Fortunella_venosa_3** **taatttgaatagttaataataataaggaatagttaataataataagattgtgccttggtc**

32110	32120	32130	32140	32150	32160

....|....|....|....|....|....|....|....|....|....|....|....|

**Atalantia_kwangtungens** **aagtcacatatttcgcacttaccaccggttttattatt----------tgagtattttag**

**Citrus_reticulata_1** **aagtcacatatttcgcacttaccaccgattttattattttttattatttgagtattttag**

**Citrus_reticulata_2** **aagtcacatatttcgcacttaccaccgattttattattttttattatttgagtattttag**

**Citrus_trifoliata_3** **aagtcacatatttcgcacttaccaccgatttgattatttttgattatttgagtattttag**

**Citrus_aurantiifolia** **aagtcacatatttcgcacttaccaccgattttattatt----------------------**

**Citrus_aurantium** **aagtcacatatttcgcacttaccaccgattttattattttttattatttgagtattttag**

**Citrus_cavaleriei** **aagtcacatatttcgcacttaccaccgatttgattattttttattatttgagtattttag**

**Citrus_hongheensis** **aagtcacatatttcgcacttaccaccgattttattattttttattatttgagtattttag**

**Citrus_limon** **aagtcacatatttcgcacttaccaccgattttattattttttattatttgagtattttag**

**Citrus_maxima** **aagtcacatatttcgcacttaccaccgattttattattttttattatttgagtattttag**

**Citrus_medica** **aagtcacatatttcgcacttaccaccgattttattattttttattatttgagtattttag**

**Citrus_polytrifolia** **aagtcacatatttcgcacttaccaccgatttgattatttttgattatttgagtattttag**

**Citrus_reticulata** **aagtcacatatttcgcacttaccaccgattttattattttttattatttgagtattttag**

**Citrus_sinensis** **aagtcacatatttcgcacttaccaccgattttattattttttattatttgagtattttag**

**Fortunella_bawangica_1** **aagtcacatatttcgcacttaccaccgatttgattattttttattatttgagtattttag**

**Fortunella_bawangica_2** **aagtcacatatttcgcacttaccaccgatttgattattttttattatttgagtattttag**

**Fortunella_hindsii_1** **aagtcacatatttcgcacttaccaccgatttgattattttttattatttgagtattttag**

**Fortunella_hindsii_2** **aagtcacatatttcgcacttaccaccgatttgattattttttattatttgagtattttag**

**Fortunella_hindsii_3** **aagtcacatatttcgcacttaccaccgatttgattattttttattatttgagtattttcg**

**Fortunella_japonica_1** **aagtcacatatttcgcacttaccaccgatttgattattttttattatttgagtattttag**

**Fortunella_japonica_2** **aagtcacatatttcgcacttaccaccgatttgattattttttattatttgagtattttag**

**Fortunella_japonica_3** **aagtcacatatttcgcacttaccaccgatttgattattttttattatttgagtattttag**

**Fortunella_japonica_4** **aagtcacatatttcgcacttaccaccgatttgattattttttattatttgagtattttag**

**Fortunella_japonica_5** **aagtcacatatttcgcacttaccaccgatttgattattttttattatttgagtattttag**

**Fortunella_japonica_6** **aagtcacatatttcgcacttaccaccgatttgattattttttattatttgagtattttag**

**Fortunella_japonica_7** **aagtcacatatttcgcacttaccaccgatttgattattttttattatttgagtattttag**

**Fortunella_japonica_8** **aagtcacatatttcgcacttaccaccgatttgattattttttattatttgagtattttag**

**Fortunella_japonica_9** **aagtcacatatttcgcacttaccaccgatttgattattttttattatttgagtattttag**

**Fortunella_japonica_10** **aagtcacatatttcgcacttaccaccgatttgattattttttattatttgagtattttag**

**Fortunella_japonica_11** **aagtcacatatttcgcacttaccaccgatttgattattttttattatttgagtattttag**

**Fortunella_japonica_12** **aagtcacatatttcgcacttaccaccgatttgattattttttattatttgagtattttag**

**Fortunella_margarita_1** **aagtcacatatttcgcacttaccaccgatttgattattttttattatttgagtattttag**

**Fortunella_margarita_2** **aagtcacatatttcgcacttaccaccgatttgattattttttattatttgagtattttag**

**Fortunella_venosa_1** **aagtcacatatttcgcacttaccaccgatttgattattttttattatttgagtattttag**

**Fortunella_venosa_2** **aagtcacatatttcgcacttaccaccgatttgattattttttattatttgagtattttag**

**Fortunella_venosa_3** **aagtcacatatttcgcacttaccaccgatttgattattttttattatttgagtattttag**

32170	32180	32190	32200	32210	32220

....|....|....|....|....|....|....|....|....|....|....|....|

**Atalantia_kwangtungens** **aaatttgctttctgctatgctttattga-ccgtttcatatcatggctccagggttgaaaa**

**Citrus_reticulata_1** **aaatttgctttctgctatgctttattgacccgtttcatatcatggctccagggttgacaa**

**Citrus_reticulata_2** **aaatttgctttctgctatgctttattgacccgtttcatatcatggctccagggttgacaa**

**Citrus_trifoliata_3** **aaatttgctttatgctatgctttattgacccctttcatatcatggctccagggttgacaa**

**Citrus_aurantiifolia** **------------------------------------------------------------**

**Citrus_aurantium** **aaatttgctttctgctatgctttattgacccgtttcatatcatggctccagggttgacaa**

**Citrus_cavaleriei** **aaatttgctttctgctatgctttattgacccgtttcatatcatggctccagggttgacaa**

**Citrus_hongheensis** **aaatttgctttctgctatgctttattgacccgtttcatatcatggccccagggttgacaa**

**Citrus_limon** **aaatttgctttctgctatgctttattgacccgtttcatatcatggctccagggttgacaa**

**Citrus_maxima** **aaatttgctttctgctatgctttattgacccgtttcatatcatggctccagggttgacaa**

**Citrus_medica** **aaatttgctttctactatgctttattgacccgtttcatatcatggctccagggttgacaa**

**Citrus_polytrifolia** **aaatttgctttatgctatgctttattgacccctttcatatcatggctccagggttgacaa**

**Citrus_reticulata** **aaatttgctttctgctatgctttattgacccgtttcatatcatggctccagggttgacaa**

**Citrus_sinensis** **aaatttgctttctgctatgctttattgacccgtttcatatcatggctccagggttgacaa**

**Fortunella_bawangica_1** **aaatttgctttctgctatgctttattgacccgtttcatatcatggctccagggttgacaa**

**Fortunella_bawangica_2** **aaatttgctttctgctatgctttattgacccgtttcatatcatggctccagggttgacaa**

**Fortunella_hindsii_1** **aaatttgctttctgctatgctttattgacccgtttcatatcatggctccagggttgacaa**

**Fortunella_hindsii_2** **aaatttgctttctgctatgctttattgacccgtttcatatcatggctccagggttgacaa**

**Fortunella_hindsii_3** **aaatttgctttctgctatgctttattgacccgtttcatatcatggctccagggttgacaa**

**Fortunella_japonica_1** **aaatttgctttctgctatgctttattgacccgtttcatatcatggctccagggttgacaa**

**Fortunella_japonica_2** **aaatttgctttctgctatgctttattgacccgtttcatatcatggctccagggttgacaa**

**Fortunella_japonica_3** **aaatttgctttctgctatgctttattgacccgtttcatatcatggctccagggttgacaa**

**Fortunella_japonica_4** **aaatttgctttctgctatgctttattgacccgtttcatatcatggctccagggttgacaa**

**Fortunella_japonica_5** **aaatttgctttctgctatgctttattgacccgtttcatatcatggctccagggttgacaa**

**Fortunella_japonica_6** **aaatttgctttctgctatgctttattgacccgtttcatatcatggctccagggttgacaa**

**Fortunella_japonica_7** **aaatttgctttctgctatgctttattgacccgtttcatatcatggctccagggttgacaa**

**Fortunella_japonica_8** **aaatttgctttctgctatgctttattgacccgtttcatatcatggctccagggttgacaa**

**Fortunella_japonica_9** **aaatttgctttctgctatgctttattgacccgtttcatatcatggctccagggttgacaa**

**Fortunella_japonica_10** **aaatttgctttctgctatgctttattgacccgtttcatatcatggctccagggttgacaa**

**Fortunella_japonica_11** **aaatttgctttctgctatgctttattgacccgtttcatatcatggctccagggttgacaa**

**Fortunella_japonica_12** **aaatttgctttctgctatgctttattgacccgtttcatatcatggctccagggttgacaa**

**Fortunella_margarita_1** **aaatttgctttctgctatgctttattgacccgtttcatatcatggctccagggttgacaa**

**Fortunella_margarita_2** **aaatttgctttctgctatgctttattgacccgtttcatatcatggctccagggttgacaa**

**Fortunella_venosa_1** **aaatttgctttctgctatgctttattgacccgtttcatatcatggctccagggttgacaa**

**Fortunella_venosa_2** **aaatttgctttctgctatgctttattgacccgtttcatatcatggctccagggttgacaa**

**Fortunella_venosa_3** **aaatttgctttctgctatgctttattgacccgtttcatatcatggctccagggttgacaa**

32230	32240	32250	32260	32270	32280

....|....|....|....|....|....|....|....|....|....|....|....|

**Atalantia_kwangtungens** **tcgccggggtacccctttttgaaatcggaagaagttatagaatagaactctttggatcat**

**Citrus_reticulata_1** **tcgccggggtacccctttttgaaatcggaagaagttatagaatagaactccttggatcat**

**Citrus_reticulata_2** **tcgccggggtacccctttttgaaatcggaagaagttatagaatagaactccttggatcat**

**Citrus_trifoliata_3** **tcgccggggtacccctttttgaaatcggaagaagttatagaatagaactccttggatcat**

**Citrus_aurantiifolia** **------------------------------------------------------------**

**Citrus_aurantium** **tcgccggggtacccctttttgaaatcggaagaagttatagaatagaactccttggatcat**

**Citrus_cavaleriei** **tcgccggggtacccctttttgaaatcggaagaagttatagaatagaactccttggatcat**

**Citrus_hongheensis** **tcgccggggtacccctttttgaaatcggaagaagttatagaatagaactccttggatcat**

**Citrus_limon** **tcgccggggtacccctttttgaaatcggaagaagttatagaatagaactccttggatcat**

**Citrus_maxima** **tcgccggggtacccctttttgaaatcggaagaagttatagaatagaactccttggatcat**

**Citrus_medica** **tcgccggggtacccctttttgaaatcggaagaagttatagaatagcactccttggatcat**

**Citrus_polytrifolia** **tcgccggggtacccctttttgaaatcggaagaagttatagaatagaactccttggatcat**

**Citrus_reticulata** **tcgccggggtacccctttttgaaatcggaagaagttatagaatagaactccttggatcat**

**Citrus_sinensis** **tcgccggggtacccctttttgaaatcggaagaagttatagaatagaactccttggatcat**

**Fortunella_bawangica_1** **tcgccggggtacccctttttgaaatcggaagaagttatagaatagaactccttggatcat**

**Fortunella_bawangica_2** **tcgccggggtacccctttttgaaatcggaagaagttatagaatagaactccttggatcat**

**Fortunella_hindsii_1** **tcgccggggtacccctttttgaaatcggaagaagttatagaatagaactccttggatcat**

**Fortunella_hindsii_2** **tcgccggggtacccctttttgaaatcggaagaagttatagaatagaactccttggatcat**

**Fortunella_hindsii_3** **tcgccggggtacccctttttgaaatcggaagaagttatagaatagaactccttggatcat**

**Fortunella_japonica_1** **tcgccggggtacccctttttgaaatcggaagaagttatagaatagaactccttggatcat**

**Fortunella_japonica_2** **tcgccggggtacccctttttgaaatcggaagaagttatagaatagaactccttggatcat**

**Fortunella_japonica_3** **tcgccggggtacccctttttgaaatcggaagaagttatagaatagaactccttggatcat**

**Fortunella_japonica_4** **tcgccggggtacccctttttgaaatcggaagaagttatagaatagaactccttggatcat**

**Fortunella_japonica_5** **tcgccggggtacccctttttgaaatcggaagaagttatagaatagaactccttggatcat**

**Fortunella_japonica_6** **tcgccggggtacccctttttgaaatcggaagaagttatagaatagaactccttggatcat**

**Fortunella_japonica_7** **tcgccggggtacccctttttgaaatcggaagaagttatagaatagaactccttggatcat**

**Fortunella_japonica_8** **tcgccggggtacccctttttgaaatcggaagaagttatagaatagaactccttggatcat**

**Fortunella_japonica_9** **tcgccggggtacccctttttgaaatcggaagaagttatagaatagaactccttggatcat**

**Fortunella_japonica_10** **tcgccggggtacccctttttgaaatcggaagaagttatagaatagaactccttggatcat**

**Fortunella_japonica_11** **tcgccggggtacccctttttgaaatcggaagaagttatagaatagaactccttggatcat**

**Fortunella_japonica_12** **tcgccggggtacccctttttgaaatcggaagaagttatagaatagaactccttggatcat**

**Fortunella_margarita_1** **tcgccggggtacccctttttgaaatcggaagaagttatagaatagaactccttggatcat**

**Fortunella_margarita_2** **tcgccggggtacccctttttgaaatcggaagaagttatagaatagaactccttggatcat**

**Fortunella_venosa_1** **tcgccggggtacccctttttgaaatcggaagaagttatagaatagaactccttggatcat**

**Fortunella_venosa_2** **tcgccggggtacccctttttgaaatcggaagaagttatagaatagaactccttggatcat**

**Fortunella_venosa_3** **tcgccggggtacccctttttgaaatcggaagaagttatagaatagaactccttggatcat**

32290	32300	32310	32320	32330	32340

....|....|....|....|....|....|....|....|....|....|....|....|

**Atalantia_kwangtungens** **ctgtcaaccgctcaatcaacgacttcttcggaatgct-aaaaaaaacgattactttattt**

**Citrus_reticulata_1** **ctgtcaaccgctcaatcaacgacttcttcggaatgct-aaaaaaaacgattactttattt**

**Citrus_reticulata_2** **ctgtcaaccgctcaatcaacgacttcttcggaatgct-aaaaaaaacgattactttattt**

**Citrus_trifoliata_3** **ctgtcaaccgctcaatcaacgacttcttcggaatgct-aaaaaaaacgattactttattt**

**Citrus_aurantiifolia** **------------------------------------------------------------**

**Citrus_aurantium** **ctgtcaaccgctcaatcaacgacttcttcggaatgct-aaaaaaaacgattactttattt**

**Citrus_cavaleriei** **ctgtcaaccgctcaatcaacgactttttcggaatgct-aaaaaaaacgattactttattt**

**Citrus_hongheensis** **ctgtcaaccgctcaatcaacgacttcttcggaatgct-aaaaaaaacgattactttattt**

**Citrus_limon** **ctgtcaaccgctcaatcaacgacttcttcggaatgct-aaaaaaaacgattactttattt**

**Citrus_maxima** **ctgtcaaccgctcaatcaacgacttcttcggaatgct-aaaaaaaacgattactttattt**

**Citrus_medica** **ctgtcaaccgctcaatcaacgacttcttcggaatgctaaaaaaaaacgattactttattt**

**Citrus_polytrifolia** **ctgtcaaccgctcaatcaacgacttcttcggaatgct-aaaaaaaacgattactttattt**

**Citrus_reticulata** **ctgtcaaccgctcaatcaacgacttcttcggaatgct-aaaaaaaacgattactttattt**

**Citrus_sinensis** **ctgtcaaccgctcaatcaacgacttcttcggaatgct-aaaaaaaacgattactttattt**

**Fortunella_bawangica_1** **ctgtcaaccgctcaatcaacgacttcttcggaatgct-aaaaaaaacgattactttattt**

**Fortunella_bawangica_2** **ctgtcaaccgctcaatcaacgacttcttcggaatgct-aaaaaaaacgattactttattt**

**Fortunella_hindsii_1** **ctgtcaaccgctcaatcaacgacttcttcggaatgct-aaaaaaaacgattactttattt**

**Fortunella_hindsii_2** **ctgtcaaccgctcaatcaacgacttcttcggaatgct-aaaaaaaacgattactttattt**

**Fortunella_hindsii_3** **ctgtcaaccgctcaatcaacgacttcttcggaatgct-aaaaaaaacgattactttattt**

**Fortunella_japonica_1** **ctgtcaaccgctcaatcaacgacttcttcggaatgct-aaaaaaaacgattactttattt**

**Fortunella_japonica_2** **ctgtcaaccgctcaatcaacgacttcttcggaatgct-aaaaaaaacgattactttattt**

**Fortunella_japonica_3** **ctgtcaaccgctcaatcaacgacttcttcggaatgct-aaaaaaaacgattactttattt**

**Fortunella_japonica_4** **ctgtcaaccgctcaatcaacgacttcttcggaatgct-aaaaaaaacgattactttattt**

**Fortunella_japonica_5** **ctgtcaaccgctcaatcaacgacttcttcggaatgct-aaaaaaaacgattactttattt**

**Fortunella_japonica_6** **ctgtcaaccgctcaatcaacgacttcttcggaatgct-aaaaaaaacgattactttattt**

**Fortunella_japonica_7** **ctgtcaaccgctcaatcaacgacttcttcggaatgct-aaaaaaaacgattactttattt**

**Fortunella_japonica_8** **ctgtcaaccgctcaatcaacgacttcttcggaatgct-aaaaaaaacgattactttattt**

**Fortunella_japonica_9** **ctgtcaaccgctcaatcaacgacttcttcggaatgct-aaaaaaaacgattactttattt**

**Fortunella_japonica_10** **ctgtcaaccgctcaatcaacgacttcttcggaatgct-aaaaaaaacgattactttattt**

**Fortunella_japonica_11** **ctgtcaaccgctcaatcaacgacttcttcggaatgct-aaaaaaaacgattactttattt**

**Fortunella_japonica_12** **ctgtcaaccgctcaatcaacgacttcttcggaatgct-aaaaaaaacgattactttattt**

**Fortunella_margarita_1** **ctgtcaaccgctcaatcaacgacttcttcggaatgct-aaaaaaaacgattactttattt**

**Fortunella_margarita_2** **ctgtcaaccgctcaatcaacgacttcttcggaatgct-aaaaaaaacgattactttattt**

**Fortunella_venosa_1** **ctgtcaaccgctcaatcaacgacttcttcggaatgct-aaaaaaaacgattactttattt**

**Fortunella_venosa_2** **ctgtcaaccgctcaatcaacgacttcttcggaatgct-aaaaaaaacgattactttattt**

**Fortunella_venosa_3** **ctgtcaaccgctcaatcaacgacttcttcggaatgct-aaaaaaaacgattactttattt**

32350	32360	32370	32380	32390	32400

....|....|....|....|....|....|....|....|....|....|....|....|

**Atalantia_kwangtungens** **ctttcctatttcattttcttttattaacttgattttcttttagtaatatatgcccaattt**

**Citrus_reticulata_1** **ctttcctatttcattttcttttattaacttgattttcttttagtaatatatgcccaaatt**

**Citrus_reticulata_2** **ctttcctatttcattttcttttattaacttgattttcttttagtaatatatgcccaaatt**

**Citrus_trifoliata_3** **ctttcctatttcattttcttttattaacttgattttcttttagtaatatatgcccaaatt**

**Citrus_aurantiifolia** **------------------ttttattaacttgattttcttttagtaatatatgccaaaatt**

**Citrus_aurantium** **ctttcctatttcattttcttttattaacttgattttcttttagtaatatatgcccaaatt**

**Citrus_cavaleriei** **ctttcctatttcattttcttttattaacttgattttcttttagtaatatatgcccaaatt**

**Citrus_hongheensis** **ctttcctatttcattttcttttattaacttgattttcttttagtaatatatgcccaaatt**

**Citrus_limon** **ctttcctatttcattttcttttattaacttgattttcttttagtaatatatgcccaaatt**

**Citrus_maxima** **ctttcctatttcattttcttttattaacttgattttcttttagtaatatatgcccaaatt**

**Citrus_medica** **ctttcctatttcattttcttttattaacttgattttcttttagtaatatctgcccaaatt**

**Citrus_polytrifolia** **ctttcctatttcattttcttttattaacttgattttcttttagtaatatatgcccaaatt**

**Citrus_reticulata** **ctttcctatttcattttcttttattaacttgattttcttttagtaatatatgcccaaatt**

**Citrus_sinensis** **ctttcctatttcattttcttttattaacttgattttcttttagtaatatatgcccaaatt**

**Fortunella_bawangica_1** **ctttcctatttcattttcttttattaacttgattttcttttagtaatatatgcccaaatt**

**Fortunella_bawangica_2** **ctttcctatttcattttcttttattaacttgattttcttttagtaatatatgcccaaatt**

**Fortunella_hindsii_1** **ctttcctatttcattttcttttattaacttgattttcttttagtaatatatgcccaaatt**

**Fortunella_hindsii_2** **ctttcctatttcattttcttttattaacttgattttcttttagtaatatatgcccaaatt**

**Fortunella_hindsii_3** **ctttcctatttcattttcttttattaacttgattttcttttagtaatatatgcccaaatt**

**Fortunella_japonica_1** **ctttcctatttcattttcttttattaacttgattttcttttagtaatatatgcccaaatt**

**Fortunella_japonica_2** **ctttcctatttcattttcttttattaacttgattttcttttagtaatatatgcccaaatt**

**Fortunella_japonica_3** **ctttcctatttcattttcttttattaacttgattttcttttagtaatatatgcccaaatt**

**Fortunella_japonica_4** **ctttcctatttcattttcttttattaacttgattttcttttagtaatatatgcccaaatt**

**Fortunella_japonica_5** **ctttcctatttcattttcttttattaacttgattttcttttagtaatatatgcccaaatt**

**Fortunella_japonica_6** **ctttcctatttcattttcttttattaacttgattttcttttagtaatatatgcccaaatt**

**Fortunella_japonica_7** **ctttcctatttcattttcttttattaacttgattttcttttagtaatatatgcccaaatt**

**Fortunella_japonica_8** **ctttcctatttcattttcttttattaacttgattttcttttagtaatatatgcccaaatt**

**Fortunella_japonica_9** **ctttcctatttcattttcttttattaacttgattttcttttagtaatatatgcccaaatt**

**Fortunella_japonica_10** **ctttcctatttcattttcttttattaacttgattttcttttagtaatatatgcccaaatt**

**Fortunella_japonica_11** **ctttcctatttcattttcttttattaacttgattttcttttagtaatatatgcccaaatt**

**Fortunella_japonica_12** **ctttcctatttcattttcttttattaacttgattttcttttagtaatatatgcccaaatt**

**Fortunella_margarita_1** **ctttcctatttcattttcttttattaacttgattttcttttagtaatatatgcccaaatt**

**Fortunella_margarita_2** **ctttcctatttcattttcttttattaacttgattttcttttagtaatatatgcccaaatt**

**Fortunella_venosa_1** **ctttcctatttcattttcttttattaacttgattttcttttagtaatatatgcccaaatt**

**Fortunella_venosa_2** **ctttcctatttcattttcttttattaacttgattttcttttagtaatatatgcccaaatt**

**Fortunella_venosa_3** **ctttcctatttcattttcttttattaacttgattttcttttagtaatatatgcccaaatt**

32410	32420	32430	32440	32450	32460

....|....|....|....|....|....|....|....|....|....|....|....|

**Atalantia_kwangtungens** **tgtttttctttcatcgatttgattct-------ttttt-------taatggataccg---**

**Citrus_reticulata_1** **tttttttctttcatcgatttgattct-------ttttt-------taatgtataccg---**

**Citrus_reticulata_2** **tttttttctttcatcgatttgattct-------ttttt-------taatgtataccg---**

**Citrus_trifoliata_3** **cgtttttctttcatcgatttgattct-------ttttt-------taatggataccg---**

**Citrus_aurantiifolia** **tgtttttctttcatcgatttgattct-------ttttt-------taatggataccg---**

**Citrus_aurantium** **tgtttttctttcatcgatttgattct-------ttttt-tatttttaatggataccg---**

**Citrus_cavaleriei** **tgtttttctttcatcgatttgattct-------ttttt-------taatggataccg---**

**Citrus_hongheensis** **tgtttttctttcatcgatttgattct-------ttttt-tatttttaatggataccg---**

**Citrus_limon** **tgtttttctttcatcgatttgattct-------ttttt-tatttttaatggataccg---**

**Citrus_maxima** **tgtttttctttcatcgatttgattct-------ttttt-tatttttaatggataccg---**

**Citrus_medica** **tgtttttctttcatcgatttgattct-------ttttt-------taatggataccg---**

**Citrus_polytrifolia** **cgtttttctttcatcgatttgattct-------ttttt-------taatggataccg---**

**Citrus_reticulata** **tttttttctttcatcgatttgattct-------ttttt-------taatgtataccg---**

**Citrus_sinensis** **tgtttttctttcatcgatttgattct-------tttttttatttttaatggataccg---**

**Fortunella_bawangica_1** **tgtttttctttcatcgatttgattct-------ttttt-------taatggataccgaga**

**Fortunella_bawangica_2** **tgtttttctttcatcgatttgattct-------ttttt-------taatggataccgaga**

**Fortunella_hindsii_1** **tgtttttctttcatcgatttgattct-------ttttt-------taatggataccgaga**

**Fortunella_hindsii_2** **tgtttttctttcatcgatttgattct-------ttttt-------taatggataccgaga**

**Fortunella_hindsii_3** **tgtttttctttcatcgatttgattct-------ttttt-------taatggataccgaga**

**Fortunella_japonica_1** **tgtttttctttcatcgatttgattct-------ttttt-------taatggataccgaga**

**Fortunella_japonica_2** **tgtttttctttcatcgatttgattct-------ttttt-------taatggataccgaga**

**Fortunella_japonica_3** **tgtttttctttcatcgatttgattct-------ttttt-------taatggataccgaga**

**Fortunella_japonica_4** **tgtttttctttcatcgatttgattct-------ttttt-------taatggataccgaga**

**Fortunella_japonica_5** **tgtttttctttcatcgatttgattct-------ttttt-------taatggataccgaga**

**Fortunella_japonica_6** **tgtttttctttcatcgatttgattct-------ttttt-------taatggataccgaga**

**Fortunella_japonica_7** **tgtttttctttcatcgatttgattct-------ttttt-------taatggataccgaga**

**Fortunella_japonica_8** **tgtttttctttcatcgatttgattct-------ttttt-------taatggataccgaga**

**Fortunella_japonica_9** **tgtttttctttcatcgatttgattct-------ttttt-------taatggataccgaga**

**Fortunella_japonica_10** **tgtttttctttcatcgatttgattct-------ttttt-------taatggataccgaga**

**Fortunella_japonica_11** **tgtttttctttcatcgatttgattct-------ttttt-------taatggataccgaga**

**Fortunella_japonica_12** **tgtttttctttcatcgatttgattct-------ttttt-------taatggataccgaga**

**Fortunella_margarita_1** **tgtttttctttcatcgatttgattct-------ttttt-------taatggataccgaga**

**Fortunella_margarita_2** **tgtttttctttcatcgatttgattct-------ttttt-------taatggataccgaga**

**Fortunella_venosa_1** **tgtttttctttcatcgatttgattctttttttattttt-------taatggataccgaga**

**Fortunella_venosa_2** **tgtttttctttcatcgatttgattctttttttattttt-------taatggataccgaga**

**Fortunella_venosa_3** **tgtttttctttcatcgatttgattctttttttattttt-------taatggataccgaga**

32470	32480	32490	32500	32510	32520

....|....|....|....|....|....|....|....|....|....|....|....|

**Atalantia_kwangtungens** **----------agattcatattgaaaataatcaga-----------------aataaattt**

**Citrus_reticulata_1** **----------agattcatattgaaaataatcaga-------------aataaatcaattt**

**Citrus_reticulata_2** **----------agattcatattgaaaataatcaga-------------aataaatcaattt**

**Citrus_trifoliata_3** **----------agattcatattgaaaataatcaga-------------aataaataaattt**

**Citrus_aurantiifolia** **----------agattcatattgaaaataatcaga-------------aataaataaattt**

**Citrus_aurantium** **----------agattcatattgaaaataatctga-------------aataaataaattt**

**Citrus_cavaleriei** **----------agattcatattgaaaataatcaga-------------aataaataaattt**

**Citrus_hongheensis** **----------agattcatattgaaaataatcaga-------------aataaataaattt**

**Citrus_limon** **----------agattcatattgaaaataatctga-------------aataaataaattt**

**Citrus_maxima** **----------agattcatattgaaaataatctga-------------aataaataaattt**

**Citrus_medica** **----------agattcatattgaaaataatcaga-------------aataaataaattt**

**Citrus_polytrifolia** **----------agattcatattgaaaataatcaga-------------aataaataaattt**

**Citrus_reticulata** **----------agattcatattgaaaataatcaga-------------aataaatcaattt**

**Citrus_sinensis** **----------agattcatattgaaaataatctga-------------aataaataaattt**

**Fortunella_bawangica_1** **ttcatattgaagattcatattgaaaataatcagaaataaataaatttaataaataaattt**

**Fortunella_bawangica_2** **ttcatattgaagattcatattgaaaataatcagaaataaataaatttaataaataaattt**

**Fortunella_hindsii_1** **ttcatattgaagattcatattgaaaataatcagaaataaataaatttaataaataaattt**

**Fortunella_hindsii_2** **ttcatattgaagattcatattgaaaataatcagaaataaataaatttaataaataaattt**

**Fortunella_hindsii_3** **ttcatattgaagattcatattgaaaataatcagaaataaataaatttaataaataaattt**

**Fortunella_japonica_1** **ttcatattgaagattcatattgaaaataatcagaaataaataaatttaataaataaattt**

**Fortunella_japonica_2** **ttcatattgaagattcatattgaaaataatcagaaataaataaatttaataaataaattt**

**Fortunella_japonica_3** **ttcatattgaagattcatattgaaaataatcagaaataaataaatttaataaataaattt**

**Fortunella_japonica_4** **ttcatattgaagattcatattgaaaataatcagaaataaataaatttaataaataaattt**

**Fortunella_japonica_5** **ttcatattgaagattcatattgaaaataatcagaaataaataaatttaataaataaattt**

**Fortunella_japonica_6** **ttcatattgaagattcatattgaaaataatcagaaataaataaatttaataaataaattt**

**Fortunella_japonica_7** **ttcatattgaagattcatattgaaaataatcagaaataaataaatttaataaataaattt**

**Fortunella_japonica_8** **ttcatattgaagattcatattgaaaataatcagaaataaataaatttaataaataaattt**

**Fortunella_japonica_9** **ttcatattgaagattcatattgaaaataatcagaaataaataaatttaataaataaattt**

**Fortunella_japonica_10** **ttcatattgaagattcatattgaaaataatcagaaataaataaatttaataaataaattt**

**Fortunella_japonica_11** **ttcatattgaagattcatattgaaaataatcagaaataaataaatttaataaataaattt**

**Fortunella_japonica_12** **ttcatattgaagattcatattgaaaataatcagaaataaataaatttaataaataaattt**

**Fortunella_margarita_1** **ttcatattgaagattcatattgaaaataatcagaaataaataaatttaataaataaattt**

**Fortunella_margarita_2** **ttcatattgaagattcatattgaaaataatcagaaataaataaatttaataaataaattt**

**Fortunella_venosa_1** **ttcatattgaagattcatattgaaaataatcagaaataaataaatttaataaataaattt**

**Fortunella_venosa_2** **ttcatattgaagattcatattgaaaataatcagaaataaataaatttaataaataaattt**

**Fortunella_venosa_3** **ttcatattgaagattcatattgaaaataatcagaaataaataaatttaataaataaattt**

32530	32540	32550	32560	32570	32580

....|....|....|....|....|....|....|....|....|....|....|....|

**Atalantia_kwangtungens** **gaaaatcgtaagagcagcctttcgattatttcagattgattggaatgaacaaaaagaaaa**

**Citrus_reticulata_1** **gaaaatcgtaagagcagcctttcgattatttcagattgattggaatgaacaaaaagaaaa**

**Citrus_reticulata_2** **gaaaatcgtaagagcagcctttcgattatttcagattgattggaatgaacaaaaagaaaa**

**Citrus_trifoliata_3** **gaaaatcgtaagagcagcctttcgattatttcagattgattggaatgaacaaaaagaaaa**

**Citrus_aurantiifolia** **gaaaatcgtaagagcagcctttcgattatttcagattgattggaatgaacaaaaagaaaa**

**Citrus_aurantium** **gaaaatcgtaagagcagcctttcgattatttcagattgattggaatgaacaaaaagaaaa**

**Citrus_cavaleriei** **gaaaatcgtaagagcagcctttcgattatttcagattgattggaatgaacaaaaagaaaa**

**Citrus_hongheensis** **gaaaatcgtaagagcagcctttcgattatttcagattgattggaatgaacaaaaagaaaa**

**Citrus_limon** **gaaaatcgtaagagcagcctttcgattatttcagattgattggaatgaacaaaaagaaaa**

**Citrus_maxima** **gaaaatcgtaagagcagcctttcgattatttcagattgattggaatgaacaaaaagaaaa**

**Citrus_medica** **gaaaatcataagagcagcctttcgattatttcagattgattggaatgaacaaaaagaaaa**

**Citrus_polytrifolia** **gaaaatcgtaagagcagcctttcgattatttcagattgattggaatgaacaaaaagaaaa**

**Citrus_reticulata** **gaaaatcgtaagagcagcctttcgattatttcagattgattggaatgaacaaaaagaaaa**

**Citrus_sinensis** **gaaaatcgtaagagcagcctttcgattatttcagattgattggaatgaacaaaaagaaaa**

**Fortunella_bawangica_1** **gaaaatcgtaagagcagcctttcgattatttcagattgattggaatgaacaaaaagaaaa**

**Fortunella_bawangica_2** **gaaaatcgtaagagcagcctttcgattatttcagattgattggaatgaacaaaaagaaaa**

**Fortunella_hindsii_1** **gaaaatcgtaagagcagcctttcgattatttcagattgattggcatgaacaaaaagaaaa**

**Fortunella_hindsii_2** **gaaaatcgtaagagcagcctttcgattatttcagattgattggaatgaacaaaaagaaaa**

**Fortunella_hindsii_3** **gaaaatcgtaagagcagcctttcgattatttcagattgattggaatgaacaaaaagaaaa**

**Fortunella_japonica_1** **gaaaatcgtaagagcagcctttcgattatttcagattgattggaatgaacaaaaagaaaa**

**Fortunella_japonica_2** **gaaaatcgtaagagcagcctttcgattatttcagattgattggaatgaacaaaaagaaaa**

**Fortunella_japonica_3** **gaaaatcgtaagagcagcctttcgattatttcagattgattggaatgaacaaaaagaaaa**

**Fortunella_japonica_4** **gaaaatcgtaagagcagcctttcgattatttcagattgattggaatgaacaaaaagaaaa**

**Fortunella_japonica_5** **gaaaatcgtaagagcagcctttcgattatttcagattgattggaatgaacaaaaagaaaa**

**Fortunella_japonica_6** **gaaaatcgtaagagcagcctttcgattatttcagattgattggaatgaacaaaaagaaaa**

**Fortunella_japonica_7** **gaaaatcgtaagagcagcctttcgattatttcagattgattggaatgaacaaaaagaaaa**

**Fortunella_japonica_8** **gaaaatcgtaagagcagcctttcgattatttcagattgattggaatgaacaaaaagaaaa**

**Fortunella_japonica_9** **gaaaatcgtaagagcagcctttcgattatttcagattgattggaatgaacaaaaagaaaa**

**Fortunella_japonica_10** **gaaaatcgtaagagcagcctttcgattatttcagattgattggaatgaacaaaaagaaaa**

**Fortunella_japonica_11** **gaaaatcgtaagagcagcctttcgattatttcagattgattggaatgaacaaaaagaaaa**

**Fortunella_japonica_12** **gaaaatcgtaagagcagcctttcgattatttcagattgattggaatgaacaaaaagaaaa**

**Fortunella_margarita_1** **gaaaatcgtaagagcagcctttcgattatttcagattgattggaatgaacaaaaagaaaa**

**Fortunella_margarita_2** **gaaaatcgtaagagcagcctttcgattatttcagattgattggaatgaacaaaaagaaaa**

**Fortunella_venosa_1** **gaaaatcgtaagagcagcctttcgattatttcagattgattggaatgaacaaaaagaaaa**

**Fortunella_venosa_2** **gaaaatcgtaagagcagcctttcgattatttcagattgattggaatgaacaaaaagaaaa**

**Fortunella_venosa_3** **gaaaatcgtaagagcagcctttcgattatttcagattgattggaatgaacaaaaagaaaa**

32590	32600	32610	32620	32630	32640

....|....|....|....|....|....|....|....|....|....|....|....|

**Atalantia_kwangtungens** **tacaaatagacgaagcaaagaaatagaattgagatactatgtatatattaacaaatagac**

**Citrus_reticulata_1** **t-------------------------------------------------acaaatagac**

**Citrus_reticulata_2** **t-------------------------------------------------acaaatagac**

**Citrus_trifoliata_3** **t-------------------------------------------------acaaatagac**

**Citrus_aurantiifolia** **t-------------------------------------------------acaaatagac**

**Citrus_aurantium** **t-------------------------------------------------acaaatagac**

**Citrus_cavaleriei** **t-------------------------------------------------acaaatagac**

**Citrus_hongheensis** **t-------------------------------------------------acaaatagac**

**Citrus_limon** **t-------------------------------------------------acaaatagac**

**Citrus_maxima** **t-------------------------------------------------acaaatagac**

**Citrus_medica** **t-------------------------------------------------acaaatagac**

**Citrus_polytrifolia** **t-------------------------------------------------acaaatagac**

**Citrus_reticulata** **t-------------------------------------------------acaaatagac**

**Citrus_sinensis** **t-------------------------------------------------acaaatagac**

**Fortunella_bawangica_1** **t-------------------------------------------------acaaatagac**

**Fortunella_bawangica_2** **t-------------------------------------------------acaaatagac**

**Fortunella_hindsii_1** **t-------------------------------------------------acaaatagac**

**Fortunella_hindsii_2** **t-------------------------------------------------acaaatagac**

**Fortunella_hindsii_3** **t-------------------------------------------------acaaatagac**

**Fortunella_japonica_1** **t-------------------------------------------------acaaatagac**

**Fortunella_japonica_2** **t-------------------------------------------------acaaatagac**

**Fortunella_japonica_3** **t-------------------------------------------------acaaatagac**

**Fortunella_japonica_4** **t-------------------------------------------------acaaatagac**

**Fortunella_japonica_5** **t-------------------------------------------------acaaatagac**

**Fortunella_japonica_6** **t-------------------------------------------------acaaatagac**

**Fortunella_japonica_7** **t-------------------------------------------------acaaatagac**

**Fortunella_japonica_8** **t-------------------------------------------------acaaatagac**

**Fortunella_japonica_9** **t-------------------------------------------------acaaatagac**

**Fortunella_japonica_10** **t-------------------------------------------------acaaatagac**

**Fortunella_japonica_11** **t-------------------------------------------------acaaatagac**

**Fortunella_japonica_12** **t-------------------------------------------------acaaatagac**

**Fortunella_margarita_1** **t-------------------------------------------------acaaatagac**

**Fortunella_margarita_2** **t-------------------------------------------------acaaatagac**

**Fortunella_venosa_1** **t-------------------------------------------------acaaatagac**

**Fortunella_venosa_2** **t-------------------------------------------------acaaatagac**

**Fortunella_venosa_3** **t-------------------------------------------------acaaatagac**

32650	32660	32670	32680	32690	32700

....|....|....|....|....|....|....|....|....|....|....|....|

**Atalantia_kwangtungens** **gaagcaaagaaatagaattgagatactatgtatatattaacatgtataagg------atc**

**Citrus_reticulata_1** **gaagcaaagaaatagaattgagatactatgtatctattaacatatataaggatccatatc**

**Citrus_reticulata_2** **gaagcaaagaaatagaattgagatactatgtatctattaacatatataaggatccatatc**

**Citrus_trifoliata_3** **gaagcaaagaaatagaattgagatactatgtatctattaacatgtataaggatccatatc**

**Citrus_aurantiifolia** **gaagcaaagaaatagaattgagatactatgtatctattaacatgtataaggatccatatc**

**Citrus_aurantium** **gaagcaaagaaatagaattgagatactatgtatctattaacatgtataaggatccatatc**

**Citrus_cavaleriei** **gaagcaaagaaatagaattgagatactatgtatctattaacatgtataaggatccatatc**

**Citrus_hongheensis** **gaagcaaagaaatagaattgagatactatgtatctattaacatgtataaggatccatatc**

**Citrus_limon** **gaagcaaagaaatagaattgagatactatgtatctattaacatgtataaggatccatatc**

**Citrus_maxima** **gaagcaaagaaatagaattgagatactatgtatctattaacatgtataaggatccatatc**

**Citrus_medica** **gaagcaaagaaatagaattgagatactatgtatctattaacatgtataaggatccatatc**

**Citrus_polytrifolia** **gaagcaaagaaatagaattgagatactatgtatctattaacatgtataaggatccatatc**

**Citrus_reticulata** **gaagcaaagaaatagaattgagatactatgtatctattaacatatataaggatccatatc**

**Citrus_sinensis** **gaagcaaagaaatagaattgagatactatgtatctattaacatgtataaggatccatatc**

**Fortunella_bawangica_1** **gaagcaaagaaatagaattgagatactatgtatctattaacatgtataaggatccatatc**

**Fortunella_bawangica_2** **gaagcaaagaaatagaattgagatactatgtatctattaacatgtataaggatccatatc**

**Fortunella_hindsii_1** **gaagcaaagaaatagaattgagatactatgtatctattaacatgtataaggatccatatc**

**Fortunella_hindsii_2** **gaagcaaagaaatagaattgagatactatgtatctattaacatgtataaggatccatatc**

**Fortunella_hindsii_3** **gaagcaaagaaatagaattgagatactatgtatctattaacatgtataaggatccatatc**

**Fortunella_japonica_1** **gaagcaaagaaatagaattgagatactatgtatctattaacatgtataaggatccatatc**

**Fortunella_japonica_2** **gaagcaaagaaatagaattgagatactatgtatctattaacatgtataaggatccatatc**

**Fortunella_japonica_3** **gaagcaaagaaatagaattgagatactatgtatctattaacatgtataaggatccatatc**

**Fortunella_japonica_4** **gaagcaaagaaatagaattgagatactatgtatctattaacatgtataaggatccatatc**

**Fortunella_japonica_5** **gaagcaaagaaatagaattgagatactatgtatctattaacatgtataaggatccatatc**

**Fortunella_japonica_6** **gaagcaaagaaatagaattgagatactatgtatctattaacatgtataaggatccatatc**

**Fortunella_japonica_7** **gaagcaaagaaatagaattgagatactatgtatctattaacatgtataaggatccatatc**

**Fortunella_japonica_8** **gaagcaaagaaatagaattgagatactatgtatctattaacatgtataaggatccatatc**

**Fortunella_japonica_9** **gaagcaaagaaatagaattgagatactatgtatctattaacatgtataaggatccatatc**

**Fortunella_japonica_10** **gaagcaaagaaatagaattgagatactatgtatctattaacatgtataaggatccatatc**

**Fortunella_japonica_11** **gaagcaaagaaatagaattgagatactatgtatctattaacatgtataaggatccatatc**

**Fortunella_japonica_12** **gaagcaaagaaatagaattgagatactatgtatctattaacatgtataaggatccatatc**

**Fortunella_margarita_1** **gaagcaaagaaatagaattgagatactatgtatctattaacatgtataaggatccatatc**

**Fortunella_margarita_2** **gaagcaaagaaatagaattgagatactatgtatctattaacatgtataaggatccatatc**

**Fortunella_venosa_1** **gaagcaaagaaatagaattgagatactatgtatctattaacatgtataaggatccatatc**

**Fortunella_venosa_2** **gaagcaaagaaatagaattgagatactatgtatctattaacatgtataaggatccatatc**

**Fortunella_venosa_3** **gaagcaaagaaatagaattgagatactatgtatctattaacatgtataaggatccatatc**

32710	32720	32730	32740	32750	32760

....|....|....|....|....|....|....|....|....|....|....|....|

**Atalantia_kwangtungens** **catattgtagattgatctctattcagtttctatctttatacat--gaaaataataaaaat**

**Citrus_reticulata_1** **catattgtagattgatctctattcagtttctatctttatacat--aaaaataataaaaat**

**Citrus_reticulata_2** **catattgtagattgatctctattcagtttctatctttatacat--aaaaataataaaaat**

**Citrus_trifoliata_3** **catattgtagattgatctctattcagtttctatctttatacat--aaaaataataaaaat**

**Citrus_aurantiifolia** **catattgtagattgatctctattcagtttctatctttatacat--aaaaaaaataaaaat**

**Citrus_aurantium** **catattgtagattgatctctattcagtttctatctttatacataaaaaaaaaataaaaat**

**Citrus_cavaleriei** **catattgtagattgatctctattcagtttctatctttctacat--aaaaataataaaaat**

**Citrus_hongheensis** **catattgtagattgatctctattcagtttctatctttatacat--aaaaaaaataaaaat**

**Citrus_limon** **catattgtagattgatctctattcagtttctatctttatacataaaaaaaaaataaaaat**

**Citrus_maxima** **catattgtagattgatctctattcagtttctatctttatacat-aaaaaaaaataaaaat**

**Citrus_medica** **catattgtagattgatctctattcagtttctatctttatacat--aaaaataataaaaat**

**Citrus_polytrifolia** **catattgtagattgatctctattcagtttctatctttatacat--aaaaataataaaaat**

**Citrus_reticulata** **catattgtagattgatctctattcagtttctatctttatacat--aaaaataataaaaat**

**Citrus_sinensis** **catattgtagattgatctctattcagtttctatctttatacat-aaaaaaaaataaaaat**

**Fortunella_bawangica_1** **catattgtagattgatctctattcagtttctatctttatacat--aaaaataataaaaat**

**Fortunella_bawangica_2** **catattgtagattgatctctattcagtttctatctttatacat--aaaaataataaaaat**

**Fortunella_hindsii_1** **catattgtagattgatctctattcagtttctatctttatacat--aaaaataataaaaat**

**Fortunella_hindsii_2** **catattgtagattgatctctattcagtttctatctttatacat--aaaaataataaaaat**

**Fortunella_hindsii_3** **catattgtagattgatctctattcagtttctatctttatacat--aaaaataataaaaat**

**Fortunella_japonica_1** **catattgtagattgatctctattcagtttctatctttatacat--aaaaataataaaaat**

**Fortunella_japonica_2** **catattgtagattgatctctattcagtttctatctttatacat--aaaaataataaaaat**

**Fortunella_japonica_3** **catattgtagattgatctctattcagtttctatctttatacat--aaaaataataaaaat**

**Fortunella_japonica_4** **catattgtagattgatctctattcagtttctatctttatacat--aaaaataataaaaat**

**Fortunella_japonica_5** **catattgtagattgatctctattcagtttctatctttatacat--aaaaataataaaaat**

**Fortunella_japonica_6** **catattgtagattgatctctattcagtttctatctttatacat--aaaaataataaaaat**

**Fortunella_japonica_7** **catattgtagattgatctctattcagtttctatctttatacat--aaaaataataaaaat**

**Fortunella_japonica_8** **catattgtagattgatctctattcagtttctatctttatacat--aaaaataataaaaat**

**Fortunella_japonica_9** **catattgtagattgatctctattcagtttctatctttatacat--aaaaataataaaaat**

**Fortunella_japonica_10** **catattgtagattgatctctattcagtttctatctttatacat--aaaaataataaaaat**

**Fortunella_japonica_11** **catattgtagattgatctctattcagtttctatctttatacat--aaaaataataaaaat**

**Fortunella_japonica_12** **catattgtagattgatctctattcagtttctatctttatacat--aaaaataataaaaat**

**Fortunella_margarita_1** **catattgtagattgatctctattcagtttctatctttatacat--aaaaataataaaaat**

**Fortunella_margarita_2** **catattgtagattgatctctattcagtttctatctttatacat--aaaaataataaaaat**

**Fortunella_venosa_1** **catattgtagattgatctctattcagtttctatctttatacat--aaaaataataaaaat**

**Fortunella_venosa_2** **catattgtagattgatctctattcagtttctatctttatacat--aaaaataataaaaat**

**Fortunella_venosa_3** **catattgtagattgatctctattcagtttctatctttatacat--aaaaataataaaaat**

32770	32780	32790	32800	32810	32820

....|....|....|....|....|....|....|....|....|....|....|....|

**Atalantia_kwangtungens** **agatagtatggtagaaagattcatatatgaatctttctaccatactatcgaatctcatag**

**Citrus_reticulata_1** **agatagtatggtagaaagattcatatatgaatctttctaccatactatcgaatctcatag**

**Citrus_reticulata_2** **agatagtatggtagaaagattcatatatgaatctttctaccatactatcgaatctcatag**

**Citrus_trifoliata_3** **agatagtatggtagaaagattcatatatgaatctttctaccatactatctaatctcatag**

**Citrus_aurantiifolia** **agatagtatggtagaaagattcatatatgaatctttctaccatactatcgaatctcatag**

**Citrus_aurantium** **agatagtatggtagaaagattcatatatgaatctttctaccatactatcgaatctcatag**

**Citrus_cavaleriei** **cgatagtatggtagaaagattcatatatgaatctttctaccatactatcgaatctcatag**

**Citrus_hongheensis** **agatagtatggtagaaagattcatatatgaatctttctaccatactatcgaatctcatag**

**Citrus_limon** **agatagtatggtagaaagattcatatatgaatctttctaccatactatcgaatctcatag**

**Citrus_maxima** **agatagtatggtagaaagattcatatatgaatctttctaccatactatcgaatctcatag**

**Citrus_medica** **agatagtatggtagaaagattcatatatgaatctttctaccatactatcgaatctcatag**

**Citrus_polytrifolia** **agatagtatggtagaaagattcatatatgaatctttctaccatactatctaatctcatag**

**Citrus_reticulata** **agatagtatggtagaaagattcatatatgaatctttctaccatactatcgaatctcatag**

**Citrus_sinensis** **agatagtatggtagaaagattcatatatgaatctttctaccatactatcgaatctcatag**

**Fortunella_bawangica_1** **agatagtatggtagaaagattcatatatgaatctttctaccatactatcgaatctcatag**

**Fortunella_bawangica_2** **agatagtatggtagaaagattcatatatgaatctttctaccatactatcgaatctcatag**

**Fortunella_hindsii_1** **agatagtatggtagaaagattcatatatgaatctttctaccatactatcgaatctcatag**

**Fortunella_hindsii_2** **agatagtatggtagaaagattcatatatgaatctttctaccatactatcgaatctcatag**

**Fortunella_hindsii_3** **agatagtatggtagaaagattcatatatgaatctttctaccatactatcgaatctcatag**

**Fortunella_japonica_1** **agatagtatggtagaaagattcatatatgaatctttctaccatactatcgaatctcatag**

**Fortunella_japonica_2** **agatagtatggtagaaagattcatatatgaatctttctaccatactatcgaatctcatag**

**Fortunella_japonica_3** **agatagtatggtagaaagattcatatatgaatctttctaccatactatcgaatctcatag**

**Fortunella_japonica_4** **agatagtatggtagaaagattcatatatgaatctttctaccatactatcgaatctcatag**

**Fortunella_japonica_5** **agatagtatggtagaaagattcatatatgaatctttctaccatactatcgaatctcatag**

**Fortunella_japonica_6** **agatagtatggtagaaagattcatatatgaatctttctaccatactatcgaatctcatag**

**Fortunella_japonica_7** **agatagtatggtagaaagattcatatatgaatctttctaccatactatcgaatctcatag**

**Fortunella_japonica_8** **agatagtatggtagaaagattcatatatgaatctttctaccatactatcgaatctcatag**

**Fortunella_japonica_9** **agatagtatggtagaaagattcatatatgaatctttctaccatactatcgaatctcatag**

**Fortunella_japonica_10** **agatagtatggtagaaagattcatatatgaatctttctaccatactatcgaatctcatag**

**Fortunella_japonica_11** **agatagtatggtagaaagattcatatatgaatctttctaccatactatcgaatctcatag**

**Fortunella_japonica_12** **agatagtatggtagaaagattcatatatgaatctttctaccatactatcgaatctcatag**

**Fortunella_margarita_1** **agatagtatggtagaaagattcatatatgaatctttctaccatactatcgaatctcatag**

**Fortunella_margarita_2** **agatagtatggtagaaagattcatatatgaatctttctaccatactatcgaatctcatag**

**Fortunella_venosa_1** **agatagtatggtagaaagattcatatatgaatctttctaccatactatcgaatctcatag**

**Fortunella_venosa_2** **agatagtatggtagaaagattcatatatgaatctttctaccatactatcgaatctcatag**

**Fortunella_venosa_3** **agatagtatggtagaaagattcatatatgaatctttctaccatactatcgaatctcatag**

32830	32840	32850	32860	32870	32880

....|....|....|....|....|....|....|....|....|....|....|....|

**Atalantia_kwangtungens** **gctactgctgattctagtccgttcgtttcatttaagacttaagactaagacatgaaattg**

**Citrus_reticulata_1** **gctactgctgattctagtccgttggtttcatttaagactaa------------------g**

**Citrus_reticulata_2** **gctactgctgattctagtccgttggtttcatttaagactaa------------------g**

**Citrus_trifoliata_3** **gctactgctgattctagtccgttggtttcatttaagactaa------------------g**

**Citrus_aurantiifolia** **gctactgctgattctagtccgttggtttcatttaagactaa------------------g**

**Citrus_aurantium** **gctactgctgattctagtccgttggtttcatttaagactaa------------------g**

**Citrus_cavaleriei** **gctactgctgattctagtccgttggtttcatttaagactaa------------------g**

**Citrus_hongheensis** **gctactgctgattctagtccgttggtttcatttaagactaa------------------g**

**Citrus_limon** **gctactgctgattctagtccgttggtttcatttaagactaa------------------g**

**Citrus_maxima** **gctactgctgattctagtccgttggtttcatttaagactaa------------------g**

**Citrus_medica** **gctactgctgattctagtccgttggtttcatttaagactaa------------------g**

**Citrus_polytrifolia** **gctactgctgattctagtccgttggtttcatttaagactaa------------------g**

**Citrus_reticulata** **gctactgctgattctagtccgttggtttcatttaagactaa------------------g**

**Citrus_sinensis** **gctactgctgattctagtccgttggtttcatttaagactaa------------------g**

**Fortunella_bawangica_1** **gctactgctgattctagtccgttggtttcatttaagactaa------------------g**

**Fortunella_bawangica_2** **gctactgctgattctagtccgttggtttcatttaagactaa------------------g**

**Fortunella_hindsii_1** **gctactgctgattctagtccgttggtttcatttaagactaa------------------g**

**Fortunella_hindsii_2** **gctactgctgattctagtccgttggtttcatttaagactaa------------------g**

**Fortunella_hindsii_3** **gctactgctgattctagtccgttggtttcatttaagactaa------------------g**

**Fortunella_japonica_1** **gctactgctgattctagtccgttggtttcatttaagactaa------------------g**

**Fortunella_japonica_2** **gctactgctgattctagtccgttggtttcatttaagactaa------------------g**

**Fortunella_japonica_3** **gctactgctgattctagtccgttggtttcatttaagactaa------------------g**

**Fortunella_japonica_4** **gctactgctgattctagtccgttggtttcatttaagactaa------------------g**

**Fortunella_japonica_5** **gctactgctgattctagtccgttggtttcatttaagactaa------------------g**

**Fortunella_japonica_6** **gctactgctgattctagtccgttggtttcatttaagactaa------------------g**

**Fortunella_japonica_7** **gctactgctgattctagtccgttggtttcatttaagactaa------------------g**

**Fortunella_japonica_8** **gctactgctgattctagtccgttggtttcatttaagactaa------------------g**

**Fortunella_japonica_9** **gctactgctgattctagtccgttggtttcatttaagactaa------------------g**

**Fortunella_japonica_10** **gctactgctgattctagtccgttggtttcatttaagactaa------------------g**

**Fortunella_japonica_11** **gctactgctgattctagtccgttggtttcatttaagactaa------------------g**

**Fortunella_japonica_12** **gctactgctgattctagtccgttggtttcatttaagactaa------------------g**

**Fortunella_margarita_1** **gctactgctgattctagtccgttggtttcatttaagactaa------------------g**

**Fortunella_margarita_2** **gctactgctgattctagtccgttggtttcatttaagactaa------------------g**

**Fortunella_venosa_1** **gctactgctgattctagtccgttggtttcatttaagactaa------------------g**

**Fortunella_venosa_2** **gctactgctgattctagtccgttggtttcatttaagactaa------------------g**

**Fortunella_venosa_3** **gctactgctgattctagtccgttggtttcatttaagactaa------------------g**

32890	32900	32910	32920	32930	32940

....|....|....|....|....|....|....|....|....|....|....|....|

**Atalantia_kwangtungens** **acatgaaattgaaa---tttttttcttaaatccttga----------------taagaac**

**Citrus_reticulata_1** **acatgaaattgaat---tttttttcttaaatccttgataagaact-------ataagaac**

**Citrus_reticulata_2** **acatgaaattgaat---tttttttcttaaatccttgataagaact-------ataagaac**

**Citrus_trifoliata_3** **acatgaaattgaaa---tttttttcttaaatacttgataagaactaagaaggataagaac**

**Citrus_aurantiifolia** **acatgaaattgaaa---tttttttcttaaatccgtga----------------taagaac**

**Citrus_aurantium** **acatgaaattgaaa---tttgtttcttaaatccttga----------------taagaac**

**Citrus_cavaleriei** **acatgaaattgaaa---tttttttcttaaatccttga----------------taagaac**

**Citrus_hongheensis** **acatgaaattgaaa---tttgtttcttaaatccttga----------------taagaac**

**Citrus_limon** **acatgaaattgaaa---tttgtttcttaaatccttga----------------taagaac**

**Citrus_maxima** **acatgaaattgaaa---tttgtttcttaaatccttga----------------taagaac**

**Citrus_medica** **acatgaaattgaaattttttttttcttaaatccttga----------------taagaac**

**Citrus_polytrifolia** **acatgaaattgaaa---tttttttcttaaatccttgataagaactaagaaggataagaac**

**Citrus_reticulata** **acatgaaattgaat---tttttttcttaaatccttgataagaact-------ataagaac**

**Citrus_sinensis** **acatgaaattgaaa---tttgtttcttaaatccttga----------------taagaac**

**Fortunella_bawangica_1** **acatgaaattgaaa---tttttttcttaaatccttga----------------taagaac**

**Fortunella_bawangica_2** **acatgaaattgaaa---tttttttcttaaatccttga----------------taagaac**

**Fortunella_hindsii_1** **acatgaaattgaaa---tttttttcttaaatccttga----------------taagaac**

**Fortunella_hindsii_2** **acatgaaattgaaa---tttttttcttaaatccttga----------------taagaac**

**Fortunella_hindsii_3** **acatgaaattgaaa---tttttttcttaaatccttga----------------taagaac**

**Fortunella_japonica_1** **acatgaaattgaaa---tttttttcttaaatccttga----------------taagaac**

**Fortunella_japonica_2** **acatgaaattgaaa---tttttttcttaaatccttga----------------taagaac**

**Fortunella_japonica_3** **acatgaaattgaaa---tttttttcttaaatccttga----------------taagaac**

**Fortunella_japonica_4** **acatgaaattgaaa---tttttttcttaaatccttga----------------taagaac**

**Fortunella_japonica_5** **acatgaaattgaaa---tttttttcttaaatccttga----------------taagaac**

**Fortunella_japonica_6** **acatgaaattgaaa---tttttttcttaaatccttga----------------taagaac**

**Fortunella_japonica_7** **acatgaaattgaaa---tttttttcttaaatccttga----------------taagaac**

**Fortunella_japonica_8** **acatgaaattgaaa---tttttttcttaaatccttga----------------taagaac**

**Fortunella_japonica_9** **acatgaaattgaaa---tttttttcttaaatccttga----------------taagaac**

**Fortunella_japonica_10** **acatgaaattgaaa---tttttttcttaaatccttga----------------taagaac**

**Fortunella_japonica_11** **acatgaaattgaaa---tttttttcttaaatccttga----------------taagaac**

**Fortunella_japonica_12** **acatgaaattgaaa---tttttttcttaaatccttga----------------taagaac**

**Fortunella_margarita_1** **acatgaaattgaaa---tttttttcttaaatccttga----------------taagaac**

**Fortunella_margarita_2** **acatgaaattgaaa---tttttttcttaaatccttga----------------taagaac**

**Fortunella_venosa_1** **acatgaaattgaaa---tttttttcttaaatccttga----------------taagaac**

**Fortunella_venosa_2** **acatgaaattgaaa---tttttttcttaaatccttga----------------taagaac**

**Fortunella_venosa_3** **acatgaaattgaaa---tttttttcttaaatccttga----------------taagaac**

32950	32960	32970	32980	32990	33000

....|....|....|....|....|....|....|....|....|....|....|....|

**Atalantia_kwangtungens** **taagaagtcaagtttcggtcaaattaatcactttgactgaccgtttttacatatattata**

**Citrus_reticulata_1** **taagaagtcaagtttcgttcaaattaatcactttgactgaccgtttttacgtatattata**

**Citrus_reticulata_2** **taagaagtcaagtttcgttcaaattaatcactttgactgaccgtttttacgtatattata**

**Citrus_trifoliata_3** **taagaagtcaagtttcgttcaaattaatcactttgactgaccgtttttacgtatattata**

**Citrus_aurantiifolia** **taagaagtcaagtttcgttcaaattaatcactttgactgaccgtttttacgtatattata**

**Citrus_aurantium** **taagaagtcaagtttcgttcaaattaatcactttgactgaccgtttttacgtatattata**

**Citrus_cavaleriei** **taagaagtcaagtttcgttcaaattaatcactttgactgaccgtttttacgtatattata**

**Citrus_hongheensis** **taagaagtcaagtttcgttcaaattaatcactttgactgaccgtttttacgtatattata**

**Citrus_limon** **taagaagtcaagtttcgttcaaattaatcactttgactgaccgtttttacgtatattata**

**Citrus_maxima** **taagaagtcaagtttcgttcaaattaatcactttgactgaccgtttttacgtatattata**

**Citrus_medica** **taagaagtcaagtttcgttcaaattaatcactttgactgaccgtttttacgtatattata**

**Citrus_polytrifolia** **taagaagtcaagtttcgttcaaattaatcactttgactgaccgtttttacgtatattata**

**Citrus_reticulata** **taagaagtcaagtttcgttcaaattaatcactttgactgaccgtttttacgtatattata**

**Citrus_sinensis** **taagaagtcaagtttcgttcaaattaatcactttgactgaccgtttttacgtatattata**

**Fortunella_bawangica_1** **taagaagtcaagtttcgttcaaattaatcactttgactgaccgtttttacgtatattata**

**Fortunella_bawangica_2** **taagaagtcaagtttcgttcaaattaatcactttgactgaccgtttttacgtatattata**

**Fortunella_hindsii_1** **taagaagtcaagtttcgttcaaattaatcactttgactgaccgtttttacgtatattata**

**Fortunella_hindsii_2** **taagaagtcaagtttcgttcaaattaatcactttgactgaccgtttttacgtatattata**

**Fortunella_hindsii_3** **taagaagtcaagtttcgttcaaattaatcactttgactgaccgtttttacgtatattata**

**Fortunella_japonica_1** **taagaagtcaagtttcgttcaaattaatcactttgactgaccgtttttacgtatattata**

**Fortunella_japonica_2** **taagaagtcaagtttcgttcaaattaatcactttgactgaccgtttttacgtatattata**

**Fortunella_japonica_3** **taagaagtcaagtttcgttcaaattaatcactttgactgaccgtttttacgtatattata**

**Fortunella_japonica_4** **taagaagtcaagtttcgttcaaattaatcactttgactgaccgtttttacgtatattata**

**Fortunella_japonica_5** **taagaagtcaagtttcgttcaaattaatcactttgactgaccgtttttacgtatattata**

**Fortunella_japonica_6** **taagaagtcaagtttcgttcaaattaatcactttgactgaccgtttttacgtatattata**

**Fortunella_japonica_7** **taagaagtcaagtttcgttcaaattaatcactttgactgaccgtttttacgtatattata**

**Fortunella_japonica_8** **taagaagtcaagtttcgttcaaattaatcactttgactgaccgtttttacgtatattata**

**Fortunella_japonica_9** **taagaagtcaagtttcgttcaaattaatcactttgactgaccgtttttacgtatattata**

**Fortunella_japonica_10** **taagaagtcaagtttcgttcaaattaatcactttgactgaccgtttttacgtatattata**

**Fortunella_japonica_11** **taagaagtcaagtttcgttcaaattaatcactttgactgaccgtttttacgtatattata**

**Fortunella_japonica_12** **taagaagtcaagtttcgttcaaattaatcactttgactgaccgtttttacgtatattata**

**Fortunella_margarita_1** **taagaagtcaagtttcgttcaaattaatcactttgactgaccgtttttacgtatattata**

**Fortunella_margarita_2** **taagaagtcaagtttcgttcaaattaatcactttgactgaccgtttttacgtatattata**

**Fortunella_venosa_1** **taagaagtcaagtttcgttcaaattaatcactttgactgaccgtttttacgtatattata**

**Fortunella_venosa_2** **taagaagtcaagtttcgttcaaattaatcactttgactgaccgtttttacgtatattata**

**Fortunella_venosa_3** **taagaagtcaagtttcgttcaaattaatcactttgactgaccgtttttacgtatattata**

33010	33020	33030	33040	33050	33060

....|....|....|....|....|....|....|....|....|....|....|....|

**Atalantia_kwangtungens** **agcaaaaacgcagtaggaactagaacgaacagtgtagtagcaataaatgcgagaatattt**

**Citrus_reticulata_1** **agcaaaaacgcagtaggaactagaacgaacagtgtagtagcaataaatgcgagaatattt**

**Citrus_reticulata_2** **agcaaaaacgcagtaggaactagaacgaacagtgtagtagcaataaatgcgagaatattt**

**Citrus_trifoliata_3** **agcaaaaacgcagtaggaactagaacgaacagtgtagtagcaataaatgcgagaatattt**

**Citrus_aurantiifolia** **agcaaaaacgcagtaggaactagaacgaacagtgtagtagcaataaatgcgagaatattt**

**Citrus_aurantium** **agcaaaaacgcagtaggaactagaacgaacagtgtagtagcaataaatgcgagaatattt**

**Citrus_cavaleriei** **agcaaaaacgcagtaggaactagaacgaacagtgtagtagcaataaatgcgagaatattt**

**Citrus_hongheensis** **agcaaaaacgcagtaggaactagaacgaacagtgtagtagcaataaatgcgagaatattt**

**Citrus_limon** **agcaaaaacgcagtaggaactagaacgaacagtgtagtagcaataaatgcgagaatattt**

**Citrus_maxima** **agcaaaaacgcagtaggaactagaacgaacagtgtagtagcaataaatgcgagaatattt**

**Citrus_medica** **agcaaaaacgcagtaggaactagaacgaacagtgtagtagcaataaatgcgagaatattt**

**Citrus_polytrifolia** **agcaaaaacgcagtaggaactagaacgaacagtgtagtagcaataaatgcgagaatattt**

**Citrus_reticulata** **agcaaaaacgcagtaggaactagaacgaacagtgtagtagcaataaatgcgagaatattt**

**Citrus_sinensis** **agcaaaaacgcagtaggaactagaacgaacagtgtagtagcaataaatgcgagaatattt**

**Fortunella_bawangica_1** **agcaaaaacgcagtaggaactagaacgaacagtgtagtagcaataaatgcgagaatattt**

**Fortunella_bawangica_2** **agcaaaaacgcagtaggaactagaacgaacagtgtagtagcaataaatgcgagaatattt**

**Fortunella_hindsii_1** **agcaaaaacgcagtaggaactagaacgaacagtgtagtagcaataaatgcgagaatattt**

**Fortunella_hindsii_2** **agcaaaaacgcagtaggaactagaacgaacagtgtagtagcaataaatgcgagaatattt**

**Fortunella_hindsii_3** **agcaaaaacgcagtaggaactagaacgaacagtgtagtagcaataaatgcgagaatattt**

**Fortunella_japonica_1** **agcaaaaacgcagtaggaactagaacgaacagtgtagtagcaataaatgcgagaatattt**

**Fortunella_japonica_2** **agcaaaaacgcagtaggaactagaacgaacagtgtagtagcaataaatgcgagaatattt**

**Fortunella_japonica_3** **agcaaaaacgcagtaggaactagaacgaacagtgtagtagcaataaatgcgagaatattt**

**Fortunella_japonica_4** **agcaaaaacgcagtaggaactagaacgaacagtgtagtagcaataaatgcgagaatattt**

**Fortunella_japonica_5** **agcaaaaacgcagtaggaactagaacgaacagtgtagtagcaataaatgcgagaatattt**

**Fortunella_japonica_6** **agcaaaaacgcagtaggaactagaacgaacagtgtagtagcaataaatgcgagaatattt**

**Fortunella_japonica_7** **agcaaaaacgcagtaggaactagaacgaacagtgtagtagcaataaatgcgagaatattt**

**Fortunella_japonica_8** **agcaaaaacgcagtaggaactagaacgaacagtgtagtagcaataaatgcgagaatattt**

**Fortunella_japonica_9** **agcaaaaacgcagtaggaactagaacgaacagtgtagtagcaataaatgcgagaatattt**

**Fortunella_japonica_10** **agcaaaaacgcagtaggaactagaacgaacagtgtagtagcaataaatgcgagaatattt**

**Fortunella_japonica_11** **agcaaaaacgcagtaggaactagaacgaacagtgtagtagcaataaatgcgagaatattt**

**Fortunella_japonica_12** **agcaaaaacgcagtaggaactagaacgaacagtgtagtagcaataaatgcgagaatattt**

**Fortunella_margarita_1** **agcaaaaacgcagtaggaactagaacgaacagtgtagtagcaataaatgcgagaatattt**

**Fortunella_margarita_2** **agcaaaaacgcagtaggaactagaacgaacagtgtagtagcaataaatgcgagaatattt**

**Fortunella_venosa_1** **agcaaaaacgcagtaggaactagaacgaacagtgtagtagcaataaatgcgagaatattt**

**Fortunella_venosa_2** **agcaaaaacgcagtaggaactagaacgaacagtgtagtagcaataaatgcgagaatattt**

**Fortunella_venosa_3** **agcaaaaacgcagtaggaactagaacgaacagtgtagtagcaataaatgcgagaatattt**

33070	33080	33090	33100	33110	33120

....|....|....|....|....|....|....|....|....|....|....|....|

**Atalantia_kwangtungens** **acttccatagtctcatcgtttgg----ttttttttacttcgcaataactcgggatttaat**

**Citrus_reticulata_1** **acttccatagtctcatcgtttggttttttttttttacttcgcaataactcgggatttaat**

**Citrus_reticulata_2** **acttccatagtctcatcgtttggttttttttttttacttcgcaataactcgggatttaat**

**Citrus_trifoliata_3** **acttccatagtctcatcgtttgg--ttttttttttacttcgcaataactcgggatttaat**

**Citrus_aurantiifolia** **acttccatagtctcatcgtttgg-tttttttttttacttcgcaataactcgggatttaat**

**Citrus_aurantium** **acttccatagtctcatcgtttgg--ttttttttttacttcgcaataactcgggatttaat**

**Citrus_cavaleriei** **acttccatagtctcatcgtttgg--ttttttttttacttcgcaataactcgggatttaat**

**Citrus_hongheensis** **acttccatagtctcatcgtttgg-tttttttttttacttcgcaataactcgggatttaat**

**Citrus_limon** **acttccatagtctcatcgtttgg--ttttttttttacttcgcaataactcgggatttaat**

**Citrus_maxima** **acttccatagtctcatcgtttgg--ttttttttttacttcgcaataactcgggatttaat**

**Citrus_medica** **acttccatagtctcatcgtttgg-tttttttttttacttcgcaataactcgggatttaat**

**Citrus_polytrifolia** **acttccatagtctcatcgtttgg---tttttttttacttcgcaataactcgggatttaat**

**Citrus_reticulata** **acttccatagtctcatcgtttggttttttttttttacttcgcaataactcgggatttaat**

**Citrus_sinensis** **acttccatagtctcatcgtttgg--ttttttttttacttcgcaataactcgggatttaat**

**Fortunella_bawangica_1** **acttccatagtctcatcgtttgg---tttttttttacttcgcaataactcgggatttaat**

**Fortunella_bawangica_2** **acttccatagtctcatcgtttgg---tttttttttacttcgcaataactcgggatttaat**

**Fortunella_hindsii_1** **acttccatagtctcatcgtttgg---tttttttttacttcgcaataactcgggatttaat**

**Fortunella_hindsii_2** **acttccatagtctcatcgtttgg---tttttttttacttcgcaataactcgggatttaat**

**Fortunella_hindsii_3** **acttccatagtctcatcgtttgg---tttttttttacttcgcaataactcgggatttaat**

**Fortunella_japonica_1** **acttccatagtctcatcgtttgg---tttttttttacttcgcaagaactcgggatttaat**

**Fortunella_japonica_2** **acttccatagtctcatcgtttgg---tttttttttacttcgcaagaactcgggatttaat**

**Fortunella_japonica_3** **acttccatagtctcatcgtttgg---tttttttttacttcgcaagaactcgggatttaat**

**Fortunella_japonica_4** **acttccatagtctcatcgtttgg---tttttttttacttcgcaagaactcgggatttaat**

**Fortunella_japonica_5** **acttccatagtctcatcgtttgg---tttttttttacttcgcaagaactcgggatttaat**

**Fortunella_japonica_6** **acttccatagtctcatcgtttgg---tttttttttacttcgcaagaactcgggatttaat**

**Fortunella_japonica_7** **acttccatagtctcatcgtttgg---tttttttttacttcgcaagaactcgggatttaat**

**Fortunella_japonica_8** **acttccatagtctcatcgtttgg---tttttttttacttcgcaagaactcgggatttaat**

**Fortunella_japonica_9** **acttccatagtctcatcgtttgg---tttttttttacttcgcaagaactcgggatttaat**

**Fortunella_japonica_10** **acttccatagtctcatcgtttgg---tttttttttacttcgcaataactcgggatttaat**

**Fortunella_japonica_11** **acttccatagtctcatcgtttgg---tttttttttacttcgcaagaactcgggatttaat**

**Fortunella_japonica_12** **acttccatagtctcatcgtttgg---tttttttttacttcgcaagaactcgggatttaat**

**Fortunella_margarita_1** **acttccatagtctcatcgtttgg---tttttttttacttcgcaataactcgggatttaat**

**Fortunella_margarita_2** **acttccatagtctcatcgtttgg---tttttttttacttcgcaataactcgggatttaat**

**Fortunella_venosa_1** **acttccatagtctcatcgtttgg---tttttttttacttcgcaataactcgggatttaat**

**Fortunella_venosa_2** **acttccatagtctcatcgtttgg---tttttttttacttcgcaataactcgggatttaat**

**Fortunella_venosa_3** **acttccatagtctcatcgtttgg---tttttttttacttcgcaataactcgggatttaat**

33130	33140	33150	33160	33170	33180

....|....|....|....|....|....|....|....|....|....|....|....|

**Atalantia_kwangtungens** **cccatagagatgataaccctttcgcttgtaaattcaatgggatgaattacatttcgatgc**

**Citrus_reticulata_1** **cccatagagatgataaccctttcgcttgtaaattcaatgggatgaattacatttcgatga**

**Citrus_reticulata_2** **cccatagagatgataaccctttcgcttgtaaattcaatgggatgaattacatttcgatga**

**Citrus_trifoliata_3** **cccatagagatgataaccctttcgcttgtaaattcaatgggatgaattacatttcgatga**

**Citrus_aurantiifolia** **cccatagagatgataaccctttcgcttgtaaattcaatgggatgaattacatttcgatga**

**Citrus_aurantium** **cccatagagatgataaccctttcgcttgtaaattcaatgggatgaattacatttcgatga**

**Citrus_cavaleriei** **cccatagagatgataaccctttcgcttgtaaattcaatgggatgaattacatttcgatga**

**Citrus_hongheensis** **cccatagagatgataaccctttcgcttgtaaattcaatgggatgaattacatttcgatga**

**Citrus_limon** **cccatagagatgataaccctttcgcttgtaaattcaatgggatgaattacatttcgatga**

**Citrus_maxima** **cccatagagatgataaccctttcgcttgtaaattcaatgggatgaattacatttcgatga**

**Citrus_medica** **cccatagagatgataaccctttcgcttgtaaattcaatgggatgaattacatttcgatga**

**Citrus_polytrifolia** **cccatagagatgataaccctttcgcttgtaaattcaatgggatgaattacatttcgatga**

**Citrus_reticulata** **cccatagagatgataaccctttcgcttgtaaattcaatgggatgaattacatttcgatga**

**Citrus_sinensis** **cccatagagatgataaccctttcgcttgtaaattcaatgggatgaattacatttcgatga**

**Fortunella_bawangica_1** **cccatagagatgataaccctttcgcttgtaaattcaatgggatgaattacatttcgatga**

**Fortunella_bawangica_2** **cccatagagatgataaccctttcgcttgtaaattcaatgggatgaattacatttcgatga**

**Fortunella_hindsii_1** **cccatagagatgataaccctttcgcttgtaaattcaatgggatgaattacatttcgatga**

**Fortunella_hindsii_2** **cccatagagatgataaccctttcgcttgtaaattcaatgggatgaattacatttcgatga**

**Fortunella_hindsii_3** **cccatagagatgataaccctttcgcttgtaaattcaatgggatgaattacatttcgatga**

**Fortunella_japonica_1** **cccatagagatgataaccctttcgcttgtaaattcaatgggatgaattacatttcgatga**

**Fortunella_japonica_2** **cccatagagatgataaccctttcgcttgtaaattcaatgggatgaattacatttcgatga**

**Fortunella_japonica_3** **cccatagagatgataaccctttcgcttgtaaattcaatgggatgaattacatttcgatga**

**Fortunella_japonica_4** **cccatagagatgataaccctttcgcttgtaaattcaatgggatgaattacatttcgatga**

**Fortunella_japonica_5** **cccatagagatgataaccctttcgcttgtaaattcaatgggatgaattacatttcgatga**

**Fortunella_japonica_6** **cccatagagatgataaccctttcgcttgtaaattcaatgggatgaattacatttcgatga**

**Fortunella_japonica_7** **cccatagagatgataaccctttcgcttgtaaattcaatgggatgaattacatttcgatga**

**Fortunella_japonica_8** **cccatagagatgataaccctttcgcttgtaaattcaatgggatgaattacatttcgatga**

**Fortunella_japonica_9** **cccatagagatgataaccctttcgcttgtaaattcaatgggatgaattacatttcgatga**

**Fortunella_japonica_10** **cccatagagatgataaccctttcgcttgtaaattcaatgggatgaattacatttcgatga**

**Fortunella_japonica_11** **cccatagagatgataaccctttcgcttgtaaattcaatgggatgaattacatttcgatga**

**Fortunella_japonica_12** **cccatagagatgataaccctttcgcttgtaaattcaatgggatgaattacatttcgatga**

**Fortunella_margarita_1** **cccatagagatgataaccctttcgcttgtaaattcaatgggatgaattacatttcgatga**

**Fortunella_margarita_2** **cccatagagatgataaccctttcgcttgtaaattcaatgggatgaattacatttcgatga**

**Fortunella_venosa_1** **cccatagagatgataaccctttcgcttgtaaattcaatgggatgaattacatttcgatga**

**Fortunella_venosa_2** **cccatagagatgataaccctttcgcttgtaaattcaatgggatgaattacatttcgatga**

**Fortunella_venosa_3** **cccatagagatgataaccctttcgcttgtaaattcaatgggatgaattacatttcgatga**

33190	33200	33210	33220	33230	33240

....|....|....|....|....|....|....|....|....|....|....|....|

**Atalantia_kwangtungens** **tagcgaatgggatcaatatcatgaattcaatatcatgaataacaatatttgactgtcaag**

**Citrus_reticulata_1** **tagcgaatggga--------------tcaatatcatgaataacaatatttgactgtcaag**

**Citrus_reticulata_2** **tagcgaatggga--------------tcaatatcatgaataacaatatttgactgtcaag**

**Citrus_trifoliata_3** **tagcgaatggga--------------tcaatatcatgaataacaatatttgactgtcaag**

**Citrus_aurantiifolia** **tagcgaatggga--------------tcaatatcatgaataacaatatttgactgtcaag**

**Citrus_aurantium** **tagcgaatggga--------------tcaatatcatgaataacaatatttgactgtcaag**

**Citrus_cavaleriei** **tagcgaatggga--------------tcaatatcatgaataacaatatttgactgtcaag**

**Citrus_hongheensis** **tagcgaatggga--------------tcaatatcatgaataacaatatttgactgtcaag**

**Citrus_limon** **tagcgaatggga--------------tcaatatcatgaataacaatatttgactgtcaag**

**Citrus_maxima** **tagcgaatggga--------------tcaatatcatgaataacaatatttgactgtcaag**

**Citrus_medica** **tagcgaatggga--------------tcaatatcatgaataacaatatttgactgtcaag**

**Citrus_polytrifolia** **tagcgaatggga--------------tcaatatcatgaataacaatatttgactgtcaag**

**Citrus_reticulata** **tagcgaatggga--------------tcaatatcatgaataacaatatttgactgtcaag**

**Citrus_sinensis** **tagcgaatggga--------------tcaatatcatgaataacaatatttgactgtcaag**

**Fortunella_bawangica_1** **tagcgaatggga--------------tcaatatcatgaataacaatatttgactgtcaag**

**Fortunella_bawangica_2** **tagcgaatggga--------------tcaatatcatgaataacaatatttgactgtcaag**

**Fortunella_hindsii_1** **tagcgaatggga--------------tcaatatcatgaataacaatatttgactgtcaag**

**Fortunella_hindsii_2** **tagcgaatggga--------------tcaatatcatgaataacaatatttgactgtcaag**

**Fortunella_hindsii_3** **tagcgaatggga--------------tcaatatcatgaataacaatatttgactgtcaag**

**Fortunella_japonica_1** **tagcgaatggga--------------tcaatatcatgaataacaatatttgactgtcaag**

**Fortunella_japonica_2** **tagcgaatggga--------------tcaatatcatgaataacaatatttgactgtcaag**

**Fortunella_japonica_3** **tagcgaatggga--------------tcaatatcatgaataacaatatttgactgtcaag**

**Fortunella_japonica_4** **tagcgaatggga--------------tcaatatcatgaataacaatatttgactgtcaag**

**Fortunella_japonica_5** **tagcgaatggga--------------tcaatatcatgaataacaatatttgactgtcaag**

**Fortunella_japonica_6** **tagcgaatggga--------------tcaatatcatgaataacaatatttgactgtcaag**

**Fortunella_japonica_7** **tagcgaatggga--------------tcaatatcatgaataacaatatttgactgtcaag**

**Fortunella_japonica_8** **tagcgaatggga--------------tcaatatcatgaataacaatatttgactgtcaag**

**Fortunella_japonica_9** **tagcgaatggga--------------tcaatatcatgaataacaatatttgactgtcaag**

**Fortunella_japonica_10** **tagcgaatggga--------------tcaatatcatgaataacaatatttgactgtcaag**

**Fortunella_japonica_11** **tagcgaatggga--------------tcaatatcatgaataacaatatttgactgtcaag**

**Fortunella_japonica_12** **tagcgaatggga--------------tcaatatcatgaataacaatatttgactgtcaag**

**Fortunella_margarita_1** **tagcgaatggga--------------tcaatatcatgaataacaatatttgactgtcaag**

**Fortunella_margarita_2** **tagcgaatggga--------------tcaatatcatgaataacaatatttgactgtcaag**

**Fortunella_venosa_1** **tagcgaatggga--------------tcaatatcatgaataacaatatttgactgtcaag**

**Fortunella_venosa_2** **tagcgaatggga--------------tcaatatcatgaataacaatatttgactgtcaag**

**Fortunella_venosa_3** **tagcgaatggga--------------tcaatatcatgaataacaatatttgactgtcaag**

33250	33260	33270	33280	33290	33300

....|....|....|....|....|....|....|....|....|....|....|....|

**Atalantia_kwangtungens** **tcgattcatcgtcgagaattcaatagtataacataggcagctcttttatccacacaccaa**

**Citrus_reticulata_1** **tcgattcatcgtcgagaattcaatagtataacataggcagctcttttatccacacaccaa**

**Citrus_reticulata_2** **tcgattcatcgtcgagaattcaatagtataacataggcagctcttttatccacacaccaa**

**Citrus_trifoliata_3** **tcgattcatcgtcgagaattcaatagtataacataggcagctcttttatccacacaccaa**

**Citrus_aurantiifolia** **tcgattcatcgtcgagaattcaatagtataacataggcagctcttttatccacacaccaa**

**Citrus_aurantium** **tcgattcatcgtcgagaattcaatagtataacataggcagctcttttatccacacaccaa**

**Citrus_cavaleriei** **tcgattcatcgtcgagaattcaatagtataacataggcagctcttttatccacacaccaa**

**Citrus_hongheensis** **tcgattcatcgtcgagaattcaatagtataacataggcagctcttttatccacacaccaa**

**Citrus_limon** **tcgattcatcgtcgagaattcaatagtataacataggcagctcttttatccacacaccaa**

**Citrus_maxima** **tcgattcatcgtcgagaattcaatagtataacataggcagctcttttatccacacaccaa**

**Citrus_medica** **tcgattcatcgtcgagaattcaatagtataacataggcagctcttttatccacacaccaa**

**Citrus_polytrifolia** **tcgattcatcgtcgagaattcaatagtataacataggcagctcttttatccacacaccaa**

**Citrus_reticulata** **tcgattcatcgtcgagaattcaatagtataacataggcagctcttttatccacacaccaa**

**Citrus_sinensis** **tcgattcatcgtcgagaattcaatagtataacataggcagctcttttatccacacaccaa**

**Fortunella_bawangica_1** **tcgattcatcgtcgagaattcaatagtataacataggcagctcttttatccacacaccaa**

**Fortunella_bawangica_2** **tcgattcatcgtcgagaattcaatagtataacataggcagctcttttatccacacaccaa**

**Fortunella_hindsii_1** **tcgattcatcgtcgagaattcaatagtataacataggcagctcttttatccacacaccaa**

**Fortunella_hindsii_2** **tcgattcatcgtcgagaattcaatagtataacataggcagctcttttatccacacaccaa**

**Fortunella_hindsii_3** **tcgattcatcgtcgagaattcaatagtataacataggcagctcttttatccacacaccaa**

**Fortunella_japonica_1** **tcgattcatcgtcgagaattcaatagtataacataggcagctcttttatccacacaccaa**

**Fortunella_japonica_2** **tcgattcatcgtcgagaattcaatagtataacataggcagctcttttatccacacaccaa**

**Fortunella_japonica_3** **tcgattcatcgtcgagaattcaatagtataacataggcagctcttttatccacacaccaa**

**Fortunella_japonica_4** **tcgattcatcgtcgagaattcaatagtataacataggcagctcttttatccacacaccaa**

**Fortunella_japonica_5** **tcgattcatcgtcgagaattcaatagtataacataggcagctcttttatccacacaccaa**

**Fortunella_japonica_6** **tcgattcatcgtcgagaattcaatagtataacataggcagctcttttatccacacaccaa**

**Fortunella_japonica_7** **tcgattcatcgtcgagaattcaatagtataacataggcagctcttttatccacacaccaa**

**Fortunella_japonica_8** **tcgattcatcgtcgagaattcaatagtataacataggcagctcttttatccacacaccaa**

**Fortunella_japonica_9** **tcgattcatcgtcgagaattcaatagtataacataggcagctcttttatccacacaccaa**

**Fortunella_japonica_10** **tcgattcatcgtcgagaattcaatagtataacataggcagctcttttatccacacaccaa**

**Fortunella_japonica_11** **tcgattcatcgtcgagaattcaatagtataacataggcagctcttttatccacacaccaa**

**Fortunella_japonica_12** **tcgattcatcgtcgagaattcaatagtataacataggcagctcttttatccacacaccaa**

**Fortunella_margarita_1** **tcgattcatcgtcgagaattcaatagtataacataggcagctcttttatccacacaccaa**

**Fortunella_margarita_2** **tcgattcatcgtcgagaattcaatagtataacataggcagctcttttatccacacaccaa**

**Fortunella_venosa_1** **tcgattcatcgtcgagaattcaatagtataacataggcagctcttttatccacacaccaa**

**Fortunella_venosa_2** **tcgattcatcgtcgagaattcaatagtataacataggcagctcttttatccacacaccaa**

**Fortunella_venosa_3** **tcgattcatcgtcgagaattcaatagtataacataggcagctcttttatccacacaccaa**

33310	33320	33330	33340	33350	33360

....|....|....|....|....|....|....|....|....|....|....|....|

**Atalantia_kwangtungens** **atacaaactttgattcctgattcaatcaaaagaattcctttactttaatactaatacaat**

**Citrus_reticulata_1** **atacaaactttgattcctgattcaatcaaaagaattcctttactttaatactaatac---**

**Citrus_reticulata_2** **atacaaactttgattcctgattcaatcaaaagaattcctttactttaatactaatac---**

**Citrus_trifoliata_3** **atacaaactttgattcctgattcaatcaaaagaattcctttactttaatactaatac---**

**Citrus_aurantiifolia** **atacaaactttgattcctgattcaatcaaaagaattcctttactttaatactaatac---**

**Citrus_aurantium** **atacaaactttgattcctgattcaatcaaaagaattcctttactttaatactaatac---**

**Citrus_cavaleriei** **atacaaactttgattcctgattcaatcaaaagaattcctttactttaatac---------**

**Citrus_hongheensis** **atacaaactttgattcctgattcaatcaaaagaattcctttactttaatactaatac---**

**Citrus_limon** **atacaaactttgattcctgattcaatcaaaagaattcctttactttaatactaatac---**

**Citrus_maxima** **atacaaactttgattcctgattcaatcaaaagaattcctttactttaatactaatac---**

**Citrus_medica** **atacaaactttgattcctgattcaatcaaaagaattcctttactttaatactaatac---**

**Citrus_polytrifolia** **atacaaactttgattcctgattcaatcaaaagaattcctttactttaatactaatac---**

**Citrus_reticulata** **atacaaactttgattcctgattcaatcaaaagaattcctttactttaatactaatac---**

**Citrus_sinensis** **atacaaactttgattcctgattcaatcaaaagaattcctttactttaatactaatac---**

**Fortunella_bawangica_1** **atacaaactttgattcctgattcaatcaaaagaattcctttactttaatactaatac---**

**Fortunella_bawangica_2** **atacaaactttgattcctgattcaatcaaaagaattcctttactttaatactaatac---**

**Fortunella_hindsii_1** **atacaaactttgattcctgattcaatcaaaagaattcctttactttaatactaatac---**

**Fortunella_hindsii_2** **atacaaactttgattcctgattcaatcaaaagaattcctttactttaatactaatac---**

**Fortunella_hindsii_3** **atacaaactttgattcctgattcaatcaaaagaattcctttactttaatactaatac---**

**Fortunella_japonica_1** **atacaaactttgattcctgattcaatcaaaagaattcctttactttaatactaatac---**

**Fortunella_japonica_2** **atacaaactttgattcctgattcaatcaaaagaattcctttactttaatactaatac---**

**Fortunella_japonica_3** **atacaaactttgattcctgattcaatcaaaagaattcctttactttaatactaatac---**

**Fortunella_japonica_4** **atacaaactttgattcctgattcaatcaaaagaattcctttactttaatactaatac---**

**Fortunella_japonica_5** **atacaaactttgattcctgattcaatcaaaagaattcctttactttaatactaatac---**

**Fortunella_japonica_6** **atacaaactttgattcctgattcaatcaaaagaattcctttactttaatactaatac---**

**Fortunella_japonica_7** **atacaaactttgattcctgattcaatcaaaagaattcctttactttaatactaatac---**

**Fortunella_japonica_8** **atacaaactttgattcctgattcaatcaaaagaattcctttactttaatactaatac---**

**Fortunella_japonica_9** **atacaaactttgattcctgattcaatcaaaagaattcctttactttaatactaatac---**

**Fortunella_japonica_10** **atacaaactttgattcctgattcaatcaaaagaattcctttactttaatactaatac---**

**Fortunella_japonica_11** **atacaaactttgattcctgattcaatcaaaagaattcctttactttaatactaatac---**

**Fortunella_japonica_12** **atacaaactttgattcctgattcaatcaaaagaattcctttactttaatactaatac---**

**Fortunella_margarita_1** **atacaaactttgattcctgattcaatcaaaagaattcctttactttaatactaatac---**

**Fortunella_margarita_2** **atacaaactttgattcctgattcaatcaaaagaattcctttactttaatactaatac---**

**Fortunella_venosa_1** **atacaaactttgattcctgattcaatcaaaagaattcctttactttaatactaatac---**

**Fortunella_venosa_2** **atacaaactttgattcctgattcaatcaaaagaattcctttactttaatactaatac---**

**Fortunella_venosa_3** **atacaaactttgattcctgattcaatcaaaagaattcctttactttaatactaatac---**

33370	33380	33390	33400	33410	33420

....|....|....|....|....|....|....|....|....|....|....|....|

**Atalantia_kwangtungens** **actaatactttttttatttaccgtcttttccagtctttactttaatactaatacaatact**

**Citrus_reticulata_1** **---------tttttt---------------------------------------aatact**

**Citrus_reticulata_2** **---------tttttt---------------------------------------aatact**

**Citrus_trifoliata_3** **---------tttttt---------------------------------------aatact**

**Citrus_aurantiifolia** **---------tttttg---------------------------------------aatact**

**Citrus_aurantium** **---------tttttt---------------------------------------aatact**

**Citrus_cavaleriei** **--------------t---------------------------------------aatact**

**Citrus_hongheensis** **---------tttttt---------------------------------------aatact**

**Citrus_limon** **---------tttttt---------------------------------------aatact**

**Citrus_maxima** **---------tttttt---------------------------------------aatact**

**Citrus_medica** **---------tttttg---------------------------------------aatact**

**Citrus_polytrifolia** **---------tttttt---------------------------------------aatact**

**Citrus_reticulata** **---------tttttt---------------------------------------aatact**

**Citrus_sinensis** **---------tttttt---------------------------------------aatact**

**Fortunella_bawangica_1** **---------tttttt---------------------------------------aatact**

**Fortunella_bawangica_2** **---------tttttt---------------------------------------aatact**

**Fortunella_hindsii_1** **---------tttttt---------------------------------------aatact**

**Fortunella_hindsii_2** **---------tttttt---------------------------------------aatact**

**Fortunella_hindsii_3** **---------tttttt---------------------------------------aatact**

**Fortunella_japonica_1** **---------tttttt---------------------------------------aatact**

**Fortunella_japonica_2** **---------tttttt---------------------------------------aatact**

**Fortunella_japonica_3** **---------tttttt---------------------------------------aatact**

**Fortunella_japonica_4** **---------tttttt---------------------------------------aatact**

**Fortunella_japonica_5** **---------tttttt---------------------------------------aatact**

**Fortunella_japonica_6** **---------tttttt---------------------------------------aatact**

**Fortunella_japonica_7** **---------tttttt---------------------------------------aatact**

**Fortunella_japonica_8** **---------tttttt---------------------------------------aatact**

**Fortunella_japonica_9** **---------tttttt---------------------------------------aatact**

**Fortunella_japonica_10** **---------tttttt---------------------------------------aatact**

**Fortunella_japonica_11** **---------tttttt---------------------------------------aatact**

**Fortunella_japonica_12** **---------tttttt---------------------------------------aatact**

**Fortunella_margarita_1** **---------tttttt---------------------------------------aatact**

**Fortunella_margarita_2** **---------tttttt---------------------------------------aatact**

**Fortunella_venosa_1** **---------tttttt---------------------------------------aatact**

**Fortunella_venosa_2** **---------tttttt---------------------------------------aatact**

**Fortunella_venosa_3** **---------tttttt---------------------------------------aatact**

33430	33440	33450	33460	33470	33480

....|....|....|....|....|....|....|....|....|....|....|....|

**Atalantia_kwangtungens** **aatactttttttatttacc-gtcttttccagtctgtcttttctataatcgaccgccttac**

**Citrus_reticulata_1** **aatac-ttttttatttaccagtcttttccagtctgtcttttctataatcgaccgccttac**

**Citrus_reticulata_2** **aatac-ttttttatttaccagtcttttccagtctgtcttttctataatcgaccgccttac**

**Citrus_trifoliata_3** **aatactttttttatttaccagtcttttccagtctgtcttttctataatcgaccgccttac**

**Citrus_aurantiifolia** **aatactttttttatttaccagtcttttccagtctgtcttttctataatcgaccgccttac**

**Citrus_aurantium** **aatactttttttatttaccagtcttttccagtctgtcttttctataatcgaccgccttac**

**Citrus_cavaleriei** **aatac-ttttttatttaccagtcttttccagtctgtcttttctataatcgaccgccttac**

**Citrus_hongheensis** **aatactttttttatttaccagtcttttccagtctgtcttttctataatcgaccgccttac**

**Citrus_limon** **aatactttttttatttaccagtcttttccagtctgtcttttctataatcgaccgccttac**

**Citrus_maxima** **aatactttttttatttaccagtcttttccagtctgtcttttctataatcgaccgccttac**

**Citrus_medica** **aatactttttttatttaccagtcttttccagtctgtcttttctataatcgaccgccgtac**

**Citrus_polytrifolia** **aatactttttttatttaccagtcttttccagtctgtcttttctataatcgaccgccttac**

**Citrus_reticulata** **aatac-ttttttatttaccagtcttttccagtctgtcttttctataatcgaccgccttac**

**Citrus_sinensis** **aatactttttttatttaccagtcttttccagtctgtcttttctataatcgaccgccttac**

**Fortunella_bawangica_1** **aatactttttttatttaccagtctttttcagtctgtcttttctataatcgaccgccttac**

**Fortunella_bawangica_2** **aatactttttttatttaccagtctttttcagtctgtcttttctataatcgaccgccttac**

**Fortunella_hindsii_1** **aatactttttttatttaccagtctttttcagtctgtcttttctataatcgaccgccttac**

**Fortunella_hindsii_2** **aatactttttttatttaccagtctttttcagtctgtcttttctataatcgaccgccttac**

**Fortunella_hindsii_3** **aatactttttttatttaccagtctttttcagtctgtcttttctataatcgaccgccttac**

**Fortunella_japonica_1** **aatactttttttatttaccagtctttttcagtctgtcttttctataatcgaccgccttac**

**Fortunella_japonica_2** **aatactttttttatttaccagtctttttcagtctgtcttttctataatcgaccgccttac**

**Fortunella_japonica_3** **aatactttttttatttaccagtctttttcagtctgtcttttctataatcgaccgccttac**

**Fortunella_japonica_4** **aatactttttttatttaccagtctttttcagtctgtcttttctataatcgaccgccttac**

**Fortunella_japonica_5** **aatactttttttatttaccagtctttttcagtctgtcttttctataatcgaccgccttac**

**Fortunella_japonica_6** **aatactttttttatttaccagtctttttcagtctgtcttttctataatcgaccgccttac**

**Fortunella_japonica_7** **aatactttttttatttaccagtctttttcagtctgtcttttctataatcgaccgccttac**

**Fortunella_japonica_8** **aatactttttttatttaccagtctttttcagtctgtcttttctataatcgaccgccttac**

**Fortunella_japonica_9** **aatactttttttatttaccagtctttttcagtctgtcttttctataatcgaccgccttac**

**Fortunella_japonica_10** **aatactttttttatttaccagtctttttcagtctgtcttttctataatcgaccgccttac**

**Fortunella_japonica_11** **aatactttttttatttaccagtctttttcagtctgtcttttctataatcgaccgccttac**

**Fortunella_japonica_12** **aatactttttttatttaccagtctttttcagtctgtcttttctataatcgaccgccttac**

**Fortunella_margarita_1** **aatactttttttatttaccagtctttttcagtctgtcttttctataatcgaccgccttac**

**Fortunella_margarita_2** **aatactttttttatttaccagtctttttcagtctgtcttttctataatcgaccgccttac**

**Fortunella_venosa_1** **aatactttttttatttaccagtctttttcagtctgtcttttctataatcgaccgccttac**

**Fortunella_venosa_2** **aatactttttttatttaccagtctttttcagtctgtcttttctataatcgaccgccttac**

**Fortunella_venosa_3** **aatactttttttatttaccagtctttttcagtctgtcttttctataatcgaccgccttac**

33490	33500	33510	33520	33530	33540

....|....|....|....|....|....|....|....|....|....|....|....|

**Atalantia_kwangtungens** **tggtacaaccacctaaccgctttacactttccttgtttacaaagggtttagaaaaaaacc**

**Citrus_reticulata_1** **ttgtacaaccccctaaccgctttacactttccttgtttacaaagggtttagaaaaaaacc**

**Citrus_reticulata_2** **ttgtacaaccccctaaccgctttacactttccttgtttacaaagggtttagaaaaaaacc**

**Citrus_trifoliata_3** **ttgtacaaccccctaaccgctttacactttccttgtttacaaagggtttagaaaaaaacc**

**Citrus_aurantiifolia** **ttgtacaaccccctaaccgctttacactttccttgtttacaaagggtttagaaaaaaacc**

**Citrus_aurantium** **ttgtacaaccccctaaccgctttacactttccttgtttacaaagggtttagaaaaaaacc**

**Citrus_cavaleriei** **ttgtacaaccccctaaccgctttacactttccttgtttacaaagggtttagaaaaaaacc**

**Citrus_hongheensis** **ttgtacaaccccctaaccgctttacactttccttgtttacaaagggtttagaaaaaaacc**

**Citrus_limon** **ttgtacaaccccctaaccgctttacactttccttgtttacaaagggtttagaaaaaaacc**

**Citrus_maxima** **ttgtacaaccccctaaccgctttacactttccttgtttacaaagggtttagaaaaaaacc**

**Citrus_medica** **ttgtacaaccccctaaccgctttacactttccttgtttacaaagggtttagaaaaaaacc**

**Citrus_polytrifolia** **ttgtacaaccccctaaccgctttacactttccttgtttacaaagggtttagaaaaaaa--**

**Citrus_reticulata** **ttgtacaaccccctaaccgctttacactttccttgtttacaaagggtttagaaaaaaacc**

**Citrus_sinensis** **ttgtacaaccccctaaccgctttacactttccttgtttacaaagggtttagaaaaaaacc**

**Fortunella_bawangica_1** **ttgtacaaccccctaaccgctttacactttccttgtttacaaagggtttagaaaaaaacc**

**Fortunella_bawangica_2** **ttgtacaaccccctaaccgctttacactttccttgtttacaaagggtttagaaaaaaacc**

**Fortunella_hindsii_1** **ttgtacaaccccctaaccgctttacactttccttgtttacaaagggtttagaaaaaaacc**

**Fortunella_hindsii_2** **ttgtacaaccccctaaccgctttacactttccttgtttacaaagggtttagaaaaaaacc**

**Fortunella_hindsii_3** **ttgtacaaccccctaaccgctttacactttccttgtttacaaagggtttagaaaaaaacc**

**Fortunella_japonica_1** **ttgtacaaccccctaaccgctttacactttccttgtttacaaagggtttagaaaaaaacc**

**Fortunella_japonica_2** **ttgtacaaccccctaaccgctttacactttccttgtttacaaagggtttagaaaaaaacc**

**Fortunella_japonica_3** **ttgtacaaccccctaaccgctttacactttccttgtttacaaagggtttagaaaaaaacc**

**Fortunella_japonica_4** **ttgtacaaccccctaaccgctttacactttccttgtttacaaagggtttagaaaaaaacc**

**Fortunella_japonica_5** **ttgtacaaccccctaaccgctttacactttccttgtttacaaagggtttagaaaaaaacc**

**Fortunella_japonica_6** **ttgtacaaccccctaaccgctttacactttccttgtttacaaagggtttagaaaaaaacc**

**Fortunella_japonica_7** **ttgtacaaccccctaaccgctttacactttccttgtttacaaagggtttagaaaaaaacc**

**Fortunella_japonica_8** **ttgtacaaccccctaaccgctttacactttccttgtttacaaagggtttagaaaaaaacc**

**Fortunella_japonica_9** **ttgtacaaccccctaaccgctttacactttccttgtttacaaagggtttagaaaaaaacc**

**Fortunella_japonica_10** **ttgtacaaccccctaaccgctttacactttccttgtttacaaagggtttagaaaaaaacc**

**Fortunella_japonica_11** **ttgtacaaccccctaaccgctttacactttccttgtttacaaagggtttagaaaaaaacc**

**Fortunella_japonica_12** **ttgtacaaccccctaaccgctttacactttccttgtttacaaagggtttagaaaaaaacc**

**Fortunella_margarita_1** **ttgtacaaccccctaaccgctttacactttccttgtttacaaagggtttagaaaaaaacc**

**Fortunella_margarita_2** **ttgtacaaccccctaaccgctttacactttccttgtttacaaagggtttagaaaaaaacc**

**Fortunella_venosa_1** **ttgtacaaccccctaaccgctttacactttccttgtttacaaagggtttagaaaaaaacc**

**Fortunella_venosa_2** **ttgtacaaccccctaaccgctttacactttccttgtttacaaagggtttagaaaaaaacc**

**Fortunella_venosa_3** **ttgtacaaccccctaaccgctttacactttccttgtttacaaagggtttagaaaaaaacc**

33550	33560	33570	33580	33590	33600

....|....|....|....|....|....|....|....|....|....|....|....|

**Atalantia_kwangtungens** **aaacaaacaatcgaaacgaaatagaaaaagaaaaaggg--------aagttctaaaccct**

**Citrus_reticulata_1** **aaacaaacaatcgaaacgaaatagaaaaagaaaaagggaaggggttaagttcgaaacccc**

**Citrus_reticulata_2** **aaacaaacaatcgaaacgaaatagaaaaagaaaaagggaaggggttaagttcgaaacccc**

**Citrus_trifoliata_3** **aaacaaacaatcgaaacgaaatagaaaaagaaaaagggaaggggttaagttctaaacccc**

**Citrus_aurantiifolia** **aaacaaacaatcgaaacgaaatagaaaaagaaaaagggaaggggttaagttctaaacccc**

**Citrus_aurantium** **aaacaaacaatcgaaacgaaatagaaaaagaaaaagggaaggggttaagttctaaacccc**

**Citrus_cavaleriei** **aaacaaacaatcgaaacgaaatagaaaaagaaaaagggaaggggttaagttctaaacccc**

**Citrus_hongheensis** **aaacaaacaatcgaaacgaaatagaaaaagaaaaagggaaggggttaagttctaaacccc**

**Citrus_limon** **aaacaaacaatcgaaacgaaatagaaaaagaaaaagggaaggggttaagttctaaacccc**

**Citrus_maxima** **aaacaaacaatcgaaacgaaatagaaaaagaaaaagggaaggggttaagttctaaacccc**

**Citrus_medica** **aaacaaacaatcgaaacgaaatagaaaaagaaaaagggaaggggttaagttctaaacccc**

**Citrus_polytrifolia** **aaacaaacaatcgaaacgaaatagaaaaagaaaaagggaaggggttaagttctaaacccc**

**Citrus_reticulata** **aaacaaacaatcgaaacgaaatagaaaaagaaaaagggaaggggttaagttcgaaacccc**

**Citrus_sinensis** **aaacaaacaatcgaaacgaaatagaaaaagaaaaagggaaggggttaagttctaaacccc**

**Fortunella_bawangica_1** **aaacaaacaatcgaaacgaaatagaaaaagaaaaagggaaggggttaagttctaaacccc**

**Fortunella_bawangica_2** **aaacaaacaatcgaaacgaaatagaaaaagaaaaagggaaggggttaagttctaaacccc**

**Fortunella_hindsii_1** **aaacaaacaatcgaaacgaaatagaaaaagaaaaagggaaggggttaagttctaaacccc**

**Fortunella_hindsii_2** **aaacaaacaatcgaaacgaaatagaaaaagaaaaagggaaggggttaagttctaaacccc**

**Fortunella_hindsii_3** **aaacaaacaatcgaaacgaaatagaaaaagaaaaagggaaggggttaagttctaaacccc**

**Fortunella_japonica_1** **aaacaaacaatcgaaacgaaatagaaaaagaaaaagggaaggggttaagttctaaacccc**

**Fortunella_japonica_2** **aaacaaacaatcgaaacgaaatagaaaaagaaaaagggaaggggttaagttctaaacccc**

**Fortunella_japonica_3** **aaacaaacaatcgaaacgaaatagaaaaagaaaaagggaaggggttaagttctaaacccc**

**Fortunella_japonica_4** **aaacaaacaatcgaaacgaaatagaaaaagaaaaagggaaggggttaagttctaaacccc**

**Fortunella_japonica_5** **aaacaaacaatcgaaacgaaatagaaaaagaaaaagggaaggggttaagttctaaacccc**

**Fortunella_japonica_6** **aaacaaacaatcgaaacgaaatagaaaaagaaaaagggaaggggttaagttctaaacccc**

**Fortunella_japonica_7** **aaacaaacaatcgaaacgaaatagaaaaagaaaaagggaaggggttaagttctaaacccc**

**Fortunella_japonica_8** **aaacaaacaatcgaaacgaaatagaaaaagaaaaagggaaggggttaagttctaaacccc**

**Fortunella_japonica_9** **aaacaaacaatcgaaacgaaatagaaaaagaaaaagggaaggggttaagttctaaacccc**

**Fortunella_japonica_10** **aaacaaacaatcgaaacgaaatagaaaaagaaaaagggaaggggttaagttctaaacccc**

**Fortunella_japonica_11** **aaacaaacaatcgaaacgaaatagaaaaagaaaaagggaaggggttaagttctaaacccc**

**Fortunella_japonica_12** **aaacaaacaatcgaaacgaaatagaaaaagaaaaagggaaggggttaagttctaaacccc**

**Fortunella_margarita_1** **aaacaaacaatcgaaacgaaatagaaaaagaaaaagggaaggggttaagttctaaacccc**

**Fortunella_margarita_2** **aaacaaacaatcgaaacgaaatagaaaaagaaaaagggaaggggttaagttctaaacccc**

**Fortunella_venosa_1** **aaacaaacaatcgaaacgaaatagaaaaagaaaaagggaaggggttaagttctaaacccc**

**Fortunella_venosa_2** **aaacaaacaatcgaaacgaaatagaaaaagaaaaagggaaggggttaagttctaaacccc**

**Fortunella_venosa_3** **aaacaaacaatcgaaacgaaatagaaaaagaaaaagggaaggggttaagttctaaacccc**

33610	33620	33630	33640	33650	33660

....|....|....|....|....|....|....|....|....|....|....|....|

**Atalantia_kwangtungens** **tttcat----------------tttttttttcaagaaaattatatcatt-aaaaaaaaaa**

**Citrus_reticulata_1** **ttttcatttt--tttttttt--tttttttttcaagaaaattatatcatt---aaaaaaaa**

**Citrus_reticulata_2** **ttttcatttt--tttttttt--tttttttttcaagaaaattatatcatt---aaaaaaaa**

**Citrus_trifoliata_3** **ttttcatttttttttttttttttttttttttcaagaaaattatatcatt---aaaaaaaa**

**Citrus_aurantiifolia** **ttttca-----------ttt--tctttttttcaagaaaattatatcatt-aaaaaaaaaa**

**Citrus_aurantium** **ttttca-----------ttt--tctttttttcaagaaaattatatcattaaaaaaaaaaa**

**Citrus_cavaleriei** **ttttca----------tttt--tttttttttcaagaaaattatatcatt---aaaaaaaa**

**Citrus_hongheensis** **ttttca-----------ttt--tctttttttcaagaaaattatatcatt-aaaaaaaaaa**

**Citrus_limon** **ttttca-----------ttt--tctttttttcaagaaaattatatcattaaaaaaaaaaa**

**Citrus_maxima** **ttttca-----------ttt--tctttttttcaagaaaattatatcattaaaaaaaaaaa**

**Citrus_medica** **ttttcatttt----tctttttttttttttttcaagaaaattatatcatt----aaaaaaa**

**Citrus_polytrifolia** **ttttca-ttttctttttttttttttttttttcaagaaaattatatcatt----aaaaaaa**

**Citrus_reticulata** **ttttcatttt--tttttttt--tttttttttcaagaaaattatatcatt---aaaaaaaa**

**Citrus_sinensis** **ttttca-----------ttt--tctttttttcaagaaaattatatcattaaaaaaaaaaa**

**Fortunella_bawangica_1** **ttttca--------tttttctttttttttttcaagaaaataatatcatt-aaaaaaaaaa**

**Fortunella_bawangica_2** **ttttca--------tttttctttttttttttcaagaaaataatatcatt-aaaaaaaaaa**

**Fortunella_hindsii_1** **ttttca--------tttttc--tttttttttcaagaaaataatatcatt-aaaaaaaaaa**

**Fortunella_hindsii_2** **ttttca--------tttttc--tttttttttcaagaaaataatatcatt--aaaaaaaaa**

**Fortunella_hindsii_3** **ttttca--------tttttc-ttttttttttcaagaaaataatatcatt-aaaaaaaaaa**

**Fortunella_japonica_1** **ttttca--------tttttc--tttttttttcaagaaaataatatcatt-aaaaaaaaaa**

**Fortunella_japonica_2** **ttttca--------tttttc-ttttttttttcaagaaaataatatcatt-aaaaaaaaaa**

**Fortunella_japonica_3** **ttttca--------tttttc--tttttttttcaagaaaataatatcatt-aaaaaaaaaa**

**Fortunella_japonica_4** **ttttca--------tttttc--tttttttttcaagaaaataatatcatt-aaaaaaaaaa**

**Fortunella_japonica_5** **ttttca--------tttttc--tttttttttcaagaaaataatatcatt-aaaaaaaaaa**

**Fortunella_japonica_6** **ttttca--------tttttc--tttttttttcaagaaaataatatcatt-aaaaaaaaaa**

**Fortunella_japonica_7** **ttttca--------tttttc--tttttttttcaagaaaataatatcatt-aaaaaaaaaa**

**Fortunella_japonica_8** **ttttca--------tttttc--tttttttttcaagaaaataatatcatt-aaaaaaaaaa**

**Fortunella_japonica_9** **ttttca--------tttttc--tttttttttcaagaaaataatatcatt-aaaaaaaaaa**

**Fortunella_japonica_10** **ttttca--------tttttc-ttttttttttcaagaaaataatatcatt-aaaaaaaaaa**

**Fortunella_japonica_11** **ttttca--------tttttc--tttttttttcaagaaaataatatcatt-aaaaaaaaaa**

**Fortunella_japonica_12** **ttttca--------tttttc--tttttttttcaagaaaataatatcatt-aaaaaaaaaa**

**Fortunella_margarita_1** **ttttca--------tttttc-ttttttttttcaagaaaataatatcatt-aaaaaaaaaa**

**Fortunella_margarita_2** **ttttca--------tttttc-ttttttttttcaagaaaataatatcatt-aaaaaaaaaa**

**Fortunella_venosa_1** **ttttca--------tttttc--tttttttttcaagaaaataatatcatt--aaaaaaaaa**

**Fortunella_venosa_2** **ttttca--------tttttc--tttttttttcaagaaaataatatcatt-aaaaaaaaaa**

**Fortunella_venosa_3** **ttttca--------tttttc--tttttttttcaagaaaataatatcatt-aaaaaaaaaa**

33670	33680	33690	33700	33710	33720

....|....|....|....|....|....|....|....|....|....|....|....|

**Atalantia_kwangtungens** **aac---------------------------------------------------------**

**Citrus_reticulata_1** **aacgaaaaagaagtgtgataaagacgagtcccagtagaaaagaatcgaatatttcataaa**

**Citrus_reticulata_2** **aacgaaaaagaagtgtgataaagacgagtcccagtagaaaagaatcgaatatttcataaa**

**Citrus_trifoliata_3** **aacgaaaaagaagtgtgataaagacgagtcccagtagaaaagaatcgaatatttcataaa**

**Citrus_aurantiifolia** **aacgaaaaagaagtgtgataaagacgagtcccagtagaaaagaatcgaatatttcataaa**

**Citrus_aurantium** **aacgaaaaagaagtgtgataaagacgagtcccagtataaaagaatcgaatatttcataaa**

**Citrus_cavaleriei** **aacgaaaaagaagtgtgataaagacgagtcccagtagaaaagaatcgaatatttcataaa**

**Citrus_hongheensis** **aacgaaaaagaagtgtgataaagacgagtcccagtataaaagaatcgaatatttcataaa**

**Citrus_limon** **aacgaaaaagaagtgtgataaagacgagtcccagtataaaagaatcgaatatttcataaa**

**Citrus_maxima** **aacgaaaaagaagtgtgataaagacgagtcccagtataaaagaatcgaatatttcataaa**

**Citrus_medica** **aacgaaaaagaagtgtgataaagacgagtcccagtagaaaagaatcgaatatttcataaa**

**Citrus_polytrifolia** **aacgaaaaagaagtgtgataaagacgagtcccagtataaaagaatcgaatatttcataaa**

**Citrus_reticulata** **aacgaaaaagaagtgtgataaagacgagtcccagtagaaaagaatcgaatatttcataaa**

**Citrus_sinensis** **aacgaaaaagaagtgtgataaagacgagtcccagtataaaagaatcgaatatttcataaa**

**Fortunella_bawangica_1** **aacgaaaaagaagtgtgataaagacgagtcccagtagaaaagaatcgaatatttcataaa**

**Fortunella_bawangica_2** **aacgaaaaagaagtgtgataaagacgagtcccagtagaaaagaatcgaatatttcataaa**

**Fortunella_hindsii_1** **aacgaaaaagaagtgtgataaagacgagtcccagtagaaaagaatcgaatatttcataaa**

**Fortunella_hindsii_2** **aacgaaaaagaagtgtgataaagacgagtcccagtagaaaagaatcgaatatttcataaa**

**Fortunella_hindsii_3** **aacgaaaaagaagtgtgataaagacgagtcccagtagaaaagaatcgaatatttcataaa**

**Fortunella_japonica_1** **aacgaaaaagaagtgtgataaagacgagtcccagtagaaaagaatcgaatatttcataaa**

**Fortunella_japonica_2** **aacgaaaaagaagtgtgataaagacgagtcccagtagaaaagaatcgaatatttcataaa**

**Fortunella_japonica_3** **aacgaaaaagaagtgtgataaagacgagtcccagtagaaaagaatcgaatatttcataaa**

**Fortunella_japonica_4** **aacgaaaaagaagtgtgataaagacgagtcccagtagaaaagaatcgaatatttcataaa**

**Fortunella_japonica_5** **aacgaaaaagaagtgtgataaagacgagtcccagtagaaaagaatcgaatatttcataaa**

**Fortunella_japonica_6** **aacgaaaaagaagtgtgataaagacgagtcccagtagaaaagaatcgaatatttcataaa**

**Fortunella_japonica_7** **aacgaaaaagaagtgtgataaagacgagtcccagtagaaaagaatcgaatatttcataaa**

**Fortunella_japonica_8** **aacgaaaaagaagtgtgataaagacgagtcccagtagaaaagaatcgaatatttcataaa**

**Fortunella_japonica_9** **aacgaaaaagaagtgtgataaagacgagtcccagtagaaaagaatcgaatatttcataaa**

**Fortunella_japonica_10** **aacgaaaaagaagtgtgataaagacgagtcccagtagaaaagaatcgaatatttcataaa**

**Fortunella_japonica_11** **aacgaaaaagaagtgtgataaagacgagtcccagtagaaaagaatcgaatatttcataaa**

**Fortunella_japonica_12** **aacgaaaaagaagtgtgataaagacgagtcccagtagaaaagaatcgaatatttcataaa**

**Fortunella_margarita_1** **aacgaaaaagaagtgtgataaagacgagtcccagtagaaaagaatcgaatatttcataaa**

**Fortunella_margarita_2** **aacgaaaaagaagtgtgataaagacgagtcccagtagaaaagaatcgaatatttcataaa**

**Fortunella_venosa_1** **aacgaaaaagaagtgtgataaagacgagtcccagtagaaaagaatcgaatatttcataaa**

**Fortunella_venosa_2** **aacgaaaaagaagtgtgataaagacgagtcccagtagaaaagaatcgaatatttcataaa**

**Fortunella_venosa_3** **aacgaaaaagaagtgtgataaagacgagtcccagtagaaaagaatcgaatatttcataaa**

33730	33740	33750	33760	33770	33780

....|....|....|....|....|....|....|....|....|....|....|....|

**Atalantia_kwangtungens** **------------------------------------------------------------**

**Citrus_reticulata_1** **attgactattttatttagatttatttcgagtttattcttctttggcaatacgcttaaaaa**

**Citrus_reticulata_2** **attgactattttatttagatttatttcgagtttattcttctttggcaatacgcttaaaaa**

**Citrus_trifoliata_3** **attgactattttatttagatttatttagagtttattcttctttggcaatacgcttaaaaa**

**Citrus_aurantiifolia** **attgactattttatttagatttatttagagtttcttcttctttggcaatacgcttaaaaa**

**Citrus_aurantium** **attgactattttatttagatttatttagagtttcttcttctttggcaatacgcttaaaaa**

**Citrus_cavaleriei** **attgacgattttatttagatttatttagagtttattcttctttggcaatacgcttaaaaa**

**Citrus_hongheensis** **attgactattttatttagatttatttagagtttcttcttctttggcaatacgcttaaaaa**

**Citrus_limon** **attgactattttatttagatttatttagagtttcttcttctttggcaatacgcttaaaaa**

**Citrus_maxima** **attgactattttatttagatttatttagagtttcttcttctttggcaatacgcttaaaaa**

**Citrus_medica** **attgactattttatttagatttatttagagtttattcttctttggcaatacgcttaaaaa**

**Citrus_polytrifolia** **attgactattttatttagatttatttagagtttattcttctttggcaatacgcttaaaaa**

**Citrus_reticulata** **attgactattttatttagatttatttcgagtttattcttctttggcaatacgcttaaaaa**

**Citrus_sinensis** **attgactattttatttagatttatttagagtttcttcttctttggcaatacgcttaaaaa**

**Fortunella_bawangica_1** **attgactattttatttagatttatttagagtttattcttctttggcaatacgcttaaaaa**

**Fortunella_bawangica_2** **attgactattttatttagatttatttagagtttattcttctttggcaatacgcttaaaaa**

**Fortunella_hindsii_1** **attgactattttatttagatttatttagagtttattcttctttggcaatacgcttaaaaa**

**Fortunella_hindsii_2** **attgactattttatttagatttatttagagtttattcttctttggcaatacgcttaaaaa**

**Fortunella_hindsii_3** **attgactattttatttagatttatttagagtttattcttctttggcaatacgcttaaaaa**

**Fortunella_japonica_1** **attgactattttatttagatttatttagagtttattcttctttggcaatacgcttaaaaa**

**Fortunella_japonica_2** **attgactattttatttagatttatttagagtttattcttctttggcaatacgcttaaaaa**

**Fortunella_japonica_3** **attgactattttatttagatttatttagagtttattcttctttggcaatacgcttaaaaa**

**Fortunella_japonica_4** **attgactattttatttagatttatttagagtttattcttctttggcaatacgcttaaaaa**

**Fortunella_japonica_5** **attgactattttatttagatttatttagagtttattcttctttggcaatacgcttaaaaa**

**Fortunella_japonica_6** **attgactattttatttagatttatttagagtttattcttctttggcaatacgcttaaaaa**

**Fortunella_japonica_7** **attgactattttatttagatttatttagagtttattcttctttggcaatacgcttaaaaa**

**Fortunella_japonica_8** **attgactattttatttagatttatttagagtttattcttctttggcaatacgcttaaaaa**

**Fortunella_japonica_9** **attgactattttatttagatttatttagagtttattcttctttggcaatacgcttaaaaa**

**Fortunella_japonica_10** **attgactattttatttagatttatttagagtttattcttctttggcaatacgcttaaaaa**

**Fortunella_japonica_11** **attgactattttatttagatttatttagagtttattcttctttggcaatacgcttaaaaa**

**Fortunella_japonica_12** **attgactattttatttagatttatttagagtttattcttctttggcaatacgcttaaaaa**

**Fortunella_margarita_1** **attgactattttatttagatttatttagagtttattcttctttggcaatacgcttaaaaa**

**Fortunella_margarita_2** **attgactattttatttagatttatttagagtttattcttctttggcaatacgcttaaaaa**

**Fortunella_venosa_1** **attgactattttatttagatttatttagagtttattcttctttggcaatacgcttaaaaa**

**Fortunella_venosa_2** **attgactattttatttagatttatttagagtttattcttctttggcaatacgcttaaaaa**

**Fortunella_venosa_3** **attgactattttatttagatttatttagagtttattcttctttggcaatacgcttaaaaa**

33790	33800	33810	33820	33830	33840

....|....|....|....|....|....|....|....|....|....|....|....|

**Atalantia_kwangtungens** **------------------------------------------------------------**

**Citrus_reticulata_1** **agattattcattccctcttcgggaggggttggcgccttgcttgatctttattttattaag**

**Citrus_reticulata_2** **agattattcattccctcttcgggaggggttggcgccttgcttgatctttattttattaag**

**Citrus_trifoliata_3** **agattattcattccctcttcgggaggggttggcgccttgcttgatctttattttattacg**

**Citrus_aurantiifolia** **agattattcattccctcttcgggaggggttggcgccttgcttgatctttattttattaag**

**Citrus_aurantium** **agattattcattccctcttcgggaggggttggcgccttgcttgatctttattttattaag**

**Citrus_cavaleriei** **agattattcattccctcttcgggaggggttggcgccttgcttgatctttattttattaag**

**Citrus_hongheensis** **agattattcattccctcttcgggaggggttggcgccttgcttgatctttattttattaag**

**Citrus_limon** **agattattcattccctcttcgggaggggttggcgccttgcttgatctttattttattaag**

**Citrus_maxima** **agattattcattccctcttcgggaggggttggcgccttgcttgatctttattttattaag**

**Citrus_medica** **agattattcattccctcttcgggaggggttgtcgccttgcttgatctttattttattaag**

**Citrus_polytrifolia** **agattattcattccctcttcgggaggggttggcgccttgcttgatctttattttattacg**

**Citrus_reticulata** **agattattcattccctcttcgggaggggttggcgccttgcttgatctttattttattaag**

**Citrus_sinensis** **agattattcattccctcttcgggaggggttggcgccttgcttgatctttattttattaag**

**Fortunella_bawangica_1** **agattattcattccctcttcgggaggggttggcgccttgcttgatctttattttattaag**

**Fortunella_bawangica_2** **agattattcattccctcttcgggaggggttggcgccttgcttgatctttattttattaag**

**Fortunella_hindsii_1** **agattattcattccctcttcgggaggggttggcgccttgcttgatctttattttattaag**

**Fortunella_hindsii_2** **agattattcattccctcttcgggaggggttggcgccttgcttgatctttattttattaag**

**Fortunella_hindsii_3** **agattattcattccctcttcgggaggggttggcgccttgcttgatctttattttattaag**

**Fortunella_japonica_1** **agattattcattccctcttcgggaggggttggcgccttgcttgatctttattttattaag**

**Fortunella_japonica_2** **agattattcattccctcttcgggaggggttggcgccttgcttgatctttattttattaag**

**Fortunella_japonica_3** **agattattcattccctcttcgggaggggttggcgccttgcttgatctttattttattaag**

**Fortunella_japonica_4** **agattattcattccctcttcgggaggggttggcgccttgcttgatctttattttattaag**

**Fortunella_japonica_5** **agattattcattccctcttcgggaggggttggcgccttgcttgatctttattttattaag**

**Fortunella_japonica_6** **agattattcattccctcttcgggaggggttggcgccttgcttgatctttattttattaag**

**Fortunella_japonica_7** **agattattcattccctcttcgggaggggttggcgccttgcttgatctttattttattaag**

**Fortunella_japonica_8** **agattattcattccctcttcgggaggggttggcgccttgcttgatctttattttattaag**

**Fortunella_japonica_9** **agattattcattccctcttcgggaggggttggcgccttgcttgatctttattttattaag**

**Fortunella_japonica_10** **agattattcattccctcttcgggaggggttggcgccttgcttgatctttattttattaag**

**Fortunella_japonica_11** **agattattcattccctcttcgggaggggttggcgccttgcttgatctttattttattaag**

**Fortunella_japonica_12** **agattattcattccctcttcgggaggggttggcgccttgcttgatctttattttattaag**

**Fortunella_margarita_1** **agattattcattccctcttcgggaggggttggcgccttgcttgatctttattttattaag**

**Fortunella_margarita_2** **agattattcattccctcttcgggaggggttggcgccttgcttgatctttattttattaag**

**Fortunella_venosa_1** **agattattcattccctcttcgggaggggttggcgccttgcttgatctttattttattaag**

**Fortunella_venosa_2** **agattattcattccctcttcgggaggggttggcgccttgcttgatctttattttattaag**

**Fortunella_venosa_3** **agattattcattccctcttcgggaggggttggcgccttgcttgatctttattttattaag**

33850	33860	33870	33880	33890	33900

....|....|....|....|....|....|....|....|....|....|....|....|

**Atalantia_kwangtungens** **------------------------------------------------------------**

**Citrus_reticulata_1** **aatatcatcccccctcccttggattagtactagaacgtatccctcaaaagttcggcgtga**

**Citrus_reticulata_2** **aatatcatcccccctcccttggattagtactagaacgtatccctcaaaagttcggcgtga**

**Citrus_trifoliata_3** **aatatcatcccccctcccttggattagtactagaacgtatccctcaaaagttcggcgtga**

**Citrus_aurantiifolia** **aatatcatcccccctcccttggattagtactagaacgtatccctcaaaagttcggcgtga**

**Citrus_aurantium** **aatatcatcccccctcccttggattagtactagaacgtatccctcaaaagttcggcgtga**

**Citrus_cavaleriei** **aatatcatcccccctcccttggattagtactagaacgtatccctcaaaagttcggcgtga**

**Citrus_hongheensis** **aatatcatcccccctcccttggattagtactagaacgtatccctcaaaagttcggcgtga**

**Citrus_limon** **aatatcatcccccctcccttggattagtactagaacgtatccctcaaaagttcggcgtga**

**Citrus_maxima** **aatatcatcccccctcccttggattagtactagaacgtatccctcaaaagttcggcgtga**

**Citrus_medica** **aatatcatcccccctcccttggattagtactagaacgtatccctcaaaagttcggcgtga**

**Citrus_polytrifolia** **aatatcatcccccctcccttggattagtactagaacgtatccctcaaaagttcggcgtga**

**Citrus_reticulata** **aatatcatcccccctcccttggattagtactagaacgtatccctcaaaagttcggcgtga**

**Citrus_sinensis** **aatatcatcccccctcccttggattagtactagaacgtatccctcaaaagttcggcgtga**

**Fortunella_bawangica_1** **aatatcatcccccctcccttggattagtactagaacgtattcctcaaaagttcggcgtga**

**Fortunella_bawangica_2** **aatatcatcccccctcccttggattagtactagaacgtattcctcaaaagttcggcgtga**

**Fortunella_hindsii_1** **aatatcatcccccctcccttggattagtactagaacgtattcctcaaaagttcggcgtga**

**Fortunella_hindsii_2** **aatatcatcccccctcccttggattagtactagaacgtattcctcaaaagttcggcgtga**

**Fortunella_hindsii_3** **aatatcatcccccctcccttggattagtactagaacgtattcctcaaaagttcggcgtga**

**Fortunella_japonica_1** **aatatcatcccccctcccttggattagtactagaacgtattcctcaaaagttcggcgtga**

**Fortunella_japonica_2** **aatatcatcccccctcccttggattagtactagaacgtattcctcaaaagttcggcgtga**

**Fortunella_japonica_3** **aatatcatcccccctcccttggattagtactagaacgtattcctcaaaagttcggcgtga**

**Fortunella_japonica_4** **aatatcatcccccctcccttggattagtactagaacgtattcctcaaaagttcggcgtga**

**Fortunella_japonica_5** **aatatcatcccccctcccttggattagtactagaacgtattcctcaaaagttcggcgtga**

**Fortunella_japonica_6** **aatatcatcccccctcccttggattagtactagaacgtattcctcaaaagttcggcgtga**

**Fortunella_japonica_7** **aatatcatcccccctcccttggattagtactagaacgtattcctcaaaagttcggcgtga**

**Fortunella_japonica_8** **aatatcatcccccctcccttggattagtactagaacgtattcctcaaaagttcggcgtga**

**Fortunella_japonica_9** **aatatcatcccccctcccttggattagtactagaacgtattcctcaaaagttcggcgtga**

**Fortunella_japonica_10** **aatatcatcccccctcccttggattagtactagaacgtattcctcaaaagttcggcgtga**

**Fortunella_japonica_11** **aatatcatcccccctcccttggattagtactagaacgtattcctcaaaagttcggcgtga**

**Fortunella_japonica_12** **aatatcatcccccctcccttggattagtactagaacgtattcctcaaaagttcggcgtga**

**Fortunella_margarita_1** **aatatcatcccccctcccttggattagtactagaacgtattcctcaaaagttcggcgtga**

**Fortunella_margarita_2** **aatatcatcccccctcccttggattagtactagaacgtattcctcaaaagttcggcgtga**

**Fortunella_venosa_1** **aatatcatcccccctcccttggattagtactagaacgtattcctcaaaagttcggcgtga**

**Fortunella_venosa_2** **aatatcatcccccctcccttggattagtactagaacgtattcctcaaaagttcggcgtga**

**Fortunella_venosa_3** **aatatcatcccccctcccttggattagtactagaacgtattcctcaaaagttcggcgtga**

33910	33920	33930	33940	33950	33960

....|....|....|....|....|....|....|....|....|....|....|....|

**Atalantia_kwangtungens** **------------------------------------------------------------**

**Citrus_reticulata_1** **aatttgaatgagataaattcttt----------caaattcaaactagtaatttaagttag**

**Citrus_reticulata_2** **aatttgaatgagataaattcttt----------caaattcaaactagtaatttaagttag**

**Citrus_trifoliata_3** **aatttgaatgagataaattcttt----------caaattcaaactagtaatttaagttag**

**Citrus_aurantiifolia** **aatttgaatgagataaattcttt----------caaattcaaactagtaatttaagttag**

**Citrus_aurantium** **aatttgaatgagataaattcttt----------caaattcaaactagtaatttaagttag**

**Citrus_cavaleriei** **aatttgaatgagataaattcttt----------caaattcaaactagtaatttaagttag**

**Citrus_hongheensis** **aatttgaatgagataaattcttt----------caaattcaaactagtaatttaagttag**

**Citrus_limon** **aatttgaatgagataaattcttt----------caaattcaaactagtaatttaagttag**

**Citrus_maxima** **aatttgaatgagataaattcttt----------caaattcaaactagtaatttaagttag**

**Citrus_medica** **aatttgaatgagataaattctttcaaattcaaacaaattcaaactagtaatttaagttag**

**Citrus_polytrifolia** **aatttgaatgagataaattcttt----------caaattcaaactagtaatttaagttag**

**Citrus_reticulata** **aatttgaatgagataaattcttt----------caaattcaaactagtaatttaagttag**

**Citrus_sinensis** **aatttgaatgagataaattcttt----------caaattcaaactagtaatttaagttag**

**Fortunella_bawangica_1** **aatttgaatgagataaattcttt----------caaattcaaactagtaatttaagttag**

**Fortunella_bawangica_2** **aatttgaatgagataaattcttt----------caaattcaaactagtaatttaagttag**

**Fortunella_hindsii_1** **aatttgaatgagataaattcttt----------caaattcaaactagtaatttaagttag**

**Fortunella_hindsii_2** **aatttgaatgagataaattcttt----------caaattcaaactagtaatttaagttag**

**Fortunella_hindsii_3** **aatttgaatgagataaattcttt----------caaattcaaactagtaatttaagttag**

**Fortunella_japonica_1** **aatttgaatgagataaattcttt----------caaattcaaactagtaatttaagttag**

**Fortunella_japonica_2** **aatttgaatgagataaattcttt----------caaattcaaactagtaatttaagttag**

**Fortunella_japonica_3** **aatttgaatgagataaattcttt----------caaattcaaactagtaatttaagttag**

**Fortunella_japonica_4** **aatttgaatgagataaattcttt----------caaattcaaactagtaatttaagttag**

**Fortunella_japonica_5** **aatttgaatgagataaattcttt----------caaattcaaactagtaatttaagttag**

**Fortunella_japonica_6** **aatttgaatgagataaattcttt----------caaattcaaactagtaatttaagttag**

**Fortunella_japonica_7** **aatttgaatgagataaattcttt----------caaattcaaactagtaatttaagttag**

**Fortunella_japonica_8** **aatttgaatgagataaattcttt----------caaattcaaactagtaatttaagttag**

**Fortunella_japonica_9** **aatttgaatgagataaattcttt----------caaattcaaactagtaatttaagttag**

**Fortunella_japonica_10** **aatttgaatgagataaattcttt----------caaattcaaactagtaatttaagttag**

**Fortunella_japonica_11** **aatttgaatgagataaattcttt----------caaattcaaactagtaatttaagttag**

**Fortunella_japonica_12** **aatttgaatgagataaattcttt----------caaattcaaactagtaatttaagttag**

**Fortunella_margarita_1** **aatttgaatgagataaattcttt----------caaattcaaactagtaatttaagttag**

**Fortunella_margarita_2** **aatttgaatgagataaattcttt----------caaattcaaactagtaatttaagttag**

**Fortunella_venosa_1** **aatttgaatgagataaattcttt----------caaattcaaactagtaatttaagttag**

**Fortunella_venosa_2** **aatttgaatgagataaattcttt----------caaattcaaactagtaatttaagttag**

**Fortunella_venosa_3** **aatttgaatgagataaattcttt----------caaattcaaactagtaatttaagttag**

33970	33980	33990	34000	34010	34020

....|....|....|....|....|....|....|....|....|....|....|....|

**Atalantia_kwangtungens** **------------------------------------------------------------**

**Citrus_reticulata_1** **ccaaactagaaaccagaaaagaagatactttgaatcttaatcttaaaagtattctatcaa**

**Citrus_reticulata_2** **ccaaactagaaaccagaaaagaagatactttgaatcttaatcttaaaagtattctatcaa**

**Citrus_trifoliata_3** **ccaaactagaaaccagaaaagacgatactttgaatcttaatcttaaaagtattctatcaa**

**Citrus_aurantiifolia** **ccaaactagaaaccagaaaagaagatactttgaatcttaatcttaaaagtattctatcaa**

**Citrus_aurantium** **ccaaactagaaaccagaaaagaagatactttgaatcttaatcttaaaagtattctatcaa**

**Citrus_cavaleriei** **ccaaactagaaaccagaaaagaagatactttgaatcttaatcttaaaagtattctatcaa**

**Citrus_hongheensis** **ccaaactagaaaccagaaaagaagatactttgaatcttaatcttaaaagtattctatcaa**

**Citrus_limon** **ccaaactagaaaccagaaaagaagatactttgaatcttaatcttaaaagtattctatcaa**

**Citrus_maxima** **ccaaactagaaaccagaaaagaagatactttgaatcttaatcttaaaagtattctatcaa**

**Citrus_medica** **ccaaactagaaaccagaaaagaagatactttgaatcttaatcttaaaagtattctatcaa**

**Citrus_polytrifolia** **ccaaactagaaaccagaaaagacgatactttgaatcttaatcttaaaagtattctatcaa**

**Citrus_reticulata** **ccaaactagaaaccagaaaagaagatactttgaatcttaatcttaaaagtattctatcaa**

**Citrus_sinensis** **ccaaactagaaaccagaaaagaagatactttgaatcttaatcttaaaagtattctatcaa**

**Fortunella_bawangica_1** **ccaaactagaaaccagaaaagaagatactttgaatctgaatcttaaaagtattctatcaa**

**Fortunella_bawangica_2** **ccaaactagaaaccagaaaagaagatactttgaatctgaatcttaaaagtattctatcaa**

**Fortunella_hindsii_1** **ccaaactagaaaccagaaaagaagatactttgaatctgaatcttaaaagtattctatcaa**

**Fortunella_hindsii_2** **ccaaactagaaaccagaaaagaagatactttgaatctgaatcttaaaagtattctatcaa**

**Fortunella_hindsii_3** **ccaaactagaaaccagaaaagaagatactttgaatctgaatcttaaaagtattctatcaa**

**Fortunella_japonica_1** **ccaaactagaaaccagaaaagaagatactttgaatctgaatcttaaaagtattctatcaa**

**Fortunella_japonica_2** **ccaaactagaaaccagaaaagaagatactttgaatctgaatcttaaaagtattctatcaa**

**Fortunella_japonica_3** **ccaaactagaaaccagaaaagaagatactttgaatctgaatcttaaaagtattctatcaa**

**Fortunella_japonica_4** **ccaaactagaaaccagaaaagaagatactttgaatctgaatcttaaaagtattctatcaa**

**Fortunella_japonica_5** **ccaaactagaaaccagaaaagaagatactttgaatctgaatcttaaaagtattctatcaa**

**Fortunella_japonica_6** **ccaaactagaaaccagaaaagaagatactttgaatctgaatcttaaaagtattctatcaa**

**Fortunella_japonica_7** **ccaaactagaaaccagaaaagaagatactttgaatctgaatcttaaaagtattctatcaa**

**Fortunella_japonica_8** **ccaaactagaaaccagaaaagaagatactttgaatctgaatcttaaaagtattctatcaa**

**Fortunella_japonica_9** **ccaaactagaaaccagaaaagaagatactttgaatctgaatcttaaaagtattctatcaa**

**Fortunella_japonica_10** **ccaaactagaaaccagaaaagaagatactttgaatctgaatcttaaaagtattctatcaa**

**Fortunella_japonica_11** **ccaaactagaaaccagaaaagaagatactttgaatctgaatcttaaaagtattctatcaa**

**Fortunella_japonica_12** **ccaaactagaaaccagaaaagaagatactttgaatctgaatcttaaaagtattctatcaa**

**Fortunella_margarita_1** **ccaaactagaaaccagaaaagaagatactttgaatctgaatcttaaaagtattctatcaa**

**Fortunella_margarita_2** **ccaaactagaaaccagaaaagaagatactttgaatctgaatcttaaaagtattctatcaa**

**Fortunella_venosa_1** **ccaaactagaaaccagaaaagaagatactttgaatctgaatcttaaaagtattctatcaa**

**Fortunella_venosa_2** **ccaaactagaaaccagaaaagaagatactttgaatctgaatcttaaaagtattctatcaa**

**Fortunella_venosa_3** **ccaaactagaaaccagaaaagaagatactttgaatctgaatcttaaaagtattctatcaa**

34030	34040	34050	34060	34070	34080

....|....|....|....|....|....|....|....|....|....|....|....|

**Atalantia_kwangtungens** **------------------------------------------------------------**

**Citrus_reticulata_1** **agtaacgatcttaaattcagttgtgtatacgctcccgggaacgatatggtactcgattcc**

**Citrus_reticulata_2** **agtaacgatcttaaattcagttgtgtatacgctcccgggaacgatatggtactcgattcc**

**Citrus_trifoliata_3** **agtaacgatcttaaattcagttgtgtatacgctcccgggaacgatatggtactcgattcc**

**Citrus_aurantiifolia** **agtaacgatcttaaattcagttgtgtatacgctcccgggaacgatatggtactcgattcc**

**Citrus_aurantium** **agtaacgatcttaaattcagttgtgtatacgctcccgggaacgatatggtactcgattcc**

**Citrus_cavaleriei** **agtaacgatcttaaattcagttgtgtatacgctcccgggaacgatatggtactcgattcc**

**Citrus_hongheensis** **agtaacgatcttaaattcagttgtgtatacgctcccgggaacgatatggtactcgattcc**

**Citrus_limon** **agtaacgatcttaaattcagttgtgtatacgctcccgggaacgatatggtactcgattcc**

**Citrus_maxima** **agtaacgatcttaaattcagttgtgtatacgctcccgggaacgatatggtactcgattcc**

**Citrus_medica** **agtaacaatcttaaattcagttgtgtatacgctcccgggaacgatatggtactcgattcc**

**Citrus_polytrifolia** **agtaacgatcttaaattcagttgtgtatacgctcccgggaacgatatggtactcgattcc**

**Citrus_reticulata** **agtaacgatcttaaattcagttgtgtatacgctcccgggaacgatatggtactcgattcc**

**Citrus_sinensis** **agtaacgatcttaaattcagttgtgtatacgctcccgggaacgatatggtactcgattcc**

**Fortunella_bawangica_1** **agtaacgatcttaaattcagttgtgtatacgctcccgggaacgatatggtactcgattcc**

**Fortunella_bawangica_2** **agtaacgatcttaaattcagttgtgtatacgctcccgggaacgatatggtactcgattcc**

**Fortunella_hindsii_1** **agtaacgatcttaaattcagttgtgtatacgctcccgggaacgatatggtactcgattcc**

**Fortunella_hindsii_2** **agtaacgatcttaaattcagttgtgtatacgctcccgggaacgatatggtactcgattcc**

**Fortunella_hindsii_3** **agtaacgatcttaaattcagttgtgtatacgctcccgggaacgatatggtactcgattcc**

**Fortunella_japonica_1** **agtaacgatcttaaattcagttgtgtatacgctcccgggaacgatatggtactcgattcc**

**Fortunella_japonica_2** **agtaacgatcttaaattcagttgtgtatacgctcccgggaacgatatggtactcgattcc**

**Fortunella_japonica_3** **agtaacgatcttaaattcagttgtgtatacgctcccgggaacgatatggtactcgattcc**

**Fortunella_japonica_4** **agtaacgatcttaaattcagttgtgtatacgctcccgggaacgatatggtactcgattcc**

**Fortunella_japonica_5** **agtaacgatcttaaattcagttgtgtatacgctcccgggaacgatatggtactcgattcc**

**Fortunella_japonica_6** **agtaacgatcttaaattcagttgtgtatacgctcccgggaacgatatggtactcgattcc**

**Fortunella_japonica_7** **agtaacgatcttaaattcagttgtgtatacgctcccgggaacgatatggtactcgattcc**

**Fortunella_japonica_8** **agtaacgatcttaaattcagttgtgtatacgctcccgggaacgatatggtactcgattcc**

**Fortunella_japonica_9** **agtaacgatcttaaattcagttgtgtatacgctcccgggaacgatatggtactcgattcc**

**Fortunella_japonica_10** **agtaacgatcttaaattcagttgtgtatacgctcccgggaacgatatggtactcgattcc**

**Fortunella_japonica_11** **agtaacgatcttaaattcagttgtgtatacgctcccgggaacgatatggtactcgattcc**

**Fortunella_japonica_12** **agtaacgatcttaaattcagttgtgtatacgctcccgggaacgatatggtactcgattcc**

**Fortunella_margarita_1** **agtaacgatcttaaattcagttgtgtatacgctcccgggaacgatatggtactcgattcc**

**Fortunella_margarita_2** **agtaacgatcttaaattcagttgtgtatacgctcccgggaacgatatggtactcgattcc**

**Fortunella_venosa_1** **agtaacgatcttaaattcagttgtgtatacgctcccgggaacgatatggtactcgattcc**

**Fortunella_venosa_2** **agtaacgatcttaaattcagttgtgtatacgctcccgggaacgatatggtactcgattcc**

**Fortunella_venosa_3** **agtaacgatcttaaattcagttgtgtatacgctcccgggaacgatatggtactcgattcc**

34090	34100	34110	34120	34130	34140

....|....|....|....|....|....|....|....|....|....|....|....|

**Atalantia_kwangtungens** **------------------------------------------------------------**

**Citrus_reticulata_1** **attccatacacagatgggggacagtcaaaaaaaccagaccccctacggtagctcttggaa**

**Citrus_reticulata_2** **attccatacacagatgggggacagtcaaaaaaaccagaccccctacggtagctcttggaa**

**Citrus_trifoliata_3** **attccatacacagatgggggacagtcaaaaaaaccagaccccctacggtagctcttggaa**

**Citrus_aurantiifolia** **attccatacacagatgggggacagtcaaaaaaaccagaccccctacggtagctcttggaa**

**Citrus_aurantium** **attccatacacagatgggggacagtcaaaaaaaccagaccccctacggtagctcttggaa**

**Citrus_cavaleriei** **attccatacacagatgggggacagtcaaaaaaaccagaccccctacggtagctcttggaa**

**Citrus_hongheensis** **attccatacacagatgggggacagtcaaaaaaaccagaccccctacggtagctcttggaa**

**Citrus_limon** **attccatacacagatgggggacagtcaaaaaaaccagaccccctacggtagctcttggaa**

**Citrus_maxima** **attccatacacagatgggggacagtcaaaaaaaccagaccccctacggtagctcttggaa**

**Citrus_medica** **attccatacacagatgggggacagtcaaaaaaaccagaccccctacggtagctcttggaa**

**Citrus_polytrifolia** **attccatacacagatgggggacagtcaaaaaaaccagaccccctacggtagctcttggaa**

**Citrus_reticulata** **attccatacacagatgggggacagtcaaaaaaaccagaccccctacggtagctcttggaa**

**Citrus_sinensis** **attccatacacagatgggggacagtcaaaaaaaccagaccccctacggtagctcttggaa**

**Fortunella_bawangica_1** **attccatacacagatgggggacagtcaaaaaaaccagaccccctacggtagctcttggaa**

**Fortunella_bawangica_2** **attccatacacagatgggggacagtcaaaaaaaccagaccccctacggtagctcttggaa**

**Fortunella_hindsii_1** **attccatacacagatgggggacagtcaaaaaaaccagaccccctacggtagctcttggaa**

**Fortunella_hindsii_2** **attccatacacagatgggggacagtcaaaaaaaccagaccccctacggtagctcttggaa**

**Fortunella_hindsii_3** **attccatacacagatgggggacagtcaaaaaaaccagaccccctacggtagctcttggaa**

**Fortunella_japonica_1** **attccatacacagatgggggacagtcaaaaaaaccagaccccctacggtagctcttggaa**

**Fortunella_japonica_2** **attccatacacagatgggggacagtcaaaaaaaccagaccccctacggtagctcttggaa**

**Fortunella_japonica_3** **attccatacacagatgggggacagtcaaaaaaaccagaccccctacggtagctcttggaa**

**Fortunella_japonica_4** **attccatacacagatgggggacagtcaaaaaaaccagaccccctacggtagctcttggaa**

**Fortunella_japonica_5** **attccatacacagatgggggacagtcaaaaaaaccagaccccctacggtagctcttggaa**

**Fortunella_japonica_6** **attccatacacagatgggggacagtcaaaaaaaccagaccccctacggtagctcttggaa**

**Fortunella_japonica_7** **attccatacacagatgggggacagtcaaaaaaaccagaccccctacggtagctcttggaa**

**Fortunella_japonica_8** **attccatacacagatgggggacagtcaaaaaaaccagaccccctacggtagctcttggaa**

**Fortunella_japonica_9** **attccatacacagatgggggacagtcaaaaaaaccagaccccctacggtagctcttggaa**

**Fortunella_japonica_10** **attccatacacagatgggggacagtcaaaaaaaccagaccccctacggtagctcttggaa**

**Fortunella_japonica_11** **attccatacacagatgggggacagtcaaaaaaaccagaccccctacggtagctcttggaa**

**Fortunella_japonica_12** **attccatacacagatgggggacagtcaaaaaaaccagaccccctacggtagctcttggaa**

**Fortunella_margarita_1** **attccatacacagatgggggacagtcaaaaaaaccagaccccctacggtagctcttggaa**

**Fortunella_margarita_2** **attccatacacagatgggggacagtcaaaaaaaccagaccccctacggtagctcttggaa**

**Fortunella_venosa_1** **attccatacacagatgggggacagtcaaaaaaaccagaccccctacggtagctcttggaa**

**Fortunella_venosa_2** **attccatacacagatgggggacagtcaaaaaaaccagaccccctacggtagctcttggaa**

**Fortunella_venosa_3** **attccatacacagatgggggacagtcaaaaaaaccagaccccctacggtagctcttggaa**

34150	34160	34170	34180	34190	34200

....|....|....|....|....|....|....|....|....|....|....|....|

**Atalantia_kwangtungens** **------------------------------------------------------------**

**Citrus_reticulata_1** **tcctgaatatgatgctaccaataattgtagcaattcgcacatgtctctttctcatcaatc**

**Citrus_reticulata_2** **tcctgaatatgatgctaccaataattgtagcaattcgcacatgtctctttctcatcaatc**

**Citrus_trifoliata_3** **gcctgaatatgatgctacctataagtgtagcaattcgcacatgtctctttctcatcaatc**

**Citrus_aurantiifolia** **tcctgaatatgatgctaccaataattgtagcaattcgcacatgtctctttctcatcaatc**

**Citrus_aurantium** **tcctgaatatgatgctaccaataattgtagcaattcgcacatgtctctttctcatcaatc**

**Citrus_cavaleriei** **tcctgaatatgatgctaccgataattgtagcaattcgcacatgtctctttctcatcaatc**

**Citrus_hongheensis** **tcctgaatatgatgctaccaataattgtagcaattcgcacatgtctctttctcatcaatc**

**Citrus_limon** **tcctgaatatgatgctaccaataattgtagcaattcgcacatgtctctttctcatcaatc**

**Citrus_maxima** **tcctgaatatgatgctaccaataattgtagcaattcgcacatgtctctttctcatcaatc**

**Citrus_medica** **tcctgaatatgatgctaccaataattgtagcaattcgcacatgtctctttctcatcaatc**

**Citrus_polytrifolia** **gcctgaatatgatgctacctataagtgtagcaattcgcacatgtctctttctcatcaatc**

**Citrus_reticulata** **tcctgaatatgatgctaccaataattgtagcaattcgcacatgtctctttctcatcaatc**

**Citrus_sinensis** **tcctgaatatgatgctaccaataattgtagcaattcgcacatgtctctttctcatcaatc**

**Fortunella_bawangica_1** **tcctgaatatgatgctaccaataattgtagcaattcgcacatgtctctttctcatcaatc**

**Fortunella_bawangica_2** **tcctgaatatgatgctaccaataattgtagcaattcgcacatgtctctttctcatcaatc**

**Fortunella_hindsii_1** **tcctgaatatgatgctaccaataattgtagcaattcgcacatgtctctttctcatcaatc**

**Fortunella_hindsii_2** **tcctgaatatgatgctaccaataattgtagcaattcgcacatgtctctttctcatcaatc**

**Fortunella_hindsii_3** **tcctgaatatgatgctaccaataattgtagcaattcgcacatgtctctttctcatcaatc**

**Fortunella_japonica_1** **tcctgaatatgatgctaccaataattgtagcaattcgcacatgtctctttctcatcaatc**

**Fortunella_japonica_2** **tcctgaatatgatgctaccaataattgtagcaattcgcacatgtctctttctcatcaatc**

**Fortunella_japonica_3** **tcctgaatatgatgctaccaataattgtagcaattcgcacatgtctctttctcatcaatc**

**Fortunella_japonica_4** **tcctgaatatgatgctaccaataattgtagcaattcgcacatgtctctttctcatcaatc**

**Fortunella_japonica_5** **tcctgaatatgatgctaccaataattgtagcaattcgcacatgtctctttctcatcaatc**

**Fortunella_japonica_6** **tcctgaatatgatgctaccaataattgtagcaattcgcacatgtctctttctcatcaatc**

**Fortunella_japonica_7** **tcctgaatatgatgctaccaataattgtagcaattcgcacatgtctctttctcatcaatc**

**Fortunella_japonica_8** **tcctgaatatgatgctaccaataattgtagcaattcgcacatgtctctttctcatcaatc**

**Fortunella_japonica_9** **tcctgaatatgatgctaccaataattgtagcaattcgcacatgtctctttctcatcaatc**

**Fortunella_japonica_10** **tcctgaatatgatgctaccaataattgtagcaattcgcacatgtctctttctcatcaatc**

**Fortunella_japonica_11** **tcctgaatatgatgctaccaataattgtagcaattcgcacatgtctctttctcatcaatc**

**Fortunella_japonica_12** **tcctgaatatgatgctaccaataattgtagcaattcgcacatgtctctttctcatcaatc**

**Fortunella_margarita_1** **tcctgaatatgatgctaccaataattgtagcaattcgcacatgtctctttctcatcaatc**

**Fortunella_margarita_2** **tcctgaatatgatgctaccaataattgtagcaattcgcacatgtctctttctcatcaatc**

**Fortunella_venosa_1** **tcctgaatatgatgctaccaataattgtagcaattcgcacatgtctctttctcatcaatc**

**Fortunella_venosa_2** **tcctgaatatgatgctaccaataattgtagcaattcgcacatgtctctttctcatcaatc**

**Fortunella_venosa_3** **tcctgaatatgatgctaccaataattgtagcaattcgcacatgtctctttctcatcaatc**

34210	34220	34230	34240	34250	34260

....|....|....|....|....|....|....|....|....|....|....|....|

**Atalantia_kwangtungens** **--------------------------gaaaaagaaaaaagagaaaaggagagcagtaggc**

**Citrus_reticulata_1** **ggtactggttgatgagaaagagaaatgaaaaagaaaaaagagcaaaggagagcagtaggc**

**Citrus_reticulata_2** **ggtactggttgatgagaaagagaaatgaaaaagaaaaaagagcaaaggagagcagtaggc**

**Citrus_trifoliata_3** **ggtactggttgatgagaaatcgaaatgaaaaagaaaaaagagcaaaggagagcagtaggc**

**Citrus_aurantiifolia** **ggtactagttgatgagaaatcgaaatgaaaaagaaaaaagagcaaaggagagcagtaggc**

**Citrus_aurantium** **ggtactagttgatgagaaatcgaaatgaaaaagaaaaaagagcaaaggagagcagtaggc**

**Citrus_cavaleriei** **ggtactggttgatgagaaagagaaatgaaaaagaaaaaagagcaaaggagagcagtaggc**

**Citrus_hongheensis** **ggtactagttgatgagaaatcgaaatgaaaaagaaaaaagagcaaaggagagcagtaggc**

**Citrus_limon** **ggtactagttgatgagaaatcgaaatgaaaaagaaaaaagagcaaaggagagcagtaggc**

**Citrus_maxima** **ggtactagttgatgagaaatcgaaatgaaaaagaaaaaagagcaaaggagagcagtaggc**

**Citrus_medica** **ggtactggttgatgagaaagagaaatgaaaaagaaaaaagagcaaaggagagcagtaggc**

**Citrus_polytrifolia** **ggtactggttgatgagaaatcgaaatgaaaaagaaaaaagagcaaaggagagcagtaggc**

**Citrus_reticulata** **ggtactggttgatgagaaagagaaatgaaaaagaaaaaagagcaaaggagagcagtaggc**

**Citrus_sinensis** **ggtactagttgatgagaaatcgaaatgaaaaagaaaaaagagcaaaggagagcagtaggc**

**Fortunella_bawangica_1** **ggtactggttgatgagaaatcgaaatgaaaaagaaaaaagagcaaaggagagcagtaggc**

**Fortunella_bawangica_2** **ggtactggttgatgagaaatcgaaatgaaaaagaaaaaagagcaaaggagagcagtaggc**

**Fortunella_hindsii_1** **ggtactggttgatgagaaatcgaaatgaaaaagaaaaaagagcaaaggagagcagtaggc**

**Fortunella_hindsii_2** **ggtactggttgatgagaaatcgaaatgaaaaagaaaaaagagcaaaggagagcagtaggc**

**Fortunella_hindsii_3** **ggtactggttgatgagaaatcgaaatgaaaaagaaaaaagagcaaaggagagcagtaggc**

**Fortunella_japonica_1** **ggtactggttgatgagaaatcgaaatgaaaaagaaaaaagagcaaaggagagcagtaggc**

**Fortunella_japonica_2** **ggtactggttgatgagaaatcgaaatgaaaaagaaaaaagagcaaaggagagcagtaggc**

**Fortunella_japonica_3** **ggtactggttgatgagaaatcgaaatgaaaaagaaaaaagagcaaaggagagcagtaggc**

**Fortunella_japonica_4** **ggtactggttgatgagaaatcgaaatgaaaaagaaaaaagagcaaaggagagcagtaggc**

**Fortunella_japonica_5** **ggtactggttgatgagaaatcgaaatgaaaaagaaaaaagagcaaaggagagcagtaggc**

**Fortunella_japonica_6** **ggtactggttgatgagaaatcgaaatgaaaaagaaaaaagagcaaaggagagcagtaggc**

**Fortunella_japonica_7** **ggtactggttgatgagaaatcgaaatgaaaaagaaaaaagagcaaaggagagcagtaggc**

**Fortunella_japonica_8** **ggtactggttgatgagaaatcgaaatgaaaaagaaaaaagagcaaaggagagcagtaggc**

**Fortunella_japonica_9** **ggtactggttgatgagaaatcgaaatgaaaaagaaaaaagagcaaaggagagcagtaggc**

**Fortunella_japonica_10** **ggtactggttgatgagaaatcgaaatgaaaaagaaaaaagagcaaaggagagcagtaggc**

**Fortunella_japonica_11** **ggtactggttgatgagaaatcgaaatgaaaaagaaaaaagagcaaaggagagcagtaggc**

**Fortunella_japonica_12** **ggtactggttgatgagaaatcgaaatgaaaaagaaaaaagagcaaaggagagcagtaggc**

**Fortunella_margarita_1** **ggtactggttgatgagaaatcgaaatgaaaaagaaaaaagagcaaaggagagcagtaggc**

**Fortunella_margarita_2** **ggtactggttgatgagaaatcgaaatgaaaaagaaaaaagagcaaaggagagcagtaggc**

**Fortunella_venosa_1** **ggtactggttgatgagaaatcgaaatgaaaaagaaaaaagagcaaaggagagcagtaggc**

**Fortunella_venosa_2** **ggtactggttgatgagaaatcgaaatgaaaaagaaaaaagagcaaaggagagcagtaggc**

**Fortunella_venosa_3** **ggtactggttgatgagaaatcgaaatgaaaaagaaaaaagagcaaaggagagcagtaggc**

34270	34280	34290	34300	34310	34320

....|....|....|....|....|....|....|....|....|....|....|....|

**Atalantia_kwangtungens** **atgaaattataccatttt------tttttgccccagtttaacagaaacggcgagatctat**

**Citrus_reticulata_1** **atgaaattctaccatttt------attttgccccagtttaacagaaacggcgagatatat**

**Citrus_reticulata_2** **atgaaattctaccatttt------attttgccccagtttaacagaaacggcgagatatat**

**Citrus_trifoliata_3** **atgaaattctaccatttt------attttgccccagtttaacagaaacggcgagatatat**

**Citrus_aurantiifolia** **atgaaattctaccatttt------attttgccccagtttaacagaaacggcgagatatat**

**Citrus_aurantium** **atgaaattctaccatttt------attttgccccagtttaacagaaacggcgagatatat**

**Citrus_cavaleriei** **atgaaattctaccatttt------attttgccccagtttaacagcaacggcgagatatat**

**Citrus_hongheensis** **atgaaattctaccatttt------attttgccccagtttaacagaaacggcgagatatat**

**Citrus_limon** **atgaaattctaccatttt------attttgccccagtttaacagaaacggcgagatatat**

**Citrus_maxima** **atgaaattctaccatttt------attttgccccagtttaacagaaacggcgagatatat**

**Citrus_medica** **atgaaattctaccatttt------attttgccccagtttaacagaaacggcgagatatat**

**Citrus_polytrifolia** **atgaaattctaccatttt------attttgccccagtttaacagaaacggcgagatatat**

**Citrus_reticulata** **atgaaattctaccatttt------attttgccccagtttaacagaaacggcgagatatat**

**Citrus_sinensis** **atgaaattctaccatttt------attttgccccagtttaacagaaacggcgagatatat**

**Fortunella_bawangica_1** **atgaaattctaccattttattttgattttgccccagtttaacagaaacggcgagatatat**

**Fortunella_bawangica_2** **atgaaattctaccattttattttgattttgccccagtttaacagaaacggcgagatatat**

**Fortunella_hindsii_1** **atgaaattctaccattttattttgattttgccccagtttaacagaaacggcgagatatat**

**Fortunella_hindsii_2** **atgaaattctaccattttattttgattttgccccagtttaacagaaacggcgagatatat**

**Fortunella_hindsii_3** **atgaaattctaccattttattttgattttgccccagtttaacagaaacggcgagatatat**

**Fortunella_japonica_1** **atgaaattctaccattttattttgattttgccccagtttaacagaaacggcgagatatat**

**Fortunella_japonica_2** **atgaaattctaccattttattttgattttgccccagtttaacagaaacggcgagatatat**

**Fortunella_japonica_3** **atgaaattctaccattttattttgattttgccccagtttaacagaaacggcgagatatat**

**Fortunella_japonica_4** **atgaaattctaccattttattttgattttgccccagtttaacagaaacggcgagatatat**

**Fortunella_japonica_5** **atgaaattctaccattttattttgattttgccccagtttaacagaaacggcgagatatat**

**Fortunella_japonica_6** **atgaaattctaccattttattttgattttgccccagtttaacagaaacggcgagatatat**

**Fortunella_japonica_7** **atgaaattctaccattttattttgattttgccccagtttaacagaaacggcgagatatat**

**Fortunella_japonica_8** **atgaaattctaccattttattttgattttgccccagtttaacagaaacggcgagatatat**

**Fortunella_japonica_9** **atgaaattctaccattttattttgattttgccccagtttaacagaaacggcgagatatat**

**Fortunella_japonica_10** **atgaaattctaccattttattttgattttgccccagtttaacagaaacggcgagatatat**

**Fortunella_japonica_11** **atgaaattctaccattttattttgattttgccccagtttaacagaaacggcgagatatat**

**Fortunella_japonica_12** **atgaaattctaccattttattttgattttgccccagtttaacagaaacggcgagatatat**

**Fortunella_margarita_1** **atgaaattctaccattttattttgattttgccccagtttaacagaaacggcgagatatat**

**Fortunella_margarita_2** **atgaaattctaccattttattttgattttgccccagtttaacagaaacggcgagatatat**

**Fortunella_venosa_1** **atgaaattctaccattttattttgattttgccccagtttaacagaaacggcgagatatat**

**Fortunella_venosa_2** **atgaaattctaccattttattttgattttgccccagtttaacagaaacggcgagatatat**

**Fortunella_venosa_3** **atgaaattctaccattttattttgattttgccccagtttaacagaaacggcgagatatat**

34330	34340	34350	34360	34370	34380

....|....|....|....|....|....|....|....|....|....|....|....|

**Atalantia_kwangtungens** **tgatg---ttttttttattggatttggatccgtcgggactgacggggctcgaacccgcag**

**Citrus_reticulata_1** **tgatg-ttttttttttattggatttggatccgtcgggactgacggggctcgaacccgcag**

**Citrus_reticulata_2** **tgatg-ttttttttttattggatttggatccgtcgggactgacggggctcgaacccgcag**

**Citrus_trifoliata_3** **tgatgtttttttttttattggatttggatccgtcgggactgacggggctcgaacccgcag**

**Citrus_aurantiifolia** **tgatg--tttttttttattggatttggatccgtcgggactgacggggctcgaacccgcag**

**Citrus_aurantium** **tgatg--tttttttttattggatttggatccgtcgggactgacggggctcgaacccgcag**

**Citrus_cavaleriei** **tgatg-ttttttttttattggatttggatccgtcgggactgacggggctcgaacccgcag**

**Citrus_hongheensis** **tgatg--tttttttttattggatttggatccgtcgggactgacggggctcgaacccgcag**

**Citrus_limon** **tgatg--tttttttttattggatttggatccgtcgggactgacggggctcgaacccgcag**

**Citrus_maxima** **tgatg--tttttttttattggatttggatccgtcgggactgacggggctcgaacccgcag**

**Citrus_medica** **tgatgtttttttttttattggatttggatccgtcgggactgacggggctcgaacccgcag**

**Citrus_polytrifolia** **tgatg--tttttttttattggatttggatccgtcgggactgacggggctcgaacccgcag**

**Citrus_reticulata** **tgatg-ttttttttttattggatttggatccgtcgggactgacggggctcgaacccgcag**

**Citrus_sinensis** **tgatg--tttttttttattggatttggatccgtcgggactgacggggctcgaacccgcag**

**Fortunella_bawangica_1** **ttatg--tttttttttattggatttggatccgtcgggactgacggggctcgaacccgcag**

**Fortunella_bawangica_2** **ttatg--tttttttttattggatttggatccgtcgggactgacggggctcgaacccgcag**

**Fortunella_hindsii_1** **ttatg--tttttttttattggatttggatccgtcgggactgacggggctcgaacccgcag**

**Fortunella_hindsii_2** **ttatg--tttttttttattggatttggatccgtcgggactgacggggctcgaacccgcag**

**Fortunella_hindsii_3** **ttatg--tttttttttattggatttggatccgtcgggactgacggggctcgaacccgcag**

**Fortunella_japonica_1** **ttatg--tttttttttattggatttggatccgtcgggactgacggggctcgaacccgcag**

**Fortunella_japonica_2** **ttatg--tttttttttattggatttggatccgtcgggactgacggggctcgaacccgcag**

**Fortunella_japonica_3** **ttatg--tttttttttattggatttggatccgtcgggactgacggggctcgaacccgcag**

**Fortunella_japonica_4** **ttatg--tttttttttattggatttggatccgtcgggactgacggggctcgaacccgcag**

**Fortunella_japonica_5** **ttatg--tttttttttattggatttggatccgtcgggactgacggggctcgaacccgcag**

**Fortunella_japonica_6** **ttatg--tttttttttattggatttggatccgtcgggactgacggggctcgaacccgcag**

**Fortunella_japonica_7** **ttatg--tttttttttattggatttggatccgtcgggactgacggggctcgaacccgcag**

**Fortunella_japonica_8** **ttatg--tttttttttattggatttggatccgtcgggactgacggggctcgaacccgcag**

**Fortunella_japonica_9** **ttatg--tttttttttattggatttggatccgtcgggactgacggggctcgaacccgcag**

**Fortunella_japonica_10** **ttatg--tttttttttattggatttggatccgtcgggactgacggggctcgaacccgcag**

**Fortunella_japonica_11** **ttatg--tttttttttattggatttggatccgtcgggactgacggggctcgaacccgcag**

**Fortunella_japonica_12** **ttatg--tttttttttattggatttggatccgtcgggactgacggggctcgaacccgcag**

**Fortunella_margarita_1** **ttatg--tttttttttattggatttggatccgtcgggactgacggggctcgaacccgcag**

**Fortunella_margarita_2** **ttatg--tttttttttattggatttggatccgtcgggactgacggggctcgaacccgcag**

**Fortunella_venosa_1** **ttatg--tttttttttattggatttggatccgtcgggactgacggggctcgaacccgcag**

**Fortunella_venosa_2** **ttatg--tttttttttattggatttggatccgtcgggactgacggggctcgaacccgcag**

**Fortunella_venosa_3** **ttatg--tttttttttattggatttggatccgtcgggactgacggggctcgaacccgcag**

34390	34400	34410	34420	34430	34440

....|....|....|....|....|....|....|....|....|....|....|....|

**Atalantia_kwangtungens** **cttccgccttgacagggcggtgctctgaccaattgaactacaatcccggggcggtaaaat**

**Citrus_reticulata_1** **cttccgccttgacagggcggtgctctgaccaattgaactacaatcccggggcggtaaaat**

**Citrus_reticulata_2** **cttccgccttgacagggcggtgctctgaccaattgaactacaatcccggggcggtaaaat**

**Citrus_trifoliata_3** **cttccgccttgacagggcggtgctctgaccaattgaactacaatcccggggcggtaaaat**

**Citrus_aurantiifolia** **cttccgccttgacagggcggtgctctgaccaattgaactacaatcccggggcggtaaaat**

**Citrus_aurantium** **cttccgccttgacagggcggtgctctgaccaattgaactacaatcccggggcggtaaaat**

**Citrus_cavaleriei** **cttccgccttgacagggcggtgctctgaccaattgaactacaatcccggggcggtaaaat**

**Citrus_hongheensis** **cttccgccttgacagggcggtgctctgaccaattgaactacaatcccggggcggtaaaat**

**Citrus_limon** **cttccgccttgacagggcggtgctctgaccaattgaactacaatcccggggcggtaaaat**

**Citrus_maxima** **cttccgccttgacagggcggtgctctgaccaattgaactacaatcccggggcggtaaaat**

**Citrus_medica** **cttccgccttgacagggcggtgctctgaccaattgaactacaatcccggggcggtaaaat**

**Citrus_polytrifolia** **cttccgccttgacagggcggtgctctgaccaattgaactacaatcccggggcggtaaaat**

**Citrus_reticulata** **cttccgccttgacagggcggtgctctgaccaattgaactacaatcccggggcggtaaaat**

**Citrus_sinensis** **cttccgccttgacagggcggtgctctgaccaattgaactacaatcccggggcggtaaaat**

**Fortunella_bawangica_1** **cttccgccttgacagggcggtgctctgaccaattgaactacaatcccggggcggtaaaat**

**Fortunella_bawangica_2** **cttccgccttgacagggcggtgctctgaccaattgaactacaatcccggggcggtaaaat**

**Fortunella_hindsii_1** **cttccgccttgacagggcggtgctctgaccaattgaactacaatcccggggcggtaaaat**

**Fortunella_hindsii_2** **cttccgccttgacagggcggtgctctgaccaattgaactacaatcccggggcggtaaaat**

**Fortunella_hindsii_3** **cttccgccttgacagggcggtgctctgaccaattgaactacaatcccggggcggtaaaat**

**Fortunella_japonica_1** **cttccgccttgacagggcggtgctctgaccaattgaactacaatcccggggcggtaaaat**

**Fortunella_japonica_2** **cttccgccttgacagggcggtgctctgaccaattgaactacaatcccggggcggtaaaat**

**Fortunella_japonica_3** **cttccgccttgacagggcggtgctctgaccaattgaactacaatcccggggcggtaaaat**

**Fortunella_japonica_4** **cttccgccttgacagggcggtgctctgaccaattgaactacaatcccggggcggtaaaat**

**Fortunella_japonica_5** **cttccgccttgacagggcggtgctctgaccaattgaactacaatcccggggcggtaaaat**

**Fortunella_japonica_6** **cttccgccttgacagggcggtgctctgaccaattgaactacaatcccggggcggtaaaat**

**Fortunella_japonica_7** **cttccgccttgacagggcggtgctctgaccaattgaactacaatcccggggcggtaaaat**

**Fortunella_japonica_8** **cttccgccttgacagggcggtgctctgaccaattgaactacaatcccggggcggtaaaat**

**Fortunella_japonica_9** **cttccgccttgacagggcggtgctctgaccaattgaactacaatcccggggcggtaaaat**

**Fortunella_japonica_10** **cttccgccttgacagggcggtgctctgaccaattgaactacaatcccggggcggtaaaat**

**Fortunella_japonica_11** **cttccgccttgacagggcggtgctctgaccaattgaactacaatcccggggcggtaaaat**

**Fortunella_japonica_12** **cttccgccttgacagggcggtgctctgaccaattgaactacaatcccggggcggtaaaat**

**Fortunella_margarita_1** **cttccgccttgacagggcggtgctctgaccaattgaactacaatcccggggcggtaaaat**

**Fortunella_margarita_2** **cttccgccttgacagggcggtgctctgaccaattgaactacaatcccggggcggtaaaat**

**Fortunella_venosa_1** **cttccgccttgacagggcggtgctctgaccaattgaactacaatcccggggcggtaaaat**

**Fortunella_venosa_2** **cttccgccttgacagggcggtgctctgaccaattgaactacaatcccggggcggtaaaat**

**Fortunella_venosa_3** **cttccgccttgacagggcggtgctctgaccaattgaactacaatcccggggcggtaaaat**

34450	34460	34470	34480	34490	34500

....|....|....|....|....|....|....|....|....|....|....|....|

**Atalantia_kwangtungens** **gtaccgaataccgatttttatgatttcattcaaaccatttcatttatatttagataaaaa**

**Citrus_reticulata_1** **gtaccgaatacctatttttatgatttcattcaaacaatttcatttatatttagataaata**

**Citrus_reticulata_2** **gtaccgaatacctatttttatgatttcattcaaacaatttcatttatatttagataaata**

**Citrus_trifoliata_3** **gtaccgaatacctatttttatgatttcattcaaacaatttcatttatatttagataaata**

**Citrus_aurantiifolia** **gtaccgaatacctatttttatgatttcattcaaacaatttcatttatatttcgataaata**

**Citrus_aurantium** **gtaccgaatacctatttttatgatttcattcaaacaatttcatttatatttagataaata**

**Citrus_cavaleriei** **gtaccgaatacctatttttatgatttcattcaaacaatttcatttctatttagataaata**

**Citrus_hongheensis** **gtaccgaatacctatttttatgatttcattcaaacaatttcatttatatttagataaata**

**Citrus_limon** **gtaccgaatacctatttttatgatttcattcaaacaatttcatttatatttagataaata**

**Citrus_maxima** **gtaccgaatacctatttttatgatttcattcaaacaatttcatttatatttagataaata**

**Citrus_medica** **gtaccgaatacctatttttatgatttcattcaaacaatttcatttatatttagataaata**

**Citrus_polytrifolia** **gtaccgaatacctatttttatgatttcattcaaacaatttcatttatatttagataaata**

**Citrus_reticulata** **gtaccgaatacctatttttatgatttcattcaaacaatttcatttatatttagataaata**

**Citrus_sinensis** **gtaccgaatacctatttttatgatttcattcaaacaatttcatttatatttagataaata**

**Fortunella_bawangica_1** **gtaccgaatacctatttttatgatttcattcaaacaatttcatttatatttagataaata**

**Fortunella_bawangica_2** **gtaccgaatacctatttttatgatttcattcaaacaatttcatttatatttagataaata**

**Fortunella_hindsii_1** **gtaccgaatacctatttttatgatttcattcaaacaatttcatttatatttagataaata**

**Fortunella_hindsii_2** **gtaccgaatacctatttttatgatttcattcaaacaatttcatttatatttagataaata**

**Fortunella_hindsii_3** **gtaccgaatacctatttttatgatttcattcaaataatttcatttatatttagataaata**

**Fortunella_japonica_1** **gtaccgaatacctatttttatgatttcattcaaacaatttcatttatatttagataaata**

**Fortunella_japonica_2** **gtaccgaatacctatttttatgatttcattcaaacaatttcatttatatttagataaata**

**Fortunella_japonica_3** **gtaccgaatacctatttttatgatttcattcaaacaatttcatttatatttagataaata**

**Fortunella_japonica_4** **gtaccgaatacctatttttatgatttcattcaaacaatttcatttatatttagataaata**

**Fortunella_japonica_5** **gtaccgaatacctatttttatgatttcattcaaacaatttcatttatatttagataaata**

**Fortunella_japonica_6** **gtaccgaatacctatttttatgatttcattcaaacaatttcatttatatttagataaata**

**Fortunella_japonica_7** **gtaccgaatacctatttttatgatttcattcaaacaatttcatttatatttagataaata**

**Fortunella_japonica_8** **gtaccgaatacctatttttatgatttcattcaaacaatttcatttatatttagataaata**

**Fortunella_japonica_9** **gtaccgaatacctatttttatgatttcattcaaacaatttcatttatatttagataaata**

**Fortunella_japonica_10** **gtaccgaatacctatttttatgatttcattcaaacaatttcatttatatttagataaata**

**Fortunella_japonica_11** **gtaccgaatacctatttttatgatttcattcaaacaatttcatttatatttagataaata**

**Fortunella_japonica_12** **gtaccgaatacctatttttatgatttcattcaaacaatttcatttatatttagataaata**

**Fortunella_margarita_1** **gtaccgaatacctatttttatgatttcattcaaacaatttcatttatatttagataaata**

**Fortunella_margarita_2** **gtaccgaatacctatttttatgatttcattcaaacaatttcatttatatttagataaata**

**Fortunella_venosa_1** **gtaccgaatacctatttttatgatttcattcaaacaatttcatttatatttagataaata**

**Fortunella_venosa_2** **gtaccgaatacctatttttatgatttcattcaaacaatttcatttatatttagataaata**

**Fortunella_venosa_3** **gtaccgaatacctatttttatgatttcattcaaacaatttcatttatatttagataaata**

34510	34520	34530	34540	34550	34560

....|....|....|....|....|....|....|....|....|....|....|....|

**Atalantia_kwangtungens** **tcgacgtgttggaacagcgacgtgagtgagatattgatatacacacgggtatac-----a**

**Citrus_reticulata_1** **tcgacgtgttggaaccgagacgtgagtgagatattgatatacacacggatatac-----a**

**Citrus_reticulata_2** **tcgacgtgttggaaccgagacgtgagtgagatattgatatacacacggatatac-----a**

**Citrus_trifoliata_3** **tcgacgtgttggaaccgagacgtgagtgagatattgatatacacacggatatac-----a**

**Citrus_aurantiifolia** **tcgacgtgttggaaccgagacgtgagtgagatattgatatacacacggatatac-----a**

**Citrus_aurantium** **tcgacgtgttggaaccgagacgtgagtgagatattgatatacacacggatatac-----a**

**Citrus_cavaleriei** **tcgacgtgttggaaccgagacgtgagtgagatattgatatacacacggatatac-----a**

**Citrus_hongheensis** **tcgacgtgttggaaccgagacgtgagtgagatattgatatacacacggatatac-----a**

**Citrus_limon** **tcgacgtgttggaaccgagacgtgagtgagatattgatatacacacggatatac-----a**

**Citrus_maxima** **tcgacgtgttggaaccgagacgtgagtgagatattgatatacacacggatatac-----a**

**Citrus_medica** **tcgacgtgttggaaccgagacgtgagtgagatattgatatacacacggatatacacttta**

**Citrus_polytrifolia** **tcgacgtgttggaaccgagacgtgagtgagatattgatatacacacggatatac-----a**

**Citrus_reticulata** **tcgacgtgttggaaccgagacgtgagtgagatattgatatacacacggatatac-----a**

**Citrus_sinensis** **tcgacgtgttggaaccgagacgtgagtgagatattgatatacacacggatatac-----a**

**Fortunella_bawangica_1** **tcgacgtgttggaaccgagacgtgagtgagatattgatatacacacggatatac-----a**

**Fortunella_bawangica_2** **tcgacgtgttggaaccgagacgtgagtgagatattgatatacacacggatatac-----a**

**Fortunella_hindsii_1** **tcgacgtgttggaaccgagacgtgagtgagatattgatatacacacggatatac-----a**

**Fortunella_hindsii_2** **tcgacgtgttggaaccgagacgtgagtgagatattgatatacacacggatatac-----a**

**Fortunella_hindsii_3** **tcgacgtgttggaaccgagacgtgagtgagatattgatatacacacggatatac-----a**

**Fortunella_japonica_1** **tcgacgtgttggaaccgagacgtgagtgagatattgatatacacacggatatac-----a**

**Fortunella_japonica_2** **tcgacgtgttggaaccgagacgtgagtgagatattgatatacacacggatatac-----a**

**Fortunella_japonica_3** **tcgacgtgttggaaccgagacgtgagtgagatattgatatacacacggatatac-----a**

**Fortunella_japonica_4** **tcgacgtgttggaaccgagacgtgagtgagatattgatatacacacggatatac-----a**

**Fortunella_japonica_5** **tcgacgtgttggaaccgagacgtgagtgagatattgatatacacacggatatac-----a**

**Fortunella_japonica_6** **tcgacgtgttggaaccgagacgtgagtgagatattgatatacacacggatatac-----a**

**Fortunella_japonica_7** **tcgacgtgttggaaccgagacgtgagtgagatattgatatacacacggatatac-----a**

**Fortunella_japonica_8** **tcgacgtgttggaaccgagacgtgagtgagatattgatatacacacggatatac-----a**

**Fortunella_japonica_9** **tcgacgtgttggaaccgagacgtgagtgagatattgatatacacacggatatac-----a**

**Fortunella_japonica_10** **tcgacgtgttggaaccgagacgtgagtgagatattgatatacacacggatatac-----a**

**Fortunella_japonica_11** **tcgacgtgttggaaccgagacgtgagtgagatattgatatacacacggatatac-----a**

**Fortunella_japonica_12** **tcgacgtgttggaaccgagacgtgagtgagatattgatatacacacggatatac-----a**

**Fortunella_margarita_1** **tcgacgtgttggaaccgagacgtgagtgagatattgatatacacacggatatac-----a**

**Fortunella_margarita_2** **tcgacgtgttggaaccgagacgtgagtgagatattgatatacacacggatatac-----a**

**Fortunella_venosa_1** **tcgacgtgttggaaccgagacgtgagtgagatattgatatacacacggatatac-----a**

**Fortunella_venosa_2** **tcgacgtgttggaaccgagacgtgagtgagatattgatatacacacggatatac-----a**

**Fortunella_venosa_3** **tcgacgtgttggaaccgagacgtgagtgagatattgatatacacacggatatac-----a**

34570	34580	34590	34600	34610	34620

....|....|....|....|....|....|....|....|....|....|....|....|

**Atalantia_kwangtungens** **ctttagtgagagcggcacggattcctttcgttattgccaaatcgattgataattcgtttt**

**Citrus_reticulata_1** **ctttagtgagagcggcacggattcctttcgttattgccaaatcaattgataattcgtttt**

**Citrus_reticulata_2** **ctttagtgagagcggcacggattcctttcgttattgccaaatcaattgataattcgtttt**

**Citrus_trifoliata_3** **ctttagtgagagcggcacgtattcctttcgttattgccaaatcaattgataattcgtttt**

**Citrus_aurantiifolia** **ctttagtgagagcggcacggattcctttcgttattgccaaatcaattgagaattcgtttt**

**Citrus_aurantium** **ctttagtgagagcggcacggattcctttcgttattgccaaatcaattgataattcgtttt**

**Citrus_cavaleriei** **ctttagtgagagcggcacggattcctttcgttattgccaaatcaattgataattcgtttt**

**Citrus_hongheensis** **ctttagtgagagcggcacggattcctttcgttattgccaaatcaattgataattcgtttt**

**Citrus_limon** **ctttagtgagagcggcacggattcctttcgttattgccaaatcaattgataattcgtttt**

**Citrus_maxima** **ctttagtgagagcggcacggattcctttcgttattgccaaatcaattgataattcgtttt**

**Citrus_medica** **ctttagtgagagcggcacggattcctttcgttattgccaaatcaattgataattcgtttt**

**Citrus_polytrifolia** **ctttagtgagagcggcacggattcctttcgttattgccaaatcaattgataattcgtttt**

**Citrus_reticulata** **ctttagtgagagcggcacggattcctttcgttattgccaaatcaattgataattcgtttt**

**Citrus_sinensis** **ctttagtgagagcggcacggattcctttcgttattgccaaatcaattgataattcgtttt**

**Fortunella_bawangica_1** **ctttagtgagagcggcacggattcctttcgttattgccaaatcaattgataattcgtttt**

**Fortunella_bawangica_2** **ctttagtgagagcggcacggattcctttcgttattgccaaatcaattgataattcgtttt**

**Fortunella_hindsii_1** **ctttagtgagagcggcacggattcctttcgttattgccaaatcaattgataattcgtttt**

**Fortunella_hindsii_2** **ctttagtgagagcggcacggattcctttcgttattgccaaatcaattgataattcgtttt**

**Fortunella_hindsii_3** **ctttagtgagagcggcacggattcctttcgttattgccaaatcaattgataattcgtttt**

**Fortunella_japonica_1** **ctttagtgagagcggcacggattcctttcgttattgccaaatcaattgataattcgtttt**

**Fortunella_japonica_2** **ctttagtgagagcggcacggattcctttcgttattgccaaatcaattgataattcgtttt**

**Fortunella_japonica_3** **ctttagtgagagcggcacggattcctttcgttattgccaaatcaattgataattcgtttt**

**Fortunella_japonica_4** **ctttagtgagagcggcacggattcctttcgttattgccaaatcaattgataattcgtttt**

**Fortunella_japonica_5** **ctttagtgagagcggcacggattcctttcgttattgccaaatcaattgataattcgtttt**

**Fortunella_japonica_6** **ctttagtgagagcggcacggattcctttcgttattgccaaatcaattgataattcgtttt**

**Fortunella_japonica_7** **ctttagtgagagcggcacggattcctttcgttattgccaaatcaattgataattcgtttt**

**Fortunella_japonica_8** **ctttagtgagagcggcacggattcctttcgttattgccaaatcaattgataattcgtttt**

**Fortunella_japonica_9** **ctttagtgagagcggcacggattcctttcgttattgccaaatcaattgataattcgtttt**

**Fortunella_japonica_10** **ctttagtgagagcggcacggattcctttcgttattgccaaatcaattgataattcgtttt**

**Fortunella_japonica_11** **ctttagtgagagcggcacggattcctttcgttattgccaaatcaattgataattcgtttt**

**Fortunella_japonica_12** **ctttagtgagagcggcacggattcctttcgttattgccaaatcaattgataattcgtttt**

**Fortunella_margarita_1** **ctttagtgagagcggcacggattcctttcgttattgccaaatcaattgataattcgtttt**

**Fortunella_margarita_2** **ctttagtgagagcggcacggattcctttcgttattgccaaatcaattgataattcgtttt**

**Fortunella_venosa_1** **ctttagtgagagcggcacggattcctttcgttattgccaaatcaattgataattcgtttt**

**Fortunella_venosa_2** **ctttagtgagagcggcacggattcctttcgttattgccaaatcaattgataattcgtttt**

**Fortunella_venosa_3** **ctttagtgagagcggcacggattcctttcgttattgccaaatcaattgataattcgtttt**

34630	34640	34650	34660	34670	34680

....|....|....|....|....|....|....|....|....|....|....|....|

**Atalantia_kwangtungens** **tcaatgtccc-----caatgaaaaaaaaaagactcttttttttgcgttactttattcttt**

**Citrus_reticulata_1** **tcaatgtcccaatgaaaaaaaaaaaaaaaaagagtctttttttgcgttactttattcttt**

**Citrus_reticulata_2** **tcaatgtcccaatgaaaaaaaaaaaaaaaaagagtctttttttgcgttactttattcttt**

**Citrus_trifoliata_3** **tcaatgtcccaatg---aaaaaaaaaaaaaagagtctttttttgcgttactttattcttt**

**Citrus_aurantiifolia** **tcaatgtcccaatg--aaaaaaaaaaaaaaagagtctttttttgcgttactttattcttt**

**Citrus_aurantium** **tcaatgtccc-----------aaaaaaaaaagagtctttttttgcgttactttattcttt**

**Citrus_cavaleriei** **tcaatgtcccaatg----aaaaaaaaaaaaagagtctttttttgcgttactttagtcttt**

**Citrus_hongheensis** **tcaatgtccc-----------aaaaaaaaaagagtctttttttgcgttactttattcttt**

**Citrus_limon** **tcaatgtccc-----------aaaaaaaaaagagtctttttttgcgttactttattcttt**

**Citrus_maxima** **tcaatgtccc-----------aaaaaaaaaagagtctttttttgcgttactttattcttt**

**Citrus_medica** **tcaatgtcccaatg------aaaaaaaaaaagagtctttttttgcgttactttattcttt**

**Citrus_polytrifolia** **tcaatgtcccaatg---aaaaaaaaaaaaaagagtctttttttgcgttactttattcttt**

**Citrus_reticulata** **tcaatgtcccaatgaaaaaaaaaaaaaaaaagagtctttttttgcgttactttattcttt**

**Citrus_sinensis** **tcaatgtccc-----------aaaaaaaaaagagtctttttttgcgttactttattcttt**

**Fortunella_bawangica_1** **tcaatgtcccaatg---aaaaaaaaaaaaaagagtctttttttgcgttactttattcttt**

**Fortunella_bawangica_2** **tcaatgtcccaatg---aaaaaaaaaaaaaagagtctttttttgcgttactttattcttt**

**Fortunella_hindsii_1** **tcaatgtcccaatg------aaaaaaaaaaagagtctttttttgcgttactttattcttt**

**Fortunella_hindsii_2** **tcaatgtcccaatg-------aaaaaaaaaagagtctttttttgcgttactttattcttt**

**Fortunella_hindsii_3** **tcaatgtcccaatg------aaaaaaaaaaagagtctttttttgcgttactttattcttt**

**Fortunella_japonica_1** **tcaatgtcccaatg-----aaaaaaaaaaaagagtctttttttgcgttactttattcttt**

**Fortunella_japonica_2** **tcaatgtcccaatg----aaaaaaaaaaaaagagtctttttttgcgttactttattcttt**

**Fortunella_japonica_3** **tcaatgtcccaatg-----aaaaaaaaaaaagagtctttttttgcgttactttattcttt**

**Fortunella_japonica_4** **tcaatgtcccaatg-----aaaaaaaaaaaagagtctttttttgcgttactttattcttt**

**Fortunella_japonica_5** **tcaatgtcccaatg-----aaaaaaaaaaaagagtctttttttgcgttactttattcttt**

**Fortunella_japonica_6** **tcaatgtcccaatg-----aaaaaaaaaaaagagtctttttttgcgttactttattcttt**

**Fortunella_japonica_7** **tcaatgtcccaatg-----aaaaaaaaaaaagagtctttttttgcgttactttattcttt**

**Fortunella_japonica_8** **tcaatgtcccaatg-----aaaaaaaaaaaagagtctttttttgcgttactttattcttt**

**Fortunella_japonica_9** **tcaatgtcccaatg-----aaaaaaaaaaaagagtctttttttgcgttactttattcttt**

**Fortunella_japonica_10** **tcaatgtcccaatg----aaaaaaaaaaaaagagtctttttttgcgttactttattcttt**

**Fortunella_japonica_11** **tcaatgtcccaatg----aaaaaaaaaaaaagagtctttttttgcgttactttattcttt**

**Fortunella_japonica_12** **tcaatgtcccaatg-----aaaaaaaaaaaagagtctttttttgcgttactttattcttt**

**Fortunella_margarita_1** **tcaatgtcccaatg----aaaaaaaaaaaaagagtctttttttgcgttactttattcttt**

**Fortunella_margarita_2** **tcaatgtcccaatg----aaaaaaaaaaaaagagtctttttttgcgttactttattcttt**

**Fortunella_venosa_1** **tcaatgtcccaatg-----aaaaaaaaaaaagagtctttttttgcgttactttattcttt**

**Fortunella_venosa_2** **tcaatgtcccaatg----aaaaaaaaaaaaagagtctttttttgcgttactttattcttt**

**Fortunella_venosa_3** **tcaatgtcccaatg----aaaaaaaaaaaaagagtctttttttgcgttactttattcttt**

34690	34700	34710	34720	34730	34740

....|....|....|....|....|....|....|....|....|....|....|....|

**Atalantia_kwangtungens** **taattagactttatgctttcgatttcatgatcctgatatgaatctagatcattattagca**

**Citrus_reticulata_1** **tcattagactttatgctttcgatttcatgatcctgatatgaatctagatcattattagca**

**Citrus_reticulata_2** **tcattagactttatgctttcgatttcatgatcctgatatgaatctagatcattattagca**

**Citrus_trifoliata_3** **tcattagactttatgcgttcgatttcatgatcctgatatgaatctagatcattattagca**

**Citrus_aurantiifolia** **tcattagactttatgctttcgatttcatgatcctgatatgaatctagatcattattagca**

**Citrus_aurantium** **tcattagactttatgctttcgatttcatgatcctgatatgaatctagatcattattagca**

**Citrus_cavaleriei** **tcattagactttatgctttcgatttcatgatcctgatatgaatctagatcattattagca**

**Citrus_hongheensis** **tcattagactttatgctttcgatttcatgatcctgatatgaatctagatcattattagca**

**Citrus_limon** **tcattagactttatgctttcgatttcatgatcctgatatgaatctagatcattattagca**

**Citrus_maxima** **tcattagactttatgctttcgatttcatgatcctgatatgaatctagatcattattagca**

**Citrus_medica** **tcattagactttatgctttcgatttcatgatcctgatatgaatctagatcattattagca**

**Citrus_polytrifolia** **tcattagactttatgcgttcgatttcatgatcctgatatgaatctagatcattattagca**

**Citrus_reticulata** **tcattagactttatgctttcgatttcatgatcctgatatgaatctagatcattattagca**

**Citrus_sinensis** **tcattagactttatgctttcgatttcatgatcctgatatgaatctagatcattattagca**

**Fortunella_bawangica_1** **tcattagactttatgctttcgatttcatgatcctgatatgaatctagatcattattagca**

**Fortunella_bawangica_2** **tcattagactttatgctttcgatttcatgatcctgatatgaatctagatcattattagca**

**Fortunella_hindsii_1** **tcattagactttatgctttcgatttcatgatcctgatatgaatctagatcattattagca**

**Fortunella_hindsii_2** **tcattagactttatgctttcgatttcatgatcctgatatgaatctagatcattattagca**

**Fortunella_hindsii_3** **tcattagactttatgctttcgatttcatgatcctgatatgaatctagatcattattagca**

**Fortunella_japonica_1** **tcattagactttatgctttcgatttcatgatcctgatatgaatctagatcattattagca**

**Fortunella_japonica_2** **tcattagactttatgctttcgatttcatgatcctgatatgaatctagatcattattagca**

**Fortunella_japonica_3** **tcattagactttatgctttcgatttcatgatcctgatatgaatctagatcattattagca**

**Fortunella_japonica_4** **tcattagactttatgctttcgatttcatgatcctgatatgaatctagatcattattagca**

**Fortunella_japonica_5** **tcattagactttatgctttcgatttcatgatcctgatatgaatctagatcattattagca**

**Fortunella_japonica_6** **tcattagactttatgctttcgatttcatgatcctgatatgaatctagatcattattagca**

**Fortunella_japonica_7** **tcattagactttatgctttcgatttcatgatcctgatatgaatctagatcattattagca**

**Fortunella_japonica_8** **tcattagactttatgctttcgatttcatgatcctgatatgaatctagatcattattagca**

**Fortunella_japonica_9** **tcattagactttatgctttcgatttcatgatcctgatatgaatctagatcattattagca**

**Fortunella_japonica_10** **tcattagactttatgctttcgatttcatgatcctgatatgaatctagatcattattagca**

**Fortunella_japonica_11** **tcattagactttatgctttcgatttcatgatcctgatatgaatctagatcattattagca**

**Fortunella_japonica_12** **tcattagactttatgctttcgatttcatgatcctgatatgaatctagatcattattagca**

**Fortunella_margarita_1** **tcattagactttatgctttcgatttcatgatcctgatatgaatctagatcattattagca**

**Fortunella_margarita_2** **tcattagactttatgctttcgatttcatgatcctgatatgaatctagatcattattagca**

**Fortunella_venosa_1** **tcattagactttatgctttcgatttcatgatcctgatatgaatctagatcattattagca**

**Fortunella_venosa_2** **tcattagactttatgctttcgatttcatgatcctgatatgaatctagatcattattagca**

**Fortunella_venosa_3** **tcattagactttatgctttcgatttcatgatcctgatatgaatctagatcattattagca**

34750	34760	34770	34780	34790	34800

....|....|....|....|....|....|....|....|....|....|....|....|

**Atalantia_kwangtungens** **agatcagaatttatgggaac------aaataaatggagcaaac-----------------**

**Citrus_reticulata_1** **agatcagaatttatgggaac------aaataaatggagcaaac-----------------**

**Citrus_reticulata_2** **agatcagaatttatgggaac------aaataaatggagcaaac-----------------**

**Citrus_trifoliata_3** **agatcaaaatttatgggaac------aaataaatggagcaaac-----------------**

**Citrus_aurantiifolia** **agatcagaatttatgggaac------aaataaatggagcaaac-----------------**

**Citrus_aurantium** **agatcagaatttatgggaac------aaataaatggagcaaac-----------------**

**Citrus_cavaleriei** **agatcagaatttatgggaac------aaataaatggagcaaac-----------------**

**Citrus_hongheensis** **agatcagaatttatgggaac------aaataaatggagcaaac-----------------**

**Citrus_limon** **agatcagaatttatgggaac------aaataaatggagcaaac-----------------**

**Citrus_maxima** **agatcagaatttatgggaac------aaataaatggagcaaac-----------------**

**Citrus_medica** **agatcagaatttatgggaac------aaataaatggagtaaacaaaataaataaatggag**

**Citrus_polytrifolia** **agatcaaaatttatgggaac------aaataaatggagcaaacaaaataaataaatagcg**

**Citrus_reticulata** **agatcagaatttatgggaac------aaataaatggagcaaac-----------------**

**Citrus_sinensis** **agatcagaatttatgggaac------aaataaatggagcaaac-----------------**

**Fortunella_bawangica_1** **agatcagaatttatgggaacaaataaaaataaatggagcaaac-----------------**

**Fortunella_bawangica_2** **agatcagaatttatgggaacaaataaaaataaatggagcaaac-----------------**

**Fortunella_hindsii_1** **agatcagaatttatgggaac------aaataaatggagcaaac-----------------**

**Fortunella_hindsii_2** **agatcagaatttatgggaac------aaataaatggagcaaac-----------------**

**Fortunella_hindsii_3** **agatcagaatttatgggaac------aaataaatggagcaaac-----------------**

**Fortunella_japonica_1** **agatcagaatttatgggaac------aaataaatggagcaaac-----------------**

**Fortunella_japonica_2** **agatcagaatttatgggaac------aaataaatggagcaaac-----------------**

**Fortunella_japonica_3** **agatcagaatttatgggaac------aaataaatggagcaaac-----------------**

**Fortunella_japonica_4** **agatcagaatttatgggaac------aaataaatggagcaaac-----------------**

**Fortunella_japonica_5** **agatcagaatttatgggaac------aaataaatggagcaaac-----------------**

**Fortunella_japonica_6** **agatcagaatttatgggaac------aaataaatggagcaaac-----------------**

**Fortunella_japonica_7** **agatcagaatttatgggaac------aaataaatggagcaaac-----------------**

**Fortunella_japonica_8** **agatcagaatttatgggaac------aaataaatggagcaaac-----------------**

**Fortunella_japonica_9** **agatcagaatttatgggaac------aaataaatggagcaaac-----------------**

**Fortunella_japonica_10** **agatcagaatttatgggaacaaataaaaataaatggagcaaac-----------------**

**Fortunella_japonica_11** **agatcagaatttatgggaac------aaataaatggagcaaac-----------------**

**Fortunella_japonica_12** **agatcagaatttatgggaac------aaataaatggagcaaac-----------------**

**Fortunella_margarita_1** **agatcagaatttatgggaacaaataaaaataaatggagcaaac-----------------**

**Fortunella_margarita_2** **agatcagaatttatgggaacaaataaaaataaatggagcaaac-----------------**

**Fortunella_venosa_1** **agatcagaatttatgggaac------aaataaatggagcaaac-----------------**

**Fortunella_venosa_2** **agatcagaatttatgggaac------aaataaatggagcaaac-----------------**

**Fortunella_venosa_3** **agatcagaatttatgggaac------aaataaatggagcaaac-----------------**

34810	34820	34830	34840	34850	34860

....|....|....|....|....|....|....|....|....|....|....|....|

**Atalantia_kwangtungens** **-----aaaataaataaatagcggtagggatctatcggagaattggactctttttattctt**

**Citrus_reticulata_1** **-----aaaataaataaatagcggtagggatctatcggagaattggactctttttattctt**

**Citrus_reticulata_2** **-----aaaataaataaatagcggtagggatctatcggagaattggactctttttattctt**

**Citrus_trifoliata_3** **-----aaaataaataaatagcggtagggatctatcggagaattggactctttttattctt**

**Citrus_aurantiifolia** **-----aaaataaataaatagcggtagagatctatcggagaattggactctttttattcct**

**Citrus_aurantium** **-----aaaataaataaatagcggtagggatctatcggagaattggactcttttgattctt**

**Citrus_cavaleriei** **-----aaaataaataaatagcggtagggatctatcggagaattggactctttttattctt**

**Citrus_hongheensis** **-----aaaataaataaatagcggtagggatctatcggagaattggactcttttgattctt**

**Citrus_limon** **-----aaaataaataaatagcggtagggatctatcggagaattggactcttttgattctt**

**Citrus_maxima** **-----aaaataaataaatagcggtagggatctatcggagaattggactcttttgattctt**

**Citrus_medica** **caaacaaaataactaaatagcggtagggatctatcggagaattggactctttttattctt**

**Citrus_polytrifolia** **---gtaaaataaataaatagcggtagggatctatcggagaattggactctttttattctt**

**Citrus_reticulata** **-----aaaataaataaatagcggtagggatctatcggagaattggactctttttattctt**

**Citrus_sinensis** **-----aaaataaataaatagcggtagggatctatcggagaattggactcttttgattctt**

**Fortunella_bawangica_1** **-----aaaataaataaatagcggtagggatctatcggagaattggactctttttattctt**

**Fortunella_bawangica_2** **-----aaaataaataaatagcggtagggatctatcggagaattggactctttttattctt**

**Fortunella_hindsii_1** **-----aaaataaataaatagcggtagggatctatcggagaattggactctttttattctt**

**Fortunella_hindsii_2** **-----aaaataaataaatagcggtagggatctatcggagaattggactctttttattctt**

**Fortunella_hindsii_3** **-----aaaataaataaatagcggtagggatctatcggagaattggactctttttattctt**

**Fortunella_japonica_1** **-----aaaataaataaatagcggtagggatctatcggagaattggactctttttattctt**

**Fortunella_japonica_2** **-----aaaataaataaatagcggtagggatctatcggagaattggactctttttattctt**

**Fortunella_japonica_3** **-----aaaataaataaatagcggtagggatctatcggagaattggactctttttattctt**

**Fortunella_japonica_4** **-----aaaataaataaatagcggtagggatctatcggagaattggactctttttattctt**

**Fortunella_japonica_5** **-----aaaataaataaatagcggtagggatctatcggagaattggactctttttattctt**

**Fortunella_japonica_6** **-----aaaataaataaatagcggtagggatctatcggagaattggactctttttattctt**

**Fortunella_japonica_7** **-----aaaataaataaatagcggtagggatctatcggagaattggactctttttattctt**

**Fortunella_japonica_8** **-----aaaataaataaatagcggtagggatctatcggagaattggactctttttattctt**

**Fortunella_japonica_9** **-----aaaataaataaatagcggtagggatctatcggagaattggactctttttattctt**

**Fortunella_japonica_10** **-----aaaataaataaatagcggtagggatctatcggagaattggactctttttattctt**

**Fortunella_japonica_11** **-----aaaataaataaatagcggtagggatctatcggagaattggactctttttattctt**

**Fortunella_japonica_12** **-----aaaataaataaatagcggtagggatctatcggagaattggactctttttattctt**

**Fortunella_margarita_1** **-----aaaataaataaatagcggtagggatctatcggagaattggactctttttattctt**

**Fortunella_margarita_2** **-----aaaataaataaatagcggtagggatctatcggagaattggactctttttattctt**

**Fortunella_venosa_1** **-----aaaataaataaatagcggtagggatctatcggagaattggactctttttattctt**

**Fortunella_venosa_2** **-----aaaataaataaatagcggtagggatctatcggagaattggactctttttattctt**

**Fortunella_venosa_3** **-----aaaataaataaatagcggtagggatctatcggagaattggactctttttattctt**

34870	34880	34890	34900	34910	34920

....|....|....|....|....|....|....|....|....|....|....|....|

**Atalantia_kwangtungens** **gagtctttcgtatctggcatttctggaaaacaaagtgggttatatcatttcctggtggat**

**Citrus_reticulata_1** **gagtctttcgtatctggcatttctggaaaacaaagggggttatatcatttcctggtggat**

**Citrus_reticulata_2** **gagtctttcgtatctggcatttctggaaaacaaagggggttatatcatttcctggtggat**

**Citrus_trifoliata_3** **gagtctttcgtatctggcatttctggaaaacaaagggggttatatcatttcctggtggat**

**Citrus_aurantiifolia** **gagtctttcgtatctggcatttctggaaaacaaagggggttatatcatttcctggtggat**

**Citrus_aurantium** **gagtctttcgtatctggcatttctggaaaacaaagggggttatatcatttcctggtggat**

**Citrus_cavaleriei** **gagtctttcgtatctggcatttctgtaaaacaaagggggttatatcatttcctggtggat**

**Citrus_hongheensis** **gagtctttcgtatctggcatttctggaaaacaaagggggttatatcatttcctggtggat**

**Citrus_limon** **gagtctttcgtatctggcatttctggaaaacaaagggggttatatcatttcctggtggat**

**Citrus_maxima** **gagtctttcgtatctggcatttctggaaaacaaagggggttatatcatttcctgttggat**

**Citrus_medica** **gagtctttcgtatctggcatttctggaaaacaaagggggttatatcatttcctggtggat**

**Citrus_polytrifolia** **gagtctttcgtatctggcatttctggaaaacaaagggggttatatcatttcctggtggat**

**Citrus_reticulata** **gagtctttcgtatctggcatttctggaaaacaaagggggttatatcatttcctggtggat**

**Citrus_sinensis** **gagtctttcgtatctggcatttctggaaaacaaagggggttatatcatttcctgttggat**

**Fortunella_bawangica_1** **gagtctttcgtatctggcatttctggaaaacaaagggggttatatcatttcctggtggat**

**Fortunella_bawangica_2** **gagtctttcgtatctggcatttctggaaaacaaagggggttatatcatttcctggtggat**

**Fortunella_hindsii_1** **gagtctttcgtatctggcatttctggaaaacaaagggggttatatcatttcctggtggat**

**Fortunella_hindsii_2** **gagtctttcgtatctggcatttctggaaaacaaagggggttatatcatttcctggtggat**

**Fortunella_hindsii_3** **gagtctttcgtatctggcatttctggaaaacaaagggggttatatcatttcctggtggat**

**Fortunella_japonica_1** **gagtctttcgtatctggcatttctggaaaacaaagggggttatatcatttcctggtggat**

**Fortunella_japonica_2** **gagtctttcgtatctggcatttctggaaaacaaagggggttatatcatttcctggtggat**

**Fortunella_japonica_3** **gagtctttcgtatctggcatttctggaaaacaaagggggttatatcatttcctggtggat**

**Fortunella_japonica_4** **gagtctttcgtatctggcatttctggaaaacaaagggggttatatcatttcctggtggat**

**Fortunella_japonica_5** **gagtctttcgtatctggcatttctggaaaacaaagggggttatatcatttcctggtggat**

**Fortunella_japonica_6** **gagtctttcgtatctggcatttctggaaaacaaagggggttatatcatttcctggtggat**

**Fortunella_japonica_7** **gagtctttcgtatctggcatttctggaaaacaaagggggttatatcatttcctggtggat**

**Fortunella_japonica_8** **gagtctttcgtatctggcatttctggaaaacaaagggggttatatcatttcctggtggat**

**Fortunella_japonica_9** **gagtctttcgtatctggcatttctggaaaacaaagggggttatatcatttcctggtggat**

**Fortunella_japonica_10** **gagtctttcgtatctggcatttctggaaaacaaagggggttatatcatttcctggtggat**

**Fortunella_japonica_11** **gagtctttcgtatctggcatttctggaaaacaaagggggttatatcatttcctggtggat**

**Fortunella_japonica_12** **gagtctttcgtatctggcatttctggaaaacaaagggggttatatcatttcctggtggat**

**Fortunella_margarita_1** **gagtctttcgtatctggcatttctggaaaacaaagggggttatatcatttcctggtggat**

**Fortunella_margarita_2** **gagtctttcgtatctggcatttctggaaaacaaagggggttatatcatttcctggtggat**

**Fortunella_venosa_1** **gagtctttcgtatctggcatttctggaaaacaaagggggttatatcatttcctggtggat**

**Fortunella_venosa_2** **gagtctttcgtatctggcatttctggaaaacaaagggggttatatcatttcctggtggat**

**Fortunella_venosa_3** **gagtctttcgtatctggcatttctggaaaacaaagggggttatatcatttcctggtggat**

34930	34940	34950	34960	34970	34980

....|....|....|....|....|....|....|....|....|....|....|....|

**Atalantia_kwangtungens** **ccgcgaattattgggccgagctggatttgaaccagcgtagacatattgccaacgaattta**

**Citrus_reticulata_1** **cagcgaattattgggccgagctggatttgaaccagcgtagacatattgccaacgaattta**

**Citrus_reticulata_2** **cagcgaattattgggccgagctggatttgaaccagcgtagacatattgccaacgaattta**

**Citrus_trifoliata_3** **cagcgaattattgggccgagctggatttgaaccagcgtagacatattgccaacgaattta**

**Citrus_aurantiifolia** **cagcgaattattgggccgagctggatttgaaccagcgtagacatattgccaacgaattta**

**Citrus_aurantium** **cagcgaattattgggccgagctggatttgaaccagcgtagacatattgccaacgaattta**

**Citrus_cavaleriei** **cagcgaattattgggccgagctggatttgaaccagcgtagacatattgccaacgaattta**

**Citrus_hongheensis** **cagcgaattattgggccgagctggatttgaaccagcgtagacatattgccaacgaattta**

**Citrus_limon** **cagcgaattattgggccgagctggatttgaaccagcgtagacatattgccaacgaattta**

**Citrus_maxima** **cagcgaattattgggccgagctggatttgaaccagcgtagacatattgccaacgaattta**

**Citrus_medica** **cagcgaattattgggccgagctggatttgaaccagcgtagacatattgccaacgaattta**

**Citrus_polytrifolia** **cagcgacttattgggccgagctggatttgaaccagcgtagacatattgccaacgaattta**

**Citrus_reticulata** **cagcgaattattgggccgagctggatttgaaccagcgtagacatattgccaacgaattta**

**Citrus_sinensis** **cagcgaattattgggccgagctggatttgaaccagcgtagacatattgccaacgaattta**

**Fortunella_bawangica_1** **cagcgaattattgggccgagctggatttgaaccagcgtagacatattgccaacgaattta**

**Fortunella_bawangica_2** **cagcgaattattgggccgagctggatttgaaccagcgtagacatattgccaacgaattta**

**Fortunella_hindsii_1** **cagcgaattattgggccgagctggatttgaaccagcgtagacatattgccaacgaattta**

**Fortunella_hindsii_2** **cagcgaattattgggccgagctggatttgaaccagcgtagacatattgccaacgaattta**

**Fortunella_hindsii_3** **cagcgaattattgggccgagctggatttgaaccagcgtagacatattgccaacgaattta**

**Fortunella_japonica_1** **cagcgaattattgggccgagctggatttgaaccagcgtagacatattgccaacgaattta**

**Fortunella_japonica_2** **cagcgaattattgggccgagctggatttgaaccagcgtagacatattgccaacgaattta**

**Fortunella_japonica_3** **cagcgaattattgggccgagctggatttgaaccagcgtagacatattgccaacgaattta**

**Fortunella_japonica_4** **cagcgaattattgggccgagctggatttgaaccagcgtagacatattgccaacgaattta**

**Fortunella_japonica_5** **cagcgaattattgggccgagctggatttgaaccagcgtagacatattgccaacgaattta**

**Fortunella_japonica_6** **cagcgaattattgggccgagctggatttgaaccagcgtagacatattgccaacgaattta**

**Fortunella_japonica_7** **cagcgaattattgggccgagctggatttgaaccagcgtagacatattgccaacgaattta**

**Fortunella_japonica_8** **cagcgaattattgggccgagctggatttgaaccagcgtagacatattgccaacgaattta**

**Fortunella_japonica_9** **cagcgaattattgggccgagctggatttgaaccagcgtagacatattgccaacgaattta**

**Fortunella_japonica_10** **cagcgaattattgggccgagctggatttgaaccagcgtagacatattgccaacgaattta**

**Fortunella_japonica_11** **cagcgaattattgggccgagctggatttgaaccagcgtagacatattgccaacgaattta**

**Fortunella_japonica_12** **cagcgaattattgggccgagctggatttgaaccagcgtagacatattgccaacgaattta**

**Fortunella_margarita_1** **cagcgaattattgggccgagctggatttgaaccagcgtagacatattgccaacgaattta**

**Fortunella_margarita_2** **cagcgaattattgggccgagctggatttgaaccagcgtagacatattgccaacgaattta**

**Fortunella_venosa_1** **cagcgaattattgggccgagctggatttgaaccagcgtagacatattgccaacgaattta**

**Fortunella_venosa_2** **cagcgaattattgggccgagctggatttgaaccagcgtagacatattgccaacgaattta**

**Fortunella_venosa_3** **cagcgaattattgggccgagctggatttgaaccagcgtagacatattgccaacgaattta**

34990	35000	35010	35020	35030	35040

....|....|....|....|....|....|....|....|....|....|....|....|

**Atalantia_kwangtungens** **cagtccgtccccattaaccgctcgggcatcgacccaggaagaataaagtctaggcttatt**

**Citrus_reticulata_1** **cagtccgtccccattaaccgctcgggcatcgacccaggaagaataaagtctaggcttatt**

**Citrus_reticulata_2** **cagtccgtccccattaaccgctcgggcatcgacccaggaagaataaagtctaggcttatt**

**Citrus_trifoliata_3** **cagtccgtccccattaaccgctcgggcatcgacccaggaagaataaagtctaggcttatt**

**Citrus_aurantiifolia** **cagtccgtccccattaaccgctcgggcatcgacccaggaagaagaaagtctaggcttatt**

**Citrus_aurantium** **cagtccgtccccattaaccgctcgggcatcgacccaggaagaataaagtctaggcttatt**

**Citrus_cavaleriei** **cagtccgtccccattaaccgctcgggcatcgacccaggaagaataaagtctaggcttatt**

**Citrus_hongheensis** **cagtccgtccccattaaccgctcgggcatcgacccaggaagaataaagtctaggcttatt**

**Citrus_limon** **cagtccgtccccattaaccgctcgggcatcgacccaggaagaataaagtctaggcttatt**

**Citrus_maxima** **cagtccgtccccattaaccgctcgggcatcgacccaggaagaataaagtctaggcttatt**

**Citrus_medica** **cagtccgtccccattaaccgctcgggcatcgacccaggaagaataaagtctaggcttatt**

**Citrus_polytrifolia** **cagtccgtccccattaaccgctcgggcatcgacccaggaagaataaagtctaggcttatt**

**Citrus_reticulata** **cagtccgtccccattaaccgctcgggcatcgacccaggaagaataaagtctaggcttatt**

**Citrus_sinensis** **cagtccgtccccattaaccgctcgggcatcgacccaggaagaataaagtctaggcttatt**

**Fortunella_bawangica_1** **cagtccgtccccattaaccgctcgggcatcgacccaggaagaataaagtctaggcttatt**

**Fortunella_bawangica_2** **cagtccgtccccattaaccgctcgggcatcgacccaggaagaataaagtctaggcttatt**

**Fortunella_hindsii_1** **cagtccgtccccattaaccgctcgggcatcgacccaggaagaataaagtctaggcttatt**

**Fortunella_hindsii_2** **cagtccgtccccattaaccgctcgggcatcgacccaggaagaataaagtctaggcttatt**

**Fortunella_hindsii_3** **cagtccgtccccattaaccgctcgggcatcgacccaggaagaataaagtctaggcttatt**

**Fortunella_japonica_1** **cagtccgtccccattaaccgctcgggcatcgacccaggaagaataaagtctaggcttatt**

**Fortunella_japonica_2** **cagtccgtccccattaaccgctcgggcatcgacccaggaagaataaagtctaggcttatt**

**Fortunella_japonica_3** **cagtccgtccccattaaccgctcgggcatcgacccaggaagaataaagtctaggcttatt**

**Fortunella_japonica_4** **cagtccgtccccattaaccgctcgggcatcgacccaggaagaataaagtctaggcttatt**

**Fortunella_japonica_5** **cagtccgtccccattaaccgctcgggcatcgacccaggaagaataaagtctaggcttatt**

**Fortunella_japonica_6** **cagtccgtccccattaaccgctcgggcatcgacccaggaagaataaagtctaggcttatt**

**Fortunella_japonica_7** **cagtccgtccccattaaccgctcgggcatcgacccaggaagaataaagtctaggcttatt**

**Fortunella_japonica_8** **cagtccgtccccattaaccgctcgggcatcgacccaggaagaataaagtctaggcttatt**

**Fortunella_japonica_9** **cagtccgtccccattaaccgctcgggcatcgacccaggaagaataaagtctaggcttatt**

**Fortunella_japonica_10** **cagtccgtccccattaaccgctcgggcatcgacccaggaagaataaagtctaggcttatt**

**Fortunella_japonica_11** **cagtccgtccccattaaccgctcgggcatcgacccaggaagaataaagtctaggcttatt**

**Fortunella_japonica_12** **cagtccgtccccattaaccgctcgggcatcgacccaggaagaataaagtctaggcttatt**

**Fortunella_margarita_1** **cagtccgtccccattaaccgctcgggcatcgacccaggaagaataaagtctaggcttatt**

**Fortunella_margarita_2** **cagtccgtccccattaaccgctcgggcatcgacccaggaagaataaagtctaggcttatt**

**Fortunella_venosa_1** **cagtccgtccccattaaccgctcgggcatcgacccaggaagaataaagtctaggcttatt**

**Fortunella_venosa_2** **cagtccgtccccattaaccgctcgggcatcgacccaggaagaataaagtctaggcttatt**

**Fortunella_venosa_3** **cagtccgtccccattaaccgctcgggcatcgacccaggaagaataaagtctaggcttatt**

35050	35060	35070	35080	35090	35100

....|....|....|....|....|....|....|....|....|....|....|....|

**Atalantia_kwangtungens** **ta----------taatccacgatcaacttcctttcgtagtaccctacccccaggggaagt**

**Citrus_reticulata_1** **ta----------taatccacgatcaacttcctttcgtagtaccctacccccaggggaagt**

**Citrus_reticulata_2** **ta----------taatccacgatcaacttcctttcgtagtaccctacccccaggggaagt**

**Citrus_trifoliata_3** **ta----------gaatccacgatcaacttcctttcgtagtaccctacccccaggggaagt**

**Citrus_aurantiifolia** **ta----------taatccacgatcaacttcctttcgtagtaccctacccccaggggaagt**

**Citrus_aurantium** **ta----------taatccacgatcaacttcctttcgtagtaccctacccccaggggaagt**

**Citrus_cavaleriei** **tataatccacgataatccacgatcaacttcctttcgtagtaccctacccccaggggaagt**

**Citrus_hongheensis** **ta----------taatccacgatcaacttcctttcgtagtaccctacccccaggggaagt**

**Citrus_limon** **ta----------taatccacgatcaacttcctttcgtagtaccctacccccaggggaagt**

**Citrus_maxima** **ta----------taatccacgatcaacttcctttcgtagtaccctacccccaggggaagt**

**Citrus_medica** **ta----------taatccacgatcaacttactttcgtagtaccctacccccaggggaagt**

**Citrus_polytrifolia** **ta----------gaatccacgatcaacttcctttcgtagtaccctacccccaggggaagt**

**Citrus_reticulata** **ta----------taatccacgatcaacttcctttcgtagtaccctacccccaggggaagt**

**Citrus_sinensis** **ta----------taatccacgatcaacttcctttcgtagtaccctacccccaggggaagt**

**Fortunella_bawangica_1** **ta----------taatccacgatcaacttcctttcgtagtaccctacccccaggggaagt**

**Fortunella_bawangica_2** **ta----------taatccacgatcaacttcctttcgtagtaccctacccccaggggaagt**

**Fortunella_hindsii_1** **ta----------taatccacgatcaacttcctttcgtagtaccctacccccaggggaagt**

**Fortunella_hindsii_2** **ta----------taatccacgatcaacttcctttcgtagtaccctacccccaggggaagt**

**Fortunella_hindsii_3** **ta----------taatccacgatcaacttcctttcgtagtaccctacccccaggggaagt**

**Fortunella_japonica_1** **ta----------taatccacgatcaacttcctttcgtagtaccctacccccaggggaagt**

**Fortunella_japonica_2** **ta----------taatccacgatcaacttcctttcgtagtaccctacccccaggggaagt**

**Fortunella_japonica_3** **ta----------taatccacgatcaacttcctttcgtagtaccctacccccaggggaagt**

**Fortunella_japonica_4** **ta----------taatccacgatcaacttcctttcgtagtaccctacccccaggggaagt**

**Fortunella_japonica_5** **ta----------taatccacgatcaacttcctttcgtagtaccctacccccaggggaagt**

**Fortunella_japonica_6** **ta----------taatccacgatcaacttcctttcgtagtaccctacccccaggggaagt**

**Fortunella_japonica_7** **ta----------taatccacgatcaacttcctttcgtagtaccctacccccaggggaagt**

**Fortunella_japonica_8** **ta----------taatccacgatcaacttcctttcgtagtaccctacccccaggggaagt**

**Fortunella_japonica_9** **ta----------taatccacgatcaacttcctttcgtagtaccctacccccaggggaagt**

**Fortunella_japonica_10** **ta----------taatccacgatcaacttcctttcgtagtaccctacccccaggggaagt**

**Fortunella_japonica_11** **ta----------taatccacgatcaacttcctttcgtagtaccctacccccaggggaagt**

**Fortunella_japonica_12** **ta----------taatccacgatcaacttcctttcgtagtaccctacccccaggggaagt**

**Fortunella_margarita_1** **ta----------taatccacgatcaacttcctttcgtagtaccctacccccaggggaagt**

**Fortunella_margarita_2** **ta----------taatccacgatcaacttcctttcgtagtaccctacccccaggggaagt**

**Fortunella_venosa_1** **ta----------taatccacgatcaacttcctttcgtagtaccctacccccaggggaagt**

**Fortunella_venosa_2** **ta----------taatccacgatcaacttcctttcgtagtaccctacccccaggggaagt**

**Fortunella_venosa_3** **ta----------taatccacgatcaacttcctttcgtagtaccctacccccaggggaagt**

35110	35120	35130	35140	35150	35160

....|....|....|....|....|....|....|....|....|....|....|....|

**Atalantia_kwangtungens** **cgaatccccgctgcctccttgaaagagagatgtcctgaaccgctagacgatgggggcata**

**Citrus_reticulata_1** **cgaatccccgctgcctccttgaaagagagatgtcctgaaccgctagacgatgggggcata**

**Citrus_reticulata_2** **cgaatccccgctgcctccttgaaagagagatgtcctgaaccgctagacgatgggggcata**

**Citrus_trifoliata_3** **cgaatccccgctgcctccttgaaagagagatgtcctgaaccgctagacgatgggggcata**

**Citrus_aurantiifolia** **cgaatccccgctgcctccttgaaagagagatgtcctgaaccgctagacgatgggggcata**

**Citrus_aurantium** **cgaatccccgctgcctccttgaaagagagatgtcctgaaccgctagacgatgggggcata**

**Citrus_cavaleriei** **cgaatccccgctgcctccttgaaagagagatgtcctgaaccgctagacgatgggggcata**

**Citrus_hongheensis** **cgaatccccgctgcctccttgaaagagagatgtcctgaaccgctagacgatgggggcata**

**Citrus_limon** **cgaatccccgctgcctccttgaaagagagatgtcctgaaccgctagacgatgggggcata**

**Citrus_maxima** **cgaatccccgctgcctccttgaaagagagatgtcctgaaccgctagacgatgggggcata**

**Citrus_medica** **cgaatccccgctgcctccttgaaagagagatgtcctgaaccgctagacgatgggggcata**

**Citrus_polytrifolia** **cgaatccccgctgcctccttgaaagagagatgtcctgaaccgctagacgatgggggcata**

**Citrus_reticulata** **cgaatccccgctgcctccttgaaagagagatgtcctgaaccgctagacgatgggggcata**

**Citrus_sinensis** **cgaatccccgctgcctccttgaaagagagatgtcctgaaccgctagacgatgggggcata**

**Fortunella_bawangica_1** **cgaatccccgctgcctccttgaaagagagatgtcctgaaccgctagacgatgggggcata**

**Fortunella_bawangica_2** **cgaatccccgctgcctccttgaaagagagatgtcctgaaccgctagacgatgggggcata**

**Fortunella_hindsii_1** **cgaatccccgctgcctccttgaaagagagatgtcctgaaccgctagacgatgggggcata**

**Fortunella_hindsii_2** **cgaatccccgctgcctccttgaaagagagatgtcctgaaccgctagacgatgggggcata**

**Fortunella_hindsii_3** **cgaatccccgctgcctccttgaaagagagatgtcctgaaccgctagacgatgggggcata**

**Fortunella_japonica_1** **cgaatccccgctgcctccttgaaagagagatgtcctgaaccgctagacgatgggggcata**

**Fortunella_japonica_2** **cgaatccccgctgcctccttgaaagagagatgtcctgaaccgctagacgatgggggcata**

**Fortunella_japonica_3** **cgaatccccgctgcctccttgaaagagagatgtcctgaaccgctagacgatgggggcata**

**Fortunella_japonica_4** **cgaatccccgctgcctccttgaaagagagatgtcctgaaccgctagacgatgggggcata**

**Fortunella_japonica_5** **cgaatccccgctgcctccttgaaagagagatgtcctgaaccgctagacgatgggggcata**

**Fortunella_japonica_6** **cgaatccccgctgcctccttgaaagagagatgtcctgaaccgctagacgatgggggcata**

**Fortunella_japonica_7** **cgaatccccgctgcctccttgaaagagagatgtcctgaaccgctagacgatgggggcata**

**Fortunella_japonica_8** **cgaatccccgctgcctccttgaaagagagatgtcctgaaccgctagacgatgggggcata**

**Fortunella_japonica_9** **cgaatccccgctgcctccttgaaagagagatgtcctgaaccgctagacgatgggggcata**

**Fortunella_japonica_10** **cgaatccccgctgcctccttgaaagagagatgtcctgaaccgctagacgatgggggcata**

**Fortunella_japonica_11** **cgaatccccgctgcctccttgaaagagagatgtcctgaaccgctagacgatgggggcata**

**Fortunella_japonica_12** **cgaatccccgctgcctccttgaaagagagatgtcctgaaccgctagacgatgggggcata**

**Fortunella_margarita_1** **cgaatccccgctgcctccttgaaagagagatgtcctgaaccgctagacgatgggggcata**

**Fortunella_margarita_2** **cgaatccccgctgcctccttgaaagagagatgtcctgaaccgctagacgatgggggcata**

**Fortunella_venosa_1** **cgaatccccgctgcctccttgaaagagagatgtcctgaaccgctagacgatgggggcata**

**Fortunella_venosa_2** **cgaatccccgctgcctccttgaaagagagatgtcctgaaccgctagacgatgggggcata**

**Fortunella_venosa_3** **cgaatccccgctgcctccttgaaagagagatgtcctgaaccgctagacgatgggggcata**

35170	35180	35190	35200	35210	35220

....|....|....|....|....|....|....|....|....|....|....|....|

**Atalantia_kwangtungens** **cttgcccgactgccatcatacttagtatgaacagttttttgaaattgtcaatataatgga**

**Citrus_reticulata_1** **tttgcccgactgccatcatacttagtatgaacagttttttgaaattgtcaatataatgga**

**Citrus_reticulata_2** **tttgcccgactgccatcatacttagtatgaacagttttttgaaattgtcaatataatgga**

**Citrus_trifoliata_3** **tttgcccgactgccatcatacttagtatgaacagttttttgaaattgtcaatataatgga**

**Citrus_aurantiifolia** **tttgcccgactgccatcatacttagtatgaacagttttttgaaattgtcaatataatgga**

**Citrus_aurantium** **tttgcccgactgccatcatacttagtatgaacagttttttgaaattgtcaatataatgga**

**Citrus_cavaleriei** **tttgcccgactgccatcatacttagtatgaacagttttttgaaattgtcaatataatgga**

**Citrus_hongheensis** **tttgcccgactgccatcatacttagtatgaacagttttttgaaattgtcaatataatgga**

**Citrus_limon** **tttgcccgactgccatcatacttagtatgaacagttttttgaaattgtcaatataatgga**

**Citrus_maxima** **tttgcccgactgccatcatacttagtatgaacagttttttgaaattgtcaatataatgga**

**Citrus_medica** **tttgcccgactgccatcatacttagtatgaacagttttttgaaattgtcaatataatgga**

**Citrus_polytrifolia** **tttgcccgactgccatcatacttagtatgaacagttttttgaaattgtcaatataattga**

**Citrus_reticulata** **tttgcccgactgccatcatacttagtatgaacagttttttgaaattgtcaatataatgga**

**Citrus_sinensis** **tttgcccgactgccatcatacttagtatgaacagttttttgaaattgtcaatataatgga**

**Fortunella_bawangica_1** **tttgcccgactgccatcatacttagtatgaacagttttttgaaattgtcaatataatgga**

**Fortunella_bawangica_2** **tttgcccgactgccatcatacttagtatgaacagttttttgaaattgtcaatataatgga**

**Fortunella_hindsii_1** **tttgcccgactgccatcatacttagtatgaacagttttttgaaattgtcaatataatgga**

**Fortunella_hindsii_2** **tttgcccgactgccatcatacttagtatgaacagttttttgaaattgtcaatataatgga**

**Fortunella_hindsii_3** **tttgcccgactgccatcatacttagtatgaacagttttttgaaattgtcaatataatgga**

**Fortunella_japonica_1** **tttgcccgactgccatcatacttagtatgaacagttttttgaaattgtcaatataatgga**

**Fortunella_japonica_2** **tttgcccgactgccatcatacttagtatgaacagttttttgaaattgtcaatataatgga**

**Fortunella_japonica_3** **tttgcccgactgccatcatacttagtatgaacagttttttgaaattgtcaatataatgga**

**Fortunella_japonica_4** **tttgcccgactgccatcatacttagtatgaacagttttttgaaattgtcaatataatgga**

**Fortunella_japonica_5** **tttgcccgactgccatcatacttagtatgaacagttttttgaaattgtcaatataatgga**

**Fortunella_japonica_6** **tttgcccgactgccatcatacttagtatgaacagttttttgaaattgtcaatataatgga**

**Fortunella_japonica_7** **tttgcccgactgccatcatacttagtatgaacagttttttgaaattgtcaatataatgga**

**Fortunella_japonica_8** **tttgcccgactgccatcatacttagtatgaacagttttttgaaattgtcaatataatgga**

**Fortunella_japonica_9** **tttgcccgactgccatcatacttagtatgaacagttttttgaaattgtcaatataatgga**

**Fortunella_japonica_10** **tttgcccgactgccatcatacttagtatgaacagttttttgaaattgtcaatataatgga**

**Fortunella_japonica_11** **tttgcccgactgccatcatacttagtatgaacagttttttgaaattgtcaatataatgga**

**Fortunella_japonica_12** **tttgcccgactgccatcatacttagtatgaacagttttttgaaattgtcaatataatgga**

**Fortunella_margarita_1** **tttgcccgactgccatcatacttagtatgaacagttttttgaaattgtcaatataatgga**

**Fortunella_margarita_2** **tttgcccgactgccatcatacttagtatgaacagttttttgaaattgtcaatataatgga**

**Fortunella_venosa_1** **tttgcccgactgccatcatacttagtatgaacagttttttgaaattgtcaatataatgga**

**Fortunella_venosa_2** **tttgcccgactgccatcatacttagtatgaacagttttttgaaattgtcaatataatgga**

**Fortunella_venosa_3** **tttgcccgactgccatcatacttagtatgaacagttttttgaaattgtcaatataatgga**

35230	35240	35250	35260	35270	35280

....|....|....|....|....|....|....|....|....|....|....|....|

**Atalantia_kwangtungens** **atgggatgactaactc-----taaaggatctttccaccttttctgatcccataccaatag**

**Citrus_reticulata_1** **atgggatgactaactctaaagtaaaggatctttccaccttttctgatcccataccaatag**

**Citrus_reticulata_2** **atgggatgactaactctaaagtaaaggatctttccaccttttctgatcccataccaatag**

**Citrus_trifoliata_3** **atgggatgactaactctaaagtaaaggatctttccaccttttcggatcccataccaatag**

**Citrus_aurantiifolia** **atgggatgactaactctaaagtaaaggatctttccaccttttctgatcccataccaatag**

**Citrus_aurantium** **atgggatgactaactctaaagtaaaggatctttccaccttttctgatcccataccaatag**

**Citrus_cavaleriei** **atgggatgactaactctaaagtaaaggatctttccaccttttctgatcccataccaatag**

**Citrus_hongheensis** **atgggatgactaactctaaagtaaaggatctttccaccttttctgatcccataccaatag**

**Citrus_limon** **atgggatgactaactctaaagtaaaggatctttccaccttttctgatcccataccaatag**

**Citrus_maxima** **atgggatgactaactctaaagtaaaggatctttccaccttttctgatcccataccaatag**

**Citrus_medica** **atgggatgactaactctaaagtaaaggatctttccaccttttctgatcccataccaatag**

**Citrus_polytrifolia** **atgggatgactaactctaaagtaaaggatctttccaccttttcggatcccataccaatag**

**Citrus_reticulata** **atgggatgactaactctaaagtaaaggatctttccaccttttctgatcccataccaatag**

**Citrus_sinensis** **atgggatgactaactctaaagtaaaggatctttccaccttttctgatcccataccaatag**

**Fortunella_bawangica_1** **atgggatgactaactctaaagtaaaggatctttccaccttttctgatcccataccaatag**

**Fortunella_bawangica_2** **atgggatgactaactctaaagtaaaggatctttccaccttttctgatcccataccaatag**

**Fortunella_hindsii_1** **atgggatgactaactctaaagtaaaggatctttccaccttttctgatcccataccaatag**

**Fortunella_hindsii_2** **atgggatgactaactctaaagtaaaggatctttccaccttttctgatcccataccaatag**

**Fortunella_hindsii_3** **atgggatgactaactctaaagtaaaggatctttccaccttttctgatcccataccaatag**

**Fortunella_japonica_1** **atgggatgactaactctaaagtaaaggatctttccaccttttctgatcccataccaatag**

**Fortunella_japonica_2** **atgggatgactaactctaaagtaaaggatctttccaccttttctgatcccataccaatag**

**Fortunella_japonica_3** **atgggatgactaactctaaagtaaaggatctttccaccttttctgatcccataccaatag**

**Fortunella_japonica_4** **atgggatgactaactctaaagtaaaggatctttccaccttttctgatcccataccaatag**

**Fortunella_japonica_5** **atgggatgactaactctaaagtaaaggatctttccaccttttctgatcccataccaatag**

**Fortunella_japonica_6** **atgggatgactaactctaaagtaaaggatctttccaccttttctgatcccataccaatag**

**Fortunella_japonica_7** **atgggatgactaactctaaagtaaaggatctttccaccttttctgatcccataccaatag**

**Fortunella_japonica_8** **atgggatgactaactctaaagtaaaggatctttccaccttttctgatcccataccaatag**

**Fortunella_japonica_9** **atgggatgactaactctaaagtaaaggatctttccaccttttctgatcccataccaatag**

**Fortunella_japonica_10** **atgggatgactaactctaaagtaaaggatctttccaccttttctgatcccataccaatag**

**Fortunella_japonica_11** **atgggatgactaactctaaagtaaaggatctttccaccttttctgatcccataccaatag**

**Fortunella_japonica_12** **atgggatgactaactctaaagtaaaggatctttccaccttttctgatcccataccaatag**

**Fortunella_margarita_1** **atgggatgactaactctaaagtaaaggatctttccaccttttctgatcccataccaatag**

**Fortunella_margarita_2** **atgggatgactaactctaaagtaaaggatctttccaccttttctgatcccataccaatag**

**Fortunella_venosa_1** **atgggatgactaactctaaagtaaaggatctgtccaccttttctgatcccataccaatag**

**Fortunella_venosa_2** **atgggatgactaactctaaagtaaaggatctgtccaccttttctgatcccataccaatag**

**Fortunella_venosa_3** **atgggatgactaactctaaagtaaaggatctttccaccttttctgatcccataccaatag**

35290	35300	35310	35320	35330	35340

....|....|....|....|....|....|....|....|....|....|....|....|

**Atalantia_kwangtungens** **cattttttgattcgtccatcaatcgctcattcgaatcgccattctattctaaaatcgaa-**

**Citrus_reticulata_1** **cattttttgattcgtccatcaatcgctcattctaatcgccattctagtctaaaatcgaa-**

**Citrus_reticulata_2** **cattttttgattcgtccatcaatcgctcattctaatcgccattctagtctaaaatcgaa-**

**Citrus_trifoliata_3** **cattttttgattcgtccatcaatcgctcattctaatccccattctagtctaaaatcgaa-**

**Citrus_aurantiifolia** **ctttttttgattcgtctatcaatcgctcattctaatcgccattctagtctaaaatcgaaa**

**Citrus_aurantium** **ctttttttgattcgtccatcaatcgctcattctaatcgccattctagtctaaaatcgaa-**

**Citrus_cavaleriei** **cattttttgattcgtccatcaatcgctcattctaatcgccattctagtctaaaatcgaa-**

**Citrus_hongheensis** **ctttttttgattcgtccatcaatcgctcattctaatcgccattctagtctaaaatcgaa-**

**Citrus_limon** **ctttttttgattcgtccatcaatcgctcattctaatcgccattctagtctaaaatcgaa-**

**Citrus_maxima** **ctttttttgattcgtccatcaatcgctcattctaatcgccattctagtctaaaatcgaa-**

**Citrus_medica** **cattttttgattcgtccatcaatcgctcattctaatcgccattctattctaaaatcgaa-**

**Citrus_polytrifolia** **cattttttgattcgtccatcaatcgctcattctaatccccattctagtctaaaatagaa-**

**Citrus_reticulata** **cattttttgattcgtccatcaatcgctcattctaatcgccattctagtctaaaatcgaa-**

**Citrus_sinensis** **ctttttttgattcgtccatcaatcgctcattctaatcgccattctagtctaaaatcgaa-**

**Fortunella_bawangica_1** **cattttttgattcgtccatcaatcgctcattctaatcgccattctagtctaaaatcgaa-**

**Fortunella_bawangica_2** **cattttttgattcgtccatcaatcgctcattctaatcgccattctagtctaaaatcgaa-**

**Fortunella_hindsii_1** **cattttttgattcgtccatcaatcgctcattctaatcgccattctagtctaaaatcgaa-**

**Fortunella_hindsii_2** **cattttttgattcgtccatcaatcgctcattctaatcgccattctagtctaaaatcgaa-**

**Fortunella_hindsii_3** **cattttttgattcgtccatcaatcgctcattctaatcgccattctagtctaaaatcgaa-**

**Fortunella_japonica_1** **cattttttgattcgtccatcaatcgctcattctaatcgccattctagtctaaaatcgaa-**

**Fortunella_japonica_2** **cattttttgattcgtccatcaatcgctcattctaatcgccattctagtctaaaatcgaa-**

**Fortunella_japonica_3** **cattttttgattcgtccatcaatcgctcattctaatcgccattctagtctaaaatcgaa-**

**Fortunella_japonica_4** **cattttttgattcgtccatcaatcgctcattctaatcgccattctagtctaaaatcgaa-**

**Fortunella_japonica_5** **cattttttgattcgtccatcaatcgctcattctaatcgccattctagtctaaaatcgaa-**

**Fortunella_japonica_6** **cattttttgattcgtccatcaatcgctcattctaatcgccattctagtctaaaatcgaa-**

**Fortunella_japonica_7** **cattttttgattcgtccatcaatcgctcattctaatcgccattctagtctaaaatcgaa-**

**Fortunella_japonica_8** **cattttttgattcgtccatcaatcgctcattctaatcgccattctagtctaaaatcgaa-**

**Fortunella_japonica_9** **cattttttgattcgtccatcaatcgctcattctaatcgccattctagtctaaaatcgaa-**

**Fortunella_japonica_10** **cattttttgattcgtccatcaatcgctcattctaatcgccattctagtctaaaatcgaa-**

**Fortunella_japonica_11** **cattttttgattcgtccatcaatcgctcattctaatcgccattctagtctaaaatcgaa-**

**Fortunella_japonica_12** **cattttttgattcgtccatcaatcgctcattctaatcgccattctagtctaaaatcgaa-**

**Fortunella_margarita_1** **cattttttgattcgtccatcaatcgctcattctaatcgccattctagtctaaaatcgaa-**

**Fortunella_margarita_2** **cattttttgattcgtccatcaatcgctcattctaatcgccattctagtctaaaatcgaa-**

**Fortunella_venosa_1** **cattttttgattcgtccatcaatcgctcattctaatcgccattctagtctaaaatcgaa-**

**Fortunella_venosa_2** **cattttttgattcgtccatcaatcgctcattctaatcgccattctagtctaaaatcgaa-**

**Fortunella_venosa_3** **cattttttgattcgtccatcaatcgctcattctaatcgccattctagtctaaaatcgaa-**

35350	35360	35370	35380	35390	35400

....|....|....|....|....|....|....|....|....|....|....|....|

**Atalantia_kwangtungens** **------atctattatagaaattgctataaaa-----------aaaaataataaaaatagg**

**Citrus_reticulata_1** **------atctattatagaaattgctataa--------ataataataaaaataaaaatagg**

**Citrus_reticulata_2** **------atctattatagaaattgctataa--------ataataataaaaataaaaatagg**

**Citrus_trifoliata_3** **------atctattatagaaattgctataa-------------aataaaaataaaaatagg**

**Citrus_aurantiifolia** **tctattatctattatagaaattgctataa--------ataataataaaaataaaaatagg**

**Citrus_aurantium** **------atctattatagaaattgctataa--------ataataataaaaataaaaatagg**

**Citrus_cavaleriei** **------atctattatagaaattgctagaa--------ataataataaaaataaaaatagg**

**Citrus_hongheensis** **------atctattatagaaattgctata--------------aataaaaataaaaatagg**

**Citrus_limon** **------atctattatagaaattgctataa--------ataataataaaaataaaaatagg**

**Citrus_maxima** **------atctattatagaaattgctataa--------ataataataaaaataaaaatagg**

**Citrus_medica** **------atctattatagaaattgctataa-------------------aataaaaatagg**

**Citrus_polytrifolia** **------atctattatagaaattgctataaaaaaaaa-ataataataaaaataaaaatagg**

**Citrus_reticulata** **------atctattatagaaattgctataa--------ataataataaaaataaaaatagg**

**Citrus_sinensis** **------atctattatagaaattgctataa--------ataataataaaaataaaaatagg**

**Fortunella_bawangica_1** **------atctattatagaaattgctataaaaaaaaa-ataataataaaaagaaaaatagg**

**Fortunella_bawangica_2** **------atctattatagaaattgctataaaaaaaaa-ataataataaaaagaaaaatagg**

**Fortunella_hindsii_1** **------atctattatagaaattgctataaaaaaaaa-ataataataaaaataaaaatagg**

**Fortunella_hindsii_2** **------atctattatagaaattgctataaaaaaaaa-ataataataaaaataaaaatagg**

**Fortunella_hindsii_3** **------atctattatagaaattgctataaaaaaaaa-ataataataaaaataaaaatagg**

**Fortunella_japonica_1** **------atctattatagaaattgctataaaaaaaaaaataataataaaaataaaaatagg**

**Fortunella_japonica_2** **------atctattatagaaattgctataaaaaaaaaaataataataaaaataaaaatagg**

**Fortunella_japonica_3** **------atctattatagaaattgctataaaaaaaaaaataataataaaaataaaaatagg**

**Fortunella_japonica_4** **------atctattatagaaattgctataaaaaaaaaaataataataaaaataaaaatagg**

**Fortunella_japonica_5** **------atctattatagaaattgctataaaaaaaaaaataataataaaaataaaaatagg**

**Fortunella_japonica_6** **------atctattatagaaattgctataaaaaaaaaaataataataaaaataaaaatagg**

**Fortunella_japonica_7** **------atctattatagaaattgctataaaaaaaaaaataataataaaaataaaaatagg**

**Fortunella_japonica_8** **------atctattatagaaattgctataaaaaaaaaaataataataaaaataaaaatagg**

**Fortunella_japonica_9** **------atctattatagaaattgctataaaaaaaaaaataataataaaaataaaaatagg**

**Fortunella_japonica_10** **------atctattatagaaattgctataaaaaaaaa-ataataataaaaagaaaaatagg**

**Fortunella_japonica_11** **------atctattatagaaattgctataaaaaaaaaaataataataaaaataaaaatagg**

**Fortunella_japonica_12** **------atctattatagaaattgctataaaaaaaaaaataataataaaaataaaaatagg**

**Fortunella_margarita_1** **------atctattatagaaattgctataaaaaaaaa-ataataataaaaagaaaaatagg**

**Fortunella_margarita_2** **------atctattatagaaattgctataaaaaaaaa-ataataataaaaagaaaaatagg**

**Fortunella_venosa_1** **------atctattatagaaattgctataaaaaaaa-------aataaaaataaaaatagg**

**Fortunella_venosa_2** **------atctattatagaaattgctataaaaaaaaa-ataataataaaaataaaaatagg**

**Fortunella_venosa_3** **------atctattatagaaattgctataaaaaaaaa-ataataataaaaataaaaatagg**

35410	35420	35430	35440	35450	35460

....|....|....|....|....|....|....|....|....|....|....|....|

**Atalantia_kwangtungens** **acgaaagacgaaagtagaggactcttttgggaagtgattggtccgacataaaagaagatt**

**Citrus_reticulata_1** **c---------aaagtagaggactcttttgggaagtgattgatccgacataaaagaagatt**

**Citrus_reticulata_2** **c---------aaagtagaggactcttttgggaagtgattgatccgacataaaagaagatt**

**Citrus_trifoliata_3** **ccgaaagacgaaagtagaggactcttttgggaagtgattggtccgacataaaagaagatt**

**Citrus_aurantiifolia** **cc-------gaaagtagaggactcttttgggaagtgattggtccgacataaaagaagatt**

**Citrus_aurantium** **ccgaaagacgaaagtagaggactcttttgggaagtgattggtccgacataaaagaagatt**

**Citrus_cavaleriei** **c---------aaagtagaggactcttttgggaagtgattggtccgacataaaagaagatt**

**Citrus_hongheensis** **ccgaaagacgaaagtagaggactcttttgggaagtgattggtccgacataaaagaagatt**

**Citrus_limon** **ccgaaagacgaaagtagaggactcttttgggaagtgattggtccgacataaaagaagatt**

**Citrus_maxima** **ccgaaagacgaaagtagaggactcttttgggaagtgattggtccgacataaaagaagatt**

**Citrus_medica** **ccgaaagacgaaagtagaggactctttggggaagtgattggtccgacataaaagaagatt**

**Citrus_polytrifolia** **ccgaaagacgaaagtagaggactcttttgggaagtgattggtccgacataaaagaagatt**

**Citrus_reticulata** **c---------aaagtagaggactcttttgggaagtgattgatccgacataaaagaagatt**

**Citrus_sinensis** **ccgaaagacgaaagtagaggactcttttgggaagtgattggtccgacataaaagaagatt**

**Fortunella_bawangica_1** **tcgaaagacgaaagtagaggactcttttgggaagtgattggtccgacataaaagaagatt**

**Fortunella_bawangica_2** **tcgaaagacgaaagtagaggactcttttgggaagtgattggtccgacataaaagaagatt**

**Fortunella_hindsii_1** **tcgaaagacgaaagtagaggactcttttgggaagtgattggtccgacataaaagaagatt**

**Fortunella_hindsii_2** **tcgaaagacgaaagtagaggactcttttgggaagtgattggtccgacataaaagaagatt**

**Fortunella_hindsii_3** **tcgaaagacgaaagtagaggactcttttgggaagtgattggtccgacataaaagaagatt**

**Fortunella_japonica_1** **tcgaaagacgaaagtagaggactcttttgggaagtgattggtccgacataaaagaagatt**

**Fortunella_japonica_2** **tcgaaagacgaaagtagaggactcttttgggaagtgattggtccgacataaaagaagatt**

**Fortunella_japonica_3** **tcgaaagacgaaagtagaggactcttttgggaagtgattggtccgacataaaagaagatt**

**Fortunella_japonica_4** **tcgaaagacgaaagtagaggactcttttgggaagtgattggtccgacataaaagaagatt**

**Fortunella_japonica_5** **tcgaaagacgaaagtagaggactcttttgggaagtgattggtccgacataaaagaagatt**

**Fortunella_japonica_6** **tcgaaagacgaaagtagaggactcttttgggaagtgattggtccgacataaaagaagatt**

**Fortunella_japonica_7** **tcgaaagacgaaagtagaggactcttttgggaagtgattggtccgacataaaagaagatt**

**Fortunella_japonica_8** **tcgaaagacgaaagtagaggactcttttgggaagtgattggtccgacataaaagaagatt**

**Fortunella_japonica_9** **tcgaaagacgaaagtagaggactcttttgggaagtgattggtccgacataaaagaagatt**

**Fortunella_japonica_10** **tcgaaagacgaaagtagaggactcttttgggaagtgattggtccgacataaaagaagatt**

**Fortunella_japonica_11** **tcgaaagacgaaagtagaggactcttttgggaagtgattggtccgacataaaagaagatt**

**Fortunella_japonica_12** **tcgaaagacgaaagtagaggactcttttgggaagtgattggtccgacataaaagaagatt**

**Fortunella_margarita_1** **tcgaaagacgaaagtagaggactcttttgggaagtgattggtccgacataaaagaagatt**

**Fortunella_margarita_2** **tcgaaagacgaaagtagaggactcttttgggaagtgattggtccgacataaaagaagatt**

**Fortunella_venosa_1** **tcgaaagacgaaagtagaggactcttttgggaagtgattggtccgacataaaagaagatt**

**Fortunella_venosa_2** **tcgaaagacgaaagtagaggactcttttgggaagtgattggtccgacataaaagaagatt**

**Fortunella_venosa_3** **tcgaaagacgaaagtagaggactcttttgggaagtgattggtccgacataaaagaagatt**

35470	35480	35490	35500	35510	35520

....|....|....|....|....|....|....|....|....|....|....|....|

**Atalantia_kwangtungens** **ttttcttcatttcttccacttaactttcattcatttatccactccttggtaagatgagat**

**Citrus_reticulata_1** **ttttcttcatttcttccacttaactttcattcatttatccactccttggtaagatgagat**

**Citrus_reticulata_2** **ttttcttcatttcttccacttaactttcattcatttatccactccttggtaagatgagat**

**Citrus_trifoliata_3** **ttttcttcatttcttccacttaactttcatttatttatccactccttggtaagatgagat**

**Citrus_aurantiifolia** **ttttcttcatttcttccacttaactttcattcatttatccactccttggtaagatgagat**

**Citrus_aurantium** **ttttcttcatttcttccacttaactttcattcatttatccactccttggtaagatgagat**

**Citrus_cavaleriei** **ttttcttcatttcttccacttaactttcattcatttatccactccttggtaagatgagat**

**Citrus_hongheensis** **ttttcttcatttcttccacttaactttcattcatttatccactccttggtaagatgagat**

**Citrus_limon** **ttttcttcatttcttccacttaactttcattcatttatccactccttggtaagatgagat**

**Citrus_maxima** **ttttcttcatttcttccacttaactttcattcatttatccactccttggtaagatgagat**

**Citrus_medica** **ttttcttcatttcttccacttaactttcattcatttatccactccttggtaagatgagat**

**Citrus_polytrifolia** **ttttcttcatttcttccacttaactttcatttatttatccactccttggtaagatgagat**

**Citrus_reticulata** **ttttcttcatttcttccacttaactttcattcatttatccactccttggtaagatgagat**

**Citrus_sinensis** **ttttcttcatttcttccacttaactttcattcatttatccactccttggtaagatgagat**

**Fortunella_bawangica_1** **ttttcttcatttcttccacttaactttcattcatttatccactccttggtaagatgagat**

**Fortunella_bawangica_2** **ttttcttcatttcttccacttaactttcattcatttatccactccttggtaagatgagat**

**Fortunella_hindsii_1** **ttttcttcatttcttccacttaactttcattcatttatccactccttggtaagatgagat**

**Fortunella_hindsii_2** **ttttcttcatttcttccacttaactttcattcatttatccactccttggtaagatgagat**

**Fortunella_hindsii_3** **ttttcttcatttcttccacttaactttcattcatttatccactccttggtaagatgagat**

**Fortunella_japonica_1** **ttttcttcatttcttccacttaactttcattcatttatccactccttggtaagatgagat**

**Fortunella_japonica_2** **ttttcttcatttcttccacttaactttcattcatttatccactccttggtaagatgagat**

**Fortunella_japonica_3** **ttttcttcatttcttccacttaactttcattcatttatccactccttggtaagatgagat**

**Fortunella_japonica_4** **ttttcttcatttcttccacttaactttcattcatttatccactccttggtaagatgagat**

**Fortunella_japonica_5** **ttttcttcatttcttccacttaactttcattcatttatccactccttggtaagatgagat**

**Fortunella_japonica_6** **ttttcttcatttcttccacttaactttcattcatttatccactccttggtaagatgagat**

**Fortunella_japonica_7** **ttttcttcatttcttccacttaactttcattcatttatccactccttggtaagatgagat**

**Fortunella_japonica_8** **ttttcttcatttcttccacttaactttcattcatttatccactccttggtaagatgagat**

**Fortunella_japonica_9** **ttttcttcatttcttccacttaactttcattcatttatccactccttggtaagatgagat**

**Fortunella_japonica_10** **ttttcttcatttcttccacttaactttcattcatttatccactccttggtaagatgagat**

**Fortunella_japonica_11** **ttttcttcatttcttccacttaactttcattcatttatccactccttggtaagatgagat**

**Fortunella_japonica_12** **ttttcttcatttcttccacttaactttcattcatttatccactccttggtaagatgagat**

**Fortunella_margarita_1** **ttttcttcatttcttccacttaactttcattcatttatccactccttggtaagatgagat**

**Fortunella_margarita_2** **ttttcttcatttcttccacttaactttcattcatttatccactccttggtaagatgagat**

**Fortunella_venosa_1** **ttttcttcatttcttccacttaactttcattcatttatccactccttggtaagatgagat**

**Fortunella_venosa_2** **ttttcttcatttcttccacttaactttcattcatttatccactccttggtaagatgagat**

**Fortunella_venosa_3** **ttttcttcatttcttccacttaactttcattcatttatccactccttggtaagatgagat**

35530	35540	35550	35560	35570	35580

....|....|....|....|....|....|....|....|....|....|....|....|

**Atalantia_kwangtungens** **aggactattcctatatcgccctaagctgggaaattaaaaaatgagaaatccgaaaatctg**

**Citrus_reticulata_1** **cggactattcctatatcgccctaagctgggaaattaacaaatgagaaatccgaaaatctg**

**Citrus_reticulata_2** **cggactattcctatatcgccctaagctgggaaattaacaaatgagaaatccgaaaatctg**

**Citrus_trifoliata_3** **cggactattcctatatcgccctaagctgggaaattaacaaatgagaaatccgaaaatcgg**

**Citrus_aurantiifolia** **cggactattcctatatcgccctaagctgtgaaattaacaaatgagaaatccgaaaatctg**

**Citrus_aurantium** **cggactattcctatatcgccctaagctgggaaattcacaaatgagaaatccgaaaatctg**

**Citrus_cavaleriei** **cggactattcctatatcgccctaagctgggaaattaacaaatgagaaatccgaaaatctg**

**Citrus_hongheensis** **cggactattcctatatcgccctaagctgggaaattcacaaatgagaaatccgaaaatctg**

**Citrus_limon** **cggactattcctatatcgccctaagctgggaaattcacaaatgagaaatccgaaaatctg**

**Citrus_maxima** **cggactattcctatatcgccctaagctgggaaattcacaaatgagaaatccgaaaatctg**

**Citrus_medica** **cggactattcctatatcgccctaagctgggaaattaacaaatgagaaatctgaaaatcgg**

**Citrus_polytrifolia** **cggactattcctatatcgccctaagctgggaaattaacaaatgagaaatccgaaaatcgg**

**Citrus_reticulata** **cggactattcctatatcgccctaagctgggaaattaacaaatgagaaatccgaaaatctg**

**Citrus_sinensis** **cggactattcctatatcgccctaagctgggaaattcacaaatgagaaatccgaaaatctg**

**Fortunella_bawangica_1** **cggactattcctatatcgccctaagctgggaaattaacaaatgagaaatccgaaaatctg**

**Fortunella_bawangica_2** **cggactattcctatatcgccctaagctgggaaattaacaaatgagaaatccgaaaatctg**

**Fortunella_hindsii_1** **cggactattcctatatcgccctaagctgggaaattaacaaatgagaaatccgaaaatctg**

**Fortunella_hindsii_2** **cggactattcctatatcgccctaagctgggaaattaacaaatgagaaatccgaaaatctg**

**Fortunella_hindsii_3** **cggactattcctatatcgccctaagctgggaaattaacaaatgagaaatccgaaaatctg**

**Fortunella_japonica_1** **cggactattcctatatcgccctaagctgggaaattaacaaatgagaaatccgaaaatctg**

**Fortunella_japonica_2** **cggactattcctatatcgccctaagctgggaaattaacaaatgagaaatccgaaaatctg**

**Fortunella_japonica_3** **cggactattcctatatcgccctaagctgggaaattaacaaatgagaaatccgaaaatctg**

**Fortunella_japonica_4** **cggactattcctatatcgccctaagctgggaaattaacaaatgagaaatccgaaaatctg**

**Fortunella_japonica_5** **cggactattcctatatcgccctaagctgggaaattaacaaatgagaaatccgaaaatctg**

**Fortunella_japonica_6** **cggactattcctatatcgccctaagctgggaaattaacaaatgagaaatccgaaaatctg**

**Fortunella_japonica_7** **cggactattcctatatcgccctaagctgggaaattaacaaatgagaaatccgaaaatctg**

**Fortunella_japonica_8** **cggactattcctatatcgccctaagctgggaaattaacaaatgagaaatccgaaaatctg**

**Fortunella_japonica_9** **cggactattcctatatcgccctaagctgggaaattaacaaatgagaaatccgaaaatctg**

**Fortunella_japonica_10** **cggactattcctatatcgccctaagctgggaaattaacaaatgagaaatccgaaaatctg**

**Fortunella_japonica_11** **cggactattcctatatcgccctaagctgggaaattaacaaatgagaaatccgaaaatctg**

**Fortunella_japonica_12** **cggactattcctatatcgccctaagctgggaaattaacaaatgagaaatccgaaaatctg**

**Fortunella_margarita_1** **cggactattcctatatcgccctaagctgggaaattaacaaatgagaaatccgaaaatctg**

**Fortunella_margarita_2** **cggactattcctatatcgccctaagctgggaaattaacaaatgagaaatccgaaaatctg**

**Fortunella_venosa_1** **cggactattcctatatcgccctaagctgggaaattaacaaatgagaaatccgaaaatctg**

**Fortunella_venosa_2** **cggactattcctatatcgccctaagctgggaaattaacaaatgagaaatccgaaaatctg**

**Fortunella_venosa_3** **cggactattcctatatcgccctaagctgggaaattaacaaatgagaaatccgaaaatctg**

35590	35600	35610	35620	35630	35640

....|....|....|....|....|....|....|....|....|....|....|....|

**Atalantia_kwangtungens** **ggaacggggattaagattaacaagttatcaa-a-----------tttttttttttttctc**

**Citrus_reticulata_1** **ggaacggggattaagattaacaagttatcaa-t---tttttttttttttttttttttctc**

**Citrus_reticulata_2** **ggaacggggattaagattaacaagttatcaa-t----ttttttttttttttttttttctc**

**Citrus_trifoliata_3** **ggaacggggattaagattaacaagttatcaa-----------tttttttttttttttctc**

**Citrus_aurantiifolia** **ggaacggggattaagattaacaagttatcaa-t--ttttttttttttttttttttttctc**

**Citrus_aurantium** **ggaacggggattaagattaacaagttatcaatt--ttttttttttttttttttttttctc**

**Citrus_cavaleriei** **ggaacggggattaagattaacaagttatcaa-tttttttttttttttttttttttttctc**

**Citrus_hongheensis** **ggaacggggattaagattaacaagttatcaa------ttttttttttttttttttttctc**

**Citrus_limon** **ggaacggggattaagattaacaagttatcaa-t--ttttttttttttttttttttttctc**

**Citrus_maxima** **ggaacggggattaagattaacaagttatcaa-t--ttttttttttttt--tttttttctc**

**Citrus_medica** **ggaacggggattaagattaacaagttatcaa------ttttttttttttttttttttctc**

**Citrus_polytrifolia** **ggaacggggattaagattaacaagttatcaa-------tttttttttttttttttttctc**

**Citrus_reticulata** **ggaacggggattaagattaacaagttatcaa-t---tttttttttttttttttttttctc**

**Citrus_sinensis** **ggaacggggattaagattaacaagttatcaa-t--ttttttttttttttttttttttctc**

**Fortunella_bawangica_1** **ggaacggggattaagattaacaagttatcaa-t-----tttttttttttttttttttctc**

**Fortunella_bawangica_2** **ggaacggggattaagattaacaagttatcaa-t-----tttttttttttttttttttctc**

**Fortunella_hindsii_1** **ggaacggggattaagattaacaagttatcaa-t--ttttttttttttttttttttttctc**

**Fortunella_hindsii_2** **ggaacggggattaagattaacaagttatcaa-----tttttttttttttttttttttctc**

**Fortunella_hindsii_3** **ggaacggggattaagattaacaagttatcaa-tt-ttttttttttttttttttttttctc**

**Fortunella_japonica_1** **ggaacggggattaagattaacaagttatcaa-t-------tttttttttttttttttctc**

**Fortunella_japonica_2** **ggaacggggattaagattaacaagttatcaa---------tttttttttttttttttctc**

**Fortunella_japonica_3** **ggaacggggattaagattaacaagttatcaa-t-------tttttttttttttttttctc**

**Fortunella_japonica_4** **ggaacggggattaagattaacaagttatcaa-t-------tttttttttttttttttctc**

**Fortunella_japonica_5** **ggaacggggattaagattaacaagttatcaa-t-------tttttttttttttttttctc**

**Fortunella_japonica_6** **ggaacggggattaagattaacaagttatcaa-t-------tttttttttttttttttctc**

**Fortunella_japonica_7** **ggaacggggattaagattaacaagttatcaa-t-------tttttttttttttttttctc**

**Fortunella_japonica_8** **ggaacggggattaagattaacaagttatcaa-t-------tttttttttttttttttctc**

**Fortunella_japonica_9** **ggaacggggattaagattaacaagttatcaa-t-------tttttttttttttttttctc**

**Fortunella_japonica_10** **ggaacggggattaagattaacaagttatcaa-t-----tttttttttttttttttttctc**

**Fortunella_japonica_11** **ggaacggggattaagattaacaagttatcaa----------ttttttttttttttttctc**

**Fortunella_japonica_12** **ggaacggggattaagattaacaagttatcaa-t-------tttttttttttttttttctc**

**Fortunella_margarita_1** **ggaacggggattaagattaacaagttatcaa-t-----tttttttttttttttttttctc**

**Fortunella_margarita_2** **ggaacggggattaagattaacaagttatcaa-t-----tttttttttttttttttttctc**

**Fortunella_venosa_1** **ggaacggggattaagattaacaagttatcaa-tt-ttttttttttttttttttttttctc**

**Fortunella_venosa_2** **ggaacggggattaagattaacaagttatcaa-tt-ttttttttttttttttttttttctc**

**Fortunella_venosa_3** **ggaacggggattaagattaacaagttatcaa-tt-ttttttttttttttttttttttctc**

35650	35660	35670	35680	35690	35700

....|....|....|....|....|....|....|....|....|....|....|....|

**Atalantia_kwangtungens** **taaaatttaggttcggggaacaaacagaatctctttatcacatgtgaaatctcgtggatc**

**Citrus_reticulata_1** **taaaatttgggttcggggaacaaacagaatctctttatcacatgtgaaatctggtggatc**

**Citrus_reticulata_2** **taaaatttgggttcggggaacaaacagaatctctttatcacatgtgaaatctggtggatc**

**Citrus_trifoliata_3** **taaaattttggttcggggaacaaacagaatctctttatcacatgtgaaatctggtggatc**

**Citrus_aurantiifolia** **taaaatttgggttcggggaacaaacagaatctctttatcacatgtgaaatctggtggatc**

**Citrus_aurantium** **taaaatttgggttcggggaacaaacagaatctctttatcacatgtgaaatccggtggatc**

**Citrus_cavaleriei** **taaaattttggttcggggaacaaacagaatctctttatcacatgtgaaatctggtggatc**

**Citrus_hongheensis** **taaaatttgggttcggggaacaaacagaatctctttatcacatgtgaaatccggtggatc**

**Citrus_limon** **taaaatttgggttcggggaacaaacagaatctctttatcacatgtgaaatccggtggatc**

**Citrus_maxima** **taaaatttgggttcggggaacaaacagaatctctttatcacatgtgaaatccggtggatc**

**Citrus_medica** **taaaattttggttcggggaacaaacagaatctctttatcacatgtgaaatctggtggatc**

**Citrus_polytrifolia** **taaaattttggttcggggaacaaacagaatctctttatcacatgtgaaatctggtggatc**

**Citrus_reticulata** **taaaatttgggttcggggaacaaacagaatctctttatcacatgtgaaatctggtggatc**

**Citrus_sinensis** **taaaatttgggttcggggaacaaacagaatctctttatcacatgtgaaatccggtggatc**

**Fortunella_bawangica_1** **taaaattttggttcggggaacaaacagaatctctttatcacatgtgaaatctggtggatc**

**Fortunella_bawangica_2** **taaaattttggttcggggaacaaacagaatctctttatcacatgtgaaatctggtggatc**

**Fortunella_hindsii_1** **taaaattttggttcggggaacaaacagaatctctttatcacatgtgaaatctggtggatc**

**Fortunella_hindsii_2** **taaaattttggttcggggaacaaacagaatctctttatcacatgtgaaatctggtggatc**

**Fortunella_hindsii_3** **taaaattttggttcggggaacaaacagaatctctttatcacatgtgaaatctggtggatc**

**Fortunella_japonica_1** **taaaattttggttcggggaacaaacagaatctctttatcacatgtgaaatctggtggatc**

**Fortunella_japonica_2** **taaaattttggttcggggaacaaacagaatctctttatcacatgtgaaatctggtggatc**

**Fortunella_japonica_3** **taaaattttggttcggggaacaaacagaatctctttatcacatgtgaaatctggtggatc**

**Fortunella_japonica_4** **taaaattttggttcggggaacaaacagaatctctttatcacatgtgaaatctggtggatc**

**Fortunella_japonica_5** **taaaattttggttcggggaacaaacagaatctctttatcacatgtgaaatctggtggatc**

**Fortunella_japonica_6** **taaaattttggttcggggaacaaacagaatctctttatcacatgtgaaatctggtggatc**

**Fortunella_japonica_7** **taaaattttggttcggggaacaaacagaatctctttatcacatgtgaaatctggtggatc**

**Fortunella_japonica_8** **taaaattttggttcggggaacaaacagaatctctttatcacatgtgaaatctggtggatc**

**Fortunella_japonica_9** **taaaattttggttcggggaacaaacagaatctctttatcacatgtgaaatctggtggatc**

**Fortunella_japonica_10** **taaaattttggttcggggaacaaacagaatctctttatcacatgtgaaatctggtggatc**

**Fortunella_japonica_11** **taaaattttggttcggggaacaaacagaatctctttatcacatgtgaaatctggtggatc**

**Fortunella_japonica_12** **taaaattttggttcggggaacaaacagaatctctttatcacatgtgaaatctggtggatc**

**Fortunella_margarita_1** **taaaattttggttcggggaacaaacagaatctctttatcacatgtgaaatctggtggatc**

**Fortunella_margarita_2** **taaaattttggttcggggaacaaacagaatctctttatcacatgtgaaatctggtggatc**

**Fortunella_venosa_1** **taaaattttggttcggggaacaaacagaatctctttatcacatgtgaaatctggtggatc**

**Fortunella_venosa_2** **taaaattttggttcggggaacaaacagaatctctttatcacatgtgaaatctggtggatc**

**Fortunella_venosa_3** **taaaattttggttcggggaacaaacagaatctctttatcacatgtgaaatctggtggatc**

35710	35720	35730	35740	35750	35760

....|....|....|....|....|....|....|....|....|....|....|....|

**Atalantia_kwangtungens** **tatggtcgaattggtaggt--------ccatgtatcaatcaaataaatttcgttccgttc**

**Citrus_reticulata_1** **tat-gtcgaattggtaggtccatgtatccatgtatcaatcaaataagtttcgttccgttc**

**Citrus_reticulata_2** **tat-gtcgaattggtaggtccatgtatccatgtatcaatcaaataagtttcgttccgttc**

**Citrus_trifoliata_3** **tat-gtcgaattggtaggtccatgtatccatgtatcaatcaaataagtttcgttccgttc**

**Citrus_aurantiifolia** **tat-gtcgaattggtaggtccatgtatccatgtatcaatcaaataagtttcgttccgttc**

**Citrus_aurantium** **tat-gtcgaattggtaggtccatgtatccatgtatcaatcaaataagtttcgttccgttc**

**Citrus_cavaleriei** **tat-gtcgaattggtaggtccatgtatccatgtatcaatcaaataagtttcgttccgttc**

**Citrus_hongheensis** **tat-gtcgaattggtaggtccatgtatccatgtatcaatcaaataagtttcgttccgttc**

**Citrus_limon** **tat-gtcgaattggtaggtccatgtatccatgtatcaatcaaataagtttcgttccgttc**

**Citrus_maxima** **tat-gtcgaattggtaggtccatgtatccatgtatcaatcaaataagtttcgttccgttc**

**Citrus_medica** **tat-gtcgaattggtaggtccatgtatccatgtatcaatcaaataagtttcgttccgttc**

**Citrus_polytrifolia** **tat-gtcgaattggtaggtccatgtatccatgtatcaatcaaataagtttcgttccgttc**

**Citrus_reticulata** **tat-gtcgaattggtaggtccatgtatccatgtatcaatcaaataagtttcgttccgttc**

**Citrus_sinensis** **tat-gtcgaattggtaggtccatgtatccatgtatcaatcaaataagtttcgttccgttc**

**Fortunella_bawangica_1** **tat-gtcgaattggtaggtccatgtacccatgtatcaatcaaataagtttcgttccgttc**

**Fortunella_bawangica_2** **tat-gtcgaattggtaggtccatgtacccatgtatcaatcaaataagtttcgttccgttc**

**Fortunella_hindsii_1** **tat-gtcgaattggtaggtccatgtacccatgtatcaatcaaataagtttcgttccgttc**

**Fortunella_hindsii_2** **tat-gtcgaattggtaggtccatgtacccatgtatcaatcaaataagtttcgttccgttc**

**Fortunella_hindsii_3** **tat-gtcgaattggtaggtccatgtacccatgtatcaatcaaataagtttcgttccgttc**

**Fortunella_japonica_1** **tat-gtcgaattggtaggtccatgtacccatgtatcaatcaaataagtttcgttccgttc**

**Fortunella_japonica_2** **tat-gtcgaattggtaggtccatgtacccatgtatcaatcaaataagtttcgttccgttc**

**Fortunella_japonica_3** **tat-gtcgaattggtaggtccatgtacccatgtatcaatcaaataagtttcgttccgttc**

**Fortunella_japonica_4** **tat-gtcgaattggtaggtccatgtacccatgtatcaatcaaataagtttcgttccgttc**

**Fortunella_japonica_5** **tat-gtcgaattggtaggtccatgtacccatgtatcaatcaaataagtttcgttccgttc**

**Fortunella_japonica_6** **tat-gtcgaattggtaggtccatgtacccatgtatcaatcaaataagtttcgttccgttc**

**Fortunella_japonica_7** **tat-gtcgaattggtaggtccatgtacccatgtatcaatcaaataagtttcgttccgttc**

**Fortunella_japonica_8** **tat-gtcgaattggtaggtccatgtacccatgtatcaatcaaataagtttcgttccgttc**

**Fortunella_japonica_9** **tat-gtcgaattggtaggtccatgtacccatgtatcaatcaaataagtttcgttccgttc**

**Fortunella_japonica_10** **tat-gtcgaattggtaggtccatgtacccatgtatcaatcaaataagtttcgttccgttc**

**Fortunella_japonica_11** **tat-gtcgaattggtaggtccatgtacccatgtatcaatcaaataagtttcgttccgttc**

**Fortunella_japonica_12** **tat-gtcgaattggtaggtccatgtacccatgtatcaatcaaataagtttcgttccgttc**

**Fortunella_margarita_1** **tat-gtcgaattggtaggtccatgtacccatgtatcaatcaaataagtttcgttccgttc**

**Fortunella_margarita_2** **tat-gtcgaattggtaggtccatgtacccatgtatcaatcaaataagtttcgttccgttc**

**Fortunella_venosa_1** **tat-gtcgaattggtaggtccatgtacccatgtatcaatcaaataagtttcgttccgttc**

**Fortunella_venosa_2** **tat-gtcgaattggtaggtccatgtacccatgtatcaatcaaataagtttcgttccgttc**

**Fortunella_venosa_3** **tat-gtcgaattggtaggtccatgtacccatgtatcaatcaaataagtttcgttccgttc**

35770	35780	35790	35800	35810	35820

....|....|....|....|....|....|....|....|....|....|....|....|

**Atalantia_kwangtungens** **tgatggggtcagatcaattcaagtcaattcc------------------attagggtcgg**

**Citrus_reticulata_1** **tgatggggtcagatcaatttaagtcaattcc------------------attagggtcgg**

**Citrus_reticulata_2** **tgatggggtcagatcaatttaagtcaattcc------------------attagggtcgg**

**Citrus_trifoliata_3** **tgatggggtcagatcaatttaagtcaattcc------------------attagggtcgg**

**Citrus_aurantiifolia** **tgatggggtcagatcaatttaagtcaattcc------------------attagggtcgg**

**Citrus_aurantium** **tgatggggtcagatcaatttaagtcaattccattagggtcggtcttgaaattagggtcgg**

**Citrus_cavaleriei** **tgatggggtcagatcaatttaagtcaattcc------------------attagggtcgg**

**Citrus_hongheensis** **tgatggggtcagatcaatttaagtcaattccattagggtcggtcttgaaattagggtcgg**

**Citrus_limon** **tgatggggtcagatcaatttaagtcaattccattagggtcggtcttgaaattagggtcgg**

**Citrus_maxima** **tgatggggtcagatcaatttaagtcaattccattagggtcggtcttgaaattagggtcgg**

**Citrus_medica** **tgatggggtcagatcaatttaagtcaattcc------------------attagggtcgg**

**Citrus_polytrifolia** **tgatggggtcagatcaatttaagtcaattcc------------------attagggtcgg**

**Citrus_reticulata** **tgatggggtcagatcaatttaagtcaattcc------------------attagggtcgg**

**Citrus_sinensis** **tgatggggtcagatcaatttaagtcaattccattagggtcggtcttgaaattagggtcgg**

**Fortunella_bawangica_1** **tgatggggtcagatcaatttaagtcaattcc------------------attagggtcgg**

**Fortunella_bawangica_2** **tgatggggtcagatcaatttaagtcaattcc------------------attagggtcgg**

**Fortunella_hindsii_1** **tgatggggtcagatcaatttaagtcaattcc------------------attagggtcgg**

**Fortunella_hindsii_2** **tgatggggtcagatcaatttaagtcaattcc------------------attagggtcgg**

**Fortunella_hindsii_3** **tgatggggtcagatcaatttaagtcaattcc------------------attagggtcgg**

**Fortunella_japonica_1** **tgatggggtcagatcaatttaagtcaattcc------------------attagggtcgg**

**Fortunella_japonica_2** **tgatggggtcagatcaatttaagtcaattcc------------------attagggtcgg**

**Fortunella_japonica_3** **tgatggggtcagatcaatttaagtcaattcc------------------attagggtcgg**
[truncated: 7,233,665 more chars]
